# Active Macropinocytosis, Lipid Catabolism, and Exhausting Immune Microenvironment of Ascites Tumor Cells Are Involved in Resistance to Platinum‐Based Therapy in Patients With High‐Grade Serous Ovarian Cancer

**DOI:** 10.1002/mco2.70657

**Published:** 2026-03-07

**Authors:** Ruiqi Zheng, Ying Cui, Xun Hu, Xin Dong, Bo Meng, Luhong Wen, Anqi Chen, Zijng Wang, Guifen Qiang, Shujun Cheng, Yang Zhao, Huiqin Guo, Ting Xiao

**Affiliations:** ^1^ State Key Laboratory of Molecular Oncology Department of Etiology and Carcinogenesis National Cancer Center/National Clinical Research Center for Cancer/Cancer Hospital Chinese Academy of Medical Sciences ＆ Peking Union Medical College Beijing China; ^2^ Department of Pathology National Cancer Center/National Clinical Research Center for Cancer/Cancer Hospital Chinese Academy of Medical Sciences ＆ Peking Union Medical College Beijing China; ^3^ Department of Imaging Diagnosis National Cancer Center/National Clinical Research Center for Cancer/Cancer Hospital Chinese Academy of Medical Sciences ＆ Peking Union Medical College Beijing China; ^4^ Department of Clinical Laboratory National Cancer Center/National Clinical Research Center for Cancer/Cancer Hospital Chinese Academy of Medical Sciences ＆ Peking Union Medical College Beijing China; ^5^ Technology Innovation Center of Mass Spectrometry for State Market Regulation, Center for Advanced Measurement Science National Institute of Metrology Beijing China; ^6^ China Innovation Instrument Company Ltd Ningbo Zhejiang China; ^7^ Beijing Key Laboratory of Innovative Drug Discovery and Polymorphic Druggability Research for Cerebrovascular Diseases Institute of Materia Medica Chinese Academy of Medical Sciences ＆ Peking Union Medical College Beijing China

**Keywords:** immune microenvironment, lipid metabolism, macropinocytosis, ovarian cancer, platinum resistance

## Abstract

Platinum resistance remains a clinical challenge in ovarian cancer. Ascites represents an important mediator and a unique tumor microenvironment (TME) for invasion and metastasis. This study performed high‐resolution mass spectrometry (MS) on pre‐chemotherapy ascites cells from ovarian cancer patients. Integrating proteomic profiling, clinical data, and single‐cell analysis revealed that platinum‐resistant ascites displayed a distinct microenvironmental: the macropinocytosis‐related protein Src homology 3 domain‐containing YSC84‐like 1 (SH3YL1) was upregulated, whereas the immune‐activation marker CD44 was downregulated in resistant cases. Single‐cell analyses and pathway enrichment indicated immune exhaustion in resistant ascites, alongside enhanced macropinocytosis and lipid catabolism in tumor cells. Clinical data also showed that resistant ascites are lipid‐rich, with immunofluorescence plus flow cytometry confirming its association with immune exhaustion. Cellular experiments confirmed that SH3YL1‐mediated macropinocytosis promoted lipid uptake, and its inhibition partially restored cisplatin sensitivity. A combined model of immune exhaustion, macropinocytosis, and lipid catabolism suggests these ascites‐associated features could somewhat predict the platinum sensitivity in ovarian cancer tissues. We therefore propose the hypothesis that, in a lipid‐rich ascites microenvironment, immune exhaustion occurs while tumor cells activate macropinocytosis and lipid catabolism—forming a network of resistance mechanisms that may serve as potential predictive markers or intervention targets for platinum resistance.

## Graphical abstract

Platinum‐resistant patients exhibited ascites suffer from immune exhaustion and enriched in lipids, which is used by tumor cells as energy resources to counteract platinum damage through active macropinocytosis and lipid catabolism. These resistant features in ascites can also predict, to a certain extent, the efficacy of platinum‐based therapy in ovarian tissues.

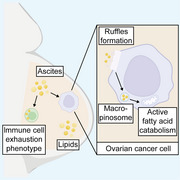

## 1 Introduction

Epithelial ovarian cancers comprise serous, mucinous, endometrioid, clear cell, transitional, squamous, mixed, and undifferentiated subtypes, with high‐grade serous ovarian cancer (HGSOC) being most prevalent [1]. Global Cancer Statistics 2020 indicate ovarian tumors account for 3.4% of female cancer incidence and 4.7% of mortality, reflecting their high lethality [2]. This is due to the deep pelvic location of ovaries and the lack of effective early screening. In addition to surgery, platinum‐based chemotherapy is standard treatment regimen; most patients initially respond, but the majority develop resistance, defined as progression during therapy or within 6 months after treatment [1].

Platinum resistance arises when ovarian cancer cells adapt to DNA damage and survive [3]. Adaptation mechanisms include: (1) limiting platinum‐DNA adduct formation, for example, inactivation of platinum compounds through conjugation with reducing substances like glutathione (GSH); (2) evading cell death, for example, activating DNA damage repair pathway and entering dormancy [4, 5].

Accumulating evidence suggests metabolic reprogramming, notably lipid metabolism alterations, supports tumor survival under chemotherapy by supplying energy and biosynthetic precursors [6]. For example, platinum‐resistant ovarian cancer cells often show diminished biosynthesis but enhanced utilization of lipids [7, 8]. These intrinsic changes are deeply influenced by the tumor microenvironment (TME); in fluid environments, tumor cells form spheroids to withstand hypoxia and nutrient deprivation while promoting survival and metastasis [9]. Ascites is a humoral environment in contact with tumor lesions and the omentum majus, containing tumor cells from multiple lesions along with adipocyte‑derived lipids and adipokines from the omentum majus [10]. Consequently, ascites composition differs markedly from blood and other biospecimens and critically influences ovarian cancer behavior [11, 12].

Abundant lipids in malignant ascites not only serve as metabolic substrates for tumor cells but also impair immune cell function. Lipid metabolite (e.g., prostaglandin E_2_ [13]), as well as lipid accumulation and peroxidation, can disrupt immune effectors, reducing interferon‑γ (IFN‑γ) production and cytotoxic activity [14], resulting in an immunosuppressive and exhausted TME [15].

This study profiled pre‐chemotherapy ascites cell pellets from 18 HGSOC patients. Patients were classified as platinum‑sensitive or platinum‑resistant based on subsequent chemotherapy response. We identified three significant features predictive of HGSOC platinum resistance: active micropinocytosis, lipid catabolism, together with an exhausted immune microenvironment, and propose hypotheses explaining how these features interact to promote adaptation to platinum‑induced damage, offering new insights into ascites'role and potential clinical targets.

## 2 Results

### 2.1 Proteomic Comparison of Ascites From Platinum‐Sensitive vs. Platinum‐Resistant HGSOC Patients

**FIGURE 1:**
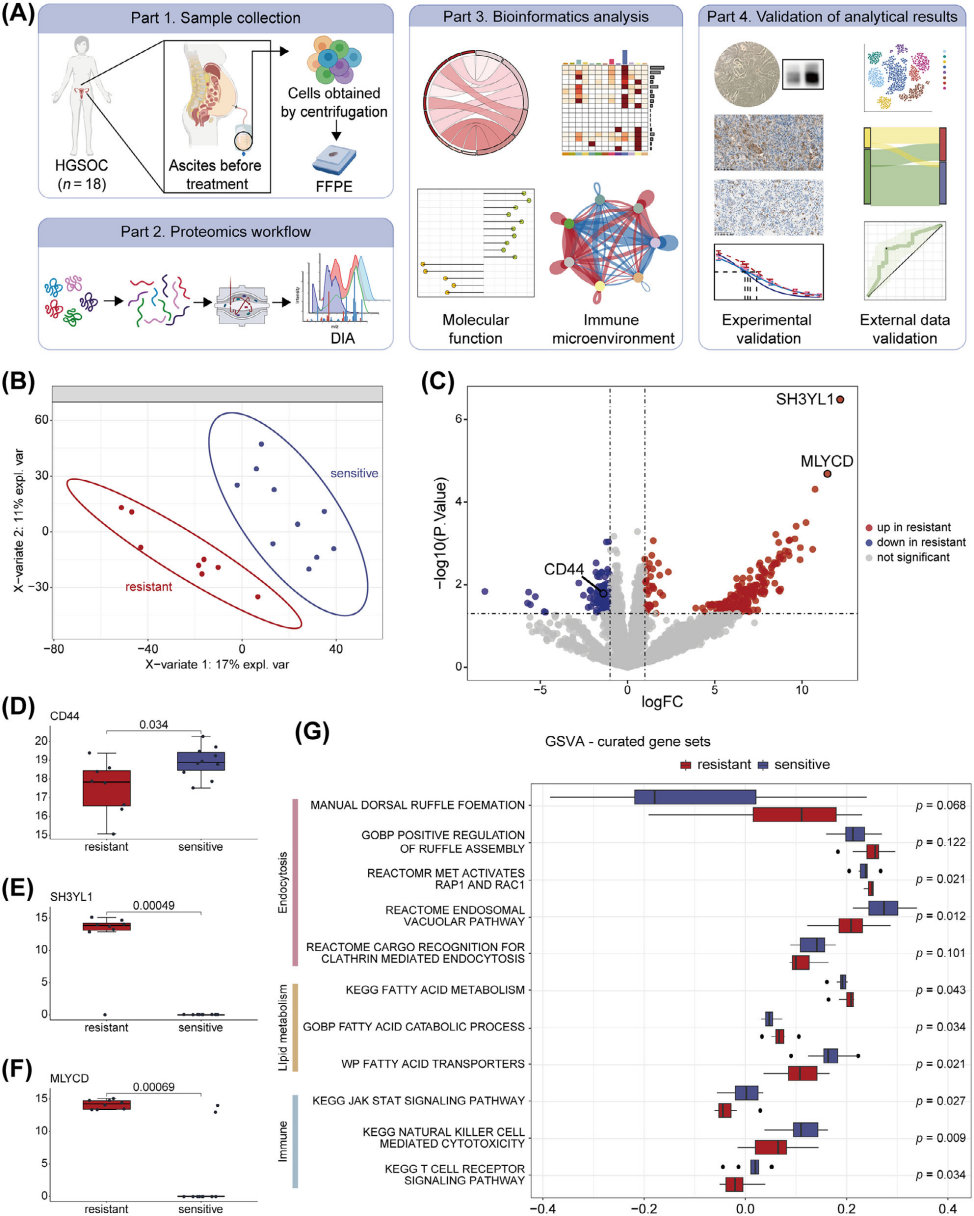
Platinum resistance‐related pathways and molecules in HGSOC patients. (A) Schematic overview of the overall study design. (B) PLS‐DA on proteomic data discriminates platinum‐resistant (*n* = 8) and sensitive (*n* = 10) ascites samples. (C) Volcano plot demonstrating differential protein expression between resistant and sensitive groups (screening criteria: *p *< 0.05, |logFC| > 1). (D–F) Box‐and‐whisker plots demonstrating expression of the differential proteins CD44 (D), SH3YL1 (E), and MLYCD (F) in the resistant group versus the sensitive group. (G) Box‐and‐whisker plot demonstrating the difference in pathway scores between resistant and sensitive groups after ssGSEA annotation. For B–G, all *n* = 18 HGSOC ascites samples were analyzed (*n* = 8 for resistant and *n* = 10 for sensitive). For B and D–G, two‐sided Wilcoxon rank‐sum tests were used. The schematic illustration in Panel A was created by the authors using Adobe Illustrator.

To identify protein markers in ascites predictive of platinum‐based chemotherapy efficacy, we performed quantitative proteomics on formalin fixed paraffin embedded (FFPE) cell‐pellet samples collected before treatment from ascites of 18 HGSOC patients, who were stratified into platinum‐sensitive (*n* = 10) and platinum‐resistant (*n* = 8) groups based on subsequent clinical outcomes (Figure 1A; Table S1). Partial least squares discriminant analysis (PLS‐DA) showed clear separation of global protein profiles between sensitive and resistant groups (Figure 1B). Differential expression analysis identified three characteristic molecules: CD44, SH3YL1, and Malonyl‐CoA decarboxylase (MLYCD) (Figure 1C).

CD44 is implicated in immune cell migration and activation [16], such as enhancing STAT3 binding to interferon‐stimulated response element (ISRE) in the JAK‐STAT3 pathway to facilitate its transcription [17, 18]. SH3YL1 critically regulates dorsal ruffles formation, the structural basis of macropinocytosis, and a special form of endocytosis [19]. MLYCD mediates a switch toward fatty acid catabolism [20]. In the resistant group, CD44 (*p* = 0.0343, Figure 1D) was downregulated, while SH3YL1 (*p* = 0.00049, Figure 1E) and MLYCD (*p* = 0.00069, Figure 1F) were upregulated, suggesting reduced immunoreactivity, enhanced macropinocytosis, and active lipid catabolism.

To explore associated biological processes, up‐ and downregulated proteins in the resistant group were subjected to Kyoto Encyclopedia of Genes and Genomes (KEGG) enrichment. The sensitive group was enriched for “natural killer (NK) cell‐mediated cytotoxicity,” “Fc gamma R‐mediated phagocytosis,” “antigen processing and presentation,” “B cell receptor signaling pathway,” and “chemokine signaling pathway” that are all immune activation pathways. The resistant group was enriched for “PI3K‐Akt signaling pathway,” “ErbB signaling pathway,” “regulation of actin cytoskeleton” that related to cell membrane remodeling, promoting the macropinocytosis [21, 22, 23]; and “AMPK signaling pathway” activates fatty acid oxidation [20, 24], along with “central carbon metabolism” in cancer suggesting active energy metabolism (all *p* < 0.05; Figure S1A; Tables S2 and S3).

Using single‐sample gene set enrichment analysis (ssGSEA), we converted protein expression into pathway enrichment scores with gene sets from gsea‐msigdb (“MANUAL dorsal ruffle formation” refers to a custom gene set comprising “Dynamin‐2, SH3YL1, inositol polyphosphate phosphatase‐like Protein 1, and pleckstrin homology domain‐containing family A member 1” based on existing studies [19, 25, 26], (Table S4). In the sensitive group, “KEGG JAK STAT signaling pathway,” “KEGG NK cell mediated cytotoxicity,” and “KEGG T cell receptor signaling pathway” were significantly higher (all *p* < 0.05)—consistent with active immune microenvironment [18], while “WP fatty acid transporters” (*p* = 0.021) and “REACTOME cargo recognition for clathrin mediated endocytosis” (*p* = 0.101) suggested selective endocytosis [21, 27]. In the resistant group, enrichment of “REACTOME MET activates RAP1 and RAC1” (*p* = 0.021, involved in remodeling the actin cytoskeleton) [28, 29], “MANUAL dorsal ruffle formation” (*p* = 0.068) [27, 30], and “GOBP positive regulation of ruffle assembly” (*p* = 0.0122) indicated ruffles formation and micropinocytosis, “KEGG fatty acid metabolism” (*p* = 0.043) and “GOBP fatty acid catabolic process” (*p* = 0.034) suggested active lipid catabolism (Figure 1G and Table S5).

A range of biological features of tumors can be explained by their microenvironment, so we further investigated the ascites environment.

### 2.2 Tumor‐Immune Landscape in Ascites of Platinum‐Sensitive vs. Platinum‐Resistant HGSOC Patients

**FIGURE 2:**
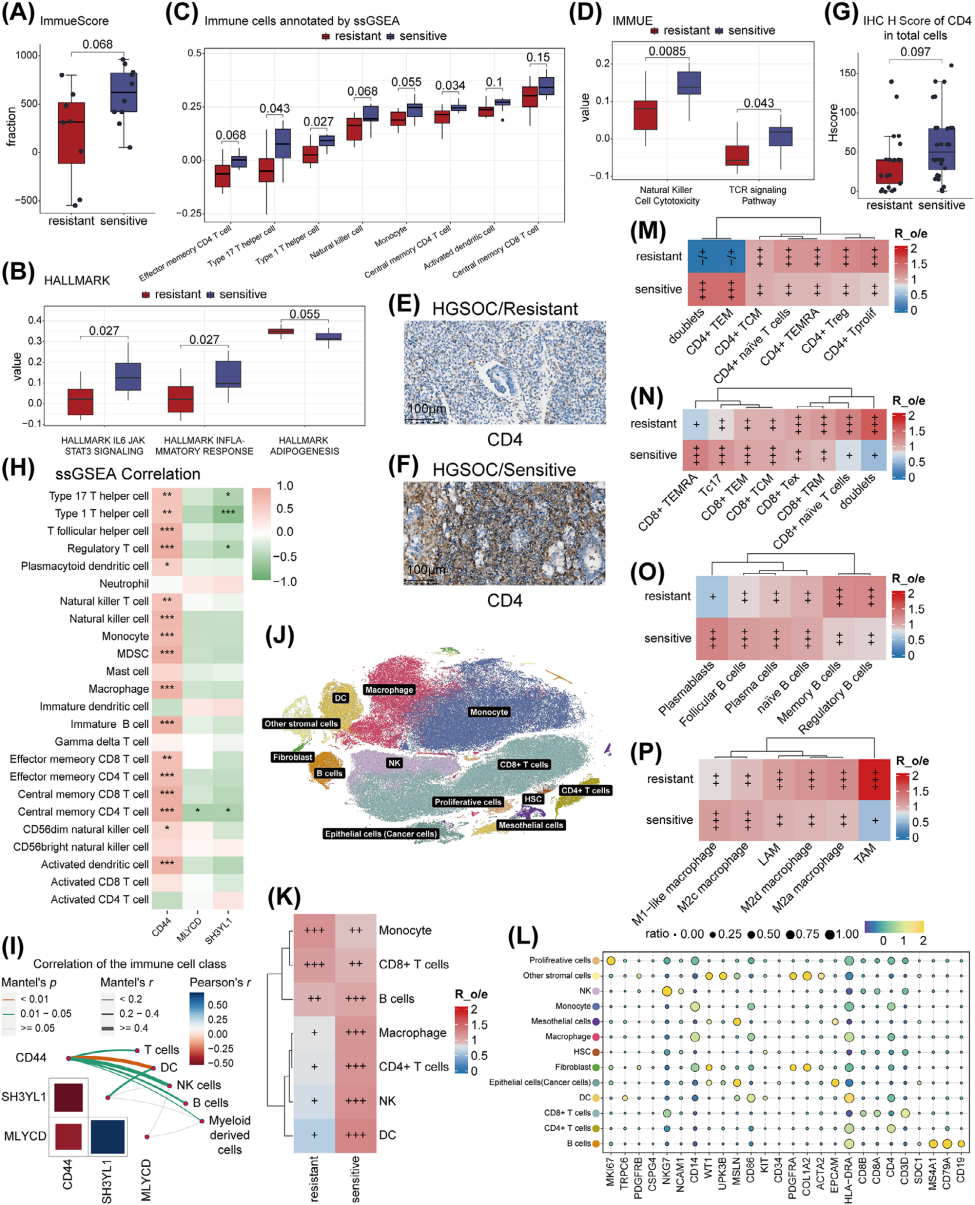
Immune microenvironment characterization of ovarian cancer ascites in platinum‐resistant versus sensitive patients. (A) Box‐and‐whisker plot comparing overall immunity scores between resistant and sensitive groups as calculated by ESTIMATE. (B–D) Box‐and‐whisker plot comparing HALLMARK‐related pathway scores (B), immune cell type scores (C), and IMMUNE‐related pathway scores (D) between two groups after ssGSEA annotation. (E, F) Representative IHC images of CD4‐staining in extended ascites‐derived cell samples from resistant (E) and sensitive patients (F). Scale bar = 100 µm. (G) Box‐and‐whisker plot comparing CD4 IHC H score between resistant and sensitive groups (*n* = 25 for resistant and *n* = 32 for sensitive, two‐sided Wilcoxon rank‐sum tests). (H) Heatmap demonstrating the correlation between expression of characteristic differential protein and scores for each immune cell type after ssGSEA annotation (Spearman correlation). (I) Correlation between differential protein expression and aggregated immune cell type scores after ssGSEA annotation (Pearson correlation). (J) t‐SNE plot of single‐cell transcriptomic data from 10 ascites samples, showing 13 clusters (0.5 resolution). Each dot corresponds to a single cell, colored by assigned clusters. (K) Distribution preferences of each immune cell type in the resistant and sensitive groups were estimated by the Ro/e. (L) Bubble plot summarizing cell markers used for cell‐type annotation. (M–P) Distribution preferences of CD4+ T cells (M), CD8+ T cells (N), B cells (O), and Macrophages (P) in the resistant and sensitive groups, estimated by the Ro/e. For A–D and H, I, all *n* = 18 HGSOC ascites samples were analyzed (*n* = 8 for resistant and *n* = 10 for sensitive). For A–D, two‐sided Wilcoxon rank‐sum tests were used. For J–L, all *n* = 10 ovarian cancer ascites samples were analyzed.

A study reported a paucity of immunoreactive cell infiltration in platinum‐resistant ovarian cancer tissues [31], leading us to speculate that the platinum‐resistant ascites might exhibit a similar immunosuppressive microenvironment. Protein expression profiles were converted to immune scores by the ESTIMATE algorithm; the scores tended to be higher in the sensitive group (*p* = 0.068, Figure 2A; Table S6).

Next, using ssGSEA (Figure 2B; Table S7), we evaluated enrichment of tumor‐feature–associated pathways. “HALLMARK IL6 JAK STAT3 signaling” (*p* = 0.027) and “HALLMARK inflammatory response” (*p* = 0.027) were scored lower in the resistant group, implying suppressed immune activation and inflammation, while “HALLMARK adipogenesis” showed a trend toward higher score (*p* = 0.055), suggesting that resistant cells may adopt adipocyte‐like traits such as enhanced lipid storage [32, 33]. Using immune cell marker sets (Figure 2C, Tables S4 and S8), the sensitive group showed higher infiltration across multiple cell types; particularly, Type 17 T helper cell (*p* = 0.043) and Type 1 T helper cell (*p* = 0.027) differed significantly, with effector memory CD4^+^ T cell (*p* = 0.068) and NK cells (*p* = 0.068) also trending higher. Using an immune pathway‐related gene set, enrichment scores for “NK cell cytotoxicity” (*p* = 0.0085) and “TCR signaling pathway” (*p* = 0.043) were significantly upregulated in the sensitive group, pointing to stronger cytotoxic and adaptive immune activity (Figure 2D; Table S9).

Immunohistochemistry (IHC) validation in 57 HGSOC ascites cell pellets FFPE samples (collected before platinum‐based therapy) confirmed that CD4 expression was higher in the sensitive group (*p* = 0.097, Figure 2E–G; Table S10). Correlation analysis between CD44, SH3YL1, and MLYCD expression with 28 immune cell types showed that CD44 positively correlated with most immune cells, while SH3YL1 and MLYCD were negatively correlated (Figures 2H, S4A–D). When grouped into major categories (T cells, B cells, dendritic cells [DCs], NK cells, and myeloid‐derived cells), the strongest correlation emerged between CD44 and DCs (Figure 2I), suggesting a role for CD44 in DCs migration and subsequent activation of adaptive immunity.

To further characterize the immune landscape in ascites, we re‐analyzed published single‐cell transcriptome data of ovarian cancer ascites, including 10 untreated samples: eight HGSOC (six platinum‐sensitive, two platinum‐resistant), one ovarian clear cell carcinoma (OCCC, platinum‐sensitive), and one endometrioid carcinoma of the ovary (ECO, platinum‐resistant) [34]. Using provided markers (Figure 2L; Table S11), we categorized cells into 13 major clusters: B cells, CD4^+^ T cells, CD8^+^ T cells, NK, monocyte, macrophage, DC, fibroblast, mesothelial cells, other stromal cells, epithelial cells (cancer cells), hematopoietic stem cell (HSC), and proliferative cells (Figure 2J). To dissect functional heterogeneity, we subdivided immune lineages (Table S11): CD4^+^ T cells into naïve, regulatory (Treg), effector memory (TEM), central memory (TCM), terminal effector memory (TEMRA), proliferating (Tprolif), and doublets [35] (Figure S2A); CD8^+^ T cells into naïve, TEM, TEMRA, TCM, exhausted (Tex), Tc17, tissue‐resident memory (TRM), and doublets [35] (Figure S2B); B cells into naïve, memory, follicular, regulatory (Breg), plasma cells, and plasmablasts [36] (Figure S2C); and macrophages into M1‐like, M2a, M2c, M2d, tumor‐associated macrophages (TAM), and lipid‐associated macrophages (LAM) [37] (Figure S2D).

We quantified relative enrichment (R_o/e_) of seven major immune categories in sensitive versus resistant groups (Figure 2K). Immunoreactive populations—CD4^+^ T cells, DC, NK, and B cells—were more abundant in the sensitive group. Among CD4^+^ subsets, cytotoxic CD4^+^ TEM cells showed higher infiltration in the sensitive group, while immunosuppressive CD4^+^ Tregs were enriched in the resistant group (Figure 2M). Among CD8^+^ subsets, cytotoxic CD8^+^ TEM, and CD8^+^ TEMRA showed greater infiltration in the sensitive group, whereas CD8^+^ Tex were more abundant in the resistant group (Figure 2N). Among B cells, plasma cells and plasmablasts infiltrated more in the sensitive group, while immunosuppressive Bregs infiltrated more in the resistant group (Figure 2O). Among macrophages, immunologically activated M1 cells infiltrated more in the sensitive group, whereas more tumor‑promoting M2a/M2d and TAM in the resistant group (Figure 2P). Differential expression analysis revealed that the sensitive group exhibited higher expression of immune activation markers—CD44, CD69, GZMB, and IFN‐γ—in CD4^+^ TEMRA, CD8^+^ TEM, CD8^+^ TEMRA, and CD8^+^ Tex cells, indicating stronger immune activation and lower exhaustion. (Figure S4E–H). We then analyzed intercellular interactions among 34 clusters (excluding doublets) to assess how cellular composition and crosstalk within ascites may influence response to platinum‑based chemotherapy.

### 2.3 Reduced Intercellular Pro‑Immune Signaling in Ascites From Platinum‑Resistant Patients

For convenience in analysis and narration, we re‑classified individual cell subtypes into broader functional categories. We defined “immune effector‐related CD4 T cells” (comprising CD4^+^ TCM, TEM, TEMRA, and Tprolif), “immune effector‐related CD8 T cells” (CD8^+^ TCM, TEM, TEMRA, TRM, and Tc17), “immune effector‐related B cells” (memory B, plasma cells, and plasmablasts), “immunosuppression related cells” (CD4^+^ Treg, Breg, CD8^+^ Tex, M2a, M2c, M2d, and TAM), “antigen‐presenting cells (APCs)” (DC, monocyte), “innate effector related cells” (NK, M1, and LAM), “stromal cells” (fibroblast, mesothelial cells, and other stromal cells), “undifferentiated types” (naïve B cells, follicular B cells, CD4^+^, and CD8^+^ naïve T cells), and “other cell types” (proliferative cells and HSC).

Comparing cell–cell communication between groups, we found that in the sensitive ascites, both the number and strength of interactions among epithelial cells (cancer cells) and effector immune cells were higher. In contrast, resistant ascites showed dominant interactions involving stromal and suppressive networks, with stronger communication between epithelial cells (cancer cells) and stromal cells, as well as among stromal cells, immune effector‐related CD4T, CD8T, B, innate effector‐related cells, and APCs (Figure S3A). Subdividing to all 34 cell types also revealed stronger interactions among epithelial cells (cancer cells), CD8^+^ TEM, CD8^+^ TEMRA, and CD4^+^ TEM (Figure S4M) as well as among CD8^+^ TEM, CD8^+^ TEMRA, NK, monocyte, and CD4^+^ TEM (Figure S4N,O) in the sensitive group.

We normalized communication intensities across signaling pathways and visualized differences via heatmaps. For overall signals (Figure S3B), the sensitive group showed elevated chemotaxis/haptotaxis‑related signals (C‐X‐C motif ligand [CXCL], C‐C motif ligand [CCL], LAMININ [38]), innate immunity signals (RESISTIN [proinflammatory]) [39], COMPLEMENT, XC‐chemokine receptor (XCR, antigens cross‐presented by DCs) [40], Fms‐like tyrosine kinase 3 (FLT3, DC expansion) [41], and adaptive immunity signals (B‐cell activating factor [BAFF, B‐cell activation]) [42], CD23 (B‐cell activation [42]), lymphocyte‐specific protein tyrosine kinase (LCK, TCR initiation [43]), major histocompatibility complex class I (MHC I), and CD6 (T‐cell costimulation) [44]). In contrast, extracellular matrix‐associated COLLAGEN signaling was stronger in the resistant group. Permutation tests confirmed that in the sensitive group, CXCL (*p *= 0.054), CCL (*p* = 0.027), RESISTIN (*p* = 0.027), and BAFF (*p* = 0.082) exhibited higher signaling intensity in immune effector related CD4T cells (Figures S3E and S4I), LAMININ was elevated in both immune effector related CD4 (*p* = 0.033) and CD8T cells (*p* = 0.037) (Figure S3E), COMPLEMENT displayed a higher mean intensity in the tumor and stromal cells (*p* = 0.173, Figure S4J), which integrates epithelial cells (cancer cells), fibroblast, mesothelial cells, and other stromal cells.

For incoming signals (Figure S3C), the sensitive group showed stronger CXCL, LAMININ, RESISTIN, BAFF, CD23, and poliovirus receptor (PVR, triggering cytotoxicity and cytokine secretion [45]) signals. Permutation testing revealing that in the sensitive group, CXCL (*p* = 0.059), CCL (*p* = 0.054), and RESISTIN (*p* = 0.057) tended higher in immune effector related CD4T cells (Figure S3F), while LAMININ significantly elevated in both immune effector related CD4 (*p* = 0.032) and CD8T cells (*p* = 0.040) (Figure S3F).

For outgoing signals (Figure S3D), the sensitive group showed stronger innate immunity‐related COMPLEMENT, XCR, and PVR signals, whereas COLLAGEN was stronger in the resistant group. Permutation testing showed that in the sensitive group, BAFF (*p* = 0.082) exhibited higher mean intensity in immune effector related CD4T cells (Figure S4I), while COMPLEMENT showed higher mean intensity in tumor and stromal cells (*p* = 0.173) (Figure S4J). Detailed pairwise receptor–ligand differences are in Figure S5.

Chord plots visually summarize differences. In the sensitive group, CXCL and CCL signaling exhibit stronger communication among immune‐active subtypes (Figure S3G,H). LAMININ signaling exhibits stronger communication between epithelial cells (cancer cells) and effector T cells (Figure S3I), consistent with CD44 as a laminin receptor (Figure S5C,D), raising the possibility that laminin may mediate haptotactic recruitment of T cells. RESISTIN and BAFF signaling also exhibit stronger communication among immune‐active subtypes in sensitive ascites (Figures S3J and S4K), while COMPLEMENT signaling between epithelial cells (cancer cells) and DC/M1/monocyte was elevated (Figure S4L).

The above results suggest that in the sensitive group, tumor cells are more immunogenic, with effective innate and adaptive immune communication, and immune effector cells dominate the microenvironment. In the resistant group, immunity appears exhausted, and stromal signaling predominates. Since cell phenotypes are strongly shaped by their surrounding substances, we speculate that substances in ascites may drive this immune exhaustion. The pathway enrichment of adipogenesis and the widely reported intracellular lipid accumulation causing tumor growth and immune exhaustion [10, 17, 46, 47, 48] turned our attention to lipids.

### 2.4 Lipid‐Driven Immune Exhaustion in Ascites of Platinum‐Resistant HGSOC Patients

Ascites serves as a transitional environment for ovarian cancer dissemination to the omentum majus, housing components from both organs—tumor cells and lipid. Given clinical observations that hyperlipidemia favors ovarian cancer progression [49, 50, 51], we hypothesized that ascitic lipids contribute to platinum resistance.

**FIGURE 3:**
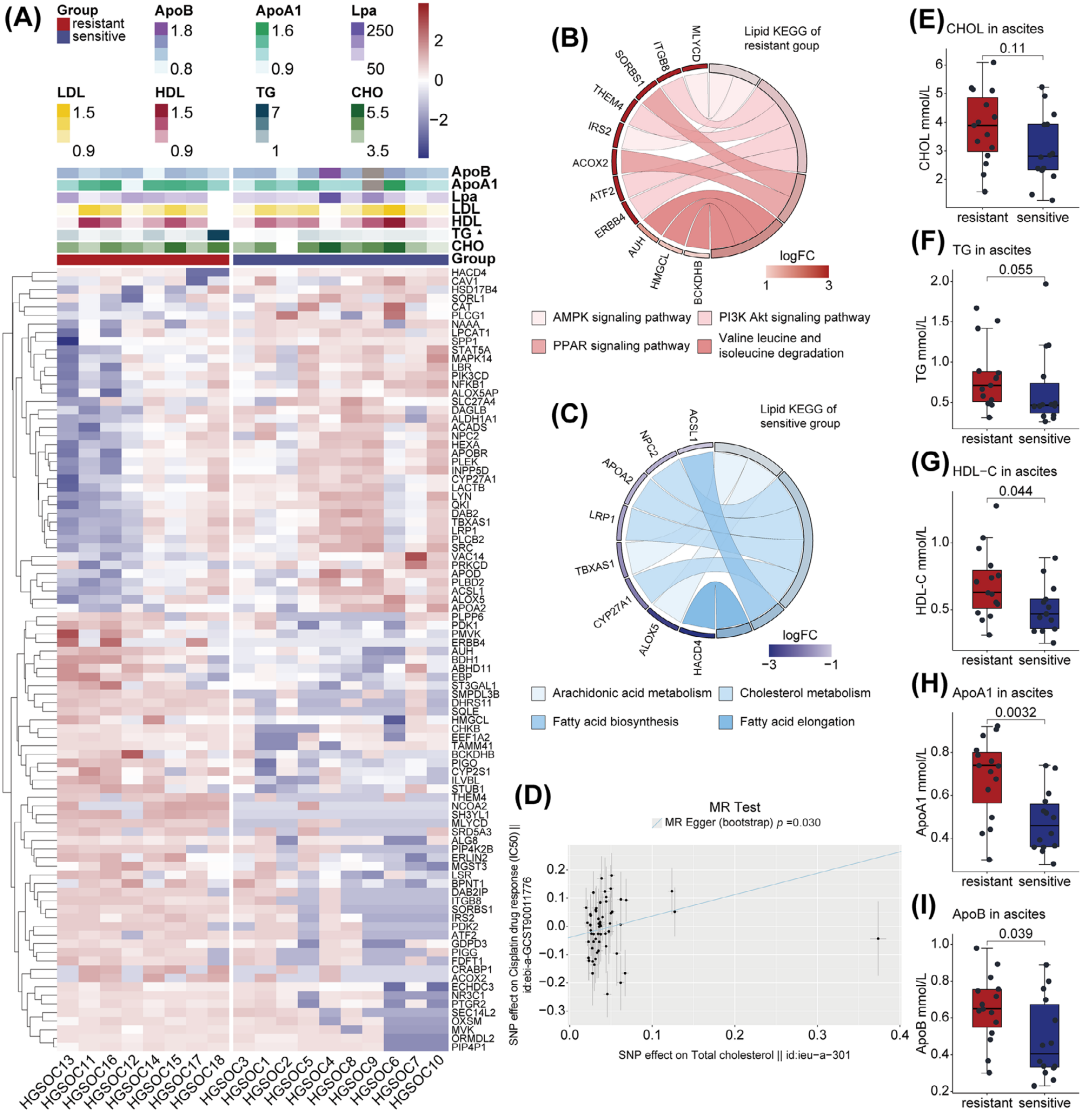
Lipid‐rich ascites environment in HGSOC patients. (A) Heatmap of differential lipid‐metabolism‐related proteins detected in ascites samples, alongside corresponding blood lipid panel levels (▲: *p* = 0.1, two‐sided Wilcoxon rank‐sum tests). (B, C) Chord plots demonstrating the KEGG‐enriched pathways from upregulated lipid metabolism‐related proteins in the resistant (B) and sensitive (C) groups, respectively. (D) Mendelian randomization (MR) analysis demonstrating the correlation between total CHOL levels and cellular cisplatin IC50 (half maximal inhibitory concentration). (E–I) Box‐and‐whisker plots comparing ascites levels of: CHOL (E, two‐tailed *t*‐test), TG (F, two‐sided Wilcoxon rank‐sum tests), HDL‐C (G, two‐tailed *t*‐test), ApoA1 (H, two‐tailed *t*‐test), and ApoB (I, two‐tailed *t*‐test) between resistant and sensitive groups. For A–C, all *n* = 18 HGSOC ascites samples were analyzed (*n* = 8 for resistant and *n* = 10 for sensitive). For E–I, *n* = 30 HGSOC ascites supernatant samples were analyzed (*n* = 16 for resistant and *n* = 14 for sensitive).

We retrospectively reviewed blood lipid levels at ascites collection in 18 samples (Figure 3A, Table S1), and blood triglyceride (TG) tended to be elevated in the resistant group (*p* = 0.1000). Intersecting the Gene Ontology (GO) gene set of the lipid metabolic process (GO:0006629) with our protein expression profiles identified differentially expressed lipid metabolism‑related proteins (Figure 3A). KEGG enrichment revealed up‑regulated proteins in the resistant group were enriched in the “AMPK signaling pathway,” “PI3K AKT signaling pathway,” and “PPAR signaling pathway” (Figure 3B, Table S12), all promoting lipid catabolism [24]. Proteins up‑regulated in the sensitive group showed enrichment in “fatty acid biosynthesis” and “fatty acid elongation”, suggesting more active fatty acid anabolism (Figure 3C, Table S13). Mendelian randomization analysis of total cholesterol (CHOL) (ieu‐a‐301) and cell line response to cisplatin—half maximal inhibitory concentration (IC50) (ebi‐a‐GCST90011776) showed a positive correlation between CHOL and cisplatin IC_50_ (Figure 3D, *p* = 0.0300), indicating CHOL may promote cellular resistance to platinum. In a validation cohort of 30 additional ascites samples (Table S14), the resistant group exhibited higher mean CHOL (Figure 3E, *p* = 0.110) and TG (Figure 3F, *p* = 0.055) levels, as well as significantly higher high‐density lipoprotein cholesterol (HDL‐C), apolipoprotein A1 (ApoA1), and apolipoprotein B (ApoB) levels (Figure 3G–I, all *p* < 0.05). Although HDL‐C is conventionally protective in systemic circulation, it cannot perform CHOL transport to the liver in ascites and may serve as a lipid reservoir. Thus, it appears that resistant ascites constitutes a lipid‐rich microenvironment.

**FIGURE 4:**
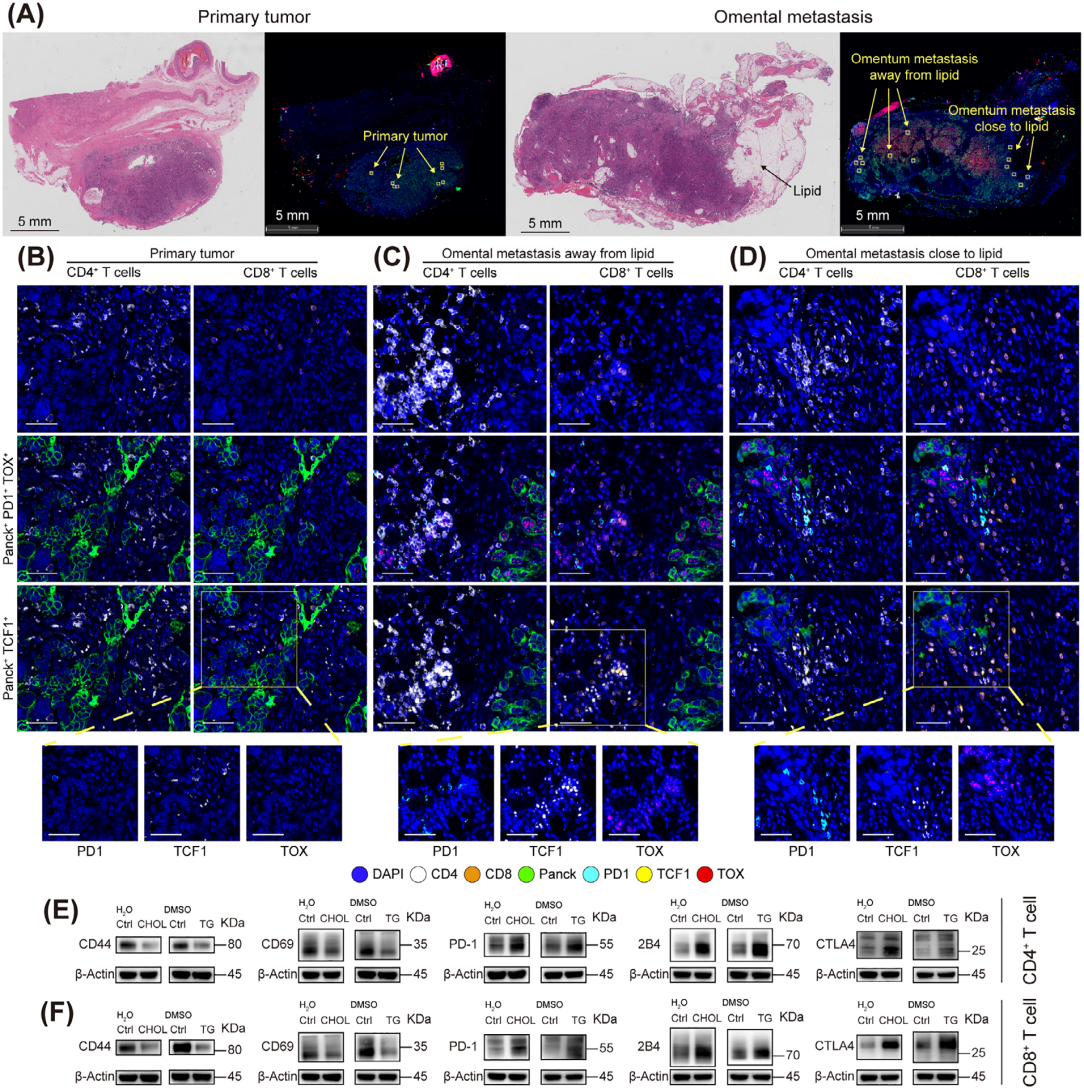
A lipid‐rich environment promotes an immune‐exhausted phenotype in ovarian cancer. (A) Hematoxylin–Eosin (HE) and overall immunofluorescence (IF) images of the primary tumor site and omental metastasis, with three types of spatially distinct regions selected for display. Scale bar = 5 mm (B, D) Representative merged IF images showing co‐staining for CD4/CD8, Pan‐Cytokeratin (PanCK), T‐cell factor 1 (TCF1), programmed death protein 1 (PD‐1), and thymocyte selection‐associated high mobility group box protein (TOX) in three contexts: primary tumor site (B), omental metastasis away from lipid (C), and omental metastasis close to lipid (D). Below each merged image are selected partial single‐channel images for the exhaustion markers PD‐1, TCF1, and TOX for clarity. Scale bar = 50 µm. (E) Representative WB results of CD4^+^ T cells treated in vitro with CHOL or TG, showing expression of activation (CD44 and CD69) and exhaustion markers (PD‐1, 2B4, Cytotoxic T‐lymphocyte–associated protein 4 [CTLA4]). (F) Representative WB of CD8+ T cell treated in vitro with CHOL or TG, showing expression of activation (CD44 and CD69) and exhaustion markers (PD‐1, 2B4, and CTLA4). Experiments were repeated at least three times.

To observe the situation in tissues, we collected FFPE sections of primary ovarian tumors and omental metastases. The omental metastases exhibited distinct lipid‐rich regions. Following multiplex immunofluorescence (IF) staining, we selected three representative fields: primary tumor, omental metastasis away from lipid, and omental metastasis close to lipid (Figure 4A). Markers included DAPI for nuclei; CD4 and CD8 for T‐cells; Pan‐Cytokeratin (PanCK) for tumor cells; CD44 and GZMB for T‐cell activation; programmed death protein 1 (PD‐1), T‐cell factor 1 (TCF1, indicating early reversible exhaustion), and thymocyte selection‐associated high mobility group box protein (TOX, indicating late irreversible exhaustion) for exhaustion [52]. At the primary site, immune cells spatially infiltrate the tumor focus, with lower PD‐1, TCF1, and TOX expression (Figure 4B). In both metastatic fields, immune cells were largely segregated from tumor cells, with minimal infiltration and demonstrated increased PD‑1, TCF1, and TOX expression. Furthermore, tumor cells in metastases showed stronger TOX expression than primary tumors, which is a poor prognostic marker in ovarian cancer [53]. Comparing metastatic zones, T cells near lipids exhibited higher PD‐1 and lower TCF1 expression, with frequent membrane‑ruptured (dead) T cells near lipids, suggesting greater exhaustion (Figure 4C,D). Differences in GZMB and CD44 were not apparent (Figure S6A–C). Single‑channel fluorescence images for each marker are shown in Figure S6D.

To verify whether lipids induce immune exhaustion, we cultured peripheral CD4^+^ and CD8^+^ T cells from healthy donors with soluble CHOL (0.75 µg/ml) or TG (1.25 mM) or corresponding solvents. After 7 days, Western blot (WB) showed CHOL and TG reduced the expression of activation markers CD44 and CD69, as well as increased exhaustion markers PD‐1, 2B4 (CD244), and cytotoxic T‐lymphocyte‐associated protein 4 (CTLA4) in both T cell types. (Figures 4E,F and S6E–H; Table S15).

Flow cytometry (FCM) (Table S16) with zombie (viability dye), CD4, CD8, GZMB, IFN‐γ, CD44, PD‐1, TCF‐1, and TOX showed that compared to the control group (Ctrl), lipid‐treated CD4^+^ T cells exhibited decreased expression of IFN‐γ and CD44 (all *p* < 0.05; Figure S7A,B,D). TOX (Ctrl vs. CHOL *p* = 0.015; Ctrl vs. TG *p* = 0.041), TCF1 (Ctrl vs. CHOL *p* = 0.1; Ctrl vs. TG *p* = 0.12), and PD‐1 (Ctrl vs. CHOL *p* = 0.18; Ctrl vs. TG *p* = 0.022) trended upward (Figures S7C,D and S8D,E). In CD8^+^ T cells, IFN‐γ tended downward (Figure S8F, Ctrl vs. CHOL *p* = 0.1; Ctrl vs. TG *p* = 0.4), while PD‐1 was significantly upregulated (Figure S7E,G, Ctrl vs. CHOL *p *< 0.001; Ctrl vs. TG *p *< 0.001). TOX also showed a slight upward trend (Figure S7F,G, Ctrl vs. CHOL *p* = 0.077; Ctrl vs. TG *p* = 0.077). The t‐SNE distributions and density plots for each biomarker are shown in Figure S8A–H.

The above results demonstrate that the lipid‐rich ascites environment likely contributes to immune exhaustion in platinum‐resistant patients.

### 2.5 Impact of CD44 Knockdown on CD4^+^ and CD8^+^ T‐Cell Phenotype

To investigate the role of CD44 in T‐cell phenotypes, we knocked down CD44 in peripheral blood‐derived CD4^+^ and CD8^+^ T cells using siRNA. All three siCD44 variants achieved significant CD44 knockdown (KD) versus negative control (siCtrl) (Figures S7H,I and S9A,B); siCD44‐1 was selected for formal experiments. FCM revealed that in CD4^+^ T cells, siCD44 markedly reduced CD44 surface expression, decreased IFN‐γ production, and increased TCF1 expression (all *p* < 0.05; Figures S7J,K and S8K). In CD8^+^ T cells, siCD44 also reduced CD44, decreased GZMB and IFN‐γ, along with increased PD‐1 expression (all *p* < 0.05; Figure S7L–O); TOX (Figure S7P, *p* = 0.072) and TCF1 (Figure S8Q, *p* = 0.077) trended upward. However, the low proportion of TCF1^+^ cells (< 1%) may reflect detection limits (Table S16).

These data demonstrate that CD44 contributes to maintaining an immunologically active T‐cell phenotype, and its downregulation may promote T‐cell exhaustion. The t‐SNE distributions and density plots for each biomarker are shown in Figure S8I–Q.

### 2.6 Increased Ruffle Formation in Tumor and Immune Cells of Platinum‑Resistant Group

To explore how lipids from ascites might be internalized, we focused on membrane ruffles, especially “dorsal ruffles” that initiate macropinocytosis.

**FIGURE 5:**
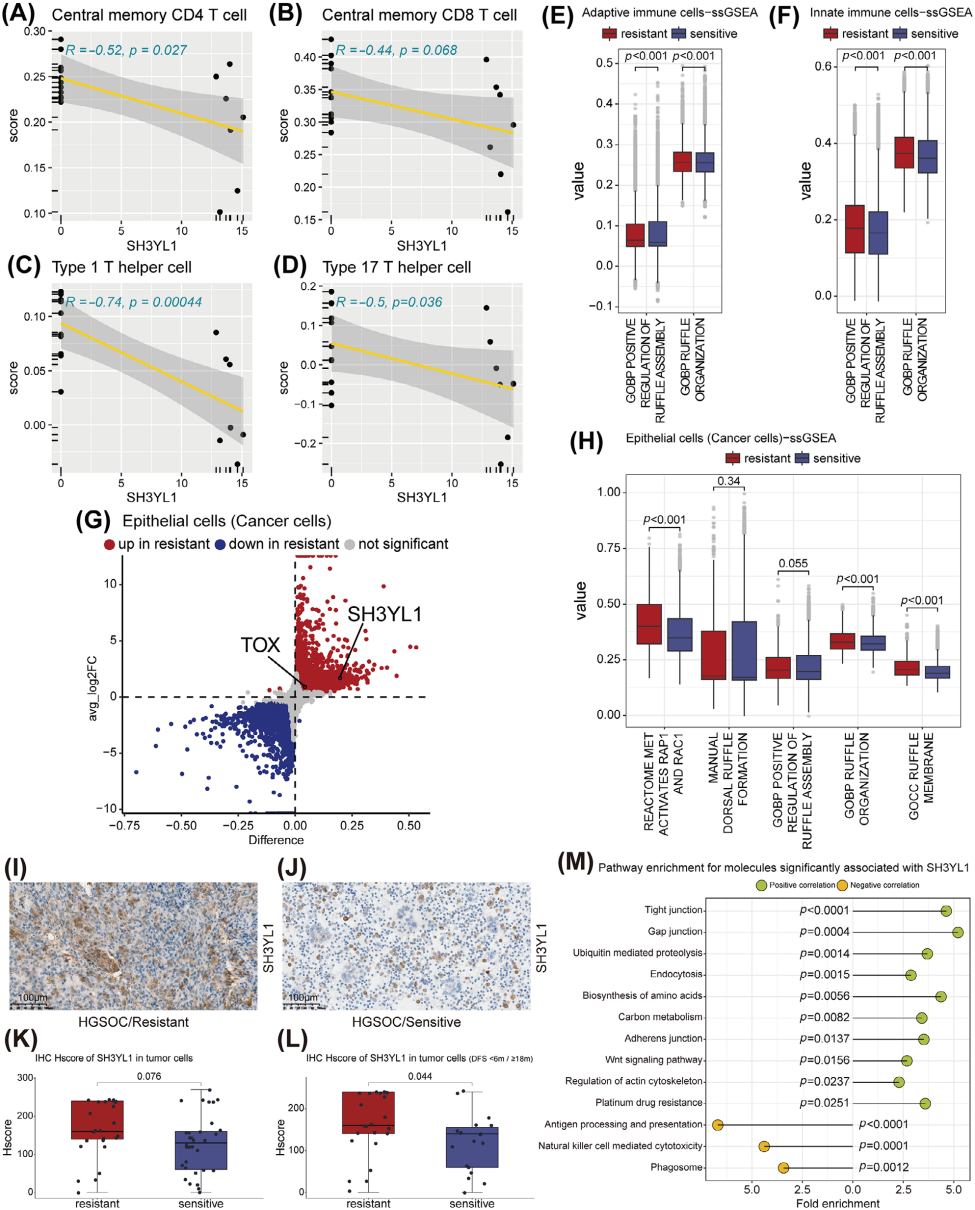
The phenotype of immune cells and tumor cells in the resistant group may be partially implicated in SH3YL1‐mediated ruffle formation activity. (A–D) Correlation between SH3YL1 expression and the score of different T‐cell subtypes: central memory CD4+ T cells (A), central memory CD8+ T cells (B), Type 1 T helper cell (C), and Type 17 T helper cell (D) (Spearman correlation). (E, F) Box‐and‐whisker plots comparing scores of pathways related to ruffle formation between resistant and sensitive groups after ssGSEA annotation of a subset of adaptive immune cells (E) and innate immune cells (F) in ascites single‐cell transcriptome data. (G) Volcano plot demonstrating differential expression of SH3YL1 and TOX between two groups in the epithelial cells (cancer cells) cluster (significant difference criteria: adjusted *p* < 0.05, |log2FC| > 0.5). (H) Box‐and‐whisker plot comparing scores of pathways related to ruffle formation between two groups after ssGSEA annotation of epithelial cells (cancer cells) cluster. (I, J) Representative IHC images of SH3YL1‐staining in extended ascites‐derived cell samples from resistant (I) and sensitive patients (J). Scale bar = 100 µm. (K, L) Box‐and‐whisker plot showing the difference in SH3YL1 IHC H score between resistant and sensitive groups, under two different clinical sensitivity criteria: Keep disease stable (no progression) within 12 months after the last treatment course (K, *n* = 25 for resistant and *n* = 32 for sensitive, two‐sided Wilcoxon rank‐sum tests) and within 18 months after the last treatment course (L, *n* = 25 for resistant and *n* = 19 for sensitive, two‐sided Wilcoxon rank‐sum tests). (M) Molecules significantly positively/negatively correlated with SH3YL1 expression were calculated for KEGG pathway enrichment. For A–D and M, all *n* = 18 HGSOC ascites samples were analyzed (*n* = 8 for resistant and *n* = 10 for sensitive). For G‐H, all *n* = 10 ovarian cancer ascites samples were analyzed (*n* = 3 for resistant and *n* = 7 for sensitive).

SH3YL1 contains a lipid‑binding “SH3YL1, Ysc84p/Lsb4p, Lsb3p, and plant FYVE proteins” (SYLF) domain that binds membrane lipids, and a Src homology 3 (SH3) domain that recruits phosphatases such as src‐homology 2 containing inositol‐5‐phosphatase 2 (SHIP2, alters membrane lipid composition) [19] and actin regulators like dynamin‐2 [25], driving actin remodeling to form dorsal ruffles [19] that move like water waves and close after contacting to internalize extracellular fluid and initiate macropinocytosis [22, 23, 29]. SH3YL1 expression inversely correlated with enrichment scores of immunoreactive T cells: central memory CD4 T cell (*R* = −0.52, *p* = 0.027), central memory CD8 T cell (*R* = −0.44, *p* = 0.068), Type 17 T helper cell (*R* = −0.74, *p* = 0.00044), and Type 1 T helper cell (*R* = −0.5, *p* = 0.036) (Figure 5A–D), suggesting dorsal ruffles formation may impede immune cell presence in ascites.

Converting single‐cell RNA expression of adaptive immune cells (B cells, CD4^+^ T cells, CD8^+^ T cells) and innate immune cells (NK, monocyte, macrophage, DC) to pathway enrichment scores showed significantly elevated enrichment for “GOBP positive regulation of ruffle assembly” and “GOBP ruffle organization” in the resistant group (all *p* < 0.05; Figure 5E,F), indicating active ruffles formation. DCs, macrophages, B cells, and T cells have been found to undergo active macropinocytosis [54]. This nonspecific endocytosis pathway may partially explain how immune cells ingest lipids from lipid‐rich ascites.

Meanwhile, tumor cells can be induced by their surroundings to undergo active macropinocytosis [55]. Differential analysis revealed that in the epithelial cells (cancer cells) cluster, SH3YL1 expression trends consistent with proteomic data, and increased TOX expression consistent with prior multiplex IF results (Figure 5G). Converting RNA expression of epithelial cells (cancer cells) clusters into pathway enrichment scores (Figure 5H), the resistant group showed higher enrichment for “REACTOME MET activates RAP1 and RAC1” (*p* < 0.001), “MANUAL dorsal ruffle formation” (*p* = 0.340), “GOBP positive regulation of ruffle assembly” (*p* = 0.055),“GOBP ruffle organization” (*p *< 0.001), and “GOCC ruffle membrane” (*p *< 0.001), suggests adequate structural basis for macropinocytosis occurrence.

IHC of 57 ascitic cell‐pellet FFPE samples found a trend toward higher SH3YL1 expression in the resistant group (*p* = 0.076; Figure 5I–K; Table S10) that reached significance when redefining “sensitive” as progression‐free survival > 18 months (*p* = 0.044; Figure 5L).

Selecting the 500 proteins most correlated with SH3YL1 and performing KEGG enrichment. Positively correlated proteins were enriched in endocytosis, “regulation of actin cytoskeleton”, “carbon metabolism” and “platinum drug resistance”, while negatively correlated proteins were enriched in “phagosome,” “NK cell mediated cytotoxicity,” “antigen processing and presentation” (all *p* < 0.05; Figure 5M; Tables S17 and S18), suggesting SH3YL1 drives macropinocytosis‐fueled metabolism for platinum resistance, whereas lipid accumulation in immune cells impairs activation.

Given the active lipid catabolism in the resistant group, we propose that SH3YL1 upregulation promotes membrane ruffling to drive the non‐specific uptake of ascitic fluid—including its constituent lipids—via macropinocytosis; these entrapped lipids are subsequently catabolized to support tumor survival and evasion of platinum‐induced damage.

### 2.7 SH3YL1‐Driven Macropinocytosis Facilitates Lipid Uptake and Cisplatin Resistance in Ovarian Cancer

**FIGURE 6:**
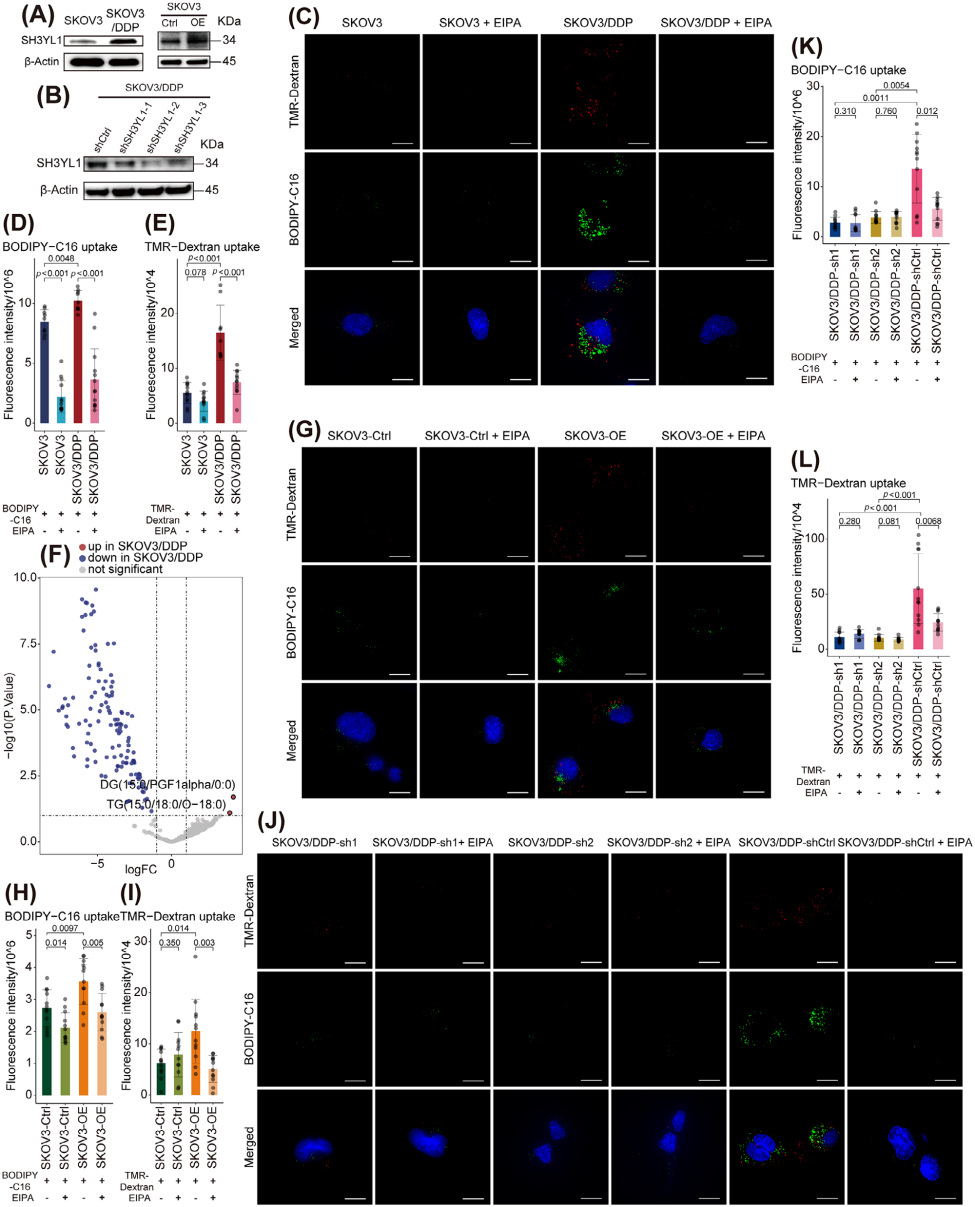
Investigation of SH3YL1 and its mediated macropinocytosis in lipid uptake and development of platinum‐resistance phenotypes in ovarian cancer. (A) WB showing differential SH3YL1 expression in platinum‐resistant and sensitive ovarian cancer cell lines and confirmation of SH3YL1 overexpression in SKOV3 cell lines. (B) WB confirming successful SH3YL1 KD in platinum‐resistant SKOV3/DDP cell lines. (C) Representative fluorescence microscopy images (single‐channel and merged) showing uptake of fluorescent tracer in SKOV3 cells or SKOV3/DDP cells, with or without pretreatment by the macropinocytosis inhibitor 5‑(N‑ethyl‑N‑isopropyl) ‑Amiloride (EIPA). Scale bar = 20 µm. (D, E) Quantification of fluorescence intensity for lipid uptake (using BODIPY‑C6; D, *n* = 9, two‐sided Wilcoxon rank‐sum tests, *p*‐values adjusted by the BH method) and macropinocytosis (using TMR‑Dextran; E, *n* = 12, two‐sided Wilcoxon rank‐sum tests, *p*‐values adjusted by BH method) in SKOV3 and SKOV3/DDP cells under indicated conditions. (F) Volcano plot demonstrating differential metabolites identified at the single‐cell level between resistant and sensitive group (screening criteria: *p* < 0.1, |logFC| > 1). (G) Representative fluorescence microscopy images (single‐channel and merged) showing uptake of fluorescent tracer in SKOV3‐SH3YL1‐OE cells (via lentiviral transduction) or vector control, with or without pretreatment by EIPA. Scale bar = 20 µm. (H, I) Quantification of fluorescence intensity for lipid uptake (using BODIPY‑C6; H, *n* = 12, two‐tailed *t*‐test, *p*‐values adjusted by the BH method) and macropinocytosis (using TMR‑Dextran; I, *n* = 12, two‐sided Wilcoxon rank‐sum tests, *p*‐values adjusted by BH method) comparing SKOV3‐SH3YL1‐OE versus control under indicated conditions. (J) Representative fluorescence microscopy images (single‐channel and merged) showing uptake of fluorescent tracer in SKOV3/DDP‐SH3YL1‐KD cells (via lentiviral transduction) or short hairpin RNA (shRNA) control, with or without pretreatment by EIPA. Scale bar = 20 µm. (K, L) Quantification of fluorescence intensity for lipid uptake (using BODIPY‑C6; K, *n* = 12, two‐sided Wilcoxon rank‐sum tests, *p*‐values adjusted by the BH method) and macropinocytosis (using TMR‑Dextran; L, *n* = 12, two‐sided Wilcoxon rank‐sum tests, *p*‐values adjusted by BH method) in SKOV3/DDP‐SH3YL1‐KD versus control under indicated conditions. Experiments were repeated at least three times.

We retrieved transcriptomic datasets from Gene Expression Omnibus (GEO) for platinum‐sensitive and ‐resistant ovarian cancer cell lines and verified the differential expression of SH3YL1 in SKOV3 cells (Figure S10A). The platinum‐sensitive SKOV3 line was subsequently used for experimental verification. Gradient cisplatin induction generated the resistant SKOV3/DDP line. Cell Counting Kit 8 (CCK‐8) assays demonstrated that SKOV3/DDP exhibited a significantly elevated IC_50_ to cisplatin relative to SKOV3 (P<0.001; Figure S10B; Table S19). WB confirmed higher SH3YL1 expression in SKOV3/DDP (Figures 6A and S9C; Table S15).

To compare the activity of macropinocytosis and lipid uptake, SKOV3 and SKOV3/DDP were starved and then incubated with the macropinocytosis tracer tetramethylrhodamine‐Dextran (TMR‐Dextran, red fluorescence; internalized solely via macropinocytosis due to high‐MW) and the lipid tracer BODIPY FL C16 (BODIPY‐C16, green fluorescence; fatty acid analog), with or without the macropinocytosis inhibitor 5‐(N‐ethyl‐N‐isopropyl)‐amiloride (EIPA). Confocal imaging showed significantly stronger TMR‐Dextran and BODIPY‐C16 fluorescence in SKOV3/DDP than in SKOV3, while EIPA substantially reduced both in SKOV3/DDP (Figure 6C). In parallel, quantitative microplate readings showed SKOV3/DDP incorporated BODIPY‐C16 significantly more efficiently than SKOV3 (*p* = 0.0048), while EIPA suppressed BODIPY‐C16 uptake in both SKOV3 (*p* < 0.001) and SKOV3/DDP (*p *< 0.001) (Figure 6D; Table S20). Similarly, SKOV3/DDP exhibited greater TMR‐Dextran uptake than SKOV3 (*p* < 0.001), with EIPA markedly reducing uptake in SKOV3/DDP (*p* < 0.001), whereas reduction in SKOV3 did not reach statistical significance (*p* = 0.078) (Figure 6E; Table S20). These results indicate enhanced macropinocytosis as a primary route for lipid acquisition in platinum‐resistant cells.

Single‐cell sorting enabled individual cell metabolite identification for SKOV3 (*n* = 8) and SKOV3/DDP (*n* = 31) (Table S21). Differential analysis revealed that SKOV3/DDP contained more diacylglycerol (DG) and TG (Figure 6F), suggesting esterification of fatty acids as reserves.

**FIGURE 7:**
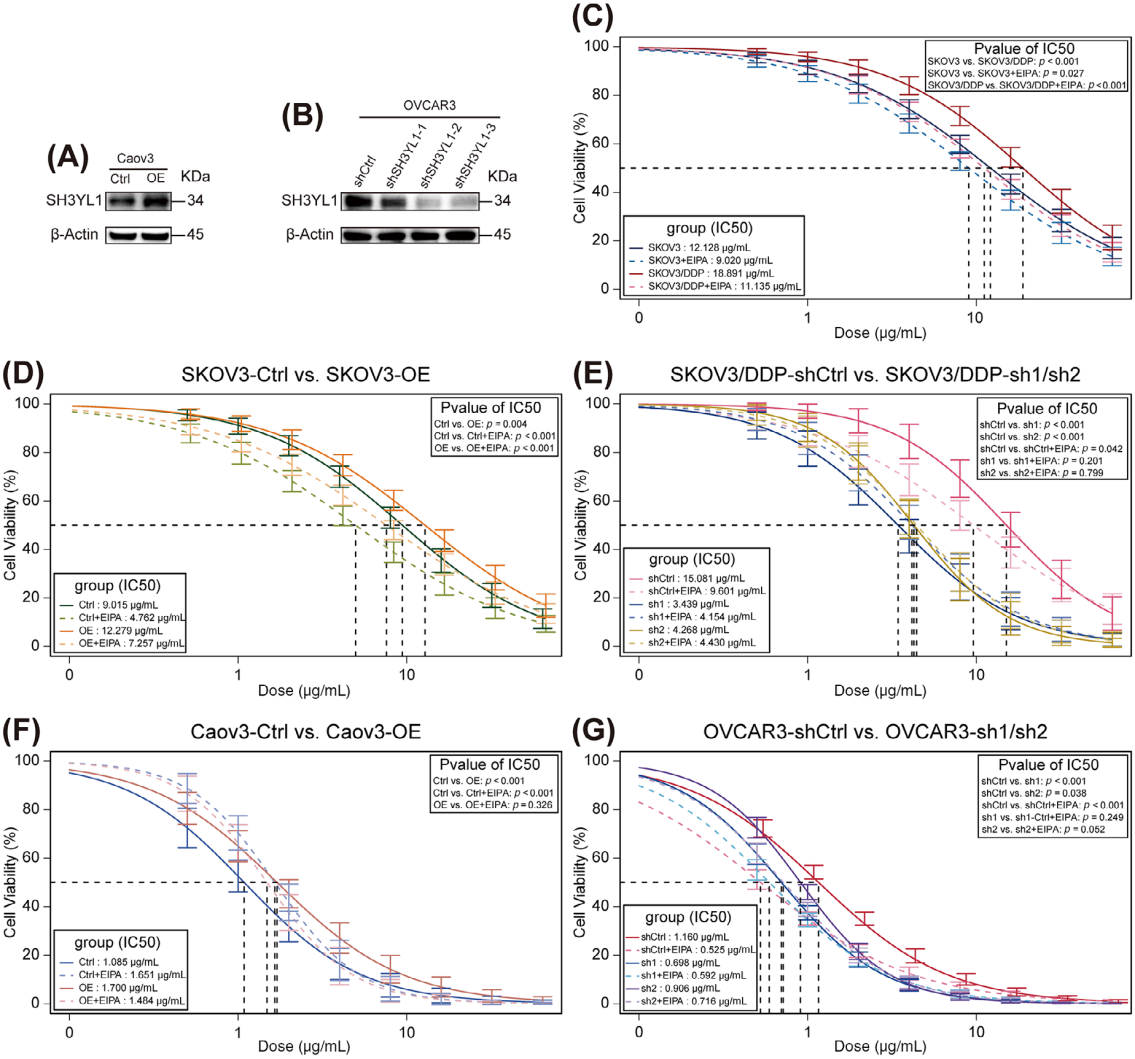
Effects of genetic manipulation targeting SH3YL1 and macropinocytosis inhibition on cisplatin sensitivity in multiple ovarian cancer cell lines. (A) WB confirming successful SH3YL OE in Caov3 cell lines. (B) WB confirming successful SH3YL1 KD in OVCAR3 cell lines. (C) CCK8 assays evaluating the effect of macropinocytosis inhibitor EIPA on cisplatin sensitivity in SKOV3 and SKOV3/DDP cells. (D) CCK8 assays evaluating the effect of SH3YL1 OE and EIPA on cisplatin sensitivity in SKOV3. (E) CCK8 assays evaluating the effect of SH3YL1 KD and EIPA on cisplatin sensitivity in SKOV3/DDP. (F) CCK8 assays evaluating the effect of SH3YL1 OE and EIPA on cisplatin sensitivity in Caov3. (G) CCK8 assays evaluating the effect of SH3YL1 KD and EIPA on cisplatin sensitivity in OVCAR3. Experiments were repeated at least three times.

To further validate that active macropinocytosis characterizes platinum‐resistant cells, four conditions were tested: SKOV3 or SKOV3/DDP in standard culture medium with or without EIPA. CCK‐8 assays showed that EIPA partially restored cisplatin sensitivity in both SKOV3 and SKOV3/DD (all *p* < 0.05; Figure 7C, Table S19), underscoring the importance of macropinocytosis in platinum resistance.

Aiming to clarify whether SH3YL1 facilitates lipid uptake via macropinocytosis, we selected two additional ovarian cancer cell lines, Caov3 and OVCAR3, alongside SKOV3 and SKOV3/DDP. WB showed SKOV3 and OVCAR3 have comparable baseline SH3YL1 expression, both significantly higher than Caov3 (Figure S9D; Table S15). Consequently, SH3YL1 overexpression (OE) was performed in SKOV3 and Caov3, as well as SH3YL1 KD in SKOV3/DDP and OVCAR3 via lentiviral transduction (Table S24). WB confirmed robust SH3YL1 OE in SKOV3 and Caov3 (Figures 6A and 7A; Figure S9E,G; Table S15). For KD lines, lentiviruses derived from three short hairpin RNAs (shRNAs), in SKOV3/DDP, shSH3YL1‐1 (sh1) and shSH3YL1‐2 (sh2) achieved substantial KD versus shCtrl, whereas shSH3YL1‐3 (sh3) had minimal effects (Figures 6B and S9F). In OVCAR3, all three shRNAs produced significant KD (Figures 7B and S9H; Table S15). Lines with effective KD (sh1 and sh2) were selected for subsequent assays.

Using these modified lines, we repeated fluorescence tracer and CCK‐8 assays. In SKOV3, SH3YL1‐OE cells (SKOV3‐OE) internalized significantly more BODIPY‐C16 (*p* = 0.0097) and TMR‐Dextran (*p* = 0.014) compared with controls (SKOV3‐Ctrl). EIPA significantly reduced BODIPY‐C16 uptake in both SKOV3‐Ctrl (*p* = 0.014) and SKOV3‐OE (*p* = 0.005), and decreased TMR‐Dextran uptake in SKOV3‐OE (*p* = 0.003). But SKOV3‐Ctrl exhibited low macropinocytosis levels, rendering non‐significant EIPA effect (*p* = 0.350) (Figure 6G–I; Table S20). Caov3‐OE similarly showed enhanced uptake of both tracers (BODIPY‐C16, *p* = 0.0058; TMR‐Dextran, *p* < 0.001) with EIPA significantly inhibiting uptake (*p* < 0.001). However, in Caov3‐Ctrl, basal lipid uptake and macropinocytosis were low, reflected by negligible EIPA effect (BODIPY‐C16, *p* = 0.620; TMR‐Dextran, *p* = 0.590) (Figure S10C–E; Table S20).

CCK8 assays revealed that SH3YL1 OE elevated IC_50_ for cisplatin in both SKOV3 (*p* = 0.004) and Caov3 (*p* < 0.001), indicating greater resistance. In SKOV3‐Ctrl (*p* < 0.001) and SKOV3‐OE (*p* < 0.001), EIPA partially restored cisplatin sensitivity (Figure 7D; Table S19). However, in Caov3, EIPA did not significantly reduce IC_50_ in Caov3‐OE (*p* = 0.326) and unexpectedly increased IC_50_ in Caov3‐Ctrl (*p* < 0.001) (Figure 7F; Table S19), possibly due to Caov3's inherently high cisplatin sensitivity, as inhibition of macropinocytosis reduces cisplatin entry.

SH3YL1 KD significantly impaired BODIPY‐C16 uptake in both SKOV3/DDP and OVCAR3 (all *p* < 0.05), and similarly reduced TMR‐Dextran uptake (all *p* < 0.05). After KD, EIPA no longer significantly inhibited tracer uptake in these lines due to the impairment of lipid uptake and macropinocytosis (BODIPY‐C16, all *p* > 0.05; TMR‐Dextran, all *p* > 0.05) (Figures 6J–L and S10F–H; Table S20).

CCK8 assays revealed that SH3YL1 KD significantly reduced IC_50_ for cisplatin in SKOV3/DDP and OVCAR3 (all *p* < 0.05), indicating restored sensitivity. Although EIPA partially restored cisplatin sensitivity in SKOV3/DDP‐shCtrl and OVCAR3‐shCtrl (all *p* < 0.05), after SH3YL1 KD, EIPA no longer had a significant effect (all *p* > 0.05) (Figure 7E,G; Table S19). Hence, we speculate that greater platinum resistance correlates with increased dependency on macropinocytosis.

These experiments indicate that SH3YL1 is a core mediator of macropinocytosis. Its up‑regulation enhances macropinocytosis activity, enabling greater lipid uptake from the environment and thereby promoting platinum resistance in ovarian cancer.

### 2.8 Predictive Performance of a Logistic‐Regression Model Using Ascites‐Derived Signatures in Ovarian Cancer

We integrated the proteomic profiles with pathway annotations to synthesize our findings, defining representative features across four modules: (1) immune microenvironment (CD44; “WP complement system”; “Hoegerkorp CD44 targets direct DN” (high expression inversely correlates with CD44 downstream signaling [56]); “TCR signaling pathway”); (2) lipid metabolism (MLYCD; “KEGG fatty acid metabolism”; “GOBP fatty acid catabolic”); (3) macropinocytosis (SH3YL1; “Liu ovarian cancer tumors and xenografts kinases DN” (comprising 34 druggable kinases with therapeutic potential [57]); “REACTOME MET activates RAP1 and RAC1”; “MANUAL dorsal ruffle formation”; “GOBP positive regulation of ruffle assembly”), and (4) DNA damage repair (“KEGG mismatch repair”; “WP DNA repair pathways full network”). These data were normalized to construct a discovery set (Table S22).

We then utilized proteomic data from the Clinical Proteomic Tumor Analysis Consortium (CPTAC)—primary foci FFPE samples of HGSOC patients (PDC000360, 75 post‐platinum samples: 55 platinum‐sensitive, 20 platinum‐resistant) [58]. Platinum‐sensitivity was defined as no progression within 2 years after last therapy, and resistance as progression within 6 months. The CPTAC data were annotated with the same gene‑set collections to obtain expression of our selected features, forming a validation set (Table S22).

We predicted cisplatin IC_50_ for all discovery set and validation samples. Resistant features (SH3YL1, MLYCD, “REACTOME MET activates RAP1 and RAC1,” “MANUAL dorsal ruffle formation,” “GOBP positive regulation of ruffle assembly,” “KEGG fatty acid metabolism,” “GOBP fatty acid catabolic,” “Hoegerkorp CD44 targets direct DN,” “WP DNA repair pathways full network”) and sensitive features (CD44, “WP complement system,” “KEGG mismatch repair,” “TCR signaling pathway,” “Liu ovarian cancer tumors and xenografts kinases DN”) were used as annotation sets for ssGSEA. In both sets, predicted cisplatin IC_50_ of correlated positively with resistant feature score (Discovery: *R* = 0.66, *p* = 0.0036; Validation: *R* = 0.54, *p* < 0.001), and negatively with sensitive score (Discovery: *R* = −0.66, *p* = 0.0037; Validation: *R* = −0.55, *p *< 0.001), supporting the credibility of the selected features (Figure S11A,B).

**FIGURE 8:**
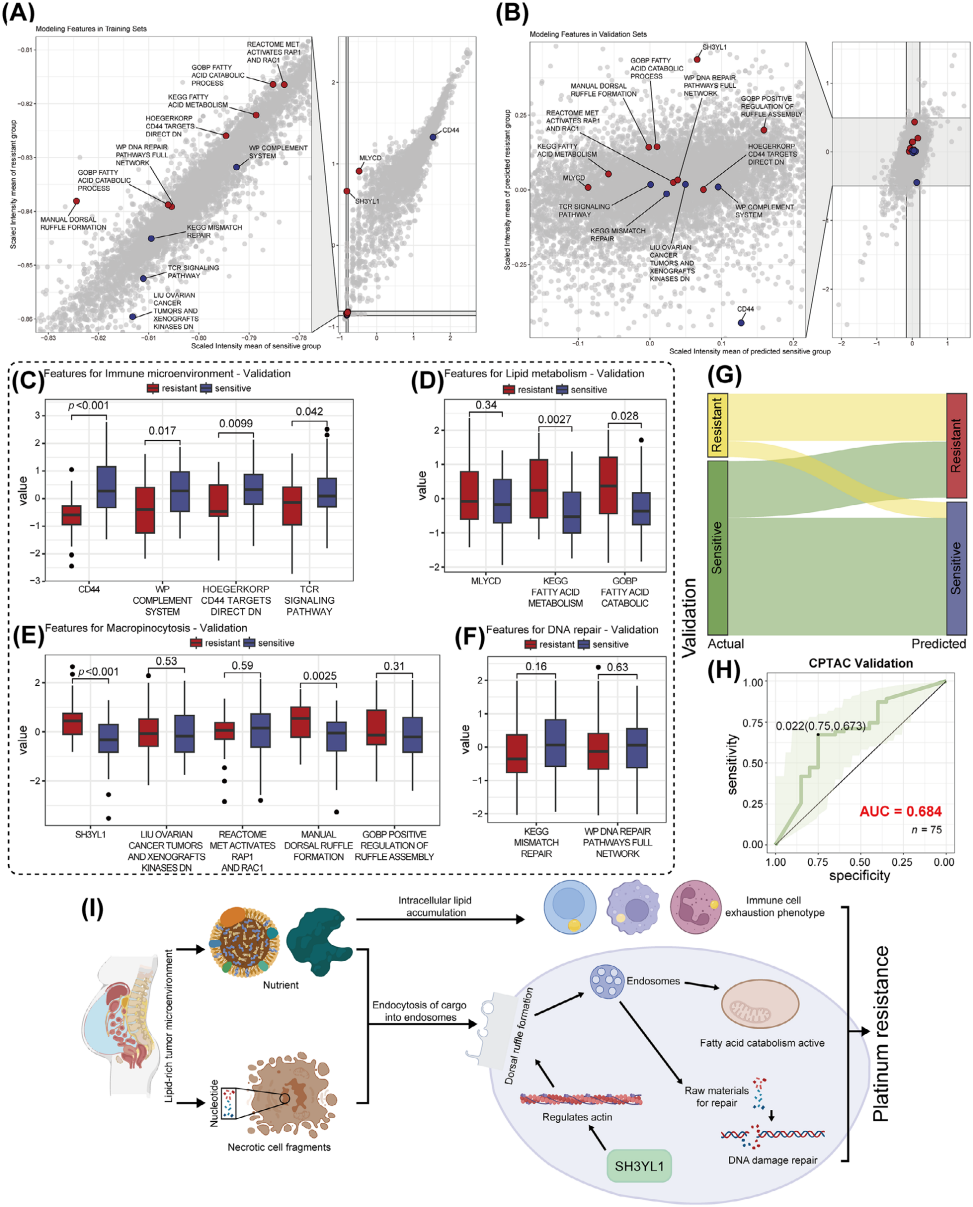
A logistic‐regression model based on mechanistic hypotheses predicts platinum sensitivity of ovarian cancer tissues with relatively good accuracy. (A, B) Scatterplot demonstrating the expression levels of the characteristic proteins and pathways used for modeling in the discovery set (A, *n* = 18 HGSOC ascites samples, actual platinum‑sensitivity/resistance) and validation set (B, *n* = 75 HGSOC tissue samples, model‑predicted sensitivity/resistance). (C–F) Box‐and‐whisker plots comparing model‐predicted platinum‐resistant versus sensitive subgroups in the validation set in terms of immune microenvironment (C), lipid metabolism (D), micropinocytosis (E), and DNA repair (F). (G) Sankey diagram comparing model‐predicted platinum sensitivity with actual platinum response in the validation set. (H) ROC curves showing performance of the logistic regression model in predicting platinum sensitivity, evaluated on an external validation set. (I) Schematic hypothesis diagram summarizing the proposed platinum‐resistance mechanism. For C–F, all *n* = 18 HGSOC ascites samples were analyzed (*n* = 8 for resistant and *n* = 10 for sensitive, two‐sided Wilcoxon rank‐sum tests). The schematic illustration in Panel I was created by the authors using Adobe Illustrator.

Subsequently, we constructed a logistic regression model (Table S22) using platinum sensitivity/resistance in the discovery set as the dependent variable, characteristic features as independent variables. Applying this model to the validation set produced predicted sensitivity/resistance classifications. Mean expression values for each feature in sensitive and resistant groups were plotted: resistance‑ (red) and sensitivity‑related (blue) features separated clearly in the discovery set (Figure 8A), and although overlap occurred in the validation set, groups remain largely distinguishable (Figure 8B).

Differential expression analyses showed three characteristic molecules with consistent trends in both sets: in the resistant group, CD44 was downregulated (Discovery: *p* = 0.034; Validation: *p* < 0.001), SH3YL1 (Discovery: *p* = 0.00049; Validation: P<0.001) and MLYCD (Discovery: *p* = 0.00069; Validation: *p* = 0.34) were upregulated.

For immune features (Figures 8C and S11C), “WP complement system” (Discovery: *p* = 0.15; Validation: *p* = 0.017) and “TCR signaling pathway” (Discovery: *p* = 0.043; Validation: *p* = 0.042) were significantly upregulated in the sensitive group in both datasets. “Hoegerkorp CD44 targets direct DN” was upregulated in the resistant group in the discovery set (*p* = 0.27) but in sensitive group in the validation set (*p* = 0.0099), reflecting CD44's context‐specific tumor‐promoting role as a cancer stem cell marker in platinum‐resistant HGSOC after therapy [17].

Lipid metabolism‐related features—“KEGG fatty acid metabolism” (Discovery: *p* = 0.043; Validation: *p* = 0.0027) and “GOBP fatty acid catabolic” (Discovery: *p* = 0.034; Validation: *p* = 0.028) were upregulated in resistant cases in both sets, highlighting the importance of fatty acid catabolism (Figures 8D and S11D).

Macropinocytosis‐related features (Figures 8E and S11E) largely trended similarly: “MANUAL dorsal ruffle formation” (Discovery: *p* = 0.068; Validation: *p* = 0.0025) and “GOBP positive regulation of ruffle assembly” (Discovery: *p* = 0.12; Validation: *p* = 0.31) were higher in the resistant group, indicating structural support for macropinocytosis. However, upstream signaling “Liu ovarian cancer tumors and xenografts kinases DN” (Discovery: *p* = 0.016; Validation: p = 0.53) and “REACTOME MET activates RAP1 and RAC1” (Discovery: *p* = 0.021; Validation: *p* = 0.59) [27, 59] were higher in resistant only in discovery but not in validation sets, suggesting reduced activation in dormant, post‐therapy tumor cells.

DNA repair features (Figures 8F and S11F)—“KEGG mismatch repair” (Discovery: *p* = 0.7; Validation: *p* = 0.16) and “WP DNA repair pathways full network” (Discovery: *p* = 0.57; Validation: *p* = 0.63) showed no significant differences, median trended higher in resistant tumors in the discovery set but in sensitive tumors in the validation set. Potentially, downregulated repair pathways may prevent apoptosis [3, 60], allowing resistant tumors to adaptively evade platinum‐induced cell death.

In the validation set, 15 platinum‐resistant and 37 sensitive patients were correctly classified (AUC = 0.684, sensitivity = 0.75, specificity = 0.673; Figure 8G,H; Table S23). These results suggest that platinum‑resistant features identified in pretreatment ascites are reflected in tumor tissues, supporting the prediction of clinical platinum sensitivity based on ascites content.

## 3 Discussion

For ovarian cancer patients with limited treatment options, resistance to platinum‐based chemotherapy after initial benefit occurs in the vast majority [61]. Malignant ascites, a common accompanying symptom [62], serves as an environmental driver of platinum resistance that warrants attention. By synthesizing our ascites proteomics with public tissue proteomic and ascites single‐cell data, we provide a multi‐dimensional profile to identify the molecular and microenvironmental drivers of platinum resistance.

We observed that patients with lipid‐rich ascites tended to develop resistance to subsequent platinum‐based chemotherapy. Pathway enrichment showed that tumor cells in resistant patients relied more on non‐selective micropinocytosis for nutrient uptake, whereas sensitive tumors favored selective receptor‐mediated endocytosis and transporter proteins [63, 64]. Experimental validation demonstrates that SH3YL1 OE enhanced macropinocytosis activity, facilitating extracellular lipid uptake and exacerbating platinum‐resistant phenotypes; while SH3YL1 KD or pharmacological inhibition of macropinocytosis partially restored cisplatin sensitivity. Unlike normal growth‐factor‐dependent cells, tumor cells appear to possess constitutive macropinocytosis capacity—akin to phagocytes in an “idling” state [54]—enabling continuous scavenging of extracellular nutrients [23]; in lipid‐rich ascites, this provides an abundant and sustained nutrient reservoir, reducing dependence on de novo fatty acid synthesis. High expression of MLYCD, which relieves the inhibitor of carnitine O‐palmitoyltransferase 1 (CPT‐1) to promote fatty acid oxidation [20, 65], alongside upregulated fatty acid catabolic pathways suggest ingested lipids are metabolized for energy in resistant tumor cells.

Lipid‐rich environments also drive lipid accumulation in immune cells [46, 47, 66] and induce an exhaustion phenotype. Lipids, particularly oxidized [67] or peroxidized polyunsaturated fatty acids [68], cause endoplasmic reticulum stress in immune effectors, leading to exhaustion of innate immunity and impaired activation of adaptive immunity [46], resulting in an exhausted immune microenvironment with poor intercellular immunoreactivity, a phenomenon we reproduced in single‐cell data analysis, in clinical samples, and in vitro. Furthermore, CD44 KD revealed that this molecule helps maintain an immune‐active T cell phenotype, and its reduced expression may indicate the degree of exhaustion.

We proposed a hypothetical network in which platinum‐resistance mechanisms rely on macropinocytosis, enhanced fatty‐acid catabolism, and immune exhaustion driven by lipid‐rich ascites (Figure 8I): macropinocytosis and robust intracellular fatty acid oxidation provide tumor cells with ample energy to fuel proliferation, migration, and DNA damage repair. In addition, fatty acid oxidation generates nicotinamide adenine dinucleotide phosphate (NADPH) [69, 70] to support GSH production to inactivate platinum‐based drugs. Necrotic debris ingested by macropinocytosis in TME provides carbohydrates, proteins, lipids, and nucleotides for central carbon metabolism, membrane synthesis, and DNA repair, protecting tumors from platinum‐induced damage [55, 71]. Besides, apoptotic bodies provide oxidized phosphocholine‐containing phospholipids, a key source of oxidized lipids for immune cells [67]. Furthermore, macropinocytosis promotes immune evasion by internalizing surface antigens and antibody complexes [27], which, alongside lipid‐driven exhaustion [17], creates an immunosuppressive microenvironment. Logistic regression models using features according to the hypotheses exerted good predictive performance in HGSOC tissues, implying that predictive features are present in ascites before treatment. This suggests opportunities for early intervention, for example, by controlling blood lipid levels or combining platinum with inhibitors of endocytosis or fatty acid oxidation. Owing to feature interdependence, interventions must be comprehensive. Using reversible endocytosis inhibitors may temporarily block tumor nutrient uptake while preserving antigens for immune recognition [27].

Due to strict criteria, this study is limited by a small sample size. These preliminary findings require further validation to define the specific lipid species and macropinocytosis mechanisms driving platinum resistance across diverse clinical settings.

In summary, this multi‐dimensional study links lipid‐macropinocytosis axes to platinum resistance, offering novel therapeutic strategies and potential biomarkers to improve HGSOC clinical outcomes.

## 4 Materials and Methods

### 4.1 Clinical Samples

A total of 1583 ascites samples were collected from patients between June 2017 and November 2019. Among these, 436 were adenocarcinoma, and 330 were associated with gynecologic tumors. Fifty‐eight cases had complete treatment and follow‐up data; 43 met criteria for platinum sensitivity (progression‐free ≥ 12 months after the last treatment course) or resistance (progression < 6 months). Twenty‐two FFPE cell‐pellet samples with tumor cell content > 30% underwent proteomic MS identification, and 18 passed quality control. These 18 cases (Table S1) were pathologically diagnosed as HGSOC with ascites, and all received paclitaxel plus carboplatin chemotherapy. All specimens were obtained from the Cancer Hospital of the Chinese Academy of Medical Sciences with ethics approval.

### 4.2 Isolation and Functional Assays of Immune Cells

Peripheral blood CD4^+^ and CD8^+^ T cells were isolated from healthy donors by magnetic bead separation and activated in vitro. FCM (Figure S12) assessed surface markers and cytokine expression; multiplex IF was conducted on FFPE sections to evaluate immune‐tumor spatial relationships.

### 4.3 Cell Culture and Genetic Perturbation

Human ovarian cancer cell lines (SKOV3, Caov3, and OVCAR3) were maintained under standard conditions. A cisplatin‐resistant SKOV3/DDP line was generated by stepwise cisplatin exposure. T cell gene silencing was performed by siRNA transfection; SH3YL1 OE and KD were achieved via lentiviral delivery (Table S24). Stable or transient manipulation was verified by WB.

The details of all used reagents are shown in Table S24.

### 4.4 Fluorescent Tracer Assay and Imaging

For quantitative assay, ovarian cancer cells were plated in 96‐well black‐walled, clear‐bottom plates at 1.5 × 10^4^ cells/well. After overnight culture, cells were glucose‐starved ± 10 µM EIPA (Selleck) overnight. Thereafter, cells were incubated in serum‐free medium containing 2 µM BODIPY‐C16 (Invitrogen) or 2 mg/mL TMR‐Dextran (MW∼70 000, Invitrogen) ± EIPA, for 2 h. After aspiration and two washes with 0.1% BSA in PBS, extracellular fluorescence was quenched with 0.08% Trypan Blue, then intracellular fluorescence was measured using a microplate reader (PE Victor Nivo Alpha S). Wells without fluorescent tracers served as background controls.

For imaging, cells on glass coverslips were glucose‐starved ± 12.5 µM EIPA overnight. Cells were first incubated with 4 µM BODIPY‐C16 for 1 h, then with 4 µM BODIPY‐C16 plus 0.3 mg/mL TMR‐Dextran (±EIPA) for another hour. After washing with 0.5% BSA in PBS and quenching extracellular fluorescence with 0.08% Trypan Blue, cells were fixed in 4% paraformaldehyde and counterstained with DAPI (Abclonal). Confocal images were acquired with a laser‐scanning microscope (DELTAVISION OMXSR) using 405 nm (DAPI), 488 nm (BODIPY‐C16), and 561 nm (TMR‐Dextran) excitation.

### 4.5 Quantification and Statistical Analysis

Statistical analyses were performed using R. Pairwise comparisons used two‐sided Wilcoxon rank‐sum tests by default; when normality and homogeneity of variance were met, two‑tailed *t*‑tests were applied. For three or more groups, the Kruskal–Wallis *H* test was applied or one‑way ANOVA if normality and homogeneity of variance were satisfied. Multiple comparisons adjusted by the Benjamini–Hochberg (BH) method. Statistical significance was accepted at *p*‐value < 0.05 or a BH adjusted *p*‐value < 0.05 (*p‐value ≤ 0.05; **p‐value ≤ 0.01; ***p‐value ≤ 0.001, ns, not significant, *p*‐value > 0.05).

The processed protein expression profiles are summarized in Table S25. Software and R packages used in these analyses are summarized in Table S26.

### 4.6 Access to Method Details

Full experimental procedures, reagent details, and software parameters are provided in the Supplementary Materials.

## Author Contributions

R.Z. conceptualized the study, performed formal analysis, conducted investigations, curated data, visualized the results, and drafted, reviewed, and edited the manuscript. Y.C. conducted investigations, validated data, and reviewed and edited the manuscript. X.H. validated data, drafted the original manuscript, and performed reviewing and editing. X.D. developed the methodology and reviewed and edited the manuscript. B.M. developed the methodology and curated data. L.W. and A.C. developed the methodology. Z.W. provided resources. G.Q. and S.C. reviewed and edited the manuscript. Y.Z. developed the methodology and curated data. H.G. conceptualized the study, provided resources, managed project administration, acquired funding, and reviewed and edited the manuscript. T.X. conceptualized the study, provided resources, supervised the project, managed project administration and funding acquisition, and reviewed and edited the manuscript. All authors have read and approved the final manuscript.

## Ethics Statement

Ethical approval was obtained from the Ethics Committee of Cancer Hospital, Chinese Academy of Medical Sciences (25/659‐5605).

## Conflicts of Interest

The authors declare no conflicts of interest.

## Supporting information



Supporting File 1

## Data Availability

The mass spectrometry proteomics data generated in this study have been deposit‐ed to the ProteomeXchange Consortium (http://proteomecentral.proteomexchange.org)via the iProX partner repository (http://iprox.cn) [72, 73] with the dataset identifiers PXD044439 and IPX0006688000.
