# Supplementary material for: Active Macropinocytosis, Lipid Catabolism, and Exhausting Immune Microenvironment of Ascites Tumor Cells Are Involved in Resistance to Platinum‐Based Therapy in Patients With High‐Grade Serous Ovarian Cancer
Source: MedComm (2020). 2026 Mar 7;7(3):e70657. doi: 10.1002/mco2.70657 (PMC12966806; doi:10.1002/mco2.70657)
Supplement: Supplementary file 1 — Supporting File 1 [file MCO2-7-e70657-s001.pdf]

Active macropinocytosis, lipid catabolism, and exhausting immune microenvironment of ascites tumor cells are involved in resistance to platinum-based therapy in patients with high-grade serous ovarian cancer

Ruiqi Zheng[1, #] | Ying Cui[2, #] | Xun Hu[3, #] | Xin Dong[4, #] | Bo Meng[5] | Luhong Wen[6] | Anqi Chen[6] | Zijng Wang[7] | Guifen Qiang[7] | Shujun Cheng[1] | Yang Zhao[5, *] | Huiqin Guo[2, *] | Ting Xiao[1, *]

[1]State Key Laboratory of Molecular Oncology, Department of Etiology and Carcinogenesis, National Cancer Center/National Clinical Research Center for Cancer/Cancer Hospital, Chinese Academy of Medical Sciences and Peking Union Medical College, Beijing, 100021, China | [2]Department of Pathology, National Cancer Center/National Clinical Research Center for Cancer/Cancer Hospital, Chinese Academy of Medical Sciences and Peking Union Medical College, No. 17 Panjiayuan Nanli, Beijing, 100021, China | [3]Department of Imaging Diagnosis, National Cancer Center/National Clinical Research Center for Cancer/Cancer Hospital, Chinese Academy of Medical Sciences and Peking Union Medical College, No. 17 Panjiayuan Nanli, Beijing, 100021, China | [4]Department of Clinical Laboratory, National Cancer Center/National Clinical Research Center for Cancer/Cancer Hospital, Chinese Academy of Medical Sciences and Peking Union Medical College, No. 17 Panjiayuan Nanli, Beijing, 100021, China | [5]Technology Innovation Center of Mass Spectrometry for State Market Regulation, Center for Advanced Measurement Science, National Institute of Metrology, Beijing, 100029, China | [6]China Innovation Instrument Company Ltd, Ningbo, Zhejiang 315000, China | [7]Institute of Materia Medica, Chinese Academy of Medical Sciences and Peking Union Medical College and Beijing Key Laboratory of Drug Target and Screening Research, Beijing 100050, China

* Correspondence: Yang Zhao (zynellj@126.com) | Huiqin Guo (ghqin2006@163.com) | Ting Xiao (xiaot@cicams.ac.cn)

[#] These authors contributed equally to this work.

Abstract

Platinum resistance remains a clinical challenge in ovarian cancer. Ascites represents an important mediator and a unique tumor microenvironment (TME) for invasion and metastasis. This study performed high-resolution mass spectrometry (MS) on pre-chemotherapy ascites cells from ovarian cancer patients. Integrating proteomic profiling, clinical data, and single-cell analysis, revealed that platinum-resistant ascites displayed a distinct microenvironmental: the macropinocytosis-related protein Src homology 3 domain-containing YSC84-like 1 (SH3YL1) was up-regulated, whereas the immune-activation marker CD44 was down-regulated in resistant cases. Single-cell analyses and pathway enrichment indicated immune exhaustion in resistant ascites,

alongside enhanced macropinocytosis and lipid catabolism in tumor cells. Clinical data also showed that resistant ascites are lipid-rich, with immunofluorescence plus flow cytometry confirming its association with immune exhaustion. Cellular experiments confirmed that SH3YL1-mediated macropinocytosis promoted lipid uptake, and its inhibition partially restored cisplatin sensitivity. A combined model of immune exhaustion, macropinocytosis, and lipid catabolism suggests these ascites-associated features could somewhat predict the platinum sensitivity in ovarian cancer tissues. We therefore propose the hypothesis that, in a lipid-rich ascites microenvironment, immune exhaustion occurs while tumor cells activate macropinocytosis and lipid catabolism — forming a network of resistance mechanisms that may serve as potential predictive markers or intervention targets for platinum resistance.

KEYWORDS

ovarian cancer | platinum resistance | macropinocytosis | lipid metabolism | immune microenvironment

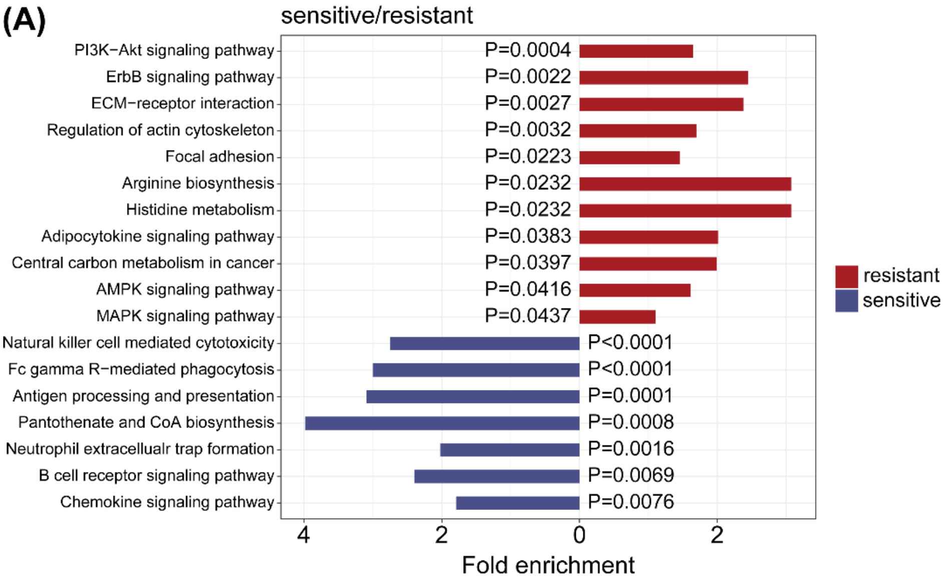

F I G U R E S1 | KEGG pathway enrichment results in HGSOC patients. (A) Bar plot demonstrating the pathways obtained by KEGG enrichment of up-regulated differential molecules in the resistant and sensitive groups, respectively (n = 8 for resistant and n = 10 for sensitive).

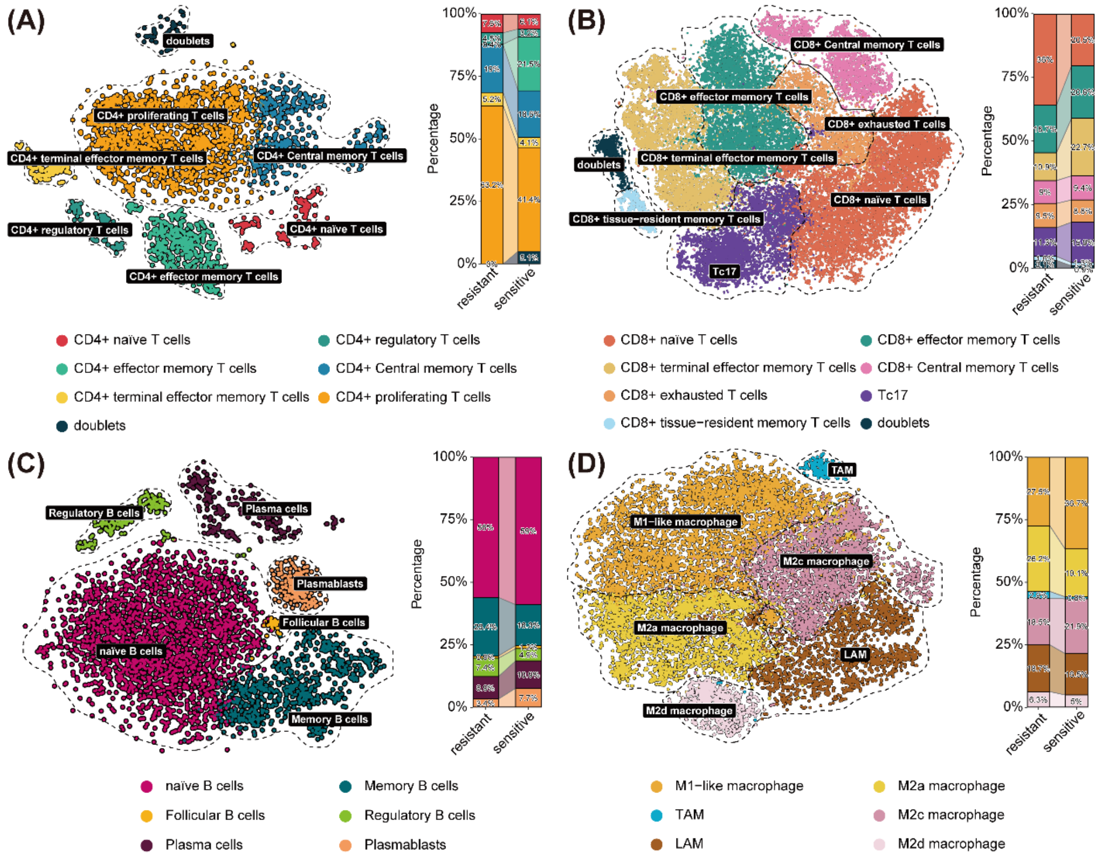

F I G U R E S2 | Functional subclusters of immune cells. (A-D) t-Distributed Stochastic Neighbor Embedding (tSNE) plots showing functional subcluster composition of $CD4^+$ T cells (A, 0.1 resolution), $CD8^+$ T cells (B, 0.8 resolution), B

cells (C, 0.05 resolution) and macrophages (D, 0.15 resolution), respectively. Each dot represents a single cell; colors denote different functional subclusters. And bar plots showing the proportion (%) of each functional subcluster within CD4^{+} T cells (A), CD8^{+} T cells (B), B cells (C) and macrophages (D), respectively. For A-D, all n = 10 ovarian cancer ascites samples were analyzed.

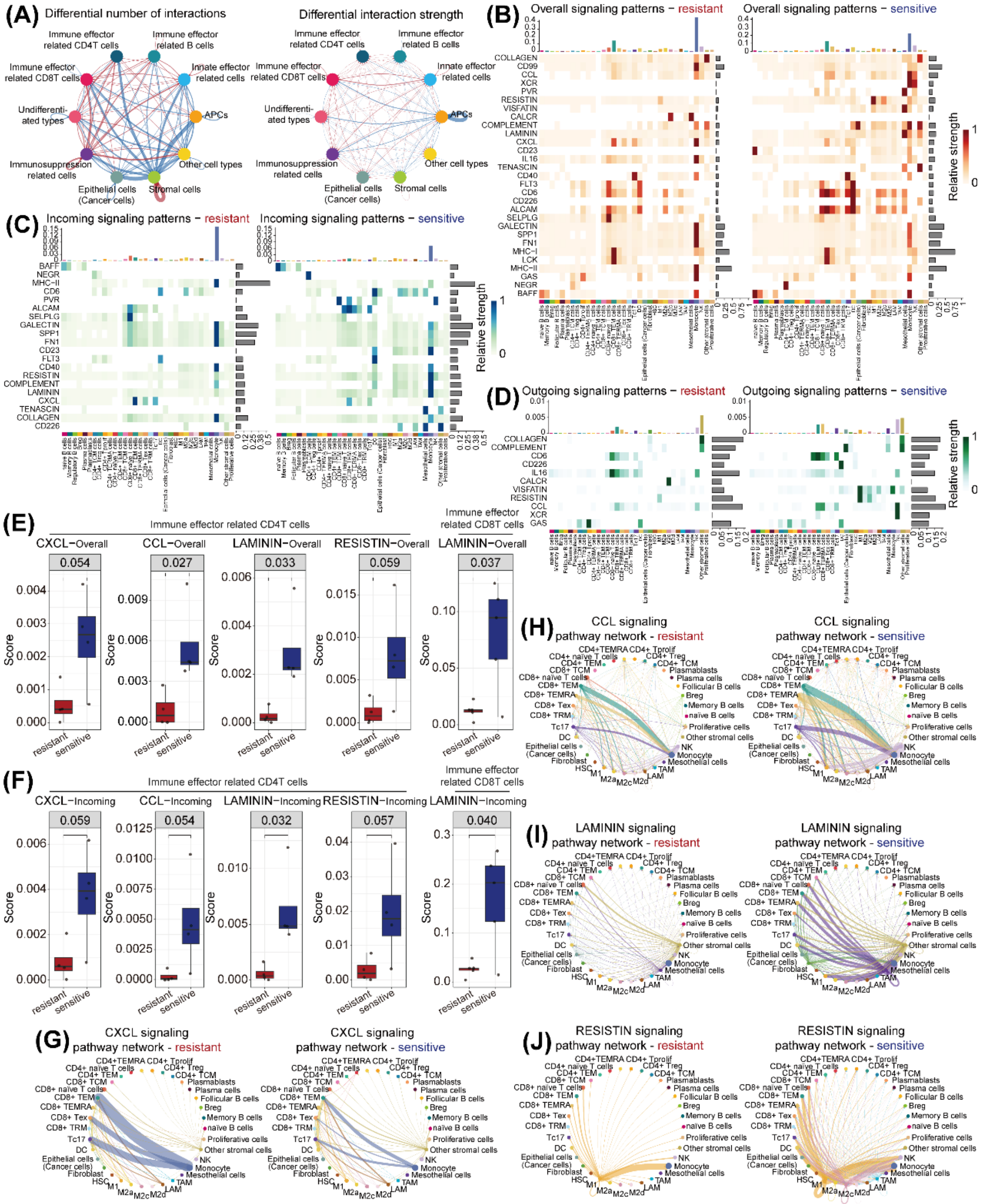

F I G U R E S3 | Altered intercellular communication networks in ascites between platinum-resistant and sensitive ovarian cancer patients. (A) Chord plot comparing aggregated cell-class communication between resistant and sensitive groups in the number and strength of communication between the major cell classes of ascites cells in n=10 ovarian cancer patients. Red connecting line representing stronger

communication in the sensitive group than in the resistant group, and the blue connecting line the other way round. (B-D) Heatmaps showing the overall (B), outgoing (C) and incoming (D) signaling intensities for each cell type across the two groups. (E-F) Box-and-whisker plots comparing overall (E) and incoming (F) signaling intensity scores of CXCL, CCL, LAMININ and RESISTIN signaling pathway in immune effector-related CD4+ T cells (n = 4, permutation test) and immune effector-related CD8+ T cells between resistant and sensitive groups (n = 5, permutation test). (G-J) Chord plots demonstrating the communication strength of four selected signaling pathways — CXCL (G), CCL (H), LAMININ (I), and RESISTIN (J) — among cell types between two groups. For A-J, all n = 10 ovarian cancer ascites samples were analyzed.

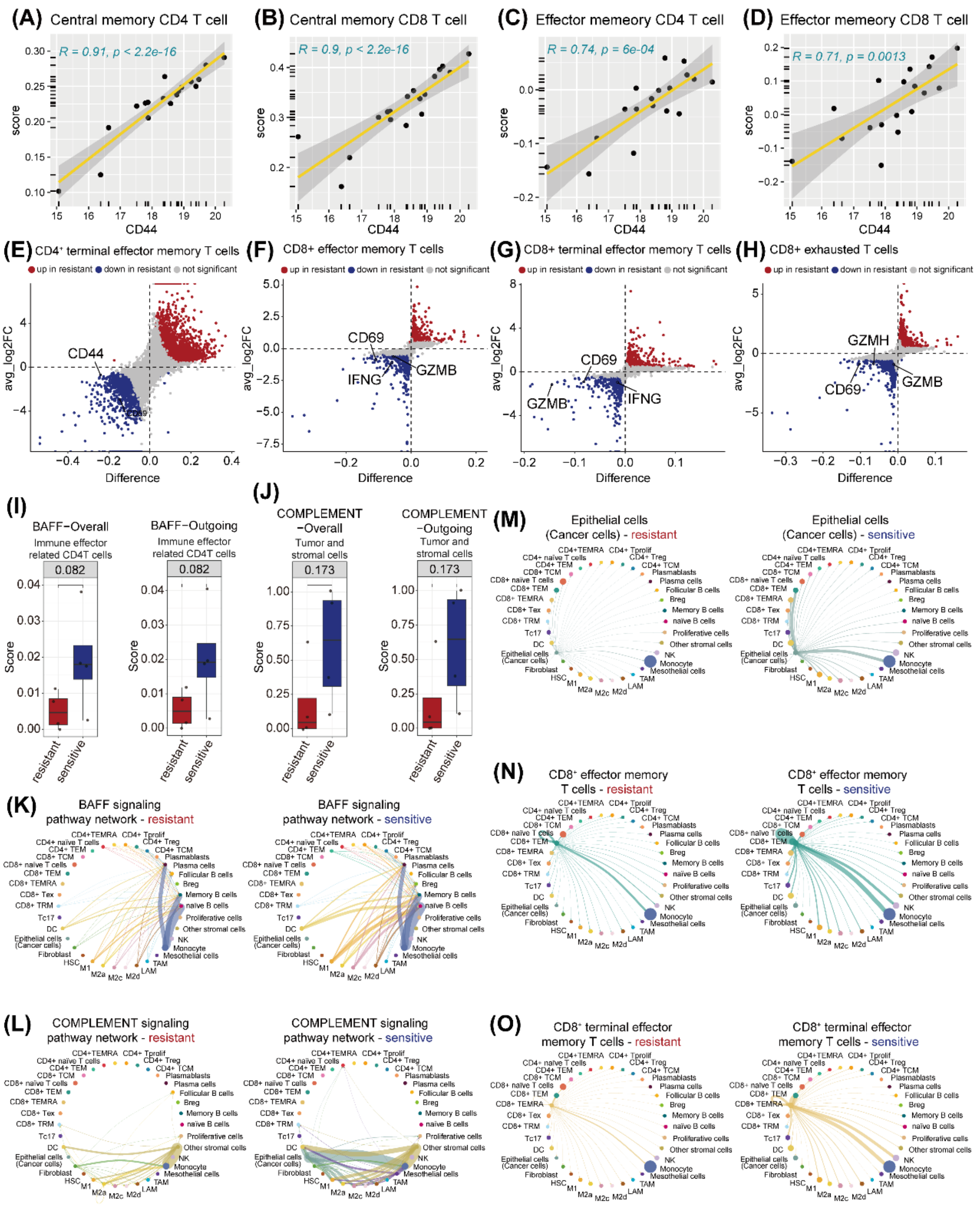

FIGURE S4 | Altered immune effector molecules and signaling pathways in platinum-resistant versus platinum-sensitive ovarian cancer microenvironments. (A-D) Correlation analysis of CD44 expression with central memory $CD4^+$ T cell (A), central memory $CD8^+$ T cell (B), effector memory $CD4^+$ T cell (C) and effector memory $CD8^+$ T cell (D) (n = 18, Spearman correlation). (E-H) Volcano plots demonstrating differential expression of immune effector molecules in $CD4^+$ terminal effector memory T cells cluster (E), $CD8^+$ effector memory T cells cluster (F), $CD8^+$ terminal effector memory T cells cluster (G), and $CD8^+$ exhausted T cells cluster (H) between resistant and sensitive groups of the single-cell transcriptome data (significant difference criteria: adjusted P<0.05, |log2FC| >0.5). (I) Box-and-whisker plots comparing overall and output intensity scores of the B-cell activating factor

(BAFF) signaling pathway in immune effector-related CD4$^+$ T cells between resistant and sensitive groups (n = 4, permutation test). (J) Box-and-whisker plots comparing overall and output intensity scores of the COMPLEMENT signaling pathway in tumor and stromal cells between resistant and sensitive groups (n = 3, permutation test). (K-L) Chord plot demonstrating the difference in the communication intensity of BAFF signaling pathway (K) and COMPLEMENT signaling pathway (L) among cell types between resistant and sensitive groups. (M-O) Chord plot demonstrating the difference in the communication intensity of Epithelial cells (Cancer cells) cluster and other cell types (M), CD8$^+$ effector memory T cells cluster and other cell types (N), CD8$^+$ terminal effector memory T cells cluster and other cell types (O) between resistant and sensitive groups. For A-D, data were summarized from all n = 18 HGSOC samples. For E-O, all n = 10 ovarian cancer ascites samples were analyzed (n = 3 for resistant and n = 7 for sensitive).

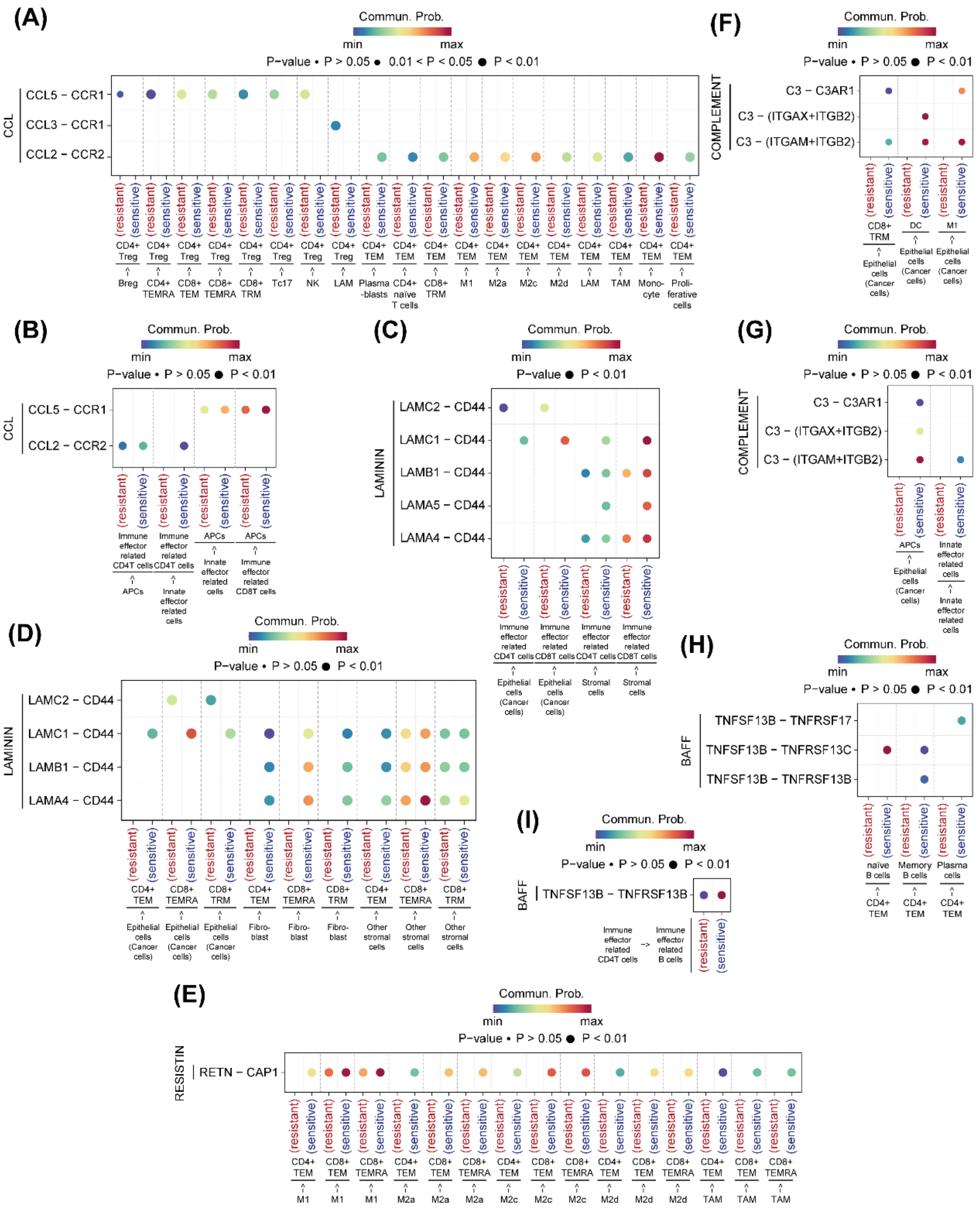

F I G U R E S5 | Differential receptor − ligand communications across cell types. (A-B) Bubble plot demonstrating difference in specific receptor-ligand pairs mediating the CC-chemokine ligand (CCL) signaling pathway communication intensity of detailed (A, e.g. CD4$^+$ Treg, CD4$^+$ TEM, etc.) and aggregated (B, e.g. APCs, innate effector cells, etc.) clusters between resistant and sensitive groups. (C-D) Bubble plot demonstrating the difference in the specific receptor-ligand pairs of LAMININ signaling pathway communication intensity of detailed (C, e.g. CD4$^+$ TEM, CD8$^+$ TEMRA, etc.) and aggregated (D, e.g. stromal cells, immune effector related CD8$^+$ T cell, etc.) clusters between the resistant and sensitive groups. (E) Bubble plot demonstrating the difference in the specific receptor-ligand pairs of RESISTIN signaling pathway communication intensity of detailed (e.g. CD4$^+$ TEM, CD8$^+$ TEM,

etc.) clusters between the resistant and sensitive groups. (F-G) Bubble plot demonstrating the difference in the specific receptor-ligand pairs of COMPLEMENT signaling pathway communication intensity of detailed (F, e.g. $CD8^+$ TRM, DC, etc.) and aggregated (G, e.g. APCs, innate effector cells, etc.) clusters between the resistant and sensitive groups. (H-I) Bubble plot demonstrating the difference in the specific receptor-ligand pairs of BAFF signaling pathway communication intensity of detailed (H, e.g. $CD4^+$ TEM, memory B cells etc.) and aggregated (I, e.g. immune effector related $CD4^+$ T cell and immune effector related B cell) clusters between the resistant and sensitive groups. For A-I, all n = 10 ovarian cancer ascites samples were analyzed (n = 3 for resistant and n = 7 for sensitive). X-axis labels in red represent communication in the resistant group and blue in the sensitive group.

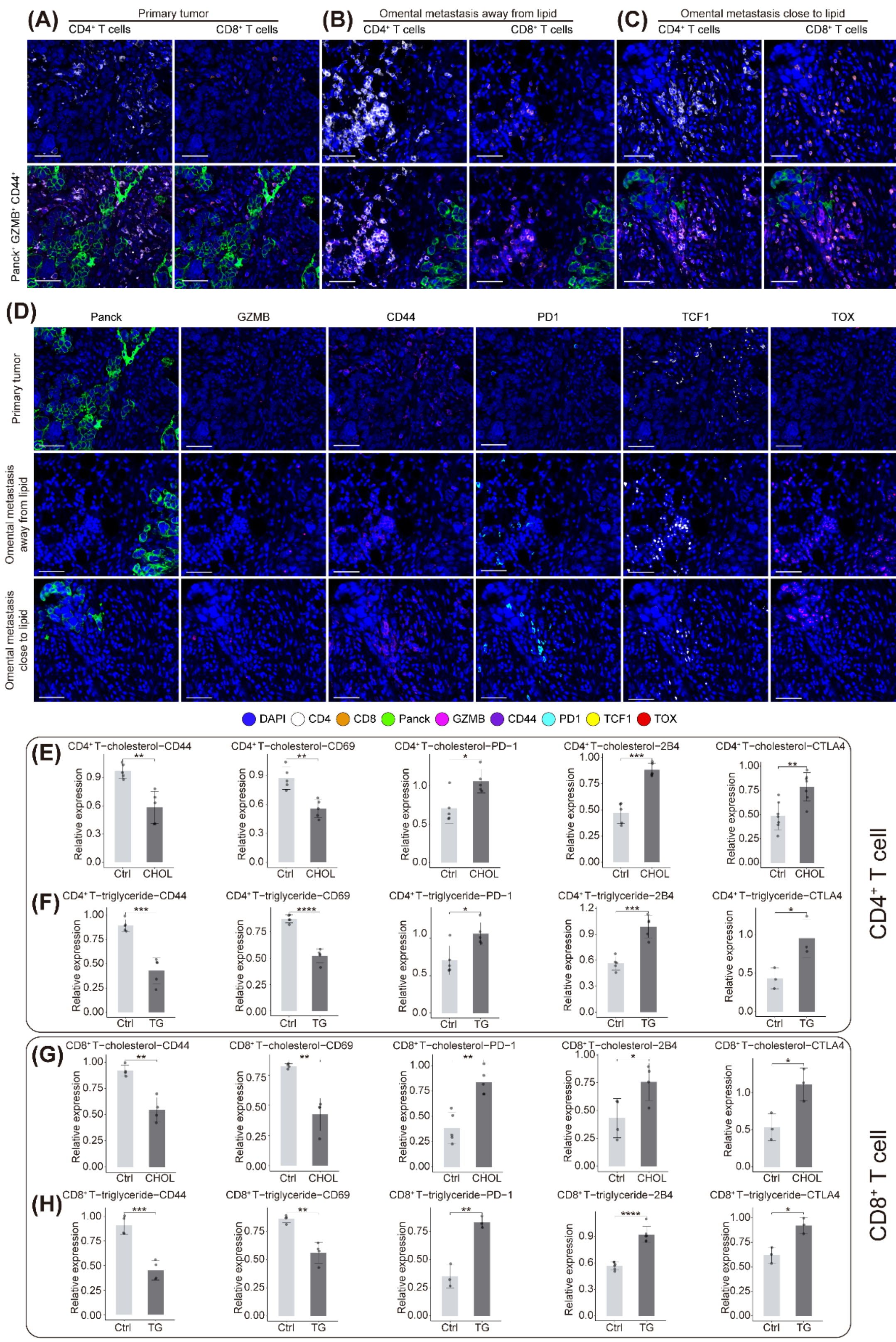

F I G U R E S6 | Merged immunofluorescence (IF) staining images of immune effector molecules alongside single-channel staining images of individual markers and quantitative results from Western blot (WB) analysis of immune cells. (A-C) Representative merged IF staining image of CD4, CD8, Pan-Cytokeratin (PanCK), Granzyme B (GZMB) and CD44 in three contexts: primary tumor site (A), omental

metastasis away from lipid (B), and omental metastasis close to lipid (C). Scale bar = 50 μm. (D) Representative single-channel staining images of individual markers (Panck, GZMB, CD44, Programmed cell death protein 1 (PD1), T-cell factor 1 (TCF1) and Thymocyte selection-associated high mobility group box protein (TOX) in three contexts. Scale bar = 50 μm. (E) Quantitative results of the effect of cholesterol (CHOL) treatment on $CD4^+$ T cell activation (CD44, n = 6; CD69, n = 5) and exhaustion markers (PD-1, n = 5; 2B4, n = 5; Cytotoxic T-lymphocyte–associated protein 4 (CTLA4), n = 7). (F) Quantitative results of the effect of triglyceride (TG) treatment on $CD4^+$ T cell activation (CD44, n = 6; CD69, n = 5) and exhaustion markers (PD-1, n = 4; 2B4, n = 5; CTLA4, n = 3). (F) Quantitative results of the effect of CHOL treatment on $CD8^+$ T cell activation (CD44, n = 4; CD69, n = 5) and exhaustion markers (PD-1, n = 5; 2B4, n = 4; CTLA4, n = 3). (G) Quantitative results of the effect of TG treatment on $CD8^+$ T cell activation (CD44, n = 4; CD69, n = 4) and exhaustion markers (PD-1, n = 3; 2B4, n = 6; CTLA4, n = 3). All statistical tests used in this figure are two-tailed *t*-test.

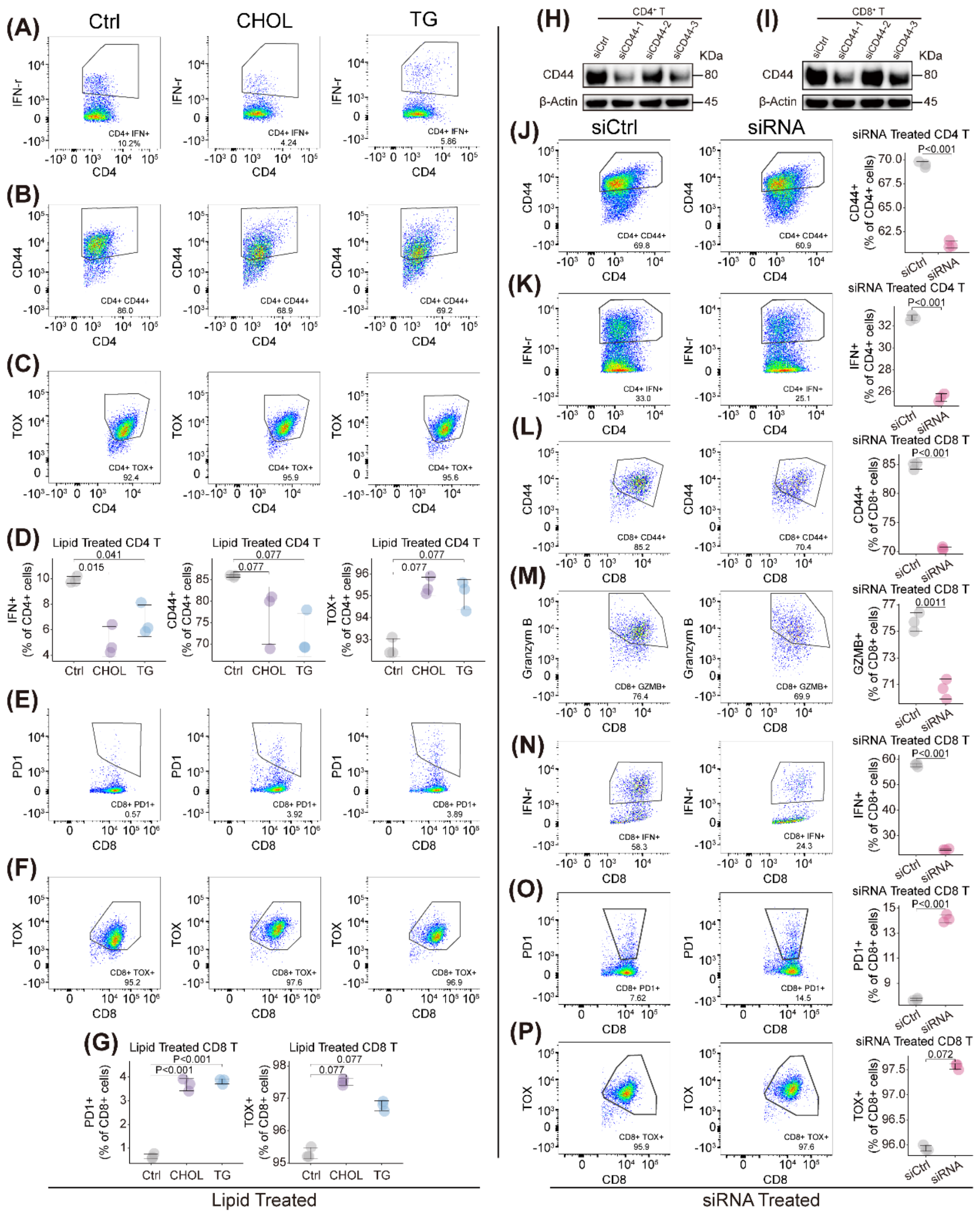

F I G U R E S7 | Effects of lipid treatment and siRNA-mediated knockdown (KD) on immune effector–related molecule expression in human CD4^{+} and CD8^{+} T cells assessed by flow cytometry (FCM) and WB. (A-C) Following treatment with CHO, TG, or blank controls, FCM was used to assess the expression of IFN-γ (A), CD44 (B) and TOX (C) in CD4^{+} T cells. (D) Scatter plots showing intergroup differences in IFN-γ (n = 3, two-tailed *t*-test), CD44 (n = 3, two-sided Wilcoxon rank-sum tests) and TOX (n = 3, two-sided Wilcoxon rank-sum tests) among the three treatment groups. (E-F) Following treatment with CHO, TG, or blank controls, FCM was used to assess the expression of PD-1 (E) and TOX (F) in CD8^{+} T cells. (G) Scatter plots showing intergroup differences in PD-1 (n = 3, two-tailed *t*-test) and TOX (n = 3, two-sided Wilcoxon rank-sum tests) among the three treatment groups. (H-I)

Representative WB results showing CD44 protein levels in $CD4^+$ T cells (H) and $CD8^+$ T cells (I) after small interfering RNA (siRNA)-mediated KD. (J-K) Following treatment with siRNA mediated KD , FCM analysis was used to assess the expression of CD44 (J) and IFN-γ (K). Intergroup differences of CD44 (n = 3, two-tailed *t*-test) and IFN-γ (n = 3, two-tailed *t*-test) were visualized through scatter plots. (L-P) Following treatment with siRNA mediated KD, FCM analysis was used to assess the expression of CD44 (L), GZMB (M), IFN-γ (N), PD-1 (O) and TOX (P) in $CD8^+$ T cells. Intergroup differences of CD44 (n = 3, two-tailed *t*-test), GZMB (n = 3, two-tailed *t*-test), IFN-γ (n = 3, two-tailed *t*-test), PD-1 (n = 3, two-tailed *t*-test) and TOX (n = 3, two-sided Wilcoxon rank-sum tests) were visualized through scatter plots.

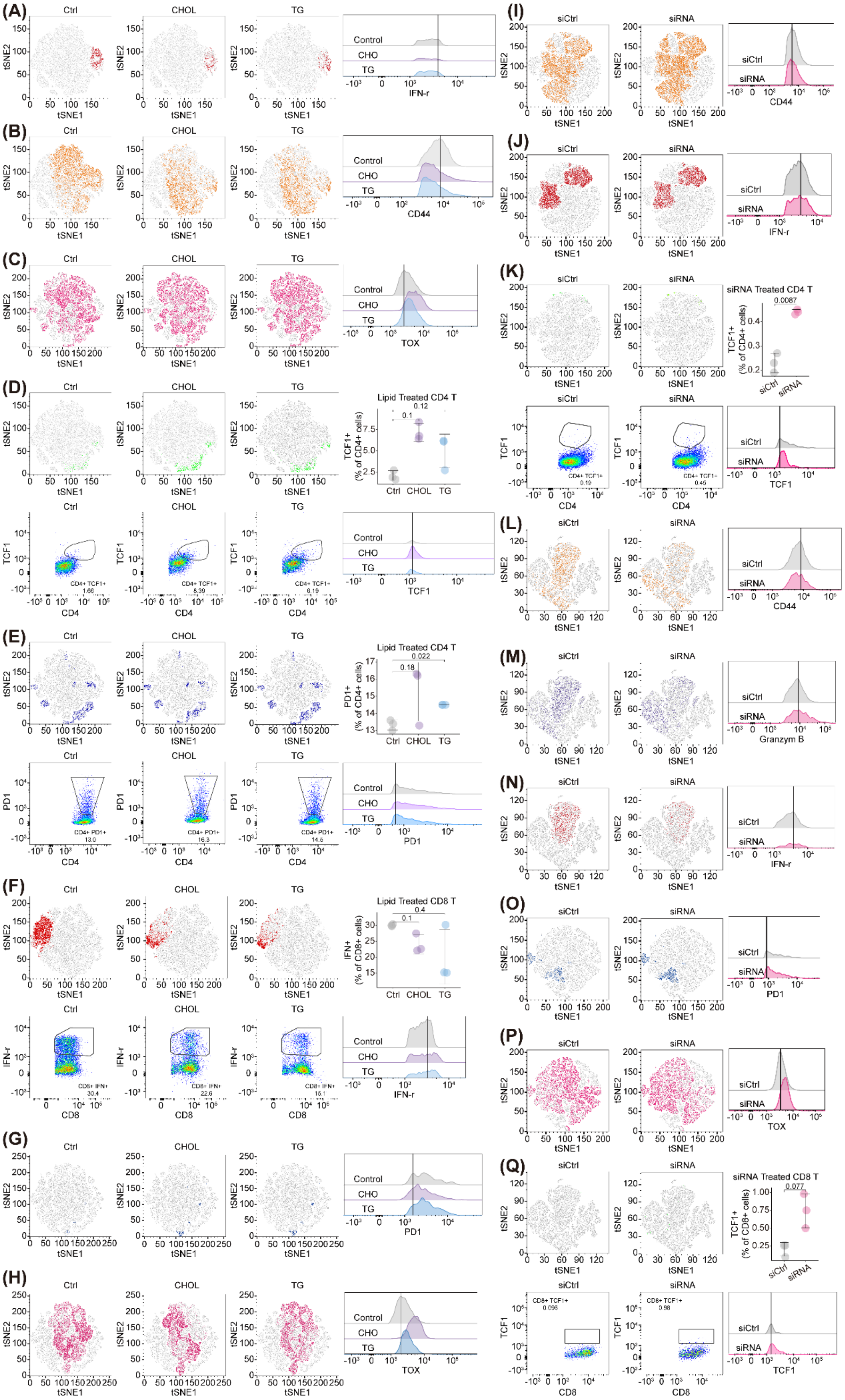
(A)
Ctrl
CHOL
TG
Control
CHO
TG
IFN-r
(B)
CD44
(C)
TOX
(D)
Lipid Treated CD4 T
0.12
0.1
TCF1+ (% of CD4+ cells)
CD4+ TCF1+ 1.66
CD4+ TCF1+ 8.39
CD4+ TCF1+ 6.19
TCF1
CD4
(E)
Lipid Treated CD4 T
0.022
0.18
PD1+ (% of CD4+ cells)
CD4+ PD1+ 13.0
CD4+ PD1+ 16.3
CD4+ PD1+ 14.5
PD1
(F)
Lipid Treated CD8 T
0.4
0.1
IFN+ (% of CD8+ cells)
CD8+ IFN+ 30.4
CD8+ IFN+ 22.6
CD8+ IFN+ 15.1
CD8
(G)
PD1
(H)
TOX
(I)
siCtrl
siRNA
CD44
(J)
IFN-r
(K)
siRNA Treated CD4 T
0.0087
TCF1+ (% of CD4+ cells)
CD4+ TCF1+ 0.19
CD4+ TCF1+ 0.45
TCF1
(L)
CD44
(M)
Granzym B
(N)
IFN-r
(O)
PD1
(P)
TOX
(Q)
siRNA Treated CD8 T
0.077
TCF1+ (% of CD8+ cells)
CD8+ TCF1+ 0.096
CD8+ TCF1+ 0.98
TCF1
CD8
tSNE1
tSNE2

F I G U R E S8 | Flow cytometry (FCM) analysis of the effects of lipid treatment and siRNA-mediated KD on immune effector-related molecules in CD4$^+$ and CD8$^+$ T cells. (A-E) Following treatment with CHO, TG, or blank controls, FCM was used to assess the expression of IFN- γ (A), CD44 (B), TOX (C), TCF1 (D, n = 3, two-sided Wilcoxon rank-sum tests), and PD1 (E, n = 3, two-tailed t-test) in CD4$^+$ T cells. Intergroup differences were visualized through t-SNE dimensionality reduction, density plots, and scatter plots. (F-H) Following treatment with CHO, TG, or blank controls, FCM was used to assess the expression of IFN- γ (F, n = 3, two-sided Wilcoxon rank-sum tests), PD1 (G) and TOX (H) in CD8$^+$ T cells. Intergroup differences were visualized through t-SNE dimensionality reduction, density plots, and scatter plots. (I-K) Following treatment with siRNA mediated KD , FCM analysis was used to assess the expression of CD44 (I), IFN- γ (J), and TCF1 (K, n = 3, two-tailed t-test) in CD4$^+$ T cells. Intergroup differences were visualized through t-SNE dimensionality reduction, density plots, and scatter plots. (M-Q) Following treatment with siRNA mediated KD, FCM analysis was used to assess the expression of CD44 (L), GZMB (M), IFN- γ (N), PD-1 (O), TOX (P) and TCF1 (Q, n = 3, two-sided Wilcoxon rank-sum tests) in CD8$^+$ T cells. Intergroup differences were visualized through t-SNE dimensionality reduction, density plots, and scatter plots.

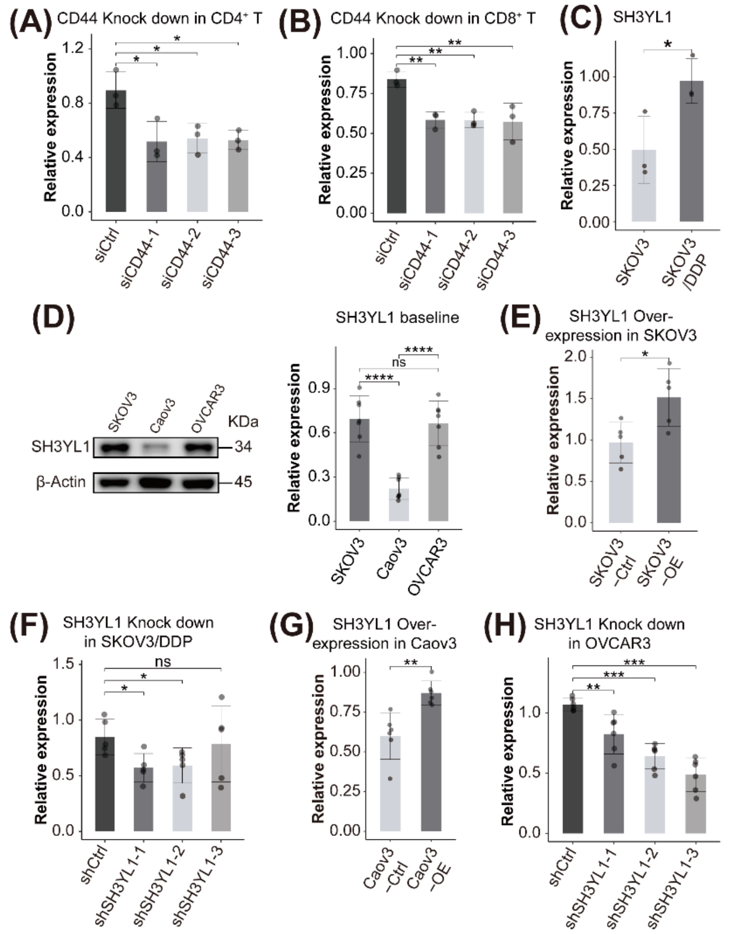

F I G U R E S9 | Quantitative WB analysis of cell lines with genetic manipulation. (A-B) Quantification of CD44 protein levels in CD4$^+$ (A, n= 3, one-way ANOVA)

and CD8^{+} T cells (B, n= 3, one-way ANOVA) with small interfering RNA (siRNA)-mediated knockdown (KD). (C) Quantification of Src homology 3 domain-containing YSC84-like 1 (SH3YL1) protein levels between platinum-sensitive ovarian cancer cell line SKOV3 and resistant line SKOV3/DDP on SH3YL1 (n= 3, two-tailed t-test). (D) WB results and quantification of SH3YL1 protein levels across three ovarian cancer cell lines (SKOV3, Caov3 and OVCAR3) (n= 7, one-way ANOVA). (E) Quantification of SH3YL1 protein levels in SKOV3 with lentivirus-mediated overexpression (OE) (n= 5, two-tailed t-test). (F) Quantification of SH3YL1 protein levels in SKOV3/DDP with lentivirus-mediated KD (n= 5, one-way ANOVA). (G) Quantification of SH3YL1 protein levels in Caov3 with lentivirus-mediated OE (n= 6, two-tailed t-test). (H) Quantification of SH3YL1 protein levels in OVCAR3 with lentivirus-mediated KD (n= 5, one-way ANOVA).

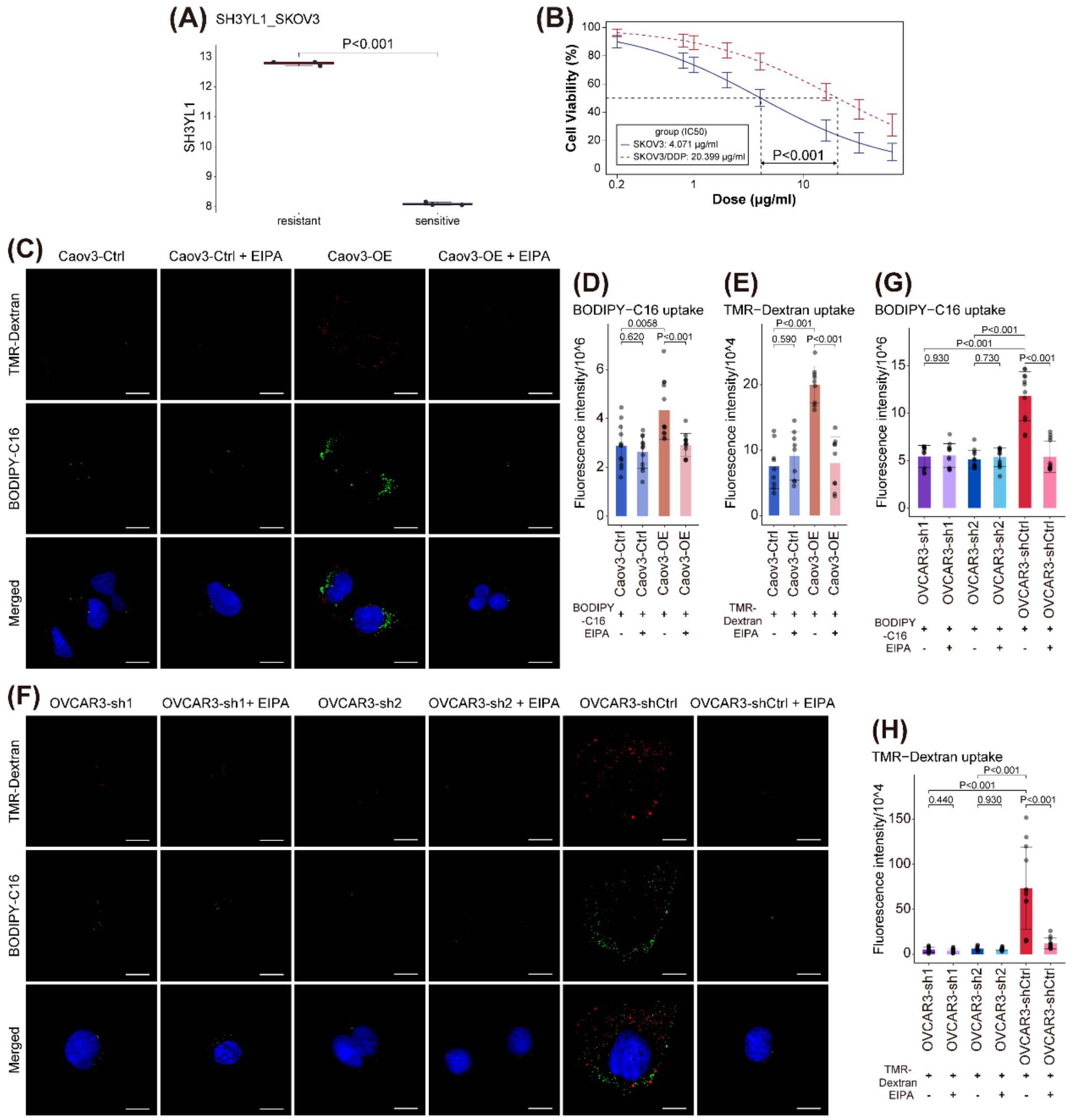

F I G U R E S10 | Validation of differentially expressed proteins in cell line transcriptomic data, confirmation of successful domestication of resistant cell lines, and representative images and quantification of fluorescent tracer uptake to measure

lipid uptake via macropinocytosis. (A) Using publicly available transcriptomic data of SKOV3 cell line, the differential expression of SH3YL1 was validated (n = 3, two-tailed t-test). (B) Verification that the resistant cell line SKOV3/DDP displays stronger platinum resistance compared to the parental sensitive SKOV3 line. (C) Representative fluorescence microscopy images (single-channel and merged) showing uptake of fluorescent tracer in Caov3-SH3YL1-OE cells (via lentiviral transduction) or vector control, with or without pretreatment by the macropinocytosis inhibitor EIPA. Scale bar = 20 μm. (D-E) Quantification of fluorescence intensity for lipid uptake (using BODIPY-C6; D, n = 12, two-sided Wilcoxon rank-sum tests, p-values adjusted by the BH method) and macropinocytosis (using TMR-Dextran; E, n = 9, two-sided Wilcoxon rank-sum tests, p-values adjusted by BH method) in Caov3-SH3YL1-OE vs. control under indicated conditions. Experiments were repeated at least 3 times. (F) Representative fluorescence microscopy images (single-channel and merged) showing uptake of fluorescent tracer in OVCAR3-SH3YL1-KD cells (via lentiviral transduction) or short hairpin RNA (shRNA) control, with or without pretreatment by EIPA. Scale bar = 20 μm. (G-H) Quantification of fluorescence intensity for lipid uptake (using BODIPY-C6; G, n = 12, two-sided Wilcoxon rank-sum tests, p-values adjusted by the BH method) and macropinocytosis (using TMR-Dextran; H, n = 12, two-sided Wilcoxon rank-sum tests, p-values adjusted by BH method) in OVCAR3/DDP-SH3YL1-KD vs. control under indicated conditions. Experiments were repeated at least 3 times.

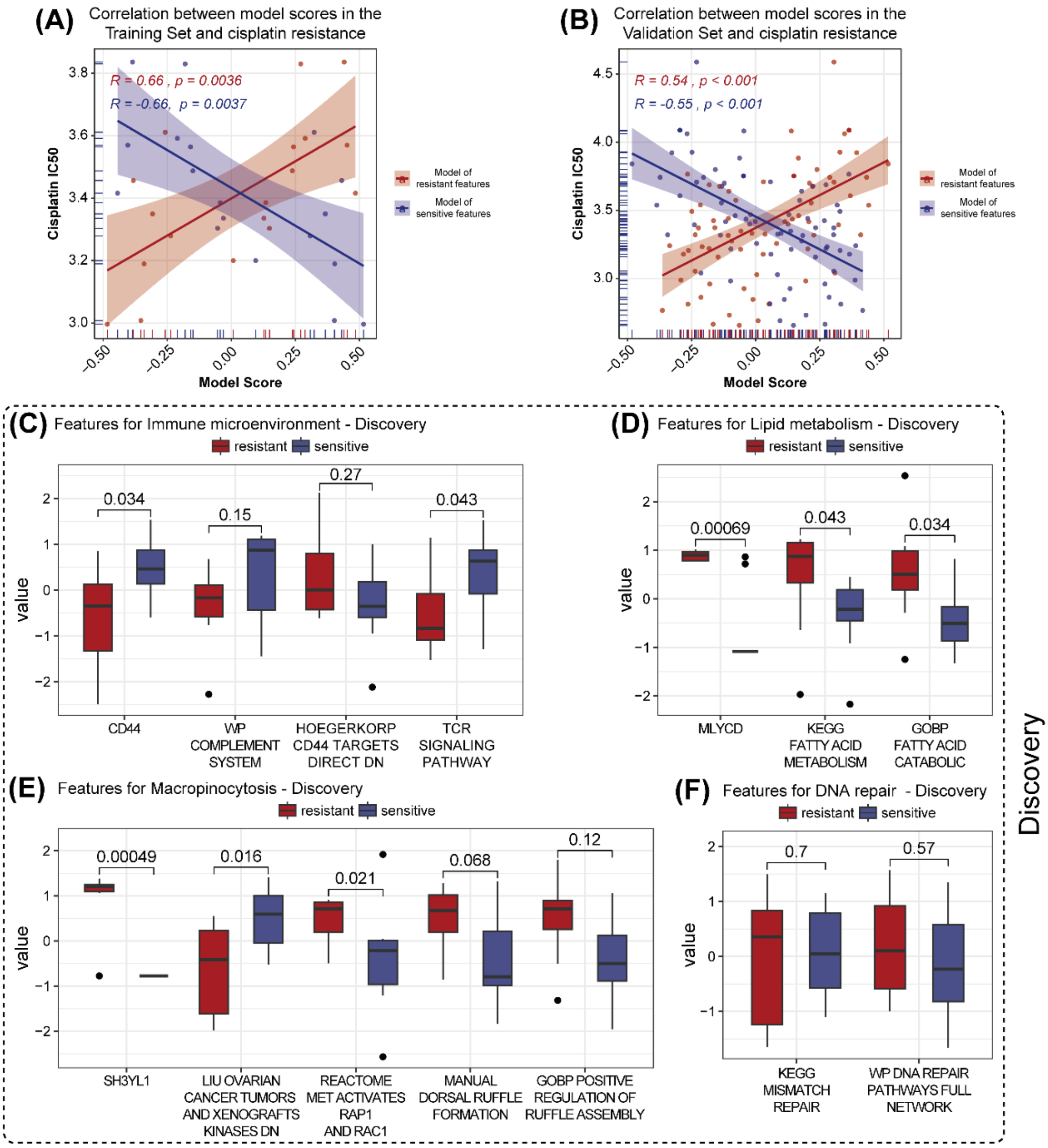

F I G U R E S11 | Characteristic molecules and pathways used for model construction and their association with cisplatin response in ovarian cancer patients. (A) Correlation between the model-derived "resistant modeling score" / "sensitive modeling score" and algorithm predicting cisplatin IC_{50} (half-maximal inhibitory concentration) in the discovery set (A, n = 18, Spearman correlation) and validation set (B, n = 75, Spearman correlation). (C) Box-and-whisker plots demonstrating the differences in expression levels of characteristic molecules and pathways related to the immune microenvironment in the discovery set between the sensitive/resistant groups. (D) Box-and-whisker plots demonstrating the difference in expression levels of lipid metabolism-related characteristic molecules and pathways between the sensitive/resistant groups in the discovery set. (E) Box-and-whisker plots Box-and-whisker plots demonstrating the differences in expression levels of macropinocytosis-related signature molecules and pathways between the sensitive/resistant groups in the discovery set. (F) Box-and-whisker plots Box-and-whisker plots demonstrating the

difference in expression levels of DNA repair-related signature molecules and pathways between the sensitive/resistant groups in the discovery set. For C-F, n = 75 HGSOC tissue samples were analyzed (n = 20 for resistant and n = 55 for sensitive, two-sided Wilcoxon rank-sum tests).

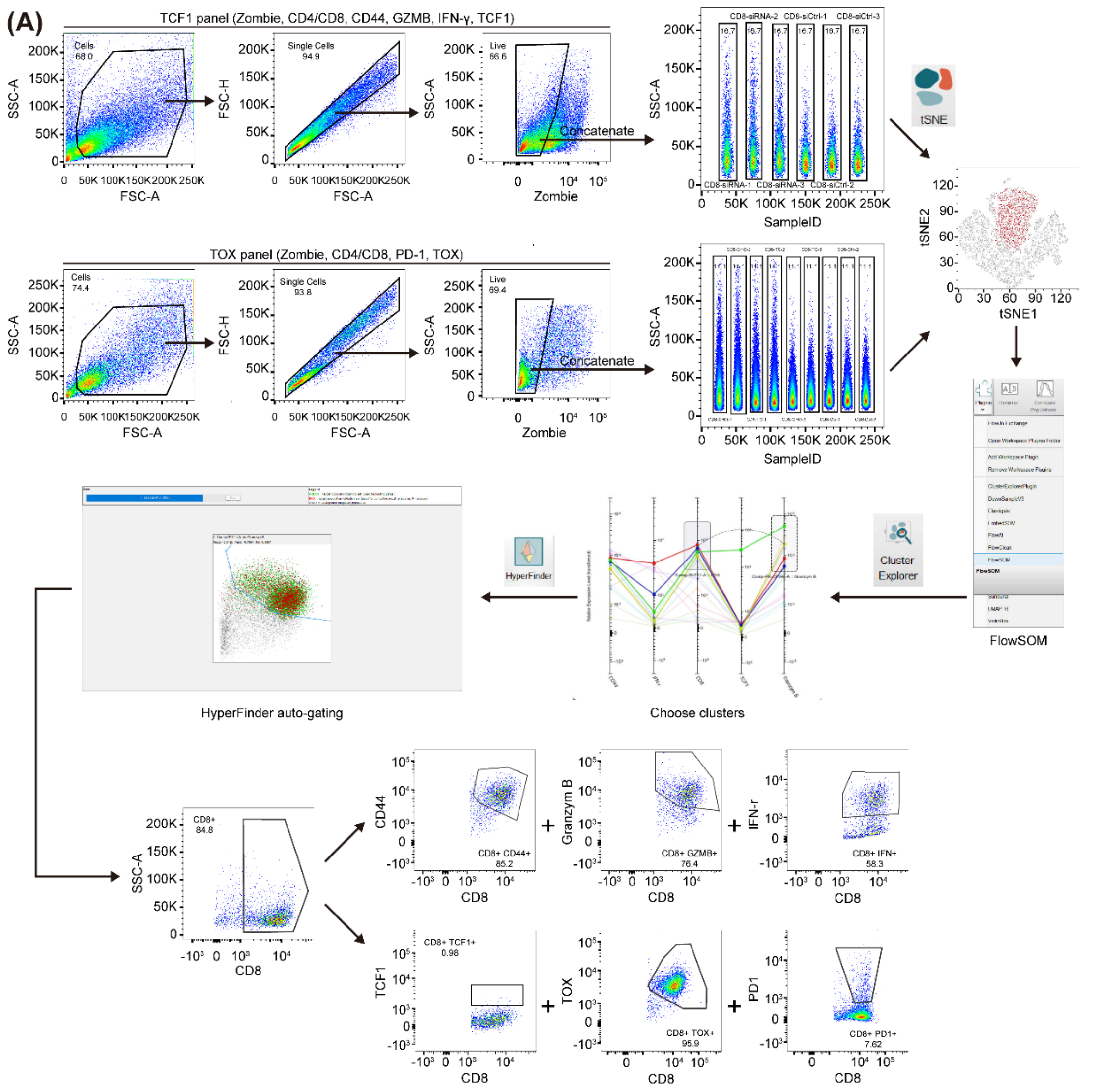

F I G U R E S12 | Gating strategy for sorting immune-effector marker-expressing T cells from peripheral blood. (A) Samples were first gated on forward scatter (FSC) vs side scatter (SSC) to select cells and exclude debris, then singlets were gated using FSC-A (area) vs FSC-H (height) to remove doublets. Next, live cells were selected by excluding dead-cell dye. After basic gating, populations of interest were defined based on marker expression. For multidimensional data, an automated gating tool (HyperFinder) was used: populations identified from clustering / dimensionality reduction (e.g. t-SNE and FlowSOM) served as "positive population" input; HyperFinder then generated optimized gating boundaries (polygon gates) maximizing both purity and yield, which were applied uniformly across all samples for downstream quantification or sorting.

Descriptions of Supplementary Tables

Sheet	Description	Note
S1-Clinicopathologic features	Clinicopathologic features of the proteomic set samples.	Related to Figure 1, 3
S2-KEGG resistant group	Kyoto Encyclopedia of Genes and Genomes enrichment analysis of upregulated genes in the platinum-resistant group	Related to Figure S1
S3-KEGG sensitive group	Kyoto Encyclopedia of Genes and Genomes enrichment analysis of upregulated genes in the platinum-sensitive group	Related to Figure S1
S4-SSGSEA annotated gene list	List of gene symbols manually annotated to the ssGSEA pathway	Related to Figure 2
S5-ssGSEA curated annotation	Expression spectrum of the ssGSEA-transformed platinum dataset annotated by c2.all.v2023.2.Hs.symbols.gmt	Related to Figure 1
S6-Estimate score	Estimate score	Related to Figure 2
S7-ssGSEA hallmark annotation	Expression spectrum of the ssGSEA-transformed platinum dataset annotated by h.all.v2023.2.Hs.symbols.gmt	Related to Figure 2
S8-ssGSEA immuepath annotation	Expression spectrum of the ssGSEA-transformed platinum dataset annotated by c7.all.v2023.2.Hs.symbols.gmt	Related to Figure 2
S9-ssGSEA immuecell annotation	Expression spectrum of the ssGSEA-transformed TCGA-GTEX dataset annotated by manual gene list of immue cells	Related to Figure 2
S10-Immunohistochemical results	Immunohistochemical results	Related to Figure 2, 5
S11-cellmarker	List of Signature Genes used for cell type annotation	Related to Figure 2 and Figure S2
S12-KEGG.lipid resistant group	Kyoto Encyclopedia of Genes and Genomes enrichment analysis of upregulated lipid process related genes in the platinum-resistant group	Related to Figure 3
S13-KEGG.lipid sensitive group	Kyoto Encyclopedia of Genes and Genomes enrichment analysis of upregulated lipid process related genes in the platinum-resistant group	Related to Figure 3
S14-Ascites lipid levels	Ascites lipid levels in patients with ovarian cancer ascites	Related to Figure 3
S15-WB Quantitative Statistics	Quantitative Statistics of Western Blot results	Related to Figure S9
S16-FCM Quantitative Statistics	Quantitative Statistics of Flow Cytometry results	Related to Figure S7, 8
S17-KEGG SH3YL1 positively	Kyoto Encyclopedia of Genes and Genomes enrichment analysis of genes positively associated with SH3YL1 expression	Related to Figure 5
S18-KEGG SH3YL1 negatively	Kyoto Encyclopedia of Genes and Genomes enrichment analysis of genes negatively associated with SH3YL1 expression	Related to Figure 5
S19-CCK8 results	Cell Counting Kit-8 results	Related to Figure 7 and Figure S10
S20-FT Quantitative Statist	Quantitative Statistics of Fluorescent tracer results	Related to Figure 6 and Figure S10
S21-single cell metabolome	Single-cell metabolome of ovarian cancer cell lines	Related to Figure 6
S22-Logistic model construction	Training and Validation Sets for Logistic regression model	Related to Figure 8 and Figure S11
S23-Prediction results of model	Prediction results of the logistic regression model	Related to Figure 8
S24-List of reagent or resource	List of reagents and resource	Related to Figure S12
S25-Protein expression profile	Protein expression profiles	Related to Figure 1

Supplementary Table 1. Clinicopathologic features of the proteomic set samples
"1" in DFS represents death or recurrence

Patient ID	Purity	group	Pathological types	Gender	Age	DFS time (month)	DFS	CHO (mmol/L)	TG (mmol/L)	HDL (mmol/L)	LDL (mol/L)	Lpa (nmol/L)	ApoA1 (g/L)	ApoB (g/L)
HGSOC1	80%	sensitive	high-grade serous ovarian cancer	Female	49	18	1	4.87	1.06	1.23	3.12	77.7	1.29	1.22
HGSOC2	80%	sensitive	high-grade serous ovarian cancer	Female	62	29.8	1	3.36	0.87	1.08	2.03	62.3	1.19	0.79
HGSOC3	80%	sensitive	high-grade serous ovarian cancer	Female	55	12.6	1	4.21	0.79	0.89	3.11	68.4	0.97	1.2
HGSOC4	50%	sensitive	high-grade serous ovarian cancer	Female	51	16.1	1	5.83	1.64	0.83	4.45	273.1	1.13	1.8
HGSOC5	50%	sensitive	high-grade serous ovarian cancer	Female	71	20	1	4.98	2.08	1.17	3.29	52.8	1.38	1.24
HGSOC6	50%	sensitive	high-grade serous ovarian cancer	Female	64	24	1	5.69	1.48	1.58	3.66	86.8	1.61	1.34
HGSOC7	50%	sensitive	high-grade serous ovarian cancer	Female	50	40.1	1	4.23	1.48	1	2.68	23.1	1.14	1.15
HGSOC8	40%	sensitive	high-grade serous ovarian cancer	Female	54	18.7	1	4.64	1.5	1	3.2	70.3	1.14	1.24
HGSOC9	30%	sensitive	high-grade serous ovarian cancer	Female	71	29	1	5.07	1.3	1.29	3.73	180	NA	NA
HGSOC10	30%	sensitive	high-grade serous ovarian cancer	Female	41	13.6	1	3.73	1.08	0.89	2.5	75.1	1.06	1.06
HGSOC11	80%	resistant	high-grade serous ovarian cancer	Female	55	2.5	1	4.02	1.18	1.42	2.19	32.4	1.4	0.99
HGSOC12	80%	resistant	high-grade serous ovarian cancer	Female	53	3	1	4.54	1.17	0.89	3.05	136.5	0.88	1.2
HGSOC13	80%	resistant	high-grade serous ovarian cancer	Female	46	3	1	4.59	2.35	0.85	2.97	148.7	1.16	1.24
HGSOC14	80%	resistant	high-grade serous ovarian cancer	Female	66	5	1	4.15	2.47	1.06	2.35	119.3	1.43	0.75
HGSOC15	80%	resistant	high-grade serous ovarian cancer	Female	41	4.4	1	5.5	1.34	1.32	3.89	91.8	1.45	1.22
HGSOC16	80%	resistant	high-grade serous ovarian cancer	Female	54	0	1	4.95	1.57	1.23	3.24	66.2	1.48	1.13
HGSOC17	80%	resistant	high-grade serous ovarian cancer	Female	61	0	1	4.14	1.63	1.06	2.59	100.9	1.4	1.13
HGSOC18	50%	resistant	high-grade serous ovarian cancer	Female	48	4	1	5.28	7.68	0.81	1.86	3.8	1.19	0.94
							reference value	2.85-5.69	0.45-1.69	1.15-1.68	<3.34	0-75	1.08-2.25	0.6-1.17

Supplementary Table 2. Kyoto Encyclopedia of Genes and Genomes enrichment analysis of upregulated genes in the platinum-resistant group

ID	Description	GeneRatio	BgRatio	pvalue	p.adjust	qvalue	geneID	Count	Fold_enrichment
hsa05223	Non-small cell lung cance	33756	72/8521	0.00012	0.01751	0.01468	7157/3845/6654/3800/572/6257	6	2.948600847
hsa05165	man papillomavirus infect	33939	331/8521	0.00021	0.01751	0.01468	/3673/56288/3845/3910/6198/6654/	12	1.748461233
hsa04919	oid hormone signaling pat	33786	121/8521	0.00032	0.01751	0.01468	862/7157/10499/3845/5214/572/625	7	2.422233001
hsa05205	Proteoglycans in cancer	33848	205/8521	0.00035	0.01751	0.01468	365/7157/6382/3673/2066/3845/61	9	2.025028794
hsa04151	PI3K-Akt signaling pathwa	33939	354/8521	0.0004	0.01751	0.01468	57/3673/2066/3845/3910/6198/6654	12	1.650764559
hsa05222	Small cell lung cancer	33756	92/8521	0.00045	0.01751	0.01468	7157/3673/7186/3910/3913/6257	6	2.59454855
hsa04012	ErbB signaling pathway	33725	85/8521	0.00219	0.05908	0.04954	2066/3845/6198/6654/572	5	2.44625623
hsa05225	Hepatocellular carcinoma	33786	168/8521	0.00219	0.05908	0.04954	323/7157/3845/6198/6654/9817/57	7	1.948600847
hsa05210	Colorectal cancer	33725	86/8521	0.0023	0.05908	0.04954	7157/3845/6198/6654/572	5	2.427606173
hsa04512	ECM-receptor interactior	33725	89/8521	0.00267	0.06179	0.05181	3696/6382/3673/3910/3913	5	2.378511623
hsa04810	gulation of actin cytoskele	33817	229/8521	0.00322	0.06327	0.05305	23365/3673/8396/3845/10787/6654/	8	1.695993813
hsa05213	Endometrial cancer	33695	58/8521	0.00346	0.06327	0.05305	7157/3845/6654/572	4	2.675815931
hsa04910	Insulin signaling pathway	33756	137/8521	0.00356	0.06327	0.05305	10580/8660/3845/6198/6654/572	6	2.021479727
hsa01522	Endocrine resistance	33725	98/8521	0.00406	0.06396	0.05363	7157/3845/6198/6654/572	5	2.241840184
hsa04213	egulating pathway - multi	33695	61/8521	0.00415	0.06396	0.05363	8660/3845/6198/1410	4	2.601696516
hsa05226	Gastric cancer	33756	149/8521	0.00537	0.07746	0.06495	8323/7157/3845/6198/6654/6257	6	1.89917563
hsa05221	Acute myeloid leukemia	33695	67/8521	0.00581	0.07893	0.06618	3845/6198/6654/572	4	2.46727948
hsa05216	Thyroid cancer	33664	37/8521	0.00726	0.09315	0.07811	7157/3845/6257	3	2.908812908
hsa01230	iosynthesis of amino acic	33695	75/8521	0.00863	0.09939	0.08334	95/2875/65263/5214	4	2.304511042
hsa05212	Pancreatic cancer	33695	76/8521	0.00904	0.09939	0.08334	7157/3845/6198/572	4	2.283921772
hsa05220	Chronic myeloid leukemia	33695	76/8521	0.00904	0.09939	0.08334	7157/3845/6654/572	4	2.283921772
hsa01521	rosine kinase inhibitor re	33695	79/8521	0.01033	0.10738	0.09003	3845/6198/6654/572	4	2.229587923
hsa05163	ıan cytomegalovirus infec	33786	225/8521	0.01069	0.10738	0.09003	365/7157/7186/3845/6198/6654/559	7	1.526068812
hsa04360	Axon guidance	33756	182/8521	0.0137	0.13184	0.11054	2049/23365/56288/3845/5364/8461	6	1.608809243
hsa04211	ongevity regulating pathwa	33695	89/8521	0.01547	0.13893	0.11649	8660/7157/3845/6198	4	2.056583528
hsa04210	Apoptosis	33725	136/8521	0.01564	0.13893	0.11649	7157/7186/3845/153090/572	5	1.769771739
hsa01210	xocarboxylic acid metabc	33635	19/8521	0.0175	0.14973	0.12554	95/2875	2	3.285402219
hsa05215	Prostate cancer	33695	97/8521	0.02058	0.16207	0.13589	7157/3845/6654/572	4	1.933572638
hsa05224	Breast cancer	33725	147/8521	0.02117	0.16207	0.13589	8323/7157/3845/6198/6654	5	1.655351829
hsa05231	ıoline metabolism in canc	33695	98/8521	0.02129	0.16207	0.13589	3845/6198/6654/1120	4	1.918386234
hsa04510	Focal adhesion	33756	203/8521	0.02226	0.16207	0.13589	3696/3673/3910/6654/3913/572	6	1.454175893
hsa00220	Arginine biosynthesis	33635	22/8521	0.02315	0.16207	0.13589	95/2875	2	3.073820233
hsa00340	Histidine metabolism	33635	22/8521	0.02315	0.16207	0.13589	138199/219	2	3.073820233
hsa05170	ımmunodeficiency virus 1	33756	212/8521	0.02684	0.17712	0.1485	7186/3845/6198/10053/572/55970	6	1.389566812
hsa05207	carcinogenesis - receptoı	33756	212/8521	0.02684	0.17712	0.1485	3845/6198/6654/688/572/6257	6	1.389566812
hsa05160	Hepatitis C	33725	158/8521	0.02785	0.17868	0.14982	7157/7186/3845/6654/572	5	1.550900665
hsa05145	Toxoplasmosis	33695	111/8521	0.03178	0.19841	0.16636	3910/3125/3913/572	4	1.739848103
hsa04920	pocytokine signaling path	33664	69/8521	0.03827	0.21989	0.18437	8660/7186/6257	3	2.010779839
hsa05211	Renal cell carcinoma	33664	69/8521	0.03827	0.21989	0.18437	3845/6654/572	3	2.010779839
hsa04722	urotrophin signaling path	33695	119/8521	0.03952	0.21989	0.18437	7157/3845/6654/572	4	1.63691458
hsa05230	al carbon metabolism in c	33664	70/8521	0.03968	0.21989	0.18437	7157/3845/5214	3	1.989139007
hsa04152	AMPK signaling pathway	33695	121/8521	0.0416	0.21989	0.18437	23417/8660/6198/5214	4	1.613531653
hsa04137	Mitophagy - animal	33664	72/8521	0.04258	0.21989	0.18437	7157/55669/3845	3	1.948600847
hsa05218	Melanoma	33664	72/8521	0.04258	0.21989	0.18437	7157/3845/572	3	1.948600847
hsa04010	MAPK signaling pathway	33786	301/8521	0.04368	0.21989	0.18437	57/51295/7186/2066/3845/6654/559	7	1.10433666
hsa00410	beta-Alanine metabolism	33635	31/8521	0.04379	0.21989	0.18437	23417/219	2	2.580145484
hsa05214	Glioma	33664	75/8521	0.04712	0.23161	0.1942	7157/3845/6654	3	1.887525271
hsa05412	enic right ventricular carc	33664	77/8521	0.05028	0.24198	0.20289	3696/3673/1829	3	1.851998837
hsa03018	RNA degradation	33664	79/8521	0.05354	0.25239	0.21162	51013/25904/5214	3	1.815575429
hsa04062	ıemokine signaling pathw	33725	192/8521	0.05635	0.26033	0.21827	56288/3845/6654/572/55970	5	1.269033146
hsa05130	genic Escherichia coli inf	33725	198/8521	0.06266	0.28059	0.23526	23365/147179/7186/10787/55930	5	1.22650853
hsa04371	Apelin signaling pathway	33695	139/8521	0.06316	0.28059	0.23526	4635/3845/6198/55970	4	1.416839742
hsa04140	Autophagy - animal	33695	141/8521	0.06586	0.28706	0.24069	8660/3845/6198/572	4	1.3950628
hsa05219	Bladder cancer	33635	41/8521	0.07209	0.30838	0.25856	7157/3845	2	2.176322773
hsa05417	Lipid and atherosclerosis	33725	215/8521	0.08262	0.347	0.29094	7157/7186/3845/572/6257	5	1.10433666
hsa04150	mTOR signaling pathway	33695	156/8521	0.08799	0.36294	0.30431	8323/3845/6198/6654	4	1.244887059
hsa04514	Cell adhesion molecules	33695	158/8521	0.09118	0.36951	0.30982	3696/1001/6382/3125	4	1.22650853
hsa05208	cinogenesis - reactive ox	33725	223/8521	0.09306	0.37016	0.31036	4509/3845/6654/9817/572	5	1.056583528
hsa00280	eucine and isoleucine deg	33635	48/8521	0.09454	0.37016	0.31036	549/219	2	1.948600847
hsa05161	Hepatitis B	33695	162/8521	0.09773	0.37626	0.31548	7157/3845/6654/572	4	1.195347598
hsa00330	inine and proline metabol	33635	50/8521	0.10129	0.38356	0.3216	219/65263	2	1.887525271
hsa04014	Ras signaling pathway	33725	236/8521	0.11141	0.40874	0.34271	9610/3845/6654/572/55970	5	0.970853654
hsa04530	Tight junction	33695	170/8521	0.11147	0.40874	0.34271	54566/9414/56288/84612	4	1.124328135
hsa04310	Wnt signaling pathway	33695	171/8521	0.11325	0.40876	0.34273	8323/81839/56998/7157	4	1.117695043
hsa04141	rocessing in endoplasmic	33695	173/8521	0.11684	0.41271	0.34604	23640/7186/7466/1410	4	1.097610797
hsa05206	MicroRNAs in cancer	33756	310/8521	0.11792	0.41271	0.34604	8660/6541/7157/3845/6654/5268	6	0.839959587
hsa05010	Alzheimer disease	33786	384/8521	0.12047	0.41536	0.34826	323/8660/4509/7186/3845/3800/57	7	0.757023247
hsa01200	Carbon metabolism	33664	115/8521	0.12757	0.43337	0.36336	2875/2731/5214	3	1.275007047
hsa04144	Endocytosis	33725	250/8521	0.13299	0.4393	0.36834	84440/147179/56288/3800/84612	5	0.887525271
hsa04370	VEGF signaling pathway	33635	59/8521	0.13312	0.4393	0.36834	3845/572	2	1.650764559
hsa04935	none synthesis, secretioı	33664	120/8521	0.13982	0.45491	0.38143	8660/3845/6654	3	1.214124805
hsa04071	hingolipid signaling pathw	33664	121/8521	0.14232	0.45661	0.38285	7157/7186/3845	3	1.201633861
hsa05217	Basal cell carcinoma	33635	63/8521	0.14792	0.46808	0.39247	8323/7157	2	1.555816155
hsa04814	Motor proteins	33695	193/8521	0.15535	0.48285	0.40485	4635/6990/3800/55930	4	0.941106311
hsa05167	oma-associated herpesv	33695	194/8521	0.15739	0.48285	0.40485	7157/7186/3845/55970	4	0.933572638
hsa05012	Parkinson disease	33725	266/8521	0.15978	0.48285	0.40485	7157/4509/55669/3800/9817	5	0.799087306
hsa04926	Relaxin signaling pathway	33664	129/8521	0.16282	0.48285	0.40485	3845/6654/55970	3	1.10433666
hsa00010	ycolysis / Gluconeogenes	33635	67/8521	0.16304	0.48285	0.40485	219/5214	2	1.464668267
hsa04664	epsilon RI signaling path	33635	68/8521	0.16686	0.48532	0.40692	3845/6654	2	1.443606651
hsa04068	FoxO signaling pathway	33664	131/8521	0.16808	0.48532	0.40692	8660/3845/6654	3	1.084064265
hsa04917	Prolactin signaling pathwa	33635	70/8521	0.17455	0.4959	0.41579	3845/6654	2	1.40599236
hsa05203	Viral carcinogenesis	33695	204/8521	0.17834	0.4959	0.41579	7157/7186/3845/572	4	0.86393845
hsa04915	Estrogen signaling pathwa	33664	137/8521	0.18415	0.4959	0.41579	10499/3845/6654	3	1.021479727
hsa05135	Yersinia infection	33664	137/8521	0.18415	0.4959	0.41579	23365/147179/7186	3	1.021479727
hsa01524	Platinum drug resistance	33635	73/8521	0.1862	0.4959	0.41579	7157/572	2	1.344828497
hsa00785	Lipoic acid metabolism	33604	19/8521	0.18655	0.4959	0.41579	2731	1	2.283921772
hsa05418	hear stress and atherosc	33664	139/8521	0.18959	0.4959	0.41579	7157/6382/9817	3	1
hsa04115	p53 signaling pathway	33635	74/8521	0.1901	0.4959	0.41579	7157/5268	2	1.321928095
hsa04015	Rap1 signaling pathway	33695	210/8521	0.19135	0.4959	0.41579	26037/56288/3845/84612	4	0.815575429
hsa03320	PPAR signaling pathway	33635	75/8521	0.19402	0.4959	0.41579	10580/6257	2	1.304511042
hsa00100	Steroid biosynthesis	33604	20/8521	0.19536	0.4959	0.41579	6713	1	2.211012193
hsa05014	nyotrophic lateral scleros	33756	364/8521	0.19945	0.50079	0.41989	7157/4509/7186/2066/3800/572	6	0.613531653
hsa00770	othenate and CoA biosynt	33604	21/8521	0.20406	0.50687	0.42499	219	1	2.140778656
hsa05166	T-cell leukemia virus 1 ir	33695	222/8521	0.21822	0.53626	0.44963	7157/996/3845/3125	4	0.739848103
hsa04146	Peroxisome	33635	83/8521	0.22568	0.54877	0.46012	23417/5824	2	1.15704371
hsa04662	ell receptor signaling path	33635	84/8521	0.22967	0.54919	0.46048	3845/6654	2	1.14404637
hsa04390	Hippo signaling pathway	33664	157/8521	0.24025	0.54919	0.46048	8323/56288/84612	3	0.82374936
hsa04714	Thermogenesis	33695	232/8521	0.24136	0.54919	0.46048	4509/3845/6198/6654	4	0.678071905
hsa04540	Gap junction	33635	88/8521	0.24568	0.54919	0.46048	3845/6654	2	1.070389328
hsa00563	hatidylinositol (GPI)-anch	33604	26/8521	0.24623	0.54919	0.46048	84720	1	1.831877241
hsa04727	GABAergic synapse	33635	89/8521	0.24969	0.54919	0.46048	54407/55970	2	1.056583528
hsa05235	n and PD-1 checkpoint p	33635	89/8521	0.24969	0.54919	0.46048	3845/6198	2	1.056583528
hsa05410	ypertrophic cardiomyopat	33635	90/8521	0.2537	0.54919	0.46048	3696/3673	2	1.042644337
hsa05022	neurodegeneration - mult	33786	476/8521	0.25388	0.54919	0.46048	323/4509/55669/7186/3845/3800/57	7	0.443606651
hsa00062	Fatty acid elongation	33604	27/8521	0.25439	0.54919	0.46048	117145	1	1.778208576
hsa00650	Butanoate metabolism	33604	27/8521	0.25439	0.54919	0.46048	622	1	1.778208576
hsa00790	Folate biosynthesis	33604	27/8521	0.25439	0.54919	0.46048	5805	1	1.778208576
hsa04520	Adherens junction	33635	93/8521	0.26574	0.56317	0.4722	10580/56288	2	0.992768431
hsa04912	GnRH signaling pathway	33635	93/8521	0.26574	0.56317	0.4722	3845/6654	2	0.992768431
hsa05414	Dilated cardiomyopathy	33635	96/8521	0.27778	0.56903	0.47711	3696/3673	2	0.948600847
hsa00030	entose phosphate pathwa	33604	30/8521	0.27836	0.56903	0.47711	5214	1	1.627606838
hsa00053	rbate and aldarate metab	33604	30/8521	0.27836	0.56903	0.47711	219	1	1.627606838
hsa00630	ate and dicarboxylate met	33604	30/8521	0.27836	0.56903	0.47711	2731	1	1.627606838
hsa05132	Salmonella infection	33695	249/8521	0.2819	0.57121	0.47894	6990/7186/10787/3800	4	0.575312331
hsa05310	Asthma	33604	31/8521	0.28618	0.57143	0.47912	3125	1	1.580145484
hsa04640	Hematopoietic cell lineage	33635	99/8521	0.28982	0.57143	0.47912	3673/3125	2	0.90303827
hsa00052	Galactose metabolism	33604	32/8521	0.29391	0.57143	0.47912	5214	1	1.531069493
hsa00640	Propanoate metabolism	33604	32/8521	0.29391	0.57143	0.47912	23417	1	1.531069493
hsa04916	Melanogenesis	33635	101/8521	0.29783	0.57143	0.47912	8323/3845	2	0.871843649
hsa04914	erone-mediated oocyte m	33635	102/8521	0.30183	0.57143	0.47912	996/3845	2	0.86393845
hsa05146	Amoebiasis	33635	102/8521	0.30183	0.57143	0.47912	3910/3913	2	0.86393845
hsa05152	Tuberculosis	33664	180/8521	0.30772	0.57143	0.47912	23365/3125/572	3	0.622930351
hsa00051	ose and mannose metab	33604	34/8521	0.30913	0.57143	0.47912	5214	1	1.443606651
hsa03020	RNA polymerase	33604	34/8521	0.30913	0.57143	0.47912	51082	1	1.443606651
hsa04625	lectin receptor signaling p	33635	104/8521	0.30983	0.57143	0.47912	23365/3845	2	0.831877241
hsa04621	like receptor signaling pa	33664	186/8521	0.32554	0.57143	0.47912	55669/7186/2635	3	0.575312331
hsa04659	Th17 cell differentiation	33635	108/8521	0.32576	0.57143	0.47912	3125/6257	2	0.782408565
hsa04931	Insulin resistance	33635	108/8521	0.32576	0.57143	0.47912	8660/6198	2	0.782408565
hsa04066	HIF-1 signaling pathway	33635	109/8521	0.32973	0.57143	0.47912	6198/5214	2	0.765534746
hsa00250	spartate and glutamate m	33604	37/8521	0.33136	0.57143	0.47912	2875	1	1.321928095
hsa01250	ynthesis of nucleotide su	33604	37/8521	0.33136	0.57143	0.47912	80146	1	1.321928095
hsa04960	one-regulated sodium rea	33604	37/8521	0.33136	0.57143	0.47912	3845	1	1.321928095
hsa05143	African trypanosomiasis	33604	37/8521	0.33136	0.57143	0.47912	3910	1	1.321928095
hsa05034	Alcoholism	33664	188/8521	0.33148	0.57143	0.47912	3845/6654/55970	3	0.565597176
hsa05330	Allograft rejection	33604	38/8521	0.33861	0.5794	0.48581	3125	1	1.286881148
hsa04725	Cholinergic synapse	33635	113/8521	0.34554	0.58311	0.48891	3845/55970	2	0.713695815
hsa04668	TNF signaling pathway	33635	114/8521	0.34947	0.58311	0.48891	7186/153090	2	0.695993813
hsa00260	serine and threonine me	33604	40/8521	0.35288	0.58311	0.48891	2731	1	1.214124805
hsa04724	Glutamatergic synapse	33635	115/8521	0.3534	0.58311	0.48891	54407/55970	2	0.687060688
hsa04726	Serotonergic synapse	33635	115/8521	0.3534	0.58311	0.48891	3845/55970	2	0.687060688
hsa04216	Ferroptosis	33604	41/8521	0.3599	0.58962	0.49438	7157	1	1.176322773
hsa00380	Tryptophan metabolism	33604	42/8521	0.36685	0.59129	0.49577	219	1	1.14404637
hsa05332	Graft-versus-host disease	33604	42/8521	0.36685	0.59129	0.49577	3125	1	1.14404637
hsa05169	Epstein-Barr virus infectio	33664	202/8521	0.37298	0.59129	0.49577	7157/7186/3125	3	0.464668267
hsa00071	Fatty acid degradation	33604	43/8521	0.37372	0.59129	0.49577	219	1	1.10433666
hsa04940	Type I diabetes mellitus	33604	43/8521	0.37372	0.59129	0.49577	3125	1	1.10433666
hsa04660	ell receptor signaling path	33635	121/8521	0.37679	0.59209	0.49645	3845/6654	2	0.613531653
hsa04611	Platelet activation	33635	124/8521	0.38836	0.60615	0.50823	23365/3673	2	0.575312331
hsa04930	Type II diabetes mellitus	33604	46/8521	0.39389	0.61065	0.51201	8660	1	1.007195501
hsa00620	Pyruvate metabolism	33604	47/8521	0.40047	0.61317	0.51412	219	1	0.97819563
hsa00520	ar and nucleotide sugar r	33604	49/8521	0.41341	0.61317	0.51412	80146	1	0.918386234
hsa04672	immune network for IgA p	33604	49/8521	0.41341	0.61317	0.51412	3125	1	0.918386234
hsa04142	Lysosome	33635	132/8521	0.41873	0.61317	0.51412	10947/10053	2	0.485426827
hsa04650	al killer cell mediated cytot	33635	132/8521	0.41873	0.61317	0.51412	3845/6654	2	0.485426827
hsa04728	Dopaminergic synapse	33635	132/8521	0.41873	0.61317	0.51412	3800/55970	2	0.485426827
hsa00565	Ether lipid metabolism	33604	50/8521	0.41978	0.61317	0.51412	79153	1	0.887525271
hsa05110	Vibrio cholerae infection	33604	50/8521	0.41978	0.61317	0.51412	9414	1	0.887525271
hsa05144	Malaria	33604	50/8521	0.41978	0.61317	0.51412	6382	1	0.887525271
hsa05016	Huntington disease	33695	306/8521	0.42205	0.61317	0.51412	7157/4509/7186/3800	4	0.275007047
hsa05320	utoimmune thyroid diseas	33604	53/8521	0.43849	0.63277	0.53056	3125	1	0.807354922
hsa05162	Measles	33635	138/8521	0.44102	0.63277	0.53056	7157/572	2	0.422233001
hsa03460	Fanconi anemia pathway	33604	55/8521	0.45062	0.64177	0.5381	57599	1	0.748461233
hsa04120	biquitin mediated proteoly	33635	142/8521	0.45563	0.64177	0.5381	996/9817	2	0.378511623
hsa04936	Alcoholic liver disease	33635	142/8521	0.45563	0.64177	0.5381	23417/219	2	0.378511623
hsa04550	ays regulating pluripoten	33635	143/8521	0.45924	0.64294	0.53908	8323/3845	2	0.378511623
hsa04923	llation of lipolysis in adipo	33604	58/8521	0.46834	0.64884	0.54403	8660	1	0.678071905
hsa04072	pholipase D signaling pat	33635	148/8521	0.47712	0.64884	0.54403	3845/6654	2	0.321928095
hsa04730	Long-term depression	33604	60/8521	0.47984	0.64884	0.54403	3845	1	0.622930351
hsa05416	Viral myocarditis	33604	60/8521	0.47984	0.64884	0.54403	3125	1	0.622930351
hsa00590	rachidonic acid metabolis	33604	61/8521	0.48549	0.64884	0.54403	8529	1	0.604071324
hsa00140	eroid hormone biosynthes	33604	62/8521	0.49109	0.64884	0.54403	79154	1	0.575312331
hsa04145	Phagosome	33635	152/8521	0.49117	0.64884	0.54403	3673/3125	2	0.286881148
hsa01240	Biosynthesis of cofactors	33635	153/8521	0.49465	0.64884	0.54403	219/5805	2	0.275007047
hsa00310	Lysine degradation	33604	63/8521	0.49662	0.64884	0.54403	219	1	0.555816155
hsa00561	Glycerolipid metabolism	33604	63/8521	0.49662	0.64884	0.54403	219	1	0.555816155
hsa05131	Shigellosis	33664	247/8521	0.50151	0.64884	0.54403	7157/7186/6198	3	0.163498732
hsa04932	n-alcoholic fatty liver dise	33635	155/8521	0.50155	0.64884	0.54403	8660/7186	2	0.263034406
hsa04929	GnRH secretion	33604	64/8521	0.5021	0.64884	0.54403	3845	1	0.5360529
hsa04218	Cellular senescence	33635	156/8521	0.50498	0.64884	0.54403	7157/3845	2	0.250961574
hsa05321	nflammatory bowel diseas	33604	65/8521	0.50751	0.64884	0.54403	3125	1	0.50589093
hsa04110	Cell cycle	33635	157/8521	0.5084	0.64884	0.54403	7157/996	2	0.23878686
hsa00970	ninoacyl-tRNA biosynthes	33604	66/8521	0.51287	0.65095	0.5458	57038	1	0.485426827
hsa04720	Long-term potentiation	33604	67/8521	0.51817	0.65409	0.54843	3845	1	0.464668267
hsa00830	Retinol metabolism	33604	68/8521	0.52342	0.65711	0.55096	29785	1	0.443606651
hsa04622	like receptor signaling pa	33604	71/8521	0.53881	0.66918	0.56108	7186	1	0.378511623
hsa03010	Ribosome	33635	167/8521	0.54172	0.66918	0.56108	51069/6183	2	0.150559677
hsa04022	GMP-PKG signaling pathw	33635	167/8521	0.54172	0.66918	0.56108	8660/572	2	0.150559677
hsa00562	ositol phosphate metaboli	33604	73/8521	0.5488	0.67432	0.56539	8396	1	0.344828497
hsa04623	osolic DNA-sensing path	33604	75/8521	0.55857	0.6827	0.57242	51082	1	0.298658316
hsa05020	Prion disease	33664	272/8521	0.56711	0.68563	0.57487	4509/3800/572	3	0.028569152
hsa05140	Leishmaniasis	33604	77/8521	0.56814	0.68563	0.57487	3125	1	0.263034406
hsa00980	of xenobiotics by cytoch	33604	78/8521	0.57284	0.68563	0.57487	29785	1	0.250961574
hsa04612	en processing and preser	33604	78/8521	0.57284	0.68563	0.57487	3125	1	0.250961574
hsa04260	Cardiac muscle contractio	33604	87/8521	0.61297	0.72988	0.61197	4635	1	0.084064265
hsa05202	riptional misregulation in	33635	193/8521	0.62093	0.73556	0.61674	7157/6257	2	-0.058893689
hsa05032	Morphine addiction	33604	91/8521	0.62959	0.73604	0.61714	55970	1	0.028569152
hsa04658	1 and Th2 cell differentiat	33604	92/8521	0.63363	0.73604	0.61714	3125	1	0.014355293
hsa05323	Rheumatoid arthritis	33604	93/8521	0.63763	0.73604	0.61714	3125	1	0
hsa04657	IL-17 signaling pathway	33604	94/8521	0.64158	0.73604	0.61714	7186	1	-0.01449957
hsa05415	Diabetic cardiomyopathy	33635	203/8521	0.6485	0.73604	0.61714	4509/5164	2	-0.13606155
hsa05150	phylococcus aureus infec	33604	96/8521	0.64937	0.73604	0.61714	3125	1	-0.058893689
hsa03015	RNA surveillance pathwa	33604	97/8521	0.6532	0.73604	0.61714	23708	1	-0.074000581
hsa04070	hatidylinositol signaling s	33604	97/8521	0.6532	0.73604	0.61714	8396	1	-0.074000581
hsa04666	mma R-mediated phagoc	33604	97/8521	0.6532	0.73604	0.61714	6198	1	-0.074000581
hsa04713	Circadian entrainment	33604	97/8521	0.6532	0.73604	0.61714	55970	1	-0.074000581
hsa00564	cerophospholipid metabo	33604	99/8521	0.66073	0.74092	0.62123	1120	1	-0.089267338
hsa04933	gnaling pathway in diabeti	33604	100/8521	0.66444	0.74147	0.6217	3845	1	-0.104697379
hsa04974	tein digestion and absorp	33604	103/8521	0.67532	0.74999	0.62884	54407	1	-0.152003093
hsa04064	-kappa B signaling pathw	33604	104/8521	0.67887	0.75033	0.62912	7186	1	-0.168122759
hsa04928	ormone synthesis, secret	33604	106/8521	0.68585	0.75443	0.63256	6257	1	-0.200912694
hsa04922	Glucagon signaling pathwa	33604	107/8521	0.68928	0.75462	0.63272	5214	1	-0.200912694
hsa04350	GF-beta signaling pathwa	33604	108/8521	0.69268	0.75476	0.63284	6198	1	-0.217591435
hsa04380	Osteoclast differentiation	33604	128/8521	0.75341	0.81708	0.68509	7186	1	-0.473931188
hsa04114	Oocyte meiosis	33604	131/8521	0.76143	0.82192	0.68915	996	1	-0.49410907
hsa00190	Oxidative phosphorylatior	33604	134/8521	0.76919	0.82261	0.68973	4509	1	-0.535331733
hsa04270	ular smooth muscle contr	33604	134/8521	0.76919	0.82261	0.68973	23365	1	-0.535331733
hsa05322	stemic lupus erythematos	33604	137/8521	0.77671	0.82682	0.69325	3125	1	-0.556393349
hsa05017	Spinocerebellar ataxia	33604	143/8521	0.79102	0.83819	0.70279	7186	1	-0.621488377
hsa04723	rade endocannabinoid si	33604	148/8521	0.80224	0.8462	0.70951	55970	1	-0.666576266
hsa04261	rgic signaling in cardiomy	33604	154/8521	0.81493	0.85009	0.71276	4635	1	-0.736965594
hsa04921	Oxytocin signaling pathwa	33604	154/8521	0.81493	0.85009	0.71276	3845	1	-0.736965594
hsa04934	Cushing syndrome	33604	155/8521	0.81697	0.85009	0.71276	8323	1	-0.736965594
hsa04217	Necroptosis	33604	159/8521	0.82489	0.85448	0.71645	7186	1	-0.785875195
hsa03008	some biogenesis in eukar	33604	164/8521	0.83431	0.86029	0.72132	10199	1	-0.836501268
hsa04630	AK-STAT signaling pathwa	33604	166/8521	0.83794	0.86029	0.72132	6654	1	-0.836501268
hsa05164	Influenza A	33604	171/8521	0.84667	0.8654	0.72561	3125	1	-0.888968688
hsa03040	Spliceosome	33604	216/8521	0.90699	0.92297	0.77388	1659	1	-1.217591435
hsa04080	active ligand-receptor inte	33635	367/8521	0.91164	0.92363	0.77443	56288/2908	2	-1
hsa04024	cAMP signaling pathway	33604	225/8521	0.91587	0.92387	0.77462	572	1	-1.286304185
hsa04020	Calcium signaling pathway	33604	253/8521	0.93846	0.94254	0.79028	2066	1	-1.434402824
hsa04740	Olfactory transduction	33604	439/8521	0.9925	0.9925	0.83217	83988	1	-2.251538767

Supplementary Table 3. Kyoto Encyclopedia of Genes and Genomes enrichment analysis of upregulated genes in the platinum-sensitive group

ID	Description	GeneRatio	BgRatio	pvalue	p.adjust	qvalue	geneID	Count	Fold_enrichment
hsa04650	killer cell mediated cyt	28338	132/8521	2.3E-05	0.0014	0.00113	7/3683/7305/3937/962/3105/6850/7	8	2.746312766
hsa04666	ma R-mediated phagc	28307	97/8521	2.5E-05	0.0014	0.00113	082/4067/2209/7454/3635/6850/740	7	2.998195503
hsa05135	Yersinia infection	28338	137/8521	3E-05	0.0014	0.00113	16/3937/834/3678/7454/7456/2335/7	8	2.691534165
hsa04664	psilon RI signaling pat	28277	68/8521	3.1E-05	0.0014	0.00113	4067/240/3937/3635/6850/7409	6	3.286881148
hsa05171	navirus disease - COV	28399	232/8521	4.1E-05	0.00145	0.00118	13/834/2162/1536/6850/6159/714/72	10	2.253989266
hsa04612	processing and prese	28277	78/8521	6.9E-05	0.00203	0.00165	3107/1520/10437/3105/1508/5641	6	3.089159132
hsa04610	nent and coagulation c	28277	86/8521	0.00012	0.00301	0.00244	713/11326/3818/2162/714/728	6	2.948600847
hsa05143	frican trypanosomiasi	28216	37/8521	0.00032	0.00722	0.00585	3039/3040/3620/5330	4	3.580145484
hsa05205	Proteoglycans in cance	28338	205/8521	0.0005	0.00981	0.00795	17/967/960/3059/3678/2335/286/74(	8	2.111031312
hsa00770	henate and CoA biosy	28185	21/8521	0.00084	0.01389	0.01125	217/1806/1807	3	3.982765463
hsa04611	Platelet activation	28277	124/8521	0.00086	0.01389	0.01125	4067/3937/5330/83706/6850/6916	6	2.419538892
hsa05144	Malaria	28216	50/8521	0.00104	0.01537	0.01245	3039/3040/3683/4035	4	3.145677455
hsa04142	Lysosome	28277	132/8521	0.00119	0.01628	0.01319	967/1520/8905/1522/1508/5641	6	2.3305584
hsa04613	hil extracellular trap fc	28307	191/8521	0.00163	0.02021	0.01637	3683/2209/834/5330/1536/6850/728	7	2.021479727
hsa05150	hylococcus aureus infe	28246	96/8521	0.0017	0.02021	0.01637	3683/2209/713/714/728	5	2.526068812
hsa05169	stein-Barr virus infecti	28307	202/8521	0.00224	0.02493	0.0202	3107/4067/3683/4939/960/3105/685(	7	1.937344392
hsa04145	Phagosome	28277	152/8521	0.00244	0.02556	0.02071	3107/1520/2209/3105/3678/1536	6	2.12763328
hsa00410	eta-Alanine metabolis	28185	31/8521	0.00266	0.02629	0.0213	217/1806/1807	3	3.420886575
hsa04810	ulation of actin cytoske	28307	229/8521	0.0045	0.04216	0.03416	583/10788/3678/3071/2335/7409/94	7	1.757023247
hsa05133	Pertussis	28216	76/8521	0.00484	0.04295	0.0348	713/834/3678/714	4	2.541019153
hsa05100	ial invasion of epitheli	28216	77/8521	0.00507	0.04295	0.0348	63916/3059/3678/2335	4	2.523561956
hsa00380	Tryptophan metabolism	28185	42/8521	0.00632	0.05117	0.04146	3620/217/8942	3	2.981852653
hsa04662	receptor signaling pa	28216	84/8521	0.00689	0.05333	0.04321	4067/3635/6850/7409	4	2.397802962
hsa04062	mokine signaling path	28277	192/8521	0.00763	0.05657	0.04583	4067/1794/5330/57580/7454/7409	6	1.790772038
hsa04640	ematopoietic cell linea	28216	99/8521	0.01215	0.0865	0.07008	2209/960/952/3678	4	2.160274831
hsa05417	pid and atherosclero	28277	215/8521	0.01289	0.08826	0.07151	817/4067/834/5330/1536/7409	6	1.627606838
hsa04514	ell adhesion molecule	28246	158/8521	0.01393	0.09182	0.07439	3107/3683/1462/3105/6614	5	1.807354922
hsa00240	Pyrimidine metabolism	28185	58/8521	0.01534	0.0975	0.07899	1890/1806/1807	3	2.516015147
hsa05416	Viral myocarditis	28185	60/8521	0.01679	0.10308	0.08351	3107/3683/3105	3	2.46727948
hsa05152	Tuberculosis	28246	180/8521	0.02314	0.13711	0.11108	817/1520/2209/4046/6850	5	1.618238656
hsa05131	Shigellosis	28277	247/8521	0.02388	0.13711	0.11108	63916/960/834/5330/3059/3678	6	1.427606173
hsa04144	Endocytosis	28277	250/8521	0.02515	0.13992	0.11336	3107/3105/2350/7456/1601/30011	6	1.411426246
hsa04621	ke receptor signaling p	28246	186/8521	0.0262	0.14134	0.11451	4939/834/5330/1536/1508	5	1.570462931
hsa04380	steoclast differentiatio	28216	128/8521	0.02834	0.14836	0.1202	7305/2209/3937/6850	4	1.790772038
hsa05167	na-associated herpes	28246	194/8521	0.03068	0.15603	0.12641	3107/4067/57580/3105/6850	5	1.510961919
hsa00983	netabolism - other en	28185	80/8521	0.03549	0.17549	0.14218	1890/1806/1807	3	2.053111336
hsa04936	Alcoholic liver disease	28216	142/8521	0.03928	0.18895	0.15309	713/217/714/728	4	1.641546029
hsa04015	Rap1 signaling pathwa	28246	210/8521	0.04101	0.19212	0.15565	3683/3937/5330/51466/7409	5	1.3950628
hsa05330	Allograft rejection	28157	38/8521	0.04596	0.20621	0.16707	3107/3105	2	2.541019153
hsa04512	CM-receptor interactic	28185	89/8521	0.04634	0.20621	0.16707	960/3678/2335	3	1.89917563
hsa01240	iosynthesis of cofacto	28216	153/8521	0.04935	0.21427	0.17359	3620/217/51004/8942	4	1.531069493
hsa04216	Ferroptosis	28157	41/8521	0.05269	0.22332	0.18092	3162/1536	2	2.432959407
hsa05332	raft-versus-host disea	28157	42/8521	0.05501	0.22772	0.18449	3107/3105	2	2.397802962
hsa04940	Type I diabetes mellitu	28157	43/8521	0.05736	0.23207	0.18801	3107/3105	2	2.364572432
hsa04933	aling pathway in diabe	28185	100/8521	0.06156	0.2435	0.19727	5330/1536/2335	3	1.731183242
hsa05142	Chagas disease	28185	102/8521	0.06455	0.24977	0.20236	713/5330/714	3	1.700439718
hsa04625	ctin receptor signaling	28185	104/8521	0.0676	0.25603	0.20743	834/4046/6850	3	1.673556424
hsa04066	HIF-1 signaling pathwa	28185	109/8521	0.07553	0.27393	0.22193	817/3162/1536	3	1.608809243
hsa04979	Cholesterol metabolism	28157	51/8521	0.07738	0.27393	0.22193	4035/1593	2	2.117695043
hsa05165	an papillomavirus infe	28277	331/8521	0.0781	0.27393	0.22193	3107/4799/3105/3678/2335/4600	6	1.007195501
hsa03266	Virion - Herpesvirus	28126	2418516	0.07849	0.27393	0.22193	3678	1	3.62058641
hsa05320	oimmune thyroid disea	28157	53/8521	0.08269	0.28304	0.22931	3107/3105	2	2.063502942
hsa04670	yte transendothelial m	28185	115/8521	0.08555	0.28733	0.23279	3683/1536/7409	3	1.531069493
hsa00130	other terpenoid-quinc	28126	2418577	0.09508	0.31342	0.25393	51004	1	3.3305584
hsa04730	Long-term depression	28157	60/8521	0.10207	0.32771	0.2655	4067/5330	2	1.883620816
hsa05130	enic Escherichia coli i	28216	198/8521	0.10381	0.32771	0.2655	834/3059/7456/3071	4	1.163498732
hsa00590	chidonic acid metabol	28157	61/8521	0.10494	0.32771	0.2655	240/6916	2	1.859969548
hsa03250	Viral life cycle - HIV-1	28157	63/8521	0.11074	0.33962	0.27515	25939/4600	2	1.811471031
hsa05203	Viral carcinogenesis	28216	204/8521	0.11257	0.33962	0.27515	3107/4067/3105/6850	4	1.117695043
hsa04720	Long-term potentiatior	28157	67/8521	0.12257	0.35767	0.28977	817/5330	2	1.722466024
hsa04210	Apoptosis	28185	136/8521	0.12468	0.35767	0.28977	1520/1522/1508	3	1.286881148
hsa05170	munodeficiency virus	28216	212/8521	0.12475	0.35767	0.28977	3107/25939/8905/3105	4	1.063502942
hsa05322	emic lupus erythemat	28185	137/8521	0.12668	0.35767	0.28977	2209/713/714	3	1.275007047
hsa04924	Renin secretion	28157	69/8521	0.1286	0.35767	0.28977	5330/1508	2	1.682573297
hsa00562	itol phosphate metabc	28157	73/8521	0.14086	0.38575	0.31252	5330/3635	2	1.599317794
hsa00120	nary bile acid biosynth	28126	17/8521	0.14313	0.38601	0.31273	1593	1	2.702657543
hsa04623	solic DNA-sensing pat	28157	75/8521	0.14709	0.39077	0.31659	25939/834	2	1.560714954
hsa04971	Gastric acid secretion	28157	76/8521	0.15022	0.39322	0.31858	817/5330	2	1.541019153
hsa05140	Leishmaniasis	28157	77/8521	0.15337	0.39564	0.32054	2209/1536	2	1.521050737
hsa04921	ytocin signaling pathw	28185	154/8521	0.16244	0.41306	0.33465	817/952/5330	3	1.111031312
hsa04217	Necroptosis	28185	159/8521	0.17349	0.43496	0.35239	817/834/1536	3	1.063502942
hsa00340	Histidine metabolism	28126	22/8521	0.18123	0.44432	0.35998	217	1	2.3305584
hsa04911	Insulin secretion	28157	86/8521	0.18222	0.44432	0.35998	817/5330	2	1.361768359
hsa05410	ertrophic cardiomyop	28157	90/8521	0.1953	0.46825	0.37936	3678/7273	2	1.298658316
hsa04310	Wnt signaling pathway	28185	171/8521	0.20085	0.46825	0.37936	817/7090/5330	3	0.956056652
hsa05164	Influenza A	28185	171/8521	0.20085	0.46825	0.37936	4939/834/4600	3	0.956056652
hsa04912	nRH signaling pathwa	28157	93/8521	0.20519	0.46825	0.37936	817/5330	2	1.250961574
hsa04970	Salivary secretion	28157	93/8521	0.20519	0.46825	0.37936	952/5330	2	1.250961574
hsa05414	Dilated cardiomyopath	28157	96/8521	0.21513	0.47563	0.38535	3678/7273	2	1.207892852
hsa04070	atidylinositol signaling	28157	97/8521	0.21845	0.47563	0.38535	5330/3635	2	1.189033824
hsa04713	Circadian entrainment	28157	97/8521	0.21845	0.47563	0.38535	817/5330	2	1.189033824
hsa04750	mediator regulation of	28157	98/8521	0.22178	0.47563	0.38535	817/5330	2	1.176322773
hsa04925	rone synthesis and se	28157	98/8521	0.22178	0.47563	0.38535	817/5330	2	1.176322773
hsa04916	Melanogenesis	28157	101/8521	0.2318	0.48293	0.39125	817/5330	2	1.13093087
hsa04972	Pancreatic secretion	28157	102/8521	0.23514	0.48293	0.39125	952/5330	2	1.117695043
hsa05146	Amoebiasis	28157	102/8521	0.23514	0.48293	0.39125	5330/2335	2	1.117695043
hsa00053	ate and aldarate meta	28126	30/8521	0.23875	0.48293	0.39125	217	1	1.883620816
hsa01523	Antifolate resistance	28126	30/8521	0.23875	0.48293	0.39125	2350	1	1.883620816
hsa04064	kappa B signaling path	28157	104/8521	0.24184	0.48368	0.39187	4067/6850	2	1.09085343
hsa04922	ucagon signaling pathv	28157	107/8521	0.25191	0.49822	0.40364	817/5330	2	1.049630768
hsa04725	Cholinergic synapse	28157	113/8521	0.27207	0.52792	0.42771	817/5330	2	0.970853654
hsa04510	Focal adhesion	28185	203/8521	0.27782	0.52792	0.42771	3678/2335/7409	3	0.713695815
hsa05415	iabetic cardiomyopath	28185	203/8521	0.27782	0.52792	0.42771	817/5330/1536	3	0.713695815
hsa04726	Serotonergic synapse	28157	115/8521	0.27879	0.52792	0.42771	240/5330	2	0.941106311
hsa00760	e and nicotinamide me	28126	37/8521	0.28579	0.53549	0.43384	952	1	1.580145484
hsa04660	receptor signaling pa	28157	121/8521	0.29893	0.55427	0.44905	3937/7409	2	0.871843649
hsa05206	MicroRNAs in cancer	28216	310/8521	0.30726	0.56383	0.4568	4082/960/3162/3678	4	0.516015147
hsa05219	Bladder cancer	28126	41/8521	0.31138	0.56556	0.4582	1890	1	1.432959407
hsa00071	Fatty acid degradation	28126	43/8521	0.32383	0.57269	0.46398	217	1	1.361768359
hsa00860	Porphyrin metabolism	28126	43/8521	0.32383	0.57269	0.46398	3162	1	1.361768359
hsa05166	T-cell leukemia virus 1	28185	222/8521	0.32495	0.57269	0.46398	3107/3683/3105	3	0.584962501
hsa05163	an cytomegalovirus inf	28185	225/8521	0.33242	0.57998	0.46989	3107/5330/3105	3	0.565597176
hsa04728	Dopaminergic synapse	28157	132/8521	0.33561	0.57998	0.46989	817/5330	2	0.748461233
hsa03050	Proteasome	28126	46/8521	0.34209	0.58448	0.47353	5699	1	1.269033146
hsa00620	Pyruvate metabolism	28126	47/8521	0.34806	0.58448	0.47353	217	1	1.232660757
hsa04973	drate digestion and ab	28126	47/8521	0.34806	0.58448	0.47353	5330	1	1.232660757
hsa00280	ucine and isoleucine d	28126	48/8521	0.35399	0.58575	0.47456	217	1	1.207892852
hsa05162	Measles	28157	138/8521	0.3554	0.58575	0.47456	4939/4600	2	0.678071905
hsa00520	and nucleotide sugar	28126	49/8521	0.35986	0.58766	0.4761	80896	1	1.176322773
hsa00330	ine and proline metab	28126	50/8521	0.36568	0.59173	0.4794	217	1	1.14404637
hsa04913	Ovarian steroidogenesi	28126	51/8521	0.37144	0.59564	0.48257	240	1	1.117695043
hsa04961	er factor-regulated cal	28126	53/8521	0.38282	0.60841	0.49291	5330	1	1.063502942
hsa04072	nolipase D signaling p	28157	148/8521	0.38792	0.61106	0.49506	5330/6850	2	0.584962501
hsa05132	Salmonella infection	28185	249/8521	0.39193	0.61197	0.4958	63916/834/3071	3	0.411426246
hsa05134	Legionellosis	28126	56/8521	0.3995	0.61649	0.49946	834	1	0.98550043
hsa04020	alcium signaling pathw	28185	253/8521	0.40176	0.61649	0.49946	817/952/5330	3	0.389566812
hsa04261	gic signaling in cardior	28157	154/8521	0.4071	0.6184	0.50101	817/5330	2	0.526068812
hsa04934	Cushing syndrome	28157	155/8521	0.41027	0.6184	0.50101	817/5330	2	0.516015147
hsa04218	Cellular senescence	28157	156/8521	0.41343	0.6184	0.50101	3107/3105	2	0.50589093
hsa05160	Hepatitis C	28157	158/8521	0.41973	0.61941	0.50183	4939/4600	2	0.485426827
hsa04978	Mineral absorption	28126	60/8521	0.42106	0.61941	0.50183	3162	1	0.879705766
hsa04330	Notch signaling pathwa	28126	62/8521	0.43155	0.62569	0.50691	7090	1	0.831877241
hsa00310	Lysine degradation	28126	63/8521	0.43673	0.62569	0.50691	217	1	0.815575429
hsa00561	Glycerolipid metabolisr	28126	63/8521	0.43673	0.62569	0.50691	217	1	0.815575429
hsa04929	GnRH secretion	28126	64/8521	0.44185	0.62569	0.50691	5330	1	0.790772038
hsa04927	sol synthesis and secr	28126	65/8521	0.44693	0.62569	0.50691	5330	1	0.765534746
hsa05020	Prion disease	28185	272/8521	0.44777	0.62569	0.50691	713/1536/714	3	0.286881148
hsa04530	Tight junction	28157	170/8521	0.45679	0.62569	0.50691	3059/7454	2	0.378511623
hsa00010	colysis / Gluconeogen	28126	67/8521	0.45696	0.62569	0.50691	217	1	0.722466024
hsa05221	Acute myeloid leukemi	28126	67/8521	0.45696	0.62569	0.50691	2209	1	0.722466024
hsa05031	Amphetamine addictio	28126	69/8521	0.46681	0.63429	0.51388	817	1	0.678071905
hsa05120	gnaling in Helicobacte	28126	70/8521	0.47167	0.63604	0.5153	4067	1	0.659924558
hsa04360	Axon guidance	28157	182/8521	0.49249	0.64827	0.52521	817/1808	2	0.286881148
hsa03320	PPAR signaling pathwa	28126	75/8521	0.49531	0.64827	0.52521	1593	1	0.565597176
hsa04918	yroid hormone synthe	28126	75/8521	0.49531	0.64827	0.52521	5330	1	0.565597176
hsa05214	Glioma	28126	75/8521	0.49531	0.64827	0.52521	817	1	0.565597176
hsa05212	Pancreatic cancer	28126	76/8521	0.49991	0.64952	0.52622	9459	1	0.545968369
hsa05412	nic right ventricular ca	28126	77/8521	0.50447	0.65069	0.52717	3678	1	0.526068812
hsa01232	Nucleotide metabolism	28126	85/8521	0.5395	0.68593	0.55572	1890	1	0.378511623
hsa04012	ErbB signaling pathwa	28126	85/8521	0.5395	0.68593	0.55572	817	1	0.378511623
hsa04742	Taste transduction	28126	86/8521	0.5437	0.68638	0.55608	5330	1	0.367371066
hsa04540	Gap junction	28126	88/8521	0.552	0.69194	0.56059	5330	1	0.333423734
hsa05222	Small cell lung cancer	28126	92/8521	0.56814	0.70228	0.56897	2335	1	0.263034406
hsa04520	Adherens junction	28126	93/8521	0.57208	0.70228	0.56897	7454	1	0.250961574
hsa05323	Rheumatoid arthritis	28126	93/8521	0.57208	0.70228	0.56897	3683	1	0.250961574
hsa05231	line metabolism in ca	28126	98/8521	0.59128	0.72088	0.58403	7454	1	0.176322773
hsa04914	one-mediated oocyte r	28126	102/8521	0.60603	0.73103	0.59226	6793	1	0.111031312
hsa04024	AMP signaling pathwa	28157	225/8521	0.60782	0.73103	0.59226	817/7409	2	-0.029146346
hsa04928	mone synthesis, secre	28126	106/8521	0.62024	0.74096	0.60031	5330	1	0.056583528
hsa04151	3K-Akt signaling pathw	28185	354/8521	0.6263	0.74321	0.60213	3678/6850/2335	3	-0.089267338
hsa05145	Toxoplasmosis	28126	111/8521	0.63731	0.75126	0.60865	240	1	0
hsa04724	Glutamatergic synapse	28126	115/8521	0.65041	0.76167	0.61708	5330	1	-0.058893689
hsa04722	otrophin signaling pat	28126	119/8521	0.66305	0.76357	0.61862	817	1	-0.104697379
hsa04935	one synthesis, secreti	28126	120/8521	0.66614	0.76357	0.61862	5330	1	-0.120294234
hsa04071	ngolipid signaling path	28126	121/8521	0.6692	0.76357	0.61862	5330	1	-0.13606155
hsa04919	d hormone signaling p	28126	121/8521	0.6692	0.76357	0.61862	5330	1	-0.13606155
hsa05010	Alzheimer disease	28185	384/8521	0.68115	0.77226	0.62566	5330/1536/4035	3	-0.217591435
hsa04926	elaxin signaling pathw	28126	129/8521	0.6927	0.78038	0.63225	5330	1	-0.217591435
hsa04114	Oocyte meiosis	28126	131/8521	0.69831	0.78176	0.63336	817	1	-0.251538767
hsa04270	ar smooth muscle con	28126	134/8521	0.70655	0.78601	0.6368	5330	1	-0.268816758
hsa04915	trogen signaling pathw	28126	137/8521	0.71455	0.78601	0.6368	5330	1	-0.304006187
hsa04371	pelin signaling pathwa	28126	139/8521	0.71977	0.78601	0.6368	5330	1	-0.321928095
hsa05418	ear stress and atheros	28126	139/8521	0.71977	0.78601	0.6368	3162	1	-0.321928095
hsa04140	Autophagy - animal	28126	141/8521	0.7249	0.78678	0.63743	1508	1	-0.358453971
hsa05017	Spinocerebellar ataxia	28126	143/8521	0.72993	0.78744	0.63796	5330	1	-0.377069649
hsa04723	ade endocannabinoid s	28126	148/8521	0.74212	0.79577	0.64471	5330	1	-0.415037499
hsa03010	Ribosome	28126	167/8521	0.78368	0.82751	0.67043	6159	1	-0.59946207
hsa04022	MP-PKG signaling path	28126	167/8521	0.78368	0.82751	0.67043	5330	1	-0.59946207
hsa05225	epatocellular carcinom	28126	168/8521	0.78567	0.82751	0.67043	3162	1	-0.59946207
hsa05022	eurodegeneration - mu	28185	476/8521	0.81233	0.85056	0.6891	817/5330/1536	3	-0.514573173
hsa05202	ptional misregulation i	28126	193/8521	0.83003	0.864	0.69999	2209	1	-0.810966176
hsa05208	inogenesis - reactive c	28126	223/8521	0.87143	0.90183	0.73063	3162	1	-1
hsa05012	Parkinson disease	28126	266/8521	0.91398	0.94039	0.76188	817	1	-1.251538767
hsa04060	e-cytokine receptor int	28126	297/8521	0.9357	0.95705	0.77538	3603	1	-1.434402824
hsa05016	Huntington disease	28126	306/8521	0.94092	0.95705	0.77538	5330	1	-1.473931188
hsa05014	yotrophic lateral scler	28126	364/8521	0.96585	0.97227	0.78771	834	1	-1.736965594
hsa04080	tive ligand-receptor in	28126	367/8521	0.96681	0.97227	0.78771	728	1	-1.736965594
hsa04740	Olfactory transduction	28126	439/8521	0.98329	0.98329	0.79663	817	1	-2

Supplementary Table 4. List of gene symbols manually annotated to the ssGSEA pathway

MANUAL_DORSAL_RUFFLE_FORMATION	Activated B cell	Activated CD4 T cell	Activated CD8 T cell	Activated dendritic cell
DNM2	ADAM28	AIM2	ADRM1	ABCD1
SH3YL1	CD180	BIRC3	AHSA1	C1QC
INPPL1	CD79B	BRIP1	C1GALT1C1	CAPG
PLEKHA1	BLK	CCL20	CCT6B	CCL3L3
	CD19	CCL4	CD37	CD207
	MS4A1	CCL5	CD3D	CD302
	TNFRSF17	CCNB1	CD3E	ATP5B
	IGHM	CCR7	CD3G	ATP5L
	GNG7	DUSP2	CD69	ATP6V1A
	MICAL3	ESCO2	CD8A	BCL2L1
	SPIB	ETS1	CETN3	C1QB
	HLA-DOB	EXO1	CSE1L	SNURF
	IGKC	EXOC6	GEMIN6	SPCS3
	PNOC	IARS	GNLY	CCNA1
	FCRL2	ITK	GPT2	CEACAM8
	BACH2	KIF11	GZMA	NOS2
	CR2	KNTC1	GZMH	SRA1
	TCL1A	NUF2	GZMK	TNFRSF6B
	AKNA	PRC1	IL2RB	TREM1
	ARHGAP25	PSAT1	LCK	TREML1
	CCL21	RGS1	MPZL1	RHOA
	CD27	RTKN2	NKG7	SLC25A37
	CD38	SAMSN1	PIK3IP1	TNFSF14
	CLEC17A	SELL	PTRH2	TREML4
	CLEC9A	TRAT1	TIMM13	VNN2
	CLECL1		ZAP70	XPO6
				CLEC4C
				TNFAIP2
				UBD
				ACTR3
				RAB1A
				SLA
				HLA-DQA2
				SIGLEC5
				SLAMF9

CD56bright natural killer cell	CD56dim natural killer cell	Central memory CD4 T cell	Central memory CD8 T cell	Effector memeory CD4 T cell
ABAT	CYP27A1	ABHD3	ACTN4	ATM
C11orf75	DDX55	AHNAK	ADAM12	CASP3
C5orf15	DYRK2	ANXA2P2	ADCY9	CASQ1
CDHR1	RPL37A	AQP3	F13A1	CD300E
DCAF12	NOTCH3	ATHL1	FCER1G	DARS
DYNLL1	AKR7A3	BMI1	FCGR3B	DOCK9
GPR137B	GPRC5C	BZW2	FGF7	EXOSC9
HCP5	GRIN1	CD63	FKBP4	EZH2
HDGFRP2	HLA-E	COL4A1	GLUD1	GDE1
KRT86	PORCN	CYLD	GM2A	IL34
MLST8	PSMC4	ELMO2	GUSB	NCOA4
ELMOD3	UPP1	FYN	IL1RN	NEFL
ENTPD5	IL21R	GLIPR1	NOL11	PDGFRL
FAM119A	KIR2DS1	GSS	NTRK1	PTGS1
FAM179A	KIR2DS2	IFITM2	RARA	REPS1
CLIC2	KIR2DS5	ITGB1	RNF128	SCG2
COX7A2L		ITGB2	SIGLEC1	SDPR
CREB3L4		KLF5	TNFRSF11A	SIGLEC14
CSF1		LSP1	TOX4	SIGLEC6
CSNK2A2		NDUFB9	UBA52	TAL1
CSTA		PKM2	ULBP1	TFEC
CSTB		SFXN3		TIPIN
CTPS		SIRPG		TPK1
CTSD		SMAD4		UQCRB
FST		STX4		USP9Y
GATA2		TRADD		WIPF1
GMPR		VIM		ZCRB1
HDC		XRCC6		
HEY1				
HOXA1				
HS2ST1				
HS3ST1				
BCL11B				
CDH3				
MYL6B				
NAA16				
CIQA				
CIQB				
CYP27B1				
EIF3M				

Effector memeory CD8 T cell	Eosinophil	Gamma delta T cell	Immature B cell	Immature dendritic cell
ACAP1	GIPR	ACP5	CD22	ACADM
APOL3	KRT18P50	AQP9	CYBB	AHCYL1
ARHGAP10	LRMP	BTN3A2	FAM129C	ALDH1A2
ATP10D	FOSB	C1orf54	FCRL1	ALDH3A2
C3AR1	RRP12	CARD8	FCRL3	ALDH9A1
CCR5	GPR183	CCL18	FCRL5	ALOX15
CD160	NR4A3	CD209	FCRLA	AMT
CD55	ST3GAL6	CD33	HDAC9	ARL1
CFLAR	DEPDC5	CD36	HLA-DQA1	ATIC
CMKLR1	PDE6C	CDK5	HVCN1	ATP5A1
DAPP1	PKD2L2	IL10RB	KIAA0226	CAPZA1
FCRL6	GPR65	KLRF1	NCF1	LILRA5
FLT3LG	IL5RA	LGALS1	NCF1B	RDX
GZMM	P2RY14	MAPK7	P2RY10	RRAGD
HAPLN3	DACH1	KLHL7	SP100	TACSTD2
HLA-DMB	DAPK2	KRT80	TXNIP	INPP5F
HLA-DPA1	EMR3	LAMC1	STAP1	RAB38
HLA-DPB1		LCORL	TAGAP	PLAU
IFI16		LMNB1	ZCCHC2	CSF3R
LIME1		MEIS3P1		SLC18A2
LTK		MPL		AMPD2
NFKBIA		FABP1		CLTB
SETD7		FABP5		C1orf162
SIK1		FADD		
TRIB2		MFAP3L		
		MINPP1		
		RPS24		
		RPS7		
		RPS9		
		DBNL		
		CCL13		

Macrophage	Mast cell	MDSC	Memory B cell	Monocyte
AIF1	ADAMTS3	CCR2	AICDA	ASGR2
CCL1	CPA3	CD14	CCNA2	CFP
CCL14	CMA1	CD2	CDKN3	ASGR1
CCL23	CTSG	CD86	CLCN5	CD1D
CCL26	ARHGAP15	CXCR4	ENPP1	UPK3A
CD300LB	CPM	FCGR2A	FCER1A	ACTG1
CNR1	FCN1	FCGR2B	FCRL4	ANXA5
CNR2	FTL	FCGR3A	MYC	ATP6V1B2
EIF1	HSPA6	FERMT3	RUNX2	CFL1
EIF4A1	ITGA9	GPSM3	SORL1	DAZAP2
FPR1	RNASE3	IL18BP	SOX5	CTBS
FPR2	S100A4	IL4R	STAT5A	EMR4P
FRAT2	SIGLEC8	ITGAL	STAT5B	HIVEP2
GPR27	SLC8A4	ITGAM	TLR9	MARCKSL1
GPR77	PTGS2	PARVG		MBP
RNASE2	EGR3	PSAP		MMP15
MS4A2	PILRA	PTGER2		PNPLA6
BASP1		PTGES2		TMBIM6
IGSF6		S100A8		PQBP1
HK3		S100A9		TEX264
VNN1				IKZF1
FES				
NPL				
FZD2				
FAM198B				
HNMT				
SLC15A3				
CD4				
TXNDC3				
FRMD4A				
CRYBB1				
HRH1				
WNT5B				

Natural killer cell	Natural killer T cell	Neutrophil	Plasmacytoid dendritic cell	Regulatory T cell
AKT3	BTN2A2	CREB5	CBX6	CCL3L1
AXL	CD101	CDA	DAB2	CD72
BST2	CD109	CHST15	DDX17	CLEC5A
CDH2	CNPY3	S100A12	HIGD1A	FOXP3
CRTAM	CNPY4	APOBEC3A	IDH3A	ITGA4
CSF2RA	CREB1	CASP5	IL3RA	L1CAM
CTSZ	CRTC2	MMP25	MAGED1	LIPA
CXCL1	CRTC3	HAL	NUCB2	LRP1
CYTH1	CSF2	C1orf183	OFD1	LRRC42
DAXX	KLRC1	FFAR2	OGT	MARCO
DGKH	FUT4	MAK	PDIA4	MMP12
DLL4	ICAM2	CXCR1	SERTAD2	MNDA
DPYD	IL32	STEAP4	SIRPA	MRC1
ERBB3	LAMP2	MGAM	TMED2	MS4A6A
F11R	LILRB5	BTNL8	ENG	PELO
FAM27A	KLRG1	CXCR2	FCAR	PLEK
FAM49A	HSPA4	TNFRSF10C	IGF1	PRSS23
FASLG	HSPB6	VNN3	ITGA2B	PTGIR
FCGR1A	ISM2		GABARAP	ST8SIA4
FN1	ITIH2		GPX1	STAB1
FSTL1	KDM4C		KRT23	
FUCA1	KIR2DS4		PROK2	
GBP3	KIRREL3		RALB	
GLS2	SDCBP		RETNLB	
GRB2	NFATC2IP		RNF141	
LST1	MICB		SEC14L1	
BCL2	KIR2DL1		SEPX1	
CDC5L	KIR2DL3		EMP3	
FGF18	KIR3DL1		CD300LF	
FUT5	KIR3DL2		ABTB1	
FZR1	NCR1		KLHL21	
GAGE2	FOSL1		PHRF1	
IGFBP5	TSLP			
KANK2	SLC7A7			
LDB3	SPP1			
	TREM2			
	UBASH3A			
	YBX2			
	CCDC88A			
	CLEC1A			
	THBD			
	PDPN			
	VCAM1			
	EMR1			

T follicular helper cell	Type 1 T helper cell	Type 17 T helper cell	Type 2 T helper cell
B3GAT1	CD70	IL17A	ASB2
CDK5R1	TBX21	IL17RA	CSRP2
PDCD1	ADAM8	C2CD4A	DAPK1
BCL6	AHCYL2	C2CD4B	DLC1
CD200	ALCAM	CA2	DNAJC12
CD83	B3GALNT1	CCDC65	DUSP6
CD84	BBS12	CEACAM3	GNAI1
FGF2	BST1	IL17C	LAMP3
GPR18	CD151	IL17F	NRP2
CEBPA	CD47	IL17RC	OSBPL1A
CECR1	CD48	IL17RE	PDE4B
CLEC10A	CD52	IL23A	PHLDA1
CLEC4A	CD53	ILDR1	PLA2G4A
CSF1R	CD59	LONRF3	RAB27B
CTSS	CD6	SH2D6	RBMS3
DMN	CD68	TNIP2	RNF125
DPP4	CD7	ABCA1	TMPRSS3
LRRC32	CD96	ABCB1	GATA3
MC5R	CFHR3	ADAMTS12	BIRC5
MICA	CHRM3	ANK1	CDC25C
NCAM1	CLEC7A	ANKRD22	CDC7
NCR2	COL23A1	B3GALT2	CENPF
NRP1	COL4A4	CAMTA1	CXCR6
PDCD1LG2	COL5A3	CCR9	DHFR
PDCD6	DAB1	CD40	EVI5
PRDX1	DLEU7	GPR44	GSTA4
RAE1	DOC2B	IFT80	HELLS
RAET1E	EMP1		IL26
SIGLEC7	F12		LAIR2
SIGLEC9	FURIN		
TYRO3	GAB3		
CHST12	GATM		
CLIC3	GFPT2		
IVNS1ABP	GPR25		
KIR2DL2	GREM2		
LGMN	HAVCR1		
	HSD11B1		
	HUNK		
	IGF2		
	RCSD1		
	RYR1		
	SAV1		
	SELE		
	SELP		
	SH3KBP1		
	SIT1		
	SLC35B3		
	SIGLEC10		
	SKAP1		
	THUMPD2		
	TIGIT		
	ZEB2		
	ENC1		
	FAM134B		
	FBXO30		
	FCGR2C		
	STAC		
	LTC4S		
	MAN1B1		
	MDH1		
	MMD		
	RGS16		
	IL12A		
	P2RX5		
	CD97		
	ITGB4		
	ICAM3		
	METRNL		
	TNFRSF1A		
	IRF1		
	HTR2B		
	CALD1		
	MOCOS		
	TRAF3IP2		
	TLR8		
	TRAF1		
	DUSP14		

Supplementary Table 5. Expression spectrum of the ssGSEA-transformed platinum dataset annotated by c2.all.v2023.2.Hs.symbols.gmt

	ABBUD_LIF_SIGNALING_1_DN	F_SIGNAL	F_SIGNAL	EN_ELAV	KIDNEY_C	_INNER_	EGFA_TAR	FA_TARG	FA_TARGE	S_MULTI	S_MULTI	INTERACT	LIVER_C	LIVER_C	ANCER_W	ANCER_V
HGSOC1	0.162481784	0.09025	0.22488	0.18499	0.06687	0.2249	0.16569	0.06778	0.0716	0.17733	0.03849	0.26416	0.08047	0.08076	0.24849	-0.00601
HGSOC2	0.152503541	0.0217	0.22593	0.19358	0.02376	0.22281	0.05127	0.05081	0.04856	0.2467	0.02864	0.29805	0.05614	0.08654	0.24472	-0.01979
HGSOC3	0.221782696	0.09303	0.20826	0.21851	0.16261	0.23583	0.09534	0.08801	0.11026	0.12272	0.08344	0.31796	0.07616	0.07754	0.24615	0.00879
HGSOC4	0.112133193	0.17431	0.27194	0.195	0.14574	0.28187	0.08359	0.10232	0.09023	0.114	0.12735	0.26714	0.09308	0.07966	0.25305	0.02679
HGSOC5	0.151738542	0.16094	0.1738	0.18581	0.14887	0.24535	0.08416	0.05157	0.14665	0.18406	0.10421	0.26964	0.08011	0.07749	0.26132	0.0118
HGSOC6	0.184014305	0.09597	0.17355	0.20455	0.0817	0.26815	0.06072	0.07474	0.13094	0.21444	0.11943	0.30346	0.11973	0.07863	0.24062	-0.01803
HGSOC7	0.127949854	0.14283	0.11021	0.21641	-0.02464	0.24725	0.12657	0.04774	0.04389	0.16515	0.1395	0.28036	0.10871	0.07951	0.24226	-0.01663
HGSOC8	0.15289423	0.17386	0.19257	0.21834	0.20362	0.25113	0.013	0.07131	0.06316	0.11506	0.14026	0.21556	0.1038	0.08213	0.24886	0.04258
HGSOC9	0.19080507	0.16226	0.2383	0.26466	0.17491	0.28144	0.16559	0.07718	0.13946	0.17063	0.13513	0.23665	0.10069	0.07577	0.2466	0.01409
HGSOC10	0.099119734	0.17645	0.1987	0.20578	0.15834	0.23821	0.00607	0.01508	0.07349	0.17466	0.14959	0.23923	0.10527	0.07944	0.24302	0.00744
HGSOC11	0.134742673	0.1473	0.13244	0.20716	-0.02269	0.24225	0.19554	0.09751	0.0716	0.1479	0.01282	0.32402	0.07493	0.08789	0.24433	0.00832
HGSOC12	0.241829731	0.16024	0.15025	0.20914	0.01931	0.2379	0.11041	0.07723	0.13632	0.14972	0.05656	0.30281	0.08936	0.08944	0.27727	0.01997
HGSOC13	0.2306874	0.03474	0.11178	0.20833	-0.04292	0.21274	0.10501	0.11616	0.05585	0.16177	-0.01311	0.3095	0.0818	0.07862	0.25265	-0.01749
HGSOC14	0.12647086	0.17499	0.25475	0.19719	0.15252	0.25666	0.01119	0.06771	0.09513	0.17176	0.07192	0.25736	0.07861	0.08406	0.25293	-0.01044
HGSOC15	0.15244701	0.13268	0.21871	0.19324	0.08237	0.2788	0.20377	0.09437	0.06785	0.13347	0.04445	0.28062	0.082	0.08373	0.23855	-0.0148
HGSOC16	0.185586699	0.10934	0.19344	0.19524	-0.05756	0.22601	0.11407	0.11762	0.06602	0.19556	-0.08213	0.31242	0.07406	0.08555	0.22815	-0.01027
HGSOC17	0.170798183	0.13419	0.23807	0.20353	0.07792	0.26838	0.08013	0.09198	0.08903	0.16492	0.11872	0.24354	0.08625	0.07911	0.24496	0.02016
HGSOC18	0.160523493	0.14839	0.16324	0.21301	0.14494	0.25491	0.03428	0.00513	0.06445	0.11658	0.10184	0.24471	0.08599	0.07995	0.24474	0.03353

	ANCER_V	ANCER_W	S_NORM	S_NORM	ATED_IN_LE	ADJACE	ADJACE	INDEPEN	INDEPEN	DIFFERE	YY1_TARG	YY1_TARG	T_PATHW	_CANCE	_CANCE
HGSOC1	0.29432	-0.00712	0.01472	0.09817	0.02827	0.13823	0.14701	0.099	0.05667	0.13916	0.09567	0.05622	0.01715	0.06463	0.10449
HGSOC2	0.27695	-0.03558	-0.01077	0.10347	0.02461	0.12249	0.15073	0.08287	0.09871	0.12382	0.06272	0.04736	0.01187	0.08245	0.10324
HGSOC3	0.28869	-0.03012	-6.3E-05	0.09187	0.03794	0.11965	0.14512	0.09501	0.07845	0.1427	0.10707	0.04483	-0.00537	0.06846	0.11597
HGSOC4	0.30299	0.02376	0.02472	0.09187	0.0529	0.11686	0.15036	0.12768	0.0666	0.18151	0.07727	0.06033	0.03622	0.06708	0.09955
HGSOC5	0.28393	0.03074	0.00445	0.08587	0.06837	0.12943	0.16041	0.12864	0.08314	0.13647	0.08425	0.03947	-0.08138	0.06711	0.08684
HGSOC6	0.29262	0.0062	0.04094	0.08961	0.05187	0.13208	0.16691	0.10434	0.10303	0.09198	0.12046	0.05565	-0.1688	0.07686	0.10491
HGSOC7	0.28627	-0.02851	0.03587	0.08669	0.04924	0.12735	0.15383	0.09123	0.09726	0.1199	0.10923	0.05227	0.04883	0.07332	0.09632
HGSOC8	0.28281	0.07187	0.0307	0.08929	0.05939	0.1156	0.15767	0.1477	0.0442	0.19195	0.04799	0.05798	0.02054	0.07197	0.08884
HGSOC9	0.28935	0.05404	0.02766	0.08644	0.06941	0.10981	0.15505	0.14329	0.05534	0.17963	0.0726	0.07613	0.06908	0.07247	0.07857
HGSOC10	0.28594	0.04151	0.03498	0.08519	0.06097	0.12496	0.14763	0.13001	0.05932	0.18132	0.07081	0.04958	-0.00689	0.07135	0.08206
HGSOC11	0.29759	-0.04884	0.00456	0.10254	0.0208	0.11099	0.13847	0.06647	0.08033	0.08241	0.12513	0.08074	-0.06522	0.05808	0.11242
HGSOC12	0.29943	-0.02735	0.01946	0.1092	0.02028	0.13994	0.16067	0.06073	0.08203	0.10858	0.10757	0.07231	-0.11986	0.0651	0.11497
HGSOC13	0.27669	-0.05342	0.01545	0.10142	0.02064	0.12647	0.1396	0.08064	0.05569	0.10573	0.10394	0.04214	-0.15531	0.0888	0.12529
HGSOC14	0.29865	0.02654	-0.00031	0.09214	0.03783	0.11333	0.15489	0.12218	0.05016	0.16356	0.0651	0.06082	-0.05619	0.07311	0.10216
HGSOC15	0.30315	0.0152	0.00686	0.09943	0.03091	0.12809	0.15746	0.08892	0.07502	0.11793	0.09413	0.06834	-0.0303	0.06975	0.09298
HGSOC16	0.28229	-0.07721	0.00211	0.10605	0.016	0.13198	0.12969	0.03299	0.09047	0.09074	0.10768	0.06581	-0.08967	0.06937	0.11968
HGSOC17	0.2957	0.06597	0.00982	0.09339	0.04433	0.11767	0.15776	0.10881	0.06483	0.15351	0.04749	0.06026	0.01352	0.08556	0.10952
HGSOC18	0.28559	0.03872	0.02041	0.0932	0.05542	0.11507	0.15163	0.13842	0.05774	0.17484	0.06821	0.04807	-0.2941	0.07383	0.09511

	ZEB1_T	BRA1_TAR	BRA1_TAR	V1_INFEC	V1_INFEC	NPM1_L	NPM1_L	LA_APOP	D_MYC_TA	METAST	ETASTASI	TASTASIS	_METAST	PS_WITH
HGSOC1	0.1885	0.12237	0.12802	0.06733	0.2203	0.0762	0.04329	0.18708	0.16433	0.03724	0.13194	-0.0199	0.17636	0.04138
HGSOC2	0.11518	0.14629	0.08417	0.024	0.19179	0.0715	0.00672	0.15826	0.17532	0.09989	0.152	0.07967	0.16434	0.03241
HGSOC3	0.12806	0.16694	0.09455	0.05055	0.18331	0.08786	0.03001	0.16328	0.1619	0.10831	0.13762	-0.02939	0.1592	0.0587
HGSOC4	0.08747	0.14039	0.13951	0.0585	0.20601	0.10601	0.03739	0.16764	0.16458	0.17761	0.13596	0.06108	0.16431	0.12872
HGSOC5	-0.00729	0.16881	0.07165	0.07226	0.21177	0.09406	0.0016	0.17612	0.15219	0.1347	0.10133	-0.00235	0.14972	0.08149
HGSOC6	0.03372	0.14763	0.15141	0.05501	0.20685	0.0999	0.05677	0.16727	0.16887	0.13233	0.16008	0.05979	0.16518	0.01684
HGSOC7	0.07463	0.13695	0.13723	0.0241	0.19863	0.11586	0.03673	0.17754	0.17589	0.17482	0.15093	0.03243	0.16411	0.08895
HGSOC8	-0.01815	0.15461	0.10005	0.06334	0.2072	0.11679	0.05686	0.16432	0.14794	0.11277	0.15803	0.06079	0.16337	0.12559
HGSOC9	-0.08933	0.16305	0.10353	0.07811	0.21069	0.12972	0.05394	0.1746	0.14591	0.17627	0.15618	0.06102	0.16054	0.17358
HGSOC10	-0.05472	0.12538	0.07815	0.073	0.2103	0.12582	0.02686	0.18649	0.15108	0.18061	0.12987	0.02846	0.15836	0.14807
HGSOC11	0.14489	0.17885	0.15149	0.05679	0.19526	0.07638	0.05595	0.18368	0.16846	0.02411	0.12594	0.04403	0.15428	-0.02023
HGSOC12	0.15414	0.13944	0.16648	0.06358	0.21304	0.09105	0.01418	0.18481	0.15685	0.13675	0.13278	0.02981	0.16269	0.02797
HGSOC13	0.23116	0.14255	0.10961	0.07019	0.18072	0.0573	0.06068	0.17006	0.17747	0.029	0.15015	0.04837	0.16852	-0.04039
HGSOC14	0.14058	0.1567	0.10781	0.06016	0.19976	0.07487	0.0331	0.17046	0.16476	0.06427	0.13659	-0.00909	0.15518	0.06675
HGSOC15	0.13061	0.14455	0.15566	0.03865	0.19535	0.09845	0.0508	0.16328	0.1738	0.08034	0.15778	-0.02588	0.16725	0.06183
HGSOC16	0.22948	0.13143	0.09469	0.00561	0.18982	0.0663	0.00511	0.18065	0.1806	0.10175	0.09088	-0.01936	0.15749	-0.02671
HGSOC17	0.13444	0.11963	0.11123	0.0474	0.22307	0.06368	0.03554	0.16502	0.17453	0.06526	0.15595	0.07305	0.17032	0.03781
HGSOC18	0.05251	0.14771	0.10198	0.06043	0.21768	0.12179	0.03873	0.17969	0.14694	0.23248	0.15174	0.02571	0.16311	0.12005

	PIRIDOL	ED_EAR	ESPONSE	ESPONSE	RESPONSE	SPONSE	ESPONSE	SPONSE	RESPONSE	SPONSE	ESPONSE	SPONSE	RESPONSE	SPONSE	RESPONS
HGSOC1	0.00474	-0.08031	-0.05631	0.03955	-0.25028	0.18671	0.13331	-0.07208	-0.08134	0.07163	0.09313	-0.01493	0.09189	0.05269	0.02622
HGSOC2	-0.03978	-0.11862	-0.05138	0.04578	-0.32037	0.20006	0.09537	-0.05997	-0.06967	0.0236	0.07288	-0.06237	0.08027	0.06264	0.02005
HGSOC3	0.00731	-0.13774	-0.05217	0.05401	-0.22308	0.1516	0.10746	-0.07751	-0.09448	0.03529	0.08218	-0.01168	0.10813	0.02058	0.04855
HGSOC4	0.00462	-0.1511	-0.05915	0.05374	-0.15031	0.2346	0.1267	0.05071	-0.09751	0.02599	0.08235	-0.0203	0.08855	0.01101	0.02623
HGSOC5	0.00954	-0.20972	-0.06033	0.04809	-0.23685	0.20362	0.09854	-0.00179	-0.09705	0.01012	0.0548	-0.03792	0.06176	-0.03013	0.02548
HGSOC6	0.047	-0.3286	-0.11044	0.04375	-0.2117	0.19817	0.10235	-0.16457	-0.10056	-0.04694	0.06609	-0.07593	0.04564	0.06736	-0.00554
HGSOC7	0.00712	-0.26492	-0.07672	0.04663	-0.15469	0.21074	0.09799	-0.12946	-0.18218	0.02242	0.07969	-0.001	0.09374	0.01843	-0.01422
HGSOC8	0.01304	-0.14816	-0.07279	0.0906	-0.12882	0.22595	0.11951	0.05941	-0.12499	0.03594	0.06473	-0.01376	0.08402	-0.0008	0.03936
HGSOC9	0.00269	-0.03307	-0.05567	0.09374	-0.23438	0.24659	0.09238	-0.0381	-0.0575	0.05777	0.05991	-0.01711	0.07855	0.06971	0.08114
HGSOC10	-0.01165	-0.20365	-0.04981	0.0539	-0.11507	0.21709	0.10568	-0.07264	-0.10405	0.00599	0.05213	-0.0284	0.06136	-0.03567	0.03863
HGSOC11	0.01786	-0.19933	-0.02583	0.07587	-0.08095	0.19445	0.12064	-0.06961	-0.17482	0.00611	0.08745	-0.0178	0.10364	0.03783	-0.00735
HGSOC12	0.01006	-0.31144	-0.01552	0.06501	-0.13671	0.21356	0.12687	0.00061	-0.16433	0.0738	0.07627	-0.05502	0.09073	0.07614	0.03971
HGSOC13	0.0454	-0.16104	-0.01325	0.06965	0.00924	0.1729	0.15554	-0.0069	-0.10605	0.01022	0.08414	-0.03876	0.12372	-0.02119	0.044
HGSOC14	0.01085	-0.10581	-0.04851	0.05712	-0.12804	0.2055	0.10783	-0.07285	-0.09509	0.0036	0.06458	-0.01478	0.12491	0.02511	0.02606
HGSOC15	0.02213	-0.16516	-0.02268	0.07441	-0.1242	0.18095	0.10997	-0.06233	-0.12312	-0.00904	0.06463	-0.02959	0.08538	0.07247	0.04021
HGSOC16	0.00323	-0.1178	0.00352	0.09722	-0.11756	0.10345	0.16842	0.03914	-0.18743	-0.00459	0.07641	-0.05403	0.0971	0.10546	0.06024
HGSOC17	0.02233	-0.1459	-0.08604	0.05253	-0.2387	0.15655	0.11235	-0.00527	-0.09879	0.04592	0.07523	-0.07007	0.08124	0.0098	0.01633
HGSOC18	-0.01272	-0.21893	-0.06137	0.058	-0.27879	0.20446	0.10274	-0.01047	-0.10942	0.03211	0.07441	-0.02633	0.08867	0.0244	0.03748

	RESPONS	RESPONS	RESPONS	RESPONS	RESPONS	_DAMAGE	ESPONSE	CER_IN	ANGIOCA	ANGIOCA	ER_CANC	ER_CAN	ATINIB_R	INSE_TO
HGSOC1	-0.13731	0.06891	0.00291	0.06978	0.01466	-0.00963	0.06468	-0.10803	0.08248	0.03604	0.05844	0.31689	0.18083	0.22061
HGSOC2	-0.17472	0.06488	0.05206	0.11984	-0.03935	0.01506	0.05836	-0.07308	0.06914	0.0015	0.04055	0.34099	0.15159	0.21212
HGSOC3	-0.03527	0.10051	0.0612	0.1393	-0.0627	0.04958	0.06973	-0.01718	0.05174	0.02091	0.05441	0.33599	0.20645	0.25612
HGSOC4	-0.07567	0.07243	0.0979	0.08481	-0.03374	0.0269	0.08038	-0.01481	0.0534	0.03208	0.07672	0.32748	0.29055	0.21607
HGSOC5	-0.01295	0.02062	0.04592	0.09683	-0.10695	-0.01751	0.06136	-0.09436	0.00493	-0.03719	0.09002	0.33358	0.22226	0.21974
HGSOC6	-0.05013	0.02447	0.02244	0.0615	-0.08752	0.05768	0.05993	0.03858	0.10553	-0.05556	0.10805	0.3348	0.16627	0.24577
HGSOC7	-0.22741	0.05444	0.03959	0.07804	-0.05504	0.14564	0.06197	0.1604	0.07698	-0.00232	0.09899	0.34237	0.19633	0.24247
HGSOC8	-0.1022	0.09044	0.12816	0.08578	-0.05363	0.04347	0.08752	0.05794	0.08086	-0.01956	0.10027	0.33291	0.28448	0.22763
HGSOC9	-0.07909	0.0671	0.13812	0.06811	-0.04247	0.02138	0.07868	0.05049	-0.00076	-0.00239	0.10282	0.32618	0.30403	0.21396
HGSOC10	-0.12005	0.04293	0.08143	0.07728	-0.0913	0.0507	0.07438	-0.00592	0.03023	-0.00128	0.11705	0.32778	0.27813	0.22019
HGSOC11	-0.09196	0.08979	0.02322	0.137	-0.00604	-0.03966	0.06021	-0.1115	0.06976	0.05541	0.0664	0.32877	0.05831	0.24943
HGSOC12	-0.08511	0.07504	0.02116	0.0984	-0.01577	0.03424	0.0484	0.03291	0.04424	0.02297	0.06229	0.33039	0.17286	0.25652
HGSOC13	-0.08948	0.08782	0.09335	0.12853	-0.05756	0.10356	0.04784	-0.07463	-0.00096	0.06902	0.04837	0.3135	0.07502	0.22508
HGSOC14	-0.18174	0.05801	0.05743	0.13113	-0.0406	0.02325	0.08737	-0.02261	0.05922	0.05848	0.08137	0.33812	0.23431	0.21641
HGSOC15	-0.11154	0.08985	0.01333	0.10901	-0.04315	-0.0234	0.07148	-0.00952	0.08733	0.018	0.08736	0.34363	0.18417	0.2289
HGSOC16	-0.00742	0.13711	0.05954	0.16192	-0.00037	0.06344	0.05691	-0.11075	0.03987	0.05669	0.04941	0.32257	0.04002	0.239
HGSOC17	-0.0865	0.08783	0.07989	0.0915	-0.03855	-0.01657	0.07041	-0.02833	0.05504	0.0681	0.05903	0.34081	0.23532	0.24763
HGSOC18	-0.04125	0.0704	0.09214	0.06573	-0.03096	0.05825	0.06003	-0.01699	0.03781	-0.01697	0.09415	0.32757	0.26795	0.22092

	NSE_TO	VA_BRC	BLASTOM	TEGRIN	PRESSIVE	PRESSIV	AND_IL17	ASTRIC	RANSF	RANSFOR	STAT3_TA	E_TO_AL	E_TO_AL	ETIC_NEP	ETIC_NEP
HGSOC1	0.1463	0.28076	0.06512	0.08971	0.09485	-0.02063	-0.22537	0.08701	0.06423	0.09505	0.20021	0.25086	0.04914	0.13559	0.14913
HGSOC2	0.16634	0.27368	0.12171	0.09803	0.10048	-0.01296	-0.42931	0.0653	0.01799	0.09419	0.21245	0.29514	0.0414	0.13892	0.10446
HGSOC3	0.14018	0.28488	0.08748	0.10224	0.11567	0.03573	-0.33006	0.05079	0.02239	0.09217	0.1937	0.28396	-0.08168	0.14006	0.14606
HGSOC4	0.14465	0.26991	0.00997	0.07007	0.11819	0.03133	-0.2055	0.08174	0.00713	0.12824	0.18698	0.24534	-0.07668	0.14375	0.18001
HGSOC5	0.1229	0.25659	0.12893	0.07718	0.0955	-0.00474	-0.17838	0.06779	-0.07603	0.09704	0.14779	0.24976	-0.05898	0.12597	0.14653
HGSOC6	0.13145	0.2432	0.10664	0.05953	0.11302	0.09769	-0.34073	0.05187	-0.10877	0.1227	0.19087	0.26191	-0.04288	0.13813	0.11983
HGSOC7	0.13737	0.24946	0.0378	0.08835	0.10073	0.05941	-0.33141	0.05223	0.0823	0.11478	0.22303	0.25276	-0.08775	0.14096	0.11472
HGSOC8	0.11401	0.25693	-0.0062	0.06704	0.1094	0.02607	-0.18293	0.03774	-0.13237	0.14024	0.20765	0.22614	-0.06456	0.13213	0.18129
HGSOC9	0.13201	0.26096	0.00201	0.06865	0.11856	0.05726	-0.13642	0.0665	-0.11544	0.12016	0.18036	0.2353	-0.05812	0.13419	0.17433
HGSOC10	0.1396	0.23988	0.05285	0.06544	0.09439	0.07382	-0.23192	0.07224	-0.12237	0.1108	0.14116	0.23629	-0.05953	0.13067	0.15023
HGSOC11	0.12012	0.28225	0.08596	0.06777	0.10437	0.07593	-0.06594	0.0469	0.11627	0.07793	0.19412	0.24372	-0.02453	0.13407	0.13962
HGSOC12	0.12214	0.29242	-0.01238	0.08682	0.08186	0.02366	-0.33428	0.05619	-0.05358	0.11615	0.20407	0.2707	-0.03543	0.15124	0.13056
HGSOC13	0.15487	0.26743	0.06442	0.08371	0.12407	-0.01241	-0.42847	0.02278	0.09388	0.05661	0.19851	0.24517	-0.07106	0.15937	0.11173
HGSOC14	0.14998	0.26304	0.01139	0.09036	0.11015	0.03774	0.18207	0.05291	0.06791	0.11177	0.20765	0.24061	-0.06501	0.13358	0.1669
HGSOC15	0.14886	0.28487	0.04589	0.0923	0.08833	0.01815	0.09492	0.06227	-0.03116	0.09178	0.17826	0.27083	-0.08343	0.13645	0.17206
HGSOC16	0.16684	0.29434	0.09838	0.09303	0.07933	0.01797	-0.18356	0.02191	0.08504	0.0463	0.16452	0.26869	-0.15028	0.13968	0.11026
HGSOC17	0.12123	0.27751	0.03702	0.07877	0.10147	0.02014	-0.08062	0.0494	0.01694	0.08244	0.16163	0.26476	-0.03005	0.13408	0.16896
HGSOC18	0.11209	0.26189	0.07257	0.07179	0.11358	0.04136	-0.29517	0.05591	0.01215	0.12687	0.19286	0.21658	-0.06696	0.13459	0.16997

	CA1_TARG	CA1_TAR	POESIS	POIESIS	OXO3_TA	OXO3_TA	PRKCI_TA	L_DISAB	TO_CARM	TO_CARM	TO_CARM	TO_CARM	ARMUSTI	ARMUSTI
HGSOC1	0.15624	0.20692	0.02005	-0.06282	0.05965	0.09706	0.01571	0.07227	0.09486	0.20594	0.13086	0.10741	0.20057	0.24885
HGSOC2	0.16926	0.18888	0.04206	0.06052	0.06718	0.02056	0.00811	0.01961	0.14537	0.18077	0.13343	0.14638	0.18829	0.27633
HGSOC3	0.13438	0.20781	0.09469	-0.07202	0.05844	0.04656	0.04635	0.08896	0.09817	0.17916	0.11828	0.14945	0.18659	0.23561
HGSOC4	0.14098	0.21376	0.06887	-0.10736	0.03868	0.06001	0.04232	0.10513	0.12376	0.1869	0.13408	0.17282	0.15638	0.25112
HGSOC5	0.12078	0.1902	0.10193	-0.073	0.04425	0.09695	0.04555	0.1018	0.1148	0.14817	0.11481	0.11186	0.145	0.2348
HGSOC6	0.14725	0.18679	-0.04234	-0.27462	0.08252	0.10164	0.06289	0.13123	0.09124	0.20296	0.12434	0.17134	0.14081	0.25543
HGSOC7	0.13256	0.19971	0.14111	-0.00225	0.04739	0.02834	0.01772	0.10459	0.07716	0.17179	0.10368	0.16142	0.17893	0.25329
HGSOC8	0.12974	0.20594	0.08306	-0.077	0.0308	0.09435	0.06511	0.12896	0.12332	0.17795	0.12535	0.13909	0.15067	0.22956
HGSOC9	0.11244	0.20264	0.10767	-0.1129	0.03562	0.06211	0.06683	0.10939	0.11242	0.14163	0.11309	0.13849	0.12755	0.23078
HGSOC10	0.1083	0.19829	0.12109	-0.05967	0.04322	0.07687	0.03712	0.15845	0.11685	0.16991	0.10542	0.14142	0.12384	0.2379
HGSOC11	0.14342	0.15316	0.0567	0.02021	0.07426	0.05034	0.04718	0.04301	0.09172	0.19482	0.10152	0.15309	0.20862	0.24336
HGSOC12	0.15223	0.17689	0.00313	-0.071	0.07179	0.04064	0.02518	0.09638	0.07823	0.13594	0.1086	0.1507	0.12247	0.26825
HGSOC13	0.14624	0.16353	-0.04479	-0.00196	0.08339	0.0273	0.04944	0.02037	0.06338	0.23756	0.09925	0.14499	0.19476	0.26329
HGSOC14	0.13123	0.19289	0.05999	-0.00929	0.04864	0.02194	0.07051	0.073	0.1316	0.22128	0.12202	0.15288	0.18995	0.26724
HGSOC15	0.11671	0.19742	0.07655	-0.0631	0.06055	0.06188	0.03462	0.07514	0.04817	0.11726	0.10643	0.15929	0.16131	0.29427
HGSOC16	0.15596	0.15504	-0.01045	0.03461	0.08338	0.06084	-0.0104	0.04349	0.0944	0.2063	0.10527	0.15077	0.23459	0.26855
HGSOC17	0.14018	0.20401	0.06081	-0.05343	0.05305	0.06322	0.01309	0.08101	0.10951	0.22673	0.13963	0.16383	0.15875	0.25659
HGSOC18	0.10895	0.21502	-0.01842	-0.27049	0.02822	0.08328	0.04972	0.0984	0.10798	0.1902	0.10727	0.13306	0.14557	0.23577

	ARMUSTI	ARMUSTI	NDOMETR	NDOMETR	ROID_CA	ROID_CA	ELAPSE	ELAPSE	ELAPSE	ELAPSE	CANCER	CANCER	BX1_TAR	BX1_TAR	MPHOCY
HGSOC1	0.28533	0.0696	-0.25912	0.0761	0.16273	0.16794	-0.02178	0.08395	0.14523	0.09641	0.25123	0.14725	0.04811	0.10633	0.17375
HGSOC2	0.26193	-0.02136	0.2725	0.02915	0.15575	0.19339	0.00203	0.04131	0.11278	0.06609	0.21069	0.11881	0.03122	0.08944	0.18851
HGSOC3	0.20952	0.10166	-0.2489	0.02983	0.14351	0.18701	0.00049	0.03658	0.15287	0.07423	0.25882	0.09327	0.02344	0.11018	0.19299
HGSOC4	0.2619	0.14728	-0.43491	0.04377	0.15549	0.16938	-0.01254	0.04979	0.14326	0.10988	0.23121	0.14211	0.01809	0.09203	0.17545
HGSOC5	0.21721	0.04216	-0.41096	-0.01002	0.14231	0.18899	0.01248	0.0744	0.1211	0.08597	0.20122	0.11399	0.02413	0.07846	0.1702
HGSOC6	0.22388	0.02634	-0.32975	0.05607	0.13888	0.19461	-0.00243	0.06177	0.12722	0.05139	0.18661	0.14821	0.0173	0.09681	0.19744
HGSOC7	0.19005	0.11107	-0.40937	0.05997	0.16152	0.166	-0.01894	0.02695	0.13223	0.05659	0.17812	0.12356	0.02383	0.08896	0.19757
HGSOC8	0.2002	0.07364	-0.42716	0.00982	0.14246	0.18618	0.00598	0.04876	0.1115	0.09724	0.19753	0.12554	0.02271	0.06441	0.15778
HGSOC9	0.20071	0.02739	-0.4062	0.07221	0.13745	0.2024	0.01203	0.04598	0.11549	0.10264	0.20326	0.14003	0.01625	0.06566	0.17522
HGSOC10	0.21868	0.04148	-0.35987	-0.01779	0.13853	0.1794	0.01187	0.05072	0.12783	0.09695	0.21457	0.12363	0.02717	0.05979	0.17976
HGSOC11	0.22967	0.06441	0.2075	0.00984	0.14515	0.18709	-0.06991	0.10007	0.17711	0.0432	0.20613	0.14736	0.03682	0.13703	0.19515
HGSOC12	0.24573	0.03794	-0.34966	0.05169	0.15646	0.18639	-0.02402	0.02942	0.15948	0.05497	0.12283	0.16224	0.04352	0.11321	0.18693
HGSOC13	0.19221	0.04875	-0.38911	0.1272	0.16121	0.17834	-0.02758	0.04223	0.18395	0.07204	0.2079	0.14491	0.04906	0.13332	0.17726
HGSOC14	0.22711	0.12231	-0.24379	-0.00828	0.16118	0.17648	-0.03362	0.0439	0.13125	0.05033	0.24457	0.14683	0.02687	0.07777	0.18899
HGSOC15	0.23677	0.08332	-0.1115	0.00789	0.15456	0.16984	-0.02062	0.05069	0.16929	0.0771	0.21851	0.12543	0.03044	0.10964	0.18928
HGSOC16	0.21775	0.05773	-0.42135	0.00082	0.16214	0.15943	-0.01849	0.07263	0.22504	0.03243	0.20689	0.10749	0.03034	0.1408	0.18876
HGSOC17	0.28169	0.05623	-0.39792	0.03896	0.16904	0.18963	-0.02418	0.04892	0.1046	0.11892	0.25004	0.12942	0.03135	0.06402	0.17536
HGSOC18	0.24348	0.08714	-0.43914	0.02785	0.14085	0.18486	0.01575	0.08125	0.12032	0.10854	0.23172	0.11574	0.01824	0.08483	0.17223

	D40_SIGN	D40_SIGN	CELL_L	CELL_L	F2_TARG	F2_TARG	XIFEN_R	XIFEN_R	PAX3_FO	F_PAX3_	F_PAX3_	OMA_STE	OMA_STE	SOME_I	C_LUPU
HGSOC1	0.01773	0.13032	-0.07886	0.16601	0.09809	0.2162	0.01099	0.11823	0.17098	-0.0483	-0.14197	0.03343	-0.05291	-0.08784	0.20418
HGSOC2	-0.05977	0.08938	-0.15127	0.16258	0.04743	0.23752	0.04491	0.1497	0.143	-0.03086	-0.16479	0.00294	-0.17606	-0.0301	0.1876
HGSOC3	0.00805	0.13209	-0.29002	0.16154	0.1108	0.20726	0.04771	0.1608	0.1613	-0.05588	-0.15243	0.04906	-0.06852	-0.0637	0.2447
HGSOC4	0.04092	0.17806	-0.08098	0.2092	0.17791	0.24606	0.06301	0.17203	0.21037	-0.08107	-0.10784	0.11129	-0.01834	-0.12453	0.27768
HGSOC5	0.04371	0.18372	-0.267	0.20586	0.14321	0.25749	0.05397	0.10992	0.21087	-0.09265	-0.12819	0.07905	-0.07532	-0.03624	0.25181
HGSOC6	-0.04748	0.11614	-0.17071	0.15297	0.16883	0.25414	0.05244	0.13485	0.19141	-0.0785	-0.10744	0.01157	-0.08072	0.02382	0.2303
HGSOC7	-0.03225	0.14874	-0.28912	0.18806	0.12686	0.26856	0.03235	0.19407	0.19758	0.05553	-0.11049	0.02268	-0.03501	-0.0809	0.31702
HGSOC8	0.09049	0.19639	-0.11686	0.22885	0.17151	0.2637	0.01654	0.15755	0.21472	-0.11374	-0.05887	0.1035	-0.04001	-0.10143	0.26774
HGSOC9	0.05589	0.25138	-0.16653	0.21807	0.1863	0.26825	0.05448	0.16773	0.27445	-0.16497	-0.1101	0.13397	-0.02283	-0.06383	0.30244
HGSOC10	0.06153	0.20972	-0.11735	0.1946	0.15404	0.30163	0.04695	0.20078	0.25086	-0.15822	-0.07981	0.11844	-0.01521	-0.10577	0.34281
HGSOC11	-0.01649	0.06111	-0.35731	0.15063	0.17526	0.18312	0.06694	0.12512	0.15054	-0.01725	-0.0587	-0.02923	-0.08666	0.00427	0.18554
HGSOC12	-0.00797	0.13174	-0.35978	0.16013	0.15913	0.23999	0.02529	0.14969	0.17335	-0.06591	-0.02154	0.02037	-0.0739	-0.0581	0.28411
HGSOC13	-0.08908	0.02843	-0.05356	0.14611	0.02373	0.17684	-0.01484	0.13856	0.17277	0.03252	-0.10967	-0.04462	-0.08806	-0.03596	0.15971
HGSOC14	0.01405	0.15994	-0.30616	0.18507	0.11495	0.20331	0.04532	0.10601	0.16543	-0.12255	-0.08381	0.07331	-0.06831	-0.11306	0.20619
HGSOC15	0.01633	0.15074	-0.0947	0.16164	0.12458	0.19871	0.04663	0.16549	0.17018	-0.14139	-0.17049	0.0386	-0.15917	-0.04639	0.24688
HGSOC16	-0.1191	0.04396	-0.31884	0.10141	0.01758	0.22112	-0.00629	0.18088	0.07507	-0.03585	-0.11485	-0.02723	-0.13508	-0.00595	0.17531
HGSOC17	-0.01869	0.13556	-0.06577	0.18246	0.14301	0.21127	0.0458	0.12895	0.16661	-0.05744	-0.11423	0.03733	-0.0616	-0.09815	0.18883
HGSOC18	0.04498	0.18509	-0.26935	0.21024	0.14916	0.25558	0.04499	0.19244	0.19646	-0.05314	-0.10663	0.11076	-0.00524	-0.08635	0.30577

	H_CYCL	TH_EED	PORATH	PORATH	TH_ES_C	CORE_NI	ES_WITH	MYC_M	C_TARGE	H_NANO	TH_NOS	TH_OC
HGSOC1	0.08439	0.1175	0.11056	-0.03617	0.29453	0.08562	-0.01359	0.15811	0.24031	0.11875	0.06242	[illegible]
HGSOC2	0.09353	0.10646	0.12461	0.06003	0.32175	0.11968	-0.02429	0.16535	0.23557	0.13007	0.08861	[illegible]
HGSOC3	0.08454	0.1059	0.13928	0.06669	0.33102	0.12118	-0.03627	0.16007	0.23526	0.11618	0.06753	[illegible]
HGSOC4	0.07063	0.10399	0.09066	-0.01922	0.27151	0.08643	-0.01286	0.15656	0.23439	0.11062	0.06695	[illegible]
HGSOC5	0.07174	0.09715	0.09757	-0.01904	0.29452	0.10042	-0.03367	0.1598	0.23439	0.11036	0.07474	[illegible]
HGSOC6	0.08095	0.0857	0.12594	-0.0348	0.30185	0.11383	-0.03007	0.15838	0.23475	0.11532	0.07525	[illegible]
HGSOC7	0.06827	0.10506	0.10932	0.04055	0.26027	0.0792	-0.01197	0.14923	0.24275	0.11223	0.08091	[illegible]
HGSOC8	0.05346	0.10341	0.04963	-0.0442	0.2798	0.07817	-0.02088	0.15554	0.22378	0.10224	0.05807	[illegible]
HGSOC9	0.05332	0.10312	0.06437	-0.02529	0.28779	0.08174	-0.02339	0.1502	0.22133	0.10562	0.05093	[illegible]
HGSOC10	0.05714	0.09266	0.0636	-0.04904	0.29142	0.0985	-0.0192	0.15744	0.23313	0.10326	0.06065	[illegible]
HGSOC11	0.08783	0.10014	0.132	-0.03065	0.2828	0.09511	-0.03221	0.16322	0.23006	0.12269	0.05766	[illegible]
HGSOC12	0.0811	0.1066	0.12911	-0.03214	0.30266	0.09521	-0.03873	0.16325	0.23988	0.11006	0.05571	[illegible]
HGSOC13	0.10057	0.09498	0.13198	0.09194	0.31282	0.11651	-0.0135	0.14998	0.22571	0.12723	0.08336	[illegible]
HGSOC14	0.07145	0.09081	0.09091	0.03447	0.30581	0.09691	-0.03263	0.15399	0.22958	0.11438	0.06709	[illegible]
HGSOC15	0.08251	0.10206	0.11323	0.02019	0.29679	0.08013	-0.0178	0.16154	0.23973	0.11704	0.06365	[illegible]
HGSOC16	0.07984	0.09977	0.13923	0.11732	0.32454	0.11585	-0.03704	0.16194	0.23832	0.11832	0.06558	[illegible]
HGSOC17	0.06556	0.10283	0.08846	0.04506	0.31271	0.08411	-0.02417	0.15805	0.23105	0.12435	0.07105	[illegible]
HGSOC18	0.06355	0.09541	0.07786	-0.02458	0.29178	0.07928	-0.02695	0.15728	0.22985	0.11029	0.06835	[illegible]

SIGNALIN	SIGNALIN	NSFORM	RMED_B	RMED_B	MED_BY	MED_BY	NSFORM	APDC1B_	APDC1B_	CINOMA_	CINOMA_	VS_DUCT	VS_DUCT	BY_2MET	BY_2MET	EMBRYO	RGETS_NO	GETS_NO	ARGETS_	ARGETS_
0.16236	0.164	0.06999	-0.01404	0.12017	0.02256	0.02929	0.13799	0.02248	0.18568	-0.03467	0.19917	0.08054	0.11603	0.11726	0.15292	0.28388	-0.04916	-0.00885	-0.04506	0.01683
0.15253	0.20269	0.04687	0.01625	-0.06389	0.08785	-0.09405	0.16067	-0.0416	0.16678	-0.04237	0.19941	0.09082	0.13591	0.10498	0.13578	0.29366	-0.07037	-0.0011	-0.07057	0.03205
0.17856	0.18695	0.07095	-0.01734	0.0182	0.05536	-0.05262	0.16929	-0.01125	0.15428	0.02328	0.17458	0.10631	0.14555	0.10032	0.1511	0.30198	-0.06096	-0.00227	-0.04886	0.03258
0.16801	0.18969	0.0848	-0.00265	0.07004	0.0192	0.09053	0.13103	0.00436	0.1584	0.09105	0.14423	0.08809	0.13215	0.10522	0.14854	0.26663	-0.05694	-0.01231	-0.07269	0.01449
0.15192	0.14989	0.06946	0.03005	0.1068	-0.03418	0.06274	0.13811	0.001	0.19431	0.07739	0.151	0.06219	0.13942	0.11147	0.15118	0.27018	-0.09751	-0.05985	-0.12447	-0.01672
0.15602	0.13892	0.07081	0.00602	0.08522	0.05737	-0.04808	0.16241	0.06822	0.14367	0.0994	0.15122	0.06768	0.15502	0.13498	0.15134	0.27371	-0.04684	-0.04075	-0.07308	-0.00158
0.15336	0.16659	0.07401	-0.01046	-0.07832	0.07186	0.03165	0.16254	0.06126	0.13951	0.02617	0.18061	0.06859	0.15238	0.14367	0.14202	0.29403	-0.0942	0.02215	-0.04864	0.0467
0.16623	0.13406	0.10223	0.00892	0.18611	0.028	0.04532	0.11183	0.03957	0.1719	0.11941	0.15053	0.07888	0.13372	0.11695	0.14259	0.24554	-0.07173	-0.0435	-0.07484	-0.02121
0.17174	0.14806	0.10177	0.02453	0.10376	0.03373	0.05091	0.11441	0.03598	0.16861	0.11631	0.13931	0.08376	0.14599	0.11304	0.13997	0.26372	-0.06729	-0.04632	-0.08876	-0.01633
0.14391	0.10534	0.07706	-0.0353	0.09522	-0.02152	-0.09943	0.11461	0.0515	0.19918	0.12229	0.14692	0.04503	0.14942	0.12891	0.14391	0.24869	-0.08671	-0.05928	-0.11501	-0.02023
0.16249	0.18638	0.05431	0.02201	0.06596	0.05636	0.12614	0.17245	-0.00762	0.12766	-0.0176	0.20405	0.12184	0.10589	0.10418	0.17703	0.2953	-0.06659	-0.00992	-0.0346	0.02542
0.16697	0.15689	0.08523	0.0439	0.04265	0.05404	0.07701	0.16216	-0.0111	0.14074	0.01562	0.19242	0.0979	0.12782	0.13553	0.15837	0.2966	-0.06088	-0.0146	-0.03602	0.01994
0.13653	0.1271	0.04097	0.10977	-0.06317	0.05592	-0.07979	0.17181	-0.01584	0.15431	-0.02712	0.23399	0.10666	0.1055	0.11497	0.13812	0.28364	-0.00735	0.01043	-2.6E-05	0.04634
0.1636	0.21516	0.07513	-0.00031	0.11163	-0.00825	-0.05767	0.13212	-0.00283	0.14929	0.04517	0.16572	0.06389	0.11576	0.11231	0.14679	0.27877	-0.05704	-0.0054	-0.04017	0.00983
0.20264	0.1956	0.08032	0.01907	0.15749	0.07406	0.02163	0.15735	0.01051	0.13917	0.03886	0.14996	0.10238	0.12325	0.12227	0.16031	0.29514	-0.07741	-0.01319	-0.07509	0.02364
0.15332	0.1628	0.03932	0.08023	-0.03519	0.05249	-0.0956	0.18691	-0.05022	0.13716	-0.02467	0.22002	0.10277	0.12694	0.13089	0.1387	0.28663	-0.07672	0.02917	-0.07239	0.06533
0.18169	0.1816	0.08354	0.03784	0.08513	0.05338	0.11242	0.13414	0.03701	0.15982	0.04809	0.17127	0.06767	0.11997	0.11324	0.14184	0.27939	-0.05695	-0.01184	-0.06882	0.02466
0.15537	0.18605	0.08191	-0.02272	0.15497	0.04405	0.07657	0.1199	-0.00232	0.20177	0.06244	0.14982	0.09305	0.14227	0.10984	0.13897	0.27189	-0.08357	-0.02743	-0.06849	0.00019

METASTA	METASTA	MYCIN_S	SENSITI	RAPAMY	RESPON	RUM_SEN	UM_SEN	UM_SENS	B_CLL_	B_CLL_	ONCOGEN	NCOGEN	NCOGEN	COGEN	COGEN	HEIMER	ERS_DIS
0.0269	0.19872	0.16907	0.2852	0.34795	0.3664	0.19747	0.14008	0.14005	0.13226	0.00627	0.06542	0.01474	0.04977	0.06449	-0.01358	0.17951	0.20961
0.00591	0.22454	0.19561	0.30643	0.36933	0.38662	0.22101	0.15586	0.1533	0.13695	-0.01572	0.08487	0.03742	0.02697	0.08534	-0.01915	0.18152	0.22127
0.06711	0.20892	0.1849	0.2864	0.35258	0.37565	0.20903	0.12696	0.15685	0.16777	-0.00302	0.06058	0.02451	0.03269	0.0952	0.0044	0.17766	0.20996
0.09085	0.17823	0.17308	0.29979	0.36976	0.39255	0.23164	0.13205	0.14783	0.18091	0.06966	0.05557	-0.01189	0.05634	0.06306	-0.00162	0.16964	0.20495
0.08276	0.18435	0.15462	0.28434	0.3599	0.38799	0.2311	0.11049	0.15058	0.18096	0.05908	0.05595	0.01391	0.02845	0.05032	-0.03591	0.17606	0.20538
0.04908	0.20075	0.14723	0.30235	0.3717	0.38277	0.20221	0.10972	0.14631	0.14255	0.06038	0.07494	0.029	0.03125	0.06666	-0.01883	0.17793	0.20507
0.07805	0.18833	0.17979	0.27831	0.36941	0.37856	0.17674	0.12949	0.14264	0.13381	0.07222	0.03556	0.04005	0.04556	0.10312	-0.01347	0.17311	0.2169
0.11888	0.17054	0.16813	0.29976	0.36606	0.39394	0.23122	0.13188	0.13687	0.19778	0.03445	0.03312	-0.0385	0.05326	0.03685	0.02033	0.17179	0.21364
0.11025	0.17884	0.17319	0.2874	0.35888	0.37796	0.23009	0.1473	0.13022	0.20608	0.01538	0.04132	-0.0139	0.05497	0.03433	-0.01424	0.16834	0.21092
0.11671	0.17623	0.17563	0.27978	0.35707	0.37091	0.22504	0.11077	0.13269	0.2119	0.06144	0.03522	-0.00363	0.04532	0.03988	-0.02944	0.16664	0.21167
0.00829	0.20422	0.16377	0.31768	0.35958	0.37103	0.20582	0.13071	0.15308	0.1211	0.00659	0.02795	0.04304	0.03633	0.09618	-0.00432	0.19307	0.22996
0.06241	0.1853	0.15644	0.30376	0.36457	0.38532	0.20753	0.11957	0.12501	0.1277	0.00813	0.05577	0.01829	0.05659	0.07624	0.00752	0.18229	0.20942
0.01935	0.2118	0.18916	0.29612	0.35681	0.37579	0.21345	0.15228	0.14705	0.07854	0.01017	0.09288	0.04999	0.05194	0.10161	-0.02948	0.17695	0.20105
0.07972	0.1921	0.17884	0.30061	0.36848	0.38174	0.2205	0.16592	0.15642	0.16089	0.02229	0.04195	-0.03207	0.04532	0.07784	-0.02079	0.17322	0.20866
0.04843	0.20248	0.15711	0.31756	0.37236	0.39315	0.20165	0.12048	0.13598	0.15148	0.03888	0.0546	0.01102	0.04017	0.09609	-0.02088	0.17906	0.20798
0.02531	0.20355	0.17247	0.30159	0.36405	0.3891	0.21686	0.12573	0.14076	0.07054	0.00062	0.05817	0.02518	0.06088	0.13888	0.04203	0.18813	0.20984
0.08991	0.18592	0.16347	0.29953	0.37494	0.39991	0.21128	0.12015	0.14949	0.15723	0.01966	0.07743	-0.00799	0.05492	0.07303	-0.00769	0.18438	0.21348
0.1063	0.17682	0.16115	0.28333	0.35438	0.38005	0.22589	0.12203	0.13565	0.18271	0.05414	0.04387	0.00194	0.03401	0.05394	-0.04472	0.1778	0.2156

ERS_DIS	HEIMER	TREATED_	TREATED_	_CELLS_	CELLS_	ELIAL_C	ELIAL_C	EPITHELI	EPITHELI	INFECTIO	TION_A549	ION_A549	INFECTI	S_COV_2	S_COV_2	S_COV_2_	COV_2_
0.04786	0.06879	-0.05155	0.05286	-0.03853	0.04771	-0.00832	0.01492	-0.13296	0.09067	0.03249	0.12003	-0.0424	-0.0261	0.03176	-0.08151	0.20046	0.00415
0.06669	0.07642	-0.05937	0.0421	-0.00631	0.0449	-0.08825	-0.00454	-0.10116	0.08391	0.0441	0.13274	-0.04874	-0.0297	-0.00867	-0.17747	0.02026	-0.01641
0.07316	0.08667	-0.08282	0.06924	-0.01661	0.071	-0.02615	0.02835	-0.07851	0.11308	0.05425	0.1461	-0.02861	-0.00538	0.0916	-0.09026	0.17315	0.05172
0.0632	0.07522	0.00576	0.07994	-0.01308	0.09321	-0.03663	0.02306	-0.06764	0.12443	0.06497	0.18705	-0.03421	-0.01092	0.07829	-0.12424	0.3028	0.06839
0.06301	0.07515	-0.04344	0.08084	-0.0724	0.08416	-0.06479	0.00034	-0.08149	0.10979	0.0603	0.13665	-0.04602	-0.02249	0.02695	-0.09199	0.24908	0.03825
0.06867	0.07193	-0.0392	0.0738	-0.03047	0.06707	-0.08905	0.04525	-0.03506	0.10248	0.11866	0.16667	-0.03895	-0.0162	0.11923	-0.08812	0.12877	0.02753
0.05734	0.0712	-0.06116	0.08566	-0.03314	0.10366	-0.03018	0.04841	-0.08245	0.16237	0.07559	0.1689	-0.02262	0.01916	0.07523	-0.0796	-0.00018	0.1032
0.07274	0.07795	-0.0069	0.09024	-0.09098	0.10655	-0.11759	0.04315	-0.05074	0.13671	0.09305	0.19695	-0.01731	-0.00162	0.03341	-0.08062	0.34085	0.06186
0.06535	0.07809	-0.04689	0.10883	-0.07562	0.12989	-0.09501	0.04042	-0.04375	0.13978	0.10218	0.19732	-0.01232	0.01648	0.06925	-0.02784	0.28871	0.11969
0.06451	0.07194	-0.05339	0.11535	-0.07507	0.13656	-0.12668	0.03106	-0.03902	0.16683	0.08585	0.18845	0.00188	0.03171	0.04335	-0.06596	0.22036	0.13262
0.03999	0.0649	-0.02278	0.0275	0.04425	0.00818	0.00362	0.06151	-0.10699	0.10366	0.0949	0.12604	-0.04018	-0.02886	0.11296	-0.06994	0.08896	-0.00352
0.06416	0.07116	-0.06364	0.08565	-0.03568	0.07015	-0.03784	0.03914	-0.05412	0.12999	0.08045	0.15641	-0.04473	0.005	0.11444	-0.06617	-0.07046	0.07289
0.06835	0.08241	-0.00853	0.02485	-0.0112	0.02004	-0.02462	0.02491	-0.14815	0.09764	0.07567	0.12236	-0.06736	-0.04699	0.07035	-0.09875	-0.06799	-0.01349
0.05983	0.07489	0.04524	0.04891	-0.08136	0.05597	-0.01856	0.00576	-0.04747	0.11979	0.08249	0.14971	-0.04182	-0.03286	0.03813	-0.0633	0.30897	0.00892
0.05943	0.07324	0.00647	0.05566	-0.01136	0.05421	-0.0193	0.03479	-0.07028	0.1381	0.09071	0.15308	-0.04812	-0.01322	0.07744	-0.08329	0.29206	0.02301
0.04965	0.06782	-0.03713	0.03517	0.02132	0.03527	0.04061	0.02392	-0.08689	0.07725	0.03388	0.11147	-0.06188	-0.02694	0.06512	-0.11294	0.00423	0.00287
0.05916	0.07472	0.0283	0.03644	-0.13646	0.04201	-0.03902	-0.0092	-0.10677	0.09771	0.07511	0.14365	-0.03978	-0.02411	-0.02646	-0.11566	0.21525	-0.00699
0.06794	0.07419	-0.06189	0.10134	-0.07979	0.11562	-0.05418	0.02825	-0.09954	0.13531	0.06813	0.15802	-0.02017	0.01229	0.02634	-0.09685	0.30509	0.08482

MOI_INF	MOI_INF	COV_2_P	COV_2_	RENZA_VI	RENZA_VI	ZA_A_INF	ZA_A_INF	OV_INFE	OV_INFE	CYTIAL_	CYTIAL_	V_1_INF	V_1_INF	NSE_TO_	NSE_TO_	S_FOXA2	LATED_	ELATED_M
0.14582	0.01808	-0.08961	0.0349	-0.04307	0.03948	0.12541	0.13466	0.14908	0.09114	0.02468	0.03656	0.04097	-0.33486	0.18361	0.13646	0.07498	0.09483	0.23807
0.00132	0.03475	-0.06163	-0.00525	-0.0109	0.03955	0.16218	0.16917	0.1383	0.11822	0.02735	0.0261	-0.13701	-0.11767	0.19817	0.11975	0.07322	0.06587	0.2179
0.03955	0.03365	-0.07233	0.05429	-0.07541	0.08113	0.15961	0.11569	0.14207	0.10411	0.00426	0.04979	-0.19097	-0.18918	0.20048	0.12389	0.08717	0.10656	0.23138
0.28953	0.07217	-0.11536	0.11414	-0.0287	0.10945	0.13628	0.13543	0.17245	0.10684	0.08726	0.08824	-0.00371	-0.29047	0.17798	0.12492	0.10215	0.10947	0.26063
0.20909	0.04475	-0.09103	0.11942	-0.08517	0.08756	0.11243	0.13039	0.19791	0.08107	-0.021	0.06552	0.0037	-0.19694	0.1692	0.1196	0.07857	0.06365	0.2706
0.1975	0.05296	-0.05514	0.03652	0.02298	0.06872	0.12756	0.16643	0.23329	0.1254	0.09153	0.04814	0.16916	-0.1795	0.19888	0.13035	0.11868	0.04564	0.29733
0.11058	0.09846	-0.07754	0.09588	-0.01851	0.13741	0.13448	0.12969	0.2179	0.10759	0.08222	0.09888	-0.2633	-0.29417	0.18746	0.12744	0.11676	0.0443	0.26374
0.15553	0.03254	-0.131	0.13831	-0.08061	0.10519	0.13402	0.1502	0.18551	0.11965	-0.00096	0.08704	0.01985	-0.3634	0.14614	0.13531	0.10826	0.0862	0.27141
0.26159	0.09901	-0.11635	0.15704	-0.043	0.15967	0.13902	0.1473	0.17914	0.09877	0.01738	0.12039	0.09917	-0.31284	0.15283	0.11719	0.10726	0.09188	0.25536
0.21499	0.10098	-0.11524	0.19063	-0.05174	0.16674	0.1348	0.12238	0.17939	0.09363	0.05673	0.11668	-0.15614	-0.48811	0.15624	0.12334	0.1117	0.09299	0.25805
-0.02922	0.08697	-0.05507	0.0214	-0.02359	0.02047	0.14313	0.17297	0.17881	0.1556	0.05234	0.03882	-0.23416	-0.13317	0.21179	0.13717	0.09532	0.06154	0.26939
0.13265	0.1076	-0.06703	0.04487	-0.01983	0.12394	0.12824	0.10362	0.1735	0.09771	0.0812	0.08871	-0.20665	-0.257	0.18803	0.14545	0.11388	0.09608	0.2938
0.02898	0.05823	-0.03977	-0.05756	0.07561	0.02014	0.13115	0.10423	0.17028	0.10758	0.07366	0.01417	0.05796	-0.0879	0.20656	0.13715	0.08028	0.07743	0.23353
0.05813	0.07579	-0.09632	0.07357	0.02251	0.04665	0.11911	0.07259	0.12372	0.13757	0.0858	0.06228	-0.26914	-0.39228	0.16541	0.12789	0.09848	0.0928	0.23269
0.0923	0.05455	-0.07382	0.08894	-0.00023	0.0618	0.13874	0.12435	0.17184	0.10653	0.07147	0.05125	-0.42402	-0.23445	0.18901	0.13832	0.10068	0.10229	0.27294
0.13868	0.06097	-0.04607	-0.05138	0.00919	0.05307	0.16236	0.09982	0.11263	0.10054	0.05143	0.02543	-0.3155	-0.10146	0.20387	0.13851	0.08813	0.0882	0.21988
0.02345	0.06884	-0.09754	0.05527	0.01634	0.0364	0.13429	0.1236	0.1369	0.13165	0.09015	0.03327	-0.46212	-0.40338	0.16024	0.11675	0.0867	0.07257	0.22518
0.2032	0.06496	-0.11716	0.14021	-0.0815	0.1428	0.15225	0.13694	0.19091	0.10564	-0.02306	0.1061	0.16246	-0.34895	0.15277	0.11834	0.08476	0.09479	0.24737

NODEFIC	NODEFIC	OF_MIR	CANCER_	CANCER_	IR_SURV	SURVIVA	L_CULT	LL_CULT	UT_STEM	CT_STEM	GNANT_M	NANT_M	ER_CAN	UCED_TH	_DIFFER	INDUCE	CYTOTO
0.09541	0.13333	0.04152	0.0925	0.13632	0.09977	0.04939	0.01824	0.08364	0.05462	-0.05452	-0.00136	0.19772	0.12994	0.13783	0.11422	-0.02774	-0.09995
0.10069	0.18246	0.0585	0.0838	0.07725	0.0954	0.03705	0.07658	0.07958	0.03374	-0.07254	-0.0262	0.21859	0.16011	0.10537	0.02524	0.0006	-0.08748
0.12152	0.15561	0.02263	0.16087	0.05708	0.08301	0.03376	0.08964	0.09748	0.0747	-0.01249	0.02373	0.19652	0.16779	0.1461	-0.01921	0.05941	-0.05194
0.18046	0.11614	0.07082	0.07647	0.17404	0.1032	0.03398	0.13488	0.10239	0.09545	0.03424	0.02919	0.1931	0.15764	0.16349	0.07061	0.07651	-0.0248
0.12721	0.12638	0.06785	0.077	0.05819	0.08111	0.04315	0.0628	0.08755	0.08687	-0.02878	0.03597	0.19314	0.14317	0.14824	-0.05849	0.04002	0.02696
0.06204	0.1659	0.08294	0.03482	0.21971	0.09608	0.04628	0.09087	0.12007	0.07499	0.04838	0.03524	0.20284	0.17545	0.13658	-0.05625	0.03438	0.01288
0.10041	0.12346	0.02809	0.16152	0.17027	0.11505	0.05432	0.14633	0.12676	0.08937	0.08557	0.04284	0.19727	0.16878	0.15036	-0.01272	0.13701	0.01072
0.19303	0.10693	0.04744	0.08099	0.19233	0.09105	0.0488	0.09831	0.10767	0.07471	0.04694	0.03408	0.18554	0.14348	0.17269	-0.01511	0.04521	-0.01165
0.20318	0.1051	0.056	0.08555	0.14019	0.09167	0.04242	0.1064	0.14188	0.11008	0.04799	0.05652	0.18339	0.17786	0.1653	-0.07451	0.11623	0.08841
0.16815	0.09134	0.03736	0.06046	0.16736	0.10969	0.03901	0.1029	0.11705	0.09964	0.0352	0.06583	0.19002	0.11826	0.15735	-0.09096	0.15842	0.08882
0.03302	0.17679	0.02332	0.10746	0.06984	0.08362	0.02791	0.05329	0.08871	0.07441	-0.04534	-7.2E-05	0.20007	0.14411	0.08833	0.11655	-0.02933	-0.12287
0.07244	0.16944	0.03126	0.12469	0.1792	0.09541	0.04035	0.10048	0.11638	0.07167	0.03161	0.0393	0.20042	0.14525	0.10432	0.08038	0.08961	-0.01062
0.03525	0.17196	0.07906	0.09049	0.07213	0.1076	0.02373	-0.00886	0.06987	0.07061	-0.08836	0.02253	0.21263	0.14507	0.10263	0.09912	-0.00943	-0.15933
0.12848	0.13029	0.05778	0.09957	0.07352	0.09822	0.03113	0.13126	0.09857	0.08554	-0.00174	0.00963	0.19562	0.12615	0.14716	0.13639	0.00671	-0.02788
0.0763	0.16253	0.03108	0.10554	0.14185	0.08487	0.02975	0.06096	0.09669	0.06867	0.00689	-0.00718	0.20637	0.13486	0.13541	0.03669	0.02773	-0.05847
-0.0206	0.18327	0.02489	0.14261	0.01409	0.06774	0.03625	-0.03709	0.06663	0.05133	-0.09111	-0.02649	0.19685	0.12577	0.09488	0.08196	0.0103	-0.13984
0.15718	0.12409	0.07443	0.09774	0.09246	0.09263	0.04052	0.12101	0.08541	0.06867	-0.0068	0.03727	0.18857	0.15117	0.13646	0.14876	-0.02371	-0.06485
0.1638	0.11256	0.03387	0.06376	0.11834	0.08836	0.03547	0.12704	0.1013	0.127	0.00751	0.04126	0.18824	0.14327	0.16586	-0.05624	0.11206	0.06225

A_BOUND	TO_EXTR	ONSE_TO	NCER_S	ANCER_S	ANCER_S	ANCER_S	ANCER_S	ANCER_	CANCER	ANCER_S	ANCER_S	ANCER_S	ANCER_	ANCER_S	ANCER_S	ANCER_	CANCER_
0.19227	0.23528	0.1958	0.09293	0.12842	0.10544	-0.05899	0.09582	0.09758	-0.01791	0.1917	0.13163	0.02193	0.17525	-0.0058	0.01325	0.15853	0.11937
0.18606	0.24905	0.22613	0.06011	0.15894	-0.02303	-0.02812	0.07948	0.10862	0.03128	0.14168	0.08014	-0.02663	0.1965	-0.0281	0.10073	0.1606	0.0823
0.21389	0.30756	0.31476	0.14447	0.13843	0.01855	-0.02789	0.1318	0.09325	-0.03196	0.09742	0.14376	0.04485	0.169	-0.0748	0.10386	0.20823	0.06596
0.19183	0.32198	0.29918	0.12015	0.13778	0.05681	-0.10073	0.10175	0.0723	0.03791	0.16418	0.12223	0.07227	0.15428	0.05699	0.19304	0.27114	0.07513
0.19498	0.2834	0.25438	0.10239	0.12338	0.09782	-0.0674	0.13107	0.06652	-0.00979	0.15205	0.11088	0.05831	0.17073	0.02918	0.18376	0.23669	-0.00464
0.21322	0.24257	0.22575	0.15624	0.16544	0.11632	-0.06977	0.12107	0.06535	-0.01281	0.16727	0.1619	0.09925	0.18736	0.11268	0.1465	0.15692	0.02497
0.20016	0.33357	0.34459	0.19386	0.12181	-0.0092	-0.05831	0.07441	0.08577	-0.01194	0.10348	0.10386	0.10519	0.17372	0.12393	0.14438	0.19917	0.03473
0.21828	0.30574	0.2854	0.13589	0.10265	0.09098	-0.08732	0.13235	0.0349	0.02851	0.21161	0.07639	0.11998	0.14788	0.06375	0.22799	0.26341	0.03004
0.20474	0.33738	0.31486	0.13188	0.11106	0.06776	-0.09064	0.13462	0.03561	0.04557	0.14154	0.08632	0.11564	0.14631	0.02274	0.12392	0.29493	-0.01967
0.19018	0.38935	0.38194	0.11857	0.08769	0.15136	-0.07791	0.12545	0.04061	0.02908	0.14737	0.03493	0.13494	0.15105	0.03079	0.1234	0.27196	0.06476
0.2233	0.20231	0.15417	0.10272	0.14208	0.0164	0.0012	0.10431	0.08095	9.1E-05	0.05242	0.16806	0.02469	0.17169	0.02816	0.13853	0.13313	0.12129
0.2176	0.32891	0.30059	0.16518	0.13976	0.02108	0.01708	0.10543	0.07793	-0.03504	0.15772	0.16613	0.09693	0.17146	0.05103	0.1393	0.18315	0.03718
0.18461	0.19314	0.20606	0.08859	0.17245	0.06619	0.02048	0.05731	0.09392	-0.06499	0.15112	0.13986	0.01211	0.19055	0.04462	0.00519	0.03274	0.13375
0.20128	0.19199	0.16402	0.12472	0.13328	0.00011	-0.05544	0.11577	0.0858	-0.0188	0.17317	0.08289	0.04187	0.16743	-0.03806	0.1272	0.23343	0.04982
0.20281	0.26217	0.24828	0.14054	0.12859	0.10763	-0.04943	0.14147	0.07888	0.01459	0.1648	0.12548	0.05313	0.17056	0.02893	0.13581	0.20737	0.07874
0.20034	0.27232	0.23468	0.1146	0.14257	0.08333	-0.01068	0.10144	0.09356	-0.07937	0.12627	0.18641	0.00541	0.18508	-0.11778	0.09933	0.06574	0.13927
0.21491	0.19557	0.16528	0.12198	0.13489	0.03432	-0.04274	0.09745	0.1096	-0.02526	0.1985	0.07263	0.04421	0.16599	0.04968	0.13301	0.20175	0.08072
0.2147	0.36883	0.35131	0.12298	0.09731	0.06242	-0.04862	0.13673	0.0394	0.01111	0.12346	0.08563	0.09689	0.15779	-0.05828	0.19452	0.26638	0.04868

CANCER_	FETAL_TA	MYELOM	MYELOM	TIPLE_MY	IPLE_MYE	PLE_MYE	TIPLE_MY	MYELOM	MYELOM	TIPLE_MY	TIPLE_MY	PLE_MYE	PLE_MYE	PLE_MYE	PLE_MYE	SE_TO_C	SE_TO_C
0.09522	0.10728	0.13275	0.09931	0.02468	0.08342	0.08141	0.07201	0.04232	0.07543	0.1199	0.05503	0.11464	0.1693	0.11125	0.02882	0.22938	0.01642
0.06424	0.06683	0.11016	0.11216	0.02408	0.0118	0.09044	0.08594	0.04357	0.08595	0.11186	0.03148	0.08325	0.0754	0.08272	0.05034	0.21312	0.00702
0.08329	0.07959	0.11809	0.05327	-0.01044	0.04369	0.09802	0.05608	0.06191	0.03059	0.12786	0.06554	0.09386	0.13733	0.09697	0.06911	0.22901	0.08131
0.10504	0.10991	0.19236	0.07737	0.02797	0.08587	0.07685	0.04617	0.05072	0.03275	0.16728	0.0468	0.06166	0.16563	0.13063	-0.01207	0.22643	0.07907
0.0922	0.08994	0.09027	0.11857	-0.05321	0.10425	0.05753	0.0591	0.05696	0.11004	0.15942	0.07255	0.03891	0.19517	0.13664	0.05496	0.23062	0.05776
0.03195	-0.01376	0.12817	0.06076	-0.00562	0.05732	0.09423	0.04591	0.05567	0.07077	0.10313	0.07095	0.17105	0.15722	0.10268	0.0873	0.21923	0.10501
0.06126	-0.02657	0.13467	0.04198	-0.04148	0.06632	0.08204	0.0284	0.06617	0.00904	0.14952	0.04214	0.08017	0.1739	0.10964	0.00117	0.24073	0.16662
0.09725	0.03804	0.19159	0.07543	-0.00785	0.10621	0.06535	0.0151	0.05907	0.03641	0.14494	0.0183	0.09764	0.20086	0.11339	-0.07183	0.23038	0.08988
0.09463	0.03681	0.17686	0.06409	0.00509	0.09577	0.06063	0.02181	0.0938	0.11376	0.16983	0.05083	0.09214	0.17774	0.11927	0.03801	0.221	0.085
0.0845	-0.00815	0.17435	0.04641	-0.01921	0.10836	0.05053	0.00475	0.05961	0.09678	0.15766	0.02501	-0.00965	0.21632	0.12983	-0.01919	0.22249	0.06476
0.06187	0.07611	0.11279	0.06494	0.05011	0.06692	0.10652	0.05499	0.05597	0.06355	0.11376	0.07573	0.1412	0.16268	0.10805	0.10984	0.22168	0.04614
0.07143	0.06232	0.09174	0.07965	0.02859	0.06331	0.09647	0.02901	0.06596	0.00044	0.12079	0.05706	0.09589	0.17292	0.112	0.07332	0.23953	0.10767
0.05683	-0.03336	0.0892	0.07991	-0.02276	0.00174	0.11928	0.05689	0.08392	0.03041	0.08788	0.06055	0.13823	0.06547	0.09768	0.04067	0.23833	0.08969
0.10434	0.11874	0.12699	0.11223	-0.0627	0.07818	0.07326	0.04374	0.09209	0.07946	0.16352	0.02504	0.01155	0.15637	0.14017	-0.00592	0.2104	0.08497
0.08416	0.03263	0.12601	0.0661	-0.02047	0.09331	0.09425	0.04511	0.07388	0.07795	0.14849	0.06966	0.04272	0.18666	0.1214	0.00965	0.23387	0.0626
0.08522	0.01334	0.07505	0.08971	-0.01173	-0.01659	0.11134	0.08075	0.07748	0.0906	0.07721	0.04995	0.12574	0.02164	0.10912	0.07999	0.22094	0.09093
0.09071	0.20524	0.1464	0.04443	0.01222	0.07913	0.09152	-0.01359	0.04302	0.05933	0.1329	0.01374	0.00193	0.1628	0.08946	0.00763	0.22176	0.05759
0.07717	0.03743	0.13399	0.08417	0.01235	0.06312	0.06064	0.03404	0.09245	0.04688	0.17938	0.04786	0.14051	0.1314	0.13414	0.00364	0.22376	0.08641

SPONSE_	SE_TO_M	SE_TO_M	SIGNALIN	SIGNALIN	ALING_VI	ALING_VI	TOR_SIG	IMPRINT	OSIS_RE	V_INFEC	V_INFEC	V_INFEC	V_INFEC	V_INFEC	V_INFEC	V_INFEC	V_INFEC	V_INFEC	V_INFEC
-0.01943	0.20595	-0.09552	0.09997	0.00318	0.08119	0.004	0.01319	-0.04938	0.07932	0.08996	0.05282	0.057	0.01753	0.06267	0.08681	0.07842	-0.00152	0.11155	0.03722
-0.04621	0.1858	-0.10205	0.06137	-0.00383	0.04501	0.01787	0.01819	-0.02835	0.07849	0.04652	0.04914	0.05979	0.01731	0.041	0.11079	0.03322	0.01769	0.06611	0.04889
0.10185	0.19739	0.08189	0.0436	-0.00812	0.031	-0.01012	-0.06339	-0.04892	0.06391	0.05704	0.05548	0.03576	0.03627	0.04128	0.09782	0.05598	0.00713	0.10214	0.03068
0.08461	0.21654	0.04989	0.08248	-0.0062	0.08153	0.04632	-0.1351	-0.00443	0.09498	0.08537	0.06077	0.05954	0.02908	0.06403	0.08906	0.08084	0.00783	0.12688	0.0211
0.05151	0.2262	0.01323	0.06532	-0.02103	0.06554	0.01511	-0.04342	-0.02386	0.06804	0.05859	0.04744	0.01899	0.0169	0.06219	0.10122	0.03348	-0.0092	0.10035	0.03576
0.04592	0.17928	0.01041	0.0483	-0.02982	0.00831	-0.03555	-0.03082	-0.02318	0.06074	0.04834	0.0426	0.03313	-0.01494	0.03527	0.10678	0.02954	0.00345	0.09742	0.02588
0.07035	0.23697	0.01231	0.01166	-0.04973	0.04986	0.01255	-0.00312	0.01674	0.05888	0.03857	0.05994	0.074	0.05453	0.02497	0.08999	0.05068	0.00184	0.11615	0.0186
0.09797	0.21421	0.07022	0.089	0.01183	0.06421	0.03801	-0.16614	0.00932	0.10503	0.03946	0.05083	0.02067	0.0148	0.06443	0.08831	0.06094	-0.02189	0.12574	0.01018
0.08421	0.2154	0.05435	0.10201	0.0172	0.05903	0.03895	-0.15111	0.00231	0.09897	0.05356	0.06035	0.01252	0.04201	0.05797	0.09435	0.0519	-0.01015	0.11273	0.01936
0.07315	0.22355	0.02839	0.09206	0.00854	0.07031	-0.00944	-0.18298	-0.05625	0.09842	0.04361	0.06857	0.01381	0.06133	0.05128	0.09465	0.02703	-0.00329	0.11068	0.02641
0.0626	0.16362	-0.00197	0.04007	-0.01732	0.01287	0.00188	-0.01162	0.0045	0.04978	0.07203	0.06143	0.03455	-0.00986	0.03929	0.09553	0.04904	0.00592	0.08313	0.03231
0.04749	0.20515	0.02024	0.06397	-1E-05	0.03831	0.07566	-0.04631	-0.05986	0.06444	0.07203	0.0505	0.05704	0.03101	0.07258	0.07393	0.04694	-0.00013	0.10301	0.01253
0.10646	0.19801	0.0374	0.06556	-0.00891	0.04051	0.01733	-0.02836	0.03302	0.06023	0.0146	0.0347	0.06639	-0.00212	0.04361	0.08101	0.03271	0.0169	0.09608	0.03054
0.05478	0.19139	0.02095	0.06735	-0.02618	0.06943	-0.00419	-0.09666	-0.00643	0.07357	0.08604	0.07102	0.05553	-0.00197	0.0422	0.0972	0.06985	0.00285	0.09687	0.02155
0.01903	0.21089	-0.00096	0.09106	-0.00605	0.08436	0.00485	-0.09576	-0.01395	0.06403	0.06313	0.06189	0.01887	0.0173	0.04869	0.10554	0.06318	0.01582	0.11222	0.03262
0.03841	0.18064	-0.01363	0.0259	0.01823	0.04713	-0.01143	0.09041	-0.012	0.02456	0.08208	0.00264	0.04481	0.01387	0.07029	0.07735	0.06455	0.0096	0.07683	0.02709
0.06986	0.20387	0.044	0.0649	0.01365	0.05412	0.0347	-0.01427	0.01665	0.06708	0.08043	0.03976	0.06935	-0.01777	0.05938	0.08366	0.0726	-0.00076	0.1114	0.0176
0.06114	0.2199	0.02833	0.08148	-0.00903	0.07262	-0.02624	-0.1154	-0.03568	0.08728	0.05109	0.05508	0.02332	0.03689	0.06204	0.10313	0.04852	-0.02567	0.11052	0.00119

V_INFEC	V_INFEC	V_INFEC	V_INFEC	V_INFEC	V_INFEC	V_INFEC	V_INFEC	V_INFEC	V_INFEC	V_INFEC	V_INFEC	V_INFEC	V_INFEC	ERON_RE
0.10392	0.10637	0.12157	0.01564	-0.01966	-0.04423	0.04406	0.08502	-0.01397	0.04905	-0.06296	0.01979	0.03438	0.11777	0.0832
0.07344	0.09444	0.09765	0.00203	-0.00664	-0.06634	0.02176	0.04646	0.01917	0.01557	-0.05477	0.0271	-0.05128	0.10498	0.08707
0.0763	0.09086	0.10215	-0.01828	0.02237	-0.03759	0.03232	0.06945	0.02122	0.05297	-0.05217	0.0727	-0.01767	0.1406	0.1452
0.09053	0.06933	0.11709	0.04918	0.01645	-0.00922	0.03971	0.07859	-0.00557	0.06664	-0.06165	0.06908	-0.00902	0.09908	0.16836
0.06956	0.08521	0.10803	0.0655	0.01847	-0.06563	0.02438	0.06654	0.02196	0.0138	-0.06717	0.08634	-0.01233	0.09898	0.14742
0.10316	0.11036	0.10129	0.04835	0.01348	0.03767	0.04956	0.06293	0.04495	0.02357	-0.06862	0.0634	-0.0248	0.12179	0.11603
0.09577	0.08548	0.10227	0.07856	0.00525	0.06793	0.03175	0.0561	0.02315	0.07572	-0.06044	0.12841	0.04606	0.13202	0.19229
0.09145	0.07176	0.07119	0.04473	0.00324	0.00768	0.04116	0.05864	-0.00705	0.05063	-0.07699	0.07421	0.04513	0.08812	0.16504
0.09191	0.06144	0.07031	0.03178	0.01694	0.02201	0.03766	0.06944	0.007	0.09623	-0.07205	0.09141	0.05533	0.10121	0.20911
0.08366	0.06328	0.08386	0.02227	0.01533	-8.4E-05	0.03168	0.06797	0.01273	0.09552	-0.07749	0.13091	0.05228	0.11196	0.23888
0.07899	0.11939	0.06939	0.01202	-0.00621	0.001	0.03205	0.06117	0.00845	0.07621	-0.02026	0.07107	-0.0591	0.10619	0.04647
0.12077	0.08633	0.1029	0.03676	0.00697	0.01353	0.04346	0.0716	0.02369	0.10337	-0.04925	0.10546	0.00589	0.13763	0.15665
0.04813	0.09908	0.1032	-0.00775	-0.00371	-0.03162	0.03755	0.0823	0.03826	0.04128	-0.01777	0.01927	-0.05651	0.09662	0.02917
0.08151	0.08228	0.03844	0.05661	0.00799	-0.00336	0.03609	0.05221	0.01561	0.02216	-0.02843	0.09635	0.02942	0.11212	0.09832
0.10546	0.10612	0.07948	-0.03363	0.00419	0.0008	0.03552	0.06603	0.01347	0.08631	-0.07348	0.05577	-0.03549	0.12519	0.11707
0.09435	0.09914	0.064	-0.06609	-0.01455	-0.02969	0.02075	0.04241	0.01594	0.0761	-0.03768	0.03526	-0.11306	0.09403	0.09371
0.08029	0.07405	0.11396	0.0722	0.00534	-0.07618	0.03253	0.06602	0.00602	-0.00724	-0.05079	0.04933	0.07555	0.10356	0.07632
0.09339	0.06524	0.0802	0.03269	0.01628	-0.09991	0.02834	0.0706	0.0133	0.04185	-0.05381	0.11281	0.05498	0.09449	0.20577

_CELL_D	_CELL_D	RGETS_O	RGETS_O	RESPONS	C_RESP	_RESPO	PONSE_V	PONSE_V	PONSE_V	PONSE_V	_VESSEL	PTATION_	_HEMATO	OCYTE_
0.15157	0.14999	0.17187	0.09953	0.02557	0.05084	-0.05871	0.02942	0.06415	0.09491	0.09256	-0.13617	-0.13466	0.09663	-0.04219
0.15937	0.11734	0.15835	0.08927	0.03763	0.04191	-0.03559	0.00104	0.02647	0.10347	0.08329	-0.16362	-0.07593	0.11551	-0.09993
0.18624	0.13792	0.18064	0.07619	0.0186	0.0509	-0.02208	0.02299	0.03276	0.11222	0.0553	-0.14099	-0.12038	0.10245	-0.04792
0.14059	0.2076	0.17977	0.06923	0.01563	0.03977	-0.04418	0.03562	0.04605	0.10556	0.05573	-0.22001	-0.20777	0.12387	-0.01838
0.14854	0.1903	0.13172	0.07249	0.02309	0.0441	-0.03902	0.02965	0.03378	0.11355	0.0746	-0.19528	-0.05911	0.0917	0.00368
0.18474	0.12532	0.15841	0.06592	0.01203	0.03829	-0.05569	0.02352	0.00346	0.06252	0.08551	-0.12515	-0.23851	0.10946	-0.1349
0.19895	0.14753	0.16643	0.07729	0.00616	0.04509	-0.04085	0.02947	0.02016	0.12575	0.05943	-0.24657	-0.25785	0.12834	-0.10275
0.13104	0.23136	0.18009	0.04727	0.01662	0.04792	-0.04648	0.03692	0.0608	0.11293	0.05705	-0.16653	-0.22697	0.09446	-0.02163
0.1498	0.22054	0.17732	0.02929	0.01576	0.04207	-0.04786	0.03775	0.05064	0.12084	0.05689	-0.24162	-0.2546	0.11495	-0.02969
0.15358	0.21182	0.13606	0.0575	0.00793	0.0387	-0.03164	0.03754	0.03831	0.11522	0.07111	-0.17242	-0.23265	0.08972	-0.05323
0.17886	0.11673	0.145	0.05424	0.01109	0.04251	-0.05376	0.01769	0.0445	0.10796	0.06334	-0.26183	-0.22011	0.12229	-0.14023
0.17388	0.14213	0.17784	0.07727	0.02863	0.05835	-0.04124	0.0305	0.04059	0.08709	0.08433	-0.22985	-0.24197	0.11488	-0.07757
0.17851	0.05877	0.12101	0.10425	0.03459	0.04484	-0.02807	0.02866	0.04315	0.12051	0.05342	-0.13742	-0.05982	0.09346	-0.04736
0.14526	0.17964	0.16275	0.05	0.01282	0.03516	-0.01879	0.02998	0.04572	0.12637	0.04862	-0.30497	-0.15787	0.11571	-0.04514
0.16739	0.16253	0.18168	0.05561	0.02138	0.04725	-0.03805	0.02703	0.05115	0.11884	0.06814	-0.1488	-0.24325	0.10849	-0.03139
0.18237	0.05771	0.1293	0.08223	0.03571	0.05575	-0.04316	0.01107	0.03087	0.11563	0.08297	-0.21396	-0.19296	0.11253	-0.10494
0.15815	0.16521	0.14428	0.08037	0.00784	0.03917	-0.03983	0.03049	0.04965	0.11572	0.05345	-0.24198	-0.13264	0.12829	-0.08237
0.15247	0.1796	0.16872	0.06169	0.0145	0.04686	-0.04433	0.03228	0.03523	0.10562	0.05894	-0.22891	-0.18154	0.09948	0.03128

OCYTE_I	YPOXIA_M	ADIPOGE	ADIPOGE	ADIPOGE	ADIPOGE	ADIPOGE	ADIPOGE	ADIPOGE	ADIPOGE	ADIPOGE	ADIPOGE	ADIPOGE	ADIPOG	GENESIS
0.05011	0.22987	-0.13968	0.05842	0.00577	0.00142	0.15337	0.04252	0.09021	0.18632	0.18448	0.12731	0.15427	0.05599	0.13259
0.02227	0.21529	-0.09431	0.06493	0.00792	0.05819	0.13315	0.04981	0.13792	0.16597	0.14017	0.13871	0.17696	0.03203	0.12892
0.06788	0.23148	-0.15131	0.04158	0.00999	0.05368	0.1486	0.09195	0.1578	0.19471	0.1632	0.17024	0.2012	0.08707	0.15959
0.07495	0.21644	-0.15227	0.03941	-0.02989	0.0064	0.13911	0.06371	0.10458	0.16917	0.16265	0.1688	0.20109	0.08272	0.19784
0.05352	0.20516	-0.18316	0.06819	-0.02235	0.04076	0.11243	0.05062	0.11324	0.15659	0.16234	0.16086	0.18613	0.04391	0.13355
0.06941	0.22771	-0.12253	0.04515	-0.04648	0.07688	0.12643	0.11847	0.12359	0.16681	0.16592	0.16713	0.16902	0.03117	0.13122
0.05373	0.22258	-0.21514	0.03256	-0.03536	-0.0265	0.14974	0.07577	0.20437	0.20292	0.16157	0.18613	0.21578	0.06232	0.16113
0.08076	0.19554	-0.14281	0.01847	-0.03189	0.00349	0.15763	0.00954	0.09762	0.16622	0.17637	0.16061	0.21526	0.08666	0.20239
0.07626	0.18328	-0.14716	0.02894	-0.00982	0.01329	0.12041	0.0309	0.12038	0.14857	0.15314	0.17813	0.21868	0.07823	0.19344
0.1119	0.19214	-0.2251	0.04365	-0.05127	-0.00265	0.12885	0.01691	0.0887	0.16237	0.16273	0.14188	0.20797	0.06725	0.16823
0.06727	0.25057	-0.07785	0.0458	-0.07119	0.06922	0.13702	0.11007	0.12541	0.19226	0.19387	0.18558	0.1622	0.04057	0.09394
0.07442	0.23807	-0.20957	0.05326	-0.01282	0.03303	0.1219	0.07961	0.13592	0.18976	0.18163	0.1561	0.17357	0.05465	0.13789
0.02224	0.25318	-0.10499	0.10468	-0.03048	0.08779	0.16563	0.09939	0.12703	0.18248	0.13078	0.1105	0.14379	0.04294	0.04481
0.09025	0.21962	-0.05042	0.06507	-0.04079	0.00632	0.16929	0.00597	0.13446	0.18721	0.17366	0.18177	0.21692	0.07189	0.15738
0.08982	0.2242	-0.05262	0.08198	-0.05245	0.02356	0.1624	0.06381	0.11334	0.16759	0.18247	0.15748	0.18337	0.0936	0.14673
0.07023	0.23864	-0.15434	0.05949	-0.04107	0.03886	0.17079	0.09542	0.16669	0.22115	0.1674	0.13604	0.15721	0.04929	0.08987
0.03421	0.22416	-0.11255	0.04998	-0.02351	0.0375	0.14446	-0.01635	0.11839	0.19721	0.1751	0.15892	0.19779	0.08413	0.16848
0.08887	0.21616	-0.18344	0.04958	-0.03235	0.02613	0.13133	0.03082	0.1011	0.17711	0.16898	0.17482	0.21414	0.07918	0.18582

GENESIS	GENESIS	GENESIS	GENESIS	NAMIC_T	NAMIC_TR	RELATE	RELATE	_STEM_C	STEM_C	POIESIS	OPOIESIS	OPOIESIS	POIESIS_S	IESIS_ST
0.16274	0.07824	0.0305	0.15645	0.04077	0.02209	0.09448	0.16646	0.10274	0.07887	-0.41996	0.19432	0.41432	0.12187	0.07296
0.11517	0.07855	0.02421	0.16952	0.03786	0.01563	0.09686	0.083	0.10463	0.07758	-0.04425	0.19228	0.4035	0.10348	0.08303
0.17144	0.09961	-0.02976	0.17303	0.04321	0.0243	0.14235	0.08937	0.09897	0.0982	-0.41806	0.13663	0.3799	0.11283	0.09686
0.1713	0.08553	-0.00228	0.17217	0.0443	0.02017	0.15385	0.11632	0.12593	0.08959	-0.43908	0.18459	0.44034	0.15104	0.09728
0.18257	0.07099	-0.05249	0.16254	0.03482	0.01647	0.13298	0.09436	0.11331	0.09994	-0.44098	0.19098	0.40976	0.13213	0.09482
0.22331	0.09551	0.0482	0.12367	0.03983	0.0051	0.12349	0.09729	0.10758	0.08844	-0.35421	0.09916	0.41143	0.12824	0.08134
0.17919	0.07975	0.01783	0.14251	0.05902	0.00646	0.12572	0.08043	0.11936	0.09473	-0.37992	0.0197	0.42302	0.1295	0.08419
0.1272	0.06017	-0.00932	0.15348	0.03659	0.02808	0.14817	0.15504	0.11082	0.09265	-0.38071	0.16461	0.42532	0.1461	0.09517
0.13378	0.05682	0.04834	0.15225	0.03261	0.02146	0.16033	0.10035	0.09608	0.09079	-0.43746	0.16429	0.44194	0.13789	0.09993
0.14256	0.05917	-0.04044	0.14847	0.0313	0.01286	0.15216	0.14736	0.11767	0.08847	-0.39024	0.165	0.42493	0.13762	0.09488
0.20035	0.13644	0.02799	0.12493	0.0718	0.02424	0.10451	0.09924	0.09342	0.09666	-0.24392	0.08268	0.39509	0.12233	0.07912
0.20662	0.11829	-0.03226	0.13938	0.06193	0.00863	0.10642	0.09662	0.08611	0.10156	-0.43452	0.0641	0.41881	0.12362	0.08558
0.16659	0.11469	0.05306	0.08325	0.04436	0.02614	0.07307	0.0178	0.11106	0.07793	-0.17181	0.12396	0.42224	0.10638	0.08198
0.11329	0.06171	0.05708	0.18446	0.03192	0.02544	0.12583	0.12337	0.11148	0.099	-0.46411	0.12757	0.43702	0.14305	0.09103
0.18992	0.11886	0.01443	0.16992	0.04771	0.02287	0.09694	0.15762	0.09557	0.08328	-0.4277	0.15956	0.38309	0.12632	0.08957
0.18546	0.13388	-0.02248	0.16402	0.06699	0.01459	0.06576	0.03907	0.11194	0.09518	-0.32207	0.07732	0.39957	0.10924	0.07598
0.10319	0.10189	0.00434	0.17198	0.02877	0.02177	0.11541	0.11915	0.13304	0.09074	-0.39564	0.09411	0.42351	0.14428	0.09374
0.10461	0.03014	-0.02861	0.16681	0.044	0.01951	0.14193	0.12016	0.10897	0.09014	-0.44873	0.13602	0.45263	0.13878	0.09607

POIESIS_	SIS_STEM	YKH_SC	TG16L1_T	TG16L1_T	ONSE_TO	ONSE_TO	ONSE_TO	ONSE_TO	ONSE_TO	ONSE_TO	ONSE_TO	ONSE_TO	ESPONSE	ESPONSE
-0.10587	-0.01104	0.22261	0.09549	0.00048	0.27884	0.23667	0.20475	0.2603	0.24492	-0.04192	0.27221	0.12058	0.21357	0.17359
-0.24972	0.01192	0.23016	0.05588	-0.00493	0.20654	0.2367	0.21082	0.26181	0.23271	-0.06973	0.31478	0.13715	0.19629	0.13363
-0.15377	0.03891	0.1725	0.11643	0.04079	0.23847	0.21812	0.2122	0.25951	0.26062	-0.06805	0.29675	0.17177	0.26477	0.15788
-0.17373	0.03384	0.15321	0.07699	0.08442	0.28324	0.27139	0.17398	0.26635	0.24663	-0.05829	0.26755	0.1642	0.22028	0.10755
-0.27513	0.03376	0.26661	0.13485	0.07074	0.23655	0.23942	0.17833	0.2595	0.26682	-0.12346	0.22895	0.12746	0.20539	0.11918
-0.28732	-0.01196	0.22327	0.12291	0.07357	0.267	0.2242	0.21171	0.27471	0.26623	-0.05269	0.29141	0.18341	0.22337	0.17121
-0.35733	0.02036	0.1703	0.10323	0.11862	0.25922	0.17304	0.21537	0.24475	0.281	-0.01098	0.2663	0.16622	0.22398	0.15512
-0.25716	0.06856	0.15863	0.13327	0.1035	0.2452	0.25389	0.18244	0.26646	0.21808	-0.11705	0.24869	0.15902	0.19086	0.12545
-0.34294	0.06094	0.15872	0.1084	0.10822	0.27606	0.24053	0.17874	0.24849	0.23972	-0.09763	0.25491	0.14914	0.19701	0.09889
-0.28122	0.03976	0.12007	0.14455	0.11254	0.20653	0.20854	0.16126	0.2502	0.24661	-0.09367	0.25466	0.18018	0.15905	0.11101
-0.21753	0.07472	0.22316	0.1138	0.01785	0.28265	0.19952	0.23296	0.27038	0.26162	-0.13131	0.33056	0.12621	0.28513	0.15835
0.05731	0.00895	0.05909	0.10125	0.06104	0.29055	0.21301	0.22088	0.25596	0.22967	-0.07327	0.28595	0.18111	0.24421	0.16436
-0.03129	0.02894	0.20658	0.09127	0.03564	0.31333	0.23651	0.21921	0.25083	0.26628	-0.07191	0.29975	0.10638	0.23877	0.15098
-0.29669	0.04601	0.20242	0.07225	0.0673	0.25846	0.20425	0.18404	0.25309	0.25856	-0.03699	0.29756	0.13781	0.18526	0.11897
-0.20983	0.0478	0.21413	0.09146	0.03685	0.28677	0.20629	0.21398	0.27216	0.22184	-0.0409	0.33108	0.1484	0.24641	0.15029
-0.15041	-0.05472	0.08437	0.10971	-0.00754	0.27378	0.20603	0.23072	0.25484	0.26956	-0.00754	0.28298	0.09324	0.26902	0.15923
-0.18293	0.05407	0.23326	0.10207	0.0458	0.2453	0.28303	0.18052	0.24976	0.26545	-0.14313	0.25809	0.13771	0.19738	0.11252
-0.34445	0.00742	0.13243	0.10525	0.0717	0.22411	0.24256	0.16413	0.25736	0.24947	-0.09826	0.21756	0.11951	0.17373	0.10071

BLASTOM	BLASTOM	PATOBLA	ASTOMA_	PATOBL	AR_DEVEL	R_DEVEL	ETS_BO	ETS_BO	_SENSITI	SENSITI	SENSITI	ANCER_C	ANCER_C	TO_GAM
0.0261	0.17837	0.02761	0.13726	0.07028	0.11787	0.0409	0.13971	0.14771	-0.16809	-0.06289	0.34107	0.12541	0.10056	0.04884
0.00866	0.20583	-0.01959	0.15875	0.10538	0.06405	0.0499	0.08791	0.16833	-0.12921	0.11972	0.29728	0.12908	0.12094	0.06861
0.04272	0.1986	0.02574	0.29356	0.07712	0.12383	-0.01691	0.08222	0.14523	-0.15952	0.09051	0.33413	0.15975	0.07428	0.09408
0.08792	0.16736	0.07179	0.33895	0.04534	0.17871	-0.00261	-0.01946	0.19069	-0.08737	0.10408	0.31413	0.20515	0.10841	0.03804
0.05439	0.17046	0.04067	0.28264	0.0507	0.13987	0.00085	0.00759	0.17167	-0.13022	0.03829	0.3344	0.15763	0.09566	0.0851
0.10689	0.19926	0.08705	0.39974	0.0665	0.19018	0.02961	0.11422	0.21529	-0.10173	0.11717	0.33226	0.18012	0.11946	0.05761
0.11146	0.19416	0.1005	0.39063	0.04623	0.1793	0.00815	0.0712	0.17791	-0.22761	0.07739	0.33092	0.20505	0.11838	0.07332
0.08805	0.14595	0.07671	0.34213	0.01718	0.18832	-0.01374	0.01047	0.20371	-0.22746	0.00933	0.35405	0.17104	0.07993	0.0792
0.1075	0.14758	0.0607	0.37017	0.0302	0.18737	-0.02078	0.02474	0.18736	-0.18345	0.07125	0.3407	0.1741	0.09937	0.06789
0.08803	0.15588	0.0673	0.37719	0.01727	0.19661	-0.01238	0.06567	0.19855	-0.07898	-0.0143	0.32904	0.2192	0.10706	0.09044
0.06244	0.19669	0.0423	0.29032	0.06553	0.13529	0.02861	0.09062	0.18941	-0.1942	0.13145	0.33777	0.13674	0.11112	0.03335
0.0795	0.18775	0.07336	0.28739	0.06419	0.17066	0.01693	0.09546	0.1715	-0.09991	0.10913	0.33043	0.15674	0.13836	0.11794
0.04885	0.20533	0.01776	0.16161	0.09062	0.08795	0.0213	0.1279	0.21917	-0.09195	0.04112	0.31697	0.09585	0.13533	0.08352
0.0666	0.1778	0.04793	0.36108	0.05717	0.15798	-0.005	-0.00622	0.18451	-0.19386	0.15752	0.3604	0.15666	0.0875	0.11258
0.05091	0.19312	0.04632	0.34362	0.05832	0.17067	0.0169	-0.00017	0.20568	-0.08977	0.10978	0.29182	0.17511	0.10761	0.10459
0.01477	0.22182	0.0024	0.10143	0.06225	0.05091	0.02279	0.05304	0.1656	-0.12557	0.05638	0.33824	0.14907	0.17158	0.12692
0.04906	0.17653	0.02791	0.19125	0.07064	0.14126	0.02149	0.07489	0.13981	-0.22718	0.02585	0.31851	0.16864	0.11728	0.07874
0.06382	0.15524	0.03333	0.29212	0.02868	0.14798	0.00032	-0.02245	0.12175	-0.16546	0.04666	0.34549	0.1449	0.08507	0.07364

IR302A_T	PATOBL	MA_VS_N	OMA_VS_N	PATOBLA	S_LOWER	_LOWER	ROMYEL	ROMYEL	CONDAR	S_AND_N	S_AND_N	_HRAS_T	_HRAS_T	NRAS_T
0.06486	0.04207	-0.00839	0.05494	0.14865	0.08776	0.165	0.1885	-0.00101	0.07432	0.09426	0.2029	-0.09421	0.22653	-0.0746
0.06598	-0.00959	-0.05893	0.07396	0.16642	0.08292	0.13091	0.19313	-0.04302	0.09962	0.08559	0.15432	0.04219	0.23623	-0.04702
0.02749	0.03528	0.07722	0.08291	0.1514	0.07933	0.15817	0.2004	-0.0147	0.10077	0.03408	0.28581	0.29134	0.14947	-0.07038
0.00157	0.04076	0.0724	0.08708	0.1501	0.13324	0.1131	0.1807	-0.01667	0.16946	0.01981	0.25052	0.03404	0.2143	-0.09137
0.02205	0.02211	0.11185	0.0707	0.14651	0.12395	0.11196	0.17792	-0.00748	0.13955	-0.06833	0.20898	-0.03508	0.20974	-0.07412
0.0612	0.04076	0.14562	0.09853	0.15326	0.05936	0.09204	0.1924	-0.01542	0.11785	-0.05926	0.29466	0.22773	0.18142	-0.09422
0.0573	0.0553	0.14286	0.11354	0.14182	0.09181	0.12822	0.19255	-0.0197	0.13304	-0.09189	0.3264	0.25647	0.2669	-0.09223
-0.01143	0.04879	0.06889	0.07644	0.13854	0.1458	0.0486	0.16371	0.01379	0.18325	-0.04112	0.24581	0.02751	0.19312	-0.20263
0.00861	0.03305	0.13381	0.10701	0.14503	0.16234	0.05692	0.16664	0.00605	0.17837	-0.09691	0.18376	0.0105	0.16802	-0.15148
-0.00446	0.04298	0.09847	0.08563	0.13362	0.14799	0.05569	0.16585	-0.0197	0.16847	-0.06134	0.20916	0.1059	0.2172	-0.18111
0.09036	0.04635	0.14524	0.05692	0.16656	0.04364	0.15461	0.20024	-0.03594	0.09778	-0.03621	0.20164	0.27323	0.13556	-0.05198
0.04421	0.06739	0.15188	0.06876	0.14969	0.09341	0.13999	0.19067	0.02864	0.12314	-0.05351	0.21372	0.15001	0.18605	-0.05926
0.08938	0.0346	0.03331	0.0825	0.15571	0.03902	0.19555	0.2011	-0.05665	0.05671	0.01063	0.30921	0.26085	0.22418	0.06975
0.01216	0.04231	0.06112	0.0683	0.15228	0.122	0.12983	0.18494	0.00399	0.1704	-0.07538	0.27991	0.1067	0.13843	-0.16204
0.03665	0.05058	0.09902	0.04836	0.15115	0.07835	0.12491	0.19448	-0.00785	0.15712	0.00084	0.28023	0.15385	0.16817	-0.11323
0.04644	0.03433	0.1145	0.03745	0.1465	0.03194	0.18914	0.2052	-0.04955	0.051	0.09188	0.24423	0.22046	0.17746	-0.05129
0.01597	0.02691	0.05745	0.07244	0.1593	0.11934	0.14152	0.17506	-0.01292	0.14848	0.08238	0.26975	0.07405	0.21423	0.01265
0.00582	0.04259	0.09349	0.06404	0.13843	0.14046	0.06744	0.17339	-0.02806	0.15995	0.03094	0.22284	0.01337	0.21128	-0.18661

NRAS_1	CER_MAL	TS_OF_T	ETS_OF_T	NACTIVE_	_INACTIV	DHB_TAR	DHB_TAR	SDHB_TA	N_METAS	METASTA	METASTA	N_METAS	R_H2AFY	R_H2AFY
0.15188	0.23261	0.05597	0.12624	0.01455	0.02654	0.12721	0.03742	0.00497	-0.02698	0.07729	0.24157	0.13032	-0.03413	0.07141
0.12119	0.24577	0.08801	0.13111	0.1243	0.09679	0.09452	-0.018	-0.04502	-0.03359	0.09553	0.24242	0.128	0.01378	0.06968
0.17294	0.24012	0.12537	0.15036	0.06177	0.09701	0.06758	-0.05579	-0.02978	-0.02931	0.05651	0.21445	0.11498	0.00885	0.05534
0.20118	0.29635	0.10198	0.12581	0.03019	0.14137	0.17183	0.02435	0.0107	-0.00922	0.08646	0.23285	0.10809	-0.02643	0.04979
0.18382	0.26657	0.13762	0.12486	0.10696	0.12653	0.12876	-0.02718	-0.05206	-0.03422	0.08704	0.22497	0.10272	0.03016	0.00549
0.13303	0.25398	0.12353	0.1549	0.03149	0.12433	0.21891	-0.00886	0.02141	-0.03866	0.07679	0.23995	0.11444	0.01894	0.07431
0.19637	0.27315	0.08589	0.14396	0.13229	0.11741	0.17497	-0.02697	0.00186	-0.02657	0.04227	0.24522	0.11893	0.06709	0.03855
0.19108	0.30627	0.14512	0.12413	0.04483	0.08953	0.19387	-0.00751	0.01335	-0.01488	0.09391	0.21004	0.11639	-0.02023	0.0387
0.18974	0.30533	0.13176	0.13887	0.06435	0.12525	0.17284	-0.00591	0.0117	-0.00537	0.08522	0.20546	0.10647	-0.01265	0.02756
0.20196	0.27708	0.09391	0.1453	0.0764	0.1425	0.16871	-0.02685	-0.00063	-0.0102	0.07242	0.21732	0.11632	0.00083	0.01607
0.14792	0.22918	0.04898	0.17136	0.01205	0.15631	0.13071	0.05017	0.01738	-0.01909	0.07096	0.20273	0.10726	0.04628	0.06598
0.2054	0.24555	0.07973	0.19563	0.03374	0.12477	0.1517	0.01016	0.03423	-0.02653	0.08674	0.21996	0.10561	0.03916	0.02368
0.12789	0.18165	0.04359	0.1676	0.07784	0.11518	0.10306	-0.0062	-0.0082	-0.02273	0.08277	0.25006	0.11887	0.04647	0.0928
0.1904	0.27366	0.0859	0.16344	0.01705	0.11807	0.10913	-0.00039	-0.00161	-0.0371	0.08791	0.18915	0.10186	0.02596	0.03692
0.18792	0.25678	0.10848	0.13934	0.00432	0.09827	0.14975	0.00101	-0.00657	-0.02622	0.08343	0.23351	0.13098	0.01348	0.03937
0.16751	0.23366	0.06764	0.14916	0.16679	0.08178	0.06443	-0.01006	-0.04452	-0.01222	0.05591	0.21921	0.09962	0.05636	0.09276
0.18199	0.26016	0.11069	0.12402	0.03663	0.08506	0.12809	0.02535	-0.00861	-0.00142	0.1009	0.23453	0.11545	-0.0128	0.04839
0.20333	0.29453	0.13275	0.11291	0.08124	0.09389	0.11786	-0.01188	0.01199	-0.0173	0.08176	0.20262	0.09577	-0.00145	0.05147

TALIZED	TALIZED	USF1_TA	USF1_TA	ER_BAS	ER_BAS	NCER_LU	NCER_LU	R_LUMIN	R_LUMIN	T_SKIN	E_TO_ME	E_TO_ME	V_GR_TA	V_GR_TA
0.06105	-0.0063	0.05377	0.19607	-0.00197	0.07445	0.07167	0.04064	0.02384	0.03617	-0.27705	0.28421	0.23795	-0.00982	-0.01568
0.04475	0.04528	0.12071	0.21221	-0.00015	0.01885	0.02553	0.05383	0.01935	2E-05	-0.25036	0.28816	0.23088	-0.05991	-0.02085
0.12643	0.015	0.15214	0.24994	-0.02315	0.05388	0.0595	0.0009	0.02817	-0.00364	-0.33751	0.2852	0.26529	-0.05165	-0.04331
0.1376	-0.01988	0.03897	0.22018	-0.01709	0.05162	0.06569	0.01171	0.05194	-0.02745	-0.31881	0.30234	0.23922	-0.03189	-0.02671
0.07737	-0.00755	0.18927	0.2062	0.02342	-0.01397	0.03735	0.0094	0.04662	-0.05507	-0.24156	0.30424	0.21519	-0.03156	-0.01401
0.07498	-0.00141	0.09004	0.22555	-0.01385	-0.0234	0.05217	0.01825	0.04333	-0.02787	-0.33621	0.28815	0.23516	0.03019	-0.03144
0.08846	-0.02091	0.16783	0.19152	0.00885	0.0069	0.04814	0.03751	0.02815	-0.01248	-0.39798	0.27719	0.26503	0.01143	-0.05744
0.11049	0.00812	0.11372	0.19303	0.06482	-0.04995	0.06969	0.00578	0.07186	-0.06482	-0.36012	0.30207	0.21611	-0.01964	-0.00295
0.13304	0.04616	0.13226	0.20008	0.06865	-0.05647	0.0733	0.0093	0.07572	-0.07413	-0.131	0.3059	0.22249	-0.03274	0.00788
0.11449	0.03877	0.12212	0.20537	0.01692	-0.02445	0.05751	-0.0065	0.055	-0.06383	-0.19601	0.29247	0.2118	-0.02649	-0.0251
0.10845	0.02336	0.16292	0.20642	0.00704	0.08984	0.08334	0.0261	0.02528	0.02099	-0.35616	0.27107	0.24864	-0.03635	-0.05097
0.14488	-0.07094	0.14847	0.20468	-0.06992	0.07052	0.09147	0.00772	0.03925	-0.01711	-0.3123	0.2769	0.26648	-0.00996	0.00049
0.07087	-0.04015	0.21188	0.22338	-0.04863	0.10212	0.03959	0.0556	-0.02192	0.07293	-0.39861	0.24838	0.26248	-0.02549	-0.06797
0.08539	0.02162	0.1009	0.23297	0.03553	0.03679	0.05028	0.02491	0.04029	-0.00091	-0.24275	0.28933	0.22887	-0.04922	-0.05675
0.13284	-0.00792	0.06322	0.17358	0.02751	0.05963	0.06546	0.02082	0.03965	0.00327	-0.35955	0.2815	0.2351	-0.01996	-0.03434
0.09975	0.04193	0.12732	0.20669	-0.05604	0.12467	0.05871	0.04554	-0.02342	0.04483	-0.35196	0.24395	0.26471	-0.06938	-0.03981
0.07836	0.0311	0.04847	0.23731	-0.036	0.06283	0.04857	0.03918	0.03606	0.0022	-0.28412	0.29729	0.24263	-0.03617	-0.0486
0.1245	0.01519	0.09115	0.16897	0.01067	-0.03176	0.0755	0.00951	0.05268	-0.04763	-0.37083	0.30585	0.22926	-0.03084	-0.0434

S_VS_E	DE_TO_PR	E_TO_PR	INTED_B	SE_TO_N	TAF7L_T	_TARGET	S_TARGE	S_TARGE	S_TARGE	S_TARGE	METABOL	_CANCE	ORT_OF	ORT_OF_
0.13494	0.04263	0.0496	-0.00776	0.24088	0.05302	-0.04033	-0.02311	0.31465	0.12634	-0.03372	0.02997	0.38488	0.17738	-0.05702
0.13006	0.01641	0.04197	0.01139	0.24149	0.44213	-0.01617	0.00475	0.41439	0.11309	-0.03057	-0.02446	0.37302	0.16772	-0.08982
0.11207	0.01769	0.0551	0.01552	0.23052	0.28149	0.09755	-0.01141	0.37018	0.09848	-0.05106	0.02929	0.39584	0.23556	-0.08421
0.11375	0.01622	0.0229	-0.00064	0.22383	0.29593	0.18758	-0.00122	0.29962	0.1243	-0.06385	0.07365	0.38262	0.27553	-0.04357
0.03217	0.00019	0.03277	0.01961	0.20667	0.40109	0.07958	-0.0552	0.34754	0.12694	-0.05636	0.02716	0.37045	0.19145	-0.09593
0.10976	0.00699	0.07672	0.01951	0.19056	0.34455	0.05227	0.03118	0.34264	0.08158	-0.06271	0.04235	0.38314	0.2218	-0.088
0.12644	-0.0235	0.05954	0.02167	0.19726	0.25771	0.0612	0.01287	0.3452	0.08272	-0.06033	0.04542	0.37049	0.27249	-0.09197
0.10427	0.00985	0.007	0.00941	0.22192	0.36498	0.20693	-0.09532	0.31098	0.12075	-0.05925	0.07014	0.39302	0.29511	-0.05329
0.12657	-0.01618	0.02077	0.01332	0.22115	0.39475	0.23196	-0.09415	0.32065	0.11283	-0.04949	0.06771	0.38562	0.3215	-0.04827
0.02756	-0.01072	0.03346	-0.00187	0.2261	0.26634	0.1997	-0.01161	0.33063	0.11322	-0.04653	0.08106	0.37585	0.26511	-0.05394
0.14632	0.04825	0.07053	-0.00786	0.2216	0.28483	-0.10692	0.0866	0.34068	0.10868	-0.05109	0.02387	0.39404	0.16552	-0.08612
0.19602	0.02787	0.05777	-0.00617	0.21848	-0.08068	-0.0449	0.00485	0.32745	0.12169	-0.05187	0.10258	0.38945	0.21575	-0.0718
0.10323	0.07323	0.06647	0.00195	0.23135	0.22987	-0.2372	0.08491	0.34971	0.08495	-0.03573	0.01325	0.38864	0.06872	-0.11782
0.09634	0.01642	0.02241	0.00574	0.21037	0.38859	0.09492	-0.08319	0.31938	0.10993	-0.04819	0.04663	0.37702	0.26191	-0.09208
0.15867	0.01981	0.05419	0.01282	0.2234	0.23252	0.01735	0.0045	0.3059	0.12324	-0.04416	0.03652	0.3845	0.21787	-0.06145
0.19178	0.04026	0.07066	0.03434	0.22811	-0.21385	-0.22626	0.02103	0.32054	0.07783	-0.05248	0.02398	0.38571	0.07488	-0.09908
0.10836	0.03042	0.01687	0.00767	0.22215	0.35494	0.0807	-0.08444	0.36827	0.11628	-0.05166	0.05068	0.37548	0.2261	-0.08078
0.12569	-0.00683	0.03382	0.01954	0.20879	0.31636	0.14895	-0.10035	0.34308	0.12184	-0.05786	0.05455	0.39076	0.26189	-0.05148

ONSE_TO	ERCAPTO	ERCAPTO	ERCAPTO	ERCAPTO	_TO_MER	E_TO_MER	AIN_D6M	IN_D6MIT	N_HIGHE	IGHEST_(	R_BRAIN	_BRAIN_Q	CER_SUB	CER_SUB
0.24068	0.05917	-0.02133	0.09192	-0.03859	-0.01698	0.02092	0.41765	0.13337	0.22522	0.15443	0.17186	0.33631	0.01572	0.05623
0.18298	0.02954	-0.22204	-0.00377	-0.03725	-0.07542	0.09554	0.38489	0.41186	0.22555	0.13572	0.17936	0.29681	0.02211	0.02249
0.2552	0.00026	-0.19073	0.09948	-0.01902	0.00098	0.06828	0.39493	0.15539	0.17343	0.15376	0.18271	0.25572	0.03481	0.04623
0.26414	-0.0199	-0.31589	0.11746	-0.02377	-0.00202	0.00997	0.46997	0.10197	0.20022	0.15081	0.16819	0.2332	0.07427	0.05495
0.26131	0.02677	-0.33809	0.14474	-0.04117	0.06656	0.0003	0.43914	0.1479	0.17922	0.16103	0.18532	0.26882	0.05805	0.02846
0.24768	0.09626	-0.23012	-0.02649	0.04703	0.04786	-0.00596	0.486	0.20058	0.16789	0.15115	0.17822	0.25568	0.04723	0.00263
0.22975	0.00378	-0.23979	0.07739	-0.02165	0.01205	0.0264	0.46521	0.11899	0.19729	0.15363	0.17727	0.25965	0.10019	0.02423
0.27329	-0.02014	-0.3629	0.18664	-0.04676	0.03468	0.00498	0.48476	0.12118	0.17182	0.13553	0.15985	0.28594	0.08117	0.02053
0.27473	-0.01589	-0.33735	0.03751	-0.04696	0.08884	0.01001	0.48283	0.12889	0.16196	0.13437	0.14769	0.25276	0.06098	0.03798
0.27526	0.02544	-0.34898	0.15592	-0.05043	0.0529	0.00148	0.47877	0.12008	0.16821	0.14958	0.15687	0.26965	0.09026	0.01987
0.28925	-0.01807	-0.20778	0.18602	0.02	-0.03643	0.03813	0.27744	0.08828	0.21732	0.16594	0.17913	0.29199	0.02615	0.04733
0.22751	0.01191	-0.17024	0.08757	-0.00677	0.00694	0.01787	0.43879	0.12979	0.18521	0.11234	0.15762	0.38127	0.0905	0.01064
0.17108	0.00544	-0.01891	-0.02384	-0.05246	-0.02557	0.0138	0.4247	0.11478	0.2115	0.147	0.18876	0.31908	-0.03497	0.04317
0.2554	-0.01147	-0.34855	0.19299	-0.01358	0.0212	0.02446	0.44971	0.3714	0.20301	0.163	0.18099	0.28368	0.06474	0.06869
0.30487	0.0314	-0.19161	0.20437	0.00538	-0.06301	0.04075	0.45957	0.13121	0.21567	0.12847	0.16897	0.26552	0.054	0.04848
0.13881	0.0211	-0.22661	0.09414	-0.01646	-0.049	0.09748	0.16171	0.42347	0.19461	0.15352	0.18443	0.32945	-0.03275	0.03762
0.26206	-0.00752	-0.09466	0.10712	-0.03465	-0.01082	0.03153	0.46961	0.1251	0.22452	0.15416	0.17969	0.22424	0.06057	0.03871
0.2313	-0.02617	-0.37648	0.05487	-0.0579	0.05516	0.01029	0.45652	0.13111	0.19351	0.14431	0.17205	0.28821	0.04098	0.01561

R_SUBCL	LR_SUBC	IER_SUBC	ER_SUBC	_SUBCL	A_SUBCL	R_SUBCL	LR_SUBCL	TIC_TRAN	TIC_TRAN	_TRANSF	_TRANSF	TIC_TRA	TIC_TRA	PHOBLA	REFRAC	L_RELAP	ESPONSE	SPONSE_T	SPONSE_1	TARGETS
-0.08623	0.20272	0.16308	0.06334	-0.00364	0.08075	0.17947	0.0169	0.1244	0.08104	-0.11184	0.10603	0.08062	0.0765	0.02998	0.12932	0.43043	0.17813	0.05677	0.15975	0.12032
0.01181	0.19066	0.14939	0.07955	-0.05438	0.08726	0.19209	0.0181	0.10422	0.08461	-0.16745	0.05502	0.07768	0.10538	0.01636	0.15388	0.43674	0.19513	-0.0326	0.13143	0.09633
-0.08325	0.24811	0.09734	0.0883	-0.00277	0.08526	0.1957	0.0122	0.12921	0.0664	-0.10425	0.10292	0.10022	0.09647	0.03338	0.1674	0.44535	0.18349	0.03458	0.15592	0.12886
-0.05145	0.22267	0.12417	0.0789	-0.00036	0.07741	0.17926	0.08464	0.13421	0.07621	-0.13788	0.11078	0.11398	0.08473	0.05606	0.14942	0.42137	0.17194	-0.0022	0.16709	0.13924
-0.0714	0.15717	0.0543	0.08066	-0.02192	0.07481	0.18337	0.0134	0.10207	0.07401	-0.15619	0.07707	0.06571	0.0787	0.03831	0.19002	0.45	0.18157	0.0054	0.14359	0.11081
-0.11421	0.1958	0.0996	0.08389	0.04766	0.06926	0.18838	0.03622	0.07717	0.10238	-0.09929	0.10268	0.06948	0.0971	0.07644	0.21168	0.44168	0.18425	0.01159	0.18851	0.12516
-0.04996	0.27655	0.12208	0.04306	0.02176	0.05982	0.1813	0.04335	0.1237	0.1019	-0.08693	0.11931	0.08991	0.11484	0.05105	0.16985	0.42404	0.19663	0.03825	0.16465	0.14351
-0.16352	0.22796	0.14892	0.02702	0.02205	0.06226	0.17954	0.04154	0.12164	0.08637	-0.05964	0.10129	0.11041	0.08915	0.05439	0.14764	0.42086	0.16646	-0.02263	0.14292	0.14463
-0.1273	0.19874	0.06484	0.06886	0.02387	0.05747	0.17753	0.04939	0.13163	0.08012	-0.07069	0.09849	0.10711	0.08288	0.05156	0.16659	0.42278	0.18282	-0.03879	0.16331	0.14137
-0.14178	0.19554	0.05478	0.10175	0.00149	0.06699	0.18234	0.03928	0.0983	0.07276	-0.10952	0.0908	0.07962	0.07095	0.06346	0.16542	0.43032	0.16334	0.00501	0.15025	0.12394
-0.06403	0.16386	0.15512	0.04804	0.00408	0.08683	0.19327	0.03595	0.10627	0.07942	-0.09172	0.08332	0.06379	0.10746	0.02981	0.14564	0.44972	0.17621	0.07395	0.14332	0.13376
-0.0505	0.14479	0.13934	0.05628	0.03311	0.06802	0.18566	0.08501	0.11919	0.07479	-0.0932	0.08382	0.09745	0.09637	0.03824	0.20319	0.44111	0.18687	0.05531	0.1262	0.13958
-0.09042	0.06982	0.10469	0.0458	0.0018	0.05467	0.18754	0.00337	0.10998	0.04064	-0.17638	0.12246	0.0855	0.10076	0.05043	0.21518	0.4382	0.1606	0.10146	0.14353	0.10488
-0.06525	0.16168	0.1011	0.08607	0.00115	0.10745	0.1798	0.0407	0.1349	0.06751	-0.12911	0.11222	0.10705	0.08508	0.05626	0.12095	0.4498	0.17616	0.04201	0.12491	0.12224
-0.12867	0.20918	0.14801	0.08501	-0.00095	0.08394	0.19186	0.01401	0.10608	0.09815	-0.08087	0.08176	0.06795	0.08376	0.02379	0.15059	0.44249	0.19926	0.04951	0.13593	0.14245
0.00793	0.12423	0.09887	0.08363	-0.01497	0.06856	0.18605	-0.00203	0.1196	0.05559	-0.16662	0.08009	0.0766	0.08188	0.02672	0.14839	0.43135	0.17552	0.05734	0.10083	0.11123
-0.0062	0.11787	0.04278	0.07836	-0.01964	0.0593	0.17786	0.04624	0.13454	0.09922	-0.13395	0.10768	0.12878	0.07959	0.05251	0.09425	0.42839	0.19568	0.01565	0.17102	0.10905
-0.04466	0.25214	0.063	0.05659	-0.00646	0.04447	0.18573	0.03014	0.13025	0.07884	-0.11117	0.08864	0.11189	0.08466	0.03284	0.2178	0.43138	0.19512	-0.0175	0.15244	0.12524

_TARGET	TARGETS	_TARGETS	NCER_C	NCER_C	MYELOMA	MYELOMA	L_ACUT
0.04982	0.2422	0.08499	0.31566	0.08582	0.10515	0.38249	-0.40796
0.04003	0.17455	0.08068	0.32227	0.04068	0.07443	0.39128	-0.34296
0.09116	0.19531	0.08502	0.31638	0.06112	0.08928	0.37291	-0.43984
0.05506	0.21504	0.07825	0.30068	8E-05	0.12401	0.3845	-0.41008
0.05962	0.21787	0.0861	0.24141	0.04146	0.1134	0.3753	-0.39387
0.10016	0.19893	0.08194	0.25885	0.05405	0.11717	0.39318	-0.48547
0.08737	0.18645	0.09487	0.28852	0.06736	0.14973	0.38122	-0.26017
0.04549	0.18909	0.07306	0.28217	0.01017	0.15374	0.37252	-0.4948
0.04987	0.19731	0.07275	0.28053	0.03222	0.12664	0.37022	-0.32165
0.04177	0.1905	0.08289	0.30456	-0.00363	0.14624	0.37688	-0.30879
0.10117	0.22665	0.09651	0.31011	0.08378	0.094	0.38016	-0.21156
0.10254	0.22191	0.10305	0.29191	0.06975	0.10091	0.38497	-0.35811
0.06038	0.19078	0.08423	0.31096	0.10507	0.13775	0.37629	0.02061
0.04333	0.17931	0.07099	0.29338	0.03804	0.11637	0.38169	-0.26757
0.09424	0.21019	0.10165	0.29826	0.03154	0.11292	0.39171	-0.4985
0.07493	0.191	0.09077	0.29927	0.11497	0.08423	0.387	-0.34754
0.02787	0.17999	0.07509	0.34065	0.03784	0.11948	0.38842	-0.49797
0.03187	0.19618	0.0637	0.29909	0.02649	0.12655	0.36689	-0.35318

RONIC_V	STAGE_P	SSF1_TA	SSF1_TA	R4AI_TA	VE_STRE	VE_STRE	ER_CYT	TER_CYT	FA2T3_TA	FA2T3_TA	ESSELS_D	ESSELS_D	SITIVE_M	SITIVE_M
0.27234	0.20678	0.10591	0.10443	0.09496	0.2525	0.13647	0.07966	0.15732	0.0697	0.11116	-0.07218	0.01929	-0.03796	0.23503
0.2758	0.1979	0.12892	0.1467	-0.01065	0.25724	0.02613	0.01473	0.14318	0.0123	0.04071	-0.12667	0.05561	-0.08666	0.18436
0.26694	0.19141	0.08865	0.11229	0.15706	0.24688	0.07011	0.04296	0.15912	0.0498	0.09857	-0.06132	-0.02891	-0.05834	0.22173
0.24983	0.19686	0.12453	0.09005	0.11885	0.24691	0.07197	0.0894	0.17199	0.04195	0.1254	-0.045	-0.03768	-0.07259	0.26513
0.25819	0.18156	0.1126	0.06426	0.06728	0.2433	0.04038	0.13644	0.14614	0.08054	0.12729	-0.1029	-0.10058	-0.23621	0.22735
0.25126	0.18701	0.13975	0.07081	0.13554	0.21596	0.01704	0.03827	0.14358	0.04277	0.07116	-0.00364	-0.02246	-0.19326	0.15868
0.2446	0.18589	0.14759	0.04128	0.13636	0.20636	0.04966	0.04866	0.15417	0.03416	0.09154	0.12791	0.1353	-0.27849	0.21251
0.23051	0.18815	0.12463	0.06063	0.15127	0.24178	0.06021	0.13464	0.16234	0.07451	0.13513	-0.00039	0.00663	-0.03271	0.16147
0.24644	0.18003	0.11993	0.11076	0.12089	0.23752	0.05326	0.12593	0.16525	0.04163	0.13499	0.00833	-0.04413	-0.05599	0.16357
0.24473	0.18177	0.12699	0.06907	0.10702	0.24588	0.0643	0.13874	0.16437	0.0564	0.152	-0.04074	-0.08896	-0.18992	0.18396
0.25627	0.1952	0.10897	0.0283	0.07149	0.22834	0.01461	0.04379	0.14737	0.05147	0.09	-0.12617	0.02536	-0.10937	0.18965
0.27137	0.17963	0.11256	0.10093	0.142	0.2181	0.0875	0.04855	0.15731	0.06913	0.08779	-0.03111	-0.0584	-0.16229	0.20465
0.30535	0.22721	0.14302	0.088	-0.0014	0.21987	0.00203	0.03095	0.14232	-0.0083	0.01283	-0.15627	0.04055	-0.127	0.16805
0.24729	0.19363	0.10199	0.04815	0.07907	0.2178	0.07509	0.07464	0.15488	0.05155	0.12683	-0.0628	-0.05124	-0.15982	0.19401
0.26714	0.18788	0.12381	0.11849	0.11271	0.27973	0.04972	0.08204	0.14726	0.09024	0.12433	-0.00903	-0.01255	-0.205	0.15872
0.29096	0.22472	0.15059	0.0444	0.01832	0.28183	0.01216	-0.03038	0.14106	0.02801	0.00711	-0.08898	0.04983	0.00616	0.14826
0.24904	0.19125	0.10662	0.07297	0.04942	0.2349	0.07781	0.05708	0.14	0.03713	0.12199	-0.07316	-0.04402	-0.04061	0.17781
0.23222	0.18331	0.1316	0.04317	0.10919	0.26506	0.05003	0.09348	0.16024	0.02274	0.13587	-0.08043	0.01742	-0.0508	0.18666

CANCER_	CANCER_	OPHAGE_	OPHAGE_	TINIB_R	TINIB_R	IS_OF_4E	OCYTOMA	OCYTOMA	MYC_TAR	MYC_TAR	KDC_RE	KDC_SU	TOSIS_B
-0.08913	0.04758	0.10913	0.00594	0.01497	0.05555	0.08094	0.2066	0.26611	0.14496	0.32117	0.16255	0.14537	0.11935
-0.20707	-0.06013	0.07688	-0.00077	-0.04378	0.10877	0.06349	0.14772	0.23539	0.11718	0.31463	0.17623	0.23414	0.13985
-0.11271	0.02644	0.1029	-0.00864	-0.044	0.07122	0.0826	0.13702	0.2325	0.1439	0.3389	0.16725	0.15713	0.12487
-0.01473	-0.04659	0.13792	0.07231	-0.02133	0.03982	0.08743	0.18588	0.30919	0.15725	0.29864	0.18238	0.14538	0.12741
-0.09474	0.00947	0.12541	0.03467	-0.09503	0.02867	0.08075	0.10885	0.25934	0.13383	0.26186	0.19747	0.17675	0.11059
-0.25572	-0.00221	0.1383	0.00133	-0.04936	0.08925	0.08599	0.18337	0.21614	0.22336	0.28342	0.20874	0.16058	0.1307
-0.21878	0.03932	0.13218	0.01445	-0.03825	0.07265	0.09902	0.17493	0.25423	0.17426	0.33701	0.21971	0.10572	0.13047
-0.07723	0.00792	0.12562	0.09655	-0.09189	0.04721	0.08932	0.15829	0.28797	0.1951	0.26259	0.17535	0.12683	0.10616
-0.04163	-0.01788	0.13605	0.08072	-0.08008	0.05262	0.09006	0.15039	0.27647	0.19214	0.22229	0.19244	0.14433	0.11833
-0.06935	0.00199	0.14355	0.06856	-0.0897	0.03896	0.09469	0.13674	0.26934	0.18382	0.27677	0.19062	0.16064	0.11001
-0.36129	-0.03854	0.1216	0.0241	0.00711	0.09322	0.0836	0.12353	0.23004	0.12113	0.33622	0.18606	0.12994	0.13646
-0.22423	0.0188	0.13557	0.04786	0.01476	0.06031	0.08112	0.15056	0.28036	0.17548	0.29716	0.16021	0.15296	0.13426
0.01418	0.032	0.07665	-0.01116	0.05477	0.04324	0.06797	0.08918	0.20472	0.18664	0.32674	0.24798	0.16138	0.12659
-0.11629	-0.01769	0.117	0.02911	-0.01712	0.05226	0.0741	0.12695	0.23342	0.20715	0.27903	0.18489	0.14125	0.09805
-0.31274	-0.01765	0.13986	0.00347	-0.03652	0.09265	0.0873	0.1617	0.25292	0.15135	0.31337	0.16348	0.13788	0.12403
-0.14584	0.06342	0.0799	-0.03028	0.02152	0.11536	0.06481	0.11401	0.22415	-0.05153	0.36118	0.19166	0.12072	0.11083
-0.07277	-0.01338	0.09294	0.02048	-0.00735	0.07135	0.06605	0.16292	0.27169	0.20116	0.31923	0.21653	0.16749	0.09932
-0.05659	-0.01304	0.09929	0.05633	-0.07875	0.04117	0.08839	0.1243	0.28268	0.17491	0.27975	0.18969	0.14222	0.10331

TOSIS_B	RMLINE_A	D_STEM	7_DIRECL	TIPLE_M	TIPLE_M	ORAL_TG	ORAL_TG	G_MYCN_	_SIGNALI	_SIGNALI	RINE_THE	RINE_THE	RINE_THE	RINE_THE	RINE_THE	R_METAST	R_METAST	UMORIGE	UMORIGE
0.09992	0.11414	0.00998	-0.01066	-0.01141	-0.07915	0.08156	0.04041	0.00186	0.28772	0.12041	0.08127	0.06016	0.04936	0.06496	0.05865	0.1604	0.12709	0.15666	-0.01682
0.09406	0.15325	0.01188	0.07729	-0.04299	-0.08678	0.05976	0.00672	0.01408	0.28727	0.16432	0.09264	0.06071	0.02094	0.06736	0.02836	0.14531	0.15549	0.07574	0.01692
0.10696	0.09651	0.02293	0.07539	0.00108	0.03164	0.07525	0.0155	0.05433	0.28786	0.1301	0.08856	0.04728	0.02725	0.06359	0.0445	0.14791	0.12719	0.08276	-0.04208
0.11056	0.03164	-0.04048	0.01134	0.07286	-0.0008	0.0643	0.01151	0.06027	0.30256	0.12364	0.08002	0.04518	0.04545	0.05106	0.04228	0.11618	0.12571	0.1545	-0.00225
0.09889	0.07436	-0.01289	0.03464	0.03466	-0.0249	0.05179	-0.01602	-0.11748	0.28366	0.10375	0.08089	0.06219	0.02931	0.04883	0.02297	0.09648	0.11821	0.11627	-0.05476
0.10563	0.0832	0.0141	0.032	0.03824	0.00172	0.06645	0.01264	-0.01488	0.26842	0.0893	0.09087	0.05384	0.02252	0.04355	0.01654	0.12394	0.12741	0.05399	0.00519
0.0949	0.06178	0.01896	0.01925	0.07565	-0.00491	0.06106	-0.02075	-0.07623	0.27967	0.14054	0.07607	0.05989	0.02562	0.0597	0.01503	0.14502	0.10596	0.08542	0.025
0.12694	0.11595	-0.05852	0.04138	0.08995	0.08558	0.04718	-0.00269	-0.01609	0.28475	0.09249	0.06557	0.05137	0.04587	0.06309	0.04531	0.05083	0.11074	0.11103	-0.01673
0.1134	0.08113	-0.04553	0.02219	0.07443	0.09845	0.06864	-0.00969	0.03977	0.2831	0.10134	0.07529	0.05902	0.02988	0.05128	0.03304	0.07391	0.1332	0.10932	0.08316
0.10573	0.06812	-0.03575	0.02619	0.0804	0.00861	0.0438	-0.02318	-0.03575	0.28407	0.10949	0.06788	0.05539	0.02803	0.04501	0.02392	0.08021	0.12504	0.12485	-0.04456
0.0988	0.1033	0.01941	0.06467	0.03663	-0.04517	0.0668	0.00633	0.092	0.28794	0.11207	0.1031	0.05395	0.03373	0.06564	0.03558	0.17744	0.12488	0.11201	0.02833
0.10121	0.12292	-0.05021	-0.02441	0.03582	0.03248	0.07511	-0.00687	0.02785	0.28579	0.09976	0.09561	0.03614	0.03466	0.05243	0.04536	0.13863	0.1408	0.15117	-0.01999
0.08185	0.06006	0.08741	0.03238	0.01678	-0.1331	0.11886	0.0589	-0.01662	0.29109	0.1668	0.08536	0.03615	0.02825	0.05678	0.04003	0.19257	0.08358	0.10938	-0.14182
0.11203	0.1223	0.01136	0.07123	-0.0106	-0.01147	0.09603	0.0075	-0.00744	0.28579	0.14746	0.08464	0.04883	0.05269	0.07201	0.05575	0.11135	0.12083	0.12051	-0.00474
0.10397	0.13906	-0.03076	0.10082	0.0614	0.02753	0.06788	0.00206	0.03259	0.28082	0.11649	0.09879	0.06402	0.0377	0.0745	0.0502	0.12217	0.13672	0.13293	0.00977
0.08283	0.09559	0.06317	0.0504	-0.02838	-0.06679	0.06665	0.02512	0.04013	0.2964	0.14111	0.10401	0.02555	0.01525	0.05956	0.04116	0.18899	0.10384	0.10507	-0.01188
0.10116	0.1801	0.01295	0.07708	0.00689	-0.01877	0.08266	0.01327	0.0076	0.31186	0.12661	0.09065	0.04512	0.03699	0.06436	0.04208	0.14968	0.12382	0.12471	0.01538
0.10436	0.12049	-0.00374	0.0235	0.0125	-0.02934	0.0407	-0.00619	0.05204	0.28325	0.10814	0.08631	0.04161	0.02526	0.05391	0.0266	0.08858	0.13738	0.06459	-0.02425

IL6_DEP	IL6_DEP	NRAS_SI	NRAS_SI	S_STRO	S_STRO	ROMAL_	ROMAL_	OSE_DE	21_TARGE	21_TARGE	21_TARG	21_TARG	URAL_K
0.05667	0.22116	0.085	0.06808	-0.00712	0.03769	-0.05022	0.01953	0.17089	0.01117	0.08427	0.16011	-0.00674	0.03301
0.09957	0.2129	0.11535	0.04686	-0.01662	0.02382	-0.01691	-0.00147	0.15637	0.00133	0.09995	0.16472	-0.01592	-0.41179
0.11006	0.26671	0.13386	0.08998	-0.01873	0.07976	0.01581	0.01254	0.18158	0.02098	0.09537	0.13594	-0.03786	0.00786
0.07483	0.35185	0.08564	0.19304	-0.03166	0.12262	0.08576	0.03393	0.15625	0.0417	0.06912	0.18089	0.00607	-0.22559
0.0538	0.36065	0.06272	0.16972	-0.06208	0.10844	0.0411	-0.04864	0.14819	0.01194	0.06944	0.09425	-0.05556	0.21688
0.11037	0.22556	0.13141	0.03818	0.02561	0.06238	0.0646	0.09371	0.17325	-0.01121	0.10196	0.05655	0.11678	0.10013
0.09918	0.29034	0.12193	0.09608	0.05834	0.10093	0.09943	0.14854	0.16787	0.01257	0.1344	0.07249	0.1598	-0.09675
0.03062	0.36176	0.04139	0.20904	-0.02386	0.12399	0.05871	0.07469	0.15306	0.03857	0.05825	0.11497	-0.05025	-0.13829
0.04675	0.36797	0.05509	0.21237	-0.01702	0.11554	0.05513	0.07024	0.1562	0.04173	0.07007	0.10738	0.02046	0.09952
0.04522	0.36139	0.06017	0.20556	-0.02886	0.11484	0.0508	-0.00693	0.1555	0.02561	0.07427	0.0923	-0.00149	0.20535
0.11585	0.12729	0.14649	0.06808	-0.01019	0.04311	-0.01228	-0.03378	0.20417	0.01125	0.09741	0.08724	0.01923	-0.08138
0.07853	0.26192	0.10936	0.04287	-0.02098	0.06326	0.03088	0.04465	0.1905	0.0162	0.09775	0.11559	0.02756	0.12971
0.0851	0.12015	0.13049	-0.09161	-0.00193	0.00656	0.06262	-0.02665	0.18391	0.00591	0.09878	0.13461	-0.04436	-0.14521
0.03362	0.28707	0.04443	0.1621	-0.04112	0.08932	-0.0017	0.02379	0.1547	0.02402	0.07473	0.13133	0.02824	0.11982
0.09526	0.24627	0.11762	0.1276	-0.01343	0.07988	0.03428	0.02241	0.18835	0.00875	0.08887	0.11517	0.02233	-0.12022
0.11209	0.17093	0.14754	-0.06735	-0.03025	-0.02867	-0.01492	-0.06932	0.19447	0.00099	0.10432	0.15221	-0.08906	-0.13576
0.01738	0.29953	0.01467	0.1297	-0.04085	0.08166	0.00371	0.02442	0.13683	0.01515	0.08607	0.17141	0.01062	-0.12336
0.03255	0.28266	0.04813	0.16653	-0.01424	0.09038	0.02156	0.02715	0.15504	0.03514	0.05737	0.09918	-0.03262	0.11552

ALLELE_	ALLELE_	RESPON	_RESPON	NSE_VIA	NSE_VIA	ESPONSE	PONSE_V	PONSE_V	ESPONSE	ONSE_VI	ONSE_VI	R_PRONE	PRONE_	ER_PRONE
0.06643	0.01823	0.37451	-0.09308	0.00022	0.0836	0.00658	-0.08998	0.14204	0.1263	-0.03143	-0.11083	0.23025	0.24146	0.12819
0.02635	-0.0697	0.39027	0.00391	-0.01378	0.07509	-0.00121	-0.0754	0.11705	0.1234	-0.05412	-0.10767	0.23407	0.23712	0.1284
0.13617	-0.00696	0.38923	0.08388	0.00748	0.08486	0.01451	-0.03334	0.14617	0.14524	-0.00825	-0.08459	0.24715	0.23125	0.15536
0.21673	0.00587	0.39578	0.07276	-0.01102	0.098	-0.0021	-0.07039	0.12642	0.13424	-0.07485	-0.09765	0.23199	0.2458	0.13383
0.07331	-0.00057	0.39016	0.11342	-0.01537	0.07539	-0.0099	-0.09609	0.10727	0.12861	-0.04985	-0.10223	0.236	0.23665	0.11655
0.03831	-0.01138	0.3743	0.09099	-0.01154	0.10055	-0.00288	-0.10031	0.10071	0.12491	-0.04633	-0.07117	0.23769	0.24233	0.15018
0.08882	0.03104	0.40348	0.03397	-0.02206	0.08447	-0.01084	-0.09664	0.11864	0.13456	-0.04882	-0.0913	0.25124	0.22687	0.16884
0.22685	0.04742	0.38229	0.10285	-0.02669	0.0791	-0.01245	-0.08583	0.1301	0.12433	-0.06918	-0.07498	0.21471	0.23317	0.09757
0.19192	0.04381	0.37469	0.13141	-0.02943	0.08202	-0.01071	-0.07426	0.12703	0.12679	-0.03878	-0.06433	0.21726	0.23974	0.07129
0.14783	0.0021	0.3863	0.10577	-0.02648	0.08302	-0.01697	-0.10115	0.13708	0.13113	-0.06795	-0.05196	0.22979	0.23208	0.14044
-0.13989	-0.05327	0.37417	0.01132	0.00727	0.10194	0.01112	-0.05312	0.13657	0.14658	-0.00687	-0.07038	0.28529	0.22856	0.16512
0.04719	0.04479	0.39145	-0.04915	-0.00968	0.11592	-0.00873	-0.1033	0.13542	0.14471	-0.04781	-0.04266	0.23393	0.24762	0.17204
-0.11428	-0.10193	0.39667	-0.06054	0.01194	0.09694	0.01601	-0.05144	0.12219	0.12511	-0.01396	-0.13809	0.26024	0.23032	0.16173
0.10902	-0.04221	0.37505	0.08578	-0.01092	0.11088	-0.00385	-0.05728	0.11341	0.12956	-0.04032	-0.07664	0.2194	0.22432	0.12303
0.08993	-0.04208	0.37371	0.0512	-0.01239	0.09986	-0.00303	-0.08435	0.138	0.13976	-0.02258	-0.01268	0.2291	0.2405	0.16205
-0.00806	-0.12642	0.40273	0.03041	0.00612	0.10965	0.01127	-0.0515	0.16614	0.13438	-0.01703	-0.09186	0.26162	0.2284	0.19724
0.21696	-0.01893	0.38273	0.00333	-0.00503	0.07953	0.00423	-0.05532	0.12024	0.12123	-0.03747	-0.11438	0.24404	0.23184	0.15668
0.114	0.0221	0.3928	0.1211	-0.03023	0.10199	-0.02013	-0.10777	0.13756	0.13751	-0.05805	-0.00149	0.23775	0.2325	0.11274

D_TSA_RE	BOUND_B	YC_TARG	YC_TARG	ULATED_B	ULATED_	LOMA_PR	PILLOMA_	PILLOMA_	PILLOMA_	PILLOMA_	_TUMOR	N_TUMOR	IOUS_CEL
0.19808	0.17393	0.1778	0.27813	0.12732	0.11585	0.05964	0.15651	0.159	0.14288	0.16864	0.13355	0.18241	0.10386
0.14389	0.1799	0.14598	0.29905	0.10432	0.15721	0.0566	0.13907	0.1421	0.12119	0.15485	0.12165	0.1601	0.09244
0.16995	0.17193	0.15709	0.29099	0.13974	0.13714	0.07083	0.14268	0.15179	0.13103	0.15977	0.11141	0.18422	0.08948
0.16269	0.17047	0.18857	0.28851	0.14881	0.10409	0.03393	0.15312	0.14558	0.13894	0.15627	0.11965	0.17862	0.10151
0.29356	0.17427	0.1728	0.28178	0.13005	0.11337	0.05426	0.13244	0.16633	0.1148	0.17691	0.11396	0.1881	0.09324
0.16757	0.17282	0.15098	0.28993	0.1356	0.13827	0.09656	0.14743	0.1486	0.12301	0.16586	0.11581	0.18034	0.10025
0.16458	0.16896	0.16445	0.30023	0.13938	0.15732	0.071	0.14862	0.14307	0.12527	0.1602	0.12462	0.16718	0.09167
0.17657	0.16842	0.20003	0.27468	0.16364	0.09693	0.06534	0.14203	0.14648	0.12267	0.16006	0.11327	0.17563	0.10468
0.14984	0.16243	0.17129	0.27014	0.17185	0.09774	0.03885	0.14101	0.1501	0.13382	0.15486	0.11552	0.17662	0.09207
0.16363	0.16961	0.19278	0.2848	0.14975	0.11016	0.07665	0.12723	0.15062	0.10465	0.16501	0.11109	0.16945	0.08612
0.15801	0.17215	0.10778	0.29783	0.0979	0.1362	0.06643	0.13716	0.15223	0.112	0.16956	0.13045	0.16082	0.09606
0.16223	0.17485	0.16789	0.29509	0.14439	0.12176	0.0993	0.13613	0.15923	0.12832	0.16372	0.11682	0.18148	0.09418
0.20222	0.16768	0.13252	0.29435	0.0944	0.14101	0.09415	0.1417	0.13029	0.12294	0.1447	0.12457	0.14668	0.09356
0.18948	0.16898	0.16386	0.29262	0.14233	0.11385	0.04634	0.14514	0.16314	0.13699	0.1682	0.13827	0.17223	0.11798
0.19157	0.17465	0.14667	0.30324	0.13601	0.14397	0.09446	0.14632	0.16324	0.13158	0.17292	0.13635	0.17523	0.10112
0.16199	0.1734	0.13483	0.30814	0.10752	0.18159	0.11249	0.13397	0.15167	0.11683	0.16342	0.1324	0.15529	0.09068
0.19516	0.17218	0.1999	0.28283	0.13963	0.10909	0.04173	0.14399	0.1502	0.13523	0.15623	0.111	0.18368	0.09317
0.17488	0.16658	0.18525	0.27052	0.15252	0.10373	0.05386	0.13746	0.14698	0.11729	0.1608	0.1126	0.17299	0.09216

IOUS_CEL	6_SIGNAL	IGNALIN	SIGNALIN	6_SIGNAL	TAT3_TAR	TAT3_TAR	ULAR_A	CULAR_A	RO1_SIGN	O1_SIGN	ARCOMA	ARCOMA	ARCOMA_OF_PAX
0.20116	0.17679	0.04208	0.00669	0.18509	0.07014	0.16127	-0.01362	0.06134	0.13948	0.0096	0.12708	0.08256	0.17804
0.17971	0.09784	-0.01263	0.03539	0.15359	0.10084	0.13383	-0.02898	0.0828	0.09136	-0.01799	0.09994	0.04306	0.16148
0.19477	0.15901	0.04103	-0.01131	0.1939	0.13271	0.15364	-0.01441	0.06464	0.0425	0.01141	0.13124	0.07655	0.17018
0.18717	0.1215	0.12062	-0.09964	0.16763	0.13148	0.22006	0.00469	0.05082	0.10651	0.01783	0.05349	0.08103	0.15421
0.1976	0.10279	0.02087	-0.03728	0.15001	0.09657	0.2048	-0.03602	0.07387	0.06697	0.02644	0.06837	0.11949	0.15997
0.18666	0.21681	0.10438	0.00575	0.12421	0.07622	0.2115	0.00549	0.08375	0.16346	0.00888	0.10153	0.08487	0.17726
0.18916	0.182	0.09065	0.08023	0.17995	0.17068	0.17952	0.01673	0.07142	0.11006	0.02488	0.08634	0.08481	0.17189
0.17657	0.11958	0.10934	-0.02082	0.16944	0.12125	0.20348	-0.00181	0.06528	0.08994	0.0208	0.05412	0.10911	0.18575
0.18867	0.07034	0.08421	0.02991	0.17308	0.14716	0.24061	-0.0012	0.06726	0.08449	0.00098	0.02005	0.07891	0.17403
0.18351	0.09494	0.0855	-0.04889	0.14112	0.19153	0.20918	-0.0037	0.06444	0.06101	-0.01484	0.08955	0.08745	0.17246
0.18547	0.15387	-0.00787	0.03495	0.10878	0.06762	0.16783	-0.01486	0.06954	0.11087	0.04548	0.07586	0.07197	0.18388
0.19292	0.22414	0.01378	-0.00417	0.182	0.15066	0.19876	-0.00286	0.06611	0.15014	-6.4E-05	0.16752	0.07312	0.19453
0.17011	0.09263	-0.02439	-0.0048	0.09515	0.08665	0.17996	-0.01592	0.0871	0.06608	0.09209	-0.00554	0.09571	0.13746
0.18556	0.09985	0.03049	-0.0315	0.16073	0.08046	0.19271	-0.00581	0.07291	0.07441	0.03691	0.10727	0.10777	0.1725
0.19909	0.07958	-0.00383	-0.0133	0.15709	0.09472	0.18885	-0.00029	0.0608	0.10196	0.0261	0.13851	0.11235	0.18705
0.18675	0.12179	-0.02593	0.03731	0.16149	0.11304	0.11213	0.0102	0.06641	0.04492	0.03533	0.12321	0.02568	0.18379
0.19165	0.10354	0.05208	0.03368	0.1753	0.03399	0.21208	0.00754	0.0611	0.13267	-0.00775	0.12034	0.07141	0.18906
0.18345	0.14182	0.06424	-0.01148	0.14749	0.17556	0.21463	-0.01767	0.05181	0.07726	0.0163	0.0724	0.10035	0.18012

ONSE_TO	ONSE_TO	ONSE_TO	PONSE_TO	ESPONSE	ESPONSE	ESPONSE_	ESPONSE_	ESPONSE	ESPONSE	ESPONSE	ESPONSE	BY_REOV	BY_REOV	S_REPRE	RARG_B	X_RAR_B	RAR_TAX	RAR_TA	MYOD_TA	MYOD_T
-0.08818	0.21007	-0.09395	0.22722	0.24356	0.10806	0.10431	0.23024	0.15177	0.28446	0.38349	-0.06065	0.0622	0.07289	0.38876	0.10326	0.05388	0.12425	0.06995	0.09758	0.05076
-0.07052	0.20156	-0.03769	0.20615	0.24783	0.04679	0.11645	0.11089	0.10333	0.27684	0.38773	-0.04576	0.07091	0.06512	0.13775	0.09863	0.05835	0.12736	0.03407	0.03604	0.04027
-0.07623	0.19328	-0.05439	0.23101	0.22469	0.03877	0.08946	0.04033	0.13443	0.27228	0.39091	-0.04479	0.06877	0.07664	0.40884	0.10573	0.05028	0.15374	0.0389	0.02906	0.06261
-0.09805	0.20586	-0.09454	0.21558	0.24885	0.0266	0.09532	0.11753	0.13321	0.24247	0.39176	-0.07788	0.06813	0.07727	0.3789	0.11722	0.05532	0.14658	0.04788	0.06174	0.02552
-0.08577	0.18411	-0.08535	0.20275	0.25003	-0.0105	0.10785	-0.00989	0.137	0.25092	0.37072	-0.05813	0.06248	0.06146	0.31848	0.10503	0.03016	0.10561	0.0179	0.03132	0.02014
-0.07212	0.18111	-0.06724	0.20129	0.23177	0.02219	0.03053	0.0631	0.14143	0.26208	0.3552	-0.03834	0.07141	0.07184	0.18654	0.08496	0.05559	0.14379	0.00331	0.0347	0.05292
-0.09746	0.20877	-0.07674	0.21107	0.25208	0.10498	0.17866	0.15095	0.10322	0.30058	0.36527	-0.07392	0.0753	0.08829	0.31795	0.10007	0.05671	0.09838	0.04433	0.05542	0.0304
-0.11396	0.19782	-0.09989	0.19872	0.25455	-0.01238	0.08956	0.06145	0.12379	0.2479	0.39099	-0.09795	0.05578	0.06125	0.39035	0.10135	0.03801	0.09753	0.00362	0.03166	0.03619
-0.10406	0.19699	-0.11201	0.21489	0.2406	0.01486	0.12821	0.06367	0.11887	0.28396	0.39013	-0.09356	0.06893	0.0738	0.42998	0.1022	0.0419	0.09496	-0.01531	0.03562	0.01527
-0.09284	0.20141	-0.1153	0.20329	0.25072	0.02282	0.0711	0.0896	0.12511	0.27272	0.38264	-0.07937	0.05639	0.09116	0.36886	0.08877	0.04433	0.11349	-0.02347	0.04201	-0.0004
-0.08017	0.2029	-0.04721	0.23541	0.24164	0.10169	0.08664	0.20692	0.14546	0.26077	0.32342	-0.04108	0.05786	0.0614	0.38753	0.1096	0.05169	0.1126	0.02706	0.04957	0.05869
-0.10783	0.20437	-0.10446	0.22137	0.25238	0.11279	0.12532	0.15244	0.1168	0.28259	0.35992	-0.07975	0.07386	0.08746	0.40356	0.10689	0.04298	0.18634	-0.03339	0.09691	0.01758
-0.05432	0.1979	-0.0338	0.22617	0.23935	0.06594	-0.01969	0.2027	0.13948	0.31346	0.32189	-0.02946	0.10174	0.0776	0.34332	0.1163	0.08181	0.09747	0.06516	0.00881	0.07894
-0.08516	0.20728	-0.05504	0.21344	0.2415	0.10275	0.1205	0.11564	0.14143	0.30964	0.36802	-0.06312	0.06513	0.06702	0.37432	0.11856	0.04339	0.02896	0.06826	0.04124	0.04524
-0.09589	0.20755	-0.07434	0.21129	0.24483	0.05485	0.06255	0.14635	0.1411	0.2764	0.35786	-0.07219	0.06099	0.07536	0.31407	0.09955	0.03576	0.1047	0.00552	0.03293	0.03954
-0.08505	0.19164	-0.03559	0.23044	0.23155	0.10322	-0.03315	0.15957	0.08954	0.25642	0.35609	-0.04587	0.06424	0.07444	0.19095	0.09088	0.05346	0.11061	0.05536	0.00798	0.03326
-0.07425	0.20861	-0.05453	0.2073	0.24114	0.07151	0.12439	0.11097	0.12846	0.28149	0.39598	-0.04542	0.07181	0.0541	0.33363	0.11998	0.05732	0.16578	0.04358	0.05098	0.05912
-0.11911	0.20753	-0.10268	0.22055	0.25707	0.05653	0.0722	0.11021	0.12783	0.28188	0.38636	-0.09741	0.05637	0.07926	0.41835	0.10704	0.04468	0.11333	0.02861	0.03867	0.01804

TS_OF_	SE_TO_TS	FOXO3_T	FOXO3_T	YROID_C	YROID_C	LHAES_A	LHAES_A	RACT_WL	PHA_RES	PHA_RES	ETA_RES	ETA_RES	AMMA_RE	AMMA_RE	EPATOCE
-0.12796	0.28186	0.11969	0.12034	0.0618	0.1107	0.17958	0.25523	0.30595	0.03422	0.19291	0.01036	0.19057	-0.03455	0.25363	0.14782
-0.10977	0.27882	0.17797	0.09501	-0.00256	0.07152	0.15832	0.20424	0.31489	0.02168	0.19603	-0.1224	0.19748	-0.0841	0.25153	0.10655
0.06015	0.29966	0.11946	0.08015	0.04131	0.08133	0.09267	0.26076	0.28614	0.04166	0.22598	-0.11127	0.21614	0.00524	0.26281	0.20298
-0.10291	0.29211	0.08855	0.0848	0.04123	0.13239	0.1388	0.32592	0.29614	0.08238	0.2534	-0.0481	0.22984	-0.0382	0.27949	0.13115
0.05801	0.29207	0.09876	0.09998	0.02148	0.08203	0.02778	0.27518	0.29616	0.02807	0.21875	0.03762	0.20334	-0.05955	0.25628	0.11982
0.03265	0.26521	0.17772	0.08365	0.03641	0.08848	0.08937	0.3047	0.30796	0.04097	0.19892	-0.05543	0.19471	-0.06967	0.2424	0.14022
-0.02871	0.26627	0.16292	0.06789	0.01663	0.11359	0.06358	0.28784	0.28862	0.01884	0.25669	0.02641	0.228	-0.04729	0.2746	0.16531
-0.06944	0.27374	0.0829	0.10676	0.06533	0.10907	0.15069	0.32645	0.29986	0.0369	0.2401	-0.01351	0.21746	-0.05395	0.27487	0.16815
-0.03286	0.28732	0.0933	0.10842	0.06537	0.11183	0.10288	0.31672	0.28245	0.06869	0.25352	-0.02869	0.23051	-0.0253	0.28404	0.18764
0.00816	0.27885	0.09739	0.11741	0.04503	0.10015	0.04961	0.32122	0.29365	0.01737	0.27637	-0.00661	0.24219	-0.0572	0.29214	0.13546
-0.04976	0.253	0.19208	0.13167	0.02639	0.0892	0.15307	0.21743	0.34097	0.04799	0.15995	-0.15019	0.16686	-0.00269	0.23616	0.19824
-0.04803	0.30449	0.17906	0.10144	0.04003	0.09955	0.09895	0.28475	0.31897	0.02414	0.23579	0.00028	0.22082	0.00498	0.27153	0.16184
0.07613	0.27353	0.16932	0.12913	0.02923	0.0765	0.06124	0.19798	0.32832	0.12608	0.15361	-0.19379	0.17194	-0.00866	0.22263	0.05782
0.01828	0.29572	0.11137	0.11104	0.02565	0.10347	0.02817	0.28325	0.31062	0.08047	0.18463	0.01695	0.17261	-0.01946	0.22593	0.1335
0.01116	0.28851	0.17137	0.11123	0.05455	0.08608	0.13226	0.26806	0.31485	0.05971	0.19258	-0.05319	0.18699	0.00871	0.24593	0.18194
-0.04055	0.27172	0.19318	0.10642	-0.01183	0.05715	0.11641	0.14921	0.3124	0.0801	0.17768	-0.00918	0.18237	0.01298	0.2367	0.1724
-0.02639	0.29636	0.10952	0.08292	0.03997	0.10978	0.13304	0.27713	0.30688	0.0313	0.17541	0.04856	0.1739	-0.05342	0.23349	0.14856
-0.03281	0.27414	0.09553	0.06812	0.05722	0.09808	0.13982	0.30221	0.28376	0.03778	0.25033	-0.02733	0.22772	-0.04422	0.27508	0.14367

CELLUL	CELLUL	CELLUL	LYMPHO	LYMPHO	1_TARGE	1_TARGE	ELOGEN	ELOGEN	ONIN_FU	NCER_BY	XPRESS	ER_MUT	ER_MUT	ER_MUT	P1_RES	P1_RESP
-0.02627	0.03454	0.20085	0.03817	0.10397	0.15711	0.05862	0.12174	0.17074	-0.2459	-0.08077	0.01345	-0.05804	-0.0343	-0.01768	0.24223	0.31691
-0.07192	0.02235	0.2018	0.0084	0.08672	0.15627	0.00501	0.11208	0.17383	-0.2031	-0.13738	0.02279	-0.15157	-0.03962	-0.05521	0.25666	0.31087
-0.05107	0.04376	0.18604	0.03985	0.11192	0.17249	-0.01294	0.13363	0.17822	-0.12806	-0.02633	0.0232	-0.0272	-0.07197	-0.01307	0.21759	0.28075
0.04854	0.0387	0.1884	0.05205	0.08636	0.15317	0.05837	0.18627	0.1611	-0.3368	0.00678	0.00949	-0.24605	-0.059	-0.06745	0.22464	0.35003
0.00924	0.04389	0.17816	0.06371	0.07951	0.13609	-0.04874	0.1601	0.17001	-0.20874	-0.01837	0.02576	-0.04857	0.00901	-0.01808	0.21599	0.33284
0.11295	0.03687	0.18583	0.05355	0.08856	0.15103	-0.0081	0.13617	0.17176	-0.13986	-0.10188	0.01646	-0.08314	-0.11065	-0.09165	0.2242	0.31549
0.08076	0.03782	0.19657	0.04402	0.09461	0.13694	0.03015	0.15954	0.16341	-0.25559	-0.10111	0.02923	-0.08803	-0.13264	-0.10948	0.23128	0.30734
0.06341	0.04151	0.17373	0.0604	0.08915	0.13912	-0.01688	0.20327	0.15951	-0.3146	0.00605	-0.01339	-0.11222	-0.12179	-0.06579	0.22053	0.34177
0.06436	0.02606	0.18203	0.07873	0.082	0.13684	-0.07227	0.20981	0.15958	-0.322	0.01214	0.00401	-0.08464	-0.15916	-0.05294	0.26426	0.33545
0.06019	0.0324	0.17956	0.08284	0.08439	0.12036	-0.06333	0.19844	0.15906	-0.23586	0.00085	0.00514	-0.082	-0.14067	-0.05396	0.21784	0.36042
-0.0231	0.0561	0.18367	0.01152	0.10055	0.16132	0.1048	0.10655	0.17942	-0.1492	-0.16249	0.04103	-0.19268	-0.00765	-0.04699	0.21263	0.26089
0.05175	0.07494	0.187	0.04121	0.10034	0.16543	0.08	0.12742	0.1692	-0.25718	-0.11953	0.05254	-0.05765	-0.10322	-0.05261	0.24129	0.30589
-0.04707	0.04012	0.17364	-0.01808	0.09028	0.1846	0.11274	0.08521	0.1734	-0.08755	-0.17222	0.05189	-0.09282	0.02963	-0.00902	0.22972	0.27411
0.01143	0.04455	0.1922	0.04348	0.08075	0.14586	0.0219	0.15077	0.17041	-0.2762	-0.04549	0.04514	-0.11323	-0.04581	-0.05883	0.24939	0.30904
0.02367	0.05989	0.1882	0.03025	0.09766	0.14864	0.05359	0.14231	0.17089	-0.17879	-0.0836	0.03435	-0.07906	-0.13081	-0.07327	0.23605	0.31982
-0.07813	0.06541	0.18696	-0.01443	0.11786	0.16703	0.10499	0.05873	0.17512	-0.2179	-0.18433	0.06566	-0.02587	0.04398	-0.04262	0.22926	0.24646
-0.00647	0.05462	0.18374	0.02966	0.08557	0.13528	0.05795	0.144	0.16635	-0.2947	-0.03321	0.04575	-0.17593	-0.00156	0.00301	0.24858	0.31732
0.00451	0.03111	0.17638	0.0606	0.0896	0.13334	0.00892	0.17082	0.16286	-0.26845	0.00478	0.01262	-0.22175	-0.06732	-0.08871	0.22154	0.34947

P1_RESP	CED_FRO	THLH_TA	THLH_TA	HIGHLY	OLIFERA	T_CANCE	T_CANCE	ST_CANC	ST_CANC	NSE_TO	NSE_TO	RYNGEA	RYNGEA	LL_CYCLE	ELAVL1
0.0897	0.35765	0.08163	0.18386	0.07031	0.13158	0.13116	0.08329	0.20172	0.00963	0.0287	0.08057	0.11578	0.02131	0.04647	0.03749
0.09588	0.38367	0.03685	0.18379	0.03813	0.11439	0.16147	0.06365	0.09918	-0.02739	0.03413	0.04988	0.13533	-0.00093	0.02368	0.07122
0.10571	0.35246	0.0621	0.18491	0.08453	0.1243	0.13027	0.09085	0.17066	0.01603	0.02569	0.10797	0.14042	0.00445	0.01599	0.03266
0.13051	0.3566	0.05385	0.17832	0.13276	0.14403	0.11728	0.06462	0.11284	-0.00999	0.03094	0.07235	0.1128	0.01481	0.12343	0.03356
0.13768	0.35408	0.04828	0.16619	0.12022	0.13039	0.09833	0.06258	0.07363	-0.04454	0.03149	0.08181	0.11246	-0.00353	0.09015	0.02479
0.10447	0.37708	0.05147	0.18419	0.19065	0.19632	0.14063	0.09704	0.12636	-0.01857	0.0292	0.06878	0.12979	-0.0104	0.02169	0.0515
0.13424	0.3655	0.06235	0.1727	0.2635	0.27415	0.07599	0.0818	0.08999	-0.01291	0.04116	0.08241	0.13107	-0.01037	0.03795	0.06479
0.14128	0.36401	0.06941	0.16001	0.16558	0.18254	0.13676	0.07899	0.08687	-0.01649	0.02322	0.08327	0.10243	0.0064	0.1263	0.04268
0.15478	0.35829	0.04863	0.1661	0.18076	0.20118	0.14401	0.10212	0.11121	-0.00823	0.03011	0.07663	0.11077	-0.00716	0.12341	0.02581
0.15649	0.35611	0.06541	0.15259	0.16217	0.15552	0.10286	0.08052	0.02589	-0.03515	0.02438	0.07034	0.1105	-0.01749	0.17197	0.01763
0.06499	0.38014	0.06203	0.17749	0.0983	0.13772	0.15279	0.015	0.20786	0.02108	0.03415	0.11029	0.13739	0.02443	-0.00323	0.10592
0.10253	0.38286	0.06249	0.17557	0.15858	0.18785	0.02986	0.10672	0.14754	0.04232	0.02505	0.08922	0.13269	0.01525	-0.02358	0.05072
0.03048	0.39031	0.05174	0.17258	0.08067	0.12849	0.0028	0.04097	0.135	0.0285	0.02866	0.08281	0.12032	0.05865	0.09997	0.08273
0.10738	0.35892	0.0461	0.16141	0.10132	0.13299	0.11917	0.06553	0.14219	0.01604	0.01427	0.10857	0.11363	0.02695	0.01892	0.04628
0.09277	0.37736	0.05164	0.14959	0.08432	0.11552	0.11799	0.06852	0.13769	-0.00019	0.01923	0.09398	0.12683	0.00159	0.02751	0.06608
0.04853	0.35256	0.0537	0.1695	0.03816	0.10477	0.02125	0.03583	0.20161	-0.01387	0.00826	0.07871	0.13796	0.02251	0.04878	0.01906
0.11839	0.34387	0.06287	0.17987	0.10459	0.13152	0.10191	0.08301	0.17274	0.02967	0.02523	0.08198	0.11145	0.03291	0.06404	0.05645
0.15108	0.34371	0.0641	0.16771	0.13743	0.19178	0.11202	0.04475	0.12438	-0.02944	0.02584	0.06675	0.11272	-0.01496	0.0757	0.02947

US_INFE	US_INFE	US_INFE	US_INFE	US_INFE	US_INFE	US_INFE	US_INFE	HOXA9_TA	HOXA9_TA	Y_GAB2_T	BMI1_TA	BMI1_TA	PRDM5_TA	OF_AML1	OF_AML1	_STROM
0.18492	0.19138	0.20149	0.14162	0.17297	0.20307	0.16202	0.17638	0.11228	0.19987	0.04318	0.05089	0.05072	-0.1009	0.01767	0.16008	0.0592
0.20784	0.22233	0.24179	0.11719	0.20129	0.22461	0.18292	0.2226	0.16702	0.22098	0.03273	0.04384	0.06231	-0.05387	-0.02579	0.02701	0.0297
0.19426	0.1819	0.18601	0.13594	0.13927	0.24393	0.11644	0.2353	0.10649	0.20527	0.05845	0.0633	0.06814	-0.17106	0.06584	0.09753	0.04423
0.18013	0.20712	0.1812	0.15254	0.15484	0.21167	0.14661	0.17708	0.15821	0.22299	0.10225	0.06622	0.05964	-0.09302	0.09807	0.14908	0.04612
0.18479	0.17918	0.17926	0.12763	0.14984	0.22389	0.14115	0.19822	0.12177	0.20938	-0.00466	0.05339	0.05268	-0.0969	0.12129	0.1864	0.00107
0.14298	0.1573	0.1477	0.09013	0.10987	0.16977	0.10209	0.16735	0.09544	0.199	0.06424	0.01867	0.0512	-0.0905	0.08205	0.09809	-0.00407
0.16986	0.16514	0.15257	0.0927	0.12717	0.13848	0.11213	0.16799	0.09941	0.19932	0.12882	0.04661	0.06628	-0.10909	0.14502	0.07083	0.01466
0.16603	0.18977	0.16544	0.1403	0.1381	0.18603	0.12384	0.16306	0.14724	0.22826	0.08579	0.0665	0.05466	-0.15762	0.07458	0.20044	0.0091
0.16309	0.22109	0.16832	0.14504	0.15609	0.19389	0.14254	0.17847	0.14245	0.20114	0.11831	0.07522	0.05368	-0.11695	0.13556	0.18122	0.01394
0.16863	0.23622	0.17474	0.15146	0.17023	0.18462	0.16135	0.16755	0.15385	0.2254	0.06995	0.06891	0.04999	-0.11744	0.11606	0.20814	-0.01399
0.15609	0.23314	0.16952	0.22912	0.15883	0.24689	0.14977	0.22043	0.02407	0.2136	-0.01154	0.05847	0.06771	-0.11482	0.02879	0.13987	0.03701
0.17244	0.16906	0.16587	0.13592	0.125	0.21804	0.11628	0.18684	0.07043	0.1702	0.11441	0.04719	0.04806	-0.07439	0.11472	0.08163	0.03974
0.19277	0.20228	0.23688	0.04489	0.21061	0.15022	0.2046	0.15067	0.03242	0.21089	-0.02379	0.04701	0.06288	-0.12764	0.02351	-0.04395	0.04501
0.17946	0.1236	0.14079	0.11574	0.12735	0.0965	0.12081	0.09675	0.13007	0.21735	0.06731	0.05925	0.06111	-0.17043	0.04364	0.15913	0.02413
0.18785	0.2111	0.19509	0.16791	0.16999	0.22623	0.15428	0.21819	0.0839	0.21713	0.09197	0.04926	0.05734	-0.07513	-0.04106	0.16783	0.03171
0.1679	0.19162	0.19784	0.07313	0.16489	0.16989	0.14791	0.18614	0.02865	0.18486	0.01964	0.05363	0.06616	-0.13438	-0.02967	-0.07099	0.08331
0.18645	0.20685	0.19136	0.18877	0.18456	0.17399	0.1764	0.16028	0.1286	0.21466	0.04339	0.04844	0.06247	-0.10857	0.08103	0.15165	0.05378
0.19564	0.2528	0.19193	0.21011	0.18961	0.21193	0.1846	0.18751	0.12106	0.21217	0.05822	0.07936	0.05457	-0.13739	0.05638	0.14153	0.0281

D_STROM	KIN_CAR	KIN_CAR	DIOL_RE	DIOL_RE	DIOL_RE	DIOL_RE	OPTOSIS	RGETS	ODF_PAX3	OF_PAX3	_CYCLE
-0.01788	0.0605	0.08743	0.02248	0.00818	-0.0198	0.07254	-0.03479	0.01189	-0.03385	0.05921	0.12998
-0.04388	0.04678	0.108	-0.00656	0.01028	-0.09973	0.07357	-0.00782	0.04828	-0.06533	0.0778	0.02362
-0.03441	0.03427	0.07995	0.00151	0.03629	-0.0553	0.08689	-0.0031	0.08227	-0.11067	0.09714	0.19524
-0.0158	0.042	0.05779	0.004	0.01275	-0.03879	0.07386	-0.01488	0.03557	0.01535	0.08982	0.13286
-0.03243	0.0244	0.07806	-0.03141	-0.00997	-0.08441	0.03226	0.0334	-0.02564	-0.02064	0.07849	0.10681
-0.02014	0.05498	0.05002	-0.03426	0.0203	-0.09842	0.07283	-0.00969	0.04479	-0.11742	0.09048	0.17302
0.01484	0.03205	0.05941	-0.02072	0.03641	-0.06918	0.10674	0.00846	0.0263	-0.03906	0.09236	0.13871
-0.0154	0.04039	0.06436	-0.00327	-0.02392	-0.03846	0.02886	0.00696	0.02943	0.0393	0.10281	-0.02568
0.01269	0.04325	0.03679	0.00332	-0.01905	-0.01713	0.03008	0.02266	0.09666	-0.00602	0.12584	0.0316
-0.01512	0.02355	0.06209	-0.02729	-0.00937	-0.07011	0.02664	0.00348	0.00748	-0.02907	0.09046	0.01832
-0.03935	0.04423	0.04881	0.0052	0.06065	-0.05357	0.11239	0.04873	0.01504	0.00219	0.07429	0.23293
-0.02068	0.05024	0.07765	0.01697	0.01008	-0.00555	0.08252	0.02459	0.03787	-0.04771	0.06511	0.16437
-0.01644	0.0466	0.09294	0.0078	0.02997	-0.03804	0.09177	0.00063	0.03502	-0.04488	0.07746	0.16889
-0.01801	0.04372	0.07324	-0.00376	-0.04091	-0.09193	0.05307	0.0081	0.02188	0.01602	0.07618	-0.00478
-0.03769	0.05058	0.06503	-0.00345	0.02572	-0.07515	0.08836	-0.02191	0.07659	0.02109	0.07842	0.16682
-0.05177	0.05488	0.05754	0.00605	0.04312	-0.05574	0.11069	0.04467	0.10075	-0.06361	0.08754	0.22349
-0.02289	0.04215	0.05914	-0.00486	-0.03691	-0.02539	0.05363	-0.01524	0.01773	-0.03334	0.06996	-0.09998
0.00675	0.04297	0.08168	0.00084	-0.01834	-0.02991	0.05733	0.03561	0.08511	0.00107	0.11042	0.03304

ANEUPL	ANEUPL	ICF_SYN	ICF_SYN	N_SIGNA	MYC_TAR	MYC_TA	AND_HIF	AND_HIF	HIF1A_TA	HIF1A_TA	HIF2A_TA	POXIA_B	YPOXIA_B
-0.16965	0.11768	0.12227	0.13066	0.08099	0.16508	-0.03485	0.06886	0.10166	0.06841	0.02922	-0.38005	0.10408	0.06027
-0.21888	0.09612	0.08027	0.07542	0.14208	0.16836	-0.03186	0.01613	0.11065	0.0126	0.05169	-0.30035	0.11741	0.00397
-0.10839	0.06455	0.16664	0.08267	0.17467	0.17512	-0.05674	0.01659	0.12882	0.00295	0.05222	-0.18388	0.10268	0.00632
-0.07952	0.10899	0.22468	0.14791	0.19464	0.17553	0.0185	0.07142	0.09195	0.05863	0.03765	-0.17728	0.12131	0.03925
-0.30083	0.11606	0.0886	0.04493	0.12388	0.16422	0.00961	0.02435	0.10757	0.00457	0.01971	-0.29047	0.10993	0.0046
-0.1056	0.07645	0.10018	0.09919	0.13265	0.17258	0.04618	0.06482	0.11846	0.07696	0.05426	-0.21082	0.13394	0.05216
-0.10571	0.04103	0.11103	0.05742	0.23347	0.14746	0.0287	0.03216	0.14509	0.03098	0.06531	-0.0723	0.13237	0.04735
-0.02052	0.10498	0.19812	0.06679	0.17085	0.19305	0.05098	0.06021	0.08007	0.05141	0.02747	-0.0018	0.09994	0.02771
-0.08412	0.10468	0.16324	0.09327	0.20109	0.17761	0.02669	0.04938	0.08576	0.04907	0.02315	-0.14279	0.11238	0.0263
-0.11717	0.09814	0.14368	-0.00793	0.25331	0.17788	0.03906	0.02398	0.08724	0.03193	0.01839	-0.20291	0.1193	0.00992
-0.05392	0.09193	0.0328	0.02283	0.05585	0.17545	-0.03332	0.08904	0.13741	0.07434	0.08554	-0.29466	0.13693	0.08304
-0.03474	0.08191	0.04735	0.1513	0.17084	0.1827	-0.02498	0.0443	0.11155	0.04722	0.0584	-0.16414	0.13438	0.067
-0.19994	0.0326	-0.02973	0.09897	0.09612	0.14738	-0.08628	0.05511	0.11075	0.03666	0.03123	-0.35659	0.11481	0.06244
-0.10186	0.08194	0.1261	0.06372	0.10859	0.17426	0.01653	0.03841	0.1386	0.02893	0.0677	-0.20777	0.13323	0.03292
-0.03684	0.13681	0.11805	0.09687	0.14142	0.19576	0.03131	0.06109	0.16962	0.06307	0.08072	-0.19079	0.14857	0.04919
-0.16749	0.05506	0.01911	0.07923	0.12117	0.15895	-0.05464	0.06383	0.17963	0.06822	0.09469	-0.37087	0.13016	0.052
-0.08585	0.1066	0.17942	0.10914	0.07743	0.18823	0.00954	0.03661	0.09957	0.02933	0.0323	-0.32831	0.08567	0.02092
-0.0948	0.11543	0.14623	0.03072	0.22181	0.18883	0.03951	0.01787	0.09151	0.01024	0.03367	-0.26133	0.12389	0.01746

SE_HYPO	SE_HYPO	NCER_PR	NCER_PR	PONSE_E	PONSE_E	NSE_KE	NSE_KE	T_CE_HS	ERT_HS	ERT_LS	CT_PROG	LL_WITH	LL_WITH
0.11419	0.04483	-0.00557	0.05492	0.05999	0.14955	0.11862	0.20649	-0.07115	0.04374	0.01271	0.09458	0.17481	0.15138
0.12422	-1.8E-05	-0.02803	0.0004	0.04141	0.1527	0.1237	0.20599	-0.07716	0.019	0.01621	0.11578	0.16754	0.15736
0.12741	-0.01473	-0.00618	-0.00182	0.04027	0.14573	0.13507	0.20497	-0.03583	0.06609	0.0171	0.11586	0.12221	0.16561
0.11834	0.04454	-0.00972	0.04924	0.05505	0.13395	0.10689	0.20824	-0.19135	0.01896	0.02154	0.07392	0.14396	0.18552
0.10916	-0.00802	0.00538	0.00617	0.04052	0.13608	0.10565	0.19358	-0.08878	0.05367	-0.03071	0.09917	0.16932	0.16508
0.12288	0.03164	0.02791	0.01719	0.05547	0.13896	0.11692	0.19662	-0.11382	0.05587	0.00737	0.11278	0.17172	0.16483
0.12614	0.01898	-0.02398	0.01741	0.04429	0.14604	0.1229	0.1949	-0.15437	0.05955	-0.03589	0.09044	0.12045	0.17485
0.11605	0.03118	0.04014	0.02831	0.04737	0.13489	0.10008	0.19638	-0.18525	0.02064	-0.02937	0.07942	0.13534	0.16763
0.10533	0.02523	-0.00875	0.04435	0.05414	0.13142	0.0956	0.19759	-0.08448	0.06028	-0.02656	0.06359	0.1494	0.16361
0.12342	0.00588	0.0213	0.0139	0.04209	0.12706	0.09545	0.19456	-0.21953	0.03263	-0.03805	0.06892	0.16186	0.1761
0.13489	0.06538	0.07871	0.02464	0.04115	0.14563	0.1279	0.20022	-0.1236	0.04929	-0.04009	0.10707	0.12218	0.14811
0.13014	0.03981	0.01074	0.06355	0.05885	0.1484	0.1231	0.206	-0.00591	0.08247	-0.02747	0.09719	0.14785	0.14989
0.12826	0.02739	0.01778	-0.00743	0.05753	0.14818	0.12949	0.21054	0.01297	0.08849	-0.03946	0.10454	0.1371	0.1637
0.126	0.01458	0.07059	0.01845	0.04591	0.15471	0.11747	0.2043	-0.15276	0.04493	-0.01804	0.07134	0.15575	0.17464
0.13718	0.03631	0.01944	0.04244	0.0562	0.14979	0.11235	0.20467	-0.0709	0.05123	-0.01622	0.10206	0.147	0.13164
0.14487	0.03316	-0.00355	-0.02867	0.04762	0.14363	0.11962	0.20826	-0.05609	0.04988	2E-06	0.12007	0.09637	0.14321
0.11738	0.00821	0.00487	0.05522	0.04528	0.14143	0.11409	0.21083	-0.02271	0.05969	0.02418	0.07655	0.15318	0.19258
0.12165	-0.00035	-0.00355	0.03331	0.04402	0.13831	0.10941	0.20043	-0.21511	0.01545	-0.0346	0.07868	0.16584	0.17233

VH_REA	VH_REA	RS_WITH	N_HYPO	IN_HYPO	NCER_A	NCER_A	NCER_B	ST_CANC	ST_CANC	ST_CANC	ST_CANC	ST_CANC
0.17467	0.16935	0.19174	0.14112	0.26478	0.10385	0.12326	0.08752	-0.01058	0.10725	0.16489	-0.29207	-0.13716
0.16171	0.16342	0.17923	0.08041	0.2185	0.09335	0.10603	0.05858	-0.0152	0.01647	0.12956	-0.28872	-0.07438
0.13229	0.16991	0.1997	0.04293	0.12232	0.10144	0.10875	0.09496	0.11454	0.0998	0.16169	-0.24591	-0.11632
0.13959	0.16368	0.21289	0.08932	0.14647	0.1018	0.12382	0.06766	0.13152	-0.02599	0.19505	-0.10246	-0.13689
0.15918	0.17902	0.18228	0.04592	0.11298	0.07477	0.10785	0.04824	0.15458	-0.05229	0.16929	-0.19331	-0.1653
0.16888	0.17486	0.18557	0.07451	0.20232	0.09291	0.11427	0.06969	0.14147	0.04911	0.12999	-0.06423	-0.10963
0.13787	0.17318	0.19949	0.09389	0.18856	0.08963	0.11907	0.0615	0.21163	0.14382	0.09786	0.07482	-0.03683
0.13551	0.17701	0.19261	0.07652	0.0732	0.09063	0.11583	0.06722	0.1215	-0.06034	0.19969	-0.05201	-0.14738
0.15157	0.19201	0.21506	0.04081	0.07502	0.08586	0.11198	0.07127	0.22028	-0.01515	0.17052	-0.07472	-0.16965
0.13038	0.17179	0.20568	0.06348	0.13132	0.08715	0.11136	0.05606	0.28146	0.02798	0.19115	-0.12098	-0.14683
0.16323	0.14095	0.20516	0.1199	0.24852	0.10305	0.12667	0.07386	-0.07611	0.07514	0.13958	-0.22194	-0.21876
0.15977	0.15987	0.21107	0.09685	0.18127	0.11061	0.12214	0.07479	0.14703	0.03721	0.13831	-0.07018	-0.09672
0.15703	0.13294	0.19418	0.14048	0.30144	0.09705	0.12824	0.0574	-0.05689	0.03543	0.10501	-0.1662	-0.07628
0.14023	0.16325	0.20817	0.07163	0.1315	0.09935	0.1195	0.07827	0.07333	0.06657	0.15743	-0.14608	-0.13476
0.1805	0.1479	0.20708	0.07048	0.14575	0.10073	0.12873	0.07219	0.09221	0.10144	0.12731	-0.1241	-0.08809
0.14742	0.15409	0.20576	0.12138	0.25139	0.08645	0.11685	0.07155	-0.07732	0.103	0.16173	-0.28045	-0.07379
0.16054	0.18383	0.19385	0.0885	0.18593	0.09995	0.12044	0.0834	0.02037	0.02299	0.172	-0.15402	-0.07552
0.15792	0.18779	0.18483	0.05581	0.07941	0.09343	0.11727	0.068	0.21317	0.0433	0.21828	-0.08503	-0.19461

ST_CANC	ST_CANC	ST_CANC	EZ_BOUN	HOX11_N	LYL1_N	D_TAL1_N	WITH_ML	WITH_ML	NSE_TO_
0.03832	0.16302	-0.16319	0.18906	0.11629	0.27356	0.16201	0.22826	0.17563	0.03364
-0.07241	0.15251	-0.1015	0.1898	0.1112	0.24594	0.05628	0.21943	0.18342	0.14568
-0.06883	0.21723	-0.2502	0.19129	0.15095	0.27869	0.18073	0.24163	0.17149	0.18108
-0.04009	0.10387	-0.35243	0.19225	0.11386	0.32111	0.1175	0.24158	0.19333	0.1913
-0.08595	0.08077	-0.22305	0.18666	0.10526	0.31009	0.1689	0.24687	0.19976	0.08702
0.00513	0.124	-0.24643	0.18988	0.15619	0.24817	0.20554	0.24036	0.17349	0.19042
0.01505	0.11295	-0.26359	0.19986	0.07894	0.27485	0.22759	0.23578	0.17052	0.09759
-0.07208	0.12385	-0.316	0.18399	0.04126	0.34476	0.08466	0.23221	0.18835	0.13863
-0.02844	0.1488	-0.32296	0.18563	0.04608	0.3344	0.10269	0.22864	0.20388	0.19958
-0.07441	0.12862	-0.34431	0.19463	0.06707	0.33423	0.21213	0.24483	0.2069	0.16364
-0.07151	0.08034	-0.09897	0.18553	0.15594	0.16917	0.20393	0.24395	0.1591	0.30298
0.05832	0.19883	-0.22365	0.18953	0.1145	0.25921	0.18898	0.23618	0.17299	0.17368
0.00371	0.18612	-0.03748	0.18522	0.17543	0.06361	0.12292	0.23627	0.14279	0.28748
0.01267	0.11365	-0.25768	0.18768	0.0096	0.28593	0.13073	0.22316	0.18596	0.1566
-0.06112	0.15575	-0.32894	0.18868	0.11243	0.27137	0.17921	0.2265	0.17278	0.1765
0.01186	0.14262	-0.06493	0.18872	0.15427	0.13483	0.16094	0.22334	0.14318	-0.02184
-0.07072	0.16219	-0.22453	0.19023	-0.00251	0.29609	0.1454	0.22727	0.16205	0.12454
-0.05135	0.10086	-0.30579	0.18736	0.07204	0.32641	0.14385	0.23859	0.20332	0.05038

NNB1_TAR	THYLATIC	THYLATIC	THYLATIC	THYLATIC	THYLATIC	THYLATIC	THYLATIC	THYLATIC	THYLATIC	THYLATIC	THYLATIC	THYLATIC	THYLATIC	ANCER_S	CANCER	_CANCER	CANCER	T_CANCE	_CTNNB	PATHWAY
0.08349	0.0472	0.01433	0.00769	-0.00483	0.05293	0.02931	-0.17694	0.01824	0.15278	-0.08192	0.19115	0.00693	0.01814	0.03883	-0.16954	-0.02583	-0.10692	-0.11843	0.03251	-0.09422
0.07158	0.03618	0.01754	-0.01366	0.05444	0.0455	0.02964	-0.09087	0.01628	0.13232	0.02178	0.2091	0.00982	0.04762	-0.07493	-0.16341	-0.03156	-0.16353	-0.06085	0.01811	0.03112
0.08946	0.04423	0.0233	0.06242	0.00947	0.06602	0.02878	-0.18222	0.04325	0.11036	0.11011	0.19514	0.03034	0.03875	-0.01911	-0.15481	-0.01994	-0.08136	-0.0261	0.01958	-0.06053
0.10842	0.09223	0.00382	0.03174	-0.0399	0.075	0.01453	-0.18433	0.02271	0.15976	0.03771	0.22654	0.00667	0.01257	0.06378	-0.10989	-0.06237	0.0648	-0.16119	-0.00066	-0.09495
0.08893	0.08972	-0.00278	0.12878	0.00349	0.07659	0.02511	-0.10035	0.02588	0.16965	0.04844	0.2371	0.0219	0.03121	0.11661	-0.0943	-0.11222	0.08074	-0.1246	-0.02627	-0.07813
0.08612	0.0722	0.03975	0.12201	0.05875	0.04229	0.03335	-0.2172	0.05228	0.12487	0.02898	0.20769	0.03039	0.05649	0.08024	-0.15053	-0.06122	-0.03169	0.00786	-0.03983	-0.10586
0.08903	0.05157	0.01148	0.11993	0.03887	0.09472	0.01128	-0.21451	0.03709	0.11037	0.09629	0.21787	-0.00387	0.04352	0.04109	-0.14954	-0.10404	0.00713	-0.06416	0.01479	0.00749
0.11388	0.08283	-0.00286	0.04864	-0.02416	0.07569	0.01549	-0.09863	0.03863	0.15875	0.01861	0.20449	0.01305	0.02025	0.08389	-0.08055	-0.07077	0.08964	-0.24322	-0.00993	-0.12741
0.10986	0.08599	-0.01683	0.06153	-0.00194	0.08948	0.00358	-0.17624	0.03065	0.1702	0.0448	0.22843	-0.00553	0.00835	0.14721	-0.11024	-0.07891	0.11012	-0.21527	-0.00607	-0.11486
0.1028	0.10564	-0.03706	0.15233	-0.01582	0.07088	-0.00077	-0.13607	0.02911	0.20262	0.07163	0.26723	-0.01492	0.01221	0.19216	-0.08441	-0.09205	0.11681	-0.20237	-0.00191	-0.12439
0.06788	0.07239	0.03339	0.0394	0.03782	0.08996	0.03277	-0.22806	0.03208	0.12317	0.13685	0.14488	0.05216	0.03462	-0.10589	-0.19778	-0.05725	-0.1691	-0.04015	0.0404	-0.00195
0.08856	0.07423	0.0226	0.03718	-0.01053	0.02287	0.04003	-0.26526	0.02696	0.12491	0.02762	0.16906	0.02985	0.0277	-0.00859	-0.10883	0.00273	-0.10103	-0.17224	0.00863	-0.02801
0.07261	0.02669	0.04975	-0.11344	0.0392	0.09275	0.04504	-0.1297	0.08469	0.0243	0.03656	0.16965	0.06589	0.06281	-0.13986	-0.20265	-0.01051	-0.30247	0.01776	0.03002	0.00193
0.08966	0.08306	0.0177	0.02922	-0.00189	0.11874	0.02057	-0.22962	0.02889	0.14349	0.0389	0.1798	0.01368	0.03025	-0.0031	-0.10044	-0.04035	-0.03523	-0.24019	0.03368	0.00189
0.07817	0.06329	0.03893	0.02605	0.04957	0.02804	0.04274	-0.278	0.06423	0.11079	0.03076	0.1525	0.02837	0.05098	-0.04174	-0.12972	-0.01208	-0.10307	-0.16071	0.02277	-0.03232
0.05955	0.03944	0.03419	-0.04164	0.00882	0.08918	0.03548	-0.20226	0.03457	0.06286	0.04227	0.14109	0.03553	0.03698	-0.17728	-0.21354	0.02729	-0.36584	0.02278	0.06082	0.02981
0.08944	0.06836	0.02696	-0.00324	-0.00617	0.07472	0.01626	-0.12658	0.02429	0.13037	0.00021	0.21567	0.00085	0.02691	0.01948	-0.17792	-0.03079	-0.02109	-0.23375	-0.01564	-0.03132
0.10582	0.09949	0.02424	0.03609	-0.02739	0.07306	0.02525	-0.11616	0.03971	0.18152	0.03898	0.23472	0.02275	0.01942	0.1027	-0.10492	-0.06188	0.04946	-0.18671	-0.00268	0.01912

N_PROLI	P53_TARG	_DREAM_	G1_S_CE	G2_M_CE	Y_TRANS	Y_TRANS	_TRANSP	_TRANSP	_TRANSP	_TRANSP	TRANSPL	TRANSPL	UMAN_NE	EX_BASA	EX_BASA	ALL_THE
0.0452	0.00145	0.11674	0.03985	0.08569	0.13319	0.2036	0.17438	0.16011	0.05691	0.19878	0.15783	0.16242	-0.03904	0.03184	-0.06399	-0.05105
0.02047	-0.02704	0.14969	0.04135	0.10678	0.16496	0.19306	0.15333	0.10739	0.04243	0.2015	0.1361	0.16331	-0.12841	0.06209	-0.12484	-0.00879
0.04773	0.00044	0.14918	0.06957	0.09469	0.17311	0.19937	0.16966	0.17383	0.07067	0.20551	0.15839	0.15778	-0.21364	0.0668	-0.09726	0.00726
0.03914	0.01002	0.11152	0.04304	0.0648	0.14423	0.20304	0.15632	0.24567	0.08336	0.21291	0.14146	0.146	-0.0862	0.07423	-0.04795	0.02448
0.06163	-0.01571	0.12341	0.03067	0.06658	0.16092	0.19512	0.14281	0.23538	0.08287	0.20944	0.1497	0.15846	0.12779	0.01471	-0.05555	-0.02251
0.03004	-0.01282	0.1387	0.04485	0.08541	0.1397	0.19491	0.15356	0.14551	0.06537	0.19657	0.15965	0.15198	0.02343	0.09461	-0.04589	0.08994
0.05711	-0.01335	0.14057	0.0235	0.08342	0.15118	0.18509	0.15628	0.2077	0.06038	0.20823	0.16226	0.12873	0.01308	0.09935	-0.04147	0.07059
0.04899	-0.00257	0.08954	0.00731	0.05824	0.16921	0.20501	0.15993	0.25857	0.09053	0.21691	0.13609	0.14773	-0.08212	0.06202	-0.06703	0.02391
0.06909	-0.0182	0.09565	0.02571	0.05848	0.16693	0.20609	0.14802	0.25529	0.10807	0.21137	0.14195	0.1478	0.00045	0.05654	-0.04062	0.00341
0.0765	-0.00988	0.11012	0.01057	0.06396	0.16364	0.2008	0.1434	0.2828	0.09588	0.21002	0.15618	0.14066	0.11289	0.04947	-0.02612	0.0466
-0.00631	0.01407	0.14711	0.07487	0.10792	0.1801	0.19867	0.17898	0.08508	0.02732	0.20228	0.15156	0.14151	-0.36031	0.07991	-0.10307	-0.01702
0.01853	0.01558	0.13257	0.07227	0.06467	0.14569	0.20543	0.16856	0.15908	0.0225	0.20081	0.16365	0.15	-0.07438	0.04132	-0.02764	0.01827
0.0216	0.00567	0.15086	0.06409	0.09686	0.15295	0.18696	0.16364	-0.00911	0.01268	0.19853	0.14849	0.14002	-0.08369	0.0575	-0.09644	0.04937
0.03577	0.00088	0.11998	0.02321	0.0931	0.1558	0.20429	0.16859	0.20814	0.07972	0.20502	0.13636	0.13544	-0.08233	0.06647	-0.09667	-0.00272
0.01914	0.0015	0.13332	0.05094	0.08612	0.13709	0.20394	0.17482	0.17167	0.04763	0.20156	0.13891	0.13974	-0.11184	0.06369	-0.09389	-0.01464
0.0222	0.01949	0.15426	0.06146	0.09011	0.17316	0.18838	0.17515	0.00636	0.01167	0.19618	0.13686	0.13217	-0.04557	0.02037	-0.11774	0.06433
0.04771	0.00209	0.11216	-0.00442	0.07274	0.13623	0.19841	0.16743	0.19586	0.0866	0.21158	0.1349	0.1475	-0.1869	0.04682	-0.11077	0.02005
0.05745	-0.00222	0.10627	0.00694	0.05957	0.15778	0.20392	0.1598	0.21986	0.09304	0.20853	0.14324	0.16359	-0.07017	0.04875	-0.05741	0.00456

ALL_THE	LAR_THY	LAR_THY	Y_THYR	Y_THYRO	OR_UNC	OR_UNC	EGRATED	EGRATED	ODRANK_	ODRANK_	AN_TGFB	AN_TGFB	ER_PHF8	ER_PHF8	DMIA_TA
0.27442	0.15758	0.06084	-0.06728	0.20039	0.07262	0.12808	0.09117	-0.03397	0.08788	0.02354	0.09344	0.019	0.05524	0.02186	0.05175
0.27302	0.11776	0.08396	-0.0487	0.17679	0.06969	0.07867	0.03213	-0.03051	0.05502	0.02169	0.04128	0.01819	0.04344	0.03772	0.03932
0.26886	0.17257	0.0822	-0.0267	0.18869	0.08689	0.15158	0.04081	-0.00982	0.07925	0.01792	0.05615	0.02055	0.04756	0.05006	0.0492
0.28439	0.19963	0.0573	-0.01022	0.20632	0.12591	0.09676	0.04171	0.00145	0.01131	0.03871	0.03113	0.03326	0.04996	0.03357	0.04449
0.26913	0.16776	0.04986	-0.05493	0.18989	0.05021	0.15109	0.03403	-0.08228	0.03878	-0.0018	0.03759	-0.01036	0.04305	0.02775	0.03286
0.27129	0.15449	0.08639	-0.11744	0.22682	0.08044	0.1635	0.02406	-0.00161	0.02887	0.02345	0.03485	0.02622	0.03323	0.02639	0.03859
0.26774	0.16586	0.06576	-0.03963	0.21022	0.10474	0.15012	0.04172	0.03578	0.03548	0.04736	0.03338	0.05316	0.04341	0.0331	0.05724
0.2802	0.19394	0.05449	-0.0199	0.17912	0.06864	0.097	0.01621	0.00027	-0.01562	0.04528	0.00265	0.03961	0.0468	0.03271	0.03582
0.25963	0.18584	0.05182	-0.00379	0.19311	0.1119	0.11926	-0.01504	0.0475	-0.04096	0.06516	-0.02282	0.06698	0.04093	0.01579	0.03203
0.27758	0.18084	0.06899	-0.03084	0.16926	0.09252	0.11159	0.0139	-0.0403	-0.01436	0.00627	0.01123	0.00441	0.03314	0.0251	0.03902
0.2781	0.08546	0.07886	-0.10774	0.16153	-0.04366	0.15904	0.08131	-0.0232	0.07264	0.02673	0.08666	0.02032	0.0616	0.05464	0.04266
0.2767	0.15489	0.07003	-0.08246	0.20906	0.06731	0.15649	0.10776	0.02489	0.06255	0.05674	0.08289	0.05665	0.04948	0.03125	0.04223
0.28973	0.0838	0.07488	-0.0638	0.16433	-0.0116	0.09897	0.08604	-0.10891	0.09197	-0.01654	0.08333	-0.01708	0.06488	0.04812	0.06011
0.28006	0.18902	0.05651	-0.01712	0.19456	0.07014	0.12394	0.04126	-0.03363	0.03578	0.01866	0.03514	0.0187	0.05068	0.02479	0.05342
0.2829	0.14078	0.06945	-0.05867	0.17969	0.05639	0.1189	0.08313	0.02065	0.06257	0.048	0.08277	0.04454	0.04931	0.02433	0.04016
0.28498	0.08888	0.08515	-0.09289	0.15213	-0.04236	0.11362	0.0809	-0.07011	0.08061	-0.00141	0.0805	-0.00249	0.05666	0.05968	0.07561
0.28292	0.15774	0.08411	-0.04385	0.20807	0.1072	0.14165	0.04773	-0.0429	0.05481	0.01542	0.05623	0.01192	0.05069	0.01978	0.07338
0.27621	0.19019	0.06608	-0.00694	0.16265	0.1067	0.12362	-0.00781	-0.01452	0.00607	0.02373	-0.00368	0.02118	0.05382	0.04598	0.04337

DM1A_TA	ANT_MA	ANT_MA	_DEVEL	_DEVEL	AR_DEVE	R_DEVE	R_DEVEL	NSE_TO	NSE_TO	O_SERM	O_SERM	XIFEN_R	XIFEN_R	K_PRKC	MMORTAL
-0.02415	0.03035	0.08011	0.02901	0.12405	0.15821	0.11794	0.25065	0.16219	-0.10487	0.04829	0.06192	0.14667	0.10977	-0.02718	-0.07241
-0.04169	0.02265	0.06937	0.07226	0.0617	0.16776	0.19417	0.26909	0.05008	-0.09379	0.00432	0.06289	0.12596	0.06552	0.00569	-0.17067
-0.00947	0.02564	0.09368	-0.02638	0.04136	0.16397	0.14429	0.33022	0.09189	-0.13223	0.10124	0.03163	0.17718	0.08222	0.10777	-0.09619
0.00049	0.03264	0.14262	0.07683	0.04221	0.14353	0.12065	0.33087	0.12278	-0.12739	0.07229	0.06546	0.15751	0.10532	0.08827	-0.12167
-0.00048	0.02745	0.07726	-0.0244	0.02526	0.15024	0.12036	0.21034	0.09603	-0.1583	0.0345	0.03204	0.18021	0.09881	0.17073	-0.10402
0.01583	0.02619	0.07784	0.18013	0.05821	0.15933	0.20302	0.34162	0.05099	-0.0915	0.08476	0.00164	0.12334	0.08141	0.13965	-0.09566
0.01644	0.0339	0.12648	0.14852	0.14752	0.14716	0.14765	0.29711	0.08416	-0.13239	0.05874	0.0116	0.14556	0.07201	-0.06164	-0.08381
0.01065	0.03534	0.1495	0.07332	0.00271	0.12163	0.09867	0.30856	0.07999	-0.09408	-0.06515	0.04244	0.19585	0.10451	0.14972	-0.06768
0.01836	0.04942	0.14791	0.12287	-0.03507	0.12585	0.12915	0.35506	0.07285	-0.13728	-0.02469	0.0793	0.1806	0.08743	0.16195	-0.07411
0.01298	0.04138	0.13993	0.078	-0.00133	0.12493	0.12866	0.29446	0.06539	-0.08154	-0.03041	0.07628	0.20382	0.10245	0.12476	-0.08548
-0.00854	0.01209	0.06935	0.13158	0.10977	0.16439	0.16294	0.27139	0.12493	-0.05973	0.1183	0.11956	0.16979	0.06648	-0.13151	-0.08363
-0.0068	0.03849	0.08622	0.13126	0.11382	0.16736	0.14156	0.34522	0.13526	-0.1648	0.07957	0.06432	0.13847	0.08666	-0.0964	-0.03848
-0.04563	0.01189	0.05679	0.0686	0.12998	0.17697	0.17983	0.18236	0.16867	-0.14818	0.09181	0.01879	0.04485	0.08509	-0.0679	-0.11662
0.01081	0.02675	0.08608	0.0447	0.04437	0.14025	0.1087	0.32002	0.13891	-0.20861	-0.06614	0.06262	0.23115	0.10975	0.19822	-0.10596
-0.01409	0.01484	0.08735	0.09809	0.04455	0.14235	0.17636	0.30159	0.10274	-0.166	0.08095	0.11202	0.25694	0.08876	0.08121	-0.09213
-0.03383	0.0091	0.05644	0.01464	0.12257	0.17539	0.21699	0.23813	0.13176	-0.20762	0.13386	0.12322	0.13273	0.10597	-0.10835	-0.13334
-0.01275	0.0147	0.08461	0.0188	0.05918	0.12873	0.08392	0.31542	0.14921	-0.16744	-0.11088	0.03514	0.18239	0.1037	0.11939	-0.10478
0.01784	0.04175	0.12039	-0.00746	0.0258	0.13245	0.117	0.3314	0.13168	-0.11973	-0.02313	0.08123	0.1794	0.10402	0.14295	-0.07971

I_SENESC	SENESC	X1_TARG	X1_TARG	PATOCYT	PATOCYT	2_IN_LIV	_TNFSF1	Y_RESPO	Y_RESPO	L2_SIGN	L2_SIGN	WITH_ST	WITH_STA	SP6_TAR	SP6_TAR
0.08044	0.10828	0.06349	0.14018	-0.04459	0.07693	-0.10607	0.02752	0.04286	0.06536	0.19131	0.03068	0.12139	-0.43174	-0.03596	0.0641
0.22545	0.06237	0.0837	0.09825	0.04151	-0.01614	-0.00303	0.05061	0.02644	0.05514	0.25142	-0.07207	0.14497	-0.41924	-0.08782	0.03682
0.12886	0.11075	0.10119	0.1103	0.0102	0.14887	-0.02198	0.01481	0.08575	0.06584	0.22884	0.00773	0.12406	-0.44161	-0.02175	0.00956
0.13367	0.10793	0.05577	0.14347	0.05808	0.09318	-0.02779	0.0036	0.12919	0.07023	0.16917	0.03606	0.03103	-0.45376	-0.0762	0.05595
0.17711	0.09696	0.05875	0.12819	0.16351	0.05795	0.02566	0.07541	0.0869	0.06701	0.20228	-0.05198	0.1287	-0.4284	-0.08214	0.0208
0.10362	0.10189	0.0939	0.13791	0.10863	0.23552	0.02951	0.02068	0.05196	0.04699	0.24334	-0.00958	0.14976	-0.3257	-0.0333	-0.00169
-0.023	0.11079	0.06473	0.16152	0.0889	0.18742	0.05909	0.00802	0.08349	0.08104	0.27516	0.00438	0.1595	-0.40602	-0.04971	0.02307
0.22311	0.12743	0.02169	0.14721	-0.00071	0.09044	-0.01823	0.07541	0.14132	0.08135	0.10824	0.0043	-0.01063	-0.39845	-0.09046	0.06487
0.20355	0.13834	0.03039	0.12016	0.09029	0.11881	0.08178	0.02288	0.15587	0.07311	0.15722	0.1158	0.03709	0.11767	-0.0709	0.07255
0.21045	0.10917	0.0215	0.15764	0.04322	0.12383	0.00887	0.0384	0.1559	0.07069	0.1411	-0.00104	0.01366	-0.40814	-0.08263	0.10304
-0.21893	0.11374	0.09953	0.09967	-0.03512	0.27479	-0.0661	0.07214	-0.00163	0.08205	0.2312	0.1	0.12425	-0.46873	0.0222	0.02697
-0.05587	0.12667	0.09035	0.13421	0.02722	0.32143	-0.03982	0.01485	0.05112	0.05757	0.22149	0.08425	0.16317	-0.4564	-0.09304	0.06051
-0.23924	0.11594	0.09369	0.06292	-0.01503	0.16931	0.12428	0.04045	-0.02396	0.0489	0.23412	0.08106	0.17449	-0.44689	-0.04713	-0.11349
0.07797	0.11103	0.03688	0.1563	-0.03878	0.19377	-0.03444	0.042	0.06658	0.07123	0.22905	-0.02261	0.19479	-0.47736	-0.04943	0.05452
0.06445	0.11337	0.08906	0.11801	0.02682	0.17744	-0.04357	0.05951	0.05	0.07404	0.25684	-0.08157	0.14974	-0.46186	0.00295	0.06958
-0.21537	0.08294	0.10478	0.09568	-0.03849	0.13497	-0.05068	0.04479	-0.01156	0.05838	0.25484	0.13559	0.16627	-0.4631	-0.01937	-0.01594
0.08902	0.11247	0.02141	0.12397	0.01819	0.00058	-0.0274	0.01365	0.06628	0.06115	0.20924	0.00723	0.06335	-0.46063	-0.09548	0.0666
0.18271	0.11468	0.03901	0.13651	0.04565	0.09084	-0.01376	0.00746	0.12898	0.06831	0.17211	-0.01835	0.07847	-0.4372	-0.08154	0.10643

ACT_WIT	Y_MIR21_	SE_TO_T	SE_TO_T	TH_FLT3	TH_FLT3	IOR_ANG	R_STEM	RESPONS	RGETS_A	RGETS_A	EABILIZE	TOCHON	MIC_STE	MIC_STE	S_OF_FL
0.34999	-0.02759	0.12659	0.16285	0.08591	0.06439	-0.33969	-0.12145	0.03862	-0.11119	-0.17483	0.12848	0.27284	0.08459	-0.06599	0.08353
0.33697	-0.02092	0.07387	0.12924	0.07526	0.07326	-0.42385	-0.06628	0.06253	0.17185	-0.08522	0.08471	0.27962	0.04924	-0.05204	0.09978
0.34399	0.00631	0.10243	0.18262	0.07296	0.09076	-0.31439	-0.25134	0.03842	-0.10243	-0.08792	0.1235	0.21586	0.08471	-0.07373	0.08941
0.32531	-0.03135	0.10731	0.17213	0.13026	0.07046	-0.3839	-0.18828	0.09635	0.15654	0.00679	0.07878	0.25025	0.12488	-0.08006	0.05394
0.3469	-0.03944	0.08771	0.16146	0.08473	0.06015	-0.298	-0.30403	0.07012	-0.2149	-0.01418	0.1078	0.23534	0.11603	-0.10754	0.07111
0.33315	-0.03273	0.1243	0.20627	0.096	0.1028	-0.23828	-0.06285	0.04169	0.20021	-0.08698	0.08852	0.25804	0.03773	-0.06276	0.09898
0.34379	-0.03856	0.1133	0.21807	0.10556	0.09173	-0.32765	0.09317	0.05135	-0.09622	-0.05928	0.09287	0.29267	0.08632	-0.07578	0.10618
0.32898	-0.04194	0.0808	0.18331	0.08929	0.05923	-0.33209	0.03636	0.10011	0.17412	0.00968	0.12073	0.25376	0.15068	-0.08004	0.05012
0.34296	-0.03398	0.05938	0.19507	0.13727	0.0468	-0.11652	-0.13951	0.14706	-0.09611	0.0244	0.0992	0.21611	0.14901	-0.09397	0.05369
0.33368	-0.04437	0.10868	0.20267	0.10946	0.06656	-0.28474	-0.0603	0.0852	-0.08844	0.06585	0.11051	0.2597	0.14202	-0.08431	0.06548
0.36054	0.01483	0.11508	0.17878	0.12668	0.07395	-0.26431	-0.05694	0.02228	0.16242	-0.12513	0.11658	0.27077	0.07134	-0.07821	0.08432
0.34503	-0.01642	0.12488	0.22272	0.11129	0.09068	-0.40482	-0.26875	0.03092	-0.1008	-0.11799	0.10889	0.29838	0.05944	-0.06537	0.0834
0.3556	-0.00417	0.10113	0.16869	0.00375	0.07161	-0.28621	-0.24811	-0.01736	-0.02334	-0.00454	0.14347	0.28052	0.00811	-0.00178	0.09436
0.33959	-0.03312	0.0676	0.18629	0.09197	0.07886	-0.37725	-0.34803	0.07582	-0.12137	-0.04449	0.09348	0.29179	0.10288	-0.1014	0.06086
0.35724	-0.01598	0.12507	0.17539	0.09893	0.0879	-0.40381	-0.1757	0.05762	0.15965	-0.07025	0.12357	0.25536	0.08814	-0.07587	0.08305
0.36824	0.00188	0.14371	0.14665	0.13616	0.06747	-0.42546	-0.25586	0.00922	-0.12474	-0.074	0.10826	0.27913	0.00568	-0.04619	0.10422
0.33285	-0.03013	0.06792	0.15889	0.07574	0.08042	-0.42539	0.03619	0.04948	-0.19433	-0.06152	0.10002	0.26831	0.08179	-0.04528	0.07163
0.31714	-0.03102	0.09453	0.17431	0.08764	0.0675	-0.28009	0.32814	0.05163	-0.30616	0.00894	0.10978	0.23076	0.1073	-0.11666	0.06131

S_OF_FL	OXIDIZED	OXIDIZED	OXIDIZE	OXIDIZE	XIDIZED_	OXIDIZE	XIDIZED	XIDIZED	OXIDIZE	OXIDIZE	IDIZED_P	DIZED_PH	XIDIZED_	XIDIZED_	OXIDIZE
0.04274	-0.06466	0.00412	-0.13707	0.02871	0.02445	0.04088	0.02803	-0.12171	-0.05291	-0.20754	0.1792	-0.05112	-0.1018	0.17315	-0.06256
0.04482	-0.07308	-0.03723	-0.08898	0.02568	0.01598	-0.01429	0.03851	-0.14613	-0.06025	-0.19792	0.28538	-0.06442	0.05115	0.14997	-0.04518
0.00365	-0.12717	-0.06784	-0.14904	0.02822	-0.17167	0.02246	0.05513	-0.11423	-0.04459	-0.19419	0.27793	0.03647	-0.0067	0.17329	-0.00461
0.03383	-0.11885	-0.0017	-0.12527	-0.00447	-0.02893	0.10486	0.01153	-0.08308	0.00103	-0.31828	0.28692	-0.01903	-0.09265	0.13847	0.01042
0.03564	-0.17285	0.03644	-0.14974	0.01359	0.00349	0.02281	0.02783	-0.11814	-0.04262	-0.3071	0.26	-0.13246	-0.06437	0.1741	0.00837
0.02268	-0.12799	-0.05161	-0.14809	0.02745	-0.11977	0.05719	0.00113	-0.05832	-0.13099	-0.18328	0.24645	0.02997	-0.03107	0.16901	-0.00258
0.02994	-0.18396	-0.02288	-0.16913	0.00083	-0.02188	0.06152	0.01386	-0.09317	-0.03277	-0.26398	0.21062	-0.0346	0.02256	0.11449	-0.03913
0.02891	-0.11931	0.00944	-0.10725	0.02646	-0.08921	0.0662	-0.01259	-0.0881	-0.00507	-0.42426	0.30224	0.03959	-0.19318	0.16357	0.03139
0.06302	-0.13523	-0.01467	-0.13161	0.03146	-0.0806	0.04057	-0.01757	-0.13917	-0.00135	-0.14448	0.33204	-0.06138	-0.19383	0.19419	0.06011
0.03974	-0.1346	-0.00409	-0.14284	0.01003	-0.08633	0.06329	-0.00708	-0.13809	0.01243	-0.18525	0.35437	-0.036	-0.13563	0.14487	0.08446
0.04288	-0.1536	-0.01212	-0.09733	0.02551	0.0181	-0.0376	0.05881	-0.12963	-0.06288	-0.18794	0.1259	-0.0469	0.07185	0.11377	-0.08519
0.0451	-0.15163	0.00536	-0.17512	0.00695	0.01197	0.00359	0.04193	-0.07877	-0.00285	-0.29222	0.2284	-0.03312	-0.10919	0.18008	-0.11474
0.08958	-0.16672	0.06485	-0.12203	0.05001	0.01715	0.02547	0.0275	-0.07259	-0.08961	-0.06588	0.20219	0.01009	0.06825	0.12675	-0.09164
0.07017	-0.14048	-0.03744	-0.11495	0.046	-0.0408	0.04759	0.05481	-0.11855	-0.04303	-0.23143	0.27983	-0.00516	0.0545	0.17997	-0.03319
0.01333	-0.19033	-0.02208	-0.12963	0.04391	0.05803	0.04072	0.02471	-0.12476	-0.03053	-0.23974	0.22639	0.06951	0.01672	0.15194	-0.09389
0.02156	-0.11042	0.02815	-0.16943	0.02524	-0.05045	0.05291	0.08099	-0.06387	-0.05668	-0.20032	0.12393	0.01609	0.08866	0.11715	-0.13442
0.0237	-0.1229	-0.02141	-0.12601	0.02034	-0.05769	0.09668	0.05242	-0.12759	-0.07738	-0.2588	0.2572	0.01345	-0.02742	0.13127	-0.03321
0.02911	-0.1549	-0.00727	-0.1226	0.00606	-0.02533	0.02358	0.02563	-0.11897	-0.01233	-0.24175	0.29891	-0.04535	-0.11769	0.13237	0.02962

XIDIZED_	OXIDIZE	OXIDIZE	XIDIZED_	OXIDIZE	IDIZED_P	DIZED_P	XIDIZED	XIDIZED	D5_TARG	D5_TARG	_PSMD4
0.25176	-0.02692	-0.05539	-0.01153	0.44989	0.01219	-0.07256	-0.06068	0.01731	0.10851	0.10761	0.0585
0.25264	-0.05724	-0.05683	0.00488	0.43879	0.04188	-0.06108	-0.05709	0.01673	0.13412	0.08968	0.02655
0.25883	-0.058	0.05817	-0.02024	0.48142	0.02186	-0.13809	-0.13273	0.06115	0.13154	0.10046	0.07717
0.24215	-0.00245	0.09277	-0.03284	0.49287	-0.02439	-0.15188	-0.06435	0.04937	0.10541	0.0966	0.12291
0.26357	-0.05061	0.04216	0.02141	0.4911	-0.00452	-0.10747	-0.12224	0.1027	0.11277	0.10845	0.14776
0.25593	-0.06979	-0.03235	-0.10396	0.49586	0.05076	-0.08973	-0.10448	0.08432	0.13182	0.10106	0.08602
0.27698	-0.00492	0.13537	-0.04799	0.49991	0.04242	-0.14848	-0.15486	-0.04054	0.12261	0.10322	0.12948
0.26276	0.0014	0.05106	0.0178	0.49075	-0.04588	-0.12866	-0.08832	0.10219	0.10504	0.09956	0.14076
0.27594	-0.06479	0.09161	0.01653	0.4904	-0.05369	-0.12768	-0.07682	0.0683	0.09841	0.10552	0.22878
0.28273	0.02028	0.18878	0.02906	0.49181	-0.05561	-0.15629	-0.10149	0.06598	0.10826	0.10264	0.16587
0.23336	-0.10537	-0.04693	-0.15477	0.46592	0.06242	-0.04391	-0.07791	0.01558	0.12438	0.08862	0.03975
0.29335	-0.08439	0.01661	-0.09285	0.4897	-0.02143	-0.06287	-0.09684	0.01842	0.12195	0.10466	0.11741
0.21054	0.01103	-0.15536	-0.09051	0.46204	0.07734	-0.07107	-0.12377	-0.07646	0.12718	0.09702	-0.07805
0.24199	-0.04181	0.0558	-0.05862	0.48705	0.00242	-0.10809	-0.09762	0.0484	0.1076	0.09692	0.11303
0.2703	-0.08401	0.01453	-0.08622	0.4793	-0.01867	-0.10424	-0.12972	-0.01521	0.12459	0.10494	0.13035
0.28276	-0.07065	-0.06913	0.01407	0.353	0.05858	-0.11216	-0.09292	-0.00763	0.13562	0.10265	0.04151
0.21298	-0.03837	-0.01511	-0.07106	0.48723	-0.01682	-0.1632	-0.10417	0.04374	0.10994	0.10636	0.05959
0.27411	-0.02524	0.04212	-0.00299	0.47455	0.00176	-0.12167	-0.10015	0.08806	0.10715	0.10123	0.16172

F4_FUSI	F4_FUSI	F4_FUSI	F4_FUSI	F4_FUSI	F4_FUSI	F4_FUSI	F4_FUSI	F4_FUSI	F4_FUSI	F4_FUSI	F4_FUSI	F4_FUSI	F4_FUSI	I_SRC_S	TARGETS	TARGETS	TARGETS	TARGETS	TARGETS
0.04897	0.04765	-0.043	0.11905	0.27571	0.11915	-0.05769	-0.07484	0.03897	0.03884	0.0979	0.0251	0.16876	0.05764	-0.32513	-0.02109	0.07407	0.01861	0.09026	0.01775
0.0578	0.03836	-0.08823	0.09417	0.19349	0.11305	-0.08954	-0.12795	-0.05612	0.04789	-0.0278	-0.02132	0.20698	0.05347	-0.41358	-0.0537	0.05861	-0.00311	0.03528	0.01762
0.06888	0.04077	0.0547	0.09192	0.21056	0.11055	-0.00203	-0.03416	0.04056	0.04128	0.12483	0.03841	0.19303	0.03514	-0.18203	-0.07295	0.07318	0.02998	0.06264	0.0338
0.0556	0.02644	0.09801	0.07986	0.16148	0.09253	0.00972	-0.01177	0.04066	0.07515	0.14708	0.05933	0.2348	0.0496	-0.16221	0.00828	0.10643	0.0076	0.06981	0.05329
0.05287	0.02836	0.12604	0.0768	0.20445	0.07849	0.06509	-0.00299	-0.02998	0.01649	0.06818	0.01109	0.19745	0.05332	-0.24795	-0.03871	0.08122	0.04128	0.04783	0.07745
0.06051	0.03764	0.0391	0.07045	0.28299	0.0916	-0.019	0.03849	-0.00638	0.09809	0.09891	0.00892	0.23015	0.06708	-0.26297	-0.04866	0.04411	-0.02172	0.07579	0.06531
0.04603	0.03576	0.03511	0.09305	0.2176	0.08123	-0.1779	-0.02804	0.05309	0.10197	0.08645	0.06666	0.23698	0.05103	-0.08433	0.01066	0.07211	0.01767	0.06465	0.07181
0.05119	0.0455	0.14718	0.06359	0.16154	0.0844	0.07751	0.05938	0.04259	0.09326	0.22053	0.06383	0.23581	0.04072	-0.18416	-0.05005	0.1112	0.01922	0.06995	0.03552
0.06644	0.03819	0.16982	0.06908	0.22511	0.08053	0.08178	0.04443	0.06965	0.08561	0.18968	0.04857	0.2493	0.06561	-0.27059	0.01792	0.11658	0.044	0.09523	0.07415
0.0354	0.04138	0.17226	0.08469	0.20433	0.09483	0.05824	-0.03167	0.08883	0.05672	0.06997	0.07074	0.23414	0.06315	-0.11093	0.0064	0.1182	0.01391	0.05666	0.06852
0.04352	0.03134	0.03326	0.16183	0.29703	0.11712	-0.21782	-0.02903	0.00373	0.10507	0.05972	0.00653	0.14939	0.04759	-0.36857	-0.08766	0.05592	0.04564	0.03832	0.02632
0.04593	0.05616	0.06815	0.09038	0.25463	0.11297	-0.10266	-0.00709	0.07611	0.12761	0.10891	0.05182	0.20955	0.03292	-0.19001	-0.05451	0.07222	0.08514	0.05405	0.0779
0.05357	0.03048	-0.07058	0.17147	0.23633	0.11466	-0.27808	-0.0005	-0.14679	0.03768	-0.11935	-0.00264	0.14107	0.04785	-0.44309	-0.0975	0.07296	0.02957	0.03446	-0.00365
0.07253	0.03933	0.03527	0.15357	0.26121	0.09487	0.0058	-0.01089	0.10606	0.07184	0.10022	0.02669	0.22523	0.042	-0.2786	-0.03992	0.06902	0.03832	0.06058	0.04298
0.06677	0.0349	0.01016	0.04991	0.18686	0.10862	0.01195	0.00247	0.04028	0.09495	0.0619	0.0243	0.19473	0.03007	-0.31979	-0.05668	0.07289	-0.0012	0.07377	-0.00377
0.05123	0.03328	-0.23646	0.15905	0.25248	0.11742	-0.23556	-0.04958	-0.17173	0.08442	0.02297	-0.00206	0.10232	0.03587	-0.45895	-0.08173	0.05687	0.04267	0.03961	0.01308
0.04643	0.03962	0.12293	0.03919	0.19779	0.09344	0.00628	0.0027	0.07415	0.06775	0.06752	0.0487	0.20852	0.03962	-0.25473	-0.06964	0.0784	0.02324	0.05191	0.07297
0.05404	0.03424	0.13572	0.09884	0.18236	0.09971	0.02174	-0.01134	0.08729	0.06338	0.13583	0.05104	0.20879	0.04391	-0.327	0.0123	0.09378	0.06792	0.05514	0.06135

PDE3B_T	CKFAN_A	CKFAN_A	ACKFAN_	ACKFAN_	KFAN_AN	KFAN_AN	TIC_SILE	ONSE_TO	ONSE_TO	SPONSE_	UV_HIGH	UV_HIGH	UV_LOW_	UV_LOW_	ESPONSE	ESPONSE	ESPONSE	ESPONSE	ESPONSE	ESPONSE
0.14059	0.06215	0.09095	0.18206	0.025	0.14335	0.08904	-0.07775	0.01258	0.18358	-0.07649	0.04772	0.14712	0.0274	0.13112	0.12511	0.06259	0.06858	0.04972	0.03204	0.08788
0.11711	0.04586	0.08059	0.18786	0.01091	0.14779	0.06918	-0.01167	0.03518	0.22179	-0.00851	0.06173	0.1594	-0.00148	0.18125	0.06041	0.08554	0.08094	0.07369	0.0021	0.09871
0.18357	0.07469	0.07972	0.18574	0.0427	0.15441	0.08499	-0.10019	0.04852	0.23099	-0.09181	0.03845	0.07664	-0.00735	0.16054	0.1148	0.03039	0.06879	0.07737	0.01955	0.07616
0.22221	0.07574	0.04465	0.16086	0.04258	0.13055	0.10209	-0.12239	0.06095	0.21811	-0.13115	0.02844	0.02612	-0.01114	0.13123	0.05349	0.01495	0.08635	0.04185	-0.01302	0.10851
0.20213	0.08919	0.08541	0.17267	0.07909	0.14299	0.12109	-0.09745	-0.00485	0.21434	-0.09722	0.02473	0.07389	-0.04156	0.13047	0.07571	-0.01578	0.06106	0.07304	-0.00159	0.06375
0.1386	0.0642	0.05833	0.17987	0.0859	0.14185	0.07169	-0.01613	0.04184	0.17923	-0.07568	0.02966	0.08897	-0.04901	0.10284	0.03777	0.07375	0.05585	0.06165	-0.02355	0.0659
0.17034	0.06875	0.06872	0.1738	0.04506	0.12372	0.11704	-0.05487	0.03186	0.22483	-0.03938	0.03951	0.22574	-0.0065	0.16827	0.11644	0.06242	0.03137	0.04262	-0.02731	0.09134
0.22672	0.09243	0.04276	0.18813	0.07649	0.13559	0.14298	-0.15839	0.04272	0.22631	-0.11432	0.02091	0.04533	-0.01956	0.10917	0.06516	-0.00373	0.07039	0.05254	-0.01612	0.07231
0.24127	0.08559	0.06647	0.19146	0.08517	0.13041	0.16736	-0.13684	0.03919	0.25212	-0.08575	0.02596	0.06014	-0.00382	0.11109	0.08922	0.03004	0.05327	0.05337	-0.01075	0.0706
0.23177	0.09474	0.0789	0.16567	0.06977	0.1141	0.14931	-0.09023	-0.01089	0.24424	-0.07224	0.03019	0.11831	-0.03426	0.14313	0.07598	0.01761	0.0564	0.0664	-0.02219	0.07404
0.17244	0.06556	0.07519	0.18402	0.02938	0.13504	0.08482	-0.08102	0.04414	0.18467	-0.06538	0.03698	0.12237	0.01463	0.12804	0.09674	0.0775	0.05139	0.03746	0.00358	0.07906
0.17048	0.07233	0.09575	0.18084	0.05085	0.13585	0.08688	-0.08786	0.07562	0.21796	-0.11828	0.02481	0.1479	-0.02949	0.19046	0.12213	0.00358	0.06966	0.05403	-0.00727	0.0485
0.0766	0.04751	0.05128	0.18042	0.07799	0.13147	0.05719	-0.08563	-0.02879	0.17411	-0.05421	0.05015	0.18184	-0.00414	0.17448	0.07603	0.06733	0.08056	0.04759	-0.00981	0.10004
0.21906	0.076	0.06628	0.15616	0.05324	0.13987	0.1244	-0.09085	0.04606	0.2018	-0.099	0.04314	0.11249	-0.01143	0.15718	0.11943	0.03647	0.06962	0.04859	0.03515	0.10544
0.17918	0.07199	0.05747	0.19485	0.01555	0.14103	0.11814	-0.08942	0.02192	0.20683	-0.0874	0.04052	0.10931	0.00317	0.14988	0.11351	0.02862	0.08682	0.0609	-0.01218	0.08293
0.04808	0.05028	0.07003	0.1661	0.02099	0.14672	0.06251	-0.10335	0.04499	0.19097	-0.06196	0.04159	0.14413	0.0164	0.14456	0.10611	0.03035	0.07077	0.05223	0.00103	0.08138
0.18342	0.07792	0.07433	0.17101	0.03071	0.14166	0.07545	-0.15087	0.04836	0.16792	-0.07572	0.03922	0.04461	0.02638	0.10216	0.0539	0.04658	0.08894	0.05208	0.01097	0.07654
0.20074	0.08444	0.07484	0.16269	0.10496	0.12841	0.14058	-0.13951	0.02753	0.21324	-0.10827	0.0242	0.17674	-0.04923	0.15183	0.05582	0.01077	0.04777	0.0585	-0.03057	0.0824

ESPONSE	ESPONSE	KEMIC_S	KEMIC_S	CYCLE	ITS_OF_MI	ADIPOGE	ADIPOGE	ESPONSE	ESPONSE	CEBP_TA	TERT_TA	ANDER_I	ANDER_I	RECT_IRR	RECT_IRR	F5_TARG
0.08778	0.06083	0.11329	0.00045	0.07078	0.03412	0.22926	0.18853	-0.01896	-0.08984	0.1355	-0.26441	0.13692	0.04526	0.17575	0.01489	0.15804
0.11506	0.07466	-0.30992	-0.04982	0.04537	0.02087	0.25028	0.16495	0.06907	-0.06958	0.10201	-0.22836	0.09625	-0.11769	0.13772	-0.088	0.16841
0.09328	0.0975	-0.14517	-0.02246	0.07923	0.04383	0.26174	0.17439	0.10428	-0.06835	0.14203	-0.36871	0.10104	-0.03313	0.13447	-0.05147	0.15532
0.07852	0.08734	-0.00715	-0.03649	0.06141	0.03075	0.25818	0.20677	0.12424	0.17562	0.15946	-0.34764	0.01341	-0.06103	0.09755	-0.04983	0.19896
0.10533	0.06492	0.03075	-0.11848	0.0523	0.02785	0.25078	0.18852	-0.00964	0.10446	0.13233	-0.33484	-0.03421	-0.11988	0.12087	-0.07083	0.20767
0.0806	0.07137	-0.16775	-0.11313	0.09431	0.02528	0.26882	0.21056	0.16862	-0.03664	0.14975	-0.33342	-0.34291	-0.08725	0.08125	-0.06122	0.13705
0.08581	0.04804	-0.12802	-0.18879	0.0461	0.02351	0.30026	0.20904	0.24028	-0.00264	0.14369	-0.38988	-0.00184	-0.08791	0.06621	-0.08968	0.12434
0.0592	0.08606	0.0654	-0.1285	0.04786	0.03334	0.25415	0.21938	0.13377	-0.05003	0.17936	-0.28338	0.02159	-0.06772	0.1477	-0.00353	0.19372
0.06546	0.07514	0.04446	-0.09642	0.03326	0.02784	0.26814	0.2022	0.18166	0.02995	0.15062	-0.02267	-0.03693	-0.01391	0.08754	0.01723	0.18169
0.11711	0.06122	0.14004	-0.09217	0.01735	0.02672	0.25388	0.20949	0.10279	0.05549	0.14338	-0.34569	0.14307	-0.10067	0.17421	-0.03517	0.15323
0.06754	0.06893	0.0558	-0.10735	0.09971	0.03934	0.24342	0.14409	0.14497	0.00476	0.14198	-0.3827	0.07834	-0.09632	0.08156	-0.05565	0.13887
0.0689	0.06737	-0.12032	-0.03812	0.07615	0.03696	0.27684	0.17641	0.14272	-0.0997	0.17051	-0.38977	0.05501	-0.07196	0.08539	-0.0254	0.16367
0.06997	0.05064	-0.16137	-0.0649	0.14817	0.03799	0.24669	0.13768	0.13367	0.11591	0.10224	-0.39837	0.16125	-0.09214	0.12017	-0.1127	0.19124
0.06669	0.09039	-0.06232	-0.11789	0.06522	0.02593	0.26164	0.1723	0.13694	0.04069	0.16404	-0.22808	0.11824	-0.04791	0.16056	-0.03884	0.17163
0.05296	0.07255	0.07953	-0.10055	0.07705	0.02961	0.25012	0.17186	0.12435	0.03012	0.1842	-0.28334	0.13186	-0.06422	0.12456	-0.05128	0.14116
0.01834	0.04196	-0.37586	-0.09257	0.11497	0.03577	0.26367	0.14254	0.14407	0.07927	0.11292	-0.41541	0.09362	-0.07446	0.11981	-0.08023	0.16816
0.08425	0.06287	-0.11104	0.01897	0.03963	0.0193	0.25529	0.1889	0.12823	0.04879	0.16589	-0.34852	-0.02597	-0.05159	0.14702	-0.03934	0.17046
0.11556	0.08256	0.09101	-0.04712	0.06978	0.02907	0.24394	0.18034	0.10348	-0.00088	0.12813	-0.41161	-0.36398	-0.04755	0.04794	-0.04632	0.16655

F5_TARG	A_METAS	ORE_NF	NCER_20	NCER_20	ANCER_Z	ANCER_Z	P_TARGET	CANCER_PL	ATELET	DENDRO	ROCYTE_I	ROCYTE_	DL_CHO	L_CHOL	ANTIGEN	OSTATIC
-0.05904	0.15633	-0.11103	0.06765	0.08559	0.05637	0.01855	0.10615	-0.08044	0.29582	-0.119	-0.00676	0.04667	0.09827	0.1814	0.16957	0.13437
0.04267	0.11018	-0.06225	0.08132	0.10332	0.08489	0.02099	0.10642	0.11525	0.28554	-0.03489	-0.0071	0.07949	0.10941	0.10811	0.15443	0.12949
0.01096	0.16626	-0.0925	0.09335	0.09093	0.06598	0.02514	0.12562	0.0749	0.29675	0.0002	-0.00175	0.07995	0.09767	0.18538	0.18028	0.12476
0.04468	0.18198	-0.1008	0.06648	0.0764	0.04547	0.04482	0.08423	-0.02054	0.3103	0.01299	0.00489	0.04652	0.11304	0.10117	0.18898	0.11982
-0.03306	0.09994	-0.05588	0.07033	0.07686	0.06128	0.02152	0.09734	0.03781	0.30637	-0.01868	-0.00567	0.04258	0.14148	0.05693	0.1906	0.12375
0.09416	0.1037	-0.11343	0.07508	0.09496	0.0606	-0.00557	0.10401	0.18279	0.31685	-0.04648	-0.01244	0.07285	0.15704	0.14097	0.1801	0.1225
0.05501	0.13143	-0.08668	0.05699	0.10793	0.06042	0.01363	0.08952	0.08087	0.3336	0.00655	-0.00515	0.06667	0.16728	0.13993	0.17092	0.12304
0.09045	0.14009	-0.07721	0.03908	0.05976	0.0318	0.0528	0.08726	-0.05767	0.33054	0.03518	0.00821	0.01542	0.14227	0.0728	0.17987	0.11827
0.03122	0.15551	-0.06759	0.06595	0.05512	0.03475	0.03824	0.09357	-0.00821	0.33021	-0.06148	0.01573	0.02017	0.15559	0.07567	0.19305	0.11508
0.02794	0.14348	-0.0239	0.05346	0.04896	0.04168	0.0347	0.08576	-0.03066	0.33275	-0.04878	-0.00021	0.02965	0.18826	0.08193	0.18708	0.1136
0.04102	0.18975	-0.13985	0.07448	0.11645	0.06031	0.00589	0.12261	0.113	0.29889	-0.09199	-0.01155	0.08623	0.16068	0.17145	0.16508	0.14386
0.0685	0.1235	-0.15137	0.06043	0.08047	0.04632	0.00335	0.09216	0.08956	0.31085	-0.06114	-1.7E-05	0.06684	0.15504	0.11334	0.17974	0.14792
0.02015	0.16655	-0.16168	0.05339	0.11727	0.06109	0.01656	0.10373	0.10934	0.28799	-0.12279	0.00136	0.08968	0.03427	0.08927	0.15326	0.13282
-0.02361	0.12383	-0.08159	0.03149	0.11565	0.05425	0.03553	0.11306	-0.00727	0.31214	-0.00717	0.00288	0.02838	0.17739	0.12064	0.17019	0.12807
0.05435	0.17322	-0.10003	0.06603	0.08709	0.05951	0.02514	0.09405	-0.04817	0.32059	0.03562	-0.01945	0.06422	0.16536	0.11292	0.18702	0.13384
0.14277	0.16054	-0.13154	0.07058	0.11521	0.06951	-0.01725	0.09332	0.06169	0.28571	-0.03671	-0.01376	0.0883	0.18133	0.06754	0.14372	0.14527
0.10697	0.19036	-0.12496	0.0467	0.10826	0.05464	0.03426	0.09123	-0.03115	0.32207	-0.01162	0.00735	0.03194	0.15572	0.16747	0.16958	0.13422
0.02082	0.17824	-0.08488	0.04947	0.0931	0.04246	0.04672	0.08671	-0.00234	0.31862	0.06641	0.00679	0.03688	0.17024	0.03008	0.18108	0.1184

IMMUNE	ALL_VS_	ALL_VS	ETS_OF_	KO_MESEN	O_MESEN	TATI_TAR	TATI_TAR	MOR_INV	SR1_TAR	SR1_TAR	BCL11B	BCL11B	ND_REC	AND_REC	VS_RECT
-0.01677	0.18555	0.12938	0.01847	0.20364	0.04687	0.21374	0.36401	0.14751	0.05441	0.03611	0.02443	0.15335	-0.04409	0.16683	0.03001
-0.04032	0.15075	0.14471	0.05356	0.18938	0.02558	0.20331	0.00116	0.19124	0.01343	-0.01237	-0.00867	0.11641	-0.06683	0.18126	0.0496
0.00291	0.16423	0.1275	0.04979	0.2107	0.07825	0.21136	0.17203	0.23326	0.03598	0.03527	0.02346	0.14742	-0.09807	0.18642	0.01118
-0.02977	0.22347	0.11899	0.03666	0.20761	0.04507	0.23497	0.3847	0.26253	0.03933	0.0619	0.01725	0.15374	-0.09847	0.164	0.04
-0.03403	0.21521	0.16378	0.04489	0.19446	0.0622	0.15601	0.31026	0.22699	0.03053	0.01579	0.04571	0.16547	-0.08653	0.16611	0.03339
-0.04823	0.16725	0.15103	0.05495	0.19971	0.10467	0.15515	-0.18745	0.21104	0.04515	0.0013	-0.00457	0.16512	-0.02401	0.18636	0.0104
-0.03074	0.19627	0.09825	0.00749	0.19876	0.12166	0.24165	0.07705	0.2116	0.05651	-0.00287	0.00747	0.17462	-0.07452	0.19286	0.03181
-0.00958	0.23762	0.11946	0.04363	0.19762	0.03758	0.10659	0.29628	0.17694	0.05562	0.07173	0.03359	0.18021	-0.04812	0.14673	0.05809
-0.01703	0.21845	0.13167	0.04574	0.2024	0.07833	0.10762	0.10434	0.21631	0.05188	0.06009	0.02721	0.17284	-0.06662	0.15704	0.04251
-0.04577	0.24902	0.13002	0.0297	0.21296	0.04217	0.13795	0.25883	0.2119	0.04481	0.04326	0.04307	0.17554	-0.06972	0.15247	0.04379
-0.01645	0.15406	0.144	0.0171	0.2063	0.10237	0.24096	0.3289	0.24512	0.03254	0.02073	0.02581	0.15391	-0.07167	0.20185	0.04768
0.01749	0.14827	0.14742	0.00857	0.21276	0.10341	0.15873	0.35103	0.22585	0.06647	0.04744	0.05236	0.1892	-0.06424	0.19409	0.03894
-0.00683	0.0769	0.17313	0.0093	0.2038	0.07892	0.25142	-0.16911	0.08149	0.02297	-0.0256	-0.0348	0.13809	-0.06819	0.19181	0.03144
0.00641	0.17425	0.12892	0.03112	0.19852	0.04097	0.19071	0.32361	0.16357	0.0325	0.02382	0.01664	0.15973	-0.10567	0.16486	0.03371
0.01509	0.19621	0.13095	0.0372	0.20556	0.04826	0.23075	0.40877	0.12365	0.04816	0.02934	-0.0023	0.14088	-0.05371	0.18189	0.04045
0.00223	0.10912	0.1238	0.0659	0.19782	0.04414	0.23936	0.16205	0.11204	0.02114	-0.00281	-0.05147	0.10602	-0.045	0.20591	0.04239
-0.01844	0.20503	0.13623	0.02873	0.20205	0.03677	0.22117	0.24508	0.206	0.03138	0.04131	-0.01501	0.14651	-0.08001	0.15727	0.03776
-0.01807	0.17863	0.11335	0.02626	0.20793	0.06613	0.19519	0.03398	0.19109	0.03579	0.04396	0.04321	0.17593	-0.08625	0.16171	0.0376

VS_RECT	TOSIS_BY	TOSIS_BY	IS_BY_SE	IS_BY_SE	E_TO_MC	E_TO_MC	O_MC_AN	IO_MC_AN	NG_VS_N	NG_VS_N	G_VS_NO	G_VS_NO	CENT_VS	CENT_V	ENT_VS_
0.04045	0.05559	0.06758	0.08612	0.07103	0.05967	0.07463	0.10112	0.111	0.03592	0.0827	0.06679	0.10597	0.17395	0.13055	0.07783
0.10619	0.05983	0.06221	0.06587	0.07455	0.07071	0.05979	0.09722	0.10433	-0.1107	-0.20107	0.01877	0.08839	0.02343	0.0742	-0.01397
0.05109	0.05778	0.07445	0.0533	0.08745	0.06662	0.07706	0.07382	0.13357	0.07302	-0.14133	0.0512	0.12367	0.02525	0.09465	0.09546
0.02253	0.03587	0.07957	0.05624	0.09828	0.04449	0.07568	0.07323	0.16305	0.20435	-0.06638	0.07279	0.09149	0.03287	0.08502	0.18395
0.03616	0.04152	0.06827	0.07327	0.06465	0.04558	0.06589	0.07607	0.12375	0.24919	0.02447	0.10079	0.09179	0.135	0.08247	0.20179
0.0712	0.03135	0.05832	0.08322	0.07995	0.05147	0.0533	0.07934	0.13291	0.16339	-0.00416	0.06395	0.11433	-0.01082	0.06716	0.13288
0.02629	0.03855	0.07167	0.08602	0.09634	0.05544	0.06725	0.0888	0.16623	0.15093	0.02987	0.05831	0.13017	0.06253	0.1248	0.07616
0.0183	0.02801	0.07004	0.05096	0.09045	0.03208	0.06594	0.04755	0.15185	0.17658	0.05451	0.0839	0.08852	0.05866	0.08559	0.21829
0.02334	0.0265	0.06779	0.05055	0.09228	0.02802	0.06332	0.03824	0.17463	0.24959	0.00351	0.10947	0.0839	0.04086	0.20988	0.22939
0.00935	0.03068	0.06886	0.05584	0.10892	0.04145	0.05729	0.04875	0.19406	0.19034	0.11993	0.08076	0.09919	0.1338	0.1259	0.12597
0.09507	0.05754	0.06012	0.07593	0.07539	0.07146	0.06253	0.08712	0.08026	-0.16905	-0.06359	0.00452	0.11242	0.16712	0.14224	-0.04013
0.04989	0.04915	0.06661	0.0612	0.09895	0.06341	0.06491	0.0656	0.1427	0.10853	-0.05005	0.04991	0.11646	0.06451	0.09096	0.15652
0.07767	0.06421	0.06988	0.08377	0.07	0.08091	0.06341	0.14662	0.08601	0.03759	-0.14482	-0.02377	0.08414	0.05493	0.00283	0.03438
0.01449	0.04068	0.07559	0.06707	0.07629	0.04956	0.07341	0.08214	0.12484	0.09124	-0.09008	0.06783	0.07582	-0.01381	0.12809	0.13279
0.04285	0.04726	0.07822	0.07567	0.08739	0.06214	0.07436	0.10515	0.12324	0.21031	0.03937	0.07084	0.1286	0.12514	0.15385	0.20235
0.07744	0.06254	0.0749	0.05457	0.07571	0.08113	0.07949	0.10994	0.09888	-0.2183	-0.33725	-0.00894	0.12693	-0.06033	0.12205	-0.06496
0.0215	0.04491	0.06608	0.08119	0.07506	0.05326	0.06966	0.09735	0.10506	-0.00535	-0.06215	0.06582	0.07667	-0.00538	0.0746	0.1443
0.02545	0.03823	0.0753	0.08136	0.09433	0.04146	0.07623	0.10433	0.17133	0.22375	0.03001	0.07829	0.08601	0.16217	0.1136	0.15689

ENT_VS_	NT_VS_N	NT_VS_N	CENT_VS	CENT_V	RESPON	X_IRF3_TA	IRF3_TA	STOMA_W	INOBLAS	M_E2A_TA	M_E2A_TA	TIC_LETH	NCER_CO	LK3_TAR	LK3_TAR
0.08038	0.00826	0.12234	0.03567	0.10807	0.1257	-0.0564	0.06829	0.26239	-0.01763	0.13889	0.07945	0.16217	0.12	0.10815	0.08337
0.06251	-0.00151	0.12646	0.04949	0.06538	0.17799	-0.07815	0.0977	0.29357	-0.07017	0.08101	0.14399	0.14949	0.122	0.07065	0.04702
0.08404	0.0264	0.15825	0.11146	0.09562	0.22398	-0.0195	0.19371	0.27428	0.076	0.135	0.16255	0.15812	0.12297	0.06733	0.05597
0.08277	0.0656	0.14733	0.0488	0.10569	0.20643	0.04512	0.22212	0.24749	0.02077	0.18906	0.05788	0.15372	0.09755	0.11299	0.06813
0.08237	0.10549	0.13644	0.05541	0.14186	0.18462	0.02078	0.16354	0.27337	-0.00269	0.21661	0.06438	0.15123	0.08834	0.07565	0.04854
0.09062	0.0157	0.14992	0.08506	0.09864	0.23194	-0.04474	0.16486	0.27861	-0.05182	0.21396	0.11138	0.17	0.10469	0.1025	0.13506
0.05355	0.01654	0.14407	0.08392	0.05792	0.29076	-0.02183	0.28159	0.23214	0.06091	0.1773	0.13297	0.15478	0.10366	0.12601	0.09354
0.10937	0.08686	0.15605	-0.013	0.1317	0.1871	0.09071	0.20121	0.22894	0.05146	0.19442	0.03499	0.15756	0.07045	0.09264	0.08063
0.15468	0.11361	0.15458	0.01157	0.16955	0.23563	0.02968	0.29268	0.21116	0.06366	0.24528	0.05386	0.15444	0.08129	0.12557	0.10354
0.09235	0.07514	0.13543	0.03496	0.13376	0.28218	0.00319	0.2937	0.25292	0.06426	0.22669	0.06821	0.14988	0.09686	0.11616	0.04275
0.08989	-0.04581	0.13663	0.09284	0.03532	0.10858	-0.04291	0.10065	0.27577	0.01379	0.06361	0.12725	0.14369	0.10984	0.09359	0.04834
0.09486	-0.02232	0.1407	0.06598	0.067	0.24445	-0.01462	0.22573	0.26546	0.018	0.18773	0.05704	0.15813	0.10776	0.13346	0.05008
0.0624	-0.09034	0.1185	0.05977	-0.00078	0.17322	0.02947	0.09031	0.27848	0.02035	0.06822	0.15885	0.131	0.13495	0.14619	0.00415
0.0886	0.0381	0.11695	-0.01954	0.1282	0.14706	0.03585	0.12532	0.26744	0.05222	0.1891	0.10574	0.15983	0.09071	0.11758	0.09262
0.10795	0.06915	0.14575	0.07485	0.10312	0.14717	-0.02886	0.17946	0.28113	0.0211	0.14034	0.06124	0.16006	0.10015	0.1074	0.08444
0.06956	-0.05577	0.13424	0.1213	0.00203	0.14039	-0.00508	0.11843	0.26695	0.01631	0.08111	0.1263	0.13974	0.12483	0.11575	0.07442
0.09266	0.06341	0.12883	-0.03511	0.12396	0.1706	0.04071	0.04489	0.24816	-0.02993	0.1515	0.04793	0.15192	0.11443	0.09286	0.08074
0.08131	0.07635	0.13795	0.02921	0.10945	0.25491	0.01459	0.26369	0.25243	0.06532	0.21886	0.09346	0.15003	0.08487	0.10232	0.05637

IF1A_TAR	IF1A_TAR	VIA_ELK	VIA_ELK	POXIA_VI	XIA_VIA_E	XIA_VIA_E	POXIA_VI	POXIA_VI	OXIA_VIA
0.33457	-0.02843	0.12985	0.13058	0.09746	0.08421	0.03024	0.09839	0.14857	0.15957
0.3024	-0.02361	0.15146	0.11436	0.05395	0.10693	-0.04832	0.11689	0.11233	0.07124
0.29961	0.00972	0.13499	0.12285	0.06906	0.07977	-0.04414	0.11518	0.10691	0.1947
0.29571	0.09994	0.14193	0.12963	0.08539	0.08757	0.00329	0.10818	0.12375	0.12861
0.28536	0.02814	0.14481	0.11972	0.09503	0.07378	-0.00882	0.11089	0.10735	0.15342
0.313	0.01471	0.12349	0.11922	0.07242	0.09461	0.00375	0.12166	0.1142	0.19303
0.30292	0.02653	0.1416	0.11518	0.07901	0.0774	-0.06409	0.1266	0.07605	0.13398
0.28205	0.12575	0.14082	0.13805	0.10363	0.09302	0.03368	0.11432	0.1144	0.11277
0.26986	0.10565	0.14246	0.1195	0.10863	0.08944	0.0216	0.11249	0.11535	0.13346
0.28158	0.11169	0.14656	0.10195	0.07872	0.08093	-0.02348	0.1095	0.09307	0.14441
0.33094	0.02602	0.1514	0.1569	0.08798	0.07949	0.00577	0.11865	0.12114	0.19976
0.31996	0.0259	0.13387	0.12166	0.08793	0.08386	0.00012	0.1116	0.10879	0.19695
0.3602	0.00469	0.14391	0.15044	0.0761	0.06196	-0.01649	0.1142	0.15027	0.13518
0.30239	0.03955	0.13637	0.13119	0.08859	0.06929	0.00424	0.10755	0.12907	0.12552
0.30529	0.01309	0.15496	0.12244	0.09598	0.08458	0.04064	0.11244	0.13095	0.16523
0.33055	-0.03409	0.14423	0.13847	0.08542	0.06709	0.01332	0.1186	0.11382	0.18181
0.29876	0.03561	0.12478	0.13374	0.07566	0.0805	0.0215	0.11244	0.15215	0.13712
0.28385	0.08603	0.12722	0.13429	0.09637	0.0978	-0.01997	0.10963	0.11249	0.13754

POXIA_VI	PANCREA	PANCREA	XO1_ENH	O1_ENHA	OXO1_TO	SPHERIC	SPHERIC	D_KLF10	D_KLF10	EX_TARG	EX_TARG	S_OF_IR	5_LYMPH	_LYMPH	ULTIPLE	DEMS_MA	DEMS_MA	ROEMS_M	EF_TARG	EF_TARG	ORUBICI	XANTRON
0.12056	0.13024	0.17402	0.08762	0.07747	0.10776	0.22028	-0.06423	0.1644	0.21158	0.09646	0.0858	0.1033	0.10891	0.15373	0.18701	0.20092	-0.11009	0.19816	0.23103	-0.00074	0.01321	-0.00224
0.14279	0.14296	0.16338	0.09694	0.09297	0.11407	0.16975	0.09112	0.17177	0.11268	0.14651	0.06701	0.06589	0.07504	0.15255	0.17698	0.16679	-0.01233	0.07017	0.2097	-0.00901	0.01615	0.01526
0.15483	0.14659	0.17635	0.08461	0.08199	0.09439	0.1931	-0.19186	0.18808	0.16107	0.16507	0.04956	0.09203	0.11479	0.20374	0.17984	0.18046	-0.17879	0.11949	0.16577	-0.00085	0.03059	0.00223
0.12801	0.15268	0.19484	0.07645	0.06581	0.09861	0.20361	-0.26591	0.20904	0.15644	0.15931	0.06968	0.09589	0.1031	0.26943	0.18764	0.31031	-0.39845	0.15704	0.17814	0.01861	0.03281	0.01654
0.12457	0.13333	0.1743	0.07712	0.07417	0.09967	0.20791	-0.16242	0.18162	0.11404	0.15723	0.09648	0.08094	0.14686	0.27865	0.16885	0.27408	-0.40655	0.21869	0.14737	-0.01625	-0.00104	-0.01862
0.16368	0.16454	0.17329	0.08528	0.08961	0.10102	0.19156	-0.25603	0.17605	0.16225	0.19998	0.04991	0.06516	0.15992	0.2135	0.19872	0.17395	-0.36252	0.07928	0.17372	0.03803	-0.00628	-0.09287
0.16378	0.14633	0.17881	0.08113	0.08393	0.10025	0.23244	-0.28531	0.16554	0.17972	0.17023	0.07258	0.06763	0.11888	0.25136	0.18235	0.22126	-0.14321	0.17909	0.1745	0.10129	-0.057	-0.07181
0.12421	0.16283	0.19109	0.06837	0.06668	0.09243	0.21028	-0.39781	0.20589	0.13423	0.17466	0.08124	0.07301	0.14648	0.30197	0.17758	0.3069	-0.25753	0.18594	0.18617	0.01621	0.03781	0.01246
0.12858	0.15956	0.18571	0.07101	0.07181	0.09839	0.25303	-0.26542	0.20832	0.10289	0.17022	0.0716	0.08318	0.14568	0.28039	0.16005	0.3387	-0.20698	0.19038	0.19209	0.0386	-0.00758	0.0187
0.11019	0.13959	0.18459	0.06756	0.05562	0.08809	0.20524	-0.09337	0.17176	0.10732	0.17348	0.05622	0.06808	0.14762	0.29913	0.17627	0.28015	-0.32341	0.27326	0.17048	0.0075	-0.04868	0.00501
0.15842	0.14434	0.16806	0.09472	0.09649	0.11388	0.12508	-0.10535	0.11851	0.23991	0.13713	0.06945	0.09352	0.10359	0.11348	0.2006	0.1789	-0.05126	0.2113	0.21647	-0.01399	-0.04246	-0.01615
0.13106	0.15089	0.18092	0.07387	0.07771	0.0831	0.22059	0.02193	0.15243	0.2572	0.16286	0.05265	0.10428	0.1126	0.19675	0.19544	0.16387	-0.13123	0.14657	0.20326	0.03341	-0.04489	-0.02083
0.16264	0.14085	0.16582	0.09448	0.09078	0.09921	0.11278	-0.40961	0.18246	0.14756	0.12891	0.09349	0.08981	0.0674	0.06452	0.18536	0.13208	0.16118	0.00022	0.22571	-0.01415	-0.03335	-0.03378
0.13317	0.15496	0.17778	0.07981	0.0808	0.10104	0.18605	-0.23642	0.19031	0.18467	0.13517	0.07657	0.08242	0.11406	0.23554	0.18818	0.25334	-0.38911	0.19264	0.1687	-0.00459	0.00434	-0.03104
0.16416	0.15316	0.17947	0.07832	0.08251	0.09871	0.17602	-0.17165	0.15085	0.18596	0.14099	0.05395	0.10048	0.0881	0.22366	0.18298	0.18997	-0.36023	0.20856	0.1929	-0.02101	-0.02613	0.00457
0.17219	0.13863	0.15648	0.09595	0.10383	0.10978	0.15569	-0.24532	0.12568	0.20778	0.14611	0.08574	0.08869	0.09442	0.02276	0.19897	0.09885	-0.2415	0.09036	0.19662	-0.00872	-0.00871	-0.07219
0.12154	0.14348	0.17325	0.08555	0.0811	0.11065	0.19848	-0.25462	0.18958	0.14671	0.15458	0.07365	0.06918	0.11103	0.17512	0.16298	0.22169	-0.0044	0.08638	0.18289	0.01242	-0.00533	-0.0402
0.11673	0.14214	0.18063	0.07308	0.07118	0.09269	0.18839	0.02255	0.17635	0.11328	0.16794	0.08045	0.06643	0.14886	0.25177	0.18931	0.26305	-0.28008	0.26927	0.17101	0.01189	0.03003	0.01946

PHOCYT	TE_AND	YTE_AND	_RESPON	CTCL_CU	TCL_PAT	IS_FUN	SIS_FUNC	OSIS_FU	SIS_FUNG	IS_FUNG	OSIS_FU	EZARY_SY	EZARY_S
0.06257	0.14348	0.06181	0.00273	0.24556	0.25929	0.12328	0.04196	0.17541	0.08464	0.20325	0.21873	-0.02944	0.17441
0.05599	0.07134	0.05977	0.00347	0.26891	0.24221	0.14308	0.0014	0.16448	0.09503	0.21125	0.23408	-0.1109	0.15338
0.0589	0.12649	0.0592	-0.04315	0.26663	0.18196	0.1214	0.00707	0.20015	0.13369	0.20436	0.21566	0.00811	0.17215
0.05135	0.21867	0.05277	0.12864	0.26823	0.01568	0.1314	0.08232	0.28189	0.09939	0.20678	0.19219	0.01844	0.19341
0.08401	0.1777	0.05249	0.03844	0.31183	0.35212	0.15213	0.04201	0.25951	0.10336	0.20353	0.27346	0.02494	0.1857
0.08424	0.14152	0.06603	-0.03348	0.24473	-0.33574	0.13841	0.00568	0.28554	0.12204	0.21121	0.18024	0.07816	0.16704
0.04618	0.1708	0.07506	-0.03609	0.25331	-0.06958	0.13737	0.00259	0.21138	0.09496	0.21406	0.16764	0.07263	0.15325
0.06335	0.22829	0.07751	0.10325	0.29168	0.21067	0.12569	0.07143	0.27125	0.08804	0.20339	0.23178	-0.02738	0.2041
0.06524	0.24731	0.07367	0.10211	0.2625	-0.12736	0.12942	0.14791	0.26085	0.0933	0.20848	0.19692	0.13296	0.19041
0.06574	0.226	0.04692	0.0356	0.28105	0.2725	0.14729	0.03589	0.28812	0.08557	0.20878	0.22833	0.09775	0.19682
0.06039	0.11069	0.06905	-0.00965	0.26358	0.27374	0.12653	0.00247	0.14715	0.12373	0.20583	0.1902	0.01729	0.17817
0.06265	0.13284	0.07499	0.16571	0.26437	0.07803	0.13842	0.02398	0.21425	0.12656	0.2114	0.19495	-0.01271	0.17104
0.04976	0.03571	0.08578	-0.06469	0.28138	0.28906	0.13	0.00383	0.21071	0.13828	0.20478	0.18649	-0.05396	0.14883
0.04183	0.14748	0.07596	-0.00053	0.2925	0.44548	0.12716	0.06064	0.21283	0.10989	0.20366	0.23911	-0.02184	0.18491
0.07308	0.17656	0.0846	-0.06858	0.27917	0.35547	0.13203	0.06237	0.2211	0.10712	0.20338	0.23606	0.01299	0.19967
0.05419	-0.03797	0.03048	-0.05353	0.27104	0.20275	0.13148	0.04943	0.1481	0.11148	0.17931	0.17905	-0.09742	0.14598
0.05254	0.15827	0.09519	-0.07479	0.26752	-0.00194	0.13504	0.07351	0.18846	0.11168	0.21385	0.18274	0.05577	0.1702
0.05726	0.14738	0.05386	0.06101	0.24834	-0.25859	0.13846	0.03354	0.25241	0.10822	0.20848	0.17243	0.01896	0.1692

E_TO_BC	IE_TO_BC	S_SIGNAL	K_SINGA	LK_SINGA	UB1_TARG	TB1_TARG	CANCER	RIS_HYP	OL7_TAR	OL7_TAR	L_WITH	LL_WITH	LL_WITH	LL_WITH	TH_CHR	WITH_M	ED_IN_L	TED_IN_L	LINE_BRAI	UNG_LIV
0.19249	0.05121	-0.16434	0.15212	-0.06762	0.01649	0.02469	0.07294	0.23461	-0.03069	-0.07082	0.02896	-0.00356	0.1289	0.15923	0.11435	-0.11365	-0.15982	0.05729	0.21676	0.32517
0.13737	0.11993	-0.12009	0.12128	-0.10201	0.00296	0.03765	-0.01496	0.22531	-0.1531	-0.13925	0.00477	0.0215	0.13419	0.10673	0.11866	-0.17024	-0.06829	0.03253	0.22328	0.32316
0.11312	0.06001	0.00489	0.16089	-0.04467	0.01002	0.04764	0.02799	0.20707	-0.05052	-0.17129	0.02917	0.0359	0.10483	0.11173	0.15039	-0.18399	-0.03607	0.04794	0.21475	0.30373
0.14642	0.07499	-0.03219	0.18899	-0.08136	0.00747	0.06567	0.03618	0.23553	0.06113	-0.09595	0.07396	0.10825	0.10243	0.15467	0.1916	-0.05554	-0.13966	0.08004	0.23067	0.33846
0.16824	0.15044	0.00419	0.19731	-0.10228	0.00341	0.04455	0.03704	0.21	-0.00334	-0.02505	0.00198	0.05473	0.10032	0.12477	0.16468	-0.15178	-0.18435	0.06841	0.27059	0.37148
0.13224	0.07081	-0.03872	0.15739	-0.02557	0.00341	0.04392	0.03547	0.23373	-0.04385	-0.08756	0.04445	0.05822	0.11138	0.09721	0.15097	-0.20094	-0.07168	0.0155	0.20062	0.30581
0.12489	0.09482	-0.11356	0.24167	0.15063	0.01475	0.0461	0.10637	0.23784	-0.06461	-0.0295	0.02413	0.03937	0.11399	0.11543	0.15677	-0.1517	-0.11066	0.05738	0.24506	0.34815
0.10978	0.19302	0.11861	0.11594	0.02005	0.01237	0.06674	0.05931	0.21944	0.02954	0.036	0.04461	0.09897	0.1176	0.142	0.19968	-0.09859	-0.16733	0.08191	0.22472	0.32526
0.0942	0.15155	0.0708	0.16127	0.06272	0.00199	0.0685	0.04789	0.22722	-0.00154	-0.003	0.03995	0.14383	0.10662	0.12937	0.20781	-0.11243	-0.29257	0.08515	0.3054	0.40045
0.10465	0.12528	-0.07276	0.15729	-0.00107	0.00816	0.05851	0.03195	0.21469	0.01638	-0.09309	0.07915	0.07776	0.09657	0.14493	0.1661	-0.11832	-0.25823	0.06628	0.22301	0.32598
0.11893	0.09133	-0.12845	0.20499	-0.04573	-0.0036	0.03032	-0.00509	0.22295	-0.00205	-0.09828	0.00288	-0.0507	0.12181	0.11451	0.08384	-0.16872	0.06706	0.03643	0.23339	0.3312
0.09776	0.02228	-0.03717	0.19609	0.00231	0.01433	0.04116	0.00548	0.21977	0.04328	-0.0026	0.01363	-0.04605	0.11881	0.10864	0.08022	-0.19122	-0.11348	0.03384	0.22518	0.31676
0.12287	0.08238	-0.17451	0.09278	-0.08302	0.0058	0.01793	0.03332	0.21335	-0.05952	-0.17863	0.00238	0.02516	0.14331	0.03799	0.08351	-0.19479	-0.02702	0.00933	0.23849	0.34733
0.15942	0.1187	-0.04594	0.22307	-0.09022	-0.00754	0.03517	0.0332	0.21829	0.01255	-0.06167	0.03207	0.05447	0.10867	0.15854	0.13213	-0.14711	-0.21433	0.06116	0.22267	0.32489
0.17272	0.07788	-0.04183	0.18279	-0.06832	0.00316	0.04619	-0.00027	0.21325	-0.08679	-0.06088	0.02905	0.0381	0.101	0.14825	0.1472	-0.22641	-0.07873	0.04556	0.21172	0.30928
0.18396	-0.00178	-0.0723	0.14774	-0.07903	-0.01337	0.04659	0.01422	0.2209	0.07238	-0.16045	-0.05234	-0.06262	0.11254	0.1101	0.00955	-0.23625	0.01126	0.03112	0.20337	0.30209
0.12458	0.10426	-0.02187	0.20344	-0.163	0.00216	0.03573	-0.00512	0.23541	0.03471	-0.11902	0.02971	0.0712	0.09446	0.13862	0.15376	-0.18419	-0.03043	0.07179	0.23663	0.34296
0.1253	0.13451	0.00188	0.16318	0.01542	0.00913	0.05296	0.0342	0.21635	0.05485	-0.05193	0.03064	0.08059	0.11966	0.13007	0.15333	-0.13827	-0.14574	0.08485	0.20809	0.31409

E_TNBC	INE_META	E_TNBC	AIN_META	E_TNBC	E_TNBC	NG_META	IFNB1_T	EAST_CAN	AST_CAN	LF4_TAR	AMPLICON	MPLICO	B_AMPLIC	AMPLIC
0.23284	0.2993	0.18305	0.24025	0.25235	-0.1335	0.19862	0.07119	0.20481	0.25372	0.17628	0.10755	0.18308	0.07955	0.0791
0.19171	0.31925	0.19496	0.24775	0.23926	-0.114	0.29086	0.09471	0.18676	0.25617	0.08009	0.03512	0.17281	0.09762	0.06059
0.19727	0.29269	0.23254	0.24532	0.30673	-0.16664	0.17503	0.18039	0.17779	0.22373	0.02788	-0.00219	0.19764	0.08119	0.03362
0.25646	0.33343	0.19842	0.18883	0.35791	-0.13553	0.23374	0.19346	0.17331	0.22469	0.07413	0.05072	0.20901	0.12422	0.02657
0.25109	0.32214	0.20578	0.22171	0.34805	-0.14254	0.1949	0.14221	0.13695	0.21982	0.05609	0.04135	0.23007	0.08242	0.0169
0.30588	0.34743	0.2342	0.21382	0.37962	-0.02678	0.24879	0.14186	0.15677	0.23646	0.02424	-0.04329	0.17453	0.14937	0.05622
0.28149	0.35869	0.26292	0.25329	0.41706	-0.05836	0.19001	0.23957	0.1686	0.25834	0.00426	0.06335	0.20422	0.10657	0.04398
0.27275	0.34263	0.18909	0.1751	0.37842	-0.12667	0.28552	0.1573	0.15339	0.2286	0.12444	-0.02107	0.22248	0.09699	0.04898
0.28191	0.36033	0.21551	0.19864	0.34396	-0.0388	0.209	0.22642	0.13152	0.23127	0.06343	-0.00585	0.23051	0.09422	0.07437
0.29717	0.39813	0.20571	0.19374	0.36207	-0.16477	0.16896	0.25144	0.15716	0.23268	0.09646	0.01446	0.22641	0.09515	0.03253
0.26591	0.30846	0.25286	0.29563	0.30843	-0.10006	0.25017	0.09895	0.15472	0.25008	0.09932	0.0243	0.16949	0.11058	0.03573
0.26307	0.32491	0.23222	0.23264	0.35641	-0.04892	0.28053	0.1921	0.13583	0.22408	0.06223	0.07518	0.19333	0.10553	0.06345
0.20075	0.31896	0.27208	0.3041	0.28705	-0.1538	0.3031	0.07784	0.18645	0.24099	0.13534	0.07194	0.09596	0.065	0.09078
0.24054	0.29382	0.20227	0.20232	0.33271	-0.20726	0.17155	0.11832	0.15821	0.23687	0.01255	0.04159	0.19128	0.11668	0.03802
0.29567	0.34408	0.18658	0.19318	0.3265	-0.13861	0.28807	0.15191	0.14321	0.24573	0.12298	0.03835	0.1961	0.1047	0.08005
0.16953	0.31944	0.22235	0.28651	0.25635	-0.05411	0.38957	0.09808	0.14479	0.2548	0.04076	0.01897	0.13518	0.09238	0.04454
0.22308	0.29122	0.18123	0.20364	0.34371	-0.08189	0.34511	0.09025	0.15458	0.23673	0.03177	0.07667	0.19858	0.10574	0.03351
0.22965	0.32034	0.2079	0.21633	0.34967	-0.18958	0.18643	0.22058	0.15921	0.2187	0.11577	-0.02765	0.22248	0.0739	0.01525

LIFIED_I	IED_IN_P	IED_IN_S	D_DURIN	HDAC_TAR	SILENC	SILENC	HDAC_TAR	ED_BY_M	ED_BY_M	SMARCA4	SMARCA4	MITOSIS	MITOSIS
-0.27057	0.06137	0.15113	0.16607	0.13402	0.09159	0.14373	0.12038	0.16801	0.08012	0.17887	0.13574	0.15366	0.01546
-0.24626	-0.0104	0.1252	0.12906	0.1453	0.0828	0.12602	0.13061	0.16497	0.07346	0.09992	0.11646	0.12956	-0.00617
-0.3685	0.04653	0.1581	0.111	0.16138	0.11026	0.12329	0.1119	0.18778	0.09515	0.13941	0.10639	0.16829	0.0461
-0.33239	0.02891	0.20993	0.18019	0.14609	0.10217	0.14266	0.13115	0.18059	0.13316	0.13531	0.15162	0.1688	0.12407
-0.0953	0.0459	0.1917	0.09421	0.15992	0.11504	0.13172	0.1258	0.181	0.13031	0.11208	0.06571	0.1023	0.05898
-0.49181	0.0261	0.21265	0.12632	0.15647	0.10636	0.1258	0.11536	0.18667	0.08107	0.17681	0.06495	0.16014	0.09128
-0.25806	0.02719	0.17908	0.15242	0.1655	0.11373	0.12542	0.11131	0.17539	0.11154	0.18966	0.07505	0.17241	0.13068
-0.32024	0.04383	0.20703	0.11901	0.15469	0.12235	0.13692	0.12863	0.20894	0.13835	0.13466	0.08248	0.15435	0.09677
-0.23216	-0.01774	0.21649	0.13576	0.15711	0.12674	0.1476	0.14088	0.19423	0.16395	0.15538	0.1074	0.17761	0.11727
-0.33715	0.02255	0.22998	0.13441	0.1598	0.12198	0.12268	0.11968	0.18699	0.1561	0.11747	0.0895	0.16576	0.12241
-0.24027	0.07518	0.10065	0.09094	0.1269	0.07846	0.14133	0.1223	0.17853	0.07026	0.17386	0.12237	0.18216	0.0225
-0.39827	0.05934	0.13608	0.12712	0.14157	0.10139	0.13405	0.11703	0.20358	0.09449	0.21781	0.14626	0.15902	0.05011
-0.34525	0.08309	0.15046	0.11293	0.10589	0.06032	0.122	0.11291	0.12944	0.0493	0.15036	0.10428	0.15693	-0.03742
-0.14409	0.07743	0.14603	0.11521	0.14328	0.11113	0.13988	0.13329	0.1856	0.10847	0.14799	0.09438	0.18141	0.04268
-0.36674	0.0272	0.17956	0.17009	0.1474	0.0998	0.13973	0.12031	0.20714	0.10743	0.17731	0.11992	0.18958	0.02852
-0.28317	0.05105	0.06519	0.0937	0.13075	0.06512	0.11622	0.10591	0.11926	0.04123	0.12768	0.13044	0.09463	-0.07785
-0.21684	0.03586	0.20151	0.17067	0.14828	0.10002	0.14437	0.12994	0.17105	0.0876	0.15401	0.13191	0.12958	0.05707
-0.1957	0.02079	0.19023	0.11498	0.14816	0.11099	0.13354	0.12368	0.17037	0.13687	0.12236	0.08441	0.11839	0.07656

MITOSIS	MITOSIS	ARREST	ARREST	ARREST	ARREST	PPRESS	OF_HOXA	OF_HOXA	N_TARGE	N_TARGE	HMGA1B	HMGA1	TARGETS
0.38635	-0.05308	0.12509	0.01954	0.22748	0.1301	0.08717	0.15065	0.11292	0.14237	-0.112	0.28404	0.29882	0.12686
0.33735	-0.09759	0.11466	0.00453	0.2538	0.04725	0.05698	0.08542	0.12417	0.16433	-0.09813	0.26615	0.27888	0.11904
0.38798	0.01634	0.10613	0.03227	0.23473	0.08796	0.07663	0.13574	0.19035	0.11035	-0.04233	0.27445	0.29734	0.12341
0.34105	0.05864	0.11537	0.12589	0.26373	0.1175	0.01946	0.2165	0.10951	0.09944	-0.02017	0.29294	0.30926	0.09877
0.26635	0.18472	0.06757	0.11214	0.16271	0.08739	0.02531	0.16771	0.12984	0.11785	-0.08996	0.2792	0.28749	0.09588
0.34873	0.0796	0.10671	0.1262	0.22478	0.12784	0.07706	0.07286	0.18731	0.10296	-0.07529	0.28866	0.30727	0.10135
0.32293	0.05386	0.11398	0.05355	0.24915	0.12154	0.01102	0.13234	0.17401	0.09014	-0.06157	0.28415	0.29799	0.1116
0.30207	0.06424	0.10095	0.13812	0.29156	0.06615	0.01708	0.23755	0.10499	0.10498	-0.07711	0.28995	0.29798	0.11119
0.26527	0.06723	0.07326	0.14576	0.25294	0.0565	0.04525	0.21932	0.10371	0.19362	-0.08285	0.28629	0.28882	0.12511
0.27909	0.04702	0.07195	0.10594	0.28514	0.08392	0.0087	0.21903	0.10808	0.09506	-0.11151	0.2848	0.29258	0.10591
0.35935	0.0758	0.14063	0.06992	0.13172	0.15525	0.05337	0.08998	0.20175	0.11283	-0.0649	0.27871	0.31	0.1326
0.33922	0.18518	0.10242	-0.00705	0.30762	0.17062	0.04871	0.09581	0.16853	0.11079	-0.07426	0.27979	0.30435	0.10972
0.34504	-0.10542	0.11333	-0.04079	0.1978	0.10263	0.06453	0.04785	0.15622	0.10155	-0.02441	0.27313	0.2943	0.08779
0.30232	0.08257	0.08479	0.0067	0.25656	0.09141	0.10794	0.16217	0.13658	0.16718	-0.09235	0.29332	0.29778	0.09968
0.37501	-0.04373	0.11888	0.004	0.24699	0.14186	0.06257	0.14237	0.17563	0.10884	-0.17818	0.28466	0.30571	0.12649
0.39112	-0.04549	0.13665	-0.02625	0.09962	0.05933	0.06919	0.03544	0.20847	0.08147	-0.00678	0.28207	0.30684	0.11791
0.33301	0.06456	0.06483	0.11627	0.26561	0.07035	0.0797	0.16494	0.11672	0.10774	0.02042	0.29939	0.3076	0.11233
0.2851	0.05174	0.07734	0.01637	0.21546	0.05228	0.005	0.16519	0.11718	0.09816	-0.10327	0.28592	0.29333	0.10291

RGETS_K	RGETS_K	ANSFORM	ANSFORM	PHOID_S	PHOID_S	D44_TARG	D44_TAR	44_TARGE	44_TARGE	NF1_TAR	NF1_TAR	TO_MATU	TO_MATU	MALL_P
0.05323	0.0892	0.10989	0.02803	0.19742	0.00189	0.19463	0.24421	0.00644	-0.2008	-0.0249	0.00789	0.02453	0.02761	0.0461
-0.05006	0.07329	0.09055	-0.00142	0.2006	-0.00644	0.16916	0.18533	-0.00522	-0.02417	-0.05107	-0.03584	0.06281	0.02123	0.06156
-0.01014	0.09474	0.11397	0.03903	0.20471	0.01165	0.20032	0.23552	0.04328	-0.08296	-0.01235	4.2E-05	0.01108	0.03422	0.02707
0.08351	0.0796	0.09755	0.04855	0.21116	0.0232	0.13581	0.2666	0.07439	-0.25604	-0.00982	0.02213	0.04388	0.09067	0.05673
-0.12336	0.07077	0.07526	0.0533	0.1702	0.04667	0.12491	0.23372	0.11595	-0.20504	0.0181	0.00761	0.03172	0.07315	0.066
-0.00887	0.06247	0.0831	0.02685	0.19698	0.02323	0.14372	0.28373	0.10876	-0.05508	0.08515	-0.01231	0.02131	0.01791	0.06828
0.03351	0.10746	0.1169	0.06123	0.17246	0.01235	0.13737	0.29736	0.0852	-0.16543	0.09677	0.02294	0.0495	0.04923	0.06759
-0.01954	0.1028	0.10887	0.08235	0.2041	0.04707	0.05845	0.30136	0.13359	-0.22624	0.01047	0.03872	0.0652	0.07739	0.08225
-0.01403	0.12002	0.09342	0.07961	0.1996	0.05782	0.11162	0.29387	0.12187	-0.17052	0.05488	0.00891	0.05462	0.13403	0.08639
-0.03935	0.11674	0.08429	0.06687	0.19395	0.05031	0.13978	0.31842	0.12373	-0.18468	0.04786	-0.00147	0.03957	0.07017	0.07729
0.13976	0.10031	0.10455	0.03865	0.20642	-0.03753	0.17601	0.20174	0.04243	-0.01484	0.00461	-0.01323	-0.0172	-0.05533	-0.02237
0.05513	0.09678	0.10736	0.058	0.18541	0.00321	0.13739	0.26512	0.11773	-0.11383	0.08036	0.05173	0.00125	0.01933	0.05
0.20122	0.07925	0.10881	0.01054	0.22388	-0.00652	0.25138	0.12465	-0.03149	-0.08979	-0.00147	-0.00049	0.02401	-0.04208	0.00915
0.10007	0.07806	0.08749	0.04936	0.19699	-0.00197	0.12689	0.24171	0.08503	-0.25473	0.01727	0.03211	0.05133	0.07153	0.02829
0.00587	0.10333	0.12691	0.04572	0.20447	-0.00929	0.14996	0.25603	0.09296	-0.14432	-0.01473	0.00907	0.03848	0.02307	0.02691
-0.00852	0.07565	0.08765	0.01977	0.21752	-0.01158	0.1601	0.1394	-0.02567	0.01705	-0.05068	-0.00672	0.04165	-0.0294	0.00949
0.09415	0.08916	0.099	0.033	0.20838	0.02083	0.23596	0.23338	0.07435	-0.22696	-0.01898	0.0027	0.01723	0.02738	0.0638
-0.03526	0.10413	0.09163	0.06343	0.17757	0.01313	0.12911	0.23102	0.12614	-0.16576	-0.01377	0.01604	0.01326	0.09987	0.06039

MALL_PR	ARGE_PI	ARGE_PI	_TO_IMM	_TO_IMM	CLOCK_T	CLOCK_T	ELL_LY	ELL_LY	ASTIC_SY	ASTIC_SY	ASTIC_SY	ASTIC_SY	PLASTIC
0.11977	0.14329	0.10508	0.17226	0.15259	0.03243	0.29617	-0.01151	0.04855	0.12862	-0.10389	0.30037	0.01152	0.12856
0.14906	0.16049	0.10418	0.20698	0.0902	0.04137	0.3145	-0.06209	0.00205	-0.14932	-0.04635	0.30373	-0.0808	0.05966
0.13414	0.174	0.07648	0.14657	0.15774	0.07989	0.35491	-0.00644	0.06965	-0.05133	-0.23912	0.30398	-0.09942	0.13465
0.11998	0.14338	0.07689	0.14797	0.18532	0.06721	0.38287	-0.02351	0.01829	-0.02227	-0.19098	0.31022	-0.06966	0.14596
0.11965	0.17022	0.12515	0.16018	0.17202	0.11444	0.33702	-0.04004	0.05216	0.0175	-0.15348	0.28624	-0.08028	0.16736
0.14315	0.17745	0.00256	0.14032	0.1467	-0.01083	0.33242	-0.02855	0.05468	0.04424	-0.12424	0.25967	-0.10917	0.09908
0.12493	0.13295	-0.02127	0.13588	0.16263	0.12261	0.37269	0.00442	0.07118	0.06924	-0.15904	0.27239	-0.06178	0.13787
0.09903	0.13437	0.0396	0.14789	0.19019	0.08585	0.36306	-0.04648	0.03381	0.09742	-0.21544	0.29893	-0.09602	0.17148
0.09916	0.15832	0.01195	0.16716	0.20729	0.07634	0.38744	-0.08628	0.05081	0.03485	-0.19463	0.29465	-0.0902	0.17505
0.11362	0.13253	0.04026	0.14898	0.19515	0.10265	0.37431	-0.00382	0.06459	0.18564	-0.14378	0.28271	-0.06998	0.18739
0.15756	0.17572	0.04265	0.14094	0.13146	0.09398	0.29522	-0.07858	0.0508	-0.0126	-0.17356	0.32142	-0.03502	0.0616
0.13128	0.16997	0.05149	0.14823	0.14577	0.06693	0.29632	-0.02583	0.04837	-0.0685	-0.14011	0.30535	-0.05355	0.02934
0.14837	0.1566	0.09867	0.17255	0.10708	0.13031	0.33388	-0.01852	0.06037	-0.11781	-0.13517	0.31252	-0.03706	0.01253
0.10706	0.1521	0.07648	0.16033	0.19413	0.16854	0.32718	-0.08854	0.01942	-0.04957	-0.1701	0.32717	-0.10114	0.13807
0.14547	0.1483	0.08838	0.13248	0.1859	0.12204	0.28908	-0.0611	0.01695	0.11957	-0.16318	0.31146	-0.08291	0.14448
0.1429	0.19047	0.06979	0.14904	0.10267	0.04408	0.32507	-0.073	0.05795	-0.19737	-0.09775	0.27839	-0.07388	-0.01437
0.11191	0.1754	0.09424	0.12276	0.17989	-0.00592	0.33036	-0.07251	0.02275	-0.02201	-0.18091	0.30278	-0.09146	0.11254
0.10544	0.17506	0.07161	0.17074	0.17913	-0.04866	0.29612	-0.03453	0.04517	0.05746	-0.19359	0.30847	-0.11733	0.16302

PLASTIC	INASE_R	INASE_R	INASE_RE	INASE_RE	UNORUB	UNORUB	UNORUB	UNORUB	OLONE_	OLONE_	LONE_R	LONE_R	STINE_RE	STINE_RE
-0.01063	0.12034	0.18767	0.06243	0.29654	0.06209	-0.0712	0.06982	0.25261	0.07784	0.18044	0.32795	0.18938	0.36746	-0.01368
0.01855	0.12465	0.1857	0.04068	0.30614	0.14733	-0.01155	0.14456	0.28701	0.06732	0.21952	0.28338	0.23155	0.39638	-0.01493
0.04873	0.09364	0.17507	0.01259	0.29836	0.03644	0.07464	0.0625	0.26063	0.16421	0.17332	0.34073	0.18868	0.38604	0.03494
0.02875	0.15311	0.18056	0.08736	0.3083	-0.00206	-0.04724	0.1061	0.23659	0.16653	0.1463	0.35377	0.15631	0.38136	-0.01438
-0.02358	0.12793	0.1789	0.04088	0.2952	0.07808	0.09596	0.11811	0.33235	0.13627	0.18351	0.33708	0.18609	0.38756	-0.02003
0.0349	0.09587	0.18812	-0.00426	0.30795	-0.00336	-0.06045	0.06696	0.23618	0.11241	0.17325	0.29741	0.17867	0.39272	-0.02917
0.02444	0.1142	0.17778	0.01568	0.31475	-0.01226	-0.00263	0.12091	0.23526	0.11856	0.17319	0.3126	0.18416	0.39383	-0.03396
-0.01879	0.14775	0.17708	0.0515	0.29607	-0.02283	-0.004	0.13568	0.21145	0.20427	0.14631	0.3527	0.1401	0.3776	-0.01155
-0.03525	0.15466	0.18389	0.08198	0.29581	-0.00237	0.00249	0.08885	0.22337	0.2159	0.15376	0.36906	0.15045	0.38816	-0.01129
-0.04415	0.14443	0.18065	0.08009	0.29683	0.04791	-0.04765	0.11941	0.21998	0.1724	0.16184	0.33779	0.16927	0.38237	-0.02007
0.04185	0.08055	0.16401	0.01486	0.30336	0.04113	0.02005	0.12515	0.25948	0.12778	0.15804	0.3247	0.14731	0.36719	-0.01616
0.01568	0.11081	0.19473	0.01724	0.3023	-0.00432	-0.02688	0.1205	0.27952	0.10367	0.16062	0.2938	0.16776	0.38171	-0.00663
0.04799	0.10017	0.18313	0.03686	0.2924	0.08345	0.03489	0.10227	0.29102	0.0458	0.21754	0.30313	0.21103	0.35762	0.01988
0.08058	0.13357	0.18886	0.09241	0.30836	0.08917	0.05868	0.15913	0.22511	0.18972	0.15956	0.34409	0.16327	0.38418	-0.0089
0.02646	0.12069	0.20527	0.07079	0.31374	0.0789	-0.00755	0.10721	0.26628	0.11034	0.17465	0.32939	0.17348	0.38131	-0.00106
0.0105	0.06216	0.19809	0.01299	0.31129	0.14747	0.05767	0.09183	0.32042	0.01999	0.18683	0.27798	0.17576	0.37336	-0.00309
0.02201	0.13321	0.1952	0.05891	0.31309	0.00578	-0.03676	0.10674	0.25661	0.12063	0.19624	0.32095	0.20004	0.39079	-0.06449
0.01759	0.1467	0.16513	0.0889	0.29173	-0.01548	-0.07563	0.11704	0.18846	0.16718	0.18902	0.35994	0.17956	0.38579	-0.01452

STINE_RE	STINE_RES	OEPITHE	IT_BREA	IT_BREA	ACINAR_B	CINAR_B	ILLARY_B	ODULAR_	ODULAR_	AMOUS_	POPTOSIS	OPTOSIS	ETAXEL_I	CANCER
0.40941	-0.06298	-0.12173	0.06389	0.01344	-0.01954	0.02857	0.07904	-0.10817	-0.03911	0.07816	0.13485	0.10742	0.30151	0.11304
0.42556	-0.0576	-0.10577	-0.02213	0.03193	0.03419	0.02675	0.05723	-0.1363	-0.36991	-0.00181	0.12299	0.10855	0.28302	0.21195
0.415	-0.05336	-0.05037	0.02587	0.05158	0.01925	0.01825	0.07854	-0.11489	-0.1344	-0.0241	0.12457	0.1272	0.28428	0.18809
0.42023	-0.12777	-0.00351	-0.02162	0.06807	-0.05203	0.04253	0.09705	-0.13662	-0.12066	0.06934	0.13249	0.12893	0.29792	0.13355
0.40366	-0.05794	-0.09125	-0.05955	0.06286	-0.12907	0.05796	0.05789	-0.13909	-0.33838	-0.04989	0.13063	0.13382	0.283	0.1455
0.42374	-0.0888	0.03016	-0.02353	0.01334	-0.10257	0.08698	0.11588	-0.15162	-0.19253	-0.0287	0.12692	0.12189	0.29433	0.20203
0.42533	-0.09999	0.04129	-0.03882	0.04608	0.05218	0.00387	0.09094	-0.14551	-0.46257	-0.04785	0.14063	0.1204	0.2885	0.15058
0.41062	-0.11735	-0.00991	-0.12648	0.12934	-0.05788	0.04441	0.19408	-0.03809	-0.21261	-0.01625	0.13791	0.13167	0.28116	0.09838
0.40777	-0.06504	0.01243	-0.11948	0.12101	-0.10859	0.04538	0.11171	-0.0837	-0.06306	0.00068	0.14454	0.12606	0.28367	0.12168
0.39169	-0.10137	-0.04527	-0.08804	0.09728	-0.1255	0.0488	0.10044	-0.11479	-0.36762	-0.00255	0.15196	0.13137	0.27923	0.1085
0.40414	-0.06592	-0.05697	0.02002	-0.04496	-0.10092	-0.03075	0.14842	-0.08019	-0.09142	0.06158	0.11305	0.11099	0.29425	0.28678
0.41689	-0.0769	-0.11891	-0.00521	0.0085	-0.07624	0.04067	0.14265	-0.10319	-0.3486	0.00197	0.1375	0.10249	0.30072	0.21765
0.39496	-0.02318	0.06541	0.08648	-0.04445	-0.02773	-0.02253	0.07251	-0.15684	-0.32024	0.04919	0.10744	0.11626	0.29044	0.22813
0.41064	-0.06823	-0.07583	-0.00613	0.05205	-0.03589	0.01423	0.10153	-0.12882	-0.3264	0.04715	0.13119	0.11892	0.28032	0.10509
0.42	-0.06488	-0.04777	0.00172	0.02982	-0.01484	0.02824	0.19327	-0.08142	-0.40655	0.05657	0.13873	0.1138	0.28291	0.12137
0.39474	-0.05474	-0.02516	0.09863	-0.08904	-0.01547	-0.00558	0.05101	-0.17534	-0.11503	0.03774	0.12766	0.10454	0.27986	0.24952
0.42041	-0.12084	-0.06314	0.02488	0.03798	-0.07381	0.02802	0.06455	-0.11829	-0.04597	0.05564	0.12681	0.13244	0.31001	0.10911
0.41145	-0.0653	-0.02091	-0.05649	0.06201	0.00967	0.03242	0.09759	-0.09658	-0.07839	0.02727	0.13847	0.13055	0.28846	0.15657

T7_TARG	T7_TARG	HYLATED	WTAP_TA	WTAP_TA	SREBF_	CER_LAT	CER_LA	_CANCER	_CANCER	_CANCER	_CANCER	_CANCER	OUSEKEE
0.05945	-0.01438	0.11253	0.10739	0.01079	0.00198	0.10733	0.10425	0.18327	0.18485	0.11527	0.08529	0.10113	0.14782
0.02883	-0.0075	0.13044	0.12717	-0.01719	-0.03139	0.10062	0.12572	0.17368	0.21461	0.07057	0.06626	0.11612	0.16862
0.03147	0.02089	0.12163	0.12023	0.02719	-0.01585	0.12156	0.14943	0.19588	0.20633	0.12352	0.06998	0.14442	0.14653
0.04239	-0.04394	0.16515	0.10701	0.05242	-0.0468	0.08836	0.12043	0.22413	0.17624	0.15081	0.0789	0.09529	0.13672
-0.00496	-0.05543	0.1025	0.10146	0.02838	-0.01788	0.12879	0.10064	0.20652	0.18445	0.11731	0.07278	0.09379	0.14336
0.0315	-0.0213	0.10223	0.12723	0.00047	-0.022	0.07117	0.14504	0.18985	0.20491	0.16336	0.10676	0.07917	0.14579
0.03547	0.02316	0.12059	0.10266	0.0351	-0.01824	0.08519	0.1452	0.20621	0.19142	0.15297	0.10961	0.10726	0.13905
0.02769	-0.03207	0.13874	0.09821	0.05975	-0.0155	0.10865	0.10357	0.22656	0.15797	0.16112	0.08519	0.1155	0.12885
0.01956	-0.01543	0.17552	0.09961	0.06862	-0.0065	0.08049	0.11541	0.23225	0.16513	0.15325	0.08952	0.14234	0.12609
0.01347	-0.07021	0.10665	0.0996	0.06806	-0.01901	0.08593	0.1451	0.2278	0.16925	0.14685	0.1074	0.11008	0.12954
0.02542	0.02943	0.10973	0.12731	0.01956	-0.03957	0.12212	0.12467	0.14778	0.18902	0.1374	0.10581	0.07448	0.15458
0.06969	-0.00042	0.12342	0.1172	0.0217	-0.02529	0.10618	0.14781	0.17836	0.19634	0.14473	0.12503	0.10692	0.14498
0.02925	-0.02987	0.10218	0.11669	-0.01642	0.07152	0.09195	0.10825	0.107	0.20198	0.10406	0.09246	0.0544	0.16773
0.02257	-0.02651	0.11358	0.10892	0.03275	-0.03476	0.10821	0.09845	0.19865	0.18354	0.14637	0.09525	0.09699	0.14782
0.01272	-0.01599	0.15929	0.1224	0.02937	0.02451	0.12135	0.1317	0.18195	0.18198	0.14031	0.09253	0.09331	0.14905
0.01763	0.01235	0.07789	0.1235	0.01884	0.02239	0.12021	0.11794	0.09257	0.1968	0.10866	0.09749	0.10696	0.15835
0.03333	-0.01655	0.16893	0.10707	0.00684	-0.02366	0.11945	0.1077	0.20517	0.19899	0.12939	0.06783	0.11999	0.14823
0.01777	-0.05154	0.14081	0.10396	0.06908	-0.0383	0.09608	0.12483	0.21647	0.15726	0.14184	0.06761	0.11795	0.13204

DUSTIS_R	TED1_TA	TED1_TA	TED1_TA	TED1_TAR	RTAL_MA	ER_VASC	SEKEEP	R_SPECI	FINIB_RE	TINIB_SE	OXA2_TA	OXA2_TA
0.2389	0.09146	0.07387	0.08265	0.16107	0.12967	0.00937	0.3394	0.08684	0.03602	0.11503	0.03183	0.10528
0.23854	0.0828	0.12355	0.09556	0.11409	0.08259	0.02637	0.34632	0.05109	0.03761	0.05762	-0.03249	0.08346
0.25068	0.08408	0.11682	0.13901	0.12942	0.13532	0.00878	0.33626	0.11101	0.00874	0.07486	0.02248	0.11491
0.26693	0.09948	0.1779	0.13818	0.22067	0.18993	0.01113	0.34538	0.17506	-0.05058	0.1174	0.04442	0.10266
0.24524	0.03373	0.15339	0.15519	0.14707	0.15319	0.05016	0.34347	0.10448	-0.0275	0.08053	-0.03446	0.06161
0.24409	0.06824	0.12112	0.1409	0.18225	0.11998	0.00584	0.34029	0.22562	-0.00429	0.0819	0.04292	0.16098
0.25941	0.04981	0.12815	0.06461	0.1453	0.13897	0.02434	0.3384	0.1915	0.01197	0.08254	0.13378	0.12328
0.27269	0.06422	0.12048	0.13529	0.12481	0.20172	0.06444	0.34166	0.18087	-0.08407	0.13583	0.065	0.09529
0.25799	0.10722	0.10912	0.14827	0.17397	0.22489	0.05598	0.34071	0.18272	-0.07778	0.12162	0.0482	0.0789
0.27329	0.02879	0.09834	0.12321	0.14596	0.18774	0.04011	0.33691	0.1779	-0.03759	0.09973	0.03527	0.06278
0.23141	0.10548	0.08861	0.05761	0.14561	0.08058	-0.0183	0.3358	0.13514	0.01804	0.06703	0.00265	0.11571
0.25819	0.10558	0.076	0.09667	0.14662	0.13444	0.00552	0.34496	0.17363	-0.00928	0.12393	0.07001	0.18477
0.22268	0.08901	0.07009	0.06645	0.16705	0.0465	0.06182	0.33558	0.1111	0.05523	0.04421	-0.02023	0.12831
0.25884	0.07618	0.09349	0.11402	0.19752	0.16877	0.03813	0.3426	0.1384	-0.00213	0.07712	0.04831	0.06702
0.2459	0.1034	0.12822	0.13339	0.11434	0.09652	0.0223	0.34243	0.14506	-0.02518	0.07256	0.02422	0.11246
0.2307	0.0974	0.13473	0.11029	0.0921	0.01999	-0.03787	0.33241	0.06014	0.08003	0.06131	-0.04668	0.07323
0.24596	0.06229	0.12492	0.07861	0.12184	0.14427	0.00101	0.35208	0.11712	0.02834	0.05555	0.04983	0.11323
0.24524	0.07105	0.14269	0.12181	0.19107	0.168	0.05659	0.34439	0.1247	-0.05391	0.13988	0.02306	0.0777

RKITTS_L	ENIGN_S	ENIGN_S	LIGNANT_	LIGNANT_	CANCER_	CANCER_	RCISE_R	BASAL_V	BASAL_V	LL_POOR	LL_POOR	IOGENE	IOGENE	OXIC_DA	OXIC_DA	N_DIREC	ON_DIRE	ATE_CAN	IM_NRF1	HIM_NRF
0.24411	0.08471	0.23299	-0.04332	0.28595	0.09732	0.16732	0.18575	0.20851	0.08554	0.03969	0.1105	0.29795	0.06472	0.16377	0.0448	0.19353	0.08141	0.33638	0.00526	0.14402
0.31363	0.18269	0.19568	0.01471	0.19753	0.10554	0.14993	0.10145	0.18766	0.01526	-0.03364	0.09018	0.26303	0.02348	0.18619	0.03345	0.212	0.1601	0.30694	0.00683	0.15154
0.26065	0.16963	0.17739	0.01598	0.25147	0.11518	0.17497	0.17334	0.19105	0.00113	0.08242	0.09062	0.26073	-0.00312	0.22109	0.06415	0.19412	0.12011	0.28811	0.00278	0.14849
0.21664	0.13898	0.24617	-0.02195	0.30801	0.1163	0.20912	0.1748	0.20981	0.08198	-0.01439	0.13315	0.25503	0.0833	0.18146	0.04958	0.18004	0.1477	0.32212	-0.01833	0.14268
0.25945	0.12872	0.2255	0.07128	0.31542	0.08978	0.17399	0.19035	0.16088	-0.01585	-0.00132	0.13568	0.27268	-0.0525	0.16102	0.02474	0.18451	0.17	0.2956	0.01081	0.14065
0.24075	0.21468	0.24356	0.13974	0.29835	0.10597	0.1437	0.13967	0.15992	-0.02157	0.03935	0.10094	0.28329	0.02756	0.17659	0.0725	0.1995	0.10301	0.34378	-0.01253	0.14657
0.21024	0.27249	0.22345	0.08488	0.29735	0.12833	0.19974	0.16565	0.17973	0.00191	0.01509	0.13244	0.28332	0.0393	0.22804	0.06981	0.18354	0.09442	0.30862	0.04696	0.14065
0.21621	0.14529	0.24391	0.05348	0.31891	0.11263	0.1932	0.16532	0.15605	0.00414	0.00076	0.12852	0.24025	0.02319	0.18205	-0.00905	0.16813	0.13885	0.29936	-0.01205	0.14723
0.23889	0.14131	0.2546	-0.01843	0.33545	0.10673	0.1989	0.16185	0.20129	-0.01463	0.00338	0.13548	0.24552	0.029	0.19228	-0.00025	0.1689	0.11368	0.28644	0.00071	0.13474
0.23384	0.13163	0.25703	-0.00062	0.31071	0.11605	0.1944	0.16128	0.17252	-0.02565	-0.0132	0.13689	0.22984	0.00728	0.17414	0.03616	0.1769	0.16339	0.30542	-0.01231	0.13652
0.2719	0.14338	0.20254	0.01322	0.25354	0.12112	0.13765	0.12969	0.21508	0.06651	-0.01065	0.07208	0.28047	0.02948	0.22224	0.08616	0.19056	0.0978	0.33725	-0.00416	0.14774
0.23567	0.14302	0.23894	-0.0004	0.28636	0.10638	0.1597	0.20405	0.24877	0.06119	0.03937	0.10907	0.27852	0.09093	0.20434	0.04898	0.19204	0.10104	0.34056	0.04035	0.14226
0.21888	0.20631	0.17224	0.028	0.17243	0.10881	0.11302	0.13136	0.18001	0.0766	0.0186	0.07196	0.27688	0.00522	0.15956	0.05091	0.20087	0.12608	0.32079	0.02132	0.14676
0.26364	0.15246	0.21241	-0.00353	0.28449	0.11632	0.18666	0.17555	0.17284	0.09313	-0.00265	0.11348	0.24021	-0.01377	0.22542	0.00747	0.18809	0.15424	0.30037	0.01446	0.15166
0.244	0.13972	0.23176	-0.03738	0.27105	0.10898	0.16195	0.17774	0.23375	0.10269	0.02187	0.08992	0.26758	0.0078	0.19792	0.01763	0.19185	0.14466	0.31557	0.00139	0.15661
0.29431	0.21897	0.12687	0.02151	0.14557	0.10191	0.11858	0.08986	0.19735	0.05911	0.03628	0.06081	0.27795	0.06105	0.18351	0.07334	0.22071	0.13585	0.3205	0.05089	0.14545
0.21099	0.14524	0.2504	-0.01293	0.30369	0.12577	0.18394	0.18682	0.15838	0.05765	0.01576	0.12285	0.27985	0.06504	0.18682	0.0157	0.17116	0.12761	0.30778	-0.00606	0.14225
0.22744	0.14537	0.23495	0.03507	0.26411	0.11418	0.16822	0.16821	0.16752	-0.00139	-0.01264	0.13279	0.25472	0.00267	0.18541	0.00613	0.17343	0.13315	0.31177	0.01347	0.1373

IM_NRF2	HIM_NRF	M_NRF3	HIM_NRF	SUS_HOS	SUS_HOS	SUS_HOS	SUS_HO	ATF4_TAR	ATF4_TAR	E2F_TAR	E2F_TAR	CER_EA	ER_PROG	ER_PROG	ER_PROG
-0.01117	0.11781	-0.02731	0.18162	0.00106	0.19526	0.07899	0.15401	0.07039	-0.18611	0.1318	0.16692	0.18604	0.19983	0.30346	0.19078
-0.02165	0.10263	0.08403	0.17718	0.01696	0.14341	0.10769	0.13132	-0.00798	-0.17968	0.04258	0.17761	0.15881	0.19469	0.233	0.15822
-0.04476	0.12047	0.12848	0.15201	0.02416	0.22355	0.07465	0.18865	0.05265	-0.04729	0.06219	0.19891	0.19578	0.19383	0.34551	0.12819
-0.01983	0.10538	0.09043	0.19426	0.05684	0.27927	0.07777	0.22925	0.04027	-0.26039	0.18837	0.21816	0.24282	0.18992	0.32841	0.1991
-0.01757	0.10611	0.11757	0.24195	0.01297	0.25716	0.09645	0.21367	0.06301	-0.23629	0.13593	0.18784	0.23449	0.17887	0.29207	0.15518
-0.05136	0.1124	0.07837	0.16864	0.09396	0.21793	0.12692	0.18782	0.06532	-0.2613	0.12553	0.22273	0.19125	0.1785	0.29265	0.10369
-0.00429	0.10143	0.0825	0.09774	0.04876	0.23324	0.16943	0.19622	0.0665	-0.09188	0.08598	0.22906	0.20794	0.16664	0.364	0.1688
-0.01573	0.11922	0.10838	0.2268	0.02021	0.29861	0.10795	0.24249	0.08282	-0.27197	0.15775	0.21335	0.23361	0.18166	0.3352	0.17
-0.02054	0.10588	0.07455	0.17319	0.04646	0.2915	0.12508	0.26666	0.06241	-0.36379	0.129	0.22513	0.26825	0.17842	0.35836	0.14139
-0.02041	0.09921	0.10433	0.17847	0.08944	0.28567	0.12392	0.25163	0.04809	-0.18391	0.24897	0.19911	0.29384	0.16951	0.35449	0.18521
-0.02696	0.12403	0.06726	0.12559	0.01906	0.17784	0.1036	0.12567	0.07013	-0.10682	0.06192	0.19299	0.14895	0.19231	0.20214	0.06364
-0.00534	0.1128	0.11086	0.16883	0.01628	0.20467	0.08876	0.17861	0.07027	-0.04948	0.1272	0.20631	0.20057	0.20259	0.32982	0.07987
0.00645	0.10921	0.05049	0.06262	-0.01089	0.08331	0.07442	0.09145	0.07371	-0.29303	-0.01425	0.18922	0.14953	0.20934	0.21257	0.00975
-0.01535	0.11684	0.0872	0.20787	0.06074	0.24546	0.09183	0.20477	0.06387	-0.08964	0.03947	0.19769	0.22444	0.18504	0.27673	0.1757
-0.03393	0.12548	0.14325	0.17331	0.0537	0.22502	0.10728	0.17338	0.06357	-0.00076	0.13461	0.19544	0.20888	0.20604	0.31198	0.14137
0.01755	0.11345	0.05634	0.01496	0.04223	0.08153	0.11907	0.09491	0.05149	-0.00521	-0.05258	0.17297	0.16969	0.18016	0.32653	0.01726
-0.01742	0.10388	0.03575	0.17526	0.03026	0.21708	0.07814	0.16936	0.0331	-0.18905	0.1573	0.18651	0.188	0.18274	0.24012	0.18806
-0.01271	0.11043	0.02834	0.19519	0.06853	0.26481	0.08217	0.22947	0.04697	-0.19168	0.11554	0.21027	0.22118	0.16731	0.34177	0.18101

ER_PROG	ER_PROG	ER_PROG	ER_PROG	ER_PROG	R133_TAR	R133_TAR	IR1_TARG	IR1_TARG	R30_TAR	R30_TAR	EBF1A_TA	NG_CAN	CANCER_S	NG_CANC	TP53_TAR
0.05851	0.21491	0.21506	0.23743	0.01868	-0.00302	0.16167	0.09379	0.23617	0.03498	0.03754	0.21614	-0.04551	0.14104	0.07967	-0.02174
0.00867	0.21903	0.20131	0.23588	-0.17005	-0.06925	0.15909	0.09573	0.2394	-0.0182	0.03465	0.10939	0.1038	0.09095	0.03698	-0.0186
0.06063	0.25139	0.20015	0.23275	-0.10313	-0.03378	0.16662	0.06736	0.22962	0.0043	0.06813	-0.03083	0.04382	0.12618	0.02042	0.00045
0.1013	0.27268	0.23917	0.22316	-0.03816	0.01652	0.17222	0.09612	0.23833	0.04348	0.05151	0.21525	-0.01865	0.10148	-0.00156	0.09082
0.05859	0.31406	0.18623	0.21904	-0.11727	-0.07367	0.15389	0.08166	0.21826	0.0391	0.03245	0.21948	-0.00977	0.09877	-0.12776	-0.04217
0.15657	0.23899	0.12039	0.20961	0.00651	-0.23116	0.15491	0.09694	0.23854	0.00514	0.03172	0.22882	0.06467	0.13197	-0.0458	0.09546
0.11125	0.25906	0.14845	0.26233	0.0264	-0.03624	0.14871	0.10899	0.21038	-0.02445	0.01097	0.30403	0.07461	0.10025	-0.09398	0.07741
0.10888	0.28728	0.1533	0.21113	-0.0393	0.03529	0.18422	0.14313	0.23841	0.0551	0.03925	0.23252	0.00531	0.06744	0.04888	0.04815
0.07943	0.31075	0.15788	0.20914	-0.04321	0.02594	0.1663	0.0984	0.23188	0.06454	0.04865	0.26317	-0.00155	0.08175	0.02413	0.02195
0.12806	0.30727	0.08542	0.20203	-0.05326	-0.03247	0.16756	0.08584	0.22103	0.02965	0.04289	0.25683	0.02147	0.08005	-0.00299	0.00854
0.08215	0.21884	0.24816	0.19441	-0.04932	0.06087	0.16965	0.07171	0.23968	0.02092	0.03113	0.17967	0.02253	0.11466	0.12792	0.11414
0.08095	0.27773	0.23674	0.23886	-0.04429	0.05528	0.16443	0.19256	0.23041	0.06549	0.03002	0.19165	-0.06302	0.11098	-0.03177	0.13415
0.06044	0.15797	0.16706	0.22038	-0.11654	-0.08072	0.15249	0.09061	0.22966	-0.02909	0.01892	0.30403	0.07675	0.10347	-0.01431	0.04272
0.10769	0.27309	0.16866	0.22946	-0.06028	0.07388	0.17506	0.0758	0.23754	0.00782	0.04196	0.1913	0.08342	0.05032	-0.00725	0.03845
0.07262	0.22605	0.22381	0.24716	-0.0495	0.02102	0.17197	0.08902	0.25147	-0.00764	0.02588	0.2563	0.02528	0.07622	0.08359	0.09702
0.07922	0.17431	0.15878	0.21415	-0.10751	-0.02061	0.15698	0.11737	0.22104	-0.00295	-0.02245	0.20539	0.11889	0.14163	0.05035	0.05714
0.07681	0.25607	0.14228	0.21483	-0.07976	0.00597	0.19451	0.09268	0.23674	0.02419	0.04276	0.18689	0.03128	0.10113	0.03774	0.00488
0.09756	0.30989	0.14657	0.20315	-0.07412	0.00914	0.17794	0.11472	0.23923	0.03405	0.0456	0.26722	0.08134	0.08814	0.08768	-0.00343

SHH_TAR	SHH_TAR	AD1_TAR	AD1_TAR	E2F_TAR	S_OF_SY	STING_SA	MLL2_T	G1_TARG	G1_TARG	OIESIS_E	IS_INTER	OIESIS_L	OPOIESIS	TOPOIESIS	SIS_STEM	ESIS_STE
0.07779	0.0573	0.26036	-0.04249	-0.03456	-0.00623	0.01107	0.12288	0.24146	0.17841	0.06333	0.0947	0.07189	0.01104	-0.05544	-0.00637	0.0216
0.07726	0.02843	0.27562	-0.03975	0.04492	0.12219	0.06584	0.15796	0.28358	0.15138	0.06797	0.09493	0.05673	-0.00245	-0.04827	0.00568	0.0516
0.07201	0.0411	0.28264	0.06211	0.07337	0.10984	0.02016	0.17324	0.29295	0.1891	0.0841	0.10423	0.07349	0.03781	-0.08113	-0.02125	0.054
0.1077	0.05877	0.2688	0.022	0.00517	0.09274	-0.00417	0.1596	0.33848	0.15698	0.05476	0.09297	0.09253	0.07069	-0.08333	-0.02911	0.03
0.05239	0.04584	0.26444	-0.0151	0.00508	0.12765	-0.00519	0.13686	0.36029	0.17705	0.06813	0.10686	0.0766	0.06904	-0.05866	-0.00751	0.03651
0.08078	0.06682	0.28753	-0.02381	0.07254	0.11311	0.08093	0.13874	0.23703	0.23934	0.05969	0.07887	0.06316	0.05216	-0.06814	-0.01141	-0.01889
0.09883	0.05996	0.3006	0.01918	0.02583	0.14845	0.05841	0.13583	0.25946	0.16878	0.06352	0.10401	0.06322	0.03672	-0.08128	-0.01231	0.00518
0.09074	0.06938	0.24999	0.08487	-0.04607	0.12824	0.02305	0.13776	0.34089	0.18048	0.05572	0.10155	0.1002	0.09684	-0.10007	-0.03725	0.02026
0.10528	0.07144	0.23998	0.04139	-0.03315	0.12823	0.0104	0.12758	0.34449	0.25621	0.04547	0.0966	0.09704	0.08303	-0.08791	-0.03287	0.04333
0.07273	0.08011	0.25367	0.00497	-0.04103	0.13403	0.0532	0.10902	0.31002	0.16745	0.05313	0.1016	0.09287	0.0784	-0.09079	-0.01356	-0.01412
0.0911	0.02741	0.30649	0.0707	0.09878	0.15325	-0.02904	0.16515	0.25129	0.13212	0.07938	0.097	0.06495	0.02911	-0.09108	-0.01121	0.01113
0.12448	0.05983	0.28148	-0.01429	0.05681	0.23142	0.02612	0.18487	0.23626	0.18984	0.07886	0.09237	0.07108	0.03256	-0.06747	-0.00265	-0.0053
0.08614	0.02657	0.2919	0.08606	0.05705	0.13524	0.0898	0.14021	0.28726	0.09367	0.073	0.07745	0.0553	-0.01483	-0.06615	0.00219	0.04513
0.11517	0.03741	0.25307	-0.00974	-0.06216	0.13433	-0.03841	0.14921	0.31894	0.18605	0.05357	0.10973	0.07995	0.05149	-0.08062	-0.0293	0.02817
0.09716	0.04914	0.31113	-0.0391	0.03405	0.0666	0.01849	0.13254	0.26456	0.17007	0.0719	0.09482	0.07456	0.04426	-0.09445	-0.02245	0.03916
0.06658	0.00774	0.32806	-0.0586	0.07919	0.02772	-0.04434	0.18652	0.18986	0.07294	0.0717	0.11507	0.06174	-0.02403	-0.06659	0.00298	0.04601
0.104	0.0533	0.27779	0.09577	-0.05811	0.10028	-0.01256	0.16354	0.31779	0.15404	0.07268	0.09335	0.07006	0.02049	-0.08799	-0.01557	0.04364
0.10735	0.05117	0.25232	0.11428	-0.03024	0.12159	0.02369	0.16943	0.30312	0.15969	0.06147	0.10357	0.08543	0.07002	-0.08927	-0.02904	0.03264

ESIS_STE	MIR106	ED_IN_C	NOGENES	GENESIS	GENESIS	NOGENES	GETS_IN	ETS_NOT	E_TO_AD	ELL_ADI	ELL_ADI	OPOIETIC	OPOIETIC	DNMT1_TA	DNMT1_TA
0.06179	-0.03851	0.22638	0.08785	0.07157	0.04186	0.05156	0.14539	-0.34333	-0.12378	-0.02775	0.11603	0.11995	0.07656	0.09835	0.13247
-0.00467	-0.03377	0.22514	0.06654	0.06259	0.06208	0.00335	0.13186	-0.31839	-0.05841	-0.04778	0.0958	0.05648	0.07571	0.15667	0.12903
0.01103	-0.05971	0.18201	0.07272	0.06124	0.04017	0.04583	0.16471	-0.17909	-0.17594	-0.01415	0.08655	0.11717	0.0978	0.09173	0.13414
0.01205	-0.07961	0.19499	0.06048	0.06226	0.06002	0.04112	0.08057	-0.35304	-0.17301	-0.0076	0.13631	0.16962	0.06786	0.08078	0.12809
0.01796	-0.10914	0.16087	0.06478	0.07178	0.0725	0.02472	0.18716	-0.12763	-0.01277	-0.03645	0.12867	0.19051	0.05807	0.09354	0.10761
0.03825	-0.08714	0.15401	0.0367	0.07236	0.05376	0.01535	0.15713	-0.06692	0.03549	0.01358	0.06909	0.12522	0.07562	0.17633	0.13151
-0.02359	-0.08659	0.1721	0.0565	0.03848	0.0453	0.03212	0.13044	-0.11628	0.08967	-0.01577	0.11406	0.12853	0.0594	0.2442	0.15001
-0.04366	-0.0618	0.14563	0.0555	0.06418	0.05803	0.03445	0.11956	-0.22072	-0.12922	0.0023	0.15297	0.19493	0.04031	0.07428	0.10954
-0.03845	-0.07877	0.1467	0.0566	0.06745	0.05573	0.0273	0.13162	-0.1772	-0.11819	-0.00905	0.14085	0.20591	0.05035	0.08333	0.1523
0.00327	-0.06592	0.13629	0.05935	0.06242	0.07585	0.03334	0.12951	-0.04793	-0.02285	-0.04165	0.14896	0.20335	0.04116	0.07503	0.13025
0.00187	-0.04141	0.16837	0.05524	0.0411	0.05303	0.0306	0.16097	-0.16889	-0.25507	0.02709	0.08081	0.07449	0.112	0.11592	0.12363
0.01086	-0.05373	0.1804	0.06259	0.06011	0.05378	0.03004	0.14929	-0.12996	-0.14804	0.02432	0.10548	0.09914	0.09263	0.10194	0.1382
0.06945	-0.0477	0.15312	0.07565	0.0565	0.04167	0.03858	0.09585	-0.27348	-0.29356	0.0557	0.03042	0.0022	0.10827	0.10974	0.14379
-0.02233	-0.08178	0.18626	0.04688	0.05077	0.0627	0.03811	0.13331	-0.27884	-0.09675	0.01154	0.11509	0.15765	0.07644	0.08814	0.1143
-0.01809	-0.0648	0.15809	0.04265	0.06139	0.05152	0.0389	0.11605	-0.15131	-0.12587	-0.00447	0.10302	0.13122	0.08413	0.1099	0.12012
0.01203	-0.06327	0.18612	0.04662	0.03734	0.02995	0.05039	0.1597	-0.24664	-0.17439	-0.03446	0.07013	-0.01725	0.10225	0.108	0.14623
0.02049	-0.07441	0.21385	0.07388	0.05217	0.06047	0.02681	0.14677	-0.30683	-0.06374	-0.00587	0.10116	0.14802	0.08074	0.12684	0.11659
-0.0305	-0.08071	0.18252	0.05012	0.06322	0.07139	0.03961	0.20024	-0.12108	-0.07172	-0.04424	0.14647	0.16506	0.07522	0.0853	0.1301

METAST	METAST	IED_IN_L	FKB_SIG	NCER_B	NCER_B	FB1_SIGN	FB1_SIGN	IGNALING	IGNALING	TO_MESE	TO_MESE	AD6_TAR	AD6_TAR	SMRT_T	CEREBRA
0.09714	0.09255	-0.03455	0.0999	-0.06198	0.14508	0.17622	0.09001	0.00911	0.10483	0.14949	0.06318	0.00757	-0.08455	0.08077	0.28608
0.04423	0.10065	-0.05596	0.11481	-0.07077	0.16234	0.20641	0.11849	0.02227	0.095	0.12052	0.03909	0.02487	-0.12156	0.11228	0.28878
0.05201	0.06493	-0.01522	0.11339	-0.00979	0.16595	0.12571	0.08584	-0.02705	0.11834	0.11513	0.08798	0.04852	-0.10549	0.13867	0.2951
0.06669	0.09607	-0.04842	0.08006	-0.01038	0.137	0.15944	0.08607	0.02061	0.12983	0.16	0.07528	-0.02954	-0.02713	0.12738	0.29872
0.00355	0.04504	-0.00838	0.09541	-0.0527	0.14159	0.11129	0.07966	0.02694	0.10552	0.09757	0.07863	-0.07873	-0.1021	0.15066	0.29239
0.03359	0.09094	-0.04992	0.1098	0.04327	0.13861	0.25235	0.09306	0.05301	0.07593	0.0932	0.10896	-0.04016	0.00398	0.12756	0.30296
0.00065	0.06449	-0.04536	0.10421	0.00462	0.14439	0.1557	0.0686	0.07968	0.09381	0.1178	0.16795	-0.01801	-0.01639	0.16947	0.2861
0.00079	0.082	-0.02341	0.06543	0.00071	0.1423	0.15554	0.06023	0.00967	0.138	0.12642	0.11145	-0.07226	-0.02413	0.15211	0.29518
-0.00831	0.10939	-0.04632	0.08465	0.00171	0.15935	0.13746	0.07072	0.02063	0.10904	0.14162	0.10921	-0.07348	-0.03374	0.15404	0.29376
0.00071	0.08765	-0.05913	0.07946	0.02873	0.13692	0.17193	0.06803	0.01437	0.12026	0.11325	0.09256	-0.0197	-0.02032	0.07322	0.29445
0.08872	0.0832	-0.01771	0.10976	-0.06054	0.14431	0.13963	0.09346	0.01484	0.10603	0.13029	0.06683	0.10247	-0.0238	0.07488	0.28889
0.07792	0.03801	-0.03513	0.10789	-0.02601	0.14537	0.20407	0.08777	0.03487	0.11096	0.10613	0.09512	0.06084	0.00834	0.05968	0.29005
0.09791	-0.00091	0.00216	0.12214	-0.04079	0.17523	0.19694	0.09676	0.03782	0.07688	0.096	0.06739	0.02323	-0.10015	-0.02093	0.2937
0.06509	0.08227	-0.01365	0.09859	-0.05791	0.14838	0.11771	0.09966	0.00737	0.12024	0.12541	0.0851	-0.03162	-0.09709	0.11392	0.3032
0.0761	0.07456	-0.00642	0.09908	-0.0324	0.14466	0.16167	0.09904	0.02906	0.10642	0.10712	0.09191	0.04011	-0.03065	0.17142	0.29005
0.08736	-0.00044	0.018	0.14421	-0.04626	0.14278	0.1181	0.108	-0.00044	0.06038	0.09516	0.08387	0.08233	-0.03606	0.05281	0.29523
0.07962	0.04483	-0.02725	0.0935	-0.04428	0.13858	0.16911	0.08915	-0.00978	0.13364	0.15258	0.05898	-0.03018	-0.05066	0.15268	0.30941
0.0298	0.06273	-0.02176	0.09652	-0.01401	0.13323	0.13866	0.07664	0.02988	0.12605	0.15847	0.10109	-0.04538	-0.11211	0.1495	0.29736

CEREBRA	G_HYPOT	G_HYPOT	HE_DUPLI	YPOXIA_	YPOXIA_	YPOXIA_	P30_TARG	P30_TAR	VHL_TAR	IMMUNE_	IMMUNE_	NSFORM	NSFORM	ELD_VS	ELD_VS	E_CELL
0.22516	0.21256	0.32605	-0.21261	0.19847	0.12939	0.17692	0.18088	0.17691	0.12725	0.06996	0.14367	0.06563	0.12867	0.14623	0.09838	0.25404
0.22803	0.22503	0.31486	-0.47155	0.21132	0.12018	0.17849	0.20078	0.11333	0.14243	0.06873	0.11017	0.05597	0.11705	0.13452	0.07935	0.26731
0.2251	0.22721	0.33784	-0.38753	0.19727	0.10089	0.18868	0.16031	0.12901	0.14758	0.10013	0.11742	0.04575	0.14168	0.1478	0.09094	0.26287
0.24281	0.23972	0.31938	-0.48265	0.19498	0.12518	0.2026	0.18335	0.11182	0.13814	0.12509	0.12489	0.07044	0.14859	0.17299	0.09149	0.25677
0.24079	0.22612	0.32984	-0.47419	0.18884	0.10617	0.17423	0.18263	0.11575	0.13238	0.11351	0.10288	0.08032	0.1373	0.15083	0.0925	0.26477
0.26727	0.21403	0.32628	-0.43403	0.18632	0.11661	0.20244	0.16621	0.10118	0.14039	0.09997	0.11519	0.04862	0.11508	0.14114	0.0765	0.27797
0.26529	0.21948	0.32706	-0.46697	0.20381	0.10502	0.18093	0.18344	0.09145	0.14315	0.12129	0.11684	0.06622	0.12625	0.15108	0.0886	0.25963
0.25891	0.24033	0.33145	-0.47138	0.18538	0.13448	0.2058	0.16374	0.10063	0.13221	0.12964	0.12348	0.07366	0.17833	0.1616	0.10646	0.24622
0.25038	0.23665	0.33356	-0.47208	0.17727	0.11351	0.19395	0.13438	0.11321	0.13462	0.12869	0.11519	0.07267	0.13623	0.17274	0.09052	0.25089
0.25528	0.22405	0.33118	-0.46592	0.18706	0.10397	0.19699	0.155	0.11467	0.1344	0.13286	0.09162	0.07063	0.14636	0.15974	0.08519	0.25693
0.23363	0.1992	0.33137	-0.32887	0.19756	0.10696	0.16898	0.146	0.06655	0.11882	0.06347	0.12986	0.02964	0.11371	0.14039	0.07233	0.26205
0.23949	0.21795	0.31204	-0.35917	0.2142	0.10788	0.21235	0.15248	0.14143	0.15405	0.1087	0.14471	0.03627	0.13474	0.16111	0.09417	0.27128
0.24401	0.21128	0.30809	-0.48265	0.18959	0.11941	0.17449	0.1721	0.10703	0.13707	0.03505	0.13831	0.05869	0.09112	0.10458	0.08315	0.26045
0.24081	0.23213	0.32589	-0.30403	0.18648	0.10842	0.16493	0.18411	0.0631	0.13002	0.09366	0.13068	0.05144	0.15927	0.15391	0.08739	0.26445
0.23519	0.213	0.32241	-0.2142	0.20803	0.10698	0.18123	0.178	0.11071	0.13426	0.09171	0.128	0.04885	0.15104	0.15398	0.08357	0.2714
0.23457	0.20941	0.32454	-0.23146	0.21001	0.10644	0.17574	0.16947	0.09949	0.11996	0.06982	0.13534	0.05677	0.11336	0.1407	0.08941	0.2636
0.24765	0.2288	0.34292	-0.42716	0.19074	0.11622	0.18525	0.20793	0.12539	0.1321	0.10497	0.12154	0.06796	0.12737	0.14616	0.09915	0.27173
0.23793	0.2335	0.34384	-0.36252	0.19387	0.10307	0.18272	0.16465	0.11783	0.13077	0.12512	0.13084	0.08032	0.14896	0.17073	0.10094	0.24435

E_CELL	IS_BY_KR	IS_BY_KR	REPRES	ONSE_TO	ONSE_TO	CANCER_	CANCER	MAGENES	MAGENES	PARVB_T	PARVB_T	PARVB_T	PARVB_T	PARVB_T	PARVB_T
0.21508	-0.10696	0.24963	0.05785	0.1915	0.10255	0.19509	-0.10423	0.30533	0.20317	0.15135	0.00874	0.08122	0.20107	0.09167	0.13838
0.18614	-0.14623	0.2224	0.04328	0.19654	0.09876	0.16219	-0.0481	0.23575	0.22607	0.12501	0.01088	0.0835	0.20374	0.10094	0.12473
0.21911	-0.06768	0.25615	0.03201	0.19914	0.11172	0.17999	-0.28443	0.29309	0.21585	0.12607	0.06869	0.08983	0.21241	0.10336	0.12838
0.26015	-0.02365	0.21382	0.04629	0.20032	0.12234	0.18354	-0.14825	0.28068	0.23327	0.0814	0.07302	0.06254	0.22115	0.06803	0.15376
0.22193	-0.08445	0.06651	0.0287	0.19792	0.12523	0.16384	-0.32068	0.25161	0.22202	0.10091	0.04377	0.07335	0.21188	0.08197	0.13682
0.18425	0.09112	0.27415	0.01606	0.20094	0.12355	0.1967	-0.06191	0.25544	0.23088	0.08582	0.08422	0.07288	0.1956	0.08441	0.12085
0.21785	0.18919	0.26069	0.02841	0.19126	0.16788	0.19066	-0.12506	0.27899	0.20945	0.1281	0.13041	0.05345	0.22994	0.081	0.13744
0.25901	0.04543	0.32513	0.02747	0.20048	0.10673	0.19367	-0.22182	0.26401	0.21448	0.11634	0.09084	0.06626	0.2303	0.05944	0.15009
0.27824	0.07173	0.34577	-0.01695	0.19847	0.12505	0.19102	-0.20722	0.26562	0.20891	0.10938	0.06269	0.07339	0.23408	0.06059	0.15323
0.25587	-0.05049	0.25432	0.02693	0.20178	0.12097	0.18325	-0.29565	0.22735	0.21923	0.09724	0.05401	0.0662	0.22997	0.0689	0.1442
0.17632	0.0189	0.26655	0.05541	0.18363	0.09323	0.21102	-0.06567	0.33349	0.21048	0.12325	0.08429	0.0978	0.20306	0.10318	0.12279
0.19874	0.01135	0.19277	0.02336	0.20901	0.1268	0.18973	-0.24931	0.2736	0.20066	0.10208	0.08623	0.0852	0.21559	0.09863	0.13764
0.14966	-0.08879	0.14242	0.04895	0.20749	0.11357	0.14662	-0.04811	0.32563	0.16475	0.08635	0.08006	0.08684	0.20101	0.09718	0.1128
0.22068	-0.0373	0.11011	0.08948	0.18641	0.12208	0.20781	-0.16651	0.27104	0.1805	0.1118	0.06671	0.07506	0.20177	0.07621	0.15132
0.20795	-0.01079	0.24278	0.05542	0.20246	0.11561	0.21039	-0.15104	0.30514	0.20376	0.10143	0.0645	0.08056	0.20926	0.09225	0.13304
0.15956	-0.17543	0.26504	0.01805	0.17593	0.11034	0.22143	-0.16185	0.33541	0.18969	0.14157	0.07866	0.08998	0.20281	0.102	0.11689
0.21973	-0.10762	0.1765	0.05014	0.1873	0.11542	0.20075	-0.23842	0.27693	0.18528	0.11239	0.06594	0.07171	0.20364	0.07298	0.1399
0.25436	-0.02584	0.24637	0.03839	0.18364	0.1269	0.17119	-0.25877	0.24715	0.20238	0.13058	0.0871	0.07451	0.22578	0.07369	0.15563

TO_SOC	E_TO_SOC	TERC_TA	D_HEART	D_HEART	HEART_V	RIUM_VS	RIUM_VS	E2F1_TA	LOID_CE	ELOID_M	GA_EZH2	GA_SENE	AR_TARG	AR_TARG
0.12985	0.21495	-0.38087	0.18263	-0.06193	0.15151	0.18596	0.1148	0.07408	0.32022	0.29239	0.05408	-0.03893	0.01334	-0.18127
0.13117	0.17664	-0.42785	0.19562	-0.0107	0.17385	0.1631	0.11981	0.14033	0.1838	0.20562	0.10554	-0.00383	-0.00738	-0.17073
0.1658	0.1881	-0.45425	0.18986	0.01041	0.18734	0.17636	0.10061	0.17544	0.29031	0.21152	0.11828	-0.03653	-0.03455	-0.17494
0.14218	0.21185	-0.44156	0.17541	0.00966	0.20228	0.1533	0.13058	0.14545	0.32711	0.24077	0.07797	-0.05378	0.082	-0.14307
0.17351	0.19106	-0.43428	0.18421	0.01945	0.20352	0.15736	0.10257	0.14124	0.34699	0.25621	0.0694	-0.01898	-0.01491	-0.14237
0.15933	0.19083	-0.31171	0.19436	0.03536	0.21328	0.16319	0.10457	0.21774	0.22253	0.20939	0.15104	0.00703	-0.01586	-0.09936
0.14353	0.21319	-0.15379	0.17416	0.01949	0.21745	0.17901	0.12852	0.1369	0.27488	0.21624	0.09699	-0.04584	0.01637	0.0466
0.16247	0.22373	-0.26479	0.17044	0.09479	0.20783	0.16399	0.12334	0.05004	0.33588	0.25461	0.02957	-0.02463	0.08307	-0.12274
0.19391	0.18293	-0.43068	0.17833	0.09274	0.20992	0.14457	0.12813	0.07127	0.30002	0.21649	0.03243	-0.01227	0.03071	-0.04342
0.17368	0.18811	-0.32329	0.15958	0.03698	0.20247	0.14934	0.11197	0.08262	0.35682	0.24311	0.01138	-0.04024	0.01787	-0.16301
0.0897	0.19951	-0.16984	0.20086	-0.01179	0.1635	0.19271	0.11914	0.24103	0.3337	0.25754	0.16579	-0.0973	-0.00415	-0.14772
0.137	0.199	-0.39173	0.18111	0.03172	0.18554	0.18477	0.1166	0.196	0.27924	0.25199	0.10466	-0.0797	-0.03252	-0.12957
0.09293	0.17689	-0.45393	0.19797	0.0459	0.13136	0.16666	0.11846	0.18091	0.1249	0.23897	0.13987	-0.05039	-0.05994	-0.12249
0.15707	0.24595	-0.32521	0.17475	-0.034	0.18088	0.17413	0.11335	0.00334	0.33868	0.24493	0.02729	-0.06592	0.00199	-0.17298
0.13891	0.21121	-0.0327	0.18673	0.00043	0.1839	0.16923	0.12368	0.15911	0.38183	0.2689	0.08304	-0.05758	0.01327	-0.15399
0.0981	0.17891	-0.46705	0.18565	-0.02611	0.13493	0.18661	0.0892	0.18466	0.14374	0.24453	0.11666	-0.05048	0.01815	-0.17677
0.14894	0.22987	-0.4443	0.17136	-0.00269	0.17743	0.17529	0.12752	-0.01651	0.30403	0.246	-0.00297	-0.02089	0.02008	-0.15716
0.14807	0.20548	-0.44212	0.19578	0.03667	0.21806	0.15729	0.11287	0.05684	0.27508	0.23543	0.03017	-0.05252	-0.02356	-0.28643

URACIL_FU	URACIL_F	GLIS3_TA	TALIZED_	TALIZED_	TP53_TAR	TP53_TAR	E_TO_AR	SER_CANC	ER_CANC	TGFB1_S	TGFB1_T	TGFB1_T	AC7_TAR	AC7_TAR	AC7_TAR	AC7_TAR	ELAVL1_T	ELAVL1_T	DNA_REN	A_REPLI
0.16069	0.10817	-0.04166	0.06488	0.08704	-0.03006	0.06512	0.15663	0.06385	0.25876	-0.02082	0.12371	0.20402	0.12971	-0.00016	0.09966	-0.09917	-0.05214	0.07295	0.05591	0.08454
0.13539	0.08684	-0.04965	0.07637	0.06107	-0.04345	0.04235	0.12034	-0.02841	0.2359	-0.10089	0.11917	0.22139	0.12531	-0.00102	0.15615	-0.28111	-0.03849	0.04464	0.11254	0.12009
0.17995	0.06978	-0.0781	0.09635	0.08639	-0.03336	0.03946	0.09868	0.19277	0.25699	-0.02594	0.1244	0.22666	0.12028	0.01602	0.10832	-0.22963	-0.00115	0.08362	0.09421	0.13731
0.10286	0.05362	0.13378	0.0665	0.09523	-0.07513	0.07245	0.09889	0.0946	0.26542	-0.11313	0.13761	0.1958	0.13306	0.04851	0.07286	-0.25584	-0.0368	0.11453	0.05736	0.09435
0.06694	0.05898	-0.01382	0.09692	0.04667	-0.01907	-0.00974	0.07747	0.0876	0.23114	-0.07223	0.12416	0.19885	0.03521	0.03963	0.08838	-0.14594	-0.02579	0.0482	0.06541	0.09718
0.07735	0.1148	0.02026	0.06386	0.06212	-0.00986	0.05038	0.12739	0.1299	0.23237	-0.01316	0.08846	0.22244	0.10643	0.0514	0.10674	-0.21271	-0.00957	0.07621	0.09978	0.13104
0.13957	0.10911	0.01588	0.11199	0.0995	0.03852	0.08814	0.12453	0.03014	0.25786	0.03723	0.11676	0.22757	0.10496	0.02229	0.1464	-0.19897	0.04397	0.0795	0.07785	0.11406
0.13093	0.04015	0.14705	0.09695	0.11894	-0.08437	0.04146	0.08988	0.13988	0.26501	0.00442	0.13711	0.18608	0.10683	0.04989	0.08285	-0.25308	-0.02202	0.09082	0.02034	0.05309
0.09742	0.06537	0.06711	0.09071	0.09341	-0.03019	0.02272	0.09329	0.14874	0.25997	-0.03215	0.13347	0.18415	0.14182	0.04763	0.06002	-0.17042	-0.01527	0.08951	0.02983	0.07211
0.03763	0.09621	0.11656	0.07175	0.04031	-0.06244	0.02532	0.05957	0.1045	0.229	-0.09813	0.12638	0.18145	0.11477	0.04074	0.09209	-0.11798	-0.01126	0.08623	0.05507	0.06921
0.22957	0.05499	-0.07404	0.06011	0.05567	-0.06462	0.00998	0.13108	0.07941	0.23876	-0.03767	0.10731	0.21831	0.06666	0.01934	0.12468	-0.41359	-0.02452	0.06815	0.08894	0.15546
0.14248	0.08878	0.02813	0.04056	0.11266	-0.03973	0.10669	0.13968	0.10669	0.25888	-0.06884	0.12057	0.21115	0.14273	0.03602	0.15665	-0.22619	-0.02201	0.08357	0.09054	0.12204
0.2151	0.07981	-0.11892	0.06134	0.05298	-0.00456	0.06434	0.09005	0.00854	0.24863	-0.09977	0.11504	0.21091	0.13089	0.02596	0.13029	-0.43803	-0.05744	0.0446	0.11154	0.13204
0.15966	0.04952	-0.04274	0.08101	0.07251	-0.08407	0.04636	0.07835	0.07393	0.25381	-0.06478	0.13765	0.21345	0.1017	0.02173	0.15082	-0.31022	-0.00923	0.06992	0.05781	0.06877
0.19157	0.0209	-0.02382	0.06524	0.04563	-0.13587	0.04743	0.11028	0.14983	0.26549	-0.00138	0.11955	0.23326	0.05096	0.02939	0.1214	-0.31437	-0.03247	0.06903	0.08525	0.11728
0.20537	0.01352	-0.09818	0.02225	0.06359	0.0006	0.02828	0.08909	0.06967	0.24994	-0.12621	0.09795	0.24128	0.1005	-0.0024	0.15058	-0.38378	-0.00533	0.02613	0.11822	0.15057
0.11762	0.11226	0.00846	0.06781	0.0719	-0.06687	0.08558	0.08874	0.1377	0.25718	-0.10739	0.14082	0.20378	0.13615	0.0375	0.12878	-0.27508	-0.018	0.0617	0.05351	0.05207
0.10754	0.05421	0.05089	0.06246	0.07851	-0.10401	-0.01051	0.07867	0.1377	0.25763	-0.06002	0.13815	0.19152	0.12301	0.02635	0.07312	0.02081	-0.01941	0.07866	0.03697	0.06826

ELANOMA	ELANOMA	ING_MU	SING_MU	ESTRICT	ESTRICT	NNB1_TA	NNB1_TA	ONSE_TO	ONSE_TO	TIPLE_DR	N_TRIM24	N_TRIM24	DERS_CA	DERS_CA	ERS_DUR	ERS_DUR	ODENDR	ODENDR
-0.3473	0.08646	0.1685	0.10208	0.22116	0.14662	0.14183	0.12875	-0.27426	0.07778	0.14973	0.08297	0.07994	0.18675	0.18374	0.19454	-0.3796	-0.04687	0.21451
-0.36551	0.1285	0.17259	0.0898	0.22067	0.15579	0.14034	0.14239	-0.22318	0.05676	0.13437	0.1344	0.09262	0.0649	0.19097	0.19979	0.15836	0.02214	0.22447
-0.33139	0.18128	0.1649	0.10545	0.22841	0.17308	0.16035	0.12992	-0.35018	0.07922	0.14925	0.32676	0.10866	0.15007	0.17678	0.18969	0.03999	-0.16566	0.2115
-0.31062	0.11945	0.17799	0.10565	0.21202	0.17251	0.16394	0.15093	-0.30403	0.05441	0.15879	0.1846	0.1655	0.20481	0.17233	0.18716	-0.27796	-0.13986	0.21053
-0.27091	0.10369	0.17328	0.11104	0.19622	0.15448	0.1716	0.15017	-0.23727	0.0785	0.15208	0.34332	0.09424	0.1706	0.17824	0.17975	-0.28836	-0.13908	0.21471
-0.22579	0.16163	0.16708	0.11135	0.19817	0.15191	0.17036	0.13317	-0.2755	0.11528	0.15514	0.35459	0.08031	0.09459	0.17395	0.1771	-0.49921	-0.0658	0.21092
-0.21171	0.12131	0.18629	0.1132	0.21102	0.17568	0.19824	0.16181	-0.42681	0.11176	0.14607	-0.31777	0.1466	0.14141	0.17298	0.18599	-0.07416	-0.06487	0.21405
-0.33681	0.02776	0.17333	0.12765	0.21363	0.18471	0.15723	0.13157	-0.12278	0.12563	0.15773	0.41994	0.15938	0.18405	0.17194	0.16888	-0.31584	-0.12093	0.20408
-0.27828	0.06157	0.1629	0.12384	0.20766	0.20007	0.14184	0.13377	-0.16347	0.07931	0.14719	0.39035	0.18245	0.18543	0.17423	0.17983	-0.32482	-0.11758	0.21082
-0.22137	0.04373	0.16475	0.12099	0.20232	0.15929	0.14964	0.14972	-0.22758	0.10746	0.18156	0.3449	0.13188	0.2015	0.16804	0.17366	-0.19535	-0.10714	0.20353
-0.18283	0.20871	0.17304	0.10292	0.24524	0.16098	0.15047	0.11714	-0.43791	0.01608	0.11563	-0.2489	0.06325	0.13146	0.19084	0.19799	-0.09406	0.02215	0.22627
-0.21303	0.16974	0.16691	0.11488	0.22844	0.14501	0.12392	0.10767	-0.32077	0.11418	0.14176	-0.03047	0.06755	0.15673	0.18023	0.19755	-0.1078	-0.07876	0.21448
-0.12714	0.18013	0.1712	0.08175	0.18418	0.11702	0.15479	0.0964	-0.3021	0.02567	0.12056	-0.29047	0.02029	0.07818	0.19404	0.19026	0.07205	0.08467	0.21815
-0.24731	0.03761	0.18258	0.12223	0.20686	0.17756	0.16829	0.15303	-0.4062	0.08421	0.14439	0.27127	0.11753	0.17875	0.1828	0.18674	-0.19165	-0.11354	0.21848
-0.15993	0.13368	0.17081	0.11819	0.20701	0.1744	0.14678	0.12215	-0.04615	0.09581	0.14443	0.32288	0.09298	0.17487	0.18461	0.19265	-0.04474	-0.11124	0.21888
-0.38649	0.18867	0.18822	0.081	0.22359	0.13303	0.13383	0.09669	-0.29346	0.0396	0.11082	-0.21015	0.02532	0.01891	0.18778	0.202	-0.24379	-0.10732	0.22406
-0.29253	0.01945	0.17776	0.10492	0.22488	0.16962	0.16326	0.1294	-0.22952	0.07174	0.14526	0.19746	0.12229	0.16207	0.18545	0.19992	-0.18707	-0.07087	0.22692
-0.27593	0.06159	0.18297	0.10399	0.22168	0.16985	0.15256	0.11847	-0.39264	0.07906	0.17813	0.32253	0.1292	0.17596	0.17828	0.18071	-0.36833	-0.14774	0.21541

IGODEND	IGODEND	NCER_CH	CENTER_	CENTER_	IS2_TARG	IS2_TARG	M_HYPOX	ER_POO	ER_POO	RC3B_TA	LIFICATIO	LIFICATIO	LIFICATIO	LIFICATIO	IFICATIO	IFICATIO	EN_TARG
0.07623	0.2235	-0.07601	0.16912	0.05841	-0.11275	0.0659	0.23111	-0.03555	0.19434	0.27897	0.06987	0.41695	0.01761	0.0384	0.05815	0.15252	0.06896
-0.03977	0.2302	-0.02271	0.06905	0.05719	-0.32103	0.04819	0.20291	-0.10232	0.15165	0.24194	-0.0438	0.33486	0.00198	0.0822	0.01786	0.1714	0.07544
0.03712	0.21985	-0.02336	0.07942	0.05667	-0.17522	0.0777	0.16306	-0.06912	0.14038	0.29619	-0.00778	0.17862	-0.01596	0.07409	0.04958	0.1895	0.04575
0.07993	0.21569	-0.0328	0.09187	0.07399	-0.14591	0.13791	0.20214	0.00281	0.18095	0.34149	-0.04218	0.36163	0.0344	0.05481	0.03065	0.16552	-0.00427
0.039	0.22479	-0.01257	0.06968	0.09478	-0.00896	0.09816	0.18353	-0.02096	0.1511	0.3315	-0.0353	0.32869	0.00163	0.06912	0.03647	0.17397	0.00806
0.03016	0.218	-0.00321	0.01036	0.07247	0.12005	0.11059	0.18578	0.04894	0.18922	0.29984	-0.01235	0.24009	-0.01166	0.07446	0.02457	0.18351	-0.02357
0.05638	0.2187	-0.01332	0.07748	0.04798	0.09945	0.15794	0.20645	0.03246	0.18484	0.34048	-0.01269	0.1078	0.00058	0.10444	0.02493	0.18367	-0.05003
0.05151	0.21334	-0.02778	0.04452	0.10448	0.00379	0.13338	0.18491	0.05328	0.18578	0.32624	-0.05426	0.31214	0.01754	0.06901	0.0393	0.15743	-0.06629
0.04424	0.21743	-0.00964	0.03438	0.08844	0.0646	0.18786	0.19124	0.03251	0.19709	0.36541	-0.06153	0.23569	-0.00199	0.04308	0.03998	0.1621	-0.01728
0.07042	0.21135	-0.04734	0.02297	0.07298	0.09131	0.14168	0.14815	0.02748	0.17515	0.37281	-0.10609	0.38577	0.00426	0.04268	0.03842	0.17375	-0.12937
0.12351	0.22896	-0.02022	0.11455	0.05015	-0.19053	0.04782	0.187	-0.06956	0.16483	0.25704	0.03302	0.17298	-0.00485	0.06235	0.02017	0.18532	0.04132
0.0777	0.2198	-0.02969	0.07558	0.05379	-0.05395	0.10679	0.14164	-0.00714	0.18642	0.35805	-0.02616	0.29699	-0.00568	0.04856	0.0727	0.18482	-0.02757
0.07093	0.2241	-0.03208	0.1859	0.06538	-0.09065	-0.02049	0.19391	-0.08653	0.12624	0.21084	0.05568	0.18531	0.00359	0.08414	0.09005	0.1538	0.05295
0.07299	0.22218	-0.03445	0.1155	0.07419	-0.07442	0.09158	0.19547	-0.03069	0.16711	0.28926	-0.01592	0.30632	-0.02327	0.07749	0.02834	0.17507	0.00858
0.05638	0.22689	-0.02108	0.11379	0.05014	-0.10227	0.0798	0.18872	-0.00838	0.16325	0.29993	-0.0563	0.25982	-0.00407	0.07792	0.04455	0.17556	0.059
0.00451	0.21994	-0.01205	0.14017	0.03564	-0.34135	-0.02643	0.15953	-0.10842	0.12049	0.2598	0.07094	0.31901	0.00444	0.10257	0.0335	0.18993	0.06354
0.05738	0.22922	-0.06125	0.04328	0.06487	-0.12386	0.10267	0.22865	-0.01473	0.16637	0.23517	0.01418	0.37943	0.00795	0.05635	0.02772	0.17024	0.0183
0.00605	0.22196	-0.06405	0.06602	0.08113	-0.15752	0.12908	0.1786	-0.03116	0.17295	0.35331	-0.05761	0.29452	-0.01509	0.06975	0.04171	0.17294	-0.09376

EN_TARG	D_TSA_AN	D_TSA_AN	AL1_TAR	TARGETS	TARGETS	TARGETS	TARGETS	ST1_TAR	T1_TARG	DF_EWSR	DF_EWSR	EFFUSION	EFFUSION	S_OF_BC	FR_SIGNA	FR_SIGNA	FR_SIGNA
-0.05554	0.0604	0.1386	0.19621	0.11653	0.10888	0.07546	0.14493	0.05077	0.07856	0.05968	0.08319	0.10297	0.13499	0.06088	0.08762	0.14662	-0.20936
0.0412	0.0556	0.0628	0.17811	0.12197	0.10657	0.08055	0.1632	0.05212	0.08146	0.04837	0.09284	0.07838	0.16205	0.04517	0.12212	0.1371	-0.2887
0.01843	0.09363	0.0755	0.13879	0.13386	0.11228	0.08806	0.14887	0.06713	0.05711	0.04981	0.09491	0.11226	0.14832	0.10237	0.14578	0.13469	-0.44573
0.03925	0.09168	0.12704	0.17765	0.12463	0.09227	0.08293	0.14074	0.04179	0.07224	0.06806	0.07304	0.12596	0.15285	0.13873	0.09748	0.14721	-0.23081
0.03218	0.05924	0.05569	0.18556	0.1093	0.08195	0.0828	0.13037	0.04218	0.04784	0.04753	0.07841	0.11242	0.1453	0.1238	0.0956	0.12799	-0.31806
0.04095	0.01747	0.0671	0.13529	0.11543	0.07299	0.06776	0.13303	0.03329	0.08563	0.0418	0.0923	0.05123	0.14808	0.09001	0.13568	0.09849	-0.33874
0.06942	0.12791	0.07402	0.1344	0.13293	0.09457	0.064	0.16617	0.04863	0.06606	0.0479	0.08769	0.05694	0.14323	0.08276	0.13093	0.11261	-0.31325
0.04636	0.07651	0.06686	0.15647	0.10909	0.08733	0.09944	0.14572	0.03621	0.04555	0.0803	0.05719	0.12039	0.14722	0.15835	0.05358	0.15095	-0.30301
0.05867	0.05347	0.06316	0.12631	0.11816	0.08377	0.09988	0.14697	0.03819	0.05199	0.0717	0.05878	0.14229	0.1572	0.16931	0.07011	0.12159	-0.42102
0.08214	0.08109	0.05995	0.14286	0.10765	0.06499	0.05752	0.1305	0.04968	0.03972	0.05621	0.06706	0.12603	0.14132	0.16864	0.07885	0.13726	-0.41562
-0.00827	0.10181	0.11549	0.12868	0.12696	0.10175	0.05963	0.14188	0.06239	0.04808	0.04385	0.10528	0.0549	0.1531	0.04493	0.15099	0.10823	-0.1923
0.05989	0.07722	0.12915	0.1477	0.13347	0.09406	0.07606	0.12819	0.06193	0.06167	0.07054	0.0947	0.06977	0.14637	0.06445	0.1267	0.11708	-0.21829
-0.04652	0.15505	0.10542	0.13768	0.10555	0.10403	0.06274	0.13027	0.05538	0.05969	0.04484	0.1043	0.03146	0.16037	-0.04386	0.13764	0.10178	-0.13056
0.00733	0.09779	0.1333	0.1447	0.13456	0.08715	0.08448	0.14208	0.04208	0.04974	0.06211	0.07583	0.09957	0.14534	0.0969	0.07247	0.15612	-0.22928
-0.0003	0.10256	0.10621	0.14523	0.12242	0.12364	0.06413	0.14591	0.04503	0.08454	0.0637	0.09116	0.08803	0.12958	0.08571	0.12428	0.1245	-0.45837
-0.00288	0.14754	0.10539	0.15514	0.1148	0.09694	0.06443	0.13615	0.05619	0.08627	0.0296	0.11331	0.0371	0.12723	-0.06089	0.16649	0.09342	-0.16759
-0.00894	0.055	0.12245	0.19754	0.1187	0.10928	0.062	0.15168	0.03567	0.08923	0.07173	0.07264	0.09169	0.16608	0.08646	0.0661	0.16033	-0.09617
0.07984	0.09612	0.08618	0.163	0.12259	0.07641	0.09535	0.13171	0.04932	0.04357	0.08093	0.07159	0.12596	0.13183	0.13001	0.07352	0.13668	-0.38903

FR_SIGN	PONSE_T	EMT_EPIT	MT_MESE	K_CCNT1	K_CCNT2	LON_CA	LON_CA	TS_OF_S	NINE_DE	NINE_DE	NINE_DE	NINE_DE	TP63_GA	NCER_H	NCER_H	EZH2_TA	DO_HYP	CANCER_H
0.19873	-0.09091	-0.03413	0.05226	0.18381	-0.02479	0.28564	0.06095	0.10274	0.00135	0.0314	0.07478	-0.01428	0.20138	0.13216	-0.49833	-0.01567	-0.07416	-0.05536
0.10874	-0.10358	-0.06006	0.07932	0.10615	-0.02802	0.28439	0.10436	0.09727	-0.0023	0.01843	0.06767	-0.02699	0.04315	0.08103	-0.4978	-0.01937	-0.21014	-0.02668
0.23807	-0.11662	-0.10564	0.05636	0.14437	-0.01146	0.15865	0.09843	0.10092	-0.00646	0.0337	0.08301	-0.0233	-0.02869	0.14576	-0.49956	-0.0094	-0.10812	-0.02124
0.25246	-0.13966	-0.09892	0.12594	0.16663	-0.00465	0.19582	0.05298	0.09421	-0.00199	0.03909	0.07839	-0.00894	0.11217	0.10735	-0.49886	-0.0126	0.04986	-0.03823
0.29631	-0.17565	-0.23148	0.08396	0.11525	0.03065	0.15115	0.05361	0.07403	0.01364	0.03701	0.08317	-0.00904	0.01808	0.11246	-0.49797	-0.05256	0.01932	-0.05846
0.23025	-0.1998	-0.17408	0.03507	0.14252	-0.00896	0.17507	0.10988	0.07729	0.02345	0.06467	0.10075	0.01895	0.11257	0.14323	-0.49322	-0.01225	0.19652	0.03398
0.14989	-0.21328	-0.04382	0.03748	0.2067	-0.00145	0.11571	0.07523	0.06923	0.01868	0.08296	0.09703	0.03988	0.03834	0.13085	-0.49727	-0.00365	0.20379	-0.01978
0.25417	-0.2561	-0.21589	0.16084	0.11039	0.01602	0.15516	0.02878	0.07945	-0.01023	0.04226	0.06555	0.00108	0.09187	0.07417	-0.49833	0.00397	0.14765	-0.03241
0.2736	-0.19241	-0.2821	0.14704	0.12725	0.01825	0.12744	0.09371	0.07756	-0.01371	0.05433	0.07727	0.00545	0.07955	0.07706	-0.4978	-0.01569	0.0909	-0.0611
0.27261	-0.15847	-0.22648	0.12898	0.10929	-0.02838	0.16098	0.02608	0.06525	0.00892	0.05417	0.07098	0.01166	-0.04509	0.11552	-0.49886	-0.02695	0.06615	-0.05606
0.16782	-0.1367	-0.02661	-0.03651	0.20615	-0.07697	0.24491	0.13697	0.10576	0.03659	0.05786	0.12017	-0.0002	0.11561	0.1252	-0.49868	0.02166	0.06917	-0.0333
0.09961	-0.08031	-0.06017	0.03695	0.22916	-0.00068	0.14849	0.06938	0.09913	0.06503	0.05535	0.13118	0.00442	0.21356	0.16177	-0.49921	-0.0054	0.05623	-0.00638
-0.01027	-0.18185	0.07801	-0.0772	0.19249	-0.09315	0.24644	0.15191	0.10914	0.04868	0.05481	0.12941	-0.00474	-0.09922	0.11223	-0.3264	-0.01491	-0.01293	-0.00029
0.29822	-0.21804	-0.10726	0.09781	0.16068	-0.00441	0.33794	0.11293	0.08675	0.04017	0.02571	0.08838	-0.01947	0.07675	0.13197	-0.49974	-0.04215	0.00817	-0.04076
0.26608	-0.25025	-0.10209	0.05446	0.20101	-0.03725	0.39043	0.04773	0.08869	0.0285	0.04886	0.09912	0.00173	0.16936	0.06281	-0.49938	-0.01336	0.07477	-0.00109
0.1741	-0.21097	0.0486	-0.05709	0.18612	-0.10669	0.16078	0.06915	0.09867	0.08447	0.03825	0.11661	-0.00446	0.13504	0.13658	-0.49903	-0.00568	-0.11569	-0.06509
0.26007	-0.12321	-0.06136	0.09784	0.18653	-0.02661	0.17303	0.07641	0.09196	0.01837	0.03058	0.08044	-0.01315	0.08819	0.1277	-0.49974	-0.05322	-0.00993	-0.02077
0.27609	-0.11581	-0.25505	0.08795	0.15162	0.02368	0.14705	0.0995	0.09158	-0.02207	0.03057	0.06267	-0.01797	0.07006	0.13719	-0.49921	-0.01769	0.10024	-0.02586

_CANCER	E2F1_TA	E2F3_TA	HORIOCA	HORIOCA	RELATED	BRYONA	RYONAL_	RYONAL_	_CARCIN	_CARCIN	A_SEMIN	A_SEMIN	OLA_TER	LA_TERAT	_YOLK_S	OLK_SAC	MA3B_TA	MA3B_TA
-0.09622	-0.06084	0.11263	-0.46926	0.30075	0.03936	-0.03883	-0.46926	0.1738	-0.33925	0.10965	-0.46926	0.18168	-0.0348	0.18072	0.01197	0.23043	0.07904	0.05687
-0.1821	-0.13068	0.14204	-0.46609	0.28509	0.02196	-0.01538	-0.46609	0.17721	-0.1696	0.06755	-0.46609	0.20599	-0.16525	0.15724	-0.05126	0.25532	0.06358	0.05427
-0.07296	-0.15045	0.1358	-0.48459	0.29597	0.09138	-0.02719	-0.48459	0.19224	-0.11762	0.05519	-0.48459	0.20367	-0.1088	0.18299	0.01659	0.26432	0.07023	0.05756
-0.02398	-0.10684	0.10093	-0.47895	0.35008	0.06061	0.18633	-0.47895	0.19548	-0.25467	0.0807	-0.47895	0.21989	-0.05095	0.19793	0.0609	0.25862	0.06121	0.06273
-0.05281	-0.18979	0.09851	-0.47032	0.32921	0.08517	0.25949	-0.47032	0.20694	-0.21895	0.09146	-0.47032	0.21039	-0.10761	0.20495	0.01708	0.2769	0.04691	0.03831
-0.04369	-0.11269	0.14917	-0.42047	0.28998	0.08764	0.14815	-0.42047	0.20451	0.08488	0.14391	-0.42047	0.19642	-0.10056	0.16645	0.10991	0.25545	0.0573	0.06506
-0.05643	-0.00558	0.15192	-0.45993	0.31705	0.15678	0.14956	-0.45993	0.19291	-0.03273	0.08154	-0.45993	0.21576	-0.05802	0.16782	0.11224	0.26725	0.07614	0.06012
-0.02079	-0.15073	0.08465	-0.46627	0.30002	0.04752	0.19425	-0.46627	0.16721	-0.29492	0.15054	-0.46627	0.18812	-0.12166	0.20068	0.08463	0.25635	0.057	0.04994
0.02118	-0.24764	0.09942	-0.46539	0.33018	0.04941	0.19385	-0.46539	0.15751	-0.22809	0.10262	-0.46539	0.19274	-0.14387	0.20801	0.09959	0.25215	0.06292	0.05052
0.03637	-0.04562	0.11594	-0.45905	0.31798	0.02372	0.20313	-0.45905	0.17546	-0.15362	0.12348	-0.45905	0.19133	-0.11167	0.21743	0.07756	0.25564	0.05201	0.04092
-0.17897	-0.07275	0.13614	-0.48829	0.3333	0.01885	-0.3459	-0.48829	0.21232	-0.1495	0.09164	-0.48829	0.2463	-0.06853	0.10868	-0.01452	0.25575	0.063	0.0763
-0.04248	-0.04691	0.10309	-0.4823	0.24607	-0.00409	0.06109	-0.4823	0.15543	-0.11553	0.05379	-0.4823	0.17069	0.03593	0.13558	0.07981	0.23936	0.06338	0.05537
-0.20717	-0.0243	0.14434	-0.28431	0.27978	0.17213	0.12983	-0.28431	0.21373	-0.22106	0.17773	-0.28431	0.20357	-0.1081	0.1321	-0.01002	0.27224	0.05805	0.06192
-0.06006	-0.0425	0.12905	-0.49005	0.37537	0.0513	0.24042	-0.49005	0.24669	-0.29186	0.13721	-0.49005	0.25053	-0.07158	0.20622	0.04303	0.27511	0.08072	0.0514
-0.09629	-0.19822	0.11434	-0.48547	0.33606	-0.02698	-0.10987	-0.48547	0.19615	-0.41335	0.13103	-0.48547	0.20421	-0.0634	0.18863	0.02224	0.27192	0.07149	0.05285
-0.23858	-0.0758	0.12274	-0.418	0.27108	0.17886	-0.00725	-0.418	0.12357	-0.19738	0.01269	-0.418	0.13922	-0.05523	0.05167	-0.05591	0.20261	0.06163	0.03918
-0.09161	-0.06331	0.10123	-0.48212	0.35888	-0.01285	0.14079	-0.48212	0.19883	-0.27107	0.0932	-0.48212	0.20663	-0.12817	0.18878	0.02249	0.26764	0.05724	0.06329
-0.01179	-0.17941	0.10912	-0.47173	0.32778	0.0393	0.20583	-0.47173	0.18363	-0.3422	0.05663	-0.47173	0.21357	-0.20609	0.20529	0.01425	0.27482	0.05213	0.05883

KAYA_ILF	KAYA_ILF	CD99_TA	OXIA_NO	YPOXIA_V	TARGETS	O_ACID_	_TO_TOS	_TO_TOS	_TO_TOS	_TO_TOS	FURIN_T	FURIN_T	ONSE_TO	ONSE_T	TOPHAGY	N_LOAD	TS_OF_ML
0.05024	0.18187	-0.36621	0.04607	0.04896	0.07679	0.2013	0.07764	0.06327	0.08748	0.04808	0.04704	0.38746	-0.35348	-0.05867	0.08665	0.05171	0.08488
0.05635	0.17639	-0.35159	0.03779	0.03286	0.05234	0.11972	0.09152	0.03291	0.09952	0.03236	0.01222	0.37163	-0.15465	-0.0781	0.06169	0.05455	0.06062
0.09036	0.26317	-0.3338	0.03713	0.06101	0.06828	0.19432	0.09361	0.05318	0.0993	0.0506	0.04048	0.3823	-0.07351	-0.05197	0.08357	0.0589	0.09349
0.02903	0.26846	-0.22952	0.04583	0.06014	0.06613	0.09566	0.06458	0.07518	0.07378	0.05761	0.00073	0.39688	-0.27915	0.01398	0.08853	0.04936	0.09334
0.03407	0.23485	-0.13317	0.02523	0.0446	0.0649	0.18146	0.06994	0.07877	0.08586	0.05317	-0.37036	0.38382	-0.25733	0.03167	0.06163	0.03772	0.07937
0.08681	0.24921	-0.15501	0.05734	0.0093	0.07404	0.17512	0.08015	0.04141	0.08567	0.04015	-0.0995	0.37106	-0.10676	-0.06265	0.06748	0.03204	0.08846
0.07923	0.30071	-0.26211	0.03625	0.05561	0.07887	0.19897	0.10531	0.05129	0.10988	0.0529	-0.07018	0.37304	-0.31858	-0.01955	0.05963	0.03828	0.09775
0.02432	0.26243	-0.19605	0.03857	0.07146	0.06401	0.13396	0.05178	0.08746	0.0656	0.05872	-0.22903	0.38856	-0.3784	0.0188	0.10088	0.06104	0.08939
0.02927	0.2848	-0.11714	0.02771	0.06836	0.07517	0.08092	0.04587	0.08003	0.0596	0.05189	-0.01631	0.36867	-0.34303	0.04724	0.07947	0.0525	0.08949
0.01551	0.33511	-0.12084	0.02932	0.0546	0.09724	0.12367	0.06406	0.06816	0.07813	0.06025	-0.3054	0.37635	-0.47639	-0.04284	0.08065	0.05438	0.10812
0.1108	0.16406	-0.38083	0.0468	0.03889	0.08998	0.24021	0.09577	0.04761	0.09851	0.0531	-0.09722	0.39868	-0.07543	-0.0567	0.0874	0.05414	0.07409
0.11871	0.31158	-0.27867	0.04205	0.04187	0.09891	0.21021	0.08785	0.06019	0.09665	0.05064	-0.01321	0.39098	-0.22076	0.17605	0.09021	0.05124	0.10282
0.04448	0.16456	-0.41501	0.05843	-0.04139	0.07684	0.16648	0.08668	0.03309	0.08871	0.03388	0.01698	0.36652	-0.24299	-0.32619	0.04254	0.04969	0.05558
0.01538	0.20096	-0.22441	0.03484	0.05942	0.07165	0.1905	0.07857	0.06434	0.08598	0.05265	-0.26374	0.37041	-0.45976	0.04344	0.07166	0.05205	0.0839
0.05085	0.21972	-0.34191	0.04266	0.02112	0.07532	0.23619	0.08796	0.0668	0.09584	0.05741	-0.0473	0.37182	-0.25277	-0.03141	0.06273	0.03414	0.09669
0.09114	0.17233	-0.37379	0.05222	0.00619	0.09233	0.22371	0.11149	0.02635	0.10748	0.04692	0.12301	0.36591	-0.23206	-0.12961	0.03502	0.03313	0.06699
-0.01303	0.17958	-0.20821	0.03518	0.0613	0.06226	0.16625	0.08088	0.04997	0.08666	0.0457	-0.21534	0.39818	-0.4334	0.01841	0.06668	0.04637	0.09289
0.00898	0.29864	-0.21067	0.03401	0.05351	0.08625	0.07941	0.06309	0.07223	0.07485	0.05118	0.03194	0.39145	-0.35757	0.00199	0.07695	0.05547	0.0938

VS_PDG	VS_PDG	NCER_E	ANCER_E	NSE_TO	ONCOCYT	_CANCER	A_STABIL	A_DAMAGE	Y_4NQO	MAGE_BY	BY_GAMM	AGE_BY_G	A_DAMAG	DNA_DAM	NA_DAM	TAL_STR	ESS_RESP
-0.02353	-0.02194	0.24597	0.25664	0.07074	-0.43773	0.14896	-0.013	0.08916	0.02152	0.05761	0.06532	0.09358	0.11401	0.14152	0.05401	0.13516	0.10699
-0.10484	0.02033	0.20987	0.34701	-0.02393	-0.42998	0.11174	-0.05899	0.01424	-0.00127	0.07682	0.08288	0.06216	0.04665	0.11746	0.03118	0.13743	0.03426
0.01141	-0.01909	0.2003	0.35053	0.03208	-0.44724	0.15448	0.02704	0.03239	-0.0469	0.1133	0.12648	0.07862	0.11396	0.13809	0.06011	0.14095	0.04322
-0.01384	-0.02619	0.24876	0.24687	0.09573	-0.28571	0.14737	0.14538	0.05186	0.03729	0.10761	0.07515	0.08334	0.11661	0.13112	0.06484	0.16992	0.07353
-0.07905	-0.02458	0.25676	0.25196	0.03242	-0.43527	0.1375	0.06385	0.03911	0.01149	0.06559	0.07561	0.08915	0.13431	0.11703	0.07205	0.14799	0.11143
-0.05798	-0.00835	0.23077	0.29618	0.04854	-0.33962	0.15597	-0.12185	0.04307	-0.00939	0.06281	0.07828	0.08457	0.09748	0.11663	0.05194	0.13238	0.07334
0.03199	-0.03992	0.22027	0.28606	0.10143	-0.41624	0.13851	-0.06245	0.07408	0.00749	0.0779	0.10828	0.08075	0.07482	0.14009	0.04356	0.1453	0.07564
0.064	-0.02289	0.23453	0.25904	0.06961	-0.43227	0.1509	0.15801	0.08976	0.06275	0.12473	0.09672	0.08866	0.12883	0.13724	0.07991	0.15951	0.05845
0.00168	-0.03154	0.25485	0.27845	0.22334	-0.43174	0.13975	0.22889	0.08769	0.0171	0.10391	0.09213	0.06147	0.13354	0.12575	0.07174	0.16342	0.02881
-0.036	-0.07905	0.22998	0.2322	0.13291	-0.41642	0.18847	0.09654	0.03886	0.00444	0.08535	0.07645	0.08903	0.13898	0.10846	0.07913	0.14797	0.08141
-0.05824	-0.0417	0.22276	0.31015	-0.0193	-0.47349	0.10199	-0.27474	0.01451	0.0113	0.07615	0.07886	0.06828	0.1	0.13712	0.03396	0.17707	0.10996
-0.02175	-0.0365	0.25664	0.30976	0.05684	-0.46292	0.10847	-0.1941	0.05582	0.06214	0.08296	0.11	0.06437	0.11266	0.1415	0.05838	0.14249	0.07509
-0.12652	-0.00535	0.16278	0.33881	-0.12157	-0.45535	0.11909	-0.41675	0.04019	0.00421	0.0838	0.05919	0.07911	0.04838	0.12727	0.03199	0.13257	0.08761
0.05308	-0.07358	0.1578	0.29345	0.06419	-0.47983	0.11147	0.0516	0.0457	-0.01504	0.07774	0.0976	0.09569	0.14153	0.1432	0.05383	0.13364	0.07713
-0.05972	-0.01574	0.22405	0.30853	0.00727	-0.46609	0.12081	-0.05241	0.03397	0.01121	0.07805	0.07742	0.08997	0.10767	0.12775	0.05115	0.12922	0.09551
-0.1208	-0.0015	0.23951	0.30766	-0.07778	-0.46909	0.0829	-0.32981	0.02777	0.02362	0.08443	0.09444	0.04797	0.06076	0.13357	0.02405	0.12983	0.04004
0.06041	0.00837	0.2156	0.30263	0.0422	-0.44037	0.17626	0.06441	0.03122	0.00217	0.10528	0.07471	0.07941	0.12119	0.13473	0.05203	0.1388	0.10788
0.02359	-0.05233	0.22207	0.25058	0.06609	-0.18038	0.16235	0.05755	0.06755	0.02741	0.09309	0.08007	0.09044	0.1003	0.11988	0.0696	0.16251	0.0994

RESS_RE	NTAL_STR	DRMAL_AI	DRMAL_A	SPONSE_	SE_TO_H2	_TO_H2O2	_TO_H2O	ROM_AND	ROM_AN	NER_SYM	NER_SY	AL_CANC	AL_CANC	CND1_TA	E_LUNG_	ANCER_F	ANCER_F	REAST_PR	REAST_PR	BREAST_1
0.15336	0.16174	0.1037	0.08507	0.21158	0.30047	0.24762	0.16506	0.12787	0.10323	0.06482	0.1116	0.08574	0.14383	0.17906	-0.00861	0.07176	0.10713	0.12377	0.16782	0.10711
0.06798	0.13532	0.03764	0.07346	0.1997	0.29381	0.25107	0.14871	0.11993	0.10797	-0.03701	0.10543	0.1323	0.15485	0.17802	-0.08218	0.03791	0.05657	0.09541	0.09329	0.08969
0.05857	0.12691	0.06025	0.05358	0.20699	0.29588	0.25822	0.16392	0.13076	0.12537	-0.04877	0.11035	0.09066	0.15712	0.19485	0.03485	0.09518	0.00135	0.1406	0.07985	0.11353
0.06726	0.12963	0.04446	0.15145	0.21793	0.29911	0.22226	0.15046	0.12652	0.10033	-0.03656	0.16726	0.08333	0.16157	0.19924	-0.17326	0.10646	0.05212	0.15986	0.15719	0.13135
0.13156	0.13518	0.04079	0.07551	0.20948	0.26926	0.21905	0.1343	0.13303	0.11423	0.0041	0.0876	0.09716	0.15825	0.21096	-0.1193	0.07394	0.02882	0.1203	0.1335	0.10661
0.13924	0.12529	0.02869	0.07045	0.22492	0.31716	0.23371	0.11227	0.1209	0.1038	-0.0456	0.09859	0.08444	0.16364	0.19851	-0.03218	0.0909	0.00277	0.16009	0.13107	0.11556
0.10015	0.13352	0.04394	0.06505	0.20755	0.2773	0.22163	0.07261	0.12666	0.08987	-0.04067	0.10459	0.09047	0.17184	0.23636	-0.16538	0.11846	-0.01679	0.14039	0.05499	0.13118
0.02418	0.11502	0.06432	0.1451	0.2147	0.2776	0.21482	0.10421	0.11901	0.08459	-0.00413	0.16394	0.07037	0.16572	0.1863	0.02276	0.12858	-0.01632	0.16034	0.08664	0.14335
0.06472	0.11654	0.05462	0.13771	0.21292	0.30052	0.21625	0.09745	0.12317	0.07953	-0.04106	0.16844	0.06958	0.15783	0.23175	-0.06807	0.14189	-0.04862	0.15204	0.0711	0.14308
0.04547	0.11032	0.03447	0.11516	0.19331	0.23551	0.18052	0.11612	0.12511	0.08208	-0.04751	0.14016	0.09628	0.15325	0.21281	-0.1478	0.0884	0.01814	0.14877	0.09014	0.1188
0.08358	0.14037	0.064	0.01542	0.21765	0.29791	0.26581	0.15406	0.13831	0.11968	0.00897	0.05524	0.09077	0.16262	0.19987	0.09965	0.05533	0.07699	0.11866	0.20426	0.08584
0.08065	0.14904	0.00189	0.03369	0.22514	0.31339	0.24992	0.12361	0.11707	0.10301	-0.09374	0.07616	0.08437	0.16003	0.19603	-0.12599	0.09547	0.07632	0.14107	0.12706	0.11051
0.10572	0.14844	0.03296	0.03508	0.18997	0.28314	0.23839	0.14408	0.13202	0.14069	-0.07376	0.09367	0.09697	0.14326	0.22458	-0.05791	0.05065	0.06092	0.12765	0.11169	0.08319
0.10012	0.13692	0.08786	0.11113	0.21956	0.31464	0.24167	0.17333	0.12801	0.11327	0.01242	0.14496	0.09827	0.16614	0.19712	0.05333	0.11808	0.07108	0.15269	0.13711	0.13838
0.12784	0.13217	0.00626	0.05696	0.20737	0.28772	0.26294	0.16748	0.12552	0.1114	-0.03933	0.07091	0.10137	0.15774	0.19497	0.04091	0.07985	0.07475	0.13518	0.18279	0.10874
0.07195	0.13622	0.05991	0.06783	0.18873	0.25513	0.24724	0.16372	0.11781	0.10152	0.01359	0.08999	0.09979	0.15043	0.19655	-0.02311	0.05476	0.07305	0.12151	0.14548	0.07564
0.10964	0.13739	0.04637	0.11162	0.20408	0.30249	0.24053	0.15037	0.12789	0.10266	-0.03154	0.13779	0.07453	0.15519	0.20488	-0.09247	0.09219	0.06362	0.15182	0.11676	0.11859
0.15442	0.11864	0.10405	0.11369	0.20427	0.28744	0.21819	0.11063	0.12579	0.10035	0.00785	0.16172	0.08327	0.16429	0.20542	-0.0485	0.09627	-0.00333	0.13514	0.10441	0.13359

BREAST_	BREAST_	BREAST_	5_FAMILY	AMPLIFI	BLASTOM	BLASTOM	TOSIS_C	TOSIS_C	EN1_TARG	CEREB	G_CEREB	ING_MUS	NG_MUS	G_NEOC	G_NEOC	P2_TARG	MP2_TARG
0.12891	0.09233	0.23288	0.21295	-0.01764	0.04678	0.07452	-0.08074	0.04234	-0.00459	0.08199	0.16462	0.21125	0.21217	0.14204	0.19754	0.09231	0.07119
0.12801	0.11962	0.14514	0.20218	0.02374	0.07086	0.08409	0.04543	0.05173	-0.04163	0.09463	0.13593	0.20202	0.23452	0.16634	0.17709	0.11865	0.03484
0.09364	0.14755	0.13548	0.2242	-0.02409	0.04932	0.0606	-0.08166	0.06334	-0.04708	0.09816	0.18727	0.18096	0.22756	0.17431	0.20333	0.12138	0.06342
0.10274	0.13138	0.17402	0.20326	-0.01576	0.04349	0.06704	-0.18604	0.06683	0.02055	0.11293	0.22959	0.2205	0.23521	0.18812	0.22031	0.07206	0.08831
0.09881	0.08352	0.175	0.22361	-0.02036	0.05067	0.06644	-0.12326	0.06268	0.00607	0.11853	0.19296	0.19461	0.23922	0.19791	0.17899	0.08678	0.07211
0.09246	0.12938	0.16437	0.21105	0.03218	0.0476	0.08776	-0.28355	0.07356	-0.07825	0.17109	0.22075	0.17603	0.23459	0.21056	0.2337	0.11849	0.05206
0.0908	0.14893	0.10069	0.24692	0.01096	0.04884	0.09113	-0.10866	0.07697	-0.08867	0.14063	0.20747	0.17497	0.19111	0.21775	0.21542	0.12147	0.07334
0.06943	0.12444	0.14243	0.20557	-0.0068	0.04686	0.06874	-0.12706	0.03798	-0.02878	0.1409	0.24561	0.19407	0.2332	0.20709	0.21796	0.05754	0.0831
0.06794	0.13082	0.1344	0.19411	0.00255	0.04528	0.06022	-0.13654	0.0558	0.00027	0.13954	0.23786	0.19665	0.22936	0.20457	0.21463	0.05913	0.08552
0.07893	0.10639	0.11645	0.22027	-0.01877	0.04457	0.07458	-0.10826	0.0746	0.00012	0.12502	0.22724	0.17884	0.22679	0.1909	0.19609	0.07082	0.08384
0.13284	0.08716	0.16776	0.20943	-0.01162	0.06254	0.06802	-0.20653	0.07932	-0.01181	0.08497	0.13959	0.18048	0.21183	0.1417	0.20588	0.12804	0.06296
0.11713	0.15034	0.18146	0.21567	0.00667	0.03903	0.07673	-0.12561	0.05848	-0.01956	0.08329	0.18355	0.19511	0.21032	0.17028	0.19672	0.1111	0.08932
0.14192	0.09728	0.18338	0.19073	-0.01433	0.08154	0.07977	0.07873	0.06039	-0.04723	0.05833	0.13463	0.17397	0.20558	0.14636	0.16006	0.12066	0.04526
0.10875	0.14775	0.19325	0.21805	0.01217	0.0467	0.06968	-0.05513	0.05784	0.03113	0.13271	0.19937	0.1995	0.22207	0.18393	0.20924	0.08759	0.07729
0.12036	0.12536	0.19048	0.20314	0.05503	0.04565	0.06281	-0.19201	0.07805	-0.02126	0.09853	0.17112	0.19759	0.2293	0.16957	0.19972	0.11185	0.05978
0.13258	0.08977	0.13188	0.20262	0.05557	0.08065	0.09094	-0.05619	0.0834	-0.06731	0.08311	0.07814	0.19862	0.17522	0.14394	0.18279	0.1424	0.03826
0.11197	0.14298	0.17813	0.21257	0.00357	0.03839	0.09693	-0.10418	0.06537	-0.00095	0.11177	0.19001	0.18639	0.23651	0.17102	0.21406	0.08508	0.0768
0.08781	0.13084	0.16233	0.20683	-0.04554	0.05	0.06949	-0.08515	0.05148	0.02991	0.13449	0.22039	0.20444	0.2275	0.17223	0.21032	0.07133	0.08464

ESTRICT	HESTRICT	TRICTION	TRICTION	IATING_	E_POLAR	T_LYMPH	T_LYMPH	HYMIC_T_F	CANCER	CANCER	CER_CIP	NCER_CIP	_CANCER	CANCER	_CANCER	CANCER	ANCER_H
0.1525	0.28061	0.26002	0.06564	0.00702	0.03038	0.16377	-0.0173	-0.06458	0.04089	0.17808	-0.03705	0.01922	0.08146	0.12065	0.12603	0.12545	0.12173
0.15244	0.27709	0.24775	0.10131	-0.02408	-0.04682	0.13439	0.0259	-0.18814	0.00907	0.10922	-0.13053	-0.03494	-0.01787	0.06991	0.05448	0.13992	0.11145
0.14788	0.27508	0.23971	0.09785	0.04636	-0.11394	0.18554	0.08071	-0.13605	0.08498	0.13835	-0.02391	0.01501	0.05527	0.10162	0.13152	0.11678	0.1133
0.0989	0.3143	0.22905	0.07084	0.0719	0.04903	0.22819	0.04164	0.02302	0.10184	0.20242	-0.00875	0.06956	0.06733	0.16376	0.14558	0.1714	0.13607
0.13067	0.27917	0.22641	0.05719	0.13514	0.05657	0.24693	0.01701	-0.02811	0.08973	0.16618	-0.02774	0.02476	0.07497	0.102	0.11155	0.14095	0.13296
0.11303	0.2623	0.2579	0.07751	0.09813	0.01276	0.20446	0.06858	0.02888	0.17913	0.17111	0.05773	0.00599	0.09617	0.1212	0.17699	0.10255	0.14407
0.14456	0.28387	0.2198	0.07815	0.07224	-0.05045	0.20303	0.002	0.00477	0.11349	0.17184	-0.04798	0.03642	0.01186	0.1546	0.14929	0.13174	0.18787
0.11834	0.28885	0.22848	0.04347	0.10948	-0.03697	0.24472	0.00185	0.01994	0.13748	0.183	0.03119	0.05652	0.07485	0.14782	0.16482	0.16529	0.20069
0.09728	0.29008	0.22872	0.05576	0.11683	-0.08235	0.27008	0.03186	-0.01202	0.12303	0.19171	0.01735	0.05527	0.07421	0.14492	0.17332	0.15718	0.16204
0.12357	0.28747	0.22683	0.07591	0.14482	-0.00874	0.27876	0.00704	-0.01018	0.09458	0.1829	0.02152	0.04987	0.06614	0.15229	0.15911	0.16704	0.10752
0.1428	0.25381	0.2343	0.07336	-0.02871	0.05622	0.10526	0.04635	-0.1378	0.15775	0.15144	0.01623	0.03161	0.05683	0.12279	0.13493	0.15357	0.13634
0.13717	0.26876	0.24419	0.09261	0.04875	-0.10232	0.19452	0.03978	-0.04989	0.11308	0.20904	-0.02403	0.05892	0.08355	0.11645	0.16156	0.17161	0.16577
0.10753	0.24819	0.22709	0.00322	-0.09167	-0.06964	0.05057	0.01497	0.09642	0.0363	0.13683	-0.04964	-0.03218	0.0199	0.06542	0.06499	0.09133	0.0435
0.13843	0.28091	0.2212	0.05413	0.06465	0.11234	0.19646	-0.00865	-0.02015	0.09966	0.17464	0.00156	0.05529	0.08592	0.12441	0.15176	0.15686	0.12867
0.12298	0.28973	0.23567	0.04431	0.0091	-0.10803	0.15711	0.00434	-0.05489	0.10772	0.19392	0.012	0.0267	0.08449	0.13868	0.15518	0.14594	0.14392
0.12771	0.25615	0.206	0.0959	-0.09012	-0.11634	0.09731	0.02408	-0.2194	0.03213	0.13688	-0.08936	-0.05224	-0.0026	0.06844	0.06351	0.13125	0.0916
0.13989	0.29285	0.24103	0.05283	0.06276	0.01511	0.1868	-0.03279	-0.10422	0.08038	0.18473	-0.05501	0.05236	0.06417	0.12607	0.10524	0.15672	0.14352
0.11471	0.29044	0.23928	0.06427	0.10635	0.07219	0.2607	0.02007	-0.04035	0.09837	0.17907	-0.00413	0.02381	0.063	0.09894	0.12655	0.1527	0.17712

_CANCE	ANCER_	ANCER_	ANCER_	ANCER_M	_CANCE	ANCER_S	ANCER_	D_ALTER	O_ALTER	AND_RNA	E_T_LYM	CREST_S	CREST_S	IT_THYM	CP4_THYM	F_PTCH1	F_PTCH1
0.11315	0.05212	0.13818	0.00749	0.17908	0.17818	0.2499	0.00572	0.15368	0.0106	0.06906	-0.02539	-0.02186	0.12981	0.11863	-0.11238	0.03631	0.14941
0.05822	-0.02236	0.1332	-0.02091	0.14931	0.14641	0.26194	-0.04928	0.13613	0.01454	0.15941	-0.08165	-0.03797	0.14234	0.1106	-0.01291	0.04778	0.12325
0.11691	0.0592	0.14226	0.02216	0.13849	0.16921	0.25322	-0.01815	0.11214	0.03202	0.12589	-0.08016	-0.03071	0.13942	0.12433	0.00234	0.06891	0.18417
0.14562	0.06808	0.18759	0.04446	0.15818	0.17808	0.24124	0.0348	0.07159	0.04357	0.07006	-0.06165	-0.04869	0.14562	0.12048	0.17306	0.02829	0.22309
0.11266	0.03954	0.16095	0.01547	0.14425	0.16949	0.24737	-0.01117	0.07389	0.00251	0.09706	-0.02966	-0.0578	0.11597	0.12594	-0.01751	0.05649	0.19662
0.20095	0.12281	0.12504	0.08501	0.15891	0.15939	0.25434	0.05787	0.14065	0.0496	0.12193	-0.19516	-0.02192	0.13752	0.1004	-0.03834	0.04189	0.23732
0.20558	0.09309	0.15227	0.02468	0.17381	0.1545	0.23749	0.0559	0.12537	0.06969	0.10833	-0.13244	-0.02875	0.16883	0.11542	0.17269	0.00332	0.21492
0.14765	0.08354	0.16157	0.0598	0.14561	0.16066	0.22834	0.03612	0.05508	0.03449	0.07122	-0.09695	-0.05595	0.12823	0.1132	0.06492	0.02488	0.19223
0.1499	0.09678	0.1643	0.04617	0.13251	0.14701	0.22717	0.02776	0.08979	0.04329	0.05226	-0.07769	-0.03269	0.14757	0.13224	0.26834	0.06512	0.22008
0.17142	0.08003	0.14951	0.02635	0.15546	0.17067	0.22262	0.04318	0.0846	0.00804	0.06233	-0.03536	-0.04631	0.12123	0.12754	0.26233	0.03544	0.20456
0.1391	0.09358	0.13948	0.06037	0.19548	0.17635	0.26964	-0.00646	0.12173	0.02877	0.12417	-0.15674	-0.03485	0.1385	0.11808	-0.20817	0.07835	0.15598
0.15665	0.09106	0.15695	0.03642	0.21154	0.19536	0.25445	0.04333	0.13027	0.02311	0.09152	-0.17696	0.00477	0.13094	0.13559	0.14234	0.03837	0.19425
0.08524	0.01987	0.10611	-0.00106	0.14887	0.17614	0.26213	-0.00244	0.13952	0.06357	0.12724	0.06489	-0.00576	0.11528	0.09789	-0.16517	0.00713	0.11207
0.14548	0.07683	0.12434	0.05273	0.15163	0.16884	0.23995	-0.00109	0.08389	0.00914	0.10442	-0.0497	-0.05153	0.15905	0.10536	0.00335	0.01228	0.17888
0.13762	0.07743	0.15899	0.06829	0.17155	0.17952	0.24848	0.00507	0.14771	0.01833	0.09728	-0.14381	-0.05201	0.13782	0.12133	0.12629	0.04787	0.16943
0.06935	-0.02483	0.11186	0.00284	0.19931	0.21812	0.26628	-0.01379	0.1166	0.05925	0.16317	-0.00203	-0.02376	0.09234	0.11336	-0.13278	0.08531	0.09291
0.13583	0.05117	0.16031	0.02071	0.18838	0.16645	0.24816	0.00126	0.0923	0.00623	0.11225	-0.04023	-0.05078	0.12914	0.10699	-0.05461	0.0816	0.14817
0.10451	0.02401	0.15019	0.01296	0.11927	0.14323	0.24041	-0.00096	0.0559	0.02463	0.06068	-0.00036	-0.03293	0.13844	0.13927	0.21846	0.01283	0.2172

ROCYTE	EBELLUM	ID_PLEX	STAL_AND	TO_PROX	DULLA_M	BRAIN_	EURON_M	ENDROC	ONS_MAR	C8_TARG	C8_TARG	MYB_TAR	TIC_CEL	TIC_CEL	ARD_HYP	R2_TARG	R2_TARG
0.11159	-0.037	0.02572	0.0947	0.20086	0.27284	0.00841	0.11044	0.09395	0.18145	0.03942	0.36326	0.18187	0.11252	0.16656	0.12627	0.02472	0.07372
0.12005	-0.0677	0.02265	0.18544	0.21904	0.2685	0.07272	0.08569	0.1271	0.18081	0.05906	0.32031	0.16184	0.11665	0.13975	0.09809	-0.0123	0.09417
0.17757	0.03162	0.06205	0.14551	0.19958	0.23652	-0.00574	0.0818	0.11886	0.16229	0.04896	0.27061	0.16407	0.14087	0.16289	0.08226	0.03929	0.10825
0.22434	-0.05546	0.07801	0.08396	0.16401	0.24972	0.05969	0.07504	0.13849	0.19212	0.11626	0.31116	0.16916	0.1692	0.19948	0.1196	0.02386	0.10484
0.16537	-0.04579	0.06879	0.08027	0.18734	0.26064	0.06271	0.13695	0.11336	0.16316	-0.0219	0.20024	0.14661	0.15639	0.16835	0.07057	0.05731	0.08147
0.20999	-0.11572	0.11625	0.07346	0.1454	0.24132	0.08892	0.13984	0.13281	0.16727	0.08051	0.12666	0.15383	0.10231	0.18362	0.09125	0.02801	0.11662
0.24283	-0.02448	0.13903	0.08811	0.13092	0.25737	0.03046	0.10353	0.1254	0.18717	0.11871	0.29152	0.16442	0.12833	0.20276	0.11181	0.03687	0.10421
0.24896	-0.02578	0.07865	-0.0293	0.15882	0.25557	0.06692	0.12139	0.14371	0.17685	0.01789	0.15088	0.16031	0.19888	0.19065	0.09747	0.04251	0.0763
0.23418	-0.03496	0.12391	0.03776	0.17877	0.24504	0.08106	0.15671	0.13939	0.17068	0.13715	0.16519	0.16271	0.1942	0.20543	0.07733	0.03426	0.08235
0.21623	-0.08189	0.08121	-0.03706	0.13931	0.26508	0.0745	0.12656	0.11845	0.15857	0.07939	0.18656	0.15794	0.1926	0.1674	0.09284	0.01471	0.06771
0.16707	0.01125	0.07088	0.21713	0.22556	0.22615	0.02228	0.06509	0.08826	0.18888	0.10065	0.27215	0.18019	0.09254	0.12304	0.11165	0.01954	0.10544
0.18091	-0.04429	0.10932	0.15839	0.1917	0.26209	0.0198	0.09909	0.11076	0.17351	0.01347	0.28921	0.17639	0.09781	0.1747	0.11065	0.05392	0.08083
0.10147	-0.01303	0.05166	0.07564	0.17257	0.2168	0.02818	0.08688	0.09779	0.18977	0.04711	0.32717	0.15292	0.03303	0.12522	0.12355	0.07154	0.09665
0.18366	-0.00226	0.1093	0.12991	0.18467	0.25318	0.05584	0.13144	0.12487	0.18523	0.01183	0.29373	0.17745	0.14705	0.1767	0.08148	0.01519	0.06596
0.18492	-0.0318	0.08159	0.14374	0.14761	0.24649	0.04698	0.09151	0.11608	0.1726	0.09021	0.16852	0.17349	0.13248	0.16048	0.06497	0.05302	0.07943
0.06914	-0.04427	-0.00269	0.17586	0.15738	0.22363	-0.0033	0.07516	0.09042	0.21029	0.07261	0.2894	0.17236	0.05187	0.12808	0.07634	0.02768	0.09856
0.22822	-0.06293	0.09143	0.2009	0.15008	0.2587	0.02412	0.08064	0.14536	0.18799	0.05191	0.36213	0.17143	0.13549	0.18119	0.10846	0.03174	0.07014
0.23396	-0.05428	0.10767	0.07555	0.22335	0.26003	0.03781	0.07509	0.15172	0.18092	0.0601	0.30328	0.16254	0.16068	0.17397	0.08867	0.04745	0.07842

L_DIFFER	I_TARGET	I_TARGET	CELL_N	CELL_N	IS_STEM	CELL_N	CELL_N	ED_BY_M	ED_BY_M	ED_BY_M	PA_TARG	PA_TARG	GRANUL	SOX4_BIN	METAST	ARCINO	MA_META
-0.30132	-0.25233	0.17652	0.12195	0.20407	0.32017	0.18777	0.18752	0.1338	0.08849	0.17572	0.03289	-0.00098	0.30543	0.11137	0.10069	-0.07905	0.09531
-0.20935	-0.06027	0.06978	0.10743	0.17953	0.28914	0.13713	0.14821	0.13503	0.09302	0.18479	-0.00539	-0.01892	0.15894	0.13882	0.10893	-0.00405	-0.01073
-0.25652	-0.1753	0.04193	0.13985	0.23697	0.27472	0.18686	0.13328	0.17267	0.16691	0.16606	0.07607	0.0445	0.25923	0.12986	0.08112	-0.04634	0.02045
-0.24447	-0.16371	0.07825	0.11516	0.25715	0.27237	0.2015	0.14446	0.21791	0.1091	0.21513	0.1612	0.14354	0.31485	0.08364	0.07438	-0.0286	0.00653
-0.31858	-0.00028	0.05361	0.16222	0.23351	0.28498	0.1992	0.12492	0.14209	0.05785	0.14018	0.09808	0.06131	0.32301	0.07907	0.08067	-0.00576	-0.05447
-0.25194	0.10166	0.11674	0.16895	0.20129	0.29883	0.26313	0.12188	0.14666	0.17705	0.18229	0.02629	0.003	0.1786	0.10394	0.08733	0.09213	0.06954
-0.21769	0.05065	0.09096	0.11033	0.17872	0.26531	0.22609	0.13186	0.20375	0.15895	0.17397	0.06617	0.03564	0.21875	0.07166	0.08272	0.14961	0.07845
-0.32186	-0.03608	0.0727	0.12809	0.2949	0.32485	0.1749	0.15377	0.17006	-0.02593	0.22988	0.19791	0.17525	0.34652	0.06676	0.0675	0.00489	-0.01466
-0.24936	0.10847	0.03343	0.14099	0.26768	0.28542	0.1817	0.13269	0.22596	0.00663	0.228	0.1856	0.15802	0.30254	0.04086	0.06768	0.04298	-0.04571
-0.36327	-0.07688	0.01125	0.13757	0.27101	0.25096	0.18281	0.11717	0.24288	0.07702	0.19317	0.19104	0.16042	0.341	0.04611	0.06794	-0.00857	-0.00776
-0.15539	-0.10521	0.02095	0.12289	0.21335	0.31049	0.15844	0.14835	0.1581	0.19653	0.19665	-0.0152	-0.03653	0.29214	0.09323	0.09169	-0.01814	0.07596
-0.22019	0.02599	0.12912	0.16309	0.22214	0.32	0.1868	0.18374	0.23271	0.12383	0.21491	0.02107	-0.02783	0.23437	0.07662	0.08678	0.02248	0.07821
-0.27902	-0.19212	0.08349	0.1306	0.1513	0.35879	0.17814	0.21414	0.03847	0.14279	0.13173	-0.08228	-0.11247	0.06359	0.09088	0.10712	-0.05838	0.1164
-0.3607	-0.22853	0.10919	0.1275	0.24966	0.27992	0.17752	0.11902	0.16693	0.10945	0.21218	0.08984	0.05378	0.31206	0.07897	0.08424	-0.01712	0.0363
-0.27034	-0.11103	0.02541	0.1381	0.24325	0.30765	0.14766	0.16467	0.19089	0.12258	0.20271	0.05949	0.03278	0.30087	0.10552	0.08743	-0.00601	0.05862
-0.32234	-0.16076	0.00651	0.0971	0.13109	0.2684	0.15258	0.17426	0.14828	0.13937	0.18917	-0.12697	-0.14471	0.06977	0.10052	0.10276	-0.07663	0.07703
-0.28958	-0.18192	0.11082	0.11871	0.25241	0.30724	0.1571	0.1628	0.15528	-0.01307	0.21527	0.03931	0.03236	0.29068	0.11612	0.08117	-0.01685	0.03765
-0.25367	-0.08287	0.06501	0.15641	0.23111	0.28689	0.19993	0.13481	0.19871	0.03063	0.19304	0.13072	0.10885	0.26112	0.04824	0.07484	-0.0545	0.01506

MA_META	F_LUMIN	Y_LUMINA	UMINAL_	UMINAL_	RY_STEM	RY_STEM	ER_CANCER	CANCER	R_CANCER	CANCER	DER_CAN	CANCER	CANC	_CANCER	CANCER	RITIC_CE	RITIC_CE
0.19242	0.01688	0.02197	-0.05062	0.12309	0.07473	-0.00657	0.05451	0.10976	0.10947	0.20365	0.03663	0.06321	0.09356	0.06227	0.10161	0.05312	0.04122
0.1606	0.04385	0.01151	-0.07722	0.04773	0.04403	-0.01348	0.07669	0.11675	0.13431	0.01229	0.02117	0.06403	0.10435	0.07815	0.12569	0.00408	0.06547
0.18225	0.08203	0.00195	-0.05993	0.14743	0.04355	-0.01086	0.06939	0.114	0.13336	0.15458	0.0622	0.06736	0.10529	0.06322	0.11473	0.05749	0.01585
0.18815	0.07744	-0.0088	-0.01448	0.1639	0.04157	0.00857	0.06607	0.11519	0.12613	0.22159	0.09466	0.0909	0.06793	0.07862	0.09319	0.0978	0.04916
0.18411	0.02573	-0.00853	-0.12577	0.14613	0.0286	-0.0499	0.06842	0.12907	0.09999	0.05001	0.08006	0.07367	0.08273	0.0907	0.11092	0.08128	0.03981
0.12525	0.04794	0.00064	-0.15797	0.12235	0.0219	0.00853	0.08419	0.112	0.08614	0.06361	0.08487	0.08172	0.09996	0.05253	0.08973	0.03889	-0.00115
0.1366	0.07531	-0.01182	0.01112	0.12714	0.02795	0.00163	0.08066	0.11791	0.09674	0.13147	0.09863	0.06877	0.08987	0.12416	0.12969	0.10759	0.01303
0.19552	0.08606	-0.03589	-0.04028	0.13831	0.02078	0.00446	0.06024	0.13023	0.13308	0.01625	0.12105	0.10039	0.06813	0.08531	0.101	0.11577	0.03509
0.18604	0.10765	-0.01946	-0.05459	0.1281	0.01559	0.02985	0.0658	0.12412	0.12777	-0.02864	0.13175	0.09219	0.06149	0.0985	0.07962	0.17551	0.05243
0.15171	0.06302	-0.03834	-0.14467	0.11926	0.02026	-0.02945	0.0659	0.12476	0.11702	-0.03368	0.10923	0.09716	0.06752	0.0738	0.0963	0.14379	0.07819
0.14591	0.05826	-0.00463	-0.01125	0.12468	0.04991	-0.00465	0.07655	0.12697	0.08885	0.20357	0.03392	0.04054	0.11703	0.04061	0.10627	0.00225	-0.07044
0.14566	0.07902	-0.02802	-0.13264	0.15401	0.04991	-0.00245	0.06245	0.11618	0.09417	0.23404	0.06617	0.07928	0.09897	0.05171	0.115	0.06439	-0.0439
0.14549	-0.03426	0.05	0.03103	0.02062	0.07666	-0.01386	0.06523	0.07956	0.05778	0.2335	-0.02622	0.02435	0.11235	0.07901	0.12865	0.0037	-0.06532
0.20448	0.06041	-0.00806	-0.01619	0.14156	0.05289	-0.0159	0.06131	0.11334	0.12358	0.11878	0.07174	0.07484	0.07671	0.07845	0.13189	0.085	0.0024
0.15065	0.07142	-0.01296	-0.0376	0.17927	0.035	-0.00918	0.06985	0.11928	0.12567	0.15996	0.06851	0.05768	0.10668	0.08902	0.11539	0.03715	0.02318
0.12113	0.01087	0.02667	-0.09734	0.06298	0.06515	-0.02113	0.07534	0.10459	0.05081	0.20262	-0.03433	0.04056	0.11227	0.04488	0.09824	0.01657	0.00436
0.16805	0.04714	0.03577	-0.0324	0.15419	0.03805	-0.00557	0.0478	0.13015	0.11355	0.07786	0.06385	0.10091	0.06096	0.08772	0.11818	0.03753	-0.01104
0.1805	0.08148	-0.01858	-0.08907	0.15177	0.03219	-0.0138	0.06095	0.13982	0.10958	0.06588	0.10237	0.09576	0.07154	0.0801	0.10127	0.15634	0.03685

RITIC_CE	RITIC_CE	DRTALIZE	DRTALIZE	Y_MIR16	APC_TARG	MA_COPY	MA_COPY	A54_TARG	A54_TARG	TUMOR_M	PE_FROM	REAST_C	X2_TARG	X2_TARG	YB_TARG	YB_TARG	N_CANC	EMORY	MEMORY	I_PRIMING
0.10529	0.13411	0.04506	0.15742	-0.00866	0.22601	0.07763	0.0233	0.03196	0.03816	0.02961	-0.19739	0.15643	0.31944	0.09568	0.25993	0.0604	0.12535	-0.18412	-0.00199	-0.11774
0.04924	0.10749	0.08151	0.13789	0.00043	0.24295	0.0931	0.06199	0.02752	0.02735	-0.02868	-0.20167	0.177	0.2723	-0.12117	0.27094	0.03959	0.12251	-0.13495	-0.03676	-0.17981
0.06497	0.14004	0.05778	0.17686	-0.01461	0.23845	0.09738	0.01877	0.00622	0.02199	-0.01727	-0.26333	0.16063	0.23911	-0.01329	0.29987	0.03461	0.13212	-0.04048	-0.00553	-0.23481
0.07866	0.17602	0.08381	0.14272	-0.00806	0.23323	0.07745	6.8E-05	0.0532	0.01342	0.00205	-0.36394	0.19799	0.34966	0.01004	0.31631	0.01243	0.13401	-0.02266	0.07683	-0.11164
0.09194	0.13489	0.0436	0.15298	0.00245	0.20854	0.06624	0.04435	-0.00938	0.03798	-0.03361	-0.18519	0.16695	0.27156	-0.02282	0.31853	0.02504	0.12566	-0.01867	0.12561	-0.14816
0.07693	0.12267	0.09334	0.14122	-0.00628	0.22407	0.08545	0.05217	-0.02065	0.02438	-0.04745	-0.17956	0.14991	0.26738	0.06746	0.26769	0.0399	0.10074	-0.05688	0.00662	-0.16089
0.07161	0.1844	0.09172	0.1632	-0.0292	0.20411	0.06244	0.01552	0.03644	-0.0023	0.00133	-0.15734	0.15659	0.19763	0.04562	0.2726	0.06652	0.1166	-0.15203	0.07393	-0.28383
0.08545	0.19997	0.06827	0.13151	-0.01227	0.20254	0.09168	0.02051	-0.00037	0.01953	-0.02172	-0.29632	0.19054	0.25599	-0.02005	0.29709	0.01881	0.12996	0.00083	0.12478	-0.15113
0.09261	0.19755	0.07388	0.1415	-0.00455	0.2126	0.07258	0.02528	0.05194	0.01912	-0.02644	-0.28229	0.17599	0.36374	-0.00909	0.27577	0.02188	0.12441	0.06571	0.09049	-0.14926
0.09215	0.19248	0.00557	0.1514	-0.01373	0.19288	0.06094	0.02574	-0.02495	0.00849	-0.02934	-0.22411	0.18488	0.39341	-0.03954	0.29966	0.01989	0.13825	-0.04514	0.1001	-0.13638
0.07709	0.07322	0.06634	0.12104	-0.00413	0.24228	0.01479	-0.01756	0.04569	0.01963	0.06043	-0.0336	0.16904	0.19893	-0.0244	0.32477	0.05322	0.12476	-0.05013	-0.00233	-0.11076
0.12023	0.1248	0.06716	0.12527	-0.00667	0.24251	0.07083	0.03698	0.06565	0.01536	-0.00519	-0.13208	0.15317	0.2798	0.11808	0.2492	0.03312	0.11622	-0.09563	0.03022	-0.17613
0.03193	0.06353	0.0762	0.13303	0.00993	0.23068	0.03714	0.07615	0.07717	0.04359	0.07326	-0.21736	0.13971	0.23312	-0.09207	0.28326	0.0378	0.09541	-0.15264	0.04205	-0.11599
0.07977	0.17475	0.05513	0.17381	0.00992	0.21576	0.04865	0.03163	0.03111	-0.00207	0.06313	-0.25935	0.17777	0.17404	-0.02826	0.31051	0.04199	0.11201	-0.09042	0.05041	-0.15401
0.10792	0.1574	0.04607	0.15463	-0.02003	0.21505	0.06987	0.00436	0.06143	0.03486	0.04125	-0.24037	0.16935	0.2996	0.00257	0.30029	0.04969	0.1124	-0.08265	0.03013	-0.1661
0.04799	0.02979	0.06465	0.13946	-0.00419	0.24279	0.08151	0.02881	0.03175	0.0258	0.03493	-0.24543	0.15151	0.28857	-0.09431	0.19675	0.05943	0.134	-0.17163	-0.01247	-0.1332
0.05688	0.15725	0.06026	0.15534	-0.01703	0.24017	0.12916	0.03617	0.00877	0.00381	-0.00635	-0.30379	0.1851	0.28796	-0.00876	0.29957	0.02388	0.14107	-0.08101	0.03959	-0.19153
0.09502	0.15957	0.06294	0.14931	-0.01502	0.21154	0.08774	0.01809	0.02491	0.01256	-0.04416	-0.27624	0.1937	0.39715	-0.03966	0.29528	0.00987	0.1214	-0.03869	0.09472	-0.17604

MORS_AN	STATE_CA	STATE_CA	ARCA4_TA	X4_TARG	X4_TARG	OF_VMYB	OF_VMYB	PBP1_TA	ATE_CAR	CATE_CAR	YB_TARG	IS_BY_AC	D_IN_LU	IN_RESIS	TIN_RESIS	NALOGS_0	E_ANALO	2_BOUN	TO_NATU	TO_NATU
-0.09574	0.04981	0.08447	0.12532	0.10149	0.01238	0.05062	0.01699	-0.17954	0.10484	0.20814	0.01422	0.12459	0.32602	-0.02108	-0.08722	-0.07072	-0.09073	0.16735	0.00547	0.17189
-0.04619	0.05489	0.0603	0.09101	0.10994	0.01412	-0.01539	-0.08177	-0.32991	0.0729	0.1747	0.00083	-0.01792	0.31642	-0.05006	-0.21412	-0.12378	-0.13233	0.18933	0.02443	0.13743
0.01533	0.03978	0.07556	0.14055	0.10583	0.04044	0.03037	0.01832	-0.2379	0.17162	0.23239	0.02615	0.07859	0.29889	-0.04972	-0.1793	-0.05913	-0.13131	0.18404	0.03554	0.16468
-0.0228	0.06537	0.04607	0.18464	0.09013	0.03358	0.08583	0.03381	-0.32144	0.1001	0.28708	-0.00168	0.14511	0.30752	0.01244	-0.18637	-0.09441	-0.10941	0.15801	0.02985	0.20282
0.0239	0.02772	0.03715	0.07121	0.09814	0.02681	0.08353	-0.03491	-0.36793	0.1483	0.24919	0.01984	0.10395	0.29425	0.01788	-0.18292	-0.07303	-0.06874	0.1767	0.04999	0.19183
0.09097	0.04876	0.07012	0.09244	0.07729	0.02245	0.06806	-0.02309	-0.29657	0.24593	0.27048	0.00684	0.1181	0.30366	0.06759	-0.22498	-0.07121	-0.04131	0.17031	0.0327	0.13621
-0.1154	0.05112	0.08444	0.1893	0.09028	0.01523	0.02973	0.00406	-0.23124	0.21573	0.27619	0.04289	0.15999	0.30744	0.04516	-0.20751	-0.08279	-0.07688	0.17151	0.07028	0.17085
-0.01376	0.04044	0.04786	0.10865	0.08481	0.04692	0.10309	0.02295	-0.35726	0.16122	0.30457	0.02252	0.1361	0.29929	0.03663	-0.1602	-0.10165	-0.07831	0.15805	0.03703	0.20495
-0.02962	0.06231	0.06199	0.13175	0.08192	0.05957	0.1267	0.03521	-0.34916	0.11258	0.2965	0.03528	0.0941	0.2818	-0.00172	-0.15582	-0.07658	-0.06738	0.14994	0.03296	0.20309
-0.08266	0.03645	0.04862	0.09409	0.08736	0.04746	0.12926	0.00233	-0.3321	0.10383	0.30081	0.02163	0.16226	0.29735	0.04084	-0.16052	-0.06275	-0.07084	0.16377	0.04311	0.19683
-0.037	0.07163	0.06886	0.15893	0.10588	0.009	-0.01618	0.08956	-0.3058	0.24212	0.1925	0.00786	0.11065	0.3187	-0.05015	-0.20023	-0.05168	-0.04511	0.15963	-0.00339	0.14246
-0.00486	0.02577	0.0946	0.14151	0.09616	0.03513	0.06802	0.09999	-0.17389	0.14968	0.24517	0.02734	0.1014	0.31042	0.08209	-0.17686	-0.0183	-0.04552	0.18914	0.02664	0.16612
0.02745	0.03132	0.07069	0.12907	0.11906	0.03041	-0.05846	0.0782	-0.34054	0.20652	0.09754	-0.00329	0.06589	0.31348	-0.08072	-0.18382	-0.05799	-0.08945	0.16532	0.00286	0.11423
-0.04251	0.04576	0.04959	0.15832	0.0996	0.01753	0.05268	-0.00519	-0.37785	0.14364	0.22577	0.004	0.13102	0.31801	-0.03048	-0.18558	-0.06886	-0.11849	0.15962	0.03619	0.18854
-0.04007	0.0704	0.09713	0.12531	0.09781	0.02386	0.02358	-0.06622	-0.39684	0.15703	0.22734	0.01936	0.12953	0.32044	-0.03241	-0.17554	-0.02526	-0.01289	0.16398	0.01239	0.1679
-0.12414	0.05897	0.1083	0.10422	0.11489	0.02367	-0.01594	-0.02913	-0.23526	0.17531	0.09069	-0.00369	0.04786	0.31685	-0.07495	-0.26541	-0.01252	-0.09352	0.20605	-0.00219	0.11018
-0.10906	0.05844	0.04222	0.15287	0.10145	0.04027	0.04703	0.04896	-0.25686	0.15919	0.22674	0.00497	0.11979	0.31787	-0.02064	-0.23178	-0.09026	-0.14746	0.17186	0.04827	0.19102
-0.00286	0.0518	0.04478	0.15479	0.08566	0.01682	0.05818	0.05899	-0.38614	0.12154	0.2714	-0.00106	0.04738	0.29527	-0.0396	-0.16576	-0.02012	-0.10269	0.17381	0.03231	0.20225

ING_CAN	CANCER	ILMS_TU	MOR_AN	MOR_AN	VS_FET	VS_FET	VS_FET	VS_FET	LIFIED_I	ED_IN_C	ED_IN_C	ELIOID_M	MBD_TA	S_IMPRI	IOMA_SU	IOMA_SU	HELIOMA
0.30199	0.00675	0.03492	-0.04981	-0.0976	0.12798	0.15961	-0.07774	0.23892	0.09787	-0.00148	0.16114	-0.01643	0.17343	0.14124	0.10132	0.12553	0.11025
0.27662	-0.03072	-0.10946	-0.03658	0.04049	0.15355	0.11331	-0.04942	0.27025	0.08728	0.03274	0.09208	0.04792	0.17865	0.19443	0.00588	0.15351	0.21984
0.26984	-0.02745	-0.00475	0.02247	0.10311	0.15556	0.14859	-0.02536	0.21908	0.085	0.01655	0.04742	-0.00788	0.17469	0.15866	0.13109	0.13754	0.09996
0.31896	-0.09927	0.08759	-0.05246	-0.04026	0.09746	0.16499	-0.00747	0.23328	0.07665	0.00302	0.01352	-0.00674	0.1776	0.14723	0.14163	0.16402	0.19057
0.29146	-0.12483	0.04213	-0.05035	-0.01842	0.10539	0.11693	-0.109	0.23135	0.06748	-0.02342	-0.00222	-0.1687	0.17133	0.13923	0.06679	0.12652	0.09682
0.28729	-0.06934	0.07103	-0.06018	0.05497	0.16553	0.12615	0.02959	0.25148	0.06833	0.02153	-0.09066	-0.11798	0.17688	0.15224	0.18033	0.25392	0.1641
0.26995	-0.08116	0.07062	-0.00977	-0.02077	0.13738	0.11345	-0.00645	0.25515	0.06876	-0.01834	0.02514	-0.13233	0.17486	0.20681	0.17829	0.20899	0.11135
0.28512	-0.09057	-0.04261	-0.0986	-0.0676	0.06416	0.14973	-0.02031	0.20535	0.0705	-0.05809	0.00778	-0.1049	0.17112	0.1424	0.16863	0.10211	0.18712
0.29045	-0.04635	-0.13355	-0.03384	-0.05393	0.07468	0.15578	0.02922	0.20475	0.0634	0.01847	0.00438	-0.17411	0.17192	0.13228	0.15101	0.1022	0.19248
0.28671	-0.09279	-0.04358	-0.01329	-0.07484	0.09029	0.13318	-0.04183	0.20715	0.06304	-0.03756	0.01062	-0.15709	0.17025	0.12068	0.16822	0.12148	0.15084
0.28278	-0.05303	-0.03025	-0.11518	0.05708	0.1649	0.11712	-0.0317	0.15824	0.09078	0.03475	0.06741	0.09405	0.17212	0.13103	0.09862	0.20953	0.10792
0.27587	0.01498	-0.11074	-0.04478	-0.00636	0.13496	0.14424	-0.0363	0.17781	0.08295	-0.01369	0.09146	0.00499	0.16965	0.13412	0.10436	0.1598	0.16531
0.2583	-0.10693	0.09109	0.01536	0.07686	0.16117	0.115	-0.00651	0.2077	0.10215	-0.00613	0.06848	0.15085	0.16998	0.14407	0.03048	0.23864	0.11459
0.28858	-0.10837	0.15585	0.0006	-0.05057	0.11007	0.14299	-0.04967	0.26026	0.06986	-0.03942	0.00325	0.01055	0.16769	0.18413	0.13605	0.18779	0.11308
0.31092	-0.0586	0.06542	-0.10076	-0.07053	0.14585	0.1356	-0.04353	0.24315	0.08451	-0.0243	0.05309	0.00197	0.17284	0.12777	0.1518	0.1806	0.18626
0.27512	-0.00306	-0.02145	-0.0999	0.00126	0.15693	0.11923	-0.04264	0.21467	0.07325	0.02971	-0.05007	0.07317	0.17774	0.19654	-0.04878	0.30773	0.07559
0.28806	-0.02993	0.16467	-0.08285	-0.10627	0.10559	0.15992	-0.02088	0.30073	0.06783	0.00393	0.04617	0.01128	0.17556	0.16813	0.08021	0.14112	0.19306
0.29274	-0.06209	-0.02406	-0.03699	-0.0774	0.08267	0.13902	-0.06362	0.18914	0.06159	-0.05775	0.06049	-0.13878	0.17315	0.16817	0.09803	0.10423	0.13824

DMA_SUR	DMA_SUR	HELIOMA_SUR	A_SURVIV	VATION_VIA	NF4A_TAR	NF4A_TAR	EATING_I	DF_PAX8	I_CANCER	CANCER	CANCER	CANCER	CANCER	CANCER	ANCER_F	ANCER_F
0.11075	0.00397	0.11438	-0.00584	0.05101	0.09524	0.12714	0.08313	0.3636	0.34873	0.18116	0.05912	0.3492	0.17902	0.36482	0.34388	0.1531
0.16851	-0.09929	0.18209	-0.171	0.07446	0.12954	0.14368	0.08775	0.23925	0.35084	0.18911	0.04112	0.34489	0.14615	0.3163	0.33423	0.18028
0.04588	0.00861	0.15309	-0.03706	0.10315	0.11174	0.00364	0.09414	0.28785	0.33467	0.13378	0.05301	0.34732	0.24421	0.41271	0.33867	0.15389
0.13018	0.13119	0.21716	-0.01269	0.03587	0.12277	0.13981	0.05158	0.39763	0.34209	0.15561	0.09302	0.35412	0.30727	0.39481	0.3498	0.15846
0.11689	0.02242	0.13714	-0.03885	0.00869	0.09261	0.01341	0.07573	0.30682	0.32968	0.12002	0.08414	0.34766	0.34496	0.3238	0.35723	0.15083
0.06369	0.11394	0.27211	0.10873	0.12533	0.1441	0.21744	0.06401	0.30434	0.33448	0.14318	0.02069	0.34997	0.22335	0.38945	0.35671	0.14184
0.06152	0.09531	0.20229	0.08413	0.14136	0.13935	0.18986	0.04998	0.31401	0.33299	0.14364	0.06858	0.34029	0.27217	0.37581	0.34404	0.14912
0.14583	0.12759	0.19236	-0.00015	0.04452	0.08657	0.14793	0.05586	0.3467	0.3337	0.1282	0.10877	0.35007	0.27966	0.3777	0.35396	0.1513
0.1216	0.11504	0.22754	-0.00872	0.01555	0.09973	0.13107	0.05625	0.25937	0.32205	0.13487	0.08231	0.34131	0.35699	0.42168	0.3478	0.16612
0.08487	0.10696	0.20877	0.02056	-0.0048	0.0957	0.1584	0.0542	0.31856	0.33336	0.14678	0.06569	0.33951	0.30917	0.35342	0.34783	0.14541
0.11745	0.06405	0.12854	0.03883	0.13166	0.13035	-0.32886	0.07518	0.42366	0.34904	0.1333	0.06223	0.36131	0.26046	0.40392	0.34398	0.16789
0.10764	0.04537	0.11314	0.01059	0.10622	0.11687	0.16384	0.03701	0.38241	0.35061	0.1277	0.03648	0.36595	0.277	0.42021	0.36723	0.14429
0.12188	0.01076	0.00242	-0.02047	0.14451	0.13857	0.06279	0.07093	0.28175	0.34969	0.18171	0.0347	0.35581	0.09228	0.34609	0.34458	0.20508
0.14204	0.11659	0.17627	-0.03409	0.06393	0.12657	0.00697	0.09722	0.36182	0.34992	0.13469	0.08485	0.359	0.3011	0.41242	0.35995	0.15303
0.09369	0.1128	0.16923	0.0789	0.11883	0.1335	0.26967	0.07288	0.4095	0.33703	0.12219	0.03321	0.3449	0.2511	0.41012	0.35244	0.16042
0.11561	-0.12162	0.15457	-0.19273	0.1365	0.14326	-0.14347	0.10011	0.36674	0.35507	0.14055	-0.02136	0.35461	0.15909	0.39427	0.35795	0.15163
0.12814	0.04732	0.1653	-0.06521	0.01327	0.10606	0.16988	0.0661	0.33001	0.346	0.16449	0.07453	0.36225	0.21527	0.36559	0.35668	0.18483
0.1526	0.07153	0.14868	-0.06587	0.04446	0.08832	-0.03589	0.09001	0.18696	0.33262	0.12211	0.1322	0.34915	0.32267	0.40655	0.35937	0.14904

CED_BY	SING_BRA	ING_BRA	H2_TARG	H2_TARG	L4_SIGNA	_ANGIOG	OTHELIA	_VASCUL	_VASCUL	NG_MIDD	NG_MIDD	GING_OL	GING_OL	G_PREMA	MC_RESP	_BRCA1	1_BRCA1
0.09855	0.16548	0.15784	0.05799	0.13434	0.14515	0.14905	0.06932	0.01179	0.08625	0.05647	-0.15886	0.20365	0.07432	0.17208	-0.01229	0.27493	0.23461
0.01253	0.16903	0.11971	0.04591	0.10553	0.11277	0.13611	0.1756	0.19088	0.16887	0.06717	-0.05687	0.22271	0.11141	0.20183	0.01058	0.28492	0.26127
-0.00071	0.15359	0.14763	0.05832	0.11872	0.08984	0.17134	0.07688	0.10956	0.08454	-0.00396	0.02291	0.24657	0.20644	0.2108	0.00636	0.24741	0.24878
0.13503	0.15177	0.14938	0.0525	0.10967	0.12878	0.15498	0.10994	0.02897	0.12308	0.06086	-0.08487	0.21908	0.04111	0.15106	0.0153	0.22796	0.23805
-0.00473	0.14881	0.11722	0.04303	0.10398	0.10741	0.10005	0.0977	0.11789	0.07724	-0.0784	-0.08367	0.21545	0.00383	0.15975	-0.00076	0.20649	0.23809
0.00184	0.14853	0.14242	0.04472	0.10973	0.13697	0.10443	0.09692	0.1892	0.09867	0.01575	-0.04512	0.23992	0.1586	0.1932	0.05324	0.19146	0.2937
0.02717	0.1221	0.1407	0.03161	0.13624	0.10122	0.18801	0.16172	0.13164	0.16333	0.03912	0.08809	0.22021	0.21602	0.16215	-0.00705	0.22486	0.28661
0.03676	0.15963	0.1465	0.04363	0.09594	0.11595	0.18636	0.166	0.23448	0.16355	-0.09337	0.07023	0.20685	0.04929	0.16173	0.03195	0.22469	0.23201
-0.00344	0.15383	0.14963	0.04897	0.10364	0.1299	0.14875	0.14078	0.14267	0.11392	-0.00083	-0.03406	0.21422	0.10764	0.16376	0.01717	0.25006	0.22121
0.08958	0.1576	0.12478	0.04116	0.12228	0.12858	0.16934	0.15229	0.05558	0.16468	0.03406	-0.153	0.21774	-0.07211	0.14454	0.01951	0.23674	0.25179
-0.04472	0.15927	0.15397	0.05609	0.11634	0.10497	0.02666	0.04233	0.01759	0.05579	-0.0048	0.04478	0.24379	0.25853	0.19298	0.01369	0.27232	0.27669
-0.08755	0.15626	0.16183	0.04922	0.12881	0.08479	0.12474	0.1199	0.06536	0.11979	-0.04816	-0.07344	0.24209	0.12456	0.19949	0.02129	0.25643	0.2475
0.00871	0.15679	0.15255	0.04632	0.13071	0.08961	0.06675	0.00939	-0.03281	0.11579	-0.01704	-0.02312	0.22691	0.12016	0.21486	-0.01031	0.23764	0.27219
0.07075	0.14764	0.14017	0.04905	0.10374	0.10457	0.13889	0.11439	0.15562	0.10814	0.08335	-0.00615	0.23773	0.20095	0.16911	0.00903	0.22567	0.22067
0.04347	0.15957	0.1408	0.04919	0.11178	0.11694	0.14698	0.1046	0.1324	0.10096	0.06252	-0.07351	0.23873	0.1002	0.20118	0.03971	0.23879	0.25218
0.08914	0.14444	0.13493	0.04858	0.15048	0.08127	0.07241	-0.031	-0.13879	0.02393	-0.00137	0.01777	0.22442	0.24694	0.20667	-0.00412	0.26055	0.21778
0.07326	0.15536	0.13797	0.04484	0.10233	0.13589	0.16617	0.07927	0.01977	0.10563	-0.04108	0.01028	0.20364	0.05962	0.18366	-0.00992	0.22398	0.24432
0.06157	0.15471	0.14202	0.04468	0.1102	0.09835	0.05269	0.01982	0.07707	0.04422	-0.07738	-0.06159	0.2073	0.14786	0.13234	0.02658	0.24336	0.24216

_DPPA4_T	A_ATF2	PLE_MYEL	PLE_MYEL	E_MYEL	E_MYEL	MORPHO	ATINIB_R	ATINIB_R	SPONSE	SPONSE	IA_VHL	HL_TARG	HL_TARG	ESSED_B	SPONSE	SPONSE	D_STEM
0.05417	-0.05987	0.16373	0.15847	0.12051	-0.07497	0.03847	0.04188	0.13915	0.13366	0.01103	-0.06526	-0.02106	-0.01886	0.20389	0.29348	0.27199	0.07584
0.04469	0.00982	0.15245	0.1962	0.12585	-0.13394	0.11649	0.04558	0.09096	0.13334	0.00784	-0.08919	-0.01436	0.01555	-0.02537	0.30685	0.26719	0.17115
0.08999	0.03846	0.17654	0.17909	0.14674	-0.10299	0.09023	0.12651	0.25952	0.15312	0.01542	-0.12326	-0.07981	0.11646	-0.05147	0.29319	0.2851	0.09042
0.10786	0.03558	0.19144	0.20897	0.09738	-0.11539	0.10829	0.02998	0.20855	0.1536	0.0133	-0.011	-0.03614	0.11909	0.09981	0.29846	0.27183	0.03843
0.10777	-0.02091	0.20337	0.22535	0.09386	-0.0384	0.02707	0.01148	0.16803	0.12276	-0.0016	-0.14732	-0.06527	0.02122	0.11347	0.30438	0.28816	0.06117
0.0434	0.01591	0.2097	0.20925	0.05171	-0.06293	0.09316	0.05157	0.26023	0.13958	0.02332	-0.13306	-0.02062	0.04998	0.21013	0.29089	0.28076	0.02139
0.04857	0.07254	0.21031	0.17934	0.0926	-0.10494	0.08361	-0.15026	0.03314	0.13796	0.04396	0.04981	0.02575	-0.16722	0.10031	0.28414	0.28732	0.07346
0.18304	0.04307	0.20294	0.24609	0.06857	-0.11	0.07849	0.21215	0.2988	0.14179	0.03546	-0.0567	0.0143	0.10591	0.07472	0.28436	0.27516	-0.04246
0.13554	-0.02267	0.24036	0.23161	0.08343	-0.05739	0.03772	0.16542	0.30616	0.13618	0.09656	-0.16279	-0.06751	0.12948	-0.06358	0.28608	0.28972	-0.01985
0.10651	0.04759	0.19176	0.22637	0.0526	-0.12471	-0.00215	0.08047	0.14107	0.12204	0.03868	-0.05282	-0.06503	0.07107	0.08065	0.28705	0.27797	0.05959
0.03308	0.03931	0.19262	0.20005	0.09909	-0.03682	0.10981	0.07339	-0.12688	0.14178	0.04134	-0.21359	0.05842	-0.02274	0.07172	0.29718	0.27994	0.04037
0.02439	0.02225	0.206	0.20099	0.10359	-0.09039	0.02846	0.06366	0.01063	0.15289	0.06243	0.0228	-0.00103	-0.04291	0.28644	0.30029	0.27067	0.07219
0.00503	0.05785	0.12386	0.1702	0.12066	-0.09676	0.02288	0.00056	-0.15815	0.15984	-0.03076	-0.1743	-0.00296	-0.02869	0.16762	0.30208	0.25361	-0.08605
0.10117	0.00138	0.19941	0.22539	0.09152	-0.1361	0.06257	0.09877	0.22904	0.15601	0.0276	-0.10041	0.00914	0.05121	0.10108	0.29742	0.29563	0.01946
0.14041	0.03315	0.15173	0.21382	0.08646	-0.13665	0.056	0.12903	0.16935	0.13659	0.02551	-0.16442	0.0575	0.15532	0.16279	0.29946	0.25958	0.06473
0.01072	0.0346	0.13033	0.20442	0.09312	-0.11251	-0.03024	-0.11277	-0.07243	0.15291	0.03466	-0.21382	-0.02726	0.03961	0.14712	0.29963	0.21713	0.00776
0.10171	0.00741	0.19961	0.20762	0.09687	-0.14565	0.13924	0.01162	0.02601	0.15655	-0.00075	-0.02691	0.01064	0.03812	0.12676	0.2927	0.29869	-0.00833
0.12267	-0.0151	0.20522	0.23377	0.09676	-0.07906	0.05898	0.13225	0.29961	0.13464	0.04707	-0.14168	-0.00117	0.14171	0.09594	0.29163	0.28863	0.1135

O_HYPOX	O_HYPOX	ORT_VS	ORT_VS	_HEALTH	_HEALTH	VS_HEAL	VS_HEAL	SE_TO_A	SE_TO_A	ECTEDIN	ECTEDIN	ESS_RE	D_BY_HI	D_BY_HI	UTYRATE	UTYRATE	UTYRATE
0.08782	0.00939	0.02071	0.09891	0.03485	0.06741	0.06292	0.11952	0.23459	0.12488	0.16182	0.18987	-0.01814	0.05571	0.05863	0.29683	0.15986	0.0354
0.11392	-0.02138	0.0283	0.10003	0.02327	0.04784	0.05051	0.12987	0.28128	0.1161	0.17705	0.20253	-0.0522	0.03404	0.00513	0.18131	0.09582	-0.03318
0.12448	-0.0063	0.02132	0.10257	0.01344	0.08346	0.05092	0.13119	0.21313	0.13497	0.20592	0.19594	-0.02258	0.05951	0.00894	0.19312	0.03726	0.02512
0.07095	0.0152	0.03054	0.09876	0.00977	0.0652	0.06229	0.12327	0.23159	0.12053	0.2026	0.24925	-0.05934	0.07287	0.01583	0.22442	0.14248	0.04801
0.08894	-0.01374	0.03668	0.09141	0.03119	0.05961	0.08186	0.09963	0.2515	0.12621	0.16603	0.22343	-0.06136	0.03137	0.03165	0.21942	0.11728	0.03981
0.12298	0.00726	0.00345	0.09845	0.02241	0.05874	0.03696	0.10329	0.27557	0.09725	0.18288	0.22325	-0.06388	0.04282	-0.00019	0.12586	0.13018	-0.0978
0.12552	0.01395	0.00385	0.09804	0.00259	0.08227	0.02849	0.12598	0.19561	0.14814	0.2171	0.24789	-0.02558	0.05326	0.01539	0.15641	0.07509	-0.06487
0.0435	0.0261	0.0558	0.10552	0.02093	0.08063	0.08837	0.1123	0.23514	0.11077	0.19272	0.24021	-0.02931	0.0548	0.02084	0.22937	0.13397	0.0564
0.05262	0.01953	0.06311	0.10611	0.0329	0.08552	0.0944	0.12489	0.24961	0.11255	0.19214	0.19936	-0.04024	0.07785	0.0131	0.20433	0.1579	0.02344
0.06771	-0.01172	0.02617	0.10046	0.00526	0.0732	0.07156	0.12791	0.21535	0.13567	0.20053	0.22494	-0.05626	0.0444	0.02655	0.21465	0.11522	0.06865
0.1258	-0.01858	0.01712	0.05766	0.01846	0.03342	0.04041	0.0927	0.22386	0.11712	0.20304	0.23105	0.03114	0.08634	0.01964	0.15639	-0.03764	-0.07834
0.12036	0.00032	0.00685	0.11091	0.01748	0.07428	0.02794	0.13064	0.2439	0.16701	0.19882	0.27184	-0.03027	0.06899	0.02228	0.17603	0.07387	-0.04232
0.11428	-0.0068	0.00536	0.11084	0.02912	0.05217	0.04225	0.12198	0.27097	0.15447	0.19694	0.2309	-0.04541	0.01834	0.00582	0.10964	0.05376	0.04174
0.07558	-0.00607	0.03772	0.08668	0.01398	0.06726	0.06728	0.09279	0.23907	0.13028	0.18699	0.20143	0.03226	0.06868	-0.00285	0.186	0.08685	0.00095
0.11283	-0.00465	0.03587	0.10023	0.0345	0.07641	0.07659	0.11827	0.25347	0.11678	0.20133	0.24776	0.00725	0.04733	0.01693	0.16819	0.12284	-0.07307
0.16447	-0.02387	0.00553	0.08735	0.00479	0.06888	0.01954	0.11527	0.19803	0.11535	0.22567	0.23967	-0.07574	0.01615	-0.00423	0.07043	0.0681	-0.01076
0.06693	-0.01543	0.03304	0.08496	0.02108	0.05906	0.06019	0.10429	0.25056	0.13071	0.18564	0.2399	-0.02939	0.04823	-0.01437	0.22383	0.15165	0.03818
0.0644	-0.01408	0.05701	0.1042	0.04657	0.06865	0.08638	0.14505	0.22766	0.14843	0.18844	0.20511	-0.03261	0.03935	0.01492	0.22495	0.11622	0.04443

SE_TO_B	SE_TO_B	SE_TO_C	SE_TO_C	B1_ACUT	B1_ACUT	1_CHRON	1_CHRON	D_NON_H	DAC_TAR	DAC_TAR	INFECTI	INFECTI	_BY_E2F	O_BY_FO	BY_FOXP	_CORE_D
0.13646	0.18445	0.10767	0.08088	0.12898	0.14976	0.04349	0.04559	0.37394	-0.09103	0.03876	0.13036	0.00837	0.10493	0.09626	0.10046	-0.00926
0.16011	0.22955	0.06652	0.0849	0.09342	0.1759	0.04722	0.06134	0.36036	-0.14992	0.03844	0.14018	0.05055	0.12177	0.09138	0.10041	-0.01713
0.0786	0.20008	0.10915	0.08193	0.12898	0.18818	0.07568	0.11088	0.35875	-0.14781	0.027	0.14223	0.01668	0.13161	0.10068	0.10584	-0.03007
0.11815	0.20056	0.05054	-0.02039	0.17645	0.14902	0.13675	0.0757	0.34819	-0.16726	0.06486	0.15498	-0.05429	0.09354	0.10001	0.10601	0.00382
0.15621	0.21274	0.05963	0.04373	0.15025	0.14391	0.11368	0.05953	0.34734	-0.05221	0.05918	0.1942	0.06646	0.09499	0.11073	0.11804	0.1196
0.14758	0.22681	0.08906	0.02815	0.12546	0.19379	0.10732	0.09165	0.34662	-0.10849	-0.01122	0.20575	0.06296	0.12076	0.09591	0.1074	0.0445
0.14891	0.19994	0.10215	0.08068	0.15018	0.16936	0.11306	0.09539	0.34888	-0.06717	0.05047	0.15576	0.08544	0.112	0.08782	0.09752	0.03563
0.13737	0.18959	0.06455	-0.03815	0.18732	0.11736	0.14895	0.04815	0.32817	-0.11176	0.03245	0.13365	0.00124	0.07467	0.10895	0.10962	0.03857
0.11555	0.18579	0.04833	-0.03457	0.19329	0.13319	0.17167	0.07208	0.32727	0.12094	0.05141	0.18298	0.00817	0.0832	0.11358	0.11256	0.1044
0.17937	0.20511	0.08132	-0.03523	0.19164	0.13164	0.14129	0.05149	0.3311	-0.12291	0.07398	0.18032	0.05614	0.08665	0.11267	0.12043	0.14944
0.06665	0.20157	0.09768	0.07991	0.09383	0.18411	0.04212	0.10219	0.3676	-0.04221	0.0665	0.17995	-0.02682	0.12326	0.09514	0.09271	0.05133
0.09872	0.20382	0.13481	0.06452	0.13334	0.17394	0.06309	0.10666	0.36473	-0.05751	0.02684	0.19933	0.04985	0.11178	0.09583	0.10055	0.02028
0.0472	0.20705	0.11659	0.14323	0.03274	0.18641	-0.02469	0.09789	0.3806	-0.15718	0.08914	0.16606	0.02634	0.13483	0.08106	0.09388	-0.02073
0.06963	0.19657	0.06017	-0.0004	0.128	0.1376	0.1052	0.06126	0.35906	-0.13866	0.01355	0.13794	0.00367	0.10093	0.102	0.10383	0.00908
0.1136	0.21044	0.10145	0.03264	0.11423	0.1765	0.04451	0.07527	0.3656	-0.19677	0.0464	0.1771	-0.013	0.11114	0.08837	0.0958	-0.01448
0.0767	0.18853	0.10262	0.14081	0.0419	0.19081	-0.03776	0.10084	0.37966	-0.09503	0.07009	0.10058	-0.09231	0.13057	0.08582	0.08737	-0.0445
0.1202	0.20485	0.07584	0.07273	0.13163	0.14401	0.09407	0.03904	0.33871	-0.18984	-0.01059	0.12962	-0.00285	0.09645	0.09886	0.1015	-0.01952
0.12914	0.19396	0.072	-0.00456	0.17446	0.12794	0.11985	0.04773	0.33867	-0.05936	0.02837	0.1411	-0.02519	0.09094	0.10577	0.11043	0.06038

OXP3_TA	TARGETS	TARGETS	OXP3_TA	D_BY_PM	TINOIN_R	TINOIN_R	MATURE_N	MATURE_N	_AND_TP5	AND_TP5	RB1_TAR	RB1_TA	PONSE_T	NSE_TO	NSE_TO	TP53_TA	TP53_TA
-0.01225	-0.08612	0.08884	0.19313	0.06931	0.20716	0.04	0.2012	0.33672	0.0951	0.09921	0.10557	0.09177	-0.04012	0.10923	0.26715	0.09775	0.09464
0.02969	-0.26391	0.11294	0.16157	0.08391	0.20793	0.0172	0.14462	0.04648	0.11021	0.07846	0.11107	0.08115	-0.0545	0.11757	0.2587	0.106	0.08002
0.04616	-0.22613	0.20544	0.20636	0.09881	0.20115	0.02433	0.14779	0.20321	0.08323	0.09739	0.10609	0.08299	-0.03547	0.11022	0.25494	0.08582	0.09343
0.03076	-0.09518	0.24074	0.21924	0.12016	0.19606	0.05879	0.22116	0.27635	0.08634	0.11222	0.11209	0.09443	-0.04887	0.09557	0.27614	0.09652	0.10165
0.07052	-0.21646	0.24044	0.22404	0.12067	0.19727	0.01189	0.20361	0.34922	0.07846	0.10382	0.10368	0.08594	-0.05831	0.09569	0.26108	0.08094	0.10004
0.10404	-0.23089	0.17711	0.17549	0.0721	0.20556	0.02898	0.12917	0.12949	0.09156	0.10526	0.10155	0.09728	-0.08136	0.10525	0.25591	0.09314	0.10151
0.0972	-0.27366	0.19085	0.20389	0.10265	0.21107	0.04498	0.06768	0.14934	0.0898	0.09581	0.10215	0.08785	-0.04868	0.09214	0.26255	0.09422	0.09012
0.03457	-0.10811	0.25552	0.24618	0.12978	0.18565	0.00717	0.25274	0.32952	0.06892	0.11827	0.11091	0.08815	-0.04427	0.08416	0.27401	0.07857	0.10845
0.04222	-0.12688	0.24679	0.25795	0.12831	0.18286	0.0158	0.27901	0.23556	0.07223	0.10831	0.10652	0.08506	-0.06352	0.07943	0.27211	0.07951	0.10167
0.04992	-0.19041	0.26789	0.24525	0.12765	0.19646	0.01794	0.24433	0.32188	0.06313	0.10987	0.10591	0.07814	-0.06602	0.08616	0.2713	0.06931	0.10304
0.03119	-0.27793	0.07132	0.19117	0.05578	0.2103	0.05312	0.06357	0.38848	0.1015	0.08996	0.09952	0.09369	-0.05252	0.10773	0.24037	0.10439	0.0843
0.03423	-0.2053	0.13893	0.19615	0.07466	0.21224	0.0309	0.0548	0.20904	0.08482	0.09874	0.10316	0.08534	-0.02452	0.10175	0.25072	0.0934	0.08915
-0.01871	-0.28279	0.04691	0.13772	0.02726	0.2105	0.02304	-0.16038	-0.03424	0.09939	0.06818	0.09413	0.0787	-0.03516	0.11099	0.24494	0.09751	0.06784
0.02356	-0.24367	0.1366	0.21876	0.10481	0.20562	0.02552	0.2067	0.29261	0.08783	0.10674	0.10845	0.09338	-0.03345	0.08484	0.24205	0.09236	0.1015
0.08558	-0.2399	0.17859	0.18509	0.08573	0.20967	0.03742	0.185	0.43577	0.09372	0.10079	0.10974	0.0903	-0.01188	0.10062	0.25732	0.09728	0.09859
0.03049	-0.30264	0.1227	0.14972	0.03351	0.22174	0.04697	-0.03547	-0.00137	0.10447	0.07454	0.10287	0.07969	-0.02557	0.1102	0.24853	0.10539	0.07055
0.05176	-0.14772	0.20133	0.21428	0.11214	0.21326	0.03567	0.172	0.27147	0.09086	0.10356	0.11265	0.08761	0.00085	0.09043	0.27584	0.09315	0.0995
0.06995	-0.19532	0.24582	0.2334	0.12106	0.19454	0.0212	0.26178	0.14727	0.07184	0.10616	0.10852	0.07956	-0.07346	0.08582	0.27905	0.07941	0.0979

ERACT_M	FKB_TAR	FKB_TAR	GPCR_S	GPCR_
0.16976	0.11406	0.02319	0.10412	0.10999
0.1845	-0.10557	0.01695	0.10004	0.06144
0.17177	0.15381	0.06035	0.11557	0.10948
0.1455	0.16674	0.08179	0.08479	0.09716
0.14704	-0.01828	0.03857	0.07247	0.0617
0.14598	0.10677	0.12627	0.13107	0.09487
0.14897	0.0795	0.13965	0.13847	0.11485
0.1206	-0.02849	0.0946	0.07319	0.10265
0.11627	0.09499	0.10318	0.07432	0.09904
0.12902	0.00488	0.11858	0.07382	0.09468
0.16424	0.16096	0.06395	0.0807	0.08928
0.14807	0.07552	0.12454	0.10338	0.13609
0.1625	0.15946	0.02081	0.11353	0.09297
0.13395	0.05422	0.07727	0.03748	0.10609
0.15035	0.13552	0.06025	0.10612	0.11298
0.1737	0.07913	-0.05071	0.07887	0.08519
0.16063	-0.05772	0.06519	0.08042	0.09265
0.12642	0.05719	0.03852	0.06169	0.09878

IL2_SIGN	IL2_SIGNA	IFEN_AN	IFEN_AN	ESPONSE	OXIFEN_	OXIFEN_	_KILLER	WS_AP1_T	CARCINO	_VS_MUL	_MGUS	LE_MYEL	OMA_WITH	L_FOR_F	MULUS_	LY_ANTRA	RYONIC_	_FERTIL	ANTATION	_LUTEAL
-0.1004	0.03224	0.07569	-0.06763	-0.01487	0.03611	0.03616	0.12772	0.09794	0.1355	0.26963	0.2196	0.14932	0.07865	0.1564	-0.27081	-0.02713	-0.00987	0.24467	0.03883	0.11537
-0.15408	0.06141	0.07666	-0.0395	0.00976	0.04405	0.00673	0.12504	0.03658	0.0762	0.28679	0.21565	0.22888	0.13494	0.15355	-0.31446	-0.10604	0.16613	0.304	0.09194	0.10418
-0.07741	0.05587	-0.00544	-0.13207	-0.03019	0.05935	0.01275	0.12516	0.06905	0.10256	0.2823	0.24382	0.15542	0.16254	0.09821	0.06506	-0.13432	-0.04601	0.30608	-0.05301	0.11492
-0.07589	0.00196	0.18282	-0.04404	-0.02334	0.03871	0.02385	0.11963	0.08667	0.17379	0.27701	0.22641	0.17296	0.14144	0.10031	0.01759	0.0266	-0.09876	0.25803	-0.10788	0.14135
-0.01937	0.00726	-0.09258	-0.15893	-0.02497	0.0321	0.0005	0.11265	0.08248	0.14757	0.28214	0.23903	0.17855	0.11843	0.17128	-0.10957	-0.13469	-0.14132	0.24992	-0.11456	0.17694
-0.01245	0.04499	0.08211	-0.04888	0.00658	0.0246	0.00129	0.13068	0.09571	0.14618	0.23789	0.23574	0.20494	0.11152	0.10383	0.13563	-0.0122	-0.07532	0.17901	-0.03253	0.1524
-0.02925	0.08136	0.01767	-0.1934	-0.01313	0.04044	2.7E-05	0.12601	0.02575	0.09827	0.24245	0.23053	0.13636	0.08357	0.1059	0.08863	0.03204	-0.01869	0.41071	0.00214	0.0987
-0.05973	-5.2E-05	0.01184	0.043	-0.02206	0.04433	0.02838	0.1052	0.14873	0.16707	0.26577	0.21609	0.14795	0.1353	0.14049	0.04583	0.00968	-0.11405	0.23541	-0.10709	0.13568
-0.04402	0.02076	0.06312	-0.12333	-0.04242	0.03429	0.01675	0.12087	0.17722	0.15651	0.27554	0.21946	0.17619	0.1662	0.13415	0.11088	0.06052	-0.17157	0.26778	-0.02888	0.11078
0.01543	0.02196	0.00404	-0.14352	-0.05649	0.01959	0.00895	0.10426	0.09674	0.15266	0.28088	0.22625	0.12084	0.12579	0.13968	0.0519	0.01041	-0.19849	0.25382	-0.1097	0.17964
-0.0391	0.0689	0.1421	-0.12332	0.0202	0.08489	0.0312	0.12531	0.01577	0.11477	0.2322	0.25873	0.23183	0.1881	0.10635	0.08979	-0.02521	0.04289	0.31058	-0.0191	0.10935
-0.00339	0.04767	0.19602	0.01399	-0.01497	0.04225	0.02608	0.12151	0.06148	0.14026	0.26937	0.23831	0.1531	0.12106	0.10351	0.06582	-0.04816	-0.14008	0.22446	-0.0491	0.07696
-0.23536	0.05885	0.0616	-0.10135	0.06143	0.05628	0.01891	0.11682	-0.06659	-0.02747	0.2255	0.22444	0.225	0.03667	0.14696	-0.04356	-0.15931	0.11813	0.36907	0.04048	0.05325
-0.07169	0.01694	-0.00886	-0.03005	-0.00966	0.06967	0.03113	0.11742	0.04269	0.13176	0.29281	0.22737	0.18114	0.13336	0.10324	-0.10184	-0.03624	-0.0983	0.32156	-0.08524	0.16872
-0.10363	0.03678	0.09792	-0.01789	-0.02118	0.07494	0.02539	0.11265	0.11598	0.14068	0.28724	0.23893	0.16073	0.15162	0.11653	0.12985	-0.02138	0.0473	0.2483	-0.02264	0.12574
-0.14017	0.06086	0.14353	-0.0148	-0.02859	0.03498	0.02955	0.11993	-0.02165	-0.02841	0.26292	0.23327	0.22455	0.04033	0.13356	-0.22438	-0.13159	0.10048	0.36319	0.05834	0.05057
-0.05813	0.02644	0.06346	-0.10971	-0.02613	0.05426	0.01961	0.1085	0.03667	0.12759	0.30212	0.21867	0.19446	0.15194	0.12879	-0.0108	-0.09507	0.01156	0.31299	0.01702	0.10209
-0.047	0.02145	0.1107	-0.08318	-0.01578	0.04768	0.00382	0.11191	0.06598	0.15266	0.25576	0.21782	0.13451	0.12956	0.125	-0.08706	-0.04969	-0.10976	0.28982	-0.08585	0.09497

EPRODU	MATERNA	UTIC_AND	K_OVUL	ANTATION	OVULATO	MATID_DI	_SPERMA	SPERMA	X_SPERM	STEROID	PONSE_T	PONSE_T	DIFFERE	DIFFERE	AT_AGING	AT_AGING
0.13553	0.04636	-0.1843	-0.16825	0.06423	-0.01161	0.01243	0.08685	-0.10921	0.05097	0.24978	0.12665	0.07353	0.02333	0.18105	0.16762	0.24415
0.14478	-0.01134	-0.12028	-0.23085	0.10124	-0.04466	0.06365	0.14157	-0.06553	0.06133	-0.24062	0.14857	0.04488	0.03972	0.16384	0.17998	0.21648
0.11561	-0.01103	-0.19629	-0.18949	0.03738	-0.00205	0.07281	0.08764	-0.08737	0.03798	0.2415	0.13394	0.07459	0.02528	0.17047	0.1852	0.2349
0.09596	-0.12605	-0.21162	-0.13661	0.03643	-0.04954	-0.02228	0.05309	0.00596	0.02196	0.33504	0.14359	0.06336	0.07676	0.19249	0.20503	0.27688
0.0654	-0.11367	-0.13448	-0.11431	0.00556	-0.04542	-0.04865	0.06831	-0.00451	0.04829	-0.01691	0.12782	0.06003	0.05212	0.18076	0.1747	0.26469
0.12457	-0.04129	-0.08862	-0.20764	0.06403	-0.08234	-0.00934	0.13986	0.02651	0.01978	0.27867	0.13714	0.02752	0.08603	0.20697	0.17552	0.25436
0.09117	-0.00082	-0.20084	-0.28189	0.09069	-0.06326	0.01159	0.08152	0.02895	0.04655	0.1182	0.13756	0.02026	0.03024	0.22579	0.21444	0.27298
0.04632	-0.09561	-0.20563	-0.14505	0.02626	-0.08357	-0.05821	0.05406	0.00175	0.0405	-0.20204	0.11465	0.04423	0.09842	0.20832	0.21169	0.28992
0.04814	-0.08473	-0.18015	0.00064	0.01382	-0.07032	-0.07676	0.06415	0.04664	0.0271	0.09001	0.11171	0.05104	0.09951	0.2104	0.22335	0.29098
0.07217	-0.0352	-0.12661	-0.16205	0.03119	-0.08672	-0.06106	0.06397	0.07729	0.05542	-0.09019	0.10996	0.03459	0.10249	0.22619	0.19402	0.29118
0.07461	-0.04182	-0.14969	-0.269	0.05569	-0.0161	0.01575	0.14297	-0.00718	0.05439	0.4328	0.16316	0.05769	0.02717	0.17157	0.15641	0.2419
0.08981	-0.0443	-0.18507	-0.29082	0.07751	-0.0145	-0.06341	0.11944	-0.00566	0.06404	0.20169	0.14853	0.05923	0.04241	0.18423	0.18988	0.26813
0.09534	0.16431	-0.18125	-0.29968	0.1239	-0.00917	0.04975	0.15899	-0.03609	0.05295	0.46961	0.14299	0.07311	0.01953	0.20329	0.15957	0.19428
0.08333	-0.10844	-0.22138	-0.07823	0.03928	-0.0488	-0.01578	0.09067	-0.0921	0.03062	0.16488	0.12235	0.05322	0.016	0.18968	0.17963	0.26733
0.1214	-0.00599	-0.15266	-0.16431	0.03584	0.07011	-0.01924	0.09861	0.02759	0.03011	0.30914	0.11702	0.05026	0.03682	0.18787	0.19688	0.26322
0.13032	0.05348	-0.11814	-0.26347	0.06197	-0.00469	0.00661	0.13617	-0.02531	0.03608	0.15624	0.15385	0.04032	0.00018	0.14098	0.15298	0.20523
0.06996	-0.11544	-0.21234	-0.18308	0.03078	-0.03335	-0.03198	0.09343	-0.04233	0.0532	-0.39281	0.12929	0.06725	0.06839	0.16748	0.19393	0.24692
0.05332	-0.04117	-0.18277	-0.21751	0.00437	-0.05063	-0.04251	0.05465	-0.04932	0.04571	0.08684	0.12527	0.03668	0.04885	0.21959	0.20782	0.26725

RTAL_BR	RTAL_BR	RTAL_BR	RTAL_BR	RTAL_BR	RTAL_BR	RTAL_BR	RTAL_BR	RTAL_TG	RTAL_TG	RMINAL_E	RMINAL_E	BOUND_B	6_TARGE	6_TARGE	OXC6_TA
0.16613	0.13406	0.10146	0.11817	0.1108	0.10721	0.12272	0.11905	0.09756	0.16585	0.28783	0.06874	0.03401	0.06149	-0.03019	-0.0922
0.2232	0.07928	0.09003	0.09924	0.12375	0.10133	0.14598	0.10195	0.07452	0.16499	0.29638	0.02379	0.0239	0.00811	0.02289	-0.26112
0.2534	0.0799	0.10594	0.08465	0.11801	0.10073	0.14321	0.12902	0.12805	0.17484	0.30884	0.01885	0.03054	0.01866	0.01457	-0.15488
0.231	0.12	0.10522	0.0993	0.10508	0.08239	0.13791	0.13119	0.10904	0.17723	0.30221	0.02333	0.0327	0.01039	0.04767	-0.12171
0.23469	0.08768	0.10284	0.05872	0.08983	0.06741	0.09879	0.11852	0.10899	0.16137	0.32488	-0.14263	0.04072	-0.0094	0.01067	-0.10367
0.27398	0.08046	0.08	0.08775	0.09919	0.07172	0.14805	0.10937	0.13243	0.14982	0.37954	-0.08609	0.04144	0.00847	0.01031	-0.1842
0.26143	0.12164	0.09967	0.10471	0.08834	0.07117	0.17072	0.11229	0.11018	0.16885	0.39655	0.12519	0.02507	0.02359	-0.04466	-0.13231
0.24701	0.10981	0.11364	0.06307	0.09837	0.0566	0.11928	0.13182	0.11735	0.19222	0.29577	-0.13427	0.03839	-0.01569	0.06819	-0.02721
0.25246	0.09286	0.11342	0.06522	0.09333	0.07809	0.12404	0.13084	0.13199	0.17993	0.30476	-0.02667	0.03221	-0.02347	0.01729	-0.05176
0.2491	0.1145	0.10246	0.07466	0.08101	0.0658	0.1189	0.11336	0.12724	0.17032	0.32022	0.00892	0.03667	-0.01497	0.05097	-0.02512
0.2199	0.13083	0.11149	0.11773	0.12606	0.07338	0.12316	0.12403	0.10592	0.17151	0.31771	0.07616	0.04658	0.00844	-0.00245	-0.20538
0.25639	0.12046	0.11388	0.09049	0.08832	0.08961	0.16254	0.11283	0.10662	0.18199	0.33049	-0.04469	0.03389	0.0237	0.03777	-0.22237
0.1759	0.09844	0.09615	0.09012	0.11367	0.07744	0.12655	0.10917	0.10032	0.14691	0.21232	0.20177	0.00753	0.0487	0.02251	-0.19984
0.20889	0.10586	0.11163	0.08341	0.11346	0.08205	0.07902	0.12904	0.10255	0.16372	0.34425	-0.0555	0.03635	-0.02106	0.00284	-0.17428
0.2348	0.12653	0.10278	0.12044	0.103	0.10297	0.13721	0.11189	0.1667	0.18336	0.33481	-0.06994	0.03845	0.01591	-0.04311	-0.12235
0.24478	0.10718	0.10971	0.10798	0.11451	0.10306	0.12005	0.09539	0.08455	0.15262	0.28972	0.22644	0.0257	0.06801	-0.01108	-0.18142
0.24735	0.11262	0.1127	0.10988	0.1106	0.10039	0.12292	0.12783	0.1012	0.1665	0.30041	0.05022	0.0345	0.0096	-0.00024	-0.16923
0.25522	0.09308	0.10394	0.06556	0.08747	0.07181	0.13554	0.11657	0.10664	0.17263	0.32308	-0.10125	0.02883	0.00189	0.05484	-0.16706

OXC6_TA	OCAINE_	OCAIN_R	CREB1_T	CREB1_T	TA_FOSB	TA_FOSB	ANAMYCI	ANAMYCI	CUTE_LU	CUTE_LU	METHYL	RSP6_TA	RSP6_TA	_DENTAL	_DENTAL	_COOPER
0.21399	0.12548	0.08023	0.08215	0.13249	0.14153	0.24698	-0.19186	-0.3412	-0.01409	0.10512	0.04453	-0.30641	0.24805	0.04594	0.11689	-0.00382
0.15476	0.09669	0.07413	0.10937	0.10176	0.14264	0.24779	-0.40656	-0.43016	0.04761	0.05681	0.06851	-0.00789	0.27094	0.0077	0.07413	-0.07321
-0.03801	0.11862	0.10057	0.08664	0.07669	0.08061	0.2219	-0.44977	-0.46574	-0.0184	0.10686	-0.01719	-0.12665	0.25215	0.03982	0.14752	-0.00628
0.0806	0.10148	0.08555	0.06415	0.07778	0.12197	0.22784	-0.17168	-0.43051	0.07426	0.14934	0.0444	-0.23182	0.24439	-0.04023	0.22092	0.00641
-0.01368	0.10175	0.09325	0.11236	0.11672	0.10407	0.24935	-0.29582	-0.15484	0.02661	0.06668	0.06955	-0.16225	0.28527	-0.01177	0.18971	0.02999
-0.00381	0.09567	0.09938	0.10788	0.10782	0.06237	0.25994	-0.38724	-0.33979	0.08766	0.06986	0.09079	-0.04816	0.26321	0.02916	0.14214	0.02943
-0.03056	0.09242	0.08085	0.05111	0.11086	0.06964	0.20635	-0.32929	-0.41642	0.03258	0.14245	0.05696	-0.16153	0.23116	0.02963	0.16843	0.04038
-0.02867	0.10202	0.11939	0.07958	0.11372	0.11051	0.22157	-0.26562	-0.35142	0.06159	0.15542	0.0444	-0.34319	0.23315	-0.03522	0.23438	-0.01372
-0.03059	0.12265	0.10029	0.08011	0.09678	0.11203	0.24374	-0.45164	-0.33257	0.09138	0.10475	0.13212	-0.25309	0.28308	-0.00894	0.26072	-0.01625
-0.02213	0.09857	0.08536	0.0771	0.0766	0.07442	0.24031	-0.44124	-0.09107	0.07481	0.09957	0.06055	-0.18706	0.25427	-0.00539	0.22648	0.03495
0.10012	0.13181	0.08172	0.0607	0.14532	0.1408	0.22283	-0.30924	0.05989	-0.02015	0.08328	0.05659	-0.18135	0.2212	0.06674	0.07477	-0.01432
0.12727	0.10194	0.08468	0.08519	0.12932	0.16121	0.22522	-0.41914	-0.43438	0.01142	0.114	0.05481	-0.24737	0.30462	0.00178	0.1274	0.02121
0.07846	0.11874	0.08077	0.08284	0.16792	0.14951	0.22615	-0.31296	-0.45552	-0.04947	0.01993	0.023	-0.01628	0.10526	0.0579	-0.02103	-0.01879
0.03566	0.12484	0.07788	0.06658	0.13981	0.10821	0.22236	-0.1239	-0.22864	0.01265	0.11315	0.01195	-0.11847	0.25777	-0.01925	0.18176	0.02584
0.06668	0.10436	0.06466	0.08712	0.13712	0.13493	0.24281	-0.45645	-0.09935	0.03684	0.11734	0.02209	-0.16074	0.28789	0.00862	0.14691	-0.00824
0.12332	0.07882	0.05488	0.03237	0.12364	0.13594	0.21866	-0.30477	-0.46926	-0.01141	0.07027	-0.00387	-0.11998	0.23294	0.04301	-0.0212	-0.06933
0.12372	0.13619	0.0935	0.0766	0.11235	0.11112	0.22359	0.00412	-0.20345	0.02311	0.11598	0.03058	-0.20393	0.27282	0.04782	0.16902	0.06783
-0.03003	0.10768	0.09039	0.07972	0.09569	0.1179	0.24626	-0.35377	-0.35882	0.03366	0.09324	0.0262	-0.24555	0.25763	-0.01149	0.2015	-0.02825

_COOPER	ROGENIT	ROGENIT	_PROGEN	R_WNT_D	OR_WNT_	CTNNB1	R_WNT_	_PROGEN	R_WNT_U	OR_WNT_	CTNNB1	R_WNT_B	SMARCA4	N_CANCE	N_CANCE	N_HCP_
0.04376	-0.0134	0.06386	0.01252	-0.08975	-0.03797	-0.11946	0.0834	0.04689	0.09249	0.00724	-0.01867	0.06194	0.06301	0.24196	0.14241	0.05257
0.0182	-0.03403	0.07799	0.03187	-0.01799	-0.14034	-0.10492	0.09438	0.03742	0.07104	-0.01781	-0.07782	0.02745	0.01362	0.16633	0.09091	0.01917
0.10177	-0.01283	0.08499	0.06089	0.04403	-0.03318	0.00786	0.05764	0.04533	0.08794	0.01079	-0.00336	0.00896	0.0237	0.19931	0.10148	-0.00634
0.07441	0.05421	0.07132	0.09989	0.07487	0.14094	0.15597	-0.00767	0.06972	0.0914	0.02517	-0.02174	0.08358	0.01672	0.16175	0.19128	-0.00664
-0.00189	0.00143	0.05907	0.0844	0.0163	-0.07347	-0.0261	0.15073	0.02188	0.06728	-0.02484	-0.03828	0.01862	0.06416	0.15319	0.17134	-0.01416
0.00074	0.06245	0.08105	0.0738	-0.01597	0.00784	0.01852	0.04287	0.04008	0.09449	-0.01322	-0.00945	0.10812	0.01852	0.16873	0.10412	-0.00469
0.0433	0.04986	0.06649	0.04177	0.03533	0.04236	0.05214	0.02829	0.09345	0.12737	0.02088	-0.00487	0.18657	-0.02544	0.2249	0.06825	0.01285
0.09386	0.06005	0.03504	0.14204	0.07115	0.14217	0.16836	0.13122	0.04506	0.0692	-0.01665	-0.05331	0.13137	0.03023	0.19516	0.18099	-0.01651
0.09536	0.05406	0.03944	0.1101	0.07386	0.14441	0.17744	0.0349	0.06233	0.08651	0.00711	-0.03576	0.13388	0.03325	0.19695	0.17424	-0.04309
0.01604	0.04867	0.0476	0.11203	0.04818	0.09563	0.13878	0.13854	0.02438	0.06864	-0.05428	-0.07459	0.07159	-0.00062	0.16545	0.1653	-0.04577
0.06118	0.00596	0.09472	-0.04587	-0.05063	-0.19023	-0.10755	0.06249	0.04129	0.06164	0.02115	-0.03207	-0.08931	0.00273	0.20245	0.07218	-0.01529
0.11634	0.02972	0.08703	0.08275	0.01743	0.00682	-0.01607	0.08164	0.07705	0.11213	0.0351	0.00718	0.11629	-0.00977	0.17857	0.13399	-0.05072
0.043	0.00507	0.09423	0.0068	-0.0192	-0.24729	-0.17209	0.10195	0.02284	0.0701	-0.03905	-0.07572	-0.01367	0.02425	0.19059	0.17888	0.05106
0.06901	0.03313	0.06162	0.05429	0.00789	-0.00337	0.0543	0.15739	0.05307	0.10189	0.0327	0.04691	0.00524	0.01631	0.20902	0.18429	0.04048
0.07158	0.02764	0.08134	0.02919	-0.04295	-0.14553	-0.07855	0.13609	0.0461	0.08146	0.00098	-0.02754	0.06002	0.02287	0.24552	0.10526	0.04397
0.04308	-0.03904	0.09356	-0.0052	0.00227	-0.28008	-0.21726	0.04115	0.05149	0.08475	0.03895	0.00285	-0.09922	0.0444	0.21204	0.14881	0.07227
0.13731	0.00568	0.04976	0.06089	0.04227	-0.00974	0.02377	-0.00401	0.04349	0.09635	0.00335	0.00536	0.05973	0.0226	0.20291	0.17688	0.03349
0.03582	0.018	0.05948	0.07362	0.05254	0.02777	0.05513	0.00036	0.0567	0.08689	0.00663	-0.02969	0.02487	0.00714	0.18069	0.18484	-0.05955

N_HCP_	WITH_H3	WITH_H3	CP_WITH	N_ICP_	S_ICP_W	WITH_H3K	C_HCP_W	C_HCP_W	WITH_H3	WITH_H3	CP_WITH_	C_ICP_W	P_WITH_	TUT1_TA	TUT1_TA
-0.21031	0.05608	0.00476	0.25968	0.14052	0.08907	0.41182	-0.25012	-0.01002	0.01092	0.00973	0.04464	0.05245	0.12418	0.17875	0.21974
-0.26223	0.00499	-0.00426	0.29307	0.13931	0.0876	0.37439	-0.2366	-0.02063	-0.01065	0.01084	0.0073	0.06459	0.15597	0.17997	0.25231
-0.24026	0.02343	0.00054	0.35975	0.15304	0.11143	0.30985	-0.20143	-0.01616	0.00549	0.01995	0.02838	0.0643	0.14439	0.19985	0.24317
-0.21842	0.04537	0.01086	0.23548	0.10234	0.03993	0.46221	-0.04119	-0.02126	-0.02065	0.00128	0.04423	0.00952	0.07885	0.18987	0.24453
-0.09678	0.02268	-0.02323	0.23209	0.11851	0.03719	0.38661	-0.17473	-0.01361	-0.04857	-0.01286	0.01546	0.02606	0.04745	0.19544	0.24598
-0.15131	-0.01781	-0.02632	0.14714	0.15462	0.06886	0.46866	-0.23112	-0.01256	-0.04793	-0.03681	-0.00766	0.06861	0.11665	0.17677	0.24869
-0.38015	-0.01318	-0.00521	0.15795	0.17471	0.0713	0.46389	-0.18555	-0.02161	-0.01133	-0.03127	0.01286	0.04544	0.15994	0.17313	0.24338
-0.07546	0.04112	-0.00777	0.25653	0.11348	0.02645	0.44443	-0.16734	0.00018	-0.03673	-0.10747	0.04523	0.02626	0.0356	0.20223	0.22411
-0.1363	0.05428	-0.00251	0.2082	0.10611	0.01018	0.43963	-0.11466	0.01078	-0.06547	-0.05983	0.03148	0.02824	-0.00768	0.18774	0.24426
-0.16141	0.0104	-0.02313	0.20524	0.09572	0.00419	0.4727	-0.19751	-0.01579	-0.05564	-0.11523	0.01587	-0.00191	0.0588	0.1811	0.23357
-0.30415	-0.01553	0.00286	0.25705	0.18224	0.11296	0.28416	-0.10778	-0.03497	-0.00323	0.01593	0.02438	0.09653	0.09306	0.19854	0.25541
-0.24158	-0.01341	0.0024	0.04484	0.09867	-0.00842	0.43194	-0.20009	-0.00927	0.00601	0.05408	0.00092	-0.0135	0.03049	0.17282	0.23794
-0.26302	-0.05841	0.0183	0.22291	0.16286	0.15895	0.34725	-0.18149	-0.00101	0.01323	0.03514	0.0688	0.09962	0.24772	0.15156	0.23956
-0.27132	-0.01887	-0.00657	0.33978	0.12921	0.08903	0.39253	-0.11654	-0.02271	-0.03611	0.02357	0.05387	0.0367	0.15703	0.20141	0.23986
-0.23242	0.00655	-0.00964	0.35955	0.12687	0.07603	0.44574	-0.34071	-0.01673	-0.03609	-0.04483	0.03312	0.0232	0.16515	0.20704	0.23075
-0.41735	-0.03328	-0.00597	0.31572	0.18092	0.13275	0.34072	-0.17594	-0.0097	-0.01377	0.0211	0.04461	0.11926	0.14063	0.19205	0.26521
-0.14127	0.04226	0.00954	0.32525	0.13316	0.06978	0.4523	-0.0852	-0.01659	0.0108	0.06757	0.05957	0.03828	0.12885	0.18774	0.25295
-0.10492	0.08456	-0.00624	0.2689	0.12514	0.055	0.35344	-0.08035	-0.01125	-0.03203	-0.0225	0.05412	0.04469	0.04242	0.18886	0.22613

E_HYPOX	LIFIED_	STRESS_	STRESS_	IFFEREN	IFFEREN	S_HCP_W	P_WITH_	S_ICP_W	S_ICP_W	WITH_H3K	S_LCP_W	S_LCP_W	P_WITH_	S_ICP_W	WITH_H3
0.0687	0.13807	0.37987	0.41868	-0.4029	0.23551	-0.37493	-0.08221	0.00746	0.01092	0.09377	-0.44284	-0.02878	0.07054	0.13824	-0.00543
0.03377	0.11741	0.39947	0.41862	-0.39278	0.1189	-0.2959	0.04174	-0.04828	-0.0056	0.13361	-0.36304	-0.08262	0.18412	0.13477	0.06533
0.00616	0.0837	0.40967	0.40856	-0.28798	0.32975	-0.40519	-0.00196	-0.05651	-0.00134	0.10476	-0.46944	-0.0595	0.07032	0.15919	0.05247
0.05973	0.09668	0.357	0.42472	-0.2551	0.07064	-0.12001	0.07769	0.06515	0.01838	0.16637	-0.04016	-0.00733	0.15946	0.13433	0.02788
0.01656	0.11401	0.37536	0.41299	-0.2529	0.18672	-0.3314	-0.12956	-0.18673	0.00782	0.12045	-0.43967	-0.00872	-0.05487	0.10195	0.04352
0.05198	0.1332	0.41507	0.41698	-0.31577	0.25542	-0.29359	0.09229	-0.2697	-0.01642	0.1355	-0.35089	0.00354	-0.11906	0.1879	0.08801
0.03929	0.09415	0.401	0.40213	-0.08758	-0.10604	-0.35864	-0.04547	0.01901	0.00218	0.1607	-0.42223	-0.02759	-0.06304	0.26347	0.08002
0.06589	0.11908	0.37881	0.42432	-0.17962	-0.21631	-0.17279	-0.10496	0.0069	0.02233	0.13097	-0.10322	0.00967	-0.11115	0.06952	-0.00311
0.03371	0.1203	0.40524	0.42403	-0.17536	0.10234	-0.16621	-0.10232	0.09487	0.01615	0.09126	-0.15959	-0.00182	-0.05315	0.17727	-0.04961
0.01094	0.10857	0.36704	0.42041	-0.17574	-0.25841	-0.22855	-0.11324	0.11057	0.01042	0.12166	-0.2445	0.01866	-0.12093	0.13927	-0.00072
0.05559	0.13265	0.41757	0.4268	-0.4416	0.29945	-0.45634	-0.26397	-0.14253	-0.00038	0.0609	-0.47578	-0.05746	0.0201	0.13068	0.05703
0.03926	0.10911	0.40384	0.42384	-0.3788	0.28307	-0.40532	-0.11558	-0.06796	0.00808	0.03475	-0.43051	-0.00865	-0.07637	0.15737	-0.17081
0.03448	0.11484	0.39114	0.40641	-0.42559	0.26563	-0.46062	-0.08844	-0.02793	-0.01056	0.01432	-0.46098	-0.05792	-0.1099	0.19924	-0.00156
0.04081	0.12117	0.4119	0.41365	-0.23016	0.18742	-0.22791	0.03785	-0.05742	0.02311	0.15146	-0.32711	-0.01284	0.19176	0.13016	0.06631
0.045	0.11157	0.40522	0.42405	-0.32348	0.1795	-0.40473	0.05145	-0.06983	0.01303	0.14202	-0.46961	-0.05031	0.15416	0.16117	0.02291
0.05532	0.12547	0.40004	0.40799	-0.37574	0.33733	-0.44633	-0.15506	-0.0847	-0.00519	0.07463	-0.4483	-0.07565	0.12927	0.13909	-0.01692
0.03058	0.10195	0.38019	0.41504	-0.32531	0.15307	-0.36444	-0.02417	-0.00443	0.01545	0.13503	-0.32993	-0.00768	0.13244	0.15272	0.02424
0.0068	0.11578	0.39655	0.42045	-0.28494	0.29963	-0.14691	-0.29654	-0.15979	0.01785	0.10809	-0.44795	-0.00687	-0.07772	0.13894	0.02287

S_LCP_W	S_WITH_H	6_HCP_W	6_ICP_W	WITH_H3	6_LCP_	6_LCP_	F_HCP_W	CP_WITH_	F_ICP_W	WITH_H3	EF_LCP_W	EF_LCP_W	C_HCP_W	WITH_H3	C_ICP_W
0.00658	-0.29704	-0.07787	-0.06347	-0.05291	-0.00702	-0.00328	-0.08934	0.09297	-0.10102	0.01205	0.09788	0.02171	-0.04078	0.0764	-0.01031
-0.04418	-0.12799	-0.09775	0.02374	0.04432	-0.21008	-0.04616	-0.11014	0.09544	-0.05709	0.22489	0.10375	-0.00638	-0.04893	0.05487	-0.05427
-0.00884	-0.31741	-0.08032	0.06595	-0.11088	0.04273	0.00965	-0.04308	0.08812	-0.03201	0.0207	0.14021	0.03513	-0.01157	0.00986	0.0473
0.02381	-0.35699	-0.0795	0.06968	0.15958	0.06548	0.045	-0.06688	0.15372	-0.01644	0.15415	0.16172	0.07034	-0.00532	0.01508	-0.15536
0.0365	-0.07776	-0.09782	0.08615	-0.11366	0.18257	0.02343	-0.09223	0.09438	0.00027	0.04705	0.25627	0.05448	-0.06789	0.00474	-0.18637
0.04587	0.01094	-0.03882	0.09991	0.06848	0.05715	0.01686	-0.06877	0.06288	0.06857	0.1706	0.1628	0.07869	-0.07185	0.01122	-0.15094
0.02121	-0.11587	-0.06068	0.10617	0.0019	0.07281	0.04678	-0.07082	0.06116	0.07332	-0.00017	0.17168	0.09913	-0.02662	-0.04458	0.0783
0.05807	-0.23518	-0.06207	0.09835	0.1461	0.0609	0.0506	-0.08479	0.13336	-0.01927	0.1516	0.20206	0.10254	-0.0477	-0.03517	-0.37382
0.0471	-0.31008	-0.04835	0.11937	0.03123	0.10413	0.04499	-0.08717	0.10047	-0.00275	0.00695	0.20914	0.09419	-0.09281	-0.0527	-0.38833
0.07147	-0.31632	-0.0808	0.1357	0.05237	0.21307	0.08633	-0.11412	0.11058	0.03138	0.03693	0.24899	0.09038	-0.04318	-0.04916	-0.26151
-0.03648	-0.22715	-0.08829	0.03119	0.03548	0.09287	-0.04006	-0.04306	0.09097	-0.04527	0.16619	0.0603	0.00368	-0.01591	0.00296	-0.0194
0.00256	-0.28316	-0.10583	-0.01311	0.05789	0.08164	0.04107	-0.11715	0.04682	-0.04261	0.02896	0.09367	0.07575	-0.01493	0.05422	-0.00708
-0.02821	-0.41617	-0.09505	0.04482	-0.08842	-0.06163	-0.0315	-0.0725	0.1212	-0.03396	0.02861	-0.01279	-0.02281	-0.02885	-0.00578	0.01359
0.02316	-0.24272	-0.10746	0.05717	-0.03724	0.106	0.0574	-0.04069	0.13385	0.03057	0.05557	0.12975	0.06287	-0.02113	-0.01296	-0.09767
-0.02199	-0.20486	-0.08295	0.01133	0.08109	0.10873	-0.00481	-0.03126	0.11535	-0.02958	0.19674	0.13219	0.02112	-0.06819	-0.02538	-0.00589
-0.01196	-0.26886	-0.09349	-0.00646	-0.09169	-0.28441	-0.05288	-0.03671	0.08496	-0.08	0.05604	-0.00725	-0.00261	-0.02597	0.02248	0.08523
0.01922	-0.2742	-0.06478	0.03474	-0.08966	0.00172	0.02136	-0.01856	0.13352	-0.01513	0.03193	0.1955	0.03061	0.02252	0.06057	-0.04466
0.04131	-0.24001	-0.08884	0.07164	-0.10581	-0.03385	0.04592	-0.09577	0.11542	-0.03107	-0.06323	0.20382	0.08842	-0.02699	0.01471	-0.12763

C_ICP_W	C_LCP_	C_WITH_	EPROGRA	LURIPOT	LURIPOT	ENOMAT	ENOMAT	PSEUDO	PODIA_CH	PODIA_CH	PODIA_H	PODIA_H	CER_VA
0.02278	0.00728	0.28888	-0.11073	0.08977	-0.12172	0.09415	0.22847	0.07707	0.10195	0.10993	0.06184	0.08721	-0.16596
0.0205	0.00941	0.31672	0.10048	0.1297	-0.06183	0.17251	-0.05945	0.09067	0.11293	0.11464	0.07128	0.06899	-0.22803
0.00874	0.01459	0.2526	-0.02123	0.10624	-0.02466	0.16662	-0.02814	0.04029	0.11008	0.1029	0.07133	0.06565	-0.17811
0.02871	0.09179	0.31513	-0.24654	0.15157	-0.22371	0.16219	0.18212	0.03782	0.12433	0.11657	0.0758	0.06482	-0.12861
0.01095	0.07615	0.28413	-0.25458	0.07999	-0.08314	0.24624	-0.01645	0.04978	0.09415	0.12151	0.0475	0.07695	-0.21808
-0.01269	0.05241	0.36621	-0.16581	0.08063	-0.21983	0.32785	-0.19315	-0.00103	0.10285	0.07865	0.0552	0.06868	-0.07528
-0.00752	0.06893	0.27426	-0.2936	0.06546	-0.00599	0.2855	0.0068	0.01626	0.11237	0.08395	0.06006	0.06123	-0.12794
0.04026	0.1281	0.33592	-0.29298	0.062	-0.24608	0.19673	-0.02675	0.01505	0.12119	0.09634	0.08199	0.06094	-0.07694
0.03207	0.12166	0.38348	-0.25638	0.09693	-0.2038	0.22235	-0.28219	0.01466	0.11199	0.10026	0.07513	0.06156	-0.04763
0.01388	0.10119	0.39845	-0.32178	0.13754	-0.33433	0.19878	-0.02745	0.03689	0.10274	0.10304	0.05233	0.06694	-0.17189
0.01437	0.0371	0.18549	0.0254	-0.10394	-0.02554	0.1897	0.24623	0.08518	0.10068	0.12586	0.08175	0.06651	-0.15178
0.01681	0.07574	0.24238	-0.01966	0.00792	-0.09812	0.25624	0.18655	0.0705	0.10012	0.1216	0.05811	0.08182	-0.20266
-0.00256	0.04126	0.34191	0.02301	0.05276	0.03188	0.32317	0.06308	0.07612	0.09784	0.10839	0.07104	0.09718	-0.15931
0.02682	0.07643	0.29188	-0.26261	0.06078	-0.1226	0.20864	0.16905	0.04263	0.10789	0.10781	0.0788	0.07047	-0.21812
0.01021	0.02428	0.33645	-0.24383	0.06392	-0.01497	0.23905	0.24166	0.04641	0.09396	0.10836	0.05837	0.07997	-0.14518
0.01344	-0.02484	0.10093	-0.32038	0.02293	-0.10358	0.15605	0.20571	0.06517	0.09661	0.10749	0.05223	0.06902	-0.25628
0.02016	0.0783	0.25313	-0.03145	0.062	-0.03576	0.16623	0.17493	0.03846	0.1117	0.11703	0.06728	0.0796	-0.15877
0.02526	0.10669	0.40585	-0.31069	0.02254	-0.13634	0.21124	-0.18572	0.06057	0.10971	0.11672	0.07134	0.07203	-0.06286

C_THYRO	LATED_BY	ATED_BY	SPONSE_T	SPONSE_1	OF_EWS	OF_EWS	AMI_HYPO	AMI_HYPO	PHAGOS	LYMPH_H	CAMPUS	CAMPUS	OCAMPUS	R_HOXA	R_HOXA	S_OF_CC	S_OF_CC	IATION_T	IATION_T	CANCER_	CANCER_
0.04715	0.15001	0.11277	0.16415	0.00671	0.03092	0.01486	0.11379	-0.43129	-0.09715	0.19659	0.24386	0.30713	0.27337	0.06522	0.16108	-0.00057	0.00315	0.07963	0.06726	-0.29591	-0.33485
0.03572	0.17717	0.10201	0.18108	0.02295	-0.00479	-0.00018	0.22371	-0.41931	-0.13681	0.22903	0.2652	0.28054	0.33098	-0.01471	0.1647	-0.00186	0.031	0.06059	0.09772	-0.26057	-0.05816
0.07552	0.18854	0.0888	0.18152	0.0092	-0.02281	0.02654	0.33239	-0.4608	-0.09357	0.20678	0.24392	0.27834	0.31329	0.04415	0.17156	0.00053	0.0194	0.06326	0.08887	-0.3244	-0.35358
0.07954	0.14058	0.13267	0.1495	0.02599	0.02478	0.05153	0.39017	-0.4194	0.02082	0.16993	0.22088	0.29552	0.29802	0.0221	0.16135	-0.02708	0.01884	0.06978	0.05576	-0.31317	-0.17972
0.00862	0.16003	0.09882	0.15589	0.01703	-0.00839	0.03454	0.33046	-0.42856	-0.00012	0.19544	0.22963	0.29823	0.2947	0.01602	0.1501	-0.07145	0.04435	0.06725	0.05686	-0.39899	-0.37028
0.07743	0.17514	0.08132	0.17842	0.00017	0.00558	0.0265	0.01744	-0.05538	-0.15353	0.18714	0.2534	0.26627	0.30381	0.01084	0.16094	-0.00953	-0.00799	0.03967	0.07025	-0.33539	-0.08321
0.08986	0.17327	0.13284	0.16934	0.00915	0.01453	0.05994	0.19658	-0.09174	-0.12982	0.20241	0.24815	0.26431	0.28925	0.01366	0.15561	0.0299	0.05434	0.0563	0.0863	-0.3545	-0.3155
0.05411	0.1155	0.1003	0.11491	0.02557	0.05801	0.05131	0.41606	-0.42592	0.04058	0.16089	0.23563	0.28047	0.28111	0.02506	0.14953	-0.02647	-0.00376	0.07406	0.05284	-0.3524	-0.32635
0.04035	0.1251	0.10752	0.13301	0.03242	0.03584	0.05336	0.40321	-0.15227	0.04217	0.18679	0.24492	0.28859	0.27569	-0.00547	0.14384	-0.02074	0.02254	0.07384	0.04096	-0.36395	-0.15231
0.03529	0.13479	0.12719	0.13898	0.02161	0.01431	0.05995	0.34842	0.05948	0.03549	0.18278	0.21782	0.29432	0.2827	0.01508	0.14057	0.00379	0.03867	0.06367	0.03979	-0.40813	-0.36791
0.06321	0.19494	0.07287	0.18379	-0.00168	-0.01729	0.03959	-0.03717	-0.16116	-0.04962	0.21461	0.25188	0.26276	0.31923	0.08425	0.17822	0.0068	0.04604	0.06107	0.08566	-0.24479	-0.10432
0.08446	0.18157	0.10905	0.16856	0.01186	0.0098	0.02488	0.06007	-0.30936	-0.08753	0.20866	0.25549	0.28158	0.29971	0.08713	0.1537	-0.03853	0.06274	0.08122	0.06908	-0.31512	-0.22624
0.07311	0.19676	0.08089	0.18476	-0.00661	-0.05945	-0.02233	-0.20627	0.14379	-0.0868	0.23041	0.27263	0.29755	0.29133	0.05741	0.17817	-0.02713	0.06419	0.06402	0.08826	-0.19435	-0.1988
0.02798	0.13825	0.10852	0.15083	0.01732	0.00876	0.0374	0.33627	-0.33963	-0.05426	0.17508	0.23753	0.29092	0.30278	0.08497	0.16294	0.00351	0.00688	0.07418	0.06864	-0.33377	-0.43812
0.10505	0.15678	0.12415	0.16389	0.00545	0.03267	0.03513	0.2038	-0.39563	-0.02362	0.20844	0.26256	0.28471	0.31149	0.05059	0.16636	0.02285	0.02747	0.06657	0.08973	-0.40888	-0.12232
0.09174	0.19805	0.0876	0.17534	-0.02298	-0.05887	0.00119	-0.15942	-0.463	-0.07086	0.21469	0.27517	0.28509	0.30569	0.03344	0.1684	0.03426	0.0381	0.06817	0.10797	-0.27071	-0.20721
0.04937	0.13707	0.08243	0.13689	0.00947	0.00901	0.02716	0.3301	-0.13978	-0.03369	0.15598	0.25046	0.29489	0.29638	0.0426	0.15989	-0.04497	0.02488	0.08036	0.07002	-0.25315	-0.38792
0.019	0.11954	0.09453	0.14892	0.02398	0.01375	0.05576	0.33821	0.00331	0.01312	0.18351	0.23747	0.29314	0.28424	0.03308	0.14713	0.00596	0.06284	0.08766	0.04754	-0.35518	-0.39517

FFA_OX	LUCONE	COGEN_HA	GLYC	MAN_MIT	MITOCH	OTHA_P	OTHA_P	OTHA_R	OTHA_T	THA_VOX	CYTE_MAT	CYTE_MAT	LE_MYEL	LE_MYEL
0.24742	0.26259	0.17498	0.28754	0.13618	0.13245	0.16377	0.10797	0.35095	0.32512	0.20397	0.14555	0.07888	0.11778	0.00637
0.17946	0.25042	0.10965	0.28743	0.12616	0.12492	0.15141	0.0515	0.3645	0.29558	0.19261	0.15226	0.08824	0.11883	0.02004
0.23729	0.24023	0.1352	0.27633	0.14148	0.13441	0.14641	0.08989	0.37734	0.3395	0.18027	0.14024	0.09227	0.11621	0.02079
0.22334	0.28065	0.13403	0.32052	0.11447	0.10905	0.13713	0.01208	0.37291	0.27797	0.18401	0.12705	0.10631	0.09753	0.01485
0.21312	0.28285	0.14683	0.32266	0.1116	0.11112	0.1464	-0.02935	0.38838	0.28598	0.15458	0.13167	0.11609	0.1063	0.02412
0.21391	0.20654	0.11047	0.27491	0.11348	0.11027	0.14219	-0.04791	0.34178	0.27909	0.17805	0.12173	0.09253	0.11011	0.0081
0.23594	0.24481	0.09327	0.28226	0.14293	0.14042	0.15793	0.05404	0.37464	0.32924	0.19953	0.13545	0.10121	0.10178	0.01861
0.2307	0.28222	0.1267	0.31426	0.10726	0.10255	0.13378	-0.00775	0.39431	0.27907	0.18135	0.10071	0.12559	0.08187	0.03431
0.21197	0.28384	0.10882	0.32036	0.09695	0.09747	0.12533	-0.01961	0.39633	0.2703	0.1802	0.10299	0.11227	0.08457	0.02302
0.23054	0.27229	0.12105	0.29937	0.11333	0.11002	0.1353	0.02251	0.39066	0.30216	0.20356	0.11575	0.1069	0.08719	0.01518
0.21742	0.25168	0.12415	0.30053	0.14905	0.14441	0.15922	0.0745	0.3436	0.31387	0.218	0.15569	0.07444	0.12589	0.00828
0.23873	0.2409	0.1124	0.27802	0.14982	0.14493	0.16255	0.12387	0.37622	0.33367	0.18664	0.1508	0.11473	0.12222	0.00265
0.18524	0.21226	0.07392	0.25036	0.09727	0.09829	0.14924	0.04822	0.28999	0.26054	0.12947	0.15123	0.0994	0.12767	0.01716
0.22547	0.26399	0.15828	0.30032	0.12309	0.12072	0.14268	0.08032	0.39823	0.31707	0.16269	0.11569	0.08532	0.09963	0.02248
0.2079	0.24602	0.17719	0.29923	0.11129	0.10575	0.14183	0.06552	0.39684	0.27139	0.1669	0.13368	0.10534	0.10941	0.02851
0.24264	0.25433	0.12989	0.30111	0.15766	0.15588	0.17126	0.16463	0.36518	0.37692	0.18536	0.17925	0.06503	0.14478	-0.00791
0.24774	0.27549	0.14484	0.32675	0.15111	0.14795	0.15756	0.05496	0.3768	0.34588	0.21797	0.11952	0.09886	0.10198	0.01387
0.23737	0.28088	0.14737	0.31098	0.12616	0.12423	0.14178	0.02058	0.39316	0.308	0.21093	0.12167	0.10809	0.09002	0.01162

SPONSE	ESPONSE	MPHOMA	MPHOMA	RE_B_LY	RE_B_LY	RE_BII_L	RE_BII_L	E_B_LY	E_B_LY	LASMA_C	LASMA_C	BI_LYMP	BI_LYMP	RE_BII_L	RE_BII_L
0.016	0.27463	0.11424	0.17675	0.16686	0.18587	0.1324	0.20483	0.16085	0.05118	0.01648	0.13474	0.07786	0.15732	0.24918	0.04064
-0.02228	0.27899	0.1295	0.20988	0.2139	0.14688	0.11341	0.23541	0.17908	0.02384	0.10242	0.14315	0.06029	0.19788	0.23389	0.07336
0.02263	0.2278	0.14312	0.18678	0.20353	0.19502	0.13969	0.23267	0.17022	0.04206	0.09658	0.13321	0.05098	0.19137	0.25524	0.0816
-0.00616	0.26237	0.13895	0.14983	0.17211	0.24702	0.18783	0.20936	0.16532	0.0743	0.09741	0.14059	0.06247	0.16578	0.24326	0.1057
0.03452	0.25934	0.11424	0.18705	0.17392	0.24731	0.1788	0.20679	0.15284	0.08709	0.0985	0.15019	0.08051	0.16736	0.25317	0.04687
0.13904	0.24211	0.09004	0.1954	0.20518	0.17891	0.15306	0.23916	0.16451	0.05483	0.08522	0.1541	0.08518	0.21587	0.25901	0.06147
0.07498	0.23148	0.14449	0.17352	0.18322	0.19853	0.13925	0.21082	0.14515	0.0564	0.08598	0.1326	0.07636	0.18461	0.26416	0.06782
-0.01485	0.24757	0.13834	0.13375	0.13664	0.24634	0.18643	0.17964	0.16926	0.08111	0.14651	0.17217	0.08115	0.14063	0.24841	0.10659
0.02441	0.2394	0.13368	0.1392	0.14722	0.27398	0.22693	0.18567	0.16327	0.11096	0.15378	0.15143	0.09675	0.14934	0.25348	0.08146
0.0647	0.23845	0.10868	0.13968	0.1425	0.25625	0.19115	0.17835	0.15288	0.08157	0.15588	0.13864	0.09408	0.14433	0.25731	0.09308
0.0016	0.2406	0.1259	0.1919	0.23244	0.10774	0.06606	0.23342	0.16219	0.01426	0.06897	0.14922	0.03103	0.22204	0.24607	0.05761
0.06423	0.24899	0.0965	0.16957	0.2049	0.16898	0.11486	0.22257	0.16828	0.03404	0.06199	0.1385	0.06936	0.18338	0.2464	0.07241
-0.03319	0.25418	0.13338	0.21078	0.2268	0.0584	0.02816	0.23391	0.15569	0.01507	0.07124	0.11779	0.02859	0.21178	0.2275	0.09278
-0.01175	0.25484	0.10597	0.15798	0.14951	0.23189	0.17779	0.18383	0.1476	0.06988	0.12306	0.15747	0.07944	0.13968	0.25038	0.07077
-0.01826	0.23605	0.10361	0.16931	0.18699	0.16846	0.13843	0.21426	0.15901	0.04623	0.06567	0.14102	0.06387	0.17603	0.25384	0.07423
-0.03405	0.25811	0.10297	0.18696	0.21571	0.07111	0.03965	0.22845	0.13937	-0.04158	0.07466	0.1074	-0.00101	0.19844	0.24117	0.00851
-0.02356	0.26502	0.10874	0.17127	0.13122	0.20624	0.15324	0.18626	0.14751	0.05834	0.08206	0.14451	0.06946	0.14162	0.24452	0.08632
-0.00859	0.24949	0.1388	0.14411	0.15907	0.23598	0.18846	0.18885	0.15143	0.08468	0.1107	0.1476	0.07609	0.14707	0.24573	0.0634

HUMERAL	UMERAL	IFNA_R	ONSE_TO	ARGETS	ARGETS	LATED_IN	ER_PLU	LING_VI	LING_VI	LL_SIGN	LL_SIGN	LL_SIGN	LL_SIGN	MUTATED	MUTATED	MUTATED
0.1517	-0.14264	0.01821	-0.06265	-0.12215	0.3036	0.12982	0.17291	0.12846	0.15276	0.03181	0.08974	0.06702	0.07941	0.15858	0.02101	0.24477
0.14756	-0.11945	0.0455	-0.03939	-0.03016	0.27641	0.09793	0.20793	0.04216	0.21014	0.02483	0.07598	0.06436	0.06784	0.16535	0.02532	0.26005
0.16519	-0.16392	0.12881	-0.15753	-0.02513	0.3164	0.05038	0.20442	0.04448	0.17223	0.03573	0.09684	0.06975	0.09715	0.16733	0.04653	0.25722
0.17947	-0.18194	0.14831	-0.06452	-0.04564	0.305	0.1384	0.16616	0.08597	0.14676	0.00639	0.13298	0.0496	0.12975	0.16006	0.02079	0.25187
0.14739	-0.18746	0.09815	-0.06065	-0.13204	0.32092	0.08723	0.17295	0.082	0.16639	0.02208	0.13424	0.05434	0.12625	0.17134	0.02638	0.24966
0.14783	-0.06739	0.07768	0.0364	-0.01101	0.273	0.08616	0.19202	0.0377	0.1753	0.02907	0.07813	0.06564	0.06831	0.15566	0.02033	0.24738
0.12283	-0.04403	0.21988	-0.04619	0.06274	0.22463	0.03063	0.19315	0.04033	0.20938	0.0262	0.09118	0.05914	0.08597	0.17332	0.03291	0.2588
0.16908	-0.19517	0.10369	-0.07663	-0.10225	0.26653	0.08395	0.13659	0.07859	0.10603	-0.00054	0.14919	0.03695	0.14238	0.17072	0.04404	0.25534
0.10028	-0.17167	0.21522	-0.11223	-0.06318	0.29974	0.05062	0.14505	0.06753	0.13304	0.02642	0.14882	0.05046	0.1419	0.16009	0.03149	0.25031
0.14161	-0.15568	0.25505	-0.10754	-0.01303	0.27239	0.08688	0.15171	0.04055	0.13129	0.02461	0.14776	0.0465	0.14335	0.17322	0.02915	0.25202
0.11844	-0.24554	0.01932	-0.00597	-0.11028	0.24093	0.15169	0.2077	0.13862	0.15285	0.02966	0.05207	0.06883	0.0406	0.17479	0.03653	0.23376
0.06251	-0.03386	0.17162	-0.06465	-0.04298	0.27108	0.13124	0.19014	0.09628	0.17013	0.04522	0.07386	0.05801	0.06205	0.16923	0.02692	0.251
0.1518	-0.01768	0.0239	0.02128	-0.01078	0.29391	0.11731	0.21171	-0.0649	0.16224	0.04118	0.03651	0.07003	0.02901	0.18593	0.05122	0.2647
0.1949	-0.12839	0.03732	-0.07272	-0.06898	0.28841	0.14702	0.16829	0.09487	0.15935	0.01601	0.10951	0.04554	0.09988	0.18312	0.0262	0.23938
0.16581	-0.19612	0.10279	-0.18268	-0.05696	0.26211	0.16467	0.19496	0.09283	0.20345	0.01697	0.09404	0.05081	0.08231	0.1762	0.03031	0.24735
0.11863	-0.13663	0.07079	-0.01656	-0.02691	0.27829	0.13776	0.21241	0.01928	0.23432	0.0543	0.03465	0.07536	0.02964	0.17446	0.06074	0.27821
0.2129	-0.164	-0.00879	-0.06479	-0.1118	0.2804	0.10659	0.1702	0.10752	0.14936	0.03404	0.10648	0.07128	0.10133	0.17169	0.02623	0.26089
0.15949	-0.17477	0.21715	-0.02055	-0.05758	0.29881	0.02604	0.15368	0.11148	0.13226	0.00998	0.13494	0.04707	0.13174	0.16932	0.02661	0.24821

MUTATED	PM1_SIG	PM1_SIG	TIPLE_M	TIPLE_M	V_RESPON	V_RESPO	UV_RESP	V_RESPO	V_RESPO	LENCE	MPLIFICA	PLIFICA	PLIFICA	PLIFICA	PLIFICA	PLIFICA
0.00906	0.12074	0.03363	0.0409	0.25939	0.13842	0.29757	0.16992	0.10364	0.18125	0.15965	0.09318	0.27582	0.15384	0.45457	0.1412	0.11585
0.01136	0.13689	0.03228	0.12721	0.28136	0.02492	0.30469	0.18248	0.15502	0.18016	0.14675	-0.02255	0.25737	0.24726	0.46457	0.16966	0.13993
0.04129	0.13309	0.05239	0.01721	0.26648	0.1796	0.25673	0.16336	0.09332	0.17384	0.11898	0.08402	0.24239	0.24372	0.4568	0.18781	0.10694
0.01965	0.1363	0.03809	0.04239	0.26734	0.21948	0.24514	0.18232	0.08769	0.17028	0.16694	0.2341	0.20276	0.27957	0.44639	0.1084	0.10953
0.03053	0.12875	0.04657	0.05493	0.26585	0.21611	0.22737	0.14895	0.05689	0.14201	0.16466	0.34279	0.25751	0.36657	0.44517	0.08917	0.05579
0.00724	0.11293	0.02994	0.06605	0.28532	0.1693	0.29453	0.20021	0.17478	0.19608	0.21103	0.20891	0.23121	0.35166	0.44826	0.12587	0.08812
0.03003	0.14097	0.04203	0.00092	0.29461	0.20655	0.24259	0.12172	0.03695	0.19272	0.26113	0.12718	0.22634	0.28487	0.42964	0.17103	0.08438
0.0322	0.12915	0.05173	0.0029	0.28286	0.20293	0.21121	0.12565	0.06533	0.14789	0.18303	0.30615	0.23483	0.30055	0.42884	0.0766	0.03551
0.02043	0.14453	0.0474	0.09084	0.2799	0.23486	0.23307	0.1723	0.10251	0.14351	0.08559	0.29857	0.24273	0.33628	0.43476	0.08023	0.03831
0.01208	0.13806	0.0377	0.06009	0.29463	0.22044	0.26994	0.18757	0.11706	0.16209	0.13451	0.3331	0.24021	0.35048	0.45031	0.09324	0.10999
0.03874	0.14315	0.04011	0.02345	0.26319	0.25453	0.26562	0.14864	0.15849	0.15848	0.20411	-0.16523	0.19044	0.18172	0.45534	0.11734	0.13801
0.01671	0.12944	0.03471	-0.06029	0.28134	0.22268	0.23747	0.17537	0.11736	0.16933	0.19465	0.12824	0.28096	0.30948	0.46321	0.10271	0.14639
0.05609	0.14951	0.04033	0.0158	0.26193	0.22113	0.30253	0.22279	0.14247	0.20347	0.1468	-0.35212	0.26008	0.11621	0.46165	0.0937	0.15677
0.02848	0.14385	0.0427	0.12355	0.27747	0.20948	0.22092	0.12427	0.08869	0.16646	0.18526	0.16822	0.22717	0.31833	0.46394	0.09667	0.09803
0.02201	0.14322	0.0404	0.00254	0.26894	0.19933	0.2567	0.18364	0.1206	0.17544	0.17783	0.15166	0.22761	0.21275	0.46305	0.11453	0.08695
0.04927	0.13601	0.05906	0.1026	0.25383	0.10956	0.28216	0.12465	0.14549	0.19674	0.17038	-0.40004	0.26402	0.12263	0.45018	0.18913	0.1149
0.02217	0.14091	0.04332	0.08889	0.27578	0.15921	0.28711	0.17723	0.11456	0.18159	0.14029	0.20028	0.23832	0.3034	0.44718	0.11756	0.12169
-0.00336	0.1239	0.04062	0.02036	0.26048	0.21854	0.30519	0.24469	0.19513	0.1501	0.15574	0.26739	0.21342	0.33391	0.43976	0.12395	0.06444

PLIFICA	PLIFICA	PLIFICA	PLIFICA	PLIFICA	PLIFICA	PLIFICA	PLIFICA	PLIFICA	PLIFICA	PLIFICA	PLIFICA	PLIFICA	PLIFICA	PLIFICA
-0.35029	-0.1954	-0.04633	0.16871	0.01691	0.07092	0.40497	0.15769	-0.29516	0.00247	0.1493	-0.34568	0.38794	0.06161	0.18185
-0.14761	-0.14003	-0.43615	0.16751	0.0084	0.13187	0.36498	0.17638	-0.42715	-0.02906	0.16923	-0.31157	0.34744	0.06707	0.23698
-0.27154	-0.16014	-0.46926	0.11236	0.04337	0.05232	0.45499	0.13381	-0.18568	0.01603	0.08195	-0.31992	0.35515	0.17009	0.24249
-0.36164	-0.17277	-0.01304	0.13636	0.05321	0.07805	0.34367	0.25314	-0.30974	-0.09864	0.12418	-0.32455	0.34939	0.11801	0.14715
-0.29428	-0.08555	-0.18813	0.12437	0.02014	0.01119	0.40585	0.19846	-0.13672	-0.05566	0.14277	-0.30442	0.28805	0.21011	0.15965
-0.28569	-0.25856	-0.35071	0.11789	0.02583	0.05241	0.37203	0.12239	-0.01222	0.03224	0.12957	-0.27892	0.29697	0.12292	0.20115
-0.10944	-0.28966	-0.42205	0.14311	0.04938	0.04116	0.44125	0.20533	-0.39378	-0.18179	0.13872	-0.36731	0.32516	0.13844	0.20573
-0.29658	-0.2996	-0.1721	0.11038	0.04047	0.00301	0.37097	0.21247	-0.20543	-0.01515	0.13394	-0.32919	0.26804	0.18267	0.13793
-0.22141	-0.22121	-0.43579	0.11515	0.04147	-5.6E-05	0.38718	0.19101	-0.17096	-0.13352	0.16099	-0.28352	0.26525	0.18562	0.15601
-0.26082	-0.29825	-0.4217	0.12391	0.09395	0.08297	0.38216	0.20125	-0.16216	-0.02854	0.1222	-0.30751	0.22676	0.19495	0.1586
-0.08786	-0.13965	-0.38964	0.07929	0.01345	0.09569	0.45816	0.15277	-0.05105	-0.18549	0.03357	-0.24906	0.35324	0.04368	0.18659
-0.23549	-0.36444	-0.46644	0.12954	0.02069	0.09258	0.44372	0.16095	-0.12011	-0.14303	0.16351	-0.29495	0.36789	0.08936	0.16845
-0.15603	-0.09383	-0.46081	0.15609	0.04657	0.13034	0.41554	0.17626	-0.34689	-0.00211	0.12269	-0.16114	0.35647	0.09005	0.17456
-0.26334	-0.12246	-0.00317	0.10519	0.02195	0.04637	0.36815	0.20355	-0.23137	-0.21948	0.08144	-0.35299	0.29102	0.10897	0.15352
-0.30637	-0.26334	-0.46944	0.12648	0.03924	0.04767	0.40831	0.17938	-0.35802	0.00264	0.12957	-0.2938	0.38292	0.08118	0.14692
-0.20207	-0.22932	-0.47208	0.15906	0.02384	0.07007	0.46697	0.19204	-0.08614	-0.12295	0.13205	-0.23356	0.36781	0.06659	0.20378
-0.35589	-0.14229	0.23956	0.1261	0.04354	0.07817	0.40867	0.18549	-0.25831	-0.15202	0.15574	-0.22857	0.34234	0.10905	0.18505
-0.12825	-0.20017	-0.3183	0.11373	0.03919	0.02364	0.42663	0.19181	-0.1645	-0.10763	0.10575	-0.36673	0.25538	0.13722	0.1524

EMENT_MI	A_COLLA	ORE_MAT	CM_AFFI	GLYCO	CM_REGU	A_MATRIS	ISOME_A	GSOC_O	ILY_META	TIC_BREA	GHLY_M	STATIC_M	IC_COLO	ME_MULT	LY_META	TIC_BREA
-0.13948	-0.24249	-0.07041	0.15373	-0.05109	0.08231	0.04196	0.09293	0.14452	-0.05706	-0.00044	-0.0637	-0.23629	0.05836	0.41308	-0.10221	0.07241
-0.09222	-0.23069	-0.09429	0.15534	-0.07051	0.02216	0.00513	0.05225	0.12745	-0.04396	-0.00951	-0.07202	0.02381	0.09801	0.41303	-0.14934	-0.00103
-0.15132	-0.1352	-0.10867	0.17865	-0.08876	0.06488	0.01958	0.077	0.1188	0.01708	-0.02716	-0.19821	-0.33382	0.07573	0.42752	-0.13332	-0.02342
-0.18473	-0.10806	-0.06805	0.19453	-0.06025	0.14918	0.07583	0.1374	0.1798	0.09249	0.0399	-0.01094	-0.16132	0.11011	0.45022	-0.04837	0.04316
-0.12285	-0.1612	-0.08665	0.13609	-0.07635	0.09189	0.03364	0.08972	0.14134	0.01417	0.00288	-0.0416	-0.28673	0.0556	0.45606	-0.12471	-0.00259
-0.07517	-0.11419	0.02277	0.15383	0.05495	0.14497	0.0908	0.12253	0.27376	0.12775	0.00697	0.11079	-0.04071	0.17758	0.43861	0.06761	0.16675
-0.01305	0.06665	0.08476	0.21331	0.1023	0.15296	0.13166	0.15223	0.29276	0.1568	0.05985	0.15067	-0.26338	0.21966	0.43515	0.0839	0.11106
-0.19395	-0.14225	-0.05694	0.1947	-0.04073	0.18549	0.09297	0.1561	0.20294	0.07463	-0.00741	-0.01154	-0.12153	0.13668	0.4563	-0.00415	0.07603
-0.06274	0.05538	-0.01366	0.17637	-0.03373	0.16829	0.09673	0.14615	0.21074	0.09335	0.01126	0.05332	-0.06039	0.13924	0.46549	-0.02518	0.03037
-0.21035	-0.17552	-0.02259	0.17318	-0.01015	0.16488	0.09072	0.14078	0.20327	0.08693	0.01074	0.06117	-0.37098	0.11212	0.45413	-1.1E-05	0.11586
-0.13902	-0.369	-0.07369	0.16756	-0.01893	0.08784	0.05097	0.10467	0.14961	0.03838	0.03781	-0.03802	0.04211	0.05857	0.40624	-0.07951	0.07446
-0.05686	-0.14902	-0.00476	0.1757	0.02609	0.1349	0.08674	0.12751	0.19424	0.1221	0.06889	0.01003	-0.10169	0.07573	0.42013	-0.01794	0.12296
-0.09113	-0.26273	-0.07752	0.16914	-0.04962	0.06133	0.03683	0.08793	0.13784	0.02636	0.01004	-0.07514	-0.09747	0.06052	0.36289	-0.01421	0.12823
-0.22353	-0.18052	-0.08877	0.16534	-0.07414	0.09119	0.05166	0.11248	0.14645	0.02766	-0.01573	-0.1078	-0.33763	0.06365	0.43543	-0.1089	-0.00347
-0.18262	-0.23701	-0.09483	0.14948	-0.07292	0.1235	0.06106	0.12776	0.16161	0.03784	0.02136	-0.04792	-0.03572	0.06065	0.41779	-0.06067	0.08279
-0.05391	-0.21616	-0.10211	0.17211	-0.08143	-0.00124	-0.00171	0.0451	0.09085	-0.03887	-0.01531	-0.00194	0.04272	0.01895	0.33497	-0.11829	0.02096
-0.09817	-0.20824	-0.06054	0.17933	-0.03565	0.10912	0.06283	0.1161	0.15674	0.01326	0.00659	-0.06493	-0.02551	0.07071	0.44045	-0.09957	0.03913
-0.17728	-0.17527	-0.07659	0.17565	-0.06275	0.08876	0.04079	0.0942	0.15145	0.0405	-0.00042	-0.10663	-0.29565	0.08586	0.45308	-0.09319	0.00376

ORLY_M	STATIC_M	Y_METAST	ROTEOG	RETED	RKAR1A	RKAR1A	T_CANCER	CANCER	RGLYCEM	R_OBESI	R_OBESI	EGF_SIG	RG1_SIG	RG1_SI	COMPON
0.17336	0.2365	0.04802	-0.03401	0.05102	0.01802	0.2189	-0.00649	0.01061	0.09539	0.26457	0.20787	-0.01704	-0.02112	0.00632	-0.27744
0.13309	0.21916	0.04367	-0.10484	0.01026	-0.22477	0.29304	-0.07223	0.02286	0.0831	0.21829	0.19228	0.02829	-0.04836	0.01711	-0.00123
0.14367	0.22903	0.05324	-0.20373	-0.00856	-0.10844	0.25713	0.05826	0.03105	0.09487	0.25442	0.23918	-0.03686	0.00535	0.01123	-0.14973
0.19048	0.22336	0.06835	-0.07021	0.03841	-0.16298	0.24237	0.10613	0.03247	0.10997	0.24305	0.29971	-0.03232	0.02585	0.00264	-0.48987
0.16866	0.22235	0.02026	-0.06813	0.03333	-0.25408	0.12624	0.08443	0.03718	0.09438	0.22779	0.24438	-0.04253	0.0084	0.00985	-0.38083
0.24632	0.26187	0.07252	-0.0519	0.02726	-0.15657	0.14837	0.17201	0.08238	0.06104	0.28419	0.20335	-0.03949	-0.01998	0.00271	-0.45993
0.26528	0.29275	0.17241	-0.01418	0.07681	-0.24161	0.21498	0.18897	0.06452	0.08469	0.28614	0.23583	-0.02071	-0.00129	-0.02224	-0.19817
0.21266	0.24298	0.10303	-0.06891	0.03035	-0.26928	0.18715	0.1204	0.01992	0.07805	0.25906	0.28493	-0.01456	0.02357	0.01464	-0.35882
0.21778	0.24615	0.07787	0.03547	0.0508	-0.15493	0.15798	0.11544	0.03204	0.09423	0.24826	0.30142	0.01938	0.04423	0.01392	-0.37943
0.1996	0.2261	0.05948	0.03813	0.04016	-0.37591	0.16107	0.13694	0.02149	0.09955	0.25114	0.27319	-0.02237	0.05324	-0.00806	-0.30315
0.17298	0.23726	0.07297	-0.2219	0.0729	-0.25429	0.27568	0.07793	0.07644	0.09373	0.2744	0.16825	0.00331	-0.00739	0.02189	-0.49392
0.2374	0.25722	0.05899	-0.06224	0.05054	-0.42544	0.29474	0.18032	0.06018	0.08188	0.26926	0.22361	-0.07009	0.00704	0.02201	-0.16899
0.19376	0.24051	0.0836	-0.09453	0.06106	-0.36589	0.25416	0.16186	0.06289	0.06305	0.25372	0.11104	0.02026	-0.06701	0.02431	-0.2755
0.17274	0.21971	0.04467	-0.07918	0.1046	-0.16535	0.26278	0.08006	0.03076	0.07886	0.24656	0.25937	0.00737	0.02513	0.0222	-0.43685
0.17339	0.19855	0.06766	-0.08458	0.1112	-0.04594	0.24754	0.08375	0.04832	0.09363	0.26153	0.20028	-0.04107	0.0295	0.02827	-0.30967
0.12656	0.21633	0.022	-0.11126	0.01395	-0.46908	0.2629	-0.03199	0.05842	0.1086	0.25595	0.09885	-0.02028	-0.04499	0.03224	-0.21279
0.16368	0.20514	0.05211	-0.08085	0.05834	0.03999	0.26997	-0.02165	0.01925	0.08991	0.24857	0.2727	-0.0256	-0.00192	0.018	-0.30597
0.16969	0.21004	0.03851	-0.06002	0.01207	-0.36816	0.20608	0.08712	-0.02705	0.09877	0.23838	0.28768	-0.03158	-0.0016	0.00954	-0.28589

COMPONE	TARGETS	MA_EOSIN	MA_MAST	POGENE	IPOGENE	IPOGEN	IPOGEN	LVEOLAR	R_MICRO	R_MICRO	RA_LUNG	ER_DIFF	IRA_MET	ETASTA
-0.33877	0.03958	0.04083	0.1418	-0.04845	0.09462	-0.02356	0.06033	-0.40541	0.12075	0.02673	-0.02895	-0.08302	0.10683	0.17
-0.13699	-0.08901	-0.03093	0.04314	-0.03097	-0.01967	-0.00213	0.0429	-0.39731	0.10657	0.06739	0.00465	-0.05964	0.12196	0.13787
-0.23628	-0.03263	-0.1784	0.09419	-0.0087	0.01133	0.04046	0.02137	-0.37008	0.12646	0.01579	0.1274	0.07356	0.09239	0.13734
-0.34806	-0.089	-0.10315	0.10314	-0.01208	0.04471	0.02519	0.06544	-0.32006	0.09265	0.12896	-0.03143	-0.03446	0.14198	0.19494
-0.1767	-0.04537	-0.00329	0.09468	-0.11048	-0.0457	-0.07721	0.02973	-0.31285	0.10889	0.02767	-0.00063	-0.25125	0.08275	0.16862
-0.27835	0.11818	-0.10074	0.10699	-0.04031	-0.01022	-0.00538	0.03737	-0.04498	0.14719	0.03626	0.1116	-0.1561	0.06563	0.14642
-0.27212	0.26457	-0.04132	0.10697	0.04187	0.00336	0.09451	0.03648	-0.01371	0.11778	0.11564	-0.08269	-0.1119	0.07839	0.16027
-0.20564	0.01486	0.00417	0.17156	-0.03772	0.0446	0.0007	0.0463	-0.24801	0.09089	0.08113	-0.19996	-0.21293	0.14128	0.20031
-0.19758	-0.0548	-0.03575	0.12422	-0.0447	0.06031	0.00118	0.05765	-0.29808	0.07449	0.10303	-0.17492	-0.23906	0.13699	0.18792
-0.21999	0.03957	0.00903	0.12608	-0.09485	0.00415	-0.04389	0.06005	-0.17923	0.07705	0.04061	-0.22597	-0.16617	0.12305	0.19811
-0.43412	0.08372	-0.064	0.11215	0.00491	0.00053	0.02988	-0.01218	-0.33917	0.15158	0.01277	0.27482	0.06621	0.04728	0.11498
-0.28025	0.01977	-0.1096	0.12581	0.09738	0.00854	0.12785	0.02895	-0.25395	0.13763	0.10794	0.09089	0.07277	0.07993	0.16485
-0.26511	0.16732	-0.2396	0.03498	-0.01054	0.01825	0.01619	0.0165	-0.42284	0.15496	0.01846	0.12153	-0.36777	0.02971	0.07927
-0.32479	-0.03763	-0.01535	0.13569	-0.03973	-0.01002	-0.01168	0.0264	-0.39674	0.10574	0.12144	-0.28364	-0.40922	0.10292	0.16686
-0.34688	-0.06489	0.03665	0.15931	-0.04361	0.03904	-0.00587	0.05582	-0.39973	0.11789	0.0231	0.02173	-0.35741	0.08264	0.16606
-0.32387	0.00315	-0.16707	0.05344	-0.02456	0.03514	0.01606	0.02472	-0.27573	0.15633	0.02522	0.13884	-0.08741	0.04692	0.07591
-0.32614	0.06178	-0.18152	0.1082	-0.04532	0.04098	-0.02185	0.05941	-0.3264	0.07604	0.04841	-0.33301	0.04119	0.11562	0.17463
-0.21776	0.09356	-0.06111	0.12066	-0.09137	0.05249	-0.04068	0.0375	-0.3041	0.07646	0.09172	-0.06662	-0.26582	0.13966	0.17026

TISSUE_T	TISSUE_T	TISSUE_T	TISSUE_TY	DS_TARG	E_TO_INT	E_TO_INT	ITH_FLT	ITH_FLT	NSE_TO_	NSE_TO_	ATORY_R	ATORY_R	RCC6_TA	RCC6_TA	ANT_GLI	OTCH1_TA	OTCH1_T	_TO_PRO	_CANCER	ANCER_
-0.07267	0.08203	-0.09084	-0.04659	0.27098	0.31611	0.10925	0.45144	0.02235	0.12529	0.11168	0.10742	0.11495	0.02871	0.30483	0.45665	0.1025	0.20106	0.1967	0.12258	0.11299
-0.10546	0.06085	-0.15647	-0.01121	0.29176	0.31296	0.11434	0.42937	0.06364	0.1703	0.08326	0.20591	0.11743	0.08968	0.30582	0.46111	0.116	0.22357	0.20156	0.09459	0.01947
-0.08814	0.13972	-0.09358	-0.04856	0.25306	0.29567	0.14116	0.44053	0.00931	0.12375	0.09896	0.23409	0.12536	0.05507	0.32218	0.45349	0.10888	0.21463	0.17773	0.14092	0.07568
-0.22317	0.23625	0.01962	-0.04143	0.22125	0.31595	0.12467	0.43823	-0.04219	0.13042	0.0989	0.19244	0.1803	-0.04622	0.34492	0.4522	0.08311	0.23721	0.19555	0.09122	0.06283
-0.1621	0.17781	-0.12251	-0.0881	0.22605	0.30354	0.12016	0.45219	0.02094	0.08882	0.06368	0.17972	0.16723	0.03853	0.35648	0.4535	0.07855	0.2201	0.18958	-0.01277	0.09317
-0.10262	0.16702	0.0517	-0.00758	0.21786	0.30421	0.12845	0.44896	0.02755	0.13996	0.11198	0.21529	0.1193	0.0004	0.26196	0.44748	0.07875	0.21703	0.18614	0.0443	0.07302
-0.09105	0.23171	-0.00083	0.00033	0.24155	0.30833	0.1528	0.44503	0.06592	0.18582	0.08105	0.20589	0.12285	0.13466	0.27624	0.45534	0.11969	0.22575	0.1924	0.22472	0.06623
-0.19825	0.23654	-0.0004	-0.06216	0.21298	0.30917	0.11794	0.44961	-0.02224	0.07883	0.11097	0.1676	0.19151	-0.07376	0.34218	0.45282	0.06456	0.18171	0.17206	0.07837	0.06822
-0.14287	0.2732	0.00935	-0.04401	0.20086	0.31073	0.13931	0.44339	-0.02322	0.12809	0.08443	0.1698	0.21753	-0.01792	0.37262	0.45928	0.06191	0.2297	0.18762	-0.028	0.06769
-0.14467	0.24738	-0.02862	-0.04486	0.22049	0.29943	0.15225	0.4407	-0.00364	0.15477	0.08573	0.14744	0.1827	-0.05436	0.36436	0.45141	0.08151	0.17083	0.20887	0.18212	0.07809
-0.05531	0.01841	-0.10406	-0.00918	0.21738	0.31466	0.11772	0.45201	0.01882	0.11535	0.10313	0.17831	0.09347	0.07286	0.22262	0.46212	0.09363	0.26761	0.12921	0.04977	0.08056
-0.10788	0.14108	-0.12893	-0.03768	0.24141	0.32397	0.14326	0.45341	-0.01801	0.11032	0.12192	0.21608	0.10176	-0.02424	0.23391	0.45989	0.08393	0.22168	0.15175	-0.02402	0.06933
-0.02105	-0.06678	-0.05284	-0.03904	0.2435	0.28789	0.10834	0.41273	0.09942	0.16697	0.10192	0.1673	0.05393	-0.019	0.29995	0.45464	0.07811	0.23133	0.16521	0.03156	0.07305
-0.19032	0.16758	-0.10455	-0.06668	0.2549	0.29602	0.11782	0.45147	0.00607	0.14784	0.08896	0.13737	0.15433	-0.01215	0.26091	0.45521	0.05946	0.21491	0.18665	-0.06782	0.07002
-0.12122	0.13988	-0.05552	-0.03989	0.22713	0.32314	0.10256	0.45803	0.01102	0.11857	0.13249	0.19699	0.12127	-0.0359	0.2941	0.45386	0.08883	0.26018	0.20762	0.11273	0.08072
-0.05632	-0.09155	-0.17662	-0.048	0.24896	0.3037	0.13946	0.40833	-0.03422	0.11858	0.09223	0.20763	0.05157	0.03244	0.21551	0.45816	0.12935	0.23484	0.15566	0.15844	0.11293
-0.12551	0.14549	-0.05625	-0.06187	0.24078	0.31223	0.10911	0.45191	0.0177	0.09202	0.08094	0.19375	0.12903	-0.0046	0.31674	0.46132	0.09248	0.23619	0.18697	0.16509	0.05837
-0.14928	0.2151	-0.05535	-0.04122	0.22685	0.30978	0.12269	0.44674	-0.02592	0.05181	0.08864	0.17668	0.16531	0.01069	0.37435	0.45622	0.08164	0.23023	0.18003	0.05386	0.09637

ANCER_1	_CANCER	_CANCER	_CANCER	_CANCER	_CANCER	_CANCER	ANCER_	ANCER_1	_CANCER	CANCER	T_CANCER	_CANCE	_CANCER
0.18682	-0.11117	-0.00494	0.101	0.02621	0.08971	-0.11203	0.02052	0.08818	-0.13635	0.2901	2.4E-05	-0.37001	-0.22775
0.1285	-0.01113	0.00067	0.05657	0.06152	0.04182	-0.08586	0.07271	0.08309	-0.25428	0.3739	0.03149	-0.32977	-0.03177
0.1265	-0.07766	0.04407	0.02749	0.04381	0.12349	-0.06575	0.04164	0.04072	-0.19629	0.34712	-0.08376	-0.36948	-0.00961
0.16484	-0.13015	0.04129	-0.04461	0.05568	0.1059	-0.07924	0.01057	0.08747	-0.18693	0.24629	-0.07413	-0.2849	0.00397
0.1396	-0.11267	0.10638	-0.01801	0.02663	0.10218	-0.07601	0.05332	0.05252	-0.14085	0.3089	-0.03904	-0.28704	-0.10845
0.16742	-0.12623	0.10911	-0.00301	0.05288	0.02858	-0.1295	0.07542	0.06997	-0.18024	0.29287	-0.01541	-0.18178	-0.24126
0.14133	-0.02648	-0.01114	-0.03726	0.05341	0.12028	-0.10054	-0.02184	0.10048	-0.22574	0.31281	-0.03162	-0.00871	-0.05685
0.16948	-0.14427	0.08618	0.13167	-0.00288	0.13057	-0.05771	0.02179	0.07672	-0.14107	0.2725	-0.09679	-0.28286	0.00739
0.14893	-0.15791	0.06919	0.0108	0.00368	0.14588	-0.06515	0.05972	0.05605	-0.11031	0.28263	-0.11555	-0.26302	0.07226
0.13024	-0.15936	0.04882	-0.0274	0.04915	0.16432	-0.10731	0.01835	0.07122	-0.15974	0.27952	-0.05175	-0.24201	0.06566
0.14996	-0.18717	0.04762	0.05304	0.06582	0.07537	-0.10787	0.05461	0.01195	-0.21699	0.3229	0.01151	-0.40564	-0.32833
0.12163	-0.17751	0.09366	0.06897	0.05968	0.09723	-0.20219	0.00785	0.09236	-0.24698	0.30062	0.0013	-0.40156	-0.15506
0.10666	-0.17538	0.08137	-0.01587	0.05194	0.07485	-0.07335	0.04937	0.0638	-0.13593	0.30399	0.22853	-0.39451	-0.39021
0.1525	-0.19411	0.08484	0.01296	0.07716	0.16001	-0.0607	0.0141	0.06078	-0.19225	0.26513	-0.06179	-0.34242	-0.09831
0.14706	-0.12049	0.09569	0.14342	0.008	0.12903	-0.11082	0.01395	0.08174	-0.16718	0.26846	0.01434	-0.38595	-0.11885
0.07428	-0.1366	-0.01464	0.05617	0.12559	0.04299	-0.05913	0.0026	0.07815	-0.2465	0.29092	0.02474	-0.41007	-0.27908
0.13773	-0.13259	0.04574	-0.02641	0.05812	0.08756	-0.08333	0.03393	0.11923	-0.20633	0.31649	0.0083	-0.3177	-0.13191
0.13099	-0.20497	0.11593	0.01828	0.05169	0.15328	-0.08138	0.02611	0.05851	-0.13599	0.30183	-0.03254	-0.25788	0.047

_CANCER	ANCER_2	_CANCER	_CANCER	T_CANCE	ANCER_	T_CANCE	ANCER_	CANCER_	ANCER_	ANCER_D	_AMPLIF	NECTED	FRP2_TA	FRP2_TA
0.1235	0.00128	-0.14148	-0.18234	0.02605	0.01718	-0.02786	-0.02882	-0.0768	0.10202	0.0768	-0.0817	-0.08069	0.13292	0.20105
0.19219	0.07365	-0.12074	-0.13568	0.0013	-0.05498	-0.03155	-0.07077	-0.02652	0.03267	0.01787	-0.06493	-0.07248	0.16001	0.25621
0.12643	-0.00987	-0.06755	-0.08981	-0.06834	0.02875	-0.07454	-0.02973	-0.06526	0.10148	0.06526	-0.05799	-0.07213	0.14577	0.23592
0.06962	0.00739	-0.15738	-0.10321	-0.03278	-0.03831	-0.0637	0.02818	-0.08367	0.04304	0.00298	-0.08208	-0.10196	0.21983	0.23259
0.11367	0.02215	-0.04389	-0.12697	-0.03739	-0.00041	-0.04383	-0.0477	0.02393	0.05593	-0.02689	-0.04263	0.01177	0.12731	0.2135
0.08927	0.03376	-0.10093	-0.12375	0.02132	-0.01359	-0.02783	0.06914	-0.09531	0.04703	0.02564	-0.06123	-0.03915	0.13513	0.23818
0.10632	0.02298	0.08484	-0.04482	-0.05659	-0.06844	-0.03899	0.03097	-0.09931	0.12614	0.06239	-0.05765	-0.07448	0.18752	0.23329
0.12116	-0.00956	-0.11078	-0.13772	-0.02529	-0.06453	-0.07842	-0.00666	-0.04004	0.03417	-0.0118	-0.0169	-0.07889	0.1611	0.26565
0.081	-0.00851	-0.05491	-0.10315	-0.03538	-0.06463	-0.07597	0.01286	-0.08927	0.0298	-0.05257	-0.04455	-0.04932	0.15866	0.25571
0.07092	0.01267	-0.22226	-0.12289	-0.01519	-0.01067	-0.07455	-0.02844	-0.09159	0.02478	-0.02058	-0.04636	-0.02368	0.18511	0.24361
0.15003	0.00146	-0.08605	-0.17988	-0.00092	-0.00173	-0.13919	0.02979	-0.11119	0.05935	0.06239	-0.12465	-0.09772	0.20256	0.27829
0.13819	-0.04511	-0.08572	-0.21569	-0.0081	0.01283	-0.06325	0.03506	-0.1167	0.05747	0.07404	-0.10656	-0.07813	0.18316	0.20468
0.17366	0.07137	-0.11581	0.11832	-0.09818	0.02694	-0.07457	0.0378	0.01338	0.05925	0.11497	-0.08488	-0.1063	0.09237	0.25835
0.13481	0.0634	-0.13672	-0.12541	-0.03229	-0.00126	-0.04693	-0.00423	-0.06342	0.08546	0.0386	-0.06659	-0.12065	0.1347	0.27472
0.16021	0.02042	-0.16852	-0.20796	-0.00907	-0.02299	-0.12725	-0.03261	-0.04654	0.0853	0.05296	-0.06653	-0.03203	0.1421	0.25364
0.13177	0.052	-0.15538	-0.04234	0.00031	0.0286	-0.09082	-0.01632	-0.05667	0.14973	0.1647	-0.09323	-0.08028	0.18108	0.21923
0.11201	0.04763	-0.16198	-0.08539	-0.0687	-0.02637	-0.02278	-0.03653	-0.0596	0.09185	0.02457	-0.0656	-0.10378	0.1645	0.23678
0.09679	0.00694	-0.23924	-0.07932	-0.04597	-0.00726	-0.08118	-0.0198	-0.03876	0.09385	0.02044	-0.02561	-0.07157	0.19188	0.23887

EPC_AS	BM_GER	SILENCE	BM_SOM	ETHYLAT	OIN_AND	_SIGNAL	E_TO_DA	E_TO_DA	VS_AO_G	VS_AO_	EZH2_TA	EZH2_TA	NIPP1_TA
-0.13584	0.08984	0.03657	0.07917	-0.07621	0.07246	0.02729	-0.06677	0.01958	0.29673	0.25323	0.0533	0.07027	0.07796
-0.19111	-0.20574	0.00357	0.04682	-0.09789	0.08972	0.00048	-0.01113	0.07366	0.29846	0.24603	0.06103	0.05775	0.0681
-0.14692	0.00299	0.03432	0.07814	-0.02672	0.07491	0.0309	-0.02565	0.03255	0.30304	0.27129	0.05988	0.0675	0.0709
-0.06956	-0.0421	0.0289	0.04635	0.01399	0.06984	0.00799	0.00206	-0.02792	0.28065	0.27498	0.03895	0.08757	0.071
-0.07934	0.21156	0.05821	0.00579	-0.03627	0.06599	0.00713	0.01695	0.01622	0.26284	0.25287	0.04452	0.06655	0.05758
-0.11551	0.17368	0.04545	-0.0079	-0.05277	0.08166	0.12734	-0.00137	0.04468	0.2672	0.258	0.05653	0.05895	0.05288
-0.04765	0.05813	0.05052	0.01745	-0.01938	0.04563	0.02439	-0.01266	0.1096	0.29143	0.25272	0.05368	0.0757	0.06162
-0.05698	0.0923	0.05417	0.05112	0.00965	0.05848	0.08717	0.04498	-0.00282	0.26802	0.28687	0.02809	0.0947	0.0628
-0.04649	0.15624	0.03052	0.04959	0.02512	0.05395	0.04578	0.04671	-0.0002	0.28025	0.27622	0.02356	0.09426	0.0577
-0.04626	0.16259	0.02143	0.00649	-0.00293	0.04865	0.04441	0.05618	0.04229	0.28772	0.27095	0.03594	0.09002	0.05662
-0.13683	0.03065	0.02828	0.11643	-0.12279	0.06988	-0.04081	-0.04517	0.00833	0.27636	0.25112	0.06371	0.06445	0.08004
-0.1002	0.09459	0.05259	-0.02305	-0.03871	0.06591	0.01963	-0.03831	-0.0142	0.27524	0.27247	0.05784	0.08675	0.06669
-0.08288	-0.17985	-0.00864	0.17761	-0.14615	0.0846	0.01648	-0.06795	0.07106	0.26241	0.25803	0.07044	0.04122	0.08545
-0.10552	0.12049	0.03481	0.06606	-0.02355	0.05964	-0.007	0.00569	0.0426	0.27384	0.26269	0.04557	0.07312	0.07224
-0.14762	-0.08261	0.0722	0.05441	-0.04805	0.0639	-0.01771	-0.03792	-0.01871	0.29346	0.27426	0.05648	0.05863	0.06888
-0.18106	-0.31918	0.04951	-0.00048	-0.12446	0.07653	-0.0547	-0.09482	0.05566	0.29505	0.24189	0.0687	0.05638	0.0753
-0.11807	0.02448	0.02998	0.04082	-0.06378	0.07676	0.03032	-0.03024	0.00343	0.30694	0.25823	0.04186	0.07122	0.06853
-0.08722	0.14004	0.01405	0.05276	-0.08607	0.05797	0.01771	0.03997	0.01119	0.26673	0.2699	0.03563	0.09267	0.05985

NIPP1_T	LL_METAS	L_METAS	TS_OF_	ETS_OF_M	TFRC_T	TFRC_T	AURKA_T	AURKB_T	ER_HNF4	ER_HNF4	ONIC_CA	ONIC_CA	ED_IN_A	OMA_STE
0.03848	0.11118	-0.06573	0.13794	0.10593	0.05758	0.04485	-0.31369	-0.06609	-0.0725	-0.10328	0.08179	0.45766	0.13806	-0.00161
0.03063	0.07694	-0.12176	0.19763	0.07613	0.069	0.01311	-0.27013	-0.01011	-0.12748	-0.11762	0.2858	0.42236	0.19037	-0.04166
0.04465	-0.02949	-0.08599	0.19265	0.12664	0.08719	0.01645	-0.18177	0.06511	-0.07623	-0.04651	0.24133	0.44763	0.15472	-0.02054
0.03283	0.03952	-0.08433	0.08797	0.16359	0.02714	0.05481	-0.36871	0.02589	0.04507	-0.0292	0.08826	0.44424	0.16229	0.00244
0.02455	0.04716	-0.09416	0.09607	0.16452	0.00635	0.05575	-0.27319	-0.00626	-0.05345	-0.00219	0.13127	0.29034	0.10966	-0.00274
0.02258	0.02505	-0.10975	0.17483	0.11695	0.07724	0.03882	-0.22514	-0.01214	0.11712	-0.00571	0.04764	0.33193	0.13418	-0.02205
0.03057	0.04721	-0.12616	0.16063	0.15038	0.0691	0.03873	-0.35989	-0.02742	0.08642	0.00492	0.10727	0.38903	0.18741	0.01032
0.04044	-0.02362	-0.03207	0.06017	0.1782	0.01491	0.05975	-0.38404	0.06836	0.05282	0.03156	-0.24107	0.42497	0.08175	0.0129
0.03806	-0.02297	-0.08544	0.07299	0.16319	0.01053	0.04056	-0.39407	0.04289	0.04854	-0.01149	0.08674	0.39362	0.17669	0.00413
0.03677	0.03835	-0.06364	0.05882	0.16132	0.01562	0.04862	-0.40337	0.00135	0.07445	0.03387	-0.21532	0.43158	0.10445	0.00159
0.03688	0.06007	-0.0081	0.16674	0.05342	0.08956	0.03909	-0.26046	-0.01949	0.00773	-0.0347	0.31674	0.47234	0.21065	-0.02828
0.06329	0.08393	-0.06099	0.16561	0.05157	0.07165	0.02978	-0.31525	-0.05089	0.06141	-0.03923	0.07639	0.44169	0.17032	-0.02626
0.01407	0.13469	-0.04193	0.20605	-0.03122	0.10864	0.03123	-0.15801	-0.11553	0.00715	-0.01583	0.11656	0.45483	0.07708	-0.04041
0.02274	0.03204	-0.0813	0.0865	0.1683	0.03715	0.05948	-0.41602	0.07135	0.00316	-0.01139	0.10561	0.42302	0.14541	0.00585
0.03369	0.04568	-0.00848	0.11885	0.14938	0.0332	0.06949	-0.16317	0.04946	0.02208	-0.02674	0.18555	0.44442	0.20366	-0.02514
0.03992	0.07093	0.00026	0.22621	-0.01544	0.09957	0.01233	-0.27307	-0.01996	-0.10663	-0.05498	0.04487	0.4446	0.15067	-0.05731
0.02207	0.02938	-0.05851	0.11533	0.13528	0.03172	0.05805	-0.32783	0.02234	-0.03363	-0.03373	0.26241	0.44892	0.19191	-0.01074
0.05712	0.00953	-0.02934	0.07915	0.15884	0.00339	0.04259	-0.38493	0.02314	-0.04558	-0.03495	0.07684	0.44326	0.14549	-0.00668

OMA_STE	ASTOMA_	MMATORY	E2F3_TA	E2F3_TA	SIGNALI	DH1_TAR	DH1_TAR	DH1_TAR	DH1_TAR	DH1_TAR	DH1_TAR	BRCA1_T	BRCA1_TH
0.00865	0.00863	0.02948	0.25963	0.04994	-0.02474	0.13051	-0.00889	0.03314	0.01739	0.03917	0.25469	0.06324	0.10747
0.03213	0.07961	0.0433	0.19526	0.12063	-0.03651	0.12751	-0.02126	-0.02368	0.00248	-0.09537	0.21727	0.00462	0.04095
0.02912	0.09479	0.03828	0.13997	0.05557	-0.02641	0.14061	-0.01208	0.01424	0.03055	-0.02062	0.1958	0.03136	0.08759
-0.00845	0.05099	0.04511	0.18131	0.0167	-0.02353	0.13285	-0.00316	0.02127	0.02878	0.03869	0.1849	0.01195	0.20152
-0.00572	0.05655	0.0229	0.14245	0.0276	-0.09774	0.1261	0.00878	-0.02932	-0.00715	-0.03326	0.14038	-0.04123	0.17867
0.02547	0.05877	0.02802	0.19662	0.04253	-0.00984	0.11851	0.00862	-0.03047	0.04622	-0.14713	0.164	-0.05841	0.23458
0.01412	0.12723	0.0304	0.20684	0.10332	0.00405	0.11954	0.00105	-0.00763	0.04643	-0.08294	0.14664	-0.04537	0.25776
-0.01425	0.01312	0.04134	0.16219	0.01228	-0.071	0.1226	0.01426	-0.01684	0.04403	0.00883	0.16916	-0.09777	0.22113
-0.00481	0.06218	0.05201	0.12824	0.01223	-0.04029	0.13128	-0.01459	-0.01748	0.05484	0.02155	0.17524	-0.00068	0.22856
-0.01173	0.00918	0.05087	0.16664	0.03841	-0.04869	0.11876	0.00644	-0.02462	0.02122	-0.03876	0.18606	-0.06038	0.21948
0.04261	0.17375	0.0252	0.15931	0.0581	-0.02274	0.14384	0.01625	0.02501	-0.00983	0.04028	0.23291	-0.02374	0.0179
0.05053	0.02069	0.00055	0.17385	0.02169	0.02551	0.14523	0.01032	0.03742	0.04711	0.03585	0.20893	0.0752	0.15625
0.03115	0.13115	0.03081	0.13635	0.0327	0.00609	0.1339	0.01782	0.03849	-0.04386	0.0348	0.15918	0.07728	-0.02511
0.00886	0.12283	0.01576	0.16565	0.03921	-0.03671	0.14763	0.02767	0.0185	0.00478	0.09368	0.24112	0.04883	0.07408
0.02875	0.01487	0.02709	0.20982	0.05802	-0.03866	0.14398	0.00285	0.01633	0.03739	0.02902	0.21139	-0.06999	0.07504
0.05178	0.11838	0.01542	0.13712	0.11788	-0.01541	0.13758	-0.01138	0.0293	-0.05137	0.0315	0.19789	0.04045	-0.06387
0.01138	0.0548	0.0286	0.18123	0.00961	-0.02017	0.13368	-0.01626	0.03048	8.2E-05	0.04806	0.1894	0.02679	0.14299
-0.0098	0.01741	0.02788	0.15892	0.01191	-0.01698	0.11282	-0.02643	-0.00283	0.02886	-0.07214	0.16165	-0.07645	0.1408

A_TP53	EAL_MEL	EAL_MEL	L1_TARG	L1_TARG	XP3_TAR	XP3_TAR	CL1_TA	CL1_TA	A_TNF_TA	ADDER_C	ADDER_C	STEM_CE	STEM_CE	IAN_CAN
-0.07242	0.13971	0.12864	-0.06206	0.12429	-0.01981	0.11153	0.12102	0.03934	-0.05129	0.14298	0.15111	0.08948	0.08098	0.28342
-0.06557	0.14891	0.10699	-0.06056	0.06755	-0.07314	0.07392	0.03765	0.03744	0.01025	0.16092	0.13596	0.08377	0.09455	0.28287
0.0012	0.1401	0.1262	0.01728	0.07373	0.0169	0.08415	0.09181	-0.1195	0.00367	0.15077	0.15843	0.08001	0.10703	0.27737
-0.02029	0.1499	0.12365	0.01024	0.07885	0.09716	-0.00145	0.11411	0.00902	-0.05225	0.14559	0.1547	0.08648	0.07578	0.29887
0.00812	0.12651	0.12439	0.035	0.16531	0.11596	0.10648	0.03949	-0.10033	-0.14655	0.16182	0.15094	0.07661	0.08934	0.2887
0.01026	0.13971	0.11916	-0.07147	0.11545	0.03889	0.0748	0.09526	0.02109	0.00596	0.16195	0.13231	0.09828	0.08235	0.28945
-0.01294	0.1286	0.13125	0.03717	0.10477	0.03253	0.09923	0.16913	-0.10977	-0.04216	0.15113	0.13395	0.08186	0.0593	0.3009
0.01163	0.13947	0.13103	0.08587	0.08855	0.12246	0.05345	0.1238	0.0775	-0.12633	0.14366	0.15344	0.08711	0.09673	0.28729
0.05366	0.13335	0.1296	0.07667	0.09513	0.11654	0.05385	0.13369	0.0387	-0.03331	0.1467	0.15392	0.07645	0.11265	0.29817
-0.01994	0.13116	0.12524	0.08019	0.18904	0.17147	0.11005	0.152	0.0643	0.09908	0.15045	0.15391	0.09188	0.06988	0.29974
-0.0632	0.14352	0.11727	-0.07469	0.0265	-0.07002	-0.00365	0.05452	0.08247	0.08944	0.14066	0.14251	0.08673	0.07426	0.3145
0.03652	0.14621	0.12571	-0.0234	0.07659	0.00345	0.05957	0.128	-0.10729	0.09088	0.14893	0.1436	0.08273	0.0808	0.27099
-0.09684	0.15246	0.10292	-0.02083	0.06701	-0.06983	0.04021	-0.02334	-0.01604	0.2237	0.14166	0.123	0.09306	0.0636	0.28639
0.02596	0.13959	0.12526	-0.02818	0.04812	0.01288	0.01492	0.07075	-0.12626	-0.00205	0.14191	0.15434	0.07143	0.10344	0.29533
-0.02694	0.14747	0.12459	-0.05856	0.05188	-0.02752	0.02991	0.0572	-0.006	0.01118	0.1554	0.14577	0.09145	0.09064	0.30027
-0.02695	0.13955	0.11118	-0.00972	-0.00266	-0.14237	0.05641	0.03071	-0.02295	0.0423	0.15509	0.13662	0.08241	0.08809	0.29009
-0.01227	0.14894	0.11844	-0.02217	0.08606	0.02469	0.04352	0.09015	0.04874	-0.02784	0.15756	0.14898	0.08114	0.08902	0.30021
-0.00343	0.12871	0.13026	0.06767	0.13006	0.09768	0.1087	0.12118	0.23223	-0.01244	0.1427	0.16377	0.07338	0.07134	0.29337

RIAN_CAN	ANCER_I	ANCER_I	L_13Q14	L_13Q14	TATE_CA	CANCER_	CANCER_	AP1_TAR	R125B1_	TS_OF_IG	GSI_SEN	GSI_SEN	MT5_TAR	MT5_TAR
0.1936	0.16527	0.26412	-0.07212	0.00193	0.05583	0.16091	0.11563	0.19262	0.04769	-0.00208	0.0266	-0.02625	0.22199	0.13512
0.21727	0.19024	0.26344	-0.0762	-0.03647	0.02218	0.15879	0.11951	0.17565	0.05114	0.01206	-0.17721	0.05284	0.22263	0.15575
0.21785	0.20434	0.27343	-0.03902	-0.02282	0.03348	0.11015	0.15462	0.20534	0.03688	0.01544	0.00053	-0.39704	0.17514	0.14467
0.19919	0.22545	0.2526	-0.03766	-0.01584	0.05724	0.12485	0.1794	0.2427	-0.00303	-0.05861	-0.26035	-0.13757	0.25622	0.12921
0.20762	0.19458	0.26004	-0.09027	-0.01517	0.04909	0.11596	0.1813	0.21096	0.05048	-0.02531	0.26916	-0.13053	0.24819	0.13293
0.18944	0.22963	0.26976	-0.02292	-0.03668	0.12625	0.10276	0.09358	0.15492	0.07159	0.03951	0.26387	0.03593	0.21964	0.15988
0.19699	0.24049	0.27852	-0.01539	-0.01998	0.10439	0.1268	0.17446	0.20963	0.04945	0.08787	-0.26422	-0.03805	0.1778	0.14796
0.20306	0.18069	0.23835	-0.04034	-0.01828	0.00717	0.13304	0.20164	0.25694	0.00723	-0.03781	0.2297	-0.14885	0.1651	0.11603
0.19377	0.23458	0.24883	-0.05516	-0.02009	0.02099	0.10429	0.19476	0.24726	0.05018	-0.04782	0.20768	-0.04597	0.20507	0.11708
0.1881	0.16225	0.25498	-0.04393	-0.0129	0.05534	0.09266	0.19886	0.23612	-0.00219	-0.05235	-0.07187	-0.20169	0.22871	0.12796
0.19916	0.23038	0.2714	0.02263	-0.04588	0.09814	0.09988	0.10567	0.20452	0.01739	0.01037	-0.3685	-0.26088	0.22461	0.15327
0.19939	0.19779	0.2638	0.00463	-0.0439	0.04891	0.13153	0.13626	0.20782	0.03296	0.0379	-0.034	-0.02114	0.22671	0.15783
0.20008	0.18811	0.27427	0.02916	-0.03317	0.12891	0.20609	0.11438	0.06935	0.06118	-0.0234	-0.43315	-0.06641	0.15911	0.15164
0.21965	0.23344	0.26468	-0.04394	-0.02652	0.03895	0.14666	0.12515	0.22875	0.0283	-0.00901	-0.21226	-0.19922	0.19051	0.13878
0.21365	0.24611	0.26031	0.0065	-0.02375	0.0464	0.15187	0.12775	0.21879	0.02396	0.02424	-0.45306	-0.02537	0.21887	0.14035
0.20789	0.16493	0.26744	-0.01807	-0.02522	0.01906	0.15365	0.11211	0.15325	0.02515	0.01157	-0.32077	-0.02149	0.25594	0.14551
0.19468	0.20981	0.26107	-0.04613	-0.01037	0.07584	0.13614	0.1438	0.22152	0.0231	-0.07471	-0.05425	0.05866	0.16932	0.12981
0.1857	0.18181	0.26256	0.02864	-0.01252	0.00995	0.09425	0.18324	0.24092	0.06217	-0.04376	0.24115	-0.47578	0.20302	0.1267

SSION_B	SSION_B	LE_ATER	LE_ATER	TOR_SIG	TOR_SIG	PATHOG	PATHOG	ULTIPOTE	HSC_MAR	TIPOTEN	TIPOTEN	ERENTIA	ERENTIA	TINOIN_
0.12468	0.13073	0.04107	0.12035	0.03369	0.05489	0.17219	0.28989	0.25865	0.00468	0.23081	0.21239	0.22845	-0.20859	0.11526
0.11815	0.13957	0.02848	0.10141	0.0408	0.06447	0.09579	0.2341	0.24567	0.05779	0.20253	0.21327	0.2129	-0.22222	0.04135
0.11431	0.1526	0.02727	0.16268	0.01265	0.06049	0.14137	0.24311	0.2537	0.01438	0.24356	0.2349	0.19485	-0.31424	0.05776
0.10804	0.16301	0.03607	0.23323	0.01021	0.06118	0.21398	0.28144	0.27614	0.03407	0.2349	0.20995	0.2488	-0.10176	0.19783
0.10735	0.15165	-0.01907	0.14784	0.06728	0.06926	0.19313	0.31291	0.27458	0.02794	0.21445	0.25875	0.16166	-0.30674	0.14852
0.12719	0.17191	0.04065	0.06661	-0.01921	0.05142	0.13665	0.29862	0.26012	-0.00936	0.25176	0.21596	0.18693	0.02483	0.09324
0.10649	0.17559	-0.00513	0.12661	0.05707	0.05397	0.11488	0.23373	0.23951	-0.01226	0.21319	0.24242	0.18696	0.00369	0.10485
0.12519	0.16452	0.03963	0.24683	0.03853	0.0704	0.25122	0.27308	0.26248	0.03607	0.21543	0.21266	0.32718	-0.12712	0.22669
0.12149	0.17916	0.04483	0.25708	-0.03669	0.06999	0.21708	0.30462	0.26303	0.05986	0.20576	0.23118	0.26428	-0.00634	0.14857
0.1264	0.15816	0.00301	0.21827	0.02691	0.06495	0.22864	0.28414	0.26688	0.02784	0.25059	0.20875	0.22657	-0.0901	0.18091
0.13143	0.13435	0.0851	0.01681	0.03139	0.04722	0.12262	0.25417	0.25738	-0.01266	0.22908	0.23289	0.20131	-0.13487	0.05507
0.12395	0.1754	0.01534	0.07726	0.00459	0.05133	0.13908	0.28283	0.26755	-0.03624	0.23581	0.22166	0.1874	-0.14427	0.08644
0.10876	0.11231	0.07367	0.01088	-0.05465	0.05989	0.01892	0.20542	0.2434	-0.00625	0.18586	0.21155	0.1363	-0.21806	-0.12967
0.11743	0.15985	0.03476	0.17592	0.04673	0.06071	0.1855	0.26363	0.24293	0.04086	0.22472	0.23993	0.21084	-0.19152	0.14478
0.09887	0.14589	0.07865	0.11542	0.09313	0.05958	0.18614	0.24953	0.24181	-0.00296	0.23161	0.22513	0.18343	-0.09985	0.12061
0.09867	0.09947	0.04766	-0.01444	-0.04251	0.06307	0.00895	0.21096	0.26322	-0.01356	0.18046	0.20646	0.01507	-0.24142	-0.12351
0.12245	0.1715	0.03799	0.1672	0.0384	0.05863	0.20419	0.27747	0.28895	0.0311	0.20733	0.22788	0.14606	-0.2617	0.16616
0.11071	0.1567	-0.01293	0.21438	-0.00033	0.06815	0.16387	0.29885	0.2747	0.01266	0.24871	0.21974	0.2168	-0.23526	0.17126

PONSE_A	PONSE_A	UZ12_TA	UZ12_TA	MPHOMA	MPHOMA	RIZ1_TA	RIZ1_TA	LIVER_C	LIFERAT	LIFERAT	ARY_STE
0.14555	0.11717	0.13639	0.05997	0.12266	0.11238	-0.30033	-0.04121	0.10362	0.13866	0.03932	0.10442
0.0991	0.06673	0.12151	0.06581	0.11502	0.09804	-0.15378	-0.12727	0.10877	0.11839	0.06895	0.11397
0.13419	0.09048	0.1355	0.07365	0.10797	0.09677	-0.10551	-0.06038	0.09638	0.14234	0.03602	0.10836
0.20225	0.18609	0.14984	0.1052	0.1296	0.08901	0.15255	0.03099	0.08238	0.13232	0.07489	0.1167
0.16321	0.17878	0.11362	0.07886	0.17042	0.09113	-0.07152	-0.08479	0.0833	0.13266	0.05394	0.0887
0.12361	0.10098	0.13453	0.04756	0.15578	0.09894	-0.36727	-0.02174	0.10001	0.14269	0.09938	0.11893
0.14792	0.12633	0.11939	0.05999	0.13185	0.09506	-0.2866	-0.07299	0.09036	0.15925	0.03944	0.10655
0.21231	0.20965	0.1471	0.08592	0.14958	0.089	0.07539	0.13128	0.07061	0.11346	0.06616	0.11069
0.19115	0.1689	0.153	0.09473	0.1507	0.0887	0.13053	0.09459	0.0694	0.11993	0.06829	0.10732
0.19618	0.18308	0.11858	0.09223	0.16253	0.06171	0.14321	0.02562	0.07246	0.13561	0.06715	0.09897
0.07109	0.05648	0.14621	0.09416	0.08732	0.10538	-0.30562	-0.07736	0.11282	0.12572	0.05742	0.11396
0.10356	0.08634	0.13776	0.07274	0.12159	0.09655	-0.37185	-0.11268	0.10538	0.11346	0.0415	0.10191
0.0706	0.01487	0.11847	0.03989	0.07664	0.10719	-0.4668	-0.18835	0.11458	0.15266	0.07006	0.09995
0.16048	0.1662	0.13586	0.09438	0.12618	0.1046	0.05337	-0.05176	0.085	0.10497	0.04712	0.10096
0.13373	0.08951	0.144	0.06349	0.11431	0.09417	0.01233	-0.02882	0.10574	0.14269	0.07317	0.11107
0.03812	0.00015	0.11938	0.07205	0.05024	0.10668	-0.39457	-0.15353	0.11881	0.15746	0.04575	0.09687
0.15735	0.14891	0.13804	0.06126	0.13596	0.10425	-0.15008	-0.02441	0.08724	0.10383	0.06307	0.10961
0.17937	0.171	0.14667	0.08078	0.14731	0.09436	-0.0458	0.03447	0.07803	0.11123	0.07153	0.09794

ASIS_BY_	ASIS_BY_	ASIS_BY_	OF_611CT	MIR31_TA	MIR31_TA	N_ANTIG	N_ANTIG	LYMPHOC	LYMPHOC	_AND_TP	TP53_TA	TP63_TA	LOOD_PR	LOOD_PR	RDIAC_H	ART_MAS	ART_MAS	NTRICLE	NTRICLE	UM_LYMP
0.04666	0.06678	0.08072	0.08068	0.0691	0.05203	0.3007	0.22951	-0.04758	0.05618	-0.06651	-0.02919	-0.00509	0.11233	-0.29797	0.20596	-0.02411	0.05471	0.13065	0.05862	0.13983
0.05155	0.04801	0.06328	0.02386	0.03284	0.05432	0.29097	0.22541	-0.01609	-0.02901	-0.06155	-0.04269	-0.01014	0.15656	-0.1579	0.19786	-0.05651	0.07243	0.13678	0.11872	0.08249
0.02491	0.07419	0.06417	0.01331	0.0738	0.03805	0.28459	0.20815	0.11576	0.02682	-0.04673	-0.04133	-0.00905	0.04271	-0.10509	0.22439	-0.00868	0.12087	0.0922	0.10525	0.09852
0.04251	0.08215	0.07747	0.05946	0.09367	0.05595	0.28647	0.21624	0.03496	0.08913	-0.03828	-0.01694	0.00896	0.13877	-0.06085	0.25436	-0.0164	0.08194	0.05679	0.06967	0.12915
0.05205	0.08854	0.05196	-0.04349	0.08741	0.00141	0.30748	0.22628	0.02118	0.09899	-0.03747	-0.03383	0.00323	0.11647	-0.08021	0.21753	0.02412	0.06822	0.13717	0.11191	0.10626
0.02948	0.11554	0.03896	-0.03107	0.0428	0.03802	0.29694	0.21859	0.10881	0.01092	-0.02202	-0.0387	0.018	0.1459	-0.13836	0.23015	-0.02857	0.05698	0.10904	0.08346	0.09267
0.02376	0.07957	0.0477	-0.01882	0.07961	0.05211	0.28574	0.21203	0.16715	-0.00152	-0.02395	-0.03768	0.02279	0.13286	-0.09138	0.23053	-0.01871	0.11709	0.09545	0.13435	0.12847
0.08446	0.112	0.06434	0.02896	0.08446	0.00841	0.30403	0.22706	0.00309	0.13564	-0.05139	-0.02366	0.00938	0.08749	-0.07514	0.25098	-0.00258	0.13539	0.0353	0.08946	0.12089
0.05621	0.15732	0.0558	0.01674	0.09161	0.02417	0.31406	0.22868	0.1102	0.16914	-0.02516	-0.01805	0.01577	0.08541	-0.07221	0.24367	0.01715	0.10285	0.03139	0.09552	0.13575
0.05026	0.07812	0.03844	-0.02863	0.09085	0.04333	0.30924	0.21679	0.05962	0.12335	-0.04712	-0.03253	0.01346	0.10483	-0.06784	0.25073	-0.01302	0.10797	0.02746	0.09591	0.10206
0.03691	0.1196	0.09261	0.03907	0.06296	0.077	0.26134	0.23031	0.12358	0.03778	-0.08439	-0.051	-0.01143	0.16355	-0.14164	0.25834	-0.05273	0.1261	0.15324	0.12102	0.07659
0.04117	0.06667	0.07183	0.06019	0.06707	0.0676	0.28947	0.24211	0.19714	0.04579	-0.09529	-0.04584	-0.01376	0.06956	-0.22981	0.24822	-0.02876	0.0957	0.1372	0.12033	0.12431
0.04266	0.01015	0.04793	0.01971	0.03031	0.04551	0.25727	0.22978	0.02319	0.04089	-0.07316	-0.05567	-0.00979	0.16773	-0.16541	0.18648	-0.02003	0.09868	0.1226	0.13145	0.05225
0.01093	0.08665	0.07289	0.05888	0.067	0.03875	0.27782	0.22479	0.14606	0.06514	-0.04892	-0.02499	0.00806	0.15037	-0.0968	0.23204	-0.00143	0.10489	0.12351	0.14014	0.11634
0.08953	0.08091	0.07277	0.03161	0.06067	0.06179	0.29093	0.21729	0.10387	0.08324	-0.0726	-0.05002	-0.0073	0.02088	-0.06534	0.2618	-0.01466	0.09581	0.13084	0.10879	0.10968
0.03785	0.07167	0.06841	0.01737	0.03457	0.08778	0.28248	0.20133	-0.0242	-0.07502	-0.08497	-0.04195	-0.0169	0.08407	-0.14433	0.2158	-0.04437	0.13645	0.08625	0.11787	0.07854
0.05468	0.07567	0.07245	0.02247	0.06965	0.05963	0.26199	0.21191	-0.02255	0.04111	-0.04382	-0.02475	0.01142	0.11742	-0.29181	0.22216	-0.03247	0.08097	0.07488	0.11269	0.11334
0.02938	0.08418	0.06547	-0.00343	0.08856	0.02891	0.2972	0.22726	0.03606	0.08474	-0.06475	-0.03119	0.00269	0.09668	-0.0509	0.25411	0.02809	0.11584	0.09638	0.09793	0.11294

UM_LYMP	ROX1_TA	ROX1_TA	S_OF_AP	S_OF_AP	ESPONS	PONSE_V	PONSE_	TNF_TAR	TNF_TAR	MUNOBL	MUNOBL	LGI1_TAR	LGI1_TAR	LF1_TAR	PKD1_TA	PKD1_TA
0.06279	0.15885	-0.08041	0.2547	0.19833	0.03776	-0.02942	0.02723	-0.25351	-0.06146	0.01821	0.03258	-0.28448	0.09437	0.0521	0.3541	0.11702
0.05198	0.1063	-0.01867	0.26476	0.23772	0.02897	0.01087	0.0285	-0.35332	-0.00107	0.00113	0.00164	-0.2304	0.15801	0.04676	0.37841	0.01452
0.07763	0.14188	-0.02649	0.26481	0.20397	0.03061	-0.02307	0.02925	-0.20643	-0.06506	-0.0053	0.05774	-0.15994	0.23201	0.06144	0.36551	0.10007
0.10261	0.15026	-0.06373	0.25876	0.2382	0.03788	-0.01582	0.02575	-0.205	-0.11369	0.01053	0.09212	-0.08543	0.12927	0.07096	0.36643	0.08069
0.08139	0.13413	-0.02877	0.26713	0.21772	0.03138	-0.04263	0.013	0.00233	-0.13474	0.00752	0.0394	-0.38964	0.12836	0.05698	0.37867	0.0635
0.1026	0.14051	0.05872	0.23774	0.14371	0.00796	-0.01925	-0.00279	-0.05528	-0.12031	-0.01404	0.06348	-0.37485	0.1132	0.10699	0.38134	0.03114
0.0703	0.15188	-0.09416	0.23799	0.25138	0.04803	-0.0343	0.04441	-0.33521	-0.07368	-0.0297	0.09166	-0.31725	0.10753	0.1012	0.37114	0.01335
0.04697	0.16534	-0.14211	0.24289	0.20205	0.0428	-0.01624	0.0475	-0.06954	-0.14432	0.01458	0.10377	-0.24168	0.10608	0.08399	0.3723	0.0624
0.07424	0.16717	-0.1162	0.24791	0.20157	0.04391	-0.00257	0.04925	-0.14727	-0.06247	0.02918	0.11118	-0.19517	0.11313	0.08894	0.37393	0.03417
0.07615	0.13127	-0.16923	0.25716	0.23922	0.0472	-0.01907	0.03231	-0.16662	-0.07055	-0.00076	0.06354	-0.24344	0.09924	0.06709	0.37036	0.04834
0.06076	0.06751	0.03743	0.22023	0.17845	0.02119	-0.02899	0.04583	-0.25364	-0.04366	0.002	0.04183	-0.40373	0.20755	0.06471	0.37752	0.05574
0.04129	0.12588	-0.03305	0.24143	0.23614	0.03235	-0.0224	0.04405	-0.36527	-0.10506	0.00052	0.0629	-0.30192	0.16564	0.0753	0.38006	0.10414
0.01204	0.06543	0.0083	0.33285	0.15502	0.03016	-0.00996	0.01749	-0.14487	-0.05363	-0.00118	0.01732	-0.17773	0.1387	0.07089	0.36593	0.10086
0.06377	0.14011	-0.16444	0.21374	0.21305	0.03774	-0.03128	0.01191	-0.28109	-0.0767	0.02277	0.05993	-0.26845	0.14319	0.05046	0.36491	0.0789
0.07039	0.12755	-0.05489	0.24877	0.24826	0.02704	-0.02247	0.02121	-0.36277	-0.0939	0.01407	0.06004	-0.33785	0.21116	0.0674	0.36865	0.07242
-0.00204	0.05852	-0.01306	0.28914	0.19156	0.02792	-0.0131	0.03398	-0.27334	-0.01071	-0.02856	0.01414	-0.37326	0.23096	0.04832	0.36421	0.08899
0.05984	0.1293	-0.16479	0.22891	0.21703	0.02831	-0.02609	0.02632	-0.23479	-0.10689	0.01727	0.04858	-0.15589	0.14601	0.05338	0.38634	0.08713
0.06992	0.13381	-0.08118	0.26669	0.22683	0.0435	-0.03833	0.0391	-0.01287	-0.12286	0.0132	0.08491	-0.25542	0.0839	0.05727	0.37058	0.07555

C_TARGE	C_TARGE	NALING	NALING	NALING	NALING	B1_TARG	B1_TARG	B1_TARG	B1_TARG	SE_TO_	NSE_TO_	OMA_DES	OMA_DES	DBLASTO	DBLASTO	E_BREA
-0.04922	0.03332	-0.06688	-0.05582	0.13189	-0.10003	0.02846	-0.04096	-0.27435	-0.06488	-0.00413	0.08659	0.23641	0.15769	0.34814	0.05659	0.04056
-0.04693	-0.06862	-0.07483	-0.07522	0.12786	-0.1251	0.00835	-0.02465	-0.26969	-0.17862	-0.05026	0.07533	0.25496	0.18171	0.37457	0.06832	0.01976
-0.01575	0.06768	-0.05539	-0.01579	0.13243	-0.12728	0.0604	-0.07433	-0.1111	-0.18479	-0.01467	0.08551	0.24166	0.16858	0.33603	0.07289	0.00626
-0.01645	0.05682	-0.00641	0.00786	0.11731	-0.09681	0.07146	-0.02617	-0.22456	-0.13573	-0.01929	0.10217	0.30304	0.16993	0.33495	0.09527	0.01722
7.6E-06	0.13691	-0.05475	-0.11753	0.08644	-0.10053	0.03036	-0.05895	-0.32188	-0.15684	-0.06685	0.10327	0.26416	0.14751	0.3415	0.08798	-0.02484
-0.03371	0.10276	-0.04054	-0.04493	0.07755	-0.13091	0.01585	-0.06642	-0.37844	-0.03409	-0.00068	0.0892	0.31778	0.15937	0.3402	0.06129	0.02966
0.00919	0.10152	-0.03586	0.02201	0.11326	-0.10536	0.05277	-0.06428	-0.25959	-0.00434	-0.04998	0.08492	0.32077	0.16631	0.34436	0.07602	0.02115
-0.0387	0.06478	0.0193	-0.047	0.07036	-0.12681	0.07609	-0.04229	-0.20768	-0.11939	-0.08007	0.12772	0.31129	0.14843	0.31812	0.09611	0.00037
0.01492	0.08895	0.01254	-0.00034	0.08963	-0.11896	0.0805	-0.05803	-0.4482	-0.08604	-0.01206	0.11203	0.31573	0.13961	0.33098	0.10096	0.01362
-0.05804	0.12671	-0.07469	0.04119	0.07172	-0.14207	0.07174	-0.05136	-0.13467	-0.07377	-0.08612	0.09333	0.2978	0.13774	0.33599	0.09905	0.00853
0.01901	0.02948	-0.05651	-0.01664	0.12097	-0.04422	0.0134	-0.01791	-0.12788	-0.2408	-0.00923	0.10457	0.24646	0.18399	0.35318	0.03972	0.05346
0.00367	0.10716	-0.04077	0.02515	0.09237	-0.03463	0.05136	-0.02372	-0.10028	-0.15846	-0.00881	0.10295	0.28338	0.13358	0.34074	0.0528	0.03276
-0.02697	0.04349	-0.16032	0.01855	0.08747	-0.18264	-0.00508	-0.00254	0.11773	-0.24228	0.054	0.03554	0.23394	0.18875	0.34795	0.06349	0.03204
0.02376	0.05666	-0.01862	-0.09406	0.10347	-0.06128	0.03019	-0.04403	-0.20908	-0.22307	-0.02466	0.09347	0.28582	0.17542	0.34471	0.08183	-0.00299
-0.05626	0.05934	-0.03295	-0.02777	0.08826	-0.07018	0.01879	-0.04216	-0.31377	-0.14813	-0.05385	0.09654	0.29162	0.1613	0.34146	0.07066	0.03682
-0.0487	0.14407	-0.12541	-0.06136	0.10471	-0.12916	-0.00454	-0.05423	-0.14293	-0.26525	0.061	0.0363	0.23305	0.16738	0.36852	0.05279	0.02997
0.01279	0.05394	-0.04929	-0.07646	0.13244	-0.08935	0.03652	-0.07141	-0.23975	-0.15739	-0.03994	0.08804	0.27981	0.18362	0.35425	0.10588	0.05122
0.01733	0.14147	0.00525	-0.05054	0.08568	-0.01825	0.06028	-0.03792	-0.37957	-0.18942	-0.05918	0.09904	0.24866	0.15126	0.319	0.10541	-0.00485

E_BREA	E_RESPO	SE_TO_H	SE_TO_H	GO_SOX4	O_SOX4	NI_META	NI_META	INHIBITE	INHIBITE	M_PCC_	CA1_PCC	CA2_PCC	_CENTER	ANCER_	ER_WITH	ER_WITH
-0.00457	0.14984	0.10882	0.10399	0.13151	-0.01065	0.16557	0.09768	0.00252	0.19605	0.12408	0.16597	0.07565	0.11136	0.0747	0.26593	0.12409
-0.05324	0.1127	0.04377	0.08277	0.15893	-0.10073	0.17251	0.10162	0.02362	0.19561	0.147	0.19318	0.11467	0.1501	0.11813	0.36882	0.12676
0.02577	0.09219	0.07732	-0.01128	0.17508	-0.03187	0.16941	0.11156	0.00088	0.20556	0.15118	0.18996	0.10524	0.15552	0.10597	0.34788	0.16263
0.0664	0.1187	0.03722	0.0515	0.1748	0.00274	0.17022	0.09863	0.00492	0.17926	0.14357	0.16401	0.06858	0.10777	0.07962	0.29226	0.11503
0.07222	0.1048	0.05357	0.1006	0.16423	-0.04169	0.14007	0.10308	-0.00269	0.19404	0.15488	0.17769	0.09869	0.1267	0.0758	0.32942	0.11855
0.02149	0.19999	0.15449	0.16744	0.1765	-0.02408	0.15687	0.09629	-0.00538	0.19328	0.15395	0.19182	0.11444	0.14767	0.09855	0.34554	0.13821
0.06831	0.11339	0.14025	0.06775	0.19302	-0.03484	0.16817	0.09324	-0.00997	0.18409	0.14164	0.17934	0.09349	0.12169	0.09914	0.34346	0.11
0.09858	0.11771	0.0143	0.08042	0.15087	-0.01109	0.12509	0.09618	0.00192	0.19184	0.13292	0.15195	0.04638	0.07054	0.03251	0.26317	0.05588
0.12356	0.1231	0.02957	0.11968	0.14359	-0.00554	0.13855	0.09803	-0.00228	0.17482	0.14298	0.15966	0.06032	0.08116	0.04939	0.29043	0.07017
0.12278	0.08188	0.01969	0.10249	0.1678	-0.03261	0.13099	0.09966	0.01418	0.1849	0.15524	0.16932	0.08095	0.10031	0.06401	0.29185	0.08129
-0.0629	0.12243	0.17314	0.05828	0.12544	-0.02575	0.19673	0.09941	0.01584	0.20723	0.13121	0.18467	0.1148	0.16734	0.09994	0.32832	0.17269
-0.01139	0.14299	0.22346	0.0779	0.15318	0.01447	0.1774	0.10443	0.01643	0.17665	0.13573	0.17925	0.09719	0.14597	0.10302	0.31039	0.14684
-0.10666	0.12196	0.1833	0.05136	0.13764	-0.08051	0.17098	0.07148	-0.01082	0.18069	0.12403	0.18449	0.1022	0.14945	0.14041	0.36034	0.14188
0.04905	0.10728	0.07376	0.04512	0.15591	-0.00822	0.15124	0.09041	-0.00939	0.20062	0.13742	0.17208	0.07361	0.09862	0.061	0.32358	0.07046
0.01268	0.12627	0.16689	0.08555	0.16072	-0.02537	0.15864	0.10534	0.02687	0.19765	0.13858	0.18318	0.09372	0.12708	0.07839	0.29923	0.12773
-0.12856	0.13625	0.2097	0.03405	0.13598	-0.10304	0.18807	0.06856	0.01707	0.18278	0.12612	0.18794	0.10108	0.15643	0.12796	0.33919	0.15459
0.02418	0.08865	0.05104	0.09337	0.18251	-0.02404	0.16374	0.09551	-0.00068	0.19157	0.13403	0.16874	0.06483	0.07903	0.05549	0.30929	0.0567
0.07872	0.10284	0.06409	0.057	0.15975	-0.01103	0.14918	0.10707	0.00117	0.1916	0.13899	0.16409	0.0698	0.09348	0.05174	0.28357	0.07745

EK2_PCC	RSS_INT	_OF_CTB	OF_CTB	OF_CTB	OF_CTB	_AND_NE	AND_NE	POSITIVE	POSITIVE	_HYPOX	ETS_OF_	SMACYTO	SMACYTO	NIC_BRA	SPONSE_	SPONSE_1
0.15968	0.11146	0.09797	0.26598	0.06621	0.07041	-0.39433	0.08355	0.06655	0.05995	0.10261	0.08022	0.09549	0.12862	0.01346	0.03104	0.15297
0.194	0.15472	0.10709	0.26765	0.06344	0.06383	-0.43236	0.10891	-0.01935	0.07493	0.06346	0.11576	0.06249	0.10816	0.00339	0.01297	0.19011
0.18917	0.1461	0.09913	0.23896	0.05566	0.06158	-0.45559	0.11285	-0.04259	0.08622	0.08772	0.09692	0.07796	0.14343	0.07628	0.02204	0.20516
0.15395	0.10397	0.08748	0.25995	0.06058	0.06057	-0.38574	0.05153	-0.06494	0.00353	0.09345	0.11197	0.08981	0.18902	0.07739	0.05988	0.23325
0.1684	0.12527	0.07026	0.27258	0.06345	0.06255	-0.4393	0.07152	-0.07863	0.03692	0.06894	0.06201	0.05104	0.17425	0.07132	0.05097	0.19108
0.19047	0.14716	0.08743	0.26791	0.0549	0.04851	-0.35037	0.07386	0.0976	0.0726	0.06929	0.06188	0.10386	0.14086	0.07586	0.04249	0.22181
0.17572	0.11315	0.07734	0.25788	0.04213	0.05464	-0.3599	0.06813	0.00508	0.05245	0.08695	0.13243	0.11251	0.17043	0.07209	0.01748	0.26495
0.13722	0.06751	0.08041	0.27374	0.05586	0.05938	-0.43683	0.02998	-0.08187	-0.01395	0.09076	0.10798	0.07684	0.19353	0.0946	0.06018	0.20773
0.14444	0.08067	0.07844	0.28022	0.05373	0.0525	-0.28794	0.04432	-0.03305	0.00873	0.08323	0.06968	0.06842	0.19703	0.12037	0.06577	0.25453
0.15678	0.10035	0.08065	0.27354	0.05628	0.06918	-0.39487	0.04273	-0.03692	0.00075	0.05237	0.0661	0.05177	0.20678	0.07869	0.0665	0.27735
0.18645	0.15813	0.10551	0.27759	0.04135	0.08046	-0.39541	0.10847	0.08183	0.08908	0.11	0.12776	0.10023	0.10721	0.06711	-0.03091	0.16394
0.17937	0.13649	0.10956	0.25234	0.07232	0.06472	-0.46473	0.09642	-0.03168	0.08427	0.09214	0.06931	0.08251	0.13383	0.08199	0.02457	0.23306
0.19057	0.14205	0.0884	0.25093	0.05956	0.06354	-0.16805	0.12551	0.08101	0.07722	0.1182	0.11949	0.07348	0.093	-0.00506	-0.00475	0.20252
0.16181	0.09791	0.08358	0.27959	0.04352	0.062	-0.00568	0.06553	-0.06867	0.01667	0.09607	0.13357	0.07435	0.16926	0.0925	0.05113	0.16887
0.17719	0.12089	0.11493	0.2761	0.04968	0.07543	-0.28822	0.08445	-0.00483	0.04576	0.09786	0.06839	0.09365	0.13265	0.0998	0.01564	0.1958
0.18792	0.13991	0.10942	0.28175	0.05697	0.06488	-0.41284	0.11548	0.10329	0.10424	0.10226	0.09117	0.06342	0.06641	0.05942	-0.0233	0.19445
0.15903	0.08989	0.11203	0.27427	0.04893	0.06882	-0.32599	0.06486	0.04975	0.02121	0.09115	0.08729	0.08543	0.16556	0.08099	0.09394	0.17869
0.15143	0.09111	0.07821	0.26524	0.05587	0.055	-0.36518	0.0591	-0.08635	0.00523	0.09897	0.08425	0.05665	0.1856	0.11002	0.05484	0.23572

R_AML_	EGFA_TAR	LATELET_	RGETS_P	IO_STEM	O_STEM	MY_META	MY_META	SIS_BY_T	IS_BY_T	IS_BY_T	IS_BY_T	RNING_E	RNING_E	EARNING_E	_TARGET	_TARGET
0.07736	-0.06618	0.13225	0.3749	0.05794	0.07829	0.0063	0.10577	0.14272	0.22458	-0.18346	-0.04598	0.0883	0.248	0.24936	-0.18724	0.10213
0.04696	0.13732	0.15006	0.38994	0.01699	0.08641	0.03852	0.13533	0.0728	0.17329	-0.14963	0.06454	0.11023	0.2629	0.21647	-0.16592	-0.04139
0.01331	-0.01715	0.13966	0.38318	0.09699	0.08386	0.01941	0.12315	0.11933	0.22398	-0.08573	0.0125	0.10291	0.2582	0.2605	-0.0611	0.02574
0.08021	-0.12391	0.16844	0.35215	0.11557	0.05057	-0.01827	0.12915	0.20392	0.23366	-0.13493	0.03134	0.10771	0.26083	0.19883	-0.14804	0.07957
0.02779	-0.15962	0.15637	0.3766	0.13904	0.04571	-0.01975	0.12059	0.21646	0.22731	-0.17017	0.02146	0.13882	0.29345	0.23033	-0.24742	0.11486
0.01156	-0.10538	0.16968	0.38732	0.08488	0.05843	0.04943	0.13217	0.21891	0.20538	-0.09395	-0.00299	0.16226	0.24768	0.23359	-0.14313	0.1738
0.01807	-0.0042	0.16131	0.37795	0.09714	0.06548	0.05145	0.14236	0.05931	0.20051	-0.0784	0.02861	0.11879	0.25121	0.21361	-0.07945	0.17607
0.04455	-0.3202	0.19299	0.35449	0.14613	0.04413	0.02363	0.11609	0.19433	0.2456	-0.1526	0.07287	0.15489	0.25557	0.25966	-0.14464	0.10871
0.03004	-0.29502	0.18259	0.35295	0.15471	0.02842	0.03547	0.10835	0.20455	0.22844	-0.07449	0.0653	0.14786	0.27051	0.23799	-0.12502	0.12976
0.04674	-0.30955	0.1631	0.34535	0.15999	0.03326	0.0115	0.11588	0.20311	0.22816	-0.08421	0.08782	0.1338	0.24923	0.19928	-0.287	0.17798
0.04761	-0.06745	0.12698	0.39596	0.05681	0.08967	-0.0085	0.12067	0.163	0.18424	-0.02609	-0.0492	0.15965	0.24727	0.24197	-0.14601	0.09127
0.03406	-0.29367	0.11658	0.37382	0.06994	0.07832	-0.01119	0.11946	0.10926	0.25596	-0.13941	-0.00284	0.14956	0.2554	0.24126	-0.12972	0.12266
0.01538	0.0297	0.12854	0.38256	0.00164	0.09528	0.04296	0.12612	0.08568	0.19784	-0.13527	0.04441	0.18382	0.24225	0.21414	0.02009	0.02056
0.03919	-0.20339	0.1569	0.37147	0.1102	0.06477	-0.03126	0.12438	0.04422	0.19168	-0.07515	8.6E-05	0.14599	0.24269	0.25465	0.01663	0.04623
0.07074	-0.04326	0.16561	0.38088	0.07544	0.0807	-0.00232	0.11735	0.06958	0.23356	-0.16359	-0.0012	0.10584	0.24806	0.28821	-0.10885	0.08512
0.03983	0.01445	0.09622	0.39577	-0.02469	0.10941	0.00859	0.13547	0.22757	0.17805	-0.03245	0.00819	0.09039	0.22714	0.23762	-0.15022	0.02114
0.06613	0.00205	0.15942	0.37781	0.10537	0.06856	0.00722	0.11948	0.12991	0.21572	-0.17373	-0.02778	0.12336	0.24417	0.25156	-0.17452	0.07819
0.01661	-0.25367	0.1829	0.36496	0.13646	0.04328	-0.04613	0.12331	0.20849	0.20912	-0.08988	0.02618	0.17147	0.27129	0.22052	-0.14622	0.04846

OUND_BY	BY_SALL	BY_SALL	NFKB1_T	TO_IONI	TO_IONI	TO_IONI	TO_IONI	TO_IONI	TO_IONI	ER_BOUN	P210_BC	P210_BC	SIS_BY_E	SIS_BY_E	ULATOR	TOSIS_L
0.05625	0.09647	0.05916	-0.03603	0.06342	0.11511	0.04429	0.1303	-0.04606	0.15621	0.0273	-0.06447	0.05813	0.07043	0.0886	-0.27074	-0.02693
0.03546	0.08014	0.07283	-0.12731	0.09855	0.10604	0.07571	0.14716	-0.00176	0.10753	0.05819	-0.09015	-0.02144	0.06809	0.07265	-0.04967	0.08722
0.03033	0.09638	0.07199	-0.1064	0.10965	0.11047	0.08294	0.15475	0.00521	0.13685	0.03628	0.01209	0.02615	0.06681	0.10703	-0.22935	0.03336
0.03073	0.11318	0.06179	-0.03154	0.08378	0.12564	0.03851	0.17686	-0.00663	0.18913	0.03867	0.0683	0.04378	0.05574	0.1069	-0.24291	-0.03141
0.01842	0.08841	0.07095	-0.03821	0.12081	0.10012	0.07251	0.16124	0.00489	0.19422	0.03859	0.09522	0.10435	0.05211	0.11323	-0.24925	-0.02127
0.05236	0.10699	0.05386	-0.21518	0.08752	0.08769	0.09418	0.1732	-0.00215	0.18203	0.03561	0.04449	-0.02174	0.06237	0.12044	-0.15589	0.07193
0.03485	0.08731	0.06385	-0.11709	0.10714	0.09728	0.07821	0.16132	0.02173	0.17855	0.04868	0.06038	-0.02214	0.06002	0.11275	-0.05848	-0.01244
0.01842	0.1028	0.05083	-0.05465	0.0975	0.0975	0.0218	0.20309	-0.01207	0.19768	0.04329	0.07675	-0.04061	0.04447	0.12287	-0.21966	-0.0759
0.01467	0.11045	0.0536	-0.17873	0.08315	0.10746	0.02083	0.1912	-0.00761	0.20865	0.03339	0.1285	0.01522	0.04771	0.12097	-0.29241	0.01057
0.00897	0.1199	0.05306	-0.0222	0.06141	0.09714	0.03239	0.19318	0.00431	0.20577	0.04124	0.14703	-0.06359	0.04204	0.10288	-0.24819	-0.06835
0.05748	0.08583	0.06784	0.01458	0.13656	0.13139	0.08652	0.13304	0.01363	0.11389	0.0352	-0.12992	-0.04899	0.06825	0.11546	0.1566	0.08326
0.03234	0.12101	0.05887	-0.05471	0.08989	0.10902	0.04528	0.15646	0.00221	0.1712	0.03924	-0.04156	-0.00608	0.05604	0.11359	-0.29294	0.05655
0.07953	0.07999	0.07471	-0.16389	0.14371	0.10074	0.09517	0.12771	0.00882	0.07098	0.03978	-0.20805	-0.02999	0.09467	0.09505	0.08279	0.04164
0.03978	0.08189	0.05363	-0.05228	0.11127	0.10971	0.07267	0.16245	0.00874	0.18338	0.0427	0.03544	0.01684	0.05153	0.09324	-0.14427	-0.06208
0.03359	0.09177	0.06196	0.04196	0.10738	0.12708	0.05662	0.1563	-0.00894	0.15131	0.03901	-0.03235	-0.00312	0.06728	0.10912	-0.06253	0.00141
0.0353	0.07011	0.06004	-0.00946	0.05998	0.11558	0.09177	0.11268	0.00475	0.07603	0.04706	-0.20657	0.00327	0.06614	0.05704	-0.1987	-0.00312
0.02904	0.10132	0.06407	-0.06261	0.10421	0.12409	0.05581	0.16834	0.00884	0.18258	0.06046	0.0187	0.02808	0.0309	0.09087	-0.30368	-0.05887
0.01944	0.09113	0.05509	-0.25392	0.12987	0.09361	0.03546	0.18918	-0.01156	0.17146	0.04202	0.08418	-3.1E-05	0.06468	0.10506	-0.26035	-0.02693

RHABDO	RHABDO	OUND_B	OCORTIC	OCORTIC	CER_MET	FFERENT	_AND_NE	AND_NE	AND_NE	AND_NE	AND_NE	AND_NE	N_METAS	N_METAS	TIATED_M	TIATED_M	NTIATED
0.14747	0.01377	0.04989	0.24682	0.13409	0.28028	0.13314	-0.0857	-0.16214	0.03756	0.13366	0.02543	0.13071	0.157	0.02056	0.28921	0.09513	0.0736
0.12457	0.09749	0.10463	0.25817	0.09329	0.28941	0.15011	-0.05002	-0.02383	-0.02225	-0.09832	-0.11524	0.14864	0.11262	0.04826	0.26698	0.10766	0.07764
0.1408	0.07285	0.12931	0.25291	0.14259	0.28433	0.18321	-0.10613	-0.20627	0.01094	0.10445	-0.03515	0.19559	0.14446	0.01112	0.27259	0.09403	0.02947
0.1678	0.04224	0.06146	0.23548	0.17911	0.25656	0.16182	-0.17523	-0.31988	-0.00329	0.10928	-0.00499	0.18406	0.14944	-0.01174	0.27006	0.12281	0.10926
0.13554	0.08915	0.08347	0.24468	0.17594	0.25081	0.14829	-0.08804	-0.22429	0.02681	0.08919	-0.14045	0.17183	0.13063	0.01107	0.24821	0.09438	0.02337
0.14028	0.08629	0.12549	0.25549	0.10569	0.26315	0.16914	-0.03978	-0.21907	0.0194	0.14432	-0.10095	0.14878	0.12921	0.00971	0.27305	0.12157	0.05551
0.14786	0.08678	0.09487	0.24267	0.13321	0.27296	0.1644	-0.12991	-0.14999	0.03813	0.12572	-0.18325	0.13152	0.13397	-0.00273	0.26905	0.10711	0.02246
0.18828	0.04944	0.00955	0.22915	0.17776	0.23834	0.14076	-0.22941	-0.3749	0.0772	0.06259	-0.11354	0.23819	0.13105	-0.02073	0.2349	0.12236	0.07499
0.166	0.07865	0.01736	0.23093	0.17157	0.24346	0.14221	-0.13107	-0.14712	0.06204	0.09692	-0.03699	0.22474	0.13996	-0.00713	0.2272	0.10981	0.06163
0.15054	0.05795	0.02291	0.23527	0.17836	0.25508	0.13508	-0.13686	-0.31391	0.00221	0.12565	-0.13876	0.1741	0.13889	0.00461	0.23281	0.08691	0.03257
0.13239	0.05502	0.15802	0.26346	0.13004	0.29132	0.15721	-0.01958	-0.24603	0.10122	0.09225	-0.05693	0.1391	0.12309	0.04965	0.31663	0.1034	0.09386
0.15629	0.04205	0.12074	0.25127	0.1606	0.28677	0.16048	-0.10988	-0.16306	0.03101	0.15618	0.00901	0.12835	0.14173	0.02797	0.28239	0.10596	0.07626
0.12623	0.05018	0.13335	0.25247	0.07218	0.2848	0.15823	-0.14513	-0.24594	0.04939	0.03646	-0.04859	0.08591	0.13975	0.0319	0.27542	0.11552	0.07089
0.15423	0.09302	-0.00136	0.23606	0.16418	0.25758	0.12835	-0.08666	-0.11655	0.10628	0.03731	-0.00886	0.17311	0.13059	0.00816	0.24041	0.12491	0.05817
0.15069	0.07321	0.09979	0.25504	0.16298	0.27786	0.15522	-0.12746	-0.14666	0.09722	0.16096	-0.12983	0.19792	0.13264	0.00925	0.2743	0.10903	0.08282
0.11231	0.04132	0.13463	0.26077	0.02608	0.29744	0.16669	-0.01314	-0.02937	0.08198	0.03578	-0.05902	0.12639	0.13286	0.03251	0.30086	0.11163	0.06364
0.14498	0.07574	0.01414	0.23919	0.15716	0.2574	0.10809	-0.14066	-0.16644	0.05447	0.08039	-0.00984	0.16664	0.14341	0.00706	0.25545	0.12762	0.07473
0.15933	0.06677	0.01961	0.24509	0.15866	0.23883	0.14813	-0.17097	-0.19387	0.03061	0.11824	-0.08185	0.20315	0.14665	0.01893	0.25829	0.1003	0.07448

NTIATED_	ENTIATE	ENTIATE	P63_DIRE	XIFEN_R	XIFEN_R	RCOMA_	RCOMA_I	INVASIV
-0.03687	0.08137	0.14659	0.04813	0.04824	0.14873	-0.03112	0.01989	0.07998
0.0126	0.06186	0.18374	0.04149	0.02881	0.12156	-0.05472	0.0004	0.06985
-0.04411	0.07149	0.16692	0.06654	0.07754	0.09913	-0.03594	-0.00738	0.07121
-0.01932	0.1032	0.14108	0.08089	0.04732	0.16755	0.0064	0.04286	0.07042
-0.01449	0.06606	0.15885	0.0612	0.03112	0.12421	-0.01491	0.01276	0.08628
-0.05767	0.06238	0.16999	0.05609	0.05189	0.13806	0.03547	-0.01046	0.08628
-0.05512	0.05056	0.15863	0.06752	0.05392	0.14165	0.03901	0.03539	0.06241
-0.01877	0.09692	0.13039	0.06824	0.05887	0.15313	0.03821	0.04521	0.09248
-0.01679	0.09437	0.14037	0.05159	0.06398	0.13788	0.02323	0.03649	0.08343
-0.02021	0.06232	0.14408	0.05728	0.05949	0.12425	0.00636	0.0433	0.08643
-0.0516	0.08309	0.16244	0.06436	0.0585	0.11172	-0.01297	0.00831	0.08206
-0.05292	0.08183	0.15591	0.0726	0.07319	0.14968	0.00776	-0.00101	0.09741
-0.08711	0.0943	0.16105	0.04381	0.06246	0.11779	-0.03217	-0.04626	0.08121
-0.01539	0.08806	0.14747	0.05058	0.03361	0.11728	0.00783	0.01799	0.07581
-0.01737	0.07619	0.1594	0.08346	0.05459	0.14383	-0.01249	0.00458	0.08585
-0.04567	0.08999	0.16452	0.07054	0.06925	0.09929	-0.07102	-0.02803	0.09628
-0.02323	0.08561	0.15109	0.05176	0.03066	0.16102	-0.02584	0.02968	0.09053
-0.00834	0.09114	0.14662	0.06471	0.05562	0.13833	-0.02827	0.03198	0.09652

_DIFFER	D_DIFFER	DIFFEREN	_DIFFERE	_DIFFER	DIFFERE	S_DCC_T	S_DCC_T	TN1_AND	S_NTN1_T	S_NTN1_T	_CARCIN	D_CARCIN	HYROID_C	INOMA_P	INOMA_P	HYROID_C	AGING_K	G_KIDNEY	G_KIDNEY	AGING_K
-0.31511	0.02557	-0.26493	0.15966	0.19982	0.04575	0.05638	0.12916	0.07889	0.1068	0.16274	0.019	0.11058	0.06987	0.05588	0.11905	0.16647	0.14828	0.16594	0.12221	0.10552
-0.07357	-0.07083	-0.34314	0.19393	0.12426	0.04018	0.07261	0.07051	0.02896	0.11068	0.14808	0.00898	0.10619	0.08104	0.03788	0.11233	0.11105	0.11487	0.13498	0.11838	0.08646
-0.18644	-0.20891	-0.22424	0.13876	0.22591	0.09188	0.05139	0.10288	0.06244	0.1261	0.21717	0.01958	0.1118	0.09543	0.04373	0.12981	0.23092	0.13836	0.16231	0.14823	0.12867
-0.29466	0.00649	-0.28589	0.13227	0.17786	0.02487	0.08515	0.13263	0.00613	0.1169	0.16637	0.02147	0.09427	0.06396	0.04868	0.10338	0.17137	0.10427	0.12358	0.15595	0.1726
-0.26606	-0.1228	0.01814	0.1772	0.18195	0.02666	0.09473	0.12415	0.03325	0.1022	0.1583	0.01221	0.08632	0.06751	0.03232	0.10695	0.18164	0.13436	0.14314	0.1077	0.15984
-0.29442	-0.1783	-0.05196	0.16236	0.19314	0.05721	0.07138	0.1336	-0.00328	0.05399	0.15134	-0.01858	0.1007	0.06567	0.03583	0.13493	0.19424	0.12868	0.14506	0.17193	0.14263
-0.25897	-0.35889	-0.15166	0.14961	0.12901	-0.01379	0.05622	0.09293	-0.00677	0.10102	0.06531	-0.00358	0.09044	0.03391	0.03019	0.11559	0.20241	0.11428	0.13353	0.16094	0.1541
-0.20347	-0.06582	-0.09864	0.10539	0.1161	0.01338	0.09834	0.12896	0.02049	0.09654	0.14584	0.0332	0.07819	0.01579	0.04739	0.08769	0.22666	0.11685	0.12905	0.15895	0.18445
-0.29285	-0.14453	-0.09354	0.12751	0.1392	0.02062	0.0961	0.12776	0.02846	0.11451	0.17885	0.02472	0.083	0.07224	0.0375	0.09441	0.17401	0.11827	0.13296	0.16272	0.19934
-0.35329	-0.14135	0.034	0.13648	0.11981	0.01529	0.10243	0.09136	0.02977	0.09657	0.17495	0.01749	0.07701	0.02016	0.03803	0.08893	0.22159	0.11643	0.12403	0.12663	0.18819
-0.13688	-0.16929	-0.3331	0.1565	0.19356	-0.00893	0.02222	0.12896	-0.04833	0.10052	0.19726	0.01025	0.119	0.07618	0.03995	0.1418	0.17314	0.176	0.20328	0.15472	0.0859
-0.25855	-0.03371	-0.15783	0.14604	0.18997	0.00053	0.08055	0.22581	0.00906	0.08853	0.16379	-0.00025	0.11846	0.09685	0.04148	0.13212	0.22517	0.16124	0.17589	0.15108	0.12804
-0.15988	-0.06702	-0.46627	0.16358	0.19571	0.01986	0.006	0.15721	0.02108	0.12434	0.17281	0.02553	0.11173	0.09716	0.05476	0.13425	0.12704	0.14485	0.16239	0.09737	0.01607
-0.19584	-0.15093	-0.1307	0.154	0.05855	0.00047	0.07449	0.12047	0.01367	0.10305	0.13923	0.01925	0.09456	0.0478	0.03696	0.10461	0.22699	0.13091	0.14768	0.14162	0.14143
-0.19047	-0.12293	-0.34208	0.15907	0.18493	0.03466	0.0792	0.0789	-0.02474	0.1084	0.11628	0.02632	0.10668	0.06098	0.04094	0.11636	0.24753	0.15504	0.16169	0.15282	0.12196
-0.09932	-0.11101	-0.31372	0.15109	0.20179	0.04623	0.05744	0.15878	0.0588	0.08669	0.14302	0.02567	0.12645	0.04513	0.05751	0.15276	0.16556	0.16024	0.18279	0.10782	0.03746
-0.28376	0.03583	-0.22987	0.14441	0.07704	0.05368	0.08574	0.12055	0.01611	0.10268	0.1873	0.0325	0.09043	0.05586	0.05	0.10213	0.21071	0.14374	0.15696	0.12853	0.13046
-0.24329	-0.13851	-0.04404	0.13104	0.14809	0.01983	0.09336	0.08069	-0.00162	0.12063	0.15037	0.03003	0.09041	0.02516	0.04439	0.09577	0.20934	0.13273	0.15109	0.14208	0.17436

_CANCER	CANCE	ERT_TAR	ERT_TAR	HDAC2_	F_EWSR	F_EWSR	MYELOID_	L_OF_FA	BITH_AML1	H_CBFB	_WITH_ML	ITH_PML	IA_WITH	CER_PR	OMA_COP	OMA_COP	LIOMA_LO	ANCER_16
0.07026	0.04199	0.12651	-0.01253	0.01467	-0.05711	-0.05332	0.067	0.07342	0.01153	0.19069	0.11896	0.06883	0.12495	0.05916	-0.01146	0.02594	-0.05763	-0.011
0.07275	0.01248	-0.01953	0.01713	-0.00185	0.01889	-0.03102	0.02193	0.0914	-0.03146	0.17987	0.08369	0.04602	0.12388	0.09234	0.01466	0.0502	-0.1084	-0.0684
0.07373	0.06329	0.05962	0.15804	0.03743	0.04396	-0.03895	0.01188	0.09456	0.01942	0.20784	0.12272	0.06461	0.11955	0.09827	0.04703	0.10921	-0.01458	-0.02675
0.06167	0.05539	0.04297	0.16605	0.01558	0.06583	0.01089	0.05115	0.08373	0.03135	0.22061	0.19649	0.06363	0.17683	0.05747	0.06804	0.08215	-0.01874	0.05675
0.06016	0.05808	0.19726	0.06915	0.02053	0.06339	-0.04844	0.02127	0.09699	0.0293	0.21991	0.15947	0.06058	0.14887	0.0513	0.03445	0.05793	0.00106	-0.01008
0.03913	0.04695	0.14037	0.08601	0.00341	-0.01256	-0.00948	0.01219	0.10989	0.02491	0.19519	0.09639	0.09663	0.1303	0.1054	0.03743	0.10277	0.01004	-0.02434
0.04831	0.03016	0.11992	0.29346	0.04697	-0.02992	-0.0622	0.06313	0.04754	0.04552	0.263	0.11414	0.10458	0.11264	0.06926	0.00697	0.08637	0.01778	0.01757
0.04344	0.05933	-0.00046	0.03978	0.02478	0.07481	-0.0327	0.06657	0.10048	0.05002	0.24816	0.21634	0.08275	0.1851	0.02238	0.05569	0.05655	0.04713	0.03959
0.04857	0.04366	0.15506	0.23109	0.02043	0.06254	-0.00344	0.09707	0.08483	0.06628	0.25517	0.205	0.08897	0.18465	0.04117	0.08351	0.06408	-0.05333	0.04428
0.06074	0.06046	0.16688	0.28278	0.02758	0.06415	-0.01032	0.08524	0.0521	0.0612	0.24936	0.21098	0.05808	0.18435	0.03266	0.07103	0.07537	0.02259	0.06232
0.04533	0.05307	0.00563	0.05356	0.00221	0.00837	-0.04044	0.01385	0.07407	0.00386	0.17711	0.08368	0.06921	0.07702	0.10553	-0.00256	0.08538	0.06627	0.0139
0.05284	0.02765	0.18858	0.23996	0.02981	0.02223	-0.04985	0.06077	0.08812	0.01726	0.19334	0.08859	0.09086	0.11214	0.07276	0.00251	0.08107	0.02645	-0.03386
0.05043	0.05926	-0.0033	0.03733	0.0315	-0.1198	-0.05467	-0.02009	0.06827	0.05196	0.14309	0.01015	0.07025	0.02988	0.0998	0.02208	0.12629	0.02539	-0.05251
0.06724	0.05788	0.11073	0.06441	0.01721	0.06444	-0.04664	0.04557	0.08123	0.03124	0.22291	0.14549	0.05187	0.13747	0.05029	0.02274	0.07785	0.02078	-0.01252
0.04995	0.04079	0.09125	0.13656	0.01233	0.01912	-0.00829	0.02675	0.10237	0.04417	0.23023	0.13239	0.06625	0.12903	0.07074	-0.01777	0.05828	0.00944	0.00436
0.08889	0.04545	-0.00484	0.04255	0.02923	-0.03695	-0.09038	-0.02887	0.05734	-0.00701	0.09796	0.02246	0.02577	0.04465	0.10826	-0.05625	0.10355	0.00169	-0.06763
0.04811	0.05038	0.12624	-0.01032	0.01758	0.04861	-0.03614	0.04146	0.07589	0.02133	0.21088	0.13653	0.05974	0.14247	0.03566	0.06031	0.07804	-0.05564	-0.03692
0.05538	0.04913	0.13124	0.27077	0.01247	0.07803	-0.04325	0.04455	0.09029	0.01808	0.22309	0.17973	0.06264	0.16066	0.04654	0.07325	0.05787	0.02364	0.03688

ANCER_16	_BLOOD	I_BLOOD	MMP14_C	MMP14_TA	P14_TAR	MMP14_T	PONSE_T	TO_TNF_	TO_TNF_	SPONSE_T	E_TO_TR	E_TO_TR	NC_TARG	NC_TARG	SE_TO_C	SE_TO_C	ESPONSE	SPONSE
-0.03762	0.0661	0.04525	0.04149	0.04102	0.00751	0.0717	0.1762	0.19266	0.21857	0.27052	0.23388	0.16531	0.05685	0.05092	0.08712	0.10139	0.06985	0.10749
-0.04174	-0.01185	0.03974	0.05275	-0.0399	-0.00614	0.07293	0.1322	0.14171	0.17669	0.2282	0.24934	0.13599	0.02892	0.04815	0.06881	0.09158	0.05517	0.09358
-0.00766	0.03241	0.09965	0.06838	-0.00372	-0.02204	0.05467	0.15887	0.16148	0.17516	0.25521	0.2429	0.12207	0.09065	0.08212	0.10728	0.1009	0.08218	0.11594
-0.03176	0.05152	0.09295	0.0675	0.02855	-0.02877	0.08202	0.15847	0.17265	0.25248	0.31349	0.22551	0.16692	0.06636	0.11358	0.13907	0.11508	0.10117	0.14122
-0.04641	0.04936	0.09781	0.06995	-0.02126	-0.00824	0.06953	0.14442	0.15846	0.20323	0.28746	0.21222	0.1319	0.02671	0.08862	0.11329	0.1066	0.09464	0.11838
-0.08052	-0.07135	0.12709	0.07138	-0.07011	0.0572	0.08553	0.14507	0.14265	0.24364	0.31091	0.24431	0.12587	0.10999	0.05141	0.06809	0.11044	0.05966	0.10555
-0.01137	-0.08733	0.12347	0.04969	-0.00885	0.0997	0.09518	0.13243	0.16269	0.21367	0.2895	0.25067	0.12094	0.0788	0.08068	0.09352	0.10962	0.07882	0.11398
-0.02637	-0.0049	0.15052	0.05948	0.04835	0.01242	0.07745	0.17919	0.1821	0.23856	0.31056	0.23571	0.1564	0.01219	0.13006	0.1603	0.12433	0.11407	0.1579
0.00802	0.01774	0.15683	0.0779	0.01534	0.04932	0.09779	0.15612	0.14611	0.25473	0.3306	0.23726	0.1454	0.03949	0.12277	0.16156	0.12074	0.12003	0.15321
-0.02303	0.04438	0.10732	0.0945	0.00095	-0.03452	0.08702	0.14742	0.13951	0.25555	0.33662	0.24307	0.13196	0.00317	0.11414	0.14984	0.10836	0.12261	0.13335
0.00963	0.0502	0.07359	0.06193	-0.00952	0.01326	0.06929	0.14038	0.19587	0.17857	0.23151	0.2497	0.07808	0.11783	0.04325	0.03183	0.1124	0.02453	0.09896
-0.0533	0.07125	0.14352	0.08055	-0.00088	0.00596	0.0725	0.13028	0.12201	0.23342	0.29201	0.21734	0.09759	0.10422	0.04761	0.07014	0.10075	0.06318	0.09773
-0.01883	-0.01322	0.03722	-0.03623	0.05191	-0.02158	0.03925	0.11046	0.14297	0.17249	0.23097	0.23994	0.07406	0.08127	0.01366	0.01357	0.1152	0.01565	0.09258
0.00563	0.06677	0.08546	0.06969	0.01037	-0.03595	0.0569	0.1781	0.20199	0.23112	0.27719	0.25405	0.17644	0.00909	0.09943	0.10455	0.11157	0.08119	0.12259
-0.05565	0.07826	0.13683	0.05853	-0.03851	-0.02501	0.06745	0.14406	0.1533	0.20847	0.27171	0.21585	0.10191	0.08821	0.08384	0.07836	0.10968	0.07456	0.10495
-0.09602	0.08679	0.02564	0.07778	-0.02004	-0.05344	0.05654	0.13017	0.14867	0.17617	0.20851	0.25525	0.13009	0.12488	0.00292	0.01195	0.08787	0.00396	0.07574
-0.05272	0.07234	0.10353	0.06806	0.02896	-0.06585	0.056	0.14447	0.15394	0.17499	0.22706	0.2321	0.134	-0.00412	0.08995	0.1148	0.09655	0.09386	0.11231
-0.02903	-0.01751	0.11818	0.08659	0.04418	-0.03874	0.08711	0.15151	0.13209	0.22936	0.30796	0.25115	0.1519	0.03265	0.10176	0.13585	0.10947	0.0964	0.13724

O_HGF_V	O_HGF_V	LYMPHO	ORECTAL	ECTAL_A	ECTAL_A	ORECTAL	AY_AND_	LYMPHO	CD24_TAR	CD24_TAR	ATITIS_V	ATITIS_V	LTRATIN	LTRATIN	NF_THER	NF_THER	TS_OF_K
0.08604	0.09875	-0.01437	-0.02981	-0.44724	0.178	0.02018	0.09716	0.09242	0.04617	0.01096	0.08198	0.16178	0.24613	0.13875	0.07074	-0.08809	0.15404
0.08502	0.07794	-0.02907	-0.06055	-0.16417	0.21144	0.01334	0.07607	0.06535	-0.00569	-0.05987	0.05387	0.16727	0.25472	0.13475	0.02636	-0.02003	0.04636
0.08096	0.11606	0.01091	-0.01042	-0.47331	0.25232	-0.00872	0.19618	0.06704	-0.00278	-0.04822	0.07195	0.17597	0.24117	0.15516	0.09601	0.0309	0.06229
0.09723	0.14265	0.0659	0.07331	-0.03822	0.21948	0.04769	0.12039	0.10527	-0.00298	-0.07832	0.12749	0.17145	0.23181	0.13343	0.137	0.0214	0.13053
0.08888	0.12093	0.04368	0.026	-0.11872	0.2336	-0.03993	0.19675	0.08538	0.00419	-0.06726	0.1204	0.18598	0.23595	0.12005	0.14322	-0.016	0.07152
0.08661	0.0912	-0.00987	0.06553	0.05478	0.22951	0.02311	0.15636	0.0613	0.10133	-0.01599	0.05813	0.16948	0.23354	0.1301	0.09472	0.02152	0.13575
0.08461	0.11055	0.02909	0.09267	-0.4298	0.25697	0.04817	0.1677	0.07749	0.10238	-0.08328	0.06367	0.17539	0.22685	0.12898	0.10961	0.0114	0.1255
0.11037	0.15917	0.09208	0.08523	-0.03911	0.25403	-0.01783	0.1265	0.08234	0.0356	-0.07678	0.12382	0.16945	0.22917	0.12771	0.14679	-0.01573	0.1184
0.1082	0.15862	0.11094	0.07917	-0.07434	0.24405	-0.05428	0.13163	0.12663	0.09437	-0.03775	0.14109	0.17797	0.22545	0.11586	0.22401	0.0662	0.12236
0.10579	0.14171	0.10527	0.09368	-0.10393	0.21928	0.0016	0.17123	0.11875	0.05662	-0.06099	0.08947	0.18719	0.22457	0.10869	0.19052	0.0382	0.11942
0.08043	0.06954	-0.07888	-0.01524	-0.01268	0.25324	0.10326	0.17128	0.10239	-0.01069	-0.05864	0.02795	0.17596	0.22138	0.14608	0.06892	0.00398	0.07807
0.08259	0.08734	-0.01789	0.05575	-0.16294	0.21899	0.05167	0.23845	0.06982	0.08008	-0.10043	0.06198	0.16864	0.22331	0.15639	0.10162	-0.01922	0.10415
0.09341	0.05179	-0.14618	-0.02084	-0.46521	0.21736	0.03741	0.07987	0.10028	0.01977	-0.05601	0.0349	0.15895	0.22711	0.17512	0.04966	0.10209	0.04402
0.09258	0.11628	0.03028	0.00794	0.09072	0.23924	0.02479	0.14044	0.10545	0.04157	-0.09602	0.10459	0.18102	0.22648	0.13642	0.13928	-0.02066	0.08885
0.08998	0.09663	-0.00778	0.02349	0.06623	0.28641	0.07331	0.23933	0.0946	0.02077	-0.06298	0.10526	0.17994	0.22585	0.16888	0.09022	-0.06103	0.11381
0.05798	0.04779	-0.10531	-0.0707	-0.20292	0.26181	0.03659	0.20159	0.11978	-0.04847	-0.036	0.04326	0.15763	0.20975	0.12578	0.05302	0.07383	0.08102
0.0884	0.11477	0.01544	0.01691	-0.05302	0.22009	0.01989	0.2159	0.1075	-0.01744	-0.08246	0.09363	0.15371	0.22273	0.14228	0.07244	0.04264	0.11263
0.09087	0.13926	0.05724	0.02019	0.03347	0.28638	-0.03569	0.1175	0.10148	-0.00301	-0.06478	0.09973	0.16871	0.23067	0.13124	0.12688	0.02899	0.06996

HFS_OF_K	PONSE_T	ONSE_T	F_SIGNA	F_SIGNA	Z_MDM2	DN_PPAR	APC_MYC	M_APC_T	APC_TAR	ARGETS_	APC_TAR	PATHWAY	MESENCH	MESENCH	BY_INSUL	BY_INSUL	AT_INSUL	AT_INSUL
0.12133	0.24794	0.10589	0.10862	0.08334	-0.06202	0.19481	0.05683	-0.04885	0.04093	0.01746	0.09171	0.01515	0.14799	0.11358	-0.18661	0.02599	-0.09663	0.09086
0.06412	0.27253	0.08559	0.12196	0.08781	-0.18471	0.10344	0.08904	-0.04386	-0.00822	0.05312	0.07538	0.03737	0.12188	0.10334	-0.18075	-0.03775	-0.13144	0.15224
0.12551	0.28189	0.13875	0.13745	0.13966	-0.03961	0.15541	0.06858	-0.02935	0.03781	0.05173	0.09264	0.10088	0.15305	0.13983	-0.13914	0.00855	-0.09057	0.15128
-0.00013	0.26624	0.20029	0.15054	0.16518	-0.05251	0.17364	0.0496	-0.05119	0.09292	0.01054	0.08437	0.04035	0.16672	0.11806	-0.24517	0.01308	-0.03133	0.13485
0.00673	0.25718	0.18165	0.13376	0.1233	-0.14065	0.1296	0.04796	-0.06243	0.08634	0.02705	0.07668	0.02998	0.13378	0.10588	-0.20126	-0.03751	-0.07113	0.08897
0.03518	0.27542	0.15786	0.13905	0.10585	-0.17275	0.10168	0.06404	-0.0245	0.05798	0.0351	0.13334	0.08068	0.1635	0.13908	-0.08321	-0.01104	-0.1484	0.06274
0.05917	0.27495	0.20769	0.15206	0.17213	0.02863	0.11534	0.06339	-0.03882	0.06569	0.0279	0.09452	0.10596	0.15566	0.12621	-0.12271	0.01967	-0.08874	0.10444
0.05867	0.27225	0.19573	0.14641	0.15817	-0.06682	0.14226	0.03152	-0.05805	0.11026	-0.0118	0.06516	0.03239	0.15574	0.07241	-0.29316	0.00425	-0.04025	0.09536
-0.0029	0.26378	0.23313	0.12656	0.19021	-0.07708	0.1288	0.04259	-0.06973	0.10546	0.00298	0.06497	0.05476	0.17674	0.08869	-0.25357	-0.00186	-0.08969	0.06396
0.06423	0.25003	0.23089	0.14546	0.2006	-0.12754	0.11318	0.03054	-0.05829	0.09881	0.00231	0.05761	0.03942	0.16026	0.07738	-0.22759	0.00904	-0.00332	0.0408
0.06572	0.26752	0.09698	0.14902	0.05665	-0.12681	0.15134	0.07373	-0.0285	-0.01228	0.05258	0.10537	0.09724	0.16771	0.16125	-0.10567	-0.02359	-0.05368	0.08985
0.29657	0.2748	0.18095	0.16424	0.15595	-0.10581	0.1209	0.05375	-0.0188	0.06137	0.03095	0.10472	0.08598	0.14617	0.1631	-0.18571	0.0634	-0.08946	0.0921
0.09404	0.26425	0.08617	0.13408	0.03332	-0.09464	0.12409	0.06682	-0.04974	-0.0506	0.04229	0.06156	0.09323	0.16243	0.15999	-0.0808	0.02739	0.0397	0.06769
0.22394	0.28829	0.14006	0.13298	0.10576	-0.02287	0.18926	0.042	-0.05794	0.06521	0.00706	0.05605	0.04063	0.14952	0.09008	-0.23286	-0.04503	-0.045	0.1423
0.07636	0.27895	0.1621	0.13792	0.12551	-0.06352	0.10338	0.07202	-0.03723	0.03638	0.05387	0.09767	0.06266	0.15274	0.13876	-0.22035	-0.00058	-0.09163	0.14154
0.16786	0.27454	0.11933	0.10873	0.08699	-0.00902	0.20865	0.09301	-0.02601	-0.02333	0.07231	0.10551	0.05276	0.138	0.14552	-0.16579	-0.00481	-0.08573	0.13009
0.12704	0.2887	0.13333	0.16885	0.08146	-0.03122	0.13568	0.05222	-0.04418	0.06433	0.01656	0.05104	0.06485	0.15342	0.07136	-0.24381	-0.01727	-0.09992	0.15142
0.05947	0.26945	0.21847	0.14095	0.18426	-0.03192	0.14115	0.05032	-0.04415	0.08738	0.00349	0.08678	0.03434	0.18962	0.08319	-0.26644	-0.03595	-0.14101	0.1207

I_NEOPLA	F_CXCR	F_CXCR	LT_T_CEL	TS_OF_T	FSH_RE	NSE_TO_	NSE_TO_	E_TO_GO	E_TO_GO	TYLATION	LATION_	YLATION_	ETICALLY	L_SEN	MATE_DEV	ATE_DEV	ATE_DEV	ATE_DEV
-0.11349	0.0651	0.03741	0.14367	0.09459	-0.03725	0.13214	0.1303	0.14062	0.11497	-0.02854	0.04857	-0.03871	0.20789	0.08696	0.03753	0.14918	0.03205	0.0259
-0.10833	0.02147	0.07617	0.14672	-0.1752	-0.15997	0.11469	0.11959	0.12685	0.10067	-0.07154	0.02819	0.01251	0.20184	0.05518	0.07992	0.12573	0.00525	-0.00903
-0.16066	0.00424	0.07938	0.15154	-0.15928	-0.14442	0.11765	0.08334	0.12869	0.06481	-0.02203	0.03641	-0.09505	0.06889	0.06433	0.0708	0.13986	-0.00385	0.00258
-0.0692	0.1672	0.12397	0.1502	-0.08738	0.03913	0.15949	0.13851	0.17628	0.1163	-0.01115	0.05665	-0.04657	0.15997	0.08503	0.08341	0.14645	0.04461	0.02139
-0.15335	-0.03517	-0.03806	0.12657	-0.17652	-0.05671	0.13204	0.12139	0.14971	0.09737	0.11473	0.03401	-0.00426	0.16141	0.05444	0.05308	0.11683	0.03502	-0.02029
-0.03055	0.04585	0.14859	0.14891	-0.02494	-0.13632	0.12374	0.09524	0.12183	0.08971	-0.08183	0.05088	-0.02155	0.09726	0.04482	0.052	0.15444	0.03819	-0.00173
-0.07671	0.10387	0.208	0.14985	-0.0176	-0.18066	0.11873	0.08242	0.11929	0.07437	-0.06456	0.08435	-0.00672	0.04182	0.08658	0.06805	0.17168	0.03492	0.00829
-0.08485	0.09994	0.05193	0.1228	-0.09376	0.03088	0.15277	0.1384	0.17223	0.11326	-0.02238	0.05198	-0.06205	0.09881	0.07073	0.08197	0.1619	0.05351	4.4E-05
-0.08486	0.12561	0.0248	0.1385	-0.07256	-0.08486	0.14102	0.12228	0.16506	0.0924	-0.0584	0.06044	-0.10233	0.11243	0.10283	0.06602	0.14274	0.04618	-0.016
-0.08281	0.13581	0.0693	0.14581	-0.05747	-0.08169	0.1316	0.11197	0.14922	0.08802	-0.019	0.05948	-0.04815	0.12859	0.0749	0.05401	0.15057	0.05015	-0.01086
-0.08704	-0.08281	0.16269	0.14654	-0.14476	-0.11351	0.1007	0.10607	0.11632	0.08341	-0.09091	0.05894	0.00931	0.21503	0.05568	0.02711	0.18177	0.03771	0.0358
-0.07641	0.04831	0.18916	0.15329	-0.07188	0.07909	0.09565	0.11352	0.11238	0.0898	-0.04173	0.07571	-0.06721	0.10332	0.07381	0.07112	0.19607	0.03996	0.04154
-0.10294	-0.09907	0.22732	0.15589	-0.09154	-0.07456	0.14653	0.11999	0.14768	0.11188	-0.12056	0.0495	0.01163	0.14363	0.03807	0.05459	0.15836	-0.01508	0.038
-0.14403	-0.02332	0.10903	0.13798	-0.12395	-0.00914	0.12709	0.11113	0.14791	0.0838	-0.08492	0.04591	-0.0328	0.14592	0.07893	0.07659	0.1294	0.02534	0.01423
-0.07306	-0.02966	0.11262	0.14358	-0.17114	-0.08427	0.1234	0.10714	0.13284	0.09005	-0.05847	0.0808	-0.01153	0.20157	0.04746	0.05202	0.14998	0.04362	0.01709
-0.11719	-0.33225	0.15749	0.1475	-0.1473	-0.08374	0.11891	0.08886	0.12043	0.07955	-0.09502	0.03138	0.03859	0.18654	0.01261	0.00827	0.12723	0.00193	0.04693
-0.11372	0.14582	0.07624	0.12817	-0.11011	0.03418	0.14148	0.12086	0.1563	0.09997	0.00186	0.05497	0.01655	0.15014	0.09998	0.10827	0.13575	0.01616	0.01294
-0.13466	0.11194	0.08595	0.13931	-0.14216	-0.01392	0.148	0.1104	0.16435	0.08791	-0.02068	0.03829	-0.10831	0.05196	0.04936	0.08173	0.16878	0.03613	-0.00018

ATE_DEV	ATE_DEV	VELOPME	VELOPME	ELOPME	ELOPME	EVELOP	VELOPME	ELOPME	ELOPME	VELOPME	VELOPME	ELOPME	S_IN_PR	S_IN_PR	GETS_OF	K_INTERA	REIT_IKK	NORMAL_
0.14656	0.07925	0.20118	0.04723	0.25925	0.14288	0.01404	0.15456	-0.04868	0.25524	0.07718	0.15748	-0.08675	0.10851	-0.10859	0.09694	0.03958	0.07034	0.12419
0.16054	0.03034	0.21727	0.08244	0.24084	0.12376	0.05771	0.16906	-0.02589	0.16047	0.09928	0.30351	-0.01108	0.13701	0.1004	0.10261	-0.03153	0.05812	0.15953
0.13963	0.06245	0.18654	0.06285	0.1264	0.20645	0.05708	0.16695	-0.05207	0.20774	0.08041	-0.12507	0.16581	0.10007	0.07839	0.09338	-0.01395	0.08349	0.22631
0.13513	0.06663	0.19496	0.00805	0.18845	0.1933	-0.00068	0.15641	-0.02964	0.23628	0.01728	-0.11943	0.20405	0.09102	0.01811	0.10576	0.02449	0.05007	0.3193
0.13599	0.04834	0.1927	0.07852	0.19614	0.22396	0.0386	0.16007	-0.03955	0.17079	0.06895	0.09459	0.23682	0.12574	0.02371	0.06751	0.00724	0.05604	0.19986
0.12502	0.03264	0.17697	-0.02677	0.03911	0.33382	0.07431	0.17453	-0.02703	0.09852	0.11014	-0.37168	0.25995	0.12361	-0.00575	0.09225	-0.00377	0.07741	0.09449
0.11675	0.05224	0.19208	0.07117	0.10447	0.31608	0.06649	0.15885	0.00336	0.12873	-0.18562	0.04263	0.09205	0.10311	0.08827	0.13841	0.0057	0.10249	0.18659
0.13121	0.08253	0.18731	-1E-05	0.16226	0.23606	0.02531	0.16599	-0.05236	0.20509	0.12223	-0.24044	0.34685	0.08163	0.09763	0.09889	0.0007	0.04369	0.34372
0.11699	0.07346	0.18783	0.00221	0.16709	0.22053	0.02325	0.15771	-0.02507	0.25324	0.10584	-0.44583	0.31861	0.09733	0.03259	0.1063	0.02231	0.05497	0.31283
0.11559	0.07836	0.20041	0.02193	0.17174	0.24742	0.06857	0.16077	-0.00523	0.21266	0.07135	-0.22406	0.27879	0.09866	-0.0247	0.07857	0.01388	0.04702	0.23348
0.13986	0.07704	0.18116	0.11998	0.25614	0.18136	0.01709	0.1681	-0.04386	0.15054	-0.28273	0.29258	-0.18907	0.13946	-0.04875	0.109	0.03343	0.0668	0.15254
0.13075	0.06104	0.20129	0.07904	0.22301	0.36156	0.03419	0.18826	-0.03815	0.19173	-0.06786	0.21349	0.04198	0.11027	-0.00151	0.17964	-0.00421	0.03289	0.21922
0.14451	0.0719	0.15389	0.09268	0.26938	0.26721	0.0198	0.17317	-0.03524	0.27195	-0.09223	0.28395	-0.12958	0.11521	-0.02351	0.05389	0.03485	0.04907	0.04184
0.1308	0.05743	0.20574	0.08138	0.20048	0.25747	0.02126	0.19423	-0.06807	0.22391	0.12652	0.11238	0.18109	0.08457	-0.03065	0.07708	0.02754	0.04552	0.24385
0.13729	0.05607	0.18384	0.03788	0.18552	0.25225	0.01122	0.14635	-0.03705	0.1562	0.05569	0.14973	0.2115	0.11685	0.03369	0.16293	0.01713	0.07089	0.2342
0.13204	0.07092	0.19387	0.10438	0.21967	0.22013	0.02274	0.17977	-0.05129	0.16603	-0.13219	0.25295	-0.31284	0.12139	-0.00229	0.11457	0.02946	0.05894	0.14203
0.14125	0.05011	0.19483	0.01614	0.13762	0.22236	-0.00713	0.1811	-0.02294	0.17694	0.02958	-0.22001	0.13653	0.10122	-0.03066	0.09321	0.00165	0.04035	0.18716
0.12495	0.08604	0.18619	0.02648	0.20172	0.1893	0.03181	0.14823	-0.04087	0.21478	0.05687	0.02114	0.22967	0.08416	0.06708	0.07746	0.01456	0.08158	0.2598

YLATED_D	YLATED_IN	_CARCI	IN_CARC	D_SERUM	ETS_AND	ETS_AND	GETS_RE	ESPONSE	SERUM_R	SERUM_R	PDGF_S	ARGETS_I	ARGETS_I	NFKB_S	MLL_TA
0.2301	-0.25788	-0.01016	0.15125	0.07011	0.15714	0.10627	0.1836	0.09673	0.13246	0.09049	-0.0698	-0.07915	-0.04414	0.05278	0.02966
0.2098	-0.12507	-0.07965	0.17692	0.08358	0.19528	0.14052	0.19965	0.11733	0.134	0.08046	-0.04106	-0.16372	-0.09835	0.04786	-0.03071
0.17379	-0.32799	0.00512	0.09556	0.10646	0.20077	0.14779	0.19619	0.11568	0.13608	0.07627	-0.10326	-0.17971	-0.07995	0.06549	0.00017
0.20094	-0.2637	0.12029	0.14559	0.06775	0.14391	0.11273	0.16827	0.09881	0.14393	0.09663	0.01722	-0.29895	-0.11966	0.08807	0.02436
0.14845	-0.07363	0.05131	0.14972	0.07597	0.14545	0.10492	0.17855	0.10169	0.14142	0.10334	-0.10919	-0.22144	-0.04337	0.07069	-0.03988
0.15391	-0.15977	-0.00167	0.14829	0.11109	0.18562	0.13664	0.19204	0.10996	0.13483	0.08441	-0.10052	-0.22514	-0.08303	0.09122	0.078
0.19983	-0.02995	0.06535	0.15106	0.11204	0.19119	0.16827	0.19135	0.12125	0.13016	0.0791	-0.0993	-0.25235	-0.06684	0.10144	0.17814
0.1898	-0.33028	0.09525	0.14619	0.05596	0.13154	0.10976	0.15889	0.09968	0.13984	0.09665	-0.03315	-0.11169	-0.10147	0.07817	0.04927
0.16295	-0.15765	0.08303	0.12844	0.06424	0.1308	0.10431	0.15798	0.10103	0.1354	0.0881	-0.08336	-0.17803	-0.0928	0.16363	0.06602
0.15828	-0.24256	0.06468	0.13882	0.06835	0.14362	0.10618	0.16918	0.10388	0.14577	0.09432	-0.01737	-0.18121	-0.10915	0.09069	0.04173
0.19024	-0.32464	0.00311	0.14311	0.08706	0.18714	0.14902	0.21279	0.11823	0.12527	0.06937	-0.08248	-0.15557	-0.08536	0.09141	-0.06549
0.17846	0.11362	0.03297	0.15348	0.11261	0.17332	0.15085	0.20573	0.11082	0.13441	0.06855	0.01686	-0.15016	-0.03741	0.07734	0.00821
0.1452	-0.30985	-0.01097	0.14659	0.10215	0.19072	0.15621	0.20299	0.13195	0.1267	0.06592	0.02168	-0.14087	0.03826	0.05039	-0.00099
0.1586	-0.18196	0.06196	0.15193	0.05893	0.16662	0.12624	0.18699	0.10568	0.13707	0.10625	-0.14635	-0.2315	-0.10194	0.07409	0.04831
0.17782	0.01286	0.0225	0.18756	0.08321	0.18056	0.14364	0.20097	0.08861	0.13817	0.07803	-0.16682	-0.19121	-0.02687	0.07589	-0.04205
0.15958	0.06923	-0.00032	0.14097	0.12917	0.2004	0.18448	0.20357	0.11478	0.1161	0.06817	-0.0561	-0.26329	-0.04109	-0.02946	-0.0976
0.19637	-0.14268	0.0182	0.17645	0.0791	0.15609	0.10506	0.17538	0.12314	0.13672	0.08729	-0.08076	-0.25955	-0.11041	0.07911	0.01495
0.17873	-0.1913	0.06699	0.13106	0.06421	0.14176	0.12737	0.16976	0.10403	0.14691	0.09602	-0.0916	-0.02265	-0.10952	0.00847	0.03845

ER_MYC_	MYB_POL	MYB_POL	ARGETS_(	ARGETS_(	GETS_O	RGETS_O	KDM5B_TA	KDM5B_TA	D_HEMAN	MET_SIG	COGENES	FORMATI	FORMATI	TORY_RES	TORY_RES	HIT_TAR	HIT_TAR	CA_HIF1_T	AND_HDA	AND_HDA
0.1322	-0.16321	-0.15023	0.11562	0.12321	0.19189	0.18449	0.10549	0.08401	-0.01289	0.19829	0.17231	-0.067	-0.01162	0.09368	-0.0679	0.02248	0.00014	0.30435	0.05413	0.08617
0.13891	-0.09518	-0.02645	0.04527	0.02114	0.16176	0.23272	0.07874	0.09577	-0.03038	0.18996	0.16501	0.02581	0.00173	0.05243	-0.03554	0.12213	0.12729	0.27578	0.0277	0.06971
0.15778	-0.13186	-0.18115	0.07697	0.03834	0.17364	0.24854	0.08562	0.00531	-0.0611	0.19362	0.18555	-0.03056	0.09023	0.16742	-0.05109	0.28589	0.13272	0.25306	0.03261	0.10205
0.11573	-0.09479	-0.13485	0.04747	0.15618	0.18438	0.24315	0.07576	0.05156	-0.06354	0.1772	0.15967	-0.10456	0.11737	0.02012	-0.02751	0.22798	0.29459	0.29149	0.06253	0.0917
0.11077	-0.06351	-0.05585	0.03978	0.01368	0.15632	0.26034	0.04859	0.09252	-0.06512	0.18931	0.16046	-0.07714	0.07765	0.1003	-0.02721	0.11932	0.12875	0.27124	-0.03027	0.06141
0.14488	-0.12665	-0.17695	0.07353	0.0206	0.15899	0.2803	0.09756	0.03765	-0.09273	0.20567	0.15566	0.04173	0.05545	0.11153	-0.07892	0.05367	0.14096	0.2885	0.03472	0.07061
0.17758	-0.14247	-0.1955	0.11347	0.00307	0.18152	0.23096	0.07438	0.01287	-0.02847	0.21019	0.12632	0.07114	0.15557	0.04636	-0.04105	0.24649	0.20044	0.29476	0.00707	0.09802
0.09313	-0.11365	-0.12726	0.06789	0.11234	0.19696	0.25012	0.07458	0.05365	-0.10988	0.17836	0.16732	-0.0761	0.09469	0.08109	-0.00934	0.2003	0.20503	0.27693	0.02396	0.08636
0.09342	-0.08916	-0.09453	0.08204	-0.01605	0.15722	0.23872	0.07135	0.03593	-0.09305	0.18183	0.15279	-0.02783	0.14947	0.0805	0.06523	0.2304	0.19705	0.26869	0.03758	0.08906
0.11986	-0.06905	-0.08395	0.08103	0.00768	0.14688	0.21623	0.05789	0.04991	-0.10518	0.16956	0.14419	-0.01525	0.17183	0.09987	0.01211	0.17076	0.25241	0.26338	-0.02184	0.08919
0.16125	-0.12014	-0.15337	0.14132	0.0952	0.17525	0.23021	0.10389	0.04402	-0.04535	0.19416	0.15743	0.06546	0.02072	0.08248	-0.0338	0.11582	-0.04463	0.27749	0.04618	0.08825
0.15799	-0.13158	-0.16142	0.03016	0.18721	0.17769	0.2508	0.09848	0.0306	0.01663	0.19699	0.17796	0.06486	0.09032	0.0296	-0.04659	0.03126	0.19102	0.26684	0.05207	0.089
0.13583	-0.06989	-0.14042	0.10269	0.13668	0.21176	0.14704	0.10741	0.01089	-0.03185	0.19287	0.19527	-0.00049	0.00419	0.11851	-0.08349	0.12383	0.02046	0.27326	0.04638	0.06967
0.12874	-0.14357	-0.13095	0.05506	0.08755	0.17709	0.21526	0.06604	0.05303	-0.08948	0.19705	0.14853	-0.03446	0.05389	0.03916	-0.0233	0.16472	0.07969	0.28101	0.04612	0.08128
0.15908	-0.1387	-0.22426	0.13607	0.1632	0.18335	0.23174	0.08111	0.05835	-0.04098	0.21499	0.16089	-0.01082	0.06524	0.11077	-0.02865	0.15457	0.1024	0.2726	0.06619	0.07281
0.18719	-0.16967	-0.04061	0.10961	0.14369	0.18231	0.23074	0.08788	0.01344	-0.07377	0.17318	0.1665	0.02766	0.05295	0.10986	-0.05603	0.24443	0.06388	0.27166	0.07802	0.069
0.11676	-0.14428	-0.17486	-0.00186	0.10344	0.19351	0.22216	0.05135	0.05642	-0.04576	0.17443	0.1442	-0.01097	0.00545	0.15284	-0.06455	0.15767	-0.01671	0.30459	0.04937	0.09137
0.10085	-0.11877	-0.03589	0.08836	0.03376	0.17587	0.2544	0.05756	0.0105	-0.08598	0.17543	0.15864	-0.06318	0.17654	0.03255	-0.0025	0.16963	0.28624	0.26798	0.02873	0.08005

DAC1_TA	DAC1_TA	DAC2_TA	DAC2_TA	DAC3_TA	DAC3_TA	NA1_ANT	HARYNGE	HARYNGE	GEAL_CA	GEAL_CA	ET_HNF1A	ET_HNF1A	R_HNF1A	R_HNF1A	SPONSE_	SPONSE_	SPONSE_
0.07839	0.10889	0.0993	0.17234	0.02916	0.0888	-0.06361	0.08865	0.04756	0.06867	0.09336	0.1042	0.12512	-0.06299	0.04884	0.1272	0.20549	0.14747
0.07128	0.0882	0.07771	0.16592	0.0164	0.06969	-0.08265	0.03127	0.06241	-0.00249	0.11569	0.08034	0.12771	-0.10659	0.00162	0.1232	0.18781	0.15581
0.07619	0.11097	0.09771	0.17934	0.02999	0.08991	-0.03736	0.05018	0.07896	0.02053	0.13049	0.0965	0.15208	-0.05306	0.02813	0.13983	0.21656	0.14424
0.06823	0.10274	0.10539	0.17351	0.0149	0.08211	0.01496	0.04174	0.07006	0.0432	0.09551	0.15086	0.1728	0.05584	0.05331	0.11501	0.19894	0.10464
0.05224	0.08791	-0.01646	0.15415	0.00513	0.07684	0.02637	0.03026	0.05222	-0.00042	0.09134	0.0822	0.16093	-0.02462	0.03306	0.11325	0.1735	0.10656
0.05964	0.08148	0.08056	0.14201	0.01614	0.05751	-0.01874	0.03306	0.06951	0.01492	0.08826	0.10554	0.18989	0.1292	0.04158	0.14255	0.17461	0.11849
0.06869	0.08582	0.07475	0.15891	0.0192	0.07656	-0.00299	0.01732	0.06388	0.03371	0.10118	0.13161	0.19453	0.13745	0.05442	0.12561	0.18206	0.1211
0.05887	0.09259	0.02428	0.16082	0.01275	0.08333	0.03895	0.02369	0.05797	0.00719	0.08687	0.12175	0.15934	0.036	0.05837	0.11244	0.19121	0.10023
0.05393	0.09278	0.07005	0.15616	0.01198	0.09224	0.04274	-0.0015	0.06984	0.00214	0.10409	0.12389	0.18055	0.06296	0.04689	0.10453	0.1793	0.09635
0.05136	0.08328	0.01369	0.15636	-0.00412	0.08072	0.06187	0.01225	0.06836	0.00745	0.09968	0.10289	0.15188	0.09088	0.0454	0.11492	0.18387	0.10736
0.06687	0.09972	0.10189	0.15669	0.03683	0.07158	-0.07757	0.09409	0.07431	0.10161	0.09457	0.12909	0.1388	0.01361	0.03553	0.13555	0.2273	0.15587
0.07497	0.10199	0.10654	0.16906	0.02672	0.08497	-0.03541	0.07814	0.07071	0.06113	0.12235	0.13006	0.14609	0.06667	0.04878	0.12664	0.21567	0.16071
0.08208	0.08617	0.0792	0.14489	0.05355	0.05361	-0.08706	0.09728	0.03828	0.06552	0.08517	0.08338	0.12645	0.00517	0.01098	0.14561	0.20056	0.17328
0.07136	0.09557	0.07123	0.1816	0.0161	0.07783	-0.00816	0.05348	0.04821	0.09525	0.09707	0.09964	0.1439	0.0048	0.06212	0.11653	0.22438	0.13854
0.08507	0.08858	0.09226	0.15333	0.03752	0.06372	-0.0295	0.04813	0.07649	0.07427	0.10787	0.08267	0.15997	-0.01392	0.0699	0.12983	0.21452	0.13452
0.0914	0.08816	0.10775	0.13857	0.03913	0.05449	-0.09859	0.05414	0.06836	0.04627	0.10459	0.10759	0.11763	-0.10437	0.03306	0.12146	0.22118	0.16589
0.08016	0.10263	0.11041	0.179	0.01125	0.07771	-0.018	0.07323	0.04311	0.03537	0.09676	0.13914	0.12731	-0.01969	0.05584	0.11946	0.20848	0.11613
0.05582	0.09094	0.08743	0.15702	0.01093	0.07872	0.02987	0.01445	0.06224	0.02524	0.10523	0.10467	0.15968	-0.00132	0.02035	0.11874	0.17884	0.10975

SPONSE_	SPONSE_	SPONSE_	SPONSE_	SPONSE_	SPONSE_	CER_TMR	CER_TMR	TIPLE_MS	IN_ACT	IN_ACT	MYELOMA	ASMA_CE	AL1_TAR	TOMA_WI	ASTROCY	ASTROCY	ANCER_G
0.14497	0.17273	0.05717	0.11951	0.26344	0.13318	0.15434	0.07707	0.12426	0.17822	0.09073	0.1844	0.15454	-0.21607	-0.4342	0.11593	-0.08202	-0.02321
0.15269	0.20941	0.05648	0.13961	0.24295	0.14043	-0.01808	0.10575	0.10866	0.16521	0.081	0.16747	0.14587	-0.1295	-0.42442	0.00911	-0.07393	-0.04315
0.17453	0.17846	0.0641	0.14753	0.27923	0.13157	0.01144	0.0736	0.12014	0.1456	0.09075	0.17425	0.15011	-0.15722	-0.43908	-0.10928	-0.02503	-0.00884
0.15422	0.15878	0.02554	0.1245	0.25213	0.10378	0.10037	0.04071	0.09996	0.15191	0.04003	0.1528	0.1338	-0.2467	-0.3528	-0.0994	-0.03195	0.00488
0.14393	0.16749	0.00797	0.14542	0.24244	0.10379	0.16475	0.05256	0.17931	0.15951	0.0847	0.16355	0.13737	-0.20011	-0.43155	-0.10226	-0.0936	-0.00155
0.14701	0.21868	0.03146	0.11335	0.25714	0.13505	0.04524	0.06093	0.1528	0.16773	0.10574	0.18673	0.17766	-0.24042	-0.12497	0.01294	-0.06091	0.00693
0.1233	0.17378	0.04937	0.18153	0.27183	0.13292	0.04661	0.05047	0.15201	0.1558	0.06318	0.16121	0.13112	0.00192	-0.10504	0.1432	-0.03748	0.01372
0.15318	0.15957	0.0248	0.12816	0.23663	0.08047	0.16877	0.03666	0.12935	0.15675	0.07154	0.15902	0.15001	-0.10584	-0.2612	-0.04871	-0.04704	0.03743
0.22075	0.15389	0.01399	0.11679	0.22116	0.10003	0.11452	0.05618	0.1342	0.15131	0.07477	0.1606	0.15861	-0.06501	-0.1398	0.04469	-0.01138	0.02019
0.14394	0.15315	0.0232	0.08602	0.23764	0.07477	0.13702	0.02573	0.1625	0.16141	0.09166	0.16464	0.13971	-0.1576	-0.30619	-0.0408	-0.04604	0.04287
0.14914	0.20206	0.07188	0.18913	0.25582	0.13397	0.09384	0.08375	0.14239	0.15871	0.05673	0.174	0.14148	0.05032	-0.39199	-0.15358	-0.10324	-0.01398
0.13	0.2335	0.04543	0.12509	0.25755	0.13957	0.04281	0.06323	0.1406	0.15876	0.08959	0.19419	0.1595	-0.08645	-0.41042	0.13682	-0.05929	0.01053
0.16054	0.20293	0.10174	0.17948	0.24386	0.16693	-0.09152	0.07213	0.13684	0.18475	0.04598	0.17882	0.16149	-0.09683	-0.3818	-0.01798	-0.05989	-0.02565
0.16321	0.1854	0.02063	0.1604	0.26809	0.1096	0.1229	0.04422	0.10496	0.15644	0.05036	0.15115	0.12576	-0.11536	-0.36398	-0.11937	-0.01648	0.02089
0.15672	0.18749	0.02273	0.15137	0.26823	0.108	0.17406	0.08635	0.1267	0.15512	0.10562	0.16808	0.15146	-0.30227	-0.40422	-0.09228	-0.11636	0.00263
0.13611	0.21788	0.08268	0.15376	0.27667	0.14595	-0.03771	0.05425	0.09318	0.16986	0.06271	0.16841	0.13221	-0.02901	-0.231	0.1595	-0.07614	-0.03275
0.15374	0.18385	0.05131	0.11879	0.25343	0.13146	0.07334	0.07233	0.0882	0.16018	0.05805	0.15671	0.12601	-0.23553	-0.40567	-0.03353	-0.06152	0.00249
0.14367	0.16591	0.02482	0.12978	0.26464	0.10427	0.14526	0.03779	0.11757	0.15142	0.07213	0.16005	0.12815	-0.02538	-0.4401	-0.09819	-0.08207	0.01099

ANCER_G	ANCER_G	ANCER_P	RCA2_TA	RCA2_TA	MYB_MOR	MYB_MOR	D_BMYB_T	AND_BL	AND_BL	CER_VS	CER_VS	CER_VS	CER_VS	CER_VS	CER_VS	LYMPHO	LYMPHO
0.11186	-0.00258	0.13308	0.03971	0.07659	0.1286	0.12483	0.13246	0.125	0.06372	0.09165	0.05116	0.09811	0.06254	-0.00942	0.02416	-0.43174	-0.04701
0.04053	-0.00637	0.13681	-0.02174	0.08337	0.10409	0.13652	0.12265	0.12952	0.09084	0.07439	0.06167	0.07397	0.03443	-0.00082	0.02752	-0.41924	-0.02125
0.06491	-0.04456	0.14157	0.04069	0.07187	0.11765	0.11246	0.08659	0.11139	0.06229	0.10524	0.03119	0.11281	0.05618	-0.05729	0.03622	-0.44161	-0.05674
0.08952	-0.04705	0.11391	0.05261	0.05823	0.12885	0.1233	0.07962	0.09979	0.0664	0.09251	0.07119	0.08213	0.05384	-0.0196	0.03181	-0.45376	-0.04953
0.06557	-0.00936	0.11023	0.07649	0.06758	0.09685	0.12838	0.04055	0.07957	0.05343	0.10684	0.04557	0.09154	0.04666	-0.06397	-0.01188	-0.4284	-0.03689
0.05024	-0.03673	0.14335	0.05136	0.05289	0.12342	0.12442	0.0783	0.1066	0.07822	0.0787	-0.00523	0.07959	0.06313	-0.04743	0.07147	-0.3257	-0.04456
0.03488	-0.04855	0.12803	0.02991	0.04165	0.14546	0.12052	0.11957	0.10692	0.08906	0.09379	0.03154	0.09116	0.07022	-0.06105	-0.01673	-0.40602	-0.00785
0.07887	-0.06493	0.08888	0.08601	0.05439	0.09754	0.1164	0.06095	0.09526	0.03957	0.08681	0.06876	0.09489	0.05805	-0.03383	-0.0134	-0.39845	-0.05951
0.07313	-0.04972	0.09236	0.06234	0.05015	0.10331	0.11228	0.06814	0.0966	0.04026	0.09088	0.06461	0.06261	0.05673	-0.01368	0.00739	0.11767	-0.01756
0.04023	-0.04985	0.09369	0.06942	0.05749	0.11817	0.11382	0.08937	0.10748	0.03956	0.07845	0.03386	0.06429	0.04968	-0.0229	-0.01156	-0.40814	-0.01269
0.0712	-0.05002	0.15188	0.03319	0.08347	0.13448	0.10345	0.08134	0.1035	0.07587	0.11406	0.06158	0.14993	0.05163	-0.02736	0.06079	-0.46873	-0.1371
0.09363	-0.02946	0.12459	0.0409	0.07106	0.12343	0.1149	0.06063	0.08583	0.07571	0.11097	0.01895	0.12207	0.07746	-0.03127	0.01862	-0.4564	-0.06755
0.10223	-0.01505	0.15565	0.03334	0.08201	0.12296	0.12618	0.11498	0.12945	0.08275	0.106	0.05343	0.06315	0.03372	0.01794	0.02397	-0.44689	-0.01082
0.08382	-0.0523	0.11766	0.0678	0.0682	0.09728	0.12113	0.08954	0.10321	0.07951	0.10151	0.03536	0.07584	0.04503	-0.01459	0.01351	-0.47736	-0.06067
0.05879	-0.02259	0.12496	0.09549	0.07864	0.11972	0.1287	0.07795	0.09464	0.08391	0.1013	0.06085	0.1409	0.05997	-0.04898	0.00418	-0.46186	-0.09541
0.04166	-0.00657	0.1553	-0.00146	0.09369	0.10958	0.11879	0.10101	0.10017	0.1142	0.09309	0.0326	0.11951	0.04808	-0.03944	0.06373	-0.4631	-0.09604
0.08838	-0.0026	0.1162	0.03029	0.0694	0.10929	0.12477	0.11066	0.11213	0.07902	0.09164	0.06471	0.09462	0.04437	-0.04249	0.01861	-0.46063	-0.08683
0.07113	-0.04305	0.10427	0.04416	0.06229	0.09551	0.11981	0.05355	0.08847	0.03541	0.09062	0.06919	0.0539	0.05292	-0.04254	-0.01451	-0.4372	-0.04845

LYMPHO	LYMPHO	LYMPHO	LYMPHO	LYMPHO	LYMPHO	_CURED_	_CURED	FOLLICU	FOLLICU	PLZF_TA	PLZF_TA	RC_TARG	RC_TARG	D_BY_EW	OF_EWS	OF_EWS	PARP1_TA
-0.05813	0.14167	0.05958	-0.15213	0.0986	0.05414	0.168	-0.1098	-0.07609	0.33312	0.01799	0.047	-0.00688	-0.20392	-0.02714	-0.02841	0.15421	0.28674
-0.06806	0.18438	0.01673	-0.18237	0.07157	0.13866	0.15093	-0.04938	-0.09775	0.32718	0.15607	0.19933	0.00585	-0.20003	-0.15306	-0.05056	0.10712	0.29722
-0.03979	0.20019	0.03953	-0.14634	0.09869	0.11123	0.19012	-0.13417	-0.01684	0.35772	0.11575	0.07094	0.05707	-0.10341	-0.05505	-0.00013	0.15603	0.3096
-0.02681	0.19027	0.06031	-0.13679	0.12684	0.10451	0.17127	-0.05407	-0.01509	0.34956	0.06897	0.10767	0.01531	-0.04442	-0.01773	-0.07568	0.18748	0.29807
0.0072	0.23638	0.04954	-0.13193	0.09843	0.12691	0.14873	-0.09673	-0.00597	0.32462	0.05736	0.07259	-0.01296	-0.04994	-0.00987	-0.05679	0.1424	0.27678
-0.01136	0.20218	0.03732	-0.13741	0.00949	0.06915	0.15266	-0.05071	0.08393	0.34092	0.02647	0.13505	-0.00534	-0.1743	-0.15857	0.00562	0.11984	0.30845
0.01689	0.15208	0.03554	-0.21467	0.09618	0.1036	0.17357	-0.08162	0.01709	0.35673	0.00723	0.10202	0.06336	0.01797	-0.15456	-0.00433	0.15054	0.29988
-0.02048	0.20946	0.04982	-0.10818	0.1455	0.1308	0.16826	-0.04069	-0.007	0.32446	0.04241	0.09401	0.08094	-0.04885	0.00482	-0.02454	0.1644	0.2822
0.00822	0.20396	0.31097	-0.07038	0.14354	0.21002	0.16763	-0.03703	-0.00875	0.33684	0.01744	0.09404	0.02769	-0.04504	-0.00077	-0.07833	0.2134	0.2747
0.03912	0.21844	0.04411	-0.12358	0.14871	0.21868	0.17277	-0.037	0.07562	0.33303	0.00443	0.12325	0.02977	-0.07484	-0.00926	-0.0496	0.18	0.27764
-0.04273	0.13051	0.05787	-0.15463	0.06561	0.0757	0.15498	-0.099	-0.01246	0.35077	0.22649	-0.00795	0.05378	-0.04242	-0.08242	0.03191	0.09964	0.30186
-0.06339	0.14006	0.03006	-0.21743	0.06694	0.07956	0.16558	-0.16819	-0.0063	0.35489	0.02845	0.12291	0.00041	-0.17646	-0.11793	0.03438	0.14692	0.28775
-0.09744	0.09086	0.01965	-0.14874	0.00524	0.14141	0.12131	-0.13723	-0.03506	0.35223	0.12146	0.07533	-0.0812	-0.1757	-0.14128	0.00247	0.03098	0.28464
-0.04673	0.22072	0.09157	-0.17519	0.0849	0.1264	0.16498	-0.0936	-0.00436	0.33412	0.11146	0.10134	0.00664	-0.16725	-0.0538	-0.01479	0.16959	0.28422
-0.04083	0.16488	0.06928	-0.19698	0.10014	0.0967	0.15253	-0.18419	0.02115	0.34222	0.06471	0.06473	0.0516	-0.22149	-0.03234	0.00507	0.12861	0.29692
-0.12503	0.14407	-0.00174	-0.14436	0.00318	0.10944	0.16057	-0.16153	-0.00555	0.36261	0.08043	0.0987	-0.12189	-0.1796	-0.14835	0.04531	0.11947	0.2791
-0.00898	0.16557	0.04402	-0.16493	0.09331	0.09325	0.16438	-0.12001	-0.02124	0.3266	0.12923	0.08129	0.06971	-0.04281	-0.04686	-0.0128	0.16661	0.28498
-0.00659	0.17147	0.03394	-0.13856	0.1368	0.12115	0.15948	-0.04907	-0.04431	0.33733	0.01982	0.1484	0.06466	0.00736	-0.03438	-0.02766	0.13074	0.26131

PARP1_T	SPONSE_I	RESPONS	RESPONS	NFE2L2_T	NECK_CA	T_CANCE	ST_CANCE	T_CANCE	CANCER_	CANCER_	ANCER_	CANCER_	ANCER_N	ANCER_N	CER_REL	CER_REL	ER_REL
0.19815	0.14426	0.1165	-0.26555	0.1372	0.03336	0.03043	0.09956	0.13062	0.05224	0.03684	0.05004	-0.03075	0.17927	-0.03595	0.13081	-0.00964	0.08218
0.17429	0.16008	0.1352	-0.29748	0.1117	0.03057	-0.01431	0.0786	0.10615	0.15098	-0.00863	-0.00025	-0.02214	0.0334	-0.07521	0.11564	-0.05266	-0.01357
0.18866	0.15562	0.11843	-0.33182	0.16309	0.05849	0.01143	0.09548	0.10487	0.05972	0.01927	0.05078	-0.00777	0.12001	0.01737	0.13568	-0.02075	0.03791
0.15834	0.16281	0.09816	-0.28863	0.14422	0.0184	0.00701	0.07649	0.13811	-0.0253	0.04576	0.07368	-0.05227	0.14051	0.07172	0.12989	0.01119	0.01409
0.18827	0.11971	0.00927	-0.3175	0.17466	0.0108	0.00227	0.05969	0.11764	-0.01215	0.02039	0.05608	-0.03679	0.02308	0.07883	0.09999	-0.02725	-0.00075
0.20294	0.17289	0.00832	-0.24903	0.13089	0.00231	0.00952	0.08177	0.09969	0.03612	0.08047	0.07055	-0.05544	0.10218	0.05904	0.11552	-0.02682	0.02776
0.22937	0.17318	0.06417	-0.37963	0.11586	-0.0227	0.01362	0.06985	0.09524	0.14389	0.12282	0.05649	-0.01199	0.1773	0.0645	0.11541	-0.01795	0.03543
0.20707	0.14235	0.05541	-0.34086	0.16756	-0.06245	0.01659	0.05253	0.12338	0.10151	0.04156	0.06004	-0.03965	0.06766	0.10332	0.09022	0.01643	0.02965
0.21643	0.15241	0.05903	-0.22877	0.15701	-0.05975	0.01454	0.0775	0.11213	0.11053	0.0655	0.08123	-0.05232	0.03276	0.13796	0.11448	-0.03043	0.02824
0.17288	0.11858	0.04839	-0.06249	0.17028	-0.04444	0.00844	0.04243	0.11437	0.10587	0.08698	0.073	-0.04975	0.01203	0.13902	0.08236	0.01543	-0.00096
0.21124	0.19835	0.0916	-0.1134	0.12121	0.06063	0.0014	0.13076	0.10972	0.09606	-0.01453	0.05884	0.00427	0.26698	-0.05566	0.16753	0.01202	0.01263
0.19916	0.19331	0.06935	-0.31712	0.10206	0.04534	0.01505	0.0937	0.10672	-0.02788	0.09063	0.07056	-0.03719	0.1589	0.01905	0.12148	0.02705	0.04362
0.22643	0.15918	0.11905	0.12692	0.07409	0.06501	0.02073	0.07204	0.10076	0.03518	0.00307	-0.01059	-0.0066	0.32967	-0.11706	0.10623	-0.04865	0.0448
0.1814	0.14351	0.07231	-0.30942	0.12446	-0.00017	0.02798	0.07059	0.15607	0.08633	0.03162	0.05987	0.01427	0.23403	0.03263	0.12401	0.01949	0.04161
0.20803	0.1775	0.04	-0.35261	0.14714	0.02316	0.01728	0.09885	0.14854	0.06052	0.05645	0.07839	0.00243	0.0645	-0.00166	0.13397	0.03744	0.00769
0.22306	0.17164	0.11043	-0.39752	0.10634	0.04253	-0.00031	0.11088	0.06356	0.00936	0.03319	-0.00079	0.00267	0.11388	-0.15183	0.12608	-0.05719	-0.00949
0.21433	0.13725	0.10539	-0.10903	0.13734	-0.02487	0.01935	0.07895	0.13616	0.05839	0.04732	0.05515	-0.02108	0.14023	0.02601	0.13294	0.02079	0.03184
0.15028	0.13161	0.05996	-0.00777	0.15323	-0.02843	-0.0038	0.06958	0.09458	0.14437	0.01984	0.06665	-0.04419	0.14938	0.08775	0.1185	-0.05478	0.00494

CER_REL	CER_REL	CER_REL	CER_REL	CER_REL	ER_RELA	ER_RELA	NDOTHE	ENDOTHE	PONSE_T	PONSE_T	PONSE_T	PONSE_T	LIVER_C	ERT_TAR	ERT_TAR	IN_HEA	CTAL_CA
0.03473	0.35529	0.24468	0.02948	0.0971	0.10484	-0.32008	0.49005	0.04646	-0.00058	-0.08921	0.04106	-0.00572	0.00379	0.16141	0.11211	0.0456	0.08463
-0.09534	0.14145	0.2067	-0.11603	0.15668	-0.06463	0.02705	0.49163	-0.01488	-0.05102	-0.04675	0.00905	-0.06399	0.02516	0.12694	0.11695	-0.01519	0.03504
0.0759	0.27004	0.25714	-0.08392	0.06078	0.02884	-0.18437	0.49234	0.02925	-0.02453	-0.04872	0.01341	-0.04573	0.02586	0.17681	0.10958	0.02896	0.07257
0.00655	0.36075	0.25107	-0.01834	0.05223	0.08112	-0.3198	0.4911	0.10146	-0.00352	-0.03197	0.071	-0.0209	-0.00225	0.1899	0.07353	0.01314	0.09933
-0.0389	0.29575	0.27412	0.00549	0.04462	0.13972	-0.24444	0.49251	0.05508	-0.00543	-0.07714	0.07048	-0.05896	-0.02272	0.16829	0.06859	0.06039	0.08954
0.00503	0.43738	0.27472	0.07694	0.19363	0.12566	-0.39279	0.49181	6.1E-05	-0.06644	-0.06571	0.04861	-0.06078	0.02616	0.16738	0.09443	-0.01318	0.02964
-0.01031	0.45341	0.22654	0.13071	0.11761	0.09595	-0.14312	0.49216	0.05716	-0.03115	-0.0713	0.06993	-0.06166	0.01483	0.16163	0.10468	0.00889	0.04451
-0.08623	0.37555	0.26803	-0.18498	0.00951	0.11227	-0.14305	0.49146	0.11772	-0.07011	-0.05675	0.07515	-0.02747	-0.02189	0.17576	0.06862	0.04629	0.11619
-0.06482	0.38189	0.28778	-0.06483	0.07653	0.0459	-0.1833	0.49163	0.12029	-0.04336	-0.00394	0.0992	-0.04015	0.0059	0.18617	0.06355	0.00308	0.11436
-0.06897	0.39493	0.31781	0.06628	0.04786	0.10932	-0.35545	0.49163	0.09503	-0.01227	-0.04452	0.11253	-0.05331	0.00667	0.17192	0.08797	-0.00917	0.08765
0.13914	0.36586	0.17003	-0.08274	0.10129	0.11011	-0.20737	0.49163	-0.00128	-0.03994	-0.02715	-0.04171	-0.04274	0.02183	0.15858	0.10737	0.06796	0.07333
0.03603	0.41483	0.21603	0.06851	0.01338	0.12228	-0.43941	0.49199	-0.01017	-0.01731	0.00061	0.02764	-0.02699	0.03367	0.18523	0.11335	0.05545	0.05562
0.01864	0.3331	0.17747	-0.1946	0.10902	0.00252	-0.19742	0.49234	-0.07587	-0.04257	-0.09375	-0.07276	-0.0418	0.04757	0.16113	0.11997	0.01216	0.06425
-0.09573	0.29752	0.34175	0.02733	0.09734	0.15769	0.03875	0.49163	0.07001	-0.03353	-0.02127	0.06466	-0.04902	-0.01765	0.19576	0.08253	0.01242	0.10054
0.05713	0.38312	0.21138	-0.07229	0.06869	0.17799	-0.3167	0.49199	0.03754	-0.04269	-0.09089	0.01374	-0.0141	0.0384	0.16696	0.11199	0.03308	0.08308
0.08027	0.26651	0.21243	0.08388	0.13903	-0.05817	-0.28317	0.49269	-0.05335	-0.05736	-0.03277	-0.05352	-0.04836	0.06146	0.14027	0.12694	0.0956	0.05281
-0.04844	0.19676	0.25104	-0.1118	0.0848	0.12034	-0.21213	0.49181	0.05897	-0.03052	-0.05172	0.05579	-0.0337	-0.01727	0.16228	0.09442	-0.00695	0.06377
-0.08816	0.3183	0.32189	-0.14861	0.0577	-0.01161	-0.17987	0.49304	0.07729	0.00393	-0.04086	0.08137	-0.04398	-0.0436	0.20707	0.07811	0.02898	0.10446

CTAL_CA	OF_IE86	CANCER_	CANCER_	K_MYC_T	T_EURO	RT_EURO	AST_EUR	AST_EUR	RS_LUNG	RS_LUNG	ING_FAM	UND_BY	PONSE_T	PONSE_T	ETS_OF	ETS_OF	E_E2F1	S_TRANS	IN_22Q11	US_22Q
0.08895	0.0984	-0.02594	0.12425	-0.34525	-0.08828	0.298	0.12272	0.18477	-0.13544	0.19081	-0.43123	-0.00629	0.01371	0.06571	0.11582	0.09494	0.1532	0.34958	-0.02645	0.10992
0.10432	0.07594	-0.05878	0.13845	-0.43826	-0.0405	0.32917	0.11909	0.19691	-0.04704	0.12758	-0.32185	-0.00161	0.10217	0.04734	0.1332	0.11462	0.08611	0.31927	-0.0369	0.11136
0.13889	0.13816	-0.01919	0.13831	-0.47049	-0.07936	0.33202	0.11864	0.17694	-0.13168	0.15074	-0.32899	-0.07211	0.03701	0.07125	0.16285	0.10517	0.05949	0.41829	-0.00426	0.08254
0.09169	0.06019	-0.03253	0.10727	-0.43298	0.03951	0.3173	0.13169	0.19132	-0.16774	0.17495	-0.42041	-0.09425	0.08974	0.09064	0.19705	0.09915	0.12195	0.4137	0.13382	0.05492
0.09681	0.09179	-0.04722	0.10409	-0.44161	-0.00914	0.31747	0.12425	0.18497	-0.00121	0.14795	-0.31365	-0.09364	0.05296	0.04475	0.12376	0.10304	0.08188	0.3061	-0.03186	0.08828
0.1451	0.12047	-0.08289	0.13865	-0.35617	-0.03043	0.28932	0.11383	0.18497	-0.10406	0.20439	-0.34002	-0.06685	0.08729	0.05915	0.13146	0.09042	0.08351	0.35612	0.10771	-0.03161
0.1799	0.12075	-0.05111	0.14192	-0.42505	-0.08039	0.33013	0.12112	0.17041	-0.0523	0.17894	0.08531	-0.05455	0.10909	0.08	0.1907	0.07097	0.09907	0.3712	0.02578	0.072
0.09471	0.01037	-0.00393	0.07294	-0.16787	0.10341	0.32666	0.1336	0.17809	-0.00355	0.17018	-0.33127	-0.06406	0.04189	0.07276	0.1262	0.10639	0.08082	0.39943	0.10059	0.08217
0.09639	0.04073	-0.02387	0.08565	-0.43826	0.08544	0.3278	0.12968	0.17762	-0.06785	0.14391	-0.26849	-0.07625	0.05294	0.07544	0.16507	0.10478	0.10262	0.4276	0.11876	0.05731
0.12674	0.06563	-0.04094	0.09183	-0.26475	0.03039	0.32048	0.12954	0.17714	-0.0354	0.17013	-0.31918	-0.03898	0.09363	0.05039	0.21333	0.0902	0.13125	0.33072	0.15231	0.07055
0.18048	0.14712	-0.05236	0.15572	-0.03382	-0.10038	0.28146	0.11143	0.18967	0.0126	0.17177	-0.16551	0.01423	0.06658	0.09831	0.13247	0.08342	0.06847	0.35454	0.00972	0.02738
0.16907	0.12337	-0.03504	0.12735	-0.35054	-0.14672	0.30939	0.11742	0.18094	-0.05387	0.19367	-0.27351	-0.06523	0.10568	0.07764	0.17757	0.0593	0.09312	0.37226	0.07024	0.10034
0.14171	0.12215	-0.02874	0.14975	-0.46239	-0.12957	0.22411	0.1079	0.18487	-0.1929	0.1915	-0.32073	-0.02033	0.09835	0.07374	0.14637	0.09688	0.12747	0.24171	0.03038	0.09779
0.11173	0.02982	-0.00739	0.10164	-0.24027	-0.07685	0.32053	0.12676	0.19042	-0.03053	0.14242	-0.26936	-0.07387	0.07303	0.09472	0.12399	0.07658	0.07761	0.41015	0.07527	0.08588
0.14916	0.11821	-0.04614	0.13539	0.15572	-0.09113	0.30777	0.11925	0.18388	-0.09138	0.16356	-0.28262	-0.07787	0.05667	0.1136	0.15824	0.09141	0.15723	0.37728	0.04579	0.0863
0.18717	0.12723	0.03047	0.1563	-0.47278	-0.17697	0.26442	0.10965	0.17906	-0.20489	0.13416	-0.29523	0.00702	0.0105	0.06424	0.17663	0.07634	0.10597	0.33926	0.01144	0.08894
0.08407	0.00488	0.00392	0.08058	-0.43245	-0.08426	0.3103	0.12268	0.1828	-0.11388	0.16963	-0.38767	-0.09136	0.07221	0.08929	0.12863	0.09912	0.09005	0.39567	0.07247	0.0705
0.11447	0.04942	-0.05424	0.08837	-0.44918	0.02661	0.31668	0.13079	0.17877	-0.05093	0.17783	-0.16429	-0.0856	0.0524	0.06467	0.2109	0.10785	0.0512	0.406	-9.7E-06	0.07187

CANCER_	MOR_FIEL	ADIPOGEN	ADIPOGEN	IC_CELL_	OCYTE_M	ESR1_TA	ESRRA_TA	RRA_TAR	RESPONS	RESPONS	RRA_TAR	RESPONS	EAL_CAR	EAL_CAR	PONSE_TC	LSM1_TA	LSM1_TA	OGESTIN	ED_WITH	HNF4A_TA
0.08973	0.16556	-0.08221	0.20842	-0.35129	0.19725	0.05711	0.08878	-0.0089	0.1004	-0.01775	0.11021	-0.1105	-0.03189	0.23142	-0.03954	-0.04863	-0.01308	0.09862	0.36332	-0.29761
0.11548	0.10594	-0.01598	0.08729	-0.19399	0.20373	0.01543	0.07301	0.02557	0.06035	-0.03683	0.08484	-0.16276	-0.01036	0.20778	-0.08398	-0.35306	0.01028	0.11478	0.29659	-0.25515
0.11857	0.16016	-0.02689	0.18765	-0.39819	0.22594	0.07741	0.09431	0.02068	0.15776	0.01444	0.11094	-0.16938	0.06361	0.18358	-0.02707	-0.23638	0.04018	0.08959	0.29101	-0.24561
0.09723	0.21925	-0.00354	0.27102	-0.38774	0.17686	0.06644	0.06908	-0.02028	0.14346	-0.06797	0.09261	-0.16085	-0.00174	0.18957	-0.01232	-0.19928	0.00575	0.0947	0.32377	-0.13145
0.10594	0.20331	-0.02565	0.25148	-0.35603	0.34472	0.04298	0.071	-0.0344	0.11962	-0.05564	0.09591	-0.2195	-0.03293	0.2126	-0.04165	-0.21915	0.02201	0.10512	0.34894	-0.21251
0.11277	0.18904	0.02384	0.20665	-0.40029	0.37352	0.05604	0.06906	0.00171	0.11214	-0.01753	0.08612	-0.15061	-0.09186	0.182	0.07922	-0.28543	0.01259	0.11848	0.31004	-0.25711
0.11639	0.19762	0.11799	0.20335	-0.42176	0.14681	0.03609	0.0856	0.01942	0.10708	-0.04674	0.10219	-0.17787	0.02887	0.18413	0.04611	-0.15162	0.00518	0.10248	0.31039	-0.19836
0.10459	0.23473	0.01295	0.26697	-0.41682	0.28538	0.04221	0.06468	-0.04425	0.08068	-0.04688	0.09182	-0.14631	-0.01585	0.21805	-0.0206	-0.26064	0.01216	0.07091	0.2906	-0.23677
0.10786	0.21445	0.02609	0.25185	-0.39967	0.25423	0.02604	0.05643	-0.0363	0.10208	-0.07231	0.07954	-0.18432	-0.02106	0.1822	0.00091	-0.25206	-0.00501	0.09953	0.26269	-0.32108
0.10668	0.23264	-0.04581	0.28754	-0.39039	0.23968	0.00109	0.06657	-0.01582	0.05494	-0.09812	0.08992	-0.2204	-0.0499	0.19929	0.01793	-0.19439	-0.00394	0.13097	0.289	-0.22262
0.11943	0.17518	-0.03247	0.21998	-0.30552	0.10598	0.08346	0.0904	0.00706	0.17143	-0.0133	0.10927	-0.09938	-0.01886	0.20787	-0.03458	-0.12819	0.03387	0.07545	0.32372	-0.15929
0.1203	0.14372	-0.01636	0.19816	-0.33906	0.21396	0.06099	0.09683	-0.01385	0.13759	-0.02357	0.12141	-0.09021	0.02302	0.21572	0.0014	-0.24791	0.00129	0.13598	0.36023	-0.18634
0.12929	0.10434	-0.15213	0.10013	-0.32374	0.23949	0.07063	0.07597	0.01937	0.11004	0.01433	0.0877	-0.14245	-0.01093	0.2393	0.01185	-0.08582	0.05072	0.10777	0.23987	-0.17988
0.09869	0.1915	0.00876	0.27146	-0.38346	0.19367	0.01121	0.07932	0.00055	0.06391	-0.02287	0.09787	-0.18662	-0.00848	0.19103	-0.04343	-0.24678	0.0027	0.09314	0.30048	-0.2194
0.09997	0.17287	0.02016	0.24723	-0.38184	0.20179	0.08334	0.07948	0.00554	0.14773	-0.01226	0.09672	-0.02211	-0.03987	0.219	-0.06402	-0.22369	0.02122	0.11585	0.35839	-0.20789
0.10306	0.06503	-0.09773	0.04126	-0.26617	0.08127	0.05394	0.10978	0.00354	0.13924	-0.00505	0.13401	-0.15324	-0.03039	0.2206	-0.04852	-0.47622	0.00334	0.1018	0.24587	-0.15157
0.10889	0.17883	-0.0074	0.20926	-0.37264	0.20204	-0.01329	0.09202	-0.02518	0.03312	-0.08494	0.12217	-0.13147	-0.02568	0.19603	-0.03009	-0.26371	0.0149	0.0988	0.35034	-0.20446
0.1054	0.17289	-0.01058	0.16909	-0.39867	0.25941	0.03074	0.07233	-0.04265	0.08882	-0.0428	0.09912	-0.18091	-0.03814	0.20697	-0.03231	-0.3221	0.05124	0.0909	0.31195	-0.31149

STASIS_S	STASIS_S	FIED_IN_	TCFL_TA	RESPONS	TO_TSA_	TO_TSA_	GU_LIVER	PANCRE	PLACEN	LIVARY_G	U_TESTIS	U_THYMU	ONCOGEN	RAS_TAR	RAS_TAR	G_CANCE
0.09244	0.04593	-0.15511	0.14427	0.03609	-0.01864	0.15661	-0.08558	-0.02747	0.09977	-0.19922	-0.06379	-0.25183	0.24912	0.08811	-0.00048	0.05839
0.01156	0.0531	-0.08415	0.12733	0.01383	0.01586	0.22165	-0.07714	-0.07515	0.10162	-0.24626	-0.06248	-0.28023	0.21874	0.08832	-0.00384	0.06071
0.07944	0.05589	-0.07458	0.19045	0.02342	0.01241	0.20294	-0.02201	0.03425	0.16637	-0.10648	-0.07114	-0.16019	0.23339	0.071	0.01488	0.08599
0.07737	0.03413	-0.05482	0.09185	0.01448	-0.08251	0.1034	0.04436	-0.00363	0.12177	-0.10522	-0.05916	-0.19362	0.253	0.08807	0.03914	0.09928
0.04723	0.00079	-0.09247	0.19736	0.01601	-0.09228	0.16558	0.00101	0.05858	0.02063	-0.16256	-0.05235	-0.05072	0.23658	0.09742	-0.00558	0.0725
0.07282	0.02413	-0.07101	0.23059	0.04223	-0.03807	0.20552	0.18472	-0.0077	-0.01901	-0.24384	-0.07795	-0.03777	0.23065	0.05676	0.01512	0.096
0.06049	0.0829	-0.19138	0.21278	-0.02008	-0.07307	0.23117	0.10293	-0.03422	0.04547	-0.11776	-0.08333	-0.07865	0.24104	0.0756	0.03334	0.08805
0.03262	0.04691	-0.13769	0.1101	0.02844	-0.06309	0.14524	0.07347	0.00287	0.20025	-0.18184	-0.07394	-0.10527	0.24914	0.06452	0.02729	0.11146
0.03151	0.05222	-0.14835	0.13823	0.02868	0.02338	0.08402	0.08418	0.06495	0.13199	-0.10745	-0.09827	-0.04482	0.24497	0.05353	0.06705	0.12415
0.02657	0.00708	-0.08624	0.21581	0.05186	-0.06211	0.15932	0.10833	-0.01321	0.01977	-0.04427	-0.08457	-0.05316	0.23256	0.05115	-0.00873	0.09864
0.11191	0.03173	0.03074	0.03084	-0.01424	-0.01778	0.15761	-0.02565	-0.04479	0.22516	-0.10047	-0.0384	-0.27526	0.23071	0.06318	0.01191	0.06742
0.07661	0.03556	-0.03894	0.21284	-0.00628	0.03271	0.11064	0.09293	-0.0218	0.15769	-0.2861	-0.08245	-0.20391	0.23309	0.06765	0.03257	0.09325
0.07774	0.04035	-0.02181	0.06052	0.01515	-0.01773	0.11404	0.00069	-0.02585	0.1324	-0.21019	-0.0637	-0.23944	0.20169	0.08757	-0.01348	0.04777
0.04429	0.03281	-0.16459	0.13383	-0.00153	-0.04364	0.18579	-0.00216	0.0481	0.19072	-0.21818	-0.06912	-0.16429	0.25004	0.10982	-0.01278	0.08699
0.07133	0.05378	-0.09666	0.10302	0.0023	-0.06995	0.11224	0.0371	-0.01562	0.10717	-0.0881	-0.05566	-0.21326	0.24248	0.0967	0.01271	0.07665
0.07976	0.04166	0.05178	0.17202	-0.08631	-0.05834	0.1622	-0.0815	0.00356	0.25137	-0.14058	-0.0276	-0.1907	0.20741	0.06576	-0.01114	0.03925
0.03676	0.03108	-0.2094	0.17016	-0.00368	0.00268	0.20364	-0.0276	-0.02275	0.21458	-0.13742	-0.08836	-0.16284	0.23986	0.1044	-0.01452	0.08077
0.04718	0.01196	-0.13098	0.1976	0.03101	-0.0378	0.13601	-0.01308	0.07381	0.03851	-0.1442	-0.10835	-0.14677	0.23221	0.05035	0.00889	0.10481

G_CANCE	RADIOL_	RASTIC_TR	CANCER_	CANCER_	E_TO_UV	E_TO_UV	A_BOUN	NUP98_H	NUP98_H	NUP98_H	NUP98_H	NUP98_H	NUP98_H	NUP98_H	NUP98_H
0.20833	0.21031	0.21964	-0.14812	-0.05633	0.16538	0.28475	0.089	0.03749	0.04365	0.0378	0.06809	0.21039	0.04038	-0.03745	-0.04368
0.17845	0.20707	0.30567	-0.18175	-0.12593	0.16771	0.27647	0.01714	0.01696	0.0499	-0.01328	0.07781	0.16655	0.02813	0.02752	-0.21311
0.19339	0.17983	0.24334	-0.10137	-0.19553	0.16499	0.26168	0.07368	0.04058	0.10275	0.00775	0.09623	0.17356	0.0905	0.01064	-0.09069
0.21655	0.21763	0.16436	-0.25222	-0.06366	0.15531	0.27143	0.01891	0.09148	0.10224	0.01769	0.1159	0.25821	0.09672	0.00844	-0.08145
0.20191	0.19842	0.23571	-0.22198	-0.0509	0.1344	0.27406	0.04885	0.04894	0.06844	0.07705	0.08063	0.20816	0.05579	-0.03026	-0.08411
0.20182	0.23595	0.19605	-0.28136	-0.32461	0.1531	0.27037	0.04386	0.05473	0.0986	-0.00926	0.10807	0.20755	0.05622	-0.04017	-0.08379
0.198	0.17752	0.21194	-0.15896	-0.40618	0.14348	0.26907	-0.0014	0.08765	0.13454	0.03481	0.17119	0.24186	0.11262	-0.00342	-0.08861
0.20557	0.19377	0.2271	-0.22662	-0.42495	0.14188	0.27869	0.0748	0.12342	0.09493	0.02991	0.11367	0.29541	0.08282	-0.01782	-0.1294
0.20564	0.1959	0.25767	-0.23959	-0.42451	0.14362	0.26723	0.11006	0.10454	0.14422	0.00142	0.1674	0.28005	0.12012	0.01424	-0.13045
0.20644	0.21409	0.15725	-0.24314	-0.40786	0.13503	0.27125	0.07449	0.10636	0.14826	0.04747	0.15951	0.28331	0.14473	-0.02054	-0.08599
0.19367	0.14498	0.25149	-0.06383	-0.26134	0.14719	0.27133	-0.02654	0.06496	0.04508	0.10585	0.04335	0.04205	0.00783	-0.05748	-0.07798
0.1981	0.18495	0.22365	-0.07232	-0.44738	0.14539	0.27798	-0.04673	0.01884	0.1121	0.0035	0.12545	0.12331	0.10061	-0.01974	-0.00125
0.17791	0.20426	0.23444	-0.15181	-0.01824	0.15759	0.2689	-0.04976	-0.0263	0.04671	-0.06212	0.03235	0.03961	0.04558	-0.07504	-0.05848
0.20913	0.18191	0.20666	-0.20516	0.02153	0.15334	0.27019	-0.06032	0.07862	0.05088	0.03877	0.06259	0.20532	0.04138	0.00686	-0.09309
0.20731	0.20371	0.23228	-0.22181	-0.37512	0.15038	0.27294	0.10781	0.08801	0.09199	0.12477	0.11058	0.19377	0.07078	-0.026	-0.14122
0.17572	0.14034	0.29571	-0.1388	0.15825	0.14764	0.25805	-0.12716	-0.04769	0.05796	-0.0419	0.05236	0.03193	0.03589	-0.11614	-0.07374
0.20831	0.19582	0.20821	-0.11249	0.00951	0.14445	0.27703	0.0197	0.05354	0.03817	0.03445	0.04368	0.21971	0.02761	-0.02185	-0.12342
0.19628	0.18287	0.17191	-0.2333	-0.06231	0.1417	0.27083	0.03017	0.04652	0.11382	-0.01088	0.13937	0.25406	0.12226	-0.0183	-0.08802

NUP98_H	NUP98_H	IN_ESO	NCE_TP	ENCE_TP	ELL_VS_B	LL_VS_B	ELL_VS_F	ELL_VS_	DIE_META	EBPA_TA	EBPA_TA	CUTE_LY	TOMA_CO	TOMA_CO	BLASTOM	AKARYO
0.0552	0.0779	0.02089	-0.01627	0.1267	-0.00711	0.18671	0.21694	0.15625	-0.04961	0.1697	0.092	0.11197	0.09398	0.0573	-0.06347	-0.04287
0.01378	0.07544	0.05235	0.01476	0.073	-0.05835	0.17163	0.21909	0.15043	-0.14472	0.18385	0.02847	0.11859	0.08703	0.02274	-0.19561	-0.0326
0.07258	0.11398	0.04048	-0.02021	0.08052	0.0079	0.19294	0.23216	0.167	-0.04617	0.16838	0.08928	0.11529	0.14265	0.01128	-0.0614	-0.04348
0.14513	0.14266	0.00615	-0.06874	0.13641	0.0287	0.17619	0.21604	0.19162	0.00716	0.16973	0.10363	0.10303	0.06122	0.0183	-0.14143	-0.07356
0.11607	0.09391	0.01544	-0.10986	0.17641	0.07337	0.17245	0.21894	0.18174	-0.06869	0.17619	0.14266	0.09091	0.08357	0.01619	-0.09382	-0.04227
0.05128	0.10592	0.02677	-0.07191	0.18879	-0.00203	0.18969	0.22514	0.17251	-0.08045	0.16353	0.07246	0.15138	0.02514	0.02824	-0.12827	-0.04797
0.11081	0.12351	0.02481	-0.0234	0.08878	0.00794	0.17983	0.2303	0.18333	-0.0307	0.19066	0.08479	0.11887	0.04368	0.05072	-0.07115	-0.01196
0.17975	0.12503	0.00887	-0.12356	0.17603	0.04551	0.20724	0.20221	0.1981	0.02214	0.19789	0.12151	0.11297	0.10009	0.03843	-0.08898	-0.05378
0.16917	0.14649	-0.00971	-0.12599	0.19393	0.08443	0.20881	0.21088	0.20134	0.00029	0.21997	0.13028	0.12101	0.10028	0.04146	-0.08064	-0.00763
0.16	0.1529	0.03403	-0.08172	0.14914	0.11474	0.18745	0.2152	0.19799	-0.03041	0.17522	0.129	0.12917	0.08109	0.02632	-0.14121	-0.01627
0.04539	0.07379	0.01742	0.01511	0.03708	-0.08174	0.17762	0.23228	0.15063	-0.09416	0.13751	0.04944	0.1297	0.10003	0.06086	-0.00956	-0.04006
0.0209	0.14555	0.04346	-0.04999	0.07399	-0.01801	0.18291	0.23181	0.17461	-0.03391	0.1492	0.03714	0.11621	0.10881	-0.00369	-0.05173	-0.0641
-0.07459	0.08833	0.04236	-0.02907	0.1319	-0.06157	0.18597	0.21051	0.12048	-0.0709	0.15243	0.04795	0.10832	0.03734	0.03583	-0.07329	0.00439
0.11703	0.0929	0.01121	-0.03735	0.07913	0.02733	0.19182	0.20822	0.1813	-0.05308	0.16661	0.07945	0.08065	0.07355	0.03167	-0.1843	-0.07818
0.12245	0.12643	0.03447	-0.01898	0.04719	-0.02626	0.16537	0.22574	0.1814	-0.05617	0.17592	0.07314	0.11672	0.09631	0.03295	-0.17197	-0.05623
-0.05952	0.0931	0.03528	0.04136	-0.0061	-0.10096	0.16162	0.22254	0.11873	-0.0792	0.06404	0.02086	0.11828	0.06131	0.06993	-0.02042	-0.0571
0.10025	0.06521	0.01891	-0.05812	0.08901	-0.01375	0.17576	0.20907	0.18184	-0.02778	0.13261	0.11902	0.09954	0.10484	0.02265	-0.06807	-0.05233
0.11651	0.15404	0.02937	-0.07159	0.19509	0.05268	0.18575	0.21451	0.18297	-0.02429	0.22834	0.11072	0.09935	0.06227	0.02213	-0.10198	-0.07503

CN_TARGE	N_TARGE	N_TARGE	N_TARGE	N_TARGE	N_TARGE	N_TARGE	X4_TARGE	X4_TARGE	4_TARGE	4_TARGE	X_TARGET	ARGETS_I	ARGETS_I	GETS_MO	OPHIL_A	OPHIL_
0.13389	-0.40989	0.28037	-0.0958	0.02093	0.17743	0.15778	-0.11093	0.07752	0.29361	0.09938	-0.12383	-0.03269	-0.03269	-0.18267	0.15504	0.1463
0.14619	-0.32952	0.27952	-0.01671	0.02233	0.17861	0.11411	-0.24123	0.12983	0.35807	0.09546	-0.02713	0.08919	0.08919	-0.13229	0.12419	0.13483
0.17732	-0.32353	0.29435	-0.03584	0.00089	0.23663	-0.04794	-0.35963	0.09765	0.20843	0.08575	-0.08085	-0.02272	-0.02272	-0.03276	0.15585	0.15127
0.15617	-0.42174	0.28184	0.04797	0.06282	0.17154	0.09619	-0.2607	0.13219	0.274	0.11561	-0.22019	-0.13999	-0.13999	0.18813	0.16905	0.17086
0.10229	-0.42338	0.31694	-0.09569	0.01363	0.12821	-0.19994	-0.32007	0.09644	0.36101	0.05912	-0.15554	-0.05289	-0.05289	0.09389	0.17847	0.167
0.11025	-0.21894	0.27087	-0.05502	0.03235	0.26888	0.0521	-0.19717	0.11376	0.33193	0.09172	0.0236	-0.07797	-0.07797	-0.23287	0.1437	0.13802
0.15727	-0.25551	0.2837	-0.02143	0.09801	0.25423	-0.01092	-0.3223	0.04778	0.24004	0.08766	0.04703	-0.0448	-0.0448	-0.33592	0.14032	0.14128
0.14041	-0.48581	0.3062	0.0867	0.04143	0.20318	0.02669	-0.27962	0.15268	0.27106	0.07324	-0.10639	-0.21994	-0.21994	0.25189	0.17244	0.17745
0.11431	-0.36931	0.29281	0.02634	0.067	0.18024	0.00667	-0.26591	0.14834	0.41529	0.05713	-0.13264	-0.22227	-0.22227	0.17069	0.17263	0.20257
0.07837	-0.37586	0.28188	0.01681	0.05232	0.19811	0.02481	-0.26219	0.12892	0.3786	0.06794	0.0377	0.14845	0.14845	0.15484	0.17607	0.18655
0.19512	-0.16227	0.32616	-0.17829	-0.00096	0.22082	-0.08767	-0.35624	0.07127	0.24956	0.11851	-0.04756	0.01028	0.01028	-0.20257	0.12771	0.11084
0.17745	-0.34012	0.27391	-0.015	0.01694	0.19358	-0.24772	-0.20593	0.0707	0.15564	0.10616	-0.14726	0.05992	0.05992	-0.04439	0.14693	0.13354
0.15947	-0.27188	0.30059	-0.1571	0.03524	0.16862	-0.29424	-0.31294	0.03616	0.12411	0.14832	0.21349	-0.01288	-0.01288	-0.04738	0.11654	0.12239
0.15827	-0.37568	0.28831	-0.06067	0.01546	0.16026	-0.13926	-0.20161	0.10098	0.38965	0.09488	0.27515	0.04366	0.04366	-0.01321	0.16177	0.16118
0.15522	-0.33639	0.28352	-0.09403	-0.02212	0.21781	-0.17331	-0.3211	0.07882	0.24648	0.10055	0.01603	0.06323	0.06323	-0.1987	0.1441	0.13363
0.20646	-0.33677	0.33489	-0.13447	-0.03011	0.23127	-0.21507	-0.20792	0.02356	0.33627	0.11501	-0.23111	-0.17487	-0.17487	-0.35265	0.09705	0.11101
0.15558	-0.39625	0.31031	0.00443	0.01524	0.15584	-0.0626	-0.23945	0.09462	0.29025	0.10585	0.17685	-0.00956	-0.00956	0.0886	0.15825	0.15403
0.06227	-0.48781	0.29261	-0.0866	0.03229	0.14984	-0.09744	-0.13501	0.13518	0.26785	0.08104	0.14691	0.15108	0.15108	0.1108	0.16685	0.18591

AMMARY_	N_ZNF21	N_ZNF21	GETS_HE	GETS_HE	C_HEART	C_HEART	LHAI5_T	ALING_N	IGNALIN
-0.07536	-0.16885	0.08187	-0.22762	-0.23187	0.0565	0.14223	0.09053	0.22823	-0.04952
0.00683	-0.0736	0.09483	-0.20275	-0.29558	0.04838	0.12157	0.10558	0.28601	-0.01978
0.07567	-0.08412	0.10235	-0.14561	-0.10431	0.04838	0.14885	0.10203	0.23958	-0.08996
-0.01142	-0.12115	0.12383	-0.0808	0.01062	0.02896	0.17738	0.16124	0.21897	-0.12146
-0.05726	-0.04696	0.10208	-0.1428	-0.17799	0.0241	0.15539	0.14069	0.05792	-0.12622
0.0514	-0.04042	0.10129	-0.03295	-0.03767	0.00795	0.14456	0.0384	0.04934	-0.065
0.0372	-0.04726	0.10592	-0.07115	0.04404	0.04965	0.14815	0.12712	0.20242	-0.03073
0.05359	0.01326	0.09679	-0.09544	0.0493	0.02561	0.18596	0.16386	0.07342	-0.1669
0.08138	-0.01906	0.11164	-0.07021	0.03729	0.01496	0.18349	0.14007	0.10442	-0.03581
-0.07862	-0.01791	0.09674	-0.07214	-0.10126	0.03868	0.18433	0.12994	0.03976	-0.02018
0.00593	-0.05346	0.07762	-0.18027	-0.22893	0.03068	0.12392	0.06938	0.03489	-0.05454
-0.0277	-0.04071	0.09872	-0.07253	0.02466	0.06758	0.13485	0.03359	0.09974	-0.1316
-0.12713	-0.13197	0.04916	-0.15114	-0.31972	0.03956	0.10685	-0.06271	0.10631	-0.05202
0.12981	-0.11042	0.12126	-0.19859	-0.04666	0.04744	0.15875	0.08492	0.27332	-0.09676
0.07453	-0.12869	0.09842	-0.11606	-0.10047	0.04174	0.14264	0.12688	0.18003	-0.10075
-0.04196	-0.12994	0.08616	-0.11774	-0.28429	0.05432	0.08625	0.03515	0.16555	-0.01389
0.03511	-0.1321	0.10465	-0.15469	-0.15227	0.04373	0.14743	0.15418	0.19596	-0.17223
-0.02767	-0.0035	0.09017	-0.06819	-0.06186	0.01881	0.1685	0.16975	0.14785	-0.10481

_PROBE	_PROBE	_PROBE	_PROBE	_PROBE	_PROBE	TH_STRE	ENCED_E	ANCER_R	_METAST	_METAST	_METAST	_METAST	OSTATE_	OSTATE_	ERACT_W
0.20704	0.13302	0.02751	0.32022	0.09272	0.33922	0.27726	-0.04296	0.30896	0.33349	0.29599	0.02996	0.07281	0.0731	0.24213	0.13912
0.20181	0.14454	0.04364	0.30311	0.09137	0.35061	0.2941	0.02445	0.37067	0.31723	0.30379	0.0042	0.0887	0.06878	0.24625	0.16384
0.20542	0.14071	0.03834	0.31444	0.09408	0.35297	0.26552	0.00206	0.26614	0.33357	0.30515	0.05784	0.06432	0.0628	0.2394	0.13052
0.19596	0.13029	0.01584	0.324	0.09534	0.36729	0.24198	0.02436	0.34789	0.34111	0.30653	0.02148	0.06151	0.06452	0.20885	0.13151
0.19646	0.13222	0.00703	0.32013	0.08437	0.35896	0.23771	0.06365	0.30519	0.29975	0.2946	0.00858	0.07602	0.0469	0.21359	0.12731
0.2129	0.13092	-0.0156	0.31581	0.06823	0.3634	0.25958	0.02307	0.33677	0.32304	0.30839	0.04712	0.01282	0.05985	0.21917	0.16321
0.20266	0.13951	-0.02895	0.32824	0.06405	0.36754	0.25346	-0.02789	0.2508	0.32352	0.29684	0.04427	0.00072	0.01973	0.24682	0.15113
0.20405	0.11402	0.03986	0.3414	0.09201	0.37313	0.23845	0.0937	0.36428	0.32239	0.29896	0.05691	0.01081	0.05695	0.22955	0.1151
0.18742	0.12024	0.02665	0.3256	0.08683	0.36266	0.24729	0.06795	0.30728	0.32477	0.29347	0.04297	0.07088	0.04816	0.22521	0.12239
0.19474	0.11528	0.02227	0.31589	0.07472	0.35208	0.26503	0.08196	0.33306	0.3277	0.28918	0.00606	0.00913	0.01086	0.20663	0.11713
0.2153	0.14957	0.00283	0.32441	0.08246	0.35737	0.24445	-0.02013	0.26415	0.32249	0.28654	0.05656	0.13776	0.08213	0.245	0.15763
0.22345	0.14026	1.6E-05	0.33339	0.07851	0.36099	0.25094	0.01778	0.25859	0.31736	0.29054	0.01461	0.0795	0.06733	0.23784	0.13603
0.20404	0.14178	-0.01759	0.33148	0.06352	0.35238	0.31132	-0.11219	0.30307	0.27811	0.31336	-0.01796	0.14656	0.13019	0.24865	0.16391
0.20722	0.12209	0.02678	0.32961	0.09454	0.36996	0.255	0.03443	0.34877	0.31598	0.28519	0.01239	0.04736	0.0473	0.2417	0.14698
0.22106	0.14579	0.04302	0.32927	0.09692	0.36965	0.25395	0.0248	0.35729	0.34747	0.29837	0.06719	0.09226	0.06255	0.26229	0.13335
0.20853	0.1545	-0.00478	0.33871	0.07002	0.36741	0.29486	-0.09108	0.18968	0.32379	0.27134	0.03967	0.06468	0.06657	0.25502	0.14586
0.19652	0.1258	0.0291	0.33727	0.09804	0.3671	0.23613	0.06566	0.34563	0.31922	0.3197	0.01672	0.02938	0.08481	0.22983	0.14583
0.18998	0.12901	0.0289	0.3311	0.09428	0.35826	0.25744	0.11693	0.31449	0.32882	0.28782	0.01932	0.08158	0.05502	0.218	0.11498

RUNX1T1	RUNX1T1	RUNX1T1	RUNX1T	UNX1_RU	UNX1_RU	1_RUNX1	1_RUNX1	1_FUSIO	T1_FUSIO	T1_FUSIO	1T1_FUS	1T1_FUS	ABINE_R	ABINE_R	OF_EWSR	EWSR1_F	EWSR1_F	OF_EWSR	_OF_MIR	RETT_SY	RETT_SY	AJB4_TA	AJB4_TA
0.06856	0.07915	0.13173	0.13549	0.07637	0.0732	0.09155	0.10912	0.10304	0.11151	0.25581	0.30011	0.07289	0.1895	0.17578	0.06327	0.16837	0.00585	0.03307	0.10406	0.11064	0.00599	0.1193	0.16051
-0.00813	0.05103	0.06473	0.18574	0.0405	0.0564	-0.01403	0.12466	0.09334	0.02745	0.24432	0.19664	0.08381	0.1876	0.18072	0.04391	0.23233	-0.03912	-0.00095	0.13074	0.07118	-0.015	0.07304	0.19236
0.0117	0.06849	0.08195	0.18935	0.06814	0.06707	0.03985	0.11977	0.05668	0.0265	0.3087	0.17936	0.00515	0.18452	0.19092	0.05942	0.23418	0.05918	0.03828	0.12491	0.2049	0.06245	0.12293	0.26054
-0.01926	0.12665	0.15932	0.10667	0.11834	0.08073	0.08632	0.10973	0.1063	0.0783	0.24841	0.17741	-0.01365	0.18288	0.17796	0.08269	0.18681	0.06481	0.03641	0.10458	0.01402	0.1237	0.1547	0.24431
0.10008	0.07958	0.1229	0.18658	0.12485	0.07966	0.08659	0.09571	0.07688	0.0734	0.29582	0.22138	0.07225	0.1666	0.17675	0.0899	0.15069	-0.03423	0.0292	0.11098	-0.01596	0.11479	0.05586	0.19158
0.09356	0.05331	0.11333	0.14612	0.07295	0.06383	0.02602	0.11077	0.09713	0.00362	0.28463	0.19292	0.01787	0.17004	0.16501	0.06918	0.21319	-0.03293	0.00119	0.1233	0.07865	0.15869	0.14773	0.15635
0.03271	0.07199	0.12974	0.1089	0.09474	0.0592	0.04198	0.11411	0.07539	0.06797	0.19677	0.22555	-0.04057	0.17305	0.2064	0.06814	0.25401	-0.01098	0.01292	0.12435	0.10453	0.17398	0.09197	0.32636
0.06767	0.13969	0.16034	0.15895	0.14043	0.1109	0.0776	0.09906	0.11143	0.08636	0.24922	0.21268	0.06938	0.18912	0.17253	0.09632	0.22004	0.11173	0.05147	0.08212	-0.0524	0.09641	0.14919	0.24705
0.04571	0.13846	0.17827	0.14454	0.13763	0.08789	0.08145	0.10662	0.12384	0.1028	0.29421	0.18896	0.03237	0.17067	0.16421	0.09688	0.23053	0.0814	0.03279	0.08623	-0.03804	0.07447	0.11303	0.28656
0.03436	0.11031	0.16714	0.17227	0.143	0.08943	0.11731	0.10392	0.12607	0.11904	0.30136	0.28411	0.08296	0.15916	0.16776	0.09401	0.19858	-0.00844	0.03158	0.08645	0.01581	0.10015	0.06397	0.34329
0.0535	0.0161	0.00682	0.15463	0.00164	0.04758	0.0517	0.12276	0.03532	-0.00884	0.19392	0.23326	0.09969	0.18755	0.17575	0.04532	0.21352	0.0108	0.02169	0.13128	0.09765	-0.00816	0.13266	0.16016
0.00649	0.06016	-0.01096	0.09492	0.05941	0.07678	0.05879	0.13076	0.1122	-0.00743	0.23903	0.18872	0.08564	0.19445	0.18485	0.06347	0.20122	0.10442	0.06087	0.11973	0.0564	0.1028	0.05154	0.27837
0.04457	0.00957	-0.12817	0.1629	0.00322	0.04164	0.02626	0.10735	0.07209	-0.22919	0.21514	0.20982	0.04448	0.19771	0.1654	0.00187	0.18777	-0.12064	-0.00974	0.12474	0.18699	0.1155	0.05393	0.21637
-0.01768	0.10775	0.10633	0.16037	0.07064	0.08873	0.04872	0.11252	0.13593	0.03467	0.276	0.1907	0.1267	0.18672	0.19165	0.07856	0.19301	0.08672	0.03741	0.10496	0.05311	0.06227	0.20248	0.18372
0.05343	0.05909	0.10585	0.15973	0.06776	0.08657	0.07507	0.11623	0.10405	-0.00178	0.28465	0.22422	0.08266	0.1912	0.179	0.05975	0.21215	0.05082	0.04121	0.12267	0.02355	0.0723	0.17249	0.23327
-0.04172	-0.01374	-0.0832	0.14294	-0.03994	0.01816	0.01393	0.13018	0.0019	-0.26654	0.21047	0.22182	-0.00399	0.15758	0.18874	0.01389	0.22079	-0.0281	0.01817	0.13536	0.18986	0.06295	0.03651	0.20434
0.04013	0.09974	0.10635	0.1362	0.08456	0.09113	0.03453	0.10375	0.10323	0.0049	0.27567	0.17916	-0.00586	0.16909	0.18368	0.08679	0.15396	0.11797	0.02995	0.10848	0.06606	0.03342	0.14391	0.11953
0.03116	0.10512	0.14627	0.16641	0.11897	0.07395	0.10237	0.10354	0.09234	0.0919	0.31897	0.27074	0.04003	0.16847	0.18803	0.08495	0.19935	-0.03206	0.02818	0.10077	0.02038	0.12271	0.06209	0.2988

_TO_ION	_TO_RAD	GENIC_P	GENIC_P	RS1_TAR	RS1_TAR	R_SOFT	LATIN_R	LATIN_R	_FBXW	UPPRESS	UPPRESS
0.07506	0.0829	-0.01263	0.11518	0.07709	0.19009	0.18114	0.09941	0.07622	-0.2991	-0.00049	0.14128
0.05273	0.11064	-0.02107	0.19289	0.072	0.21489	0.18109	0.10344	0.03225	-0.09054	-0.03884	0.12473
0.05512	0.10393	-0.01374	0.16592	0.07423	0.22442	0.17669	0.16625	0.12357	-0.13106	0.01014	0.1659
0.07236	0.14292	-0.06008	0.06576	0.06906	0.18468	0.19784	0.15373	0.1413	-0.27162	0.1978	0.15585
0.06669	0.05869	-0.04454	0.1163	0.06374	0.19117	0.19642	0.13459	0.14444	-0.18918	0.05243	0.18427
0.07944	0.17577	0.04496	0.2103	0.0799	0.22557	0.10314	0.14077	0.2595	-0.19782	0.14828	0.16811
0.08806	0.24723	0.08727	0.1592	0.09906	0.22581	0.15052	0.18631	0.24971	-0.18478	0.18576	0.162
0.07	0.13502	-0.03698	0.04993	0.08995	0.17962	0.23036	0.16843	0.16552	-0.31636	0.18705	0.17075
0.09322	0.15888	-0.00361	0.04226	0.09831	0.18662	0.20474	0.16214	0.18366	-0.2681	0.15686	0.17411
0.09029	0.17129	-0.04336	0.09584	0.08834	0.19125	0.21121	0.15256	0.18367	-0.22494	0.22227	0.1457
0.06184	0.08437	-0.0334	0.11865	0.07491	0.20723	0.04626	0.13265	0.20782	-0.3072	0.05057	0.24015
0.08259	0.12426	0.02514	0.09797	0.08625	0.20323	0.13784	0.16458	0.2917	-0.25172	0.15523	0.1302
0.04029	0.13969	-0.02589	0.1974	0.04668	0.22146	0.20586	0.10626	0.06363	-0.2038	0.09303	0.10316
0.06294	0.05345	-0.06693	0.12452	0.06985	0.19673	0.20819	0.18007	0.13189	-0.30773	0.00166	0.14654
0.07456	0.13626	-0.01743	0.14636	0.09352	0.20856	0.19675	0.16862	0.14831	-0.32729	0.10944	0.23229
0.04778	0.09842	0.03466	0.17687	0.08513	0.21543	0.11766	0.11724	0.00075	-0.32288	-0.02859	0.13521
0.05079	0.10516	-0.03685	0.0937	0.06718	0.18441	0.20368	0.11788	0.08394	-0.2533	0.11429	0.17562
0.0865	0.11468	-0.039	0.10499	0.07847	0.18244	0.20869	0.14689	0.12978	-0.25119	0.09632	0.19638

_CARCINO	CARCINO	R_CARCIN	R_CARCIN	_CARCIN	_CARCINO	RMAL_DU	I_MAPK1	I_MAPK14	K1_AND_K	I_MAPK7	K8_AND_R	_MED1_T	R_MED1_T	ENTRAL_	RIFERAL	ICING_VI
0.05521	0.02386	-0.02597	0.01255	-0.0042	0.06383	-0.05145	-0.3366	-0.36278	-0.24316	-0.42452	-0.2138	0.04373	0.14558	0.18716	0.15477	0.03056
0.0328	0.07101	-0.02957	0.0182	-0.01859	0.01691	-0.02267	-0.32878	-0.35373	-0.03798	-0.41148	-0.22636	0.02132	0.13015	0.18412	0.13733	0.03134
0.04938	0.05445	-0.035	0.02941	0.00295	0.02065	0.00973	-0.38135	-0.40288	-0.20926	-0.45658	-0.27211	0.03426	0.15069	0.18374	0.13567	0.05214
0.03054	0.06176	-0.02296	0.04376	0.03673	0.06622	-0.08617	-0.391	-0.4083	-0.20186	-0.44989	-0.3476	0.03392	0.12809	0.17055	0.1425	0.00494
-0.11163	0.03877	-0.12014	0.00133	-0.01197	-0.02681	-0.06831	-0.42653	-0.42631	-0.2259	-0.42276	-0.34703	0.02225	0.14156	0.15646	0.13706	0.02323
0.03728	0.06653	-0.01564	0.03574	0.02369	-0.00251	-0.02534	-0.32294	-0.32188	-0.24625	-0.31707	-0.26208	0.01218	0.13142	0.18247	0.14207	0.03324
0.02838	0.0648	-0.05852	0.11039	0.0828	0.00272	-0.06002	-0.40513	-0.39315	-0.14934	-0.40144	-0.30045	0.01523	0.13411	0.17203	0.12827	0.02351
-0.02627	0.05782	-0.08032	0.08043	0.04607	0.02008	-0.09089	-0.34058	-0.36441	-0.21791	-0.42117	-0.31205	0.03702	0.12305	0.16611	0.13375	0.02815
0.02774	0.06538	-0.03521	0.08359	0.05923	0.04122	-0.09111	-0.4096	-0.41334	-0.22858	-0.39528	-0.37575	0.01978	0.11549	0.16887	0.12716	0.03164
-0.01294	0.06211	-0.05301	0.06981	0.02131	0.01971	-0.09688	-0.40689	-0.40628	-0.29823	-0.40268	-0.32206	0.01972	0.12311	0.16672	0.12375	0.00442
0.11102	0.02478	0.01773	-0.00637	-0.0608	0.05494	-0.02629	-0.43521	-0.43979	-0.04592	-0.46468	-0.34823	0.02981	0.10882	0.18985	0.15021	0.00658
0.08613	0.06522	0.0049	0.05837	0.00052	0.05419	-0.06842	-0.45534	-0.45492	-0.20795	-0.4527	-0.31989	0.03203	0.14443	0.204	0.14546	0.01468
0.04244	0.02198	-0.02073	-0.0873	-0.07485	0.04992	-0.02696	-0.29431	-0.25674	-0.0322	-0.44143	-0.12404	0.04267	0.1417	0.16857	0.14142	0.06784
0.02407	0.05249	-0.06152	0.04138	0.02227	-0.00731	-0.03842	-0.36936	-0.37954	-0.17022	-0.36833	-0.32093	0.02084	0.12796	0.18529	0.13301	0.00664
0.0735	0.04808	-0.03031	0.04889	-0.00771	0.04799	-0.01852	-0.24544	-0.28433	-0.20059	-0.44654	-0.20454	0.03336	0.13372	0.18627	0.14206	0.02502
0.10604	0.01531	-0.02088	-0.05312	-0.08284	0.04888	-0.00113	-0.36921	-0.32729	-0.0544	-0.45728	-0.20524	0.02554	0.14682	0.20794	0.13591	0.05267
0.04714	0.04115	-0.03419	0.02211	0.00314	0.04811	-0.03741	-0.30479	-0.34508	-0.19744	-0.45658	-0.27729	0.03971	0.12902	0.19202	0.15298	0.02789
0.03397	0.04644	-0.03504	0.04364	0.0091	0.02936	-0.047	-0.38754	-0.40119	-0.23766	-0.43157	-0.32597	0.02848	0.1316	0.15848	0.1236	0.01513

SPONSE_	E_DIFFER	E_DIFFER	LEUKO	ML_CLUS	ML_CLUS	ML_CLUS	ML_CLUS	ML_CLUS	ML_CLUS	ML_CLUS	ML_CLU	ML_CLU	ML_CLU	ML_CLU
0.08412	-0.00338	0.05619	-0.13676	-0.2257	0.1555	0.07163	0.12531	0.0746	0.15155	0.03563	0.26032	0.0205	0.17962	0.07189
0.12711	0.01847	0.01512	-0.14984	-0.07866	0.00564	0.0796	0.12595	0.03142	0.13925	0.0393	0.13293	-0.00201	0.15322	0.0585
0.17849	0.05936	0.05682	-0.22701	-0.23682	0.06178	0.13172	0.14347	-0.02191	0.17345	-0.02399	0.227	-0.01994	0.17943	0.05808
0.16385	0.02667	0.08603	-0.25971	-0.13386	0.03977	0.11338	0.1576	0.02557	0.21373	0.06004	0.19384	0.0149	0.19363	0.15935
0.17573	0.02612	0.04434	-0.26781	-0.08396	0.05934	0.13291	0.1463	0.05033	0.18181	0.04067	0.21746	0.00736	0.17335	0.08739
0.15671	0.02207	0.06643	-0.24702	-0.13321	0.04858	0.06027	0.0962	0.09483	0.10135	0.03474	0.24269	0.03525	0.19708	0.02529
0.27365	0.16214	0.06882	-0.2607	-0.15324	0.05688	0.1285	0.10393	0.11779	0.09638	-0.03321	0.22921	-0.01926	0.19891	0.07156
0.15301	0.07031	0.07585	-0.25491	-0.06828	-0.02056	0.15493	0.17031	0.01466	0.17329	0.05604	0.1507	-0.01266	0.14622	0.19585
0.25087	0.0829	0.08121	-0.16587	-0.08503	0.01173	0.14729	0.15237	0.12272	0.18353	0.0521	0.15681	0.01645	0.20174	0.18029
0.28638	0.00914	0.07567	-0.25896	-0.1331	0.01329	0.14771	0.13424	0.07818	0.15424	0.07861	0.21272	-0.00231	0.1419	0.17413
0.05099	0.01705	0.05029	-0.20666	-0.21601	0.08357	0.06066	0.10265	-0.01286	0.12566	0.02801	0.22359	0.00619	0.21005	-0.07288
0.229	0.03964	0.08297	-0.14343	-0.24433	0.03927	0.023	0.15271	0.09682	0.10943	-0.0136	0.24591	-0.01211	0.18237	0.03208
0.12174	0.04409	0.01005	-0.14412	-0.35213	0.06696	0.00866	0.10496	0.06912	0.082	0.02845	0.28416	0.05554	0.24611	-0.05608
0.10048	0.05871	0.06828	-0.17799	-0.13656	0.02174	0.12192	0.14764	-0.02943	0.16831	-0.02391	0.19	0.05963	0.20687	0.06759
0.14488	0.05995	0.05548	-0.20217	-0.1652	0.06488	0.09448	0.0965	0.00083	0.17096	0.05195	0.21387	0.02972	0.19618	0.03545
0.1462	-0.00391	0.00657	-0.12169	-0.12754	0.05004	-0.01135	0.06859	0.01952	0.10901	0.01507	0.22044	-0.07819	0.17531	-0.07246
0.08651	0.02675	0.06357	-0.17605	-0.2337	0.12358	0.14478	0.12894	0.01841	0.18513	0.0031	0.26269	-0.00349	0.16895	0.07542
0.23021	-0.02477	0.07388	-0.25424	-0.02919	-0.0116	0.15486	0.1267	-0.05499	0.20637	0.00547	0.23543	-0.00694	0.20252	0.13856

ML_CLU	ML_CLU	ML_CLU	ML_CLU	H_11Q23	ML_WITH	ML_WITH	L_WITH_F	T_8_21_	BCL2_TA	BCL2_TA	MD1_TAR	MD1_TAR	MD1_TAR	MD1_TAR
0.09746	0.07142	-0.00168	0.04061	-0.01603	0.18385	0.171	-0.08351	0.29752	0.07957	0.18926	0.19756	0.13908	0.25501	-0.00302
0.10575	0.02934	0.05858	0.02969	-0.02506	0.01147	-0.06187	-0.12135	0.22406	0.06586	0.07997	0.07338	0.13736	0.20048	0.13623
0.15164	0.07478	0.04036	0.01213	-0.0494	0.12609	0.00686	-0.19676	0.2105	0.07965	0.11686	0.10349	0.07818	0.22052	0.0541
0.19221	0.02834	0.00076	0.06347	0.09925	0.19342	0.06299	-0.07016	0.22406	0.0967	0.18144	0.077	0.1271	0.19836	0.061
0.16936	0.17922	0.06533	-0.0357	-0.07734	0.21436	0.08565	-0.20193	-0.43597	0.12499	0.18441	-0.03236	0.12529	0.19891	0.07298
0.13541	0.2087	0.05222	-0.12041	0.01834	0.03769	0.04337	-0.10197	-0.3412	0.07554	0.20742	0.07071	0.13505	0.20532	0.06862
0.15564	0.0054	-0.04822	0.12648	-0.08918	0.05752	-0.04864	-0.08694	0.2762	0.08757	0.17938	0.10631	0.15706	0.20482	0.13198
0.16493	0.14886	0.01636	0.06318	-0.00227	0.21036	0.02744	-0.13089	0.06518	0.12508	0.19549	-0.06039	0.05299	0.18174	-0.07083
0.18205	0.09816	-0.00099	0.06272	0.02805	0.14501	0.03013	-0.11496	0.23939	0.09944	0.1935	0.03386	0.12369	0.19186	0.03937
0.16274	0.05566	0.01075	0.04033	0.04887	0.19389	0.05025	-0.11398	0.3072	0.12626	0.20811	-0.03458	0.1093	0.17992	0.02248
0.16026	-0.02918	0.00324	0.04709	0.04621	0.20227	0.12984	-0.06369	0.14779	0.09096	0.1389	0.18871	0.18591	0.20421	0.15189
0.1138	0.06193	-0.08584	0.03686	-0.03217	0.10215	0.06128	-0.19266	0.2607	0.05091	0.19645	0.13866	0.07269	0.21692	0.13965
0.13805	0.09324	0.06416	-0.02531	0.06823	-0.02963	-0.04142	-0.01178	0.36234	0.00694	0.16842	0.18745	0.13135	0.25007	-0.04526
0.17168	0.06382	0.00307	0.04651	-0.02662	0.16931	0.03638	-0.11418	0.05461	0.11147	0.14066	0.1121	0.20285	0.21325	0.01979
0.16654	0.06955	-0.00159	0.00928	-0.04955	0.2315	0.13158	-0.07199	0.2253	0.10463	0.20642	0.09981	0.10526	0.21119	-0.0331
0.14168	0.0621	0.02174	-0.02078	0.01853	0.04287	-0.05741	-0.18993	0.26281	0.00721	0.08644	0.20687	0.10354	0.22416	-0.0271
0.15952	0.06954	0.0416	0.03031	-0.00091	0.16023	0.11512	-0.12212	0.17879	0.1	0.19385	0.11025	0.17033	0.22358	0.06477
0.1813	0.1328	0.06292	0.04514	0.02405	0.1288	-0.00604	-0.17789	-0.44425	0.0877	0.17031	-0.0018	0.16142	0.21701	0.03294

MD1_TAR	NORMAL_	NORMAL_	UTERINE	UTERINE	FIBROID	FIBROID	SP3_TAR	SP3_TAR	OGENESI	AST_CAN	AST_CAN	AST_CAN	AST_CAN	NT_CANCE
0.10207	0.1286	0.49516	0.05609	-0.10358	0.13131	0.06701	0.08643	-0.01414	0.11071	0.1512	0.08401	0.13599	0.0413	0.07859
0.08966	0.13608	0.49428	0.041	-0.0375	0.13226	0.0939	0.05049	-0.19763	0.10992	0.18642	0.09131	0.11211	0.01873	0.04019
0.13234	0.12294	0.49604	0.07086	-0.12308	0.13208	0.05521	0.09378	0.13198	0.1003	0.16187	0.12552	0.16013	0.03094	0.07036
0.20981	0.10314	0.49568	0.02227	-0.04148	0.1091	0.06746	0.15625	-0.07896	0.10217	0.12041	0.07233	0.15686	0.01401	0.0537
0.15878	0.14094	0.49392	0.00781	-0.05224	0.11031	0.0719	0.10668	-0.2126	0.09207	0.10887	0.04227	0.14185	0.02422	0.06705
0.20291	0.14107	0.49304	0.07756	-0.05275	0.10727	0.08442	0.03896	-0.38513	0.07749	0.1367	0.09444	0.13393	0.01527	0.06911
0.19828	0.10902	0.49339	0.08992	-0.01544	0.13055	0.07297	0.10201	-0.17544	0.07273	0.126	0.1122	0.13719	-0.00602	0.0701
0.25407	0.13492	0.49533	0.02171	-0.06267	0.12669	0.06043	0.16005	0.12453	0.08784	0.1152	0.02438	0.13876	0.03801	0.01726
0.25579	0.12748	0.49551	0.01916	-0.02682	0.11506	0.04884	0.15397	-0.01048	0.08792	0.10732	0.05039	0.14593	0.00862	0.03667
0.20934	0.12141	0.49533	0.01332	-0.06397	0.11397	0.07143	0.1368	-0.28864	0.08408	0.1092	0.05334	0.14195	0.00635	0.0247
0.14274	0.07588	0.49604	0.06115	-0.17368	0.10038	0.06277	0.03435	-0.03961	0.09534	0.14721	0.07325	0.14708	0.03669	0.0984
0.14507	0.09955	0.486	0.05623	-0.08836	0.1225	0.05887	0.06454	0.14475	0.09778	0.16215	0.0807	0.12477	0.03201	0.10607
0.14962	0.12157	0.49533	0.08053	-0.18664	0.07337	0.0941	0.05126	-0.27828	0.10063	0.13961	0.09174	0.10606	0.05294	0.07093
0.17102	0.11341	0.49392	0.02897	-0.08903	0.12367	0.07658	0.15373	-0.10044	0.10217	0.17997	0.05988	0.13073	0.04301	0.05447
0.14943	0.12529	0.49339	0.04636	-0.12285	0.12195	0.08746	0.08797	0.01002	0.10387	0.13438	0.06616	0.1554	0.00141	0.07018
0.07213	0.09818	0.49304	0.06906	-0.11937	0.13467	0.04956	0.04605	0.21294	0.12284	0.16928	0.13051	0.12618	0.02708	0.11907
0.18378	0.16805	0.49604	0.06339	-0.07143	0.12913	0.06467	0.09259	0.09191	0.0987	0.17131	0.04461	0.12506	0.0467	0.03763
0.21238	0.13486	0.48952	0.04993	-0.08274	0.13446	0.02881	0.1424	-0.23907	0.08343	0.13281	0.05875	0.14893	0.02855	0.03425

T_CANCE	CANCER	PSTE24_T	PSTE24_T	N_ANGIO	N_ANGIO	CER_ADV	CER_ADV	IC_CANC	IC_CANC	WITH_NP	WITH_NP	OBLASTO	BLASTOMA	OBLAST
0.18769	0.1045	0.13	0.08821	-0.00979	-0.0259	0.12826	0.04499	-0.05977	0.12364	0.08033	0.09092	-0.00225	0.10205	0.09083
0.16387	0.03406	0.13951	0.08019	-0.04586	-0.00023	0.08414	0.01276	-0.12072	0.13873	0.01351	0.06707	0.01325	0.0538	0.07015
0.18693	0.05648	0.16526	0.06165	-0.02606	0.01485	0.09555	0.02944	-0.0733	0.13672	0.05811	0.10894	0.02268	0.11506	0.07371
0.16326	0.07364	0.13991	0.07858	0.00187	0.00618	0.07982	0.09942	-0.03336	0.103	0.07271	0.16718	-0.04413	0.17934	0.09091
0.18776	0.1039	0.17206	0.05936	-0.03214	-0.01254	0.10893	0.02454	-0.05183	0.09975	0.08191	0.12057	-0.04811	0.14048	0.11226
0.14959	0.02887	0.23031	0.08104	0.03452	-0.01296	0.11227	0.02419	-0.02384	0.1278	0.05601	0.08344	-0.04718	0.07354	0.07357
0.12526	0.03797	0.13045	0.07171	0.07044	0.0435	0.08506	0.05923	-0.03646	0.12881	0.09048	0.12517	-0.02972	0.10712	0.07522
0.17678	0.02589	0.17139	0.10118	-1.5E-05	0.03843	0.07743	0.1146	0.02131	0.06764	0.06463	0.17681	-0.0541	0.20527	0.11453
0.18923	0.07739	0.15879	0.05426	0.00679	0.06845	0.05516	0.09736	-0.00132	0.06864	0.08483	0.18941	-0.03834	0.18972	0.10038
0.16509	0.02006	0.166	0.07298	0.01844	0.01547	0.07361	0.07894	-0.00135	0.08274	0.08347	0.17602	-0.0558	0.16707	0.09695
0.19852	0.10916	0.1339	0.0904	0.00224	-0.02504	0.1135	-0.03038	-0.0501	0.14561	0.08874	0.02314	0.00362	0.05469	0.08237
0.16013	0.11617	0.15546	0.096	-0.03133	-0.0215	0.10986	0.01411	-0.01056	0.13076	0.07992	0.06575	-0.0073	0.08442	0.10126
0.18088	0.08133	0.11358	0.0507	-0.02027	0.03021	0.1131	-0.07248	-0.07981	0.14791	0.03314	-0.0257	0.02091	-0.0104	0.0754
0.17051	0.04737	0.16496	0.0851	-0.00663	-0.02389	0.08969	0.05353	-0.0385	0.12097	0.06838	0.11634	-0.02947	0.14352	0.08503
0.13851	0.04755	0.13348	0.07492	-0.03866	-0.0044	0.10167	0.03962	-0.04242	0.13024	0.08519	0.099	-0.01721	0.11667	0.08683
0.16154	0.09782	0.10777	0.0918	-0.05169	-0.04879	0.11577	-0.07705	-0.13096	0.17757	0.02	-0.01715	0.0425	-0.00047	0.08339
0.1613	0.03029	0.13448	0.10606	0.00079	-0.00328	0.10604	0.05306	-0.0632	0.11194	0.06883	0.1229	0.02352	0.12587	0.08561
0.18996	0.03664	0.16841	0.0796	-0.03418	-0.01208	0.07474	0.06553	-0.0298	0.08983	0.05151	0.14343	-0.04537	0.16374	0.11196

BLASTOM	OBLASTO	OBLASTO	ET_ADIP	AYED_RE	RLY_RES	RESPONS	ESPONSE	ESPONSE	ESPONSE	ESPONSE	ESPONSE_OF_PRK	OF_PRK	OF_PRK	METASTA
0.01098	-0.04205	-0.01606	0.0284	0.05552	0.13229	0.05356	0.17637	0.19135	-0.00642	0.04949	0.42487	0.26334	0.12576	-0.18462
0.06663	-0.12123	-0.01171	-0.15293	0.05458	0.10799	0.04222	0.15926	0.12535	0.0462	0.0149	0.39898	0.22169	0.14133	-0.18691
0.02911	-0.02023	0.0498	0.04065	0.03611	0.11081	0.03016	0.18467	0.11601	-0.00736	0.01512	0.42646	0.28177	0.15845	-0.2632
-0.01231	-0.02592	-0.00108	0.11277	0.05963	0.15482	0.06489	0.23445	0.16821	0.02064	0.05669	0.30615	0.28706	0.12047	-0.19294
0.03388	-0.00214	0.00616	0.0863	0.01192	0.10841	0.02911	0.17168	0.12573	-0.0343	0.00126	0.32781	0.30613	0.11595	-0.24143
0.04119	-0.10686	0.03012	0.11004	0.04754	0.11338	0.03455	0.16493	0.15475	0.08686	-0.02916	0.39317	0.2005	0.15426	-0.18288
0.011	-0.02612	-0.00252	0.12177	0.12318	0.16924	0.09889	0.23161	0.17878	0.18922	0.05323	0.28871	0.26309	0.13567	-0.29286
-0.00261	0.02224	-0.04995	0.14933	0.03273	0.14928	0.06064	0.22149	0.17125	0.06444	-0.00672	0.15166	0.27181	0.16399	-0.24174
0.04002	-0.06093	-0.03782	0.11369	0.06376	0.15121	0.05674	0.23234	0.16438	0.04036	0.05288	0.27902	0.28639	0.10345	-0.22458
-0.00105	-0.0274	-0.01521	0.19278	0.02548	0.12195	0.03351	0.19022	0.15381	0.02926	0.00348	0.17509	0.24154	0.1257	-0.2371
0.03754	0.02363	0.04876	0.13244	0.06904	0.08438	-0.02821	0.15729	0.14112	0.02534	0.05213	0.42751	0.27073	0.1622	-0.33508
0.02938	-0.00622	0.06168	0.10161	0.05362	0.12004	0.05998	0.1659	0.14194	0.03598	0.01839	0.41043	0.28002	0.1135	-0.13981
0.04413	0.00656	0.01147	-0.04452	0.09266	0.08377	0.01697	0.09819	0.17156	0.04956	0.08268	0.42029	0.22117	0.11	-0.26557
0.02137	0.00254	-0.05241	0.09817	0.04993	0.12693	0.00333	0.23099	0.14387	-0.01613	0.04553	0.41219	0.30885	0.12182	-0.27218
0.02437	-0.06278	0.04693	0.1593	0.05284	0.11141	0.03695	0.19335	0.09254	0.01726	0.03658	0.36428	0.22573	0.13456	-0.30248
0.0168	-0.03848	0.01555	-0.15236	0.0691	0.06227	-0.00247	0.10734	0.09159	0.02682	0.05291	0.41589	0.23939	0.13184	-0.34347
0.03402	-0.06788	-0.08097	0.08232	0.0139	0.14022	0.06599	0.19613	0.17115	-0.00789	-0.00913	0.31267	0.3017	0.13969	-0.23984
-0.01282	-0.03133	-0.05345	0.03064	0.08023	0.12914	0.00113	0.22002	0.17004	0.05977	0.0481	0.4328	0.2111	0.11582	-0.23226

IFRD1_T	OTCH1_T	OTCH1_T	VER_CAN	VER_CAN	NG_LYMP	NG_LYMP	HEAT_S	HEAT_S	OXIA_TAR	SE_PROM	APO2_SE	EHMT2_T	ENESIS_	GENESI
0.25411	0.28323	-0.06632	0.08192	0.00063	0.07591	0.16667	0.28428	0.19457	0.19239	-0.13036	-0.21728	-0.37661	0.17796	0.07757
0.28797	0.11923	-0.15057	0.06318	0.01888	0.07568	0.16048	0.28692	0.21583	0.1399	-0.0666	-0.1246	-0.44848	0.1601	0.07441
0.21223	0.24885	-0.04775	0.08038	0.00821	0.0442	0.12344	0.27708	0.22826	0.07157	-0.11219	-0.0015	-0.47684	0.13844	0.08728
0.2517	0.31162	-0.11767	0.07512	-0.00865	0.08125	0.19115	0.27704	0.17755	0.15356	-0.10001	-0.1314	-0.22494	0.15146	0.08608
0.19052	0.35102	0.02099	0.07382	-0.01059	0.02347	0.18575	0.28623	0.19505	0.09038	-0.24115	-0.18971	-0.30456	0.14168	0.07632
0.20702	0.18493	0.00257	0.09803	0.018	0.07636	0.2263	0.27246	0.18188	0.16782	-0.08811	-0.09263	-0.37766	0.16128	0.07326
0.2503	0.21284	-0.03994	0.08807	-0.00071	0.05379	0.14305	0.26281	0.21033	0.14819	-0.12551	-0.19515	-0.43808	0.12404	0.06611
0.21493	0.36727	-0.08691	0.07449	-0.02159	0.08459	0.21791	0.27676	0.18805	0.05676	-0.10614	-0.11489	-0.25595	0.15398	0.08696
0.17487	0.32398	-0.00446	0.09594	-0.00829	0.06033	0.19855	0.2998	0.18881	0.12114	-0.16004	-0.12485	-0.2304	0.16805	0.08351
0.21392	0.3547	0.01822	0.09198	-0.02065	0.07004	0.183	0.27971	0.19286	0.08313	-0.13302	-0.17566	-0.06148	0.15336	0.08549
0.19686	0.32499	-0.02566	0.12955	0.03983	0.02625	0.13482	0.27749	0.22611	0.19756	-0.03886	-0.01195	-0.48071	0.16725	0.06866
0.17062	0.19422	-0.01412	0.09843	-0.01381	-0.01127	0.18891	0.25373	0.21801	0.1599	-0.14105	0.01999	-0.47349	0.16474	0.07109
0.22881	-0.06934	-0.11474	0.09297	0.03431	0.06023	0.17526	0.2792	0.28842	0.17999	-0.06092	-0.09008	-0.39581	0.15687	0.07278
0.23238	0.31388	-0.05093	0.11528	-0.00063	0.066	0.18707	0.29881	0.19093	0.15819	-0.12498	-0.04752	-0.48512	0.12173	0.08489
0.2483	0.34628	-0.07117	0.11058	0.00877	0.01665	0.2089	0.27484	0.15917	0.14623	-0.13638	-0.08207	-0.4025	0.16495	0.08118
0.21291	-0.04835	-0.07465	0.07785	0.02495	0.03934	0.06958	0.26931	0.19256	0.18217	-0.12231	-0.21964	-0.47877	0.14166	0.07398
0.25434	0.28484	-0.05851	0.08031	0.01175	0.0711	0.19241	0.30141	0.18082	0.1566	-0.06347	-0.16387	-0.36269	0.13451	0.08566
0.19352	0.27194	-0.02575	0.11465	-0.00548	0.05431	0.18068	0.29375	0.17112	0.07471	-0.11139	-0.22459	-0.29223	0.14658	0.09151

ESIS_PP	NESIS_P	RG_RXR	ZNF143	JAK2_TA	OSTATE	TATE_CA	TATE_CA	OSTATE	IAN_CAN	IAN_CAN	VARIAN	OVARIAN	GENES_R	RGUS_AND
0.09483	0.08352	0.0647	0.07955	0.16022	-0.36283	0.0544	0.06219	0.09847	0.11918	0.13517	0.08921	0.12945	0.32908	0.21715
0.06672	0.07056	0.03907	0.1002	0.15692	-0.35721	-0.01012	0.04005	0.08283	0.11939	0.09422	0.08237	0.10268	0.2861	0.2128
0.11484	0.08798	0.06787	0.10501	0.09307	-0.3621	0.01529	0.10932	0.10179	0.12562	0.10045	0.09496	0.10976	0.28391	0.24579
0.12675	0.08555	0.05909	0.07573	0.06991	-0.2016	0.02308	0.15236	0.06646	0.13392	0.0777	0.08683	0.09365	0.31692	0.27516
0.08628	0.07151	0.04466	0.06847	0.13003	-0.15143	0.01255	0.16838	0.08625	0.13835	0.05256	0.09362	0.07926	0.29661	0.22981
0.08812	0.07047	0.04377	0.11224	0.13081	-0.22172	0.00625	0.12638	0.08284	0.12793	0.08931	0.0828	0.09715	0.28877	0.20865
0.09625	0.07699	0.05405	0.09569	0.12562	-0.40556	0.01194	0.15705	0.10342	0.12999	0.08333	0.08149	0.11316	0.25565	0.24868
0.11607	0.08371	0.05968	0.0664	0.05755	-0.20107	0.02826	0.15924	0.0416	0.14341	0.03841	0.11091	0.07689	0.28522	0.22928
0.09794	0.07325	0.03832	0.06852	0.08902	-0.4143	0.01925	0.2013	0.0329	0.1443	0.01898	0.10708	0.06775	0.28043	0.20167
0.09601	0.07091	0.04928	0.08495	0.09658	-0.39991	0.00676	0.20701	0.06809	0.12987	0.03738	0.09844	0.06317	0.26694	0.22997
0.11174	0.0872	0.07444	0.09203	0.09436	-0.35275	0.04421	0.00652	0.12732	0.09393	0.11092	0.07056	0.11914	0.27958	0.238
0.10413	0.08285	0.05827	0.11957	0.15147	-0.41394	0.02067	0.12056	0.14199	0.08652	0.11812	0.07539	0.13638	0.31304	0.24394
0.11013	0.08976	0.06052	0.12465	0.11102	0.04301	-0.01166	-0.04579	0.14941	0.09689	0.13007	0.03506	0.15989	0.3263	0.24913
0.1188	0.08381	0.05833	0.06848	0.10781	-0.42296	0.01237	0.10854	0.09752	0.1115	0.08585	0.08869	0.11174	0.29893	0.21859
0.10297	0.08148	0.05922	0.0999	0.11788	-0.27409	0.02715	0.08515	0.11501	0.12575	0.10604	0.09974	0.10133	0.30301	0.26925
0.11784	0.099	0.08777	0.12176	0.11214	-0.10152	0.04325	-0.04498	0.16406	0.05543	0.12173	0.04297	0.13744	0.31615	0.21862
0.10744	0.07568	0.05003	0.06782	0.09654	-0.19958	0.01839	0.08794	0.09967	0.1536	0.0935	0.08954	0.10875	0.31483	0.25028
0.10667	0.07774	0.04818	0.06653	0.09465	-0.34687	0.00418	0.17438	0.05106	0.14041	0.05495	0.11165	0.07366	0.30148	0.22069

GUS_AND	ETTS_ES	ETTS_ES	RESPON	RESPON	OGENIC	LIM2_TA	LIM2_TA	CANCERS	S_CANCE	S_CANCE	PROSTA	D_BY_HO	D_BY_HO	MO4_TAR
0.18192	0.28313	0.20732	0.08562	0.15119	0.07539	0.04829	0.05531	0.01659	0.15106	0.09587	0.10861	-0.02448	0.13162	0.07737
0.1278	0.25296	0.14024	0.0533	0.14456	-0.0186	0.05549	0.07989	0.06362	0.10665	0.0943	0.06149	0.07756	0.04449	0.08048
0.14968	0.2682	0.1636	0.07902	0.14633	0.06752	0.066	0.07375	0.05799	0.12768	0.13416	0.06404	0.01534	0.06	0.07486
0.19463	0.28174	0.16337	0.07481	0.1556	0.13712	0.04619	0.04082	0.10267	0.12493	0.15205	0.05801	-0.04811	0.10135	0.06594
0.16675	0.26161	0.16056	0.10084	0.1598	0.12374	0.04187	0.06804	0.06298	0.096	0.11371	0.07278	0.12723	0.05879	0.06864
0.20907	0.26465	0.14024	0.0695	0.17108	0.08613	0.03434	0.04642	0.02634	0.11981	0.11635	0.06103	0.11126	0.06275	0.04988
0.19225	0.21593	0.1599	0.05694	0.17655	0.11567	0.0412	0.06093	0.02788	0.10152	0.14215	0.05857	0.04594	0.09186	0.06179
0.18311	0.24553	0.13701	0.10015	0.14454	0.13785	0.0435	0.03009	0.11239	0.11806	0.1552	0.06852	-0.02143	0.10518	0.05976
0.21582	0.25789	0.14632	0.08899	0.15818	0.10671	0.0393	0.06029	0.17512	0.11071	0.16281	0.04353	0.05072	0.07946	0.06076
0.19229	0.26365	0.15778	0.08612	0.16054	0.10958	0.04726	0.04768	0.05213	0.09804	0.14471	0.05339	0.10112	0.09597	0.0533
0.16306	0.26505	0.11726	0.06503	0.14235	0.06589	0.06199	0.06121	0.09939	0.1555	0.1004	0.07891	-0.06139	0.16743	0.06318
0.22787	0.25208	0.14229	0.06693	0.15552	0.09907	0.05956	0.03724	0.01448	0.14075	0.12668	0.09356	0.05443	0.15039	0.07234
0.0953	0.23698	0.12256	0.05604	0.14624	0.0226	0.06932	0.08679	0.07992	0.13958	0.06904	0.09122	-0.04362	-0.06917	0.08378
0.16996	0.2843	0.13335	0.08089	0.14403	0.12797	0.04553	0.05977	0.08179	0.12719	0.12104	0.08405	0.01589	0.11848	0.07262
0.16493	0.27346	0.1318	0.08589	0.15391	0.14359	0.06352	0.05162	0.02685	0.14519	0.11685	0.07902	0.00685	0.16599	0.07117
0.12544	0.20807	0.09735	0.05688	0.13152	0.01486	0.06426	0.06476	0.11553	0.16561	0.04443	0.09045	-0.06527	-0.00679	0.08915
0.15842	0.27818	0.17645	0.08128	0.15129	0.10935	0.04034	0.05819	0.08099	0.11837	0.12259	0.06231	0.04611	0.08077	0.07117
0.17547	0.27318	0.15465	0.07982	0.13591	0.09047	0.04896	0.03776	0.02279	0.12868	0.13083	0.05223	0.05293	0.00988	0.0637

MO4_TAR	SD1_TAR	SD1_TAR	SIS_OF_B	F_BREA	F_BREA	TED_IN_B	MLL_TA	ANSFORM	NFKB_T	CER_AND	ENT_LIV	ENT_LIV
0.11344	-0.11175	0.16915	-0.14216	0.21829	0.17004	0.07467	0.00073	0.09904	-0.047	0.11427	-0.12134	0.02915
0.1166	-0.10194	0.19966	-0.16346	0.20062	0.23812	0.01658	-0.04967	-0.15546	-0.01431	0.07929	-0.16953	0.06549
0.10934	-0.28197	0.11685	-0.16038	0.26978	0.23786	-0.00897	-0.00183	0.06149	0.09062	0.10535	-0.11704	0.01588
0.08801	-0.2654	0.16835	-0.03612	0.24724	0.19127	0.06339	0.03909	0.08645	0.12404	0.10138	-0.08192	0.01857
0.08653	-0.21408	0.15036	-0.16248	0.26156	0.20746	0.00933	-0.01166	0.08043	0.06142	0.11659	-0.09296	0.045
0.09655	-0.17676	0.17744	-0.13472	0.20491	0.24971	0.06137	0.00187	0.09557	0.09604	0.09237	-0.16811	0.06059
0.08975	-0.18534	0.13114	-0.14644	0.2396	0.201	0.07733	0.05059	0.08288	0.1358	0.08453	-0.17567	-0.01054
0.07845	-0.21034	0.1633	-0.06322	0.28642	0.139	0.05239	0.01837	0.08375	0.13209	0.11604	-0.12776	0.03068
0.07642	-0.14962	0.1754	-0.0792	0.29065	0.14912	0.07969	0.02837	0.09764	0.15014	0.09984	-0.1127	0.0223
0.08705	-0.26903	0.19214	-0.15133	0.27903	0.16384	0.07753	0.0118	0.09781	0.13091	0.10793	-0.07636	0.03519
0.10433	-0.25372	0.1282	0.01521	0.18871	0.22065	0.05237	-0.02422	0.00817	0.01138	0.06881	-0.10275	0.00363
0.10741	-0.2471	0.05804	-0.07506	0.25556	0.19623	0.07082	0.02048	0.18319	0.01236	0.10377	-0.18129	0.01975
0.11205	-0.25546	0.11967	-0.0395	0.1791	0.22156	0.03645	-0.04374	-0.29657	0.05656	0.06848	-0.19505	0.03897
0.09449	-0.21769	0.12447	-0.02409	0.2552	0.17784	0.06179	0.03334	0.05442	0.07706	0.11721	-0.17525	0.04087
0.0957	-0.24458	0.13842	-0.10926	0.22596	0.18479	0.07276	-0.02266	-0.02312	0.00324	0.10817	-0.18123	0.0715
0.12518	-0.22493	0.05193	-0.00509	0.15053	0.22373	0.06436	-0.05811	-0.28286	0.00416	0.09032	-0.09787	0.06286
0.10231	-0.26621	0.18916	-0.03841	0.26723	0.1954	-0.0049	0.01779	0.09435	0.07497	0.12143	-0.13099	0.03076
0.08658	-0.25115	0.15804	-0.10841	0.25958	0.15245	0.0844	0.02042	0.10807	0.1479	0.09651	-0.12195	0.04545

SE_TO_F	GSK3_IN	GSK3_INI	RCE1_TA	RCE1_TA	OF_MLL_	OF_MLL_	OC1_TAR	OC1_TAR	TNF_TAR	R_INVAS	R_INVAS	R_RESP	SPONSE	_CANCE	N_CANCE	R_RADIO	R_RADIO	VE_COLI	VE_COLI	ED_HCP_I
0.13732	0.06203	0.0477	0.13091	0.00921	0.17771	0.09101	0.21203	0.49375	-0.04649	0.13805	0.24226	-0.0258	-0.01448	-0.06177	0.17309	0.17937	0.14835	-0.05277	-0.1725	-0.44644
0.10523	0.06559	0.0246	0.14419	-0.02905	0.17232	0.06444	0.18598	0.4867	-0.01573	0.13969	0.24272	0.01746	-0.04146	0.01433	0.11687	0.17299	0.15964	-0.12436	-0.07959	-0.39851
0.07501	0.07769	0.05822	0.15634	-0.00929	0.17705	0.11824	0.1602	0.46063	0.07393	0.14282	0.24151	-0.01065	-0.02872	-0.14072	0.18857	0.17657	0.14882	-0.01345	-0.20139	-0.34393
0.0848	0.0493	0.10952	0.13381	0.018	0.15879	0.10723	0.26013	0.48776	-0.01168	0.14017	0.24566	-0.02221	-0.01853	-0.11425	0.12963	0.16072	0.14873	-0.00014	-0.12555	-0.34908
0.09037	0.05636	0.07969	0.13083	-0.02223	0.17435	0.1235	0.16758	0.47049	0.05951	0.1237	0.2431	-0.03237	0.01115	-0.09517	0.1613	0.17483	0.14661	0.09732	-0.30848	-0.41409
0.15414	0.08352	0.05251	0.12609	0.00163	0.16836	0.11138	0.18772	0.48371	0.00848	0.13548	0.24942	0.01719	0.03261	-0.09858	0.21167	0.17592	0.12463	-0.03767	-0.08204	-0.36786
0.09263	0.08934	0.08216	0.14393	-0.00421	0.18189	0.08502	0.22467	0.48001	0.05095	0.13432	0.24744	0.01557	0.02677	-0.1266	0.19231	0.1814	0.14938	-0.03269	-0.22922	-0.34061
0.05061	0.03242	0.12521	0.12674	0.0203	0.14001	0.15205	0.20301	0.48124	-0.0611	0.1289	0.24045	-0.0198	0.00738	-0.22009	0.09925	0.16442	0.14069	0.03314	-0.01536	-0.4039
0.06734	0.03967	0.13234	0.12979	0.01731	0.14372	0.16391	0.224	0.47437	-0.05559	0.12908	0.23468	-0.03302	0.00965	-0.1398	0.11481	0.17365	0.13973	0.14024	-0.26905	-0.39225
0.04534	0.03864	0.12664	0.13482	0.00677	0.13497	0.1515	0.21879	0.46292	0.00402	0.12642	0.23744	-0.00925	0.02146	-0.18871	0.14058	0.15041	0.14903	0.08588	-0.24859	-0.38808
0.09778	0.09627	0.00122	0.14369	-0.00732	0.15534	0.07132	0.0639	0.486	-0.03716	0.13168	0.24281	-0.01881	-0.03745	0.0286	0.18479	0.17386	0.12909	-0.12628	-0.10333	-0.31994
0.11393	0.0761	0.06087	0.14461	0.01647	0.18115	0.0943	0.13048	0.43086	0.15948	0.13441	0.24554	0.02862	0.06946	-0.25922	0.18459	0.16652	0.14474	-0.08677	-0.13739	-0.31291
0.05523	0.08877	-0.02177	0.13514	-0.0054	0.14929	0.01268	0.12103	0.49005	-0.02493	0.13415	0.23365	-0.05876	-0.08265	-0.13055	0.13107	0.19381	0.1362	-0.20792	-0.15985	-0.23182
0.09842	0.05075	0.07965	0.13943	-0.01836	0.16851	0.11216	0.14083	0.4904	-0.03099	0.14006	0.24121	-0.0374	-0.02201	-0.10092	0.15359	0.17231	0.15614	-0.01621	-0.22324	-0.31827
0.08842	0.07879	0.05866	0.13914	-0.00495	0.17135	0.11225	0.24676	0.48265	0.08861	0.12648	0.24239	0.0028	-0.04781	-0.0807	0.15362	0.16848	0.14195	-0.10634	-0.06377	-0.4719
0.07433	0.10447	-0.04018	0.16403	-0.04461	0.16529	-0.01306	0.18571	0.48934	0.12431	0.12379	0.23432	-0.0309	-0.07932	-0.1023	0.1216	0.18238	0.14369	-0.1337	-0.17442	-0.18978
0.05776	0.03116	0.07144	0.13451	-0.01413	0.17435	0.11085	0.28216	0.47384	0.06463	0.14347	0.24586	-0.01484	-0.03903	-0.07973	0.15926	0.17108	0.13668	-0.07122	-0.26945	-0.31111
0.04669	0.04767	0.10684	0.13377	0.00942	0.15391	0.09544	0.24478	0.46398	-0.04847	0.1363	0.24218	-0.02063	0.0097	-0.12486	0.17125	0.18183	0.14647	0.04583	-0.32805	-0.3515

ATED_HC	ATED_ICP	ATED_IC	ATED_IN	D_LCP_I	ATED_LC	STRESS_	_STRESS	STRESS	IVE_STR	PTATION_	PTATION_	IR34A_TA	ARGETS_	GATAI_T	RCAI_TA	RCAI_TA
-0.44644	0.34085	0.34085	0.39863	0.40074	0.40074	0.18322	0.09946	0.19584	0.03642	0.15317	0.16918	0.07562	0.09952	0.17745	0.18827	0.20459
-0.39851	0.4335	0.4335	0.43474	0.39193	0.39193	0.16613	0.08791	0.20142	0.02454	0.11543	0.17096	0.06183	0.10977	0.15194	0.20531	0.19317
-0.34393	0.35159	0.35159	0.43192	0.37696	0.37696	0.13812	0.10991	0.19843	0.04381	0.10088	0.1518	0.09287	0.11475	0.21377	0.20458	0.1977
-0.34908	0.31636	0.31636	0.43438	0.23992	0.23992	0.12607	0.11767	0.20072	0.03752	0.11709	0.18569	0.0573	0.08934	0.16679	0.18029	0.20376
-0.41409	0.41589	0.41589	0.4062	0.3264	0.3264	0.10796	0.12116	0.17724	0.05145	0.07009	0.13456	0.07556	0.09254	0.25116	0.16909	0.19739
-0.36786	0.42646	0.42646	0.44513	0.29505	0.29505	0.10587	0.08406	0.17669	0.06209	0.10532	0.13315	0.06873	0.11014	0.27872	0.18248	0.18495
-0.34061	0.42769	0.42769	0.42611	0.13757	0.13757	0.1443	0.0934	0.21014	0.07368	0.1123	0.15158	0.04503	0.11716	0.10517	0.20264	0.18559
-0.4039	0.3493	0.3493	0.43174	0.12084	0.12084	0.10365	0.10413	0.18364	0.04403	0.07455	0.17737	0.0703	0.07881	0.23683	0.16104	0.19385
-0.39225	0.35265	0.35265	0.45006	0.24432	0.24432	0.11766	0.11078	0.21145	0.05309	0.06968	0.15833	0.06418	0.0759	0.20584	0.15842	0.19608
-0.38808	0.41853	0.41853	0.43491	0.2068	0.2068	0.12487	0.09443	0.2067	0.05357	0.06166	0.12171	0.06183	0.08428	0.17738	0.16794	0.1945
-0.31994	0.3789	0.3789	0.43667	0.39387	0.39387	0.14313	0.14734	0.16423	0.05345	0.07713	0.06303	0.07346	0.11244	0.10624	0.19854	0.19272
-0.31291	0.43315	0.43315	0.43333	0.30139	0.30139	0.10515	0.10919	0.20063	0.0332	0.16226	0.13844	0.06064	0.11849	0.19453	0.19576	0.19131
-0.23182	0.3715	0.3715	0.40303	0.43421	0.43421	0.15515	0.15364	0.20448	0.09988	0.06097	0.04282	0.05267	0.10648	0.1589	0.21225	0.18925
-0.31827	0.40567	0.40567	0.43861	0.30351	0.30351	0.11591	0.11835	0.17685	0.01176	0.08527	0.14693	0.05649	0.0939	0.16308	0.18733	0.20932
-0.4719	0.42293	0.42293	0.42276	0.30738	0.30738	0.14347	0.10039	0.16894	0.04676	0.07522	0.10828	0.06656	0.11232	0.17182	0.19514	0.19031
-0.18978	0.32323	0.32323	0.42804	0.41113	0.41113	0.14036	0.10409	0.15033	0.04554	0.08008	0.0528	0.05385	0.13038	0.09297	0.21343	0.19058
-0.31111	0.34842	0.34842	0.43615	-0.03717	-0.03717	0.11626	0.10057	0.19208	0.0273	0.08545	0.13369	0.05708	0.09337	0.17728	0.18218	0.19918
-0.3515	0.37678	0.37678	0.42505	0.38189	0.38189	0.13257	0.11005	0.1904	0.01546	0.11337	0.18649	0.0693	0.0893	0.21028	0.16207	0.19866

HESIN_T	POR_DC	TARGETS	TARGETS_	TARGET	TARGETS	VEGFA_	EGFA_TA	EGFA_TA	EGFA_TA	L_CARCI	L_CARCI	CORTIC	TICAL_TU	TICAL_TU	CORTICA
0.04973	0.10389	0.19387	-0.1106	0.14565	0.07621	0.02866	-0.0405	0.05101	-0.02457	0.13693	0.06294	0.06475	0.07217	-0.19265	0.14556
0.00983	0.07821	0.06882	-0.14484	0.08366	0.05462	-0.00467	-0.06454	-0.00994	-0.02066	0.1142	0.15208	0.04608	0.0091	-0.03146	0.1541
0.07302	0.15342	0.15881	-0.16127	0.08432	0.11426	0.07974	0.03168	0.09073	0.07228	0.19564	0.12839	0.05855	0.04068	-0.19611	0.1426
-0.0123	0.13667	0.13721	-0.10777	0.10484	0.09544	0.07774	0.01549	0.07506	0.04524	0.22848	0.02214	0.06657	0.10028	-0.19925	0.13932
0.02946	0.12553	0.14806	-0.05032	0.09691	0.06514	0.06685	-0.19289	0.10398	-0.02242	0.2164	0.06674	0.04487	0.11398	-0.2035	0.13856
0.018	0.14108	0.03402	-0.04928	0.06451	0.12612	0.12206	0.02799	0.12277	0.10336	0.19072	0.08678	0.07751	0.2316	-0.11791	0.14088
0.00543	0.11948	0.11852	-0.04694	0.07383	0.08164	0.16018	0.10329	0.13408	0.15221	0.22736	0.08158	0.07381	0.26835	-0.14887	0.12672
0.00809	0.13437	0.13345	-0.06686	0.0478	0.09854	0.07459	-0.00583	0.06955	0.05191	0.22675	-0.0017	0.07283	0.11802	-0.3127	0.14124
0.03363	0.13604	0.1299	-0.07459	0.04463	0.08385	0.07144	0.02825	0.05245	0.05915	0.26628	0.00322	0.0699	0.14704	-0.27619	0.14314
0.02457	0.18531	0.11776	-0.02096	0.09987	0.11026	0.0687	-0.02944	0.05254	0.04317	0.24651	0.02109	0.05823	0.1641	-0.33015	0.1269
0.04116	0.12907	0.1573	-0.09611	0.15893	0.09611	0.03242	-0.11579	0.06885	-0.0561	0.14124	0.04613	0.05728	0.03523	-0.12311	0.15294
0.0664	0.16113	0.18666	-0.05035	0.10776	0.10203	0.09851	-0.06139	0.09026	0.118	0.17664	0.09588	0.06603	0.11937	-0.18752	0.13322
0.10094	0.08068	0.04916	-0.04057	0.14491	0.11242	0.02564	-0.05595	0.02559	-0.00508	0.10185	0.11	0.05749	0.01555	-0.11944	0.14902
0.07226	0.1705	0.18045	-0.10736	0.16351	0.10388	0.0851	-0.00674	0.0942	0.02634	0.21198	0.05086	0.06043	0.06355	-0.27733	0.13862
0.05392	0.13417	0.13634	-0.04409	0.12278	0.13835	0.06218	0.01092	0.06412	0.05408	0.1788	0.08975	0.0783	0.09866	-0.19433	0.14714
0.07092	0.11846	0.10006	-0.09763	0.13857	0.0923	0.02515	0.00463	0.04247	0.01388	0.15668	0.10172	0.05227	-0.08806	-0.07631	0.13669
0.06055	0.12525	0.16283	-0.09623	0.13132	0.06702	0.07509	-0.06746	0.08514	0.02765	0.14845	0.05781	0.07005	0.13409	-0.21693	0.13494
0.03356	0.13984	0.17798	-0.10783	0.07488	0.0776	0.06189	-0.04567	0.07203	0.01806	0.24077	0.03186	0.06124	0.07996	-0.27025	0.13641

ATRIC_C	ACLITAX	SPLATIN	PLATIN_	TOMA_WI	CELL_C	D_CELL_	CELL_C	LL_CYCL	_CELL_C	D_CELL_	RGETS_O	P_BY_HB	WING_AN	DING_AND	PML_INT	STAT5A_T
0.13443	0.17551	0.34861	0.20234	0.00863	0.0291	0.082	0.12711	-0.06934	0.10046	-0.0185	0.07302	0.16261	0.48811	0.25554	0.26651	0.04015
0.1681	0.15877	0.31753	0.18874	0.07961	-0.00498	0.09292	0.14051	-0.00776	0.10413	0.02066	0.01162	0.11925	0.45869	0.20906	0.28004	0.02607
0.1833	0.16862	0.33353	0.20533	0.09479	0.03617	0.09257	0.11875	0.03948	0.08715	0.0002	-0.08391	0.18426	0.47085	0.20754	0.28499	0.08025
0.13559	0.14552	0.341	0.19048	0.05099	0.00735	0.06868	0.10039	-0.04813	0.09064	-0.04428	-0.0344	0.24255	0.48758	0.31569	0.27628	0.09768
0.13999	0.15297	0.332	0.04882	0.05655	-0.00372	0.06482	0.11246	-0.04358	0.10244	-0.03101	-0.08537	0.23211	0.49181	0.27939	0.27102	0.08191
0.19469	0.17517	0.35587	0.08202	0.05877	0.01008	0.07887	0.13192	0.04626	0.09855	-0.0105	-0.0296	0.18683	0.45535	0.2901	0.30192	0.02508
0.17799	0.16748	0.37058	0.19943	0.12723	-0.03178	0.07952	0.11348	-0.04247	0.05655	-0.02557	-0.05189	0.21088	0.47842	0.14271	0.28696	0.07611
0.11634	0.16582	0.34887	0.0914	0.01312	-0.03942	0.05034	0.09612	-0.1554	0.07748	-0.06215	-0.09927	0.24343	0.49163	0.32943	0.26884	0.10647
0.12482	0.16944	0.32984	0.07137	0.06218	-0.03872	0.04319	0.10737	-0.09778	0.07362	-0.04898	-0.06222	0.28126	0.48917	0.28865	0.27466	0.1001
0.12682	0.16785	0.33723	0.09341	0.00918	-0.02631	0.0548	0.11065	-0.15022	0.07223	-0.04673	-0.13943	0.28138	0.49093	0.31204	0.26987	0.10163
0.19727	0.14812	0.32272	0.08147	0.17375	0.04195	0.08114	0.12697	0.09219	0.08546	-0.00383	0.04285	0.11105	0.49199	0.19157	0.30884	0.05313
0.16453	0.15075	0.3496	0.21127	0.02069	0.04524	0.05854	0.11636	0.04671	0.08577	0.00422	0.023	0.18284	0.47507	0.20542	0.28265	0.04813
0.19561	0.17657	0.31695	0.1798	0.13115	0.05953	0.07622	0.13922	0.04557	0.13119	0.01017	-0.04139	0.04116	0.41853	0.11072	0.29294	0.00129
0.12637	0.13352	0.33877	0.18132	0.12283	-0.01712	0.06379	0.11938	-0.1689	0.07105	-0.04639	-0.11438	0.19642	0.4911	0.19181	0.29164	0.07359
0.17229	0.16074	0.34191	0.13723	0.01487	0.03876	0.08733	0.11035	-0.00495	0.08957	-0.00467	-0.00158	0.16857	0.49163	0.22928	0.28573	0.05863
0.19671	0.17179	0.32632	0.16986	0.11838	0.05101	0.07769	0.1215	0.06874	0.08208	0.00399	0.00927	0.08954	0.39035	0.13117	0.29487	-0.00774
0.10591	0.1644	0.32877	0.19953	0.0548	-0.02318	0.06276	0.10971	-0.19463	0.08572	-0.06007	-0.06586	0.15612	0.4867	0.24158	0.26714	0.07557
0.11976	0.15203	0.34012	0.13816	0.01741	-0.02137	0.06308	0.10048	-0.09319	0.09151	-0.04867	-0.10553	0.2457	0.48917	0.31067	0.28333	0.08576

TAT5A_TA	AT5A_TA	STAT5A_T	TOS_LUN	TOS_LUN	SE_TO_PR	SE_TO_PR	PONSE_T	WNT_SI	ESR1_TA	ESR1_TA	ESR2_TA	ESR2_TA	T_TUMOR	_TUMOR	MELANO	MELANO
0.03537	0.12641	0.06093	0.03929	-0.00468	0.03463	-0.00853	-0.02824	0.19282	0.07116	0.03775	0.1208	0.0629	0.32228	0.17064	-0.09431	0.08692
0.01271	0.05637	0.02001	0.08469	0.11554	0.05299	-0.01785	-0.0486	0.19988	-0.09318	0.02388	0.17126	0.091	0.24696	0.01029	-0.13904	0.12251
-0.01273	0.0466	0.00482	0.07704	0.05124	0.0121	0.02156	0.02295	0.15622	-0.06077	0.05404	0.18946	0.01117	0.2467	0.18513	-0.08976	0.13618
0.0392	0.08478	0.05507	0.11399	0.02003	0.0773	0.0118	0.1194	0.16684	-0.28536	0.02775	0.14975	0.09548	0.34042	0.22184	-0.06579	0.09418
0.0204	0.06958	0.04118	0.08503	0.08918	0.03289	0.02069	0.13919	0.14097	-0.11467	0.0668	0.14687	0.07243	0.2137	0.20392	-0.00367	0.0734
0.02305	0.11307	0.05394	0.10613	0.09008	0.04126	0.01467	0.00849	0.21273	-0.15536	0.11121	0.09999	0.07293	0.17067	0.15077	-0.07387	0.12707
0.02581	0.08221	0.01779	0.0526	0.05257	0.06593	0.00373	0.03493	0.1749	-0.17738	0.11971	0.13922	0.05499	0.18647	0.19665	-0.06649	0.11607
0.06521	0.08502	0.06208	0.1252	0.06308	0.07161	-0.0296	0.17008	0.20347	-0.23322	0.06364	0.0752	0.07372	0.33854	0.24034	-0.04239	0.06507
0.02233	0.11747	0.06066	0.12617	0.04629	0.06824	-0.02755	0.18658	0.20445	-0.31478	0.08127	0.07813	0.04817	0.35072	0.21505	-0.01274	0.06686
0.02017	0.06253	0.03607	0.15637	0.04324	0.04291	-0.02184	0.16456	0.16138	-0.31636	0.04928	0.05696	0.06001	0.30199	0.22197	-0.01989	0.06832
-0.00484	0.0518	0.00154	0.02126	0.10819	0.03143	0.03259	-0.10211	0.15059	-0.04351	0.02717	0.22008	0.03861	0.1467	0.18263	-0.1167	0.14825
0.00648	0.09614	0.02147	0.02288	0.08363	0.04006	0.00332	-0.05608	0.17004	0.04421	0.02579	0.17612	0.06401	0.13718	0.20572	-0.08393	0.13686
-0.0212	0.05159	-0.01953	0.03599	0.07729	0.08041	0.03995	-0.21717	0.18831	0.20011	0.09886	0.19775	0.12877	0.18667	0.18322	-0.11315	0.14073
0.03328	0.07251	0.03859	0.06698	0.06233	0.04103	-0.01897	0.06002	0.14742	-0.22847	0.04439	0.10038	0.07562	0.30632	0.19446	-0.07853	0.09309
0.02111	0.09984	0.03364	0.08769	0.06055	0.04117	0.0177	0.00346	0.19025	-0.29981	0.08375	0.16713	0.10827	0.19993	0.19119	-0.1122	0.11949
-0.00292	0.00276	-0.0177	0.02059	0.0775	0.01985	0.00595	-0.25715	0.13285	0.12559	0.02305	0.16819	0.08755	0.1278	0.10571	-0.13201	0.16989
0.02869	0.10765	0.05276	0.0567	0.06782	0.05269	-0.01479	0.04427	0.17118	-0.02131	-0.00145	0.17739	0.12736	0.30563	0.189	-0.09002	0.06759
0.02831	0.03717	0.04226	0.11244	0.07119	0.04278	-0.01049	0.14062	0.13708	-0.18989	0.02822	0.09057	0.07956	0.3567	0.07788	-0.05211	0.0646

R_HYPOX	YPOXIA_	R_HYPOX	GRADED_	TISSUE_	ONIC_STE	METRIUM	METRIUM	METRIAL_	ONDRIA_	ASOME_G	EBNAI_T	EBNAI_T	NCER_RE	NCER_R	ASION_A
-0.04716	0.194	0.18635	-0.0802	0.01277	0.19111	0.09341	0.22331	0.25421	0.16804	0.1735	0.11533	0.23003	0.08304	0.1122	0.02878
-0.10678	0.18193	0.17776	-0.08561	0.01255	0.21825	0.08626	0.11799	0.15886	0.14522	0.17533	0.1073	0.22473	0.04314	0.10887	-0.08845
-0.01015	0.18027	0.16883	-0.10365	0.01282	0.21042	0.0757	0.13089	0.28663	0.1613	0.15088	0.12185	0.23975	0.09309	0.08735	-0.07771
0.06867	0.18389	0.16439	-0.10013	0.02829	0.17993	0.02046	0.22464	0.40296	0.14078	0.16095	0.12216	0.216	0.09228	0.13568	-0.03279
0.13924	0.17144	0.15174	-0.11459	0.00674	0.1909	0.02737	0.13517	0.40962	0.12557	0.19241	0.10434	0.22081	0.08874	0.09836	-0.01797
0.09719	0.19302	0.18359	-0.1646	0.01286	0.21394	0.10955	0.11182	0.42035	0.12188	0.1578	0.12328	0.22983	0.11461	0.1305	-0.13974
0.04191	0.19143	0.14795	-0.07293	0.02821	0.20973	0.07724	0.12829	0.42105	0.16429	0.15146	0.1148	0.23784	0.13496	0.12741	-0.07128
0.11182	0.19399	0.14651	-0.12591	0.03226	0.15731	0.09251	0.14045	0.41168	0.12763	0.17397	0.12873	0.2206	0.12395	0.11679	-0.00146
0.15334	0.18407	0.13638	-0.02825	0.04124	0.16046	0.03405	0.06529	0.40019	0.12112	0.18055	0.11794	0.21978	0.09428	0.15641	-0.02115
0.18411	0.17792	0.13078	-0.06654	0.00243	0.17224	0.06188	0.18423	0.43608	0.14198	0.16898	0.09257	0.20954	0.0789	0.10905	0.00136
-0.16683	0.20175	0.18748	-0.1119	0.02344	0.21877	0.09047	0.32152	0.22104	0.17017	0.17103	0.08875	0.22828	0.09665	0.09787	-0.07174
0.02504	0.18721	0.17507	-0.08231	0.03399	0.20588	0.1475	0.19752	0.37699	0.16035	0.1649	0.13071	0.22846	0.10317	0.1419	-0.06135
-0.23561	0.18026	0.17625	-0.09927	0.01803	0.21778	0.05315	0.11581	0.18968	0.11126	0.16346	0.13924	0.2229	0.08983	0.06732	-0.06474
0.05513	0.17873	0.16695	-0.11175	0.02087	0.17881	-0.03096	0.16659	0.36675	0.14092	0.16541	0.12596	0.2204	0.10601	0.11867	-0.07709
-0.05244	0.18792	0.16873	-0.10876	0.0219	0.20036	0.10156	0.28295	0.33349	0.1386	0.16698	0.12427	0.24111	0.09781	0.12006	-0.02112
-0.29585	0.18139	0.18817	-0.06047	-0.00391	0.23274	-0.01121	0.16446	0.01348	0.17043	0.15611	0.08672	0.2197	0.07878	0.0806	-0.08545
0.04629	0.18476	0.17443	-0.13762	0.02486	0.19441	0.0448	0.21695	0.31668	0.1798	0.15051	0.09842	0.23809	0.09734	0.13568	-0.06262
0.12711	0.17934	0.16302	-0.09393	0.02493	0.17394	0.08437	0.08536	0.34301	0.15463	0.17701	0.08965	0.22011	0.09184	0.11458	0.0044

ASION_A	TUMOR_R	TUMOR_R	RUNX_TA	RUNX_TA	RESPONS	RESPONS	EIMER_DI	EIMER_DI	BY_CDKN	S_BY_CD	ELL_MIGR	X_TARGET	X_TARGE	X_TARGE	X_TARGE	X_TARGE
-0.03246	0.15072	-0.02808	0.02169	-0.17517	0.16964	0.15756	0.2526	0.19085	0.12238	0.02752	0.10987	0.06971	0.1582	0.08891	0.10944	0.02907
-0.01182	0.26447	-0.02386	-0.00212	-0.31806	0.14593	0.1369	0.21557	0.20985	0.17392	0.00425	0.03637	0.10149	0.19014	0.11254	0.14306	-0.03964
0.0003	0.20544	0.0556	-0.0034	-0.24185	0.22527	0.17301	0.20736	0.19514	0.14839	0.06587	0.05621	0.08199	0.16752	0.11142	0.11647	-0.01474
0.06503	0.13887	0.05452	-0.01579	-0.17196	0.26939	0.23409	0.19796	0.26227	0.15203	0.00278	0.08879	0.06993	0.16767	0.13121	0.1006	-0.09787
0.11096	0.14577	0.0792	-0.08244	-0.29827	0.28554	0.18763	0.18411	0.22342	0.14552	0.00202	0.02086	0.09858	0.17534	0.13105	0.12718	-0.03727
0.05346	0.21322	0.03468	-0.03293	-0.17997	0.28255	0.17585	0.22922	0.2058	0.19381	0.08467	0.04562	0.07242	0.16566	0.09806	0.11268	-0.07803
0.05734	0.19737	0.05574	-0.07356	-0.21003	0.30193	0.18902	0.26516	0.21089	0.2056	0.04118	0.07637	0.1035	0.15732	0.18752	0.10266	0.02269
0.06447	0.12244	0.08403	-0.02855	-0.17942	0.26177	0.22424	0.15884	0.26969	0.16301	-0.02818	0.04127	0.05373	0.18552	0.09065	0.1104	-0.0699
0.18788	0.13499	0.17172	-0.03985	-0.04828	0.30324	0.2438	0.1728	0.28479	0.18575	0.00568	0.04678	0.05522	0.17523	0.12413	0.09566	-0.10666
0.14033	0.1459	0.09892	-0.09095	-0.18202	0.28835	0.24516	0.19269	0.23734	0.21125	-0.02955	0.02292	0.08469	0.19937	0.13939	0.12749	-0.05101
-0.14616	0.19829	-0.07933	-0.03222	-0.1916	0.17971	0.11631	0.25331	0.20319	0.17975	0.08842	0.06473	0.11767	0.15916	0.05322	0.15743	-0.10451
-0.0346	0.19082	0.02875	0.07567	-0.33075	0.26043	0.17371	0.23361	0.20151	0.1774	0.03728	0.09003	0.10724	0.15236	0.07909	0.13887	-0.15473
-0.31576	0.23075	-0.04286	0.01146	-0.14026	0.17985	0.06003	0.27096	0.15582	0.16322	0.09085	0.07134	0.12147	0.0947	0.12792	0.10615	-0.04175
0.05728	0.1548	0.0368	-0.01991	-0.16593	0.17205	0.1632	0.21801	0.23016	0.13554	-0.00571	0.05622	0.10567	0.16766	0.15983	0.11894	-0.14753
-0.03375	0.13248	-0.04409	-0.03913	-0.32785	0.25833	0.17618	0.25256	0.2544	0.18012	0.03171	0.0706	0.08616	0.16746	0.09111	0.1273	-0.00769
-0.149	0.19567	-0.16566	-0.05095	-0.40327	0.15164	0.10216	0.23974	0.17119	0.19921	0.07996	0.06131	0.12664	0.14093	0.12425	0.13457	-0.01139
0.01664	0.206	0.01636	0.00229	-0.26529	0.17094	0.15095	0.23727	0.20218	0.14462	-0.0333	0.08327	0.08248	0.1669	0.12823	0.11031	-0.01754
0.10966	0.13963	0.07419	-0.04708	-0.09093	0.27722	0.22115	0.18837	0.23568	0.15232	0.00274	0.03829	0.05156	0.20571	0.09781	0.11729	-0.05885

X_TARGE	HYLATION	SC_S1PR	SC_S1PR	SC_S1PR	1_TARGET	1_TARGE	BP_TAR	BP_TAR	OCRINE_	OCRINE_	GENOUS	GENOUS	G_NOT_	G_NOT_	LING_NO	NDUCED
0.09686	0.10261	0.11675	0.17269	0.02806	-0.23516	0.17169	0.1363	0.07361	0.08481	0.17731	0.02804	-0.0567	0.03949	0.07191	0.03669	-0.03197
0.03843	0.07936	0.05555	0.13392	0.03648	-0.11996	0.20009	0.13017	0.09688	0.08875	0.14461	0.02811	-0.00411	0.03138	0.04229	-0.01298	-0.00273
0.0205	0.1203	0.09886	0.11792	0.07217	-0.10587	0.25542	0.13435	0.12964	0.07744	0.15745	0.03855	0.01876	0.05072	0.03368	0.02925	0.01507
0.08814	0.18661	0.1637	0.13525	0.07839	-0.04034	0.24582	0.14123	0.13937	0.0537	0.1492	0.0551	0.08882	0.01303	0.07227	0.07181	-0.05929
0.02735	0.112	0.12593	0.24162	0.04532	-0.13951	0.18712	0.15854	0.0819	0.05065	0.14402	0.03778	0.05535	0.02095	0.07173	0.06318	0.00674
0.06956	0.1268	0.14577	0.26992	0.04787	-0.20011	0.18854	0.13699	0.13046	0.08192	0.14403	0.07379	0.04127	0.0518	0.10028	0.03863	-0.01045
0.03363	0.15613	0.11846	0.11452	0.12752	-0.11996	0.29986	0.12905	0.15459	0.05421	0.16247	0.0679	0.01279	0.00488	0.11168	0.04603	-0.1267
0.11288	0.16855	0.16627	0.1826	0.06014	0.1425	0.2203	0.17658	0.12064	0.04265	0.17073	0.08159	0.1155	0.02792	0.07542	0.05157	-0.07067
0.09226	0.21153	0.14203	0.17265	0.11532	0.00194	0.27678	0.13996	0.11263	0.07446	0.15694	0.07616	0.15692	0.02397	0.05609	0.07933	-0.06221
0.10165	0.18722	0.13687	0.1368	0.13323	-0.06042	0.30072	0.1623	0.09603	0.03119	0.14987	0.06466	0.11988	0.02766	0.06606	0.02711	-0.04648
0.12263	0.01543	0.01197	0.1122	0.01313	-0.19024	0.19798	0.13752	0.09623	0.08688	0.16645	0.04802	-0.11491	0.0056	0.02894	0.03159	-0.00349
0.10293	0.14094	0.09164	0.09895	0.09931	-0.31478	0.2681	0.15077	0.13548	0.09821	0.17352	0.08852	-0.00095	0.00747	0.09592	0.02062	-0.04544
0.06027	-0.0112	0.16205	0.1451	0.05681	-0.31619	0.22918	0.15056	0.08191	0.05558	0.14254	0.02003	-0.04793	-0.014	0.0517	-0.01069	0.01879
0.06715	0.12461	0.06803	0.13865	0.03003	0.07856	0.19705	0.13753	0.09861	0.06616	0.16843	0.05352	-0.0112	0.01415	0.069	0.05532	-0.05581
0.05123	0.12588	0.09754	0.11625	0.03673	0.02308	0.22314	0.15744	0.11393	0.08076	0.17576	0.04236	0.00994	0.03468	0.05205	0.015	-0.02746
0.11179	-0.04791	0.01394	0.08813	0.04622	-0.31531	0.232	0.12896	0.11017	0.0854	0.1517	0.00597	-0.07537	0.02339	0.06145	0.01146	0.01707
0.07382	0.12128	0.12351	0.10108	0.038	-0.11485	0.17509	0.13828	0.10743	0.07344	0.16574	0.04238	0.02334	0.02081	0.03214	0.07849	-0.07064
0.086	0.11024	0.1166	0.21461	0.11916	-0.13528	0.27296	0.14779	0.08897	0.04648	0.16053	0.05042	0.0459	0.0215	0.07631	0.04431	-0.05487

INDUCED	S_INDUC	_REPRES	_REPRES	RETINOI	RETINOI	SE_TO_TR	SE_TO_TR	L_FAB_M	ELAPSE_	AML_SUR	H_11Q23
0.03223	0.04581	0.03471	0.2016	0.18651	0.26508	0.1649	0.22313	0.1059	0.27737	0.11807	0.10707
-0.00522	0.05992	-0.0203	0.23922	0.23937	0.21707	0.13694	0.23095	0.10554	0.25883	0.11664	0.08769
0.09904	0.11139	0.07042	0.23076	0.21306	0.2868	0.17903	0.18811	0.13164	0.23359	0.08517	0.097
0.17348	0.13847	0.09481	0.10231	0.24093	0.32739	0.16621	0.21582	0.11526	0.2748	0.10708	0.10471
0.21571	0.05389	0.06617	0.14043	0.21349	0.31167	0.14479	0.25524	0.11855	0.27046	0.102	0.11286
0.15575	0.06785	-0.00239	0.17306	0.24324	0.28036	0.16886	0.22655	0.09782	0.24009	0.08603	0.09023
0.20083	0.18565	0.07982	0.11042	0.28775	0.31491	0.17904	0.18819	0.12012	0.24553	0.09208	0.10007
0.24066	0.11655	0.11776	0.17596	0.22221	0.31538	0.17639	0.24057	0.11776	0.28102	0.11512	0.10625
0.25289	0.18373	0.10793	0.1627	0.21986	0.31972	0.14057	0.24745	0.11933	0.27851	0.11016	0.11722
0.19286	0.17104	0.1028	0.14538	0.27545	0.32955	0.15474	0.27978	0.10282	0.26714	0.0943	0.10365
-0.00184	0.07758	0.00904	0.16589	0.20729	0.2503	0.17423	0.12568	0.08801	0.24427	0.09046	0.07536
0.11529	0.16066	0.04189	0.12217	0.23629	0.29293	0.19471	0.22396	0.10281	0.2542	0.08348	0.07931
-0.12921	0.10017	-0.03111	0.07615	0.26123	0.23086	0.17887	0.08026	0.08706	0.214	0.08006	0.06976
0.15067	0.09162	0.05223	0.13805	0.22516	0.30179	0.14309	0.21709	0.11053	0.25018	0.10501	0.09464
0.0928	0.1291	0.02973	0.26179	0.2421	0.24435	0.16178	0.22278	0.1099	0.25874	0.11747	0.09257
-0.11488	0.08019	-0.03823	0.25499	0.22116	0.22075	0.18703	0.09572	0.07442	0.20157	0.07087	0.06281
0.144	0.04384	0.07407	0.27748	0.24278	0.29422	0.1435	0.20656	0.08764	0.2703	0.11225	0.10256
0.14233	0.12932	0.06574	0.17458	0.18116	0.32678	0.1747	0.21765	0.10827	0.25838	0.09312	0.10073

BLASTOM	BLASTOM	_CANCER	_CANCER	CANCER	_CANCER	RATED_IN	ENCED_BT	CEB3_TA	TCEB3_TA	RA_ESX1	G_CANCE	CL3_TAR	CL3_TAR
0.05244	0.12637	0.02077	0.08395	-0.04086	0.30317	0.01296	0.10871	0.19124	0.09769	0.1433	0.38664	-0.03266	0.07314
0.06946	0.13331	-0.01229	0.06512	-0.17591	0.31155	0.02098	0.01506	0.19924	0.10431	0.15536	0.39287	-0.11333	0.05743
0.14237	0.11189	0.01372	0.05832	-0.03895	0.29952	0.02883	-0.07908	0.19047	0.12668	0.16718	0.38886	0.11188	0.08692
0.01195	0.23727	0.11307	0.03368	0.14364	0.30041	0.10479	0.22078	0.17309	0.11997	0.19219	0.36862	0.0997	0.10355
0.06728	0.2113	0.03905	0.04685	0.05246	0.30303	-0.03205	0.02211	0.17721	0.09511	0.17207	0.37402	0.10299	0.07033
0.07525	0.07239	0.17039	-0.0083	0.27239	0.32042	-0.05235	-0.35608	0.20296	0.12737	0.10622	0.37826	0.07648	0.0954
0.09075	0.16174	0.16129	0.04418	0.04635	0.29913	0.00562	-0.19058	0.18301	0.13458	0.10262	0.36383	-0.03866	0.10035
0.04262	0.23156	0.12552	0.0282	0.17296	0.29685	0.03893	0.15885	0.1446	0.13417	0.20596	0.36215	0.23444	0.08839
0.10473	0.25832	0.13782	0.02122	0.14333	0.29228	0.04811	0.18019	0.16252	0.1396	0.17932	0.37542	0.22201	0.09778
0.05663	0.2185	0.15629	0.018	0.15335	0.28744	0.03915	0.14922	0.177	0.1031	0.17701	0.37045	0.14591	0.09162
0.01396	0.10409	0.07024	0.06406	0.04126	0.3	0.03469	-0.05569	0.19312	0.12402	0.08523	0.35277	-0.03871	0.08497
0.08134	0.11094	0.12734	0.05435	0.14414	0.30914	0.00337	-0.03411	0.18136	0.12781	0.1262	0.36971	-0.10085	0.10505
0.0029	0.07551	0.03988	0.11645	0.01854	0.30798	-0.03479	-0.24217	0.18611	0.09956	0.10508	0.3934	-0.30318	0.07024
0.06527	0.18488	0.07412	0.04463	0.06461	0.30476	0.02619	0.07651	0.18164	0.09549	0.14773	0.3549	0.15636	0.06717
0.0599	0.16021	0.05559	0.0232	0.01099	0.30032	0.05519	0.07274	0.18149	0.10996	0.13373	0.38502	0.10977	0.08216
0.00361	0.03996	-0.00441	0.06136	-0.04252	0.29791	-0.04014	-0.20171	0.18723	0.10399	0.02979	0.34083	-0.12789	0.07904
0.06031	0.17124	0.04172	0.05298	0.06342	0.31459	0.04892	0.05385	0.17853	0.11762	0.1603	0.36735	0.06213	0.07161
0.01796	0.20344	0.05575	0.02707	0.07279	0.28963	0.0517	0.16632	0.16509	0.11003	0.18505	0.3723	0.14973	0.09775

ANCER_E	ANCER_E	ST_CANCE	ANCER_E	ANCER_E	ST_CANC	PE_FROM	TS_VIA_P	TS_VIA_P	PE_TO_PR	E_TO_PR	E_TO_PR	E_TO_PR	E_TO_PR	E_TO_PR
0.05513	0.21006	0.05433	0.15362	0.01004	0.03707	0.06441	0.08708	0.00142	-0.04649	0.03074	0.20965	0.14184	0.19465	0.15293
0.03706	0.1668	0.06026	0.16565	-0.08115	0.03721	0.0161	0.07681	-0.03909	-0.11499	-0.0268	0.21877	0.18662	0.18831	0.1653
0.05675	0.14805	0.1318	0.16684	-0.03084	0.00462	0.07823	0.08553	0.02905	-0.04737	0.01394	0.20591	0.17326	0.19266	0.15035
0.12258	0.18506	0.08067	0.1344	-0.0005	0.03937	0.10246	0.04955	0.03064	-0.068	0.07138	0.19094	0.13543	0.21871	0.15173
0.05466	0.20226	0.08299	0.14894	0.01505	0.01436	0.06527	0.08716	0.05724	-0.10046	0.02832	0.19866	0.1474	0.20734	0.14683
0.0514	0.12892	0.06043	0.19378	0.04257	0.0171	0.09091	0.16518	0.01794	0.00294	0.07674	0.20295	0.16008	0.18002	0.14735
0.00913	0.16594	0.08448	0.12281	-0.009	0.01994	0.16874	0.07919	0.05059	-0.04412	0.06907	0.21472	0.17074	0.20857	0.16029
0.07454	0.18495	0.09608	0.14629	0.01879	0.05912	0.08918	0.07337	0.02423	-0.00952	0.05766	0.18897	0.13392	0.19873	0.15181
0.11359	0.1677	0.09793	0.13509	-0.01353	0.05217	0.17197	0.09629	0.02312	0.0083	0.04266	0.18714	0.12622	0.20634	0.14274
0.05784	0.15685	0.07375	0.11794	-0.00765	-0.00048	0.19079	0.05269	0.00186	-0.04906	0.05771	0.19191	0.12683	0.18285	0.15196
0.05024	0.1833	0.05407	0.16031	-0.04783	0.12392	0.04114	0.15795	0.05011	0.01592	0.03519	0.20843	0.1675	0.23257	0.16954
0.03887	0.14075	0.09381	0.13368	0.04443	0.04906	0.12876	0.15719	0.073	0.05431	0.07599	0.22035	0.16906	0.20299	0.1564
0.0141	0.20462	0.03861	0.13599	-0.00582	0.09314	-0.01735	0.07835	-0.05787	-0.10594	0.02344	0.21985	0.16334	0.19843	0.15173
0.05444	0.16788	0.08656	0.14635	0.02715	0.09845	0.07594	0.0888	0.04021	-0.04325	0.05442	0.20353	0.16347	0.2138	0.14682
0.08111	0.12494	0.07483	0.16669	-0.04435	0.06757	0.09356	0.09584	0.06512	-0.04643	0.08887	0.19265	0.17332	0.21947	0.15196
0.01223	0.15818	0.01658	0.15339	-0.02854	0.05121	0.05728	-0.00673	-0.0243	-0.11759	-0.03344	0.22069	0.19103	0.19978	0.15116
0.0836	0.2167	0.07533	0.13239	-0.02359	0.06148	0.03887	0.06053	0.02461	-0.03935	0.0177	0.21197	0.1469	0.19889	0.16749
0.04987	0.20045	0.1015	0.13973	0.00075	0.02854	0.11957	0.10874	0.00341	-0.06991	0.02492	0.20139	0.13386	0.20734	0.15918

E_TO_PR	E_TO_PR	E_TO_PR	E_TO_PR	E_TO_PR	E_TO_PR	E_TO_PR	E_TO_PR	E_TO_PR	E_TO_PR	E_TO_PR	E_TO_PR	SIGNALI	SIGNAL	INIAN_PRO	V_GR_TA
0.20937	0.0092	0.07383	0.16424	0.10827	-0.08626	0.16205	0.17542	0.03797	0.19787	0.13463	0.1563	0.0068	-0.00931	0.05311	0.00377
0.21262	0.06382	0.06747	0.17474	0.16738	-0.11318	0.12643	0.04227	-0.01791	0.18782	0.11588	0.11956	-0.0534	-0.04324	0.06933	0.00502
0.21263	0.04393	0.08514	0.17928	0.11455	-0.04861	0.05993	0.05751	0.01114	0.19018	0.13914	0.15203	0.01988	-0.02461	0.05355	0.01555
0.19134	0.0277	0.09046	0.15997	0.11597	0.06083	0.04962	0.16721	-0.00382	0.1817	0.12545	0.16131	0.0347	-0.02737	0.03103	0.00082
0.20021	0.05863	0.07227	0.16988	0.15024	0.03079	0.00919	0.14917	0.00089	0.19294	0.10968	0.16218	0.00192	-0.01337	0.02198	0.01352
0.20319	0.12973	0.10833	0.18094	0.1296	0.17121	0.13911	0.05507	0.03237	0.17775	0.13949	0.13512	0.03382	0.00442	0.07862	-0.01615
0.2008	0.08426	0.12191	0.17521	0.14929	0.05377	0.10381	0.09363	0.01478	0.19989	0.11737	0.1485	0.05071	0.00346	0.03033	0.0319
0.18756	0.0323	0.10026	0.15632	0.10655	0.11585	0.03446	0.19077	-0.01858	0.17753	0.10345	0.15548	0.01763	-0.02876	0.01122	-0.00875
0.18101	0.03892	0.13673	0.16059	0.10305	0.09337	-0.03294	0.09942	-0.02472	0.17467	0.10474	0.14641	0.03757	-0.03436	0.02336	0.02223
0.18752	-0.00133	0.06085	0.1539	0.12313	0.0964	0.0186	0.17764	0.02414	0.19264	0.10432	0.14672	0.01802	-0.02511	0.04076	-0.0093
0.21416	0.05658	0.05728	0.1792	0.14127	-0.11279	0.16022	0.12084	0.05049	0.20691	0.16651	0.13382	0.05197	0.00575	0.04157	0.01346
0.19965	0.13974	0.10789	0.17754	0.10901	0.02052	0.16044	0.20655	0.05791	0.19643	0.11563	0.16207	0.05346	-0.01786	0.05269	0.0535
0.2097	-0.00375	0.08092	0.17869	0.15788	-0.00964	0.23997	0.02188	0.02456	0.1958	0.14208	0.10915	0.01146	-0.00555	0.00743	0.04664
0.20785	0.03494	0.0773	0.1665	0.12319	-0.01627	0.061	0.14856	0.00823	0.19023	0.14411	0.14659	0.00213	-0.01241	0.03949	-0.01717
0.22011	0.05776	0.08653	0.1649	0.12349	-0.02428	0.11787	0.15866	0.03814	0.19679	0.13428	0.13027	0.02638	0.00237	0.03573	0.07188
0.2353	0.05635	0.06849	0.18373	0.15765	-0.2154	0.17075	0.08214	0.0179	0.20383	0.14044	0.12664	-0.01509	-0.0113	0.08612	0.0349
0.20644	0.01055	0.09454	0.17824	0.13479	-0.04826	0.07529	0.15938	0.02131	0.19212	0.11297	0.13904	0.01168	-0.05487	0.04112	0.00405
0.18333	0.03354	0.07661	0.17771	0.12265	0.02945	0.04485	0.07705	0.02959	0.17597	0.09589	0.15606	0.01776	-0.03321	0.02051	-0.01096

ISE_TO_A	ISE_TO_A	ISE_TO_A	ISE_TO_A	ISE_TO_A	ER_TEST	ULATION	ULATION	MAPK8_T	MAPK8_T	NF143_PA	AP1_TAR	C_TARGE	C_TARGE	_TRANSC
0.05693	0.18005	0.11771	0.19393	0.27433	0.04355	0.09827	0.02527	0.12357	0.07317	0.10571	0.15573	0.15439	-0.00109	-0.11929
0.05809	0.14762	0.15709	0.19362	0.26063	-0.01356	0.09934	-0.02257	0.11474	0.06368	0.13802	0.15311	0.06635	0.07036	-0.10607
0.05252	0.12968	0.13578	0.16512	0.20463	0.04642	0.08391	-0.05386	0.12503	0.06861	0.07009	0.19429	0.12911	0.06477	-0.02793
-0.0102	0.15912	0.10758	0.18714	0.32	0.04678	0.03875	0.06358	0.12237	0.09043	0.00047	0.13698	0.21428	0.01193	-0.15907
-0.05047	0.16582	0.12498	0.18946	0.26058	0.01611	-0.01098	0.03535	0.11465	0.08575	0.00296	0.17884	0.19629	0.03599	-0.20187
0.04483	0.14999	0.13788	0.16966	0.22638	0.06175	0.06243	0.01433	0.11214	0.09369	0.10694	0.14104	0.14122	0.09011	-0.15335
-0.02661	0.09391	0.13732	0.18946	0.24814	0.06976	0.0742	0.05067	0.11642	0.09003	0.08204	0.15043	0.16654	0.06411	-0.09331
-0.00456	0.14486	0.05946	0.17959	0.14743	-0.00021	0.055	0.06829	0.12217	0.09255	-0.03768	0.14123	0.20104	-0.00502	-0.23507
0.02855	0.12868	0.07884	0.18337	0.20167	0.0403	-0.00282	0.03482	0.12089	0.08612	-0.0024	0.16707	0.22752	-0.0082	-0.20621
-0.02857	0.13018	0.08812	0.17535	0.26644	0.00143	0.00619	0.06316	0.11305	0.09488	0.02447	0.16363	0.21816	-0.01504	-0.262
0.09173	0.14498	0.13614	0.18505	0.18965	0.07753	0.08024	0.04617	0.1237	0.07314	0.03586	0.11275	0.06122	0.1092	-0.08343
0.01292	0.13657	0.13199	0.17546	0.23552	0.05487	0.07231	0.03695	0.1212	0.08255	0.02882	0.14941	0.16054	0.07964	-0.13171
0.03942	0.1604	0.1484	0.18275	0.24902	0.07909	0.0911	0.03241	0.12698	0.06595	0.07724	0.14779	0.00408	0.13749	-0.05614
-0.0653	0.15574	0.09128	0.19155	0.20789	0.08756	0.09553	0.0377	0.12273	0.08594	0.06366	0.10128	0.17662	-0.00394	-0.14389
0.00261	0.15203	0.08322	0.17316	0.27481	0.02776	0.07415	0.05098	0.12182	0.08705	0.07696	0.11644	0.13743	0.05425	-0.03822
0.06856	0.16661	0.13158	0.2046	0.34924	0.07487	0.05701	-0.0188	0.12021	0.05939	0.08393	0.11894	0.0357	0.09272	-0.09366
0.00305	0.1497	0.12837	0.18501	0.24085	-0.00518	0.0711	0.02693	0.12799	0.08195	0.09719	0.13055	0.16642	0.00198	-0.16702
0.00713	0.1456	0.07078	0.19157	0.24254	0.04489	0.04017	0.05734	0.12151	0.08474	-0.00544	0.16293	0.2202	0.02812	-0.14367

OS2_TAR	OS2_TAR	CD44_TA	CD44_TA	ONSE_TO	ONSE_TO	OGENES	ESPONS	RESPONS	ANCER_R	ANCER_R	ATA6_TAR	ATA6_TAR	RFERON	ERATING
0.2461	0.18417	0.00431	0.02904	0.099	0.20456	0.11283	0.07283	0.15374	0.14993	0.10291	-0.00865	0.13532	0.07986	0.19626
0.22698	0.18197	-0.02455	-0.00888	0.18337	0.17884	0.07236	0.02954	0.19348	0.12543	0.12312	-0.0573	0.10111	0.13611	0.1949
0.25173	0.22205	0.08273	0.04607	0.24414	0.19995	0.14138	0.00137	0.21246	0.13329	0.11568	-0.06404	0.19229	0.19378	0.19213
0.26499	0.19426	0.08534	0.16754	0.02308	0.25004	0.18155	0.06082	0.22983	0.13541	0.07593	-0.00824	0.1459	0.18261	0.2003
0.26089	0.18739	0.10182	0.06861	0.11626	0.29731	0.07656	0.03324	0.19379	0.12222	0.09362	-0.00811	0.12216	0.15594	0.19257
0.27077	0.20553	-0.01492	0.20489	0.10164	0.14937	-0.01731	-0.03567	0.25208	0.11324	0.11564	-0.02461	0.15908	0.16832	0.194
0.26561	0.20487	0.05647	0.1795	0.17738	0.10765	0.17139	-0.00554	0.32269	0.11441	0.09459	-0.05383	0.1072	0.24143	0.19325
0.27432	0.16998	0.0964	0.17411	-0.23657	0.25825	0.15864	0.0374	0.21793	0.10289	0.0654	-0.01394	0.19815	0.16006	0.21185
0.2606	0.17418	0.11093	0.18245	-0.0805	0.26256	0.18466	0.04983	0.2406	0.11855	0.06121	-0.03056	0.18741	0.21895	0.21266
0.25621	0.16355	0.11795	0.19508	-0.2038	0.26084	0.12274	0.02909	0.25228	0.10224	0.08253	-0.04918	0.09896	0.25666	0.21863
0.24201	0.23271	0.08674	0.12802	0.22459	0.15273	0.14743	0.04272	0.20947	0.13158	0.11993	-0.01862	0.12303	0.00863	0.19574
0.26252	0.19692	0.06594	0.21754	0.16488	0.16084	0.12227	0.03408	0.23402	0.14691	0.11931	0.01321	-0.00447	0.17109	0.17017
0.2504	0.22606	-0.09896	0.12058	0.25912	0.1219	0.1301	0.00141	0.24268	0.14544	0.12511	-0.01762	0.066	0.04397	0.21057
0.26079	0.17847	0.0961	0.0775	-0.2459	0.28031	0.13241	0.01962	0.19244	0.12274	0.08948	-0.05615	0.17501	0.0942	0.20521
0.25976	0.21781	0.04838	0.12629	-0.02026	0.23734	0.0844	0.01047	0.23085	0.14566	0.10601	-0.01078	0.14549	0.11103	0.19834
0.23686	0.22716	-0.04386	0.02183	0.3264	0.20116	0.11645	0.00065	0.19255	0.13046	0.13122	-0.02442	0.09453	0.0824	0.18165
0.25923	0.1633	0.01853	0.15712	-0.04069	0.21352	0.20755	0.01868	0.21652	0.12975	0.09477	-0.03145	0.11225	0.08614	0.20494
0.25582	0.17317	0.09265	0.08164	-0.0347	0.18868	0.20977	0.04384	0.18314	0.12039	0.08173	-0.04089	0.16178	0.23309	0.20432

ISE_TO_C	ISE_TO_C	_IKK_IN	_IKK_IN	_OF_EWS	FERENTI	FERENTI	ERENTIA	ERENTIA	MYELOMA	YELOMA	LE_MYEL	LE_MYEL	MYELOMA	MYELOMA
0.24196	0.04611	-0.05186	-0.03798	0.07161	0.05617	-0.11803	0.09478	0.1606	0.18938	0.03328	-0.02963	0.07473	-0.05281	0.08372
0.23448	0.03006	-0.00112	-0.05469	0.01591	0.07975	-0.06832	0.11075	0.15864	0.18688	0.07043	-0.07738	0.08038	-0.10115	0.11571
0.25157	0.05875	-0.02106	-0.03034	0.05362	0.08004	-0.09005	0.09363	0.12245	0.16292	0.06323	-0.08507	0.07169	-0.11388	0.10228
0.23702	0.07455	-0.05401	-0.0314	0.07867	0.06263	-0.10783	0.12164	0.16253	0.19549	0.02934	-0.05585	0.11601	-0.03432	0.11715
0.23174	0.04786	-0.05036	-0.01715	0.07318	0.0803	-0.11584	0.15069	0.15913	0.18037	0.02478	-0.07369	0.09339	-0.06515	0.11586
0.24427	0.0256	-0.04289	-0.06265	0.08329	0.09701	-0.18272	0.1061	0.16674	0.13376	0.01419	-0.10242	0.05016	-0.08704	0.09408
0.24976	0.05711	-0.04357	-0.01672	0.06219	0.04315	-0.14463	0.10923	0.13664	0.15577	0.02473	-0.06996	0.08274	-0.04352	0.15422
0.21076	0.09695	-0.06312	-0.0167	0.07957	0.04387	-0.08919	0.13749	0.19355	0.16434	0.04304	-0.0603	0.08058	-0.06237	0.07254
0.21975	0.10879	-0.03869	0.03961	0.07158	0.08244	-0.10599	0.15673	0.19981	0.1677	0.03305	-0.0714	0.10973	-0.04304	0.10866
0.22353	0.07126	-0.06219	0.02089	0.10218	0.08515	-0.06451	0.16752	0.16346	0.17811	0.02945	-0.08795	0.06931	-0.05937	0.1254
0.26559	0.04709	-0.0443	-0.05317	0.04141	0.04589	-0.13177	0.08705	0.1101	0.14182	0.01468	-0.02255	0.07575	-0.06728	0.14924
0.26314	0.05047	-0.01148	-0.04178	0.0584	0.06142	-0.10103	0.06844	0.14121	0.1447	-0.01123	-0.05524	0.05917	-0.06846	0.14127
0.24481	0.01328	-0.00834	-0.07178	0.02634	0.04665	-0.06252	0.04912	0.11945	0.12	0.01467	-0.01475	0.063	-0.07462	0.04552
0.22951	0.04266	-0.03835	-0.03127	0.06869	0.04673	-0.1366	0.12415	0.1352	0.15408	0.0305	-0.07992	0.11188	-0.10027	0.12168
0.25148	0.07507	-0.02129	-0.03193	0.07807	0.06277	-0.21248	0.10016	0.13243	0.15294	0.03706	-0.11582	0.10177	-0.06461	0.12116
0.27257	-0.00703	0.00893	-0.04842	0.04082	0.06491	-0.18234	0.03692	0.1551	0.1416	-0.00594	0.00171	0.02417	-0.0776	0.06056
0.23316	-0.00667	-0.04196	-0.0483	0.06128	0.05117	-0.13971	0.06823	0.16773	0.17333	0.02413	-0.08684	0.08999	-0.03869	0.1034
0.22451	0.05651	-0.05392	0.00895	0.08456	0.04042	-0.11367	0.14819	0.19259	0.16466	0.0338	-0.05602	0.11105	-0.03924	0.13467

LE_MYEL	LE_MYEL	TIPLE_MY	PLE_MYE	PLE_MYE	PLE_MYE	PLE_MYE	PLE_MYE	PLE_MYE	PLE_MYE	PLE_MYE	PLE_MYE	PLE_MYE	PLE_MYEL	_MYELO	TIPLE_MY
0.17683	0.02597	0.16104	0.08206	0.0696	0.07749	0.17975	0.0738	0.148	0.09687	-0.21686	-0.05148	-0.08817	-0.10362	0.21468	0.23384
0.18371	0.04061	0.08742	0.06323	0.05988	0.05928	0.11145	0.08978	0.15408	0.08273	-0.11776	-0.04547	0.00875	-0.10983	0.22807	0.23548
0.1878	0.07354	0.16763	0.04212	0.1039	0.10061	0.12813	0.0418	0.20236	0.0857	-0.10355	-0.00205	-0.05333	-0.03835	0.22443	0.22973
0.15832	0.08289	0.25214	0.09922	0.08227	0.12725	0.15109	0.07728	0.20357	0.11154	-0.14415	-0.02125	-0.11589	0.03373	0.17642	0.21966
0.15925	0.05533	0.23737	0.08787	0.07886	0.12814	0.09752	0.04306	0.14833	0.08324	-0.17716	0.05233	-0.13073	-0.07827	0.20949	0.2261
0.18506	-0.01191	0.11609	0.1012	0.05923	0.08453	0.06734	0.05825	0.14974	0.08336	-0.20653	-0.03257	0.0019	0.06538	0.20121	0.2318
0.19373	0.06427	0.14162	0.06697	0.10188	0.15556	0.09054	0.05321	0.17251	0.13085	-0.13608	-0.04299	-0.07087	0.09861	0.20262	0.24433
0.15744	0.10921	0.28397	0.10672	0.0697	0.13496	0.14764	0.05584	0.19734	0.07531	-0.20984	0.01544	-0.12567	0.01726	0.1733	0.20585
0.15678	0.12828	0.26726	0.0937	0.10861	0.171	0.14232	0.05285	0.18088	0.09276	-0.18484	0.04331	-0.10297	0.06153	0.18846	0.21805
0.15246	0.10926	0.25994	0.12676	0.09206	0.21502	0.13015	0.04753	0.20272	0.12551	-0.21303	-0.00635	-0.1118	0.06295	0.19933	0.22557
0.18373	-0.01841	0.11234	0.07571	0.05807	-0.00065	0.11872	0.06368	0.1482	0.05747	-0.11611	-0.1192	-0.01527	0.00142	0.19358	0.21774
0.1824	0.02894	0.12884	0.09591	0.0881	0.138	0.14957	0.04583	0.14346	0.14715	-0.07072	-0.0233	-0.05543	0.09101	0.20231	0.23786
0.16113	-0.01991	-0.04905	0.04925	0.06027	-0.00547	0.15242	0.01805	0.10096	0.12885	-0.08694	-0.05186	-0.0347	-0.03895	0.24639	0.20748
0.17241	0.04018	0.2269	0.07796	0.09273	0.07695	0.12541	0.06728	0.15178	0.09617	-0.12936	-0.00315	-0.12151	-0.02236	0.18825	0.21286
0.19074	0.01038	0.18115	0.08447	0.11368	0.08284	0.09262	0.08986	0.16211	0.10063	-0.21399	-0.12063	-0.02722	-0.00946	0.20332	0.21395
0.20051	-0.02931	-0.00492	0.04625	0.1142	0.01592	0.14227	-0.00767	0.11456	0.1435	-0.139	-0.16826	0.01428	-0.08747	0.23214	0.22833
0.18258	0.07913	0.22727	0.06288	0.09873	0.09065	0.17326	0.07736	0.18091	0.09506	-0.143	-0.01779	-0.16086	-0.06874	0.19419	0.23806
0.14909	0.06461	0.20879	0.09555	0.08275	0.16162	0.1261	0.04434	0.18384	0.10208	-0.11061	0.02482	-0.13188	0.00242	0.18861	0.20697

FFERENT	FFERENT	DIFFEREN	_DIFFER	Y_DIFFER	BOUND_B	OXP3_TAR	TARGETS	TARGETS	GETS_IN	GETS_IN	OXP3_TAR	ASTOMA	ASTOMA	_22_SIGN
0.18815	0.08774	0.19429	0.24437	0.12403	0.02488	0.40708	0.0388	0.04039	0.0706	-0.12125	0.03307	-0.04712	0.09176	-0.00902
0.16583	0.06648	0.19694	0.2435	0.10901	0.00566	0.35275	-0.16981	0.01162	0.07207	-0.39119	-0.01712	-0.02367	0.07959	-0.00138
0.19216	0.09031	0.17632	0.23836	0.05865	0.04011	0.38331	-0.09775	0.0315	0.0578	-0.26664	-0.02038	0.00251	0.09731	-0.03676
0.2073	0.08355	0.20523	0.24188	0.13829	0.04516	0.33604	-0.16076	0.03673	0.08535	-0.13268	0.02685	-0.04264	0.0955	-0.01155
0.20688	0.07846	0.19485	0.25323	0.10395	0.07098	0.36304	-0.01973	0.05156	0.12914	-0.21587	0.06485	-0.062	0.09222	-0.04844
0.21761	0.08191	0.21229	0.22267	0.10796	0.06314	0.33541	0.18923	0.02395	0.07963	-0.12363	-0.00508	-0.07605	0.10322	-0.02043
0.22223	0.11356	0.20516	0.23081	0.10694	0.03667	0.34321	-0.09289	0.01082	0.04961	-0.19439	-0.01656	0.01486	0.107	0.011
0.18542	0.11471	0.2037	0.22881	0.08219	0.06146	0.33747	-0.05816	0.04505	0.10123	-0.10103	0.05144	-0.05675	0.06921	-0.09063
0.20117	0.12299	0.20299	0.23403	0.09936	0.06796	0.35139	-0.08806	0.05773	0.11167	-0.05106	0.05104	0.00087	0.07822	-0.00659
0.20628	0.1215	0.20053	0.22582	0.17046	0.08156	0.36135	-0.1136	0.04627	0.11827	-0.12265	0.05637	-0.10572	0.08437	-0.06492
0.17993	0.04758	0.19409	0.22771	-0.01924	0.01237	0.40368	-0.16454	0.01564	-0.03632	-0.43106	-0.08556	-0.00221	0.10739	0.06928
0.23076	0.08285	0.20012	0.24403	0.10928	0.03847	0.41583	0.13368	0.01368	0.05077	-0.37366	-0.02406	-0.07378	0.09299	0.00315
0.13033	0.05886	0.18096	0.24511	0.02935	-0.01301	0.4262	-0.12455	0.00727	-0.09344	-0.20856	-0.10924	-0.06582	0.11504	0.0416
0.19578	0.08815	0.20106	0.24041	0.11901	0.03583	0.40023	-0.14142	0.0204	0.05749	-0.35018	0.00602	-0.04018	0.06606	-0.03557
0.16907	0.06015	0.20969	0.23821	0.08372	0.01349	0.35349	-0.13694	0.0101	0.05288	-0.38158	-0.05847	-0.02408	0.10542	0.02379
0.13947	0.01784	0.18202	0.23746	0.03913	-0.01105	0.39759	-0.17408	-0.00418	-0.09366	-0.31452	-0.06251	0.00574	0.08731	0.01946
0.21121	0.11517	0.20489	0.25774	0.1022	0.03487	0.39121	-0.15064	0.01396	0.05639	-0.3454	-0.00564	-0.02239	0.06827	-0.01101
0.20221	0.09378	0.20565	0.23403	0.101	0.05503	0.38224	-0.08853	0.04714	0.11511	-0.22194	0.02217	-0.04535	0.08041	-0.10317

_22_SIGN	ONSE_TO	ONSE_TO	TO_AZACI	TO_AZA	LUNG_CA	LUNG_CA	LUNG_CA	GENES_IN	GENES_IN	GENES_IN	ORY_RES	ORY_RES	ORY_RES	ORY_RES	ORY_RES
0.14071	0.10505	0.2748	0.12516	0.0874	0.33087	0.31252	0.27473	0.06654	0.04456	0.08391	-0.12987	0.05738	-0.08921	0.07016	0.01288
0.03428	0.04784	0.06203	0.15061	0.07555	0.32092	0.29843	0.26879	0.10543	-0.23426	0.07356	-0.08445	0.03111	-0.07851	0.05366	0.02066
0.11262	0.11503	0.07336	0.11533	0.07071	0.3391	0.30311	0.27185	0.12512	-0.0041	0.13686	-0.09482	0.04084	-0.06114	0.0374	0.03455
0.14785	0.0706	0.08611	0.10822	0.1003	0.34566	0.31482	0.27838	0.09345	-0.04915	0.09064	-0.0962	0.04221	-0.06467	0.03644	0.0176
0.11311	0.00223	0.06024	0.13507	0.09923	0.33603	0.31486	0.28221	0.09142	-0.00977	0.09478	-0.11753	0.05413	-0.0843	0.03692	0.01237
0.10551	0.11033	0.01891	0.12632	0.07381	0.35699	0.31944	0.27995	0.13326	0.05934	0.12213	-0.1184	0.03264	-0.09336	0.04232	0.01201
0.13806	0.02481	0.02824	0.10825	0.05632	0.35604	0.33078	0.30347	0.13112	-0.10635	0.09707	-0.09994	0.0404	-0.07037	0.02281	0.0437
0.14015	0.04852	0.1281	0.10582	0.11153	0.35314	0.32279	0.28186	0.06865	0.0018	0.02725	-0.07751	0.04852	-0.0678	0.04327	0.0067
0.15662	0.0264	0.05425	0.11316	0.09911	0.35502	0.32635	0.28849	0.06571	-0.0531	0.0419	-0.0826	0.07125	-0.07059	0.0586	0.05539
0.16429	0.07835	0.08067	0.13208	0.06803	0.35122	0.31868	0.28355	0.0832	-0.16909	0.06332	-0.11077	0.03655	-0.0845	0.03442	0.04177
0.15249	0.0325	0.05167	0.10535	0.03305	0.34489	0.31814	0.28063	0.13525	0.08074	0.13844	-0.11138	0.07001	-0.0885	0.03568	0.00546
0.1053	0.08949	0.01924	0.12513	0.04868	0.35003	0.32698	0.28434	0.12827	0.09754	0.12444	-0.12997	0.04961	-0.10152	0.04151	0.02106
0.07805	0.09372	0.08803	0.12732	0.05448	0.35064	0.32181	0.29957	0.11481	0.05133	0.11887	-0.10813	0.03314	-0.06973	0.04875	0.00264
0.1083	0.00606	0.03212	0.1175	0.09619	0.33893	0.31326	0.27653	0.06099	-0.16922	0.04849	-0.09545	0.03939	-0.05599	0.03087	0.00516
0.15344	0.06073	0.09573	0.11842	0.07875	0.34515	0.31413	0.28482	0.11524	0.09344	0.12063	-0.1123	0.04848	-0.09045	0.04906	0.00517
0.03847	0.04997	-0.00937	0.15289	0.04322	0.32004	0.30802	0.29309	0.12418	0.0214	0.14388	-0.07634	0.0428	-0.05873	0.03966	0.00725
0.12675	0.05082	0.05136	0.11222	0.08361	0.34143	0.32309	0.29539	0.04801	-0.20592	0.02189	-0.1024	0.04529	-0.07239	0.04384	0.00236
0.13269	-0.06389	0.08841	0.1203	0.09487	0.33109	0.31399	0.28862	0.06567	-0.08161	0.05263	-0.08049	0.03561	-0.06868	0.03161	0.02982

_ENDOC	F_SIGNAL	IF_SIGNA	CMV_24_H	CMV_24_H	MV_8_H	CMV_8_H	CMV_ALL	CMV_AL	KIL_TARG	KIL_TARG	NOIN_RE	NOIN_RE
-0.49956	0.29022	0.32164	0.06475	0.23751	0.12943	0.07118	0.08351	0.20146	-0.0627	-0.04482	0.48117	-0.26324
-0.49974	0.27949	0.31323	0.05375	0.22429	0.08968	0.0992	0.0683	0.19771	-0.01187	-0.04727	0.48248	-0.3158
-0.3833	0.27718	0.28985	0.03745	0.24593	0.12696	0.16178	0.06664	0.22497	0.19265	-0.03364	0.48371	-0.26456
-0.14339	0.27395	0.30251	0.08794	0.2425	0.14313	0.16278	0.11709	0.22198	0.04153	0.00786	0.48609	-0.19421
-0.49991	0.27591	0.29818	0.01145	0.24218	0.12307	0.13159	0.05326	0.21505	0.12228	-0.02329	0.48039	-0.17767
-0.49815	0.25965	0.28709	0.10479	0.23735	0.11172	0.11942	0.12459	0.203	-0.01232	-0.0006	0.4795	-0.24359
-0.49991	0.26682	0.27924	0.12263	0.2514	0.13075	0.15331	0.14448	0.23098	0.07337	0.04173	0.47748	-0.21121
-0.16822	0.26026	0.29106	0.09985	0.23268	0.15418	0.13447	0.12555	0.21231	0.15516	0.00555	0.48503	-0.23354
-0.49938	0.26514	0.2769	0.07818	0.23187	0.14186	0.17828	0.10766	0.22575	0.03246	0.05179	0.48539	-0.2442
-0.11802	0.25319	0.28182	0.04769	0.25398	0.1468	0.19918	0.09516	0.23625	0.02383	0.01943	0.48162	-0.35325
-0.49991	0.29936	0.29966	0.03798	0.23006	0.08605	0.06176	0.062	0.19333	0.13452	-0.00859	0.4774	-0.27395
-0.49991	0.26876	0.29747	0.11329	0.26741	0.10367	0.15207	0.12019	0.23688	0.15585	0.03407	0.48301	-0.23256
-0.49974	0.28617	0.27243	0.05066	0.23449	0.10638	0.08357	0.07186	0.19169	0.04089	-0.05741	0.48266	-0.0489
-0.4298	0.26224	0.31252	0.06902	0.23916	0.13492	0.11231	0.09629	0.21278	0.18095	-0.0307	0.48503	-0.23833
-0.43579	0.28719	0.30081	0.08017	0.24662	0.09741	0.10812	0.09639	0.21478	0.0645	-0.02107	0.47889	-0.28014
-0.50009	0.28896	0.29117	0.05684	0.25607	0.04313	0.11033	0.05406	0.2194	0.05194	-0.044	0.47992	-0.13556
-0.29822	0.27196	0.28851	0.05722	0.22609	0.13088	0.0853	0.08833	0.1982	0.11739	-0.0485	0.48626	-0.22645
-0.21385	0.29216	0.29934	0.05639	0.25597	0.12187	0.18094	0.08137	0.23372	0.12271	-0.05058	0.48504	-0.1982

ANSIENT	LN_BY_2	NEGF_INTE	EGF_INTE	F_PERSIS	F_PERSIS	UP_BY_2	ECEPTOR	PASE_CA	AS_SIGNA	AND_S_P	AND_M_P	TOKINE_C	AMMED_C	TEN_PATH	ADE_OF_	KA_RECE	SIGNALIN	O40PATHW	CHEMOT	TOR_IN_B	PATHW	ALING_IN_B	NG_IN_C	TIN_CYTOT
-0.03605	-0.01545	-0.04149	0.08469	-0.00815	0.09852	0.01806	0.08409	-0.07696	0.1263	0.08255	0.099	0.03207	-0.07625	0.04471	-0.1449	0.04682	0.0262	0.01329	0.15106	-0.07043	0.09499	0.00973	0.02571	0.27471
-0.0231	0.01879	0.00918	0.0241	-0.03951	0.07402	0.02843	0.03024	-0.06473	0.13098	0.11926	0.18337	0.01618	-0.08139	0.03424	-0.05013	0.00367	-0.01897	-0.02371	0.06758	-0.08737	0.07864	-0.05048	0.01548	0.25069
-0.0141	0.02513	-0.03801	0.07222	-0.00683	0.10856	0.03133	0.08948	-0.03893	0.1122	0.11828	0.24414	0.08701	-0.04422	0.06185	-0.06524	0.07201	0.02456	-0.0105	0.14958	-0.02434	0.09629	0.00742	0.03274	0.28391
-0.01314	-0.00186	-0.0299	0.09321	-0.03448	0.07558	0.02631	0.1063	-0.05487	0.15274	0.04436	0.02308	0.10879	0.01892	0.0467	-0.25291	0.07737	0.08653	0.02769	0.13826	-0.00657	0.11735	0.06402	0.02285	0.23859
-0.05593	0.01883	-0.02798	0.08629	-0.06523	0.05743	0.03709	0.10375	-0.01439	0.17819	0.07241	0.11626	0.11926	-0.01384	0.04047	-0.17628	0.09357	0.08928	0.02281	0.12873	-0.02388	0.10554	0.05353	0.02117	0.264
-0.05369	0.02125	-0.0446	0.01551	-0.08646	0.06428	0.02114	0.02027	-0.02788	0.1465	0.08343	0.10164	0.01775	-0.07857	-0.00948	-0.13919	0.05276	-0.00794	-0.07759	0.11973	-0.09756	0.05557	-0.02871	0.01474	0.26661
-0.02796	0.05223	-0.03263	0.04325	-0.03918	0.09566	0.03545	0.06421	-0.0169	0.14361	0.08297	0.17738	0.13921	-0.03592	0.03875	-0.10911	0.0855	0.04935	0.03399	0.14997	-0.01656	0.08712	-0.0282	0.03857	0.25889
-0.03908	0.00617	-0.04373	0.03264	-0.05175	0.09428	0.04787	0.12006	-0.08023	0.14035	0.04242	-0.23657	0.18615	0.01085	0.04447	-0.26821	0.08519	0.09475	0.04173	0.14624	-0.00431	0.12568	0.05605	0.02497	0.27702
-0.03142	0.00126	-0.02382	0.05685	-0.05941	0.09492	0.0492	0.11549	0.01228	0.16945	0.0454	-0.0805	0.19928	0.03464	0.06662	-0.23532	0.09275	0.10039	0.05084	0.13869	0.00553	0.11233	0.06607	0.0183	0.27708
-0.04044	0.02534	-0.02902	0.0641	-0.05441	0.09658	0.04233	0.13086	0.02762	0.17009	0.07349	-0.2038	0.1859	0.05245	0.05725	-0.17542	0.09216	0.09328	0.05577	0.13228	-0.02641	0.10349	0.07497	0.02065	0.25078
-0.02179	0.00569	-0.03163	0.10038	-0.02686	0.08296	0.00848	-0.01071	-0.03528	0.14781	0.17371	0.22459	-0.03856	0.00613	-0.0066	0.07511	-0.01105	-0.02482	-0.0307	0.15777	-0.05037	0.10802	-0.05853	0.05337	0.28908
-0.0144	-0.00376	-0.0339	0.09335	-0.00635	0.10992	0.0144	0.03532	-0.06539	0.135	0.11408	0.16488	-0.02803	-0.05089	0.05951	-0.08105	0.03394	0.02355	-0.00276	0.15141	-0.07502	0.1103	0.02109	0.03286	0.26855
-0.02098	0.00044	-0.00656	0.04451	-0.04381	0.11137	-0.00262	0.05263	-0.04753	0.14973	0.15627	0.25912	-0.32601	-0.02098	0.06532	0.0173	0.07776	-0.01364	-0.0393	0.16285	0.00595	0.14813	-0.07777	0.09302	0.29585
-0.01356	0.01372	-0.03936	0.0566	-0.03222	0.1103	0.03057	0.06238	-0.02815	0.12088	0.04197	-0.2459	0.10438	-0.06327	0.05177	-0.2235	0.06799	0.0339	0.00561	0.13186	-0.04429	0.10379	-0.00133	0.03156	0.27525
-0.02545	0.02402	-0.03466	0.09672	-0.05005	0.11678	0.03603	0.09249	-0.06183	0.14186	0.07236	-0.02026	0.07085	-0.0478	0.05606	-0.13881	0.0576	0.00732	-0.01929	0.14106	-0.0513	0.10056	-0.01681	0.03694	0.30158
-0.00695	0.02505	-0.01278	0.032	-0.04209	0.08818	-0.0151	-0.01882	-0.06449	0.12091	0.02729	0.3264	-0.25976	0.02345	0.044	-0.30881	0.04992	-0.04854	-0.02337	0.1236	-0.05637	0.08228	-0.17301	0.05165	0.2548
-0.01934	-0.00503	-0.00911	0.03212	-0.06636	0.02713	0.02608	0.06704	-0.07642	0.14233	0.10539	-0.04069	0.09886	-0.09554	0.05007	-0.10977	0.071	0.05733	-0.01633	0.14669	-0.00165	0.12595	0.04038	0.02943	0.26827
-0.04453	0.01266	-0.02978	0.05737	-0.03529	0.11086	0.04687	0.09147	-0.0293	0.15683	0.03943	-0.0347	0.14373	0.02822	0.05774	-0.2754	0.08661	0.09693	0.04895	0.13381	0.00696	0.1159	0.03487	0.01185	0.25196

SIGNAL	TA_41BB_	TA_ACE2_	ETAMINO	TA_ACH_P	ACTINY	AGPCR	TA_AGR_P	TA_AHSP_	AKAP13_	AKAP95_	CENTRO	TA_AKT_P	TA_ALK_P	TERNATI	TA_AMAN_	TA_AMI_P	A_ARAP_	ARENRF2	TA_ARF_P	ASBCELL	TA_AT1R_	TA_ATM_P	ATRBRC	TA_BAD_P
0.08707	-0.00011	-0.33328	0.00336	-0.01477	0.22449	0.11647	0.09635	0.21141	0.11997	0.11911	0.09767	0.03252	-0.18102	-0.0642	0.04496	0.14226	0.02877	0.15084	-0.00192	0.00219	0.17915	-0.04229	-0.17227	0.1303
0.13449	-0.04325	-0.29101	0.12289	0.02328	0.14389	0.12866	0.10563	0.22651	0.16053	0.15229	0.10448	0.02367	-0.10427	-0.03734	0.01827	0.10559	-0.01546	0.12654	-0.03012	0.047	0.13955	-0.077	-0.16886	0.14704
0.16274	-0.04809	-0.28344	0.07554	0.08684	0.16451	0.07337	0.12312	0.23656	0.05868	0.10417	0.06265	0.04355	-0.18711	-0.03435	0.07091	0.16699	0.02403	0.14396	0.08485	0.04416	0.15752	-0.01373	-0.12846	0.14089
0.11421	-0.02658	-0.06142	-0.09205	0.08249	0.10915	0.14884	0.10647	0.21387	0.13843	0.10328	0.06942	0.08166	-0.21445	0.09481	0.07625	0.19076	0.05314	0.18154	0.02207	0.09241	0.16493	-0.02896	-0.12436	0.17695
0.08014	0.00932	-0.13495	0.11118	0.06388	0.11714	0.18806	0.12929	0.25903	0.15005	0.1278	0.08193	0.05677	-0.22891	0.02256	0.04853	0.19211	0.0456	0.17568	-0.03503	0.13229	0.17056	-0.05114	-0.1889	0.17431
0.11189	-0.02779	0.03882	0.05399	0.03203	0.11823	0.148	0.07386	0.28546	0.12966	0.12957	0.05782	0.00437	-0.19815	0.25799	0.0448	0.24123	0.00968	0.1231	-0.03629	0.04444	0.16307	-0.03337	-0.14676	0.10552
0.11721	0.01819	-0.04655	0.11976	0.09736	0.19631	0.16343	0.13176	0.17575	0.10942	0.11267	0.06077	0.05715	-0.17684	0.25708	0.07377	0.24482	0.00512	0.12702	0.05435	0.13216	0.16903	0.02359	-0.08001	0.16751
0.08781	0.0237	-0.09324	-0.01548	0.11083	0.11755	0.19501	0.12748	0.23426	0.14868	0.08161	0.05069	0.09547	-0.22665	0.1197	0.14617	0.21626	0.06594	0.18097	-0.02967	0.16727	0.17145	-0.03381	-0.15999	0.20604
0.09372	-0.0152	0.11797	0.10386	0.12803	0.11118	0.16738	0.11588	0.21533	0.13909	0.09144	0.07094	0.07896	-0.23393	0.15023	0.1156	0.25314	0.0487	0.16079	6.6E-05	0.24866	0.16485	-0.04278	-0.15175	0.19359
0.09275	0.02419	-0.06622	-0.06543	0.08718	0.11436	0.19838	0.11073	0.21672	0.17536	0.09802	0.07036	0.06516	-0.27527	0.16449	0.06657	0.21898	0.03548	0.17539	-0.05675	0.14012	0.18917	-0.00385	-0.12157	0.1847
0.1338	-0.08568	-0.18149	0.14325	0.02573	0.15413	0.08608	0.11152	0.20128	0.06788	0.09267	0.07526	0.02811	-0.11751	0.07626	-0.06901	0.17819	0.05162	0.12994	0.12015	0.10528	0.15256	-0.04397	-0.13789	0.13393
0.11773	-0.06506	-0.16256	-0.34121	0.00618	0.16852	0.13749	0.10782	0.211	0.11865	0.11349	0.09122	0.01363	-0.22315	0.18736	0.02939	0.24574	-0.0312	0.10804	0.05482	0.13109	0.18011	-0.03257	-0.13276	0.17429
0.19072	-0.10529	-0.18608	0.17126	0.02507	0.24352	0.12484	0.11947	0.19925	0.10519	0.12885	0.07158	0.02014	-0.04221	0.07371	-0.0452	0.17034	0.04286	0.08863	0.03652	0.00307	0.16145	-0.02033	-0.0707	0.16094
0.11005	-0.01735	-0.24442	0.12689	0.02968	0.15941	0.16066	0.08761	0.22322	0.12825	0.09532	0.07417	0.05639	-0.20025	0.0685	0.07448	0.17156	0.05233	0.15415	0.00132	0.14968	0.14659	-0.0428	-0.14896	0.1521
0.10506	-0.00427	-0.18126	-0.05882	0.03933	0.14904	0.14159	0.11087	0.19906	0.11888	0.09667	0.06205	0.0118	-0.1779	0.07531	0.01738	0.20485	-0.01329	0.1134	0.00301	0.18401	0.18951	-0.01697	-0.119	0.14163
0.16393	-0.07174	-0.30046	0.13213	0.02511	0.23094	0.08163	0.16322	0.17602	0.08975	0.13344	0.07205	-0.00418	-0.09461	-0.10844	-0.02567	0.12205	0.03501	0.09896	0.00295	0.12124	0.16442	0.07914	0.02297	0.13031
0.11877	-0.03655	-0.11535	-0.07423	-0.0069	0.16586	0.14847	0.10328	0.21975	0.11504	0.11501	0.07582	0.05749	-0.2178	-0.01636	0.10313	0.16498	0.05009	0.14921	0.13235	0.13203	0.14507	-0.02738	-0.13079	0.16783
0.1104	0.00229	-0.23119	0.04097	0.1486	0.09561	0.16766	0.12629	0.20136	0.1628	0.09911	0.10187	0.09591	-0.18762	0.02696	0.08404	0.1549	0.06899	0.14637	0.01563	0.16643	0.1489	0.02701	-0.09494	0.20852

ARRESTI	RRESTIN_	ARR_MAR	BBCELL	ELLSURV	TA_BCR_P	IOPEPTID	YMPHOC	BOTULIN	A_BTG2_	A_CACAM_	LCINEU	ARDIACE	A_CARM1_	CARM_ER	CASPASE	A_CB1R_	TA_CBL_P	A_CCR3_	A_CCR5_	A_CD40_	A_CDC25_	CDC42RA	A_CDK5_	CDMAC_
0.1165	0.12122	0.19119	0.00219	0.04125	0.12331	0.19921	0.14066	0.36445	-0.15222	0.26709	0.09583	-0.05166	0.21838	-0.02351	0.06622	0.07815	0.05016	0.2037	0.15151	-0.03135	0.07867	0.292	0.23597	0.15693
0.07321	0.02271	0.08544	0.047	0.05485	0.07253	0.18615	0.1329	0.35318	-0.05441	0.29498	0.08981	-0.08021	0.22877	0.05739	0.06482	0.09091	0.00534	0.13857	0.16967	-0.0103	0.1023	0.17261	0.22368	0.06014
0.15535	0.09804	0.16591	0.04416	0.09209	0.10867	0.17344	0.15553	0.34825	-0.02962	0.2851	0.07018	-0.06644	0.15488	0.07003	0.09366	0.09432	0.07634	0.20268	0.14149	-0.05031	0.09617	0.27664	0.19882	0.13417
0.20114	0.16351	0.18115	0.09241	0.09159	0.14762	0.21252	0.21556	0.39281	-0.08096	0.26663	0.12341	0.00397	0.15844	-0.00423	0.09224	0.14015	0.10354	0.21811	0.14144	-0.04031	0.0623	0.25035	0.19961	0.13146
0.19036	0.16941	0.20813	0.13229	0.05462	0.1679	0.21425	0.21878	0.36868	-0.0572	0.29179	0.14108	0.0075	0.22721	0.01504	0.12103	-0.03193	0.1559	0.19305	0.14665	-0.00639	0.08329	0.21835	0.21319	0.16944
0.13194	0.04703	0.09972	0.04444	0.02441	0.09984	0.24415	0.14873	0.33081	-0.01115	0.28395	0.1064	0.06815	0.19087	0.02258	0.10117	0.08504	0.08799	0.14057	0.18545	-0.14347	0.06708	0.18672	0.17889	0.09162
0.17219	0.13408	0.19086	0.13216	0.09862	0.10678	0.21706	0.20069	0.26651	-0.04847	0.26052	0.07169	0.0283	0.1754	0.02161	0.12185	0.02735	0.12216	0.19725	0.13411	-0.01856	0.06054	0.32946	0.20375	0.16446
0.22126	0.18363	0.20823	0.16727	0.05553	0.16894	0.22533	0.26461	0.35688	-0.07865	0.28679	0.13264	0.02645	0.23209	-0.01626	0.08829	0.09229	0.12573	0.2181	0.13414	0.01358	0.00967	0.2545	0.21704	0.16547
0.2087	0.17466	0.18457	0.24866	0.0981	0.16714	0.22717	0.31762	0.34014	-0.09424	0.29918	0.11755	0.01807	0.19949	-0.00593	0.13012	0.07715	0.13624	0.19437	0.12656	-0.0172	0.03496	0.26556	0.18473	0.1283
0.19273	0.16303	0.19507	0.14012	0.06027	0.16853	0.24	0.23093	0.34754	-0.0835	0.25819	0.12985	0.06635	0.23544	-0.01426	0.14533	0.08785	0.15868	0.21586	0.15791	0.0355	0.02258	0.21551	0.20603	0.17898
0.15124	0.06936	0.12943	0.10528	0.0615	0.05282	0.16268	0.14917	0.26968	-0.07527	0.28045	0.04556	0.00014	0.13163	0.04465	0.0672	-0.00554	0.03692	0.21868	0.14623	-0.11915	0.09398	0.24962	0.18488	0.08504
0.11656	0.07694	0.14744	0.13109	0.02619	0.08776	0.23549	0.21188	0.26299	-0.0676	0.28506	0.09184	-0.00326	0.23148	0.02578	0.07372	-0.05438	0.09559	0.22543	0.19151	-0.07909	0.11687	0.25035	0.2682	0.11457
0.15651	0.0781	0.15945	0.00307	0.06441	0.03701	0.1795	0.02778	0.40585	0.00898	0.18865	-0.00247	-0.07701	0.20527	0.06695	0.05592	0.15149	-0.00328	0.22624	0.08362	-0.09898	0.13128	0.2532	0.20748	0.08534
0.20328	0.13675	0.1763	0.14968	0.05964	0.10504	0.1953	0.2282	0.32429	-0.08456	0.28096	0.07102	-0.06537	0.18446	0.0052	0.10162	-0.00248	0.06275	0.20104	0.13674	-0.00367	-0.04158	0.22759	0.21582	0.10401
0.13657	0.13505	0.19187	0.18401	0.00981	0.10623	0.21435	0.23066	0.31161	-0.10245	0.25092	0.06258	-0.03336	0.18779	0.00911	0.08464	-0.10729	0.07349	0.24629	0.14273	-0.05106	0.02584	0.2397	0.25769	0.13955
0.12156	0.0854	0.15877	0.12124	0.0563	0.04277	0.17992	0.0958	0.29523	-0.02414	0.275	0.0214	-0.04359	0.16531	0.02015	0.02697	-0.0114	0.02595	0.20958	0.13405	-0.0781	0.13096	0.22651	0.27905	0.10469
0.19658	0.11912	0.16601	0.13203	0.10662	0.09231	0.18623	0.23525	0.33292	-0.01813	0.22144	0.07005	-0.01992	0.19853	0.01591	0.08067	0.04582	0.0823	0.2227	0.10992	-0.11938	0.02599	0.29446	0.22888	0.10815
0.20834	0.1497	0.19812	0.16643	0.07585	0.15752	0.19934	0.2438	0.38911	-0.09212	0.30546	0.12771	-0.00699	0.19094	-0.01202	0.11501	0.1299	0.12789	0.18786	0.1197	-0.00252	0.04253	0.25812	0.21352	0.1615

ELL2CEL	ELLCYCL	CERAMID	A_CFTR_	CHEMICA	A_CHREBP	TA_CK1_P	CLASSIC	A_COMP_	A_CPSF_	A_CREB_	A_CREM_	TA_CSK_P	A_CTBP1_	A_CTCF_	A_CTLA4_	TA_CTL_P	A_CXCR4_	CYTOKIN	A_D4GDI_	TA_DC_PA	A_DEATH_	A_DICER_	AFRAGM	A_DREAM_
0.24885	-0.12149	0.08352	0.27136	0.02881	0.11147	0.16371	-0.03764	-0.02184	0.054	0.157	0.35476	0.06928	0.3512	-0.13663	0.02269	0.0887	0.11152	0.22623	0.03901	-0.07588	-0.02422	-0.01332	0.12239	0.18189
0.24	-0.04379	0.04517	0.28667	0.04738	0.14301	0.22621	-0.06	-0.05898	0.13127	0.14599	0.37819	0.05792	0.36278	-0.07393	-0.00028	0.04368	0.07084	0.11331	0.00739	-0.11258	-0.04752	0.05956	0.26336	0.2018
0.31055	-0.02524	0.0978	0.20581	0.08878	0.09559	0.13333	0.08565	0.07866	0.03642	0.14752	0.36058	0.04876	0.39793	-0.0717	0.04293	0.10428	0.08799	0.18526	0.07655	-0.03857	0.00214	0.03734	0.22165	0.13193
0.30547	-0.21199	0.08792	0.26139	0.08535	0.11593	0.1587	0.20042	0.18837	0.00562	0.18726	0.28272	0.10726	0.31268	-0.09138	0.08738	0.08614	0.10735	0.26157	0.06909	0.10467	-0.00724	-0.01991	0.11735	0.1605
0.27238	-0.11691	0.13844	0.29196	0.10375	0.14753	0.17875	0.09497	0.09148	0.07152	0.19233	0.32887	0.19402	0.30147	-0.12389	0.15087	0.15285	0.09482	0.24339	0.12162	-0.00217	0.02842	-0.05569	0.19084	0.20345
0.24676	-0.11021	0.07497	0.25452	0.08862	0.16942	0.21208	0.34137	0.33359	0.11767	0.12978	0.33098	0.172	0.30708	-0.13748	0.13424	0.1798	0.07084	0.14327	0.0762	-0.23817	0.00729	-0.08094	0.20549	0.12599
0.26644	-0.07802	0.11174	0.23618	0.10823	0.1124	0.15898	0.30098	0.30009	0.00216	0.17576	0.32341	0.17686	0.35909	-0.06963	0.15777	0.14359	0.1205	0.22318	0.08606	-0.02715	0.03393	-0.15353	0.19362	0.15495
0.2395	-0.27334	0.11461	0.28702	0.09502	0.13066	0.18772	0.20349	0.19175	0.00779	0.20634	0.26968	0.14902	0.27973	-0.13146	0.11718	0.1099	0.1245	0.27234	0.0598	0.09094	-0.0015	-0.04805	0.1198	0.19489
0.28066	-0.22438	0.10589	0.26206	0.1281	0.13513	0.16931	0.22657	0.21458	0.01746	0.20016	0.28272	0.18356	0.30413	-0.1028	0.16919	0.15749	0.10172	0.30425	0.13143	0.08629	0.02352	-0.08294	0.14442	0.1634
0.2424	-0.20887	0.12744	0.29635	0.14509	0.1552	0.18487	0.23287	0.21956	-0.0006	0.20467	0.29822	0.20496	0.33394	-0.13916	0.15481	0.21029	0.12004	0.24913	0.15204	0.10821	0.04422	-0.06644	0.16901	0.19693
0.28115	0.05791	0.05511	0.20805	0.05082	0.08808	0.14146	0.10098	0.10215	0.05743	0.12558	0.35829	0.07987	0.37221	-0.01375	0.05919	0.07388	0.10871	0.1029	0.0233	-0.15206	-0.03682	-0.02381	0.21564	0.13683
0.24698	-0.06181	0.07853	0.28052	0.05956	0.12432	0.24104	0.24246	0.23165	0.0441	0.17697	0.3685	0.16451	0.36424	-0.10587	0.12293	0.17143	0.09605	0.26756	0.05145	-0.06238	-0.04043	-0.07332	0.13665	0.20926
0.27812	0.02406	0.11079	0.25599	0.0668	0.16748	0.19146	0.07094	0.06988	0.06549	0.12804	0.34314	-0.02463	0.38429	-0.03025	-0.03861	-0.09587	0.07557	0.08624	-0.00255	-0.17719	0.00375	0.02775	0.20455	0.17778
0.285	-0.25945	0.0919	0.28373	0.05753	0.11961	0.20141	0.13964	0.12899	0.01944	0.1577	0.31161	0.13955	0.31587	-0.12091	0.07816	0.08532	0.0683	0.17574	0.08047	-0.01609	-0.02335	-0.02791	0.1653	0.17825
0.2712	-0.14699	0.09796	0.27203	0.0776	0.15738	0.18205	0.09212	0.09667	0.03188	0.15003	0.31214	0.13831	0.31698	-0.16108	0.12241	0.03515	0.09692	0.19072	0.05594	-0.05225	-0.00014	-0.0774	0.15803	0.18119
0.28253	-0.12754	0.07864	0.23601	0.07696	0.10582	0.22198	-0.16399	-0.13873	0.09579	0.12119	0.37079	0.0495	0.36754	-0.12532	0.03545	0.00663	0.06373	-0.03075	-0.05775	-0.16756	-0.01418	-0.00831	0.18094	0.16754
0.25588	-0.13377	0.06806	0.26776	0.0592	0.15373	0.21332	0.05489	0.04606	0.02842	0.17424	0.28395	0.13205	0.35214	-0.05014	0.1287	0.06178	0.09779	0.18904	0.04234	-0.06359	-0.02751	0.00254	0.148	0.17343
0.30416	-0.23381	0.12402	0.27155	0.10597	0.11154	0.1722	0.1321	0.12376	0.0208	0.2083	0.27638	0.16828	0.33157	-0.11775	0.15103	0.15006	0.1146	0.28013	0.10362	0.04626	0.00666	-0.10762	0.14328	0.19116

TA_DSP_P	TA_ECM_P	A_EDG1_	A_EEA_	TA_EFP_P	PGFR_SM	TA_EGF_P	ICOSANO	A_EIF2_P	A_EIF4_P	TA_EIF_P	OSINOPH	A_EPHA4_	EPONFK	TA_EPO_P	TA_ERAD_	A_ERBB3_	ERBB4_	A_ERK5_	TA_ERK_P	ERYTH_	TA_ETC_P	TA_ETS_P	EXTRINS	TA_FAS_P
0.31777	0.26515	0.14718	0.24136	-0.03594	0.05824	0.05445	0.15677	0.20396	0.13205	0.29904	0.15616	0.26647	0.13272	0.07579	0.20561	0.02956	-0.14419	0.09007	0.19161	-0.42829	0.35594	-0.16781	0.17988	0.11594
0.2079	0.20702	0.10355	0.16005	0.06415	-0.02555	0.01323	0.14437	0.19132	0.1232	0.31177	0.14955	0.25909	0.03775	0.04688	0.24294	-0.02438	-0.24844	0.09032	0.13439	-0.41552	0.34215	-0.01911	0.17935	0.12377
0.25618	0.28516	0.19279	0.16462	-0.0025	0.02	0.05198	0.25032	0.21642	0.14265	0.29492	0.17265	0.29329	0.10728	0.06446	0.21995	-0.11934	-0.24432	0.10955	0.16149	-0.32776	0.33586	-0.11936	0.27007	0.14424
0.29293	0.27668	0.20237	0.20093	0.00463	0.04574	0.08501	0.20649	0.20593	0.15311	0.29644	0.23335	0.36232	0.06433	0.07763	0.22386	-0.08527	-0.0696	0.1193	0.16571	-0.36754	0.29935	-0.14032	0.24855	0.10473
0.28841	0.26144	0.16231	0.15481	-0.05895	0.04406	0.07027	0.13369	0.17015	0.14001	0.29099	0.28947	0.24667	0.1312	0.0896	0.21903	-0.09885	-0.17441	0.09744	0.16126	-0.24887	0.29556	-0.17531	0.22764	0.13677
0.07308	0.1983	0.10428	0.20002	0.03129	-0.02215	0.03931	0.16445	0.22535	0.09637	0.30818	0.18092	0.1578	0.11339	0.05566	0.22619	-0.10394	-0.1456	0.04545	0.11738	-0.32162	0.34261	-0.12992	0.30948	0.13988
0.26882	0.26812	0.20152	0.10802	-0.01785	0.0253	0.07173	0.21937	0.17347	0.15806	0.2979	0.23734	0.22655	0.13594	0.06552	0.20545	-0.12047	-0.12868	0.10017	0.15432	-0.28835	0.37064	-0.1495	0.31083	0.14457
0.30423	0.28739	0.20943	0.20515	-0.0472	0.05123	0.0926	0.17092	0.19933	0.14905	0.29084	0.28255	0.3298	0.12739	0.08325	0.21435	-0.02122	-0.11881	0.12152	0.16866	-0.29007	0.30855	-0.20116	0.28477	0.09597
0.28001	0.27425	0.21193	0.15671	-0.10923	0.0383	0.09397	0.17355	0.19729	0.15113	0.2867	0.38068	0.33374	0.09906	0.08204	0.22147	-0.07866	-0.17005	0.13401	0.15509	-0.01955	0.31289	-0.20353	0.33282	0.12628
0.29823	0.25187	0.18381	0.13654	-0.11102	0.06457	0.08602	0.15639	0.19545	0.13073	0.27536	0.21883	0.30453	0.14531	0.10182	0.19524	-0.01761	-0.06774	0.10256	0.16252	-0.23119	0.32886	-0.16598	0.27368	0.12259
0.22884	0.27092	0.13225	0.22935	0.13584	0.07432	0.04766	0.08637	0.20644	0.1161	0.28399	0.23906	0.09072	0.07776	0.02195	0.21604	0.15285	-0.37663	0.07701	0.15574	-0.43299	0.3652	-0.10221	0.23285	0.14257
0.29597	0.25591	0.16681	0.17885	0.11635	0.04263	0.04177	0.02392	0.24407	0.13535	0.32192	0.28747	0.22139	0.08501	0.04041	0.22102	0.01006	-0.25958	0.07717	0.15446	-0.37982	0.36033	-0.14724	0.32024	0.11769
0.22235	0.28075	0.18607	0.19196	0.11475	-0.06479	0.04018	0.02426	0.21414	0.12592	0.31618	0.07314	0.10526	-0.05099	0.06235	0.22993	-0.19043	-0.32982	0.12519	0.20921	-0.44433	0.34659	-0.09785	0.21592	0.13125
0.27565	0.24912	0.17188	0.16491	-0.01075	-0.00654	0.03129	0.252	0.19851	0.12324	0.2942	0.28319	0.2565	0.10951	0.06613	0.22917	-0.07736	-0.23811	0.07599	0.15483	-0.34181	0.35885	-0.1526	0.2855	0.11296
0.30302	0.25669	0.16335	0.22296	0.04688	0.03156	0.03701	0.20183	0.23347	0.12265	0.31879	0.35219	0.15735	0.10526	0.07973	0.1978	-0.06662	-0.19085	0.07573	0.1636	-0.39451	0.34155	-0.19296	0.27645	0.12941
0.26647	0.24355	0.13321	0.23763	0.14641	0.06877	0.03312	0.0628	0.25362	0.12396	0.31236	0.23959	0.14391	0.06868	0.02755	0.20936	0.08064	-0.29864	0.05482	0.18185	-0.46036	0.39238	-0.10704	0.17259	0.12767
0.24695	0.27127	0.19097	0.18355	0.01114	0.01739	0.06426	0.19476	0.178	0.1464	0.29421	0.29806	0.32278	0.07553	0.05515	0.23852	-0.07159	-0.1874	0.1086	0.16489	-0.41592	0.37652	-0.0881	0.2509	0.10584
0.29781	0.28526	0.23657	0.16611	-0.06067	0.02872	0.0874	0.19993	0.19074	0.15729	0.28435	0.28929	0.26334	0.13621	0.07638	0.20872	-0.04416	-0.18668	0.14655	0.15577	-0.32837	0.31945	-0.1896	0.23103	0.11626

A_FBW7_	A_FCER1_	FEEDER	BRINOLY	LUMAZEN	A_FMLP_	A_FOSB_	A_FREE_	TA_FXR_	TA_G1_P	TA_G2_P	A_GABA_	A_GATA3_	TA_GCR_P	GHRELIN	TA_GH_P	GLEEVEC	A_GPCR_	ANULOC	A_GSK3_	TA_HBX_	A_HCMV_	A_HDAC_	A_HER2_	TA_HES_
-0.03084	0.118	0.30619	0.19962	0.43459	0.18722	-0.2913	0.24188	-0.42311	-0.16955	0.09606	0.15502	0.14376	0.23517	-0.0751	0.05513	0.04422	0.15301	0.11123	-0.01028	0.11748	0.11658	0.10152	0.14308	-0.25761
0.023	0.06106	0.27972	0.19802	0.42078	0.11046	0.0568	0.20218	-0.36005	-0.10767	0.10668	0.1522	0.15572	0.19811	-0.23852	-0.00331	-0.01452	0.13333	0.13417	-0.06119	0.15802	0.04066	0.05215	0.05615	-0.28011
-0.00076	0.12117	0.26888	0.2367	0.44747	0.15584	-0.28675	0.25989	-0.41518	-0.10925	0.06743	0.1458	0.09598	0.2521	-0.42006	0.03474	0.04866	0.12378	0.14272	0.05354	0.08739	0.10817	0.08816	0.10624	-0.29891
-0.02759	0.15013	0.28183	0.29309	0.41002	0.16607	-0.2102	0.22595	-0.19341	-0.20254	0.09402	0.14023	0.1202	0.23991	-0.201	0.08831	0.07308	0.14856	0.22214	0.05632	0.0454	0.09596	0.13987	0.09639	-0.31386
-0.01023	0.15477	0.28053	0.2247	0.41636	0.21817	-0.27844	0.25539	-0.39317	-0.1453	0.07914	0.14881	0.22312	0.24249	-0.28148	0.07375	0.05691	0.18649	0.19611	0.04565	0.08796	0.14221	0.13843	0.09912	-0.23077
0.03051	0.08997	0.29026	0.25843	0.41714	0.13441	0.02509	0.2431	-0.31478	-0.12199	0.10268	0.15089	0.16866	0.24738	0.08308	0.02998	0.00644	0.14265	0.15433	0.03681	0.11351	0.05924	0.0794	0.02538	-0.27619
0.02694	0.1207	0.30268	0.27719	0.4221	0.17707	-0.2544	0.25116	-0.37291	-0.11983	0.06059	0.11665	0.16249	0.27587	-0.01585	0.06439	0.07647	0.13394	0.1304	0.08477	0.0829	0.12894	0.10317	0.09686	-0.346
-0.02283	0.15371	0.32549	0.31946	0.39071	0.20347	-0.23208	0.2553	-0.21843	-0.22233	0.06173	0.15716	0.17971	0.25575	-0.27567	0.08765	0.0928	0.1742	0.28284	0.00847	0.07019	0.12789	0.15422	0.12513	-0.22659
-0.04064	0.15481	0.29305	0.27445	0.41118	0.19902	-0.24425	0.22781	-0.23604	-0.17213	0.05365	0.13875	0.17461	0.26287	-0.20743	0.09173	0.08972	0.15197	0.28664	0.04484	0.09234	0.12566	0.15696	0.10458	-0.27171
-0.04425	0.15852	0.30696	0.29364	0.40626	0.20051	-0.25684	0.23506	-0.30685	-0.17116	0.05095	0.14306	0.22775	0.24063	-0.06742	0.09426	0.0826	0.17468	0.22601	0.03172	0.10546	0.12989	0.11799	0.08942	-0.27105
0.13985	0.06441	0.34428	0.19772	0.41131	0.11774	-0.10549	0.20305	-0.452	-0.06489	0.09495	0.19035	0.07409	0.25394	-0.15965	-0.00167	0.00648	0.11683	0.09703	0.02687	0.03657	0.0733	0.07496	0.10375	-0.22543
-0.02761	0.08468	0.25777	0.29269	0.43327	0.13973	0.06445	0.27498	-0.44055	-0.12729	0.1257	0.17523	0.17593	0.23579	-0.18415	0.0059	0.04349	0.16513	0.16387	0.03749	0.07708	0.10077	0.10196	0.09631	-0.32556
0.10723	0.03462	0.23115	0.21678	0.39962	0.09185	-0.17022	0.20617	-0.42998	-0.05384	0.11756	0.11178	0.13193	0.19835	-0.05314	0.01064	0.006	0.1235	-0.03713	0.02028	0.15849	0.0662	0.02663	0.14412	-0.14376
0.01556	0.0932	0.29003	0.2418	0.39844	0.14774	-0.05521	0.21004	-0.42258	-0.23333	0.02343	0.18993	0.15729	0.23821	-0.30479	0.01979	0.02582	0.13807	0.19149	0.03665	0.09143	0.07573	0.08621	0.05859	-0.33289
0.03681	0.088	0.30226	0.27523	0.43136	0.16546	-0.15123	0.22786	-0.44742	-0.17468	0.07866	0.20697	0.13817	0.22129	-0.34161	0.03011	0.02994	0.15403	0.12031	0.02034	0.08793	0.09054	0.0595	0.07703	-0.28359
-0.00697	0.02754	0.28298	0.20497	0.43191	0.11799	0.01076	0.207	-0.44073	-0.11798	0.11872	0.1553	0.0909	0.22483	-0.34807	-0.04112	0.01805	0.11068	-0.13719	-0.0057	0.06393	0.06589	0.04839	0.09548	-0.20704
-0.03323	0.10814	0.26604	0.29171	0.41743	0.12087	-0.01317	0.22584	-0.39317	-0.17222	0.06929	0.14451	0.1267	0.24164	-0.09768	0.04657	0.04958	0.12646	0.20301	0.05236	0.04941	0.09634	0.07549	0.11378	-0.29272
-0.06435	0.1693	0.30891	0.2236	0.41761	0.20319	-0.23181	0.24469	-0.3405	-0.19958	0.0814	0.10517	0.17488	0.28881	-0.37501	0.08166	0.10691	0.16547	0.22729	0.10053	0.07488	0.15078	0.17544	0.11479	-0.29504

TA_IFNA_	TA_IFNG_	IGF1MTOR	A_IGF1R_	A_IGF1_P	TA_IL10_P	TA_IL12_P	TA_IL17_P	TA_IL1R_P	A_IL22BP_	A_IL2RB_	TA_IL2_P	TA_IL3_P	TA_IL4_P	TA_IL5_P	TA_IL6_P	TA_IL7_PA	INFLAM_	INSULIN	INTEGRIN	INTRINSI
0.09527	0.36075	0.04643	0.13495	0.04704	0.29567	-0.06192	-0.29024	-0.067	0.08059	0.03397	0.03034	0.1024	-0.03492	0.04289	0.12722	-0.02435	0.04291	0.02731	0.27259	0.08754
0.05161	0.35582	0.02294	0.11557	0.00666	0.25381	-0.18328	-0.38361	-0.14208	0.00725	0.00075	-0.02606	0.02621	-0.01896	0.04258	0.09067	-0.11695	0.04884	-0.02083	0.21291	0.11817
0.13861	0.42311	0.06159	0.12215	0.08734	0.28931	-0.08897	-0.30425	-0.10766	0.0279	0.06836	0.03175	0.07145	0.03144	0.06147	0.11693	-0.08758	0.05934	0.05593	0.26015	0.17076
0.11506	0.41237	0.06851	0.15358	0.08881	0.34165	-0.06602	-0.21105	-0.10174	0.06948	0.07547	0.04791	0.09533	0.00042	0.10972	0.11771	-0.01019	0.10055	0.06934	0.25557	0.2105
0.09644	0.39634	0.02307	0.14316	0.04073	0.34973	0.01811	-0.08655	-0.064	0.1136	0.07658	0.07491	0.10975	0.0125	0.17165	0.12795	-0.00093	0.17689	0.01454	0.24964	0.16679
0.11465	0.39528	0.03588	0.0781	-0.00655	0.28524	0.032	-0.04288	-0.12465	0.09923	0.01209	0.02257	0.03558	-0.02921	0.07604	0.06562	0.01278	0.07606	-0.02814	0.22785	0.29443
0.17299	0.43491	0.04135	0.13872	0.06067	0.28698	0.01053	-0.03633	-0.06996	0.07141	0.06629	0.06172	0.07526	-0.01388	0.14325	0.07495	0.06545	0.1446	0.03177	0.26094	0.28015
0.17065	0.41571	0.05771	0.16993	0.09119	0.37312	-0.07692	-0.18079	-0.0725	0.11715	0.10372	0.05698	0.10943	0.02063	0.18317	0.12969	0.00605	0.18187	0.05947	0.25983	0.23926
0.14146	0.43896	0.05269	0.1584	0.0747	0.36392	-0.04541	-0.12467	-0.01539	0.11249	0.10814	0.05915	0.09969	0.04793	0.32519	0.10059	0.03839	0.26734	0.04669	0.26808	0.29849
0.19831	0.45605	0.02503	0.15214	0.05498	0.36588	0.09482	0.07506	-0.0582	0.1501	0.10124	0.10011	0.12127	0.01662	0.1258	0.12149	0.07143	0.13767	0.0285	0.26375	0.24753
0.0126	0.27585	0.06622	0.09526	0.04738	0.17164	-0.1035	-0.36503	-0.11991	-0.01405	0.0463	-0.01523	0.01082	-0.06028	0.12959	0.06659	-0.10076	0.11543	0.0179	0.23406	0.16373
0.11551	0.44002	0.03563	0.13156	0.00938	0.2756	0.03189	-0.09584	-0.11683	0.0874	0.06557	0.0141	0.04612	-0.01093	0.15475	0.06896	0.00085	0.15397	-0.0188	0.24023	0.25218
0.01849	0.32323	0.07657	0.15128	0.09106	0.18593	-0.1559	-0.42455	-0.07416	-0.02567	0.03492	0.02066	0.06241	0.06144	-0.02113	0.13867	-0.12967	-0.0211	0.06398	0.25411	0.16872
0.06217	0.3375	0.04442	0.12812	0.03644	0.28902	-0.078	-0.20298	-0.09694	0.04098	0.03	0.03048	0.07731	0.03869	0.17897	0.11559	-0.09405	0.1653	0.01082	0.24859	0.18689
0.10354	0.38718	0.04507	0.13158	0.0589	0.25259	-0.12116	-0.32513	-0.09651	0.02516	0.0302	0.0367	0.0885	-0.00413	0.23706	0.13515	-0.14647	0.22162	0.02919	0.26271	0.20779
0.01622	0.40796	0.06999	0.1056	0.02495	0.18765	-0.14747	-0.41093	-0.11272	0.01267	0.01393	-0.01762	0.01598	-0.0393	0.13708	0.09261	-0.15005	0.13709	-0.00385	0.25765	0.08841
0.0429	0.32552	0.06498	0.13791	0.0818	0.29611	-0.11032	-0.23952	-0.12985	0.02113	0.04684	0.03026	0.06508	-0.00395	0.18013	0.09771	-0.03737	0.1678	0.05034	0.24633	0.16202
0.13871	0.42875	0.06161	0.16626	0.0827	0.34685	-0.04167	-0.10357	-0.08736	0.10819	0.11061	0.07082	0.09474	0.03344	0.18242	0.10551	0.07721	0.17657	0.05423	0.25864	0.16701

TA_ION_P	TA_IRES_P	RATINOCY	A_KREB_	A_LAIR_	TA_LDL_P	LECTIN	A_LEPTIN	TA_LIS1_P	ONGEVIT	MPHOCY	TA_LYM_P	MALATE	TA_MAL_P	A_MAPK_	MCALPAIN	A_MCM_P
-0.05896	0.27214	0.02312	0.42189	0.09332	0.36798	0.00916	-0.02689	-0.00454	0.20772	0.2049	0.19617	0.28366	0.31268	0.05376	0.30908	0.09938
0.02146	0.30057	-0.02147	0.36952	0.13224	0.3863	-0.04977	-0.02205	0.0656	0.14723	0.21637	0.19798	0.25807	0.22361	-0.00494	0.30297	-0.07496
-0.15002	0.26706	-0.01939	0.4247	0.12214	0.43685	0.04141	-0.0379	-0.01438	0.24602	0.25898	0.23023	0.23352	0.28704	0.03187	0.28592	0.1711
-0.15207	0.26397	0.0008	0.37074	0.19396	0.39563	0.17906	-0.01538	0.06514	0.21931	0.30138	0.27722	0.23804	0.2927	0.02038	0.29997	0.0998
-0.06607	0.26523	0.02715	0.38832	0.1426	0.40462	0.10104	0.00744	0.06455	0.20488	0.23751	0.20598	0.25843	0.32011	0.06009	0.3093	0.08014
0.06903	0.26517	-0.01624	0.37073	0.2543	0.4062	0.29648	0.06762	0.06699	0.17605	0.18609	0.15011	0.19237	0.22706	0.01623	0.28115	0.17035
-0.06434	0.27932	0.00622	0.38386	0.23105	0.44002	0.195	-0.03191	-0.01587	0.22107	0.21243	0.17099	0.26928	0.29002	0.03411	0.28932	0.1063
-0.12808	0.25213	0.00243	0.37569	0.23141	0.43121	0.17115	-0.00248	0.07396	0.22735	0.33079	0.3083	0.25627	0.32326	0.04388	0.30688	-0.06499
-0.07768	0.25458	-0.00675	0.35867	0.26474	0.41695	0.18919	0.022	0.09996	0.25195	0.33516	0.30915	0.23799	0.28914	0.03951	0.30107	-0.01242
-0.02769	0.23707	0.02342	0.37717	0.20653	0.41712	0.20441	0.0224	0.04654	0.1889	0.28212	0.252	0.26591	0.30728	0.06635	0.31158	-0.06332
-0.21389	0.2499	-0.01936	0.40162	0.1548	0.40708	0.12737	-0.00992	0.12661	0.21245	0.1757	0.1788	0.2616	0.27038	-0.02801	0.30794	0.2711
-0.07269	0.28501	0.00131	0.40679	0.24045	0.42963	0.27346	-0.01019	0.05621	0.18199	0.23781	0.22298	0.25811	0.30134	0.02698	0.32381	0.15186
-0.14132	0.27845	-0.02019	0.37768	0.10932	0.41571	0.10207	0.04228	0.11559	0.19532	0.07596	0.11181	0.26362	0.2834	0.01864	0.30813	0.17477
-0.14226	0.26507	0.0002	0.40528	0.18461	0.41994	0.1331	-0.04482	0.07971	0.18711	0.26317	0.23828	0.25507	0.27646	0.01983	0.31512	-0.12141
-0.10504	0.27211	-0.01659	0.38184	0.11103	0.38806	0.0881	0.027	0.07391	0.19105	0.15899	0.12511	0.24199	0.31487	0.03402	0.29463	0.1404
-0.16511	0.27239	-0.00461	0.42675	0.01184	0.4557	-0.14067	0.00961	0.07273	0.17114	0.08282	0.07736	0.26551	0.28535	-0.00091	0.31239	0.19557
-0.19345	0.27876	-0.03213	0.42969	0.174	0.39457	0.09859	0.00869	0.1097	0.22336	0.29451	0.27969	0.23705	0.27481	-0.00857	0.31314	-0.22514
-0.14364	0.25775	0.00229	0.38929	0.17993	0.43632	0.10857	-0.06184	0.09626	0.26853	0.28138	0.25075	0.25359	0.31107	0.0429	0.30072	0.0041

A_MEF2D_	ELANOCY	TA_MET_P	TA_MHC_P	TOCHOND	TA_MITR_	MONOCY	TA_MPR_P	TA_MRP_P	TA_MSP_P	TA_MTA3_	TA_MTOR_	A_MYOSIN	DKDYNA	EUTROPH	TA_NFAT_
0.18062	-0.03103	0.16965	0.26552	0.02976	0.11187	0.22398	0.21559	0.15542	-0.43094	0.3189	0.09922	0.04634	0.23918	0.20636	0.16515
0.16176	0.01095	0.11818	0.23199	0.06492	0.1324	0.20902	0.22414	0.14126	-0.41121	0.33288	0.10337	-0.03302	0.21743	0.18644	0.13643
0.17142	-0.07467	0.14863	0.24355	0.06482	0.08672	0.26889	0.18691	0.16089	-0.42274	0.33931	0.12286	0.11195	0.25549	0.25197	0.15094
0.17918	-0.02629	0.14175	0.27677	0.06761	0.18434	0.31639	0.1854	0.15795	-0.36971	0.31176	0.13165	0.12035	0.27725	0.30877	0.19306
0.21217	-0.01984	0.13507	0.31898	0.08612	0.12277	0.26301	0.22216	0.13719	-0.42838	0.27524	0.09388	0.08288	0.26802	0.2668	0.18951
0.20476	-0.05498	0.10601	0.26762	0.02225	0.00296	0.1845	0.17405	0.1883	-0.12995	0.29564	0.10506	0.0321	0.24288	0.16976	0.16973
0.18357	-0.13187	0.15874	0.28287	0.09128	0.08598	0.21992	0.20966	0.18984	-0.13769	0.3061	0.13926	0.06624	0.22725	0.19939	0.18216
0.1893	-0.01827	0.17728	0.29891	0.05006	0.21703	0.34487	0.18727	0.12043	-0.31257	0.29495	0.11117	0.09824	0.28412	0.34993	0.21639
0.19125	-0.02748	0.15653	0.33938	0.08432	0.20848	0.3455	0.18974	0.14341	0.06011	0.29397	0.11775	0.10268	0.27675	0.35102	0.20756
0.1923	0.02012	0.14902	0.27969	0.10321	0.11896	0.30132	0.20836	0.14051	-0.27531	0.31111	0.08689	0.12068	0.25341	0.31085	0.20125
0.17046	-0.02407	0.13095	0.24999	0.04301	0.01715	0.19555	0.18019	0.21745	-0.40724	0.33637	0.11055	0.07668	0.24176	0.1663	0.14287
0.18204	-0.02868	0.12831	0.29793	0.00023	0.13034	0.23325	0.23726	0.13103	-0.3624	0.32225	0.11324	0.11262	0.24845	0.20691	0.1884
0.15678	-0.04529	0.13635	0.15537	0.07411	0.07012	0.03357	0.21877	0.14086	-0.40086	0.36906	0.12618	0.01837	0.2206	-0.03804	0.13897
0.17589	-0.06047	0.12199	0.25968	0.04152	0.07984	0.27371	0.16888	0.15029	-0.46749	0.31404	0.10562	0.07686	0.25264	0.26095	0.16218
0.16687	-0.09336	0.14251	0.30911	0.07363	0.05043	0.198	0.20878	0.18028	-0.44205	0.32318	0.09139	0.08262	0.2303	0.20026	0.17061
0.15555	-0.0581	0.13109	0.27736	0.065	0.04422	0.03912	0.22023	0.19534	-0.45389	0.32344	0.12182	-0.00255	0.25143	-0.03593	0.15109
0.14975	-0.07099	0.14078	0.26285	0.04962	0.07248	0.30311	0.18818	0.21081	-0.44095	0.31536	0.12178	0.11705	0.24869	0.28573	0.16663
0.20253	-0.04624	0.16354	0.30913	0.08307	0.16467	0.29997	0.18911	0.13611	-0.4371	0.30121	0.12254	0.0597	0.29548	0.3042	0.2079

TA_NFKB_	TA_NGF_P	NKCELL	TA_NKT_P	TA_NO1_P	NO2IL12	A_NOS1_	A_NOTCH	TA_NPC_P	TA_NPP1_	TA_NTHI_	UCLEAR	TA_P27_P	ALZHEIM	P38MAPK	53HYPOX	TA_P53_P
-0.08885	0.0597	0.26063	-0.43003	0.21691	-0.31358	0.20998	-0.07373	0.21848	-0.19415	-0.07457	-0.00335	0.01628	0.1401	0.08476	0.12206	-0.15426
-0.1282	0.0406	0.17625	-0.4184	0.21134	-0.41195	0.19619	-0.10773	0.23313	-0.20773	-0.09445	-0.09359	0.05616	0.17997	0.02944	0.11708	-0.03552
-0.07823	0.08157	0.2682	-0.34404	0.20021	-0.38635	0.16318	-0.15566	0.2742	-0.23485	-0.09572	-0.01358	0.05422	0.13223	0.07027	0.09121	-0.04104
-0.08291	0.09626	0.28438	-0.29871	0.21646	-0.2927	0.20971	-0.03781	0.1954	-0.15078	-0.10889	0.00658	0.02977	0.1328	0.06856	0.11427	-0.03294
-0.0312	0.05886	0.26911	-0.32603	0.23139	-0.139	0.24922	-0.10612	0.22738	-0.25205	-0.04799	-0.07285	0.04072	0.11291	0.08388	0.09088	-0.01946
-0.11041	0.02143	0.19014	-0.3253	0.28638	-0.04397	0.21856	-0.20854	0.24918	4.9E-05	-0.08079	0.03546	0.03975	0.19545	0.07616	0.10684	-0.0156
-0.0777	0.08815	0.27531	-0.22381	0.24893	-0.07967	0.20063	-0.14458	0.23375	-0.1162	-0.04756	-0.07507	0.08772	0.14067	0.0639	0.10692	-0.0091
-0.02762	0.08282	0.30219	-0.23723	0.24425	-0.27365	0.23982	-0.03215	0.1689	-0.20157	-0.05352	-0.04194	-0.0014	0.13827	0.05579	0.09015	-0.04804
-0.0546	0.08244	0.31024	-0.07354	0.25835	-0.20067	0.22459	-0.14905	0.16389	-0.01088	0.00042	-0.0276	-0.02	0.13071	0.05701	0.09962	-0.03087
-0.032	0.06035	0.28995	-0.19157	0.25714	0.01629	0.23079	-0.06142	0.19796	-0.2161	-0.04686	-0.07567	0.02358	0.1152	0.09941	0.10909	0.00476
-0.14503	0.06761	0.22183	-0.44947	0.2014	-0.46567	0.16539	-0.18967	0.23059	-0.3249	-0.12813	0.02454	0.13618	0.18746	0.02551	0.07642	0.05927
-0.09323	0.02392	0.23529	-0.38922	0.25929	-0.15198	0.21353	-0.24638	0.24395	-0.36314	-0.10632	0.04476	0.02854	0.19042	0.07661	0.1491	-0.06164
-0.13238	0.09765	0.14435	-0.44148	0.18517	-0.42973	0.14971	-0.21689	0.27746	-0.41077	-0.11456	-0.06352	0.15749	0.21992	0.08048	0.13703	-0.01267
-0.06152	0.04314	0.21927	-0.29805	0.23468	-0.28034	0.19886	-0.15517	0.21068	-0.3186	-0.08316	-0.10984	0.02939	0.17121	0.05826	0.08924	-0.05787
-0.07699	0.06744	0.23138	-0.42442	0.22834	-0.45106	0.20521	-0.08991	0.20628	-0.29227	-0.09516	-0.04005	0.02448	0.14377	0.0502	0.10696	0.00587
-0.13743	0.03528	0.18302	-0.46211	0.22416	-0.46304	0.17207	-0.17639	0.25512	-0.34202	-0.11459	-0.05397	0.06877	0.20895	0.03672	0.11615	-0.04398
-0.13726	0.08992	0.23914	-0.36464	0.20393	-0.3153	0.17096	-0.15283	0.20126	-0.22394	-0.12059	-0.13649	0.01599	0.22441	0.05957	0.11857	-0.09051
-0.0483	0.09996	0.29445	-0.20147	0.23902	-0.20664	0.23774	-0.17967	0.20533	-0.33729	-0.05356	-0.07063	0.02214	0.12812	0.0771	0.1084	-0.03107

TA_PAR1_	A_PARKIN	A_PCAF_	A_PDGF_	A_PDZS_	A_PELP1_	TA_PEPI_	A_PGC1A_	A_PITX2_	TA_PKC_	LATELET	A_PLCD_	A_PLCE_	TA_PLC_P	A_PLK3_	TA_PML_	A_PPARA_
0.09549	0.02794	-0.00458	0.07138	0.12311	0.25993	0.30925	0.22762	0.02219	0.01499	-0.04961	0.0617	0.14683	-0.12014	-0.04415	-0.00337	0.09196
0.05858	0.01851	-0.03858	0.03205	0.12494	0.30787	0.10171	0.19514	0.01676	-0.05181	-0.06911	-0.00156	0.23277	-0.16841	-0.056	-0.02381	0.04759
0.11686	0.02148	0.00207	0.07458	0.10415	0.30289	0.15617	0.18573	0.00415	0.01712	-0.09051	0.0332	0.1726	-0.03592	-0.09153	0.05969	0.07491
0.16421	0.03093	-0.03316	0.10682	0.00635	0.21362	0.26181	0.23312	-0.02052	0.04988	0.02822	0.0879	0.22257	0.03522	-0.02425	0.04319	0.10345
0.13311	0.17253	0.03511	0.09031	0.12228	0.25258	0.25328	0.24303	-0.04332	0.0594	-0.03738	0.13512	0.24491	0.00558	-0.04051	0.05503	0.114
0.11314	0.06268	0.00993	0.05749	0.16714	0.11726	0.14644	0.18968	0.02165	0.03943	0.01998	0.08254	0.23399	-0.01634	-0.04372	-0.00235	0.09139
0.14354	0.01629	0.04205	0.0951	0.00693	0.24449	0.13681	0.18829	0.00222	0.04069	0.08561	0.07897	0.21174	0.02114	-0.11058	0.01691	0.11418
0.15444	0.06098	0.01116	0.11507	0.08195	0.24605	0.27232	0.25579	-0.0798	0.05733	0.0295	0.11371	0.27135	0.04926	-0.00102	0.00597	0.112
0.16048	0.05468	0.00464	0.11692	0.10036	0.21226	0.24821	0.2464	-0.06067	-0.00503	0.06008	0.10562	0.24624	0.08572	-0.02027	0.03394	0.12304
0.15918	0.02216	0.0408	0.10665	0.04683	0.19844	0.27308	0.20369	-0.11205	0.1004	0.0653	0.10639	0.27294	0.03294	0.0036	0.038	0.1191
0.12096	0.0399	-0.0415	0.04097	0.07775	0.29284	0.25366	0.18196	0.03397	0.02348	-0.0583	0.01119	0.1511	-0.10625	-0.10489	0.07562	0.07168
0.15806	0.03749	-0.0332	0.05156	0.13688	0.26172	0.22428	0.21025	-0.04872	0.06331	0.01896	0.0787	0.24284	-0.10594	-0.02138	0.06073	0.11436
0.09994	0.01252	-0.03838	0.05974	0.16658	0.30794	-0.03706	0.14395	0.10244	-0.03656	0.00053	-0.04014	0.19402	-0.23708	-0.02645	0.01249	0.05345
0.10744	-0.09542	-0.01048	0.05277	0.06299	0.27619	0.22869	0.19122	-0.01983	-0.00368	-0.08906	0.07745	0.2428	-0.08909	-0.06753	0.01092	0.07494
0.12727	-0.06662	0.0004	0.0569	0.09628	0.29907	0.29271	0.19685	-0.00091	0.01491	0.00558	0.09927	0.21461	-0.13706	-0.06236	0.00405	0.08053
0.06721	0.04971	-0.00626	0.02847	0.07321	0.25714	-0.0464	0.18043	0.05121	-0.0364	-0.08125	-0.01422	0.16029	-0.27559	-0.07379	0.05582	0.0692
0.15722	-0.01284	-0.00718	0.08662	0.06937	0.24846	0.34431	0.15416	-0.01954	0.04478	0.00353	0.11003	0.21557	0.01291	-0.042	0.00447	0.09859
0.13987	0.1281	0.02133	0.10957	0.07671	0.24215	0.19265	0.25679	-0.01107	0.01428	-0.0777	0.11409	0.25885	0.08663	-0.02766	0.04549	0.11794

A_PPARG_	A_PRC2_	A_PRION_	ROTEASO	TA_PS1_P	TA_PTC1_	A_PTDINS	A_PTEN_	TA_PYK2_	TA_RAB_P	A_RAC1_	RACCYC	A_RACC_	RANBP2	A_RANKL_	A_RANMS_
-0.45218	-0.00063	0.22773	0.3107	-0.03228	0.2326	0.07002	0.02742	0.20753	0.2152	0.04046	0.09181	0.28395	0.20513	0.06939	0.35441
-0.31108	0.13597	0.22887	0.29507	-0.06964	0.28388	0.0754	0.00561	0.17961	0.11853	-0.00595	0.0662	0.21578	0.20588	-0.01627	0.31688
-0.33715	-0.01308	0.21745	0.26773	-0.08036	0.30347	0.07894	0.05952	0.18849	0.09057	0.03312	0.10196	0.22864	0.17563	0.03157	0.33242
-0.36252	-0.02984	0.20677	0.2723	-0.05582	0.15946	0.09025	0.06084	0.16617	0.19993	0.08609	0.10151	0.22917	0.17213	-0.00333	0.27353
-0.36798	-0.01903	0.21763	0.31329	-0.09988	0.22654	0.07944	0.03751	0.203	0.19843	0.05921	0.116	0.18408	0.18564	0.03494	0.28924
-0.25277	0.02968	0.23423	0.29891	-0.08902	0.22097	0.06621	-0.0204	0.17996	0.12879	0.06381	0.0454	0.26493	0.17047	0.01511	0.31482
-0.30985	0.04477	0.22545	0.28765	-0.09563	0.25222	0.03134	0.06837	0.19177	0.07485	0.06657	0.13706	0.28043	0.20505	0.09142	0.35309
-0.34085	-0.07798	0.24435	0.29674	-0.08984	0.05026	0.09901	0.06348	0.1873	0.2002	0.09913	0.12752	0.20944	0.10195	0.04714	0.30667
-0.36287	-0.05891	0.22983	0.29604	-0.13017	0.11525	0.09949	0.07142	0.18134	0.16063	0.102	0.11728	0.22794	0.14169	0.01492	0.30815
-0.35653	-0.02218	0.22485	0.28397	-0.1499	0.07814	0.08326	0.05048	0.20431	0.17419	0.07697	0.12198	0.22794	0.16312	0.08993	0.3304
-0.19799	0.0016	0.25253	0.29714	-0.07923	0.2929	0.03933	0.05602	0.15213	0.16879	0.0433	0.09375	0.30104	0.16485	0.01026	0.31334
-0.35124	-0.02495	0.22968	0.27077	-0.16736	0.27013	0.03053	0.04253	0.19223	0.18988	0.025	0.05849	0.20504	0.18227	0.05012	0.30961
-0.31425	0.04978	0.25124	0.33436	-0.01603	0.30173	0.08683	0.05489	0.17924	0.17453	-0.09377	0.06186	0.41184	0.24826	-0.02485	0.34129
-0.30474	-0.0382	0.24045	0.28191	-0.11697	0.07301	0.07549	0.02873	0.17512	0.14009	0.05022	0.09194	0.26405	0.15995	0.00454	0.32781
-0.38506	-0.02934	0.25311	0.29678	-0.06128	0.15688	0.06486	0.01485	0.20337	0.19108	0.03692	0.09424	0.28061	0.16071	0.02377	0.33354
-0.2718	0.00025	0.25778	0.3061	-0.04658	0.34881	0.07231	0.03909	0.17294	0.14604	-0.02664	0.04565	0.25454	0.21557	0.04746	0.33043
-0.3671	-0.03428	0.21066	0.28563	-0.10847	0.13248	0.08737	0.0586	0.15248	0.15858	0.09514	0.08167	0.34455	0.16784	-0.02848	0.31495
-0.3449	-0.05646	0.24344	0.30264	-0.09018	0.13079	0.08466	0.08522	0.18126	0.1882	0.09406	0.13925	0.22864	0.16299	0.04814	0.3209

TA_RAN_P	RARRXR	TA_RAS_P	TA_RB_P	A_RECK_	A_REELIN	A_RELA_	HODOPS	A_RHO_P	RNAPOL3	TA_RNA_P	TA_S1P_P	LMONEL	A_SAM68_	A_SARS_	TA_SET_P	TA_SHH_P
0.33527	-0.22893	0.13461	-0.09522	0.03941	-0.2024	-0.03007	0.00088	0.2569	-0.02472	0.16444	-0.01002	0.39073	0.22332	0.2557	0.23101	0.0847
0.33216	-0.12946	0.07068	-0.04706	-0.00143	0.29804	-0.09441	0.22882	0.19572	0.07466	0.13962	-0.02036	0.26898	0.24021	0.21018	0.23191	0.12343
0.32436	0.03336	0.12306	-0.06708	0.02173	-0.192	-0.05763	0.07134	0.2601	0.00096	0.19901	-0.04811	0.34645	0.26072	0.2321	0.23611	0.03301
0.30162	-0.06406	0.15407	-0.10231	0.07928	-0.06385	-0.05822	-0.01462	0.26817	-0.12842	0.17672	-0.04824	0.26492	0.19782	0.26103	0.18281	0.07192
0.33369	-0.04805	0.14232	-0.0715	0.10031	-0.20187	0.01338	0.12295	0.26961	-0.02523	0.17381	-0.15298	0.24482	0.21223	0.24658	0.31555	0.11381
0.32567	-0.06165	0.08892	-0.06966	-0.08662	0.24361	-0.09274	0.1425	0.23929	-0.03264	0.17512	-0.06448	0.25988	0.11647	0.18891	0.30154	0.11262
0.35061	-0.06179	0.17968	-0.0738	0.23142	-0.15501	-0.05099	0.23481	0.24896	-0.01917	0.17532	-0.15191	0.37885	0.21048	0.25053	0.24842	0.08374
0.25703	-0.10319	0.16147	-0.12829	0.14691	-0.11749	0.00061	0.01867	0.27508	-0.11986	0.19107	0.06364	0.2606	0.17537	0.24449	0.19928	0.11527
0.27323	-0.03707	0.1601	-0.12252	0.02725	-0.13423	-0.02654	-0.01075	0.25686	-0.10686	0.18697	-0.09896	0.24645	0.18383	0.24286	0.26444	0.0968
0.29695	-0.10149	0.15345	-0.12236	0.05057	-0.1573	0.00693	0.00951	0.26107	-0.09905	0.20567	-0.20144	0.23173	0.14937	0.24235	0.28177	0.12142
0.30856	-0.06932	0.11962	-0.00699	0.09114	0.08578	-0.14091	0.02043	0.26515	-0.04424	0.1627	-0.14602	0.29415	0.2232	0.23673	0.22357	0.03926
0.34195	-0.10168	0.10226	-0.06958	0.03857	0.29012	-0.05958	0.10657	0.27248	-0.08211	0.19363	-0.0433	0.38773	0.21647	0.27015	0.2589	0.11722
0.36489	-0.0582	0.1427	0.01344	-0.0495	-0.02043	-0.14071	0.10974	0.23581	-0.01457	0.14143	-0.10869	0.39252	0.25142	0.14512	0.24418	0.09852
0.32346	-0.10388	0.11983	-0.15673	0.0727	0.11168	-0.02035	0.17122	0.26286	-0.06085	0.1703	-0.09187	0.34432	0.17391	0.23826	0.29342	0.0911
0.3008	-0.0682	0.1046	-0.09104	0.14849	-0.01656	-0.0514	0	0.25425	0.00263	0.18106	0.01866	0.32884	0.20096	0.26382	0.21645	0.09529
0.35814	-0.04772	0.11686	-0.03803	-0.01032	0.25788	-0.14156	0.08297	0.2513	-0.02379	0.14778	-0.12133	0.32467	0.26123	0.2181	0.17702	0.07363
0.30998	-0.09966	0.13645	-0.12859	0.05335	0.18443	-0.11561	-0.06764	0.26695	-0.02781	0.16147	-0.05876	0.3588	0.20071	0.24433	0.22499	0.08631
0.29801	-0.05914	0.17857	-0.11553	-0.11647	-0.11362	-0.01323	0.02272	0.26356	-0.06913	0.19328	-0.18254	0.22974	0.20402	0.24095	0.26085	0.10002

SKP2E2F	A_SLRP_	TA_SM_P	A_SOOD_	A_SPPA_	A_SPRY_	SRCRPT	A_STAT3_	STATHMIN	A_STEM_	A_STRESS	A_SUMO_	A_TALL1_	APOPTOS	A_TCRA_	TA_TCR_P	CYTOTOX
-0.03084	-0.37344	0.3087	-0.21007	0.21594	0.10147	0.05096	0.20587	0.15947	-0.42505	-0.04726	0.35306	0.08806	-0.30934	-0.0109	0.05467	0.04818
0.023	-0.37097	0.35358	-0.24242	0.18907	0.05845	0.11378	0.16709	0.152	-0.41184	-0.09781	0.36574	-0.06721	-0.40333	-0.05113	-0.00578	-0.01256
-0.00076	-0.0458	0.32886	-0.16773	0.27485	0.11509	0.11748	0.162	0.09034	-0.29875	-0.10155	0.38306	0.00502	-0.3733	-0.02126	0.04163	0.10338
-0.02759	-0.18778	0.31712	-0.1386	0.25764	0.09551	0.12195	0.16789	0.12361	-0.20997	-0.08219	0.30018	-0.00349	-0.28516	0.03738	0.06972	0.07252
-0.01023	-0.34349	0.30806	-0.08867	0.26756	0.10391	0.14853	0.1823	0.16429	-0.42311	-0.01771	0.35038	0.07172	-0.13869	0.15207	0.11784	0.11371
0.03051	0.13986	0.31662	-0.14211	0.18659	0.01953	0.09022	0.07197	0.16772	-0.31777	-0.09051	0.35559	-0.0161	-0.02503	0.13581	0.09973	0.1446
0.02694	0.2105	0.32255	-0.15851	0.26826	0.07679	0.10851	0.15097	0.13802	-0.01585	-0.06456	0.38343	0.0632	-0.07942	0.14996	0.10396	0.11536
-0.02283	-0.01938	0.29711	-0.14931	0.24439	0.1158	0.09085	0.18374	0.08446	-0.04386	-0.02572	0.27494	0.05141	-0.26012	0.08648	0.08866	0.10723
-0.04064	0.07627	0.29206	-0.12514	0.23463	0.11738	0.12313	0.15249	0.09507	0.01867	-0.04389	0.32468	0.03811	-0.18365	0.14816	0.0859	0.12927
-0.04425	-0.21543	0.29635	-0.11785	0.26592	0.10465	0.11723	0.14956	0.1701	-0.00018	-0.00652	0.32922	0.08335	0.02096	0.16359	0.12698	0.18648
0.13985	-0.09195	0.30961	-0.25573	0.19104	0.08816	0.04316	0.15845	0.09708	-0.46503	-0.12412	0.3478	-0.00307	-0.30464	0.03167	0.02539	0.04809
-0.02761	0.03083	0.31588	-0.16553	0.1759	0.08208	0.0641	0.16742	0.17748	-0.356	-0.09523	0.36392	0.04772	-0.10719	0.12248	0.05025	0.10927
0.10723	-0.32394	0.32446	-0.10489	0.21214	0.11969	0.00447	0.21098	0.14028	-0.4247	-0.11814	0.37868	-0.06365	-0.43014	-0.13015	-0.00654	-0.16678
0.01556	-0.00018	0.30596	-0.17707	0.24099	0.08365	0.0434	0.19762	0.09053	-0.15554	-0.0551	0.29016	0.01518	-0.28377	0.08268	0.04306	0.05769
0.03681	-0.00881	0.31914	-0.16764	0.19722	0.10044	0.06753	0.16736	0.10122	-0.45869	-0.06691	0.3267	0.02749	-0.41599	0.09577	0.0281	0.05066
-0.00697	-0.22811	0.30071	-0.19933	0.19338	0.09483	0.09853	0.20157	0.13295	-0.45781	-0.14432	0.3598	0.04456	-0.36448	-0.03409	-0.01307	-0.08786
-0.03323	-0.28994	0.33067	-0.18424	0.23463	0.07208	0.09714	0.15749	0.0959	-0.24097	-0.10742	0.34323	-0.03349	-0.31307	0.08379	0.04261	0.1032
-0.06435	-0.14004	0.29082	-0.14823	0.27918	0.10242	0.11299	0.18078	0.10936	-0.04316	-0.03266	0.33161	0.04662	-0.1876	0.1219	0.11196	0.09645

TA_TEL_P	A_TERC_	A_TERT_	TA_TFF_P	A_TGFB_	A_TH1TH2_	THELPER	TA_TID_P	A_TNFR1_	A_TNFR2_	A_TOB1_	TA_TOLL_
0.0898	-0.1573	0.01258	0.08698	0.03502	0.14714	0.01476	0.08972	0.15359	-0.07535	-0.30038	-0.03891
0.09835	-0.07549	0.04529	0.05259	0.04181	0.13677	-0.04429	0.13606	0.14057	-0.07867	-0.32075	-0.07247
0.10986	-0.08719	-0.02905	0.10174	0.05551	0.18689	0.06337	0.09049	0.14981	-0.13558	-0.34395	-0.05658
0.09057	-0.1651	-0.0636	0.09824	0.0476	0.2312	0.04246	0.05845	0.13141	-0.11831	-0.30861	-0.05821
0.1235	-0.1175	-0.09494	0.06593	0.00623	0.2471	0.07884	0.03491	0.17608	-0.06993	-0.10569	0.01784
0.0825	-0.15586	0.00792	0.00514	-0.06505	0.1307	0.10909	0.06866	0.14907	-0.14643	-0.04832	-0.08104
0.06975	-0.22932	-0.03083	0.0928	0.05344	0.22575	0.1003	0.06676	0.15577	-0.1054	-0.08708	-0.03649
0.08243	-0.20881	-0.11176	0.0756	0.06733	0.27553	0.09007	0.03875	0.14135	-0.06361	-0.31651	0.00212
0.09208	-0.19785	-0.10356	0.09048	0.03948	0.35782	0.11703	-0.00624	0.16218	-0.10705	-0.22646	-0.03941
0.07767	-0.10172	-0.08858	0.05487	0.04545	0.24642	0.16625	0.02777	0.15582	-0.06686	-0.0095	0.01894
0.10915	-0.0894	-0.06367	0.11018	0.07245	0.2053	0.01569	0.06616	0.12464	-0.15163	-0.34679	-0.04103
0.09795	-0.20053	-0.1777	0.07203	0.02111	0.24794	0.06907	-0.01195	0.13739	-0.15228	-0.1396	-0.05101
0.13313	-0.13379	0.02127	0.13958	0.09476	0.13183	-0.19217	0.06345	0.14703	-0.15808	-0.33712	-0.10004
0.10687	-0.1328	-0.13876	0.05926	0.03519	0.26262	0.03507	0.05292	0.13591	-0.09691	-0.30704	-0.02099
0.06634	-0.09346	-0.01031	0.0617	0.06379	0.29227	0.01788	0.09682	0.15635	-0.09879	-0.40673	-0.00851
0.13357	-0.07772	-0.02656	0.11981	0.07978	0.20145	-0.11378	0.07563	0.11734	-0.16112	-0.38451	-0.06421
0.12391	-0.15447	-0.02898	0.08835	0.0289	0.24949	0.06961	0.0961	0.13792	-0.14487	-0.34122	-0.08808
0.09721	-0.2406	-0.08158	0.1101	0.0418	0.283	0.08083	0.06507	0.15312	-0.08241	-0.24631	-0.03164

UCALPAI	ITA_VDR_PA	A_VEGF_	ITA_VIP_PA	A_VITCB_	VOBESITY	A_WNT_F	C_TRANSE	MYELO	HERENS_	KINE_SIG	TE_AND_	GULATED	OGRAFT_	OLENIC_A	HEIMERS	YL_TRNA	NUCLEO
0.37301	0.08327	0.08407	0.15868	0.14341	0.05154	0.05295	-0.04425	0.07116	0.21166	0.00854	0.12886	0.03297	0.06924	-0.10103	0.17393	0.16015	0.18326
0.35222	0.1774	0.09927	0.14913	0.08189	-0.40432	0.06872	-0.09308	0.02826	0.18485	-0.01353	0.10157	-0.02924	0.03842	-0.08472	0.16685	0.15386	0.16753
0.38079	0.14678	0.10275	0.11922	0.10048	-0.02949	0.08757	-0.05833	0.05253	0.20259	-0.00025	0.16049	0.06807	0.11438	0.01749	0.16379	0.14618	0.18478
0.37276	0.07304	0.09307	0.1496	0.13727	-0.03031	0.06227	-0.01478	0.08056	0.18471	0.03069	0.10103	0.07994	0.14497	-0.00274	0.1845	0.1141	0.18125
0.35732	0.12095	0.11776	0.1925	0.16012	0.08151	0.02578	-0.03948	0.09477	0.14069	0.02867	0.12568	0.04071	0.19023	-0.06649	0.15728	0.11599	0.18699
0.34846	0.15941	0.08492	0.17627	0.136	-0.39403	0.0356	0.02238	0.01993	0.14352	0.02725	0.14747	-0.03792	0.11086	0.0589	0.17245	0.13856	0.12537
0.35235	0.11268	0.09138	0.14524	0.12715	-0.21206	0.07282	0.00457	0.06654	0.1818	0.00747	0.12871	0.04848	0.13785	0.02711	0.17935	0.14068	0.1628
0.36236	0.08088	0.10275	0.17723	0.1643	0.00811	0.02716	-0.03431	0.07436	0.15056	0.04065	0.13759	0.06881	0.16837	-0.15472	0.17177	0.10709	0.20644
0.3701	0.09427	0.0972	0.1552	0.25459	-0.08423	0.04274	0.02174	0.07142	0.15098	0.03266	0.08329	0.05785	0.2688	-0.22305	0.17733	0.10483	0.18744
0.3594	0.07244	0.07458	0.17978	0.10423	0.1167	0.01511	-0.02047	0.07076	0.14416	0.03497	0.09679	0.04147	0.20278	-0.04659	0.19109	0.10764	0.18707
0.36688	0.13372	0.11159	0.12905	0.14762	0.10104	0.10643	-0.05491	0.01439	0.21198	-0.02133	0.16923	0.04677	0.06876	0.07924	0.1842	0.18453	0.15942
0.38808	0.12907	0.09567	0.17004	0.07321	-0.02023	0.02846	-0.00519	0.02255	0.1902	-0.02885	0.14155	0.03417	0.16583	0.05632	0.16565	0.18692	0.18092
0.39045	0.19727	0.13054	0.10697	0.10353	-0.34306	0.15051	-0.09073	0.03284	0.21263	0.00335	0.12744	0.00511	-0.02458	0.02544	0.11282	0.15248	0.13371
0.3943	0.11543	0.06784	0.14784	0.14327	0.10404	0.05397	-0.06778	0.05832	0.19828	0.02304	0.12402	0.0307	0.16403	0.02231	0.16056	0.14854	0.20806
0.36795	0.10866	0.11076	0.14969	0.10657	0.12734	0.05396	-0.02324	0.03864	0.18341	0.00097	0.14972	0.05739	0.16026	-0.08423	0.15761	0.16598	0.20206
0.38054	0.15359	0.09467	0.12192	0.0632	-0.24059	0.09678	-0.04843	0.03956	0.21424	-0.03337	0.20518	0.03464	0.08744	0.08196	0.16425	0.20801	0.17198
0.38363	0.14161	0.0908	0.12564	0.14817	-0.04697	0.05533	-0.06794	0.0727	0.19606	0.00048	0.10178	0.06201	0.0817	0.02723	0.18745	0.14285	0.16036
0.36435	0.06346	0.07451	0.1757	0.15306	-0.21779	0.0485	-0.0147	0.09145	0.16101	0.0113	0.08813	0.06604	0.21507	0.0281	0.19733	0.13004	0.19979

C_LATER	CESSING	G_APOPT	IONIC_AC	AND_PRO	T_VENTRI	AND_ALD	GG_ASTH	UNE_THY	AXON_GU	L_CELL_	RANSCRI	E_EXCIS	ALANINE
0.12749	0.2398	-0.00574	0.06522	0.12503	0.19679	0.23132	0.11692	0.06924	0.06127	-0.11476	-0.10442	0.03407	0.16129
0.0969	0.24679	-0.02287	0.10477	0.12709	0.19103	0.24263	-0.01449	0.03842	0.05212	-0.1558	-0.0234	0.15264	0.14132
0.12377	0.25115	-0.00418	0.11246	0.17494	0.23298	0.30223	0.06367	0.11438	0.06096	-0.09612	0.04208	0.13408	0.18526
0.13345	0.28208	0.02175	0.11532	0.11513	0.16527	0.204	0.15454	0.14497	0.04619	-0.17959	-0.04549	0.08515	0.13337
0.16388	0.28398	0.04077	0.09649	0.12386	0.13167	0.17092	0.16943	0.19023	0.05927	-0.19495	-0.03777	0.08965	0.12834
0.11947	0.24185	0.00159	0.12736	0.15851	0.15615	0.22859	0.04824	0.11086	0.05005	-0.11543	0.00863	0.1399	0.10637
0.11676	0.25145	0.03672	0.15043	0.15495	0.16083	0.22802	0.08072	0.13785	0.06516	-0.1578	-0.02364	0.12374	0.1622
0.13236	0.28341	0.029	0.10203	0.11036	0.11088	0.24004	0.17317	0.16837	0.06635	-0.22333	-0.11186	0.05489	0.16073
0.13685	0.31778	0.04675	0.10235	0.0855	0.15798	0.20657	0.24123	0.2688	0.05674	-0.20937	-0.05634	0.06892	0.15808
0.13855	0.2922	0.03675	0.07915	0.0821	0.08509	0.2215	0.22228	0.20278	0.06098	-0.28038	-0.08898	0.09935	0.14076
0.11237	0.23191	0.0061	0.03389	0.20968	0.20721	0.29262	0.05739	0.06876	0.0727	-0.13941	-0.01566	0.12083	0.17396
0.1162	0.26082	-0.00533	0.0236	0.15481	0.16955	0.20109	0.08673	0.16583	0.05008	-0.21125	-0.06057	0.12025	0.16555
0.10299	0.16949	0.00914	0.00933	0.14639	0.24158	0.19025	-0.11325	-0.02458	0.11261	0.0088	-0.01345	0.11045	0.12879
0.12442	0.26968	0.00699	0.12519	0.14399	0.19082	0.23814	0.16463	0.16403	0.05752	-0.19838	-0.04442	0.10483	0.17583
0.11507	0.26922	-0.00469	0.11992	0.17074	0.18593	0.34156	0.20136	0.16026	0.04127	-0.1579	-0.02223	0.10824	0.20529
0.11481	0.22667	-0.01421	0.0536	0.21266	0.21869	0.31213	-0.01529	0.08744	0.065	-0.06997	0.00542	0.16417	0.19739
0.10704	0.23617	-0.0013	0.11266	0.13687	0.16044	0.2295	0.0938	0.0817	0.06625	-0.18739	-0.0426	0.08061	0.15281
0.15635	0.28599	0.04437	0.09371	0.10051	0.20267	0.28995	0.21283	0.21507	0.07099	-0.15012	-0.04918	0.0444	0.18023

OF_UNSA	LADDER	ANOATE_M	EPTOR_SM	SIGNAL	MUSCLE	ESION_M	4_CELL	INE_SIGN	IC_MYEL	DIAN_RH	TE_CYCLE	ORECTAL	AND_COA	D_METHI
-0.03373	0.13438	0.18122	0.03154	0.10431	0.30663	0.08945	0.08025	0.07248	-0.01832	-0.02026	0.30091	0.0599	0.01396	0.23556
-0.02205	0.08608	0.15365	-0.01826	0.08525	0.29278	0.07089	0.08722	0.02259	-0.02025	-0.03047	0.26627	0.05918	-0.00389	0.20113
-0.00078	0.10583	0.15534	0.0292	0.06895	0.30781	0.10075	0.10972	0.06543	0.00389	-0.05073	0.304	0.09655	0.07723	0.23241
0.051	0.09579	0.1248	0.08504	0.09625	0.29263	0.10753	0.08452	0.11135	-0.02112	-0.22477	0.26176	0.0924	0.17507	0.18528
0.02042	0.09581	0.14642	0.10185	0.09693	0.24576	0.08969	0.08282	0.10829	0.01036	-0.15026	0.25662	0.10912	0.09852	0.19701
0.01337	0.00814	0.10113	0.00083	0.08904	0.27628	0.04962	0.1246	0.0547	-0.03399	-0.0502	0.24427	0.06583	0.26072	0.17048
0.0072	0.10066	0.13782	0.05646	0.06319	0.30351	0.09822	0.09294	0.10331	0.00232	-0.20275	0.28976	0.09614	0.25402	0.25066
0.01846	0.0862	0.14386	0.11209	0.09711	0.28513	0.1167	0.03607	0.12912	-0.00829	-0.10921	0.26736	0.07822	0.18638	0.19524
0.01695	0.07509	0.09926	0.10394	0.08519	0.27909	0.15432	0.02394	0.13509	-0.00935	-0.16382	0.24125	0.09696	0.20207	0.20263
-0.00426	0.08234	0.13192	0.10239	0.0916	0.28875	0.11877	0.02555	0.13379	-0.00753	-0.13933	0.25751	0.07293	0.20231	0.18998
0.06727	0.11221	0.18784	-0.01655	0.076	0.28896	0.0518	0.1666	0.02554	-0.00972	-0.38031	0.29923	0.08344	0.08569	0.23051
0.05669	0.11243	0.16009	-0.00132	0.08781	0.31281	0.10622	0.11532	0.05138	-0.02879	0.05073	0.30383	0.06467	0.21967	0.24047
0.02613	0.11427	0.12829	-0.04113	0.07174	0.26001	0.04153	0.14604	0.03239	0.01557	0.0155	0.26599	0.10414	0.09384	0.22826
0.07179	0.06687	0.15989	0.04792	0.0967	0.2669	0.11251	0.03794	0.08853	-0.02521	-0.08896	0.29835	0.05826	0.1288	0.1901
0.03223	0.10321	0.1967	0.01539	0.08816	0.29141	0.09219	0.09129	0.06803	-0.03003	-0.07398	0.25802	0.05472	0.12684	0.22237
0.07392	0.11578	0.20141	-0.04829	0.07981	0.28784	0.04957	0.12801	0.0099	-0.01867	0.17456	0.32506	0.07522	-0.04645	0.24878
0.03414	0.12388	0.14639	0.05078	0.07607	0.32098	0.08615	0.03662	0.0981	-0.00628	0.00863	0.29262	0.09266	0.08726	0.18574
0.00964	0.04776	0.15818	0.10506	0.1008	0.30913	0.12536	0.0486	0.11246	-0.00036	-0.14761	0.28149	0.08979	0.10515	0.19634

KINE_RE	DNA	SEED_CAR	DNA_REPL	ENTRAL_	BOLISM	ABOLISM	CEPTOR_	ENDOCY	OMETRI	ANG_IN_HE	SIGNAL	IR_LIPID	Y_ACID	MN_RI_SI
-0.13614	-0.01555	0.21593	0.05235	0.08947	0.07144	-0.02252	-0.02286	0.08711	0.04962	0.16673	-0.00765	0.11878	0.20209	0.06071
-0.21527	-0.03809	0.2279	0.03705	0.05186	-0.01024	0.01642	-0.00406	0.04226	0.00617	0.12832	-0.05582	0.11251	0.16102	0.0084
-0.18996	-0.03168	0.22753	0.11083	0.07146	0.02453	0.02598	-0.01329	0.08501	0.05812	0.14833	-0.00575	0.17229	0.20066	0.05994
-0.13287	-0.007	0.21893	0.07161	0.07727	0.04424	0.08978	0.0184	0.10593	0.06863	0.19131	-0.02041	0.17211	0.19407	0.1036
-0.20541	-0.01485	0.18454	0.06651	0.10859	0.03037	0.0614	-0.06385	0.09985	0.04919	0.15722	0.00556	0.15925	0.18967	0.11596
-0.15626	-0.07621	0.18091	0.12103	-0.00273	0.02007	0.02855	0.00019	0.04645	-0.01211	0.07785	-0.01981	0.14608	0.19303	0.03978
-0.08611	-0.01941	0.21046	0.06493	0.06042	0.02962	0.04622	0.09212	0.05632	0.06175	0.11599	-0.00747	0.1458	0.19939	0.08333
-0.0993	-0.00599	0.21631	-0.01591	0.0707	0.08904	0.068	-0.01823	0.12381	0.0399	0.19	0.00088	0.13562	0.19037	0.0988
0.00793	0.03762	0.20822	0.00705	0.0564	0.05278	0.07087	0.0086	0.11398	0.04917	0.18443	-0.00066	0.12711	0.1807	0.10716
-0.04692	0.00642	0.18238	0.02026	0.06712	0.04935	0.05814	-0.0509	0.10357	0.02896	0.18094	0.0013	0.1589	0.18752	0.12186
-0.10536	-0.04352	0.19886	0.12374	0.04573	0.07308	0.03447	-0.03756	0.06129	0.05	0.11007	-0.0328	0.18798	0.21298	-0.01668
-0.16011	-0.02403	0.21611	0.10996	0.04863	0.08471	0.08088	0.00248	0.06258	0.02982	0.10036	-0.03927	0.1754	0.21396	0.03197
-0.18152	-0.11679	0.25458	0.10212	0.07332	-0.0078	0.02317	0.01404	0.08061	0.08121	0.09885	-0.00554	0.14324	0.16417	-0.03182
-0.1548	0.00393	0.20643	-0.03998	0.07652	0.07877	0.07173	-0.03684	0.08605	0.03195	0.16398	-0.03193	0.18317	0.2056	0.0652
-0.13088	-0.01503	0.19534	0.082	0.0598	0.11984	0.01288	-0.06389	0.088	0.03285	0.13178	-0.02825	0.10228	0.18502	0.02348
-0.17464	-0.0629	0.21691	0.12379	0.08314	0.07134	0.05631	0.0215	0.07673	0.07511	0.09359	-0.0041	0.07866	0.21439	-0.04603
-0.18141	-0.02096	0.21527	-0.08444	0.07451	0.04924	0.02554	0.00493	0.08854	0.06211	0.15309	-0.0156	0.14837	0.20537	0.08055
-0.13425	0.00106	0.22085	-0.00655	0.07402	0.02352	0.0862	-0.02225	0.09727	0.07558	0.17458	-0.00208	0.19657	0.21205	0.11054

R_MEDIAT	OCAL_AD	ATE_BIOS	ND_MAN	CTOSE_M	GAP_JUN	GG_GLIO	THIONE_	ROLIPID	HOSPHOL	AND_TH	SIS_GLU	BIOSYNTH	N_BIOSYN
0.14187	0.10068	-0.06496	0.16991	0.20078	0.19435	0.05113	0.1781	0.08889	0.04829	0.0266	0.28666	-0.02267	-0.10015
0.09382	0.07402	-0.05499	0.19223	0.19857	0.18383	0.04628	0.1318	0.1266	0.05124	0.0387	0.28224	-0.12321	-0.16723
0.14385	0.10192	0.00022	0.16389	0.20916	0.1872	0.0551	0.17277	0.1197	0.0621	0.08049	0.28165	0.04967	0.06074
0.18365	0.10646	-0.09415	0.19577	0.21	0.21558	0.07175	0.18395	0.10147	0.08797	-0.00198	0.27756	-0.09553	-0.17281
0.16685	0.0791	-0.00853	0.20157	0.21004	0.20017	0.07217	0.16169	0.11714	0.07783	-0.02313	0.2797	0.15437	0.0366
0.11523	0.1	-0.02898	0.13761	0.14582	0.14866	0.0443	0.16069	0.12182	0.05792	0.13732	0.25466	0.10236	-0.0228
0.16551	0.1267	0.00122	0.17192	0.21163	0.18072	0.03878	0.16918	0.11284	0.03736	0.1008	0.26273	-0.02228	-0.1208
0.20099	0.11659	-0.04242	0.18183	0.21384	0.19983	0.04968	0.17341	0.11276	0.06406	0.07172	0.27666	0.12438	0.12126
0.18863	0.11399	-0.03632	0.18362	0.2306	0.19092	0.05105	0.19185	0.11961	0.04919	-0.03962	0.27472	0.06204	-0.05009
0.19777	0.08808	-0.03686	0.16956	0.19824	0.19601	0.04685	0.1684	0.1252	0.04916	-0.09367	0.26514	-0.09321	-0.16978
0.09545	0.09883	-0.02347	0.18666	0.18566	0.20154	0.02239	0.19191	0.12425	0.07158	0.09585	0.29179	-0.00466	-0.06844
0.11564	0.0975	-0.04685	0.16147	0.1876	0.20833	0.0289	0.2142	0.10867	0.05601	0.0934	0.27044	0.03812	-0.06355
0.08575	0.10544	0.00414	0.13358	0.16805	0.22578	0.0317	0.15049	0.10998	0.07322	0.07563	0.25291	0.04147	-0.07171
0.15946	0.08543	0.00211	0.1852	0.24339	0.19734	0.06348	0.17199	0.12413	0.08434	-0.00896	0.27984	0.06822	-0.03696
0.12943	0.09071	-0.05508	0.16995	0.20752	0.18467	0.03527	0.21388	0.12357	0.04532	0.08761	0.29065	0.06255	0.01713
0.05002	0.09701	0.03977	0.1842	0.21262	0.20565	0.05449	0.18935	0.12091	0.00377	0.10847	0.28532	-0.03548	-0.09776
0.1717	0.10744	-0.07858	0.17657	0.22378	0.22309	0.06364	0.15787	0.11005	0.09098	0.04071	0.27938	-0.12813	-0.20237
0.18788	0.10739	0.00781	0.17406	0.20745	0.208	0.0702	0.16772	0.13221	0.06291	-0.05577	0.28988	-0.07103	-0.14491

N_BIOSYN	NOGLYC	D_BIOSY	ID_BIOS	YNTHESI	LINOSIT	D_DICAR	SIGNAL	VERSUS	DG_SIGN	POIETIC	IDINE_M	DGOUS_R	TINGTON	HIC_CAR
-0.30386	0.0831	0.07287	0.05493	-0.23036	-0.16471	0.30919	0.16101	0.06819	0.00057	0.05482	0.04236	0.0259	0.19112	0.16284
-0.16403	0.02845	0.0492	0.0049	-0.20171	-0.09908	0.28235	0.13455	0.01858	-0.01903	0.02257	0.05641	0.07469	0.19486	0.18802
-0.12324	0.08974	0.11793	0.08781	-0.3056	-0.12383	0.29129	0.12538	0.10514	-0.01687	0.06161	0.07834	0.05542	0.1903	0.18431
-0.31892	0.12616	0.1868	0.13041	-0.18572	-0.08995	0.27231	0.1522	0.1328	-0.02994	0.11996	0.03559	0.06747	0.19134	0.1678
-0.25487	0.13957	0.0979	0.09937	-0.23835	-0.13689	0.26388	0.14857	0.18619	-0.04189	0.0796	0.02169	0.03616	0.17363	0.14446
-0.16181	0.09589	0.08469	0.01509	-0.23004	-0.0667	0.2929	0.12204	0.11156	-0.04234	0.03944	0.00578	0.09111	0.19105	0.1546
-0.15613	0.11602	0.09725	0.06792	-0.13364	-0.22036	0.28852	0.13404	0.12405	-0.05933	0.09796	0.02362	0.11017	0.20008	0.15862
-0.19268	0.1397	0.16625	0.17771	-0.1662	-0.11018	0.27766	0.12088	0.1539	-0.00853	0.10826	0.10815	0.06215	0.18398	0.16442
-0.25956	0.17219	0.16312	0.19258	-0.21549	-0.07879	0.25549	0.10907	0.27765	-0.03163	0.15057	0.06084	0.04962	0.18687	0.16061
-0.20778	0.1499	0.14115	0.15178	-0.1952	-0.07952	0.28165	0.14848	0.18071	-0.02552	0.10781	0.04579	0.09368	0.20392	0.14072
0.00176	-0.01912	0.06799	-0.0394	-0.14366	-0.03235	0.32544	0.0891	0.06992	-0.0618	0.03081	0.04529	0.03855	0.20774	0.14652
-0.19246	0.12914	0.09632	0.05011	-0.04157	-0.157	0.31577	0.14377	0.16352	0.05587	0.09984	0.00583	0.06643	0.18465	0.16529
0.02262	-0.07735	-0.03833	-0.05882	-0.00071	-0.13122	0.29867	0.11586	-0.05293	0.01985	-0.02897	-0.01765	0.09589	0.14438	0.2108
-0.13559	0.19946	0.21493	0.17384	-0.09343	-0.16637	0.30278	0.15285	0.15717	0.00101	0.07589	0.08904	0.00946	0.17287	0.15261
-0.19427	0.09823	0.1495	0.07531	-0.27175	-0.17418	0.31709	0.12024	0.15746	-0.00628	0.08682	0.1217	0.04661	0.1699	0.15663
-0.16065	0.04185	0.09919	0.02063	-0.28247	-0.14232	0.35565	0.12979	0.07722	0.05138	0.01229	0.04486	0.12617	0.17944	0.17654
-0.27637	0.17879	0.18324	0.13679	-0.11758	-0.16326	0.28113	0.12833	0.0793	0.02231	0.08301	0.05276	0.04278	0.21058	0.16923
-0.26219	0.13476	0.16197	0.15375	-0.33219	-0.12357	0.29779	0.13911	0.20281	-0.03338	0.08548	0.08322	0.04105	0.20643	0.16888

PHOSPH	N_SIGNAL	NETWOR	T_SIGNA	HMANIA	RANSEND	AND_PINE	IC_ACID	TERM	TERM_P	INE_DEG	G_LYSOS	SIGNAL	KET_DIAB	MELANOG
-0.01177	0.08016	-0.02145	-0.0256	0.08978	0.20875	0.28216	-0.0443	0.21161	0.18012	0.23572	0.12806	0.0859	0.29399	0.16039
-0.06815	0.07024	-0.01696	-0.05609	0.04345	0.17828	0.25688	-0.03364	0.13664	0.13787	0.20508	0.08207	0.05436	0.40356	0.15212
-0.02499	0.05945	0.04534	-0.00351	0.09084	0.21204	0.29014	-0.01457	0.19659	0.13972	0.22428	0.11614	0.07778	0.16664	0.15855
-0.05911	0.08743	0.104	-0.0004	0.15734	0.21324	0.23166	-0.01429	0.22191	0.17747	0.19117	0.20124	0.08038	0.19412	0.16782
0.00142	0.08774	0.14763	0.01393	0.14653	0.19539	0.24316	-0.10992	0.21136	0.17502	0.18786	0.15393	0.10602	0.32376	0.16132
-0.04396	0.06569	0.02591	-0.02503	0.03879	0.16493	0.24008	0.21418	0.15523	0.13508	0.18878	0.09072	0.06378	0.27585	0.13902
-0.06707	0.07169	0.11858	0.00415	0.09802	0.18163	0.25997	0.20189	0.19066	0.12699	0.20622	0.10972	0.07486	0.43685	0.13344
-0.01417	0.08197	0.12374	0.03508	0.17161	0.20341	0.22244	-0.02787	0.18443	0.15745	0.19177	0.21002	0.09621	0.14233	0.15286
-0.00576	0.08098	0.23756	0.03464	0.20952	0.20716	0.24245	-0.2886	0.16874	0.1366	0.1963	0.23028	0.09546	0.17315	0.13344
-0.02673	0.08037	0.12068	0.02839	0.16334	0.19758	0.23361	-0.08616	0.20157	0.15901	0.17905	0.19307	0.10314	0.39546	0.14239
-0.00642	0.06284	0.00099	-0.04983	0.05387	0.20019	0.28721	-0.11709	0.14346	0.11238	0.22751	0.0195	0.06216	0.40585	0.11865
-0.06789	0.06278	0.07017	-0.03233	0.07095	0.17845	0.27072	0.05243	0.19645	0.1381	0.23351	0.10053	0.0771	0.21737	0.13543
-0.04904	0.07064	-0.07839	-0.04444	-0.06164	0.16366	0.21188	-0.05406	0.19444	0.12999	0.19291	0.01757	0.06146	0.20715	0.13821
-0.04604	0.09644	0.13456	-0.04464	0.14149	0.21051	0.28547	-0.08508	0.1758	0.16038	0.21721	0.16523	0.08462	0.11185	0.17054
-0.0369	0.07798	0.13936	-0.06045	0.13391	0.19557	0.28856	-0.01058	0.17339	0.14834	0.20756	0.11292	0.07494	0.32623	0.14285
-0.04139	0.07145	0.02727	-0.06089	0.00308	0.15366	0.32568	-0.10614	0.16686	0.13208	0.24886	0.0079	0.05653	0.42293	0.16824
-0.03907	0.08389	0.0577	-0.017	0.10092	0.21358	0.26475	-0.11012	0.21904	0.14439	0.19498	0.17898	0.06742	0.36252	0.14957
-0.02339	0.08679	0.16624	0.02936	0.16346	0.19798	0.29175	-0.05411	0.1886	0.16592	0.22209	0.19163	0.09552	0.22301	0.16181

G_MELAN	NOBIOTI	SMATCH	SIGNAL	IR_CELL_M	AND_RI	PHIN_SIG	D_NICOT	ROGEN_M	CEPTOR	IOLOGO	LL_CELL	SIGNAL	OTIDE_EX
0.04691	0.07942	-0.01187	0.02157	0.09897	0.14172	0.11892	0.05867	0.05548	0.088	0.27327	-0.04298	0.02199	-0.02867
-0.01083	-0.02872	0.0667	-3.4E-05	0.03774	0.03811	0.10824	-0.0288	-0.00017	0.03842	0.30297	-0.08114	0.07306	0.04359
0.05256	0.01612	0.07596	0.04276	0.08498	0.08448	0.10646	0.03922	0.10109	0.07129	0.3196	-0.05172	0.07346	0.01944
0.05446	0.04729	0.03179	0.06749	0.11493	0.15942	0.12923	0.05774	0.05659	0.0985	0.29831	-0.03309	0.06076	-0.01203
0.04218	0.0225	-0.00029	0.02149	0.15189	0.16215	0.13314	0.05804	0.09702	0.0826	0.27758	-0.01206	0.01618	-0.00999
-0.04388	0.01383	0.0718	0.01248	0.09376	0.06789	0.11671	0.01382	0.19507	0.0158	0.26173	-0.08167	0.03391	0.05022
0.03326	0.01576	0.01998	0.06341	0.105	0.1607	0.11394	0.04179	0.07017	0.06571	0.3231	-0.05595	0.04034	0.01297
0.03919	0.05742	-0.03472	0.06057	0.13789	0.18829	0.13814	0.07389	0.14674	0.10392	0.25727	-0.0406	0.04495	-0.05524
0.03553	0.04344	-0.03169	0.05792	0.14474	0.21171	0.12359	0.0921	0.07931	0.11885	0.2794	-0.0319	0.04144	-0.06615
0.02149	0.04994	0.02356	0.03657	0.16283	0.20408	0.13465	0.05315	0.06844	0.11027	0.2775	-0.03943	0.01498	-0.00048
0.04918	0.08991	0.05557	0.01723	0.03117	0.06814	0.08725	0.07256	0.04108	0.06303	0.3146	-0.05873	0.05089	0.01233
0.03205	0.08528	0.06515	0.01932	0.05573	0.09172	0.10777	0.0289	0.1079	0.07177	0.32155	-0.05677	0.03418	0.01971
0.06011	-0.00158	0.09288	0.05578	-0.01494	0.03342	0.09218	-0.00918	0.04242	0.03382	0.34034	-0.04311	0.07972	0.05068
0.0002	0.06911	-0.0364	0.02945	0.0814	0.14954	0.13057	0.05402	0.07073	0.08525	0.30135	-0.0828	0.05211	-0.03767
0.00706	0.08567	0.01822	0.02412	0.07322	0.11181	0.115	0.08056	0.0824	0.07309	0.31189	-0.08752	0.03168	-0.008
0.04288	0.07966	0.05873	0.02063	-0.01544	-0.0383	0.10336	0.02295	0.03187	0.06977	0.39321	-0.0605	0.04474	0.02897
0.08405	0.0517	-0.05754	0.04952	0.08085	0.13677	0.11484	-0.07645	0.05695	0.06037	0.29855	-0.01422	0.07778	-0.06842
0.05744	0.03724	-0.06153	0.048	0.14441	0.16192	0.14033	0.07388	0.08926	0.08432	0.31139	-0.02985	0.02633	-0.03807

YCAN_BI	TORY_TR	BON_P	ODOCYTE	GLYCAN	IVE_PHOS	YCAN_BI	SIGNALIN	CREATI	TE_AND	KINSON	ESCHERI	HWAYS_IN	CURON	E_PHOSP
0.05795	0.16798	0.13755	0.15586	0.06274	0.22475	-0.2363	-0.05678	0.05159	0.01533	0.20646	0.29582	0.06139	0.18153	0.26615
0.09972	0.21434	0.1191	0.14221	0.02532	0.21121	-0.14012	-0.08745	0.02675	0.02038	0.20814	0.27928	0.05364	0.13552	0.25845
0.02821	0.1761	0.1653	0.13124	0.07952	0.2124	-0.01509	-0.04547	0.04274	0.01908	0.18689	0.29788	0.06879	0.20556	0.27848
0.07906	0.18807	0.14236	0.15099	0.14999	0.22775	-0.19834	-0.0139	0.05297	-0.00651	0.19931	0.30313	0.04912	0.15984	0.27291
0.05149	0.18461	0.10693	0.13745	0.10459	0.18397	-0.28072	-0.06805	0.07535	0.03491	0.1794	0.28887	0.06145	0.15608	0.284
0.06941	0.20717	0.14319	0.14689	0.08345	0.19053	-0.14303	-0.0257	0.00817	0.01764	0.21499	0.2766	0.05544	0.16836	0.2449
0.07334	0.14506	0.18791	0.1192	0.0752	0.21876	-0.18557	-0.0197	0.06431	0.01524	0.21681	0.2916	0.06658	0.16258	0.25246
0.1012	0.17556	0.0786	0.13104	0.14873	0.22348	-0.13712	-0.04036	0.05906	0.07664	0.19482	0.30595	0.04128	0.21359	0.28048
0.08092	0.18134	0.0867	0.11506	0.15589	0.2235	-0.19016	-0.03343	0.05973	0.03826	0.2002	0.29546	0.04996	0.17662	0.28012
0.05435	0.17927	0.12829	0.12085	0.10956	0.23814	-0.1866	-0.05003	0.04791	0.01934	0.21853	0.28533	0.04078	0.16383	0.26536
0.08276	0.14071	0.20529	0.13568	-0.04366	0.23536	-0.09217	0.01391	0.05009	0.05641	0.22474	0.30805	0.08222	0.17564	0.29011
0.0668	0.15687	0.13719	0.13583	0.0515	0.20064	-0.04143	-0.00386	0.03817	-0.04555	0.19355	0.30762	0.06156	0.1813	0.25931
0.08927	0.0985	0.16611	0.14789	-0.05573	0.15685	-0.23283	-0.03417	0.05614	0.14228	0.13451	0.31981	0.09442	0.16969	0.25386
0.0985	0.23574	0.07433	0.14396	0.16563	0.20714	-0.06392	-0.08862	0.03711	0.10343	0.18467	0.31357	0.04419	0.20317	0.28761
0.06992	0.18803	0.16984	0.13512	0.07669	0.19553	-0.11187	-0.00856	0.00626	-0.01455	0.18371	0.30258	0.04264	0.24417	0.26958
0.0236	0.1814	0.24491	0.13668	-0.02766	0.19964	-0.19523	-0.01916	0.02896	-0.01403	0.20183	0.30191	0.0843	0.20166	0.27263
0.04543	0.13179	0.14022	0.12352	0.13512	0.24885	-0.21472	-0.05161	0.06194	-0.00301	0.22154	0.32342	0.06555	0.16794	0.27611
0.05705	0.21434	0.13929	0.13789	0.11508	0.2472	-0.23833	-0.05823	0.06252	0.0353	0.2213	0.31798	0.04842	0.2088	0.27302

PEROXI	ALANINE	INOSITO	ID_CHLOR	SIGNALI	BILE_ACID	Y_IMMUN	PRION_DI	MEDIATE	ANOATE_	ROSTATE	PROTEA	ROTEIN_	LE_BICAR	RINE_ME
0.06679	0.19914	-0.0017	0.14181	0.06428	0.11021	-0.06874	0.11176	0.08196	0.18446	0.06554	0.29479	0.16132	0.24714	0.049
0.06615	0.23232	-0.03019	0.14773	0.03847	0.08114	-0.05233	0.12054	0.06492	0.12798	0.04445	0.29142	0.17774	0.18833	0.04448
0.062	0.23682	-0.02138	0.15072	0.07594	0.07482	-0.07403	0.16255	0.07869	0.16632	0.05669	0.26996	0.11596	0.27299	0.059
0.08184	0.21389	0.01539	0.16444	0.11795	0.10574	-0.00825	0.20209	0.10594	0.11566	0.06658	0.28132	0.17568	0.21751	0.04258
0.05878	0.21138	0.04172	0.1773	0.1022	0.11348	0.0774	0.16117	0.09181	0.12246	0.08031	0.3161	0.15618	0.19897	0.07016
0.05934	0.2211	0.006	0.1738	0.07862	0.03734	0.05148	0.26573	0.05982	0.11345	0.03118	0.28932	0.17012	0.24445	0.06747
0.06644	0.22758	-0.02208	0.12994	0.06877	0.0116	0.05786	0.29278	0.09713	0.10228	0.06602	0.27786	0.13412	0.23174	0.07838
0.09106	0.28613	0.01478	0.18716	0.14574	0.13104	0.02795	0.21375	0.08812	0.12576	0.06031	0.30283	0.18403	0.22053	0.04299
0.06664	0.2226	0.02094	0.16797	0.13715	0.10909	0.05397	0.26033	0.0854	0.11128	0.05301	0.30883	0.16723	0.22618	0.04709
0.04217	0.19557	0.02436	0.15977	0.1125	0.05313	0.13436	0.24052	0.07576	0.12267	0.05001	0.29385	0.16009	0.22411	0.06862
0.08517	0.20795	-0.01196	0.11368	0.05817	0.01498	-0.13686	0.17184	0.08657	0.16957	0.06046	0.27719	0.13682	0.21824	0.08201
0.08692	0.21343	-0.01402	0.15597	0.08408	-0.04259	-0.00195	0.24193	0.0699	0.15002	0.03125	0.29404	0.15116	0.25058	0.09638
0.06455	0.15997	-0.07295	0.11052	0.02174	-0.00695	-0.17108	0.17326	0.09976	0.1121	0.07245	0.289	0.1203	0.15181	0.06946
0.10196	0.23903	0.00403	0.17656	0.12331	0.09053	-0.01463	0.1785	0.06252	0.16548	0.05241	0.28316	0.15555	0.22496	0.04631
0.04453	0.26728	-0.0132	0.12693	0.06214	0.00965	-0.09032	0.17356	0.05902	0.19228	0.04042	0.28854	0.14577	0.2419	0.06916
0.09376	0.1858	-0.03795	0.09951	0.03281	0.00588	-0.16723	0.09618	0.05202	0.19025	0.06112	0.27492	0.08851	0.26712	0.08091
0.05785	0.21954	0.00353	0.16154	0.09386	0.07134	-0.08272	0.12604	0.09748	0.1588	0.06531	0.28175	0.16245	0.21922	0.03942
0.06387	0.22038	0.03375	0.15323	0.11371	0.14406	0.0477	0.18838	0.09247	0.15247	0.07322	0.30442	0.19176	0.21052	0.05357

L_CELL_	ANGIOTE	TINOL_MB	FLAVIN_	NG_RIBOS	CEPTOR_	NA_DEGR	NA_POLY	MINO_AC	_CELL_L	IONS_IN_	GOLIPID_	_SPLICE	OID_SUCR	ROID_BIO	HORMON	LFUR_ME	_LUPUS_	ITE_TRAN	_BACKBO	TA_SIGNA
0.13179	0.11816	-0.00113	0.01281	0.40336	-0.00449	-0.00362	-0.07048	0.14113	-0.07126	0.01849	-0.01418	0.20177	0.24755	-0.03558	0.06364	0.07752	0.27562	0.2187	0.04232	0.0723
0.08093	0.01098	-0.03768	0.04092	0.41996	-0.03974	0.09349	-0.05613	0.15137	-0.08813	0.0118	-0.00948	0.27921	0.16822	-0.1344	-0.19407	-0.07254	0.27095	0.17379	-0.00636	0.07528
0.12803	0.11141	-0.01538	-0.02691	0.40608	-0.0404	0.02019	-0.04645	0.13903	-0.07484	0.06024	-0.02833	0.2294	0.23525	-0.03166	-0.10568	0.02134	0.29873	0.14347	-0.03506	0.07926
0.13137	0.19082	-0.02196	-0.00544	0.42	-0.02378	0.01773	-0.11035	0.08857	-0.09424	0.09969	0.10074	0.20091	0.21642	-0.00148	-0.12764	0.07726	0.34164	0.22485	-0.0372	0.08757
0.14033	0.11852	-0.04405	0.01749	0.41232	-0.0039	0.02675	-0.10302	0.12267	-0.09046	0.0561	0.00467	0.2149	0.21	-0.04543	-0.01369	0.08712	0.32019	0.21359	0.00949	0.07024
0.1095	0.09949	-0.03017	-0.07121	0.41978	-0.06408	0.01772	-0.09613	0.07473	-0.08397	0.02236	0.04107	0.23848	0.20101	-0.09089	-0.18586	-0.18653	0.339	0.10679	-0.01672	0.07848
0.13309	0.0859	-0.11062	0.0134	0.41818	-0.02831	0.04109	-0.03832	0.12557	-0.04983	-0.00756	-0.02138	0.2131	0.18966	-0.02719	-0.15057	-0.09351	0.35395	0.20883	-0.02629	0.13234
0.14165	0.17175	0.00343	-0.0107	0.41528	-0.02116	0.00014	-0.1613	0.09398	-0.06832	0.11537	0.07185	0.17569	0.24484	-0.08024	-0.1098	0.147	0.33672	0.20034	-0.01031	0.08423
0.12403	0.18179	-0.03227	-0.01114	0.40916	0.00493	-0.00668	-0.13549	0.1234	-0.07592	0.09961	0.10814	0.1874	0.22158	-0.03732	-0.10337	0.10613	0.3573	0.20184	0.0085	0.07437
0.12075	0.16986	-0.03641	-0.00112	0.40464	0.01791	0.02509	-0.09725	0.11026	-0.1013	0.08793	0.04369	0.1969	0.20224	-0.09264	-0.05166	0.04111	0.34763	0.20664	-0.0082	0.06085
0.12609	0.07882	-0.03482	-0.04231	0.41745	-0.0372	0.03431	-0.07772	0.12179	-0.04892	-0.00574	-0.02871	0.22516	0.24164	-0.02483	-0.00926	-0.01583	0.29908	0.14798	-0.02464	0.1365
0.09733	0.12854	-0.03931	-0.01911	0.42098	-0.00727	0.00077	-0.07885	0.11916	-0.06821	-0.00038	-0.01059	0.21268	0.20454	-0.05188	-0.05077	-0.07385	0.32994	0.18504	0.11073	0.08277
0.14409	0.0044	-0.04844	-0.01011	0.40828	-0.041	0.02692	-0.04926	0.08934	-0.05543	-0.033	-0.03421	0.22909	0.1695	0.06392	-0.11339	-0.12279	0.26051	0.17741	0.08278	0.14173
0.11919	0.11266	0.03474	-0.00142	0.42034	-0.01794	0.02286	-0.09663	0.11595	-0.10912	0.07432	0.05912	0.2173	0.22608	-0.02225	-0.07892	0.07067	0.33011	0.17004	-0.04507	0.09284
0.10338	0.13552	-0.01716	-0.00521	0.42604	-0.0246	0.0239	-0.0809	0.13989	-0.10742	0.02274	7.8E-05	0.22719	0.26582	-0.05405	-0.0959	0.12382	0.31642	0.14492	0.08914	0.09562
0.13164	0.04614	-0.12833	-0.00411	0.41667	-0.02815	0.03497	-0.04023	0.15895	-0.0438	-0.0215	-0.0266	0.20978	0.21	-0.04528	-0.05781	-0.10869	0.24701	0.08005	0.03102	0.11709
0.13928	0.16539	-0.06542	0.00765	0.42172	-0.05332	0.01247	-0.08609	0.14554	-0.06896	0.05144	0.0544	0.22697	0.21453	-0.08833	-0.08336	0.02803	0.30499	0.2239	-0.04317	0.06678
0.14429	0.17438	0.04235	0.00224	0.4031	-0.00803	0.02687	-0.11352	0.11612	-0.08135	0.09648	0.06268	0.18768	0.24775	-0.07692	-0.05949	0.11733	0.33046	0.20113	-0.02672	0.06375

HYROID_	IGHT_JU	CEPTOR_	TOPHAN_	I_DIABET	I_DIABET	OSINE_M	EPTOR_SI	_MEDIAT	AND_ISO	AND_ISO	OOTH_MU	GULATED_	SIGNALI	CHOLER	RAL_MYO
0.23804	0.17551	-0.02502	0.19078	0.16489	0.09639	0.10398	0.01032	0.00749	0.22577	0.15972	0.19445	0.25252	0.09892	0.2029	0.17732
0.22173	0.18511	-0.0572	0.18916	0.16333	0.04945	0.11158	-0.04441	0.01975	0.22167	0.12821	0.15765	0.24571	0.01903	0.19686	0.15801
0.22936	0.17119	-0.01295	0.19877	0.14063	0.13034	0.11383	-0.01366	0.01451	0.18158	0.16934	0.17832	0.20375	0.06242	0.20856	0.18224
0.1898	0.15995	0.0262	0.16816	0.20349	0.15569	0.12154	0.01337	0.00817	0.17254	0.10231	0.19875	0.26074	0.10504	0.2328	0.1992
0.18114	0.14399	0.01478	0.19591	0.1775	0.2035	0.10163	0.05239	0.01168	0.19286	0.12466	0.17501	0.25257	0.12387	0.19596	0.21177
0.1489	0.1347	-0.04391	0.17191	0.09856	0.13419	0.07561	0.01257	0.02771	0.18784	0.12088	0.16876	0.21765	0.06431	0.13599	0.1736
0.17526	0.15334	0.0082	0.1851	0.20523	0.14689	0.09897	0.02595	0.00876	0.18421	0.11823	0.17238	0.17588	0.09781	0.16215	0.19366
0.1657	0.1205	0.02723	0.17382	0.18097	0.17348	0.16936	0.02579	0.00357	0.21021	0.13198	0.16387	0.261	0.09991	0.23016	0.20682
0.14018	0.12421	0.0712	0.1815	0.19197	0.28788	0.11268	0.02608	0.00066	0.16072	0.09756	0.16547	0.25299	0.1035	0.22308	0.26459
0.15125	0.11082	0.04173	0.16961	0.1788	0.19935	0.09483	0.05152	-0.00421	0.17856	0.11642	0.18135	0.23901	0.10793	0.20596	0.23104
0.20052	0.16973	-0.06805	0.16982	0.15187	0.09836	0.06629	-0.03701	0.05238	0.24834	0.18604	0.17393	0.25055	0.04768	0.1982	0.14647
0.18713	0.15302	-0.02907	0.1775	0.11291	0.18315	0.10158	-0.01866	0.01174	0.21763	0.1707	0.19249	0.24575	0.06791	0.18044	0.20816
0.24375	0.22674	-0.07215	0.12247	0.11811	-0.01437	0.04965	-0.05081	0.06965	0.27782	0.11292	0.1987	0.24771	0.05427	0.1965	0.1282
0.19312	0.17398	-0.00097	0.21676	0.16797	0.17729	0.13204	0.0055	0.02076	0.24449	0.16936	0.18481	0.25074	0.07426	0.22211	0.19481
0.20331	0.15343	-0.02865	0.19213	0.15634	0.17745	0.17062	-0.02246	0.00252	0.21505	0.16439	0.19291	0.25549	0.04722	0.17783	0.19939
0.23978	0.20295	-0.06186	0.20661	0.12895	0.10389	0.10436	-0.0425	0.04973	0.24971	0.17426	0.18819	0.21396	0.03473	0.1609	0.16522
0.21639	0.18416	-0.01353	0.18465	0.1931	0.10541	0.12854	-0.00836	-0.00288	0.19639	0.16657	0.17529	0.25114	0.09066	0.21906	0.17734
0.19406	0.13469	0.02943	0.20725	0.19192	0.21974	0.13221	0.03909	0.00342	0.1727	0.13556	0.17388	0.25912	0.12601	0.22722	0.2155

SIGNALI	NIC_TO_E	D_A_PYRE	BPA_TO_F	ACTOR_	E2_TO_	ION_TO_AN	METALS_	TALS_TO_	METALS_	ETALS_TO	OTINE_N	OTINE_N	NNN_TO	NNN_TO	NNN_TO
0.03681	0.30208	0.0731	0.22066	0.12004	0.18251	0.19861	-0.3402	0.11345	0.04531	0.18563	0.28476	-0.02502	-0.11899	-0.07117	-0.28439
0.01736	0.28235	-0.04246	0.14629	-0.01096	0.11105	0.27058	-0.32811	0.03245	0.05344	0.10071	0.24362	-0.01589	-0.10271	-0.09503	-0.25297
0.06823	0.27007	-0.00281	0.1815	0.04128	0.14759	0.23934	-0.34438	0.08485	0.0782	0.13972	0.23074	0.03063	-0.074	-0.03828	-0.16005
0.04726	0.30682	-0.02836	0.20257	0.0633	0.16735	0.27197	-0.41211	0.12344	0.0832	0.14685	0.28597	0.06906	0.03337	-0.01209	-0.11348
0.04523	0.29145	-0.00594	0.23802	0.08558	0.19925	0.23932	-0.39848	0.12275	0.10539	0.19278	0.26593	0.06087	0.05429	0.00817	-0.12589
0.04871	0.28784	0.07626	0.09972	0.03816	0.06723	0.2371	-0.29034	0.04766	0.04495	0.05647	0.22411	0.04044	-0.10322	-0.01354	-0.14172
0.04058	0.2926	0.00311	0.18713	0.05455	0.14816	0.23294	-0.32145	0.01782	0.06552	0.13426	0.26974	0.05972	-9.5E-05	0.02008	-0.1084
0.03904	0.29511	-0.01127	0.19701	0.06956	0.15819	0.27699	-0.39503	0.14593	0.10707	0.13531	0.29182	0.06396	0.08143	-0.00645	-0.11665
0.03376	0.28934	0.00375	0.17776	0.08931	0.13383	0.27615	-0.37255	0.11591	0.08291	0.10414	0.29356	0.07545	0.06338	-0.00248	-0.08935
0.02178	0.31678	0.0125	0.18953	0.10964	0.15027	0.26485	-0.36157	0.09921	0.12052	0.12702	0.28112	0.05517	0.02972	0.00013	-0.11447
0.06411	0.27769	0.01828	0.12685	0.1301	0.09619	0.30863	-0.43056	0.0011	-0.00839	0.08904	0.26612	0.00178	-0.08062	-0.09047	-0.25252
0.04263	0.30975	0.01384	0.14453	0.16431	0.11091	0.27524	-0.31087	-0.03611	-0.00616	0.11146	0.28206	0.01765	-0.13943	-0.08029	-0.18662
0.07867	0.24108	0.05425	0.19076	-0.00488	0.15505	0.32548	-0.39542	-0.03102	-0.01385	0.1687	0.29837	0.02997	-0.20371	-0.05863	-0.21083
0.04341	0.2771	-0.00827	0.17211	0.09883	0.12979	0.29319	-0.40907	0.06466	0.08433	0.11771	0.27529	0.03861	-0.00079	-0.00492	-0.13662
0.03031	0.266	-0.00112	0.1971	0.141	0.15632	0.25681	-0.39111	0.01827	0.05082	0.15346	0.28615	-0.03763	-0.05891	-0.09421	-0.27145
0.05562	0.23949	0.0263	0.18455	0.15405	0.15099	0.29835	-0.33596	-0.0543	-0.01153	0.15954	0.18421	0.04684	-0.15185	-0.04168	-0.18681
0.03695	0.3348	-0.0153	0.19896	0.1075	0.16458	0.31069	-0.39443	0.09074	0.0497	0.15157	0.30642	0.07343	-0.03148	-0.01283	-0.10337
0.0471	0.33057	0.00371	0.1966	0.04822	0.15972	0.30767	-0.41951	0.10908	0.08174	0.14274	0.27316	0.0636	0.03671	0.00965	-0.09824

K_NNN_T	ACTOR_N	ACTOR_PR	TCDD_	N_TO_AN	IC_TO_EL	BARF1_T	EBNA1_T	NA2_TO_	EBV_EB	EBNA3C_	EBNA3C_	EBNA3C_	V_LMP1_	IA_EAE_T	CHIA_EAE
0.18563	0.36692	0.07296	0.08117	0.19667	0.31556	0.1081	-0.0489	-0.16146	-0.42875	-0.30461	-0.33377	-0.02344	-0.04468	0.33991	0.30464
0.10071	0.39616	-0.15694	-0.07313	0.23391	0.31388	0.14052	0.05384	-0.10665	-0.35124	-0.19257	-0.17977	0.02961	-0.1021	0.2951	0.26376
0.13972	0.42012	0.02146	-0.03318	0.23542	0.32354	0.14085	0.06971	-0.11711	-0.24203	-0.1998	-0.23569	0.06827	-0.06874	0.32029	0.28993
0.14685	0.39317	0.10777	-0.0144	0.26386	0.27592	0.13988	0.03371	-0.18382	-0.44178	-0.30642	-0.3242	0.02895	-0.05097	0.30861	0.28326
0.19278	0.41783	0.23265	0.00759	0.23848	0.28924	0.17959	0.05832	-0.04423	-0.21684	-0.1814	-0.23284	0.05354	-0.01538	0.30066	0.29625
0.05647	0.37837	-0.20712	-0.0277	0.21035	0.29773	0.06563	-0.02158	-0.05752	-0.32077	-0.14133	-0.22631	-0.03069	-0.11252	0.32976	0.28949
0.13426	0.41201	-0.14292	-0.0097	0.2308	0.35148	0.16111	0.05056	-0.21949	-0.40373	-0.11482	-0.18806	0.06746	-0.05086	0.32724	0.28517
0.13531	0.40743	0.27036	-0.00777	0.27342	0.27988	0.15139	0.02922	-0.09735	-0.32975	-0.25176	-0.27133	0.04166	0.00374	0.30646	0.28741
0.10414	0.42188	0.19288	0.01091	0.27252	0.31106	0.19463	0.04112	-0.12021	-0.42223	-0.29897	-0.3454	0.04979	-0.03662	0.28423	0.28175
0.12702	0.42029	0.09827	0.0464	0.26129	0.34049	0.21076	0.06815	-0.06336	-0.32588	-0.16685	-0.2853	0.07057	0.00315	0.28233	0.27649
0.08904	0.43808	0.03665	0.05015	0.29292	0.32328	0.16144	0.06792	-0.12931	-0.46662	-0.05388	-0.02777	0.04737	-0.10882	0.32944	0.27826
0.11146	0.44266	-0.0874	0.06892	0.23955	0.33246	0.10664	0.04436	-0.16332	-0.45464	-0.28406	-0.3538	0.06305	-0.06843	0.36188	0.31346
0.1687	0.39651	-0.05017	-0.07739	0.31906	0.25954	0.13948	0.05401	-0.15414	-0.44495	-0.13787	-0.09174	0.07933	-0.13598	0.37707	0.30551
0.11771	0.39704	0.12604	0.02348	0.273	0.3209	0.11298	0.06653	-0.10806	-0.42558	-0.25756	-0.25786	0.04341	-0.01451	0.33485	0.29922
0.15346	0.42681	0.18097	0.05373	0.24466	0.32111	0.14432	0.03621	-0.11359	-0.4601	-0.19897	-0.19283	-0.00658	-0.04246	0.31338	0.29091
0.15954	0.45887	-0.09953	0.07108	0.27106	0.37294	0.11013	0.05943	-0.08254	-0.24573	-0.26913	-0.22175	0.02438	-0.10478	0.34873	0.28492
0.15157	0.36886	-0.01878	0.03345	0.28779	0.34186	0.1165	0.00587	-0.15722	-0.45905	-0.3325	-0.3621	-0.01276	-0.1104	0.34668	0.30424
0.14274	0.44495	0.24596	-0.02176	0.3038	0.30704	0.15576	0.05727	-0.13172	-0.36674	-0.26942	-0.30971	0.05877	-0.01453	0.30268	0.29043

ESPG_TO	RICHIA_E	RICHIA_M	HBX_TO_	HBX_TO	LHBS_T	GB_TO_PD	GH_TO_	UL33_TO	_GNAQ_P	UL33_TO_	UL33_TO	O_CXCR4	US28_TO_	US28_TO	_GNAQ_P
0.22757	0.36592	0.36825	0.02084	0.20285	0.14997	0.2079	0.19439	0.20319	0.09151	0.17269	0.16035	0.0387	0.07643	0.29966	0.10865
0.268	0.3615	0.34247	0.13596	0.14283	0.03164	0.13761	0.17457	0.2024	0.03273	0.15728	0.14368	-0.05777	0.01144	0.28557	0.03331
0.2695	0.36543	0.3604	0.1005	0.16795	0.10188	0.16361	0.23601	0.17338	0.14456	0.12375	0.10205	-0.04055	0.1248	0.27273	0.07668
0.29849	0.37601	0.35438	0.07255	0.19292	0.12543	0.17844	0.23963	0.21223	0.17081	0.15047	0.21367	-0.08438	0.10763	0.33632	0.10564
0.26941	0.38136	0.36098	0.10833	0.22205	0.15494	0.21905	0.22303	0.23649	0.09118	0.17605	0.17286	-0.06097	0.09855	0.32449	0.13144
0.23643	0.36964	0.35326	0.11518	0.09752	0.04887	0.09182	0.20552	0.19114	0.01299	0.13334	0.10194	0.01448	0.08192	0.28061	0.06658
0.26158	0.36075	0.36615	0.06575	0.17306	0.1259	0.16421	0.24826	0.18774	0.06145	0.14092	0.11623	0.01664	0.12018	0.30015	0.06308
0.30545	0.37603	0.35861	0.04926	0.18672	0.13158	0.17088	0.24886	0.22567	0.14631	0.16853	0.17814	-0.02029	0.08437	0.34013	0.1215
0.30454	0.37015	0.34438	0.05726	0.1707	0.108	0.1498	0.25237	0.2276	0.11164	0.18029	0.17225	-0.04661	0.15109	0.32848	0.12645
0.29447	0.37606	0.34482	0.05498	0.18048	0.13897	0.16445	0.24074	0.21993	0.11253	0.1719	0.19741	0.00995	0.06693	0.3184	0.09898
0.32622	0.36798	0.36259	0.15195	0.1057	0.05223	0.10074	0.20455	0.19655	0.10729	0.1612	0.09966	-0.02176	0.13262	0.28828	0.0772
0.27383	0.38081	0.39283	0.16519	0.13285	0.10497	0.13592	0.23166	0.23821	0.13735	0.19122	0.16472	-0.01262	0.11739	0.32174	0.08236
0.35086	0.40541	0.40629	0.09072	0.16606	0.06855	0.17889	0.21999	0.18321	0.08789	0.17914	0.13811	0.02792	0.12707	0.275	0.02693
0.30748	0.37398	0.37209	0.07213	0.16397	0.05671	0.15745	0.26093	0.19239	0.09626	0.15516	0.13945	-0.01474	0.13005	0.29658	0.08052
0.27828	0.35555	0.35681	0.13493	0.17988	0.14176	0.18016	0.24495	0.19754	0.04863	0.17781	0.1154	-0.01496	0.14887	0.28747	0.0498
0.30178	0.38569	0.37225	0.09721	0.15933	0.10748	0.16752	0.14429	0.1453	0.11967	0.1016	0.07965	-0.00256	0.10837	0.22485	0.07489
0.32077	0.38242	0.38374	0.06154	0.18607	0.09173	0.17754	0.22107	0.21897	0.14011	0.1854	0.20464	-0.04577	0.10417	0.32352	0.04833
0.33775	0.38766	0.36258	0.07494	0.18616	0.12081	0.17473	0.24389	0.20755	0.06874	0.1591	0.19019	-0.04209	0.11259	0.31143	0.13245

28_TO_G	V_CORE	RE_TO_R	TO_CXCR	120_TO_C	0_TO_CX	P120_TO_	NEF_TO_L	K_BETW	AT_TO_T	PR_TAT_	HIV_VPR_	CESSIN	5_TO_EG	E6_TO_N	E6_TO_N	E6_TO_N
0.06227	0.33324	0.21244	-0.02502	0.10995	0.26297	-0.10798	-0.10798	0.08277	0.108	-0.42452	-0.03357	0.25649	0.17266	-0.28615	-0.28615	0.00744
0.0744	0.27526	0.24291	-0.01589	0.06861	0.21638	-0.11943	-0.11943	0.09167	-0.00857	-0.41148	0.03258	0.21678	0.10525	-0.2321	-0.2321	0.0521
0.05411	0.26475	0.07381	0.03063	0.06125	0.2383	-0.10677	-0.10677	0.12055	0.07729	-0.45658	0.00343	0.24185	0.12529	-0.23636	-0.23636	0.04799
0.16148	0.30091	0.21983	0.06906	0.11212	0.29874	-0.08205	-0.08205	0.1456	0.03181	-0.44989	-0.054	0.28056	0.14159	-0.29028	-0.29028	0.04219
0.12558	0.30741	0.21244	0.06087	0.15492	0.2649	-0.05622	-0.05622	0.17828	0.13545	-0.42276	0.00312	0.26933	0.18371	-0.17414	-0.17414	0.09338
0.04786	0.2646	0.18637	0.04044	0.1085	0.22229	-0.11052	-0.11052	0.05438	0.13449	-0.31707	-0.02972	0.26876	0.06174	-0.36161	-0.36161	0.03623
0.09164	0.28237	0.1499	0.05972	0.06384	0.26014	-0.09262	-0.09262	0.13316	0.15627	-0.40144	-0.00287	0.26585	0.12493	-0.30251	-0.30251	0.0017
0.14852	0.31953	0.22917	0.06396	0.12551	0.29472	-0.03101	-0.03101	0.13572	0.10801	-0.42117	-0.12213	0.29576	0.13289	-0.17692	-0.17692	0.11333
0.12855	0.29236	0.21807	0.07545	0.11303	0.27337	-0.0692	-0.0692	0.17932	0.06288	-0.39528	-0.10367	0.32044	0.11212	-0.24983	-0.24983	0.06356
0.154	0.29396	0.15906	0.05517	0.11745	0.30471	-0.04083	-0.04083	0.1952	0.14817	-0.40268	-0.07432	0.34035	0.13	-0.23926	-0.23926	0.06734
0.01687	0.28165	0.24256	0.00178	0.10171	0.22567	-0.15708	-0.15708	0.12977	0.04422	-0.46468	0.02426	0.19845	0.1039	-0.18607	-0.18607	0.02266
0.05762	0.31674	0.27479	0.01765	0.10628	0.23787	-0.1256	-0.1256	0.07927	0.04161	-0.4527	-0.01234	0.31197	0.11364	-0.28947	-0.28947	-0.07955
0.04999	0.30789	0.22882	0.02997	0.028	0.24293	-0.14541	-0.14541	0.11921	-0.02023	-0.44143	0.02717	0.16292	0.1428	-0.23799	-0.23799	0.05529
0.09813	0.27626	0.2459	0.03861	0.08593	0.22678	-0.06167	-0.06167	0.10289	0.06146	-0.36833	-0.10487	0.19592	0.11959	-0.26917	-0.26917	-0.01546
0.04877	0.32655	0.15836	-0.03763	0.08248	0.24058	-0.08857	-0.08857	0.14245	0.06846	-0.44654	-0.07995	0.23596	0.14227	-0.24205	-0.24205	0.05576
-0.00669	0.30289	0.22459	0.04684	0.05138	0.15045	-0.16401	-0.16401	0.1351	0.02481	-0.45728	0.04255	0.25255	0.1598	-0.23968	-0.23968	0.02446
0.14889	0.294	0.18196	0.07343	0.06226	0.30107	-0.12979	-0.12979	0.11237	0.02965	-0.45658	-0.06038	0.17702	0.14035	-0.28013	-0.28013	0.03937
0.15378	0.3023	0.23956	0.0636	0.1433	0.28487	-0.05422	-0.05422	0.1459	0.13078	-0.43157	-0.01858	0.29529	0.13844	-0.30215	-0.30215	0.03592

EN_HPV_E	HPV_E7	V_E7_TO	GD_TO_H	ROCESSI	_1_P12_T	1_TAX_T	1_TAX_T	1_TAX_T	TO_SPIN	K15_TO	HV_LANA	PROCESS	2_TO_CEL	SHV_VC	V_VFLIP_	VFLIP_T
-0.42875	-0.33377	0.10659	-0.08987	0.25427	-0.08261	0.04006	-0.36067	-0.33377	-0.27792	-0.08569	0.086	0.22961	0.43932	-0.42875	0.00839	-0.04656
-0.35124	-0.17977	0.00814	-0.08165	0.20955	-0.25223	0.03442	-0.23813	-0.17977	-0.17966	-0.07541	0.06435	0.17751	0.3678	-0.35124	0.01871	-0.03866
-0.24203	-0.23569	0.1025	-0.12046	0.2521	-0.22778	0.10522	-0.28443	-0.23569	-0.25584	-0.11462	0.10241	0.22644	0.35829	-0.24203	-0.02345	-0.07857
-0.44178	-0.3242	0.11277	-0.08697	0.27386	-0.08345	0.15923	-0.35671	-0.3242	-0.23317	-0.08869	0.02539	0.24894	0.33874	-0.44178	0.01131	-0.0466
-0.21684	-0.23284	0.06563	-0.05043	0.27512	-0.01435	0.20147	-0.2796	-0.23284	-0.32795	-0.0501	-0.00518	0.25155	0.24855	-0.21684	0.05075	-0.00604
-0.32077	-0.22631	0.09124	-0.12305	0.26895	-0.0218	0.0608	-0.25426	-0.22631	-0.15072	-0.11615	0.07566	0.24648	0.16717	-0.32077	-0.03609	-0.086
-0.40373	-0.18806	-0.0094	-0.07764	0.26959	-0.10357	0.11362	-0.24627	-0.18806	-0.24479	-0.07275	0.056	0.23577	0.16576	-0.40373	0.01111	-0.03443
-0.32975	-0.27133	0.07817	-0.0441	0.30315	-0.03249	0.15593	-0.30961	-0.27133	-0.24208	-0.04027	-0.05644	0.27716	0.3176	-0.32975	0.06243	0.00299
-0.42223	-0.3454	0.08294	-0.09812	0.31981	-0.01395	0.28168	-0.36749	-0.3454	-0.28545	-0.09607	-0.03138	0.29849	0.42505	-0.42223	-0.00226	-0.05598
-0.32588	-0.2853	0.04663	-0.05245	0.34851	0.05731	0.15867	-0.31711	-0.2853	-0.28445	-0.05093	-0.16682	0.33193	0.21614	-0.32588	0.05468	-0.00692
-0.46662	-0.02777	-0.01732	-0.16555	0.18693	-0.28703	0.08889	-0.1422	-0.02777	-0.15137	-0.1476	0.10943	0.16041	0.41483	-0.46662	-0.07995	-0.12877
-0.45464	-0.3538	0.00526	-0.16103	0.31685	-0.12303	0.12409	-0.3812	-0.3538	-0.28718	-0.16031	-0.02218	0.29742	0.42205	-0.45464	-0.08288	-0.12736
-0.44495	-0.09174	0.15688	-0.19419	0.09965	-0.34716	-0.05565	-0.13009	-0.09174	-0.18405	-0.18812	0.13527	0.10389	0.31178	-0.44495	-0.16666	-0.16817
-0.42558	-0.25786	0.07542	-0.04967	0.18603	-0.17189	0.20252	-0.30124	-0.25786	-0.24992	-0.04715	0.02445	0.14616	0.38471	-0.42558	0.06239	0.00013
-0.4601	-0.19283	0.06464	-0.09746	0.2327	-0.1823	0.21381	-0.25048	-0.19283	-0.27521	-0.09197	0.06138	0.21712	0.29223	-0.4601	-0.00501	-0.05964
-0.24573	-0.22175	0.08699	-0.1533	0.25408	-0.28492	0.08849	-0.23548	-0.22175	-0.25485	-0.14634	0.12981	0.22815	0.22811	-0.24573	-0.11852	-0.1279
-0.45905	-0.3621	0.09072	-0.12171	0.16689	-0.16564	0.11863	-0.38842	-0.3621	-0.1796	-0.12601	0.01658	0.12606	0.38999	-0.45905	-0.04289	-0.08618
-0.36674	-0.30971	0.03988	-0.06404	0.28982	-0.04754	0.20401	-0.34138	-0.30971	-0.26057	-0.05918	0.07839	0.26358	0.33926	-0.36674	0.04261	-0.02003

GPCR_T	CR_TO_	CR_TO_G	A_AVRA_	_TO_MIC	TO_TETH	S_TO_ANX	SOPB_TO	NELLA_S	A_SOPE_	NELLA_S	COV_2_	N_TO_LE	2_S_TO_	TO_CLAS	ALK_BET	A_TO_IT
0.20282	0.10733	0.17161	0.33468	0.15302	-0.03287	0.45574	0.22409	0.26683	0.07381	0.29118	0.40474	-0.03312	0.05735	-0.13921	0.31058	0.23989
0.17307	0.12259	0.15381	0.31865	0.18882	-0.01183	0.46709	0.21647	0.28166	0.08532	0.2896	0.42905	-0.16857	-0.01348	-0.07694	0.33228	0.21153
0.16793	0.09406	0.14537	0.38788	0.15921	0.05089	0.45527	0.22828	0.29608	0.03029	0.29858	0.41097	-0.25823	0.08984	0.16693	0.31603	0.26686
0.24844	0.22804	0.25349	0.25031	0.21382	0.06602	0.46987	0.26533	0.24909	0.08866	0.3131	0.42354	0.06729	0.08336	0.24082	0.3616	0.22
0.2295	0.15079	0.22188	0.16998	0.199	0.0833	0.46028	0.25752	0.26742	0.11581	0.30987	0.42296	0.18478	0.11895	0.08201	0.35627	0.24492
0.11669	0.07788	0.15011	0.25242	0.17881	-0.04717	0.45949	0.23781	0.27918	0.03753	0.30422	0.43059	-0.29311	0.05113	0.42609	0.29725	0.22978
0.17977	0.11805	0.18512	0.30262	0.14766	-0.01096	0.42457	0.24785	0.24894	0.0877	0.30647	0.42771	-0.1529	0.11322	0.47907	0.34277	0.21945
0.22543	0.17482	0.23956	0.06377	0.22809	0.10913	0.47086	0.27298	0.25324	0.12712	0.31819	0.42874	0.11996	0.12609	0.26438	0.35474	0.22576
0.19385	0.16494	0.22221	0.12524	0.20394	0.10847	0.46067	0.2616	0.27228	0.07251	0.30711	0.41674	0.13652	0.21651	0.29738	0.37045	0.2496
0.227	0.17723	0.25627	-0.09301	0.21761	0.05653	0.4644	0.26061	0.23755	0.12194	0.30842	0.4076	0.14832	0.14196	0.28696	0.355	0.21203
0.1335	0.07393	0.12567	0.35177	0.21597	-0.01341	0.4758	0.24508	0.32171	-0.00147	0.31054	0.42264	-0.20416	0.01883	0.04633	0.32926	0.29793
0.17099	0.11101	0.16151	0.192	0.18373	-0.05881	0.47052	0.2398	0.27884	0.00572	0.3099	0.42497	-0.15413	-0.0207	0.17752	0.32092	0.25333
0.21041	0.15464	0.16383	0.36287	0.27248	-0.05721	0.47281	0.28761	0.35222	-0.03016	0.35113	0.41416	-0.22301	-0.12359	0.00701	0.26737	0.31281
0.17335	0.12511	0.16439	0.27779	0.21437	0.05162	0.47122	0.26192	0.29475	0.11629	0.31764	0.42694	-0.02766	0.0843	0.1518	0.35474	0.25845
0.17399	0.08315	0.1497	0.30351	0.2085	0.00244	0.45743	0.21807	0.31822	0.06089	0.28869	0.42636	-0.01022	0.07296	0.09961	0.30793	0.29417
0.14995	0.07079	0.09657	0.36023	0.21523	-0.02909	0.46933	0.26813	0.34868	-0.02674	0.33283	0.42734	-0.38612	-0.0887	-0.20644	0.28337	0.29116
0.25445	0.22463	0.24601	0.20927	0.21152	0.03593	0.46375	0.26468	0.26743	0.0461	0.32374	0.42816	0.02995	0.03392	-0.03405	0.34988	0.22689
0.23338	0.19098	0.24252	0.29893	0.24335	0.10504	0.46411	0.26437	0.27445	0.11159	0.31827	0.41708	-0.00846	0.13071	0.17672	0.3706	0.24742

C_D_TO_	ELLA_I	PGD_TO_A	OSPF_T	LA_OSPG_	YOPP_J_	OTEASOM	ACH_CH	CH_CHRN	ACTH_C	ON_OF_P	F2.2_BY_	UNCE_ACT	IE_ADRB3
0.2564	0.35979	0.20948	0.33343	0.17905	0.02041	0.30292	-0.25339	0.17855	0.10872	0.10156	0.04967	-0.17583	0.14709
0.25435	0.34953	0.20338	0.22925	0.14323	-0.05752	0.3011	-0.21783	0.11291	0.1573	0.15715	0.12189	0.02903	0.19227
0.27128	0.36169	0.22158	0.21834	0.15835	-0.02116	0.28671	-0.10927	0.12927	0.09757	0.11297	0.07887	-0.09299	0.14035
0.26246	0.38042	0.26139	0.26145	0.16375	-0.0625	0.2853	-0.05665	0.13004	0.11794	0.10437	0.04454	-0.13803	0.11084
0.22626	0.3895	0.25293	0.24374	0.1526	0.04581	0.32699	-0.06944	0.19384	0.1338	0.11722	0.05406	-0.08796	0.16361
0.25379	0.37023	0.22697	0.12129	0.19774	-0.01581	0.30346	-0.09971	0.06672	0.07682	0.1322	0.12842	-0.13639	0.14482
0.22064	0.36253	0.24262	0.21419	0.17039	0.00843	0.28399	-0.04991	0.1219	0.08196	0.10261	0.06912	-0.01492	0.10558
0.26184	0.38124	0.27164	0.23614	0.10793	-0.01984	0.30868	-0.05902	0.13003	0.11138	0.08769	0.01629	-0.125	0.11701
0.25242	0.36713	0.26179	0.22225	0.03281	-0.02838	0.31419	-0.01909	0.11342	0.119	0.08401	0.03017	-0.08541	0.13362
0.24896	0.36525	0.25919	0.2464	0.08803	0.02131	0.29905	-0.0564	0.12895	0.11031	0.1154	0.05186	-0.18097	0.09619
0.25498	0.37412	0.22724	0.20326	0.24105	-0.10365	0.29359	-0.21518	0.09171	0.13504	0.137	0.069	0.03493	0.12441
0.25212	0.38211	0.22758	0.28738	0.13763	-0.02568	0.29369	-0.13909	0.12232	0.13256	0.11457	0.05217	-0.12804	0.14462
0.23965	0.39046	0.26812	0.22811	0.20811	-0.01765	0.31604	-0.16432	0.16159	0.14998	0.13905	0.11193	0.03642	0.20106
0.23864	0.37631	0.25589	0.24981	0.1612	-0.04675	0.29903	-0.07839	0.11913	0.11898	0.11336	0.0553	-0.11612	0.14581
0.23649	0.36011	0.20606	0.23907	0.07371	-0.01084	0.30346	-0.23146	0.15649	0.12295	0.14281	0.04857	-0.09792	0.14923
0.25999	0.37499	0.24849	0.24037	0.16846	-0.03131	0.28441	-0.13768	0.15065	0.06481	0.15274	0.06226	-0.01386	0.15195
0.25607	0.38832	0.25655	0.20868	0.1285	-0.05824	0.29512	-0.04572	0.14591	0.16107	0.10237	0.05329	-0.11908	0.17721
0.22842	0.39	0.26163	0.24894	0.12955	-0.01949	0.31146	-0.03737	0.13783	0.10239	0.0859	0.0235	-0.16167	0.11953

ANGII_AT1	TENSIN_&	_ANTERG	SING_AND	SING_AN	AREG_EG	EG_EGF	FED_MICR	RNO_AR	EMBLY_AN	NCE_AT	REFERENC	SOME_AN	_AND_LY	LEATION_	_NUCLEA	NUCLEA	CLEATION	EATION_	ATION_EL	E_AVP_V2
0.10858	0.13045	0.15302	0.07847	0.33684	-0.2067	0.17266	0.07208	0.24401	0.33725	-0.16693	0.0115	-0.04382	0.20999	-0.08036	-0.11001	-0.22759	-0.1691	-0.12946	0.2125	0.28476
0.0871	0.12333	0.18882	0.03923	0.30369	-0.19746	0.10525	0.10043	0.23597	0.37962	-0.12858	0.08919	-0.03009	0.2048	-0.28593	-0.14855	-0.27474	-0.33452	-0.27864	0.19924	0.24362
0.14109	0.16068	0.15921	0.12819	0.32925	-0.15994	0.12529	0.08476	0.24636	0.35624	-0.09457	-0.05431	0.02445	0.24612	-0.20924	-0.14865	-0.29197	-0.27005	-0.23557	0.12541	0.23074
0.13699	0.13998	0.21382	0.20185	0.34684	-0.117	0.14159	0.15443	0.28165	0.32273	-0.1804	-0.00462	0.03453	0.2141	-0.08513	-0.05981	-0.22245	-0.15453	-0.1436	0.17483	0.28597
0.17938	0.11746	0.199	0.24455	0.3484	-0.14203	0.18371	0.13637	0.26924	0.33681	-0.12388	0.04401	0.07054	0.21718	-0.13891	-0.07581	-0.25652	-0.18293	-0.19544	0.2594	0.26593
-0.00753	0.12009	0.17881	0.12075	0.33239	-0.15186	0.06174	0.11428	0.25134	0.34045	-0.0867	0.03567	-0.05842	0.16358	-0.4334	-0.1683	-0.3609	-0.43471	-0.29445	0.15145	0.22411
0.18663	0.10142	0.14786	0.17411	0.33205	-0.13822	0.12493	0.06284	0.2598	0.35073	-0.13306	0.03403	-0.02722	0.20025	-0.18724	-0.12707	-0.2402	-0.25682	-0.18859	0.18711	0.26974
0.17515	0.15845	0.22809	0.1965	0.36377	-0.14135	0.13289	0.16607	0.28588	0.29555	-0.2747	0.03066	0.08254	0.24011	-0.11625	-0.02434	-0.25311	-0.15082	-0.12442	0.17149	0.29182
0.12994	0.14138	0.20394	0.32284	0.37676	-0.12217	0.11212	0.15018	0.27132	0.30771	-0.20013	-0.03251	0.08173	0.22564	-0.0964	-0.02254	-0.26169	-0.17364	-0.15453	0.16499	0.29356
0.17927	0.12707	0.21761	0.19282	0.3954	-0.15954	0.13	0.15364	0.27166	0.3158	-0.20895	0.04238	0.04089	0.24762	-0.09213	-0.0306	-0.28363	-0.16365	-0.17072	0.17331	0.28112
0.09862	0.09837	0.21597	0.12015	0.26206	-0.13777	0.1039	0.13978	0.26553	0.37752	-0.11889	-0.06613	-0.01242	0.25903	-0.05283	-0.07663	-0.21638	-0.12882	-0.21896	0.21751	0.26612
0.04937	0.13808	0.18373	0.17891	0.3775	-0.15095	0.11364	0.09607	0.25824	0.36686	-0.13188	-0.02928	-0.06506	0.21117	-0.18278	-0.10927	-0.29906	-0.24526	-0.26416	0.19814	0.28206
-0.03024	0.04262	0.27248	-0.01188	0.19723	-0.14556	0.1428	0.17181	0.30831	0.37488	-0.05493	0.05557	-0.07312	0.13253	-0.1141	-0.14534	-0.10574	-0.19416	-0.14171	0.10118	0.29837
0.13596	0.12466	0.21437	0.22425	0.2863	-0.13079	0.11959	0.14905	0.27685	0.36197	-0.30407	-0.05951	0.01638	0.24176	-0.11226	-0.12347	-0.23966	-0.18906	-0.18881	0.12605	0.27529
0.1386	0.10455	0.2085	0.23551	0.30617	-0.21438	0.14227	0.1447	0.23681	0.3427	-0.19209	-0.06238	-0.02625	0.19481	-0.15769	-0.08641	-0.25229	-0.22637	-0.22729	0.22195	0.28615
0.01069	0.12314	0.21523	0.11541	0.31819	-0.06566	0.1598	0.12434	0.28858	0.39031	-0.03933	-0.06727	-0.05853	0.14667	-0.09696	-0.14991	-0.20177	-0.17874	-0.19424	0.07389	0.18421
0.11211	0.11141	0.21152	0.15611	0.25336	-0.12492	0.14035	0.14251	0.28026	0.35025	-0.20712	-0.03528	0.00506	0.21483	-0.14702	-0.10868	-0.25163	-0.21513	-0.1626	0.16057	0.30642
0.06696	0.1288	0.24335	0.25824	0.35467	-0.13093	0.13844	0.17177	0.27856	0.33503	-0.18863	-0.05576	0.08979	0.25306	-0.20068	-0.0208	-0.30133	-0.26484	-0.19517	0.18963	0.27316

ND_STRA	D_STRAN	ON_AND_	R_BCAP	_PLCG_C	BCR_PL	EFERENC	BETA_OX	IENCE_BI	KING_UB	_GROUP	NCE_BM	NCE_BMP	E_BMP_	IENCE_BN	RANCHIN	CE_BREA	ORF72_M
0.1029	-0.0025	-0.05219	-0.12937	0.10527	0.07408	0.30945	0.14545	0.10861	-0.0588	-0.31777	-0.23055	-0.23055	-0.23055	-0.23055	0.16729	0.13718	0.06723
0.24286	0.18477	0.17785	-0.1231	0.04124	-0.00392	0.26817	0.05439	0.07596	0.02928	-0.07874	0.07865	0.07865	0.07865	0.07865	0.17538	-0.09152	0.02066
0.17051	0.06835	0.02817	0.01227	0.07282	0.01382	0.32019	0.12715	0.0303	-0.00188	-0.23023	-0.24189	-0.24189	-0.24189	-0.24189	0.17251	0.20536	-0.18753
0.12072	0.04272	0.02303	0.08928	0.13644	0.14499	0.28819	0.18313	0.08906	-0.07419	-0.21138	-0.18999	-0.18999	-0.18999	-0.18999	0.18228	0.13722	0.05649
0.13581	0.06109	-0.00049	0.02058	0.17773	0.11626	0.29433	0.13811	0.07556	-0.04713	-0.18954	-0.31318	-0.31318	-0.31318	-0.31318	0.1655	0.09735	-0.00986
0.15447	0.0923	0.09885	-0.12679	0.07994	-0.01959	0.28611	0.17964	-0.00282	-0.02088	-0.18601	-0.37068	-0.37068	-0.37068	-0.37068	0.13449	0.19385	-0.20686
0.16093	0.06246	0.04981	0.06328	0.07119	-0.01081	0.30409	0.15998	-0.03954	-0.01105	0.01568	-0.11992	-0.11992	-0.11992	-0.11992	0.19082	0.09938	-0.38699
0.09867	0.05383	-0.00935	0.11832	0.16961	0.11211	0.32002	0.18544	0.08611	-0.09086	-0.13088	-0.24957	-0.24957	-0.24957	-0.24957	0.23405	-0.05704	0.03464
0.11103	-0.00723	-0.01169	0.15327	0.17192	0.128	0.30701	0.15966	0.0571	-0.08308	-0.19007	-0.26574	-0.26574	-0.26574	-0.26574	0.23747	0.01117	-0.04109
0.11659	0.05585	-0.01427	0.08837	0.15918	0.14643	0.32598	0.15512	0.00344	-0.05189	-0.13387	-0.37498	-0.37498	-0.37498	-0.37498	0.22662	-0.06842	0.02455
0.15913	0.0279	0.03612	-0.07792	0.05437	-0.04283	0.30128	0.18064	0.02534	0.04577	0.00617	0.04537	0.04537	0.04537	0.04537	0.22425	0.2728	-0.20087
0.13487	0.09943	0.01953	-0.08151	0.06978	0.06872	0.33509	0.11873	-0.04713	-0.03488	0.0229	-0.19047	-0.19047	-0.19047	-0.19047	0.17096	0.19094	-0.00709
0.17572	0.03194	0.06499	-0.15186	-0.03433	-0.12882	0.26823	0.13851	0.0124	0.0577	-0.0532	0.04338	0.04338	0.04338	0.04338	0.2369	0.18579	-0.00839
0.15693	0.08498	0.06768	-0.03151	0.0841	0.01728	0.30393	0.16585	0.09936	-0.0089	-0.13282	-0.13975	-0.13975	-0.13975	-0.13975	0.23348	-0.12987	-0.18816
0.13751	0.06018	-0.03159	-0.10817	0.07283	0.02755	0.27607	0.10017	0.01822	-0.02433	-0.28096	-0.22574	-0.22574	-0.22574	-0.22574	0.21008	0.16006	0.00198
0.15331	0.11504	0.07239	-0.15266	0.00428	-0.17898	0.30353	0.08259	0.05482	0.01767	-0.40197	0.01933	0.01933	0.01933	0.01933	0.19012	0.24798	-0.11935
0.13366	0.06772	0.04545	0.04874	0.05632	0.05556	0.33942	0.12726	0.0495	-0.03996	-0.17897	-0.2098	-0.2098	-0.2098	-0.2098	0.25141	-0.22447	0.01179
0.10612	0.03428	0.00871	0.10145	0.1749	0.09387	0.31263	0.17402	0.08598	-0.09655	-0.22882	-0.22679	-0.22679	-0.22679	-0.22679	0.25506	0.02625	0.0135

CA2_CA	E_CA2_CA	A2_CAM	A2_ENTR	2_ENTRY	_CA2_PL	CA2_PYK2	_CARDIAC	CE_CASR	2_GNB_G	GNB_G	XCR_GN	E_CD40	80_CD86	80_CD86_	ENCE_CD	PE_INTE	E_CGAS	CE_CHO
0.14982	0.18094	0.41251	0.02026	0.44619	0.06618	0.20243	0.33538	0.00138	0.06227	0.12178	0.15196	-0.22906	-0.25263	0.04216	-0.08718	0.21669	0.04689	0.02178
0.14223	0.11006	0.42467	-0.06359	0.41466	0.00177	0.15563	0.27774	-0.08708	0.0744	0.09572	0.15357	-0.02906	-0.23631	-0.03032	-0.03787	0.2198	-0.0657	-0.07539
0.16557	0.11645	0.43947	-0.05848	0.44918	-0.09583	0.15617	0.31134	0.07166	0.05411	0.10905	0.12512	-0.08402	-0.15633	0.06807	0.00181	0.19717	-0.11331	0.03029
0.13921	0.15052	0.39912	0.19517	0.39598	-0.0074	0.16753	0.31528	0.07524	0.16148	0.22865	0.26311	-0.10582	-0.10564	0.0822	-0.10799	0.21729	0.00882	-0.00754
0.12119	0.21909	0.41885	0.08297	0.41941	0.00121	0.20254	0.27553	-0.02135	0.12558	0.16999	0.20513	-0.13018	-0.10505	0.02842	-0.03768	0.18138	-0.0341	0.00674
0.18678	0.16551	0.45304	-0.44812	0.3988	-0.06287	0.10592	0.15933	-0.12262	0.04786	0.07975	0.12061	-0.16372	-0.10874	0.04773	-0.0067	0.23299	-0.22976	0.00511
0.14619	0.1415	0.41833	0.00194	0.43157	-0.06643	0.16282	0.30749	-0.06124	0.09164	0.12741	0.15622	-0.00444	-0.11162	-0.0228	-0.03874	0.18633	-0.09402	0.01256
0.18263	0.1819	0.40529	0.2984	0.42258	-0.10503	0.16541	0.34709	0.05353	0.14852	0.20566	0.22508	-0.06954	-0.0912	0.04394	-0.22728	0.17548	0.01393	-0.08909
0.14897	0.17035	0.40863	0.2637	0.42293	-0.11104	0.15515	0.34175	0.01028	0.12855	0.19196	0.24203	-0.0112	-0.06534	0.05278	-0.13372	0.17597	-0.01151	-0.03788
0.1327	0.16708	0.38837	0.16998	0.39686	-0.05158	0.17019	0.29135	0.00761	0.154	0.21003	0.24128	0.00055	-0.10005	0.01689	-0.14642	0.19005	-0.0173	-0.08494
0.10074	0.13576	0.44317	-0.32006	0.46028	-0.07505	0.09174	0.37117	0.01804	0.01687	0.06385	0.11484	-0.16429	-0.21314	-0.04952	-0.00828	0.19201	-0.04165	0.06111
0.13544	0.13696	0.43048	-0.31425	0.44196	0.07867	0.13735	0.36349	0.02337	0.05762	0.10645	0.15586	-0.18925	-0.17996	-0.01792	-0.04586	0.18634	0.0078	0.01672
0.05381	0.08262	0.32247	-0.42135	0.4099	-0.02494	0.16422	0.24996	-0.03442	0.04999	0.1103	0.16815	0.01102	-0.16056	0.10733	0.01364	0.21374	-0.22283	0.11382
0.13167	0.14004	0.44388	0.20874	0.39405	-0.02775	0.1568	0.35519	-0.00292	0.09813	0.11056	0.15879	-0.12805	-0.13758	0.04258	-0.26484	0.24498	-0.0445	0.0176
0.16908	0.12937	0.41656	-0.08825	0.42452	0.00964	0.17521	0.33087	-0.07733	0.04877	0.07617	0.13743	-0.11235	-0.24088	0.0101	-0.12395	0.20384	-0.09752	0.01981
0.12884	0.12071	0.43366	-0.3914	0.44143	-0.09997	0.15406	0.32935	0.03739	-0.00669	0.04942	0.08268	-0.02802	-0.14376	0.0579	0.05977	0.20162	-0.17371	0.00567
0.09552	0.08656	0.35982	0.07821	0.3951	0.00741	0.1588	0.30634	0.04575	0.14889	0.21138	0.25554	-0.15789	-0.08723	0.06554	-0.14108	0.23677	-0.01467	-0.07724
0.14477	0.22339	0.43683	0.26176	0.41695	-0.01632	0.16528	0.32655	-0.01515	0.15378	0.20551	0.23039	-0.06908	-0.07598	0.02402	-0.11826	0.17084	0.00569	-0.05961

E_CHRN	TRATE_CY	ATE_CYC	COMPLE	COHESI	COHESI	NFERENCE	THWAY_O	RRENCE_	NCE_CO	ENCE_CO	RENCE_	ERENCE_	CRHR_P	WEEN_EX	CR1_GN	12_CXC	CR4_GNA
-0.11899	0.30143	0.34514	-0.05217	-0.18342	-0.00807	0.01453	-0.27517	-0.08179	0.1674	0.24641	-0.07941	0.06392	0.17269	-0.01455	0.29966	0.08662	0.0737
-0.10271	0.30798	0.27683	-0.16671	-0.09879	-0.0003	0.12976	-0.18261	-0.03848	0.16037	0.22845	0.03806	0.00416	0.15728	-0.00171	0.28557	-0.00042	-0.00692
-0.074	0.34214	0.32967	0.10771	-0.15763	0.04549	0.15298	-0.19571	0.00181	0.1753	0.256	0.005	0.24951	0.12375	0.05657	0.27273	0.07053	0.12929
0.03337	0.27559	0.2896	0.21386	-0.14729	0.00919	0.02881	0.01254	-0.04864	0.19173	0.24857	-0.03437	0.18475	0.15047	0.04662	0.33632	0.09973	0.12677
0.05429	0.27675	0.30193	0.11026	-0.21337	0.03694	0.12779	-0.14051	-0.04655	0.1427	0.22944	-0.02255	0.18039	0.17605	0.07004	0.32449	0.10521	0.13347
-0.10322	0.25863	0.28612	0.35461	-0.07861	0.01305	0.09969	0.22964	-0.01607	0.14595	0.21736	0.04224	0.14957	0.13334	-0.00525	0.28061	0.01377	0.0965
-9.5E-05	0.3181	0.30993	0.29755	-0.15032	-0.02276	0.0838	0.24458	-0.10329	0.155	0.25258	0.00048	0.00748	0.14092	0.05632	0.30015	0.05405	0.13702
0.08143	0.31377	0.27363	0.22918	-0.16647	-0.02793	0.00939	-0.02372	-0.06526	0.213	0.25946	-0.05995	-0.15747	0.16853	0.02939	0.34013	0.10252	0.14235
0.06338	0.27517	0.26969	0.22364	-0.19783	-0.02902	0.0036	0.07096	-0.04499	0.18086	0.25959	-0.05727	-0.08645	0.18029	0.08085	0.32848	0.09036	0.16142
0.02972	0.28732	0.3145	0.22636	-0.18307	-0.05574	0.03642	0.09035	-0.08286	0.1567	0.21603	-0.00642	-0.01182	0.1719	0.09734	0.3184	0.09895	0.13876
-0.08062	0.2906	0.33123	0.05715	-0.08117	-0.00514	0.10086	0.00316	-0.04583	0.16148	0.23583	-0.01156	0.2726	0.1612	0.05831	0.28828	0.0349	0.13583
-0.13943	0.32141	0.34034	0.19933	-0.18479	0.017	0.08938	0.18201	-0.07303	0.12307	0.22011	-0.02861	0.12565	0.19122	-0.00047	0.32174	0.06742	0.14908
-0.20371	0.27939	0.28105	-0.01575	-0.10294	0.05895	0.08525	0.00461	0.02922	0.10925	0.21829	0.00072	0.05689	0.17914	0.0325	0.275	0.0195	0.1309
-0.00079	0.32265	0.31586	0.13604	-0.1505	-0.00957	0.07354	-0.02487	-0.08297	0.20687	0.24505	-0.05854	0.02522	0.15516	0.01573	0.29658	0.03604	0.14416
-0.05891	0.23992	0.31294	0.06031	-0.17317	-0.01843	0.1079	-0.09122	-0.07776	0.17677	0.22658	-0.03911	0.06247	0.17781	0.03813	0.28747	0.07233	0.1672
-0.15185	0.33818	0.38507	-0.26678	-0.14923	0.04018	0.11488	-0.3105	-0.00208	0.13734	0.20217	0.0023	0.16085	0.1016	0.05531	0.22485	0.05014	0.10527
-0.03148	0.30491	0.34849	0.03025	-0.10807	-0.01445	0.07762	-0.17125	-0.08256	0.16091	0.25579	-0.06772	0.24931	0.1854	0.01138	0.32352	0.06591	0.12943
0.03671	0.30641	0.32178	0.15415	-0.175	-0.04681	0.0176	-0.11732	-0.07416	0.14122	0.22931	-0.09906	-0.03709	0.1591	0.04036	0.31143	0.07673	0.12613

CR4_GNA	AQ_PLC	R4_GNB	XCR4_G	XCR_GN	R_GNB	YTOKINE	EPHOSP	E_DERMA	CE_DEU	BRENCE_	ATION_BY	NA_END	CE_DNA	CE_DNA	_DOUBL	RD1_GN	EIN_REC	E2_ER_R
-0.02502	0.09227	0.1133	0.26297	0.2271	0.10733	0.10941	0.21669	-0.04369	-0.10893	-0.18852	0.01693	-0.00794	0.14002	0.21762	0.03856	0.17269	0.06467	0.17656
-0.01589	0.0336	0.06783	0.21638	0.19868	0.12259	0.02773	0.2198	-0.10569	0.06842	-0.08578	-0.03504	0.04999	-0.05646	0.10537	0.08676	0.15728	0.09131	0.12063
0.03063	0.0625	0.07744	0.2383	0.18933	0.09406	0.08191	0.19717	-0.13458	-0.10543	-0.16308	0.04567	-0.01412	0.20855	0.24174	0.05768	0.12375	0.05234	0.13724
0.06906	0.09769	0.1461	0.29874	0.27229	0.22804	0.13174	0.21729	-0.18443	-0.16685	-0.15253	0.05915	0.00867	0.14162	0.2097	0.05573	0.15047	0.10627	0.14986
0.06087	0.13096	0.1051	0.2649	0.25445	0.15079	0.16093	0.18138	0.07169	-0.07502	-0.20423	-0.01328	0.00311	0.10238	0.18351	0.03436	0.17605	0.097	0.19953
0.04044	0.07354	0.06453	0.22229	0.13906	0.07788	0.12246	0.23299	-0.17703	-0.11483	-0.04774	0.03037	-0.03158	0.19365	0.24828	0.05101	0.13334	0.06661	0.0752
0.05972	0.04993	0.10035	0.26014	0.20524	0.11805	0.11733	0.18633	-0.13	-0.05466	-0.15188	0.08936	0.03087	0.12086	0.19225	0.06053	0.14092	0.02882	0.13576
0.06396	0.11638	0.14536	0.29472	0.25096	0.17482	0.17543	0.17548	-0.0916	-0.18693	-0.17027	0.05995	0.02117	-0.04576	0.10469	0.02883	0.16853	0.11322	0.15051
0.07545	0.10679	0.12795	0.27337	0.22285	0.16494	0.19314	0.17597	0.05284	-0.14914	-0.19221	0.09652	-0.01335	0.01405	0.11146	0.0313	0.18029	0.11511	0.13919
0.05517	0.09902	0.15254	0.30471	0.25336	0.17723	0.2075	0.19005	0.03928	-0.12907	-0.17807	0.07785	0.00936	-0.07629	0.07976	0.05577	0.1719	0.08173	0.14948
0.00178	0.07119	0.05609	0.22567	0.15346	0.07393	0.02459	0.19201	-0.13845	-0.12598	-0.09148	-0.01794	-0.00474	0.3151	0.28765	0.07692	0.1612	0.12491	0.09767
0.01765	0.07145	0.08293	0.23787	0.1931	0.11101	0.11584	0.18634	0.10939	-0.12363	-0.16456	0.00927	0.01284	0.19208	0.23153	0.07316	0.19122	0.08346	0.12066
0.02997	0.00166	0.10646	0.24293	0.23442	0.15464	-0.00608	0.21374	-0.45623	-0.08876	-0.11641	-0.022	0.05171	0.21944	0.24531	0.12936	0.17914	0.11792	0.15048
0.03861	0.06836	0.08766	0.22678	0.20105	0.12511	0.0864	0.24498	0.04703	-0.14136	-0.16902	-0.01902	-0.03026	-0.10411	0.10022	0.0496	0.15516	0.10083	0.12982
-0.03763	0.04392	0.06271	0.24058	0.19965	0.08315	0.06952	0.20384	-0.14356	-0.12435	-0.15812	0.00048	0.00186	0.18096	0.20165	0.07623	0.17781	0.10967	0.15866
0.04684	0.04567	0.05369	0.15045	0.17169	0.07079	-0.00261	0.20162	-0.13916	-0.0796	-0.10634	0.0045	0.07039	0.2482	0.27527	0.16809	0.1016	0.07125	0.14414
0.07343	0.04481	0.1477	0.30107	0.27792	0.22463	0.07219	0.23677	0.02889	-0.13287	-0.12302	0.07006	0.01098	-0.2168	0.04832	0.07029	0.1854	0.09752	0.15773
0.0636	0.1285	0.1427	0.28487	0.25818	0.19098	0.16425	0.17084	-0.08578	-0.14197	-0.18357	0.06738	0.02329	0.02958	0.14077	0.10028	0.1591	0.10761	0.15327

NCE_EA	EGF_EGF	F_EGFR	EGF_EG	FR_PLC	_EGFR_	F_EGFR	GF_EGFR	GF_EGFR	F_EGFR	_EGFR_	F_EGFR	F_ERBB2	EGF_JA	ELECTR	ELECTR	ELECTR	P_NE_AD
0.08518	0.28368	-0.10506	-0.2452	0.09354	0.11393	0.08687	0.17266	0.06445	-0.06867	0.1176	0.02478	0.16335	0.09565	0.08376	0.35563	0.28416	0.28476
-0.02228	0.2423	-0.10002	-0.26268	0.04227	0.13302	0.00106	0.10525	0.07289	-0.06108	0.09585	0.02053	0.08739	0.01531	0.13346	0.31205	0.26115	0.24362
-0.05652	0.26864	-0.08946	-0.18298	0.02944	0.12686	0.03345	0.12529	0.03774	-0.04277	0.06164	0.0023	0.10881	0.01692	0.08442	0.32192	0.24648	0.23074
0.069	0.26741	-0.04476	-0.1408	0.0758	0.12363	0.07381	0.14159	0.06171	-0.01403	0.09145	0.04202	0.10992	0.07521	0.14499	0.28285	0.28683	0.28597
0.02569	0.28063	-0.02988	-0.15242	0.14392	0.12035	0.11447	0.18371	0.1127	0.0102	0.1133	0.10645	0.15797	0.11498	0.08835	0.17556	0.26789	0.26593
-0.01643	0.2662	-0.08471	-0.18096	0.09858	0.15752	0.03131	0.06174	0.00252	-0.07093	0.06683	-0.00363	0.04294	0.09946	0.12287	0.26116	0.27016	0.22411
-0.13234	0.26656	-0.04832	-0.16087	0.05204	0.09982	0.05759	0.12493	0.01362	-0.04581	0.0839	-0.01605	0.09941	0.06491	0.12325	0.33769	0.2748	0.26974
0.08097	0.2673	-0.01898	-0.15589	0.10405	0.14946	0.08384	0.13289	0.03097	-0.03752	0.04598	0.0228	0.10343	0.11174	0.13593	0.28803	0.27229	0.29182
0.05195	0.26001	-0.04104	-0.13277	0.1027	0.14533	0.08136	0.11212	0.0067	-0.03679	0.03788	0.01453	0.08032	0.11391	0.14567	0.26135	0.26588	0.29356
0.04417	0.25443	-0.02313	-0.15552	0.09716	0.12553	0.09667	0.13	0.02532	-0.04024	0.0528	0.03319	0.09647	0.15163	0.1656	0.27464	0.29728	0.28112
0.02992	0.27366	-0.09681	-0.13204	0.09143	0.10661	0.05179	0.1039	0.0511	-0.05957	0.08775	-0.00059	0.09082	0.06009	0.16874	0.32734	0.25831	0.26612
-0.04386	0.29147	-0.105	-0.16372	0.07	0.12019	0.06481	0.11364	0.00794	-0.07627	0.02899	-0.0138	0.10709	0.10603	0.10612	0.32912	0.29146	0.28206
0.03523	0.27822	-0.17908	-0.17457	-0.01064	-0.01449	0.00927	0.1428	0.07974	-0.01637	0.1491	0.02315	0.14834	-0.0152	-0.08265	0.31832	0.22681	0.29837
0.03438	0.27794	-0.03072	-0.18209	0.05865	0.11489	0.01519	0.11959	0.07393	-0.01689	0.10202	0.05061	0.09181	0.02402	0.09254	0.25697	0.25314	0.27529
0.04693	0.26859	-0.11475	-0.27519	0.0512	0.10468	0.08897	0.14227	-0.00127	-0.11163	0.02233	-0.03019	0.10965	0.02817	0.09014	0.32014	0.24102	0.28615
-0.01636	0.27542	-0.09538	-0.0656	0.05233	0.10373	0.05916	0.1598	0.09478	0.00901	0.12102	0.04096	0.1567	0.07572	0.07832	0.32039	0.21844	0.18421
0.02828	0.28641	-0.07608	-0.15015	0.01571	0.07584	0.03776	0.14035	0.09682	0.00833	0.1189	0.07635	0.12582	0.02079	0.12043	0.36875	0.32111	0.30642
0.01448	0.28986	-0.02026	-0.13056	0.13698	0.13158	0.07514	0.13844	0.05388	-0.01338	0.05876	0.0472	0.11188	0.10473	0.16316	0.31177	0.31276	0.27316

P_NE_AD	REG_EG	EG_EGF	ERENCE_	CE_ESTA	CE_EXTR	NCE_FAS	FERENCE	FGF_FG	GF_FGFR	_FLCN_M	T3LG_FL	T3LG_FLT	FSHR_G	GATOR1	METHYL	E_GF_RT	GF_RTK	GF_RTK
0.01742	-0.2067	0.17266	0.20751	-0.17191	-0.10427	-0.30539	0.16927	-0.20796	0.2079	-0.15977	0.2079	-0.01998	0.28476	-0.2196	0.11087	-0.18666	0.06301	-0.09511
0.03116	-0.19746	0.10525	0.14035	-0.03558	-0.11333	-0.24539	0.15909	-0.16983	0.13761	-0.12707	0.13761	0.00579	0.24362	-0.19229	0.18683	-0.19575	0.02194	-0.09223
0.07935	-0.15994	0.12529	0.22561	-0.01873	-0.03208	-0.17412	0.20246	-0.12675	0.16361	-0.07863	0.16361	0.00753	0.23074	-0.20986	0.13394	-0.19938	0.00195	-0.10209
0.12633	-0.117	0.14159	0.19568	-0.12504	-0.04078	-0.25296	0.25448	-0.08827	0.17844	-0.07183	0.17844	0.0488	0.28597	-0.16694	0.10419	-0.13803	0.01842	-0.05754
0.11293	-0.14203	0.18371	0.20499	-0.00924	0.0037	-0.23923	0.15144	-0.10862	0.21905	-0.12583	0.21905	0.06359	0.26593	-0.21444	0.12103	-0.16843	0.05618	-0.05824
0.10287	-0.15186	0.06174	0.08287	-0.04856	-0.04231	-0.14046	0.26852	-0.13276	0.09182	-0.11662	0.09182	-0.01921	0.22411	-0.20006	0.14759	-0.17079	-0.02758	-0.10572
0.11857	-0.13822	0.12493	0.19738	-0.0205	-0.0247	-0.27271	0.20663	-0.10162	0.16421	-0.0257	0.16421	0.01451	0.26974	-0.16676	0.13823	-0.10079	0.02671	-0.04893
0.12054	-0.14135	0.13289	0.22395	-0.11333	-0.05197	-0.34169	0.2238	-0.11402	0.17088	-0.07473	0.17088	0.01403	0.29182	-0.18497	0.09159	-0.16712	-0.00364	-0.09051
0.13334	-0.12217	0.11212	0.19818	-0.15153	0.00494	-0.28582	0.21952	-0.08826	0.1498	-0.05712	0.1498	0.0181	0.29356	-0.18443	0.09861	-0.15007	-0.021	-0.08708
0.11319	-0.15954	0.13	0.22467	-0.09775	0.00687	-0.35415	0.19459	-0.12714	0.16445	-0.10663	0.16445	0.00345	0.28112	-0.23814	0.10524	-0.17023	0.00949	-0.09366
0.03952	-0.13777	0.1039	0.13657	-0.04574	-0.11855	8.9E-05	0.20455	-0.20634	0.10074	-0.09913	0.10074	-0.06727	0.26612	-0.17139	0.14332	-0.09762	0.01937	-0.06212
0.07183	-0.15095	0.11364	0.19739	-0.05687	-0.09334	-0.0924	0.1992	-0.16313	0.13592	-0.11191	0.13592	-0.05868	0.28206	-0.23965	0.11409	-0.20666	-0.00711	-0.13717
0.05848	-0.14556	0.1428	0.26318	-0.07034	-0.03517	-0.23325	0.16857	-0.12173	0.17889	-0.02554	0.17889	0.04282	0.29837	-0.05389	0.15193	-0.06374	0.10512	-0.00012
0.09399	-0.13079	0.11959	0.1774	-0.05359	-0.07105	-0.17774	0.16593	-0.09184	0.15745	-0.09227	0.15745	0.05814	0.27529	-0.17435	0.1208	-0.20642	0.00891	-0.0891
0.01225	-0.21438	0.14227	0.24796	-0.11426	-0.05717	-0.2429	0.22958	-0.20018	0.18016	-0.12131	0.18016	-0.06067	0.28615	-0.20981	0.10838	-0.28413	0.00591	-0.17683
0.06436	-0.06566	0.1598	0.1376	-0.04543	-0.09893	-0.08766	0.20306	-0.1016	0.16752	-0.08258	0.16752	0.01291	0.18421	-0.15503	0.14286	-0.10263	0.07994	-0.04359
0.13216	-0.12492	0.14035	0.16622	-0.09959	-0.08091	-0.34918	0.19891	-0.08477	0.17754	-0.14012	0.17754	0.07266	0.30642	-0.19639	0.13349	-0.06661	0.05557	0.00491
0.1222	-0.13093	0.13844	0.20433	-0.1095	-0.0463	-0.24503	0.19121	-0.10597	0.17473	-0.09017	0.17473	0.03306	0.27316	-0.20937	0.09705	-0.08009	0.04165	-0.0208

GHRHR_	E_GH_JA	RENCE_G	ERENCE	NCE_GLU	ENCE_GL	ENCE_GL	_GLYCO	REFEREN	GLYCAN_	H_GNRH	CE_GPCR	GPCR_PL	NCE_GPI	ERENCE_	E_HEPAR	HGF_M	GF_MET
0.17269	-0.08261	0.06012	0.29002	0.13602	0.18897	0.25123	0.09864	0.38689	-0.02267	0.17274	0.11851	0.16714	-0.16471	0.03261	0.17434	-0.2571	0.2079
0.15728	-0.25223	0.08874	0.22921	0.11198	0.07849	0.14935	0.08891	0.3885	-0.12321	0.08437	0.13108	0.08313	-0.09908	0.06716	0.07648	-0.23755	0.13761
0.12375	-0.22778	0.03624	0.22942	0.13015	0.12099	0.24345	0.11601	0.3626	0.04967	0.17481	0.11189	0.1207	-0.12383	0.08427	0.13913	-0.1453	0.16361
0.15047	-0.08345	0.02183	0.26268	0.11845	0.15058	0.20847	0.08617	0.36406	-0.09653	0.18898	0.24855	0.18587	-0.08995	0.03111	0.12615	-0.11043	0.17844
0.17605	-0.01435	0.05675	0.21389	0.18543	0.17505	0.21606	0.07466	0.38425	0.15437	0.17684	0.17039	0.11723	-0.13689	0.086	0.22061	-0.11258	0.21905
0.13334	-0.0218	0.11274	0.21563	0.20597	0.15552	0.21297	-0.11253	0.38125	0.10236	0.07702	0.09529	0.05799	-0.0667	0.12776	0.15052	-0.16363	0.09182
0.14092	-0.10357	0.0793	0.26949	0.1372	0.09726	0.17593	0.13078	0.35158	-0.02228	0.15187	0.13801	0.08132	-0.22036	-0.02136	0.17675	-0.11889	0.16421
0.16853	-0.03249	-0.00211	0.24114	0.18456	0.20151	0.24309	0.08811	0.33469	0.12438	0.19437	0.20158	0.11332	-0.11018	0.08457	0.17029	-0.12447	0.17088
0.18029	-0.01395	-0.03617	0.23415	0.17703	0.13577	0.22207	0.09604	0.35312	0.06204	0.1617	0.19029	0.08456	-0.07879	0.04436	0.22266	-0.09039	0.1498
0.1719	0.05731	0.04106	0.20864	0.15049	0.1311	0.21738	0.08983	0.35742	-0.09321	0.1852	0.2095	0.12629	-0.07952	0.04726	0.2121	-0.11589	0.16445
0.1612	-0.28703	0.04369	0.27996	0.21031	0.20949	0.26069	-0.04641	0.38049	-0.00466	0.11896	0.08642	0.10654	-0.03235	0.00475	0.04034	-0.21292	0.10074
0.19122	-0.12303	0.05448	0.25745	0.17977	0.17561	0.19605	0.09648	0.35958	0.03812	0.16751	0.13861	0.19717	-0.157	0.0847	0.21761	-0.18296	0.13592
0.17914	-0.34716	0.09836	0.22151	0.11188	0.28367	0.1319	-0.38102	0.38486	0.04147	0.13458	0.15784	0.11432	-0.13122	0.00324	0.0175	-0.15146	0.17889
0.15516	-0.17189	0.03971	0.25762	0.13948	0.23142	0.22421	0.03578	0.36044	0.06622	0.13496	0.13152	0.10207	-0.16637	0.06417	0.24957	-0.14037	0.15745
0.17781	-0.1823	0.02201	0.23697	0.16567	0.23594	0.27004	0.18702	0.37631	0.06255	0.1541	0.10052	0.08386	-0.17418	0.00849	0.12724	-0.27121	0.18016
0.1016	-0.28492	0.06625	0.26148	0.10772	0.18179	0.19972	0.01739	0.39075	-0.03548	0.16177	0.07052	0.08769	-0.14232	-0.00772	0.04421	-0.11092	0.16752
0.1854	-0.16564	0.01865	0.21142	0.14313	0.16199	0.19954	0.11386	0.37799	-0.12813	0.16042	0.24759	0.14819	-0.16326	0.06396	0.22959	-0.10368	0.17754
0.1591	-0.04754	0.05754	0.22885	0.13848	0.1727	0.21447	0.14979	0.35938	-0.07103	0.1536	0.2206	0.09188	-0.12357	0.02858	0.18704	-0.09944	0.17473

E_HYDR(	COSLG_I(	E_IFN_R	IGF2_IGF	IGF_IGFB	F_IGF1R	F_IGFR_I	IGG_FC(	BLOOD_(	FAMILY_	12_23_TO	E_IL1_IL1	E_IL1_IL1	FAMILY_T	FAMILY_T
0.13301	-0.20966	0.06094	-0.17472	-0.08223	0.18503	-0.09202	0.19201	-0.23036	0.06814	0.06814	-0.16488	-0.08885	0.1032	0.10369
0.12562	-0.23603	-0.02282	-0.08851	-0.0836	0.14828	-0.07323	0.1882	-0.20171	0.02105	0.02105	-0.27616	-0.18716	0.03902	0.08581
0.13064	-0.0603	0.01244	-0.13877	-0.03285	0.13191	-0.05923	0.18751	-0.3056	-0.00619	-0.00619	-0.21147	-0.13766	0.05421	0.10892
0.13854	0.03054	0.05709	-0.08956	0.01708	0.15308	-0.01723	0.31282	-0.18572	0.00431	0.00431	-0.21504	-0.14034	0.11388	0.12058
0.07221	-0.05373	0.06553	-0.07691	-0.04456	0.20379	0.00102	0.19266	-0.23835	0.06564	0.06564	-0.22907	-0.10378	0.15675	0.15858
0.09695	-0.10167	0.02786	-0.09289	-0.01025	0.09048	-0.06334	0.12336	-0.23004	0.04025	0.04025	-0.25601	-0.16506	0.11975	0.11109
0.09907	0.03071	0.03993	-0.03987	0.02169	0.16948	-0.01494	0.19953	-0.13364	0.0093	0.0093	-0.19512	-0.11178	0.09584	0.11367
0.13184	0.01823	0.1114	-0.11956	-0.02728	0.14149	0.0122	0.32743	-0.1662	0.08259	0.08259	-0.21112	-0.11779	0.15828	0.17003
0.14752	0.05776	0.10556	-0.1153	-0.00668	0.11455	-0.00943	0.27292	-0.21549	0.036	0.036	-0.11847	-0.01674	0.17371	0.1681
0.13377	-0.04132	0.1104	-0.14877	-0.02681	0.12843	0.00861	0.30302	-0.1952	0.04949	0.04949	-0.21072	-0.10059	0.18456	0.16104
0.05746	-0.08229	-0.04116	-0.23427	-0.06626	0.06617	-0.135	0.11949	-0.14366	0.05203	0.05203	-0.1758	-0.12276	0.02886	0.1019
0.08738	-0.12903	0.03344	-0.19536	-0.04441	0.09914	-0.10669	0.05751	-0.04157	0.06832	0.06832	-0.19081	-0.11626	0.1084	0.14705
0.08878	-0.16525	-0.05909	-0.15536	-0.00482	0.14688	-0.16575	0.00719	-0.00071	0.0182	0.0182	-0.16689	-0.0975	-0.00056	0.06316
0.15075	-0.10092	0.00703	-0.06789	-0.0039	0.14579	0.00472	0.2488	-0.09343	0.06392	0.06392	-0.23137	-0.14202	0.08405	0.14202
0.06951	-0.22086	0.00356	-0.1923	-0.09678	0.14995	-0.0929	0.20299	-0.27175	-0.01326	-0.01326	-0.19505	-0.1177	0.04892	0.0792
0.10023	-0.19219	-0.07669	-0.07094	0.01463	0.15725	-0.12668	-0.00707	-0.28247	0.01986	0.01986	-0.17939	-0.12513	0.01288	0.08116
0.1997	0.04481	-0.00668	-0.02874	-0.03384	0.18103	-0.04414	0.24457	-0.11758	0.00767	0.00767	-0.25904	-0.17357	0.06891	0.09522
0.17051	0.06439	0.08472	-0.11499	-0.02528	0.14483	0.00916	0.28314	-0.33219	0.06259	0.06259	-0.24006	-0.13691	0.14725	0.16269

E_INAC	E_INTRI	E_IRE1A	ENCE_IS	TGA_B_FA	ITGA_B_I	A_B_RH	GA_B_RH	TGA_B_RI	A_B_TAL	E_KEAP1	E_KERAT	KINETO	NETOCH	G1_KISS1
-0.18084	0.00233	-0.34926	0.28222	0.0701	0.11089	0.08045	0.11347	0.11538	0.20662	0.13112	0.12009	0.26257	0.22757	0.03836
-0.08438	0.03918	-0.30872	0.25591	0.02654	0.06373	0.06301	0.06403	0.09096	0.18326	0.01061	0.10341	0.26996	0.268	-0.02523
-0.05972	0.06801	-0.34334	0.30362	0.06966	0.11289	0.0956	0.11909	0.13318	0.20373	0.06709	0.15839	0.2647	0.2695	0.08608
-0.12897	0.02909	-0.40978	0.27598	0.07522	0.11697	0.09915	0.08961	0.15494	0.19438	0.09413	0.23332	0.2982	0.29849	0.12796
-0.11252	0.05776	-0.33461	0.29009	0.01175	0.05811	0.01252	0.01654	0.09508	0.14593	0.09622	0.17426	0.26199	0.26941	0.07291
-0.13093	-0.03332	-0.31196	0.28104	-0.00675	0.05632	0.00698	0.02569	0.07151	0.15968	0.03251	0.1803	0.24196	0.23643	0.0036
-0.10194	0.07632	-0.25546	0.2932	0.07049	0.11899	0.07621	0.10225	0.12778	0.1773	0.05136	0.17591	0.26504	0.26158	0.04053
-0.11267	0.02706	-0.40433	0.3001	0.06589	0.10874	0.06549	0.06445	0.13836	0.17677	0.09899	0.2311	0.29287	0.30545	0.1146
-0.09355	0.07507	-0.39058	0.2906	0.04653	0.09622	0.06025	0.05447	0.13375	0.16916	0.10509	0.25392	0.28535	0.30454	0.08403
-0.10902	0.07862	-0.33618	0.30413	0.02732	0.07256	0.0155	0.01817	0.09842	0.14908	0.12467	0.21373	0.27747	0.29447	0.10308
-0.06608	0.09985	-0.40574	0.28003	0.02128	0.09696	0.05585	0.08137	0.08129	0.18985	0.09785	0.01443	0.32146	0.32622	0.03803
-0.17846	0.02404	-0.28601	0.32478	0.05796	0.11213	0.09189	0.1271	0.12852	0.20978	0.12273	0.15703	0.2608	0.27383	0.08339
-0.03999	0.06262	-0.25786	0.26318	0.10895	0.13252	0.1368	0.18347	0.12169	0.22684	0.00173	-0.08208	0.34147	0.35066	0.01866
-0.11467	0.02197	-0.45557	0.30106	0.02908	0.09492	0.04723	0.06334	0.10768	0.16445	0.09724	0.25785	0.31219	0.30748	0.04208
-0.14816	0.02592	-0.35129	0.27506	0.0021	0.05387	0.00806	0.02762	0.05838	0.16057	0.12844	0.17293	0.26672	0.27828	0.00135
-0.06965	0.08543	-0.21343	0.28765	0.01245	0.05308	0.04782	0.08434	0.02217	0.18644	0.11305	0.04135	0.29362	0.30178	0.04514
-0.14325	0.00489	-0.32374	0.32682	0.0788	0.12173	0.0859	0.09791	0.13672	0.19546	0.11515	0.22807	0.30614	0.32077	0.09198
-0.08872	0.04416	-0.41076	0.29487	0.0769	0.1194	0.08178	0.09163	0.14347	0.1817	0.07359	0.20391	0.32117	0.33775	0.03932

F_KITLG	KITLG_KIT	Y_OF_CO	F_COAG	OMPLE	ION_BYP	RENCE_L	HCGR_G	IGHT_HV	LOADING	FERENCE	PAR_GN	PA_GNAI	2PA_GNA	ENCE_LY
-0.20796	0.2079	0.38026	0.21138	0.04338	-0.40985	0.12674	0.28476	-0.04656	0.17223	-0.0853	0.21283	0.0737	0.12715	0.22969
-0.16983	0.13761	0.36028	0.17228	-0.31909	-0.36357	0.08912	0.24362	-0.03866	0.15861	0.03495	0.21865	-0.00692	0.13682	0.15784
-0.12675	0.16361	0.35157	0.0844	-0.05297	-0.40937	0.09604	0.23074	-0.07857	0.17338	0.05288	0.1859	0.12929	0.16038	0.15845
-0.08827	0.17844	0.40122	0.28718	0.14307	-0.40462	0.02791	0.28597	-0.0466	0.17836	-0.00956	0.32518	0.12677	0.20957	0.19793
-0.10862	0.21905	0.42249	0.31543	0.16104	-0.39228	0.06226	0.26593	-0.00604	0.17364	-0.04731	0.25859	0.13347	0.14541	0.24555
-0.13276	0.09182	0.37286	0.18487	0.13741	-0.26898	0.02313	0.22411	-0.086	0.1433	0.03355	0.17619	0.0965	0.09131	0.19502
-0.10162	0.16421	0.36606	0.15866	-0.18144	-0.34103	0.13091	0.26974	-0.03443	0.17059	-0.00256	0.2182	0.13702	0.11617	0.17847
-0.11402	0.17088	0.41283	0.29966	0.15642	-0.37626	0.05769	0.29182	0.00299	0.18248	-0.10023	0.28683	0.14235	0.17137	0.21717
-0.08826	0.1498	0.41555	0.31196	0.10297	-0.37678	-0.00027	0.29356	-0.05598	0.17962	-0.07346	0.27213	0.16142	0.13427	0.20399
-0.12714	0.16445	0.41839	0.31594	0.12641	-0.34772	0.04913	0.28112	-0.00692	0.17026	-0.0262	0.29836	0.13876	0.1662	0.18532
-0.20634	0.10074	0.35327	0.10435	0.01295	-0.30527	0.15113	0.26612	-0.12877	0.13639	0.03506	0.17364	0.13583	0.18815	0.18595
-0.16313	0.13592	0.36498	0.17766	0.15021	-0.40985	0.11456	0.28206	-0.12736	0.16818	0.03066	0.2216	0.14908	0.23786	0.17471
-0.12173	0.17889	0.35683	0.16375	-0.09512	-0.40092	0.08799	0.29837	-0.16817	0.19053	0.02816	0.23705	0.1309	0.2269	0.12239
-0.09184	0.15745	0.38067	0.20964	0.07597	-0.37872	0.15262	0.27529	0.00013	0.17607	-0.06911	0.21072	0.14416	0.15588	0.17499
-0.20018	0.18016	0.38759	0.2537	-0.00883	-0.38207	0.05123	0.28615	-0.05964	0.21165	0.01968	0.1978	0.1672	0.16686	0.18108
-0.1016	0.16752	0.34998	0.10921	-0.40715	-0.35917	0.1445	0.18421	-0.1279	0.17451	0.05927	0.14542	0.10527	0.16591	0.16803
-0.08477	0.17754	0.39543	0.25117	0.10593	-0.4062	0.11476	0.30642	-0.08618	0.18497	-0.12278	0.32144	0.12943	0.20401	0.12611
-0.10597	0.17473	0.38497	0.22352	0.08582	-0.37432	0.05139	0.27316	-0.02003	0.17129	-0.09584	0.29873	0.12613	0.10369	0.25745

E_MACH	CE_MAGI	E_MDA5	MDM2_P	MDM2_P	INITIATE	ENCE_ME	RENCE_	MGLUR1	E_MGLUR	E_MICRO	BULE_DE	NCE_MIC	ICROTUB	FERENCE
0.25901	-0.21283	-0.01356	-0.19799	-0.21845	-0.25339	0.303	0.04232	0.08272	0.19225	0.18389	0.21661	0.18241	0.20935	-0.04066
0.20588	-0.17254	-0.03316	-0.17112	-0.13553	-0.21783	0.29382	-0.00636	-0.02033	0.15334	0.21895	0.25933	0.18562	0.22578	0.05376
0.1945	-0.05919	-0.07464	-0.12127	-0.14993	-0.10927	0.28264	-0.03506	0.03279	0.15299	0.22118	0.25392	0.18693	0.25642	0.06951
0.20684	-0.02949	-0.09174	-0.35585	-0.32141	-0.05665	0.26733	-0.0372	0.10292	0.16605	0.247	0.2821	0.19729	0.26874	0.00771
0.19292	-0.108	-0.06482	-0.26052	-0.25756	-0.06944	0.27666	0.00949	0.04814	0.15016	0.22098	0.25513	0.18222	0.25233	-0.01967
0.19002	-0.1177	-0.09517	-0.30685	-0.26225	-0.09971	0.27684	-0.01672	-0.01308	0.13884	0.19721	0.22975	0.14645	0.21836	0.04946
0.19928	-0.06806	-0.07952	-0.35745	-0.26244	-0.04991	0.31053	-0.02629	0.00456	0.16665	0.2025	0.25429	0.19881	0.24548	0.00372
0.16111	-0.08212	-0.05418	-0.37939	-0.33092	-0.05902	0.24549	-0.01031	0.03799	0.12149	0.25184	0.28374	0.24267	0.28155	-0.06756
0.13755	-0.0583	-0.06824	-0.32003	-0.31218	-0.01909	0.27164	0.0085	0.00955	0.12342	0.25306	0.28677	0.2441	0.2858	-0.05703
0.16103	-0.09866	-0.04188	-0.36283	-0.32974	-0.0564	0.25901	-0.0082	0.06732	0.14826	0.24251	0.27927	0.23681	0.27032	0.0032
0.221	-0.16192	-0.03525	-0.14873	-0.04512	-0.21518	0.32681	-0.02464	0.00382	0.17622	0.26478	0.3067	0.24211	0.30753	0.03586
0.24303	-0.10148	-0.0409	-0.12986	-0.17597	-0.13909	0.28588	0.11073	0.10037	0.16833	0.22321	0.25252	0.18244	0.25497	0.04627
0.22217	-0.07979	-0.02995	-0.10019	-0.04589	-0.16432	0.29926	0.08278	-0.00234	0.16621	0.29159	0.33056	0.25128	0.31168	0.07496
0.19393	-0.07872	-0.08428	-0.36085	-0.29513	-0.07839	0.25	-0.04507	0.00689	0.14037	0.26342	0.29222	0.23882	0.28237	-0.05076
0.17737	-0.21394	-0.01616	-0.29428	-0.22139	-0.23146	0.29859	0.08914	-0.01543	0.14257	0.23021	0.25835	0.22033	0.27495	0.00518
0.21858	-0.04645	0.0004	-0.28884	-0.26463	-0.13768	0.29019	0.03102	-0.01997	0.16541	0.24322	0.27933	0.20913	0.27283	0.05431
0.24665	-0.02179	-0.08235	-0.12766	-0.17719	-0.04572	0.24861	-0.04317	0.06117	0.1825	0.27072	0.30915	0.25235	0.2856	-0.09003
0.1763	-0.02892	-0.03639	-0.3946	-0.35427	-0.03737	0.25293	-0.02672	0.02656	0.13511	0.27921	0.32335	0.26936	0.30471	-0.09065

ONDRIAL	ICE_MLK	ODIFYIN	CE_MYOS	TERENCE	RP3_INF	NCE_NO	E_NOD_	NONICAL	E_NON_	HENCE_N	E_NOTCH	NCE_NOT	NRG_	ERENCE_NU
0.18975	-0.28342	-0.09852	-0.23055	-0.07684	0.19472	-0.17583	0.00839	0.12754	0.36687	-0.26017	0.07328	-0.28615	-0.28923	0.07775
0.18872	-0.27074	-0.03549	0.07865	-0.06767	0.1025	0.02903	0.01871	0.03195	0.38758	-0.14391	0.01295	-0.2321	-0.25919	0.09682
0.18147	-0.27867	0.00507	-0.24189	-0.13404	0.23846	-0.09299	-0.02345	0.1567	0.40752	-0.14021	-0.0041	-0.23636	-0.16278	0.11513
0.20599	-0.38524	-0.14237	-0.18999	-0.11964	0.26023	-0.13803	0.01131	0.20258	0.34731	-0.21402	0.09314	-0.29028	-0.11608	0.03486
0.15063	-0.37943	-0.08355	-0.31318	-0.02504	0.25389	-0.08796	0.05075	0.18678	0.37081	-0.02256	-0.0478	-0.17414	-0.12997	0.0626
0.18288	-0.26828	-0.11082	-0.37068	-0.20991	0.14144	-0.13639	-0.03609	0.07043	0.35599	-0.36234	-0.00371	-0.36161	-0.15254	0.07366
0.20085	-0.2651	-0.0491	-0.11992	-0.12419	0.23678	-0.01492	0.01111	0.15324	0.3611	-0.28448	0.01927	-0.30251	-0.11394	0.07294
0.197	-0.37467	-0.32916	-0.24957	-0.14625	0.27178	-0.125	0.06243	0.22425	0.31212	-0.03012	0.03975	-0.17692	-0.12086	0.01139
0.18908	-0.35159	-0.22481	-0.26574	-0.12421	0.37728	-0.08541	-0.00226	0.30388	0.31726	-0.14691	0.00294	-0.24983	-0.0866	0.0276
0.21106	-0.3301	-0.30944	-0.37498	-0.16239	0.2791	-0.18097	0.05468	0.22297	0.31201	-0.13722	0.0355	-0.23926	-0.11964	0.04301
0.22026	-0.40708	-0.00928	0.04537	-0.25235	0.18072	0.03493	-0.07995	0.09874	0.39034	-0.05848	-0.08463	-0.18607	-0.25474	0.05931
0.20446	-0.23076	-0.03704	-0.19047	-0.30095	0.19616	-0.12804	-0.08288	0.12988	0.33311	-0.24837	-0.03699	-0.28947	-0.18947	0.08642
0.07907	-0.36269	0.01331	0.04338	-0.07885	-0.02091	0.03642	-0.16666	-0.05019	0.37099	-0.17544	-0.0424	-0.23799	-0.20973	0.09898
0.16371	-0.46592	-0.32697	-0.13975	-0.00421	0.25275	-0.11612	0.06239	0.17462	0.37903	-0.16276	-0.01041	-0.26917	-0.12994	0.09023
0.18479	-0.34825	-0.17139	-0.22574	-0.08067	0.2033	-0.09792	-0.00501	0.13754	0.34373	-0.15096	0.0177	-0.24205	-0.27007	0.05645
0.18754	-0.26669	0.05949	0.01933	-0.12943	0.06224	-0.01386	-0.11852	0.00405	0.38808	-0.16769	-0.0494	-0.23968	-0.18527	0.06868
0.21497	-0.35406	-0.18629	-0.2098	-0.2324	0.22487	-0.11908	-0.04289	0.16157	0.38085	-0.21772	0.00676	-0.28013	-0.11142	0.05845
0.2158	-0.40955	-0.18567	-0.22679	-0.08773	0.277	-0.16167	0.04261	0.21362	0.34267	-0.22653	-0.00643	-0.30215	-0.10172	0.04986

CAR_INITI	AENCE_N_	AN_PREG	N_PREC	RECURS	E_OKAZ	GANIZATI	GANIZATI	ORIGIN_	URENCE_	RENCE_P	NCE_P30	E_P4_PR	P4_PR_R	PDGF_PD
0.29581	-0.02376	0.2578	0.00786	0.14103	-0.01974	-0.28237	-0.39709	0.06424	-0.11797	-0.01847	-0.39694	-0.12896	0.20285	-0.20796
0.29617	-0.03545	0.27287	0.06224	0.26481	0.01722	-0.12912	-0.26889	0.04454	-0.09372	-0.00794	-0.37862	-0.09776	0.14283	-0.16983
0.3189	-0.01454	0.22921	-0.20913	0.14632	0.04093	-0.29769	-0.35523	0.14752	-0.03683	-0.00338	-0.34143	-0.02121	0.16795	-0.12675
0.32249	0.01487	0.2682	-0.21068	0.17877	0.00619	-0.35864	-0.43258	0.09898	-0.32308	-0.03126	-0.36919	0.0464	0.19292	-0.08827
0.27442	0.02736	0.24579	-0.22032	0.18714	0.04227	-0.17051	-0.32265	0.07319	-0.20881	-0.00031	-0.27062	0.01836	0.22205	-0.10862
0.26998	0.00917	0.27017	-0.02999	0.1553	0.10734	-0.36005	-0.42091	0.1221	-0.30097	0.04718	-0.24256	-0.03308	0.09752	-0.13276
0.30033	-0.0282	0.2422	0.14016	0.19828	0.0318	-0.24485	-0.37827	0.07619	-0.34913	0.04392	-0.37663	0.03097	0.17306	-0.10162
0.30897	0.07599	0.26128	-0.15996	0.18866	-0.01095	-0.29998	-0.40368	-0.03096	-0.36255	-0.05276	-0.30892	0.04497	0.18672	-0.11402
0.30312	0.04317	0.25071	-0.17088	0.15819	0.0211	-0.32024	-0.41475	-0.01104	-0.28097	-0.11866	-0.31925	0.07928	0.1707	-0.08826
0.29334	0.01482	0.24885	-0.21994	0.19613	0.05728	-0.15149	-0.33322	-0.01646	-0.34626	-0.0295	-0.24998	0.0458	0.18048	-0.12714
0.29776	0.01993	0.29042	0.02932	0.17734	0.03539	-0.30879	-0.36302	0.16698	-0.0364	0.11475	-0.4496	-0.10868	0.1057	-0.20634
0.31193	-0.03287	0.25298	0.00235	0.17771	0.034	-0.20698	-0.35505	0.14487	-0.02916	-0.03486	-0.41059	-0.07049	0.13285	-0.16313
0.27191	-0.00242	0.29614	-0.15794	0.22532	-0.02077	-0.12877	-0.29339	0.15428	-0.0458	0.06039	-0.31386	-0.10384	0.16606	-0.12173
0.30239	0.00126	0.25353	0.1091	0.2176	-0.05768	-0.40127	-0.45284	-0.04126	-0.34205	-0.01773	-0.36346	0.00389	0.16397	-0.09184
0.27611	-0.00986	0.25945	-0.20263	0.24162	0.0286	-0.20486	-0.35335	0.10971	-0.25717	-0.04511	-0.33763	-0.10881	0.17988	-0.20018
0.30353	-0.08342	0.24331	-0.23002	0.23041	0.0701	-0.20856	-0.31798	0.15083	-0.30199	-0.02097	-0.28283	-0.07402	0.15933	-0.1016
0.28875	-0.02715	0.25928	-0.22845	0.15622	-0.04785	-0.31038	-0.40883	-0.1192	-0.02606	-0.05571	-0.36556	0.03781	0.18607	-0.08477
0.28768	0.00464	0.26718	-0.18747	0.165	-0.03469	-0.23604	-0.36046	-0.00064	-0.37974	-0.04609	-0.32024	0.05061	0.18616	-0.10597

F_PDGFR	GF_PDGF	DL_PD1_	RKIN_ME	FERENCE	PKR_EI	N_MEDIAT	CE_PP2A	ERENCE	PRNP_PI	OK_PRK	ROMOTIO	PTH_PTH	RINE_SAL	AGE_PATH
0.14739	0.2079	-5.9E-06	0.19372	0.21577	0.10983	0.12883	0.04216	0.17664	0.19761	0.35815	0.17239	0.28476	0.03435	-0.02561
0.16991	0.13761	-0.05269	0.15062	0.24612	0.14674	-0.00402	-0.03032	-0.01352	0.1382	0.32538	0.20018	0.24362	-0.01317	-0.03127
0.1699	0.16361	0.07487	0.12849	0.15493	0.15112	0.06877	0.06807	0.26477	0.17992	0.34521	0.20187	0.23074	0.07086	0.02394
0.16288	0.17844	0.12561	0.14639	0.22081	0.08577	0.14443	0.0822	0.18069	0.20981	0.35702	0.23081	0.28597	0.06744	0.0386
0.15588	0.21905	0.09781	0.21961	0.25663	0.17515	0.11986	0.02842	0.14264	0.22561	0.35309	0.2189	0.26593	0.15189	0.13605
0.1982	0.09182	0.05475	0.13206	0.21332	0.04195	0.17514	0.04773	0.22626	0.14887	0.26614	0.18521	0.22411	0.1321	0.12891
0.14041	0.16421	0.11579	0.18177	0.2255	0.07466	0.18836	-0.0228	0.15708	0.19179	0.34205	0.19928	0.26974	0.13245	0.10564
0.1917	0.17088	0.16291	0.1518	0.26031	0.10962	0.17496	0.04394	-0.02248	0.24011	0.36162	0.23716	0.29182	0.1078	0.08298
0.18796	0.1498	0.16581	0.13507	0.23949	0.09238	0.16347	0.05278	0.05198	0.25445	0.33918	0.24507	0.29356	0.12899	0.10227
0.16232	0.16445	0.11056	0.15404	0.26537	0.0881	0.21914	0.01689	-0.02031	0.25691	0.33999	0.23355	0.28112	0.09922	0.0663
0.1034	0.10074	0.04609	0.2021	0.16316	0.14138	0.11319	-0.04952	0.3197	0.15255	0.32303	0.24763	0.26612	0.20892	0.20586
0.14488	0.13592	-0.01885	0.17238	0.2597	0.14358	0.16176	-0.01792	0.24111	0.14336	0.34816	0.2017	0.28206	0.20905	0.18586
0.01378	0.17889	-0.07202	0.11696	0.20975	0.1468	0.12472	0.10733	0.22687	0.04175	0.35539	0.286	0.29837	0.05657	0.03538
0.15737	0.15745	0.01356	0.14409	0.23704	0.09665	0.09699	0.04258	-0.03918	0.20056	0.32944	0.24202	0.27529	0.11282	0.07774
0.14341	0.18016	0.00382	0.14911	0.23645	0.14542	0.11406	0.0101	0.21581	0.22012	0.34406	0.21382	0.28615	0.10954	0.08332
0.10552	0.16752	-0.14331	0.15141	0.16222	0.17842	0.09437	0.0579	0.30381	0.06297	0.34109	0.24025	0.18421	0.08199	0.04599
0.11395	0.17754	0.11833	0.14979	0.21864	0.07387	0.14661	0.06554	-0.1721	0.18686	0.34976	0.2535	0.30642	0.06867	0.01346
0.17116	0.17473	0.14915	0.16305	0.22848	0.06522	0.09082	0.02402	0.06309	0.22166	0.35755	0.2658	0.27316	0.08078	0.05403

ED_MICR	AC_CDC42	CE_RAD5	CRUITME	ULATION_	ON_OF_	GULATION	GULATIO	K_RAS_EF	RTK_R	LATION_	OTION_OF	RAS_ER	OF_GF_	RTK_RAS
0.17532	0.29484	0.09333	0.04855	-0.18761	-0.01588	-0.0202	-0.29716	0.16762	-0.03185	0.31129	0.00954	0.16312	0.19467	-0.15775
0.2065	0.18775	0.19002	0.04657	-0.30573	-0.07258	-0.10949	-0.38789	0.14364	-0.04878	0.25684	-0.04994	0.15377	0.14853	-0.21156
0.19022	0.22917	0.00871	0.03298	-0.15359	-0.02262	0.1011	-0.11375	0.13741	-0.02326	0.24192	0.0608	0.13375	0.18872	-0.21579
0.22972	0.24496	0.06913	0.07049	0.0021	0.11277	0.21597	-0.09161	0.16103	-0.06567	0.28226	0.03763	0.10884	0.07278	-0.22995
0.21088	0.27546	0.12704	-0.00215	-0.16755	0.03025	0.10543	-0.36505	0.1548	-0.05121	0.2475	0.13913	0.20722	0.23952	-0.22469
0.17706	0.17552	0.1084	0.04866	0.17042	0.25779	0.39104	-0.2678	0.18024	-0.05773	0.19759	0.06002	0.07696	0.10246	-0.2303
0.17109	0.2622	0.09328	0.05194	0.13032	0.27208	0.44749	-0.14259	0.1308	-0.07856	0.23804	-0.08671	0.10296	0.14199	-0.22333
0.23474	0.25666	0.11692	-0.01716	0.0244	0.08249	0.23638	0.01278	0.1166	-0.05237	0.29529	0.07551	0.08205	0.11332	-0.24429
0.23597	0.24774	0.03906	-0.12269	0.01167	0.14775	0.2627	-0.16497	0.13105	-0.06256	0.28684	0.035	0.05652	0.10518	-0.23168
0.21236	0.26115	0.11884	0.01785	0.06842	0.15561	0.24689	-0.1505	0.14675	-0.04565	0.3007	-0.11498	0.06658	0.12569	-0.2665
0.24846	0.18887	-0.02874	-0.00913	-0.0787	0.14517	0.14812	-0.21422	0.15029	-0.1147	0.19009	0.04589	0.12484	0.05416	-0.05632
0.20415	0.21925	0.04175	0.00195	0.0592	0.26089	0.19873	-0.04353	0.13738	-0.11311	0.297	-0.13763	0.06215	0.10834	-0.15677
0.25095	0.23357	0.11577	0.05353	-0.07737	0.13024	0.0895	-0.37299	0.16386	0.04707	0.24324	0.13374	0.22486	0.21049	-0.06322
0.23121	0.20401	-3.2E-05	0.009	-0.01539	0.10221	0.17422	-0.20646	0.16583	-0.07406	0.2689	0.11821	0.18232	0.19696	-0.31142
0.22408	0.27739	-0.0091	0.02066	-0.00276	0.0615	0.17303	-0.10521	0.15141	-0.01961	0.27363	0.02687	0.07731	0.11624	-0.2885
0.21174	0.24473	-0.04288	-0.00539	-0.28569	-0.02862	-0.13417	-0.21176	0.17306	-0.06845	0.23967	0.03763	0.15446	0.20247	-0.05635
0.23567	0.21823	0.04019	0.03112	-0.09214	0.00776	0.02801	-0.04929	0.15522	-0.07335	0.2408	0.14226	0.18803	0.17501	-0.25831
0.24718	0.25191	0.02	0.00924	-0.15946	0.05858	0.12184	-0.30187	0.11355	-0.07509	0.29265	-0.08425	0.10631	0.07214	-0.22607

RENCE_R	S_SIGNAL	ELN_VL	ENIN_AN	CE_RETRO	E_RIG_I	E_RIG_I	RTK_PL	ENCE_SE	SKELETA	NDLE_AS	RAD_S	C3_TAC	R31_MEDIA	TCR_PL
0.01988	-0.16534	-0.11314	-0.21455	0.17405	0.03764	0.05843	-0.02965	-0.05144	0.33538	-0.13094	0.07422	-0.04709	0.07728	-0.08455
-0.01175	-0.19901	-0.1095	-0.22987	0.20714	0.01676	0.10071	-0.09659	0.19095	0.27774	-0.06844	0.09707	-0.13757	0.01961	-0.14491
-0.00193	-0.19882	-0.05877	-0.15114	0.20669	-0.04146	0.02885	-0.15504	0.03505	0.31134	-0.12403	0.09984	0.01264	-0.06804	-0.13143
0.15859	-0.18247	-0.02469	0.25418	0.24798	-0.05179	0.06628	-0.09597	-0.1529	0.31528	-0.09995	0.10267	0.04286	-0.00577	-0.06643
0.11615	-0.1045	-0.07911	-0.0347	0.22519	-0.0278	0.09625	-0.0796	-0.07469	0.27553	-0.18172	0.01677	-0.01643	0.07977	0.03282
0.12369	-0.19852	-0.04371	0.38594	0.19102	-0.06272	0.0273	-0.04903	0.11485	0.15933	-0.04699	0.07486	-0.09959	-0.04518	0.04167
0.01841	-0.23032	-0.01662	0.18161	0.19576	-0.08044	0.05982	-0.10598	0.05461	0.30749	-0.10566	0.09511	-0.05745	-0.01441	0.01485
0.06229	-0.15126	-0.06157	0.24326	0.25734	-0.0181	0.09701	-0.14113	0.23974	0.34709	-0.13714	0.04096	0.03542	0.03877	-0.06691
0.08763	-0.15007	-0.04549	0.25048	0.26759	-0.04171	0.03943	-0.14163	-0.02713	0.34175	-0.21163	0.054	-0.00022	0.00037	-0.03767
0.13215	-0.10561	-0.06191	0.30368	0.23487	-0.04641	0.08138	-0.10247	-0.3264	0.29135	-0.14553	0.10646	0.02234	-0.00664	0.07684
-0.01351	-0.17407	-0.10197	0.08772	0.27038	0.01346	0.0099	-0.05507	-0.11414	0.37117	-0.07705	-0.00466	-0.05397	0.04001	-0.12545
0.07396	-0.19566	-0.06775	0.14444	0.20798	-0.03034	0.00487	-0.01196	-0.28237	0.36349	-0.164	0.01966	-0.02007	0.00778	-0.03333
-0.02303	-0.29763	-0.04246	0.07046	0.26175	-0.002	-0.05539	-0.10006	-0.06042	0.24996	-0.0706	0.0425	-0.10139	-0.09973	-0.23354
0.06111	-0.20118	-0.04357	-0.04404	0.24527	-0.03847	0.11176	-0.14826	0.10322	0.35519	-0.1424	0.00323	-0.05085	-0.05871	-0.07042
0.04776	-0.09971	-0.11571	0.10093	0.24083	0.03427	0.08247	-0.13208	0.14585	0.33087	-0.14909	0.07763	-0.10965	0.07172	-0.12501
-0.04438	-0.23231	-0.01376	-0.16417	0.22686	0.0157	-0.01997	-0.10679	-0.40849	0.32935	-0.13971	0.17957	-0.04493	-0.14849	-0.23759
0.03974	-0.26859	-0.06914	0.20627	0.25668	-0.03677	0.03013	-0.10486	-0.20609	0.30634	-0.08383	0.00506	0.0058	-0.03192	-0.08965
0.08347	-0.14605	-0.06264	-0.01621	0.27021	-0.03066	0.0719	-0.11167	-0.11203	0.32655	-0.13342	0.03138	-0.03254	0.02575	-0.01404

ENCE_T	CE_TEST	RING_OF_	TGFA_EG	A_EGFR	FA_EGFR	CE_TGF	HYROID	HT_JUNC	TLR1_2	E_TLR2_4	CE_TLR3	CE_TLR3	E_TLR3	E_TLR4_
0.12632	0.00575	-0.0527	-0.18347	-0.1893	0.17266	-0.23526	0.1775	0.31992	-0.06245	0.06618	-0.16219	-0.01761	-0.01162	-0.16219
0.23204	-0.33798	-0.01944	-0.17962	-0.21283	0.10525	-0.06764	0.22171	0.32326	-0.10392	-0.02571	-0.26176	-0.21895	-0.06092	-0.26176
0.17418	-0.17498	0.05485	-0.14537	-0.23925	0.12529	-0.14229	0.12774	0.29125	-0.08935	0.00381	-0.27243	-0.21719	-0.07878	-0.27243
0.11589	-0.34623	0.07966	-0.07675	-0.13249	0.14159	-0.19239	0.17271	0.29044	-0.06999	0.00735	-0.25208	-0.19306	-0.05838	-0.25208
0.14008	-0.03872	0.08948	-0.10671	-0.08573	0.18371	-0.10784	0.10013	0.2862	-0.02592	0.0482	-0.25568	-0.29206	-0.00958	-0.25568
0.17865	-0.25545	-0.01604	-0.12491	-0.07996	0.06174	-0.18835	0.12519	0.28846	-0.13176	-0.04476	-0.26316	-0.25612	-0.13309	-0.26316
0.19543	-0.27648	0.01737	-0.10347	-0.186	0.12493	-0.07485	0.08043	0.28465	-0.06589	0.01397	-0.34872	-0.34032	-0.05081	-0.34872
0.06916	-0.33699	0.12508	-0.1073	-0.11675	0.13289	-0.15266	0.14839	0.26586	-0.00699	0.02974	-0.14101	0.01709	-0.0058	-0.14101
0.07384	-0.31903	0.12015	-0.08455	-0.08751	0.11212	-0.11651	0.11694	0.25076	-0.04025	0.0196	-0.23465	-0.11062	-0.0522	-0.23465
0.13193	-0.22664	0.07233	-0.12564	-0.08103	0.13	-0.15988	0.14533	0.26625	-0.02132	0.04151	-0.2375	-0.18584	-0.0065	-0.2375
0.1899	-0.07162	0.00276	-0.09882	-0.09318	0.1039	-0.07408	0.14227	0.31625	-0.13312	-0.03387	-0.26039	-0.20786	-0.09943	-0.26039
0.19334	-0.16518	-0.03913	-0.13736	-0.13554	0.11364	-0.18832	0.13784	0.33292	-0.11205	0.03576	-0.29048	-0.27268	-0.08649	-0.29048
0.19417	-0.25316	-0.04201	-0.11506	-0.32659	0.1428	-0.06063	0.13592	0.35417	-0.18653	0.00032	-0.23502	-0.23428	-0.18613	-0.23502
0.18714	-0.23524	0.0756	-0.09267	-0.21984	0.11959	-0.16718	0.1036	0.31627	-0.03795	0.00785	-0.27696	-0.17562	-0.00726	-0.27696
0.13684	-0.25925	0.02358	-0.20954	-0.18578	0.14227	-0.17306	0.11564	0.28747	-0.07693	0.01314	-0.19007	-0.08508	-0.04347	-0.19007
0.20069	-0.16089	-0.00145	-0.04034	-0.20726	0.1598	-0.10077	0.08436	0.33975	-0.1168	0.02181	-0.2073	-0.31513	-0.08791	-0.2073
0.17268	-0.20435	0.05129	-0.0882	-0.18818	0.14035	-0.19608	0.11142	0.32113	-0.13192	-0.02794	-0.29784	-0.24397	-0.11594	-0.29784
0.16417	-0.23066	0.12222	-0.09438	-0.13035	0.13844	-0.19287	0.12508	0.27927	-0.02575	0.03071	-0.20976	-0.12929	-0.01944	-0.20976

CE_TNF_	NCE_TNF_	E_TNF_R	RIP_DEPE	PTIONAL_	CE_TRAN	RENCE_TR	E_TRAPPI	H_TRHR_	_TSC1_2_I	TSH_DU	NCE_TSH_	NTERFER	CE_TYPE_	NTERFER	RENCE_TY	REFEREN	RENCE_V	CE_VGC	CRENCE_V	IENCE_WI
-0.10798	-0.1051	-0.12591	0.23849	0.07593	0.0009	0.40838	0.10858	0.03836	-0.15497	-0.04709	0.12434	0.35221	0.11177	0.09527	0.01696	0.07073	0.25261	0.21662	0.43872	0.099
-0.11943	-0.12687	-0.30203	0.09279	0.18682	-0.02297	0.42587	0.04202	-0.02523	-0.15814	-0.13757	0.14927	0.31535	0.09269	0.05161	-0.1027	-0.071	0.13336	0.20257	0.46205	0.18337
-0.10677	-0.09298	-0.21856	0.27641	0.10583	-0.02931	0.41035	0.07847	0.08608	-0.16457	0.01264	0.10098	0.31331	0.17059	0.13861	-0.02272	-0.09081	0.20377	0.18608	0.45245	0.24414
-0.08205	-0.1102	-0.14517	0.22496	0.06348	-0.06388	0.42615	0.08153	0.12796	-0.13472	0.04286	0.08861	0.33237	0.14061	0.11506	0.13144	-0.04275	0.14168	0.17941	0.42705	0.02308
-0.05622	-0.03739	-0.12476	0.18276	0.0843	-0.03252	0.41857	0.0716	0.07291	-0.20513	-0.01643	0.11278	0.35276	0.11781	0.09644	0.00912	0.07114	0.14161	0.18686	0.43968	0.11626
-0.11052	-0.08744	-0.20584	0.26181	0.11601	-0.03814	0.42939	0.0538	0.0036	-0.17732	-0.09959	0.10789	0.33415	0.14369	0.11465	-0.02364	0.11424	0.13725	0.19719	0.43043	0.10164
-0.09262	-0.09166	-0.21592	0.20344	0.11735	-0.01192	0.42843	-0.02297	0.04053	-0.09769	-0.05745	0.08701	0.33743	0.2043	0.17299	-0.18365	-0.06439	0.11041	0.22488	0.43942	0.17738
-0.03101	-0.07251	-0.08299	0.08708	0.05142	-0.11264	0.42393	0.13451	0.1146	-0.19808	0.03542	0.11061	0.37376	0.17424	0.17065	0.07716	-0.02944	0.16779	0.15023	0.40405	-0.23657
-0.0692	-0.06094	-0.09229	0.12955	0.05129	-0.11931	0.41555	0.11059	0.08403	-0.19424	-0.00022	0.12028	0.36899	0.16277	0.14146	-0.05504	-0.17439	0.14655	0.16503	0.41707	-0.0805
-0.04083	-0.03946	-0.10997	0.05793	0.06315	-0.05042	0.41126	0.09568	0.10306	-0.2078	0.02234	0.11343	0.37554	0.2188	0.19831	0.00294	-0.08454	0.14661	0.18024	0.41623	-0.2038
-0.15706	-0.14942	-0.30773	0.29988	0.12844	-0.05112	0.4225	-0.02335	0.03803	-0.14038	-0.05397	0.12638	0.2701	0.06935	0.0126	-0.23305	0.12907	0.22073	0.22407	0.42967	0.22459
-0.1256	-0.08284	-0.15447	0.27182	0.09217	-0.06367	0.42496	0.13593	0.08339	-0.17106	-0.02007	0.12739	0.36988	0.16032	0.11551	-0.08992	-0.13919	0.21022	0.17741	0.43213	0.16488
-0.14541	-0.09071	-0.17573	0.25039	0.15135	-0.01988	0.41695	0.12477	0.01866	-0.00379	-0.10139	0.15619	0.29665	0.07038	0.01849	-0.10735	0.05969	0.18157	0.21065	0.45216	0.25912
-0.06167	-0.1032	-0.19624	0.06491	0.11132	-0.09076	0.42643	0.03489	0.04206	-0.15138	-0.05085	0.09117	0.30245	0.10575	0.06217	0.04692	-0.07545	0.16581	0.1785	0.43972	-0.2459
-0.08857	-0.09707	-0.18837	0.25361	0.10189	-0.09637	0.43217	0.09699	0.00135	-0.16623	-0.10965	0.15811	0.30217	0.12734	0.10354	0.13158	-0.01336	0.15163	0.19031	0.43503	-0.02026
-0.16401	-0.12758	-0.27019	0.29716	0.11943	-0.04278	0.42452	0.05427	0.04514	-0.08503	-0.04493	0.09888	0.32115	0.09902	0.01622	0.10535	0.03526	0.19835	0.21631	0.45081	0.3264
-0.12979	-0.12	-0.21267	-0.00329	0.09628	-0.06783	0.42794	0.10188	0.09198	-0.1813	0.0058	0.18258	0.28756	0.08086	0.0429	-0.01315	-0.11875	0.18506	0.21747	0.44661	-0.04069
-0.05422	-0.06251	-0.092	0.12968	0.03499	-0.05094	0.41057	0.11398	0.03932	-0.20588	-0.03254	0.10825	0.36648	0.16651	0.13871	0.01999	-0.10241	0.16913	0.16974	0.44766	-0.0347

E_WNTS	NT_SIGN	NT_SIGN	_SIGNA	IENCE_W	PLIFIED_C	PLIFIED_	PLIFIED_	FIED_EGF	_EGFR_T	D_EGFR_	IED_FGF	D_FGFR_	IFIED_M	FIED_ME	D_MYCN
-0.24948	-0.29047	-0.29047	-0.33468	-0.06795	-0.42875	-0.33377	-0.42875	-0.18347	0.11393	0.17266	-0.17914	0.2079	-0.42838	-0.17914	-0.06095
-0.40196	-0.40356	-0.40356	-0.08156	-0.10791	-0.35124	-0.17977	-0.35124	-0.17962	0.13302	0.10525	-0.14297	0.13761	-0.3788	-0.14297	-0.08355
-0.23028	-0.10041	-0.10041	-0.15748	-0.04628	-0.24203	-0.23569	-0.24203	-0.14537	0.12686	0.12529	-0.10191	0.16361	-0.31999	-0.10191	-0.12738
-0.44477	-0.44548	-0.44548	-0.41659	-0.13358	-0.44178	-0.3242	-0.44178	-0.07675	0.12363	0.14159	-0.03481	0.17844	-0.4462	-0.03481	-0.23199
-0.4157	-0.41747	-0.41747	-0.37978	-0.15327	-0.21684	-0.23284	-0.21684	-0.10671	0.12035	0.18371	-0.06193	0.21905	-0.29756	-0.06193	-0.13465
-0.29915	-0.30298	-0.30298	-0.46222	-0.07449	-0.32077	-0.22631	-0.32077	-0.12491	0.15752	0.06174	-0.09645	0.09182	-0.32021	-0.09645	-0.06053
-0.30646	-0.24943	-0.24943	-0.31214	-0.10886	-0.40373	-0.18806	-0.40373	-0.10347	0.09982	0.12493	-0.05493	0.16421	-0.38706	-0.05493	-0.07482
-0.4142	-0.41536	-0.41536	-0.37907	-0.18684	-0.32975	-0.27133	-0.32975	-0.1073	0.14946	0.13289	-0.06897	0.17088	-0.36881	-0.06897	-0.15283
-0.412	-0.41377	-0.41377	-0.48617	-0.17054	-0.42223	-0.3454	-0.42223	-0.08455	0.14533	0.11212	-0.03699	0.1498	-0.42186	-0.03699	-0.1612
-0.39218	-0.3944	-0.3944	-0.35318	-0.25575	-0.32588	-0.2853	-0.32588	-0.12564	0.12553	0.13	-0.08309	0.16445	-0.35997	-0.08309	-0.08533
-0.37751	-0.43051	-0.43051	-0.29364	-0.08754	-0.46662	-0.02777	-0.46662	-0.09882	0.10661	0.1039	-0.16743	0.10074	-0.45648	-0.16743	-0.13643
-0.40842	-0.38119	-0.38119	-0.28078	-0.17256	-0.45464	-0.3538	-0.45464	-0.13736	0.12019	0.11364	-0.1489	0.13592	-0.45428	-0.1489	-0.14173
-0.03124	-0.08455	-0.08455	0.26458	0.05403	-0.44495	-0.09174	-0.44495	-0.11506	-0.01449	0.1428	-0.07917	0.17889	-0.28856	-0.07917	-0.07899
-0.41268	-0.38224	-0.38224	-0.43086	-0.15436	-0.42558	-0.25786	-0.42558	-0.09267	0.11489	0.11959	-0.04001	0.15745	-0.41297	-0.04001	-0.17451
-0.38537	-0.34279	-0.34279	-0.41466	-0.11066	-0.4601	-0.19283	-0.4601	-0.20954	0.10468	0.14227	-0.19075	0.18016	-0.40287	-0.19075	-0.15382
-0.21313	-0.23481	-0.23481	-0.44302	-0.02752	-0.24573	-0.22175	-0.24573	-0.04034	0.10373	0.1598	-0.07772	0.16752	-0.25476	-0.07772	-0.10382
-0.42225	-0.40197	-0.40197	-0.35811	-0.14559	-0.45905	-0.3621	-0.45905	-0.0882	0.07584	0.14035	-0.03475	0.17754	-0.45869	-0.03475	-0.18862
-0.39801	-0.37872	-0.37872	-0.48864	-0.10344	-0.36674	-0.30971	-0.36674	-0.09438	0.13158	0.13844	-0.057	0.17473	-0.39514	-0.057	-0.13928

D_MYCN_	MPLIFIED	LIFIED_M	LIFIED_M	IED_PDG	PDGFR_	D_PDGFR	MPLIFIED	REXPRES	XPRESSI	FUSION_K	SION_KIN	EXPRESS	2_FUSION	ESSION_T	TO_DYST
-0.13176	-0.24185	-0.24185	-0.06164	-0.17914	0.14739	0.2079	-0.24557	-0.25815	0.04067	-0.13757	0.2079	0.39147	0.10872	-0.25339	0.18078
-0.20979	-0.27468	-0.27468	-0.06195	-0.14297	0.16991	0.13761	-0.11426	-0.26033	0.09814	-0.10981	0.13761	0.36413	0.1573	-0.21783	0.22145
-0.1654	-0.20251	-0.20251	-0.03884	-0.10191	0.1699	0.16361	-0.1596	-0.17741	0.13132	-0.03878	0.16361	0.42487	0.09757	-0.10927	0.22608
-0.14779	-0.26238	-0.26238	-0.07781	-0.03481	0.16288	0.17844	-0.19445	-0.15047	0.11372	-0.05935	0.17844	0.35124	0.11794	-0.05665	0.17742
-0.1462	-0.18057	-0.18057	-0.04621	-0.06193	0.15588	0.21905	-0.1465	-0.16604	0.13953	-0.04216	0.21905	0.14498	0.1338	-0.06944	0.13095
-0.0074	-0.13436	-0.13436	0.03459	-0.09645	0.1982	0.09182	-0.30202	-0.16609	0.10896	-0.07096	0.09182	0.23122	0.07682	-0.09971	0.16744
-0.27567	-0.32895	-0.32895	-0.00713	-0.05493	0.14041	0.16421	-0.12833	-0.1537	0.16541	-0.02739	0.16421	0.33003	0.08196	-0.04991	0.14753
-0.04686	-0.17113	-0.17113	-0.08168	-0.06897	0.1917	0.17088	-0.17199	-0.15064	0.1079	-0.00161	0.17088	0.26659	0.11138	-0.05902	0.18329
-0.06236	-0.19327	-0.19327	-0.13476	-0.03699	0.18796	0.1498	-0.07334	-0.12848	0.17095	0.00592	0.1498	0.24094	0.119	-0.01909	0.1552
-0.01497	-0.15068	-0.15068	-0.0592	-0.08309	0.16232	0.16445	-0.06697	-0.15747	0.18879	-0.01963	0.16445	0.20855	0.11031	-0.0584	0.14865
-0.21473	-0.30066	-0.30066	0.04	-0.16743	0.1034	0.10074	-0.12585	-0.15984	0.13567	-0.03081	0.10074	0.41172	0.13504	-0.21518	0.17744
-0.30016	-0.35863	-0.35863	-0.10232	-0.1489	0.14488	0.13592	-0.14897	-0.14545	0.05558	-0.0597	0.13592	0.32421	0.13256	-0.13909	0.21642
-0.46186	-0.45349	-0.45349	0.03682	-0.07917	0.01378	0.17889	-0.06403	-0.21483	0.08697	-0.03778	0.17889	0.41438	0.14998	-0.16432	0.25178
-0.23058	-0.31262	-0.31262	-0.07933	-0.04001	0.15737	0.15745	-0.16021	-0.1787	0.08749	-0.04496	0.15745	0.33817	0.11898	-0.07839	0.14378
-0.18866	-0.2828	-0.2828	-0.09492	-0.19075	0.14341	0.18016	-0.26057	-0.24431	0.10857	-0.09684	0.18016	0.36568	0.12295	-0.23146	0.23284
-0.35882	-0.29818	-0.29818	-0.05434	-0.07772	0.10552	0.16752	-0.07641	-0.09736	0.09071	-0.03064	0.16752	0.38388	0.06481	-0.13768	0.30285
-0.27603	-0.34342	-0.34342	-0.11291	-0.03475	0.11395	0.17754	-0.2345	-0.15344	0.09355	-0.04599	0.17754	0.32989	0.16107	-0.04572	0.18262
-0.12084	-0.22788	-0.22788	-0.08639	-0.057	0.17116	0.17473	-0.11588	-0.13355	0.11602	0.00387	0.17473	0.38152	0.10239	-0.03737	0.16515

TATION_AC	ATION_AC	REXPRE	REXPRES	XPRESSI	FUSION_	SION_KIN	SION_KIN	PRESSIO	REXPRE	REXPRES	1_FUSIO	REXPRES	REXPRES	XPRESS
0.2079	-0.05229	-0.2067	-0.23951	0.17266	-0.2571	0.11778	0.22935	0.16726	-0.15995	-0.25815	0.27814	-0.00505	0.26412	0.18503
0.13761	-0.03355	-0.19746	-0.25291	0.10525	-0.23755	0.03098	0.14035	0.1037	-0.18073	-0.26033	0.38506	-0.04088	0.13281	0.14828
0.16361	-0.01145	-0.15994	-0.16312	0.12529	-0.1453	0.07003	0.17809	0.09708	-0.14468	-0.17741	0.16206	-0.02598	0.2008	0.13191
0.17844	0.01443	-0.117	-0.1052	0.14159	-0.11043	0.10889	0.18603	0.07598	-0.13903	-0.15047	-0.0044	-0.07007	0.21088	0.15308
0.21905	0.04133	-0.14203	-0.12581	0.18371	-0.11258	0.14942	0.23667	0.12411	-0.13786	-0.16604	-0.09618	-0.09509	0.24114	0.20379
0.09182	-0.0494	-0.15186	-0.1348	0.06174	-0.16363	0.06071	0.09287	0.10071	-0.15242	-0.16609	0.09935	-0.01722	0.11252	0.09048
0.16421	-0.01335	-0.13822	-0.11225	0.12493	-0.11889	0.095	0.17813	0.08843	-0.14405	-0.1537	0.21473	-0.05712	0.21374	0.16948
0.17088	-0.00853	-0.14135	-0.10929	0.13289	-0.12447	0.1209	0.17856	0.10764	-0.15438	-0.15064	-0.20187	-0.13587	0.21574	0.14149
0.1498	-0.00531	-0.12217	-0.08248	0.11212	-0.09039	0.11913	0.15232	0.09117	-0.1466	-0.12848	-0.18918	-0.11608	0.19702	0.11455
0.16445	-0.01111	-0.15954	-0.11444	0.13	-0.11589	0.13177	0.1706	0.10434	-0.18089	-0.15747	-0.2334	-0.22162	0.21164	0.12843
0.10074	-0.08773	-0.13777	-0.11407	0.1039	-0.21292	0.04114	0.1238	0.16356	-0.12289	-0.15984	0.06165	-0.01196	0.15156	0.06617
0.13592	-0.07388	-0.15095	-0.12726	0.11364	-0.18296	0.08346	0.1501	0.18881	-0.12002	-0.14545	-0.37925	-0.11016	0.20002	0.09914
0.17889	0.01146	-0.14656	-0.1907	0.1428	-0.15146	0.03974	0.21014	0.15893	-0.08581	-0.21483	0.27532	0.09712	0.20018	0.14688
0.15745	0.01749	-0.13079	-0.139	0.11959	-0.14037	0.05106	0.16514	0.05723	-0.14453	-0.1787	-0.24661	-0.08763	0.1606	0.14579
0.18016	-0.09038	-0.21438	-0.24576	0.14227	-0.27121	0.12412	0.19997	0.0537	-0.22594	-0.24431	0.2651	-0.03876	0.25923	0.14995
0.16752	-0.00219	-0.06566	-0.07251	0.1598	-0.11092	0.05494	0.19826	0.21854	-0.03507	-0.09736	0.22899	0.03273	0.21966	0.15725
0.17754	0.04297	-0.12492	-0.11235	0.14035	-0.10368	0.07345	0.1902	0.09367	-0.10858	-0.15344	0.20116	-0.07514	0.17842	0.18103
0.17473	0.01458	-0.13093	-0.0884	0.13844	-0.09944	0.10998	0.18394	0.11235	-0.13931	-0.13355	-0.02501	-0.02707	0.21215	0.14483

T_FUSIO	_OF_CDK	RASSF1_	T_OF_VHL	REXPRES	XPRESSI	_FUSION_	_FUSION	AID_H_T	CTIVATED	VATED_E	IVATED_E	VATED_F	TED_GNA	TED_GNA	TED_GR
0.49356	-0.30461	0.042	0.10673	-0.06795	0.2079	-0.11543	-0.29936	0.17657	-0.2452	0.08687	0.17266	0.2079	0.10872	0.17269	0.13197
0.49603	-0.19257	0.04166	0.08314	-0.10791	0.13761	-0.00685	-0.20306	0.19388	-0.26268	0.00106	0.10525	0.13761	0.1573	0.15728	0.02657
0.49585	-0.1998	0.0295	0.10692	-0.04628	0.16361	-0.13514	-0.16625	0.19341	-0.18298	0.03345	0.12529	0.16361	0.09757	0.12375	0.07166
0.49532	-0.30642	0.06505	0.09144	-0.13358	0.17844	-0.1031	-0.37999	0.16395	-0.1408	0.07381	0.14159	0.17844	0.11794	0.15047	0.13298
0.49568	-0.1814	0.17837	0.1204	-0.15327	0.21905	-0.12321	-0.28902	0.16798	-0.15242	0.11447	0.18371	0.21905	0.1338	0.17605	0.05798
0.4948	-0.14133	0.01194	0.15242	-0.07449	0.09182	-0.10962	-0.21528	0.20226	-0.18096	0.03131	0.06174	0.09182	0.07682	0.13334	-0.00888
0.49532	-0.11482	-0.01185	0.09937	-0.10886	0.16421	-0.12279	-0.30829	0.15959	-0.16087	0.05759	0.12493	0.16421	0.08196	0.14092	0.0145
0.49444	-0.25176	0.0317	0.09619	-0.18684	0.17088	-0.11102	-0.35453	0.19613	-0.15589	0.08384	0.13289	0.17088	0.11138	0.16853	0.04947
0.49462	-0.29897	0.02229	0.04414	-0.17054	0.1498	-0.16897	-0.34776	0.18911	-0.13277	0.08136	0.11212	0.1498	0.119	0.18029	0.01774
0.49497	-0.16685	0.04963	0.06738	-0.25575	0.16445	-0.12672	-0.31311	0.17082	-0.15582	0.09667	0.13	0.16445	0.11031	0.1719	0.06776
0.49585	-0.05388	-0.05856	0.17843	-0.08754	0.10074	-0.10056	-0.22554	0.12398	-0.13204	0.05179	0.1039	0.10074	0.13504	0.1612	0.05453
0.4955	-0.28406	-0.0417	0.06168	-0.17256	0.13592	-0.20256	-0.38147	0.1801	-0.16372	0.06481	0.11364	0.13592	0.13256	0.19122	0.14601
0.49427	-0.13787	0.08645	0.16012	0.05403	0.17889	-0.14065	-0.10715	0.07031	-0.17457	0.00927	0.1428	0.17889	0.14998	0.17914	0.05036
0.49585	-0.25756	0.10164	0.10849	-0.15436	0.15745	-0.09587	-0.23168	0.17087	-0.18209	0.01519	0.11959	0.15745	0.11898	0.15516	0.04498
0.49568	-0.19897	-0.03812	0.02418	-0.11066	0.18016	-0.18103	-0.21887	0.16592	-0.27519	0.08697	0.14227	0.18016	0.12295	0.17781	0.01973
0.49515	-0.26913	0.02174	0.15042	-0.02752	0.16752	-0.1495	-0.23655	0.153	-0.0656	0.05916	0.1598	0.16752	0.06481	0.1016	0.03192
0.4955	-0.3325	0.14403	0.09679	-0.14559	0.17754	-0.07093	-0.29659	0.13527	-0.15015	0.03776	0.14035	0.17754	0.16107	0.1854	0.09462
0.49585	-0.26942	0.07858	0.103	-0.10344	0.17473	-0.15619	-0.40442	0.17969	-0.13056	0.07514	0.13844	0.17473	0.10239	0.1591	0.0479

TED_ITPR	NJ5_TO_	ACTIVATE	TIVATED_	IVATED_K	VATED_K	ACTIVATE	TIVATED_	CTIVATE	ED_PRKA	ACTIVATE	BERRANT	_ABETA_T	ABERRA
0.00138	0.17657	-0.2571	0.2079	0.22935	-0.10363	-0.2571	0.2079	-0.17808	-0.01515	-0.17914	0.19595	0.28609	0.17327
-0.08708	0.19388	-0.23755	0.13761	0.14035	-0.08362	-0.23755	0.13761	-0.13715	0.09741	-0.14297	0.16022	0.28471	0.08814
0.07166	0.19341	-0.1453	0.16361	0.17809	-0.0485	-0.1453	0.16361	-0.10495	-0.0019	-0.10191	0.17292	0.26776	0.11962
0.07524	0.16395	-0.11043	0.17844	0.18603	-0.02751	-0.11043	0.17844	-0.0817	0.01291	-0.03481	0.1541	0.26597	0.11692
-0.02135	0.16798	-0.11258	0.21905	0.23667	0.01284	-0.11258	0.21905	-0.13965	0.04946	-0.06193	0.16456	0.30861	0.17513
-0.12262	0.20226	-0.16363	0.09182	0.09287	-0.09648	-0.16363	0.09182	-0.15145	-0.04685	-0.09645	0.15359	0.28478	0.07088
-0.06124	0.15959	-0.11889	0.16421	0.17813	-0.05853	-0.11889	0.16421	-0.12645	-0.06077	-0.05493	0.16282	0.2678	0.1484
0.05353	0.19613	-0.12447	0.17088	0.17856	-0.06484	-0.12447	0.17088	-0.14299	-0.00653	-0.06897	0.13212	0.29201	0.12932
0.01028	0.18911	-0.09039	0.1498	0.15232	-0.05943	-0.09039	0.1498	-0.12898	-0.00894	-0.03699	0.11956	0.29565	0.09551
0.00761	0.17082	-0.11589	0.16445	0.1706	-0.06633	-0.11589	0.16445	-0.17197	-0.01446	-0.08309	0.12307	0.27993	0.1282
0.01804	0.12398	-0.21292	0.10074	0.1238	-0.12024	-0.21292	0.10074	-0.17589	0.0354	-0.16743	0.1987	0.27653	0.08334
0.02337	0.1801	-0.18296	0.13592	0.1501	-0.12574	-0.18296	0.13592	-0.14962	0.0214	-0.1489	0.18018	0.27435	0.08973
-0.03442	0.07031	-0.15146	0.17889	0.21014	-0.00255	-0.15146	0.17889	-0.07128	0.04114	-0.07917	0.18287	0.30526	0.15192
-0.00292	0.17087	-0.14037	0.15745	0.16514	-0.01938	-0.14037	0.15745	-0.09554	0.0123	-0.04001	0.16186	0.28067	0.10708
-0.07733	0.16592	-0.27121	0.18016	0.19997	-0.15849	-0.27121	0.18016	-0.18429	0.01838	-0.19075	0.13998	0.28453	0.13379
0.03739	0.153	-0.11092	0.16752	0.19826	-0.01715	-0.11092	0.16752	-0.04948	-0.03846	-0.07772	0.20028	0.26522	0.12502
0.04575	0.13527	-0.10368	0.17754	0.1902	0.01596	-0.10368	0.17754	-0.08201	0.05345	-0.03475	0.20752	0.28133	0.14237
-0.01515	0.17969	-0.09944	0.17473	0.18394	-0.02474	-0.09944	0.17473	-0.11991	-0.03152	-0.057	0.14018	0.29265	0.13444

ERRANT	_CROSS	ERRANT_	ERRANT_A	ABERRAN	ABERRAN	BERRANT	ED_ABER	RRANT_A	RRANT_	ABERRANT	T_HTT_TO	ABERRAN	ABERRAN
0.21677	0.00092	0.09566	0.24659	-0.20938	0.23469	0.19066	0.28834	0.28834	0.26079	0.22045	0.28696	0.14029	-0.08567
0.26715	0.01488	0.14918	0.22733	-0.18769	0.18564	0.1487	0.26566	0.26566	0.28487	0.26281	0.28437	0.17317	-0.23042
0.24576	0.03389	0.0937	0.20237	-0.33479	0.16735	0.12612	0.21927	0.21927	0.22579	0.26132	0.26957	0.14413	-0.18698
0.26615	0.04276	0.14712	0.24068	-0.36324	0.17893	0.14945	0.2041	0.2041	0.19947	0.29471	0.26686	0.19396	-0.12905
0.2408	0.08183	0.09475	0.22298	-0.38107	0.1654	0.1407	0.2226	0.2226	0.23423	0.27022	0.31066	0.18347	-0.13524
0.23692	-0.00933	0.13656	0.22683	-0.27679	0.16373	0.12599	0.25032	0.25032	0.25974	0.23355	0.28611	0.16187	-0.32997
0.24544	0.05898	0.13205	0.24134	-0.17309	0.18065	0.15199	0.26396	0.26396	0.26474	0.2476	0.26546	0.12971	-0.2031
0.27496	0.04119	0.1397	0.23403	-0.39603	0.13861	0.11448	0.18726	0.18726	0.20307	0.30234	0.29209	0.21086	-0.12096
0.27375	0.07704	0.14828	0.22151	-0.3739	0.11218	0.10507	0.16319	0.16319	0.18377	0.30373	0.29894	0.191	-0.08394
0.26177	0.0896	0.16471	0.25146	-0.31916	0.13544	0.13008	0.17202	0.17202	0.18309	0.29147	0.28204	0.20052	-0.10717
0.30596	0.0613	0.17757	0.22194	-0.2559	0.19837	0.16142	0.27068	0.27068	0.26652	0.31844	0.27912	0.20433	-0.04094
0.27389	0.0034	0.12554	0.24502	-0.29772	0.21356	0.15873	0.24967	0.24967	0.24965	0.26574	0.27606	0.16743	-0.17643
0.3283	0.05919	-0.03677	0.20877	-0.12341	0.21022	0.17048	0.28919	0.28919	0.28999	0.33577	0.30266	0.25632	-0.0999
0.28809	0.00578	0.10737	0.20954	-0.44151	0.16727	0.12163	0.23257	0.23257	0.25381	0.29938	0.28233	0.19785	-0.11863
0.25788	0.04004	0.10219	0.19722	-0.341	0.15059	0.12931	0.21779	0.21779	0.23007	0.27755	0.28507	0.18808	-0.18751
0.28961	0.04106	0.09857	0.17582	-0.32229	0.18991	0.14151	0.26937	0.26937	0.26993	0.29257	0.26748	0.19895	-0.10779
0.31115	0.0388	0.1402	0.29178	-0.14306	0.23112	0.18344	0.30065	0.30065	0.28406	0.31495	0.27653	0.19164	-0.18244
0.29805	0.04548	0.1637	0.26575	-0.39005	0.14934	0.12324	0.20333	0.20333	0.22645	0.33002	0.29518	0.22632	-0.16949

ABERRA	BERRANT_	ABERRAN	NT_HTT_	ABERRAN	D_ABERR	SED_ABE	BERRANT_	BERRANT	ABERRANT_	SNCA_T	BERRANT	ERRANT_	ABERRA
-0.0497	0.31315	0.17788	0.11899	0.16179	-0.18257	0.21714	-0.04532	0.32714	0.21662	0.28534	0.13651	0.07618	0.27188
-0.03805	0.27056	0.13911	0.17299	0.19254	-0.19815	0.19525	-0.02228	0.28053	0.20257	0.28381	0.16999	0.12372	0.27177
-0.09572	0.27876	0.13833	0.11142	0.19189	-0.20811	0.15685	-0.15007	0.22933	0.18608	0.26777	0.13981	0.07525	0.2548
-0.14559	0.23798	0.14818	0.09401	0.22956	-0.29716	0.15974	-0.02502	0.24394	0.17941	0.26865	0.19611	0.13588	0.28629
-0.05088	0.14481	0.13635	0.09	0.21046	-0.23612	0.18287	0.00558	0.25032	0.18686	0.31925	0.20123	0.0934	0.3698
-0.08394	0.2208	0.12339	0.1606	0.17574	-0.219	0.18494	0.02428	0.26901	0.19719	0.29514	0.18126	0.12617	0.36462
-0.16586	0.28843	0.14838	0.12568	0.17854	-0.25031	0.19782	-0.16098	0.26755	0.22488	0.26622	0.1293	0.11351	0.2546
-0.07912	0.24859	0.1089	0.05912	0.24131	-0.23616	0.14598	0.00851	0.1919	0.15023	0.29407	0.21495	0.13106	0.29919
-0.05719	0.22958	0.1139	0.0793	0.25382	-0.186	0.15392	-0.07028	0.17341	0.16503	0.29888	0.19087	0.1397	0.29527
-0.08156	0.23578	0.13417	0.10259	0.21945	-0.2249	0.16288	-0.00924	0.20383	0.18024	0.28134	0.19903	0.15649	0.27128
-0.03304	0.29324	0.16594	0.17985	0.25783	-0.17291	0.2121	-0.05982	0.27398	0.22407	0.27782	0.19983	0.16005	0.28783
-0.06159	0.28427	0.15209	0.11077	0.19258	-0.19639	0.17079	-0.05237	0.30062	0.17741	0.2771	0.16709	0.09759	0.29272
-0.04345	0.28073	0.15405	0.16122	0.24767	-0.19714	0.20835	0.06117	0.2877	0.21065	0.29997	0.25106	-0.08739	0.25015
-0.09212	0.21994	0.12687	0.06685	0.22987	-0.2505	0.16124	-0.06721	0.24758	0.1785	0.28008	0.19282	0.08267	0.24343
-0.07932	0.27142	0.1246	0.08495	0.22201	-0.29678	0.17505	0.00588	0.24329	0.19031	0.28758	0.19128	0.08192	0.30416
-0.07197	0.27835	0.15068	0.15778	0.21178	-0.19425	0.19281	0.00429	0.26563	0.21631	0.26796	0.19833	0.07013	0.28725
-0.0974	0.31641	0.16293	0.11302	0.2377	-0.30397	0.20375	-0.07939	0.30072	0.21747	0.27666	0.19193	0.11093	0.24312
-0.07235	0.27178	0.1227	0.09252	0.25435	-0.23343	0.16385	-0.01412	0.22839	0.16974	0.30086	0.23769	0.16241	0.34418

ABERRA	T_SOD1_T	ABERRA	ABERRA	BERRAN	ERRANT_S	ERRANT_B	BERRANT	NACTIVAT	TP1AI_TO	TP2B3_TO	VATED_A	NACTIVAT
0.21204	0.30717	0.15837	0.43861	0.13317	0.1251	0.08676	0.13839	0.086	0.21166	0.17657	-0.08707	0.086
0.19111	0.3022	0.17826	0.42188	0.14753	0.00084	0.13786	0.15845	0.06435	0.22556	0.19388	-0.22774	0.06435
0.15049	0.29245	0.18626	0.43501	0.14842	0.12264	0.09025	0.15527	0.10241	0.22656	0.19341	-0.13894	0.10241
0.16203	0.28915	0.17181	0.41159	0.19206	0.10665	0.14816	0.20778	0.02539	0.19862	0.16395	-0.08941	0.02539
0.21013	0.32723	0.21098	0.41569	0.18403	0.01569	0.09334	0.19587	-0.00518	0.20092	0.16798	-0.03781	-0.00518
0.21395	0.30884	0.12386	0.42032	0.15668	-0.03568	0.12821	0.16754	0.07566	0.23276	0.20226	-0.23197	0.07566
0.19871	0.2897	0.1942	0.40689	0.13041	0.04062	0.12742	0.14412	0.056	0.19778	0.15959	-0.14632	0.056
0.15254	0.30909	0.17475	0.39479	0.19762	0.02085	0.13799	0.21746	-0.05644	0.22164	0.19613	-0.12655	-0.05644
0.15371	0.31486	0.21713	0.41056	0.22515	-0.01131	0.14692	0.24067	-0.03138	0.21711	0.18911	-0.05748	-0.03138
0.16054	0.29848	0.22918	0.40772	0.1649	0.05233	0.16641	0.18187	-0.16682	0.20302	0.17082	-0.05359	-0.16682
0.2053	0.29723	0.19741	0.41795	0.20759	0.06339	0.17248	0.22009	0.10943	0.16728	0.12398	-0.03985	0.10943
0.17042	0.29926	0.15405	0.42822	0.14086	0.11132	0.11078	0.15252	-0.02218	0.21688	0.1801	-0.16045	-0.02218
0.19956	0.32287	0.1779	0.415	0.14806	0.06279	-0.06848	0.16124	0.13527	0.11485	0.07031	-0.08214	0.13527
0.15297	0.30279	0.14556	0.39491	0.16704	0.0549	0.09931	0.1873	0.02445	0.2025	0.17087	-0.07528	0.02445
0.17958	0.30781	0.18503	0.43958	0.21056	0.00532	0.09606	0.21945	0.06138	0.20202	0.16592	-0.10781	0.06138
0.19164	0.29288	0.15901	0.43099	0.14762	0.08524	0.08549	0.15672	0.12981	0.19386	0.153	-0.06356	0.12981
0.20419	0.29808	0.15499	0.40106	0.17823	0.11965	0.12362	0.19336	0.01658	0.17179	0.13527	-0.11016	0.01658
0.182	0.31347	0.18743	0.41261	0.18822	0.01463	0.16346	0.2033	0.07839	0.20919	0.17969	-0.1413	0.07839

CTIVATED	TIVATED_	TIVATED_	N_TO_PI	S2_TO_PI	PDE11A	ATED_PIN	ED_PINK	TED_PINK	ED_PRKA	ED_PRKA
0.16556	0.2079	0.2079	0.20292	0.21668	0.08486	0.08376	0.1167	0.06818	0.08486	0.18747
0.19968	0.13761	0.13761	0.14294	0.18021	0.14741	0.13346	0.09167	0.12056	0.14741	0.15972
0.23369	0.16361	0.16361	0.14806	0.16375	0.08658	0.08442	0.10914	0.10749	0.08658	0.14099
0.17442	0.17844	0.17844	0.14247	0.18356	0.10115	0.14499	0.09071	0.12013	0.10115	0.15788
0.19808	0.21905	0.21905	0.20843	0.24604	0.13864	0.08835	0.12945	0.17709	0.13864	0.19153
0.18999	0.09182	0.09182	0.13955	0.16651	0.05043	0.12287	0.0382	0.12892	0.05043	0.15035
0.16818	0.16421	0.16421	0.18069	0.22221	0.05836	0.12325	0.14051	0.16245	0.05836	0.16414
0.13265	0.17088	0.17088	0.15607	0.18529	0.09945	0.13593	0.04705	0.13974	0.09945	0.17815
0.1444	0.1498	0.1498	0.13354	0.17128	0.10696	0.14567	0.1015	0.18677	0.10696	0.19334
0.13254	0.16445	0.16445	0.15147	0.19336	0.09465	0.1656	0.14917	0.2044	0.09465	0.18346
0.23598	0.10074	0.10074	0.18863	0.24501	0.11186	0.16874	0.16716	0.13465	0.11186	0.17531
0.19903	0.13592	0.13592	0.16453	0.21019	0.12685	0.10612	0.07603	0.07826	0.12685	0.20207
0.21276	0.17889	0.17889	0.15436	0.14486	0.12429	-0.08265	0.07651	0.11045	0.12429	0.211
0.23377	0.15745	0.15745	0.16865	0.18167	0.09015	0.09254	0.07902	0.08261	0.09015	0.15868
0.17831	0.18016	0.18016	0.14318	0.1652	0.10548	0.09014	0.10958	0.12808	0.10548	0.19385
0.24701	0.16752	0.16752	0.21144	0.18262	0.04762	0.07832	0.15646	0.06812	0.04762	0.12585
0.14172	0.17754	0.17754	0.15554	0.18688	0.12678	0.12043	0.12234	0.11331	0.12678	0.19134
0.16431	0.17473	0.17473	0.16826	0.19251	0.07533	0.16316	0.12229	0.1423	0.07533	0.16196

TED_RAS	CTIVATED	ON_INACT	QLN2_TO_	CP_TO_2	REXPRES	RMATION	N_KINAS	RPSC_TO	MATION_P	MATION_	ITION_PR	FORMATI	RMATION_	FUSION_R	EXPRES	RG_FUSIO
0.20319	0.32714	-0.02344	0.29088	0.30807	-0.07684	-0.03451	0.22935	0.28404	0.2225	0.25593	0.19276	0.2075	-0.3331	0.18036	-0.23951	0.0315
0.2024	0.28053	0.02961	0.29027	0.30657	-0.11815	-0.00778	0.14035	0.28313	0.18908	0.26434	0.11804	0.1912	-0.21261	0.16667	-0.25291	0.09216
0.17338	0.22933	0.06827	0.2745	0.29246	-0.09952	-0.05646	0.17809	0.26865	0.14205	0.26518	0.1798	0.15373	-0.20292	0.16144	-0.16312	0.07779
0.21223	0.24394	0.02895	0.27307	0.29023	-0.24846	-0.02213	0.18603	0.26612	0.16567	0.25401	0.23134	0.15698	-0.30245	0.16952	-0.1052	0.12894
0.23649	0.25032	0.05354	0.31464	0.33112	-0.06739	0.00807	0.23667	0.30966	0.17773	0.26066	0.23171	0.17947	-0.19077	0.18851	-0.12581	0.12236
0.19114	0.26901	-0.03069	0.29335	0.30825	-0.24373	0.0323	0.09287	0.28694	0.18112	0.27362	0.17503	0.18773	-0.12454	0.19159	-0.1348	-0.30367
0.18774	0.26755	0.06746	0.27321	0.2892	-0.22828	-0.07813	0.17813	0.26646	0.18972	0.25793	0.2059	0.201	-0.16646	0.16727	-0.11225	0.13645
0.22567	0.1919	0.04168	0.29781	0.31341	-0.12453	0.01716	0.17856	0.2941	0.13779	0.29957	0.26177	0.15266	-0.02889	0.15156	-0.10929	0.17936
0.2276	0.17341	0.04979	0.3015	0.3188	-0.22414	-0.03309	0.15232	0.29774	0.13321	0.28222	0.28054	0.1499	-0.1219	0.14137	-0.08248	0.01898
0.21993	0.20383	0.07057	0.28726	0.30392	-0.21924	0.01753	0.1706	0.28332	0.16513	0.29368	0.2832	0.167	-0.07134	0.18344	-0.11444	0.15064
0.19655	0.27398	0.04737	0.28205	0.29726	-0.05097	0.02447	0.1238	0.27853	0.1953	0.29329	0.14844	0.21026	-0.04457	0.23586	-0.11407	0.07254
0.23821	0.30062	0.06305	0.28335	0.29863	-0.08805	-0.02227	0.1501	0.27543	0.19	0.271	0.11447	0.16869	-0.20416	0.16796	-0.12726	0.13807
0.18321	0.2877	0.07933	0.30618	0.32264	0.03031	0.07891	0.21014	0.30248	0.20169	0.28059	-0.03353	0.20779	-0.08156	0.09447	-0.1907	0.03693
0.19239	0.24758	0.04341	0.28663	0.30404	-0.19476	-0.02974	0.16514	0.28031	0.14827	0.24655	0.21459	0.15405	-0.28149	0.19935	-0.139	0.07592
0.19754	0.24329	-0.00658	0.29172	0.30857	-0.22027	0.03101	0.19997	0.28931	0.18362	0.3201	0.22271	0.18904	-0.00722	0.18185	-0.24576	0.18984
0.1453	0.26563	0.02438	0.27325	0.28886	-0.00949	0.04619	0.19826	0.27177	0.18121	0.32542	0.0065	0.20604	0.06553	0.09602	-0.07251	0.04588
0.21897	0.30072	-0.01276	0.2828	0.30023	-0.11307	-0.05364	0.1902	0.27619	0.19706	0.23709	0.19278	0.20254	-0.30139	0.20313	-0.11235	0.11107
0.20755	0.22839	0.05877	0.30008	0.31587	-0.20796	0.01055	0.18394	0.2967	0.16436	0.29809	0.23196	0.16883	-0.03505	0.11619	-0.0884	-0.28254

KINAS	4_INTEGI	DISS_2PA	LK1_PAT	LK2_PAT	SYNUCLE	UTROPHI	IN_RECE	THRAX_P	AP1_PAT	OWNSTRE	RF6_PATH	AFFICKI	RF_3PAT	INGENOM
0.22935	0.12037	0.26172	0.12098	-0.06604	0.05732	0.11213	0.09901	0.18437	-0.04114	0.26671	-0.06304	0.11242	0.14764	0.12958
0.14035	0.10154	0.23445	0.14076	0.17675	0.08794	0.0767	0.05441	0.09733	-0.05538	0.24406	-0.10472	0.08361	0.13632	0.09777
0.17809	0.09581	0.22506	0.11505	-0.02338	0.08053	0.14259	0.094	0.17787	-0.01618	0.26103	-0.05046	0.11221	0.13516	0.13292
0.18603	0.09252	0.24488	0.10385	-0.00248	0.10671	0.15511	0.11164	0.15955	-0.04316	0.28056	0.00406	0.10791	0.1685	0.14952
0.23667	0.04343	0.21768	0.12314	-0.03319	0.12277	0.16595	0.10445	0.19838	-0.02362	0.29272	0.00033	0.06999	0.16193	0.16615
0.09287	0.08187	0.21377	0.08639	-0.06396	0.0993	0.08566	0.07389	0.10158	-0.0437	0.22106	-0.07751	0.07032	0.13912	0.10772
0.17813	0.10071	0.24085	0.13366	0.04412	0.10727	0.14961	0.11367	0.17579	-0.01738	0.27189	-0.04676	0.09442	0.13782	0.1437
0.17856	0.09125	0.21194	0.13498	-0.0017	0.11889	0.19209	0.13671	0.18268	-0.03782	0.29868	0.01363	0.07834	0.19117	0.16871
0.15232	0.06303	0.22906	0.14419	-0.02982	0.13603	0.19335	0.13937	0.21948	-0.02294	0.30318	0.00434	0.09905	0.17406	0.16056
0.1706	0.05834	0.20628	0.1425	-0.05048	0.12393	0.16602	0.1342	0.19365	-0.04449	0.27313	0.02142	0.07819	0.16709	0.15397
0.1238	0.14248	0.22594	0.12828	0.13095	0.0539	0.0958	0.05902	0.11534	-0.05063	0.25076	-0.01686	0.10895	0.13642	0.12227
0.1501	0.14239	0.20287	0.09914	-0.01388	0.04024	0.10721	0.06897	0.1638	-0.03852	0.27801	-0.05131	0.10403	0.12711	0.11818
0.21014	0.14246	0.23631	0.15049	0.11462	0.06809	0.01536	0.0758	0.13508	-0.05337	0.28278	0.01736	0.13931	0.12876	0.09623
0.16514	0.07833	0.23923	0.11057	0.01115	0.11666	0.1419	0.0908	0.16849	-0.08637	0.25885	-0.00083	0.10191	0.17143	0.12374
0.19997	0.08329	0.22746	0.12823	0.018	0.08718	0.13325	0.07833	0.16937	-0.03838	0.27621	-0.05483	0.07788	0.12394	0.10536
0.19826	0.15775	0.24128	0.15387	0.11532	0.07438	0.02693	0.0579	0.10895	-0.06165	0.2839	-0.04666	0.12993	0.12708	0.09663
0.1902	0.12987	0.2439	0.08999	-0.02792	0.07574	0.16091	0.1035	0.14452	-0.03079	0.28237	-0.00383	0.09709	0.1498	0.15404
0.18394	0.09332	0.23951	0.11874	-0.00544	0.09337	0.15394	0.11822	0.18889	-0.07113	0.27778	0.00301	0.10722	0.16439	0.17363

AR_PATH	R_TF_PA	ATF2_PAT	ATM_PATH	ATR_PATH	ORA_A_P	ORA_B_P	INTEGRIN	3_OPN_PA	ARD1_PA	CR_5PAT	TENIN_D	TENIN_N	BMP_PATH	SPASE_P
0.14291	0.05358	0.08118	0.10708	0.1212	0.02288	0.07131	0.08769	0.14314	0.18014	0.0459	0.02452	0.20309	0.01964	0.14068
0.15523	0.03937	0.03554	0.13139	0.13611	-0.03008	0.1016	0.04636	0.12092	0.23266	-0.00812	0.03263	0.22251	0.05052	0.12154
0.12969	0.04246	0.03552	0.15583	0.15698	-0.0013	0.11241	0.11225	0.16268	0.20923	0.03802	0.05821	0.21082	0.03949	0.16169
0.09421	0.01116	-0.00423	0.11551	0.13774	-0.03772	0.07934	0.12034	0.17513	0.19932	0.08099	-0.00985	0.21228	0.0243	0.13014
0.0915	0.02839	0.08657	0.13121	0.11933	-0.02999	0.10426	0.07215	0.15167	0.18494	0.07916	-0.03063	0.15749	0.02056	0.16448
0.11475	0.01157	0.07106	0.12906	0.14293	-0.06855	0.1029	0.074	0.097	0.20485	0.00579	0.03576	0.16237	0.05399	0.15858
0.12583	0.0307	0.04342	0.14782	0.12207	-0.00922	0.07749	0.14357	0.19215	0.22814	0.03891	0.00791	0.18212	0.059	0.17104
0.06753	0.03225	0.03933	0.08967	0.06653	0.02275	0.08624	0.10933	0.19392	0.1798	0.10512	-0.02987	0.18409	0.06051	0.14538
0.07167	0.03907	0.06517	0.10516	0.06935	0.02683	0.09424	0.12727	0.18265	0.17495	0.09568	-0.04591	0.16601	0.04989	0.16005
0.07888	0.049	0.04059	0.11828	0.08478	0.0084	0.08797	0.10114	0.17568	0.17658	0.0898	-0.06045	0.17079	0.0289	0.17582
0.11399	0.03634	0.07936	0.15766	0.17301	-0.03265	0.07337	0.07998	0.14014	0.21793	0.00072	0.01997	0.22242	0.08205	0.13602
0.11243	0.03731	0.05515	0.1295	0.14736	-0.07071	0.06844	0.09715	0.14196	0.21217	0.02267	0.03652	0.22816	0.04319	0.13917
0.18386	0.03432	0.03924	0.15416	0.19786	0.00319	0.09202	0.09303	0.11946	0.23455	-0.04638	0.1438	0.24869	0.10502	0.1457
0.0974	0.03324	0.02904	0.13485	0.07473	0.02258	0.09533	0.08832	0.14975	0.19436	0.05123	0.03253	0.19017	0.02838	0.15192
0.12698	0.0169	0.04104	0.13896	0.14141	-0.01416	0.08632	0.09235	0.1646	0.2142	0.03677	0.03932	0.20737	0.04207	0.15089
0.1127	0.03546	0.02536	0.2126	0.17636	-0.05294	0.09554	0.07906	0.10966	0.25652	-0.02757	0.12619	0.19142	0.07974	0.11001
0.11684	0.04291	0.00839	0.13288	0.05076	-0.02455	0.09265	0.09739	0.1586	0.20415	0.03986	0.03629	0.21306	0.02686	0.14814
0.07649	0.0003	0.0538	0.13307	0.09048	0.01785	0.08094	0.11222	0.15523	0.20868	0.08855	-0.01966	0.13786	0.01945	0.16007

CD40_PAT	DOWNST	8_TCR_P	DC42_PA	42_REG	RAMIDE_P	ADIAN_P	MYB_PAT	XCR3_PA	XCR4_PAT	A_NP63	MA_PK_PA	E2F_PATH	KERATIN	N_NASCE
-0.0279	0.07759	0.03286	0.18821	-0.00291	0.10866	0.19526	0.16418	0.06152	0.08735	0.0284	0.16117	-0.04336	0.19494	0.18846
-0.07399	0.00448	-0.00679	0.1515	-0.00925	0.07178	0.27753	0.13815	0.03835	0.04481	0.00506	0.20557	0.01802	0.18637	0.17704
-0.01163	0.04695	0.03393	0.18649	0.01014	0.10773	0.22473	0.17053	0.05694	0.09643	0.02579	0.19492	0.00813	0.21472	0.18703
-0.01916	0.04866	0.0542	0.18249	0.06796	0.10382	0.16007	0.17839	0.10688	0.12442	-0.01442	0.1681	-0.01697	0.23251	0.18082
0.0266	0.14833	0.12519	0.164	0.0604	0.12663	0.23448	0.18343	0.12501	0.1254	-0.05567	0.16625	-0.02993	0.16997	0.12381
-0.03997	0.07918	0.09098	0.15105	0.03696	0.08894	0.34555	0.14145	0.04191	0.08339	-0.03281	0.19861	-0.00392	0.16442	0.15475
0.03993	0.08787	0.08289	0.18593	0.02736	0.12024	0.22181	0.15618	0.09148	0.12439	-0.10468	0.1877	0.00477	0.2055	0.17371
0.02379	0.06526	0.0678	0.17601	0.06242	0.11236	0.15586	0.17554	0.12028	0.13222	-0.03608	0.1499	-0.05775	0.17332	0.14251
0.03421	0.07508	0.06559	0.18469	0.07453	0.11685	0.1801	0.16983	0.12875	0.13601	-0.05568	0.15736	-0.04675	0.21057	0.15256
0.05506	0.13366	0.12995	0.16549	0.0787	0.13036	0.19211	0.17547	0.11582	0.14449	-0.06994	0.1706	-0.02958	0.17332	0.12945
-0.02999	-0.00486	-0.0085	0.16766	-0.06138	0.04982	0.25447	0.15828	0.02669	0.05594	0.04928	0.21323	0.04334	0.23146	0.21053
0.0265	0.07001	0.04924	0.18728	0.01115	0.10032	0.21079	0.15567	0.03466	0.08235	0.02103	0.17891	0.0024	0.18335	0.17738
-0.02639	-0.00814	-0.07326	0.18483	-0.04574	0.11001	0.25712	0.11824	0.0272	0.04914	0.03805	0.22133	0.03128	0.19321	0.21471
-0.00154	0.04575	0.06805	0.17736	0.033	0.10507	0.2236	0.17814	0.08089	0.1044	-0.05927	0.19759	-0.0655	0.19734	0.17902
-0.01899	0.02019	-0.00174	0.17287	-0.00041	0.11939	0.20581	0.16589	0.0415	0.0671	0.01124	0.16919	-0.01309	0.17897	0.17045
-0.02109	0.02789	-0.05058	0.1728	-0.06568	0.11393	0.22145	0.1199	0.00711	0.04624	0.03874	0.21146	0.03055	0.19134	0.20703
-0.06766	0.02921	0.02713	0.18519	0.03296	0.10456	0.20174	0.17701	0.11404	0.10602	0.02042	0.17945	-0.04064	0.21843	0.18866
0.0375	0.1049	0.09838	0.18466	0.0648	0.12829	0.17477	0.151	0.11527	0.11475	0.00603	0.18913	-0.07325	0.24592	0.17546

STABILI	DTHELIN	A2_FWD	HA_FWD	P.B_FWD	FINB_REV	EPO_PAT	GENOMIC	OWNSTR	ERNALIZ	PTOR_PR	2_ERBB3	RBB4_PA	NETWOR	GENOMIC
0.15199	0.15404	0.09689	-0.05374	0.13285	0.03788	0.02855	0.12369	0.18	0.05396	0.15615	0.09227	0.01454	0.05977	0.09865
0.13711	0.10614	0.05024	-0.02414	0.07692	0.03126	0.0057	0.17168	0.15974	0.00176	0.10751	0.05483	-0.05941	0.00046	0.19859
0.13956	0.15438	0.11018	-0.03841	0.11601	0.04525	0.04123	0.16251	0.16527	0.05006	0.1408	0.0752	-0.00361	0.00714	0.14026
0.12457	0.18374	0.08444	-0.00758	0.10707	0.09909	0.05743	0.14561	0.17793	0.06836	0.12966	0.09103	0.02135	-0.03546	0.10743
0.07033	0.16867	0.09572	-0.00795	0.11425	0.078	0.0739	0.17178	0.17101	0.0798	0.13751	0.10023	0.04333	-0.00392	0.14452
0.10073	0.11928	0.06313	-0.00977	0.03838	0.05283	0.03821	0.14063	0.15268	0.01439	0.10762	0.02075	-0.03099	-0.01092	0.08472
0.1161	0.17139	0.11865	0.01052	0.11295	0.10203	0.04766	0.15541	0.17602	0.03312	0.15513	0.07698	0.0141	-0.03137	0.11278
0.10301	0.17184	0.10312	0.021	0.10884	0.10296	0.0951	0.12516	0.18017	0.08427	0.14818	0.09699	0.02338	-0.03398	0.06368
0.11561	0.15931	0.11593	0.0376	0.10615	0.11848	0.09997	0.14322	0.16784	0.07579	0.1465	0.08099	0.01543	-0.05438	0.09142
0.08198	0.18147	0.12383	0.04389	0.08568	0.09284	0.09145	0.14277	0.15948	0.06124	0.12669	0.06898	0.0108	-0.05271	0.09887
0.16999	0.11339	0.11944	0.00608	0.10567	0.03957	0.00783	0.14257	0.16716	0.03562	0.11679	0.03783	-0.02159	0.12497	0.14861
0.13367	0.15582	0.05105	-0.0194	0.07817	0.02429	0.02389	0.15582	0.17491	0.01992	0.10957	0.04928	-0.0238	0.08458	0.10219
0.1839	0.13071	0.17678	0.00358	0.15853	0.01844	-0.02392	0.15606	0.21115	0.04533	0.13781	0.10775	0.02419	0.07991	0.19779
0.12424	0.15011	0.07051	-0.0132	0.10528	0.04825	0.01128	0.14854	0.17933	0.04029	0.1384	0.06575	-0.02763	-0.02996	0.14351
0.13368	0.14096	0.08524	-0.03488	0.10059	0.03429	0.03122	0.14866	0.1647	0.03084	0.11746	0.04811	-0.01386	-0.03745	0.13321
0.16145	0.14231	0.09371	-0.02632	0.09696	0.00299	-0.04891	0.1545	0.18791	0.02776	0.12606	0.08055	-0.01457	0.13563	0.14108
0.11377	0.15414	0.10421	0.00696	0.12982	0.0823	0.02496	0.15139	0.18884	0.07789	0.14823	0.09511	0.00601	0.01038	0.1253
0.10673	0.16462	0.14635	0.04172	0.10297	0.09897	0.08806	0.13424	0.17185	0.08847	0.13762	0.09044	0.03382	-0.01896	0.10818

NGENOMI	FAK_PATH	CONI_PA	AS_PATH	CER1_PA	GF_PATH	OXM1_PA	OXO_PAT	FRA_PATH	PICAN_1	MCSF_PA	_CLASSI	_CLASSI	_CLASSI	GEHOG_2
0.12021	0.10976	-0.07705	-0.06538	0.04225	0.06913	-0.12429	0.17455	-0.08874	-0.07524	0.12439	0.09865	0.21054	0.11871	-0.02517
0.12375	0.06569	-0.01541	-0.07102	-0.01918	0.05701	-0.05799	0.16973	-0.07931	-0.08498	0.07993	0.11116	0.20869	0.12176	-0.05867
0.15041	0.12279	-0.01898	-0.03955	0.06083	0.09122	-0.04565	0.15537	-0.06826	-0.06852	0.10691	0.08171	0.20105	0.09958	0.08043
0.18431	0.11901	-0.03393	0.00477	0.07019	0.06593	-0.13922	0.15541	-0.07435	-0.01018	0.13902	0.11742	0.21852	0.07531	0.15031
0.16956	0.10063	-0.05528	0.00492	0.08605	0.02684	-0.10299	0.13915	-0.09597	-0.00662	0.14513	0.10375	0.23557	0.1008	0.13635
0.09718	0.06923	-0.04182	-0.05947	0.01837	0.02483	-0.09511	0.16603	-0.21142	-0.04195	0.05834	0.02396	0.19271	0.11958	-0.00101
0.1513	0.1287	-0.0571	-0.04037	0.05392	0.05174	-0.05326	0.15943	-0.10174	0.03543	0.10517	0.03953	0.19734	0.11025	0.14243
0.17389	0.13624	-0.10949	0.01545	0.089	0.07317	-0.17485	0.15523	-0.0397	0.02684	0.15361	0.13266	0.20455	0.04518	0.18377
0.16322	0.12919	-0.10591	0.03425	0.08414	0.09434	-0.15881	0.12909	-0.06212	0.03338	0.14489	0.10157	0.21294	0.05933	0.20625
0.15244	0.10216	-0.05379	0.01674	0.10188	0.05444	-0.16136	0.13464	-0.11212	0.03383	0.14822	0.08626	0.2206	0.09141	0.12792
0.14058	0.09588	-0.03337	-0.06732	-0.04272	0.04323	-0.03003	0.17358	-0.11351	-0.06686	0.04725	0.05192	0.21382	0.09891	0.0506
0.12586	0.07723	-0.00899	-0.06784	0.00951	0.02824	-0.10062	0.17812	-0.07438	-0.08224	0.07799	0.09215	0.211	0.08213	0.0155
0.14335	0.11957	0.04177	-0.09939	-0.06892	0.06207	-7.5E-05	0.18426	-0.14638	-0.09205	0.07812	0.0696	0.19434	0.12745	-0.01283
0.16014	0.10935	-0.09291	-0.0411	0.03585	0.0276	-0.14567	0.18019	-0.12002	-0.04963	0.09144	0.06872	0.20318	0.07841	0.08951
0.12508	0.09429	-0.03508	-0.05606	0.0192	0.05758	-0.10588	0.18207	-0.10593	-0.06118	0.10028	0.11685	0.18861	0.09464	-0.02597
0.12026	0.09495	0.04534	-0.09635	-0.08359	0.02238	-0.06334	0.20315	-0.16495	-0.10067	0.04594	0.05688	0.20692	0.09987	-0.03521
0.19167	0.12128	-0.09345	-0.03139	0.04383	0.04909	-0.10215	0.16133	-0.03115	-0.01121	0.11909	0.10383	0.22602	0.08236	0.12939
0.1441	0.12141	-0.06666	0.01004	0.09401	0.03994	-0.16364	0.14856	-0.15165	-0.02107	0.14032	0.13367	0.224	0.06947	0.19436

HOG_GL	S_HEY_PA	IF1A_PAT	IF1_TFPA	HIF2PATH	V_NEF_PA	NF3A_PA	NF3B_PA	FNG_PAT	GF1_PATH	L12_2PAT	_STAT4_P	IL1_PATH	IL23_PATH	L27_PATH
0.10001	0.03114	0.055	0.18367	0.08808	-0.11028	0.11562	0.21871	0.10865	0.03109	0.04532	0.00396	-0.00314	0.12873	0.1042
0.11214	0.06245	0.06309	0.17096	0.11141	-0.12248	0.11784	0.16425	0.08417	0.0002	-0.01816	-0.10944	-0.04202	0.01975	-0.01209
0.09017	0.0448	0.07432	0.1672	0.10859	-0.07285	0.16998	0.18417	0.11433	0.03045	0.01781	-0.03106	-0.02355	0.09814	0.09073
0.11028	0.01632	0.06461	0.18365	0.10576	-0.09232	0.12888	0.20527	0.1224	0.02716	0.04005	-0.01951	0.02711	0.12489	0.10128
0.09968	0.03323	0.06276	0.14913	0.0982	-0.04698	0.18429	0.21177	0.11241	0.01002	0.11105	0.09019	0.03172	0.12971	0.12267
0.12879	-0.00492	0.07952	0.16582	0.12005	-0.05722	0.19134	0.20842	0.09215	0.02806	0.08107	0.04409	-0.02259	0.11362	0.0719
0.10973	0.06979	0.043	0.1627	0.11353	-0.04237	0.17969	0.22198	0.13324	0.05241	0.0921	0.0474	0.02394	0.16163	0.09837
0.10119	0.02715	0.05487	0.17442	0.1064	-0.08927	0.13312	0.21956	0.14745	0.05824	0.05551	0.00919	0.02334	0.12196	0.13905
0.09417	0.03472	0.0185	0.163	0.09544	-0.05934	0.1761	0.21914	0.15163	0.04438	0.1104	0.05111	0.05852	0.16539	0.21052
0.11133	0.0548	0.04526	0.15246	0.12543	-0.03659	0.17192	0.22531	0.14093	0.03087	0.12157	0.11421	0.00223	0.15258	0.16259
0.0602	0.00784	0.11515	0.19032	0.13037	-0.08571	0.14162	0.22587	0.04863	0.03326	0.01301	-0.03484	0.01298	0.10326	0.02676
0.10283	0.01046	0.06976	0.18184	0.10542	-0.10166	0.17983	0.24315	0.08015	0.01062	0.05472	0.03339	-0.02736	0.12849	0.09993
0.11866	2.7E-06	0.10037	0.16121	0.17233	-0.08395	0.14154	0.21037	0.07209	0.07341	-0.0194	-0.0851	-0.00824	0.02268	0.00875
0.10065	0.01075	0.05411	0.1728	0.10862	-0.09654	0.13624	0.22176	0.09212	0.03475	0.02621	0.01212	0.01498	0.07297	0.0762
0.07772	0.00063	0.02358	0.15987	0.08943	-0.08737	0.15004	0.22964	0.09654	0.0332	0.01723	-0.06971	-0.00133	0.05596	0.05952
0.0995	-0.00573	0.07114	0.17291	0.12871	-0.07926	0.09857	0.24104	0.06414	0.04164	-0.00852	-0.09172	-0.06623	0.02291	0.00307
0.11318	0.02239	0.08713	0.18931	0.1412	-0.11263	0.13894	0.22063	0.10142	0.04586	0.01569	-0.04705	0.01019	0.11924	0.04771
0.08903	0.02027	0.05338	0.1561	0.13182	-0.07419	0.12671	0.18179	0.12988	0.05064	0.0862	0.07317	0.01857	0.15096	0.13355

L2_1PAT	PI3K_P	STAT5_P	IL3_PATH	L4_2PAT	IL5_PATH	L6_7_PAT	CXCR1_P	CXCR2_P	ILK_PATH	GLUCO	SULIN_P	EGRIN1	EGRIN2	EGRIN3
0.05255	0.08244	-0.08331	0.12126	-0.04511	0.05275	0.05804	-0.01008	0.07869	0.15827	0.17265	-0.05027	0.04593	0.17134	0.08365
-0.00038	0.06783	-0.14477	0.09941	-0.07055	0.00556	0.03656	-0.05073	0.04229	0.1568	0.13283	-0.10927	0.05198	0.12964	0.08663
0.04504	0.10031	-0.06292	0.11122	-0.01658	0.06752	0.0645	0.00017	0.0925	0.16262	0.11561	-0.1043	0.05043	0.19066	0.09094
0.05526	0.08666	-0.09749	0.13683	0.01899	0.16606	0.1216	0.10088	0.1768	0.16406	0.16346	-0.07058	0.07791	0.23026	0.13825
0.08308	0.10349	-0.01141	0.14034	0.0107	0.12447	0.10273	0.11286	0.18306	0.16941	0.12436	-0.10956	0.01122	0.20657	0.06001
0.02494	0.09542	-0.02186	0.1031	-0.00858	0.02476	0.06277	-0.005	0.0947	0.14708	0.16245	-0.09544	0.06938	0.18941	0.11398
0.0522	0.12775	-0.03131	0.12351	0.01985	0.05993	0.11395	0.01157	0.10152	0.15726	0.11189	-0.09202	0.15688	0.22125	0.15581
0.07638	0.09752	-0.06178	0.1479	0.04604	0.18151	0.11178	0.1279	0.19567	0.16641	0.14701	-0.07025	0.08463	0.2584	0.1289
0.06922	0.09526	-0.00489	0.14137	0.04668	0.18613	0.12128	0.12955	0.1974	0.17081	0.11997	-0.05322	0.09096	0.26917	0.13011
0.08219	0.0952	-0.0152	0.13484	0.02041	0.1572	0.12906	0.11337	0.18527	0.16205	0.11754	-0.09399	0.04376	0.23624	0.09436
-0.00973	0.08854	-0.10233	0.11269	-0.04834	-0.06112	0.03648	-0.06596	0.0366	0.1472	0.15632	-0.07548	0.02609	0.16862	0.08815
0.02411	0.0818	-0.0703	0.10588	-0.03061	-0.02156	0.07362	-0.03441	0.06743	0.12749	0.18262	-0.09514	0.08023	0.19885	0.10322
0.02289	0.10572	-0.10949	0.12614	-0.08253	-0.11806	0.02466	-0.09891	0.00054	0.16203	0.22091	-0.05845	0.02408	0.06534	0.11944
0.02966	0.08729	-0.06625	0.11208	-0.0159	0.01986	0.10421	0.04281	0.13177	0.15412	0.16009	-0.08244	0.03221	0.19074	0.09643
0.02584	0.07041	-0.12715	0.10204	-0.02068	0.02561	0.08605	-0.02284	0.0708	0.13717	0.14959	-0.08714	0.0237	0.18693	0.0861
-0.00117	0.08303	-0.14295	0.0725	-0.09875	-0.13685	0.00342	-0.10282	-0.01628	0.15476	0.18288	-0.05522	0.03423	0.06363	0.1172
0.04124	0.09755	-0.05725	0.12978	-0.02958	0.11488	0.08474	0.03702	0.13484	0.16257	0.18238	-0.07139	0.07711	0.21993	0.09896
0.06867	0.12842	0.01361	0.14199	0.02203	0.16136	0.10941	0.10059	0.17446	0.18224	0.14327	-0.08003	0.05156	0.22107	0.11178

EGRIN4	EGRIN5	RIN_A4B	RIN_A6B	GRIN_CS	KIT_PATH	LIS1_PAT	KB1_PAT	LPA4_PAT	GIOGEN	DSPHOLI	PK_TRK_P	MET_PATH	TOR_4PAT	_ACTIV_P
0.01109	0.06338	0.17927	0.23759	0.04628	0.05825	0.22794	0.12108	0.19473	0.07997	0.11178	0.13416	0.1002	0.13489	0.24501
-0.01134	-0.01062	0.16764	0.22766	-0.00206	0.00272	0.27316	0.09791	0.20723	0.06608	0.08454	0.13458	0.04264	0.12395	0.27221
-0.06095	0.10211	0.16577	0.22785	0.07027	0.07719	0.22035	0.06096	0.13407	0.09843	0.11395	0.10614	0.08798	0.11619	0.25945
-0.11325	0.10992	0.18734	0.2947	0.05088	0.07993	0.243	0.10313	0.16875	0.1736	0.13556	0.12397	0.0909	0.13671	0.25151
-0.15872	0.04826	0.14251	0.21194	-0.02039	0.09572	0.24808	0.08108	0.2278	0.09304	0.11918	0.14701	0.07633	0.11959	0.24351
-0.05201	0.05325	0.15026	0.25037	-0.04471	0.0406	0.2462	0.09171	0.13746	0.02359	0.09821	0.08033	0.05371	0.10725	0.25244
-0.06969	0.10719	0.21431	0.30789	0.05555	0.07579	0.21221	0.07356	0.13346	0.11107	0.13286	0.10781	0.09231	0.13081	0.25162
-0.09842	0.10701	0.19233	0.30741	0.03728	0.11546	0.24606	0.10219	0.20499	0.16568	0.13411	0.12026	0.08738	0.13657	0.23675
-0.15426	0.12438	0.1905	0.26994	0.02207	0.11019	0.24484	0.08967	0.2078	0.16376	0.12981	0.11265	0.08989	0.11517	0.22552
-0.17185	0.0264	0.18875	0.25964	0.00062	0.1106	0.23585	0.08627	0.20005	0.12323	0.1401	0.13504	0.07186	0.11776	0.23547
0.01554	0.07233	0.1579	0.17676	-0.00303	0.01452	0.27741	0.09627	0.18438	0.06688	0.09968	0.08311	0.07746	0.12286	0.27099
0.05932	0.08622	0.16364	0.2108	0.0417	0.04386	0.2563	0.11751	0.23346	0.08733	0.09793	0.12064	0.06596	0.12242	0.24853
-0.0047	0.15805	0.16113	0.1797	0.02716	0.01155	0.2826	0.13623	0.16405	0.08084	0.07974	0.13924	0.1109	0.15366	0.27686
-0.16497	0.09799	0.17032	0.24139	0.00351	0.04707	0.2592	0.08794	0.17743	0.10608	0.09747	0.13165	0.09023	0.12988	0.2458
-0.08738	0.03652	0.16691	0.21019	-0.04581	0.05949	0.23023	0.10125	0.17303	0.0731	0.09449	0.10916	0.08369	0.13453	0.25946
0.07412	0.08904	0.13965	0.14037	-0.04482	0.01125	0.26801	0.10815	0.10835	0.0533	0.08586	0.12056	0.08175	0.13812	0.27603
0.02099	0.06997	0.18086	0.28328	0.03747	0.05874	0.2546	0.09967	0.17285	0.10852	0.13567	0.13575	0.0931	0.13527	0.25836
-0.08658	0.1331	0.1621	0.26976	0.05248	0.10707	0.2425	0.09342	0.18054	0.14148	0.10522	0.12142	0.09015	0.12209	0.21721

MYC_PAT	REPRESS	DHERIN_	ECTIN_PA	IN_NEPH	TRIN_PA	FAT_3PAT	FAT_TFPA	B_ATYPIC	_CANON	DTCH_PA	TA_DOWN	PHA_BET
0.15784	0.08714	0.20856	0.20961	0.05727	0.17468	0.1505	-0.14774	-0.02313	0.11074	0.05701	0.13352	0.09565
0.15507	0.06226	0.17198	0.18472	0.0222	0.09306	0.14196	-0.16651	-0.03883	0.07562	0.05292	0.10323	0.03205
0.16636	0.09105	0.2069	0.21436	0.07276	0.13574	0.13814	-0.14047	0.02968	0.0575	0.08042	0.12763	0.05474
0.11986	0.06711	0.20919	0.19989	0.05999	0.11099	0.14899	-0.14524	0.04817	0.08268	0.06392	0.11632	0.10034
0.12476	0.07366	0.18379	0.1433	0.03791	0.11955	0.16634	-0.0555	0.06363	0.11199	0.06052	0.14727	0.11166
0.12679	0.05185	0.19263	0.16275	0.03288	0.08876	0.13275	-0.06188	0.00057	0.0538	0.02715	0.1437	0.04101
0.09999	0.07782	0.1851	0.21733	0.0959	0.17473	0.1477	-0.11426	0.07159	0.06936	0.0625	0.1162	0.10676
0.07642	0.07008	0.18325	0.17576	0.04936	0.13417	0.15999	-0.08796	0.06882	0.0981	0.05461	0.10162	0.10753
0.12208	0.07413	0.20695	0.18074	0.06597	0.13656	0.13748	-0.0289	0.08812	0.0887	0.06038	0.12092	0.12628
0.10528	0.07695	0.15391	0.15661	0.02311	0.13284	0.15662	-0.05434	0.10107	0.10744	0.03233	0.13341	0.13168
0.16577	0.07897	0.19584	0.23209	0.05855	0.12488	0.15073	-0.19111	0.01846	0.06992	0.07364	0.12593	0.0252
0.17054	0.08861	0.16751	0.17406	0.06845	0.11719	0.16681	-0.13751	-0.0246	0.06021	0.03609	0.13596	0.04809
0.18498	0.07372	0.19561	0.26226	0.07458	0.11486	0.17527	-0.17646	-0.06997	0.03858	0.09216	0.11476	0.04227
0.11018	0.10354	0.19776	0.20078	0.08863	0.11166	0.13638	-0.08709	0.01581	0.10214	0.05687	0.13086	0.07197
0.13732	0.07459	0.20284	0.19208	0.03815	0.1171	0.14931	-0.17132	-0.02019	0.08337	0.0383	0.12394	0.05024
0.15463	0.10896	0.18062	0.21784	0.06626	0.10841	0.16237	-0.21527	-0.07297	0.04912	0.0755	0.11375	0.04706
0.18516	0.07965	0.20441	0.19716	0.09283	0.13148	0.13031	-0.11885	0.03166	0.04033	0.045	0.1226	0.06913
0.08788	0.06397	0.22366	0.21512	0.05097	0.14748	0.15055	-0.03281	0.08194	0.09391	0.04344	0.14193	0.08902

GULATIO	P73PATH	5_NTR_PA	GFRA_PA	GFRB_PA	CI_AKT_R	3KCI_PA	PLC_TRK	LK1_PAT	LING_EV	PS1_PATH	TP1B_PAT	AC1_PAT	1_REG_P	NBP2_PA	RAS_PATH	RB_1PATH	ELIN_PA	G_GR_PA
0.09351	0.08802	0.07723	-0.00893	0.16812	0.087	0.03	0.12767	0.20385	0.23554	0.10491	0.06486	0.22569	0.02132	0.28961	0.0618	0.0363	0.06197	0.13755
0.12819	0.05597	0.05477	-0.03032	0.12885	0.08905	-0.00312	0.12341	0.17626	0.16799	0.0797	0.01778	0.18583	-0.03389	0.28919	0.02376	0.05455	0.09141	0.14601
0.11648	0.05888	0.04847	0.01819	0.162	0.07609	0.03274	0.10159	0.16749	0.20607	0.13654	0.03125	0.20906	0.01893	0.27078	0.09137	0.0421	0.07358	0.11746
0.08341	0.06553	0.0968	-0.01837	0.17533	0.11116	0.08976	0.1335	0.15873	0.24271	0.10088	0.08987	0.22702	0.07075	0.25007	0.07337	0.0015	0.06999	0.13968
0.09841	0.05951	0.08285	-0.05879	0.15741	0.08597	0.1008	0.12253	0.18187	0.21836	0.07115	0.06361	0.22079	0.08839	0.2694	0.15646	0.04077	0.06146	0.10817
0.09131	0.04276	0.05695	0.0312	0.14502	0.09088	0.03091	0.1015	0.15961	0.17468	0.04513	0.04113	0.18597	0.04477	0.27523	0.06977	0.05075	0.08354	0.1261
0.09239	0.05055	0.08724	0.04124	0.16462	0.0903	0.06664	0.11678	0.19015	0.2121	0.07714	0.05827	0.21595	0.02535	0.29828	0.03085	0.01255	0.07099	0.08779
0.06784	0.04136	0.08331	0.01579	0.17245	0.11078	0.09208	0.12699	0.18442	0.24622	0.0851	0.10361	0.22809	0.09912	0.19611	0.1111	-0.02499	0.08416	0.12299
0.0788	0.04224	0.08819	0.02433	0.16732	0.08883	0.10426	0.11951	0.17952	0.25065	0.07735	0.12486	0.23107	0.10445	0.22874	0.09799	0.00511	0.08252	0.12827
0.08638	0.03021	0.08012	-0.00983	0.16428	0.08617	0.09638	0.09757	0.17714	0.22333	0.06671	0.10207	0.23272	0.11246	0.2482	0.06387	0.01107	0.03688	0.1172
0.12031	0.10355	0.06811	0.03326	0.14034	0.12633	0.0243	0.11326	0.18923	0.23672	0.10192	0.0155	0.19432	-0.08281	0.26493	0.03576	0.052	0.1321	0.14004
0.12328	0.09028	0.07139	-0.02987	0.15658	0.11742	0.01659	0.12133	0.13816	0.226	0.09506	0.01183	0.19568	0.016	0.278	0.02405	0.01405	0.09624	0.14739
0.12138	0.08115	0.07502	0.05953	0.16916	0.13826	-0.01012	0.15002	0.19643	0.25336	0.16163	-0.00581	0.19711	-0.07753	0.32341	0.10766	0.04716	0.08277	0.11651
0.10004	0.03856	0.06578	-0.02093	0.16504	0.09775	0.04576	0.11998	0.18641	0.20691	0.08807	0.05353	0.22819	0.04393	0.26011	0.10206	0.02205	0.06162	0.11759
0.10855	0.10026	0.07649	0.00594	0.15403	0.09674	-0.00368	0.11583	0.20742	0.22847	0.1022	0.03238	0.20524	-0.00326	0.2509	0.06596	0.03157	0.01857	0.11683
0.1418	0.08649	0.04487	-0.00693	0.15737	0.12258	-0.03749	0.1189	0.18653	0.19147	0.14754	-0.00355	0.18973	-0.07975	0.29945	0.0826	0.01173	0.08406	0.12854
0.11206	0.04388	0.09741	-0.00149	0.17177	0.10063	0.07397	0.14351	0.19576	0.23031	0.07969	0.05696	0.2192	0.04214	0.26117	0.10908	0.03978	0.11785	0.14491
0.07777	0.04367	0.07994	0.01599	0.16808	0.09734	0.08207	0.12317	0.18046	0.21942	0.07926	0.09701	0.23102	0.08488	0.24556	0.03086	-0.02187	0.10235	0.13882

OIC_ACID	RET_PATH	HOA_PAT	A_REG_P	OPSIN_R	_VDR_P	META_P	_S1P1_P	_S1P2_P	_S1P3_P	_S1P4_P	HP2_PAT	3NUCLE	AD2_3PA	ECAN_1_	ECAN_2_
0.11892	0.13708	0.21361	-0.02967	0.30914	-0.27019	0.11097	0.12117	0.2612	0.19267	0.25277	0.09136	-0.0105	0.18103	0.03854	0.13853
0.15786	0.10581	0.16926	-0.07995	0.31901	-0.23451	0.11194	0.11644	0.21979	0.1874	0.25523	0.05527	0.02027	0.2073	0.02251	0.15209
0.15163	0.12038	0.19835	-0.01504	0.14568	-0.2891	0.12577	0.14284	0.26085	0.23606	0.25056	0.0798	0.00538	0.24032	0.03236	0.1587
0.13546	0.12672	0.20273	0.00547	0.33327	-0.32449	0.20187	0.144	0.28482	0.25057	0.28258	0.08012	0.017	0.16423	0.06566	0.16477
0.1365	0.11707	0.19956	0.02205	0.28536	-0.30641	0.12641	0.13786	0.26677	0.21357	0.27587	0.08447	0.00686	0.19275	0.01475	0.18762
0.05581	0.07506	0.18251	-0.03677	0.09494	-0.32965	0.1755	0.1098	0.20916	0.17128	0.24214	0.07216	0.04692	0.16898	-0.0591	0.14402
0.14598	0.11325	0.17474	-0.03718	0.48054	-0.33228	0.12498	0.14764	0.26144	0.22648	0.27818	0.08815	0.03519	0.19369	0.09835	0.16799
0.06721	0.13858	0.19755	0.02754	0.25595	-0.30425	0.15632	0.15484	0.27671	0.24225	0.28461	0.06754	0.00772	0.16393	0.04595	0.20465
0.11852	0.13461	0.19015	0.02882	0.19588	-0.2977	0.14355	0.17099	0.25854	0.22938	0.27564	0.05857	0.01061	0.1477	0.07614	0.21037
0.07445	0.11256	0.20225	0.03785	0.3109	-0.27047	0.16069	0.13626	0.27573	0.22841	0.27455	0.06694	0.01124	0.16047	0.02367	0.18935
0.14023	0.13493	0.21041	-0.0987	0.16981	-0.33054	0.16093	0.10974	0.24026	0.20072	0.2242	0.06062	-0.00871	0.20653	-0.01846	0.11509
0.11854	0.09144	0.2049	-0.06411	0.13458	-0.32688	0.16844	0.12572	0.28174	0.25379	0.26529	0.05129	-0.01237	0.19689	0.02222	0.16254
0.11913	0.1394	0.20189	-0.08752	0.3213	-0.32019	0.14162	0.15795	0.24871	0.25443	0.2692	0.08832	0.00968	0.24752	0.00333	0.14334
0.10528	0.11928	0.19033	-0.03385	0.15871	-0.33383	0.12473	0.14515	0.24216	0.2371	0.26067	0.05911	0.00836	0.17273	0.03813	0.15589
0.13422	0.13163	0.19831	-0.05156	0.18196	-0.33629	0.10527	0.13974	0.26457	0.23186	0.26175	0.05331	-0.0176	0.20048	0.01966	0.15543
0.14058	0.10529	0.17476	-0.08827	0.18742	-0.29637	0.12853	0.11737	0.2527	0.21074	0.2529	0.06953	-0.00894	0.22558	0.08321	0.11745
0.11096	0.14222	0.20566	-0.026	0.32376	-0.33713	0.18969	0.13724	0.27379	0.23954	0.27953	0.09182	0.00251	0.16247	0.04036	0.16081
0.12666	0.13537	0.18977	0.01605	0.29558	-0.31879	0.11385	0.14576	0.24691	0.22272	0.2766	0.0832	0.00255	0.16681	0.0148	0.1681

ECAN_3_	ECAN_4_	AP63_PAT	PTP_PAT	CALCIUM	R_JNK_PA	CR_PATH	R_RAS_PA	MERASE_	GFBR_PA	BIN_PAR	BIN_PAR	TNF_PATH	DOGENO	RAIL_PAT	RKR_PAT
0.17574	0.20001	0.06639	0.06995	-0.00218	-0.01023	0.02969	0.16582	0.08796	0.05147	0.17218	0.15138	0.00643	0.24929	-0.04518	0.12256
0.15322	0.19908	0.06431	0.05014	-0.01777	-0.03363	-0.00513	0.11108	0.10513	0.05656	0.13656	0.11929	-0.02408	0.27405	-0.07522	0.0936
0.14233	0.18205	0.0034	0.07113	-0.04839	0.02176	0.02155	0.14687	0.08851	0.07294	0.18503	0.16018	-0.03608	0.2498	-0.02143	0.13009
0.14917	0.21828	0.05512	0.11495	-0.00375	0.02572	0.05982	0.15744	0.0729	0.05191	0.24355	0.2439	-0.00243	0.28112	0.02533	0.14971
0.14266	0.16702	-0.00464	0.09201	0.05269	0.07662	0.11723	0.19663	0.07316	0.06213	0.21792	0.22381	0.02125	0.2702	0.0076	0.14399
0.10611	0.1782	-0.01002	0.07821	-0.03996	0.07061	0.07744	0.06291	0.1023	0.05053	0.15724	0.16756	0.00822	0.24286	-0.08379	0.10185
0.13505	0.24252	-0.00897	0.10114	-0.01772	0.08425	0.07621	0.1416	0.10948	0.03371	0.19912	0.17557	0.01604	0.26727	-0.0016	0.1379
0.12062	0.21906	-0.00831	0.10715	0.02497	0.09852	0.07744	0.15665	0.05472	0.0604	0.24008	0.23769	0.00049	0.30402	0.02235	0.1473
0.122	0.20508	-0.01551	0.11858	0.05632	0.08249	0.08484	0.11838	0.05711	0.06813	0.23816	0.22527	-0.00392	0.28823	0.02627	0.13998
0.09682	0.21245	-0.02604	0.11383	0.0118	0.10922	0.12709	0.1536	0.06281	0.03555	0.22821	0.23224	0.017	0.29421	0.00941	0.12057
0.19238	0.19321	0.05445	0.07438	-0.03778	0.06229	-0.00828	0.08657	0.09692	0.06388	0.17768	0.17417	-0.05031	0.17418	-0.07648	0.13585
0.14925	0.19396	0.09491	0.07144	-0.05122	0.01657	0.04163	0.10523	0.07696	0.02465	0.17608	0.20453	-0.01568	0.25233	-0.04731	0.10371
0.14789	0.20431	0.03299	0.07104	-0.05159	-0.032	-0.04489	0.16332	0.11269	0.10712	0.14548	0.1672	-0.01566	0.18355	-0.05024	0.12471
0.1312	0.20669	-0.01035	0.0665	-0.00074	0.07067	0.0671	0.13798	0.07455	0.05223	0.19536	0.19967	-0.01425	0.27888	-0.01158	0.14099
0.14601	0.19584	0.03252	0.03377	-0.04039	0.04306	0.01622	0.12973	0.09221	0.07468	0.17059	0.19995	0.00571	0.25696	-0.04416	0.129
0.20251	0.18794	0.00466	0.0583	-0.07413	0.00542	-0.04789	0.14036	0.12448	0.07402	0.14784	0.13198	-0.03911	0.1694	-0.08441	0.09768
0.14781	0.22328	0.05099	0.08464	-0.03642	0.02639	0.04019	0.16567	0.08133	0.04622	0.22625	0.23764	-0.03846	0.26689	0.00173	0.14916
0.1208	0.17128	-0.01177	0.11104	0.02373	0.07496	0.09069	0.15552	0.08402	0.05701	0.22063	0.19278	-0.01895	0.26918	0.01534	0.14943

TXA2PATH	_UPAR_P	FR1_2_P	GFR1_PA	_VEGFR_	ANONICA	CANONI	IGNALIN	TR_CIRC	IE_ABACA	PROTEIN	ORTERS_	RANSPO	MITOTIC_	C_G1_S_T	_HIV_1_
0.09475	0.202	0.15212	0.06707	-0.13458	0.01964	0.0214	-0.13443	0.37653	0.05028	0.23275	0.05957	0.23983	-0.21046	-0.30461	-0.05219
0.05705	0.17745	0.10798	0.0345	0.02079	-0.05388	-0.00368	-0.24334	0.38922	-0.20205	0.22003	0.01158	0.23623	-0.1344	-0.19257	0.05871
0.10504	0.23818	0.14251	0.08628	-0.00018	-0.01259	0.06092	-0.20009	0.38186	-0.15501	0.2144	0.08771	0.22165	-0.22134	-0.1998	-0.03602
0.17803	0.26291	0.14566	0.06167	-0.31038	-0.03336	0.04629	-0.24001	0.31282	-0.09763	0.22468	0.11657	0.23559	-0.18022	-0.30642	-0.08763
0.17434	0.23317	0.14979	0.05427	-0.19306	-0.0703	0.05482	-0.23649	0.33886	-0.04911	0.24442	0.06111	0.25957	-0.25575	-0.1814	-0.04366
0.0914	0.16731	0.13765	0.05061	-0.30351	-9.9E-06	0.0167	-0.18279	0.36325	-0.04539	0.23825	0.10934	0.24055	-0.16709	-0.14133	-0.04028
0.13328	0.22855	0.15458	0.07522	0.02519	-0.03995	0.05695	-0.17469	0.35773	-0.02368	0.22189	0.11334	0.22854	-0.21954	-0.11482	-0.05199
0.17821	0.26615	0.14817	0.08582	-0.1765	-0.04747	0.06633	-0.25317	0.28105	-0.10439	0.24241	0.10951	0.25635	-0.19165	-0.25176	-0.11457
0.18914	0.25704	0.16803	0.10641	-0.19183	-0.02035	0.06029	-0.24851	0.31174	-0.05695	0.24845	0.13823	0.25569	-0.23415	-0.29897	-0.09193
0.19022	0.24331	0.1595	0.09592	0.03364	-0.06634	0.07133	-0.20622	0.32832	-0.12981	0.22707	0.07821	0.23744	-0.25378	-0.16685	-0.06307
0.08238	0.22389	0.11434	0.05652	0.10675	-0.07517	0.04042	-0.23086	0.35852	0.00271	0.22484	0.09625	0.22902	-0.14931	-0.05388	-0.07384
0.11933	0.21176	0.12884	0.05073	-0.10146	-0.10919	0.06206	-0.2503	0.36253	-0.00251	0.23559	0.09791	0.23896	-0.22884	-0.28406	-0.08939
0.04321	0.16166	0.14201	0.03432	-0.20592	0.01117	0.08677	-0.10927	0.38564	-0.2326	0.22821	0.06343	0.23826	-0.15257	-0.13787	-0.01818
0.12954	0.2381	0.13373	0.03145	-0.18619	-0.04144	0.05286	-0.27339	0.35241	0.05549	0.22708	0.0986	0.24175	-0.21813	-0.25756	-0.06392
0.09341	0.21849	0.14897	0.04001	-0.35529	-0.00413	0.03781	-0.35153	0.34664	-0.0315	0.23809	0.05633	0.24357	-0.21353	-0.19897	-0.04283
0.0386	0.13711	0.10639	0.01714	-0.23604	-0.0153	0.06956	-0.18414	0.33825	-0.1453	0.21928	0.04248	0.22938	-0.16322	-0.26913	-0.0308
0.1475	0.2513	0.12509	0.04861	-0.25137	-0.01583	0.04286	-0.31234	0.3607	-0.12436	0.21897	0.03407	0.22781	-0.18522	-0.3325	-0.06541
0.15134	0.22234	0.14811	0.0731	-0.24309	-0.03746	0.0916	-0.2016	0.34615	-0.15858	0.24167	0.12196	0.25458	-0.24625	-0.26942	-0.07829

NEUROT	RE_REGUL	AND_SP	B_TRANS	TRK2_SI	_SIGNAL	NTRK2_S	NTRK2_S	NTRK2_S	NTRK3_S	NTRK3_S	OF_AR_A	1_MEDIA	_AMPK_D	HINDBR	IN_RES
0.13771	0.12168	0.33874	0.08564	0.16899	0.10011	0.30932	0.16271	0.08734	0.03916	0.08734	0.43836	0.11648	0.15769	0.25456	0.04151
0.12457	0.09744	0.39792	0.02349	0.35823	0.10872	0.30534	0.11783	0.09229	-0.01816	0.09229	0.44145	0.08333	0.20309	0.2726	-0.00501
0.14168	0.1635	0.31707	0.10091	0.16994	0.09276	0.32836	0.24305	0.08125	0.13985	0.08125	0.44561	0.08495	0.19199	0.26244	0.09976
0.15386	0.21808	0.20944	0.16802	0.21398	0.08769	0.38528	0.35976	0.06901	0.28546	0.06901	0.44222	0.09775	0.21392	0.24395	0.06141
0.13243	0.12999	0.25947	0.10665	0.17975	0.1392	0.35909	0.21929	0.12072	0.12593	0.12072	0.43433	0.1447	0.20789	0.25425	0.03961
0.07561	0.02354	0.32464	-0.00756	0.34959	0.05547	0.30415	0.14638	0.02356	0.02619	0.02356	0.43549	0.07581	0.19914	0.25305	0.08762
0.03521	0.09181	0.38841	0.09491	0.18086	0.03563	0.34636	0.32761	0.03638	0.24427	0.03638	0.4485	0.09637	0.18066	0.26866	0.04386
0.08897	0.19853	-0.01832	0.17003	0.20795	0.07752	0.39077	0.34172	0.03627	0.26792	0.03627	0.43593	0.11638	0.21928	0.23266	-0.06053
0.09882	0.1668	0.11943	0.15088	0.19809	0.03472	0.39802	0.35829	0.02655	0.2986	0.02655	0.4409	0.12391	0.22867	0.23767	-0.04686
0.0787	0.16905	0.17773	0.09915	0.19551	0.0679	0.38975	0.2439	0.03491	0.16429	0.03491	0.43974	0.13975	0.22239	0.25322	-0.05783
0.09284	0.05178	0.34842	0.02058	0.26562	-0.01301	0.29279	0.24143	-0.00392	0.18796	-0.00392	0.44782	0.06691	0.2301	0.26407	0.14726
0.0386	0.12265	0.15307	0.03468	0.37494	-0.00182	0.29116	0.14695	-0.02175	0.00873	-0.02175	0.44483	0.11657	0.19462	0.25349	0.0991
0.16278	0.02905	0.44196	0.05208	0.22471	0.12061	0.2525	0.09984	0.19184	-0.05367	0.19184	0.4345	0.07356	0.26361	0.26333	0.13771
0.06871	0.10922	0.36163	0.10909	0.29293	0.08703	0.34097	0.16863	0.10152	0.04217	0.10152	0.4469	0.11262	0.2203	0.25042	-0.09806
0.07389	0.05183	0.25894	0.09057	0.23079	0.0492	0.31406	0.13493	0.04267	0.01114	0.04267	0.4365	0.09606	0.21663	0.249	0.07303
0.10208	0.05275	0.41835	0.04048	0.34841	0.05432	0.26988	0.04086	0.05522	-0.10555	0.05522	0.4385	0.08303	0.21657	0.24415	0.11215
0.06105	0.16727	0.34596	0.08848	0.32407	0.11595	0.35796	0.35769	0.11059	0.2696	0.11059	0.44284	0.06714	0.24142	0.25646	-0.16571
0.11277	0.13462	0.25647	0.11336	0.20659	0.07759	0.37331	0.38565	0.05681	0.30346	0.05681	0.43525	0.11109	0.24108	0.2452	-0.04254

AND_TRA	ON_OF_B	IVATION_	_THROU	GENE_EX	RF3_IRF7	E_RECEP	F_MATRI	_NIMA_K	RECEPT	AND_TR	GC1A_PG	AND_TR	ACTIVATIO	_RAC1_	ATION_O
0.172	0.11118	0.11821	0.03886	-0.09559	0.12686	0.1347	0.09471	0.02042	0.12404	-0.42787	-0.04078	-0.12026	0.11602	0.10684	0.13759
0.10303	0.05201	0.01575	0.05513	-0.11207	0.07042	0.12894	-0.0196	0.03886	0.15016	-0.41466	0.00583	-0.12236	0.04778	0.11985	0.15847
0.10987	0.04896	0.10852	0.09692	-0.10353	0.07587	0.10817	0.0485	0.12074	0.14127	-0.45834	-0.01827	-0.1337	0.09173	0.19725	0.11959
0.1773	0.1138	0.23167	0.08606	-0.15901	0.07082	0.19306	0.11966	0.08614	0.14134	-0.45129	-0.01237	-0.0986	0.04343	0.172	0.12068
0.15576	0.08466	0.19312	0.06783	-0.11068	0.06954	0.12354	0.17071	0.10272	0.1434	-0.42558	0.05004	-0.32601	0.08575	0.19277	0.22509
0.13634	0.06368	0.30634	0.04836	-0.1196	0.04317	0.07499	0.1144	0.03583	0.11441	-0.31953	0.10062	-0.09894	0.08618	0.11478	0.03581
0.1167	0.0469	0.20689	0.13654	-0.10926	0.04281	0.09545	0.18613	0.09607	0.11791	-0.37326	-0.01331	-0.16939	0.06882	0.13416	0.06529
0.19346	0.11576	0.27055	0.05669	-0.1417	0.12405	0.15851	0.18413	0.01174	0.15046	-0.42311	0.00678	-0.1691	0.07156	0.21624	0.05843
0.15467	0.08328	0.22505	0.13229	-0.13574	0.08414	0.15093	0.16192	0.04511	0.14641	-0.42135	0.03847	-0.1493	0.09331	0.21857	0.00651
0.14072	0.08734	0.25517	0.15191	-0.13091	0.10042	0.14521	0.16352	0.04057	0.14397	-0.40479	0.07612	0.0485	0.07448	0.17285	0.04008
0.16004	0.10713	0.16584	0.10026	-0.10776	0.04627	0.08192	0.14933	0.03094	0.12206	-0.4483	-0.04205	-0.13032	0.06823	0.10864	0.04695
0.17906	0.11737	0.27458	0.00033	-0.06656	0.047	0.10806	0.13815	0.10365	0.1336	-0.45376	0.01992	-0.06551	0.03179	0.13256	-0.00849
0.17067	0.12323	0.12595	0.04158	-0.01448	0.01233	0.1381	-0.04804	0.06496	0.17843	-0.19746	0.01548	-0.01011	0.09039	0.10872	0.2342
0.14441	0.08608	0.20206	0.04126	-0.13507	0.04442	0.10888	0.09779	-0.07043	0.14592	-0.40109	-0.05543	-0.12348	0.07696	0.15816	0.17599
0.17288	0.10514	0.18592	0.08346	-0.06296	0.10209	0.08793	0.17707	0.02286	0.14416	-0.36533	0.04857	-0.09841	0.08271	0.13647	0.02685
0.19574	0.12794	-0.03497	0.0684	-0.07253	0.0505	0.0925	-0.03626	0.05821	0.1275	-0.26387	-0.047	-0.05	0.12392	0.07911	0.14272
0.15322	0.10696	0.17011	0.05469	-0.1304	0.06176	0.17518	0.0912	-0.02023	0.14767	-0.45816	0.02183	-0.0905	0.04463	0.12774	0.2187
0.18561	0.10504	0.17427	0.05975	-0.19586	0.09167	0.15666	0.00707	0.04477	0.15188	-0.43333	-0.02053	-0.33283	0.05747	0.20973	0.10939

ACTIVATIO	P_1_FAM	CAP_BIN	THE_PHO	F_THE_PR	CHAIN_RE	REMODE	HAIN_RE	HAIN_RE	HAIN_RE	HAIN_RE	HAIN_RE	PTIVE_IM	_CYCL	ASSIS_JUNC	TI_INFLA
-0.03266	0.16053	0.36737	0.30914	0.12204	0.27286	0.00898	0.04135	0.01717	-0.00394	0.17056	-0.00784	0.11816	0.3139	0.17448	0.2212
-0.06435	0.09755	0.38877	0.31901	0.04289	0.24141	-0.33116	-0.03409	-0.08597	-0.13624	-0.00614	-0.06924	0.10977	0.31314	0.16941	0.2328
0.04942	0.13474	0.36874	0.14568	0.16241	0.29477	-0.26687	0.05468	0.08695	0.00893	0.20316	0.06215	0.12533	0.31244	0.17935	0.17912
0.08595	0.13593	0.38173	0.33327	0.11959	0.24824	-0.1499	0.13853	0.12581	-0.0347	0.25246	0.06342	0.15945	0.35856	0.11997	0.2739
0.11159	0.15183	0.37809	0.28536	0.10959	0.30124	-0.25823	0.09206	0.09308	-0.02498	0.19492	0.06621	0.1547	0.34337	0.05137	0.25289
-0.01157	0.03239	0.38864	0.09494	0.16513	0.34252	-0.30897	0.01772	0.034	0.04864	0.20143	0.04888	0.12169	0.35409	0.12984	0.20571
0.06082	0.13324	0.37586	0.48054	0.12196	0.32931	-0.22089	0.02088	0.02104	0.00239	0.16388	-0.0189	0.12995	0.33505	0.11129	0.21915
0.1391	0.14959	0.37936	0.25595	0.00211	0.17032	-0.19147	0.05113	-0.00168	-0.18677	0.09855	-0.05152	0.16997	0.34864	0.06067	0.26565
0.12025	0.13305	0.37409	0.19588	0.02327	0.18039	-0.33926	0.0674	0.02245	-0.16612	0.08838	-0.02891	0.17227	0.33145	0.08493	0.2505
0.12785	0.15117	0.36636	0.3109	0.00216	0.26915	-0.28149	0.08966	0.07115	-0.03578	0.21533	0.02206	0.16353	0.32954	0.07373	0.27283
-0.0515	0.10241	0.37119	0.16981	0.21926	0.31535	-0.29752	0.09319	0.12078	0.01958	0.21995	0.09298	0.1118	0.32709	0.19149	0.18913
0.04463	0.16583	0.38569	0.13458	0.15165	0.33529	-0.30914	-0.02347	-0.01851	0.03402	0.15405	0.00161	0.12131	0.32416	0.12557	0.23444
-0.04496	0.11745	0.3748	0.3213	0.18497	0.34606	-0.10199	-0.06445	0.00279	-0.0464	0.17949	0.01485	0.10478	0.31058	0.19566	0.23556
0.12867	0.10508	0.37959	0.15871	-0.03741	0.27731	-0.26123	0.1409	0.13577	-0.01838	0.21861	0.08207	0.14422	0.31199	0.14885	0.21973
0.03187	0.1657	0.38564	0.18196	0.13536	0.24282	-0.29623	0.04923	0.06667	-0.11571	0.17068	-0.0053	0.12482	0.28591	0.15126	0.20968
0.02657	0.12891	0.38215	0.18742	0.17995	0.32408	-0.0576	-0.19851	-0.14225	-0.0988	0.00417	-0.10889	0.09748	0.29792	0.18668	0.16368
0.0534	0.1219	0.39091	0.32376	-0.11829	0.30515	-0.2031	0.08599	0.11647	-0.05246	0.23864	0.07286	0.13296	0.3591	0.13123	0.2827
0.09826	0.14363	0.37231	0.29558	0.02695	0.29775	-0.19165	0.1142	0.09488	0.02251	0.20514	0.08067	0.16237	0.32758	0.12392	0.26122

G_THRO	G_THROU	RENALI	NTION_EN	ACTIVATIO	ME_AGGR	RYLATES_	YLATES_1	K_MUTAN	_ALPHA_	AS_AND_L	_OXIDATI	IN_KINA	SIVE_COM	ACIDS_R	PORT_A
0.20111	0.2226	0.20915	0.2437	0.19666	0.25193	-0.21885	-0.06817	0.2649	0.20035	0.12122	-0.12296	0.05837	0.11119	0.13261	-0.18161
0.17737	0.22816	0.21903	0.24648	0.04597	0.27731	-0.18674	-0.02399	0.23913	0.18859	0.10982	-0.0904	-0.00548	0.04554	0.09501	-0.12883
0.19612	0.19268	0.18069	0.24399	0.07124	0.25107	-0.13315	0.0039	0.28085	0.1011	0.06796	-0.07841	0.03459	0.07985	0.14667	0.03935
0.29784	0.32706	0.32813	0.22538	0.17799	0.27157	-0.12465	0.00443	0.24045	0.11678	0.16769	-0.09724	0.03218	0.14621	0.18198	-0.01706
0.23323	0.26288	0.26304	0.25245	0.11447	0.25867	-0.16973	0.00704	0.2802	0.1323	0.10912	-0.10093	0.03313	0.15375	0.127	-0.15848
0.17167	0.20035	0.18317	0.1978	0.09175	0.23395	-0.16465	-0.02139	0.26014	0.11165	0.12194	-0.19986	-0.04818	0.25854	0.07323	-0.05332
0.21344	0.22778	0.22256	0.27739	0.14102	0.23633	-0.12449	-0.03345	0.2463	0.22897	0.09168	-0.12319	0.02318	0.28758	0.10953	0.04952
0.23515	0.28882	0.28809	0.25949	0.18371	0.2736	-0.16044	-0.00854	0.23309	0.12986	0.13857	-0.09502	0.05716	0.22129	0.18871	-0.00932
0.21589	0.27035	0.27179	0.23164	0.13428	0.26925	-0.15883	0.0114	0.25003	0.16051	0.12334	-0.09267	0.05026	0.18096	0.18486	-0.01301
0.27196	0.29444	0.29777	0.26057	0.09579	0.26293	-0.11271	-0.00981	0.27079	0.17274	0.08345	-0.14194	0.04822	0.19553	0.15437	-0.04309
0.18268	0.19079	0.17317	0.29386	0.24245	0.28453	-0.16668	-0.03423	0.24642	0.23943	0.08868	-0.11395	0.06204	0.13656	0.11635	-0.04136
0.23889	0.22667	0.21597	0.27079	0.22251	0.26545	-0.19362	0.01023	0.25399	0.15452	0.0819	-0.11957	0.0635	0.15457	0.07083	-0.02798
0.2112	0.24093	0.24201	0.26483	0.18757	0.31595	-0.06073	-0.009	0.24522	0.08275	0.10541	-0.08978	0.04264	0.10335	0.15653	-0.04752
0.19231	0.21409	0.20669	0.27456	0.23492	0.2793	-0.19865	-0.00398	0.24512	0.16945	0.19753	-0.07304	0.03545	0.1544	0.17149	-0.06274
0.18285	0.19796	0.19173	0.26963	0.28003	0.26366	-0.2178	-0.09505	0.25687	0.26618	0.09245	-0.12823	0.06053	0.20881	0.12958	-0.04665
0.13831	0.15376	0.13034	0.2571	0.18455	0.28187	-0.08907	0.02516	0.24974	0.08405	0.15139	0.02237	0.11329	0.08878	0.09137	-0.00439
0.29262	0.32497	0.32576	0.26672	0.17217	0.2848	-0.14249	0.03163	0.2272	0.10973	0.11143	-0.0967	-0.00418	0.08977	0.15453	-0.10637
0.2535	0.29302	0.2966	0.25445	0.13143	0.29183	-0.14892	0.03658	0.26125	0.15258	0.13253	-0.1036	0.03594	0.12195	0.16411	-0.03571

SP_TRANS	LOID_FIB	ORING_F	BASAL_B	ROGEN_	_BCR_LI	ASSEMB	CESSING	UBIQUIT	TIMICRO	HANISM_	ESE_FAVO	EDIATED_	EDIATED_	ID_OTHE
-0.21269	0.3192	-0.20972	0.16233	-0.11448	0.09023	0.25362	0.2513	0.09802	0.23156	0.16447	0.15487	-0.06548	-0.08301	0.21983
0.02427	0.30255	-0.26299	0.17721	-0.30476	0.03504	0.22996	0.25133	0.10911	-0.00469	0.19014	0.14763	-0.00073	-0.01849	0.2278
0.00394	0.32502	-0.23488	0.15661	-0.33869	0.08783	0.24006	0.25565	0.09733	0.10792	0.20229	0.12943	-0.07752	-0.08438	0.19752
-0.11755	0.34453	-0.1018	0.16332	-0.1936	0.15936	0.26013	0.28113	0.09752	0.18372	0.18027	0.22491	-0.04452	-0.07014	0.20996
0.03056	0.33946	-0.22379	0.17468	0.00645	0.14988	0.24743	0.28817	0.11541	0.2218	0.17818	0.18162	-0.10852	-0.12941	0.22991
0.06092	0.33937	-0.18806	0.17197	-0.21383	0.08281	0.21976	0.25058	0.11748	0.09384	0.18489	0.11486	-0.03793	-0.05822	0.22231
-0.19683	0.35873	-0.17588	0.13364	-0.31561	0.11396	0.24035	0.25393	0.08815	0.11544	0.18615	0.14966	-0.07133	-0.08412	0.20401
-0.10533	0.33997	-0.11689	0.17883	-0.16132	0.16127	0.27608	0.29657	0.11037	0.22698	0.16367	0.21376	-0.05563	-0.10361	0.2285
-0.06234	0.35344	-0.0676	0.18606	-0.15613	0.18178	0.2815	0.29854	0.11009	0.1837	0.17649	0.19845	-0.08868	-0.12411	0.22677
0.00544	0.3466	-0.22885	0.16075	-0.11575	0.1697	0.28265	0.28711	0.10132	0.24109	0.18655	0.222	-0.09992	-0.1495	0.21644
-0.14402	0.34322	-0.24998	0.17673	0.05497	0.0546	0.18892	0.23306	0.11619	0.25264	0.17467	0.08474	-0.02187	-0.0316	0.20992
0.00082	0.34858	-0.10184	0.16463	-0.13571	0.07642	0.24516	0.25421	0.10522	0.15917	0.19752	0.12713	-0.08179	-0.09378	0.21499
0.00104	0.31209	-0.39996	0.2015	-0.20597	-0.03015	0.163	0.21878	0.12883	-0.03569	0.19813	0.07432	-0.02489	-0.02703	0.2195
-0.09663	0.33801	-0.25675	0.16393	-0.16889	0.10321	0.2417	0.26295	0.10207	0.24561	0.19019	0.1597	-0.0623	-0.12046	0.21391
-0.00621	0.33106	-0.2519	0.16588	-0.07957	0.06216	0.24029	0.25125	0.10275	0.27911	0.1804	0.14662	-0.06667	-0.10227	0.21511
0.01603	0.30721	-0.20701	0.17857	-0.17092	-0.05313	0.23137	0.21433	0.11422	-0.01309	0.19598	0.04522	-0.03515	-0.02884	0.2062
0.09978	0.33361	-0.17897	0.17181	-0.12957	0.1231	0.20417	0.24675	0.09031	0.18049	0.17018	0.19138	-0.04468	-0.08177	0.20901
-0.04453	0.33087	-0.17859	0.17067	-0.14979	0.16414	0.25533	0.28993	0.10225	0.12741	0.18397	0.19468	-0.09299	-0.12417	0.22523

EGRADATI	_SITES_V	ED_RESI	OME_APC
0.21001	-0.21455	0.27351	0.23219
0.22366	0.07016	0.27359	0.2192
0.1941	-0.13291	0.27442	0.21994
0.20042	-0.11792	0.21192	0.21161
0.22069	-0.12222	0.26762	0.22449
0.21605	-0.03546	0.29122	0.20989
0.2004	-0.13303	0.26419	0.20868
0.21402	-0.19053	0.19792	0.21707
0.21321	-0.18879	0.22907	0.21572
0.20188	-0.17565	0.26822	0.2141
0.21311	-0.1101	0.26142	0.2207
0.20699	-0.16785	0.27923	0.23138
0.22034	-0.06607	0.29173	0.23273
0.20379	-0.07942	0.26184	0.2164
0.20791	-0.2024	0.24138	0.22183
0.20288	-0.06795	0.23082	0.2185
0.19874	-0.08731	0.25758	0.22024
0.21127	-0.16081	0.26095	0.22967

_FACTOR	ORIN_MED	NATE_PR	IDONIC_	IS_MEDIA	CARBON	NE_N_LIN	AND_AS	ME_ASPI	RFACE_P	IVE_LPL_	FIBRILS	MBLY_OF_	_COMPLE	WITH_TA	LOCALIZ	ESPONSE	ALPHA_A	CHA_ACTIV	ATORVAS	TACHMEN	NT_OF_G	ATTENUA	O_BINDS	KA_ACTI
0.11848	0.18268	-0.03645	0.04738	0.19986	0.20411	0.14747	0.26188	0.02829	0.17885	-0.41727	-0.05323	0.12936	0.4501	0.23796	0.30351	-0.09081	0.24163	0.24163	-0.23649	0.09476	-0.12895	0.31632	0.32577	0.17608
0.12268	0.19569	-0.02366	0.06293	0.19856	0.1103	0.14405	0.16946	0.01565	0.20977	-0.35793	-0.04353	0.01827	0.45477	0.2481	0.30399	-0.03397	0.2805	0.2805	-0.41808	0.15573	-0.06508	0.3384	0.32919	0.17873
0.15715	0.13585	0.02391	0.04733	0.16826	0.12348	0.14449	0.30558	0.04706	0.18557	-0.33903	-0.01919	0.08097	0.45278	0.24697	0.28276	-0.09134	0.27629	0.27629	-0.36171	0.02523	-0.10674	0.30768	0.30894	0.15623
0.10632	0.25208	0.07612	0.06007	0.20874	0.14894	0.15719	0.13189	0.1134	0.18424	-0.22209	-0.03514	0.10766	0.45142	0.22574	0.28464	-0.11357	0.2442	0.2442	-0.02474	0.09882	-0.04926	0.2684	0.31452	0.1492
0.11089	0.21573	-0.01649	0.0327	0.17467	0.16417	0.14807	0.19623	0.01209	0.14497	-0.31452	-0.06434	0.07255	0.45073	0.23758	0.31756	-0.0862	0.25455	0.25455	-0.42715	0.10135	-0.06311	0.26935	0.3438	0.16562
0.03266	0.16455	-0.03668	0.06	0.18351	0.16383	0.1365	0.27373	0.10363	0.14696	0.00453	-0.02841	-0.01905	0.45851	0.23515	0.30387	-0.1041	0.22149	0.22149	0.08366	0.00078	-0.03891	0.2981	0.32641	0.16442
0.11193	0.18468	-0.13807	0.07057	0.18524	0.14492	0.12668	0.27386	0.03514	0.15988	-0.20769	0.06833	-0.02183	0.45919	0.22854	0.28829	-0.05899	0.22369	0.22369	-0.4061	0.10985	-0.20984	0.29685	0.31193	0.14281
0.11643	0.24269	0.1401	0.02579	0.24434	0.13622	0.17637	0.13056	0.11677	0.1795	-0.07227	-0.02293	0.08872	0.44588	0.22409	0.30631	-0.13721	0.21061	0.21061	0.00989	-0.03698	-0.04636	0.27007	0.33312	0.18533
0.13369	0.21659	0.05335	0.02958	0.19369	0.14626	0.16402	0.13397	0.09491	0.18336	-0.0657	0.03902	0.06534	0.44804	0.23121	0.30873	-0.12699	0.22082	0.22082	0.00369	0.11521	0.02203	0.26164	0.33682	0.1929
0.12944	0.243	0.0312	0.00691	0.19494	0.16553	0.14687	0.14491	0.1037	0.16731	-0.04656	-0.06844	0.00539	0.44904	0.21939	0.30029	-0.09571	0.26048	0.26048	0.00561	0.03763	-0.05522	0.26909	0.32684	0.17069
0.13857	0.15832	-0.12124	0.01668	0.24652	0.17406	0.14785	0.31272	0.05283	0.20136	-0.23712	-0.0783	0.00513	0.46065	0.25972	0.28811	-0.0875	0.27919	0.27919	-0.23895	0.02118	-0.05269	0.33104	0.31369	0.17027
0.11928	0.19401	-0.09551	0.00232	0.1649	0.16798	0.15327	0.32102	0.09817	0.1799	-0.16718	0.00782	0.03025	0.45989	0.2512	0.30057	-0.10885	0.22256	0.22256	-0.04541	0.01433	-0.17149	0.29156	0.3245	0.15404
0.13033	0.23329	-0.17747	0.00481	0.21039	0.15837	0.14847	0.25401	0.00894	0.25994	-0.08048	-0.06407	0.08098	0.45272	0.26065	0.29964	-0.0999	0.24686	0.24686	-0.09625	0.09903	-0.21837	0.32871	0.32244	0.19462
0.09875	0.1929	0.03171	0.07559	0.2007	0.1503	0.16767	0.21409	0.03146	0.20948	-0.23702	-0.0555	0.04191	0.45617	0.24401	0.29233	-0.08314	0.25786	0.25786	-0.23838	0.08803	-0.18933	0.30987	0.31938	0.17043
0.12678	0.17935	-0.11174	0.06174	0.21289	0.15394	0.15706	0.26044	0.03678	0.18682	-0.18527	-0.08993	0.03327	0.45248	0.24197	0.29687	-0.0193	0.27099	0.27099	-0.09252	0.01593	-0.14917	0.30256	0.32152	0.17476
0.12055	0.12632	-0.18821	0.00626	0.21611	0.16708	0.1315	0.28734	0.02571	0.21566	-0.38852	-0.05781	0.07803	0.45353	0.27352	0.28816	-0.05654	0.25871	0.25871	-0.42184	0.18731	-0.18138	0.30313	0.30937	0.17417
0.11102	0.25196	0.00994	0.0413	0.19658	0.16216	0.14601	0.21063	0.02532	0.20761	-0.25688	-0.03926	0.09259	0.45272	0.26066	0.28648	-0.12626	0.23938	0.23938	-0.19625	0.071	-0.13635	0.28748	0.31298	0.17693
0.13072	0.22921	0.04455	0.04178	0.20954	0.16522	0.15653	0.18102	0.10226	0.20314	-0.28861	-0.07418	0.04825	0.44881	0.22066	0.30772	-0.13582	0.22656	0.22656	-0.1081	0.01613	-0.09735	0.25818	0.33459	0.17323

OME_AUT	AZATHIO	OR_SEQUE	RIAL_INF	ASE_EXC	ON_REP	ASIGIN_I	RTED_CA	RN_INDEP	IN_PHOSE	BETA_D	_OF_BUT	DECANO	OF_HEXA	LAURO	OF_OCTA	DATION_	_OF_VER	ITH_AND	RBONATE	_AND_BI	_OF_LIGA	_CTNN	BIOLOGIC	HESIS_OF
0.11277	0.12647	0.03887	0.2631	0.19759	0.38639	0.15944	0.31472	0.24476	0.08008	-0.30527	0.2614	0.34348	0.33381	0.40336	0.39234	0.03324	-0.0086	-0.01161	0.07381	0.09108	0.19594	0.09959	0.09824	0.05074
0.09965	0.08578	0.07238	0.21435	0.25504	0.39517	0.14815	0.29383	0.23717	0.02106	-0.3331	0.2128	0.32239	0.29192	0.39315	0.36265	0.01156	-0.00193	-0.04958	0.31708	0.02859	0.20849	0.07005	0.04993	0.12282
0.08882	0.13477	-0.04886	0.25917	0.23688	0.39559	0.17048	0.33177	0.23013	0.1012	-0.24361	0.20198	0.32864	0.29671	0.38127	0.36056	-0.06564	-0.05283	-0.01727	0.30824	0.04568	0.25914	0.10532	0.09307	0.03451
0.12906	0.17288	0.00532	0.26512	0.2188	0.38963	0.15406	0.33475	0.25155	0.06883	-0.14391	0.1758	0.30269	0.2858	0.36864	0.36009	-0.00168	0.02538	-0.06103	0.15753	0.06996	0.32378	0.01014	0.0914	0.11471
0.10582	0.16301	0.05411	0.25354	0.22155	0.39584	0.09591	0.34682	0.26481	0.01986	-0.17298	0.20117	0.29509	0.30234	0.38013	0.357	-0.03174	-0.04006	0.02117	0.20352	0.04949	0.2621	0.05806	0.07709	0.08438
0.08794	0.16293	0.0268	0.23141	0.24177	0.39768	0.12604	0.33934	0.23066	0.083	-0.30421	0.18952	0.37238	0.28655	0.38103	0.36037	-0.04258	-0.03195	-0.06181	0.23537	0.03621	0.26022	0.12107	0.06757	0.19356
0.07911	0.16645	0.05499	0.2294	0.23955	0.40913	0.20152	0.35224	0.22857	-0.00312	-0.23763	0.26213	0.35825	0.33217	0.42256	0.37819	-0.05786	-0.03666	-0.02866	0.24046	-0.03266	0.31908	0.0596	0.05439	0.16782
0.12464	0.17756	-0.0593	0.26755	0.19703	0.38647	0.14685	0.309	0.25642	0.02438	-0.02431	0.2301	0.32804	0.31445	0.3805	0.36912	-0.02718	-0.02076	-0.00849	0.19611	0.09603	0.34396	-0.07854	0.11886	0.1484
0.11662	0.16014	0.0477	0.26171	0.20031	0.38862	0.14103	0.32593	0.2529	0.03497	0.00018	0.22718	0.36925	0.31319	0.38329	0.37302	-0.05659	-0.02412	-0.01189	0.15311	0.06462	0.34268	-0.01724	0.09853	0.14766
0.1119	0.18596	-0.04895	0.26265	0.2226	0.40034	0.13008	0.34414	0.24973	-0.01133	-0.13246	0.24954	0.37751	0.32518	0.39049	0.37984	-0.05816	-0.07945	-0.03722	0.18811	0.01943	0.32417	-0.11405	0.07379	0.11025
0.10587	0.15287	-0.0592	0.22752	0.22913	0.39097	0.13716	0.3025	0.22075	0.02526	-0.44865	0.24114	0.34888	0.3171	0.41235	0.38317	0.02403	0.00701	0.04611	0.13463	0.05328	0.22536	0.07512	0.09141	-0.06979
0.0889	0.21103	-0.01155	0.23018	0.23977	0.40762	0.20012	0.34196	0.23706	0.06187	-0.3981	0.2335	0.38016	0.31595	0.41479	0.40722	-0.08265	0.00067	0.00999	0.16327	-0.01111	0.27912	-0.00607	0.08466	-0.00737
0.14236	0.15079	0.09068	0.21726	0.23824	0.38694	0.11353	0.33234	0.24044	0.13077	-0.4254	0.18686	0.32417	0.27309	0.37321	0.35901	-0.01489	-0.01283	0.01436	0.24579	-0.00805	0.20442	0.08122	0.0447	-0.16748
0.0999	0.18093	0.01399	0.252	0.21854	0.40446	0.11511	0.31791	0.23947	0.07145	-0.20328	0.20832	0.31257	0.30591	0.40032	0.38364	0.01339	0.08192	-0.04485	0.24792	0.09795	0.27125	0.07033	0.10561	0.10021
0.1073	0.17285	-0.02363	0.24859	0.2266	0.39803	0.11397	0.32703	0.22784	0.05785	-0.35705	0.1717	0.3636	0.27034	0.36403	0.35673	-0.02577	-0.05798	-0.04147	0.17199	0.05622	0.24851	0.05021	0.12387	0.17378
0.10526	0.16307	0.09116	0.2236	0.23679	0.3968	0.16883	0.30458	0.21237	0.1198	-0.4247	0.2207	0.34872	0.3256	0.4315	0.39512	0.01709	0.09756	0.11555	0.05646	0.04273	0.1573	0.13314	0.09448	-0.05878
0.12636	0.1576	-0.00776	0.24973	0.20343	0.39989	0.13243	0.32199	0.24686	0.06069	-0.27638	0.23549	0.34332	0.32544	0.41513	0.39538	-0.04667	-0.03442	-0.05317	0.13319	0.03635	0.27077	-0.00295	0.07024	0.09719
0.12031	0.17272	-0.10468	0.26337	0.1951	0.3895	0.13896	0.28834	0.26793	0.02514	-0.08015	0.25763	0.32204	0.33218	0.40341	0.3809	-0.02931	-0.00172	0.0116	0.18793	0.0608	0.28192	0.08895	0.10824	0.02022

YNTHESI	IALIZED_	LIPID_LI	RANSPO	ROUP_SY	ACTIVATE	CHAIN_AN	D_MATU	TOR_1_B	ILIN_BTN	SITIVEL	YME_CA2	INEURIN	XIN_CAL	ATED_PH	TRANSLA	ARDIAC_	E_CARDI	CONCEN	_FOR_CL	ING_TO_	RNITINE	ATH_REC	NDENCE_	_EXTRINS
0.08393	0.04233	0.0277	-0.04249	-0.43509	-0.06222	0.15756	0.15335	-0.15759	-0.00953	0.35786	0.12862	0.17159	0.27053	0.18075	0.21485	0.18242	-0.01966	0.16187	0.11222	0.12983	0.02507	-0.08781	0.01247	-0.05514
-0.00389	0.10278	0.04601	-0.04084	-0.42382	-0.08296	0.09763	0.07918	-0.0682	-0.018	0.36869	0.10571	0.15352	0.24978	0.21501	0.25865	0.15815	0.14493	0.12439	0.08882	0.09111	-0.00701	-0.10896	0.04386	-0.05911
0.04789	0.00079	-0.00435	-0.05105	-0.28325	-0.0242	0.17015	0.09959	-0.16121	0.00571	0.37345	0.09288	0.15025	0.27041	0.24675	0.26028	0.15606	0.01189	0.16263	0.13411	0.09772	0.0416	-0.04875	-0.02269	-0.04079
0.14946	0.05272	0.02468	-0.16441	-0.41747	-0.05244	0.04306	0.1239	-0.16291	0.0623	0.34422	0.1428	0.16649	0.28214	0.1758	0.28226	0.16793	-0.05332	0.15427	0.14808	0.15096	0.03105	-0.02229	0.06051	0.00483
0.10091	0.06499	0.02501	-0.13966	0.00687	-0.07325	0.07114	0.11426	-0.14187	-0.12368	0.34593	0.1288	0.24502	0.27688	0.15691	0.258	0.14457	-0.07411	0.13176	0.15264	0.12629	0.06475	0.00423	0.11174	0.03786
0.23961	0.11267	-0.02309	-0.10875	0.2031	-0.05781	0.11455	0.05526	-0.17146	-0.15526	0.35989	0.05138	0.1778	0.2649	0.25929	0.2326	0.17985	0.01881	0.13308	0.10742	0.10716	0.11894	-0.04319	0.09987	0.00052
0.20241	0.13816	0.00166	-0.03527	-0.41043	0.00266	0.08267	0.01634	-0.07062	-0.09542	0.37579	0.07528	0.18255	0.22139	0.16627	0.26374	0.13612	0.04156	0.13473	0.10466	0.10909	0.01019	-0.04115	0.06112	-0.0081
0.1532	0.06769	0.04872	-0.09179	-0.06553	-0.05412	0.10414	0.13389	-0.16252	0.0188	0.33275	0.1008	0.20306	0.29537	0.18506	0.28912	0.16192	-0.13591	0.18083	0.17817	0.13659	0.06034	-0.02542	0.08597	0.01102
0.14203	0.06244	0.02588	-0.16812	-0.22987	-0.10622	0.0424	0.11808	-0.14731	-0.19338	0.34206	0.08523	0.19013	0.28739	0.13851	0.29164	0.1559	-0.10741	0.16522	0.16588	0.15083	0.04793	-0.00639	0.12508	0.03654
0.15487	0.04725	0.02952	-0.13666	-0.37079	-0.06156	0.07337	0.09251	-0.13441	-0.17769	0.34517	0.10043	0.20713	0.26527	0.17078	0.27971	0.16584	-0.19679	0.1432	0.15758	0.12214	0.07372	-0.00132	0.10514	0.03267
-0.1964	-0.00472	0.01882	-0.05664	-0.45746	-0.06319	0.19201	0.07485	-0.19902	0.00975	0.37146	0.07365	0.15975	0.25997	0.14096	0.32064	0.14456	0.12954	0.14309	0.09122	0.1309	0.02746	-0.09325	-0.00852	-0.06769
-0.14445	0.02587	0.04522	-0.10447	-0.39035	-0.00096	0.16588	0.09045	-0.14925	0.14279	0.3648	0.0854	0.14837	0.26866	0.20281	0.26017	0.17394	-0.04481	0.13733	0.0934	0.14007	0.07156	-0.02368	-0.00277	-0.01756
-0.20472	-0.09899	0.00803	-0.22143	-0.35776	-0.00177	0.14607	0.14668	-0.14675	0.01035	0.36881	0.11373	0.12628	0.24611	0.1088	0.35104	0.15	0.13964	0.09131	0.0948	0.13138	0.05413	-0.09422	0.10152	-0.03027
0.05041	0.06961	0.06851	-0.10353	-0.39722	-0.08903	0.12935	0.08254	-0.14579	0.02396	0.36459	0.08827	0.16412	0.25526	0.15831	0.29796	0.14422	0.01571	0.16347	0.13212	0.13231	0.05269	-0.05361	-0.03368	-0.04716
0.16787	0.12862	0.05378	-0.07055	-0.4409	-0.04897	0.09541	0.11249	-0.09915	-0.03953	0.35438	0.06097	0.16086	0.2696	0.1681	0.26639	0.14602	-0.01963	0.1688	0.10489	0.12611	0.03708	-0.0673	0.05969	-0.02783
0.00854	-0.03494	0.00423	-0.05128	-0.46574	-0.08854	0.18101	0.10349	-0.11393	0.02621	0.37143	0.0558	0.14552	0.25698	0.18315	0.30058	0.14239	0.10614	0.08975	0.08734	0.12254	0.06677	-0.10389	-0.02763	-0.08064
0.19582	0.07201	-0.01932	-0.0143	-0.35882	-0.05764	0.14465	0.12246	-0.16863	0.05154	0.36032	0.12983	0.13471	0.27023	0.13769	0.31358	0.14782	-0.04497	0.14746	0.12371	0.11804	0.08523	-0.06331	0.04458	-0.0285
-0.00233	0.01502	0.03381	-0.13643	-0.15448	-0.12059	0.07892	0.11099	-0.13629	-0.03743	0.3428	0.13872	0.23139	0.27853	0.15766	0.32231	0.15413	-0.04	0.13998	0.16403	0.11402	0.06006	-0.02299	0.06095	0.0038

CLEAVA	GLED_CH	LA_DEPEN	G_AN_ANT	09_DC_S	EDIATED	D28_CO	ENDENT	DEPENDE	DC42_GT	IC_AND_	LAR_HEL	AR_RESP	ESPONSE	_RESPO	LAR_RES	R_RESPO	ELLULAR	L_CELL_	LL_JUNC	ME_CELL	L_CYCLE	CELL_CY	ALLING_	ELLULA
0.22937	0.05898	0.22095	0.20549	0.2157	0.26411	0.0448	-0.22058	0.24367	0.00464	0.38679	0.24618	0.23278	0.23201	0.13757	0.24622	0.33497	0.19538	0.14629	0.1523	0.15569	0.18823	0.1713	-0.04635	0.15753
0.23856	0.16604	0.21276	0.16015	0.13161	0.23292	0.00774	-0.24354	0.18017	-0.03671	0.34016	0.17097	0.23355	0.21826	0.15195	0.24826	0.33719	0.1933	0.12997	0.15434	0.16811	0.19069	0.18004	-0.07309	0.17215
0.25535	-0.00087	0.23397	0.18436	0.16202	0.28704	0.06386	-0.17116	0.22621	0.0287	0.38261	0.21119	0.23062	0.22239	0.15306	0.21927	0.33929	0.18837	0.14564	0.14626	0.16486	0.18367	0.178	-0.05076	0.18035
0.24247	-0.25848	0.21609	0.25218	0.20067	0.37034	0.10151	-0.11838	0.2561	0.04531	0.33306	0.26373	0.22988	0.22212	0.1032	0.24082	0.35673	0.18359	0.10994	0.09252	0.14955	0.18155	0.16613	-0.0117	0.19089
0.27223	-0.23056	0.23112	0.19558	0.24535	0.32826	0.08633	-0.15522	0.28888	0.04405	0.27983	0.28324	0.23079	0.24099	0.10729	0.26509	0.33965	0.18192	0.07894	0.03834	0.15746	0.18602	0.1741	-0.01121	0.17342
0.27123	-0.24397	0.25468	0.10752	0.13895	0.18205	0.03857	-0.14149	0.23624	0.00954	0.3169	0.22501	0.2247	0.22463	0.12736	0.24229	0.33807	0.18086	0.10144	0.09738	0.16187	0.18903	0.17597	-0.05952	0.14442
0.27027	-0.03961	0.24154	0.18291	0.18416	0.22948	0.11257	-0.07528	0.28509	0.02413	0.33436	0.19702	0.22836	0.22627	0.1302	0.22559	0.34055	0.19044	0.11435	0.10182	0.14663	0.17019	0.15893	-0.05382	0.14946
0.24412	-0.22076	0.21136	0.22816	0.20994	0.37838	0.09648	-0.16052	0.27148	0.05252	0.14446	0.31034	0.23224	0.23336	0.09899	0.25147	0.35472	0.17737	0.10363	0.01572	0.13837	0.17065	0.15769	-0.001	0.22218
0.26048	-0.24113	0.19631	0.19906	0.20184	0.38341	0.10867	-0.13385	0.28484	0.06145	0.28588	0.32781	0.22758	0.22992	0.09942	0.24421	0.35085	0.17012	0.10737	0.03544	0.14084	0.1679	0.16062	0.01538	0.22251
0.26961	-0.14174	0.24243	0.23874	0.22958	0.38187	0.10481	-0.16326	0.31078	0.05879	0.18885	0.2011	0.22633	0.22914	0.09706	0.24426	0.33973	0.18856	0.09186	0.03466	0.14293	0.16548	0.15999	0.00236	0.18318
0.24536	-0.22426	0.16117	0.01894	0.11121	0.06404	0.02375	-0.17961	0.15821	-0.03669	0.36312	0.26134	0.23476	0.22665	0.13108	0.23969	0.34221	0.18158	0.13272	0.17162	0.1636	0.19739	0.17548	-0.07377	0.1515
0.23198	-0.21212	0.24466	0.14462	0.13661	0.17985	0.02223	-0.20904	0.20049	-0.00587	0.31137	0.14094	0.22844	0.22737	0.12943	0.24188	0.33703	0.18778	0.12097	0.10488	0.15644	0.1931	0.16814	-0.06817	0.16011
0.27091	-0.28402	0.19639	-0.02204	0.0963	-0.0375	-0.00476	-0.17348	0.17114	-0.04092	0.4073	0.19106	0.23879	0.21568	0.16881	0.24728	0.34448	0.18136	0.14486	0.19834	0.18466	0.21163	0.19523	-0.07059	0.13358
0.25277	-0.38211	0.21293	0.22523	0.18372	0.28862	0.05897	-0.17017	0.2438	0.01871	0.36287	0.2941	0.23845	0.22495	0.14488	0.23849	0.35628	0.1824	0.12571	0.14843	0.15097	0.17093	0.16468	-0.04725	0.19235
0.25502	-0.26358	0.2088	0.22015	0.16263	0.24494	0.02096	-0.27983	0.23314	-0.00316	0.35392	0.21622	0.2358	0.22282	0.12576	0.24249	0.35448	0.18354	0.11722	0.12159	0.15631	0.18327	0.17006	-0.05701	0.1759
0.23766	-0.00781	0.18975	0.05463	0.08524	0.00187	0.02213	-0.20708	0.18193	-0.04573	0.37494	0.14182	0.22188	0.20736	0.13667	0.23211	0.33765	0.17246	0.14404	0.18546	0.16821	0.19629	0.17776	-0.08665	0.16305
0.25082	-0.30204	0.20146	0.22022	0.19304	0.29664	0.10029	-0.06427	0.2495	0.033	0.30187	0.2731	0.23526	0.2276	0.12792	0.23711	0.35115	0.19226	0.13454	0.12584	0.14746	0.16603	0.16037	-0.03782	0.1805
0.24934	-0.11526	0.2188	0.19781	0.20221	0.30591	0.10677	-0.07399	0.28946	0.05296	0.36721	0.32157	0.22836	0.23321	0.09493	0.25551	0.33794	0.18245	0.12345	0.07689	0.14966	0.17596	0.1651	-0.01575	0.1961

JUNCTIO	ITERACTI	ONE_ME	RECEPT	D_INACT	HL1_IN	ESTERO	HOLINE	DITIN_SU	LFATE_DE	TES_MET	RING_TH	ATIN_MO	OMOSOM	YLOMICR	LOMICR	OMICRO	_CILIUM	IPROFL	C_CIRCA	DIC_ACID	RHODOP	SECRETE	ED_ANTI	SOMAL_
0.12616	0.07638	0.36206	-0.24978	0.31482	0.09895	-0.00068	-0.10292	0.38365	0.07682	0.20117	0.47561	0.1529	0.16914	0.22428	0.29141	0.18726	0.13865	0.49146	0.06306	0.31444	-0.00187	0.09337	0.13048	0.07329
0.12513	0.06136	0.33793	-0.32782	0.29539	0.11795	-0.08656	-0.12621	0.43315	0.08446	0.19818	0.47887	0.18423	0.2038	0.18684	0.36291	0.13393	0.14719	0.4911	0.04807	0.29318	0.00247	0.07898	0.13575	0.04521
0.12384	0.09202	0.34117	-0.41227	0.26655	0.1367	-0.04574	-0.02775	0.24132	0.02449	0.12982	0.4793	0.16702	0.19756	0.24486	0.3804	0.20844	0.13739	0.49304	0.05637	0.32493	-0.02183	0.06406	0.13654	0.041
0.08282	0.12314	0.3672	-0.2882	0.30518	0.11863	-0.06606	0.00925	0.44671	0.06887	0.13987	0.47721	0.13821	0.16648	0.31842	0.38711	0.303	0.16021	0.49339	0.03251	0.29141	0.06994	0.18956	0.14998	0.0459
0.03457	0.09953	0.33382	-0.43177	0.27209	0.18118	-0.03446	-0.14429	0.25718	0.11123	0.1745	0.47758	0.16001	0.17072	0.27311	0.38697	0.2603	0.1552	0.49304	0.04592	0.28943	0.00118	0.11976	0.15426	-0.01066
0.07434	0.06973	0.3289	-0.16271	0.28849	0.19411	-0.04986	-0.16475	0.49022	0.06113	0.20427	0.47779	0.16794	0.19341	0.38347	0.44028	0.40095	0.13416	0.49551	0.05851	0.25254	0.08928	0.04589	0.13674	-0.04508
0.08023	0.12847	0.32092	-0.14317	0.26588	0.07688	-0.0468	-0.05865	0.42593	0.09012	0.20602	0.48691	0.15885	0.19842	0.29928	0.41482	0.29269	0.12797	0.49621	0.04901	0.29815	0.13075	0.06654	0.12826	-0.01369
0.06051	0.13574	0.37661	-0.19843	0.3013	0.17362	-0.09856	-0.21127	0.46169	0.03625	0.15204	0.47377	0.1244	0.13669	0.35447	0.42818	0.36136	0.16299	0.4948	0.03463	0.28399	0.09772	0.17301	0.16238	0.03444
0.07177	0.11633	0.36645	-0.08113	0.26279	0.15103	-0.05938	-0.18347	0.43298	0.12336	0.14149	0.47684	0.13049	0.14596	0.35211	0.4144	0.36089	0.16889	0.49568	0.02624	0.26976	0.13218	0.14204	0.16209	0.02601
0.04705	0.12345	0.33767	-0.11308	0.24233	0.10139	-0.09351	-0.16844	0.47613	0.05729	0.20845	0.47925	0.13606	0.16814	0.37019	0.42615	0.38074	0.14929	0.49287	0.02529	0.28935	0.11993	0.16131	0.15163	-0.04687
0.13062	0.03431	0.33004	-0.24593	0.34171	0.05987	-0.02069	-0.00459	-0.24679	-0.02735	0.17174	0.48093	0.16944	0.19264	0.24998	0.32755	0.21405	0.17248	0.49287	0.05058	0.30945	0.04236	0.0555	0.13173	0.01883
0.10215	0.07066	0.33228	-0.29211	0.35813	0.12129	0.02112	-0.16524	0.418	0.05974	0.18642	0.483	0.15142	0.19275	0.3133	0.38342	0.30523	0.14841	0.49568	0.06836	0.31423	0.05139	0.10844	0.13237	-0.01161
0.13703	0.02005	0.32838	-0.33917	0.34587	0.16654	0.0618	-0.03573	0.33556	0.03461	0.14527	0.47569	0.18027	0.21308	0.30356	0.3887	0.31364	0.18661	0.49251	0.09027	0.23915	0.01603	0.14847	0.12971	-0.00144
0.10881	0.09936	0.3401	-0.29515	0.27451	0.1076	-0.04391	-0.12939	0.25048	0.09008	0.12113	0.48354	0.15308	0.16539	0.24343	0.37152	0.22037	0.15936	0.49181	0.03959	0.32257	0.04588	0.07588	0.14393	0.01755
0.09103	0.0795	0.33526	-0.18129	0.32218	0.08416	0.01777	-0.09241	0.40109	0.03582	0.16677	0.47763	0.16265	0.17889	0.31437	0.40214	0.31112	0.15233	0.49357	0.05342	0.2728	0.04684	0.06406	0.13586	-0.02899
0.1392	0.02284	0.32701	-0.29764	0.35197	0.07695	-0.01501	0.01465	0.02466	0.07799	0.20655	0.47565	0.16455	0.19691	0.1904	0.2836	0.1338	0.16108	0.49181	0.07071	0.34616	-0.05341	0.02313	0.12309	0.03092
0.11554	0.09091	0.3629	-0.30542	0.29249	0.13646	-0.10193	-0.054	0.43597	0.07378	0.16683	0.48197	0.15469	0.16233	0.29193	0.40268	0.28054	0.15772	0.49022	0.05962	0.33058	0.06901	0.1942	0.12432	-0.00302
0.09862	0.1105	0.34843	-0.29195	0.27185	0.15785	-0.09279	-0.12956	0.18725	-0.00187	0.12423	0.47587	0.12998	0.15471	0.27303	0.41562	0.26622	0.15971	0.49392	0.00272	0.31975	0.04432	0.14606	0.15068	-0.00303

MIN_MED	TIN_1_IN	7A_DECTA	_INFLAM	LAMIN_C	AMIN_B12	NZYME_A	_LOADIN	YNTHESI	GEN_CHA	LLAGEN_	OLLAGEN	WAY_OF_	OMPLEM	OMPLEX_I	_OF_PRO	N_OF_P	NALING_B	IGNALIN	TIVE_SI	GY_LIGA	GNALING_C	CDC20	P1_AND_	_AND_TR
0.14751	0.17487	0.21341	0.05965	0.02546	-0.09505	0.01251	-0.00569	0.04778	-0.24249	0.01178	0.05645	0.09384	-0.02432	0.05685	-0.10772	0.41732	0.02649	-0.18086	0.08822	0.12823	0.13545	-0.19503	0.27197	0.28589
0.12156	0.09718	0.21114	0.01564	-0.03685	-0.08852	-0.00628	0.10544	0.04993	-0.23069	-0.05684	0.0639	0.07745	-0.09351	0.0995	-0.01855	0.42385	-0.02788	-0.14736	0.09339	0.134	0.11107	-0.11546	0.27392	0.28225
0.14894	0.08296	0.19364	0.05564	-0.15635	-0.26315	0.00895	0.10179	0.07144	-0.1352	0.01086	0.0743	0.16697	0.02931	0.05973	0.02335	0.42608	0.03635	-0.13714	0.09854	0.13781	0.12273	-0.21899	0.27361	0.27503
0.15683	0.12101	0.21054	0.10626	-0.01341	-0.15874	-0.02879	0.00324	0.07745	-0.10806	0.05705	0.06083	0.18342	0.13704	0.10518	-0.06858	0.41464	0.08257	-0.12602	0.10672	0.14486	0.08966	-0.1617	0.31642	0.28908
0.16553	0.18435	0.23798	0.13488	-0.20109	-0.28833	-0.02044	0.10761	0.0283	-0.1612	0.03816	0.02791	0.1451	0.05634	0.04854	-0.04248	0.42049	0.07644	-0.14728	0.12113	0.15861	0.12091	-0.26008	0.28794	0.29622
0.13074	0.1122	0.2056	0.07456	-0.02386	-0.18841	-0.00448	0.08741	0.07552	-0.11419	-0.03602	0.05456	0.22585	0.22273	0.09002	-0.03893	0.42336	0.03979	-0.15447	0.07535	0.11718	0.07846	-0.15474	0.25334	0.27776
0.1247	0.12278	0.2055	0.10436	-0.17526	-0.13192	0.02661	0.0856	0.13424	0.06665	0.09918	0.11425	0.21984	0.22745	0.08696	-0.02929	0.43126	0.07555	-0.13682	0.08863	0.12794	0.08139	-0.20592	0.26582	0.2736
0.18137	0.10463	0.22316	0.09109	-0.0185	-0.21128	-0.00848	-0.00176	0.10782	-0.14225	0.06815	0.09604	0.23378	0.15109	0.09566	-0.11039	0.40643	0.0951	-0.15823	0.09662	0.13712	0.08134	-0.17826	0.28696	0.29281
0.1714	0.0966	0.23311	0.189	-0.14422	-0.24106	0.00212	-0.01558	0.14625	0.05538	0.09838	0.11345	0.23447	0.15736	0.1009	-0.06856	0.41376	0.10408	-0.15505	0.10972	0.1482	0.07666	-0.221	0.28543	0.30116
0.16238	0.1391	0.22664	0.13	-0.11852	-0.21015	-0.02417	0.0274	0.04147	-0.17552	0.03192	0.03128	0.21198	0.17878	0.11718	-0.09263	0.41161	0.09695	-0.12445	0.07977	0.12242	0.0798	-0.24616	0.29964	0.28992
0.1244	0.09503	0.20407	0.05093	0.07103	-0.14004	0.06589	0.08126	0.04128	-0.369	-0.04719	0.04756	0.12342	0.07059	0.13547	0.02387	0.43146	0.02487	-0.15124	0.10442	0.14244	0.07432	-0.13316	0.29671	0.32398
0.12321	0.13395	0.20757	0.01297	-0.03854	-0.17323	0.05708	0.07192	0.0569	-0.14902	-0.01369	0.08231	0.20787	0.18253	0.09578	-0.07627	0.42766	0.02758	-0.17948	0.05814	0.10293	0.08213	-0.21513	0.29754	0.29768
0.14663	0.09145	0.19812	-0.06125	-0.05112	-0.20551	0.17195	0.07306	0.0188	-0.26273	-0.06951	0.03474	0.09926	0.04386	-0.08015	0.0403	0.4277	-0.04367	-0.05907	0.12617	0.16184	0.20619	-0.13512	0.30665	0.33848
0.15381	0.10318	0.2115	0.09002	0.02635	-0.15938	0.03182	0.05883	0.03781	-0.18052	0.00306	0.04292	0.19571	0.10112	0.05061	-0.11214	0.41673	0.04285	-0.16776	0.06858	0.1117	0.112	-0.20122	0.27683	0.31494
0.13065	0.09142	0.21014	0.08111	-0.03516	-0.17043	0.00706	0.03815	0.03168	-0.23701	0.01523	0.02475	0.23186	0.08682	0.05677	-0.0937	0.41801	0.01741	-0.19919	0.05109	0.09603	0.0653	-0.19912	0.28278	0.29954
0.12135	0.05503	0.18981	0.04807	-0.07467	-0.20519	0.02955	0.10909	0.01912	-0.21616	-0.02368	0.03077	0.04853	-0.10566	0.06753	0.03901	0.42993	-0.03884	-0.0849	0.06843	0.11117	0.10473	-0.15457	0.27592	0.33339
0.14855	0.08517	0.19603	0.07675	0.00697	-0.15425	-0.04424	0.00833	0.01985	-0.20824	0.00938	0.04267	0.16533	0.03998	0.08676	-0.10188	0.42155	0.07938	-0.13566	0.12373	0.16041	0.12797	-0.16928	0.31956	0.30846
0.16723	0.16118	0.22532	0.10965	-0.13412	-0.22342	-0.00447	0.01147	0.0422	-0.17527	-0.02213	0.03056	0.11852	0.06251	0.1162	-0.07053	0.41111	0.10432	-0.14593	0.11839	0.15546	0.08465	-0.23449	0.28267	0.30319

LATION_E	REATINE_	S_OF_C4	NMDA_RE	ROUGH_	THE_AC	EB_PHO	CRISTAE	PS_IN_SE	KING_OF	RTICULA	SOLUBL	CS_DS_D	M_INHIBI	CIATED_E	SSOCIATE	D_ASSOCI	CYP2E1_	C_MEDIA	450_ARRA	SIGNALIN
0.04524	0.06392	-0.1484	0.14274	0.23863	0.16413	0.0399	0.24536	-0.01277	-0.29186	0.08922	0.27296	0.2276	0.01709	0.18511	0.246	0.12378	-0.38136	0.10712	-0.0701	0.1661
0.00338	0.00416	-0.12127	0.14201	0.26912	0.19247	0.01702	0.23843	0.16605	-0.28287	0.03963	0.27875	0.201	-0.03496	0.19873	0.2519	0.11846	0.01462	0.09141	-0.1099	0.16067
0.062	0.24951	0.06711	0.12956	0.19451	0.24384	0.06755	0.25642	0.02507	-0.24414	0.21339	0.25518	0.20218	0.04519	0.21508	0.23531	0.15124	-0.03752	0.12055	-0.04654	0.1797
0.1012	0.18475	0.20778	0.0994	0.22746	0.18776	0.03161	0.19346	0.05527	-0.10545	0.25847	0.2757	0.27942	0.05299	0.16622	0.23931	0.13232	0.14039	0.09757	-0.01774	0.18854
0.11969	0.18039	0.05605	0.10114	0.27385	0.18139	0.08206	0.21307	0.03761	-0.22551	0.18002	0.29606	0.24341	0.02215	0.17442	0.2682	0.12308	-0.21895	0.09051	-0.10607	0.19212
0.07559	0.14957	0.28456	0.07847	0.24094	0.27685	0.04183	0.19215	0.09817	-0.12101	0.04508	0.27311	0.23896	0.03169	0.17869	0.2616	0.12792	-0.11837	-0.00626	-0.14859	0.17178
0.09956	0.00748	0.35052	0.07418	0.22931	0.17532	0.01723	0.23987	0.02032	-0.03094	0.18427	0.26164	0.24059	0.01767	0.19682	0.24362	0.12282	-0.08966	0.09617	-0.13864	0.18819
0.10223	-0.15747	0.21019	0.1028	0.2636	0.19033	0.07602	0.19792	0.06935	-0.08696	0.27029	0.2985	0.29315	0.03468	0.13178	0.25844	0.12691	-0.11978	0.09251	-0.00278	0.19167
0.14631	-0.08645	0.22664	0.07652	0.25109	0.13288	0.0646	0.17498	0.07893	0.01511	0.26198	0.30007	0.32531	0.0425	0.13614	0.26201	0.12587	-0.10005	0.1138	-0.04627	0.20494
0.12224	-0.01182	0.25438	0.09125	0.26756	0.15946	0.10587	0.20329	0.04524	-0.2098	0.22325	0.28658	0.3319	0.04044	0.13256	0.25429	0.12808	-0.26581	0.107	-0.06452	0.20545
0.02184	0.2726	-0.00407	0.05229	0.20932	0.21471	0.01115	0.23642	0.07864	-0.28943	0.09512	0.25851	-0.01332	-0.06605	0.20673	0.2472	0.12997	0.2725	0.08683	-0.13758	0.15641
0.06371	0.12565	0.10719	0.10231	0.27712	0.2263	0.05764	0.20053	0.08607	-0.16164	0.02168	0.27186	0.2708	-0.00418	0.15833	0.2475	0.07646	-0.02836	0.07099	-0.1359	0.17759
-0.01542	0.05689	-0.05066	0.14485	0.2141	0.13859	0.10996	0.27032	-0.03368	-0.40074	-0.03656	0.26712	0.0044	0.00898	0.24852	0.26086	0.17742	0.03364	0.06913	-0.21035	0.1517
0.08349	0.02522	0.08677	0.11235	0.25387	0.17714	0.02759	0.22869	0.06853	-0.20875	0.20242	0.26873	0.25911	0.00706	0.1613	0.24397	0.10729	0.10041	0.07435	-0.01723	0.18191
0.07404	0.06247	0.05204	0.11838	0.24487	0.18541	0.07128	0.20836	0.04356	-0.19894	0.14354	0.27272	0.2281	-0.02311	0.19689	0.24689	0.11564	-0.30491	0.11746	-0.08484	0.17732
0.03252	0.16085	-0.25231	0.11075	0.20329	0.23243	-0.06483	0.26912	0.04757	-0.32056	-0.1486	0.2538	0.03812	-0.00691	0.26065	0.2361	0.08281	0.17386	0.09782	-0.16616	0.15374
0.08286	0.24931	-0.03638	0.10728	0.22779	0.15291	0.01374	0.26536	0.09849	-0.28208	0.16077	0.26236	0.30967	0.03705	0.16946	0.2372	0.15093	0.09635	0.06847	-0.03	0.17299
0.11797	-0.03709	0.10239	0.08423	0.25018	0.18435	0.04233	0.21879	0.06122	-0.23292	0.23645	0.28925	0.2156	0.02622	0.15196	0.25727	0.09503	0.17333	0.10776	-0.00595	0.19211

PROTEC	ON_SUL	ORS_OF_L	FONATI	LIC_TRN	LECTIN	IG_AND_I	DAP12_IN	DAP12_S	DARPP	IATED_AT	INDUCT	BETA_CA	TION_DE	ADENYLA	TH_RECE	ED_NONC
0.15317	0.00023	0.05048	0.00702	0.31153	0.22155	0.1973	0.05206	0.00067	0.19692	0.15159	0.04802	0.13135	0.01981	0.13829	-0.01481	0.28326
0.13899	-0.02232	0.0408	-0.12836	0.31592	0.21108	0.20259	0.03758	-0.013	0.18922	0.07234	0.0452	0.16259	0.09076	0.18058	-0.03442	0.29327
0.14082	0.00543	0.04305	-0.07396	0.31325	0.19593	0.16912	0.07174	0.02355	0.14505	0.16587	0.04049	0.1726	0.03403	0.16636	-0.02561	0.27163
0.15426	0.02481	0.03603	0.00592	0.28461	0.21591	0.18346	0.11358	0.0667	0.19242	0.04981	0.03277	0.13566	0.03211	0.14664	0.0026	0.2719
0.15337	-0.00409	0.04305	0.01809	0.29781	0.23796	0.18965	0.1269	0.09098	0.22097	0.06351	0.05959	0.11354	0.02909	0.12226	0.00401	0.30421
0.14676	0.03784	0.01416	-0.15656	0.30908	0.1979	0.214	0.08489	0.03884	0.19953	0.10616	0.03856	0.09878	0.02813	0.1511	-0.02617	0.29184
0.16044	0.0429	0.04962	-0.14148	0.31516	0.20359	0.18658	0.09837	0.05308	0.16065	0.14663	0.07253	0.14306	0.05556	0.14533	-0.02199	0.27746
0.15351	0.04251	0.03207	0.07738	0.28088	0.22157	0.17502	0.11988	0.07601	0.21107	0.08363	0.05834	0.08739	0.00405	0.10622	0.00547	0.29022
0.14004	0.04503	0.02674	0.01755	0.28103	0.22742	0.16348	0.13488	0.09219	0.19793	0.08511	0.05523	0.10203	-0.00018	0.09677	0.01117	0.29715
0.15907	0.04253	0.03125	-0.01087	0.28245	0.22329	0.19713	0.13925	0.09597	0.19452	0.08125	0.08437	0.11749	0.01943	0.10881	0.0106	0.28666
0.13221	-0.01611	0.01888	-0.05543	0.33898	0.19982	0.12105	0.02909	-0.01799	0.16002	0.11841	0.07325	0.14632	0.04911	0.18439	-0.03713	0.2826
0.16234	0.01802	0.03165	-0.07736	0.35057	0.20404	0.20493	0.03559	-0.02332	0.21345	0.1323	0.05455	0.10484	0.01367	0.12439	-0.04043	0.28654
0.13418	-0.01905	0.0199	-0.14735	0.30753	0.18957	0.15245	-0.00508	-0.04791	0.18313	0.15299	0.05689	0.14833	0.05102	0.16299	-0.02283	0.29227
0.14466	-0.05665	0.03587	-0.00186	0.29905	0.20806	0.17148	0.07746	0.03717	0.19222	0.12581	0.06388	0.10651	0.034	0.15558	-0.01237	0.28051
0.1259	0.01987	0.03139	0.02432	0.33901	0.20462	0.17371	0.03941	-0.01112	0.185	0.12589	0.07027	0.10535	0.0412	0.14188	-0.0156	0.28677
0.1297	0.00525	0.03836	-0.08773	0.35736	0.18178	0.13223	0.00622	-0.05821	0.17963	0.13419	0.08098	0.10534	0.04563	0.16534	-0.0387	0.27469
0.17834	-0.00518	0.03645	-0.04768	0.29484	0.20787	0.15857	0.07902	0.04175	0.17699	0.16107	0.0343	0.11844	0.02609	0.15851	-0.02	0.27052
0.1544	-0.02256	0.05698	0.03275	0.29733	0.2218	0.16495	0.11573	0.08849	0.20914	0.05687	0.05061	0.11349	0.03264	0.12722	0.00045	0.29283

DECTIN	LT1_CA	LT7_CA	IVE_C1GA	CFTR_CA	USES_E	DIVE_CHS	TIVE_CHS	TIVE_CH	E_EXT2_C	FECTIVE_	ACTOR_	ACTOR_V	TIVE_GAL	TRINSIC_	TIVE_LF
0.18836	-0.3444	0.02422	0.31357	0.27255	0.38365	0.38365	-0.37344	0.38365	-0.01798	-0.23534	-0.26142	-0.02391	0.19975	0.28877	-0.31372
0.15316	-0.20347	0.09213	0.28024	0.27013	0.43315	0.43315	-0.37097	0.43315	0.05973	-0.11538	-0.16926	0.06048	0.21294	0.28702	-0.33962
0.17293	-0.13205	-0.10258	0.24614	0.2534	0.24132	0.24132	-0.0458	0.24132	-0.17654	-0.20927	-0.11385	0.16194	0.1769	0.27786	-0.35741
0.22281	-0.1995	0.00758	0.18337	0.26288	0.44671	0.44671	-0.18778	0.44671	-0.06867	-0.04738	-0.06042	0.22478	0.09149	0.2618	-0.38612
0.15955	-0.26	0.00157	0.09842	0.29296	0.25718	0.25718	-0.34349	0.25718	-0.05105	-0.07733	-0.14043	0.20417	0.01584	0.25188	-0.39563
0.06449	-0.00863	-0.02546	0.15473	0.27112	0.49022	0.49022	0.13986	0.49022	-0.13745	0.05566	-0.02674	0.24945	0.07088	0.26764	-0.36075
0.09415	0.11701	0.04323	0.13143	0.25683	0.42593	0.42593	0.2105	0.42593	-0.01017	0.08578	-0.00856	0.25678	0.05855	0.23731	-0.3213
0.1753	-0.0733	-0.13113	0.04271	0.28679	0.46169	0.46169	-0.01938	0.46169	-0.35365	0.00247	-0.02361	0.25067	-0.03471	0.26912	-0.44055
0.15055	-0.04661	0.0117	-0.05675	0.28511	0.43298	0.43298	0.07627	0.43298	-0.0701	0.08631	-0.01286	0.24604	-0.11825	0.26277	-0.3863
0.14732	-0.1734	-0.06731	-0.073	0.26873	0.47613	0.47613	-0.21543	0.47613	-0.18452	0.20962	0.00835	0.26914	-0.10508	0.24374	-0.37132
0.12082	-0.04158	-0.09771	0.27752	0.2588	-0.24679	-0.24679	-0.09195	-0.24679	-0.04802	-0.13211	-0.16126	0.12125	0.20163	0.29784	-0.35424
0.12169	0.02687	-0.05601	0.25159	0.26986	0.418	0.418	0.03083	0.418	-0.15524	-0.06694	0.0004	0.22239	0.25284	0.29642	-0.33504
-0.04036	-0.17293	0.06081	0.23871	0.27138	0.33556	0.33556	-0.32394	0.33556	0.05371	-0.11714	-0.06039	0.10528	0.15706	0.29385	-0.30932
0.08026	-0.06395	-0.00754	0.04296	0.26936	0.25048	0.25048	-0.00018	0.25048	-0.07137	-0.25788	-0.21417	0.13174	0.05912	0.27526	-0.42276
0.08567	-0.13123	-0.07567	0.1131	0.27438	0.40109	0.40109	-0.00881	0.40109	-0.20441	0.01392	-0.11204	0.22185	0.04123	0.26965	-0.35547
0.00892	-0.30409	0.08244	0.28685	0.26091	0.02466	0.02466	-0.22811	0.02466	0.14982	-0.07786	-0.05492	0.13867	0.20206	0.28765	-0.32394
0.24134	-0.23156	-0.00925	0.35422	0.25946	0.43597	0.43597	-0.28994	0.43597	-0.07068	-0.28483	-0.20181	0.15256	0.246	0.30852	-0.35353
0.1423	-0.18289	-0.13423	0.04725	0.28404	0.18725	0.18725	-0.14004	0.18725	-0.19507	-0.10939	-0.17234	0.19147	-0.03566	0.25843	-0.39898

1_MEDIA	GAL3_CA	IN_BIOT	N_COBAL	TAMIN_	ION_SYST	ME_DEF	EGRADA	_CATENI	OF_CYST	EGRADA	N_OF_GL	_OF_THE	ATION_O
-0.15331	-0.37344	-0.04249	-0.12437	-0.07039	-0.02599	0.12548	0.30405	0.25452	0.14383	0.29108	0.28337	0.09388	0.26601
-0.24899	-0.37097	-0.04084	-0.13486	-0.07267	0.09264	0.11428	0.30779	0.25228	0.15101	0.29111	0.28217	0.04717	0.27687
-0.16741	-0.0458	-0.05105	-0.26211	-0.11955	0.11255	0.04547	0.28368	0.24287	0.16952	0.26848	0.26425	0.07723	0.2617
-0.10705	-0.18778	-0.16441	-0.11272	-0.14542	0.208	0.07289	0.2916	0.24287	0.11468	0.27682	0.2744	0.11548	0.22897
-0.07736	-0.34349	-0.13966	-0.28375	-0.18715	0.12847	0.08318	0.32599	0.26073	0.12197	0.31028	0.30204	0.09605	0.2467
-0.15916	0.13986	-0.10875	-0.17094	-0.13065	0.25655	0.05613	0.30753	0.25543	0.15183	0.29278	0.27941	0.08454	0.23994
-0.16431	0.2105	-0.03527	-0.27889	-0.11119	0.23793	0.16158	0.28949	0.23551	0.22031	0.27579	0.2678	0.14106	0.22395
-0.10605	-0.01938	-0.09179	-0.16785	-0.11732	0.21764	0.10396	0.31415	0.2482	0.13901	0.2954	0.29221	0.12654	0.19195
-0.09213	0.07627	-0.16812	-0.24037	-0.19337	0.20457	0.13293	0.31853	0.25273	0.09272	0.29789	0.29426	0.14268	0.20508
-0.10521	-0.21543	-0.13866	-0.24179	-0.17064	0.22008	0.12427	0.30716	0.24061	0.11767	0.28811	0.27779	0.10795	0.19635
-0.25584	-0.09195	-0.05664	-0.10534	-0.07321	0.09069	0.16063	0.29031	0.24429	0.18215	0.27852	0.27265	0.05583	0.25523
-0.16565	0.03083	-0.10447	-0.18973	-0.13169	0.20774	0.08401	0.30369	0.24643	0.16216	0.28857	0.28148	0.09509	0.1956
-0.11017	-0.32394	-0.22143	-0.16659	-0.20122	0.13534	0.01921	0.30102	0.26291	0.13562	0.28976	0.28152	0.03763	0.27074
-0.17441	-0.00018	-0.10353	-0.09565	-0.10077	0.10299	0.11054	0.29663	0.24613	0.15231	0.28107	0.27741	0.07521	0.21306
-0.16597	-0.00881	-0.07055	-0.18685	-0.10763	0.16377	0.1859	0.30065	0.2405	0.17559	0.28148	0.2754	0.08607	0.2434
-0.21053	-0.22811	-0.05128	-0.16905	-0.08982	0.08443	0.01421	0.28616	0.24414	0.17207	0.27279	0.26064	0.03117	0.24881
-0.17948	-0.28994	-0.0143	-0.12501	-0.05419	0.09926	0.04893	0.28945	0.24044	0.15426	0.27395	0.26947	0.09489	0.21186
-0.10926	-0.14004	-0.13643	-0.22015	-0.16484	0.14388	0.11371	0.31557	0.25258	0.12654	0.29953	0.29175	0.06997	0.23919

CONTAIN	AN_SULF	N_OF_RE	DEUBIQ	VELOPM	YPE_I_IF	NOME_DI	GESTION_A	N_OF_DI	E_COMPLEX	D_WITH_G	H_GLYCO	D_WITH_	D_WITH_	IATED_WI	F_CARB	EASES_O	ASES_OF
0.39569	0.38365	0.32308	0.1625	0.20547	0.11404	-0.29624	-0.32449	-0.41014	0.06775	0.03906	0.15094	-0.00451	-0.037	0.23833	0.14131	-0.03899	0.03964
0.40637	0.43315	0.27454	0.17297	0.21871	0.09424	-0.30113	-0.27714	-0.42239	-0.02597	0.04841	0.17912	0.01115	-0.00868	0.13299	0.07258	0.02268	0.0552
0.40257	0.24132	0.31067	0.1526	0.20799	0.12212	-0.30138	-0.29573	-0.4623	0.04579	0.01941	0.09421	-0.08972	-0.05816	0.30491	0.12907	0.03535	-0.01119
0.3926	0.44671	0.30809	0.14921	0.20325	0.07064	-0.3633	-0.2451	-0.45021	0.0132	0.06105	0.12709	0.00359	-0.10665	0.15096	0.13223	0.00097	0.02495
0.38976	0.25718	0.32133	0.18229	0.2053	0.14559	-0.17495	-0.20952	-0.20494	-0.03109	0.0655	0.13446	-0.02666	-0.16021	0.00951	0.16004	-0.03953	0.00951
0.402	0.49022	0.2856	0.15042	0.20103	0.1712	-0.31988	-0.32363	-0.33307	0.07517	0.04676	0.07043	-0.02004	-0.02842	0.19024	0.11494	-0.01225	0.01806
0.4064	0.42593	0.29986	0.15731	0.20063	0.14962	-0.17557	-0.16133	-0.41094	-0.02897	0.09906	0.14722	-0.00087	0.08848	0.28325	0.14753	-0.01227	0.07917
0.37634	0.46169	0.3294	0.16038	0.19343	0.10336	-0.28529	-0.14132	-0.32628	-0.01451	0.05065	0.14884	0.01034	-0.14229	-0.1034	0.15512	-0.07351	0.02273
0.38606	0.43298	0.32497	0.1681	0.20354	0.10148	-0.34056	-0.25619	-0.42777	0.01495	0.11653	0.10029	0.00244	-0.14255	-0.02325	0.14828	-0.07465	0.02661
0.39158	0.47613	0.3189	0.16253	0.19344	0.10908	-0.30099	-0.21804	-0.35814	-0.02865	0.0331	0.12146	-0.02616	-0.17479	0.01638	0.13343	-0.02551	-0.0062
0.40405	-0.24679	0.30182	0.16311	0.20984	0.08874	-0.14216	-0.18875	-0.09232	-0.02739	-0.03932	0.15063	0.00034	-0.03703	0.23164	0.06614	0.00581	0.0139
0.40311	0.418	0.28385	0.15811	0.19763	0.06585	-0.39635	-0.4089	-0.44741	0.01914	0.03223	0.11276	0.00888	-0.02968	-0.22477	0.173	0.02661	0.0276
0.41361	0.33556	0.24752	0.16433	0.22423	0.08422	-0.28746	-0.32169	-0.45005	0.08903	0.00335	0.19451	-0.04354	-0.02568	0.31795	0.06214	0.07778	0.02614
0.39984	0.25048	0.30688	0.16134	0.20475	0.0772	-0.32616	-0.29391	-0.369	0.01233	0.07757	0.18028	0.06013	-0.08078	-0.03664	0.19219	-0.05643	0.06398
0.39607	0.40109	0.31257	0.15732	0.20501	0.1033	-0.17561	-0.22448	-0.13899	0.02418	0.02729	0.15587	-0.00221	-0.09082	0.21138	0.15599	-0.01614	0.02563
0.3964	0.02466	0.25551	0.17027	0.20912	0.09432	-0.30877	-0.34069	-0.46432	0.08041	0.02229	0.18717	-0.08707	0.0322	0.32306	0.10513	0.06813	0.03555
0.39697	0.43597	0.30364	0.155	0.21281	0.09931	-0.44884	-0.35654	-0.4572	0.00613	0.03628	0.11351	-0.04879	-0.00663	0.27567	0.17605	-0.07098	0.02192
0.38339	0.18725	0.31856	0.16025	0.20618	0.14517	-0.39064	-0.27679	-0.4386	-0.0267	-0.0024	0.10711	-0.03745	-0.17215	0.31742	0.15453	-0.06914	-0.02279

ASES_OF	EASES_O	S_OF_MI	S_OF_MI	OF_PROG	_GROWT	ITION_OF	_TRANSM	_OF_DN	LUTION	DNA_DAM	AGE_REC	A_DAMA	MERE_ST	IBLE_STR	LE_STRA	_DNA_ME	ME_DNA	_DNA_RE	REPLICA
0.17102	0.06905	0.07471	-0.23192	0.32712	0.14652	0.0286	0.17136	0.12801	0.15982	0.04004	0.12754	-0.11739	0.29841	0.17624	0.28291	0.46221	0.10861	0.26375	0.0798
0.17526	0.05633	0.11247	-0.14292	0.3226	0.14626	0.01628	0.17096	0.23729	0.11375	0.0977	0.14153	-0.05549	0.28228	0.22682	0.30577	0.46262	0.15317	0.2687	0.21719
0.19149	0.04261	0.25029	-0.16657	0.32495	0.15235	0.06612	0.1749	0.2053	0.14505	0.09705	0.10084	-0.17552	0.29952	0.21329	0.3049	0.46201	0.12574	0.26845	0.26352
0.21729	0.05063	0.07707	-0.20222	0.32164	0.1504	0.05913	0.1628	0.15981	0.23981	0.08205	0.07669	-0.20873	0.2952	0.19323	0.28786	0.46169	0.10491	0.26474	0.11221
0.22149	0.04836	-0.00131	-0.24188	0.31705	0.16512	-0.01422	0.18469	0.20172	0.18101	0.06828	0.13655	-0.16136	0.28189	0.1976	0.28745	0.46003	0.11705	0.27039	0.10569
0.18369	0.04796	0.11223	-0.15732	0.32259	0.145	0.05387	0.17564	0.20667	0.2059	0.11757	0.13512	-0.11878	0.28962	0.21961	0.30063	0.46404	0.14166	0.28206	0.12912
0.23219	0.06701	-0.0144	-0.19526	0.31813	0.1408	0.02652	0.16342	0.19157	0.23338	0.09773	0.13772	-0.18612	0.3065	0.20279	0.29128	0.47286	0.12935	0.26539	0.20962
0.22797	0.06284	-0.10901	-0.21842	0.3194	0.15298	0.04003	0.17014	0.17822	0.30923	0.04994	0.0852	-0.20188	0.27744	0.16921	0.26976	0.45637	0.08317	0.25421	-0.03541
0.22461	0.05782	-0.02146	-0.25207	0.32017	0.15305	0.01657	0.17108	0.12627	0.26125	0.04922	0.06809	-0.12583	0.27443	0.17382	0.2739	0.46003	0.09037	0.25619	0.03875
0.22017	0.0358	0.03801	-0.24383	0.31465	0.15574	0.05928	0.15832	0.19779	0.22805	0.08675	0.10554	-0.1665	0.29749	0.19218	0.28443	0.46199	0.1136	0.25321	0.14814
0.17825	0.03882	0.1694	-0.12168	0.32979	0.13406	0.03156	0.15954	0.15545	0.17883	0.08083	0.15195	-0.08998	0.28846	0.21976	0.31229	0.46862	0.13293	0.28287	0.19218
0.1716	0.05427	0.16256	-0.22558	0.33088	0.14364	0.05294	0.1703	0.24692	0.26024	0.0939	0.12024	-0.07923	0.29902	0.20893	0.30186	0.46844	0.1247	0.27572	0.18143
0.16717	0.03371	0.24071	-0.14825	0.3324	0.14787	-0.12682	0.17733	0.17007	0.17548	0.10921	0.16249	-0.18634	0.2747	0.23118	0.31805	0.45857	0.14678	0.28017	0.13951
0.21054	0.06469	0.03878	-0.20979	0.32601	0.14675	0.01456	0.18012	0.23574	0.20117	0.05639	0.10903	-0.11414	0.2881	0.18179	0.28334	0.46615	0.10882	0.24845	0.25735
0.18905	0.05837	0.00967	-0.17038	0.32005	0.14116	0.04977	0.16611	0.2027	0.21668	0.07048	0.11087	-0.10346	0.29638	0.19887	0.28726	0.46252	0.117	0.26816	0.20909
0.13036	0.05395	0.18816	-0.17096	0.32128	0.13674	0.08212	0.17122	0.26627	0.14741	0.11346	0.12981	-0.04518	0.28289	0.22074	0.31245	0.45808	0.14472	0.27531	0.28712
0.19303	0.06246	-0.01558	-0.17942	0.33154	0.14768	0.03253	0.1634	0.20722	0.25654	0.02012	0.09021	-0.17002	0.31389	0.18124	0.29198	0.46475	0.09621	0.2359	-0.00934
0.22269	0.04483	-0.11899	-0.25	0.31783	0.14918	0.03443	0.18113	0.16504	0.16899	0.05057	0.11657	-0.18497	0.29039	0.17904	0.28877	0.45811	0.09915	0.25593	0.02906

EPLICATI	A_STRAN	RANCE_F	UROTRAN	ION_OF_	ULATION	ULATION	SMAD2_3	_OF_TGF	ING_EVE	SIGNALI	SIGNALI	SIGNALI	SIGNALI	REAM_SIG	ME_DRU	OSCAM_I	AL_INCISI	AL_INCISI	_OF_PRE
0.29986	0.03605	0.33363	0.14436	0.06551	0.01645	0.07673	0.11729	0.11773	0.22968	0.0175	0.0175	0.0175	0.0175	0.03673	0.04963	0.31377	-0.01537	-0.0056	0.099
0.29861	0.02117	0.21437	0.1323	0.023	-0.01516	0.07755	0.14197	0.13269	0.23059	0.02455	0.02455	0.02455	0.02455	0.03894	0.00898	0.18149	0.05605	0.06008	0.18337
0.29395	0.1171	0.31319	0.11224	0.0908	0.04396	0.10164	0.14685	0.14764	0.20981	0.02956	0.02956	0.02956	0.02956	0.02396	0.07098	0.17502	0.04598	0.02144	0.24414
0.29459	0.07131	0.35758	0.04248	0.07756	0.03296	0.12558	0.11559	0.14083	0.22201	0.03674	0.03674	0.03674	0.03674	0.04667	0.10448	0.21543	0.0253	0.00967	0.02308
0.30455	0.047	0.33539	0.0289	0.04451	0.00182	0.11869	0.13041	0.15189	0.26198	0.04349	0.04349	0.04349	0.04349	0.06345	0.10603	0.22127	0.02443	0.01191	0.11626
0.30921	0.11606	-0.03259	-0.00859	-0.00223	-0.01435	0.05109	0.09239	0.16421	0.2331	0.0117	0.0117	0.0117	0.0117	0.03951	0.10795	0.17213	0.06904	0.04418	0.10164
0.29465	0.06134	0.28149	0.04961	0.06973	0.02741	0.09629	0.13939	0.1602	0.22174	0.00919	0.00919	0.00919	0.00919	0.03948	0.08701	0.20505	0.04592	0.02163	0.17738
0.29119	-0.03284	0.37115	-0.02929	0.05189	0.01763	0.12226	0.13874	0.13485	0.24532	0.01708	0.01708	0.01708	0.01708	0.07787	0.1318	0.1961	-0.02006	-0.03945	-0.23657
0.29277	-0.00925	0.36586	0.01324	0.04392	0.00954	0.13227	0.13477	0.14644	0.24408	0.01324	0.01324	0.01324	0.01324	0.07808	0.11864	0.20975	-0.03224	-0.03664	-0.0805
0.28634	-0.00749	0.33785	-0.01894	0.01671	0.00784	0.1157	0.14658	0.13143	0.24247	-0.01193	-0.01193	-0.01193	-0.01193	0.05682	0.11657	0.23416	0.02619	0.01396	-0.2038
0.30844	0.13133	0.24185	0.08502	0.0588	0.0687	0.08949	0.13895	0.19643	0.21395	-0.02348	-0.02348	-0.02348	-0.02348	0.0284	0.08809	0.16098	0.0447	0.00836	0.22459
0.30393	0.10962	0.28959	0.04415	0.07566	0.05665	0.0591	0.12835	0.15757	0.21955	-0.08194	-0.08194	-0.08194	-0.08194	-0.00986	0.12828	0.21176	0.05393	0.00955	0.16488
0.30689	0.11713	0.32993	0.18121	0.09917	0.06996	0.06535	0.1343	0.17606	0.22121	0.07556	0.07556	0.07556	0.07556	0.02408	0.05676	0.14634	0.06782	0.03484	0.25912
0.28308	-0.07476	0.35388	0.07675	0.05128	0.04166	0.10477	0.12091	0.17354	0.22914	-0.01507	-0.01507	-0.01507	-0.01507	0.0355	0.11068	0.19924	-0.00801	-0.02066	-0.2459
0.29849	0.0828	0.29681	0.05594	-0.00608	-0.0114	0.08297	0.13108	0.13463	0.22348	-0.02778	-0.02778	-0.02778	-0.02778	0.00147	0.11748	0.19045	0.01028	-0.00189	-0.02026
0.30085	0.12745	0.32112	0.10075	0.15181	0.0992	0.04675	0.1294	0.17562	0.20651	-0.05023	-0.05023	-0.05023	-0.05023	0.00834	0.09099	0.19151	0.04465	0.02612	0.3264
0.27218	-0.11661	0.30104	0.02747	0.10679	0.03531	0.09794	0.11593	0.13727	0.21548	0.06119	0.06119	0.06119	0.06119	0.05545	0.08268	0.15744	-0.03549	-0.03126	-0.04069
0.29358	-0.02248	0.39299	0.05338	0.06232	0.01069	0.11042	0.13277	0.13169	0.24544	0.0381	0.0381	0.0381	0.0381	0.06855	0.11358	0.19807	-0.0133	-0.02506	-0.0347

REGULA	TASES_UBI	PHASE_	ORS_COV	ECM_PRO	TS_OF_R	FR_DOW	TS_WITH	RANSACT	D_INITIA	ME_EICOS	STIC_FIB	N_OF_CY	NDOGEN	COMPLEX	OMAL_V	AT_REGUL	_ENOS	ATED_GR
0.1733	0.14961	0.25063	0.09243	-0.09493	0.06312	0.11065	-0.21338	0.06445	-0.08139	-0.21502	-0.06089	0.19634	-0.04102	0.09253	0.24375	0.01262	0.24217	0.21396
0.16059	0.15552	0.25673	0.06158	-0.04611	0.014	0.06679	-0.14272	0.07289	0.09344	-0.1867	-0.07894	0.11836	-0.17571	0.01406	0.21241	0.0075	0.2018	0.19729
0.22623	0.14309	0.25013	-0.00337	-0.0483	-0.01372	0.14253	-0.36512	0.03774	0.08939	-0.02041	0.00413	0.02433	-0.08445	0.03311	0.23894	0.01019	0.26961	0.22528
0.16463	0.14118	0.20073	0.0687	-0.02579	0.0823	0.12205	-0.2928	0.06171	0.01993	-0.03992	0.03067	0.19082	-0.08239	0.06456	0.29015	0.05965	0.15782	0.21343
0.17988	0.13398	0.21363	0.06432	-0.07422	0.06894	0.14288	-0.29325	0.1127	0.0657	-0.18671	0.00765	0.13494	-0.12766	0.05149	0.29359	0.02922	0.15972	0.18699
0.18527	0.15585	0.26494	-0.01115	-0.01945	0.06605	0.09404	-0.08745	0.00252	0.04964	-0.19812	0.08258	0.06351	-0.17968	-0.01604	0.25742	0.05834	0.25022	0.15843
0.18894	0.13281	0.24179	-0.00144	0.09009	0.02815	0.0924	-0.29587	0.01362	0.03225	-0.22763	0.14185	0.10455	-0.21353	-0.05677	0.24447	0.0146	0.23531	0.21863
0.10119	0.14162	0.18298	0.01707	-0.00915	0.03429	0.15928	-0.33101	0.03097	0.00948	0.01825	0.06989	0.12611	0.01383	0.07891	0.30345	0.07428	0.14918	0.20418
0.14189	0.13366	0.21009	0.06223	0.02538	0.00931	0.15724	-0.26775	0.0067	-0.01209	0.00863	0.06931	0.10203	-0.06973	0.05989	0.3285	0.06923	0.17311	0.22444
0.09311	0.12478	0.22453	0.03694	-0.05754	0.09062	0.14227	-0.32037	0.02532	-0.01122	-0.09616	0.06082	0.13827	-0.06709	0.03482	0.33865	0.0608	0.18043	0.23296
0.2245	0.156	0.26067	0.01525	-0.08517	-0.06965	0.07457	-0.05519	0.0511	-0.02684	-0.15855	-0.01329	0.05204	-0.25006	-0.00915	0.13842	-0.00091	0.23869	0.23648
0.1921	0.13992	0.23435	0.02034	-0.03848	0.06496	0.09217	-0.19453	0.00794	0.03337	-0.29487	0.08414	0.13765	-0.14883	0.00797	0.28177	-0.01673	0.245	0.2023
0.26499	0.16076	0.23404	0.09377	-0.02219	-0.028	0.0831	-0.29997	0.07974	0.06138	-0.33404	0.03499	0.08764	-0.31886	0.07357	0.12235	0.0602	0.21364	0.28429
0.10898	0.12843	0.21436	0.03914	-0.0676	0.04836	0.10865	-0.3969	0.07393	-0.0294	0.02156	0.00533	0.14206	-0.07629	0.0145	0.21414	0.02291	0.19015	0.2
0.15398	0.13733	0.2333	0.03955	-0.05713	-0.0192	0.08212	-0.29333	-0.00127	0.06129	0.00426	0.00044	0.04045	-0.0972	0.03861	0.20516	0.03301	0.24226	0.21696
0.24587	0.15342	0.24363	0.10558	-0.04976	-0.10768	0.09574	-0.08753	0.09478	0.14543	-0.28576	-0.08988	0.00145	-0.19811	0.05425	0.20064	-0.00165	0.27362	0.23631
0.1788	0.12306	0.21017	0.06229	-0.03468	0.05762	0.12945	-0.36982	0.09682	0.04474	-0.05762	-0.03347	0.1383	-0.08377	0.07723	0.21256	0.03404	0.12296	0.21457
0.1228	0.13532	0.22102	0.02101	-0.0345	0.03622	0.14456	-0.29401	0.05388	0.02428	-0.05558	0.00954	0.18638	-0.04468	0.04096	0.28867	0.03773	0.23894	0.22473

DIATED_F	EPHRIN_	MEDIATE	PH_EPHR	EGULATI
0.24088	0.08796	0.16222	0.22098	0.21231
0.20261	0.10168	0.16821	0.20156	0.23749
0.24295	0.1295	0.1877	0.23798	0.21828
0.2323	0.11782	0.20945	0.23239	0.18288
0.22683	0.15126	0.17633	0.22143	0.19848
0.20777	0.06151	0.1264	0.1842	0.21748
0.24489	0.08719	0.16585	0.22086	0.22212
0.24257	0.15404	0.2113	0.23875	0.16091
0.2418	0.16865	0.19862	0.23945	0.17408
0.231	0.13411	0.20174	0.22944	0.19133
0.22969	0.06872	0.16139	0.21644	0.2244
0.24124	0.0516	0.15166	0.21558	0.21148
0.24519	0.07794	0.20067	0.2541	0.23452
0.23323	0.09977	0.18295	0.22701	0.20829
0.2331	0.10681	0.1554	0.21947	0.20414
0.22505	0.06428	0.15731	0.22135	0.21424
0.24088	0.09797	0.19557	0.23499	0.20884
0.23741	0.13911	0.19137	0.2288	0.18161

RKS_ARE	ERK_MAP	P_CARBO	P_OXYGE	IVATES_P	IVATES_F	ROPOIET	ROPOIETI	_CONTRO	GI_ANTE	_MEDIAT	_OF_SIST	ROGEN_	DEPENDE	EVENTS_	LATED_S	ETHANOL	IC_TRANS	TIC_TRA	RIBONUC	ENSION_(
0.23068	0.18753	0.34571	0.37473	-0.10085	-0.08687	0.06389	-0.04351	0.074	0.16579	0.20664	-0.01306	0.30809	0.23829	0.07899	0.13719	0.04531	0.40538	0.37034	0.10265	0.00372
0.19238	0.13447	0.31868	0.38388	-0.16786	-0.0754	0.04086	-0.14346	0.04558	0.15455	0.22104	0.0928	-0.1307	0.26599	0.04057	0.14913	0.11513	0.42051	0.38692	0.11318	0.08147
0.21593	0.18358	0.3595	0.40002	-0.03857	-0.10927	0.06848	-0.11721	0.10072	0.17119	0.22342	0.09447	-0.30985	0.26364	0.09154	0.13441	0.08599	0.40667	0.3721	0.14099	0.05798
0.22561	0.19027	0.30587	0.36993	0.08689	-0.01089	0.09258	-0.00097	0.11885	0.17417	0.21328	-0.00929	0.08843	0.23411	0.03765	0.11115	0.04698	0.42205	0.3837	0.06369	0.02419
0.20616	0.18004	0.37776	0.43112	0.00061	-0.01591	0.11568	-0.0056	0.11997	0.16278	0.21914	0.1013	0.06148	0.24771	0.0761	0.18071	0.04697	0.41678	0.38298	0.09155	0.02731
0.20765	0.16872	0.37467	0.44946	-0.12847	-0.09724	0.02146	-0.09355	0.08501	0.1576	0.20763	0.06981	-0.12965	0.25178	0.04057	0.0721	0.06503	0.42286	0.38405	0.09705	0.07699
0.16988	0.15046	0.32916	0.34745	0.02171	-0.15005	0.04773	-0.10492	-0.00293	0.14464	0.22594	0.07884	-0.22618	0.26275	0.08481	0.09207	-0.01823	0.42131	0.37706	0.09204	0.06798
0.22052	0.19518	0.35674	0.41504	0.09186	-0.00925	0.10362	0.01723	0.15037	0.19196	0.19981	-0.00488	0.07645	0.22124	0.05253	0.09126	0.10153	0.41782	0.37937	0.03022	-0.02104
0.22303	0.19423	0.33972	0.39811	0.12457	0.02491	0.09273	0.02857	0.13149	0.17989	0.20024	-0.02585	0.06694	0.2236	0.04415	0.06784	0.09069	0.41216	0.3743	0.04574	-0.01851
0.22988	0.21244	0.32216	0.3883	0.04037	0.00305	0.10015	0.0645	0.0932	0.16255	0.21141	0.02171	-0.0458	0.23509	0.07912	0.08933	0.07181	0.40822	0.36845	0.06245	0.02513
0.15579	0.11599	0.26267	0.31059	-0.12929	-0.19435	-0.03443	-0.27814	0.05199	0.16733	0.21468	0.07258	0.28624	0.24987	0.10494	0.06953	0.02509	0.42079	0.37948	0.08337	0.0749
0.2217	0.18622	0.35371	0.40041	-0.13319	-0.12707	-0.00473	-0.11621	0.0786	0.16995	0.21127	0.06589	-0.01656	0.24696	0.10574	0.06499	0.02104	0.42109	0.38753	0.11155	0.07331
0.27531	0.22155	0.36126	0.38685	-0.27094	-0.30247	0.0593	-0.30691	0.04082	0.17108	0.22634	0.05871	-0.43016	0.25737	0.09778	0.18072	-0.08878	0.41145	0.37962	0.12483	0.09857
0.22833	0.18221	0.35882	0.3852	-0.08936	-0.1011	0.09384	-0.11236	0.07375	0.18288	0.20608	0.06093	0.10833	0.24137	0.05211	0.13811	0.07927	0.42231	0.3843	0.11717	0.01187
0.20474	0.18158	0.32615	0.37551	-0.07981	-0.03517	0.07205	-0.05892	0.08759	0.17359	0.19771	0.03324	0.0414	0.23633	0.05141	0.08056	0.15828	0.42842	0.39349	0.07151	0.04082
0.2134	0.13998	0.26456	0.31748	-0.34855	-0.31135	-0.02311	-0.289	0.0733	0.15353	0.21121	0.09458	-0.24132	0.2459	0.12871	0.12643	0.01505	0.42082	0.3858	0.10492	0.08876
0.18995	0.14714	0.3361	0.38413	0.05793	-0.0676	0.10464	-0.07979	0.08382	0.17528	0.22315	0.00787	0.1374	0.24262	0.06215	0.16577	0.01904	0.42407	0.38865	0.08922	-0.00631
0.21248	0.174	0.34512	0.41027	0.08552	-0.04237	0.09889	-0.02137	0.11386	0.17306	0.20837	0.00717	0.12524	0.23258	0.08069	0.11533	0.14731	0.40572	0.38656	0.06726	0.00325

LULAR_M	CLEAR_E	HWAY_OF	YOCYTE_	CONI_AN	ASL_CD95	_PROCES	ME_FATT	GPR40_F	RTTY_ACID	ACYL_C	ANTS_AN
0.07317	0.14753	-0.34384	0.16269	0.06328	-0.25428	-0.28573	-0.46009	0.09151	0.06582	0.06694	0.17905
0.0565	0.13635	-0.26813	0.17405	0.11749	-0.25409	-0.1935	-0.45521	0.03273	0.04053	0.00562	0.14323
0.07354	0.14451	-0.13044	0.1554	0.02412	-0.18853	-0.10237	-0.37429	0.14456	0.04827	0.00648	0.15835
0.09842	0.17537	-0.15022	0.17142	0.04755	-0.17391	-0.26455	-0.46085	0.17081	0.05649	0.02893	0.16375
0.07319	0.16183	-0.14386	0.17607	0.09752	-0.14766	-0.34562	-0.41838	0.09118	0.04073	0.02849	0.1526
0.09252	0.11613	-0.03942	0.17729	0.10506	-0.14032	-0.29185	-0.39571	0.01299	0.04142	-0.01625	0.19774
0.14266	0.15913	-0.02605	0.14639	0.06212	-0.21458	-0.10417	-0.38887	0.06146	0.05572	0.01732	0.17039
0.1157	0.16159	-0.06591	0.17478	0.07326	-0.23535	-0.30802	-0.41732	0.14631	0.04079	0.01982	0.10793
0.11518	0.15688	-0.01881	0.1743	0.03259	-0.17599	-0.2871	-0.45598	0.11164	0.04433	0.03589	0.03281
0.09192	0.16769	-0.00191	0.1744	0.07609	-0.15427	-0.35109	-0.40496	0.11253	0.04357	0.04162	0.08803
0.06694	0.14719	-0.22113	0.16229	0.01306	-0.16047	0.13043	-0.23873	0.10729	0.04198	0.00775	0.24105
0.09666	0.1412	0.03094	0.15963	0.04516	-0.19318	-0.10357	-0.407	0.13735	0.04546	0.02352	0.13763
0.06107	0.16231	-0.19631	0.19486	0.09751	-0.12787	-0.14327	-0.32377	0.08789	0.02191	0.00538	0.20811
0.07053	0.13863	-0.27743	0.17044	0.0093	-0.25002	-0.21415	-0.46077	0.09626	0.07444	0.04842	0.1612
0.07983	0.12391	-0.10789	0.16231	0.01049	-0.23164	-0.28205	-0.21724	0.04863	0.04744	0.00046	0.07371
0.03791	0.14576	-0.20481	0.16495	0.00701	-0.17784	0.01963	-0.42272	0.11967	0.05892	0.02404	0.16846
0.07996	0.18943	-0.24361	0.1802	0.04554	-0.24203	-0.21418	-0.28844	0.14011	0.05739	0.02276	0.1285
0.07642	0.1602	-0.17091	0.18336	0.02999	-0.21799	-0.2277	-0.45026	0.06874	0.04983	0.02825	0.12955

EDIATED_	MEDIATED	EDIATED_	OR_FCGR	R_MEDIATE	FCGR_AN	RECEP	ME_FERTI	GAND_BIN	AND_BIN	AND_BIN	UTANT_RE	2_ALTER	UTANT_R	AND_BIN	ECTIN_M	OHYDRATE	FLT3_	SIGNALING
0.05893	0.09613	0.25218	0.17611	0.10618	-0.01408	0.19793	0.10604	0.25823	0.25823	0.25823	0.03248	0.13669	-0.01977	-0.26193	0.14498	-0.03312	-0.046	0.22923
0.01611	0.06583	0.25085	0.13901	0.10496	-0.02714	0.18306	0.07741	-0.05232	-0.05232	-0.05232	-0.03069	0.15242	0.03411	-0.01392	0.13385	-0.16857	-0.0746	0.24426
0.03607	0.10061	0.2285	0.16277	0.08368	0.02043	0.17642	0.01395	0.28695	0.28695	0.28695	0.01248	0.15451	0.01503	-0.05919	0.13951	-0.25823	-0.00098	0.31393
0.08686	0.12181	0.24339	0.20835	0.17146	0.16571	0.1938	0.07252	0.23833	0.23833	0.23833	0.05324	0.09903	-0.04954	-0.26968	0.21083	0.06729	0.0384	0.26119
0.1193	0.14857	0.26758	0.17746	0.14246	0.05548	0.22196	0.10166	0.09864	0.09864	0.09864	0.06641	0.11695	-0.02439	-0.44707	0.17639	0.18478	0.05918	0.31426
0.03985	0.0481	0.241	0.14709	0.0711	-0.09234	0.17719	0.07712	0.11767	0.11767	0.11767	0.00215	0.11726	-0.01647	-0.21332	0.14816	-0.29311	-0.00272	0.25019
0.04894	0.08626	0.23412	0.18389	0.10096	0.02029	0.1836	0.07005	0.10499	0.10499	0.10499	0.00355	0.1439	0.01112	-0.16946	0.22547	-0.1529	0.02743	0.26366
0.09449	0.13313	0.26539	0.21287	0.17569	0.17545	0.21142	0.06758	0.00264	0.00264	0.00264	0.07556	0.06244	-0.08064	-0.44583	0.21716	0.11996	0.04126	0.3002
0.10416	0.13861	0.26334	0.20994	0.16456	0.15205	0.21085	0.07776	0.13405	0.13405	0.13405	0.07197	0.07082	-0.05267	-0.34032	0.14406	0.13652	0.05506	0.33521
0.11946	0.14766	0.25929	0.21077	0.18503	0.16843	0.20941	0.13125	-0.14127	-0.14127	-0.14127	0.05421	0.09145	-0.03506	-0.19975	0.20285	0.14832	0.06288	0.30839
-0.0125	0.02564	0.22839	0.13105	0.0376	-0.1335	0.17024	0.04867	0.23534	0.23534	0.23534	-0.00593	0.14118	-0.03111	-0.05989	0.20853	-0.20416	-0.03594	0.27959
0.00886	0.03561	0.23751	0.14794	0.07429	-0.13107	0.17639	0.04341	0.23199	0.23199	0.23199	0.0202	0.12014	-0.03414	0.2563	0.10003	-0.15413	-0.04064	0.28723
-0.06532	0.01515	0.22543	0.14662	-0.00628	-0.26822	0.1654	0.08204	0.2607	0.2607	0.2607	-0.01423	0.13251	0.00135	-0.11326	0.06892	-0.22301	-0.09117	0.24245
0.05019	0.09924	0.24657	0.17917	0.1141	0.03512	0.18923	0.1144	0.19253	0.19253	0.19253	0.01369	0.12229	-0.03623	0.10129	0.23935	-0.02766	-0.01117	0.23192
0.02231	0.07728	0.24265	0.15785	0.09816	-0.01344	0.18231	0.02828	0.22459	0.22459	0.22459	0.00895	0.12358	-0.03149	-0.20346	0.22909	-0.01022	-0.06191	0.24703
-0.08301	0.00724	0.21648	0.1245	-0.01938	-0.246	0.15364	-0.01775	0.26123	0.26123	0.26123	-0.04025	0.15058	-0.01272	-0.09759	0.07728	-0.38612	-0.07085	0.21491
0.03728	0.09749	0.23351	0.19268	0.11518	0.05256	0.18102	0.08961	0.13387	0.13387	0.13387	0.03788	0.14035	-0.02419	-0.16294	0.18583	0.02995	0.01993	0.22589
0.09902	0.12202	0.25956	0.1998	0.14741	0.10129	0.21144	0.0902	0.06007	0.06007	0.06007	0.07269	0.0967	-0.04669	-0.40074	0.11757	-0.00846	0.0491	0.31273

3_SIGNAL	IG_THROU	G_OF_A	MATION_C	ATP_BY	TION_OF	N_OF_DE	FIBRIN_F	INCISI	G_OF_INT	ON_OF_P	RNA_POL	E_ASSO	TC_NER	F_THE_A	A_CATEN	OF_THE_	THE_EAR	F_THE_PC	IN_FOLD	ONTAININ	N_OF_XYL	IND_FOR	MEDIATE
0.01794	-0.18235	0.41528	0.12051	0.33558	0.09267	0.07031	0.08157	0.15759	-0.1536	0.28377	-0.01815	0.27604	0.04069	-0.418	0.36758	0.23249	-0.06416	-0.418	0.31024	0.02312	0.13451	0.01786	0.10448
0.015	-0.25604	0.40524	0.09019	0.2968	0.13202	0.18247	0.08929	0.19309	0.10921	0.28996	0.05612	0.19907	0.05785	-0.38577	0.37245	0.20475	0.04427	-0.38577	0.32207	0.06535	0.09075	-0.02639	0.07673
0.06396	-0.13629	0.41386	0.10814	0.32308	0.1782	0.18765	0.16303	0.12615	-0.01638	0.2682	0.00931	0.24252	0.00863	-0.34103	0.37054	0.16954	-0.04818	-0.34103	0.32524	0.01168	0.16616	0.00044	0.08133
0.07306	-0.00391	0.40137	0.10003	0.29825	0.08025	0.07066	0.20316	0.12152	-0.43121	0.27011	-0.03377	0.24626	0.00214	-0.09477	0.36559	0.2022	-0.09855	-0.09477	0.34147	-0.05788	0.14648	0.05587	0.08267
0.08379	0.04771	0.41577	0.08437	0.28632	0.17743	-0.01653	0.15916	0.15478	-0.26775	0.30428	-0.01412	0.21067	0.04503	-0.27286	0.36165	0.13428	-0.05562	-0.27286	0.32935	0.03503	0.15532	0.00896	0.08834
0.05194	-0.06428	0.39822	-0.0345	0.28614	0.11834	0.09277	0.24991	0.18162	-0.02167	0.28825	0.01206	0.21626	0.04209	-0.3028	0.35738	0.1751	-0.05018	-0.3028	0.30456	0.01867	0.1059	0.02624	0.07963
0.07114	0.01952	0.39963	0.076	0.30415	0.09891	0.14809	0.2404	0.1562	-0.22072	0.26743	0.00201	0.21083	0.0388	-0.39299	0.36644	0.12405	-0.06393	-0.39299	0.31467	0.03232	0.11327	0.15653	0.07661
0.08548	0.01325	0.39185	0.09639	0.29891	0.01061	0.02379	0.24125	0.11586	-0.44125	0.29075	-0.06498	0.20984	-0.00914	-0.20539	0.35047	0.10383	-0.12513	-0.20539	0.34167	-0.05916	0.16628	0.0829	0.07549
0.08108	0.07443	0.40907	0.09508	0.27484	0.00844	0.02404	0.24263	0.10434	-0.22723	0.2943	-0.04845	0.18187	-0.02095	-0.08702	0.35657	0.16174	-0.10271	-0.08702	0.34493	-0.0343	0.13209	0.06258	0.06237
0.08366	0.08748	0.39822	0.08712	0.30851	-0.12371	-0.03425	0.23614	0.14603	-0.22635	0.28119	-0.0257	0.2292	0.02493	-0.08702	0.36375	0.13289	-0.0753	-0.08702	0.33337	-0.00506	0.10778	0.10227	0.09986
0.03892	-0.19405	0.43377	0.08582	0.30639	0.23115	0.21223	0.13777	0.12995	-0.05496	0.2735	-0.00954	0.17217	0.03828	-0.46186	0.37279	0.19971	-0.08497	-0.46186	0.36608	0.03997	0.06791	0.11858	0.12199
0.0439	-0.14932	0.42338	0.08814	0.26283	0.08122	0.05147	0.22215	0.14865	-0.24361	0.28046	-0.04194	0.22793	0.00283	-0.44971	0.35985	0.14664	-0.09959	-0.44971	0.33662	0.02168	0.11402	0.12198	0.09197
0.03527	-0.38918	0.42373	0.07527	0.3383	0.23742	0.19292	0.1211	0.19064	0.03664	0.28329	0.0226	0.14548	0.04057	-0.43562	0.3683	0.20389	-0.03092	-0.43562	0.37632	0.0229	0.01904	0.06434	0.09875
0.03191	-0.06824	0.41592	0.07562	0.30882	0.12437	0.06814	0.19704	0.11803	-0.35388	0.27713	-0.01583	0.2026	0.00863	-0.12965	0.36615	0.17778	-0.07513	-0.12965	0.37147	0.00368	0.15672	0.09935	0.10857
0.01779	-0.20419	0.43163	0.11963	0.29102	0.19374	0.09591	0.22278	0.12328	0.05549	0.28283	-0.00984	0.21903	0.0086	-0.3685	0.36195	0.18899	-0.05469	-0.3685	0.3402	0.02527	0.19624	0.00313	0.11295
0.00314	-0.42122	0.44305	0.10313	0.32562	0.28202	0.16441	0.05511	0.13316	0.08643	0.26954	0.00094	0.1464	0.02951	-0.45411	0.35008	0.20331	-0.04347	-0.45411	0.35968	-0.01691	0.16186	-0.09163	0.11215
0.06451	-0.07063	0.40445	0.06713	0.36826	0.05607	0.08966	0.17055	0.12694	0.1684	0.26777	-0.02458	0.29589	-0.0024	-0.21666	0.36316	0.1803	-0.07666	-0.21666	0.35424	0.00733	0.14438	0.12118	0.07836
0.08451	0.03635	0.39166	0.1114	0.31499	0.11073	0.07227	0.1552	0.14185	-0.18531	0.29474	-0.04792	0.218	0.01358	-0.30069	0.35978	0.22601	-0.08948	-0.30069	0.35103	-0.00164	0.15464	0.06012	0.04098

RANSCRI	RANSCRI	_OXIDAT	IDS_REG	EDIATED_	EDIATED_	EDIATED_	EDIATED_	RUCTOSE	RUCTOSE	_G0_AND	NA_DAM	SPECIFIC	G2_M_CH	DNA_DAM	A_REPLI	OME_G2_	GAB1_SI	B_RECE
-0.08516	-0.1107	0.10882	0.06363	0.10011	0.10011	0.10011	0.10011	0.03647	0.18012	0.04698	0.26313	0.05285	0.26861	0.24742	0.099	-0.27074	0.07674	0.2226
-0.09184	-0.02629	0.07711	0.02476	0.10872	0.10872	0.10872	0.10872	0.02416	0.19799	0.16759	0.27033	0.17273	0.26711	0.26821	0.18337	-0.04967	0.03376	0.22816
-0.12724	-0.03617	0.08036	0.10898	0.09276	0.09276	0.09276	0.09276	0.25891	0.30705	0.13061	0.24801	0.12894	0.26512	0.25036	0.24414	-0.22935	0.09286	0.19268
-0.22548	-0.11562	0.0625	0.1659	0.08769	0.08769	0.08769	0.08769	0.14654	0.22756	0.06269	0.24807	0.12153	0.26466	0.252	0.02308	-0.24291	0.16036	0.32706
-0.14593	-0.08564	0.11251	0.09363	0.1392	0.1392	0.1392	0.1392	0.2544	0.3214	0.06634	0.28273	0.07906	0.27632	0.24681	0.11626	-0.24925	0.1034	0.26288
0.00701	-0.13043	0.04735	0.03124	0.05547	0.05547	0.05547	0.05547	0.23409	0.29268	0.12569	0.26789	0.15909	0.27472	0.24864	0.10164	-0.15589	0.07104	0.20035
-0.01543	-0.1902	0.0577	0.09666	0.03553	0.03553	0.03553	0.03553	-0.08647	0.14223	0.08986	0.2495	0.14127	0.25768	0.24218	0.17738	-0.05848	0.10397	0.22778
-0.15418	-0.18014	0.05513	0.15144	0.07752	0.07752	0.07752	0.07752	0.27277	0.27179	0.00169	0.26969	0.015	0.25775	0.23981	-0.23657	-0.21966	0.16494	0.28882
-0.07476	-0.15814	0.05974	0.13072	0.03472	0.03472	0.03472	0.03472	0.28072	0.29529	0.02202	0.27401	0.0708	0.25763	0.22635	-0.0805	-0.29241	0.15154	0.27035
-0.06866	-0.05286	0.08459	0.12974	0.0679	0.0679	0.0679	0.0679	0.25392	0.29105	0.02141	0.26711	0.01985	0.25016	0.23495	-0.2038	-0.24819	0.13116	0.29444
-0.06686	-0.03364	0.13816	0.10023	-0.01301	-0.01301	-0.01301	-0.01301	0.05549	0.1965	0.19819	0.26071	0.15479	0.28543	0.26972	0.22459	0.1566	0.09637	0.19079
-0.146	-0.16079	0.06601	0.06504	-0.00182	-0.00182	-0.00182	-0.00182	0.03633	0.14253	0.10177	0.26572	0.15286	0.28333	0.27234	0.16488	-0.29294	0.03845	0.22667
-0.09874	-0.08217	0.05174	0.08041	0.12061	0.12061	0.12061	0.12061	-0.0496	0.10095	0.1706	0.26692	0.16669	0.28912	0.28312	0.25912	0.08279	-0.02859	0.24093
-0.20134	-0.07986	0.12669	0.09639	0.08703	0.08703	0.08703	0.08703	0.25642	0.29189	0.02061	0.25204	0.04748	0.24409	0.23591	-0.2459	-0.14427	0.05693	0.21409
-0.11219	-0.03069	0.10539	0.01359	0.0492	0.0492	0.0492	0.0492	0.29491	0.32158	0.09198	0.25875	0.13104	0.27415	0.25702	-0.02026	-0.06253	0.03136	0.19796
-0.09231	-0.00207	0.07841	0.0482	0.05432	0.05432	0.05432	0.05432	0.08443	0.21858	0.12837	0.24076	0.18658	0.28201	0.27817	0.3264	-0.1987	0.02532	0.15376
-0.22375	-0.10689	0.07524	0.10288	0.11595	0.11595	0.11595	0.11595	0.1355	0.21521	0.01636	0.25245	0.05488	0.23829	0.24177	-0.04069	-0.30368	0.14004	0.32497
-0.20583	-0.21871	0.02069	0.06504	0.07759	0.07759	0.07759	0.07759	0.23723	0.29052	0.04244	0.27091	0.03763	0.26342	0.24029	-0.0347	-0.26035	0.1598	0.29302

R_RECEP	RELEASE_	ATION_HY	ORT_AND	AIR_SYN	P_JUNCT	JUNCTION	_TRAFF	NALLING	E_GASTR	_FUCOSE	_SECOND	JAK_STAT	NE_SILE	R_FORMA	UCLEOT	DE_1_GL	NALING_I
0.19981	0.23691	0.06412	-0.08876	-0.03191	0.19079	0.24853	0.22898	0.13572	0.23464	0.29556	-0.00245	0.3579	0.21831	-0.05359	0.07618	0.25293	0.21034
0.22426	0.24993	0.07155	-0.03959	0.08034	0.23174	0.27093	0.26433	0.1183	0.25112	0.24672	-0.03681	0.33526	0.22897	0.04153	0.12287	0.23999	0.22085
0.20774	0.2734	0.08059	0.04794	0.06667	0.23065	0.2747	0.25309	0.12607	0.22942	0.28208	-0.00106	0.35605	0.23383	0.00302	0.09011	0.18556	0.16166
0.30926	0.15537	0.09282	0.05741	0.02078	0.26577	0.29498	0.28693	0.11975	0.21894	0.1591	0.0539	0.35136	0.19558	-0.06283	0.05977	0.28985	0.27484
0.25216	0.22565	0.08106	0.00313	0.01544	0.23624	0.25169	0.24277	0.16265	0.24757	0.2052	0.15225	0.36284	0.20886	-0.07104	0.09375	0.25855	0.2418
0.19973	0.18606	0.08616	0.11395	0.06629	0.20271	0.24077	0.21932	0.05216	0.23983	0.272	0.09494	0.35022	0.21601	-0.04471	0.11318	0.21124	0.18525
0.24027	0.14969	0.09637	0.13838	0.03042	0.22459	0.23415	0.23581	0.09565	0.2193	0.21901	0.09726	0.3499	0.21997	0.01364	0.09803	0.23179	0.21357
0.27342	0.22615	0.08799	0.07323	-0.0332	0.26682	0.29096	0.28189	0.12623	0.2366	0.16834	0.08283	0.36512	0.18008	-0.11872	0.04226	0.26317	0.26259
0.2581	0.14019	0.1124	0.14292	-0.02951	0.2714	0.31938	0.2942	0.10535	0.23628	0.06901	0.1573	0.36309	0.19082	-0.08076	0.03913	0.24751	0.24577
0.27889	0.20206	0.08793	0.11429	0.01887	0.25743	0.26762	0.26411	0.12868	0.2243	0.1414	0.14731	0.36142	0.20028	-0.18993	0.07654	0.28117	0.27291
0.20164	0.23855	0.10992	0.01134	0.04677	0.29346	0.24916	0.27894	0.10037	0.23787	0.31671	-0.00095	0.34553	0.20986	0.06109	0.1102	0.19485	0.16674
0.21758	0.17773	0.12531	0.17013	0.04412	0.23785	0.26138	0.25889	0.11037	0.22984	0.29975	0.06593	0.36449	0.22295	-0.07484	0.09592	0.2577	0.22247
0.24429	0.18326	0.12207	0.04862	0.07571	0.32029	0.25823	0.28939	0.16084	0.25159	0.24596	-0.12833	0.33677	0.23028	0.05954	0.13416	0.24839	0.23198
0.21093	0.22547	0.11877	0.02708	-0.0176	0.2797	0.2819	0.28502	0.12325	0.22371	0.18114	0.07044	0.35522	0.22503	-0.04119	0.06119	0.22821	0.21148
0.1992	0.31518	0.10468	0.05771	0.02348	0.24391	0.27495	0.26273	0.11626	0.23901	0.24429	0.07024	0.35594	0.20261	-0.02582	0.08244	0.22876	0.2057
0.16758	0.2376	0.10885	0.01466	0.06023	0.2656	0.2266	0.25572	0.1182	0.23834	0.3299	-0.04242	0.35616	0.21909	0.03516	0.10307	0.1625	0.14108
0.31963	0.15205	0.06894	-0.04509	-0.04377	0.28887	0.29069	0.2955	0.12934	0.22572	0.10464	0.04089	0.36825	0.2099	-0.07006	0.04919	0.28804	0.28141
0.28759	0.21655	0.08445	0.00932	-0.05533	0.30151	0.26516	0.28751	0.12792	0.23918	0.22794	0.122	0.35789	0.2005	-0.061	0.05889	0.26898	0.25832

ON_TYPE_	CORTIC	GLUCON	LUCOSE_	GLUCUR	AND_GLU	ROTRAN	TATHION	NE_SYNT	PHOSPH	PHOSPH	BREAKD	LYCOGEN	OGEN_ST	LYCOGE	ME_GLY	AMINOGL	PHINGOL	SPHINGO
0.20656	-0.42928	0.28724	0.1924	0.2897	0.08297	0.03686	0.16735	0.22469	0.06654	-0.01498	0.21062	0.22373	0.1767	0.2811	0.1907	0.01229	0.26477	0.0544
0.20437	-0.41712	0.27472	0.18083	0.21796	0.12073	-0.01879	0.10468	0.21755	0.04612	-0.06795	0.12553	0.1373	0.10585	0.19884	0.18439	0.01229	0.12272	0.04055
0.16555	-0.24185	0.26244	0.19291	0.36948	0.16814	0.07515	0.15476	0.25703	0.06773	-0.1607	0.17871	0.18709	0.1377	0.21884	0.19694	0.02023	0.22856	0.04165
0.30698	-0.29417	0.27975	0.17908	0.19298	0.07371	0.05836	0.14085	0.19698	0.10388	0.07416	0.15748	0.17855	0.15485	0.24839	0.17542	0.04644	0.25667	0.14985
0.23199	-0.35107	0.27559	0.18901	0.18103	0.08235	0.03769	0.13704	0.18896	0.0739	-0.09319	0.17007	0.18698	0.16431	0.24835	0.19023	0.0494	0.23638	0.10098
0.16641	0.16118	0.24158	0.17094	0.26278	0.18539	0.03602	0.14971	0.28244	0.05727	-0.18092	0.14427	0.16723	0.09468	0.25915	0.18377	7.5E-05	0.31385	0.08139
0.20534	-0.04527	0.2804	0.17388	0.20221	0.20868	-0.02417	0.13462	0.21568	0.03537	-0.08932	0.15913	0.16396	0.1374	0.21271	0.16293	0.05696	0.27614	0.04218
0.26356	-0.04104	0.25775	0.15826	0.29156	0.14896	0.04193	0.13147	0.16318	0.06616	0.00946	0.18437	0.20689	0.18386	0.28038	0.15165	0.05428	0.25535	0.14653
0.24924	0.09917	0.25831	0.17217	0.20783	0.10968	0.04229	0.15705	0.23458	0.05675	-0.01026	0.17819	0.18756	0.13407	0.2299	0.17223	0.06567	0.23466	0.18923
0.27715	-0.1566	0.25323	0.16371	0.2412	0.10405	0.02363	0.12029	0.13505	0.06817	-0.12834	0.18368	0.1863	0.13313	0.22423	0.16378	0.02607	0.2351	0.13364
0.16872	-0.17069	0.28744	0.18633	0.29145	0.20261	0.01537	0.17427	0.25618	0.07872	0.01513	0.16818	0.20232	0.13278	0.29945	0.18294	-0.01871	0.27715	-0.03107
0.19829	-0.07028	0.27942	0.1886	0.25451	0.25652	0.0192	0.19708	0.23594	0.0478	-0.04922	0.17864	0.1949	0.17984	0.26749	0.18392	0.01841	0.23258	0.04143
0.24676	-0.19253	0.25357	0.17532	0.27133	0.14237	0.0214	0.12142	0.25471	0.07392	-0.13854	0.05276	0.15278	0.1484	0.34601	0.18616	-0.0428	0.23943	-0.0219
0.19484	-0.41747	0.27311	0.19244	0.25588	0.18851	0.05214	0.13624	0.14666	0.08092	0.1069	0.16873	0.20666	0.18179	0.29769	0.19223	0.08974	0.28927	0.12697
0.18573	-0.27955	0.26989	0.17189	0.30787	0.1605	0.02329	0.18626	0.21923	0.05923	0.02997	0.22597	0.24286	0.21404	0.30929	0.17469	0.03348	0.24388	0.07558
0.12632	-0.34314	0.29733	0.18903	0.2732	0.23893	0.05221	0.20974	0.25445	0.03964	-0.14992	0.13788	0.17968	0.15504	0.27505	0.18536	0.00273	0.1588	-0.00656
0.31869	-0.38929	0.28288	0.18816	0.27112	0.13102	0.02148	0.12618	0.1196	0.08498	-0.04974	0.15097	0.17557	0.17517	0.25052	0.18468	0.0476	0.25396	0.11748
0.27617	-0.30756	0.27723	0.17904	0.22978	0.12099	0.02904	0.14423	0.24513	0.05478	-0.0032	0.16744	0.19111	0.17428	0.25317	0.17515	0.01855	0.24605	0.12558

SPHINGO	ABOLISM	OCIATED	ERICENT	ER_RET	K_V_ACTI	PCR_LIG	GPER1_	DIATED_A	VENTS_IN	KAGE_T	VENTS_IN	ORMONE	A_12_13_S	PHA_I_SIG	HA_Q_SIG	HA_S_SIG	HA_Z_SI
0.09139	0.15302	0.16733	0.32329	0.14094	0.1214	0.03456	0.20728	0.08073	0.02054	0.25274	0.00572	0.16971	-0.04182	0.1272	0.10281	0.16148	0.16121
0.05416	0.15817	0.14479	0.27355	0.1492	0.11473	0.03204	0.22406	0.03533	-0.00316	0.24851	0.07428	0.10937	-0.06149	0.10713	0.07002	0.16913	0.14334
0.07399	0.20704	0.13469	0.28214	0.14455	0.21088	0.01272	0.19264	0.10119	-0.01848	0.30191	-0.15406	0.11979	-0.02957	0.11465	0.08616	0.15718	0.13466
0.16788	0.11598	0.17066	0.2793	0.16832	0.36199	0.11691	0.27996	0.19941	-0.02843	0.31938	-0.31454	0.19458	0.03501	0.16857	0.16367	0.24373	0.24903
0.12399	0.10588	0.1558	0.25627	0.14784	0.23489	0.04886	0.23662	0.16333	0.02499	0.31926	-0.11125	0.1676	0.00717	0.14782	0.12779	0.21593	0.20142
0.12241	0.12224	0.14381	0.19314	0.14387	0.17784	0.07356	0.19253	0.08879	-0.06362	0.28644	-0.24681	0.0868	-0.04894	0.15539	0.09294	0.14988	0.16544
0.08383	0.1187	0.10982	0.23846	0.11924	0.28939	0.10775	0.22112	0.13924	-0.06104	0.31717	-0.21525	0.14563	-0.02853	0.16225	0.12013	0.18762	0.18084
0.16501	0.15681	0.18413	0.25669	0.16399	0.33134	0.12671	0.26756	0.20364	-0.05345	0.32652	-0.28843	0.20555	0.04023	0.16536	0.14523	0.24069	0.21791
0.19679	0.09288	0.18079	0.22932	0.15721	0.31136	0.13616	0.25813	0.21104	-0.08192	0.3209	-0.24935	0.19376	0.05098	0.16247	0.13483	0.23346	0.20934
0.1508	0.09729	0.16202	0.21561	0.14936	0.25259	0.13564	0.2665	0.19428	-0.07056	0.32689	-0.33572	0.20519	0.03712	0.18306	0.15621	0.24069	0.23472
0.0255	0.18692	0.11489	0.27302	0.16873	0.21061	0.04716	0.179	0.02601	-0.00241	0.26611	-0.0131	0.04692	-0.06189	0.11269	0.07797	0.13315	0.123
0.07494	0.19798	0.13356	0.26764	0.14807	0.1973	0.07364	0.21437	0.06547	-0.02514	0.28812	-0.12287	0.12056	-0.04738	0.15274	0.11917	0.16817	0.14027
0.02465	0.151	0.136	0.31379	0.17447	0.10239	0.06801	0.23823	-0.08305	0.0513	0.25874	0.16992	0.04695	-0.0318	0.12489	0.10877	0.18322	0.17518
0.15453	0.1356	0.14275	0.25539	0.16103	0.23027	0.05759	0.22307	0.1288	-0.00848	0.32223	-0.21334	0.1427	-0.00993	0.13488	0.10662	0.19353	0.16008
0.10437	0.15381	0.14516	0.27477	0.15548	0.19061	0.05347	0.20191	0.07729	-0.08144	0.30245	-0.27803	0.14293	-0.04426	0.13195	0.07958	0.16231	0.14913
0.0216	0.19517	0.11099	0.30286	0.1398	0.15529	-0.02391	0.15277	-0.09059	0.05106	0.24203	0.07768	0.06785	-0.0868	0.08518	0.04438	0.1058	0.0927
0.14071	0.18112	0.16381	0.26172	0.15615	0.27376	0.11883	0.27355	0.13793	0.02822	0.31217	-0.1106	0.13512	0.015	0.15631	0.15533	0.22645	0.23219
0.14597	0.1124	0.16404	0.26022	0.16161	0.31196	0.08448	0.24982	0.18468	-0.02572	0.3248	-0.25609	0.17711	0.02731	0.151	0.12909	0.21999	0.20789

OTEIN_MIS	_ACETYL	HCMV_EA	E_HCMV_I	HCMV_LA	S_DEACET	E_HDL_A	E_HDL_CL	HDL_RES	_DEMETH	HOMOLOG	THROUGH	H_SINGLE	HOG_LIG	EDGEHOG	EDGEHO	HEME_BK	HEME_DE	_HEME_S	ME_HEM	CATE_HEP
0.21883	0.26593	0.24104	0.21125	0.23801	0.32704	0.18485	0.39431	0.14399	0.38871	-0.03915	0.05185	-0.07899	0.2729	0.25272	0.26448	0.10958	0.15825	0.09426	0.14148	0.00361
0.19284	0.29944	0.25621	0.20938	0.22236	0.34551	0.14415	0.30839	0.17136	0.3918	0.04251	0.14694	-0.01528	0.26939	0.26061	0.25627	0.13671	0.17987	0.09522	0.1305	0.00618
0.21743	0.28983	0.26025	0.21611	0.23812	0.3385	0.16366	0.36767	0.23009	0.39336	0.04962	0.13995	-0.02684	0.24958	0.24455	0.24867	0.15129	0.22768	0.09957	0.14675	-0.01999
0.22112	0.27185	0.24082	0.21006	0.22487	0.32351	0.22223	0.39773	0.31063	0.38717	0.0256	0.09748	-0.03327	0.26341	0.2575	0.25653	0.10856	0.23453	0.08706	0.19157	-0.00768
0.21463	0.28409	0.2442	0.20864	0.22782	0.32376	0.20529	0.3618	0.2895	0.38729	-0.00291	0.08418	-0.06564	0.28915	0.27625	0.2858	0.15221	0.23661	0.11371	0.16975	0.04889
0.21572	0.27973	0.23911	0.19211	0.21368	0.34689	0.19237	0.37143	0.3602	0.38885	0.04148	0.09492	-0.05023	0.27002	0.25594	0.26031	0.19031	0.20113	0.09499	0.18003	0.00253
0.21462	0.28198	0.24111	0.1872	0.20751	0.34147	0.2044	0.37392	0.293	0.39232	0.04319	0.14452	-0.03192	0.24943	0.25033	0.24878	0.06707	0.19965	0.0974	0.18722	0.03274
0.20011	0.25717	0.22589	0.19787	0.21254	0.30485	0.22958	0.41518	0.35353	0.38292	-0.0225	0.04153	-0.08591	0.285	0.27744	0.27712	0.1513	0.24878	0.10497	0.20274	-0.00628
0.18328	0.26908	0.23038	0.19845	0.21556	0.31031	0.23831	0.41696	0.36119	0.38436	-0.02044	0.07044	-0.10125	0.28356	0.27534	0.27781	0.12006	0.22876	0.09183	0.19689	0.03942
0.22126	0.26568	0.23392	0.19692	0.21421	0.31944	0.21573	0.39363	0.37209	0.38447	0.0238	0.07187	-0.05548	0.27162	0.2642	0.26361	0.1213	0.22647	0.10005	0.19476	-0.0213
0.16899	0.291	0.25759	0.20913	0.21181	0.33959	0.16867	0.38952	0.22868	0.39174	0.03156	0.13349	-0.04278	0.24962	0.26149	0.25042	0.0915	0.16683	0.07771	0.13687	-0.07198
0.23876	0.28567	0.25881	0.21449	0.22834	0.33073	0.21418	0.38098	0.33651	0.38431	0.04771	0.11605	-0.02	0.26507	0.26211	0.26227	0.15182	0.16446	0.10145	0.16835	0.00866
0.19243	0.29266	0.27034	0.23555	0.24487	0.35704	0.17614	0.40611	0.25678	0.39718	0.07205	0.15359	0.02416	0.26141	0.28348	0.26319	0.08955	0.12958	0.09873	0.13969	0.01621
0.19571	0.27962	0.26084	0.21775	0.23371	0.32389	0.2027	0.38551	0.27951	0.38956	-0.01997	0.10932	-0.09135	0.26048	0.27495	0.2644	0.13394	0.23872	0.07924	0.16945	0.04765
0.18849	0.27769	0.23872	0.20257	0.21913	0.33005	0.2214	0.41254	0.27288	0.38632	0.03269	0.11021	-0.04097	0.26748	0.25783	0.25519	0.09172	0.17282	0.10045	0.15856	-0.01246
0.17719	0.28266	0.25426	0.21555	0.22932	0.33363	0.07691	0.36827	0.11505	0.39397	0.08793	0.23013	0.02296	0.25092	0.24994	0.24752	0.07598	0.15075	0.06293	0.10925	0.05877
0.20326	0.27447	0.25714	0.22294	0.23498	0.33	0.19846	0.3818	0.30763	0.38519	-0.03984	0.11869	-0.09906	0.25515	0.26321	0.25	0.13353	0.24189	0.10701	0.17687	0.02902
0.19895	0.26373	0.24332	0.20712	0.21858	0.3112	0.20621	0.38151	0.28437	0.38292	-0.02201	0.09247	-0.07961	0.28088	0.27962	0.27877	0.10599	0.21922	0.07705	0.17684	-0.05488

ISTIDINE	ATION_AR	E_HIV_IN	E_HIV_LI	ANSCRIP	RANSCRI	NA_PAIR	IDLOGY_D	ERACTIO	E_HSF1_A	EPENDEN	)_HORMO	IS_GAG_B	IS_GAG_D	1_BINDS	AN_BIOSY	LURONA	AN_UPTA
-0.083	-6.2E-05	0.16275	0.09188	0.00413	-0.04455	-0.05972	0.17144	0.21246	0.28785	0.22834	0.2514	-0.05582	-0.01234	0.29968	0.12683	0.14027	0.14226
-0.27098	0.0921	0.16716	0.10718	0.09829	-0.00315	0.00651	0.22397	0.20996	0.31845	0.23561	0.26062	0.01056	0.00255	0.32797	0.031	0.16667	0.18584
-0.14087	0.01813	0.16273	0.10231	0.02389	0.00425	-0.00537	0.20482	0.21381	0.26371	0.21486	0.25471	-0.12372	-0.08048	0.29468	0.34349	0.24141	0.22505
-0.06535	-0.03345	0.14948	0.06194	-0.0293	-0.05286	-0.01218	0.19005	0.20238	0.24205	0.1792	0.27238	-0.11122	-0.01903	0.26296	0.17914	0.25272	0.26359
-0.05572	0.00967	0.16983	0.07988	0.0094	-0.02827	-0.04614	0.19661	0.22787	0.25251	0.15204	0.25768	-0.08621	0.02716	0.29893	0.32341	0.2395	0.22628
-0.38081	0.02426	0.16328	0.0812	0.02333	-0.01588	-0.03468	0.21312	0.2238	0.26557	0.20901	0.24338	-0.17309	-0.05349	0.30051	0.16047	0.11458	0.10735
-0.3931	0.02324	0.14886	0.06449	0.01437	-0.00846	-0.01189	0.2007	0.21291	0.26362	0.20389	0.24547	-0.06202	0.02503	0.31828	0.17615	0.22836	0.23601
-0.01205	-0.06442	0.14727	0.0401	-0.05508	-0.09122	-0.06771	0.17246	0.20746	0.25151	0.18231	0.27813	-0.24245	-0.12367	0.25608	-0.00687	0.22398	0.25545
-0.05276	-0.04518	0.15213	0.05004	-0.03973	-0.05521	-0.08385	0.17372	0.21222	0.23298	0.17261	0.2743	-0.11412	0.01585	0.26002	0.31126	0.22363	0.20895
0.03213	-0.01609	0.15389	0.05977	-0.00948	-0.04924	-0.03662	0.1919	0.213	0.25001	0.15866	0.26691	-0.21227	-0.04444	0.28345	0.02043	0.21707	0.24424
-0.40894	-0.00798	0.14389	0.07281	-0.00195	-0.03891	-0.02467	0.21485	0.19759	0.27915	0.22152	0.30342	-0.07323	-0.05691	0.25117	0.37995	0.17702	0.14323
-0.315	-0.03874	0.15406	0.07516	-0.02986	-0.04615	0.00176	0.2036	0.21416	0.25934	0.19868	0.27196	-0.1889	-0.02487	0.27896	0.29752	0.15843	0.13418
-0.42316	0.04456	0.17215	0.11323	0.0428	-0.00702	0.04784	0.22261	0.21798	0.2942	0.23179	0.29583	-0.00011	0.00177	0.31558	0.32922	0.02586	-0.02685
-0.18194	-0.0064	0.15565	0.07718	-0.00026	-0.06185	-0.07334	0.18164	0.21531	0.2672	0.2308	0.28579	-0.10758	0.04939	0.29126	0.30773	0.2576	0.24958
-0.3255	0.00062	0.15098	0.07565	0.00844	-0.03413	-0.02054	0.20153	0.19804	0.26151	0.21187	0.27813	-0.17839	-0.08113	0.28903	0.3678	0.22658	0.20366
-0.39618	0.03091	0.15117	0.08977	0.0229	-0.02674	0.04636	0.22012	0.19856	0.26799	0.2097	0.27134	0.09566	0.10377	0.30361	0.39686	0.09138	0.03728
-0.15539	-0.00344	0.15302	0.07367	-0.00429	-0.04392	-0.08066	0.17683	0.20769	0.2564	0.19646	0.27843	-0.11125	0.03872	0.28024	0.2459	0.23636	0.23481
-0.05182	-0.03209	0.15885	0.06007	-0.03195	-0.04331	-0.06069	0.17657	0.22063	0.23212	0.16387	0.27451	-0.21535	-0.05734	0.2545	0.34014	0.24786	0.23322

HYDROLY	IGNALING	VARIANT_I	RECRUI	NE_RECE	IS_BETWE	ATION_OF	N_OF_C	ESIS_SE	ON_OF_CH	_MYCOB	INFECTIO	E_INFLA	NFLUENZ	DNA_REC	N_INITIA	D_FOR_T	RIGGER
0.00091	0.40106	-0.01388	0.19239	-0.39387	0.03161	0.20099	0.15494	0.15838	-0.22601	0.28657	0.22404	0.07645	0.29371	0.26573	0.20075	-0.13978	0.00673
-0.19557	0.33731	0.03532	0.17547	-0.39405	0.03039	0.20049	0.11537	0.16058	-0.31181	0.22263	0.22414	0.08145	0.31362	0.27174	0.15232	-0.06141	-0.08402
0.09195	0.37716	-0.00937	0.13228	-0.46486	0.07054	0.16969	0.14424	0.18623	-0.17248	0.27729	0.22306	0.08676	0.31241	0.28366	0.21988	-0.15349	0.08463
0.16538	0.35522	-0.00496	0.16855	-0.41307	0.15475	0.24245	0.18792	0.18125	-0.0768	0.30596	0.23383	0.11243	0.29891	0.25431	0.21417	-0.11208	0.20179
0.05929	0.3625	0.03295	0.16825	-0.43421	0.06982	0.2398	0.19196	0.18324	-0.24462	0.28917	0.22877	0.12707	0.30122	0.26473	0.20108	-0.19009	0.12809
0.02687	0.17063	-0.01105	0.08711	-0.33803	0.01689	0.19935	0.13329	0.19304	-0.13935	0.25947	0.21802	0.05561	0.31121	0.26568	0.21467	-0.10333	0.28022
0.05441	0.34877	0.03392	0.12792	-0.41501	0.11864	0.22093	0.14175	0.22101	-0.19117	0.24489	0.2217	0.13076	0.31154	0.28559	0.19325	-0.14524	0.26544
0.00937	0.37425	0.04006	0.19191	-0.43121	0.17047	0.24556	0.21559	0.20211	-0.13912	0.31679	0.23186	0.15213	0.28933	0.24033	0.19528	-0.12751	0.22233
0.0369	0.34663	0.00808	0.17308	-0.39493	0.15453	0.23936	0.20739	0.19123	-0.11493	0.30196	0.23032	0.12045	0.2905	0.25164	0.21661	-0.16461	0.2044
0.1003	0.35014	0.04383	0.17819	-0.41536	0.17692	0.25553	0.22214	0.1557	-0.17844	0.30251	0.2307	0.15065	0.29339	0.26539	0.17857	-0.18277	0.22667
0.03487	0.31874	-0.05202	0.11797	-0.47261	0.00514	0.17527	0.11138	0.11998	-0.16952	0.26993	0.21756	0.08374	0.30393	0.26485	0.2244	-0.08276	0.07744
0.00314	0.37161	-0.07517	0.15072	-0.46222	0.05092	0.20119	0.13242	0.07881	-0.21921	0.26908	0.22273	0.07283	0.31182	0.26893	0.20265	-0.1564	0.17236
-0.06514	0.39875	-0.12822	0.06222	-0.3944	-0.04187	0.2139	0.07686	0.11477	-0.26546	0.22162	0.22883	0.04127	0.31032	0.26969	0.26721	-0.08782	0.02036
0.08784	0.34648	0.03282	0.17131	-0.47948	0.05644	0.21325	0.14911	0.19209	-0.09807	0.28061	0.22852	0.1031	0.30957	0.26355	0.21142	-0.13702	0.13404
0.03227	0.40002	0.0053	0.15217	-0.44936	0.02772	0.1935	0.12677	0.11903	-0.11256	0.28669	0.22442	0.07473	0.30462	0.26732	0.21651	-0.13844	0.10962
-0.11589	0.38295	-0.07829	0.1152	-0.46856	0.00131	0.1808	0.1073	0.11511	-0.24818	0.23999	0.21837	0.06087	0.30954	0.26214	0.2168	-0.10296	-0.13519
0.08602	0.34013	-0.04385	0.14187	-0.43421	0.05575	0.22924	0.14679	0.15971	-0.13498	0.28305	0.23043	0.10747	0.305	0.26801	0.2557	-0.11274	0.06661
0.08901	0.38675	0.02958	0.17425	-0.44284	0.12384	0.25028	0.18465	0.24699	-0.06094	0.28858	0.2286	0.12092	0.28921	0.25517	0.1744	-0.17453	0.13364

NUCLEAR_E	LISTERIA	_LISTER	NATE_IM	XL_PHOS	OTEINS_IN	SULIN_R	IN_RECE	ECEPTOR	ION_OF_	EGRATION	CELL_SU	INTEGRIN	REV_WIT	VPR_WIT	N_BETW	MULUS_O	F_NUCL
0.28667	0.3618	0.17434	0.16576	-0.04351	0.18088	0.1347	0.20287	-0.01975	0.22208	0.3267	0.09304	0.15119	0.13613	0.10625	0.24714	-0.31777	0.17253
0.28276	0.34695	0.10339	0.12049	-0.09536	0.18243	0.02617	0.16159	-0.07072	0.21514	0.33689	0.0831	0.15048	0.14336	0.11422	0.26272	-0.07874	0.16668
0.30523	0.38262	0.17241	0.15229	-0.0496	0.13421	0.0249	0.21905	-0.02885	0.20444	0.33078	0.12401	0.19762	0.16751	0.13291	0.30469	-0.23023	0.17351
0.2578	0.3647	0.1684	0.20391	-0.07402	0.15392	0.10693	0.24606	0.00142	0.2524	0.28097	0.15483	0.21846	0.09875	0.05492	0.23062	-0.21138	0.16166
0.26874	0.24102	0.18263	0.19117	-0.00559	0.17486	0.08146	0.178	-0.02282	0.2389	0.29556	0.10198	0.21505	0.127	0.07598	0.29906	-0.18954	0.14746
0.26433	0.26974	0.14607	0.15771	-0.04857	0.13819	0.04734	0.10218	-0.11282	0.20585	0.35199	0.11334	0.17511	0.13081	0.11244	0.3475	-0.18601	0.19763
0.24467	0.33603	0.13212	0.17632	-0.06193	0.09099	0.03117	0.15197	-0.04312	0.21012	0.31012	0.19967	0.20827	0.12879	0.09188	0.20703	0.01568	0.18418
0.23029	0.31696	0.19847	0.21657	-0.02594	0.17156	0.11455	0.23318	-0.02883	0.24196	0.2571	0.1412	0.23505	0.06569	0.02337	0.27588	-0.13088	0.1532
0.24438	0.30975	0.19718	0.21191	-0.02254	0.16875	0.10797	0.24052	-0.03672	0.23152	0.27658	0.17725	0.23324	0.08112	0.03861	0.25427	-0.19007	0.13223
0.22768	0.27903	0.18857	0.21535	-0.0424	0.13287	0.09753	0.21213	-0.04663	0.25072	0.29298	0.11368	0.22777	0.09737	0.05269	0.25016	-0.13387	0.15103
0.25353	0.36659	0.08911	0.15246	-0.07302	0.14761	0.0769	0.17417	-0.04587	0.18775	0.33109	0.08596	0.1755	0.11856	0.09443	0.2332	0.00617	0.21891
0.20177	0.30862	0.12336	0.1608	-0.09348	0.11945	0.10033	0.12733	-0.09074	0.21883	0.32803	0.12257	0.17627	0.14695	0.11506	0.28456	0.0229	0.20957
0.28036	0.3628	0.08657	0.11263	-0.09368	0.16152	0.05592	0.19322	0.01375	0.2223	0.33124	0.07887	0.16763	0.16049	0.12528	0.28846	-0.0532	0.19525
0.25563	0.34361	0.15719	0.19091	-0.09633	0.14292	0.0687	0.22856	-0.04733	0.2081	0.30617	0.11057	0.22628	0.14617	0.115	0.25143	-0.13282	0.12423
0.27317	0.34332	0.12099	0.18158	-0.06346	0.14187	0.09314	0.16498	-0.04328	0.19819	0.30471	0.07268	0.18901	0.10635	0.07386	0.24296	-0.28096	0.22648
0.26368	0.34701	0.11754	0.09948	-0.07197	0.12064	0.07995	0.15431	-0.0444	0.17895	0.30452	0.05488	0.14716	0.14222	0.10307	0.2731	-0.40197	0.20653
0.24802	0.34604	0.16523	0.17655	-0.0771	0.14355	0.0642	0.22194	0.00661	0.24883	0.31259	0.12445	0.20519	0.11966	0.09244	0.26624	-0.17897	0.1526
0.26804	0.36681	0.173	0.18922	-0.0211	0.18302	0.08921	0.23188	-0.02214	0.22923	0.3035	0.12577	0.23029	0.10235	0.06605	0.28015	-0.22882	0.15243

RON_ALP	FERON_G	TERFER	ERLEUKIN	UKIN_12	RLEUKIN	RLEUKIN	RLEUKIN	RLEUKIN	EUKIN_1	RLEUKIN	ERLEUKI	EUKIN_20	RLEUKIN	RLEUKIN	RLEUKIN	EUKIN_2	ERLEUKI	RLEUKIN
0.10778	0.09907	0.14287	-0.03467	0.34304	0.33904	0.01578	0.1079	0.3693	0.1708	0.17513	0.17898	0.07821	0.15899	0.23045	0.25638	0.02251	-0.05706	0.25638
0.12752	0.08608	0.15407	-0.13887	0.32155	0.31725	-0.04744	0.07934	0.18026	0.15597	0.05277	0.17992	-0.00348	0.07734	0.19279	0.24053	-0.01833	-0.11904	0.24053
0.17296	0.13517	0.17786	-0.09718	0.3431	0.34077	-0.04419	0.09004	0.36149	0.15689	0.20101	0.16425	0.0559	0.08912	0.19796	0.264	0.04209	-0.10307	0.264
0.18016	0.15817	0.17128	-0.04792	0.34055	0.33672	-0.01066	0.09764	0.42038	0.16352	0.17009	0.17151	0.06951	0.14508	0.19221	0.2546	0.08646	-0.10887	0.2546
0.15676	0.1508	0.16647	-0.09134	0.35021	0.34631	0.02355	0.11842	0.34848	0.1887	0.20571	0.1993	0.10018	0.18467	0.22593	0.27749	0.09117	-0.00958	0.27749
0.16324	0.10597	0.16174	-0.08806	0.33808	0.33399	-0.00472	0.0878	0.34529	0.16717	0.17268	0.17946	0.09504	0.16942	0.20144	0.26193	0.03639	0.01398	0.26193
0.21625	0.14329	0.17671	-0.03175	0.33615	0.33178	-0.01863	0.08456	0.38312	0.1637	0.20372	0.17734	0.06128	0.1405	0.1976	0.26624	0.08366	-0.0236	0.26624
0.17751	0.15971	0.16186	-0.03861	0.35669	0.35203	0.01612	0.1138	0.42464	0.18719	0.20728	0.1916	0.12054	0.18822	0.24691	0.291	0.10436	-0.07024	0.291
0.22316	0.20539	0.18926	0.13104	0.35218	0.34721	0.02279	0.107	0.43685	0.18611	0.25497	0.19379	0.10744	0.19384	0.20942	0.27362	0.10134	-0.02621	0.27362
0.25238	0.18707	0.18921	-0.0349	0.35193	0.347	0.03996	0.1256	0.42027	0.18112	0.22695	0.18567	0.15168	0.22623	0.21913	0.2855	0.12963	0.03977	0.2855
0.08777	0.05143	0.13776	-0.05076	0.33033	0.32918	-0.08008	0.058	0.18751	0.16146	0.17973	0.18102	-0.03602	0.04043	0.22907	0.24482	-0.03103	-0.17242	0.24482
0.18785	0.12112	0.17441	-0.05055	0.35274	0.34809	-0.03497	0.09704	0.1544	0.1589	0.16713	0.17545	0.06553	0.15209	0.23891	0.29518	0.01738	-0.07423	0.29518
0.10238	0.03023	0.14557	-0.06969	0.32032	0.3168	0.00227	0.10106	0.06825	0.15586	0.06602	0.18162	-0.02297	0.04164	0.19164	0.23197	-0.05523	-0.09763	0.23197
0.10342	0.11773	0.16013	-0.03557	0.34379	0.34181	-0.0204	0.10658	0.34228	0.17331	0.17769	0.18626	0.01466	0.09504	0.23947	0.27138	0.03819	-0.0871	0.27138
0.15006	0.13031	0.16444	-0.09363	0.34146	0.33945	-0.02696	0.09902	0.38528	0.17194	0.188	0.18152	0.04418	0.09304	0.18932	0.24558	0.00263	-0.09427	0.24558
0.13539	0.08909	0.1593	-0.10569	0.3409	0.33823	-0.06281	0.06504	0.11191	0.13856	0.06892	0.16511	-0.02178	0.07182	0.20514	0.26615	-0.08908	-0.20234	0.26615
0.10085	0.09491	0.14655	-0.08904	0.35067	0.35043	-0.02918	0.07029	0.39896	0.15982	0.15975	0.17108	0.00749	0.09045	0.19678	0.2318	0.05382	-0.12723	0.2318
0.21513	0.18558	0.1847	-0.03226	0.34986	0.34508	0.00403	0.0989	0.32077	0.17098	0.1959	0.18441	0.10045	0.17791	0.22878	0.28733	0.10426	-0.04194	0.28733

ERLEUKI	RLEUKIN	NTERLEUK	4_AND_IN	EUKIN_6	ERLEUKI	ERLEUKI	ERLEUKI	KIN_RECE	ESTINAL	SIGNALIN
0.14391	0.01437	0.04874	0.13889	0.09109	0.09109	0.33072	0.15899	-0.01263	-0.43615	0.12161
-0.31073	-0.04246	-0.00792	0.12759	0.07345	0.07345	0.31771	0.07734	-0.08188	-0.22019	0.12323
-0.22441	0.00399	0.07144	0.14542	0.12912	0.12912	0.32843	0.08912	0.0252	-0.28237	0.1168
-0.10463	-0.00115	0.10065	0.19855	0.0783	0.0783	0.34863	0.14508	0.05759	-0.0155	0.12232
-0.30914	0.02611	0.10583	0.17134	0.13054	0.13054	0.34496	0.18467	0.05988	-0.32887	0.13044
-0.25929	-0.01273	0.03552	0.12026	0.10524	0.10524	0.33005	0.16942	-0.01801	-0.33451	0.12876
-0.09794	-0.02834	0.0855	0.1943	0.07448	0.07448	0.3368	0.1405	0.05992	-0.12736	0.11652
-0.32147	0.04757	0.13444	0.18796	0.14199	0.14199	0.35251	0.18822	0.06674	0.17967	0.12207
-0.33521	0.03298	0.13678	0.19327	0.12662	0.12662	0.35504	0.19384	0.08283	-0.08261	0.12386
-0.27867	0.04044	0.13144	0.18042	0.1421	0.1421	0.34422	0.22623	0.05868	-0.02889	0.12902
0.05091	-0.05962	0.03128	0.1395	0.05586	0.05586	0.33756	0.04043	-0.00849	-0.47173	0.11487
-0.17703	-0.01851	0.03175	0.14074	0.11537	0.11537	0.3251	0.15209	-0.04008	-0.46081	0.11578
0.08948	-0.04467	-0.00118	0.07156	0.06837	0.06837	0.31529	0.04164	-0.01895	-0.45235	0.13258
-0.2216	-0.0163	0.05736	0.1848	0.04544	0.04544	0.32638	0.09504	0.02505	-0.21807	0.11457
-0.12242	-0.01133	0.04207	0.15073	0.07882	0.07882	0.31968	0.09304	-0.01219	-0.46556	0.11457
-0.4948	-0.08545	-0.03975	0.06351	0.05626	0.05626	0.31472	0.07182	-0.1007	-0.44161	0.10802
-0.17034	-0.04127	0.0736	0.18619	0.02979	0.02979	0.34806	0.09045	0.05668	-0.18513	0.11646
-0.40109	0.00367	0.11428	0.14899	0.1282	0.1282	0.34964	0.17791	0.07183	-0.06077	0.12355

FLAGELL	_RETROG	NTRA_GO	IC_PATH	WAY_OF	Y_RECTI	ACTIVITY	_CHANNE	ION_HO	NSPORT_I	RECRUI	ED_ACTIV	K4_DEFI	HA_ACTIV	ED_ACTI	TED_IND	UPTAKE	E_IRS_AC	MEDIATE
0.12581	0.06658	-0.09732	0.11333	0.04174	0.19493	0.08868	0.10582	0.21224	0.16553	0.23815	0.05837	0.22589	0.20025	-0.16219	0.16167	0.23946	-0.24397	-0.08954
0.163	0.06948	-0.09304	0.09124	0.07336	0.20265	0.12555	0.08865	0.19876	0.17476	0.23967	-0.00548	0.232	0.21931	-0.26176	0.16092	0.20725	-0.36869	-0.13502
0.16436	0.07394	-0.08501	0.10039	0.15951	0.15616	0.02776	0.12046	0.1929	0.1898	0.18773	0.03459	0.26077	0.22116	-0.27243	0.15445	0.2561	-0.42921	-0.09042
0.17906	0.09517	-0.04762	0.11058	0.21736	0.3177	0.00736	0.13022	0.18865	0.14849	0.21653	0.03218	0.28541	0.21574	-0.25208	0.15563	0.2837	-0.24779	-0.04259
0.17446	0.07751	-0.06429	0.09956	0.16927	0.2387	-0.077	0.10094	0.1764	0.15	0.23558	0.03313	0.29059	0.22536	-0.25568	0.13487	0.23161	-0.46309	-0.07035
0.11945	0.07291	-0.09382	0.0489	0.28575	0.15102	0.03534	0.07197	0.20479	0.19703	0.1466	-0.04818	0.26334	0.20969	-0.26316	0.10193	0.14729	-0.40874	-0.15362
0.14964	0.04716	-0.09545	0.07959	0.27447	0.19353	-0.02866	0.07922	0.17076	0.15261	0.18868	0.02318	0.30055	0.19378	-0.34872	0.16452	0.1976	-0.38955	-0.09435
0.19467	0.10189	-0.00743	0.11327	0.23715	0.27106	-0.05944	0.14434	0.18268	0.15222	0.23126	0.05716	0.28668	0.24804	-0.14101	0.14517	0.28749	-0.40793	-0.07399
0.18858	0.08982	-0.04554	0.10712	0.26076	0.25219	-0.04312	0.14875	0.17503	0.14264	0.21461	0.05026	0.2987	0.23812	-0.23465	0.14164	0.26774	-0.37909	-0.0859
0.17365	0.07851	-0.05925	0.10406	0.26551	0.284	-0.08151	0.12877	0.18097	0.15297	0.22738	0.04822	0.27625	0.21949	-0.2375	0.15425	0.25554	-0.45296	-0.09643
0.22404	0.08823	-0.09518	0.12904	0.14182	0.14822	0.04699	0.10333	0.1792	0.14276	0.17185	0.06204	0.26815	0.22982	-0.26039	0.14836	0.24005	-0.33324	-0.09034
0.14547	0.06957	-0.09122	0.12071	0.25464	0.19724	0.04709	0.08414	0.21142	0.1732	0.19765	0.0635	0.25493	0.20494	-0.29048	0.17864	0.20801	-0.34733	-0.15884
0.24877	0.08798	-0.13549	0.13264	0.14378	0.21945	0.1143	0.08483	0.16464	0.13129	0.16629	0.04264	0.25284	0.22502	-0.23502	0.13311	0.21012	-0.25066	-0.07581
0.23226	0.08734	-0.06848	0.09417	0.18332	0.18453	0.03078	0.11926	0.1806	0.15521	0.25318	0.03545	0.27892	0.24074	-0.27696	0.15127	0.2678	-0.41766	-0.10369
0.16847	0.07916	-0.07544	0.1097	0.19495	0.17148	0.04628	0.10254	0.18315	0.16898	0.21767	0.06053	0.26311	0.22491	-0.19007	0.12611	0.24134	-0.17583	-0.11661
0.18006	0.0625	-0.08687	0.12978	0.04883	0.10488	0.1086	0.07831	0.17441	0.15445	0.19763	0.11329	0.20833	0.17292	-0.2073	0.15815	0.22025	-0.40374	-0.10636
0.20781	0.0808	-0.06462	0.106	0.15384	0.31484	-0.00763	0.10778	0.18661	0.15453	0.18565	-0.00418	0.2686	0.20307	-0.29784	0.16014	0.26297	-0.36323	-0.02045
0.21363	0.09004	-0.05682	0.11615	0.18509	0.28277	-0.0656	0.13153	0.1748	0.1365	0.21648	0.03594	0.28297	0.23203	-0.20976	0.17694	0.25085	-0.42277	-0.06876

5_ANTIVI	ON_AND	SEPHIN	EAP1_NF	AN_SULF	AN_SULF	SULFATE	E_KERAT	ONE_BO	ILLING	OME_KIN	BINDS	1CAM_IN	ING_STR	AMININ	DOSOM	S_COV_2	_LDL_CL	LDL_RE	WAY_OF	EISHMAN	EIC_ACI	OME_LIPO	YTOGENE	NG_TERM	TION_OF
0.12213	0.10982	0.34216	0.23388	-0.26321	0.06016	-0.0285	0.19831	0.11133	0.22868	0.0695	-0.05491	0.17257	-0.04136	-0.05996	0.17635	0.12381	0.20445	0.45828	-0.03312	0.16851	0.03522	0.10232	0.22395	0.10782	0.10019
0.14204	0.10115	0.34588	0.23366	-0.23099	0.04439	-0.02903	0.18808	0.03336	0.20405	0.1112	0.00436	0.18514	0.05357	-0.06139	0.08784	0.11962	0.20728	0.44383	-0.16857	0.13666	-0.10325	0.06045	0.171	0.12891	0.1585
0.1666	0.09294	0.33932	0.22922	-0.2123	0.12559	0.01135	0.14188	-0.01375	0.23579	0.07173	-0.06558	0.21309	0.04666	-0.08158	0.09607	0.14177	0.22515	0.42933	-0.25823	0.15179	-0.02459	0.0637	0.23145	0.09332	0.09661
0.11573	0.09852	0.3024	0.22371	-0.23038	0.17306	0.05517	0.15876	0.0577	0.29275	0.10289	-0.05285	0.19464	0.01336	-0.11289	0.15279	0.15703	0.28993	0.41927	0.06729	0.20784	0.08559	0.1227	0.22145	0.0799	0.08411
0.12438	0.11402	0.3742	0.25006	-0.28354	0.1081	-0.00242	0.10543	0.06057	0.26869	0.09054	-0.03969	0.1995	-0.00183	-0.09364	0.11815	0.12711	0.24439	0.44514	0.18478	0.18318	-0.0156	0.08771	0.17934	0.00075	0.12612
0.14426	0.05512	0.33897	0.23505	-0.11796	0.17353	0.05817	0.13081	-0.10149	0.19168	0.08967	-0.0819	0.17776	0.05666	-0.07993	0.06379	0.16596	0.21516	0.44627	-0.29311	0.13626	-0.00468	0.13622	0.16659	0.08455	0.1066
0.14603	0.07995	0.3276	0.22939	-0.04664	0.18195	0.08277	0.08191	0.00401	0.21576	0.04666	0.0058	0.17216	0.01984	-0.0558	0.02005	0.138	0.18777	0.4428	-0.1529	0.1713	-0.01034	0.01504	0.18634	0.03747	0.11293
0.09533	0.10947	0.32287	0.23466	-0.12701	0.19207	0.08491	0.06388	0.0385	0.29228	0.08933	-0.06683	0.21266	-0.03845	-0.14762	0.14707	0.16064	0.2967	0.4623	0.11996	0.21489	0.08097	0.13785	0.22125	0.07213	0.05135
0.1115	0.10417	0.33633	0.23504	-0.15412	0.22536	0.09236	0.13116	-0.04801	0.27862	0.09126	-0.05739	0.21886	-0.0392	-0.07553	0.12651	0.14621	0.28528	0.44864	0.13652	0.20806	0.07768	0.13966	0.21753	0.05447	0.05597
0.1284	0.11118	0.34427	0.22977	-0.22005	0.15236	0.04114	0.08992	0.03406	0.28746	0.10383	-0.04441	0.19245	0.01123	-0.19698	0.09365	0.14365	0.26491	0.45229	0.14832	0.21508	0.01584	0.10988	0.1987	0.04504	0.09327
0.11653	0.09205	0.32685	0.23631	-0.0827	-0.00324	-0.02811	0.17361	0.14503	0.1742	0.11214	-0.08533	0.18839	0.02807	-0.05461	0.06746	0.16206	0.13077	0.40633	-0.20416	0.12706	-0.18992	0.01411	0.17049	0.00228	0.1224
0.15429	0.10695	0.33771	0.23206	-0.07412	0.13631	0.05937	0.12071	0.06887	0.21324	0.06084	-0.05595	0.18641	0.04164	0.00662	0.08928	0.14227	0.21573	0.44391	-0.15413	0.15443	-0.06779	0.06044	0.16752	0.06431	0.13371
0.14376	0.08873	0.35399	0.23595	-0.07855	-0.1163	-0.08059	0.17418	0.09746	0.1404	0.15929	-0.06975	0.21935	0.05906	-0.00267	0.13947	0.18004	0.16019	0.4365	-0.22301	0.12529	-0.08817	0.09522	0.1671	0.02913	0.12591
0.13172	0.12453	0.32162	0.23074	-0.06605	0.21809	0.12424	0.15076	0.12901	0.26079	0.11422	-0.04593	0.20307	-0.06452	-0.16497	0.09259	0.14159	0.23743	0.42568	-0.02766	0.18215	0.08664	0.04726	0.20514	0.07742	0.10712
0.11968	0.1067	0.33976	0.23178	-0.15998	0.14401	0.03825	0.16194	0.09874	0.25952	0.10042	-0.00035	0.18374	0.0182	-0.14267	0.10917	0.14525	0.18078	0.46137	-0.01022	0.16095	-0.06586	0.09887	0.18123	0.08284	0.10491
0.15223	0.11928	0.32773	0.22319	-0.14057	0.00197	-0.03308	0.17574	0.10482	0.11287	0.12115	-0.02902	0.18013	0.04062	-0.00984	0.10963	0.15152	0.12565	0.41366	-0.38612	0.10331	-0.10034	-0.01041	0.18052	0.0754	0.07639
0.10853	0.07132	0.32502	0.22475	-0.21299	0.16022	0.05736	0.15137	0.11837	0.26484	0.1173	-0.06712	0.20037	-0.08035	-0.03903	0.14675	0.13981	0.2791	0.44677	0.02995	0.1962	-0.0332	0.14316	0.21115	0.0571	0.11326
0.11858	0.09467	0.33166	0.23669	-0.24717	0.15233	0.03333	0.18065	0.04437	0.27574	0.11419	-0.04615	0.21144	-0.06577	-0.11993	0.11383	0.1479	0.29685	0.44861	-0.00846	0.19699	0.08056	0.06601	0.22533	0.0263	0.07594

OME_VES	K_AND_M	DEPEND	PK1_ERK	PK3_ERK	PK6_MAP	AMILY_SIG	EAR_EVE	LITATES_I	_TO_ZYG	URATION_	OF_SARS	_OF_SARS	OF_SARS_	OF_SARS	NEURONA	LATES_TI	TRANSCR	TOME_ME	IOTIC_RE	MEIOTIC
0.1959	0.23417	0.1532	0.10512	0.12227	0.28737	0.1993	0.13007	0.26552	0.31927	-0.24807	0.05655	0.34337	0.03886	0.20357	0.12719	0.20827	0.1837	0.29428	0.38167	0.29465
0.1736	0.22048	0.15427	0.03975	0.06849	0.2902	0.18626	0.0914	0.30021	0.33316	-0.28653	0.02967	0.36471	0.03303	0.20683	0.13343	0.28868	0.26375	0.328	0.39484	0.33302
0.17332	0.25874	0.13589	0.1095	0.14167	0.26906	0.20402	0.12328	0.31569	0.33385	-0.17753	0.09541	0.30356	0.08927	0.18962	0.06084	0.10109	0.10494	0.31641	0.38463	0.32766
0.22612	0.29066	0.16526	0.03286	0.06822	0.26889	0.19479	0.12531	0.26581	0.31792	-0.26148	0.11545	0.37171	0.07852	0.22515	0.12349	0.01907	0.07401	0.30301	0.38559	0.30417
0.20323	0.29682	0.17651	0.10318	0.09803	0.29957	0.21628	0.1297	0.2723	0.31751	-0.28301	0.05913	0.37206	0.03676	0.21487	0.13425	0.23893	0.17242	0.31725	0.38283	0.32908
0.18996	0.23536	0.14037	0.08335	0.01599	0.27361	0.19776	0.11493	0.31913	0.32272	-0.13074	0.07476	0.34115	0.10603	0.22118	0.13025	0.22041	0.17037	0.31611	0.38391	0.3263
0.16892	0.26131	0.15146	0.05379	0.08734	0.26995	0.18836	0.08882	0.28251	0.32507	-0.23535	0.14146	0.3152	0.11816	0.17629	0.10146	0.09671	0.11134	0.32485	0.40106	0.32909
0.24732	0.29956	0.18717	0.08774	0.05324	0.28279	0.20684	0.13346	0.22404	0.30573	-0.12779	0.09233	0.37038	0.06938	0.23652	0.10305	0.08822	0.0569	0.29695	0.38166	0.29582
0.24367	0.28419	0.14944	0.03419	0.02486	0.28638	0.20396	0.13206	0.23981	0.30462	-0.23318	0.08672	0.37365	0.05191	0.22477	0.12006	0.1118	0.08592	0.29645	0.37979	0.30071
0.21521	0.28928	0.17205	0.09068	0.03961	0.27913	0.19484	0.15166	0.19594	0.30956	-0.24008	0.12849	0.35499	0.09302	0.20084	0.1347	0.19613	0.15799	0.30866	0.38765	0.31139
0.1428	0.23404	0.12289	0.0067	0.08004	0.2757	0.19451	0.07281	0.2826	0.3375	-0.04511	0.0997	0.33078	0.09052	0.22795	0.12223	0.1076	0.14866	0.31694	0.39019	0.32378
0.16743	0.23721	0.10099	0.06889	0.08092	0.28468	0.19402	0.13639	0.26336	0.31989	-0.1931	0.12938	0.33248	0.10925	0.18576	0.1284	0.21529	0.16792	0.31529	0.3883	0.32549
0.17047	0.23997	0.09223	0.06769	0.12509	0.28575	0.20861	0.15909	0.33422	0.33268	-0.23108	0.18788	0.32631	0.17463	0.22326	0.08899	0.1314	0.16215	0.32142	0.39586	0.31936
0.19494	0.27677	0.19081	0.00858	0.00909	0.27096	0.19945	0.11738	0.22563	0.32276	-0.13968	0.04601	0.3381	0.05403	0.21096	0.14981	0.14776	0.14571	0.31659	0.38587	0.33035
0.18079	0.2602	0.15929	0.10937	0.10256	0.27981	0.19643	0.12471	0.23458	0.31624	-0.06491	0.07907	0.34811	0.06228	0.20599	0.06895	0.08874	0.10367	0.30859	0.38419	0.31427
0.10204	0.23446	0.07729	0.07778	0.14343	0.27077	0.19818	0.09006	0.33617	0.33246	-0.14269	0.13381	0.31616	0.1243	0.20652	0.07245	0.0946	0.1407	0.31745	0.39463	0.3147
0.22371	0.27157	0.12484	0.02157	0.02784	0.26906	0.19601	0.09763	0.27419	0.32545	-0.33388	0.09784	0.35557	0.08696	0.19983	0.13533	0.15768	0.15673	0.31004	0.3877	0.31714
0.22853	0.2852	0.15487	0.06673	0.06445	0.28605	0.20208	0.11656	0.22543	0.31512	-0.25451	0.12124	0.3699	0.07064	0.21644	0.13787	0.20157	0.13278	0.30109	0.38417	0.30113

MBRANE	RS_OF_B	F_AMINO	ANGIOTE	OLISM_O	ABOLISM	SM_OF_F	SM_OF_F	GESTED_	ETABOLIS	_OXIDE_	BOLISM_O	BOLISM_	BOLISM_	METABOL	ABOLISM	ISM_OF_
0.09988	0.11013	0.26592	0.11302	0.15553	0.08052	0.02236	0.06489	0.24292	0.04241	0.25734	0.0858	0.29172	0.10027	0.15953	0.05921	0.07997
0.07476	0.071	0.25995	-0.03386	0.1411	0.09097	0.01154	0.07527	0.12384	0.01624	0.18083	0.08168	0.29483	0.12755	0.2193	0.01794	-0.00189
0.0897	0.13883	0.27389	0.06883	0.16433	0.14195	0.01692	0.11885	0.14355	0.02339	0.268	0.10418	0.27567	0.16143	0.18139	0.03957	0.00527
0.11075	0.11693	0.25306	0.19337	0.15252	0.06916	0.12079	0.13629	0.11186	0.057	0.13266	0.10031	0.28103	0.14441	0.15121	0.048	0.0743
0.09959	0.10748	0.2642	0.12579	0.16801	0.08398	0.07159	0.04006	0.05205	0.03226	0.13273	0.11364	0.31108	0.1666	0.16696	0.04243	0.09234
0.08541	0.10736	0.2686	0.12439	0.14264	0.06417	0.13077	0.0779	0.09367	0.02095	0.2328	0.10271	0.29139	0.16851	0.18347	0.03628	0.04804
0.06321	0.09541	0.26837	0.08153	0.15019	0.11131	0.11206	0.12094	0.14581	0.01992	0.24008	0.11698	0.2833	0.10083	0.1782	0.02428	0.03693
0.12455	0.14054	0.26137	0.16021	0.15662	0.07378	0.08763	0.05281	0.13928	0.04892	0.12409	0.10473	0.30328	0.17484	0.12995	0.05666	0.12093
0.11555	0.14521	0.24863	0.15286	0.16029	0.07059	0.13895	0.06729	0.16163	0.0501	0.14063	0.09955	0.30803	0.14675	0.13702	0.05328	0.11165
0.10235	0.10832	0.24652	0.23008	0.14354	0.07704	0.09563	0.11065	0.0603	0.03935	0.13847	0.10524	0.29477	0.14633	0.14694	0.0326	0.09465
0.08393	0.05472	0.27749	0.07082	0.1427	0.07238	0.05437	0.10711	0.00496	0.02716	0.22242	0.14005	0.28018	0.09472	0.17175	0.04741	0.07368
0.08877	0.08651	0.28019	0.14328	0.15093	0.10563	0.05372	0.06275	0.15573	0.02262	0.25344	0.13766	0.29336	0.12651	0.16414	0.04414	0.07362
0.09973	0.06501	0.26258	-0.05557	0.12751	0.0884	0.07825	0.06968	0.08948	0.01829	0.22862	0.10667	0.29592	0.07077	0.18104	0.04771	-0.03502
0.09729	0.11206	0.26334	0.10259	0.17827	0.08152	0.05501	0.03632	0.07485	0.05129	0.18847	0.09373	0.28487	0.15723	0.16717	0.05398	0.05622
0.09603	0.09446	0.27404	0.09214	0.15803	0.05596	0.07916	0.09938	0.0547	0.0314	0.22122	0.12196	0.29269	0.09817	0.17186	0.0661	0.04972
0.07514	0.09232	0.28657	-0.01479	0.15136	0.11722	0.02664	0.16331	0.18887	0.01053	0.26156	0.1283	0.27913	0.0769	0.19047	0.02838	-0.00573
0.10324	0.09842	0.26529	0.09001	0.15865	0.06802	0.06074	0.10271	0.0491	0.04477	0.14996	0.07606	0.276	0.15996	0.17118	0.02695	0.07081
0.10775	0.17474	0.25275	0.16733	0.15658	0.11054	0.06179	0.10345	0.15467	0.03426	0.18419	0.10483	0.30223	0.13355	0.14658	0.03003	0.11812

OF_VIT	ER_SOLU	TALLOPR	OTHIONE	ION_SL	RATION_B	IONINE_S	ME_METH	IVATES_R	CTIVATES	ET_ACTIV	CTIVATES	CTIVATES	RACTS_W	PROMOTE	_RECEPT	_RECEP
0.0393	0.03313	0.1581	0.21543	0.10126	0.29038	0.18734	0.34379	0.16271	0.06874	0.20196	0.23702	0.06234	0.18535	0.11665	0.2174	0.0902
0.02553	0.01537	0.14501	-0.23003	0.0373	0.07028	0.01538	0.26958	0.11783	0.03763	0.20425	0.24126	0.05529	0.15509	0.09442	0.01858	0.06774
0.03861	0.02384	0.15125	-0.37733	0.02808	0.21596	0.13114	0.33171	0.24305	0.03055	0.21912	0.23808	0.04065	0.20736	0.09061	-0.02937	0.10295
0.05347	0.02071	0.15242	-0.25932	0.13354	0.27999	-0.02451	0.31975	0.35976	0.07143	0.20807	0.22379	0.05088	0.29014	0.12252	-0.11871	0.15098
0.02396	-0.00703	0.15584	-0.37345	0.07845	0.28511	0.09116	0.30375	0.21929	0.0019	0.20038	0.20491	0.0811	0.12051	0.06126	-0.13465	0.14172
0.04532	0.00585	0.14066	-0.3144	0.14252	0.10732	0.03775	0.26822	0.14638	0.0242	0.20336	0.22485	0.01602	0.06139	0.07385	-0.08281	0.08031
0.06605	0.03773	0.17124	-0.25329	-0.10084	0.14306	0.07472	0.31796	0.32761	0.04459	0.15108	0.24121	-0.02207	0.16224	0.09982	0.11027	0.13495
0.05637	0.03746	0.1584	-0.16741	0.132	0.2755	0.09539	0.32465	0.34172	0.05813	0.23861	0.26714	0.00764	0.2632	0.1279	-0.33269	0.20166
0.0632	0.03129	0.16333	-0.3147	0.14574	0.18653	0.07467	0.33191	0.35829	0.02247	0.19254	0.23879	0.00311	0.24186	0.09375	-0.32722	0.17127
0.04881	0.02251	0.15491	-0.22334	0.10646	0.26261	0.07986	0.27459	0.2439	-0.00921	0.23159	0.23433	0.01284	0.16561	0.068	-0.15068	0.17183
0.04607	0.03392	0.17854	-0.19294	0.03337	0.32645	0.1594	0.29521	0.24143	0.04571	0.0588	0.24851	-0.01299	0.19021	0.10501	0.05945	0.12438
0.04399	0.02779	0.16723	-0.31323	0.06418	0.26203	0.15247	0.30442	0.14695	0.10403	0.13095	0.2414	-0.02782	0.11942	0.13103	-0.09903	0.10737
0.05149	0.03001	0.14587	-0.07192	0.064	0.03696	0.12827	0.31607	0.09984	0.02016	0.16551	0.2528	0.1411	0.26021	0.09687	0.27461	0.14715
0.04001	0.02313	0.16458	-0.35498	0.13231	0.44978	0.04354	0.30152	0.16863	0.01292	0.15428	0.25221	0.05974	0.16426	0.08433	-0.00156	0.15774
0.05441	0.04102	0.15558	-0.18872	0.02478	0.34338	0.14315	0.30817	0.13493	0.02035	0.20748	0.23367	0.01912	0.0822	0.08108	-0.07199	0.13529
0.05167	0.04722	0.15576	-0.31135	-0.01056	0.09535	0.11385	0.3108	0.04086	0.09971	0.10967	0.24372	0.01778	0.33728	0.14955	0.10123	0.09793
0.02762	0.00389	0.19629	-0.31838	-0.01143	0.29098	0.05367	0.25463	0.35769	0.10615	0.17498	0.25317	0.0628	0.289	0.15098	0.02793	0.15687
0.05139	0.03252	0.16406	-0.19472	0.09134	0.13562	0.0737	0.31728	0.38565	0.03585	0.19776	0.25231	0.01354	0.22233	0.10551	-0.21194	0.1565

S_II_AN	ORNA_MI	MIRO_GT	ELLANEO	S_TRANSP	_MISMAT	CHONDRI	RIAL_CA	RIAL_FAT	D_BETA_C	BETA_OX	IRON_SU	ONDRIAL	ONDRIAL	CHONDRI	NDRIAL_
0.16559	0.02585	-0.0591	-0.43666	-0.09517	0.00031	0.13896	0.18398	0.14501	0.31564	0.4083	-0.01588	0.13428	0.08572	-0.02314	0.01892
0.16391	0.04673	-0.03585	-0.28023	-0.06066	0.08239	0.13869	0.18965	0.10765	0.27596	0.31207	-0.05895	0.13555	0.05772	-0.09637	0.0066
0.1799	0.03263	-0.2113	-0.26184	-0.09516	0.08471	0.14793	0.17684	0.15081	0.29272	0.37535	-0.09736	0.13489	0.12262	-0.04706	-0.01321
0.2226	-0.02846	-0.16882	-0.21985	-0.06447	0.0366	0.09382	0.13076	0.13016	0.27243	0.38069	-0.10668	0.07493	0.01947	-0.09691	-0.05083
0.20083	-0.02003	-0.02503	-0.35455	0.02006	0.01836	0.13035	0.16165	0.11861	0.27144	0.36471	-0.09227	0.09329	0.04644	-0.09123	-0.07145
0.16237	-0.00823	-0.15111	-0.30849	0.08899	0.10679	0.11822	0.11092	0.12827	0.31028	0.33225	-0.10966	0.11264	0.04811	-0.11603	-0.01586
0.16936	-0.01017	-0.07282	-0.29141	-0.09904	0.04571	0.13489	0.17452	0.15564	0.31601	0.3521	-0.09614	0.16987	0.08452	-0.03637	-0.02642
0.2268	-0.05798	-0.12041	-0.31993	0.01394	-0.0018	0.10379	0.10365	0.1282	0.30824	0.35994	-0.10576	0.06509	-0.01562	-0.16891	-0.06977
0.23894	-0.04557	-0.09364	-0.3268	-0.02281	0.00245	0.09381	0.06158	0.13088	0.33344	0.34243	-0.13287	0.03951	-0.02875	-0.12245	-0.08297
0.20753	-0.0171	-0.10749	-0.36334	-0.03462	0.06084	0.1169	0.11759	0.15797	0.34359	0.36549	-0.06543	0.08738	0.01337	-0.11098	-0.06163
0.16588	-0.01878	-0.13115	-0.07879	-0.05788	0.05086	0.13712	0.18738	0.14708	0.30723	0.36168	-0.17347	0.14403	0.19623	-0.08998	0.04032
0.16657	-0.0074	-0.02979	-0.27478	-0.0391	0.05514	0.12956	0.17528	0.15978	0.3273	0.38492	-0.02095	0.14888	0.12566	0.02907	0.03597
0.16763	0.03966	-0.14173	-0.20794	-0.02272	0.08761	0.14007	0.1911	0.08741	0.26414	0.29697	-0.1241	0.13572	0.14797	-0.07905	0.01141
0.22228	-0.02218	-0.12662	-0.22643	-0.07715	-0.03624	0.11917	0.15813	0.13941	0.27662	0.38201	-0.1506	0.11642	0.00975	-0.12384	-0.00092
0.19891	-0.01414	-0.20469	-0.2398	-0.08647	0.01072	0.11887	0.13621	0.15293	0.30226	0.36195	-0.10828	0.10321	0.00118	-0.14937	-0.01395
0.1488	0.01331	-0.0967	-0.19806	-0.12788	0.06585	0.15192	0.20854	0.15427	0.29577	0.40788	-0.01047	0.19774	0.21093	-0.04203	0.06113
0.20791	-0.02241	-0.138	-0.15821	-0.07617	-0.02947	0.14811	0.1902	0.16379	0.30475	0.39017	-0.06333	0.13161	0.02189	0.00374	0.00239
0.22608	-0.03578	-0.06186	-0.20899	-0.03293	-0.05634	0.11254	0.13088	0.12658	0.29875	0.37164	-0.09339	0.09529	0.04804	-0.08584	-0.02184

ME_MITO	_PHASE_	OTIC_G2	_METAPH	TOTIC_PR	MITOTIC	IC_SPIND	C_TELOPH	BY_MTB	SSOCIAT	NUM_COF	E_MRNA	Y_BY_3_T	_BY_5_T	E_MRNA	ITING_C	E_MRNA_
0.09812	0.20559	0.21962	0.1656	0.08056	0.27489	0.03839	-0.03062	0.13184	-0.03293	-0.13622	-0.03834	-0.1937	0.03018	-0.01297	-0.3028	0.1706
0.11754	0.20911	0.22527	0.17371	0.10836	0.27759	0.05071	0.07103	0.14535	-0.07344	0.02698	0.01736	-0.11037	0.13419	0.12026	-0.13106	0.24683
0.11992	0.21374	0.20549	0.1627	0.10072	0.28914	0.03031	0.068	0.16039	0.00176	-0.19203	0.00824	-0.21102	0.03791	0.02054	-0.44231	0.18812
0.11271	0.20631	0.21187	0.15675	0.08816	0.25746	0.03297	-0.0207	0.26134	0.03487	-0.26501	-0.0522	-0.20127	0.05875	0.10737	-0.02871	0.16
0.10481	0.22058	0.22939	0.17203	0.09658	0.26607	0.01769	0.07796	0.16322	0.02994	-0.12615	-0.03498	-0.15948	0.05833	0.06734	-0.14286	0.17215
0.11519	0.22649	0.21553	0.1632	0.08942	0.26413	0.03179	0.05997	0.15702	0.11224	-0.07898	-0.01529	-0.22685	0.05454	0.07403	-0.24326	0.19104
0.15908	0.20205	0.20115	0.14415	0.07785	0.26436	0.00743	0.05659	0.16981	0.14986	-0.17206	-0.01159	-0.09494	0.05862	0.0354	-0.21948	0.17393
0.06311	0.19527	0.22876	0.1547	0.0831	0.23863	0.01276	-0.0263	0.27816	0.04821	-0.1872	-0.08256	-0.20896	0.03852	-0.01088	-0.26299	0.13336
0.05854	0.20485	0.22989	0.15554	0.08535	0.24487	0.0044	-0.03974	0.29034	0.06354	-0.18629	-0.06375	-0.19639	0.02889	0.00719	-0.31249	0.14267
0.10373	0.19156	0.21988	0.15329	0.0857	0.24603	0.00933	0.0011	0.24584	0.07195	-0.1651	-0.04844	-0.17488	0.05573	0.17027	0.07557	0.16023
0.15537	0.22079	0.22746	0.15802	0.10349	0.26468	0.03048	0.05265	0.12193	0.01645	-0.15858	-0.03196	-0.23081	0.07447	0.01653	-0.47314	0.16689
0.12452	0.21312	0.2114	0.15408	0.08838	0.26525	0.02087	0.04529	0.14096	0.10096	-0.16429	-0.04676	-0.20321	0.03807	0.06492	-0.26052	0.16624
0.14565	0.23157	0.24023	0.19088	0.12831	0.28367	0.06828	0.04542	0.03824	0.07537	-0.15676	-0.01043	-0.17358	0.07805	0.10269	-0.39158	0.18753
0.08663	0.1774	0.2203	0.16728	0.09938	0.27338	0.04036	0.03511	0.16229	0.00725	-0.16191	-0.04456	-0.18933	0.04799	-0.00462	-0.29382	0.16756
0.1137	0.2088	0.22215	0.15786	0.08519	0.25976	0.01414	0.00786	0.11577	0.00725	-0.10859	-0.03686	-0.12391	0.04766	0.03135	-0.28959	0.17892
0.12741	0.21074	0.2183	0.16395	0.10902	0.27506	0.04256	0.08168	0.15378	-0.06001	-0.10285	-0.02798	-0.15438	0.04714	0.00234	-0.3715	0.17068
0.12628	0.17401	0.22324	0.16046	0.09562	0.26307	0.03226	-0.01557	0.16721	-0.01673	-0.15823	-0.03155	-0.22884	0.03945	0.00147	-0.40831	0.18364
0.09943	0.19781	0.23191	0.16806	0.091	0.25497	0.01623	-0.01367	0.21264	0.02636	-0.21796	-0.05708	-0.18834	0.08259	0.03614	-0.44372	0.15184

SPLICING	C1_MEDI	MTOR_S	COPOLYS	USCLE_C	INDEPEND	ONIC_EPI	ME_MYOG	TERNAL_	TOME_M	RDULATES	CAM1_IN	ING_FOR	CL_TRANS	ME_NEED	ND_SIGN
0.21087	0.12411	0.06784	0.06947	0.23994	0.04062	0.24321	0.0676	0.15149	0.19836	0.09667	-0.24169	0.09071	-0.03031	0.14823	0.03616
0.26316	0.12646	0.07641	0.01176	0.22555	0.02234	0.19914	0.03484	0.18638	0.20693	0.11146	-0.15157	0.08658	-0.12586	0.14791	-0.02007
0.218	0.11559	0.07894	0.05691	0.23981	0.02741	0.21438	0.08547	0.18427	0.19963	0.01691	-0.17827	0.11551	-0.01058	0.13251	0.05401
0.19423	0.16414	0.11065	0.08233	0.25046	0.02389	0.23351	0.04587	0.14731	0.19128	0.06315	-0.27146	0.05008	-0.14196	0.12596	0.12715
0.2034	0.12591	0.08241	0.13151	0.21594	0.04514	0.26135	0.0292	0.12727	0.20318	0.12413	-0.22375	0.09545	-0.21494	0.16115	0.16097
0.21636	0.13262	0.10106	0.09664	0.23184	0.01738	0.26121	0.03992	0.14788	0.19673	0.10524	-0.08715	0.09079	-0.17562	0.13429	0.12181
0.20318	0.12836	0.07984	0.10562	0.2184	0.02848	0.21319	0.07265	0.15325	0.18198	0.04933	0.04807	0.13452	-0.08585	0.12708	0.13138
0.1612	0.17464	0.11831	0.11213	0.24627	0.05595	0.25913	0.00442	0.1158	0.18998	0.13175	-0.26219	0.06452	-0.1405	0.13294	0.14112
0.17701	0.1593	0.11252	0.1518	0.24795	0.03877	0.23549	0.0652	0.11062	0.19093	0.16054	-0.02455	0.11234	-0.15854	0.13171	0.16534
0.18533	0.14349	0.10495	0.12081	0.23694	0.0617	0.23611	0.00239	0.12621	0.18894	0.13365	-0.23002	0.06969	-0.1878	0.1293	0.19338
0.1948	0.12662	0.06564	-0.03189	0.21934	0.01897	0.24025	0.05203	0.18293	0.19326	0.12308	-0.15987	0.07835	0.00659	0.15796	-0.02456
0.19615	0.08893	0.05391	0.12757	0.23051	0.02801	0.25227	0.03312	0.13259	0.19034	0.05019	-0.18598	0.08017	-0.16846	0.14186	0.06912
0.21709	0.16119	0.11313	-0.04004	0.22082	0.02839	0.32337	0.04709	0.18192	0.21847	0.08616	-0.1192	0.12323	0.11372	0.15802	-0.06381
0.18992	0.13904	0.08182	0.20306	0.22104	0.0395	0.28644	0.05108	0.15494	0.20002	0.04122	-0.24626	0.07986	-0.10548	0.14269	0.11091
0.20769	0.13776	0.08961	0.07336	0.2392	0.0417	0.28119	0.10016	0.13035	0.19314	0.1339	-0.22299	0.07508	-0.11497	0.14758	0.03109
0.19179	0.11049	0.07248	0.0139	0.22065	0.01243	0.18759	0.04978	0.17335	0.19941	0.02839	-0.10568	0.12048	0.13618	0.14648	-0.05309
0.21454	0.14	0.09777	0.16937	0.23076	0.01808	0.25171	0.00988	0.16277	0.19673	0.10802	-0.24293	0.09747	-0.06648	0.12675	0.10227
0.18205	0.14668	0.09562	0.11059	0.23547	0.03188	0.2495	0.05547	0.13443	0.19734	0.11512	-0.2326	0.08348	-0.16134	0.14198	0.16069

ATED_CD	ATED_CD	F_MHC_C	URFACE	IC_REGU	ACK_REG	ITY_OF_T	GULATIO	EGULATIO	EGULATIO	EGULATIO	IVE_REG	REGULATI	REGULAT	DA_RECE	GULATION
0.11652	0.20901	0.15769	0.13911	0.29731	0.32123	0.39088	0.28399	0.28399	0.28399	0.28399	0.15596	0.19122	0.10494	0.05986	0.29324
0.12441	0.22429	0.10269	0.11279	0.31101	0.20426	0.38987	0.26749	0.26749	0.26749	0.26749	0.13926	0.14187	0.02908	0.08964	0.29199
0.17931	0.29204	0.13309	0.15533	0.29785	0.27673	0.42741	0.31383	0.31383	0.31383	0.31383	0.23639	0.19191	0.10218	0.04422	0.26833
0.2099	0.32183	0.16699	0.18742	0.27735	0.26087	0.37486	0.30492	0.30492	0.30492	0.30492	0.2601	0.18601	0.13936	0.03614	0.2774
0.20631	0.29302	0.17557	0.19015	0.28325	0.2946	0.39988	0.30433	0.30433	0.30433	0.30433	0.272	0.19535	0.11746	-0.0213	0.30335
0.16935	0.22525	0.16595	0.16775	0.30452	0.16198	0.37763	0.24024	0.24024	0.24024	0.24024	0.17361	0.14804	0.05107	0.03925	0.2917
0.19682	0.2336	0.17423	0.18525	0.31251	0.27718	0.43173	0.27702	0.27702	0.27702	0.27702	0.18952	0.14651	0.08	-0.01429	0.27927
0.23787	0.34178	0.19958	0.21814	0.2601	0.28714	0.29336	0.32139	0.32139	0.32139	0.32139	0.27586	0.16489	0.14858	0.05802	0.29286
0.2417	0.33396	0.1955	0.21793	0.26916	0.25172	0.37537	0.31639	0.31639	0.31639	0.31639	0.30964	0.1581	0.15029	0.03506	0.29313
0.22959	0.29693	0.17664	0.20247	0.27996	0.28022	0.37841	0.31082	0.31082	0.31082	0.31082	0.28499	0.1598	0.13806	0.00034	0.28354
0.10455	0.19563	0.1022	0.10334	0.30088	0.20863	0.41612	0.24832	0.24832	0.24832	0.24832	0.18024	0.13894	0.04226	-0.06763	0.28343
0.1262	0.21627	0.1518	0.14005	0.29748	0.26255	0.43571	0.26809	0.26809	0.26809	0.26809	0.192	0.15721	0.05636	0.03039	0.28346
0.16029	0.27081	0.1178	0.13752	0.30273	0.25859	0.41518	0.28515	0.28515	0.28515	0.28515	0.05722	0.21987	0.00612	-0.01153	0.29245
0.2024	0.29448	0.10129	0.15045	0.2941	0.24074	0.3671	0.27664	0.27664	0.27664	0.27664	0.21003	0.16287	0.1104	0.00709	0.28596
0.13804	0.23136	0.1385	0.13842	0.2841	0.31793	0.39024	0.29168	0.29168	0.29168	0.29168	0.15521	0.16343	0.05726	0.02664	0.27727
0.09852	0.18715	0.08766	0.09266	0.29979	0.28422	0.41808	0.2591	0.2591	0.2591	0.2591	0.11991	0.1933	0.06284	0.03538	0.2734
0.20972	0.30716	0.13822	0.1725	0.2939	0.23976	0.38595	0.28665	0.28665	0.28665	0.28665	0.19825	0.18327	0.11317	-0.00947	0.27344
0.25717	0.33539	0.18575	0.2204	0.27027	0.28236	0.39001	0.30965	0.30965	0.30965	0.30965	0.2669	0.15799	0.1134	-0.00046	0.29811

EPENDEN	LATION_O	LATORS_	RIN_FA	MIUS_SYS	NETRIN_	EDIATED	EXINS_AN	ROFASCI	NEURON	ICITY_OF	TRANSM	ITORS_AN	RANSMITT	ROPHIL_	_PENTOS	ANTI_OX
-0.21156	0.03094	0.11339	0.28134	0.23236	0.16351	0.08306	0.09848	-0.01892	0.12404	0.36445	0.33457	0.13671	0.09188	0.20717	0.44202	0.21203
-0.40021	-0.04513	0.11988	0.21668	0.24353	0.08796	0.08354	0.1252	0.04268	0.13109	0.35318	0.30067	0.16084	0.06436	0.15745	0.43936	0.18438
-0.4328	0.02081	0.10701	0.281	0.2396	0.0951	0.11386	0.01529	0.10496	0.12893	0.34825	0.38366	0.15784	0.09901	0.18565	0.44462	0.23226
-0.4439	0.04732	0.10085	0.21951	0.24147	0.05526	0.1245	0.02031	0.08858	0.14566	0.39281	0.39476	0.17395	0.01634	0.24338	0.44547	0.21375
-0.41377	0.04357	0.10502	0.20232	0.25128	0.05446	0.10537	-0.00815	0.29779	0.13773	0.36868	0.37212	0.16318	0.0366	0.23322	0.46157	0.2447
-0.29523	0.01168	0.11986	0.22519	0.23989	0.07903	0.0896	-0.02609	0.15517	0.09639	0.33081	0.2123	0.12601	0.01145	0.17795	0.43285	0.20867
-0.38876	0.01895	0.12611	0.26782	0.23962	0.10437	0.05401	-0.01023	-0.15486	0.10657	0.26651	0.33686	0.14561	0.03343	0.19478	0.44297	0.16635
-0.41289	0.04716	0.12008	0.21138	0.24726	0.06523	0.15895	0.01159	0.25608	0.15588	0.35688	0.41696	0.17504	0.0398	0.25867	0.45432	0.24065
-0.41008	0.05945	0.13215	0.22265	0.25046	0.06042	0.12448	0.00875	0.23306	0.14217	0.34014	0.41213	0.17546	0.00703	0.24814	0.45484	0.21851
-0.38982	0.0477	0.14501	0.18713	0.23909	0.08946	0.15111	-0.02654	0.11972	0.14123	0.34754	0.39872	0.16445	0.02576	0.25001	0.45699	0.22021
-0.33468	0.00545	0.11818	0.27264	0.23696	0.05792	-0.00426	0.00504	-0.21071	0.10655	0.26968	0.26238	0.13506	0.0672	0.18804	0.43882	0.18032
-0.4476	0.01877	0.12457	0.26179	0.23951	0.07516	-0.00988	0.01543	-0.06843	0.11033	0.26299	0.23004	0.1489	0.03335	0.18403	0.43076	0.17434
0.0192	-0.00428	0.12471	0.27677	0.25596	0.09925	0.02275	0.09618	-0.0896	0.14352	0.40585	0.16131	0.18168	0.06307	0.12222	0.39691	0.16624
-0.45024	0.02418	0.10271	0.24139	0.24487	0.07899	0.02507	0.02286	0.03287	0.12925	0.32429	0.39898	0.15651	0.07303	0.23028	0.4602	0.19887
-0.45288	0.00459	0.12139	0.24942	0.24052	0.07261	0.09309	0.01437	0.03969	0.12036	0.31161	0.36978	0.14917	0.09113	0.22592	0.45436	0.22302
-0.19218	-0.0172	0.15387	0.28024	0.24046	0.07082	-0.01324	-0.00141	-0.25831	0.09719	0.29523	0.28092	0.1315	0.08497	0.12647	0.41724	0.18043
-0.45041	0.04121	0.07943	0.26781	0.24928	0.08307	0.0727	0.0113	0.0974	0.14134	0.33292	0.32988	0.17904	0.01543	0.21681	0.4405	0.18832
-0.42329	0.05396	0.11302	0.21797	0.24533	0.05265	0.10294	0.03498	0.22308	0.14794	0.38911	0.41352	0.17538	0.04331	0.22468	0.45533	0.21905

ATING_M	ULATING	DD_RIP_	TIVATED	TIMULATE	OTINAMI	COTINAT	1_2_SIGN	NICAL_A	OLOGOUS	NSE_MED	N_MEMBR	NEURO
-0.30216	-0.09583	-0.07099	0.18469	-0.06179	0.09716	0.02439	-0.00582	0.37569	0.33454	0.35636	-0.02197	0.13771
-0.16362	0.02801	-0.00776	0.12639	0.02313	0.0345	-0.0376	-0.03106	0.36769	0.35204	0.37438	-0.00819	0.12457
-0.18555	-0.04192	-0.05245	0.08878	-0.07479	0.09867	0.01153	-0.03698	0.30094	0.34389	0.35767	-0.04117	0.14168
-0.10459	-0.28624	-0.00977	0.1333	-0.09165	0.12941	0.04238	0.01887	0.39332	0.33397	0.37169	-0.00453	0.15386
-0.15827	-0.07328	0.00014	0.18357	-0.07392	0.0295	-0.01695	0.00846	0.32506	0.33891	0.36371	-0.05451	0.13243
-0.03738	-0.18161	-0.06911	0.10544	-0.01818	0.10713	-0.01604	-0.06316	0.32844	0.33641	0.37175	-0.01055	0.07561
-0.04291	0.01938	-0.0573	0.16723	-0.10354	0.10052	0.01391	-0.01542	0.1878	0.33943	0.36777	0.05562	0.03521
-0.13501	-0.17315	-0.02148	0.14701	-0.09292	0.17542	0.07657	0.01484	0.3586	0.32745	0.36154	-0.02743	0.08897
-0.15181	-0.15783	-0.02446	0.12006	-0.06735	0.16995	0.08069	0.00775	0.33946	0.32774	0.35759	0.00915	0.09882
-0.18441	-0.42769	0.00088	0.15271	-0.09338	0.09735	0.03859	0.01985	0.34306	0.33286	0.35479	-0.05722	0.0787
-0.00565	0.25912	-0.09096	0.11876	-0.05802	0.09164	0.01788	-0.01944	0.26142	0.35226	0.36904	-0.0245	0.09284
-0.15591	0.10217	-0.06268	0.14145	0.01629	0.14274	0.02107	-0.00795	0.30957	0.34192	0.36313	0.01075	0.0386
-0.19748	0.09864	-0.04237	0.03948	-0.04878	0.11862	0.02875	-0.02201	0.33616	0.35442	0.36797	0.04096	0.16278
-0.06911	-0.07715	-0.01987	0.12923	-0.02515	0.10626	0.02799	0.00502	0.28028	0.33946	0.37247	-0.04293	0.06671
-0.14964	-0.21719	-0.03302	0.16639	-0.05758	0.13191	0.06008	-0.02162	0.34399	0.34344	0.37813	-0.04754	0.07389
-0.05645	0.15959	-0.07081	0.08965	-0.03531	0.11626	0.02629	-0.01565	0.26193	0.36391	0.36818	0.03896	0.10208
-0.09539	-0.23005	-0.0596	0.10696	-0.02036	0.05235	-0.0698	-0.036	0.34508	0.34386	0.37377	-0.00804	0.06105
-0.19499	-0.00793	-0.02344	0.14745	-0.09598	0.1415	0.05674	-0.00322	0.33254	0.3458	0.35709	-0.01307	0.11277

_TRANSMI	LAR_DOM	TRANSMI	LAR_DOM	LH_TRAN	ES_EXPR	D_NR1H3	NE_EXPR	GENE_E	XESSION_L	EXPRESS	NE_EXPR	ISSION_LI	GNALS_D	IRCAM_IN	CELL_	DED_EFFEC	TRK2_AC	VR_ENVE	_ENVEL	OE_AND_T
0.28376	-0.07925	0.34797	-0.1925	-0.11065	0.18051	-0.02518	-0.01199	0.13372	-0.13511	-0.25179	-0.13511	-0.06897	-0.13299	-0.00146	0.3108	0.07695	0.44707	0.1096	0.17253	0.07602
0.2237	0.05356	0.24422	-0.12125	-0.02783	0.22429	-0.00146	-0.1738	0.15437	-0.02642	-0.13942	-0.02642	-0.01001	-0.15753	0.0696	0.24614	0.101	0.41624	0.12383	0.16565	0.08308
0.18982	0.06554	0.35674	-0.12614	-0.02693	0.17957	0.02398	-0.32328	0.08493	-0.26193	-0.24866	-0.26193	0.01853	-0.10706	-0.00429	0.2147	0.13451	0.44354	0.14829	0.17841	0.07084
0.25856	-0.01043	0.3559	-0.19102	-0.08406	0.16721	0.04845	-0.4586	0.05776	-0.45799	-0.31602	-0.45799	0.04741	-0.07281	-0.14051	0.26964	0.05734	0.45587	0.07721	0.15888	0.06734
0.17006	0.06477	0.27712	-0.12598	-0.06377	0.15877	0.0152	-0.31835	0.01757	-0.30069	-0.24454	-0.30069	0.01601	-0.08489	-0.06369	0.28971	0.07837	0.46433	0.10271	0.16471	0.06787
0.19719	0.06118	0.27984	-0.15573	-0.05694	0.15492	0.058	-0.33915	0.11016	-0.33733	-0.31048	-0.33733	0.05111	-0.12791	0.02083	0.21048	0.10729	0.44918	0.10324	0.15553	0.08364
0.19197	-0.0795	0.31278	-0.23303	-0.0762	0.1611	0.03563	-0.36032	0.14134	-0.41448	-0.28493	-0.41448	0.02277	-0.12683	-0.14563	0.25827	0.08986	0.44213	0.09846	0.14312	0.03865
0.21748	0.01374	0.3329	-0.12011	-0.11123	0.16469	0.03645	-0.43191	0.06338	-0.43069	-0.31095	-0.43069	0.03321	-0.06025	-0.07642	0.2352	0.02899	0.46609	0.03873	0.14896	0.0686
0.19221	0.01255	0.32305	-0.16843	-0.129	0.14634	0.03077	-0.3914	0.01632	-0.36023	-0.30148	-0.36023	0.03066	-0.04721	-0.09546	0.25002	0.0435	0.45922	0.05749	0.15605	0.079
0.19419	0.04579	0.30938	-0.12061	-0.09402	0.15832	0.038	-0.27298	0.09647	-0.19834	-0.23697	-0.19834	0.03539	-0.05895	-0.08306	0.24324	0.068	0.46768	0.0693	0.1501	0.08082
0.25949	0.00203	0.27289	-0.12893	-0.01658	0.20583	0.01083	-0.28051	0.01657	-0.18302	-0.23154	-0.18302	0.0134	-0.16746	-0.09185	0.26399	0.08606	0.42593	0.089	0.15993	0.03923
0.17073	-0.01368	0.3033	-0.18085	-0.07006	0.25461	0.0712	-0.31499	0.09037	-0.23111	-0.29044	-0.23111	0.06838	-0.1607	-0.03785	0.31119	0.10924	0.4557	0.09696	0.15039	0.10877
0.251	-0.00343	0.33542	-0.14133	-0.00868	0.19851	0.05108	-0.30212	0.12698	-0.2112	-0.25756	-0.2112	0.04215	-0.14234	0.03551	0.20158	0.11474	0.43227	0.12318	0.20733	0.10218
0.20396	0.01476	0.29651	-0.15687	-0.09238	0.19251	0.04367	-0.17101	0.07413	-0.36886	-0.32714	-0.36886	0.02244	-0.11058	-0.10505	0.23102	0.09815	0.45499	0.11015	0.17397	0.09002
0.19798	-0.00586	0.31243	-0.12102	-0.05689	0.19388	0.01406	-0.33905	0.111	-0.26616	-0.30302	-0.26616	-0.00077	-0.15098	-0.09198	0.29442	0.062	0.4402	0.08265	0.16283	0.07237
0.25141	0.00676	0.34485	-0.12813	-0.06131	0.24167	-0.07451	-0.31864	0.12673	-0.23498	-0.22581	-0.23498	-0.10318	-0.16169	-0.07575	0.22911	0.10432	0.43421	0.10665	0.16964	0.05987
0.21325	0.01729	0.30935	-0.17144	-0.05949	0.20451	0.04009	-0.42092	0.08272	-0.39035	-0.24119	-0.39035	0.03032	-0.10722	-0.09788	0.23698	0.06779	0.45253	0.08336	0.17087	0.07174
0.18962	-0.0033	0.2971	-0.18781	-0.12441	0.16761	0.00575	-0.35743	-0.01144	-0.31883	-0.28704	-0.31883	0.00966	-0.07756	-0.11267	0.23867	0.06425	0.46151	0.08292	0.17565	0.0559

EVENTS_1	MULATED	R_IMPOR	RE_COM	EPTOR_	EAR_SIGN	CH_REPE	LEOTIDE	CLEOTID	EOTIDE_B	UCLEOT	CAN_ANTE	ELONGAT	IG_AND_E	THE_ER_	S_ANTIVI	RTORY_SI
0.25541	0.28058	0.11682	0.05713	-0.23114	0.01972	0.02513	0.17001	-0.04942	0.0607	0.06189	-0.36563	-0.11368	-0.20074	0.28507	0.09447	0.21428
0.25122	0.26078	0.12658	0.06836	-0.23489	0.01322	0.01522	0.13831	-0.02754	0.11042	0.05505	-0.20612	-0.14737	-0.23399	0.27119	0.19274	0.21099
0.24088	0.23992	0.15095	0.10776	-0.22933	0.04129	0.00735	0.19825	-0.0178	0.06791	0.08201	-0.31671	-0.05767	-0.1861	0.26201	0.21719	0.15167
0.24273	0.2423	0.07988	0.02707	-0.30024	0.08021	0.05137	0.14435	0.03691	0.04412	0.09545	-0.30038	-0.03368	-0.10405	0.27718	0.24188	0.13447
0.27417	0.27318	0.105	0.05513	-0.24649	0.0822	0.04991	0.17519	0.03677	0.07203	0.14713	-0.24454	-0.08018	-0.11596	0.28389	0.20909	0.18553
0.25481	0.23913	0.11111	0.05312	-0.26626	0.05438	-0.02032	0.16949	-0.03426	0.09277	0.09612	-0.24799	-0.04351	-0.18509	0.27764	0.21005	0.16489
0.24164	0.23852	0.1064	0.0586	-0.28796	0.07748	0.0371	0.18322	0.01761	0.07796	0.11191	-0.13992	-0.05684	-0.17029	0.23881	0.25746	0.2221
0.25981	0.24472	0.04308	-0.01395	-0.26808	0.08823	0.0653	0.14154	0.03843	0.01842	0.13226	-0.24074	-0.01914	0.02614	0.28943	0.22828	0.09436
0.2577	0.25368	0.05883	0.00606	-0.27615	0.08583	0.04606	0.15501	0.0461	0.02205	0.11477	-0.28119	0.01774	-0.05933	0.28907	0.25237	0.14417
0.25257	0.25203	0.07504	0.02056	-0.27778	0.08484	0.06525	0.16896	0.02343	0.06003	0.12172	-0.22739	-0.07334	-0.10252	0.26995	0.30583	0.12675
0.25405	0.22693	0.0972	0.03986	-0.23344	0.0239	0.022	0.24732	0.01958	0.07646	0.06905	-0.10357	-0.03161	-0.26558	0.26565	0.06842	0.21116
0.24935	0.23997	0.12572	0.07523	-0.28481	0.02454	0.01957	0.2185	0.06247	0.06456	0.06193	-0.13273	-0.11431	-0.2458	0.2785	0.20154	0.10647
0.24673	0.2309	0.13902	0.08498	-0.20766	-0.00404	0.00805	0.22956	-0.06404	0.10254	0.0757	-0.19012	-0.03516	-0.29128	0.25758	0.12871	0.22787
0.24445	0.25506	0.12917	0.06613	-0.27528	0.04459	0.03989	0.16989	0.02334	0.04153	0.1184	-0.25213	-0.05246	-0.17556	0.26583	0.19475	0.11966
0.24776	0.22549	0.08573	0.02666	-0.29556	0.07085	0.01369	0.19781	-0.02203	0.05993	0.06882	-0.24483	-0.08045	-0.22829	0.27768	0.19753	0.17511
0.23289	0.21783	0.11831	0.06349	-0.2164	0.00273	0.01858	0.25126	-0.00211	0.07952	0.06932	-0.42818	-0.21234	-0.29621	0.27252	0.18391	0.14325
0.23941	0.24228	0.1028	0.04267	-0.26758	0.0471	0.01931	0.15577	-0.03023	0.03304	0.06014	-0.27729	-0.10787	-0.17977	0.2678	0.12806	0.13102
0.26096	0.25759	0.08219	0.02684	-0.28832	0.07414	0.04059	0.15008	0.02676	0.0376	0.13019	-0.29166	-0.12613	-0.07766	0.28201	0.28239	0.21455

ENE_INDU	DGENIC_	OPIOID_	REMOVAL_	BIOGEN	NIC_ANIO	R_INTERL	SEMAPHO	_TUMOR_	STRESS_	OF_TSR_	LINKED_	D_GLYCOS	_TO_MAPK	P38MAP	LY_REGU
0.14853	0.20524	0.2018	0.2769	0.13978	0.01146	0.12733	0.24159	0.1354	0.29462	-0.10221	-0.07939	-0.04906	0.25091	0.1867	0.1507
0.14626	0.20494	0.1801	0.25622	0.14471	0.02065	0.06132	0.1724	0.14317	0.30467	-0.17512	-0.06978	-0.02994	0.24583	0.11346	0.27996
0.15739	0.2187	0.18166	0.26754	0.14293	-0.01473	0.07996	0.25234	0.11218	0.2901	-0.17178	-0.05426	0.02055	0.30081	0.08777	0.20211
0.09827	0.23033	0.22421	0.26585	0.13352	-0.03124	0.1397	0.35469	0.10347	0.27964	-0.2002	-0.13266	-0.08084	0.31573	0.15673	0.17696
0.09808	0.23818	0.21093	0.28915	0.14579	0.00059	0.13827	0.28147	0.13989	0.28876	-0.20529	-0.18266	-0.15689	0.31079	0.15968	0.15195
0.01986	0.19602	0.19235	0.28733	0.12818	-0.00665	0.07426	0.23038	0.06959	0.27527	-0.04178	-0.06868	-0.07762	0.28497	0.11205	0.26701
0.06674	0.2141	0.18362	0.26184	0.13186	0.15772	0.0803	0.27219	0.10257	0.29012	0.12142	-0.0085	-0.11541	0.32035	0.13593	0.2211
0.08573	0.23356	0.20454	0.26056	0.13896	-0.01734	0.15579	0.33869	0.10795	0.27579	-0.13072	-0.1264	-0.10431	0.33208	0.10562	0.11636
0.08661	0.22464	0.18861	0.2683	0.13851	-0.01004	0.14525	0.31782	0.11674	0.27834	-0.09387	-0.15033	-0.16974	0.32205	0.11256	0.12732
0.08227	0.23632	0.21296	0.25042	0.13654	0.00619	0.14891	0.32064	0.1225	0.2835	-0.17452	-0.18338	-0.17449	0.32693	0.13078	0.13839
0.15057	0.18672	0.15507	0.29175	0.15876	-0.0283	0.0508	0.13007	0.1031	0.28668	-0.11634	-0.05808	-0.00077	0.29705	0.08047	0.22274
0.16207	0.20641	0.21414	0.28062	0.14138	0.05366	0.10434	0.24784	0.13123	0.29635	-0.20134	-0.05954	0.02883	0.28818	0.09949	0.18522
0.16556	0.19446	0.18834	0.28577	0.16832	0.00848	-0.0187	0.0097	0.10751	0.29001	-0.13042	-0.07172	-0.06208	0.27669	0.13443	0.28224
0.0947	0.23431	0.18039	0.24304	0.14348	-0.00478	0.11178	0.29248	0.10225	0.28507	-0.1353	-0.08962	-0.05458	0.3283	0.14765	0.20034
0.12306	0.21715	0.17158	0.2738	0.13908	-0.02837	0.11857	0.18058	0.11332	0.27983	-0.13033	-0.09416	-0.06528	0.30861	0.06069	0.16089
0.0886	0.20042	0.16115	0.27534	0.15842	0.07764	0.02434	0.05486	0.14011	0.27657	-0.14572	-0.05571	-0.0382	0.25959	0.099	0.20547
0.14433	0.22126	0.20898	0.22672	0.15481	-0.06834	0.10957	0.30001	0.09651	0.28608	-0.1561	-0.07054	-0.01526	0.31382	0.14244	0.19703
0.11227	0.22201	0.20864	0.271	0.14084	0.01378	0.1438	0.29837	0.13127	0.28355	-0.20263	-0.18472	-0.17072	0.32628	0.11892	0.12605

RUITS_SI	REGULAN	TR_SIGN	CEPTOR_	PARACET	PARASITE	_LONG_P	_PCP_CE	E_PD_1_	PECAM1_I	NOSE_PHO	E_HORM	LIGAND_	EGULATES	ISOMAL_	XISOMAL_	OME_PEX
0.12285	0.40501	0.11916	0.00904	-0.03522	0.17176	-0.01402	0.27691	0.01762	-0.02036	0.19697	0.1346	-0.00187	0.01831	-0.03356	0.06606	0.11018
0.0914	0.39186	0.06674	-0.01038	-0.10228	0.13016	0.0932	0.27512	-0.01413	-0.03684	0.19786	0.04528	0.00247	0.05478	-0.05306	0.07739	0.2058
0.04564	0.41553	0.05369	0.00231	0.00806	0.16453	0.07156	0.2711	0.03171	0.01885	0.23796	0.08387	-0.02183	0.01974	-0.08386	0.04303	0.02934
0.10599	0.42787	0.08767	0.03778	-0.00178	0.20236	0.0356	0.28245	0.08222	0.03892	0.20227	0.15445	0.06994	0.00279	-0.0398	0.0608	0.1055
0.14757	0.4284	0.14458	0.0366	0.05102	0.17754	0.02053	0.30412	0.16768	0.0366	0.24489	0.12317	0.00118	0.0128	-0.07823	0.05005	0.13432
0.0634	0.39992	0.08236	0.0054	-0.01851	0.1497	0.07548	0.28203	0.1154	0.01443	0.22255	0.11257	0.08928	0.01098	-0.09999	0.0512	0.13202
0.132	0.40143	0.13825	0.0143	0.01844	0.18409	0.04784	0.27169	0.1294	0.0446	0.22628	0.1	0.13075	0.03498	-0.08314	0.06558	0.11415
0.08865	0.4342	0.09364	0.0435	0.06838	0.21592	-0.0188	0.30123	0.10352	0.06443	0.24497	0.15318	0.09772	-0.01919	-0.06675	0.0753	0.0758
0.07075	0.4394	0.06879	0.04788	0.0167	0.20704	-0.01597	0.30142	0.18853	0.05508	0.23715	0.14509	0.13218	-0.01103	-0.06924	0.04596	0.0749
0.07508	0.43203	0.09415	0.04615	0.04449	0.20983	0.03295	0.29262	0.16346	0.07545	0.22962	0.15693	0.11993	0.0105	-0.09919	0.04226	0.09718
0.10613	0.37341	0.09073	-0.0197	0.00108	0.13832	0.05304	0.26252	0.01148	-0.09198	0.23324	0.08646	0.04236	0.02225	-0.04371	0.08828	0.10559
0.1233	0.42571	0.10294	-0.01268	0.00453	0.1563	0.06126	0.27997	0.10042	-0.01935	0.21372	0.10862	0.05139	0.01037	-0.06137	0.07308	0.11209
0.09321	0.40011	0.06562	-0.00684	-0.02892	0.15335	0.07345	0.27745	-0.10399	-0.0519	0.21779	0.03864	0.01603	0.00808	-0.05685	0.07526	0.04869
0.11825	0.42119	0.10423	0.01431	0.07501	0.18718	-0.01658	0.28307	0.1033	0.00215	0.23199	0.1137	0.04588	0.0219	-0.00584	0.10188	0.04891
0.14035	0.41301	0.12263	0.00342	0.12521	0.16384	0.04512	0.27493	0.12206	-0.01722	0.23056	0.09993	0.04684	0.07399	-0.10896	0.0318	0.13948
0.08768	0.40889	0.07228	-0.02438	0.07984	0.1388	0.06836	0.25705	0.0136	-0.13024	0.21361	0.06147	-0.05341	0.04473	0.02321	0.11217	0.01621
0.10242	0.41753	0.08438	0.01702	0.03065	0.19501	-0.04938	0.27899	0.06303	0.01127	0.21489	0.09803	0.06901	-0.01362	-0.08021	0.05629	0.10039
0.08018	0.43116	0.09327	0.03008	0.02525	0.1966	-0.03712	0.30383	0.15527	0.04741	0.239	0.15809	0.04432	-0.01827	-0.05226	0.04957	0.07162

0_RAPID	ASE_2_PL	_3_RAPID	CONJUGA	CTIONAL	INE_AND	NYLALANI	_HYDRO	D_HYDR	SE_C_M	SE_C_M	SE_C_M	SPHOLIP	SPHORYL	HORYLATI	YSIOLOGI	PI3K_AKT
0.28218	0.16487	-0.21473	0.15968	0.02385	0.14957	0.21185	-0.44196	-0.02453	-0.2267	-0.2267	-0.2267	0.01362	0.099	-0.16017	-0.19127	0.01214
0.27338	0.13586	-0.24943	0.07943	0.01642	0.14898	0.25851	-0.39493	-0.1414	0.00846	0.00846	0.00846	-0.01114	0.18337	-0.08174	-0.36024	-0.0515
0.30179	0.14796	-0.29241	0.14511	0.03365	0.18844	0.28216	-0.42205	-0.0836	-0.35512	-0.35512	-0.35512	0.00138	0.24414	-0.16145	-0.35718	0.0893
0.2451	0.1034	-0.37749	0.13612	0.03991	0.1862	0.21408	-0.02008	-0.0363	-0.30826	-0.30826	-0.30826	0.04851	0.02308	-0.14167	0.00034	0.15691
0.22036	0.16059	-0.17509	0.1301	0.01745	0.24235	0.33922	-0.43896	-0.07181	-0.21226	-0.21226	-0.21226	0.02467	0.11626	-0.21445	-0.286	0.06245
0.30028	0.0854	-0.44953	0.10097	0.02347	0.21731	0.31072	-0.34807	-0.15083	0.04844	0.04844	0.04844	-0.00775	0.10164	-0.12664	0.00636	-0.02128
0.25058	0.13431	-0.30509	0.10236	-0.01644	0.1764	0.31851	-0.421	-0.09831	-0.23798	-0.23798	-0.23798	-0.01653	0.17738	-0.16005	-0.30235	0.14289
0.28421	0.13191	-0.28765	0.15425	0.08126	0.17213	0.21731	-0.06782	-0.05202	-0.37766	-0.37766	-0.37766	0.03644	-0.23657	-0.18399	-0.05967	0.14743
0.24703	0.13699	-0.26581	0.14903	0.04076	0.16485	0.25471	0.05584	-0.06824	-0.21649	-0.21649	-0.21649	0.02972	-0.0805	-0.20599	-0.05744	0.17124
0.25571	0.13324	-0.21913	0.11256	0.03064	0.18616	0.26482	-0.11555	-0.06193	-0.26264	-0.26264	-0.26264	0.0252	-0.2038	-0.24174	0.02498	0.0609
0.19014	0.14697	-0.3826	0.13889	0.02469	0.12037	0.2565	-0.41466	0.00502	-0.30333	-0.30333	-0.30333	0.02114	0.22459	-0.09385	-0.32651	0.13508
0.25594	0.13599	-0.34173	0.14962	0.00501	0.1552	0.25498	-0.43368	-0.09898	-0.31813	-0.31813	-0.31813	-0.00998	0.16488	-0.17027	-0.3546	0.03571
0.14396	0.17239	-0.12383	0.10208	-0.04377	0.10292	0.19668	-0.45957	-0.08331	-0.32958	-0.32958	-0.32958	0.00602	0.25912	-0.09027	-0.04089	0.01165
0.25344	0.11822	-0.26828	0.13373	0.0619	0.1537	0.20538	-0.0694	-0.08115	-0.40074	-0.40074	-0.40074	0.03352	-0.2459	-0.20573	-0.4461	0.00634
0.25773	0.12655	-0.41642	0.1678	0.06146	0.22595	0.27074	-0.46891	-0.09355	-0.29646	-0.29646	-0.29646	0.00123	-0.02026	-0.17973	-0.29814	0.06899
0.23772	0.13665	-0.33239	0.16041	-0.00464	0.20223	0.24859	-0.42258	-0.11264	-0.32958	-0.32958	-0.32958	-0.0266	0.3264	-0.09571	-0.33149	-0.03661
0.22788	0.10311	-0.36674	0.10762	0.02186	0.13203	0.19772	-0.22706	-0.12328	-0.32182	-0.32182	-0.32182	0.0323	-0.04069	-0.15567	-0.28403	0.15792
0.24108	0.12442	-0.30773	0.14377	0.07529	0.17322	0.24389	-0.19658	-0.06013	-0.32077	-0.32077	-0.32077	0.02311	-0.0347	-0.21124	-0.05147	0.19497

KT_SIGN	ENTS_IN	ENTS_IN	GULATES	RKN_ME	ACTING_RI	3K_CAS	3K_CAS	3K_CAS	3K_CAS	E_PI_ME	TION_IN_	ED_PHOS	HORYLAT	METHYLAT	R_MEDIAT	A_LIPOP
-0.05895	-0.00165	-0.22542	-0.11511	0.08574	0.02021	0.11097	0.11097	0.11097	0.11097	-0.04388	0.2275	0.23863	0.28506	0.35186	0.22805	0.18211
-0.07969	-0.08475	-0.35676	-0.14073	0.10904	0.0275	0.06871	0.06871	0.06871	0.06871	-0.07153	0.24117	0.26912	0.25727	0.35197	0.2481	0.13119
-0.03636	-0.00432	-0.10088	-0.15961	0.09959	0.01749	0.17155	0.17155	0.17155	0.17155	-0.07107	0.16772	0.19451	0.23171	0.357	0.24587	0.18626
-0.00528	0.03057	0.03611	-0.18753	0.10176	-0.02846	0.22553	0.22553	0.22553	0.22553	-0.0169	0.22228	0.22746	0.31353	0.3368	0.25213	0.26183
-0.01692	-0.0263	-0.16421	-0.06784	0.09101	-0.00475	0.134	0.134	0.134	0.134	-0.02812	0.24481	0.27385	0.30517	0.34963	0.24046	0.23045
-0.03897	-0.06696	-0.21874	-0.09746	0.10874	-0.01539	0.10901	0.10901	0.10901	0.10901	-0.08037	0.22213	0.24094	0.20315	0.35549	0.23024	0.30014
-0.0106	0.01492	0.00956	-0.23421	0.15492	0.02161	0.18354	0.18354	0.18354	0.18354	-0.07204	0.23491	0.22931	0.26427	0.35435	0.23399	0.24087
-0.00802	0.02178	-0.00086	-0.14007	0.03633	-0.06151	0.23324	0.23324	0.23324	0.23324	0.00703	0.25197	0.2636	0.33133	0.33961	0.24276	0.2875
-0.00097	0.01721	0.02641	-0.09919	0.02414	-0.03904	0.21655	0.21655	0.21655	0.21655	0.00308	0.23762	0.25109	0.32528	0.33855	0.24903	0.29392
0.00505	-0.04937	-0.15806	-0.15546	0.07655	-0.00363	0.15898	0.15898	0.15898	0.15898	-0.0193	0.25924	0.26756	0.30696	0.34984	0.24892	0.29086
-0.04854	0.08794	0.02217	-0.08223	0.14176	-0.01983	0.11868	0.11868	0.11868	0.11868	-0.04706	0.18746	0.20932	0.24824	0.35171	0.25895	0.19786
-0.0563	0.00774	-0.22576	-0.08802	0.15325	-0.01545	0.0583	0.0583	0.0583	0.0583	-0.06904	0.25076	0.27712	0.31909	0.34219	0.25477	0.26281
-0.05082	-0.01465	-0.25786	-0.1936	0.15661	0.03105	0.06625	0.06625	0.06625	0.06625	-0.07052	0.24549	0.2141	0.23843	0.34332	0.2739	0.23582
-0.04365	-0.07863	-0.27076	-0.10923	0.07235	-0.04225	0.0541	0.0541	0.0541	0.0541	-0.01912	0.24166	0.25387	0.28079	0.34893	0.25619	0.21289
-0.06988	-0.10247	-0.28948	-0.11604	0.1099	-0.03472	0.09139	0.09139	0.09139	0.09139	-0.06483	0.24401	0.24487	0.28882	0.35125	0.25315	0.25453
-0.05948	0.00851	-0.37885	-0.12726	0.12789	-0.01634	-0.01544	-0.01544	-0.01544	-0.01544	-0.0997	0.17815	0.20329	0.1475	0.34157	0.26053	0.11214
-0.00987	0.06229	0.03494	-0.09052	0.12301	-0.02752	0.21219	0.21219	0.21219	0.21219	-0.02623	0.24056	0.22779	0.28688	0.34581	0.24342	0.23193
0.00203	0.05646	0.07687	-0.13885	0.07687	-0.0247	0.24268	0.24268	0.24268	0.24268	-0.00701	0.22858	0.25018	0.29489	0.34656	0.25347	0.22702

ASSEMBL	_LIPOPR	LIPOPRO	ATION_SI	HESION_	GGREGA	LET_CALC	ATELET_	LET_SENS	_LONG_P	KE_KINAS	LYMERAS	NG_ON_T	IC_REGU	EAR_POR	ONIN_TU
0.19419	0.22456	0.20321	0.16267	0.0556	0.14462	0.23682	0.18115	0.11648	0.15362	0.421	-0.14422	-0.16789	0.30612	0.12898	0.19736
0.17371	0.21274	0.18467	0.14169	0.06235	0.13944	0.21814	0.14305	0.03721	0.23109	0.44918	-0.01803	-0.06237	0.32663	0.12248	0.19768
0.21188	0.24942	0.233	0.16659	0.1218	0.18005	0.18956	0.16222	0.13738	0.17338	0.44548	0.03251	-0.01222	0.32501	0.15173	0.18527
0.27795	0.3144	0.2994	0.23364	0.31136	0.23871	0.2185	0.2156	0.15111	0.13223	0.43579	-0.02124	-0.06381	0.29175	0.08734	0.22668
0.24721	0.27485	0.29013	0.19897	0.15132	0.20625	0.21871	0.19033	0.13844	0.14008	0.40919	-0.06495	-0.08389	0.29289	0.0996	0.20584
0.24599	0.26078	0.34107	0.21768	0.06172	0.16478	0.21361	0.16534	0.12516	0.15912	0.41606	0.02369	-0.00479	0.31063	0.10152	0.19069
0.21899	0.2399	0.28907	0.23595	0.16978	0.20548	0.17165	0.14147	0.07328	0.16919	0.42681	-0.02938	-0.05447	0.31762	0.12097	0.18061
0.29048	0.32118	0.33846	0.24165	0.2638	0.24388	0.19881	0.1909	0.13488	0.09982	0.38524	-0.10736	-0.13789	0.26998	0.06106	0.22484
0.29062	0.31826	0.33983	0.23972	0.23462	0.24085	0.19644	0.18382	0.12911	0.11629	0.40655	-0.08504	-0.11261	0.28024	0.07763	0.22653
0.27756	0.29831	0.34875	0.2353	0.1987	0.22459	0.20587	0.19993	0.14235	0.13599	0.40726	-0.04232	-0.05487	0.28992	0.08299	0.21753
0.16323	0.18181	0.21569	0.16336	-0.00314	0.15458	0.17421	0.12567	0.05546	0.16517	0.4365	0.01766	-0.02626	0.32093	0.1097	0.24296
0.24433	0.25609	0.3166	0.20781	0.0968	0.17467	0.2124	0.16755	0.10964	0.15017	0.42064	0.03352	-0.00215	0.30889	0.13335	0.21524
0.18371	0.20507	0.26239	0.15348	-0.11628	0.15146	0.21366	0.18032	0.11073	0.16001	0.45129	0.03798	-0.01448	0.33462	0.14048	0.2716
0.23299	0.2714	0.25592	0.20005	0.1591	0.21994	0.20941	0.16662	0.11207	0.13153	0.4402	-0.10792	-0.13093	0.30874	0.13455	0.25905
0.21429	0.22804	0.27987	0.1865	0.08408	0.18199	0.17048	0.14735	0.10259	0.16637	0.43051	0.00293	-0.01895	0.3024	0.09643	0.21682
0.11578	0.15232	0.14596	0.11432	-0.00887	0.1387	0.14258	0.10638	0.06486	0.16303	0.44478	0.03538	-0.01892	0.32132	0.12507	0.23812
0.26131	0.2965	0.29899	0.21227	0.17195	0.19808	0.22242	0.20978	0.12281	0.10594	0.43967	-0.14708	-0.17004	0.30689	0.10614	0.24535
0.26432	0.31098	0.28144	0.21015	0.20151	0.22614	0.22387	0.19784	0.12037	0.11674	0.38541	-0.11574	-0.1365	0.28248	0.09816	0.24845

PTIONAL_	CATION_S	ATIONAL_	OTASSIUM	AL_THER	_ACTIVAT	REPRESS	PHORYLA	THYLATES	_PREDNIS	NENOLON	UNCTION	_PHAGO	_EXPRES	TCH_PRO	SSING_IN	NG_AND_
0.06133	-0.06176	0.13223	0.15217	0.13181	0.34349	-0.45235	0.26322	0.42192	0.20155	0.15231	0.15257	0.3284	0.29776	-0.01049	-0.19771	0.35308
0.15822	-0.06934	0.13434	0.16033	0.12151	0.27374	0.05707	0.20518	0.42474	0.198	0.15197	0.13021	0.28565	0.31342	-0.02085	-0.02089	0.36574
0.06553	-0.0326	0.13067	0.12096	0.14293	0.19535	-0.34772	0.29453	0.42459	0.18687	0.17556	0.13727	0.27406	0.31038	-0.022	-0.03508	0.38306
-0.03755	-0.00848	0.13071	0.28644	0.14103	0.24819	-0.46838	0.26729	0.42064	0.17606	0.19874	0.25686	0.31985	0.29808	-0.0035	-0.10137	0.30018
-0.00746	-0.1168	0.13372	0.21935	0.13603	0.30791	-0.15131	0.27947	0.41809	0.16917	0.19067	0.18785	0.31212	0.30822	0.02705	-0.09097	0.35038
-0.02048	-0.07515	0.1296	0.12486	0.13731	0.27057	-0.37432	0.26649	0.42591	0.33694	0.13892	0.09217	0.32762	0.30816	0.0294	-0.06656	0.35559
-0.10008	-0.12983	0.12167	0.17413	0.13696	0.23375	-0.43544	0.18873	0.43189	0.25195	0.19031	0.14598	0.27023	0.30158	0.03491	-0.19658	0.38343
-0.07628	-0.08316	0.13404	0.24207	0.13414	0.33063	-0.44795	0.26394	0.41311	0.25273	0.23795	0.22841	0.32758	0.30151	0.04612	-0.08699	0.27494
-0.14867	-0.04529	0.13187	0.2316	0.13661	0.29593	-0.36874	0.27169	0.41591	0.31004	0.20041	0.21447	0.30786	0.29804	0.02007	-0.04452	0.32468
-0.05606	-0.08319	0.12181	0.26602	0.13728	0.291	-0.43386	0.24725	0.42023	0.21098	0.23284	0.22531	0.30052	0.30013	0.04239	-0.1691	0.32922
0.08276	-0.0442	0.14161	0.10266	0.13181	0.26422	-0.14902	0.22405	0.42605	0.20657	0.07767	0.09683	0.29623	0.31961	0.01277	0.00703	0.3478
0.03315	-0.12828	0.13822	0.15435	0.12379	0.29664	-0.42892	0.26876	0.42655	0.24234	0.17445	0.13233	0.27575	0.31265	0.03287	-0.06914	0.36392
0.13323	-0.07443	0.14693	0.16883	0.14494	0.26951	-0.39228	0.3249	0.42188	0.1998	0.0978	0.14784	0.28716	0.31534	0.01808	-0.09786	0.37868
0.01983	-0.07062	0.13567	0.1651	0.13308	0.26651	-0.31725	0.26466	0.4265	0.17392	0.16894	0.14044	0.29891	0.30831	0.00016	-0.13192	0.29016
0.00638	-0.07051	0.13247	0.1258	0.12959	0.21226	-0.13687	0.24636	0.42229	0.17888	0.14758	0.10349	0.31267	0.30084	-0.01087	-0.12811	0.3267
0.06516	-0.05871	0.12785	0.05855	0.12256	0.22935	-0.31108	0.28675	0.41962	0.16883	0.13372	0.08501	0.30157	0.30142	-0.05745	-0.0947	0.3598
0.06679	-0.07573	0.12768	0.27918	0.13177	0.24855	-0.20169	0.24171	0.42468	0.16786	0.18417	0.24315	0.30344	0.31065	0.00542	-0.04381	0.34323
-0.07893	-0.05715	0.1265	0.25973	0.13511	0.30174	-0.2755	0.23838	0.41548	0.17424	0.25268	0.23376	0.32469	0.29728	0.00952	-0.13979	0.33161

F_CAPPE	APPED_IN	DNA_DO	G_OF_IN	ROCESSI	IS_ON_TH	NTHESIS	GRAMME	TIN_REG	ED_ERK	PIONYL_	CLING_TH
0.12852	0.14186	0.22413	0.09804	0.16275	-0.02937	0.01801	0.2173	0.0703	0.24427	-0.11029	0.20656
0.18886	0.21072	0.25898	0.16651	0.16135	0.04434	0.10472	0.19737	0.00194	0.19476	-0.08887	0.20437
0.12135	0.16269	0.2349	0.08806	0.16195	0.02454	0.07506	0.19948	0.02748	0.22358	-0.10945	0.16555
0.10518	0.12646	0.23029	0.06585	0.11772	0.00725	0.06034	0.20153	0.04542	0.21805	-0.1854	0.30698
0.13352	0.14302	0.23545	0.10433	0.1312	0.02397	0.06373	0.21201	0.06598	0.22533	-0.16108	0.23199
0.15219	0.16217	0.23976	0.13472	0.08708	0.08751	0.13249	0.18808	0.10154	0.18088	-0.16979	0.16641
0.11144	0.14177	0.23158	0.07504	0.16463	0.05424	0.09919	0.1852	0.03263	0.23526	-0.21349	0.20534
0.08218	0.09864	0.21806	0.04634	0.08666	-0.00249	0.05053	0.20748	0.04258	0.26443	-0.13165	0.26356
0.08632	0.10887	0.21187	0.05641	0.03856	-0.00146	0.04558	0.20751	-0.01165	0.21872	-0.17722	0.24924
0.09746	0.12719	0.22757	0.06997	0.0962	0.06014	0.0952	0.20231	0.05891	0.23488	-0.20361	0.27715
0.1417	0.14006	0.24907	0.11974	0.16352	0.0099	0.05943	0.20138	0.03138	0.22853	-0.06311	0.16872
0.11465	0.13951	0.23854	0.08254	0.15943	0.02471	0.06539	0.21432	0.01329	0.22083	-0.11752	0.19829
0.12401	0.16052	0.26114	0.09328	0.16094	0.01503	0.072	0.21451	0.02823	0.23188	-0.29779	0.24576
0.11288	0.13914	0.21871	0.08921	0.1181	-0.05229	-0.01496	0.20159	0.00391	0.22097	-0.06669	0.19484
0.13202	0.14428	0.23778	0.10094	0.12577	0.02574	0.05621	0.20705	-0.01539	0.26472	-0.01679	0.18573
0.14545	0.14935	0.25166	0.12379	0.18776	0.01613	0.07481	0.19869	-0.00559	0.24561	-0.12512	0.12632
0.11972	0.15051	0.21935	0.08436	0.1803	-0.04459	0.00031	0.20839	-0.00404	0.21472	0.02644	0.31869
0.09869	0.12001	0.22578	0.06485	0.0965	-0.04024	-0.00197	0.21436	0.035	0.24738	-0.11634	0.27617

TEIN_INTE	PROTEI	OTEIN_U	LED_MON	PTEN_RE	PTK6_EX	OMOTES_I	REGULAT	OTEINS_0	GTPASES	AND_TH	NALING_II	PURINE_C	OSIDE_M	PURINE	RIMIDINE	PYRIMIDIN	ME_PYRO	YRUVATE	OLISM_A	CHANGE_	GERANY	GULATIO	RAC1_GT	RAC2_GT	RAC3_GT	E_RAF_A	PENDENT_
0.09369	0.14406	0.12751	0.18435	0.16569	-0.09849	0.12042	-0.26369	0.22629	0.03478	0.12872	0.12995	-0.12104	0.21348	0.12707	-0.06444	-0.02736	0.14552	0.16286	0.22891	0.06145	0.13594	0.03826	0.03999	0.10035	0.07078	0.16412	0.14822
0.06891	0.20784	0.13544	0.17045	0.18683	0.00899	0.15088	-0.12883	0.28373	0.03003	0.11513	0.07771	-0.09498	0.17268	0.1097	-0.04848	-0.02744	0.08171	0.12429	0.19732	0.0137	0.07079	-0.00568	-0.00779	0.08014	0.04484	0.12418	0.09191
0.01236	0.14411	0.11907	0.13306	0.16483	-0.02347	0.13972	-0.17965	0.23622	0.05967	0.25148	0.1311	-0.06687	0.23841	0.17174	-0.07175	-0.05607	0.11066	0.16152	0.22716	0.02599	0.0827	0.00771	0.04745	0.10839	0.08902	0.16626	0.1807
0.01333	0.16517	0.1179	0.1874	0.16213	-0.21318	0.13861	-0.2588	0.20543	0.09066	0.17403	0.16858	-0.02613	0.19722	0.14208	-0.01923	0.02396	0.12765	0.12217	0.19801	0.08399	0.14584	0.06583	0.07911	0.11347	0.08701	0.16328	0.10065
-0.00233	0.16674	0.12372	0.08957	0.17491	-0.14961	0.06038	-0.16419	0.24674	0.08822	0.21441	0.16212	-0.01765	0.22975	0.23804	0.02178	0.03483	0.15144	0.10529	0.18617	0.06173	0.12724	0.03908	0.06309	0.10537	0.05732	0.15531	0.1548
-0.03283	0.08266	0.12241	0.08143	0.17203	-0.12576	0.16328	-0.07748	0.23928	0.02751	0.12184	0.09736	-0.06603	0.21902	0.18562	-0.10176	-0.02854	0.08464	0.08536	0.15874	0.02805	0.08303	0.01593	0.04042	0.08736	0.05226	0.15733	0.09673
-0.03577	0.10928	0.09938	0.21035	0.15907	-0.14311	0.0542	-0.02934	0.22535	0.04382	0.15668	0.15098	-0.03171	0.24146	0.19329	-0.03352	0.00583	0.07217	0.13153	0.20417	-0.01374	0.02792	-0.01366	0.04783	0.1021	0.06968	0.11996	0.14631
0.00361	0.18876	0.12036	0.19418	0.16438	-0.11324	0.14525	-0.21731	0.22079	0.10827	0.21533	0.19832	-0.03491	0.1936	0.20644	0.021	0.01593	0.15472	0.12236	0.18756	0.09214	0.14422	0.06744	0.08354	0.12012	0.08713	0.1455	0.12771
-0.01763	0.17326	0.11203	0.12351	0.16508	-0.10762	0.14902	-0.25791	0.2255	0.09012	0.25057	0.20328	-0.03501	0.20642	0.20742	0.02575	-0.01108	0.16909	0.09143	0.16912	0.08356	0.13695	0.05885	0.08675	0.1286	0.08915	0.137	0.10022
-0.01647	0.16726	0.11092	0.14198	0.16558	-0.2763	0.18186	-0.10084	0.20042	0.07931	0.21423	0.19875	-0.06023	0.2273	0.20725	0.01249	0.01672	0.15454	0.11947	0.19286	0.06278	0.10268	0.04977	0.0868	0.11893	0.07901	0.14304	0.10831
0.02514	0.15056	0.14281	0.08569	0.17253	0.0001	0.14268	0.03811	0.35443	0.01825	0.14137	0.12825	0.04471	0.27836	0.15397	-0.08039	-0.07092	0.1032	0.13533	0.2045	0.03234	0.1088	0.00428	-0.00537	0.07989	0.06628	0.10102	0.12695
0.00309	0.12311	0.11556	0.13613	0.16048	-0.05181	0.15707	-0.23529	0.23044	-0.00915	0.1591	0.12962	0.08421	0.26903	0.15102	-0.02129	-0.04413	0.1194	0.15079	0.21725	0.05651	0.09934	0.0253	0.0114	0.0614	0.03151	0.12288	0.13442
0.09366	0.17952	0.14157	0.11643	0.19444	-0.05222	-0.25653	-0.055	0.27475	0.04928	0.15642	0.06319	-0.10738	0.28427	0.13964	-0.11548	0.00811	0.11867	0.1183	0.17197	0.05816	0.12368	0.03454	-0.02561	0.05948	0.04049	0.14348	0.16349
0.00446	0.14307	0.12088	0.13657	0.16251	-0.02899	0.1331	-0.19791	0.23252	0.09397	0.16672	0.16119	-0.043	0.22581	0.1777	-0.02568	0.01511	0.1156	0.14861	0.2193	0.0378	0.0743	0.00659	0.05279	0.1117	0.08299	0.15345	0.08268
0.00253	0.13899	0.12507	0.12942	0.16704	-0.06282	0.06397	-0.13203	0.29245	0.01641	0.13887	0.1316	-0.04753	0.25508	0.1565	-0.11681	-0.06819	0.13318	0.13606	0.18865	0.04265	0.09209	0.02672	0.03072	0.08914	0.05927	0.13417	0.16235
0.001	0.12941	0.1328	0.08708	0.16707	-0.07597	0.1342	-0.28161	0.25905	0.02124	0.15975	0.06527	-0.04234	0.30681	0.1109	-0.00035	-0.01158	0.08616	0.17713	0.24867	0.01403	0.05778	-0.01261	-0.02673	0.06206	0.04892	0.15299	0.18136
0.00477	0.10822	0.10196	0.1738	0.16095	-0.08457	0.15166	-0.29474	0.25018	0.1066	0.15582	0.16046	-0.10188	0.19906	0.12103	-0.05142	-0.03731	0.1395	0.12508	0.21677	0.05721	0.10862	0.03702	0.05819	0.10593	0.07116	0.15051	0.08696
0.001	0.1568	0.11646	0.16031	0.16791	-0.14983	0.1511	-0.2285	0.22537	0.09033	0.24509	0.16148	-0.06309	0.21099	0.2193	0.03392	0.00448	0.14553	0.13425	0.21372	0.0747	0.12715	0.04753	0.07087	0.12099	0.08318	0.12539	0.13816

RAP1_SI	CA2_INF	RAS_PR	REAM_OF	BIOSYNTH	THE_COMP	TOR_MED	TYROSIN	GLYCOS	GE_BY_P	OC_CENTR	F_NUMA	G_OF_BI	CYCLING	YCLING_B	N_OF_C	IN_SIGNA	EGULATE	ED_PROT	EGULATI	ATION_ON	ON_OF_BE	ATION_OF	OF_CDH1
0.38757	0.15052	0.11382	0.19467	0.03246	0.00109	0.06327	-0.06089	0.38639	-0.02622	0.14749	0.15157	0.19445	0.15266	0.23522	0.26326	0.05971	0.10771	0.15601	-0.20669	0.26335	-0.06268	0.38679	0.18713
0.27737	0.17278	0.11613	0.14853	-0.0042	-0.11838	0.07378	-0.19286	0.39517	0.04724	0.16415	0.17429	0.18862	0.16908	0.25744	0.28451	-0.09177	0.07077	0.01794	-0.23677	0.2386	0.01806	0.34016	0.16323
0.2924	0.12201	0.12783	0.18872	-0.00185	0.04225	0.09607	-0.10078	0.39569	0.0407	0.14249	0.15856	0.21535	0.17543	0.27643	0.29165	0.08807	0.08753	0.06133	-0.16575	0.24888	-0.00227	0.38261	0.1507
0.35641	0.0929	0.09325	0.07278	-0.05097	0.06864	0.08228	-0.18796	0.38963	0.01898	0.14209	0.16943	0.21234	0.12563	0.29612	0.23663	0.15836	0.11742	0.09368	-0.13887	0.25283	-0.01351	0.33306	0.20408
0.37325	0.0702	0.12218	0.23952	-0.01531	0.0228	0.07293	-0.1161	0.39584	0.0207	0.15654	0.173	0.17203	0.19124	0.27765	0.27173	0.291	0.13434	0.08164	-0.087	0.24565	0.01992	0.27983	0.15042
0.3479	0.10746	0.06857	0.10246	0.03184	0.03684	0.05845	-0.21544	0.39768	0.05763	0.15003	0.15593	0.17947	0.09458	0.23941	0.3001	0.25066	0.07183	0.03756	-0.11814	0.2767	-0.009	0.3169	0.17941
0.33391	0.05153	0.11786	0.14199	-0.09042	-0.0989	0.10461	-0.17675	0.40913	0.02369	0.1177	0.13551	0.18988	0.09577	0.25124	0.21449	0.23586	0.06734	0.10539	-0.15448	0.25749	0.00851	0.33436	0.17781
0.37462	0.07076	0.06863	0.11332	0.03013	0.08804	0.05751	-0.16531	0.38647	-0.03105	0.15247	0.18132	0.19719	0.14728	0.30484	0.24461	0.18478	0.13142	0.09979	-0.14334	0.21427	-0.01332	0.14446	0.16292
0.34729	0.03546	0.07651	0.10518	-0.02661	0.09736	0.06712	-0.2604	0.38862	-0.04592	0.16366	0.18559	0.19635	0.12714	0.30253	0.25565	0.2341	0.14216	0.07247	-0.11439	0.17507	-0.01936	0.28588	0.20176
0.35993	0.04102	0.05303	0.12569	-0.03012	0.0236	0.09897	-0.14608	0.40034	0.01463	0.14106	0.16754	0.18783	0.11221	0.28545	0.24875	0.30051	0.12784	0.12779	-0.10581	0.20073	0.04298	0.18885	0.20362
0.29365	0.02424	0.14458	0.05416	0.003	0.06	0.12233	-0.08354	0.39397	0.03982	0.15873	0.19383	0.22568	0.17525	0.26596	0.24888	-0.09371	0.08429	0.01784	-0.18275	0.30749	0.01847	0.36312	0.20703
0.38708	0.07771	0.0991	0.10834	-0.00109	-0.09328	0.01115	-0.23515	0.40762	0.04295	0.14562	0.16335	0.2158	0.17541	0.26214	0.25848	0.15114	0.10735	0.06614	-0.12593	0.23472	-0.03454	0.31137	0.19243
0.37106	0.12825	0.14771	0.21049	-0.01194	0.03603	0.0685	-0.04193	0.38694	0.06658	0.18356	0.21441	0.16886	0.17398	0.28922	0.2914	-0.27515	0.10895	-0.01245	-0.10893	0.28433	-0.01305	0.4073	0.19416
0.37064	0.1176	0.13344	0.19696	0.03716	0.04816	0.06077	-0.23273	0.40446	-0.02458	0.14235	0.1718	0.19398	0.13537	0.28981	0.25266	0.18443	0.10248	0.06328	-0.17657	0.25097	-0.03392	0.36287	0.19913
0.36313	0.09104	0.07704	0.11624	0.0319	0.0234	0.06133	-0.20965	0.39803	0.00507	0.15274	0.16983	0.16962	0.18873	0.25825	0.24964	0.05707	0.10485	0.0867	-0.16286	0.19244	-0.0115	0.35392	0.1402
0.34523	0.13684	0.16502	0.20247	-0.06358	-0.14414	0.0725	-0.16075	0.3968	0.0504	0.16016	0.18547	0.21627	0.19643	0.24979	0.23068	-0.34807	0.07325	0.06155	-0.17821	0.25699	0.06071	0.37494	0.20517
0.36084	0.11577	0.10374	0.17501	-0.05373	0.00263	0.07615	-0.20306	0.39989	-0.04306	0.15423	0.18697	0.16778	0.1087	0.2968	0.27586	0.18637	0.11603	0.02992	-0.18658	0.22861	0.03737	0.30187	0.18035
0.38349	0.04722	0.11364	0.07214	0.04906	-0.02893	0.09348	-0.26451	0.3895	-0.02119	0.14556	0.18536	0.16937	0.09533	0.31237	0.24808	0.20909	0.1181	0.0919	-0.13542	0.23041	-0.00372	0.36721	0.14269

H11_MRN	CDH19_E	ESTEROL	URAL_AX	KING_AN	EXPRES	TRANSCR	ON_OF_FZ	XPRESSI	GENE_E	E_BRANC	NASE_BY	S_BY_FRU	HMOX1_	HOMOT	SF1_MED	ION_OF_I	IATION_OF	E_IMMUN	ORT_AND	ION_OF_L	LATION_OF	LIPID_	IZATION_
0.07793	0.38679	-0.02039	0.19796	0.0705	0.34603	0.08989	0.12832	-0.19072	-0.01336	-0.14017	0.06111	0.11759	0.27407	0.2492	0.1272	0.09696	0.12945	-0.01128	0.17185	0.22323	0.07568	-0.041	0.22376
0.14234	0.34016	-0.02926	0.16144	0.02993	0.36211	0.13446	0.10352	-0.14765	0.05431	-0.03675	0.07156	0.09087	0.27717	0.24024	0.14917	0.06135	0.10687	-0.01303	0.13001	0.21248	0.05954	-0.05398	0.18629
0.08265	0.38261	-0.02055	0.01546	0.08666	0.33898	0.05991	0.17148	-0.34379	0.02169	-0.03919	0.10618	0.13085	0.22074	0.23981	0.15003	0.13249	0.11706	-0.0162	0.14575	0.19703	0.11053	-0.05612	0.19891
-0.01349	0.33306	-0.05021	0.15576	0.11506	0.35238	0.15762	0.16875	-0.07926	0.05001	-0.11816	0.02013	0.16249	0.39668	0.1978	0.09338	0.14915	0.13831	-0.00114	0.19498	0.27181	0.12392	-0.01961	0.25187
-0.01409	0.27983	-0.03584	0.02681	0.10855	0.35745	0.10341	0.11206	-0.1256	0.0546	-0.03315	0.04955	0.15402	0.34442	0.1597	0.10738	0.13484	0.1355	0.00349	0.15999	0.23707	0.13787	-0.07569	0.19176
-0.02047	0.3169	-0.04451	-0.03075	0.07049	0.35904	0.00546	0.05737	-0.40147	0.0294	-0.07524	0.05507	0.11642	0.19753	0.18272	0.11884	0.11347	0.1078	-0.05029	0.22022	0.18744	0.07571	-0.05696	0.2229
-0.05296	0.33436	-0.03418	0.23139	0.1029	0.35337	0.15259	0.14122	-0.36595	0.12269	-0.19386	0.05087	0.07108	0.2347	0.18266	0.12176	0.13501	0.12678	0.0042	0.208	0.21407	0.14911	-0.05866	0.20804
-0.0445	0.14446	-0.03367	0.08611	0.13604	0.35182	0.15613	0.14032	-0.22876	-0.0017	-0.03083	-0.01683	0.16203	0.41049	0.08869	0.08717	0.18781	0.12582	0.0156	0.20501	0.25574	0.14223	-0.03302	0.24988
-0.09928	0.28588	-0.0414	0.06435	0.13502	0.3489	0.06873	0.1257	-0.39634	0.02478	-0.08908	0.00152	0.20026	0.36028	0.15156	0.08738	0.1691	0.1449	0.00804	0.19035	0.24004	0.15597	-0.04485	0.20073
-0.02573	0.18885	-0.04132	0.1452	0.11871	0.34658	0.19251	0.1036	-0.44952	0.08936	-0.02917	0.01829	0.12643	0.33551	0.12451	0.09378	0.17699	0.16083	0.01481	0.19671	0.25391	0.17212	-0.03603	0.18681
0.10889	0.36312	-0.03702	0.0438	0.00271	0.35451	0.14539	0.13013	-0.42243	0.05961	-0.04411	0.04082	0.12338	0.16757	0.25076	0.13067	0.04522	0.0726	-0.03345	0.15818	0.18356	0.01743	-0.06064	0.23833
0.06197	0.31137	-0.01359	-0.07298	0.06665	0.35164	0.1524	0.08959	-0.23308	0.01723	-0.11722	0.07191	0.14386	0.17588	0.20718	0.12471	0.10316	0.10616	0.00942	0.20614	0.21193	0.06827	-0.05576	0.27715
0.12019	0.4073	0.02333	0.09886	-0.00361	0.35509	0.31455	0.11773	-0.06318	0.02447	-0.07204	0.0871	0.13521	0.12456	0.2742	0.16736	0.02481	0.10489	-0.08761	0.17203	0.21899	-0.03219	-0.02072	0.25521
0.04625	0.36287	-0.03604	0.06139	0.10056	0.35629	0.16092	0.1151	-0.25196	-0.01074	-0.0696	0.08026	0.14025	0.32782	0.22818	0.13586	0.13218	0.10233	-0.0283	0.16687	0.21088	0.08528	-0.06226	0.23687
0.03427	0.35392	0.00847	-0.01737	0.05993	0.35738	0.08668	0.10736	0.03741	0.00317	-0.03269	0.02707	0.13284	0.26559	0.20917	0.11979	0.109	0.1098	-0.0482	0.18571	0.1886	0.04841	-0.04704	0.25345
0.03501	0.37494	-0.02285	-0.07127	0.02677	0.35151	0.14258	0.17887	-0.19446	0.12912	-0.04985	0.06403	0.0872	0.07679	0.23379	0.12996	0.03817	0.09471	-0.03923	0.10416	0.1485	-0.0174	-0.06176	0.2689
0.02482	0.30187	-0.04222	0.06795	0.1019	0.35212	0.04822	0.12123	-0.06979	0.11943	-0.09963	0.04954	0.13895	0.34997	0.18838	0.12283	0.08954	0.09939	-0.02419	0.20646	0.25344	0.10652	-0.03266	0.24433
0.00754	0.36721	-0.08721	0.06749	0.12592	0.3495	0.0716	0.09436	-0.11731	0.06187	-0.11133	0.02477	0.12191	0.3525	0.20843	0.08606	0.15484	0.13049	0.02033	0.16214	0.24125	0.14834	-0.08472	0.2237

F_MECP2	ITY_BY_R	OF_NFE	N_OF_N	N_OF_NP	OF_NP	N_OF_NP	PLK1_AC	OF_PTEN	ION_OF_	N_OF_PT	OF_PTEN	RUVATE_	LATION_	RUNX1_	RUNX2_	RUNX3_	TION_OF	OF_TLR	LATION_	TP53_ACT	ITY_THR	TP53_ACT	ACTIVIT
0.0816	0.24466	0.108	-0.01713	0.03301	-0.1152	0.07793	0.16466	0.01582	0.29404	0.07793	0.26399	0.08196	0.2654	0.15372	0.27186	0.2775	0.05172	0.26084	0.02192	0.04676	-0.16889	0.03327	0.01663
0.12459	0.25641	-0.00857	0.01401	0.05009	-0.34208	0.14234	0.17292	0.05544	0.35618	0.14234	0.27001	0.03427	0.25829	0.17254	0.27078	0.28096	0.0152	0.25029	0.06713	0.07666	-0.14537	0.03824	0.0691
0.0879	0.23185	0.07729	-0.0046	0.01511	-0.22917	0.08265	0.15129	0.02089	0.33703	0.08265	0.25705	0.1143	0.24911	0.16175	0.25525	0.25975	0.11318	0.29401	0.05275	0.06066	-0.05143	0.04618	0.03548
0.04903	0.23222	0.03181	0.01419	-0.07416	-0.30139	-0.01349	0.15735	0.01513	0.3358	-0.01349	0.26099	0.01519	0.24679	0.12608	0.26255	0.26264	0.15815	0.3196	0.01625	0.03569	-0.05914	0.03634	0.00127
0.07232	0.25805	0.13545	0.00153	-0.07787	-0.42505	-0.01409	0.17036	0.00556	0.32461	-0.01409	0.28823	-0.01901	0.29597	0.12774	0.2932	0.29954	0.13528	0.33084	0.02597	0.06051	-0.15013	0.05361	0.01375
0.08362	0.24222	0.13449	-0.07032	-0.07619	-0.31883	-0.02047	0.16765	0.02764	0.29877	-0.02047	0.27433	-0.03677	0.27317	0.11207	0.28047	0.27954	0.04718	0.30197	0.0516	0.0702	-0.08498	0.06449	0.05418
0.0545	0.24394	0.15627	-0.04396	-0.11159	-0.4025	-0.05296	0.1261	0.01351	0.29864	-0.05296	0.26128	0.04953	0.25151	0.09504	0.26637	0.26169	0.13929	0.33918	0.02016	0.02081	-0.15669	0.014	0.03608
0.04207	0.2422	0.10801	0.02918	-0.07469	-0.1728	-0.0445	0.16679	-0.00197	0.29055	-0.0445	0.27733	0.00827	0.27457	0.117	0.27972	0.28519	0.20177	0.29232	-0.0097	0.0033	-0.15573	0.03882	-0.00236
0.0306	0.24846	0.06288	0.00874	-0.09968	-0.10076	-0.09928	0.17057	-0.00518	0.30249	-0.09928	0.28248	-0.0368	0.27601	0.09752	0.28287	0.29218	0.21221	0.31848	0.00594	0.02088	-0.14565	0.04847	0.01062
0.0624	0.24375	0.14817	0.01105	-0.08806	-0.40409	-0.02573	0.15261	0.0039	0.32764	-0.02573	0.27416	-0.00071	0.26193	0.14132	0.27577	0.2821	0.1782	0.31826	0.01808	0.03001	-0.08939	0.06667	0.03095
0.07908	0.22664	0.04422	-0.0398	0.02016	-0.36833	0.10889	0.17893	0.02464	0.34688	0.10889	0.26645	0.07541	0.25042	0.12494	0.26375	0.26291	0.07785	0.29443	0.05685	0.05993	-0.10258	0.06037	0.0415
0.09668	0.24029	0.04161	-0.03353	0.01979	-0.1189	0.06197	0.15648	-0.01435	0.29947	0.06197	0.27324	0.08962	0.25691	0.1162	0.27569	0.27644	0.05346	0.31036	0.04293	0.043	-0.03363	0.03918	0.03946
0.06222	0.23875	-0.02023	-0.02829	0.04174	-0.25189	0.12019	0.19897	0.0811	0.27446	0.12019	0.27523	0.03655	0.26747	0.10739	0.27066	0.27002	-0.01925	0.26975	0.07305	0.07222	0.01177	0.03702	0.05846
0.08079	0.23892	0.06146	0.03842	-0.0116	-0.21561	0.04625	0.15848	-0.00405	0.33421	0.04625	0.26861	0.08002	0.26195	0.10707	0.26728	0.26614	0.08696	0.32214	0.02276	0.04107	-0.13746	0.05098	0.01143
0.0632	0.24559	0.06846	0.00444	-0.02311	-0.226	0.03427	0.16649	0.01713	0.36205	0.03427	0.26519	0.06604	0.25579	0.12943	0.26402	0.26902	0.05173	0.31485	0.04642	0.04934	-0.1757	0.05314	0.05594
0.03258	0.23641	0.02481	-0.00817	-0.00033	-0.11467	0.03501	0.16954	0.02212	0.36849	0.03501	0.26215	0.12718	0.25052	0.06203	0.25784	0.25038	-0.02659	0.22727	0.06716	0.06027	-0.02508	0.04974	0.06192
0.07076	0.23175	0.02965	-0.02662	-0.03303	-0.23903	0.02482	0.16467	0.00945	0.33246	0.02482	0.26046	0.03036	0.25287	0.12293	0.25765	0.26618	0.12496	0.31311	0.03249	0.0669	-0.05979	0.04524	0.014
0.05197	0.24595	0.13078	-0.01905	-0.03728	-0.18778	0.00754	0.15982	-0.00614	0.32822	0.00754	0.28089	0.03244	0.26798	0.14058	0.28283	0.28744	0.18193	0.32662	-0.00461	0.02191	-0.18081	0.03406	-0.00204

TP53_EX	PTIC_FAC	TH_NP_F	ROPEPTID	S_BY_NU	ION_OF_	E_REPR	ON_OF_A	MULTIPL	ION_OF_D	THROUG	OF_SIST	TORY_EL	CHEMIOS	EIF2AK1	AK4_GCN	E_OF_MT	ELEVATED	PONSE_	ID_CYCL	E_NEUR	SPORT_A	E_RET_SI	HYDRAT
-0.04584	0.19689	0.0761	-0.06233	0.45981	0.13669	0.27702	-0.01969	0.01504	-0.07031	-0.07031	0.09535	0.15615	0.1887	0.25195	0.38704	0.31982	0.18222	0.24471	-0.03396	0.26143	-0.0468	0.02503	0.00646
0.00338	0.22419	-0.0731	-0.02681	0.46177	0.18756	0.31164	0.10515	0.11732	-0.06204	-0.06204	0.12023	0.15929	0.18348	0.16593	0.39889	0.27778	0.16878	-0.06343	0.07766	0.25676	-0.07521	-0.00885	-0.00243
0.02383	0.23105	-0.17139	0.11105	0.4563	0.16473	0.29913	0.06276	0.09044	-0.00725	-0.00725	0.11568	0.14917	0.18086	0.24617	0.38887	0.30855	0.1873	-0.17521	0.06375	0.30862	-0.03195	-0.00227	0.03325
-0.01608	0.18425	0.10816	0.08374	0.45434	0.1467	0.28492	0.02846	0.05454	-0.0074	-0.0074	0.10355	0.16114	0.18596	0.20541	0.40179	0.32162	0.24708	-0.08577	0.25583	0.32573	-0.02681	0.05747	-0.0135
-0.0204	0.22362	-0.19993	0.06703	0.46049	0.12487	0.29837	0.02736	0.04669	-0.10279	-0.10279	0.11167	0.11877	0.14904	0.19892	0.39459	0.31691	0.20907	-0.16377	0.17114	0.30346	-0.03849	0.07008	0.12723
-0.01572	0.17247	-0.09829	0.18183	0.46868	0.10265	0.30196	0.07283	0.09017	-0.0758	-0.0758	0.10034	0.15135	0.17532	0.28665	0.40572	0.29605	0.27144	-0.15542	0.28047	0.2654	-0.00739	0.02598	0.12762
-0.02823	0.2072	-0.02942	0.21032	0.46481	0.15851	0.30906	0.04717	0.07396	0.02312	0.02312	0.09348	0.17497	0.19827	0.20644	0.40042	0.28125	0.27658	-0.07253	0.25556	0.27025	-0.06842	0.02756	-7.3E-05
-0.06474	0.20459	-0.03417	0.12153	0.46066	0.10421	0.2782	-0.01688	0.00629	-0.05567	-0.05567	0.08811	0.15246	0.17903	0.20815	0.39739	0.32659	0.26407	-0.05003	0.11646	0.3375	-0.01876	0.06998	0.11207
-0.03848	0.20298	-0.15114	0.22126	0.4571	0.13356	0.27752	-0.01744	0.0123	-0.04482	-0.04482	0.08806	0.15452	0.17619	0.20793	0.39377	0.31297	0.2662	-0.12969	0.26511	0.33091	-0.01303	0.0761	0.02023
-0.0657	0.20629	0.00828	0.18569	0.46259	0.14258	0.2919	0.02818	0.05593	-0.0269	-0.0269	0.09247	0.17461	0.1987	0.1989	0.38775	0.31617	0.25218	-0.09569	0.11512	0.30502	-0.04742	0.04509	0.05547
0.03475	0.20141	-0.36639	0.05119	0.46049	0.17514	0.30102	0.04441	0.07252	-0.02983	-0.02983	0.11922	0.1854	0.20729	0.26124	0.39993	0.29714	0.20427	-0.04767	0.18533	0.24666	-0.02817	-0.02031	-0.05932
-0.01023	0.20338	-0.24432	0.22455	0.46159	0.10099	0.29425	0.05629	0.082	-0.01855	-0.01855	0.1062	0.17551	0.19125	0.28462	0.40447	0.30056	0.24873	-0.17471	0.08468	0.27545	-0.04755	-0.0042	0.01294
0.05022	0.21896	-0.26158	0.04582	0.46344	0.17626	0.30565	0.074	0.09699	0.02031	0.02031	0.15254	0.06566	0.11704	0.21639	0.39349	0.29553	0.1954	0.04326	0.0462	0.2897	-0.06433	0.01202	0.01346
-0.01961	0.18946	-0.14761	0.07497	0.45952	0.12775	0.29835	0.01011	0.01563	-0.03621	-0.03621	0.12175	0.1334	0.16548	0.23995	0.40398	0.30747	0.22246	-0.11541	0.13362	0.31158	-0.03299	0.03169	0.03589
-0.03016	0.20184	-0.03312	0.13007	0.46121	0.0918	0.28863	0.0346	0.06929	0.00216	0.00216	0.09321	0.13426	0.16254	0.27606	0.41208	0.32154	0.22365	-0.02501	0.23413	0.25731	-0.0435	0.0299	0.05779
0.02801	0.20782	-0.18513	-0.00561	0.45511	0.11609	0.29863	0.07198	0.08341	0.17447	0.17447	0.12799	0.1608	0.19073	0.24068	0.40067	0.29302	0.15707	-0.10064	0.08704	0.23886	-0.0548	-0.03762	-0.09012
-0.00134	0.20991	-0.09354	-0.00176	0.45625	0.15945	0.2926	-0.01977	-0.0166	-0.00437	-0.00437	0.1122	0.18112	0.21527	0.16773	0.40167	0.31146	0.22987	-0.08835	0.16744	0.32396	-0.05113	0.05877	0.02385
-0.04589	0.2391	-0.13599	0.06911	0.45728	0.11411	0.28262	-0.0245	-0.00459	0.03333	0.03333	0.10664	0.18265	0.2065	0.18178	0.38318	0.3191	0.2189	-0.05614	0.18947	0.33235	-0.03723	0.07157	0.08428

RHOA_GT	RHOBTB1_	RHOBTB2_	RHOBTB3_	RHOBTB_G	RHOB_GT	RHOC_GT	RHOD_GT	RHOF_GT	RHOG_GT	RHOH_GT	RHOJ_GT	RHOQ_GT	RHOT1_GT	RHOU_GT	RHOV_GT	GTPASE	TPASES_A	GTPASES_	GTPASES	SES_ACTI	GTPASES	GTPASES	CTIVATE_	GTPASES	ES_ACTIV
0.05768	0.16974	0.27185	0.11282	0.21277	0.01432	0.06902	0.11357	0.11126	0.10827	0.15788	0.05754	0.04627	0.09081	0.0922	0.15637	0.29625	0.13164	0.28236	0.18247	0.2541	0.31596	0.38495	0.12674	0.29229	0.24043
0.00899	0.18491	0.31056	0.06735	0.23407	-0.03296	0.03224	0.11564	0.07756	0.0954	0.1269	0.02202	0.02448	-0.00939	0.08769	0.13598	0.29869	0.13457	0.3006	0.19288	0.18955	0.29673	0.37923	0.12721	0.2628	0.19954
0.05876	0.15115	0.27143	0.14589	0.20397	0.01451	0.08073	0.12012	0.10372	0.11962	0.18089	0.09859	0.06951	-0.14481	0.09293	0.15721	0.29521	0.1305	0.29644	0.12793	0.23408	0.29899	0.37437	0.06962	0.28295	0.22418
0.08224	0.12985	0.25019	0.08177	0.18996	0.02447	0.08324	0.12269	0.12734	0.11574	0.145	0.05604	0.03915	-0.08423	0.09699	0.12262	0.28359	0.14393	0.32831	0.22295	0.27851	0.29156	0.37776	0.10055	0.26697	0.23945
0.07157	0.18424	0.27616	0.09847	0.22017	0.00274	0.05932	0.12767	0.12096	0.08939	0.15374	0.04473	0.02152	0.05182	0.1137	0.13531	0.27651	0.14456	0.29243	0.22756	0.28037	0.29032	0.36325	0.10593	0.26138	0.22992
0.04105	0.17596	0.28639	0.08471	0.22534	-0.02611	0.03394	0.11263	0.09173	0.108	0.18869	0.03834	0.03156	-0.05277	0.08272	0.10761	0.28076	0.12833	0.27805	0.21451	0.1727	0.28638	0.36775	0.05721	0.25227	0.21559
0.03578	0.17843	0.27744	0.07793	0.21587	-0.0151	0.04687	0.10153	0.07871	0.08768	0.16574	0.06956	0.05568	-0.07955	0.10894	0.1609	0.28669	0.11993	0.29242	0.14544	0.2451	0.29206	0.37147	0.04479	0.27166	0.242
0.08833	0.1413	0.24646	0.11371	0.19659	0.02955	0.08718	0.13397	0.14091	0.1027	0.13517	0.0571	0.03306	-0.00498	0.12799	0.13721	0.26214	0.13911	0.312	0.23319	0.28236	0.27728	0.36889	0.06911	0.25356	0.24449
0.08436	0.17405	0.28064	0.10643	0.21792	0.02255	0.08943	0.13782	0.13477	0.11355	0.16265	0.067	0.04101	0.01263	0.1454	0.1419	0.28502	0.14244	0.32396	0.18903	0.28903	0.28363	0.37173	0.06073	0.28039	0.23667
0.07103	0.14244	0.24452	0.069	0.18936	-0.00782	0.06485	0.11659	0.12302	0.10311	0.15453	0.05694	0.04279	0.03372	0.11609	0.12064	0.2935	0.13822	0.31299	0.22717	0.30139	0.28728	0.3688	0.0269	0.27011	0.23632
0.04629	0.23389	0.31191	0.08922	0.26661	0.00249	0.05843	0.11771	0.09349	0.09399	0.18337	0.03452	0.01827	-0.10113	0.09921	0.16257	0.30221	0.13871	0.3215	0.1265	0.22041	0.30834	0.38528	0.08463	0.29699	0.20979
0.04326	0.18607	0.29467	0.0912	0.23474	-0.00603	0.0529	0.08606	0.07706	0.06882	0.16067	0.03451	0.02363	0.0045	0.08461	0.12772	0.28418	0.13742	0.29457	0.16933	0.21029	0.28621	0.3846	0.06661	0.26806	0.2387
0.01781	0.19286	0.30532	0.1105	0.23994	-0.00231	0.04213	0.11952	0.10493	0.08689	0.14869	0.05713	0.05963	-0.19468	0.09828	0.20761	0.33111	0.16506	0.34137	0.23225	0.10414	0.34271	0.3886	0.16296	0.31477	0.25716
0.06735	0.16417	0.27197	0.09818	0.21515	0.00912	0.06835	0.12216	0.08774	0.10717	0.1576	0.03976	0.03561	-0.01693	0.09133	0.15287	0.2938	0.15564	0.32912	0.19484	0.25587	0.28526	0.37876	0.13047	0.27887	0.24597
0.04212	0.19183	0.28432	0.09965	0.23622	-0.00904	0.05488	0.11874	0.10051	0.08444	0.16847	0.04687	0.04647	-0.16204	0.09672	0.13854	0.2851	0.12516	0.30502	0.21501	0.26275	0.30577	0.37951	0.05996	0.29287	0.22469
0.02538	0.17959	0.29636	0.06799	0.23554	0.00548	0.04708	0.10073	0.08795	0.08097	0.17522	0.06184	0.07013	-0.13316	0.09093	0.16367	0.30514	0.13893	0.30433	0.15582	0.1128	0.3298	0.38303	0.15461	0.29432	0.23906
0.06025	0.16366	0.2867	0.08636	0.22184	0.00499	0.06444	0.11894	0.11278	0.10742	0.14528	0.05404	0.05409	-0.03977	0.10051	0.14365	0.29207	0.15343	0.32401	0.17586	0.24887	0.29668	0.37865	0.1298	0.27087	0.25271
0.08582	0.15034	0.26342	0.06492	0.20466	0.01699	0.08719	0.12915	0.12411	0.11617	0.15939	0.05251	0.04086	0.04085	0.11066	0.14306	0.27149	0.15612	0.34856	0.21203	0.25978	0.27845	0.36458	0.05877	0.25773	0.23748

ED_NFK	ETHYLATE_	MERASE_	LYMERASE	SCRIPTIO	SCRIPTIO	SE_III_TRA	SE_II_PR	ASE_II_TR	LYMERASE	SE_II_TRA	MERASE_	LYMERAS	RASE_I_TR	SE_I_TRA
-0.01455	0.31754	-0.04713	-0.07159	-0.08117	-0.05412	-0.04344	-0.01647	-0.08155	0.10237	0.15793	0.37032	0.29864	0.04223	-0.02059
-0.01402	0.34313	-0.07228	-0.03249	-0.04378	-0.07976	-0.04589	0.05525	-0.00816	0.12996	0.2132	0.37668	0.31262	0.078	-0.02656
-0.02502	0.32716	-0.06155	-0.05038	-0.0725	-0.04587	-0.03256	0.01933	-0.04389	0.11189	0.18002	0.37908	0.31141	0.07617	0.02194
-0.00821	0.30362	-0.11851	-0.16772	-0.17692	-0.13021	-0.12137	-0.02408	-0.09712	0.09552	0.15544	0.35586	0.28224	-0.00689	-0.09713
0.0086	0.32238	-0.11643	-0.08593	-0.11725	-0.10967	-0.06993	-0.0042	-0.07411	0.10611	0.16587	0.36775	0.29044	0.01001	-0.06416
-0.03263	0.33731	-0.11709	-0.1158	-0.12131	-0.10667	-0.10998	0.02303	-0.01354	0.11412	0.18089	0.36946	0.29116	0.04877	-0.03534
-0.01172	0.32021	-0.0362	-0.06638	-0.05588	-0.04578	-0.05729	0.0058	-0.04355	0.10047	0.1576	0.38463	0.3063	0.04888	0.00728
0.02873	0.30348	-0.16641	-0.17326	-0.18489	-0.1702	-0.15459	-0.05669	-0.12218	0.08739	0.12252	0.34958	0.26877	-0.06022	-0.0871
0.011	0.31171	-0.17155	-0.17759	-0.18635	-0.16264	-0.16249	-0.03669	-0.09965	0.08848	0.12725	0.3529	0.26945	-0.04986	-0.11714
0.0215	0.30231	-0.13118	-0.15454	-0.16172	-0.13488	-0.12868	-0.01344	-0.07834	0.09884	0.14865	0.36083	0.28306	-0.01306	-0.10206
-0.06269	0.31489	-0.08273	-0.06839	-0.09784	-0.09434	-0.04465	0.00148	-0.05807	0.11231	0.17999	0.37196	0.30485	0.05531	-0.00511
-0.05797	0.32248	-0.07502	-0.08431	-0.1233	-0.0756	-0.03537	-0.03019	-0.09986	0.09989	0.15916	0.37521	0.29756	0.02864	-0.02823
-0.08592	0.34253	-0.06233	-0.06117	-0.07967	-0.06699	-0.04043	0.0294	-0.02875	0.1187	0.18035	0.37152	0.32346	0.1062	-0.00729
-0.00729	0.32142	-0.09093	-0.11718	-0.12853	-0.10341	-0.08544	-0.01201	-0.088	0.09795	0.15425	0.3702	0.29743	0.01533	-0.03759
-0.01338	0.3213	-0.05339	-0.04862	-0.06347	-0.06618	-0.03479	0.00396	-0.07537	0.10327	0.16004	0.36467	0.29649	0.02932	-0.02352
-0.07087	0.32671	-0.00898	-0.03618	-0.05008	-0.01707	-0.00175	0.00266	-0.05913	0.11086	0.17756	0.37803	0.31473	0.07871	0.05423
-0.04853	0.33144	-0.07452	-0.09205	-0.09977	-0.09322	-0.07044	-0.02212	-0.05453	0.10592	0.16902	0.37143	0.29493	0.02228	-0.0508
0.01833	0.30608	-0.12568	-0.1443	-0.15714	-0.12487	-0.11648	-0.03352	-0.09913	0.09105	0.14017	0.35608	0.2758	-0.03216	-0.07468

RND1_GT	RND2_GT	RND3_GT	_RECEPT	ABL_IN	RITAL_LAB	OSPHOL	_MESSENC	TIVATES	IS_PROD	CATION_	TION_IN_T	RRNA_P	ESSING_I	E_RSK_A
0.04747	0.0583	0.07396	0.13472	0.05494	-0.00169	0.07538	-0.12927	-0.05553	0.24027	-0.29937	-0.02933	0.17411	-0.0694	0.12612
0.03998	0.03914	0.03102	0.15278	0.04191	-0.0328	0.05867	0.00021	-0.13817	0.17316	-0.26661	0.08396	0.23985	0.00074	0.07489
0.02785	0.04949	0.06644	0.03386	0.07661	0.03138	0.07052	-0.22781	-0.05861	0.26356	-0.23107	0.04801	0.20338	-0.05031	0.143
0.002	0.03026	0.03258	0.161	0.07821	0.1048	0.17816	-0.18383	-0.08048	0.31227	-0.26491	-0.02865	0.17809	-0.08082	0.11475
-0.01251	-0.00258	0.01908	0.20799	0.13685	0.03787	0.08883	-0.15198	-0.07929	0.26634	-0.343	0.00766	0.18788	-0.08327	0.12054
0.03923	0.02735	0.04232	0.09623	0.1144	-0.06401	0.0409	-0.01665	-0.06881	0.1299	-0.3484	0.0692	0.21038	-0.05421	0.01946
0.00016	0.045	0.02261	0.11248	0.09539	0.02729	0.09023	-0.16067	-0.02125	0.19117	-0.29285	0.09945	0.22873	-0.03885	0.11393
0.00113	0.02556	0.01358	0.16904	0.08418	0.09714	0.15064	-0.12306	-0.06803	0.32674	-0.3694	-0.0611	0.1633	-0.14453	0.13932
0.0226	0.02261	0.04288	0.15098	0.10407	0.10942	0.14042	-0.08198	-0.10403	0.33005	-0.34322	-0.05543	0.16498	-0.1043	0.11604
-0.0302	0.0076	0.01176	0.18642	0.12332	0.08199	0.16701	-0.03067	-0.09078	0.3078	-0.33281	-0.03242	0.16975	-0.10253	0.14335
0.04649	0.04229	0.05564	0.032	0.09599	-0.05204	-0.01559	-0.32561	-0.04202	0.21312	-0.23523	0.01384	0.19999	-0.021	0.06297
-0.01831	-0.01171	0.02376	0.13584	0.07565	-0.07929	-0.00905	-0.21771	-0.01051	0.15927	-0.25462	0.01153	0.19643	-0.03169	0.11542
0.07206	0.07601	0.07973	0.09813	0.12117	-0.12401	-0.06018	-0.2924	0.01777	0.12828	-0.3895	0.03964	0.20373	-0.06741	0.17414
-0.012	0.01923	0.02082	0.14699	0.09658	-0.01454	0.06349	-0.20432	-0.09132	0.29596	-0.40061	0.01832	0.2014	-0.06708	0.10019
-0.0012	0.0392	0.02061	0.13285	0.06734	-0.03229	0.03822	-0.29164	-0.05748	0.24352	-0.34467	0.02331	0.21015	-0.06657	0.16227
0.04049	0.06324	0.03693	0.05268	0.16092	-0.1859	-0.14242	-0.32063	-0.0673	0.07749	-0.29012	0.10848	0.23517	-0.0106	0.05936
0.01316	0.03093	0.05809	0.15025	0.11157	0.06303	0.10933	-0.25902	-0.06488	0.26977	-0.27861	0.03658	0.20139	-0.05737	0.08868
0.01424	0.01891	0.04345	0.15738	0.10075	0.08157	0.14773	-0.06876	-0.13495	0.29918	-0.34219	-0.02561	0.1675	-0.0773	0.12004

EVELOP	HOSE_P	ROGEN_R	ISSION_O	MEGAKA	RIPTION_O	N_OF_GE	GENES_I	GENES_I	ION_OF_G	IPTION_O	GULATES	ATES_CH	GENES	ATES_OS
-0.0998	-0.02024	0.19447	0.17946	0.34871	0.01664	0.35812	0.19521	0.19206	0.01664	0.19447	0.05756	0.19447	-0.09854	0.07031
-0.13351	0.08658	0.16523	0.20054	0.3659	0.04783	0.36043	0.19023	0.14685	0.04783	0.16523	0.08073	0.16523	-0.02087	0.03755
-0.08139	-0.00343	0.21156	0.08014	0.35373	0.05698	0.35006	0.24706	0.20154	0.05698	0.21156	0.06363	0.21156	0.03465	0.07632
-0.0556	-0.06461	0.18637	0.02206	0.33968	0.04705	0.35085	0.29172	0.21841	0.04705	0.18637	0.08339	0.18637	0.09263	0.10089
0.04059	0.01295	0.29065	-0.04497	0.34978	0.174	0.37612	0.28894	0.25776	0.174	0.29065	0.08397	0.29065	0.01951	0.10583
0.00476	0.01052	0.25964	-0.03658	0.35374	0.12652	0.36215	0.15981	0.23563	0.12652	0.25964	-0.01483	0.25964	-0.03772	-0.01273
-0.03	-0.02139	0.26863	0.01753	0.35981	0.04043	0.35375	0.24343	0.24653	0.04043	0.26863	0.08252	0.26863	0.02187	0.08426
0.04595	-0.06475	0.226	-0.04636	0.33507	0.08265	0.36268	0.30608	0.2352	0.08265	0.226	0.06203	0.226	0.07991	0.08417
0.04089	-0.03334	0.27197	-0.04129	0.34288	0.18277	0.3674	0.34287	0.25231	0.18277	0.27197	0.05839	0.27197	0.10722	0.07947
0.07297	-0.05482	0.29769	-0.02696	0.34779	0.15397	0.3607	0.33822	0.28049	0.15397	0.29769	0.04263	0.29769	0.07766	0.06399
-0.14164	-0.01598	0.1219	0.0569	0.36121	-0.06648	0.35393	0.01045	0.14113	-0.06648	0.1219	0.09841	0.1219	-0.12212	0.07047
-0.05066	-0.02502	0.21878	0.15497	0.35444	0.03104	0.36299	0.20514	0.20921	0.03104	0.21878	0.08972	0.21878	-0.03816	0.10607
-0.23785	0.04007	-0.01022	0.10831	0.35409	-0.16082	0.35767	-0.06647	0.09171	-0.16082	-0.01022	0.0977	-0.01022	-0.11976	0.06898
-0.06645	-0.0188	0.17791	0.03241	0.35578	0.05018	0.35667	0.24484	0.17813	0.05018	0.17791	0.04391	0.17791	0.02427	0.0357
-0.12155	-0.02232	0.18108	0.07649	0.35251	0.04159	0.35974	0.22329	0.17251	0.04159	0.18108	0.06204	0.18108	-0.07617	0.07103
-0.26812	-0.0062	-0.12929	0.06098	0.34158	-0.09661	0.34968	-0.00559	0.06501	-0.09661	-0.12929	0.0969	-0.12929	-0.13408	0.07322
-0.0544	0.00642	0.18795	0.10389	0.35484	0.11491	0.35244	0.22862	0.19515	0.11491	0.18795	0.03124	0.18795	0.03378	0.03011
0.04234	-0.03916	0.29153	0.01484	0.34862	0.12695	0.36571	0.31616	0.24694	0.12695	0.29153	0.07875	0.29153	0.07557	0.09819

ATES_BC	ULATES_C	MMUNE_R	EGULATE	IX3_REGU	REGULAT	TES_YAP	S_MODUL	OV_1_H	RS_COV	LATES_H	ACELLUL	LATES_IN	OV_2_H	RS_COV
-0.29938	-0.32675	0.02535	-0.16146	-0.0758	0.09959	-0.22152	0.16243	0.28169	0.23883	0.40886	0.19466	0.10706	0.19123	0.17167
-0.23029	-0.26797	0.00023	-0.10665	-0.06015	0.07005	-0.19547	0.16854	0.29616	0.23983	0.4328	0.17122	0.10706	0.19803	0.1747
-0.34396	-0.31744	0.00178	-0.11711	-0.04712	0.10532	-0.13226	0.16796	0.27904	0.22794	0.41637	0.1547	0.13279	0.20257	0.17561
-0.37496	-0.35873	0.04014	-0.18382	-0.16914	0.01014	-0.27001	0.17737	0.2919	0.24953	0.42747	0.18481	0.10639	0.19821	0.18081
-0.26029	-0.2256	0.00021	-0.04423	-0.0128	0.05806	-0.09095	0.17713	0.29041	0.24131	0.42615	0.15278	0.11021	0.19404	0.17227
-0.20437	-0.23409	0.11109	-0.05752	-0.10188	0.12107	-0.07342	0.1496	0.29212	0.23793	0.43437	0.20731	0.09097	0.18659	0.16839
-0.24835	-0.23986	0.0564	-0.21949	-0.15961	0.0596	-0.24894	0.1824	0.29941	0.24145	0.43125	0.18086	0.10434	0.1921	0.16716
-0.33994	-0.30214	0.09419	-0.09735	-0.18517	-0.07854	-0.26514	0.1854	0.29824	0.25437	0.432	0.19735	0.10266	0.19803	0.17723
-0.26296	-0.24162	0.09805	-0.12021	-0.13261	-0.01724	-0.25969	0.17119	0.28677	0.2434	0.42162	0.17457	0.10472	0.19291	0.17458
-0.26703	-0.2266	0.12234	-0.06336	-0.11762	-0.11405	-0.27037	0.19779	0.29246	0.24779	0.41291	0.17679	0.11368	0.19033	0.16955
-0.25218	-0.31065	-0.14629	-0.12931	-0.12034	0.07512	-0.11042	0.17246	0.30119	0.24833	0.42703	0.20515	0.09452	0.18772	0.16852
-0.31501	-0.32017	-0.06662	-0.16332	-0.10298	-0.00607	-0.17855	0.17775	0.30281	0.25375	0.42836	0.22688	0.11836	0.20322	0.17826
-0.26972	-0.25009	-0.26831	-0.15414	-0.05922	0.08122	-0.06331	0.14214	0.28303	0.24532	0.41819	0.20459	0.09409	0.18725	0.17937
-0.3188	-0.30472	-0.04258	-0.10806	-0.12087	0.07033	-0.18054	0.15518	0.28734	0.23648	0.43085	0.18383	0.11687	0.20157	0.17793
-0.369	-0.35024	-0.08311	-0.11359	-0.16565	0.05021	-0.1829	0.16988	0.29605	0.25068	0.42988	0.20401	0.0978	0.19442	0.17396
-0.17584	-0.24711	-0.1786	-0.08254	-0.20258	0.13314	-0.05036	0.17996	0.30496	0.25656	0.43029	0.21172	0.10652	0.20119	0.18544
-0.36702	-0.37308	0.0286	-0.15722	-0.04813	-0.00295	-0.2833	0.16014	0.29143	0.24469	0.43193	0.18395	0.08937	0.18903	0.17034
-0.28617	-0.27703	0.00741	-0.13172	-0.12793	0.08895	-0.15445	0.18955	0.2915	0.24606	0.42135	0.16005	0.10685	0.19523	0.1734

V_2_MOD	LATES_H	ACELLUL	_PDZ_PR	RS_COV	ING_BY	ONG_BY	ONG_BY	GING_OF_P	_MEDIAT	DIATED_D	NUCLEAR	ELECTIVE	AMINO_	_DEPEN
-0.0311	0.37377	0.20133	-0.01135	0.16579	0.16419	0.12458	0.41935	0.28788	0.2983	0.27122	0.16048	0.16508	0.37193	0.3477
-0.02062	0.4005	0.1512	0.05094	0.16987	0.18	0.11569	0.43335	0.26187	0.3027	0.27827	0.15257	0.1869	0.38312	0.30999
0.00591	0.38161	0.13842	-0.14502	0.17226	0.19851	0.18911	0.42972	0.33941	0.27953	0.25622	0.13535	0.1654	0.37005	0.32894
0.04032	0.38914	0.18761	-0.09384	0.1759	0.29221	0.21875	0.39688	0.41694	0.28663	0.2597	0.17747	0.18322	0.38096	0.32075
0.03558	0.38886	0.13926	-0.29763	0.16997	0.20569	0.17837	0.41368	0.3837	0.31958	0.29375	0.1763	0.1698	0.37299	0.33827
-0.06001	0.39516	0.19484	-0.22319	0.16674	0.15046	0.21154	0.42331	0.39701	0.3038	0.28423	0.14993	0.1652	0.37662	0.30798
-0.02516	0.39502	0.15179	-0.14935	0.16909	0.24203	0.3199	0.43527	0.40836	0.28501	0.2664	0.10974	0.16298	0.37901	0.32418
0.07512	0.38915	0.20031	-0.1749	0.17178	0.31154	0.22131	0.42763	0.42906	0.30811	0.28249	0.19482	0.17236	0.37612	0.33759
0.07167	0.3784	0.17813	-0.28809	0.16972	0.32628	0.25233	0.41817	0.42062	0.31182	0.28394	0.18566	0.16992	0.37256	0.34921
0.01113	0.37121	0.16398	-0.28263	0.16954	0.2671	0.27764	0.42656	0.43053	0.30028	0.27754	0.17848	0.17625	0.3665	0.35003
-0.0473	0.38653	0.18456	0.00265	0.16713	0.17409	0.223	0.43178	0.2844	0.28893	0.27104	0.16674	0.191	0.37114	0.31074
-0.08179	0.39103	0.21971	0.07308	0.17018	0.20642	0.25011	0.42127	0.38558	0.29804	0.27135	0.15235	0.17368	0.38136	0.3163
-0.04394	0.38362	0.19333	0.03025	0.17589	0.14274	0.19817	0.41539	0.30815	0.29886	0.28306	0.23888	0.21615	0.36496	0.3222
0.01613	0.38903	0.17471	-0.03192	0.17023	0.20696	0.23626	0.42516	0.36429	0.29302	0.26721	0.17294	0.17155	0.37955	0.31255
0.00608	0.39207	0.19968	-0.06408	0.16687	0.17497	0.14173	0.43471	0.36907	0.2961	0.27164	0.17296	0.17589	0.38715	0.33183
-0.00979	0.39439	0.21044	0.0424	0.17778	0.14553	0.10922	0.40584	0.20511	0.28132	0.25832	0.16683	0.17555	0.38494	0.33892
-0.00447	0.3932	0.18811	-0.06446	0.1667	0.22412	0.21852	0.42685	0.3504	0.28429	0.25705	0.19587	0.19846	0.37875	0.30987
0.07699	0.38208	0.165	-0.19451	0.17067	0.24597	0.15685	0.41138	0.37121	0.30776	0.28053	0.20596	0.18445	0.36843	0.32663

SIGNAL	MIGRAT	_IN_SEMA	TION_OF	APHORIN	OCIATED_	_DNA_D	SENSORY	RY_PER	RY_PROC	OUND_B	TION_OF_	SERINE_B	EUROTRA	VENTS_IN
0.20216	0.20797	0.19085	0.08857	0.21193	0.27773	-0.04125	0.15287	0.05746	0.27784	0.27344	0.16047	0.11937	0.13771	0.04245
0.17322	0.18907	0.16802	0.07401	0.2128	0.27706	-0.00512	0.13979	0.00982	0.26262	0.24236	0.17789	0.1104	0.12457	0.03908
0.21101	0.20762	0.19258	0.10637	0.21652	0.2789	0.07715	0.13611	-0.01951	0.26493	0.24047	0.15952	0.22767	0.14168	0.02347
0.24083	0.18154	0.1742	0.11761	0.21795	0.27291	0.04508	0.15819	0.07003	0.24974	0.2271	0.16059	-0.01968	0.15386	0.01814
0.32409	0.17778	0.19277	0.21656	0.22084	0.27299	-0.04909	0.12747	0.06262	0.20965	0.17816	0.17408	0.15745	0.13243	0.06519
0.15638	0.16132	0.14245	0.06065	0.18937	0.26918	0.02088	0.14498	-0.09922	0.2168	0.18652	0.16499	0.35185	0.07561	-0.01965
0.16657	0.19791	0.1741	0.08814	0.20734	0.27868	0.07032	0.14995	0.05336	0.22577	0.20166	0.15117	0.28207	0.03521	-0.01914
0.25084	0.17704	0.16866	0.12763	0.21765	0.27593	-0.02493	0.13267	0.01081	0.22402	0.19522	0.16347	0.13152	0.08897	-0.01403
0.24085	0.20387	0.18784	0.11727	0.22828	0.26599	-0.03621	0.15215	0.01429	0.22356	0.19132	0.16102	0.05667	0.09882	-0.02529
0.23728	0.17948	0.16715	0.11405	0.21349	0.27709	-0.00289	0.11732	0.03456	0.19583	0.16746	0.15925	0.03575	0.0787	-0.01531
0.17167	0.22229	0.198	0.0662	0.20911	0.28225	0.03696	0.13928	-0.06433	0.26065	0.23969	0.16744	0.27377	0.09284	0.02108
0.2102	0.20889	0.19407	0.10725	0.2211	0.27428	0.0416	0.13087	0.00836	0.24496	0.23008	0.16249	0.31598	0.0386	-0.03479
0.29643	0.28015	0.27425	0.2211	0.22965	0.28137	0.07959	0.1839	-3.8E-05	0.3101	0.29814	0.18988	0.20143	0.16278	0.12871
0.21577	0.20143	0.18775	0.111	0.21965	0.27704	-0.01896	0.15801	-0.00722	0.27919	0.26338	0.1738	0.07692	0.06671	0.04166
0.19223	0.20491	0.18704	0.08828	0.20882	0.27369	0.03335	0.13198	-0.08308	0.23543	0.21441	0.15738	0.23778	0.07389	-0.00629
0.22054	0.24051	0.2256	0.14746	0.22111	0.26572	0.19453	0.13784	-0.04631	0.26816	0.25584	0.1697	0.25824	0.10208	0.06134
0.24441	0.19966	0.19334	0.14577	0.22711	0.27929	0.0072	0.15649	0.0574	0.28625	0.26519	0.16488	0.10737	0.06105	0.05111
0.22739	0.17271	0.16275	0.11044	0.20732	0.27138	0.04724	0.13005	0.03869	0.22951	0.20415	0.17055	0.00413	0.11277	0.00837

VENTS_IN	ENTS_IN	EDIATED	EDIATED	EDIATED	D_EVENT	LIC_ACI	IGNALING	SIGNALI	LING_BY	SIGNALI	_BY_BRA	_CSF1_M	NALING_B	_CTNNB1
0.01414	0.03193	0.08734	0.08734	0.08734	0.06696	-0.01012	0.18803	0.08981	0.24966	0.0994	0.20147	0.05084	0.11941	0.08008
-0.00708	0.03541	0.09229	0.09229	0.09229	0.11907	-0.00538	0.21594	0.12459	0.23417	0.20356	0.2036	0.01105	0.09106	0.02106
0.00377	0.03166	0.08125	0.08125	0.08125	0.03811	-0.05792	0.19926	0.06917	0.2279	0.03977	0.21626	0.07294	0.11305	0.1012
-0.02403	0.01876	0.06901	0.06901	0.06901	0.04162	0.04249	0.17938	0.10091	0.22864	0.06604	0.23717	0.09668	0.13707	0.06883
0.00809	0.06252	0.12072	0.12072	0.12072	0.11079	0.03711	0.21045	0.06963	0.24779	0.04459	0.2406	0.10983	0.15301	0.01986
-0.04699	-0.02807	0.02356	0.02356	0.02356	0.03387	-0.03056	0.09439	0.08861	0.22155	0.00921	0.19041	0.02078	0.08914	0.083
-0.02652	-0.01773	0.03638	0.03638	0.03638	0.07084	-0.05937	0.22544	0.09797	0.21378	0.08401	0.21457	0.07467	0.09131	-0.00312
-0.04533	-0.01713	0.03627	0.03627	0.03627	0.00496	0.04932	0.18895	0.0774	0.22425	0.08277	0.23905	0.10812	0.15815	0.02438
-0.05045	-0.02571	0.02655	0.02655	0.02655	-0.01928	0.0683	0.18258	0.09643	0.22702	0.04709	0.22841	0.11619	0.14771	0.03497
-0.0429	-0.01887	0.03491	0.03491	0.03491	-0.01148	0.04112	0.16708	0.08439	0.23142	0.0634	0.23771	0.1052	0.16235	-0.01133
-0.02695	-0.04606	-0.00392	-0.00392	-0.00392	-0.05406	-0.09035	0.19592	0.09949	0.22216	0.18106	0.18635	0.03114	0.05677	0.02526
-0.04453	-0.06748	-0.02175	-0.02175	-0.02175	-0.07293	-0.0164	0.2056	0.03849	0.22905	0.0789	0.20539	0.02597	0.07802	0.06187
0.09209	0.12417	0.19184	0.19184	0.19184	0.13508	-0.11311	0.24431	0.08268	0.21794	0.12712	0.1932	0.01996	0.08821	0.13077
-0.00159	0.05614	0.10152	0.10152	0.10152	0.09102	0.08912	0.17839	0.06021	0.22246	0.09582	0.23237	0.05002	0.11039	0.07145
-0.05265	-0.00487	0.04267	0.04267	0.04267	0.01043	0.02339	0.21748	0.03693	0.21764	0.06672	0.21713	0.02832	0.09115	0.05785
0.01364	0.00182	0.05522	0.05522	0.05522	0.05665	-0.07682	0.22377	0.00883	0.21491	0.15413	0.19428	-0.00437	0.07214	0.1198
0.00228	0.05287	0.11059	0.11059	0.11059	0.12866	0.00904	0.17346	0.12087	0.23166	0.07488	0.22386	0.07547	0.11423	0.06069
-0.037	0.005	0.05681	0.05681	0.05681	0.02181	0.05184	0.18165	0.08741	0.22846	0.06559	0.23028	0.09873	0.13025	0.02514

YTOSOL	SIGNALI	LING_BY_	SIGNALI	NG_BY_EF	ING_BY_E	SIGNALI	LING_BY_	SIGNALI	SIGNALI	ING_BY_	SIGNALI	ALING_B	ING_BY_	SIGNALI
0.02089	0.06839	0.12823	0.04957	0.08658	0.04887	0.03656	-0.01818	0.14278	0.1707	0.01844	0.1482	-0.01977	-0.00319	0.14244
-0.03433	0.02318	0.134	0.03674	0.07213	0.02996	0.02333	-0.05523	0.14529	0.14399	-0.0192	0.15441	0.03411	0.04135	0.14609
0.00674	0.08065	0.13781	0.06025	0.06255	0.04292	0.05221	-0.00398	0.16621	0.19419	-0.00546	0.16827	0.01503	0.02411	0.17304
0.05136	0.08746	0.14486	0.06131	0.06839	0.03561	0.08128	0.04426	0.12971	0.19027	0.02878	0.1352	-0.04954	-0.00316	0.16371
0.05315	0.08332	0.15861	0.04875	0.07605	0.04081	0.08538	0.06216	0.1379	0.18489	0.05721	0.14737	-0.02439	0.0126	0.16895
0.0006	0.05008	0.11718	0.0214	0.05725	0.01819	0.03577	-0.00591	0.116	0.13126	-0.00563	0.12356	-0.01647	-0.00208	0.11338
0.01601	0.05557	0.12794	0.06747	0.05755	0.02575	0.07066	0.0088	0.14406	0.16084	-0.0169	0.1526	0.01112	0.0292	0.14592
0.06914	0.09959	0.13712	0.05135	0.04779	0.02124	0.0772	0.06201	0.10376	0.17697	0.03853	0.11479	-0.08064	-0.03627	0.16628
0.07404	0.09033	0.1482	0.04707	0.05358	0.02589	0.075	0.07345	0.11112	0.18442	0.03489	0.11966	-0.05267	-0.01507	0.16312
0.05646	0.07882	0.12242	0.03436	0.03263	0.00438	0.06323	0.0607	0.1093	0.14957	0.02153	0.12296	-0.03506	-0.01722	0.14709
-0.027	0.02261	0.14244	0.06814	0.08065	0.05004	0.05105	-0.0717	0.13922	0.14738	-0.02782	0.14143	-0.03111	-0.01525	0.12896
0.01345	0.01329	0.10293	0.04095	0.04052	0.0095	0.01639	-0.05935	0.1285	0.13064	-0.0125	0.12153	-0.03414	-0.03664	0.1324
-0.03682	0.04249	0.16184	0.09144	0.13489	0.09363	0.05276	-0.07042	0.15552	0.20059	-0.01099	0.15896	0.00135	0.01234	0.17883
-0.00648	0.04544	0.1117	0.04867	0.04452	0.01814	0.04562	0.00343	0.13516	0.15421	0.00246	0.13436	-0.03623	-0.01432	0.14822
-0.01595	0.02767	0.09603	0.00773	0.00968	-0.01953	0.0468	-0.02071	0.12919	0.15412	-0.0185	0.1341	-0.03149	-0.0321	0.12971
-0.06586	0.03293	0.11117	0.0905	0.0729	0.0274	0.0241	-0.1332	0.14043	0.13925	-0.04406	0.14278	-0.01272	-0.01676	0.11664
0.02915	0.08268	0.16041	0.07048	0.10727	0.06357	0.07272	0.04331	0.15277	0.18597	0.02835	0.16069	-0.02419	0.02568	0.1701
0.06722	0.08365	0.15546	0.04704	0.06687	0.03697	0.07915	0.06347	0.12529	0.18425	0.04299	0.13642	-0.04669	0.00134	0.16791

SIGNALI	ING_BY_	LING_BY_	G_BY_FL	BY_FLT3	SIGNALI	NALING_	SIGNALI	NG_BY_I	ALING_BY	LING_BY	SIGNALIN	ALING_BY	TETHER	SIGNALI
0.16432	0.03426	0.00304	-0.00952	-0.01312	0.04331	0.22618	0.09969	0.11318	0.19105	0.09234	-0.0015	0.43544	-0.1278	0.09169
0.15642	0.0579	0.0029	-0.02537	-0.04226	0.02195	0.22336	0.12038	0.07283	0.1714	0.04801	-0.09123	0.40056	-0.2186	0.03676
0.18745	0.04462	0.00293	0.03063	-0.01547	0.04501	0.21534	0.10313	0.12305	0.18537	0.08052	-0.09233	0.449	-0.08142	0.06301
0.18678	0.09713	-0.00311	0.07649	0.0064	0.09552	0.23388	0.09086	0.1482	0.20297	0.12998	-0.05283	0.39123	-0.0283	0.09493
0.19064	0.09343	0.02462	0.0654	0.0333	0.07608	0.24956	0.13114	0.09958	0.21243	0.13534	-0.00172	0.43086	-0.01136	0.0615
0.13216	0.03088	-0.0102	0.0356	-0.04212	0.0525	0.2234	0.09681	0.0162	0.18128	0.07584	-0.00956	0.37872	-0.07516	0.03365
0.16466	0.07002	-0.00553	0.05959	0.00191	0.07033	0.21726	0.09382	0.07634	0.19645	0.13905	-0.08751	0.41272	-0.03736	0.06239
0.18846	0.0646	-0.00984	0.07348	0.01178	0.10027	0.2552	0.10765	0.13263	0.21693	0.13381	0.0064	0.42153	-0.06457	0.09342
0.18786	0.069	-0.00074	0.06822	0.00138	0.10155	0.24925	0.08214	0.13284	0.21847	0.13482	-0.0208	0.42364	-0.01989	0.07425
0.16715	0.02296	-0.00159	0.07612	-0.00941	0.1039	0.23774	0.09198	0.11041	0.21708	0.14502	0.02612	0.42522	-0.01308	0.05998
0.14096	0.02055	-0.02934	-0.00863	-0.0224	0.02137	0.22598	0.1428	0.08714	0.17269	0.01976	-0.12251	0.3722	-0.16396	0.05816
0.12303	-0.04226	-0.02152	0.00715	-0.05326	0.05084	0.2278	0.16825	0.04359	0.18676	0.05286	-0.05648	0.4298	-0.10339	0.059
0.19627	0.03676	-0.00607	-0.00405	-0.02271	0.04695	0.24692	0.14501	0.12199	0.16101	0.01476	-0.09689	0.41289	-0.1571	0.07176
0.15142	0.03434	-0.01322	0.02324	-0.0393	0.06118	0.24536	0.15894	0.12044	0.2009	0.0693	-0.10418	0.36516	-0.04388	0.06008
0.14897	-0.03318	-0.02407	-0.00518	-0.0761	0.03905	0.22832	0.1428	0.08392	0.19192	0.02126	-0.0258	0.43086	-0.12372	0.03852
0.13016	-0.02585	-0.03142	-0.03899	-0.04993	0.00418	0.21537	0.18734	0.07698	0.15204	0.01133	-0.13597	0.40285	-0.31691	0.07839
0.18946	0.1313	0.00689	0.07326	0.01866	0.07872	0.23087	0.12783	0.13816	0.19415	0.12926	-0.09193	0.37872	-0.08017	0.1077
0.19244	0.10317	0.00702	0.06549	0.03951	0.08126	0.25309	0.1143	0.13114	0.20057	0.15222	-0.02593	0.42329	-0.04394	0.07158

ERATE_K	_BY_MRA	SIGNALI	SIGNALI	SIGNALI	GNALING	TCH1_HD	H1_PES	9_NOTCH	IGNALIN	IGNALIN	GNALING	NALING_B	NALING_B	SIGNALIN
0.25449	0.18131	0.08673	0.18803	0.2476	0.02822	0.27234	0.06956	0.1511	0.13873	0.14118	0.27134	0.06072	-0.00827	0.12225
0.24689	0.15853	-0.07928	0.21594	0.25633	0.02922	0.14385	0.07754	-0.07056	0.11436	0.14952	0.26763	0.11033	0.05437	0.11733
0.27927	0.1509	-0.12363	0.19926	0.24512	0.05918	0.22352	0.10315	0.06465	0.11144	0.13397	0.25179	0.07397	0.02781	0.11737
0.29474	0.17125	-0.17758	0.17938	0.24565	0.06887	0.29548	0.08917	0.18798	0.10449	0.14506	0.25853	0.10019	0.05425	0.13178
0.3007	0.16812	-0.20638	0.21045	0.25866	0.04557	0.15921	0.06054	-0.04699	0.09638	0.12151	0.2798	0.09032	0.06536	0.13047
0.25292	0.19237	-0.05523	0.09439	0.24697	-0.00317	0.18496	0.08194	0.00191	0.06949	0.13986	0.26977	0.09568	0.0236	0.12303
0.27094	0.14672	0.07937	0.22544	0.23973	0.03591	0.23179	0.07406	0.08214	0.07073	0.08476	0.25574	0.06792	0.04778	0.11233
0.30333	0.1299	-0.30017	0.18895	0.25049	0.0471	0.24026	0.04967	0.09655	0.1183	0.13199	0.27457	0.08756	0.03892	0.13815
0.2869	0.14402	-0.31988	0.18258	0.24729	0.01864	0.20612	0.0179	0.0366	0.10127	0.1161	0.27209	0.08441	0.04942	0.13269
0.29406	0.16223	-0.16435	0.16708	0.24546	0.02811	0.23466	0.05006	0.08772	0.11016	0.12927	0.26345	0.06354	0.02965	0.13132
0.24069	0.1617	-0.05146	0.19592	0.2525	0.04032	0.13669	0.10176	-0.10312	0.09356	0.15518	0.26076	0.05329	0.00406	0.09545
0.2581	0.15123	-0.16266	0.2056	0.24687	0.00287	0.15655	0.05932	-0.05387	0.08941	0.09443	0.25994	0.0396	-0.04603	0.11889
0.23805	0.17269	0.11809	0.24431	0.25584	0.05406	0.19231	0.11078	0.00709	0.09241	0.14608	0.2714	0.10209	0.0636	0.14071
0.28623	0.17392	-0.09844	0.17839	0.24558	0.028	0.16792	0.0549	-0.02891	0.07519	0.12031	0.26388	0.073	0.03103	0.13158
0.27201	0.16725	-0.15909	0.21748	0.23952	0.01858	0.22771	0.06564	0.07541	0.08291	0.11376	0.25767	0.04586	-0.01933	0.11027
0.25276	0.18233	-0.01571	0.22377	0.23698	0.00664	0.23772	0.08074	0.08755	0.10272	0.15214	0.25506	0.04909	-0.02077	0.09935
0.27679	0.16466	-0.07841	0.17346	0.2452	0.0279	0.19504	0.06861	0.01932	0.10767	0.12622	0.25185	0.13424	0.06828	0.1302
0.28979	0.12904	-0.26749	0.18165	0.24791	0.01959	0.17067	0.02996	-0.02475	0.07817	0.10927	0.27386	0.09621	0.06205	0.13265

SIGNALIN	Y_RECEP	ALING_BY	GTPASES	ALING_BY	ING_BY_	IGNALING	G_BY_TGF	Y_TGF_BE	BETA_RE	Y_THE_B	LIN_LIKE	SIGNALIN	SIGNALIN	LING_BY	IGNALLI	SIGNALLI	IGNAL_A	SIGNAL_A	ORY_PRO	AL_TRAN	ELY_REG	OF_RE	ED_TRAN	RANSPO
0.04417	0.09637	0.10721	0.14875	-0.29047	0.31643	0.06986	0.09792	0.0976	-0.09861	0.19809	-0.05067	0.13413	0.25326	0.03703	0.196	0.15392	0.18492	0.09767	0.12032	0.08268	0.44221	0.19903	0.03525	0.07439
0.05207	0.06904	0.06571	0.13594	-0.40356	0.32754	0.05114	0.12277	0.11573	0.06139	0.18769	-0.10192	0.09186	0.24746	0.03662	0.14205	0.09554	0.16654	0.05427	0.09345	0.08119	0.44454	0.25476	0.02979	0.07715
0.06719	0.08926	0.09114	0.14533	-0.10041	0.30441	0.07986	0.11823	0.11931	-0.0101	0.1805	-0.11006	0.11901	0.2509	0.09649	0.14668	0.07886	0.18639	0.07721	0.12767	0.10534	0.44976	0.21534	0.04283	0.10882
0.05325	0.10011	0.03865	0.15454	-0.44548	0.32108	0.10041	0.11211	0.11084	-0.04663	0.20631	-0.06359	0.13818	0.25644	0.03886	0.16909	0.12604	0.27364	0.07721	0.14885	0.08519	0.44192	0.2047	0.05151	0.0567
0.06998	0.08348	0.04263	0.15333	-0.41747	0.33184	0.1284	0.10472	0.10554	0.02333	0.2363	-0.05245	0.12367	0.25896	0.00423	0.17458	0.12996	0.22125	0.0684	0.16536	0.0632	0.44553	0.20422	0.00488	0.07691
0.04391	0.0737	0.06056	0.14129	-0.30298	0.3281	0.04394	0.08906	0.0908	-0.02906	0.19805	-0.09702	0.10802	0.24705	0.0605	0.1314	0.08765	0.18184	-0.02447	0.12607	0.05235	0.44718	0.20067	0.03901	0.08126
0.04686	0.10069	0.04294	0.1396	-0.24943	0.32131	0.10261	0.12677	0.12488	0.03617	0.19583	-0.02822	0.1362	0.24211	0.00478	0.16713	0.10582	0.21716	0.04878	0.1281	0.09883	0.45401	0.19918	0.0404	0.06981
0.07933	0.09269	0.06521	0.1558	-0.41536	0.32286	0.12075	0.10931	0.10996	0.00034	0.22511	-0.10637	0.12836	0.25314	0.00293	0.17144	0.08414	0.22497	0.06099	0.20113	0.0912	0.43871	0.17894	0.0592	0.04399
0.07383	0.09573	0.01681	0.15679	-0.41377	0.32294	0.12055	0.11582	0.11844	0.00891	0.22857	-0.10894	0.13994	0.2533	0.02054	0.15275	0.09315	0.2068	0.05972	0.16981	0.10058	0.4436	0.16761	0.03986	0.04766
0.05915	0.09366	0.03557	0.15055	-0.3944	0.31891	0.13111	0.10198	0.10314	-0.01215	0.22497	-0.08232	0.13433	0.25229	-0.02545	0.16678	0.10524	0.24918	0.05756	0.20953	0.0826	0.44711	0.1867	0.01963	0.04589
0.07773	0.07303	0.07818	0.14292	-0.43051	0.31971	0.04201	0.12184	0.11749	0.03219	0.17746	-0.10791	0.10949	0.2459	0.03611	0.13464	0.05024	0.17895	0.02028	0.01896	0.1141	0.44583	0.20757	0.01808	0.06071
0.03766	0.07753	0.06857	0.14234	-0.38119	0.3185	0.04261	0.11416	0.11156	-0.05161	0.18605	-0.14924	0.1017	0.24922	0.04188	0.14072	0.0677	0.22512	0.04192	0.08649	0.09639	0.44972	0.21291	0.01972	0.07365
0.04924	0.08357	0.08263	0.14802	-0.08455	0.32058	0.00998	0.14932	0.14517	0.03224	0.1699	-0.06234	0.10433	0.25809	0.14668	0.18338	0.14159	0.19958	0.14096	0.06298	0.13597	0.44324	0.20402	0.02215	0.09054
0.07665	0.09317	0.10064	0.15592	-0.38224	0.32372	0.06744	0.11866	0.11651	-0.04275	0.19934	-0.08181	0.13703	0.25091	0.05154	0.16798	0.12208	0.18611	0.0651	0.11028	0.08273	0.44979	0.18996	0.04427	0.09149
0.03195	0.07278	0.08526	0.14029	-0.34279	0.32627	0.03824	0.10648	0.10537	-0.00239	0.18659	-0.11364	0.11542	0.23963	0.03084	0.15755	0.05881	0.17733	0.11313	0.1243	0.09123	0.44301	0.23222	0.01957	0.05616
0.04768	0.07978	0.0832	0.13793	-0.23481	0.31783	0.01744	0.12364	0.1171	0.02109	0.15252	-0.07099	0.09011	0.23613	0.09626	0.15358	0.07136	0.14451	0.05212	0.04948	0.12888	0.44255	0.17508	0.03893	0.08933
0.07284	0.09939	0.05596	0.15497	-0.40197	0.32171	0.09699	0.10199	0.10137	-0.05826	0.19377	-0.02806	0.12753	0.25502	0.05525	0.16063	0.1135	0.27161	0.0492	0.10973	0.05484	0.44926	0.19842	0.01354	0.07119
0.07579	0.08234	0.08291	0.15757	-0.37872	0.32057	0.09738	0.11578	0.11661	-0.03523	0.22565	-0.09921	0.13433	0.25988	0.0047	0.16654	0.09398	0.23103	0.07112	0.16399	0.08319	0.44295	0.18519	0.05641	0.07587

EGULATE	ETEROT	FERING_	TH_MUSC	SNRNP_	IM_CALC	LED_PHO	UM_PROT	E_SOMIT	G_MEDIAT	N_OF_PR	RM_MOTI	LIPID_DE	INGOLIP	GULATION	LATIONA	TABILIZ	ENTS_DO	STATS_A	DOWNS	ULI_SENS	DUCTION	ED_MUSC	E_OXID	R_AMINO
0.08884	0.1236	0.01895	0.28401	0.10327	0.43254	0.02348	-0.31854	0.29033	0.04986	-0.36005	0.16611	0.04441	0.07396	0.28399	0.36335	0.28084	0.16131	0.0034	-0.0134	-0.01446	0.123	0.32039	0.18812	0.17172
0.10614	0.15377	0.06539	0.27694	0.12601	0.42821	-0.06546	-0.17847	0.29484	0.06333	0.07839	-0.16787	0.07351	0.05696	0.26749	0.38266	0.28516	0.23497	-0.05858	-0.0422	-0.03097	0.12585	0.32767	0.16903	0.11011
0.11271	0.11054	0.00346	0.2961	0.13719	0.44186	0.05121	-0.17715	0.2728	0.03975	0.14691	-0.12982	0.08552	0.07581	0.31383	0.36313	0.26357	0.12793	-0.03538	-0.03659	-0.01436	0.13699	0.34651	0.18129	0.14746
0.10694	0.14826	-0.04043	0.31473	0.07081	0.37882	-0.03166	-0.21076	0.27635	0.05562	-0.49181	0.16382	0.08154	0.13981	0.30492	0.38458	0.26537	0.1366	0.0047	-0.03883	0.01783	0.14406	0.32851	0.15793	0.07571
0.11153	0.13692	-0.02884	0.26275	0.09413	0.39846	-0.15049	-0.22162	0.31128	0.10316	-0.30932	0.17879	0.05635	0.09683	0.30433	0.37498	0.29883	0.13472	0.03016	0.01296	-0.00053	0.13774	0.33292	0.12331	0.09559
0.07506	0.103	-0.03699	0.27846	0.11076	0.40602	-0.02301	-0.11327	0.2921	-0.02649	0.1691	0.03153	0.07835	0.10689	0.24024	0.38436	0.28111	0.18204	0.01337	-0.01728	-0.02564	0.08121	0.3122	0.17665	0.1035
0.14047	0.16257	-0.05969	0.2804	0.10159	0.39012	0.03948	-0.24407	0.27494	0.00747	0.05795	-0.46715	0.06255	0.06689	0.27702	0.37498	0.26061	0.14644	-0.02566	-0.01767	-0.02491	0.14741	0.33032	0.24435	0.16842
0.0695	0.13117	-0.00365	0.29887	0.03831	0.3638	-0.22775	-0.19231	0.29731	0.02412	-0.48547	0.30632	0.06491	0.12837	0.32139	0.38304	0.28683	0.10242	0.03907	0.00464	0.05369	0.1444	0.35426	0.14865	0.10836
0.12596	0.13006	-0.02736	0.31081	0.05223	0.37965	-0.1733	-0.18113	0.30101	0.0008	-0.33539	0.2526	0.0839	0.15419	0.31639	0.37479	0.29193	0.10421	0.02402	-0.00519	0.06827	0.14661	0.35135	0.105	0.08402
0.14547	0.12061	-0.02541	0.28772	0.07674	0.35775	-0.14649	-0.19511	0.28578	0.01182	-0.48318	0.30439	0.05886	0.1172	0.31082	0.36925	0.28099	0.13116	0.07166	0.0185	0.03091	0.15111	0.32297	0.13517	0.08844
0.08693	0.09692	0.01562	0.2776	0.10562	0.40337	-0.00658	-0.28284	0.27971	-0.02486	0.10974	-0.37273	0.07987	0.0484	0.24832	0.37703	0.26977	0.1891	-0.1295	-0.05072	0.01417	0.11633	0.29927	0.20289	0.15923
0.02201	0.09681	0.02185	0.27403	0.10629	0.40965	-0.31271	-0.19046	0.28648	-0.02766	-0.43808	-0.05602	0.05464	0.05633	0.26809	0.37791	0.28314	0.15683	-0.04464	-0.01621	-0.01064	0.15611	0.32803	0.19817	0.16568
0.05514	0.14276	0.02784	0.27517	0.12695	0.38998	0.1342	-0.22681	0.2888	0.08138	-0.00757	-0.48318	0.1027	0.06002	0.28515	0.36758	0.27921	0.20384	-0.09729	-0.05918	-0.04621	0.09845	0.29143	0.12189	0.14292
0.04942	0.12505	0.05014	0.27402	0.11443	0.40262	-0.16871	-0.24507	0.28262	0.07389	-0.31636	0.06148	0.06644	0.11607	0.27664	0.38246	0.26804	0.15718	-0.05447	-0.05862	-0.00188	0.13863	0.32128	0.18939	0.10609
0.11725	0.13168	0.02592	0.30403	0.09173	0.37343	0.03654	-0.32067	0.28733	0.03891	-0.4941	0.02396	0.05074	0.07098	0.29168	0.38359	0.27325	0.11124	-0.02262	-0.05323	-0.01188	0.10215	0.33965	0.22663	0.14879
0.06874	0.12142	0.03538	0.28272	0.11817	0.37718	0.22987	-0.19942	0.27414	0.02743	0.00335	-0.43227	0.0485	0.02963	0.2591	0.3676	0.25756	0.16777	-0.08776	-0.04036	-0.02992	0.12189	0.31913	0.19432	0.17077
0.098	0.12936	0.02957	0.28532	0.09059	0.35868	0.14495	-0.26136	0.2733	0.0948	-0.1108	0.03858	0.05633	0.10678	0.28665	0.38052	0.27027	0.15705	-0.0372	-0.06942	-0.03705	0.13901	0.33132	0.20519	0.11588
0.10398	0.11413	-0.01207	0.29297	0.07488	0.40566	0.0816	-0.11155	0.30104	0.046	-0.3065	0.17315	0.07636	0.11872	0.30965	0.37317	0.28828	0.10744	0.01263	0.00905	0.04518	0.17231	0.34258	0.13792	0.0966

ME_SUMO	CHROMA	DAMAGE_	OF_DNA	OF_DNA	OF_IMMU	OF_INT	ON_OF_R	N_OF_SI	OF_TRA	N_OF_TR	OF_UBI	CONJUGA	PROTEOL	ERRED_	PRESSION	N_OF_PH	FACTAN	RIGINS_T	ADHESIO	DECAN_	EICOSAT	EICOSAT	HYDROX	OF_5_EI
0.15669	0.18792	0.06729	-0.00692	0.09703	0.16661	0.22392	0.08843	0.1001	0.13307	0.09139	0.09452	0.34535	0.41538	0.35308	0.32306	0.27559	0.0154	0.22074	0.15144	0.03212	0.10878	0.03545	-0.09783	0.20184
0.20451	0.21646	0.11716	0.09621	0.12863	0.20121	0.20471	0.1234	0.10881	0.1942	0.13069	0.10273	0.34958	0.38889	0.36574	0.24344	0.24809	-0.009	0.20804	0.11511	0.01796	0.28607	0.18246	-0.44265	0.22451
0.19243	0.20605	0.10423	-0.04246	0.14563	0.2188	0.18932	0.12183	0.13932	0.13549	0.13468	0.13061	0.37148	0.39217	0.38306	0.33459	0.24533	-0.0164	0.2094	0.0722	0.03341	0.13743	0.04705	-0.36116	0.18773
0.13784	0.15885	0.02822	-0.06157	0.07347	0.17179	0.16283	0.05068	0.06078	0.10542	0.03759	0.05412	0.2722	0.35727	0.30018	0.32242	0.28034	0.03124	0.21167	0.07054	0.07574	0.18493	0.08728	-0.43391	0.32917
0.16087	0.17831	0.06671	-0.02583	0.09825	0.19456	0.1925	0.0847	0.08915	0.12663	0.05921	0.07943	0.34078	0.40155	0.35038	0.33035	0.26523	-0.07566	0.225	0.01378	-0.02839	0.15485	0.06443	-0.39107	0.2527
0.17068	0.18626	0.07701	-0.00763	0.11153	0.18227	0.2043	0.08996	0.09559	0.12296	0.06503	0.08217	0.34227	0.40587	0.35559	0.22816	0.29314	-0.01319	0.23084	0.06293	0.02318	0.31948	0.20876	-0.36593	0.33397
0.16641	0.18408	0.06895	0.00758	0.10514	0.23297	0.23171	0.09162	0.09587	0.13581	0.05433	0.08301	0.3718	0.44683	0.38343	0.29743	0.21857	-0.02619	0.20499	-0.02341	0.1207	0.32872	0.21953	-0.35245	0.26589
0.11231	0.13479	-0.00262	-0.11352	0.03256	0.14275	0.14467	0.02097	0.02401	0.07552	-0.01668	0.01178	0.25591	0.32994	0.27494	0.33343	0.2829	0.01359	0.20695	0.01406	0.0499	0.23499	0.13912	-0.39325	0.28936
0.12479	0.14805	0.01613	-0.07132	0.05384	0.18643	0.20071	0.04098	0.04806	0.09803	0.02993	0.0353	0.31384	0.41427	0.32468	0.31265	0.27065	0.00955	0.2079	0.00991	0.04548	0.16672	0.15078	-0.44292	0.29907
0.13799	0.15778	0.03693	-0.04856	0.06728	0.20393	0.1795	0.05648	0.06022	0.10242	0.01564	0.05191	0.31057	0.37378	0.32922	0.33315	0.26277	0.03319	0.19284	0.02313	0.02223	0.18685	0.09222	-0.17748	0.28525
0.17893	0.17817	0.0707	0.00068	0.1087	0.20319	0.17813	0.07753	0.08248	0.1416	0.11352	0.07256	0.33434	0.40498	0.3478	0.28734	0.25719	-0.01775	0.23546	0.08747	0.00382	0.10523	0.01272	-0.20135	0.16947
0.17423	0.18955	0.0863	-0.01134	0.12111	0.20506	0.21439	0.10076	0.11168	0.12835	0.09822	0.10011	0.3474	0.42042	0.36392	0.32248	0.23925	-0.05627	0.21945	0.07891	0.04538	0.22144	0.13265	-0.38231	0.20906
0.19473	0.20879	0.10953	-0.01514	0.13832	0.18728	0.24042	0.11185	0.12384	0.16093	0.15279	0.11815	0.36762	0.41112	0.37868	0.28768	0.25179	-0.05637	0.23114	0.19075	0.10702	0.08939	0.0014	-0.30985	0.08218
0.16403	0.18427	0.07228	-0.06659	0.11893	0.19061	0.14887	0.09584	0.11031	0.11601	0.02156	0.10056	0.25391	0.30947	0.29016	0.31944	0.25373	-0.03555	0.18997	0.06805	0.04149	0.20423	0.1255	-0.4425	0.27939
0.15108	0.16711	0.04853	-0.03772	0.08456	0.18907	0.19231	0.06755	0.07057	0.11339	0.04407	0.06012	0.31133	0.4136	0.3267	0.33741	0.27026	-0.01087	0.21526	0.06212	0.00838	0.26532	0.17424	-0.20827	0.26129
0.18128	0.18965	0.08166	-0.01868	0.1163	0.22282	0.21422	0.09792	0.10195	0.14981	0.08027	0.09686	0.3459	0.3907	0.3598	0.27227	0.2581	-0.07637	0.22203	0.10476	0.05039	0.10692	0.02505	-0.42007	0.05807
0.1608	0.17982	0.06012	-0.06033	0.09305	0.15843	0.18383	0.08065	0.08844	0.12811	0.10255	0.07992	0.32073	0.37013	0.34323	0.32489	0.25834	0.02761	0.17584	0.07656	0.01224	0.16344	0.06022	-0.23514	0.26744
0.13708	0.16001	0.03629	-0.07189	0.07666	0.19042	0.19015	0.06095	0.06794	0.09961	0.01658	0.05795	0.31956	0.40902	0.33161	0.31516	0.28065	0.0522	0.21279	0.02066	0.07493	0.15854	0.05894	-0.43383	0.22385

UBIQUIT	OF_BILE	AND_BIL	AND_BILE	D_BILE	SIS_OF	SYNTHES	IS_OF_DO	AND_DIH	HESIS_OF	LYCOSYL	IP2_IP	OF_IP3_A	ESIS_OF	LEUKOT	THESIS_C	_SYNTHE	_SYNTHE	_SYNTHE	_SYNTHE	_SYNTH	S_AT_THE	OF_PIPS	F_PIPS_A	S_AT_THE
0.11959	0.0497	-0.0393	-0.06159	0.04233	0.01671	0.1759	-0.08838	-0.08077	0.067	-0.36693	-0.04625	-0.03053	0.09446	0.03999	0.19105	-0.06005	0.07152	0.00974	0.09564	0.0662	-0.1465	0.07317	0.03564	-0.0785
0.12263	-0.02206	-0.15421	-0.18775	-0.02348	0.01328	0.18188	-0.46071	-0.37663	0.19902	-0.29064	-0.10523	-0.06144	0.02183	0.08584	0.19386	-0.03524	-0.00331	-0.04864	0.20932	0.00551	-0.1814	0.10339	0.03691	-0.15337
0.10136	-0.00431	-0.03171	-0.02378	-0.0066	0.02986	0.18219	-0.27426	-0.40616	0.11298	-0.21009	-0.06859	-0.01584	-0.00382	-0.00199	0.10719	-0.03667	0.05635	-0.00023	0.12078	0.02416	-0.17151	0.05075	0.02702	-0.12801
0.10031	0.02454	-0.0424	-0.06049	0.02362	0.02479	0.17718	-0.2956	-0.3776	0.11327	-0.29818	-0.139	-0.02859	0.04788	0.03483	0.17916	-0.04193	0.03983	0.09917	0.27454	0.18533	-0.12803	0.077	0.07439	-0.10154
0.11907	0.00185	-0.07429	-0.06387	-0.00353	0.01929	0.18584	0.01964	-0.36065	0.15776	-0.40839	-0.03566	0.02558	0.03039	0.03971	0.2071	0.0132	-0.01133	-0.06197	0.0624	0.11257	-0.1408	0.10332	0.03385	-0.08738
0.08491	-0.01248	-0.09631	-0.11093	-0.02631	0.01615	0.20001	-0.34285	-0.19586	0.09653	-0.29853	-0.1129	-0.0488	-0.12387	0.09936	0.15771	0.00682	0.02418	-0.08508	0.16438	-0.18208	-0.20579	0.08823	0.00455	-0.18457
0.08218	-0.09601	-0.3462	-0.33402	-0.13866	0.0125	0.17434	-0.32451	-0.22788	0.17316	-0.33148	-0.03375	-0.06262	0.0295	0.10882	0.21199	-0.05086	-0.01291	0.0212	0.04532	-0.10612	-0.14147	0.09395	0.03444	-0.065
0.11441	0.05307	0.08992	0.09906	0.06577	0.01467	0.16391	-0.20802	-0.33617	0.13239	-0.32702	-0.0782	0.00402	0.0619	0.04206	0.15104	-0.10977	0.04716	0.05583	0.20346	0.04266	-0.13651	0.05065	0.07793	-0.12819
0.10394	0.01796	0.02991	0.02864	0.02767	0.01571	0.16511	-0.20672	-0.36395	0.11049	-0.38595	-0.06807	0.01696	-0.02983	-0.00144	0.13556	-0.09704	0.04597	0.01545	0.20246	-0.06062	-0.10227	0.07306	0.07493	-0.09355
0.11105	-0.03419	-0.12096	-0.12624	-0.06065	0.00219	0.161	-0.09902	-0.18148	0.16065	-0.28505	-0.0558	-0.02062	0.03756	0.02724	0.16726	-0.06904	0.03662	0.05222	0.22896	-0.00558	-0.11576	0.05944	0.06306	-0.09006
0.14071	0.00543	-0.17828	-0.1808	-0.01917	0.03597	0.20032	-0.07866	-0.45139	0.15904	-0.09559	-0.05739	-0.07079	0.10497	0.02237	0.13734	-0.02616	0.07075	0.05486	0.06143	0.03794	-0.18722	0.05875	0.03297	-0.10226
0.10442	-0.0694	-0.28953	-0.29411	-0.12048	0.01417	0.18862	-0.16515	-0.2435	0.14319	-0.20823	-0.08376	-0.07847	0.02364	-0.02074	0.06504	-0.0022	0.04318	0.02503	0.05355	-0.05209	-0.20982	0.05057	0.00187	-0.17481
0.14591	-0.06864	-0.41903	-0.38471	-0.09872	0.01982	0.20067	-0.23555	-0.21594	0.16405	-0.01267	-0.03608	-0.10936	0.15619	-0.13187	-0.07106	0.04762	0.04977	0.04525	0.0514	0.01066	-0.177	0.11387	0.03496	-0.13589
0.12803	0.05718	0.00024	0.00482	0.05892	0.02549	0.14944	-0.3079	-0.38826	0.16149	-0.17819	-0.11604	-0.06641	0.17217	0.05687	0.18119	-0.05689	0.04985	0.08101	0.09028	0.0398	-0.13233	0.07072	0.06551	-0.09754
0.12751	0.00846	-0.04811	-0.09031	0.00327	0.02022	0.1821	-0.07232	-0.30313	0.19626	-0.27565	-0.05842	-0.05489	0.06749	0.12789	0.24309	-0.11082	0.06259	0.11645	0.11992	0.00031	-0.19381	0.05653	0.0231	-0.16747
0.13326	-0.00814	-0.26024	-0.25902	-0.02803	0.02231	0.19198	-0.27541	-0.1609	0.22238	-0.12325	-0.01035	-0.0933	0.12464	-0.09312	-0.02455	-0.05037	0.06039	0.06237	-0.02146	-0.00563	-0.20023	0.0845	0.00704	-0.12722
0.08927	-0.01385	-0.10593	-0.10569	-0.02496	0.04611	0.13229	-0.27946	-0.26142	0.07246	-0.25334	-0.09907	-0.03915	0.13004	0.04916	0.16015	-0.03381	0.04798	0.08145	0.20018	0.01588	-0.16473	0.07172	0.04341	-0.15012
0.10201	0.01501	-0.00356	-0.0079	0.02021	0.01907	0.16459	-0.24191	-0.38645	0.11347	-0.30428	-0.06718	0.01325	0.02939	-0.00561	0.13444	-0.06344	-0.00322	-0.00253	0.21649	-0.07306	-0.13521	0.06982	0.0619	-0.14601

PIPS_AT	TAGLAND	PYROPHO	BSTRATE	F_UDP_	VERY_LON	TION_AN	ACTIVATI	OF_GLUC	TOME_S	FT_IKK_A	ME_TBC	FT_SIGNA	E_TCR_S	STRAND_L	C_STRAN	E_EXTEN	LOMERE_L	PATHWA	N_OF_O	OF_TRAN	YNTHESI	SSOR_DU	CRIPTIO	EPTOR_SI
-0.05406	0.04748	-0.02153	0.05058	0.09252	0.07537	0.15931	0.15838	0.09409	0.1373	-0.00899	0.05835	0.28223	0.17841	-0.06801	-0.28222	0.05391	0.16608	-0.16459	0.12979	0.01288	0.09881	0.37738	-0.10685	0.07878
-0.09161	0.07795	-0.08162	0.07336	0.05508	0.00579	0.15559	0.16058	0.09805	0.15152	0.01451	0.01384	0.28039	0.16086	0.01709	-0.30329	0.11369	0.20261	-0.14119	0.11275	0.09889	0.11818	0.43054	-0.14199	0.10668
-0.09207	0.09493	-0.03242	0.03702	0.1175	-0.01134	0.18774	0.18623	0.15579	0.15573	0.00311	0.00558	0.28025	0.16369	0.01494	-0.2492	0.07112	0.19856	-0.11428	0.07504	0.10906	0.17034	0.42292	-0.10955	0.12819
-0.04482	0.05287	-0.08568	0.05999	0.09465	0.06602	0.13983	0.18125	0.14126	0.14092	-0.0079	0.09002	0.27449	0.19285	-0.01866	-0.28866	0.03662	0.16714	0.06601	0.01364	0.08785	0.09012	0.38272	-0.22537	0.11054
-0.03435	0.03915	0.02342	0.07291	0.07978	0.06058	0.17186	0.18324	0.16712	0.15828	0.02021	0.05377	0.28272	0.23661	-0.02204	-0.17137	0.05987	0.17292	-0.05729	-0.04109	0.06495	0.11983	0.39243	-0.19739	0.1023
-0.07788	-0.02334	0.06847	0.00407	-0.02646	0.01485	0.19843	0.19304	0.19856	0.17205	-0.01521	0.05359	0.28205	0.2	0.03368	-0.14222	0.10036	0.19374	0.25879	0.01057	0.12627	0.11203	0.41211	-0.12039	0.08726
-0.10155	0.04737	-0.09581	0.02192	0.05154	0.01116	0.18666	0.22101	0.15807	0.14853	0.03015	0.0002	0.27254	0.1983	-0.00253	-0.17186	0.11985	0.20213	0.26697	-0.0083	0.10832	0.15145	0.42244	-0.13202	0.13462
-0.01155	0.03033	0.00573	0.08315	0.1481	0.03557	0.21974	0.20211	0.21471	0.12994	0.03167	0.07303	0.27252	0.21459	-0.06822	-0.28871	-0.00141	0.13967	0.03138	-0.0596	0.05547	0.0978	0.36191	-0.2504	0.11073
-0.00324	0.05193	0.00623	0.06437	0.12501	0.07408	0.22209	0.19123	0.20107	0.12961	0.00523	0.06689	0.278	0.23571	-0.06298	-0.24506	0.00472	0.1473	0.11607	-0.15877	0.06825	0.10449	0.36108	-0.26184	0.12423
-0.01859	-0.03923	-0.06626	0.07211	0.10617	0.05678	0.20536	0.1557	0.1652	0.13161	0.04117	0.06567	0.27492	0.22791	-0.00366	-0.11156	0.04097	0.16936	0.13674	-0.16175	0.10641	0.08925	0.37328	-0.258	0.09392
-0.06971	-0.01162	-0.14614	0.02329	0.04543	0.04018	0.12369	0.11998	0.06439	0.16821	0.03013	0.01651	0.28017	0.1683	-0.0027	-0.25849	0.12713	0.1943	0.06368	0.14576	0.08566	0.13533	0.40542	-0.0471	0.15161
-0.09225	-0.06531	-0.17614	0.06573	0.10001	0.00524	0.08419	0.07881	0.04801	0.15579	0.00124	0.00621	0.27705	0.18853	0.01646	-0.18249	0.10889	0.19465	0.21581	0.08221	0.10077	0.15756	0.39445	-0.07952	0.13063
-0.09778	0.01461	-0.09545	0.03545	0.04992	0.01463	0.0787	0.11477	0.02306	0.17124	0.00154	0.04878	0.28745	0.12446	0.02168	-0.321	0.14357	0.20801	0.06374	0.10172	0.1177	0.16485	0.39663	-0.07003	0.17886
-0.05101	0.09752	-0.23651	0.09281	0.09912	0.06227	0.20345	0.19209	0.18258	0.12158	0.04292	0.0074	0.27554	0.19587	-0.08482	-0.24163	0.09281	0.16691	0.02902	-0.04812	0.04547	0.16583	0.4083	-0.28229	0.1235
-0.10217	0.04274	-0.04373	0.09407	0.10916	0.00968	0.10825	0.11903	0.07285	0.14669	0.02745	0.03666	0.27215	0.1907	0.00154	-0.12244	0.06624	0.17841	-0.01064	0.00801	0.08063	0.11778	0.4157	-0.22149	0.09808
-0.14755	0.04245	-0.07744	0.05344	0.05603	-0.01172	0.1115	0.11511	0.05095	0.16395	0.01327	-0.02369	0.2748	0.1409	0.00352	-0.37483	0.14395	0.19918	-0.17348	0.18175	0.12325	0.19609	0.41346	0.02979	0.12957
-0.03412	0.03035	-0.09468	0.02253	0.02288	0.00733	0.18545	0.15971	0.11994	0.10417	0.01011	0.04964	0.2726	0.18	-0.10311	-0.27619	0.07172	0.16484	-0.06839	0.14245	-0.00728	0.07811	0.39621	-0.19333	0.0966
-0.00482	0.05975	-0.04864	0.07812	0.14644	0.04977	0.22766	0.24699	0.22947	0.13149	0.00407	0.04735	0.27656	0.22409	-0.08543	-0.23664	0.07787	0.15762	-0.03392	-0.07028	0.03811	0.14368	0.37962	-0.20167	0.11972

G_IN_EM	TIVATION	CINOID_	CYLE_AND	NLRP3_	OTOTRAN	CYCLE_IN	G2_M_PR	_1_REPL	OUGH_PR	SIGNALLI	NDENT_A	DIATED_	ENDENT_	E_TIE2_S	JUNCTIO	1_DEPEN	NF_KA	CED_PRO	IATED_C	N_CANON	MEMBER	E_TNF_S	RECEP	LIKE_RE
0.13528	-0.12696	-0.09039	0.20316	0.11582	0.10358	0.15959	0.28027	0.12348	0.17943	0.14754	0.075	0.16989	0.05837	0.02593	0.07858	-0.14852	-0.09536	-0.18924	0.46556	0.2623	-0.34906	-0.00934	0.03179	0.07615
0.16082	-0.15034	-0.08234	0.18776	0.11271	0.10755	-0.08438	0.29276	0.09139	0.18263	0.15671	-0.01955	0.147	-0.00548	0.03784	0.10427	-0.21918	-0.0494	-0.17115	0.46644	0.2773	-0.18666	-0.02439	0.01395	0.04686
0.12759	-0.14791	-0.04947	0.19701	0.12356	0.07799	-0.15325	0.27591	0.14242	0.17542	0.13661	-0.03555	0.09612	0.03459	0.04814	0.02586	-0.17652	-0.11056	-0.21499	0.45605	0.25244	-0.23652	-0.03839	0.03082	0.07448
0.12801	-0.02598	0.09844	0.19123	0.15091	0.06063	0.06077	0.2857	0.18643	0.2929	0.26562	-0.01686	0.13504	0.03218	0.06054	-0.00153	-0.08976	-0.09634	-0.21393	0.46274	0.2569	-0.20449	-0.00711	0.02986	0.09487
0.13234	-0.06991	-0.00459	0.16194	0.16301	0.0756	-0.09512	0.30573	0.19078	0.23887	0.18877	-0.01261	0.13993	0.03313	0.08254	-0.0068	-0.08805	-0.05055	-0.12998	0.45658	0.28424	-0.243	-0.01638	0.04801	0.10302
0.1307	-0.06999	0.08396	0.16848	0.10342	0.09579	-0.21913	0.28348	0.15582	0.13606	0.13148	-0.04114	0.06197	-0.04818	-0.01475	-0.02142	-0.19027	-0.11144	-0.19337	0.45129	0.27036	-0.35606	-0.05013	0.01209	0.05575
0.12682	-0.14305	0.04262	0.19853	0.16719	0.1485	-0.02625	0.27436	0.1706	0.2029	0.16926	-0.03633	0.09113	0.02318	0.02623	0.06939	-0.21589	-0.10546	-0.20418	0.46081	0.25907	-0.15834	-0.0398	0.03386	0.07509
0.11107	-0.006	-0.01554	0.18282	0.18695	0.09082	-0.15959	0.29934	0.21396	0.27366	0.21955	0.04859	0.16087	0.05716	0.05364	-0.14508	-0.12213	-0.06724	-0.18057	0.4557	0.27514	-0.17186	-0.01313	0.05491	0.11461
0.12892	0.06916	0.09237	0.1728	0.15365	0.07937	-0.24538	0.30426	0.21838	0.25139	0.19236	0.01107	0.14095	0.05026	0.05326	-0.1596	-0.09711	-0.06302	-0.15462	0.45129	0.28312	-0.149	-0.0174	0.04233	0.11468
0.08281	-0.01258	-0.00949	0.19553	0.19956	0.05964	-0.29945	0.29142	0.20802	0.26872	0.22583	0.01651	0.14608	0.04822	0.02318	-0.10354	-0.14258	-0.07085	-0.17222	0.449	0.27186	-0.10205	-0.01275	0.06458	0.1132
0.13961	-0.15452	0.02892	0.20579	0.09481	0.04898	-0.06852	0.29097	0.08373	0.15823	0.14626	0.02323	0.09797	0.06204	-0.01902	0.10029	-0.24257	-0.12626	-0.24326	0.45517	0.26046	-0.34613	-0.048	0.02225	0.06319
0.11889	-0.10201	-0.02754	0.19969	0.09611	0.05417	-0.37502	0.29031	0.12994	0.20963	0.19815	-0.03555	0.11763	0.0635	-0.06527	0.03666	-0.21887	-0.11742	-0.22377	0.4638	0.26592	-0.32099	-0.06416	0.03163	0.06604
0.17928	-0.02118	-0.08658	0.13547	0.05992	0.12422	0.23128	0.30685	0.10327	0.2075	0.19257	0.02279	0.08976	0.04264	0.0824	0.20573	-0.13099	-0.09371	-0.15813	0.46063	0.27683	-0.07971	-0.0237	0.02971	0.05176
0.13108	-0.02971	0.07765	0.18413	0.13703	0.05377	0.05091	0.28812	0.14905	0.20896	0.15782	-0.0236	0.14222	0.03545	0.02518	0.14639	-0.20686	-0.09014	-0.23172	0.4527	0.26302	-0.22621	-0.01371	0.05074	0.0912
0.13797	-0.09986	0.07965	0.17049	0.11726	0.04753	-0.3877	0.28436	0.1239	0.20161	0.1575	0.04956	0.12923	0.06053	-0.03684	0.01387	-0.18583	-0.062	-0.15795	0.46592	0.26768	-0.26622	-0.00413	0.04476	0.09145
0.17275	-0.10721	-0.09204	0.20931	0.07688	0.08257	0.00018	0.28442	0.07044	0.1404	0.10804	0.03621	0.10154	0.11329	-0.04267	0.18077	-0.21151	-0.10234	-0.1963	0.46116	0.25546	-0.14093	-0.03436	0.0168	0.02114
0.12181	-0.13792	0.01205	0.21503	0.15559	0.05346	-0.18989	0.28762	0.16218	0.27311	0.26571	-0.01535	0.10563	-0.00418	0.07634	0.1074	-0.19288	-0.13113	-0.24987	0.45711	0.25179	-0.27503	-0.03995	0.02382	0.07954
0.14451	-0.07855	0.06689	0.20969	0.15268	0.05628	-0.17351	0.30944	0.21105	0.24983	0.19714	0.02234	0.14206	0.03594	0.06613	-0.08599	-0.13145	-0.07272	-0.17683	0.452	0.27634	-0.18076	-0.01151	0.03366	0.09736

PTION_O	RANSCRI	RANSCRI	IPTION_(	RANSCRI	_OF_GEN	N_OF_GEN	N_OF_GE	ATH_GEN	NDENT_I	ON_OF_TA	MEDIATED	_ACTIVA	EDIATED_	D_PROCE	KING_OF	GLUR2_C	RISTOYL	_TRAIL_	IVATION_	TY_OF_S	PTIONAL_	TIONAL_R	TIONAL_R	TIONAL_R
-0.21942	-0.01435	-0.03166	-0.14409	-0.06609	-0.10352	-0.33929	0.07785	0.17593	0.004	0.14083	-0.01268	0.28715	-0.03626	0.20251	0.17721	0.19122	0.03598	-0.22155	0.07733	0.09923	-0.08282	0.08483	0.12526	0.24647
-0.2577	0.04266	-0.11215	-0.23173	0.01779	-0.08273	-0.24951	0.13471	0.01038	0.0236	0.09986	-0.07874	0.24334	-0.01964	0.23261	0.19628	0.19282	0.05795	-0.22293	0.07994	0.12373	0.03076	0.09594	0.16202	0.26964
-0.1979	0.06636	-0.06509	-0.25237	-0.04089	-0.01613	-0.2825	0.18505	0.01945	-0.01585	0.1343	-0.10162	0.26031	-0.05169	0.24112	0.19809	0.24571	0.08694	-0.13942	0.08245	0.11084	-0.0812	0.07379	0.11788	0.24179
-0.13692	0.01843	-0.03087	-0.18839	-0.09488	-0.01449	-0.31138	0.13776	0.05251	-0.00521	0.13788	-0.08983	0.24905	-0.0646	0.31432	0.19088	0.29019	0.16568	-0.12145	0.02935	0.10662	-0.08527	0.0748	0.0798	0.22934
-0.14421	0.00194	-0.05329	-0.34498	-0.05159	-0.0369	-0.27342	0.11862	-0.01246	0.00698	0.12732	-0.05674	0.2926	-0.00076	0.26237	0.1507	0.27296	0.0623	-0.09097	0.07796	0.11333	-0.07462	0.07983	0.07725	0.25827
-0.16373	0.04308	-0.05438	-0.12927	-0.02505	-0.02723	-0.20504	0.1387	0.01571	-0.0121	0.03983	-0.13863	0.24559	-0.0641	0.17928	0.15948	0.23107	0.02095	-0.13141	0.06573	0.08759	-0.05003	0.07789	0.07497	0.24877
-0.12547	0.04589	-0.04685	-0.21935	-0.04036	-0.00068	-0.18377	0.13971	-0.06559	-0.00401	0.12397	-0.07015	0.26832	0.03021	0.21122	0.1789	0.22864	0.07464	-0.16803	0.07329	0.12251	-0.08186	0.05994	0.07021	0.23716
-0.12065	0.00088	-0.02062	-0.2482	-0.12147	0.03159	-0.30776	0.12263	0.00968	0.03712	0.15622	-0.03796	0.26901	-0.00486	0.33731	0.18702	0.31326	0.12084	-0.20997	0.04534	0.12136	-0.12077	0.06563	0.07823	0.23668
-0.09728	-0.04026	-0.01828	-0.21914	-0.10493	0.01607	-0.33492	0.07653	0.01472	0.01082	0.14933	-0.06085	0.27399	-0.04241	0.34428	0.20666	0.29725	0.15583	-0.14705	0.04079	0.12112	-0.09917	0.05823	0.05928	0.24384
-0.06906	-0.00409	0.00634	-0.1233	-0.072	0.0823	-0.23183	0.07643	0.05021	0.03249	0.14894	-0.02992	0.28267	0.02606	0.30216	0.15396	0.28494	0.06781	-0.12268	0.05505	0.12441	-0.07393	0.07609	0.07276	0.23825
-0.2038	0.10039	-0.04561	-0.13889	-0.05875	-0.08043	-0.13142	0.18687	0.0887	-0.0023	0.11526	-0.054	0.24512	0.01024	0.13701	0.13273	0.18098	0.16087	-0.23044	0.08506	0.09753	-0.04354	0.08072	0.13688	0.24023
-0.22664	0.04306	-0.04091	-0.06887	-0.08561	-0.06512	-0.32106	0.20526	0.1094	0.02665	0.1535	-0.03782	0.28392	-0.00712	0.19004	0.19275	0.21176	0.05979	-0.23007	0.09429	0.09096	-0.09167	0.09557	0.16688	0.24527
-0.22565	0.08916	-0.10619	-0.16455	-0.0057	-0.05893	-0.16022	0.21763	0.02906	0.02455	0.03118	-0.03688	0.2619	-0.04993	0.15807	0.16145	0.20826	0.13663	-0.0643	0.04616	0.1021	-0.02165	0.04667	0.14071	0.25518
-0.18845	0.01894	-0.0753	-0.23227	-0.0683	-0.06769	-0.25733	0.09042	0.03164	-0.0103	0.13112	-0.08627	0.26733	0.01337	0.24372	0.17611	0.25348	0.19752	-0.20332	0.05388	0.10424	-0.13937	0.09642	0.11829	0.24135
-0.1703	0.01492	-0.03484	-0.21402	-0.06025	-0.03204	-0.24327	0.14249	0.09391	0.04043	0.12965	-0.01825	0.27305	0.00238	0.19593	0.18509	0.19461	0.11795	-0.20162	0.05923	0.10966	-0.04319	0.06115	0.11457	0.24562
-0.2615	0.06152	-0.06561	-0.13735	-0.02772	-0.02528	-0.31091	0.19922	0.08084	0.04751	0.14182	0.0182	0.29192	-0.01376	0.08639	0.15402	0.15908	0.02361	-0.18351	0.08283	0.09555	-0.05501	0.02457	0.15272	0.2408
-0.20256	0.02066	-0.05148	-0.17959	-0.06867	-0.01343	-0.36149	0.12526	0.05705	-0.01174	0.11176	-0.09238	0.24234	-0.02257	0.26415	0.20092	0.27077	0.13401	-0.19594	0.06398	0.10008	-0.0917	0.07983	0.11656	0.24615
-0.13907	0.00522	-0.03417	-0.3326	-0.08902	-0.00292	-0.31657	0.09394	-0.01158	0.0371	0.13872	-0.03752	0.27099	-0.01166	0.30355	0.15692	0.29949	0.07657	-0.20309	0.0453	0.10994	-0.12742	0.07343	0.0926	0.24438

TIONAL_R	TIONAL_R	INAL_REG	_THE_AP	PTIONAL_	TIONAL_	AL_REGU	GULATIO	EGULATI	ATION_OF	ED_NUCL	S_UNDEF	E_CONTR	IPTION_O	N_ENDOC	ME_TRAN	SEMBLY_	SARS_CC	SARS_CC	ESION_S	ESION_S	DNA_PO	LC2A4_	N_ACRO	RT_AND_
0.21889	0.20841	0.24343	0.20114	0.05859	-0.07815	0.38793	-0.09889	0.04281	-0.09281	-0.00211	0.13418	0.28597	-0.00547	0.22788	0.22477	0.07909	0.13995	0.14223	0.10606	0.02914	0.06212	0.15792	0.13502	0.05386
0.22947	0.21663	0.25204	0.23673	0.07956	-0.04829	0.39246	0.11307	0.21583	-0.11942	0.04889	0.25905	0.33691	0.08076	0.19061	0.21927	-0.02475	0.13306	0.13953	0.18644	0.13396	0.13615	0.14506	0.15387	-0.03313
0.21815	0.20298	0.26017	0.2516	0.06951	-0.08088	0.39421	-0.05483	-0.03144	-0.10122	0.01168	0.22449	0.33253	0.02879	0.26696	0.21641	-0.05754	0.15141	0.15811	0.14779	0.09823	0.13699	0.13518	0.16058	-0.06315
0.22636	0.19483	0.22308	0.20036	0.05049	-0.08951	0.38514	-0.05758	-0.07544	-0.125	-0.00985	0.17812	0.26534	-0.02074	0.2911	0.21102	0.03634	0.17705	0.1711	0.13527	0.08368	0.11797	0.18024	0.16973	0.08088
0.24615	0.23032	0.23578	0.21888	0.05436	-0.12523	0.39903	-0.00298	0.09496	-0.12752	0.01724	0.16624	0.27343	0.01505	0.21191	0.21178	0.02048	0.14467	0.14691	0.11529	0.07116	0.0922	0.14996	0.16039	-0.01519
0.2215	0.21501	0.23885	0.23688	0.07341	-0.09279	0.39011	-0.09982	-0.0745	-0.14503	0.03436	0.23491	0.28453	0.03441	0.12897	0.2121	-0.06509	0.15967	0.18332	0.1398	0.0864	0.15196	0.1475	0.12029	-0.15524
0.22546	0.1922	0.2445	0.24616	0.05852	-0.12105	0.39097	-0.06359	0.0625	-0.10261	0.01821	0.16357	0.31729	0.01707	0.18619	0.22185	-0.11357	0.17033	0.15623	0.12659	0.06489	0.13853	0.12952	0.14041	-0.08337
0.23428	0.21419	0.20295	0.1677	0.0404	-0.11001	0.38835	-0.0817	-0.25684	-0.14267	-0.03646	0.1278	0.15783	-0.05256	0.28456	0.19561	0.02971	0.17888	0.17757	0.09148	0.02719	0.08934	0.179	0.17636	0.10713
0.23706	0.21786	0.21312	0.1833	0.03916	-0.13045	0.39054	-0.07748	-0.10798	-0.14866	-0.04319	0.13784	0.21178	-0.03305	0.28494	0.19891	0.01112	0.16714	0.1614	0.07256	0.00428	0.09844	0.17571	0.16898	0.07539
0.22587	0.20678	0.22395	0.20303	0.05638	-0.1434	0.39575	-0.12036	-0.30836	-0.13646	0.0052	0.15353	0.19268	-0.00698	0.25529	0.19881	0.01943	0.17408	0.16144	0.09844	0.04754	0.1275	0.16872	0.16175	0.0515
0.22631	0.1958	0.23435	0.2547	0.06871	-0.05378	0.39747	0.04575	-0.07159	-0.11323	0.02419	0.2743	0.33166	0.01047	0.21958	0.2224	-0.01757	0.17507	0.18032	0.11696	0.0854	0.10442	0.1828	0.13082	-0.06786
0.23333	0.20114	0.24753	0.24642	0.06389	-0.1091	0.39474	-0.09596	-0.24884	-0.14482	-0.00064	0.20011	0.29211	-0.02095	0.17081	0.24918	-0.00215	0.1776	0.16231	0.16208	0.11255	0.13249	0.17262	0.14007	-0.09643
0.23494	0.2102	0.25649	0.25106	0.07845	-0.06578	0.38378	0.02105	0.04454	-0.08438	0.03593	0.23541	0.37138	0.04266	0.22785	0.21471	0.08036	0.19873	0.2015	0.17045	0.13917	0.13972	0.19651	0.16504	-0.25922
0.21922	0.19656	0.24831	0.20343	0.05049	-0.09116	0.39397	-0.04817	-0.31952	-0.11659	-0.01929	0.1509	0.21367	0.00024	0.25457	0.20997	-0.01271	0.14678	0.15578	0.10367	0.02268	0.09036	0.16989	0.15712	0.08382
0.21569	0.19866	0.22415	0.21512	0.06339	-0.09782	0.39153	-0.05435	0.04715	-0.14403	-0.00441	0.19867	0.25548	0.01777	0.21704	0.2118	0.03274	0.16945	0.16016	0.13721	0.07387	0.1175	0.16753	0.15247	-0.01426
0.22698	0.18816	0.24026	0.26383	0.07473	-0.10159	0.38442	-0.03083	0.00423	-0.11442	0.02291	0.20238	0.36894	0.02061	0.1883	0.23495	0.03468	0.1805	0.17278	0.12258	0.07119	0.14579	0.18516	0.13156	-0.18564
0.21182	0.20042	0.23656	0.2175	0.06195	-0.07872	0.39261	-0.01502	0.03566	-0.10056	-0.03541	0.12915	0.25531	-0.00773	0.26294	0.23742	0.03561	0.15494	0.15587	0.07574	-0.00651	0.04237	0.17752	0.1704	-0.01624
0.23886	0.21368	0.22325	0.20699	0.04818	-0.11044	0.38937	-0.09635	-0.17229	-0.16547	-0.02859	0.15699	0.21229	-0.01982	0.27116	0.20884	0.00496	0.17469	0.16056	0.07147	0.01265	0.06903	0.18411	0.17172	0.0748

ORGANIC	ONNEXON	NSPORT_	ATIONS_A	NAS_DER	MATURE_	URINE_A	RT_OF_N	PORT_OF	T_OF_RC	ORT_OF_	THE_SL	BPS_NUCLE	GOLGI_A	GI_NETW	TED_PRO	LYCERID	LYCERI	LYCERID	_ZFP36_	RNA_AMI	NCATION_	ION_IN_T	TRNA_P	RESSING_
0.10245	0.19079	-0.08813	-0.02852	0.05668	0.10913	0.1923	0.03714	-0.44478	-0.36997	0.15573	0.06227	0.0364	0.14426	0.16557	-0.01168	-0.00318	0.23189	0.1996	-0.12394	0.18074	0.00711	-0.06873	0.0116	0.00494
0.03796	0.23174	-0.0331	0.02852	0.08606	0.14922	0.15491	-0.05663	-0.45129	-0.27284	0.14426	0.08616	0.00115	0.13216	0.14355	-0.04642	-0.12135	0.19714	0.15626	-0.05124	0.17801	0.07228	0.03424	0.06498	0.13203
0.03247	0.23065	0.03709	0.06321	0.08701	0.14598	0.10127	-0.21413	-0.47674	-0.32657	0.15583	0.10375	-0.0127	0.1521	0.13691	-0.01456	-0.10445	0.19546	0.15647	-0.14151	0.16473	0.02139	-0.018	0.04946	0.00383
0.10924	0.26577	0.13606	0.00253	0.0197	0.09644	0.12052	-0.1076	-0.34618	-0.23004	0.17635	0.02675	0.03976	0.15749	0.17552	0.04859	-0.05022	0.20509	0.17015	-0.13825	0.13511	0.00399	-0.08434	-0.01845	-0.01324
0.04632	0.23624	0.03582	-0.0486	0.04814	0.10997	0.08672	0.02286	-0.45701	-0.37907	0.16434	0.05218	-0.00107	0.14424	0.15311	0.03597	-0.1047	0.19384	0.15446	-0.12146	0.13688	0.00433	-0.03775	0.01074	0.0537
0.12866	0.20271	-0.03625	-0.00182	0.07116	0.12731	0.17153	-0.12769	-0.39878	-0.28558	0.1533	0.06776	-0.01683	0.14013	0.1445	-0.06605	-0.32426	0.21342	0.16083	-0.1409	0.16118	0.0573	-0.00232	0.03232	0.08755
-0.00625	0.22459	0.05801	0.06719	0.0476	0.10893	0.14791	-0.07792	-0.446	-0.2832	0.14854	0.05955	0.00795	0.12738	0.11305	-0.03797	-0.24833	0.18052	0.12833	-0.05818	0.1607	0.08416	-0.0435	0.01989	0.09426
0.12519	0.26682	0.18178	-0.00715	-0.0101	0.0606	0.12143	-0.05098	-0.28984	-0.35248	0.18919	-0.00089	0.0533	0.17774	0.18896	0.02505	0.01009	0.22667	0.19746	-0.13844	0.12716	-0.05873	-0.10183	-0.04863	-0.03038
0.09673	0.2714	0.17821	-0.00851	0.00576	0.0722	0.07522	-0.1703	-0.36002	-0.336	0.17906	0.01226	0.01002	0.16627	0.18643	0.03949	-0.03818	0.24015	0.20498	-0.11184	0.1232	-0.02926	-0.07956	-0.03517	-0.01563
0.08404	0.25743	0.07461	-0.00947	0.02258	0.09452	0.05417	-0.1301	-0.38908	-0.34723	0.16823	0.02941	-0.02603	0.14442	0.16307	0.01093	-0.06807	0.20975	0.17381	-0.11426	0.12921	0.0135	-0.07527	-0.02204	0.01165
0.02426	0.29346	-0.1435	-0.01874	0.05979	0.12471	0.16653	0.10983	-0.44321	-0.26546	0.14356	0.05427	0.02731	0.14833	0.11422	-0.09294	-0.24001	0.21071	0.15652	-0.16522	0.20581	0.00882	-0.03583	0.01901	0.08417
0.06358	0.23785	-0.03201	-0.0161	0.06031	0.11663	0.15906	-0.05567	-0.47445	-0.43321	0.15763	0.07664	0.0159	0.14823	0.13029	-0.0467	-0.09797	0.18856	0.15233	-0.1378	0.20826	0.03082	-0.07838	0.01452	0.08751
0.05056	0.32029	-0.12369	0.00567	0.07601	0.13931	0.18628	-0.09114	-0.46802	-0.34864	0.14906	0.08916	0.00135	0.15292	0.12294	-0.10426	-0.22638	0.16702	0.12457	-0.13571	0.17132	-0.02278	-0.05809	0.0358	0.04514
0.07649	0.2797	0.06747	-0.02095	0.07615	0.12141	0.15791	0.0564	-0.48679	-0.25867	0.16546	0.0876	0.06804	0.16385	0.14441	-0.03491	-0.10778	0.21881	0.17609	-0.12636	0.166	0.02052	-0.09835	0.02319	0.05495
0.03279	0.24391	-0.00681	-0.02148	0.03682	0.09867	0.18667	-0.04531	-0.47789	-0.34319	0.15511	0.04556	0.04037	0.15245	0.1414	-0.04713	-0.1325	0.21176	0.16839	-0.07637	0.18517	-0.00193	-0.04399	0.01082	0.04708
0.00173	0.2656	-0.10913	0.04934	0.0679	0.12634	0.21192	-0.00379	-0.18093	-0.25611	0.13367	0.06736	0.03892	0.12829	0.10153	-0.09507	-0.06533	0.17625	0.14329	-0.09583	0.22693	0.08978	-0.01585	0.05411	0.16006
0.03081	0.28887	0.0419	-0.03743	0.05593	0.12051	0.17551	-0.15089	-0.47648	-0.21875	0.16502	0.0573	0.0326	0.15467	0.17108	-0.02321	-0.10026	0.23498	0.18889	-0.14405	0.16375	0.05087	-0.04782	0.01028	0.05464
0.05302	0.30151	0.13845	0.03129	0.02131	0.08638	0.1485	-0.07153	-0.36473	-0.31502	0.17619	0.0325	0.05182	0.15259	0.16669	0.0174	-0.06884	0.20354	0.16619	-0.13013	0.15458	-0.00333	-0.10808	-0.02329	0.06308

OCESSIN	E_TRP_C	YPTOPHA	HEMIDES	YROSINE	EAVES_P	EIQUINOL	IFIC_PRO	UCH_PR	CEPTOR	SED_PROT	UNWINDI	_ACTION	_FUNCTI	FUNCTIO	ARY_COB	ME_UREA	RENAL_	EDIATED_	DIATED_V	ILE_MEDI	AL_INFEC	ESSENGI	AL_PHOT	IN_B1_THI
0.04228	-0.23754	-0.05766	0.07425	0.01696	0.1447	-0.04986	0.17524	0.2713	0.15559	0.13748	0.19205	0.28052	0.14053	0.42839	-0.11087	0.00117	0.18268	0.1037	0.16407	0.10569	0.22123	0.03279	0.03268	-0.16815
0.0769	-0.36016	0.00724	0.04838	-0.1027	0.21371	-0.04245	0.17887	0.28524	0.1784	0.16043	-0.05759	0.26633	0.10942	0.43185	-0.06585	-0.13566	0.19569	0.10819	0.1152	0.08335	0.23063	0.05077	0.02196	-0.32375
0.07855	-0.30061	0.0865	0.04094	-0.02272	0.17311	0.02462	0.16608	0.25572	0.12294	0.15102	0.26489	0.27367	0.13312	0.42183	-0.38319	-0.14119	0.13585	0.07003	0.15363	0.1022	0.22549	0.07375	0.01039	-0.18139
0.00614	-0.19445	0.03331	-0.04313	0.13144	0.24022	-0.09384	0.17017	0.25587	0.08133	0.14114	0.19172	0.25145	0.10393	0.39072	-0.24993	-0.09607	0.25208	0.10821	0.13906	0.12821	0.22834	-0.0069	0.08882	-0.08992
0.03123	-0.25456	0.06833	-0.13835	0.00912	0.20595	-0.07603	0.19162	0.29492	-0.00509	0.14925	0.14866	0.28528	0.17607	0.39772	-0.34127	0.00288	0.21573	0.11317	0.12419	0.11137	0.22935	0.02176	0.0535	-0.05628
0.04392	-0.38894	-0.02317	-0.01346	-0.02364	0.21814	-0.08456	0.16933	0.26831	0.13206	0.13923	0.24174	0.26719	0.1447	0.40264	-0.37016	0.03251	0.16455	0.0948	0.13467	0.09819	0.22446	0.02492	0.0945	-0.17626
0.04198	-0.39911	0.09013	-0.03797	-0.18365	0.19328	-0.04811	0.17205	0.26377	0.0468	0.13766	0.14805	0.26448	0.14463	0.41053	-0.11364	-0.1296	0.18468	0.09429	0.1383	0.08387	0.22309	0.03597	0.09975	-0.12456
-0.02751	-0.17966	0.07837	-0.01656	0.07716	0.22857	-0.1077	0.18341	0.26584	0.07513	0.15266	-0.02033	0.24586	0.13915	0.35032	-0.36296	-0.08378	0.24269	0.06689	0.09378	0.14221	0.22582	-0.04441	0.06451	0.07867
-0.01866	-0.14665	0.09025	-0.02254	-0.05504	0.21387	-0.10853	0.18133	0.28735	0.04568	0.14898	0.05416	0.2508	0.13184	0.37338	-0.30622	-0.21024	0.21659	0.07411	0.14775	0.13412	0.22625	-0.0219	0.09853	-0.01343
-0.00223	-0.20169	0.09071	-0.05663	0.00294	0.17576	-0.07165	0.17572	0.27538	0.04137	0.14467	-0.05129	0.2698	0.15575	0.39057	-0.28566	-0.13109	0.243	0.09603	0.11404	0.11999	0.2254	-0.00718	0.06167	0.05779
0.03862	-0.37898	-0.03919	0.06936	-0.23305	0.20542	-0.14393	0.1772	0.26361	0.01526	0.15659	0.33987	0.23403	0.07956	0.40415	-0.4028	0.05413	0.15832	0.02837	0.11428	0.09358	0.22473	0.009	0.02592	-0.40266
0.04822	-0.28135	-0.02857	0.08309	-0.08992	0.21084	-0.08108	0.17362	0.27021	0.10617	0.1365	0.25075	0.24524	0.13757	0.3696	-0.22956	-0.18336	0.19401	0.06333	0.10769	0.10288	0.22619	0.03249	0.02597	-0.094
0.0749	-0.28416	-0.00716	0.04816	-0.10735	0.19692	-0.1421	0.18051	0.26629	0.08212	0.14752	0.24085	0.28305	0.12424	0.44055	-0.27551	-0.01089	0.23329	0.07742	0.1343	0.10706	0.23496	0.05674	0.07577	-0.3897
0.06783	-0.27816	0.05966	-0.08596	0.04692	0.26287	-0.20998	0.1786	0.27285	0.09295	0.16209	-0.09752	0.26777	0.12871	0.40877	-0.2949	-0.12586	0.1929	0.08936	0.14528	0.11031	0.22738	0.03753	0.05052	-0.0414
0.03396	-0.34151	-0.01981	-0.0621	0.13158	0.1672	-0.19346	0.1694	0.26826	0.1155	0.17023	0.21685	0.2674	0.13998	0.4105	-0.28921	-0.06806	0.17935	0.05059	0.13597	0.10689	0.22599	-0.00185	0.04127	-0.3155
0.07904	-0.27181	0.04312	0.05213	0.10535	0.24081	-0.07482	0.17925	0.26488	0.13409	0.12646	0.30647	0.26624	0.12402	0.43282	-0.34067	-0.02947	0.12632	0.03038	0.15312	0.07893	0.22808	0.04008	0.02135	-0.30254
0.03014	-0.28713	-0.07404	0.05902	-0.01315	0.20274	-0.05493	0.16766	0.26228	0.07037	0.12698	-0.2034	0.25294	0.09883	0.4123	-0.3264	-0.16978	0.25196	0.10128	0.12612	0.11571	0.22608	0.0243	0.04091	-0.16821
0.00769	-0.19242	0.07516	0.06647	0.01999	0.22405	-0.0527	0.17384	0.27789	0.02043	0.14348	0.06866	0.26851	0.13309	0.40038	-0.27651	-0.14602	0.22921	0.07439	0.1466	0.12066	0.22566	-0.00232	0.03892	0.055

B2_RIBO	S_PANTO	C_ASCO	D_CALCI	RNALISA	TE_VLDL_	_VLDL_C	GATED_P	ARGO_TA	PLASMA	TERNAL	DENT_IN	BIOGEN	DIATED_	ME_XENO	TAZ_ST	NATED_IN	PARTME	O_CELLS	ZINC_TRA	C_GENO	RNAL_	RY_NUMB	OPY_NU	UMBER_V
0.0997	-0.04411	0.33965	0.20477	0.30656	0.33318	0.21835	-0.21772	0.04146	-0.04404	0.29801	0.21113	0.1561	0.00145	-0.22519	-0.22758	-0.03697	0.18284	0.09295	0.11738	-0.17778	0.30609	0.06653	0.11579	0.07715
0.06676	-0.04763	0.28615	0.23185	0.31531	0.27741	0.1342	-0.20539	-0.03921	0.07856	0.28645	0.20146	0.14472	0.00644	-0.20183	-0.16983	-0.05191	-0.14215	0.09294	0.03639	-0.21508	0.34342	0.11396	0.06535	0.02935
0.09424	-0.00417	0.31002	0.27384	0.35655	0.30969	0.20202	-0.16928	-0.06273	0.12841	0.33334	0.25482	0.15298	0.05988	-0.23063	-0.07615	-0.06177	-0.0347	0.00993	-0.00196	-0.11324	0.30765	0.09976	0.13582	0.0875
-0.04051	-0.06139	0.30583	0.32483	0.37665	0.36146	0.24505	0.04404	0.03196	0.15713	0.36492	0.29983	0.17713	-0.01234	-0.1566	-0.24552	-0.04395	0.13123	0.10503	0.11251	-0.18505	0.29939	0.05053	0.053	0.12232
0.03911	-0.05019	0.33968	0.24177	0.35701	0.37518	0.2081	0.07627	-0.00678	0.07768	0.33827	0.28574	0.1523	-0.00248	-0.31196	-0.10035	-0.03005	-0.10023	0.11113	0.05849	0.03273	0.29538	0.03017	0.07174	0.0768
0.065	0.00065	0.28404	0.31163	0.32739	0.42762	0.27923	-0.06923	-0.03928	-0.01497	0.30752	0.23488	0.16384	0.02551	-0.24914	-0.08999	-0.06782	0.03382	0.17932	0.14388	-0.08308	0.29643	0.05039	0.07704	0.0561
0.15237	0.02478	0.29164	0.32363	0.32827	0.37425	0.20823	0.0377	-0.05598	0.02801	0.29977	0.23861	0.12431	0.02169	-0.19881	-0.24017	-0.05861	-0.13387	-0.36913	-0.29763	-0.24286	0.30364	0.06582	0.11076	0.11557
-0.07557	-0.02676	0.35862	0.35567	0.39537	0.39477	0.20902	0.01814	0.0373	0.18989	0.37735	0.32038	0.19735	-0.0212	-0.25725	-0.2695	0.00342	0.12049	0.06263	0.07834	-0.21202	0.26609	0.01893	0.03157	0.11088
-0.04915	-0.02755	0.36422	0.38536	0.38438	0.40445	0.22078	0.07786	0.04447	0.17368	0.36487	0.3112	0.18334	-0.0033	-0.25356	-0.28887	-0.0221	0.11661	0.04702	0.06705	-0.26711	0.28181	0.02484	-0.00126	0.10069
-0.01022	-0.04177	0.28098	0.30318	0.36333	0.38656	0.22715	0.13352	-0.01198	0.10234	0.34165	0.2981	0.16436	0.02094	-0.33162	-0.32848	-0.01638	0.0414	0.05246	0.04941	-0.29767	0.27062	0.0314	0.01622	0.0742
0.16646	-0.00916	0.34368	0.26358	0.2952	0.34119	0.18749	-0.33486	0.01716	0.07751	0.27389	0.18718	0.14872	0.11201	-0.07641	-0.04637	-0.09361	0.10904	-0.05937	-0.01121	-0.04847	0.31325	0.1176	0.11312	0.04239
0.22582	0.02937	0.28228	0.27398	0.32396	0.40485	0.27257	-0.22001	0.02516	0.21578	0.30146	0.22281	0.19502	0.02021	-0.16919	-0.14826	-0.09264	0.07715	0.03364	0.04548	-0.11618	0.29776	0.07523	0.07991	0.06727
0.38489	0.14688	0.29722	0.23746	0.33807	0.37327	0.20191	-0.42012	-0.00884	0.03505	0.32051	0.23351	0.19562	0.13067	-0.21337	0.01062	-0.11086	0.04034	0.07671	0.06735	0.01216	0.3069	0.14659	0.07185	0.017
-0.03118	0.02092	0.32735	0.27134	0.36494	0.37743	0.18893	0.02026	-0.00692	0.16062	0.35211	0.27759	0.1713	0.02197	-0.17073	-0.17638	-0.04512	0.00388	0.09849	0.07497	-0.15245	0.29113	0.09681	0.05319	0.09898
0.22036	0.00082	0.29981	0.25982	0.31463	0.36211	0.21931	-0.30756	-0.00309	0.01814	0.29525	0.21966	0.17001	0.02185	-0.3721	-0.13997	-0.04041	-0.19852	0.05095	-0.01316	-0.03816	0.31823	0.07854	0.0756	0.04905
0.2341	0.07982	0.26087	0.2081	0.29062	0.27508	0.11589	-0.38806	0.04023	0.05108	0.26562	0.17721	0.12057	0.06064	-0.14635	-0.01667	-0.09281	0.04087	-0.05911	-0.03238	0.07686	0.31882	0.09415	0.17823	0.0291
0.06007	-0.0493	0.3026	0.28298	0.37248	0.35044	0.25769	-0.00317	-0.02549	0.05496	0.3555	0.27885	0.16951	-0.00228	-0.17651	-0.28037	-0.08448	0.06747	-0.08793	-0.04335	-0.24301	0.30965	0.07969	0.06276	0.11299
0.05883	-0.05366	0.35903	0.3109	0.38907	0.36544	0.23179	0.08649	0.01297	0.15255	0.37079	0.30525	0.18162	0.00227	-0.10932	-0.14729	-0.01581	0.00528	0.08345	0.06214	-0.11433	0.28848	0.05166	0.05102	0.10794

OPY_NUM	OPY_NUM	UMBER_V	UMBER_V	PY_NUMB	AL_DELE	MAL_DELE	_COPY_N	MBER_VA	MBER_VA	UMBER_V	UMBER_V	MBER_V	MBER_VA	UMBER_V	MBER_VA	MBER_VA	PY_NUM	ND_RETI	Y_NUMB	Y_NUMB	UMBER_V	_COPY_N	ND_7_BE	IMBER_V
0.16729	0.14521	0.10307	0.452	0.11614	0.03134	0.18632	0.23894	0.01477	0.02097	0.01277	0.14853	0.04355	0.11578	0.26366	0.02587	0.06191	0.03374	0.114	0.07119	-0.2737	0.16144	0.29978	0.03302	0.18099
0.17652	0.17834	0.10878	0.43192	0.1341	0.04269	0.15791	0.26439	0.03025	0.05568	0.10471	0.15372	0.03916	0.03831	0.2302	0.0195	0.03453	0.0734	0.0781	0.07626	-0.3304	0.16495	0.28481	0.00177	0.16987
0.1393	0.11571	0.07068	0.47596	0.1405	0.00069	0.16119	0.2061	-0.0307	0.02013	0.00361	0.16356	0.08031	0.10257	0.24312	0.02811	0.06366	-0.00108	0.09755	0.08156	-0.4267	0.17543	0.29395	-0.01409	0.17556
0.15908	0.08045	0.10926	0.39299	0.10104	0.07546	0.1761	0.26938	-0.02901	0.03246	-0.03977	0.14355	0.03909	0.06484	0.23582	0.05288	0.04334	0.0111	0.08524	0.09932	-0.2492	0.15424	0.32615	0.01738	0.20319
0.16625	0.07616	0.08577	0.43421	0.11262	0.05322	0.19025	0.2543	0.02846	0.02048	-0.09163	0.14051	0.02143	0.08742	0.27626	0.05198	0.01385	0.01107	0.11012	0.11564	-0.40718	0.13766	0.26475	0.0457	0.18546
0.17353	0.07515	0.02507	0.4409	0.12413	0.03609	0.13284	0.26871	0.03292	0.01001	-0.03048	0.13007	-0.01458	0.064	0.23864	0.05451	0.02411	-0.04416	0.05186	0.10779	-0.32875	0.14678	0.27653	0.021	0.10947
0.16611	0.11053	0.08296	0.42452	0.09215	0.02888	0.14386	0.20705	-0.01774	0.04862	-0.06456	0.15263	0.05022	0.12026	0.23985	0.0211	0.01693	0.0189	0.08953	0.09363	-0.25564	0.14978	0.29415	-0.0455	0.12811
0.1937	0.05465	0.05276	0.47877	0.13731	0.08919	0.16666	0.28538	-0.03223	0.00017	-0.09846	0.13704	0.05365	0.05938	0.28378	0.07633	0.0174	5.8E-05	0.08954	0.10258	-0.39619	0.12016	0.32043	0.0136	0.19806
0.17536	0.04193	0.03723	0.43773	0.1309	0.06106	0.16419	0.27937	0.0148	0.01089	-0.1092	0.11903	0.03829	0.09998	0.27505	0.09353	0.03031	-0.00974	0.08344	0.11154	-0.33715	0.1218	0.30193	0.01013	0.20513
0.15702	0.03017	0.08857	0.42082	0.11255	0.07133	0.16763	0.24774	-0.01205	0.01845	-0.10825	0.1408	0.0198	0.08213	0.2703	0.06765	0.0278	-0.01428	0.08326	0.09334	-0.41015	0.13253	0.28351	-0.01547	0.17771
0.10766	0.13711	0.11966	0.47613	0.11914	0.0155	0.17595	0.29124	0.03164	0.03939	-0.00526	0.16355	0.03391	0.08109	0.21995	0.0259	0.05491	0.06039	0.04788	0.07718	-0.44686	0.18104	0.29392	-0.02136	0.14667
0.16244	0.13613	0.10209	0.43209	0.03528	0.01848	0.18149	0.28486	0.00775	-0.0026	-0.00599	0.18121	0.01496	0.08756	0.25186	0.03947	0.04464	-0.02512	0.06593	0.06264	-0.42466	0.16133	0.30566	0.00709	0.12891
0.18121	0.1634	0.11273	0.47419	0.0928	0.04219	0.17386	0.27474	0.04599	0.06101	0.0834	0.14725	0.07174	0.10384	0.24688	0.03827	0.06286	0.04589	0.15072	0.03486	-0.31266	0.19966	0.318	-0.02314	0.19666
0.14004	0.12057	0.0852	0.46909	0.12108	0.05682	0.18887	0.26632	-0.0114	0.01482	-0.02561	0.15526	0.02942	0.0771	0.24418	0.03205	0.04746	0.02041	0.09776	0.07708	-0.46503	0.1462	0.31078	0.02065	0.16247
0.18082	0.10801	0.12344	0.47842	0.06286	0.06161	0.19096	0.27881	-0.0213	0.01578	-0.00573	0.1495	0.01524	0.07807	0.21992	0.04374	0.06835	-0.00637	0.10587	0.07935	-0.38628	0.1431	0.28595	-0.00419	0.15691
0.2017	0.16327	0.08914	0.4675	0.09379	0.04463	0.17408	0.26608	0.01231	0.04612	0.05536	0.17189	0.08991	0.11268	0.25067	0.04196	0.07586	0.04787	0.11986	0.03459	-0.39693	0.17401	0.27276	-5.5E-05	0.14251
0.16359	0.13021	0.03965	0.45024	0.11577	0.06624	0.15531	0.28281	0.00938	0.00676	-0.0002	0.1474	0.05363	0.07639	0.23676	0.05217	0.04097	0.00813	0.07264	0.09544	-0.40663	0.15748	0.29099	0.0008	0.17284
0.1796	0.07137	0.10065	0.43791	0.14082	0.063	0.16372	0.2643	-0.02707	0.02735	-0.10264	0.13884	0.05283	0.07635	0.27975	0.06943	0.01337	0.03253	0.09539	0.10409	-0.42396	0.12768	0.28918	0.01492	0.21602

TROPHY	3_INFLAM	VIRAL_M	ADIPOGEN	OBIC_GL	XIN_B1_M	RAGE_P	MUSCLE	)_ASPART	GRAFT_R	ITA_4_SIG	ROM_SYN	F_MUC1	Y_OF_FET	IMER_39	_DISEASE	:_ACID_M	TRIPLE_N	Y_EXCER	TRANSPO	F_ONCOG
0.2152	0.11099	0.1145	0.04269	0.40759	0.17404	0.14589	0.14333	0.11518	0.03027	0.10017	0.05229	0.08945	0.36368	0.16028	0.16028	0.21319	0.13362	0.12587	0.24027	-0.11206
0.23699	0.04929	0.13142	-0.02165	0.41105	0.12542	0.11691	0.09797	0.09124	-0.00275	0.06419	0.05723	0.09648	0.29742	0.14923	0.14923	0.16829	0.11423	0.06889	0.27849	-0.08632
0.22825	0.12765	0.13683	0.01656	0.42783	0.17023	0.1471	0.16136	0.10621	0.07738	0.08227	0.0653	-0.00119	0.304	0.15129	0.15129	0.20759	0.26203	0.18035	0.3796	-0.12185
0.25372	0.13527	0.13601	0.04397	0.40656	0.15755	0.15849	0.16283	0.06199	0.13738	0.06776	0.0788	0.06718	0.26724	0.16944	0.16944	0.16431	0.0653	0.11906	0.3833	-0.05106
0.22313	0.18865	0.14714	0.0265	0.41344	0.08518	0.17759	0.13173	0.09606	0.11206	0.05067	0.08892	0.08289	0.2787	0.17384	0.17384	0.18211	0.11584	0.108	0.29047	-0.09795
0.31156	0.12879	0.10749	0.02402	0.40722	0.11698	0.12664	0.14522	0.11499	0.15851	0.04467	0.0297	0.03883	0.25821	0.15063	0.15063	0.18714	0.32133	0.10597	0.37115	0.01179
0.32217	0.18411	0.16361	0.00158	0.39904	0.21402	0.1571	0.127	0.13927	0.15595	0.08934	0.01	0.06104	0.29441	0.15019	0.15019	0.18633	0.24387	0.17147	0.41466	0.17224
0.25374	0.1746	0.15381	0.0499	0.4126	0.13263	0.1634	0.15506	0.1343	0.12462	0.0781	0.07647	0.06491	0.31083	0.16595	0.16595	0.18513	0.1533	0.13444	0.41131	0.0715
0.26461	0.2482	0.168	0.04317	0.40859	0.14458	0.16244	0.15088	0.00839	0.19175	0.06421	0.09307	-0.05747	0.30295	0.16743	0.16743	0.15841	0.09441	0.11623	0.4099	-0.01861
0.26532	0.20318	0.15966	0.03353	0.39076	0.26127	0.17965	0.14842	0.03386	0.17978	0.05294	0.0718	0.07891	0.21331	0.16404	0.16404	0.15562	0.08249	0.1087	0.39105	-0.0477
0.26544	0.06981	0.10846	0.01951	0.41134	0.22928	0.12265	0.20108	0.20052	0.06325	0.07291	0.0596	0.02666	0.34501	0.15812	0.15812	0.21715	0.2657	0.20268	0.31178	-0.11583
0.26002	0.08277	0.12369	0.02008	0.39688	0.14074	0.13542	0.1957	0.0763	0.15448	0.09622	0.01213	0.03781	0.28246	0.14556	0.14556	0.21214	0.24999	0.11854	0.35829	0.0573
0.25452	-0.11707	0.08857	-0.03771	0.41736	0.20917	0.1323	0.16616	0.15218	0.02865	0.10743	0.05007	0.00772	0.32205	0.17419	0.17419	0.19805	0.22348	0.02604	0.26722	-0.20544
0.25619	0.14016	0.12363	0.00933	0.41288	0.25615	0.1416	0.13931	0.10611	0.10566	0.04822	0.0575	0.09337	0.28819	0.16244	0.16244	0.19911	0.14513	0.14063	0.32922	-0.04424
0.25919	0.11877	0.11218	0.02749	0.40654	0.20248	0.13711	0.17764	0.15676	0.07635	0.07029	0.09698	0.05079	0.21929	0.16082	0.16082	0.19403	0.21392	0.10614	0.32746	-0.13909
0.22084	0.01501	0.10943	-0.01165	0.43008	0.30257	0.12789	0.1494	0.16681	-0.00841	0.10199	0.03624	-0.00406	0.20563	0.1649	0.1649	0.22883	0.25888	0.19454	0.33222	-0.15778
0.21885	0.11576	0.13584	0.00942	0.43648	0.14947	0.13944	0.13453	0.06266	0.03351	0.10337	0.04561	0.04235	0.20921	0.16498	0.16498	0.18695	0.13482	0.17257	0.25542	-0.04108
0.22525	0.1811	0.14416	0.00765	0.40374	0.08714	0.15556	0.13217	0.05338	0.1292	0.08263	0.0894	0.05107	0.30364	0.17919	0.17919	0.17216	0.06903	0.12284	0.37555	0.12604

PROTEIN	_LATERA	R_NETWO	CEPTOR_S	ANGIOGEN	PROTEIN_	_RECEPT	Y_EFFECT	FLAMMAT	_APOPTO	ODULATIO	_MODULA	UE_TO_A	OXYGENA	SHT_VENT	REACTIVE	MINE_ME	N_RECE
-0.01971	0.10906	0.06922	0.10945	0.08835	0.00181	-0.06757	-0.03187	-0.01833	-0.09293	-0.01384	0.00714	0.17769	0.27193	0.19679	0.41379	0.43791	0.07247
-0.04216	0.08493	0.06576	0.08908	0.11514	-0.04082	-0.03312	-0.07591	-0.03718	-0.08518	-0.00689	0.00311	0.1746	0.25531	0.19103	0.35324	0.08913	0.00714
-0.01005	0.1301	0.07945	0.09145	0.10407	-0.0237	-0.00698	-0.0386	0.02314	-0.06554	0.00964	0.04043	0.1957	0.25801	0.23298	0.3778	0.34596	0.04672
0.02174	0.13416	0.06914	0.08145	0.08114	-0.0076	0.07765	-0.03437	-0.00583	-0.08299	-0.01285	0.03658	0.17748	0.26128	0.16527	0.37459	0.28254	0.03016
-0.0102	0.16791	0.07031	0.07857	0.098	-0.00884	0.02576	0.01345	0.01736	-0.046	0.00761	0.06748	0.17988	0.25966	0.13167	0.36644	0.42434	0.07987
0.01039	0.12834	0.08805	0.08807	0.02576	-0.03587	0.02326	-0.04531	-0.00349	-0.0946	-0.03771	0.05092	0.20041	0.29737	0.15615	0.33838	-0.04369	0.04609
0.00737	0.12145	0.08081	0.1125	0.11895	-0.01915	0.02502	-0.08153	0.05051	-0.03999	0.00626	0.07252	0.21019	0.28661	0.16083	0.35379	0.11784	0.05664
0.013	0.14533	0.06626	0.07482	0.09861	-0.00659	0.06876	0.01913	0.04244	-0.06793	0.01343	0.03547	0.17236	0.24259	0.11088	0.35593	0.40056	0.04829
0.03634	0.15098	0.07606	0.08027	0.0936	-0.00472	0.05525	0.03163	0.06052	-0.04803	0.01431	0.06667	0.19521	0.25893	0.15798	0.34638	0.38224	0.06415
0.00905	0.15049	0.06267	0.07706	0.10679	-0.00917	0.04447	-0.01372	0.0467	-0.0469	0.01658	0.08303	0.1987	0.24543	0.08509	0.35801	0.39616	0.07761
-0.01862	0.10763	0.06501	0.11187	0.10866	-0.03192	-0.05354	-0.07672	0.00442	-0.09129	0.00875	0.04239	0.19599	0.28352	0.20721	0.36556	0.22935	0.0465
-0.01358	0.12272	0.07364	0.09823	0.10146	-0.02458	0.01859	-0.10903	0.01762	-0.09678	-0.01115	-0.00034	0.19548	0.28764	0.16955	0.37743	0.11009	0.02133
0.02734	0.09145	0.11115	0.1324	0.08796	0.02302	0.02932	-0.14539	-0.0173	-0.08234	0.01161	0.02416	0.19071	0.29274	0.24158	0.32947	0.25119	0.03803
-0.01303	0.11795	0.05074	0.08546	0.10984	-0.01733	-0.01577	-0.02477	-0.00094	-0.07675	0.00618	0.01624	0.19171	0.26137	0.19082	0.34856	0.3058	0.03999
-0.0167	0.13205	0.08192	0.09112	0.08927	-0.01942	-0.0151	-0.05441	0.02085	-0.10413	0.0051	0.04212	0.19792	0.27144	0.18593	0.3769	0.40902	0.03056
0.001	0.12056	0.09912	0.12341	0.11456	-0.02177	-0.03664	-0.12316	0.02858	-0.09859	0.00375	0.0333	0.17697	0.32571	0.21869	0.37646	0.03364	0.04817
0.02803	0.10658	0.06314	0.09999	0.09499	-0.01245	0.00129	-0.07375	-0.02695	-0.07328	-0.00258	0.01862	0.16713	0.28957	0.16044	0.38304	0.29681	0.03801
-0.00557	0.16124	0.05704	0.08674	0.04943	-0.01071	-0.0038	-0.01107	0.03739	-0.046	0.01615	0.0486	0.18132	0.25621	0.20267	0.36593	0.42399	0.0331

N_RECE	N_DEVEL	GNALING	R_SIGN	_AUTOPH	REATIC_D	SIVE_OST	AGE_ST	NT_BIEDL	_EXCISIO	_TRKB_S	ENE_ME	_PYRENE	_AND_EN	IC_AMINE	RIMIDINE	R_UREA	HYDRO
0.04546	0.07268	-0.07401	0.01548	-0.13377	0.00274	0.06664	0.12892	-0.09451	0.02357	0.03474	0.28017	0.21434	0.36249	0.33363	-0.08659	0.02578	0.05725
-0.02009	0.11552	-0.0342	0.05685	-0.15447	0.00482	0.03895	0.11156	-0.04582	0.15579	0.03565	0.17635	0.08489	0.31423	0.21437	-0.04557	-0.06631	0.09048
0.03548	0.08475	-0.03058	-0.05286	-0.17864	-0.02488	0.03797	0.14129	-0.04254	0.12068	0.03126	0.27585	0.13278	0.29068	0.31319	-0.02048	0.00888	0.10438
0.05053	0.08196	-0.06805	0.0155	-0.11762	0.04074	0.08861	0.12415	-0.01617	0.07229	0.03092	0.26437	0.09541	0.31005	0.35758	-0.06928	-0.01655	0.00096
0.03337	0.07755	-0.04767	-0.01696	-0.13356	0.07842	0.06125	0.12149	-0.01963	0.08042	0.03604	0.22593	0.12439	0.31371	0.33539	-0.03627	0.06782	0.05962
0.00559	0.09586	-0.07033	0.01604	-0.19278	0.02601	-0.07032	0.11654	-0.04097	0.14637	0.02013	0.2421	0.14273	0.18118	-0.03259	-0.06313	0.05222	0.00309
0.03573	0.08175	-0.05086	0.00643	-0.16458	0.00948	0.04513	0.14557	-0.03028	0.1182	0.01863	0.30163	0.10169	0.2741	0.28149	-0.06594	0.01407	0.08505
0.07939	0.04338	-0.09241	-0.02248	-0.12077	0.02693	0.13724	0.12694	0.0312	0.03983	0.01073	0.25976	0.11464	0.30074	0.37115	-0.06686	-0.01229	0.09186
0.10845	0.04694	-0.07844	-0.02637	-0.10151	0.03114	0.11521	0.13815	0.00672	0.05139	0.01237	0.27686	0.13862	0.27871	0.36586	-0.03125	-0.05888	0.03254
0.06416	0.0648	-0.07825	0.03569	-0.11999	0.03383	0.13309	0.12154	-0.02602	0.0904	0.01852	0.37731	0.14596	0.25997	0.33785	-0.06112	-0.0404	-0.00318
0.06557	0.09506	-0.00415	-0.03943	-0.1309	-0.02839	-0.01708	0.13233	-0.01396	0.10614	-0.00941	0.31712	0.15118	0.29415	0.24185	-0.05967	0.11432	0.06929
0.06733	0.07973	-0.06084	-0.01712	-0.16594	0.04313	0.01016	0.11589	-0.04037	0.10636	0.00247	0.29212	0.12746	0.34871	0.28959	-0.04528	-0.01633	0.03845
-0.02103	0.08788	-0.00947	0.07769	-0.06957	0.00668	-0.04711	0.13488	0.03125	0.09717	0.07353	0.19781	0.12276	0.37626	0.32993	-0.10647	0.04799	0.13587
0.0465	0.07304	-0.06405	-0.00868	-0.16058	0.04321	0.07516	0.12475	-0.00147	0.09072	0.0341	0.34606	0.12339	0.30601	0.35388	-0.10201	-0.06493	0.10037
0.04213	0.0624	-0.04106	-0.00459	-0.11031	0.0166	0.03894	0.12594	-0.05488	0.09135	0.00954	0.31176	0.12175	0.34593	0.29681	-0.07943	0.03079	0.07964
0.0115	0.12722	0.03418	0.01897	-0.14314	-0.00676	-0.01061	0.12813	-0.11747	0.1498	0.04413	0.2571	0.11424	0.31972	0.32112	-0.07091	0.04998	0.09764
0.04703	0.09851	-0.07136	-0.00145	-0.1164	0.03036	0.00967	0.12872	-0.03349	0.06241	0.01358	0.26293	0.09416	0.32526	0.30104	-0.05653	-0.04084	-0.03195
0.05263	0.06827	-0.05473	-0.07703	-0.14425	0.02909	0.11922	0.12355	-0.0586	0.04021	0.01496	0.24196	0.13729	0.30901	0.39299	-0.04459	-0.05159	0.0635

OVER_O	BOLISM	LADDER_C	CLOTTIN	RY_ENDO	G_IN_EYE	ROTEIN_S	PHIC_FACT	CANCER	WOUND	PTOR_SI	ULATION	ESTRICT	MKK2_PA	THERAPY	THERAPY	D_RECEP	N_CANON
0.11534	-0.04249	0.10814	0.06351	0.00553	-0.03908	-0.23055	0.13321	0.01304	0.13245	0.00858	0.18826	-0.011	0.16283	0.12329	0.04432	0.20672	-0.0579
0.10477	-0.04084	0.06224	0.10069	0.11698	0.062	0.07865	0.11629	0.0012	0.12819	-0.01976	0.16912	0.01983	0.14979	0.06409	-0.02263	0.14819	-0.10301
0.12922	-0.05105	0.09729	0.13736	0.02831	-0.0944	-0.24189	0.1104	0.04056	0.14976	0.02188	0.16251	0.00597	0.14936	0.18858	0.01971	0.12879	-0.0347
0.2097	-0.16441	0.10591	0.22102	-0.0011	-0.05204	-0.18999	0.12913	0.02043	0.21084	0.04908	0.21474	0.00606	0.15926	0.2241	0.038	0.17215	-0.04261
0.12195	-0.13966	0.08659	0.13503	-0.0971	-0.11207	-0.31318	0.11888	0.01869	0.17358	0.06377	0.19599	-0.00316	0.17291	0.19294	0.15851	0.15113	0.0228
0.13119	-0.10875	0.01532	0.25475	-0.07241	-0.25117	-0.37068	0.1079	-0.00724	0.15434	0.0208	0.18238	-0.02298	0.11751	0.15414	0.1784	0.09754	-0.19094
0.13902	-0.03527	0.10724	0.23657	0.0051	-0.04623	-0.11992	0.10627	0.03529	0.20628	0.04576	0.17309	-0.0287	0.13608	0.20483	0.1451	0.12033	-0.09085
0.20262	-0.09179	0.09412	0.25894	-0.11883	-0.07509	-0.24957	0.12965	0.00434	0.21231	0.07594	0.20987	-0.01376	0.16704	0.23299	0.06666	0.18367	-0.03593
0.19293	-0.16812	0.09243	0.24119	-0.13557	-0.11149	-0.26574	0.12917	0.00861	0.22619	0.08135	0.19429	0.00053	0.16075	0.24437	0.06196	0.15844	-0.07938
0.18486	-0.13666	0.08016	0.22351	-0.26977	-0.14366	-0.37498	0.12494	0.00155	0.20266	0.09212	0.20675	-0.02581	0.14582	0.19943	0.18919	0.15595	-0.05345
0.08724	-0.05664	0.112	0.11017	0.12076	0.12479	0.04537	0.08717	0.02796	0.14131	-0.02455	0.16214	-0.04043	0.15307	0.16974	0.01108	0.11921	-0.1081
0.11515	-0.10447	0.08698	0.22713	-0.04437	-0.01849	-0.19047	0.09774	0.01443	0.18802	-0.00093	0.20322	-0.01564	0.14609	0.11171	0.09445	0.17719	-0.11528
0.12783	-0.22143	0.0779	0.12691	0.13747	0.07689	0.04338	0.08771	0.07743	0.15362	-0.05293	0.159	0.04764	0.14469	0.09001	-0.05753	0.1421	0.03677
0.16247	-0.10353	0.04631	0.15084	0.02687	-0.03778	-0.13975	0.10139	0.01027	0.17816	0.03028	0.18146	0.03018	0.17337	0.15152	-0.00539	0.15001	-0.10944
0.10129	-0.07055	0.07443	0.20397	0.00781	-0.05083	-0.22574	0.13128	0.01006	0.17417	0.01078	0.18822	-0.03339	0.1356	0.13914	0.00883	0.13948	-0.09356
0.06702	-0.05128	0.07785	0.09845	0.15678	0.13206	0.01933	0.08952	0.0712	0.11913	-0.07263	0.14966	0.02166	0.11063	0.07017	-0.02771	0.10607	-0.02258
0.17376	-0.0143	0.13127	0.19274	-0.0242	-0.07945	-0.2098	0.11027	0.03022	0.17587	0.02455	0.19507	-0.00454	0.14892	0.1817	-0.02093	0.12768	-0.04164
0.22237	-0.13643	0.06152	0.1353	-0.02723	-0.0568	-0.22679	0.11861	0.0304	0.1996	0.06482	0.18643	0.00223	0.17031	0.23099	0.11294	0.16133	-0.10837

N_CANO	CAL_NF	YPERTRO	OGENITOR	NG_PATH	SIGNALIN	D_IN_LO	LAR_PRO	CELL_CY	NTIATION	FERENTI	PANCREA	FOR_NEU	ND_INVA	_SELECT	ACIDURI	E_SIGNA	CHOLEST
-0.16154	-0.04398	0.04896	-0.05026	0.18035	0.04166	0.09332	0.16292	0.088	-0.02378	0.34349	0.02543	0.01872	0.01679	-0.09998	0.17272	0.07441	0.0991
-0.10942	-0.03426	0.03443	-0.0413	0.11908	-0.00811	0.13224	0.09842	0.09415	-0.04672	0.27374	0.00922	0.05781	0.01804	-0.09299	0.12744	0.02076	0.09532
-0.06709	-0.06937	0.04176	0.02649	0.15337	-0.04523	0.12214	0.04566	0.1157	-0.08779	0.19535	0.05733	0.04055	-0.01277	0.0472	0.16114	0.0641	0.14105
-0.12861	-0.03435	0.02286	-0.02844	0.13668	-0.11397	0.19396	0.05813	0.0896	-0.08288	0.24819	0.05938	0.04804	-0.00111	0.12608	0.12566	0.11195	0.04168
-0.09363	0.02037	0.0664	-0.01661	0.12792	-0.13636	0.1426	0.13546	0.08952	-0.03243	0.30791	0.02428	-0.0093	0.03998	-0.08728	0.13784	0.10884	0.03287
-0.10152	-0.08541	0.02771	-0.01493	0.122	0.02862	0.2543	0.14401	0.13117	-0.04582	0.27057	0.07272	0.00943	-0.05517	-0.09663	0.18585	0.05705	0.08976
0.02271	-0.04567	0.04921	-0.00123	0.13865	0.00424	0.23105	0.08317	0.10026	-0.07796	0.23375	0.06974	0.02485	0.01302	-0.07678	0.23516	0.10325	0.14141
-0.02051	0.0032	0.05194	0.01342	0.16123	-0.15234	0.23141	0.07611	0.04118	-0.04075	0.33063	0.06622	0.01384	0.04205	0.10343	0.08879	0.1295	0.09081
-0.04612	-0.00879	0.03562	0.02127	0.16228	-0.08367	0.26474	0.10256	0.02974	-0.05546	0.29593	0.16783	0.02858	0.00359	0.07988	0.09864	0.13679	0.02245
-0.09563	0.04808	0.06204	-0.04206	0.1189	-0.09382	0.20653	0.09298	0.03054	-0.0506	0.291	0.06712	0.03446	0.04264	0.12162	0.0946	0.13542	0.00628
-0.06823	-0.12087	-0.01346	0.01781	0.13167	-0.05951	0.1548	0.13941	0.17298	-0.09521	0.26422	0.1238	-0.00623	0.03877	-0.00548	0.19169	0.02087	0.20618
-0.05535	-0.10676	0.01286	-0.07174	0.19744	-0.0398	0.24045	0.12165	0.12248	-0.03941	0.29664	0.04698	0.0394	-0.03032	0.04042	0.15141	0.05035	0.20393
-0.13172	-0.20743	0.00797	-0.12403	0.16272	-0.00947	0.10932	0.17149	0.15251	-0.06893	0.26951	0.03466	0.01805	0.05496	0.02159	0.24622	0.02624	0.1418
-0.09727	0.00566	0.0366	-0.05085	0.1134	-0.1107	0.18461	0.06721	0.04346	-0.03943	0.26651	0.12183	-0.0122	-0.00392	-0.09315	0.14157	0.08836	0.15677
-0.07229	-0.0621	0.0221	0.01469	0.1797	0.00534	0.11103	0.11736	0.09747	-0.10721	0.21226	0.09318	0.01992	0.03706	-0.05364	0.15541	0.06081	0.2304
-0.11112	-0.14663	0.00842	-0.00564	0.16532	-0.07806	0.01184	0.21164	0.13475	-0.0966	0.22935	0.05828	0.04509	0.0176	-0.1023	0.21629	0.00703	0.1629
-0.16144	-0.07096	0.01754	0.01295	0.14055	-0.138	0.174	0.10423	0.03954	-0.07567	0.24855	0.03693	0.03128	0.01583	0.01153	0.17852	0.09871	0.1309
-0.14637	-0.01498	0.04551	-0.02126	0.15783	-0.19089	0.17993	0.09169	0.0558	-0.05008	0.30174	0.04135	0.07268	0.00071	-0.00046	0.13686	0.114	0.06355

L_BIOSYN	THESIS	THESIS_V	STEROL	BLOCH	L_SYNTH	LLITE_IN	A_IMPAIR	ARY_LAN	CILIOPAT	HAN_RHY	GNALING	H_GLUCO	AL_CELL	_AUTOPH	CORNEL	_UNDERL	EMENT_A
-0.12045	0.02392	0.19395	0.05636	0.02772	-0.04604	0.0597	0.07725	0.10421	-0.15603	0.12749	0.09982	0.26455	0.22379	0.01107	0.01199	0.30011	-0.07595
-0.15139	-0.04184	0.0672	0.00989	-0.03702	-0.12835	0.04277	0.05589	0.09689	-0.11993	0.15054	0.09067	0.20636	0.2214	-0.00488	0.03943	0.26436	-0.12561
-0.13273	0.00985	0.18006	0.05977	-0.00169	-0.04904	0.06836	0.05243	0.1046	-0.13176	0.14128	0.09384	0.31027	0.20669	-0.0202	0.0687	0.27208	-0.00514
-0.15769	0.00439	0.19205	0.1266	0.01005	-0.06307	0.05356	0.09719	0.10683	-0.10903	0.11726	0.09553	0.20157	0.2071	0.04546	0.01311	0.2889	0.12171
-0.10057	0.01176	0.16594	0.11039	0.0143	-0.0376	0.06509	0.13717	0.1141	-0.10313	0.13734	0.08986	0.17586	0.21318	0.03323	0.05915	0.29737	0.02303
-0.13623	-0.00707	0.19623	0.104	-0.0088	-0.05516	0.03421	0.06276	0.10304	-0.12968	0.14402	0.07101	0.11559	0.22567	-0.0263	0.04782	0.2697	0.26962
-0.11609	-0.02182	0.16511	0.08512	-0.00686	-0.03852	0.05758	0.10598	0.10875	-0.12884	0.11864	0.10802	0.19636	0.22059	0.03689	0.02453	0.27116	0.25384
-0.13897	-0.01885	0.05808	0.13085	-0.00015	-0.09868	0.03853	0.10272	0.09881	-0.0835	0.10355	0.08165	0.29069	0.20272	0.02396	-0.0162	0.29102	0.12802
-0.12966	0.00569	0.12946	0.13619	0.01512	-0.05894	0.06199	0.09437	0.10121	-0.09859	0.11707	0.08976	0.24157	0.19936	0.03504	-0.00098	0.28246	0.14337
-0.16254	-0.02314	0.07244	0.11537	-0.01451	-0.09671	0.04854	0.09731	0.09053	-0.11873	0.11415	0.09978	0.16619	0.19608	0.04216	-0.00274	0.293	0.15617
-0.12112	-0.01564	0.27395	0.03509	-0.00306	-0.02107	0.05139	0.11698	0.12986	-0.0958	0.15287	0.09189	0.30718	0.2443	0.03286	0.02634	0.2716	0.04791
-0.06406	0.03821	0.21001	0.0962	0.02799	0.0045	0.05231	0.05323	0.11181	-0.11031	0.14709	0.10261	0.20774	0.21373	0.02486	0.03583	0.28233	0.18965
0.02251	0.05975	0.21921	0.08572	0.06082	0.06468	0.09496	0.05888	0.12493	-0.04617	0.14842	0.08088	0.23324	0.23131	0.03035	0.07133	0.27933	0.02616
-0.15665	0.01084	0.18983	0.08787	0.00662	-0.0816	0.04465	0.09676	0.10521	-0.11126	0.13432	0.07474	0.28661	0.20075	0.0202	0.02193	0.27685	0.07111
-0.05373	0.02778	0.21132	0.07335	0.02757	0.01344	0.03891	0.11907	0.11391	-0.14048	0.12657	0.08247	0.21513	0.21779	0.00906	0.01302	0.29281	0.03245
-0.06563	-0.0106	0.0989	0.00076	-0.01702	-0.03375	0.07685	0.07572	0.12219	-0.14868	0.14442	0.09886	0.18297	0.23118	0.04739	0.07279	0.26853	-0.19177
-0.16584	-0.03773	0.02939	0.08907	-0.03486	-0.12162	0.05049	0.07966	0.09312	-0.11762	0.13279	0.08111	0.15856	0.20767	0.01687	0.00559	0.28476	-0.02392
-0.16765	-0.00566	0.13103	0.09279	-0.00847	-0.11018	0.03665	0.00104	0.09902	-0.12517	0.10634	0.08384	0.23355	0.19882	0.03167	-0.00777	0.28473	0.04224

ND_COAG	PLEMENT	EURONAL	NERALIZ	DROSTAN	ANCE_BY	IOTENSIN	ER_HOM	CORI_CY	ING_HO	ERSE_O	DOTHEL	BOSIS_A	EATINE_P	_METHIO	D_INFLA	C_RIBOS	SINE_MET
0.01169	0.08499	0.01604	0.02076	0.13345	-0.39307	0.45761	0.03472	0.41831	0.10452	-0.35393	-0.12031	0.34998	0.05018	0.21755	0.01274	0.39035	0.20865
-0.01415	0.06892	-0.0185	-0.07134	0.06789	-0.38258	0.27379	-0.10777	0.41127	0.08523	-0.35163	-0.44953	0.34504	0.11662	0.18473	-0.03994	0.4045	0.21045
0.08073	0.11768	0.06323	-0.0415	0.11967	-0.30826	0.44233	-0.08623	0.40887	0.09557	-0.32429	-0.01814	0.33351	0.21222	0.20258	0.05943	0.3942	0.17465
0.17311	0.18141	0.11918	-0.0253	0.09334	-0.33674	0.46028	-0.06707	0.40312	0.10656	-0.1484	-0.07187	0.4276	0.12335	0.17004	0.0867	0.40697	0.14464
0.09478	0.12948	0.05973	-0.11175	0.14109	-0.23776	0.473	-0.08846	0.40859	0.11539	-0.27907	-0.27215	0.36774	0.10514	0.18887	0.09037	0.4003	0.18576
0.25009	0.19367	0.13966	-0.04583	0.07077	-0.23175	0.37511	-0.09374	0.40753	0.08934	-0.065	-0.15572	0.46138	0.19072	0.1939	0.02751	0.41147	0.19897
0.2492	0.20253	0.18086	-0.02699	0.06889	-0.29124	0.37086	-0.06653	0.38572	0.11459	-0.17992	-0.10217	0.42279	0.12258	0.21518	0.1206	0.41165	0.18036
0.18604	0.20018	0.11194	-0.00108	0.14463	-0.25904	0.48097	-0.02529	0.38605	0.11219	-0.16572	-0.04862	0.42229	0.03882	0.17959	0.0954	0.40583	0.14544
0.20136	0.20387	0.12029	-0.02395	0.11894	-0.22405	0.44683	-0.06007	0.40329	0.116	0.23914	-0.02184	0.44235	0.14942	0.18997	0.23767	0.39717	0.1532
0.19805	0.19866	0.13564	-0.12554	0.10263	-0.18523	0.47648	-0.04848	0.38617	0.11882	-0.06258	-0.11168	0.42782	0.03846	0.15107	0.13764	0.39459	0.19364
0.08684	0.1162	0.08734	-0.02987	0.07897	-0.16264	0.44541	-0.1055	0.42681	0.08831	-0.22676	-0.11749	0.31843	0.25022	0.21094	0.07555	0.40339	0.19032
0.20407	0.17777	0.14901	-0.04169	0.11694	-0.20034	0.44936	-0.09828	0.39111	0.09162	-0.19342	-0.25225	0.3835	0.21712	0.21322	0.02997	0.40735	0.18943
0.08692	0.10381	0.0673	0.02569	0.10902	-0.41575	0.25437	-0.01192	0.41694	0.10929	-0.24179	-0.40655	0.37989	0.16331	0.17594	-0.03145	0.40059	0.17297
0.11589	0.14944	0.07843	-0.11565	0.12557	-0.34612	0.47051	-0.1384	0.41309	0.09746	-0.28632	-0.20786	0.37379	0.08179	0.17279	0.09553	0.40755	0.16786
0.12327	0.14838	0.09087	-0.05817	0.11057	-0.33671	0.47365	-0.06115	0.39921	0.09447	-0.22691	-0.05866	0.40039	0.04324	0.20995	0.08266	0.41338	0.18802
-0.02747	0.02635	-0.00168	0.01384	0.09005	-0.26338	0.23399	-0.06001	0.42341	0.07291	-0.34467	-0.24027	0.31081	0.22183	0.20298	0.05677	0.40181	0.11281
0.09104	0.12917	0.06667	-0.03576	0.09298	-0.39865	0.4713	-0.08031	0.39008	0.11091	-0.17082	-0.15395	0.39248	0.17917	0.17925	-0.00205	0.40907	0.16842
0.11054	0.13926	0.07392	-0.08696	0.11105	-0.26083	0.42378	-0.04025	0.40633	0.11991	-0.25449	-0.04756	0.36276	0.09028	0.17215	0.1198	0.39169	0.17892

DNA_SE	ONENT_C	_SPHING	AB_EFFE	ETEROG	DENDRITI	CURETER	F_WHITE	KETONE	D_SYNTH	ATE_MET	F_FRUC	F_GALAC	STSYNA	N_DUCHE	DAMAGE	PONSE_O	D_CELLU	EAKS_AN	MISMATC	PATHWAY
-0.0151	0.03701	0.09391	-0.09506	0.08187	-0.12353	0.11645	-0.04298	0.11102	0.08249	0.17944	0.35689	0.19923	0.24717	0.18235	-0.01243	0.058	0.11841	0.05305	-0.04066	0.02865
-0.03829	0.05029	0.05833	-0.2852	-0.15869	-0.1542	0.25105	0.13151	0.09879	0.03948	0.13194	0.34804	0.1506	0.23692	0.14205	0.01666	0.03368	0.17179	0.08676	0.05376	0.10055
-0.02317	0.0888	0.06495	-0.22652	0.00427	-0.10072	0.14354	-0.06393	0.09222	0.05168	0.22097	0.39761	0.14472	0.19316	0.15593	0.01532	0.06022	0.14863	0.09233	0.06951	0.07794
-0.01154	0.07384	0.2055	-0.12578	-0.00454	-0.16243	0.12233	0.06201	0.13543	0.06504	0.13298	0.38218	0.16043	0.21383	0.16299	0.00878	0.05318	0.12347	0.04435	0.00771	0.04617
-0.00745	0.02358	0.16685	-0.10847	-0.05329	-0.03486	0.03771	0.0323	0.17233	0.06178	0.15115	0.40346	0.17608	0.13876	0.13178	0.01866	0.06592	0.13396	0.07173	-0.01967	0.0513
-0.05782	0.03301	0.10239	-0.21006	-0.10053	0.00617	0.02679	-0.10771	0.02362	0.04907	0.15288	0.36077	0.13321	0.10693	0.11437	0.02449	0.03373	0.13239	0.05077	0.04946	0.09386
-0.00857	0.11225	0.0751	-0.14741	-0.04972	-0.15881	0.13188	-0.08729	0.13505	0.01363	0.20159	0.32403	0.15733	0.15288	0.16924	0.02703	0.057	0.1281	0.0725	0.00372	0.07315
-0.01604	0.03735	0.20949	-0.11579	-0.0088	-0.06281	0.01818	0.03004	0.10208	0.07082	0.09624	0.40114	0.20346	0.18045	0.13918	-0.01187	0.04947	0.07213	0.02309	-0.06756	0.00698
0.01628	0.0483	0.24463	-0.23746	0.19861	0.01811	0.0198	-0.03438	0.01971	0.06267	0.11101	0.39147	0.16239	0.13596	0.10497	-0.00179	0.06312	0.08316	0.04658	-0.05703	0.00865
0.0078	0.05837	0.1864	-0.15358	-0.02702	-0.00629	-0.06318	-0.04949	0.10667	0.01425	0.16179	0.38406	0.15436	0.16094	0.16762	0.02341	0.05349	0.09404	0.05539	0.0032	0.05158
-0.04837	0.07112	-0.06873	-0.19005	-0.01163	-0.15874	0.17684	0.07278	0.29911	0.01788	0.25159	0.33703	0.16876	0.1608	0.13119	0.04333	0.05468	0.17018	0.10266	0.03586	0.06651
-0.02205	0.07642	0.10046	-0.20633	0.01903	-0.09121	0.03529	-0.1026	0.21407	-0.01018	0.1801	0.34165	0.14791	0.21791	0.12826	0.03684	0.03924	0.13887	0.1072	0.04627	0.08359
-0.05262	0.10411	-0.08745	-0.25662	-0.04466	-0.15391	0.19945	0.00882	0.19944	-0.02451	0.24198	0.27186	0.19962	0.21227	0.12599	0.04632	0.07547	0.16688	0.11341	0.07496	0.09834
-0.01354	0.0667	0.17193	-0.16004	-0.01108	-0.08838	0.10176	0.13583	0.19046	0.0397	0.08775	0.38423	0.22885	0.21187	0.11233	-0.00679	0.04438	0.10417	0.0711	-0.05076	0.04317
-0.02265	0.09196	0.09737	-0.13422	-0.05625	-0.17278	0.10734	0.00967	0.16441	0.05552	0.20648	0.37215	0.20767	0.21323	0.14724	0.02448	0.02583	0.12616	0.09496	0.00518	0.05715
-0.05376	0.22812	-0.04263	-0.26829	-0.12482	-0.16524	0.20684	0.08616	0.2062	-0.01237	0.26736	0.33754	0.19175	0.20874	0.11932	0.0758	0.04347	0.16839	0.14243	0.05431	0.10752
-0.03448	0.07777	0.18104	-0.13582	-0.04637	-0.10808	0.16401	0.01785	0.16742	0.03147	0.1419	0.38231	0.15865	0.17302	0.16422	0.00357	0.06231	0.10127	0.06774	-0.09003	0.02755
-0.00085	0.10938	0.19219	-0.20152	0.02195	-0.06034	0.0843	-0.0452	0.10697	0.08546	0.14545	0.39585	0.16786	0.17537	0.13245	0.02043	0.06509	0.11051	0.06341	-0.09065	0.03238

ON_OF_E	DEL_OF_\	CELL_PI	US_INFE	R_VIRUS	RM_DIFF	HAIR_FC	S_OF_NI	NTICOAG	VED_IN_	KINASE_I	R_SIGNA	LISM_VIA	TOCHROI	DLISM_VI	ANOID_S	AIN_OXP	CELL_PL	ONDRAL_	CATION_	RM_DIFF
-0.43058	0.11196	-0.04733	0.14523	0.1011	0.04459	-0.38242	0.17339	-0.24485	0.23965	0.01837	0.06939	-0.00856	-0.35542	0.02111	0.09913	0.20668	0.00021	-0.0126	-0.0126	0.01675
-0.33634	0.06871	-0.02574	0.11827	0.05467	0.08811	-0.39457	0.17753	-0.42205	0.28015	-0.009	0.0325	0.02358	-0.37267	0.02334	0.14213	0.20956	-0.007	0.0016	0.0016	0.06233
-0.45365	0.1174	-0.05789	0.15141	0.05826	0.06143	-0.46116	0.22039	0.04721	0.25895	0.01551	0.0707	0.05035	-0.33406	-0.01557	0.15195	0.19198	-0.01075	0.00147	0.00147	0.06661
-0.43037	0.12006	-0.07501	0.175	0.06454	0.0665	-0.2984	0.27192	0.15237	0.22891	0.02311	0.06943	0.00683	-0.37601	0.00713	0.12857	0.21123	-0.0041	0.01537	0.01537	0.02474
-0.39286	0.16728	-0.08334	0.15758	0.08842	0.06793	-0.42875	0.20457	-0.02677	0.23011	0.06222	0.08326	-0.00448	-0.32307	-0.01387	0.12782	0.16524	0.00942	0.0095	0.0095	0.02874
-0.22952	0.1513	-0.0581	0.13386	0.0511	0.0778	-0.26193	0.19268	-0.02906	0.24233	-0.00272	0.05778	-0.06199	-0.18712	0.03696	0.14133	0.19934	0.00355	-0.00541	-0.00541	0.04205
-0.22632	0.13376	-0.0493	0.14628	0.0933	0.07688	-0.20011	0.19465	0.10657	0.23385	0.02257	0.07007	0.00119	-0.18758	0.02362	0.1757	0.2163	0.00151	0.02916	0.02916	0.03536
-0.06336	0.11876	-0.09784	0.17977	0.0817	0.02938	-0.37449	0.30117	0.09406	0.20016	0.03333	0.08571	-0.06035	-0.34445	-0.0127	0.11824	0.20375	-0.00592	0.0626	0.0626	0.01373
-0.37825	0.07556	-0.09561	0.18889	0.06675	0.04933	-0.42611	0.20296	0.16558	0.2131	0.03274	0.08723	-0.03768	-0.35974	-0.05158	0.1073	0.20031	-0.0082	0.02355	0.02355	0.02208
-0.38037	0.1446	-0.06692	0.18107	0.10932	0.02241	-0.29893	0.17139	0.11661	0.22032	0.02045	0.08443	-0.08715	-0.33266	-0.0553	0.08418	0.22302	-0.0106	0.03787	0.03787	0.01275
-0.44648	0.13796	-0.06871	0.12254	0.06064	0.07912	-0.46944	0.16952	-0.41994	0.25834	0.00668	0.04415	-0.0093	-0.21076	-0.01895	0.03731	0.23282	-0.00531	-0.06595	-0.06595	0.0627
-0.4586	0.10609	-0.08673	0.14211	0.06916	0.06546	-0.29188	0.37604	-0.41096	0.24236	0.00724	0.05387	-0.03261	-0.21548	0.02024	-0.00431	0.19978	-0.02634	-0.00909	-0.00909	0.02134
-0.42478	0.12401	0.02252	0.11509	-0.00795	0.07434	-0.3796	0.17118	-0.04967	0.29438	0.04863	0.06264	0.00437	-0.14409	-0.04229	-0.01479	0.12748	0.03794	-0.01295	-0.01295	0.07391
-0.44723	0.12364	-0.08438	0.15099	0.09703	0.05371	-0.45288	0.19625	-0.0569	0.22548	0.00618	0.06118	0.07954	-0.37442	0.0473	0.16377	0.19109	-0.02454	0.02017	0.02017	0.01437
-0.05899	0.05149	-0.06863	0.15326	0.08749	0.08529	-0.46257	0.18132	-0.35212	0.24528	-0.00583	0.04933	0.013	-0.14142	0.06753	0.1324	0.18806	-0.02202	0.00893	0.00893	0.04189
-0.42718	0.11191	-0.0434	0.13247	0.07185	0.09375	-0.4638	0.17364	-0.46521	0.24332	0.03386	0.04967	0.01933	-0.16342	0.01758	0.01693	0.19713	0.02126	-0.01751	-0.01751	0.05684
-0.27859	0.11586	-0.04713	0.14382	0.04142	0.06801	-0.41976	0.22099	-0.04721	0.25252	0.03454	0.06459	-0.00648	-0.12589	0.00616	0.11787	0.23917	-0.01848	0.03358	0.03358	0.02881
-0.41267	0.14505	-0.09777	0.18191	0.09804	0.07151	-0.43773	0.20703	-0.22336	0.20796	0.03175	0.07854	0.01331	-0.35526	-0.03493	0.1223	0.23041	-0.00224	-0.02819	-0.02819	0.03684

METRIAL	SS_RESP	THELIN_	RGY_MET	CHOLEST	CIRCULA	POTENTIAL	AL_TRANS	CEPTOR_	SIGNALIN	_HUNTIN	PORTO_	N_METAB	N_METAB	N_RECEP	N_SIGNAL	S_ON_HIS	PRODU
0.04019	0.18498	0.25294	-0.00946	0.02119	-0.41518	0.17948	0.05062	0.12297	-0.01367	0.04121	0.11542	0.23301	0.11337	0.02009	0.14067	0.00236	0.23693
0.00935	0.16486	0.22628	0.0104	-0.0419	-0.26475	0.18685	0.03696	0.04786	-0.06865	0.0488	0.12966	0.08501	-0.07407	-0.10693	0.12224	0.03853	0.05652
0.05947	0.13241	0.2307	-0.00886	0.0096	-0.43826	0.18081	0.0528	0.10432	-0.01599	0.05818	0.13649	0.15138	0.00809	-0.21536	0.10499	0.06391	0.16891
0.06355	0.13282	0.22289	-0.01556	0.02518	-0.40479	0.16563	0.02942	0.11921	-0.02548	0.00224	0.10819	0.10947	-0.00634	-0.12669	0.1422	-0.02337	0.15948
0.0498	0.13211	0.21677	0.0326	0.02946	-0.35476	0.19347	0.02796	0.12611	-0.00148	0.05423	0.14368	0.14105	0.03176	-0.18112	0.15619	0.01211	0.22343
-0.01457	0.12537	0.24986	0.03212	0.01368	-0.13194	0.14988	-0.01372	0.07107	-0.03146	0.03098	0.1628	0.06318	-0.19703	-0.11148	0.0821	0.01996	0.15333
0.064	0.09557	0.23048	-0.00748	0.01246	-0.10164	0.16802	0.0487	0.11517	-0.01478	0.03138	0.11846	0.12969	-0.01075	-0.15709	0.14567	-0.0531	0.2018
0.04014	0.09229	0.22822	-0.00662	0.00711	-0.38277	0.16202	0.00976	0.13694	-0.00413	0.02335	0.11001	0.10824	0.03704	-0.13029	0.15905	0.04478	0.19437
0.04773	0.10802	0.22477	-0.00849	0.02056	-0.33275	0.16045	0.04164	0.14248	-0.00744	0.07032	0.11197	0.1194	0.03223	-0.14969	0.13633	0.02642	0.14975
0.03469	0.12848	0.22146	0.00677	-0.00457	-0.34367	0.15408	0.01544	0.14624	-0.00443	0.06665	0.1044	0.12576	0.16673	-0.08698	0.1637	0.00484	0.20668
0.05151	0.13302	0.23646	-0.00383	0.00255	-0.45482	0.16058	0.03714	0.04508	-0.04065	0.09478	0.14787	0.13757	-0.03132	-0.11627	0.0791	0.0148	0.08831
0.0343	0.11516	0.20987	0.00411	0.02665	-0.44812	0.13774	0.0288	0.1038	-0.04468	0.03929	0.12665	0.12096	-0.0724	-0.09952	0.10728	0.00797	0.14477
0.08017	0.17751	0.2279	-0.01801	0.07684	-0.39457	0.18159	0.0532	0.07701	-0.01773	0.01994	0.05532	0.09884	-0.01435	-0.13286	0.08247	-0.00756	0.10341
0.0336	0.10755	0.20491	-0.02249	0.00069	-0.42329	0.18317	0.04454	0.11007	-0.0383	0.01095	0.15106	0.14339	0.09731	-0.03936	0.13309	-0.01083	0.12588
0.0278	0.13758	0.25429	-0.00208	0.03659	0.12559	0.16439	0.03305	0.11103	-0.03608	0.01895	0.1202	0.07786	0.01396	-0.15548	0.12032	0.05221	0.2311
0.06917	0.16942	0.22428	0.00427	0.01382	-0.39405	0.16167	0.03057	0.05911	-0.01635	0.07618	0.13266	0.17039	0.0447	-0.06275	0.0608	-0.02744	0.17852
0.05265	0.1491	0.21474	-0.00388	-0.03099	-0.17738	0.17599	0.0349	0.09908	-0.02017	-0.00191	0.08071	0.12406	0.00824	-0.12063	0.12067	-0.01782	0.13152
0.0734	0.10905	0.19656	0.00139	-0.01808	-0.38312	0.17472	0.02141	0.13053	-0.00831	0.03816	0.13174	0.16129	0.0508	-0.14877	0.15407	0.02139	0.16997

LIPID_BI	TRANSCR	CELLS_A	CED_CIR	M_OF_ACT	IN_THE_	EDIATED	CULAR_B	SULIN_L	HYPERLIP	HYPERLIP	HYPERLIP	HYPERLIP	HYPERLIP	PARTIAL_L	X_RECE	ESS_IND	ROTEINS_
-0.06787	-0.07557	-0.45129	0.19659	-0.00892	0.19244	-0.01742	0.07295	-0.0312	0.07168	0.14492	0.19873	-0.01601	0.07168	0.116	-0.13829	0.15735	0.08896
-0.05843	-0.05126	-0.44583	0.18688	0.00913	0.17178	-0.0102	0.01852	-0.05432	0.06819	0.1147	0.21811	0.00636	0.06819	0.11926	-0.30459	0.16067	0.07214
-0.03072	-0.03564	-0.47525	0.19018	0.05542	0.18276	-0.00616	0.07311	-0.02621	0.1391	0.16172	0.30318	0.04268	0.1391	0.13848	-0.19716	0.17639	0.09707
0.02084	-0.08543	-0.46785	0.1672	-0.05467	0.19671	-0.05422	0.15492	-0.07473	0.23228	0.20819	0.39016	0.13275	0.23228	0.12106	-0.10522	0.15995	0.1771
-0.04457	-0.07439	-0.4483	0.17294	-0.11288	0.16336	-0.02951	0.12011	-0.06963	0.21952	0.24054	0.3763	0.11795	0.21952	0.09626	-0.18551	0.17961	0.13782
-0.04455	-0.0661	-0.37308	0.16544	-0.02751	0.11483	-0.09268	0.08745	-0.09145	0.18537	0.23927	0.31449	0.08682	0.18537	0.12802	-0.16486	0.18125	0.14245
-0.07536	-0.04766	-0.43474	0.16077	-0.1381	0.21238	-0.0521	0.08454	0.00938	0.17537	0.22217	0.34358	0.07672	0.17537	0.10006	-0.22026	0.18062	0.12406
0.03932	-0.12941	-0.4476	0.16035	-0.09628	0.22397	-0.11071	0.13306	-0.05131	0.28104	0.23754	0.41203	0.16661	0.28104	0.09661	0.01087	0.15055	0.19794
0.08234	-0.10955	-0.44654	0.1667	-0.11529	0.1982	-0.10251	0.15637	-0.04487	0.29816	0.24061	0.41604	0.18469	0.29816	0.1066	-0.15001	0.17527	0.21552
0.00942	-0.1119	-0.4335	0.15934	-0.11297	0.19023	-0.13933	0.18338	-0.08541	0.28349	0.2424	0.40339	0.17083	0.28349	0.09199	-0.14165	0.17885	0.18446
-0.05589	-0.07602	-0.47948	0.17136	-0.048	0.18257	0.03588	0.05659	-0.03188	0.04085	0.109	0.18395	-0.0008	0.04085	0.1522	-0.14079	0.18007	0.05689
0.01526	-0.07829	-0.47155	0.18635	-0.07071	0.18189	-0.03207	0.09144	-0.01727	0.20787	0.26315	0.34756	0.12076	0.20787	0.08028	-0.11249	0.15394	0.09115
-0.10051	-0.04074	-0.3722	0.17987	0.01344	0.1219	0.06113	0.00997	0.04392	0.07644	0.15881	0.18703	-0.0091	0.07644	0.11762	-0.18025	0.16639	0.0462
0.05939	-0.08204	-0.48423	0.19397	-0.07077	0.1527	-0.03705	0.06418	-0.03978	0.17686	0.18296	0.33539	0.07861	0.17686	0.11927	-0.17906	0.15176	0.13777
-0.01751	-0.05699	-0.47543	0.16376	-0.06988	0.16682	-0.0711	0.07475	-0.04444	0.15465	0.18518	0.28796	0.06472	0.15465	0.08576	-0.09797	0.17222	0.09261
-0.10435	-0.0532	-0.35283	0.18206	0.0147	0.19454	0.04876	-0.02069	-0.00227	-0.05686	0.08108	0.06419	-0.10353	-0.05686	0.13684	-0.20437	0.16518	0.02484
-0.03258	-0.0602	-0.44689	0.19839	-0.10412	0.18911	-0.00447	0.09576	-0.02298	0.22211	0.25326	0.38129	0.11499	0.22211	0.12855	-0.17239	0.15367	0.15105
0.01495	-0.08285	-0.45411	0.1661	-0.13818	0.12965	-0.04644	0.12024	-0.08278	0.20855	0.23121	0.37283	0.1158	0.20855	0.12435	-0.14645	0.163	0.16762

CID_BETA	ACID_BI	CID_OMEG	CID_TRA	SIGNALIN	IN_CARD	FERROPT	HATEMIC	_PROLIF	RECEPT	ACETIC_A	PYRIMID	OCAL_AD	HK_AKT_M	D_CANCE	ATE_META	HORMON	OXA2_PA
0.23046	0.14604	0.08686	0.09024	0.3562	0.29487	0.12335	-0.12204	0.25086	0.1153	0.30773	-0.15138	0.09652	0.03189	0.15548	0.19114	0.14136	0.07128
0.17067	0.11721	0.12636	0.12387	0.36974	0.252	0.1255	-0.07575	0.15159	0.08208	0.2983	-0.09697	0.06854	0.01449	0.13473	0.16504	0.12329	0.0071
0.21842	0.16064	0.21813	0.15409	0.33146	0.27841	0.13555	-0.09357	0.18315	0.1308	0.34761	-0.13691	0.09535	0.02754	0.08117	0.19908	0.13916	-0.03566
0.2109	0.14088	0.19685	0.18248	0.3437	0.29706	0.14717	-0.11741	0.21833	0.18212	0.29551	-0.0922	0.11043	0.06972	0.04099	0.20738	0.16328	-0.05589
0.20977	0.13493	0.19918	0.16534	0.36153	0.2921	0.13947	-0.19334	0.19858	0.18322	0.32104	-0.08065	0.08244	0.02708	0.15058	0.17886	0.15544	-0.02058
0.1944	0.1676	0.22728	0.16162	0.32023	0.29393	0.15335	0.01518	0.146	0.09573	0.28932	-0.09477	0.09153	0.02552	0.10481	0.20121	0.11308	0.03533
0.22466	0.17377	-0.01184	0.15804	0.32935	0.26972	0.1476	-0.05273	0.19087	0.15697	0.29245	-0.0892	0.11622	0.05446	0.00851	0.23719	0.1514	0.03301
0.23813	0.16913	0.25239	0.22107	0.34096	0.30667	0.16147	-0.10434	0.20406	0.17947	0.33451	-0.09188	0.12152	0.06435	0.12121	0.19461	0.15844	-0.01888
0.21975	0.17099	0.26228	0.22288	0.34055	0.30106	0.14182	-0.11161	0.18776	0.19912	0.32951	-0.0568	0.11558	0.05631	0.14687	0.21087	0.16147	0.01255
0.2216	0.17051	0.24045	0.18081	0.33476	0.30317	0.13894	-0.08041	0.2194	0.21205	0.31558	-0.05394	0.0882	0.03789	0.1306	0.22172	0.16108	0.03964
0.21543	0.1468	-0.04255	0.12334	0.35419	0.25944	0.12044	-0.14673	0.16304	0.08298	0.26704	-0.11639	0.09688	0.03095	-0.04008	0.21445	0.12278	0.01687
0.21672	0.16448	-0.0269	0.07293	0.3475	0.29805	0.11303	-0.09497	0.22036	0.14091	0.22991	-0.09715	0.09082	0.03375	0.07305	0.23342	0.1577	0.04724
0.17815	0.10931	-0.21441	0.03603	0.34657	0.28204	0.10202	-0.22334	0.1892	-0.00155	0.22345	-0.11209	0.09842	0.05825	0.03385	0.19978	0.14118	-0.01422
0.2245	0.14591	0.23105	0.15881	0.34882	0.28369	0.12416	-0.12105	0.1867	0.16048	0.30608	-0.13823	0.0835	0.03092	0.08329	0.17254	0.1428	-0.00516
0.18544	0.14769	0.23953	0.09204	0.35924	0.26681	0.1352	-0.13268	0.19898	0.1474	0.32082	-0.11128	0.08865	0.01946	0.08576	0.20543	0.14137	0.09065
0.20391	0.15533	-0.06915	0.0894	0.36654	0.27372	0.11099	-0.18011	0.20417	0.03683	0.30165	-0.10177	0.09029	0.02579	0.07397	0.19758	0.14896	0.08747
0.22063	0.14529	0.15037	0.13569	0.35728	0.31466	0.11306	-0.1251	0.18801	0.14098	0.2926	-0.13714	0.10617	0.06044	0.09652	0.1962	0.12266	0.02636
0.2366	0.15309	0.22678	0.16623	0.3551	0.29906	0.14774	-0.1765	0.20982	0.1754	0.32297	-0.07541	0.10614	0.05831	0.11471	0.18662	0.1501	-0.05886

XP3_IN_C	GILE_X_S	ITY_VARIA	IGNALING	CELL_CY	ETABOLIS	ECEPTOR_	RECEPT	S_AND_D	SPHING	HINGOLI	CANCER_	CANCER_	SIGNALI	RET_SIGN	DEVELO	ROLLING	CILIUM_
0.08104	0.14451	-0.21825	0.22748	0.04708	0.03463	0.14052	-0.06152	0.21404	0.1267	-0.03489	0.16981	0.06076	0.0612	0.01262	0.02812	0.11102	-0.11961
-0.03867	0.11886	-0.10798	0.1595	0.01254	0.05694	0.12968	-0.06226	0.23223	0.09711	-0.12366	0.16938	0.10376	0.04282	0.14311	-0.06873	0.11618	-0.07444
-0.05626	0.12108	-0.1529	0.22905	0.0975	0.09674	0.195	-0.03653	0.26004	0.16826	-0.0726	0.13375	0.10801	0.07007	0.21635	0.06806	0.11887	-0.09039
0.04025	0.10651	-0.3678	0.21489	0.03581	-0.08142	0.20482	-0.00035	0.19678	0.20057	-0.0698	0.02738	0.04846	0.06058	-0.00956	0.06646	0.09321	-0.06333
0.10449	0.11841	-0.19799	0.23416	0.0519	-0.01242	0.20344	0.02668	0.14982	0.15254	-0.18755	0.08194	0.05719	0.05535	-0.01683	0.08168	0.02836	-0.01905
0.08428	0.0913	-0.1182	0.21369	0.09279	0.03371	0.17039	-0.00045	0.2758	0.13602	-0.24749	0.04435	0.0816	0.02379	0.09444	-0.12706	0.06479	-0.10074
0.02304	0.10281	-0.17404	0.23538	0.04468	-0.01689	0.15887	-0.03302	0.22424	0.15943	-0.17362	0.07563	0.0762	0.06627	0.09778	0.07358	0.09146	-0.06792
0.10188	0.11468	-0.20099	0.23787	-0.05387	0.08761	0.25963	-0.03048	0.12335	0.21544	-0.07306	0.06225	0.00441	0.07064	-0.10695	0.12708	0.03605	-0.06004
0.09991	0.10644	-0.15413	0.23064	-0.02809	-0.04724	0.22531	-0.06456	0.24811	0.21248	-0.14312	0.05218	0.01779	0.0646	-0.05859	0.10299	0.03334	-0.04712
0.14044	0.10671	-0.24502	0.2322	-0.04411	-0.04509	0.21205	0.01965	0.10464	0.21322	-0.13555	0.09059	0.02154	0.05554	-0.18927	0.14149	-0.02551	-0.07882
-0.05785	0.10134	0.00229	0.21449	0.15149	0.05284	0.16114	0.00702	0.24189	0.10071	0.06543	0.17198	0.09664	0.06409	0.15179	0.12391	0.0929	-0.06181
0.03734	0.11331	-0.27162	0.23439	0.09831	-0.05939	0.15278	-0.01303	0.22098	0.12192	-0.10964	0.09553	0.10501	0.05703	-0.12174	0.02985	0.05039	-0.14799
-0.08168	0.15382	-0.31918	0.18818	0.11537	-0.06671	0.15025	-0.00453	0.25382	-0.06004	-0.01726	0.1356	0.0731	0.06066	0.10979	0.07109	0.13599	0.0089
-0.00141	0.1185	-0.03541	0.23698	-0.09982	0.06831	0.22647	-0.06094	0.13067	0.22863	0.01372	0.13777	0.04711	0.05401	0.00474	0.11881	0.04575	-0.04608
-0.0283	0.11891	-0.25753	0.21888	0.04811	0.16347	0.23211	-0.02316	0.20514	0.16915	0.00304	0.16858	0.07224	0.04728	0.04649	0.13565	0.07103	-0.09474
-0.07391	0.12105	-0.40092	0.17589	0.08275	0.03204	0.12773	0.00385	0.28091	0.12725	0.07031	0.13696	0.1231	0.05054	0.09894	0.00272	0.18088	-0.18284
-0.00375	0.11274	-0.21032	0.23407	-0.09781	-0.08992	0.19947	-0.06572	0.1016	0.19388	-0.11013	0.05712	0.03009	0.07079	0.00903	-0.05029	0.13787	-0.05402
0.08162	0.09956	-0.23974	0.21184	-0.02441	-0.06545	0.23173	-0.03995	0.25711	0.19622	-0.04798	0.04623	0.0485	0.05962	0.00338	0.07736	0.03524	-0.07595

ID_BY_MIR	RTO_SIN	ELL_DIFF	MA_SIGN	RTICOID	COID_RE	RIPLE_NE	UCURON	THIONE_M	AND_GLY	OLIPID_B	NTHESIS_	AND_GL	SIS_IN_S	OGLYCA	AN_SYNT	INGOLIPI	RELATE
0.17153	0.176	0.16223	-0.01037	0.19831	0.03192	0.27789	0.2498	0.16068	0.03162	-0.03279	0.18352	0.33047	0.34302	0.0831	-0.10015	-0.21994	0.02951
0.16693	0.12853	0.33636	-0.03212	0.10374	-0.00459	0.25327	0.26122	0.12857	-0.03654	0.02901	0.12101	0.31603	0.32823	0.02845	-0.16723	-0.22276	0.07268
0.17035	0.15965	0.31693	0.00359	0.12009	0.0274	0.28336	0.29851	0.15838	0.03745	0.04601	0.16659	0.31501	0.30017	0.08974	0.06074	-0.17206	-0.06105
0.18166	0.20175	0.3783	-0.00538	0.1035	0.02704	0.23618	0.21945	0.17661	0.01718	0.08722	0.16891	0.32272	0.33188	0.12616	-0.17281	-0.01111	0.0034
0.18862	0.20002	0.3276	0.02818	0.21223	0.01837	0.22401	0.20701	0.15161	0.02865	0.04798	0.16381	0.32542	0.32481	0.13957	0.0366	-0.16142	0.00632
0.18408	0.10267	0.34895	-0.01703	0.20413	0.00585	0.24641	0.21951	0.16175	-0.1067	-0.04787	0.16169	0.29426	0.31195	0.09589	-0.0228	-0.24026	0.00643
0.12476	0.18016	0.33693	0.01269	0.13481	0.00678	0.25928	0.19742	0.16166	-0.003	0.00098	0.13164	0.31491	0.30848	0.11602	-0.1208	-0.24352	0.033
0.17706	0.2206	0.36198	-0.01248	0.13577	0.02873	0.22511	0.28846	0.16024	0.05561	0.13359	0.17485	0.31497	0.31742	0.1397	0.12126	-0.00667	0.0307
0.17682	0.2045	0.32239	0.00326	0.13855	0.04249	0.23673	0.21017	0.20246	-0.03032	0.09532	0.16921	0.3128	0.32796	0.17219	-0.05009	0.10062	-0.00484
0.16997	0.20723	0.38047	0.01393	0.12072	0.0497	0.22726	0.22069	0.19206	-0.03307	0.06482	0.15287	0.30305	0.31677	0.1499	-0.16978	-0.04911	-0.00239
0.12821	0.14452	0.176	-0.01436	0.15431	0.06245	0.29786	0.27148	0.18769	0.00114	0.00298	0.1648	0.3407	0.32485	-0.01912	-0.06844	-0.39611	0.0688
0.1842	0.12512	0.33771	-0.01048	0.13893	0.01919	0.27462	0.25148	0.19458	-0.03015	0.01929	0.14006	0.3108	0.30978	0.12914	-0.06355	-0.25395	0.02553
0.22807	0.07223	0.229	0.01011	0.13404	-0.03038	0.27889	0.30302	0.09708	0.0012	-0.08036	0.16934	0.29857	0.29594	-0.07735	-0.07171	-0.32027	0.02241
0.17525	0.17367	0.1654	-0.03071	0.11993	0.04381	0.27868	0.25888	0.16397	0.0328	0.06943	0.1801	0.32084	0.31306	0.19946	-0.03696	-0.02555	0.06444
0.17258	0.17477	0.31685	-0.03405	0.12417	0.03421	0.2743	0.3065	0.16441	0.07967	0.07878	0.18605	0.31096	0.33364	0.09823	0.01713	-0.10424	0.02618
0.07758	0.08792	0.325	-0.01814	0.14821	-0.04474	0.32521	0.27205	0.18571	0.09553	-0.00403	0.1622	0.32259	0.31784	0.04185	-0.09776	-0.24416	-0.03541
0.18198	0.16167	0.39053	0.00446	0.09726	0.02066	0.25886	0.24098	0.13305	0.07965	0.08812	0.15779	0.32651	0.33346	0.17879	-0.20237	-0.05997	-0.03306
0.1851	0.20224	0.37422	-0.01143	0.15996	0.0061	0.25283	0.30671	0.17113	-0.02152	0.07151	0.15914	0.32378	0.31368	0.13476	-0.14491	-0.05766	-0.00716

YLATE_M	ASS_A_R	ASS_B_S	BOTROPI	GPCRS_O	VD_RETIN	PR40_PAT	RMONES	_SIGNAL	ALING_A	NENT_CYT	PMENT_O	SQUAM	ORT_DEVE	GNALING	SIGNALIN	STEM_CE	REGULA
0.24235	-0.0395	-0.30601	-0.22852	-0.22598	0.0235	-0.0286	-0.03079	0.12416	0.10124	-0.00239	0.20683	-0.0287	0.04886	0.05307	0.09136	-0.16309	-0.14293
0.25777	-0.06793	-0.25801	-0.37165	-0.24201	-0.00827	-0.0595	-0.03026	0.11156	0.10575	0.00267	0.18962	-0.03563	0.11536	0.02227	0.02353	-0.23312	-0.06192
0.25525	0.01328	-0.27803	-0.20028	-0.34468	0.03588	-0.04405	-0.00616	0.10741	0.12223	-0.01656	0.19199	-0.01632	0.06673	0.06358	-0.02441	-0.09259	-0.16503
0.20877	0.01873	-0.16307	-0.18121	-0.24621	0.10528	-0.01174	-0.00989	0.1683	0.085	0.0017	0.14647	-0.03267	0.01425	0.07962	-0.00737	-0.1294	-0.18071
0.20341	0.01418	-0.12776	-0.35857	-0.23667	0.08714	0.00817	0.00031	0.17107	0.01549	-0.02394	0.07947	-0.03376	0.03738	0.10269	0.0114	-0.083	-0.13807
0.16822	-0.0256	-0.28702	-0.25008	-0.28312	0.02585	-0.08461	-0.07961	0.10121	0.09667	-0.01213	0.14057	-0.06138	0.03477	0.01941	0.19724	-0.0762	-0.1359
0.17956	0.05227	-0.34101	-0.2284	-0.37447	0.0849	-0.0804	-0.02698	0.13295	0.09709	-0.03539	0.18745	-0.02406	0.04958	0.06502	0.07691	-0.10666	-0.10499
0.29864	0.03542	-0.02177	-0.32065	-0.14414	0.1265	-0.02646	-0.04217	0.14535	0.04878	-0.05327	0.1039	-0.0579	0.00813	0.12122	-0.0423	-0.12157	-0.18531
0.20321	0.07045	-0.09571	-0.38443	-0.22497	0.11686	0.00137	-0.05035	0.12674	0.05754	-0.02738	0.14843	-0.0422	-0.00443	0.10742	-0.01308	-0.08522	-0.16388
0.16752	-0.00774	-0.12847	-0.47847	-0.26484	0.11742	-0.03558	-0.0516	0.1435	0.01979	-0.03347	0.09554	-0.07962	-0.01671	0.10576	0.04205	-0.07208	-0.14268
0.20647	-0.0553	-0.36524	-0.02604	-0.24683	0.0294	-0.06277	-0.00515	0.10474	0.14662	0.04646	0.20538	-0.00164	0.08438	0.01407	-0.03424	-0.17364	-0.07342
0.23056	-0.05767	-0.32996	-0.18599	-0.31932	0.06606	-0.03055	-0.05457	0.12948	0.08923	-0.03488	0.15066	-0.04754	0.01744	0.09777	0.0013	-0.19039	-0.1254
0.19298	-0.04795	-0.22391	-0.05492	-0.14652	0.01104	-0.10775	0.05495	0.18502	0.12611	-0.00399	0.18384	0.01694	0.12708	0.04575	-0.01453	-0.1787	-0.08473
0.19468	-0.03055	-0.24924	-0.02979	-0.29621	0.06485	-0.06051	-0.023	0.12796	0.09991	-0.00809	0.16131	-0.05496	0.03161	0.1136	0.00606	-0.14873	-0.12151
0.23582	-0.02215	-0.35442	-0.21886	-0.30577	0.02193	-0.0723	-0.06589	0.11835	0.12426	-0.00436	0.13912	-0.0585	0.03838	0.06856	-0.05575	-0.14792	-0.15824
0.21653	-0.06398	-0.33187	-0.32062	-0.28847	-0.01967	-0.10019	-0.01147	0.13221	0.11951	0.0335	0.20546	-0.0114	0.09335	0.06835	-0.00731	-0.22655	-0.11689
0.2157	-0.03292	-0.15625	-0.10335	-0.22041	0.07003	-0.04101	-0.01993	0.16977	0.09013	-0.03401	0.17936	-0.01777	-0.00247	0.0834	0.03074	-0.11785	-0.16793
0.16751	0.02718	-0.17791	-0.2033	-0.3155	0.07903	-0.01354	-0.02415	0.13982	0.10308	-0.016	0.15688	-0.04043	0.05083	0.08631	-0.02331	-0.08909	-0.16907

DEFECT	TE_BIOSY	TITIS_B_I	HEPATOC	H_FACTO	S_AND_R	S_REGUL	QUITINAT	PTOGENE	SIGNALI	NG_REGI	O_YAP_SI	NE_MODI	GOUS_RE	N_OF_HU	N_OF_HU	IUMAN_C	OF_HUMA	RTROPH
0.03261	0.03261	0.02967	0.06447	0.1732	0.14545	0.47024	0.13132	0.15285	0.06595	0.05879	-0.09812	0.39108	-0.07449	-0.04042	-0.0852	-0.00059	0.08054	0.17224
0.06716	0.06716	0.02178	0.08036	0.11119	0.11859	0.47166	0.13541	0.18126	0.04643	0.06641	-0.15359	0.38698	-0.02808	-0.01558	-0.1672	0.00789	0.04914	0.0877
0.08427	0.08427	0.02112	0.04499	0.14211	0.09612	0.48073	0.16631	0.17926	0.07067	0.06472	-0.12439	0.38978	0.00017	-0.01143	-0.16982	0.03501	0.04314	0.18053
0.03111	0.03111	0.04303	0.0402	0.12113	0.0988	0.45113	0.12453	0.14776	0.04426	0.07183	-0.18881	0.38974	0.00125	-0.0118	-0.05239	0.04617	0.05367	0.18411
0.086	0.086	0.07887	0.07556	0.12709	0.09626	0.46383	0.1816	0.18794	0.02984	0.06711	-0.14077	0.3914	-0.05994	0.01186	-0.103	0.0417	0.05686	0.11995
0.12776	0.12776	0.01583	0.05963	0.10887	0.13228	0.48306	0.18174	0.17668	0.03886	0.07805	-0.09843	0.38277	0.02482	-0.05635	-0.24772	0.00104	0.04205	0.10879
-0.02136	-0.02136	0.05303	0.07994	0.14736	0.10363	0.44946	0.15679	0.15901	0.05906	0.07498	-0.11295	0.38442	0.03187	-0.00056	-0.15657	0.04951	0.08017	0.15812
0.08457	0.08457	0.05162	0.04278	0.16066	0.10824	0.47372	0.10972	0.16647	0.02668	0.06263	-0.17647	0.3914	-0.02313	-0.02951	-0.07542	0.07147	0.0555	0.1691
0.04436	0.04436	0.06085	0.11513	0.13652	0.11352	0.46617	0.0818	0.22226	0.02959	0.0538	-0.13471	0.38756	-0.00131	0.01042	-0.05196	0.04795	0.05831	0.18232
0.04726	0.04726	0.06487	0.05678	0.14822	0.09278	0.43033	0.1407	0.17956	0.02825	0.06119	-0.14829	0.38858	0.03682	0.02352	-0.07483	0.09957	0.07795	0.157
0.00475	0.00475	0.0137	0.04693	0.11094	0.14028	0.48118	0.20762	0.2088	0.05725	0.07897	-0.11711	0.3905	-0.03021	-0.00668	-0.07928	-0.07459	-0.00464	0.13156
0.0847	0.0847	0.02618	0.04084	0.12481	0.10409	0.43484	0.14531	0.19497	0.04948	0.07379	-0.08197	0.38388	-0.00197	-0.03705	-0.13521	0.03229	0.05365	0.15385
0.00324	0.00324	0.01898	0.03888	0.13832	0.16498	0.48535	0.20494	0.13622	0.07666	0.11277	-0.07036	0.37498	0.02605	0.01346	-0.08522	-0.07311	0.0896	0.15899
0.06417	0.06417	0.02847	0.04736	0.12804	0.09766	0.46495	0.15223	0.17392	0.04898	0.07164	-0.10637	0.40057	-0.0582	-0.01533	-0.09622	0.01274	0.01971	0.1493
0.00849	0.00849	0.02275	0.04749	0.14326	0.10503	0.45077	0.10447	0.15836	0.0326	0.08543	-0.11876	0.39158	0.00659	-0.03335	-0.09262	0.02759	0.06476	0.14506
-0.00772	-0.00772	0.02508	0.0548	0.13614	0.12525	0.47487	0.11939	0.16273	0.05952	0.09918	-0.00391	0.3855	0.11633	0.03664	-0.0895	-0.00766	0.04067	0.16032
0.06396	0.06396	0.02476	0.03227	0.12733	0.10682	0.46998	0.09047	0.14405	0.04425	0.07478	-0.16254	0.39123	-0.01551	-0.04947	-0.10314	-0.04054	0.04973	0.15385
0.02858	0.02858	0.04663	0.05278	0.1404	0.08266	0.47084	0.15866	0.17949	0.05806	0.05477	-0.14678	0.39034	-0.01966	-0.00569	-0.08789	0.07362	0.05591	0.16282

ATHOGEN	IOFACIAL
0.07305	-0.00312
0.07742	-0.05084
0.0887	-0.01248
0.07472	-0.03326
0.073	0.12758
0.05486	0.00551
0.11084	-0.0295
0.07401	-0.01851
0.074	-0.01092
0.06799	-0.02301
0.09421	0.02943
0.12322	0.05076
0.14691	0.08283
0.08181	-0.03441
0.08314	-0.04358
0.08716	-0.00763
0.06235	-0.04587
0.06804	0.04942

MMATORY	SIGNALIN	SIGNALIN	NALING_I	IGNALIN	IGNALIN	IGNALIN	IGNALIN	IGNALIN	IGNALIN	IGNALIN	IGNALIN	IGNALIN	CHRONIC_	TION_IN_
0.30297	0.05482	0.09114	0.17085	0.01404	0.14258	0.19995	0.08963	0.06445	0.01407	0.06585	0.12507	0.20985	-0.11695	0.19201
0.26915	0.01348	0.06136	0.12769	-0.02316	0.15039	0.17541	0.04456	0.02114	-0.02356	0.02438	0.05342	0.14045	-0.07222	0.18099
0.33	0.01461	0.09828	0.15925	-0.00873	0.1482	0.20023	0.09381	0.0757	0.02905	0.07153	0.09554	0.17829	-0.06701	0.19895
0.36358	0.03127	0.10694	0.19432	0.00738	0.14686	0.18184	0.08972	0.11018	0.05009	0.09295	0.10642	0.20221	-0.16787	0.20151
0.35337	0.0686	0.12779	0.20006	0.03354	0.14062	0.21442	0.11154	0.12071	0.07229	0.09684	0.12184	0.21767	-0.09572	0.18837
0.29185	-0.03757	0.08278	0.12549	-0.02072	0.1182	0.12101	0.07179	0.04494	0.00623	0.02214	0.07528	0.1591	-0.07202	0.0606
0.31306	0.01181	0.12661	0.17586	0.01332	0.14447	0.20269	0.11121	0.09049	0.0382	0.05957	0.1235	0.22727	-0.03432	0.20741
0.39529	0.07278	0.10976	0.21896	0.02405	0.14453	0.20881	0.12317	0.14404	0.08467	0.10971	0.13734	0.22954	-0.17119	0.19612
0.3838	0.0463	0.11598	0.21684	0.03987	0.14708	0.24036	0.12237	0.14495	0.08257	0.10372	0.13656	0.21927	-0.15856	0.25407
0.38499	0.0645	0.11686	0.21261	0.02936	0.14495	0.21813	0.13699	0.13371	0.08293	0.10649	0.13475	0.22476	-0.08408	0.23225
0.19717	-0.01601	0.08677	0.12097	-0.02486	0.15538	0.19696	0.04134	0.03337	-0.03067	-0.00503	0.05759	0.1335	-0.08914	0.15876
0.29636	0.01194	0.07362	0.20133	-0.00809	0.14745	0.20582	0.09949	0.06165	0.00637	0.02901	0.1077	0.1687	-0.11799	0.15533
0.21411	-0.02392	0.05873	0.12418	-0.02548	0.12814	0.1608	0.06952	0.01327	-0.03831	0.04934	0.07891	0.13498	-0.11303	0.07904
0.30896	0.06245	0.09996	0.16843	-0.00965	0.13199	0.2031	0.05844	0.0712	0.03096	0.03422	0.06836	0.14755	-0.11854	0.20626
0.28251	0.03864	0.10058	0.14707	0.01381	0.14166	0.1941	0.0764	0.06199	0.01671	0.04775	0.09295	0.17398	-0.0989	0.15954
0.20239	0.01514	0.07549	0.13342	-0.04165	0.1372	0.17323	0.05133	-0.00417	-0.04721	0.0144	0.09105	0.13085	-0.09884	0.15914
0.31107	0.00105	0.10493	0.13483	-0.00987	0.1344	0.18317	0.0662	0.07809	0.02252	0.0641	0.08187	0.18673	-0.15162	0.19139
0.37036	0.06361	0.12411	0.21441	0.02311	0.1511	0.18265	0.13424	0.13071	0.07422	0.09639	0.14731	0.23054	-0.15518	0.12907

PONSE_T	ION_BOD	RECEPT	BOWEL_	DRY_RESP	NOPATH	AND_SEC	D_TRANS	ULIN_SIG	ADIPOC	ADIPOC	ED_CANC	EDIATED	S_AND_M	OXL4_AN
0.11524	0.04433	-0.06742	0.08054	-0.00268	0.12806	0.24093	-0.0017	0.05465	-0.01432	-0.01432	-0.06463	0.1074	-0.0586	0.09704
0.11414	0.07155	-0.18804	0.04439	0.00778	0.1076	0.18252	0.0378	0.01431	-0.01768	-0.01768	-0.00345	0.05623	-0.10114	0.11245
0.18394	0.04173	-0.10716	0.09102	-0.01752	0.12015	0.15806	-0.03937	0.04159	-0.02588	-0.02588	0.02689	0.10837	-0.08734	0.11266
0.18837	0.02043	-0.12228	0.09876	0.09112	0.12575	0.22345	-0.05477	0.05239	0.04467	0.04467	-0.01009	0.10343	-0.07964	0.09845
0.14804	0.03637	-0.0151	0.1385	0.02924	0.04731	0.26702	0.01179	0.04586	-0.02668	-0.02668	-0.00039	0.09183	-0.01402	0.12141
0.10723	0.07671	-0.12324	0.07636	0.07137	0.09483	0.14523	0.02956	0.0128	-0.01874	-0.01874	-0.00782	0.07124	-0.08079	0.10498
0.21023	0.11282	-0.06892	0.10347	0.11835	0.05283	0.13842	-0.01555	0.03777	-0.01649	-0.01649	0.01	0.11381	-0.0464	0.10519
0.18482	0.05186	-0.05878	0.15336	0.08112	0.08443	0.22147	-0.04104	0.05103	-0.03778	-0.03778	-0.04775	0.11243	-0.0166	0.09236
0.19169	0.05804	-0.0747	0.16127	0.08965	0.07892	0.18255	-0.0616	0.04665	-0.03668	-0.03668	-0.01131	0.11471	0.07367	0.10877
0.26469	0.06737	-0.02499	0.13781	0.08645	0.05075	0.19532	-0.01922	0.04733	-0.05301	-0.05301	-0.00063	0.11229	0.02726	0.1204
0.06678	0.03305	-0.14223	0.07945	0.0051	0.10555	0.18177	-0.01946	0.03555	-0.03432	-0.03432	0.02758	0.08164	-0.08619	0.09246
0.19154	-3.8E-05	-0.06658	0.08582	0.06213	0.03162	0.17994	-0.07083	0.01876	-0.06029	-0.06029	-0.00071	0.08099	-0.06835	0.10749
0.07835	0.05097	-0.15703	0.05356	0.01266	0.09699	0.25887	0.01692	0.06677	0.02559	0.02559	0.07728	0.09633	-0.14554	0.08698
0.0912	0.00379	-0.10671	0.10033	0.03781	0.12282	0.20319	-0.03278	0.04954	-0.00944	-0.00944	-0.04137	0.09201	-0.06109	0.11461
0.14724	0.0334	-0.08576	0.06025	0.03377	0.08113	0.22172	-0.03001	0.03467	-0.05059	-0.05059	-0.02299	0.08303	-0.09249	0.10992
0.13666	-0.02717	-0.05571	0.03095	0.03254	0.11507	0.19371	-0.01979	0.03391	0.02877	0.02877	0.05151	0.07191	-0.09629	0.06465
0.05681	0.07182	-0.16604	0.06835	0.05585	0.11611	0.22213	-0.05322	0.04178	0.01683	0.01683	-0.03818	0.09917	-0.10301	0.11206
0.21708	0.05481	-0.04709	0.13731	0.06273	0.06951	0.19423	-0.0169	0.04488	-0.04467	-0.04467	-0.02303	0.11282	-0.01254	0.11884

KILLER_	OMB_RE	YPE_I_SIG	N_11_SIG	IL_1_STR	DUCED_	PROTEIN	PORT_PR	ASE_IN_N	OTECAN_	ABOLISM	ABOLISM	IR_CLUS	N_THE_RE	BERT_SY
-0.01666	0.04156	0.08344	0.1014	0.02355	0.20256	0.16315	0.08978	0.01136	-0.41837	0.24136	0.13946	0.03354	-0.01538	0.03309
-0.09557	0.06586	0.0567	0.07612	-0.0022	0.15552	0.15082	0.13619	0.06536	-0.33791	0.19964	0.14112	0.0043	-0.03475	0.05214
-0.03701	0.05927	0.09262	0.09097	0.01968	0.10381	0.0828	0.1009	0.07308	-0.29301	0.23503	0.22394	0.00155	-0.03067	0.01849
-0.01912	0.04881	0.09993	0.09479	0.01886	0.14798	0.08866	0.11541	0.08145	-0.06507	0.24617	0.20096	0.00011	0.01236	0.0775
-0.06831	0.04732	0.13068	0.09295	0.04577	0.21179	0.1075	0.13177	0.09836	-0.30511	0.17987	0.08158	-0.02342	0.02153	0.04295
-0.02275	0.04351	0.10762	0.04634	-0.00875	0.13943	0.10781	0.06868	0.11233	-0.03794	0.16592	0.1304	-0.00361	-0.03724	0.08126
-0.00822	0.03448	0.12783	0.09391	0.04535	0.16864	0.08476	0.0622	0.06617	-0.22365	0.20852	0.11746	-0.00249	-0.02269	0.06296
-0.0335	0.0191	0.12507	0.12037	0.04998	0.14099	0.09962	0.12623	0.1327	-0.14152	0.25138	0.21634	0.00622	-0.0103	0.05293
0.00034	0.03291	0.13054	0.10932	0.02688	0.13573	0.08943	0.11221	0.09747	-0.1279	0.24127	0.13718	-0.02197	-0.01086	0.07787
0.0229	0.06214	0.14699	0.10728	0.05355	0.16412	0.07026	0.10175	0.0496	-0.06653	0.21763	0.11235	0.02148	-0.00718	0.05283
-0.12871	0.02159	0.07159	0.06067	-0.02767	0.1722	0.11175	0.14824	0.01113	-0.18518	0.25868	0.16066	-0.03434	-0.06832	0.05404
-0.13064	0.04515	0.08867	0.07538	0.01074	0.18683	0.11649	0.09149	-0.01569	-0.15295	0.24959	0.15271	0.05718	-0.04153	0.08026
-0.13934	0.07441	0.0457	0.07082	0.00165	0.09502	0.20248	0.18822	0.04104	-0.41005	0.14303	0.18817	-0.04869	0.02228	0.102
-0.0289	0.03629	0.0913	0.06711	-0.0042	0.1305	0.11683	0.14412	0.07884	-0.37614	0.20767	0.12673	-0.03493	-0.00508	0.06386
-0.0771	0.03315	0.07999	0.07388	0.04971	0.18764	0.08402	0.12338	0.04058	-0.30437	0.23185	0.18365	-0.00419	-0.07035	0.06971
-0.10392	0.02412	0.03298	0.07143	0.01677	0.11901	0.13471	0.07899	0.00201	-0.3156	0.26728	0.18941	0.07561	-0.00786	0.0566
-0.06511	0.05274	0.10114	0.08178	0.01476	0.12826	0.12532	0.11291	0.07484	-0.25447	0.24464	0.14787	0.02021	0.01638	0.07098
-0.02042	0.05034	0.12958	0.11542	0.039	0.15334	0.08951	0.14161	0.08483	-0.1211	0.23097	0.13875	-0.01244	0.0023	0.03433

MANN_SY	WAY_FR	ESIS_AN	SYNTHES	ALLIKRE	N_RECEP	TOR_SIGN	FSTRA_S	_CYCLE_	AND_LIN	HUTTLE_I	WHAT_W	NCE_ON	AND_AD	IN_SIGN
0.1828	0.08899	0.16215	0.11102	-0.04016	0.04051	0.06754	0.04113	0.27277	-0.14325	0.2637	0.00069	0.07623	0.03156	-0.06881
0.15038	0.0768	0.11178	0.09879	0.06169	-0.00516	0.03042	0.10597	0.19616	-0.16059	0.23666	0.012	0.04744	-0.0414	-0.13489
0.20587	0.12286	0.15668	0.09222	0.06673	0.05269	0.07507	0.05509	0.25359	-0.19513	0.24948	-0.10189	0.05321	-0.05294	-0.14054
0.16461	0.13265	0.14271	0.13543	0.20946	0.07614	0.09247	-0.0532	0.23837	-0.19876	0.25418	-0.10971	0.04997	0.02017	-0.10186
0.19744	0.1008	0.17187	0.17233	0.10885	0.11294	0.10427	0.05754	0.2202	-0.17181	0.27151	-0.09161	0.08324	-0.0008	-0.1237
0.1624	0.09553	0.14563	0.02362	0.37689	-0.03102	0.04955	0.05891	0.20207	-0.23687	0.24914	-0.14489	0.04806	0.06996	-0.13818
0.16558	0.09083	0.12867	0.13505	0.31144	0.07915	0.08205	0.01481	0.21264	-0.29064	0.24381	-0.05031	0.0647	0.0241	-0.13841
0.19977	0.14301	0.15781	0.10208	0.24322	0.0949	0.12631	-0.03375	0.20586	-0.24299	0.27847	-0.09491	0.05703	0.033	-0.11022
0.18102	0.10947	0.08781	0.01971	0.24058	0.08256	0.12594	-0.00933	0.18603	-0.21517	0.26675	-0.05492	0.13425	0.05112	-0.09868
0.2021	0.08745	0.12642	0.10667	0.24164	0.09556	0.12689	-0.03854	0.21786	-0.22386	0.24439	-0.11592	0.10661	0.02813	-0.11528
0.07229	0.12266	0.21925	0.29911	0.08568	-0.0157	0.01981	0.00541	0.25791	-0.30672	0.2142	0.03186	0.02343	0.03407	-0.14911
0.17109	0.09889	0.19181	0.21407	0.21906	0.00447	0.04835	0.0314	0.25476	-0.30703	0.24637	-0.06327	0.07975	-0.01409	-0.09683
0.16058	0.08492	0.15861	0.19944	0.12291	0.02539	0.02913	0.10144	0.19846	-0.20722	0.24602	0.01212	-0.04263	0.05792	-0.09513
0.14499	0.12412	0.14452	0.19046	0.09808	0.07069	0.05467	0.01906	0.24094	-0.09344	0.25362	-0.11457	0.06214	-0.02651	-0.11454
0.18965	0.13278	0.17695	0.16441	0.15961	0.01986	0.06521	0.03801	0.24032	-0.15391	0.23603	-0.04497	0.02426	0.03873	-0.10009
0.15527	0.15519	0.16682	0.2062	-0.04168	0.03347	0.0055	0.0455	0.29002	-0.20925	0.20126	-0.01327	0.02694	0.00717	-0.13742
0.1473	0.15598	0.16423	0.16742	0.09339	0.06141	0.06291	0.06362	0.25432	-0.24084	0.26038	-0.09724	0.03763	-0.02239	-0.14349
0.20401	0.10748	0.17509	0.10697	0.11252	0.05986	0.10977	-0.00443	0.25052	-0.18314	0.29407	-0.10494	0.08118	-0.06878	-0.08204

SIGNALI	CINE_AND	SIC_HIPP	NE_META	LISM_IN_	ETABOLI	RTICLES_	RECEPT	T_SIGNAL	ANISMS_	GNAL_R	ING_FIB	OPHAGE_	LE_INFE	N_CARD
0.08335	0.12191	0.11891	0.1891	0.06768	0.0555	0.18726	0.35124	0.02823	-0.2171	0.13751	-0.01226	0.31537	0.04899	0.23832
0.04697	0.07306	0.08547	0.18313	-0.09021	0.08674	0.13393	0.31231	0.06892	-0.13051	-0.02389	-0.01092	0.26491	0.03843	-0.10102
0.0824	0.12947	0.09574	0.12375	0.09902	0.06939	0.20644	0.33821	0.08033	-0.17846	0.12453	0.00832	0.33358	0.06604	0.0709
0.0722	0.01628	0.1185	0.16937	0.07667	0.06519	0.303	0.28378	0.02801	-0.3486	0.11931	0.02232	0.36955	0.06164	0.03129
0.08755	0.04775	0.14552	0.16929	0.06094	0.08494	0.2603	0.24643	0.01966	-0.20101	0.16578	0.00643	0.37666	0.05617	0.21596
0.05826	0.06342	0.14139	0.12603	0.14164	0.09904	0.40095	0.37414	0.07621	-0.24118	0.05515	0.00425	0.27347	0.10047	-0.01961
0.08426	0.0394	0.12391	0.15798	0.12751	0.0662	0.29269	0.41924	0.06233	-0.14276	0.09756	0.03468	0.34627	0.06259	-0.07299
0.10326	0.06697	0.14283	0.14906	-0.03857	0.09859	0.36136	0.30386	-0.0013	-0.26785	0.17462	0.03234	0.37137	0.05599	0.13152
0.09232	0.01711	0.11825	0.13809	0.05917	0.08637	0.36089	0.26281	0.0198	-0.25749	0.20922	0.07808	0.37705	0.05449	0.11376
0.10067	0.04433	0.13916	0.15507	0.08253	0.09685	0.38074	0.3257	-0.00397	-0.41631	0.17365	0.01486	0.37805	0.06585	0.10476
0.06813	0.15515	0.0783	0.14645	-0.09424	0.05309	0.21405	0.18143	0.11311	0.0197	0.08401	-0.00246	0.25144	0.08611	0.02644
0.06395	0.11361	0.14071	0.07722	-0.0611	0.10228	0.30523	0.33275	0.05492	-0.0734	0.12143	-0.05355	0.34219	0.06365	-0.07276
0.08188	0.10597	0.0963	-0.04769	-0.09294	0.0983	0.31364	0.38277	0.13216	-0.03683	-0.09126	-0.08252	0.06547	0.0594	0.02664
0.07417	0.11111	0.13804	0.16872	0.01372	0.06613	0.22037	0.35177	0.04137	-0.21012	0.13031	0.02662	0.37735	0.04257	0.06289
0.07851	0.08123	0.10751	0.18757	0.04572	0.09247	0.31112	0.38172	0.04566	-0.28447	0.12473	0.01174	0.33255	0.06842	0.11217
0.06079	0.13222	0.10802	0.08569	-0.04907	0.07615	0.1338	0.39369	0.11245	-0.02513	-0.00595	-0.07628	0.13812	0.0564	-0.12281
0.07155	0.12745	0.12675	0.15979	0.09962	0.09569	0.28054	0.36516	0.06412	-0.31157	0.09741	0.0234	0.37575	0.06921	0.04564
0.08823	0.05423	0.15725	0.14305	0.03066	0.06602	0.26622	0.20733	0.02739	-0.15614	0.13987	-0.01245	0.37936	0.02651	0.14695

DER_OF_	THWAY_E	ENT_PAT	HWAY_PR	MENT_PA	AND_BL	PATHWA	APK_CAS	CONGEN	SIGNALI	F_KIDNE	METALLO	RULATION	S_VIRUS	OF_YAP_
0.11742	-0.04654	0.15473	0.06031	0.23792	0.21736	0.04022	0.11926	0.05617	0.09631	-0.02871	0.02628	0.12748	0.03244	0.15456
0.15111	-0.06356	0.17919	-0.029	0.12961	0.14852	-0.00241	0.08746	-0.02194	0.06256	0.07334	-0.05032	0.10076	0.04187	0.12758
0.12689	0.01042	0.17505	-0.06074	0.17432	0.24104	-0.00264	0.12181	0.01766	0.08472	0.01573	-0.03721	0.15024	0.06182	0.15409
0.05344	-0.02351	0.16482	-0.02745	0.20632	0.19083	0.02996	0.13155	-0.01394	0.08746	-0.02247	0.02834	0.17433	0.06134	0.14252
0.0995	-0.10318	0.17809	-0.04437	0.2397	0.20298	0.12045	0.17102	0.05394	0.11197	-0.0456	0.07462	0.17136	0.07584	0.11516
-0.06781	-0.02031	0.10872	-0.03029	0.24818	0.17396	-0.10475	0.06736	-0.08969	0.07064	-0.0745	-0.03132	0.13663	0.05797	0.13784
0.07474	-0.00731	0.17022	-0.02331	0.27229	0.21177	-0.02975	0.11112	-0.02219	0.08425	-0.0179	0.13912	0.13678	0.08259	0.15031
-0.218	-0.03551	0.204	-0.03615	0.24797	0.17483	0.0592	0.12221	-0.02379	0.10036	0.02433	0.10921	0.19395	0.06978	0.12978
-0.13312	-0.02528	0.16282	-0.01731	0.21219	0.1966	0.01985	0.10694	-0.05028	0.10206	-0.05927	0.02897	0.20051	0.08188	0.13187
-0.28274	-0.0119	0.19477	-0.05791	0.22025	0.14346	0.04676	0.1227	-0.02581	0.10709	-0.07997	0.05368	0.15724	0.10893	0.12087
0.14694	-0.03106	0.21662	0.01141	0.18052	0.22638	-0.06548	0.02919	-0.06111	0.06863	0.05254	0.09102	0.15613	0.04917	0.14664
-0.09576	0.00771	0.17704	-0.00043	0.23443	0.19862	-0.00079	0.10658	-0.05199	0.08517	-0.00734	0.03733	0.13494	0.06532	0.15821
0.09252	0.01175	0.1661	0.0229	0.17313	0.23325	-0.01939	0.12405	0.1019	0.07153	0.00429	-0.07213	0.17066	0.03543	0.18252
0.01933	-0.0023	0.1607	-0.00911	0.18219	0.18781	0.05484	0.09172	-0.00083	0.0905	-0.02754	0.0151	0.13902	0.0407	0.14667
0.04506	-0.08914	0.19739	-0.04243	0.20658	0.22987	0.00731	0.10762	0.02064	0.0856	-0.00525	0.11703	0.12384	0.05392	0.13842
0.12187	-0.00452	0.13367	0.04416	0.14838	0.2112	-0.01467	0.13032	0.0246	0.06638	-0.01648	-0.00435	0.08111	0.04409	0.17025
0.1299	-0.04091	0.17799	-0.0309	0.19965	0.1703	-0.00678	0.12749	0.009	0.0745	0.01361	-0.01474	0.17135	0.02321	0.13265
-0.03499	-0.02055	0.11215	-0.03753	0.2233	0.22918	0.03926	0.14409	-0.01297	0.10047	0.05738	-0.11659	0.20532	0.0772	0.16071

SSOCIATE	_MELANO	METABOLI	L_COMM	_EPILEP	ATHWAYS	DL_HDL	GRAMMI	RAMMING	OF_ALPH	OLIPIDS_	RANSF	ORTATIC_BR	NOVO_AN	SULFU
0.11647	0.11695	0.11069	-0.01406	0.24094	0.18059	0.21831	0.32204	0.20792	0.01577	-0.14586	0.03705	0.00736	0.17149	0.24941
0.15992	0.10269	0.01238	0.03988	0.22769	0.16652	0.1895	0.30732	0.1953	0.07014	-0.12559	-0.01896	-0.0422	0.14537	0.19574
0.142	0.13011	0.02126	0.02725	0.22966	0.23077	0.22433	0.34205	0.24493	-0.04337	-0.12865	0.02501	0.03793	0.1668	0.19833
0.13025	0.12426	-0.00641	-0.01519	0.21887	0.16011	0.28793	0.29525	0.18249	0.04364	-0.07287	0.04952	0.17414	0.14739	0.19071
0.151	0.12753	0.04987	0.00504	0.22474	0.14135	0.25728	0.30597	0.18373	-0.0211	-0.13523	0.02402	0.05828	0.13929	0.13723
0.17624	0.0822	0.00902	0.00999	0.22417	0.23939	0.35219	0.32029	0.18687	0.28072	0.00362	-0.01446	0.00126	0.07667	0.15168
0.13501	0.11475	0.01781	-0.00914	0.24389	0.20879	0.27985	0.34646	0.21222	0.22224	-0.09174	0.00338	0.13695	0.15157	0.22789
0.12419	0.11479	0.04349	-0.0336	0.22135	0.20425	0.3259	0.30695	0.20189	0.12236	-0.04265	0.0696	0.15176	0.16715	0.11977
0.13414	0.1164	0.03701	-0.02404	0.19831	0.16745	0.32739	0.28797	0.17734	0.12785	-0.05425	0.0475	0.15639	0.15956	0.15124
0.15371	0.1176	0.06915	-0.04576	0.1983	0.151	0.33285	0.31092	0.18882	0.04754	-0.06395	0.03188	0.06615	0.15462	0.11442
0.1119	0.10051	-0.01269	0.06141	0.2611	0.23272	0.22002	0.33274	0.22192	-0.26429	0.00089	0.02288	0.10344	0.16488	0.17457
0.14263	0.10328	0.04345	-0.0038	0.23457	0.21361	0.28822	0.3476	0.23182	-0.1606	-0.05481	0.01513	0.00819	0.17643	0.25735
0.1356	0.10578	-0.00917	0.07249	0.2246	0.19978	0.29699	0.31953	0.23609	-0.42051	-0.13151	-0.01464	0.02778	0.16368	0.19944
0.13481	0.12567	0.03258	-0.01431	0.22122	0.17401	0.23048	0.31354	0.20202	0.01166	-0.08629	0.05124	-0.02595	0.13606	0.15439
0.1323	0.10412	0.03528	0.00954	0.23107	0.20751	0.28895	0.31442	0.1941	0.16222	-0.14308	0.03196	-0.03768	0.16918	0.14265
0.13646	0.12713	0.00654	0.0598	0.25168	0.23841	0.18111	0.34027	0.23463	-0.21552	-0.20386	0.01382	-0.07256	0.17201	0.34226
0.14907	0.11783	0.01256	0.00735	0.23792	0.16785	0.26912	0.31397	0.19931	0.0103	-0.15573	0.02218	0.18051	0.11938	0.17548
0.12836	0.13352	0.09186	-0.00602	0.21512	0.14707	0.26449	0.30595	0.2001	-0.10483	-0.05652	0.04225	0.19454	0.14374	0.16552

YLATION_	ILLARY_R	HOLESTE	LONATE_	OF_ENDO	NCER_CO	GEN_PH	ARDIOMY	GROWTH	ED_WITH	CYTOSKE	ORTICOID	D_IN_DN	NA_BIOG	N_OF_DN
0.2923	0.1161	-0.00598	0.01798	0.16734	0.12191	0.10835	0.03723	-0.20874	-0.14837	0.10769	0.00528	-0.166	0.09203	-0.00735
0.1536	0.0785	-0.0384	-0.04686	0.13286	0.07769	0.06192	0.01043	-0.12454	-0.03826	0.0762	-0.39017	-0.13943	0.09803	0.02101
0.27664	0.1077	-0.0456	-0.0675	0.14714	0.10329	0.17108	0.04696	-0.30809	-0.21362	0.08813	-0.36058	-0.15321	0.14545	0.01501
0.27692	0.11391	-0.06193	-0.05318	0.10845	0.0314	0.26695	0.04804	-0.20416	-0.21823	0.07987	-0.34543	-0.18859	0.12432	0.01139
0.24999	0.12655	-0.02509	0.00582	0.09435	0.06705	0.21322	0.06106	-0.23498	-0.11022	0.08582	-0.031	-0.0837	0.05753	0.02128
0.15145	0.08451	-0.06066	-0.05423	0.09901	0.04794	0.11809	0.03586	-0.47648	-0.15942	0.06459	0.00388	-0.05043	0.1167	0.02909
0.21539	0.11793	-0.04214	-0.05864	0.12165	0.08647	0.2006	0.05077	-0.48864	-0.11149	0.07655	-0.41853	-0.14326	0.04384	0.02969
0.29892	0.12994	-0.03449	-0.03718	0.11224	0.0366	0.28623	0.07021	-0.21296	-0.1642	0.08068	-0.23305	-0.14269	0.08592	-0.01127
0.29686	0.12587	-0.02507	-0.0165	0.08529	0.03795	0.28982	0.05803	-0.28325	-0.20142	0.08162	-0.25665	-0.13403	0.09475	-0.00313
0.23712	0.12015	-0.04062	-0.01896	0.07748	0.04326	0.27577	0.05252	-0.05848	-0.08309	0.07882	-0.35265	-0.04031	0.07511	0.02539
0.17688	0.08772	-0.03351	-0.06449	0.14437	0.08758	0.08414	0.01777	-0.39387	-0.10469	0.10832	-0.23481	-0.12891	0.03728	0.05067
0.21759	0.08487	0.05626	0.05629	0.16243	0.14187	0.08758	0.01058	-0.40233	-0.17292	0.08561	-0.32112	-0.13315	0.0487	0.0371
0.22287	0.11841	0.09992	0.09164	0.19316	0.16781	-0.10738	0.02705	-0.24943	-0.04966	0.09619	-0.45764	-0.14235	0.07798	0.0492
0.22947	0.09602	-0.03636	-0.10424	0.1269	0.08022	0.18598	0.03112	-0.44055	-0.10412	0.06045	-0.26898	-0.17067	0.08111	-0.00781
0.21064	0.10098	0.03725	0.10319	0.17222	0.10298	0.13948	0.03531	-0.15307	-0.22173	0.08997	-0.37819	-0.15847	0.0835	0.02922
0.20456	0.10496	0.01013	0.02266	0.162	0.09467	-0.12193	0.00938	-0.40056	-0.13302	0.07173	-0.33134	-0.17315	0.10541	0.07943
0.16462	0.11523	-0.07045	-0.05094	0.12496	0.04936	0.17215	0.04431	-0.32006	-0.18501	0.0776	-0.44601	-0.16572	0.03632	0.00671
0.30171	0.13082	-0.04948	-0.05848	0.10538	0.07033	0.24417	0.05334	-0.28131	-0.15882	0.07187	-0.2297	-0.12645	0.06615	0.01775

P53_PAT	ROSTATE_	MMUNE_	ECM_AND	NS_WITH	ERATION	NSHIP_WI	DRIAL_B	IAL_COMP	IAL_COM	AL_COMP	K_I_ASS	NTY_ACID	ATTY_AC	RIAL_GE
-0.10703	0.02348	0.01465	-0.06828	0.25102	-0.07888	0.35142	0.29856	0.2432	0.15986	0.16599	0.05147	0.25784	-0.13439	0.06746
-0.1245	0.00393	-0.05638	-0.03551	0.2738	-0.02626	0.34014	0.20821	0.20079	0.15351	0.12018	0.07748	0.15332	-0.1416	0.14332
-0.03887	0.01203	-0.01217	-0.04037	0.24544	0.02061	0.35705	0.29625	0.20143	0.16356	0.11077	0.0505	0.19529	-0.14855	0.11207
0.00072	0.01145	0.01989	0.02495	0.25409	0.02391	0.36128	0.23615	0.16238	0.10051	0.16801	0.08737	0.24465	-0.18396	0.00704
0.00778	0.04981	0.03333	-0.07469	0.23244	-0.00867	0.35424	0.24794	0.0819	0.11179	0.14473	0.03243	0.21725	-0.24088	0.09578
-0.01667	-0.00579	-0.08209	0.0578	0.20297	0.0049	0.33556	0.25234	0.12868	0.17937	0.05903	0.08298	0.19449	-0.03533	0.05025
0.00842	0.00383	-0.02492	0.1698	0.21617	0.08999	0.32711	0.26326	0.20541	0.2028	0.14494	0.0747	0.23185	-0.09995	0.05715
-0.00954	-0.00198	0.04726	0.02896	0.22982	0.05389	0.37802	0.23327	0.16962	0.10482	0.15435	0.07728	0.22686	-0.13027	-0.00229
0.00956	-0.01348	0.05068	0.08282	0.21931	0.00214	0.35529	0.21642	0.13869	0.14218	0.11767	0.07781	0.20708	-0.05811	0.01681
0.01751	0.00253	0.05775	-0.02406	0.19841	-0.05144	0.34895	0.24339	0.15135	0.17815	0.14979	0.08995	0.23933	-0.08548	0.05167
-0.04061	-0.03548	0.00895	-0.09497	0.23949	0.02872	0.37502	0.26373	0.22779	0.2654	0.1029	0.11552	0.20037	-0.07334	0.12108
-0.06233	-0.04689	-0.0813	-0.03688	0.19144	0.03417	0.33486	0.32418	0.21171	0.18897	0.17092	0.08541	0.23962	-0.09075	0.09797
-0.01579	0.02436	-0.12228	-0.00875	0.24968	0.02321	0.33662	0.21297	0.19569	0.11891	0.13797	-0.07036	0.18719	-0.12168	0.03647
-0.07111	0.00942	0.03175	-0.0318	0.25566	-0.01366	0.36304	0.29076	0.13411	0.18055	0.11931	0.0328	0.25105	-0.21289	0.06208
-0.02424	-0.00812	0.02017	-0.04016	0.27742	0.0422	0.34067	0.22861	0.18815	0.16661	0.13449	0.03363	0.19331	-0.05758	0.03835
-0.05687	0.00054	-0.03855	-0.03897	0.2506	-0.02489	0.31777	0.29364	0.23792	0.23571	0.13515	0.0575	0.1926	-0.04393	0.12453
-0.05178	-0.00433	-0.00876	-0.00047	0.23873	-0.05611	0.35758	0.30232	0.23446	0.19559	0.19377	0.0746	0.22073	-0.11679	0.08547
0.0059	0.01253	-0.00836	-0.01034	0.18654	0.02892	0.36974	0.25325	0.19166	0.14264	0.18432	0.09824	0.23165	-0.18809	0.04832

UNE_RE	CHAIN_F	R_SIGNA	SIGNALI	FACTOR_	AMINE_T	NA_PROC	E_INDUC	DENDRIT	R_DEF	CT_INPUT	ON_AND	ESIS_II_	SYNTHET	D_META
0.07544	0.24413	-0.03761	-0.03759	-0.13455	-0.03988	0.21124	0.00662	-0.1598	0.09805	0.07553	0.13295	0.02785	0.06917	-0.00959
0.05318	0.13505	-0.04117	-0.0527	-0.24841	-0.06012	0.27653	0.01561	-0.09364	0.04672	0.01865	0.1128	-0.03496	0.00716	-0.0268
0.06012	0.1775	-0.02571	-0.03229	-0.30045	0.01927	0.23844	0.19978	-0.12984	0.06909	0.05466	0.09331	-0.08385	-0.00513	0.02131
0.05906	0.23094	-0.01639	-0.01113	-0.20923	0.05	0.21312	0.09977	-0.10289	0.09146	0.06499	0.14757	-0.02549	0.05367	-0.00673
0.05842	0.20108	0.01352	0.03937	-0.32062	-0.04062	0.23005	-0.0245	-0.09323	0.08506	0.11159	0.14127	0.02951	0.06611	0.02943
-0.0041	0.17767	-0.01852	0.02407	-0.3227	0.04671	0.24304	0.09195	-0.10908	-0.00899	0.06097	0.13545	-0.15099	-0.02475	-0.05485
0.05924	0.21692	-0.00393	0.02998	-0.25819	0.06122	0.21346	0.25978	-0.08307	0.02965	0.07942	0.10859	-0.1066	0.01227	0.00063
0.06648	0.21207	-0.00853	-0.01126	-0.07015	0.03871	0.18994	0.08956	-0.08405	0.0477	0.12206	0.14893	-0.0542	0.06981	0.03985
0.06408	0.1904	-0.00873	0.00713	-0.34794	0.13202	0.20165	0.08923	-0.06194	0.07205	0.12964	0.14283	-0.02838	0.07399	0.05575
0.06247	0.22511	-0.00967	0.05801	-0.3421	0.08322	0.20523	0.11479	-0.03387	0.08663	0.12849	0.15365	-0.08181	0.04998	0.02457
0.0665	0.18452	-0.10144	-0.04899	-0.12094	0.01126	0.22698	0.14342	-0.13053	0.05323	0.02116	0.10888	-0.23973	-0.00869	-0.04269
0.09526	0.22572	-0.06185	-0.02999	0.039	0.00797	0.21678	0.12556	-0.07455	0.03605	0.05987	0.14552	-0.29031	-0.00409	-0.01151
0.00362	0.17149	-0.02849	-0.10046	-0.30167	0.02661	0.23515	0.14903	-0.09423	0.07208	0.02107	0.10491	0.01449	0.07321	0.00253
0.0557	0.23729	0.02823	-4E-05	-0.24477	-0.02745	0.22395	0.08729	-0.1273	0.03926	0.06848	0.12365	0.11751	0.06137	-0.02089
0.05457	0.17719	-0.0655	-0.04701	-0.02695	0.01935	0.22787	0.08105	-0.09904	0.04495	0.05622	0.1292	0.01739	0.05621	-0.01718
0.05445	0.17244	-0.04293	-0.08901	-0.10851	0.03393	0.22045	0.11932	-0.03187	0.0615	0.05102	0.07847	-0.13026	0.0592	-0.03738
0.05495	0.20404	-0.04062	-0.01694	-0.17023	-0.00462	0.23348	-0.00391	-0.1254	0.07893	0.03432	0.1369	-0.16805	-0.04611	-0.06404
0.07596	0.21667	0.03557	0.01488	-0.34699	0.00755	0.20119	0.08916	-0.03949	0.0563	0.12422	0.13267	-0.00865	0.06475	0.04004

_ACTIVATI	GERED_	AGGERED_	IGNALING	GNALING_	LARISATI	EPHROGE	ROTIC_ST	5B_SIGN	ARS_CO	MIGRATI	CELL_MI	REST_DIF	IRON_AC	ROINFLA	_AND_GL	NSMITTE
0.1758	-0.04558	0.07885	0.19103	-0.00263	0.0524	-0.11903	0.1826	0.15905	0.07152	0.03201	0.00357	-0.03022	-0.00481	0.00669	0.10839	0.33363
0.11338	-0.09938	0.01159	0.09296	0.05019	0.10189	-0.00747	0.20681	0.10511	0.0443	-0.00914	-0.03202	0.07806	-0.01875	-0.03679	0.0971	0.21437
0.13876	-0.05508	0.10963	0.11803	0.07735	0.08758	-0.04963	0.17507	0.13542	0.08572	0.02773	-0.00545	0.0634	-0.05407	-0.01989	0.14546	0.31319
0.17394	0.03431	0.09989	0.10819	-0.00713	0.05479	-0.36776	0.14675	0.12714	0.14357	0.02397	0.00397	0.01431	0.00564	0.00843	0.11188	0.35758
0.16994	-0.05722	0.12842	0.19531	-0.02774	0.10087	-0.20498	0.13854	0.14792	0.109	0.06287	0.03384	-0.02646	-0.0214	-0.00529	0.12507	0.33539
0.12302	-0.08124	0.09654	0.18138	0.05514	-0.02931	-0.1836	0.14385	0.12129	0.12927	-0.02851	-0.03796	0.03594	-0.0644	-0.00547	0.13038	-0.03259
0.158	-0.05364	0.10896	0.18265	0.05705	0.10052	-0.27401	0.1812	0.12908	0.16461	0.02994	-0.00253	0.03302	-0.02589	0.00348	0.13029	0.28149
0.16686	0.00661	0.05682	0.18618	-0.03046	0.08195	-0.13497	0.12274	0.1446	0.13645	0.05927	0.03413	0.00284	-0.0021	-0.00701	0.13409	0.37115
0.15567	0.02708	0.07203	0.14518	-0.01375	0.0809	-0.27107	0.12485	0.14929	0.15958	0.03686	0.00486	-0.00493	0.00095	-0.01382	0.12913	0.36586
0.16376	0.02727	0.11238	0.19743	-0.02689	0.09326	-0.2071	0.10643	0.1418	0.16249	0.0478	0.01846	-0.03227	-0.01078	-0.0111	0.10561	0.33785
0.12027	-0.07498	0.02977	0.12228	0.0557	0.08941	-0.00071	0.15027	0.10284	0.08217	0.03635	0.01615	0.05894	-0.01473	0.0131	0.11818	0.24185
0.15641	-0.01816	0.10884	0.13245	0.02537	0.06265	-0.00986	0.18863	0.16023	0.13645	-0.00127	-0.01836	0.00931	-0.02646	-0.01841	0.11878	0.28959
0.13025	-0.06511	0.07977	0.08565	0.12107	0.09265	-0.08981	0.20858	0.13977	0.07722	0.01291	-0.02003	0.0465	0.01128	-0.02655	0.10486	0.32993
0.15583	-0.0631	0.07765	0.13353	0.0158	0.08013	-0.17454	0.10816	0.11753	0.10075	0.02513	-0.00676	-0.00556	-0.01868	-0.00155	0.11687	0.35388
0.11781	-0.01769	0.05927	0.11833	0.01118	0.07984	-0.08428	0.1272	0.13434	0.11081	0.03499	0.01236	0.04836	-0.02863	-0.01405	0.11989	0.29681
0.13748	-0.03013	0.03418	0.09804	0.0847	0.11666	-0.27481	0.20772	0.11785	0.05186	0.03582	0.00976	0.06402	-0.02153	0.05598	0.12586	0.32112
0.15744	-0.04687	0.07464	0.10704	0.0303	0.06165	-0.20905	0.18243	0.11127	0.10162	0.02154	0.00181	0.04177	-0.01623	-0.01051	0.1142	0.30104
0.15592	0.00542	0.12624	0.19012	-0.00853	0.03783	-0.05408	0.14872	0.15471	0.12062	-0.00891	-0.03546	0.01934	-0.02214	-0.00505	0.11834	0.39299

BER_VA	ON_DO	RAGE_CO	TABOLISM	TOR_NL	LIC_FAT	CAL_ROL	NS_OF_1	LOGOUS	L_CELL_F	_ENDOT	TCH_SIG	SIGNALI	NENTS_	EDIATED	DELETIO
0.23931	0.17518	-0.08332	0.14002	-0.09486	0.10795	-0.21455	0.0236	0.43872	0.01962	0.00785	0.02533	-0.01572	0.01209	0.06409	0.32681
0.23781	0.26036	0.02324	0.15273	-0.02482	0.11712	-0.22987	-0.0133	0.46205	-0.01517	-0.00522	0.06786	0.00978	-0.01608	0.0423	0.35605
0.2433	0.1253	-0.0647	0.18976	-0.08611	0.10625	-0.15114	0.01596	0.45245	0.01403	-0.00885	0.07186	-2.7E-05	-0.01722	0.06474	0.31423
0.24328	0.251	-0.06704	0.07796	-0.05028	0.13116	0.25418	0.04318	0.42705	0.02407	0.03075	0.07316	0.01227	-0.00611	0.03169	0.27536
0.24293	0.25047	-0.01295	0.09173	-0.08053	0.10203	-0.0347	0.04133	0.43968	0.05412	-0.03867	0.01434	0.0047	0.01236	0.0563	0.29556
0.23798	0.25893	1.8E-05	0.18112	-0.14665	0.11273	0.38694	-0.0008	0.43043	-0.01201	0.13156	0.03187	-0.01355	-0.03811	0.07027	0.25734
0.23752	0.18824	-0.08295	0.09739	-0.05917	0.13678	0.18161	0.05051	0.43942	0.02357	0.0696	0.05994	0.01355	-0.01318	0.05362	0.28425
0.24005	0.24418	-0.10129	0.10336	-0.02538	0.12633	0.24326	0.06166	0.40405	0.01619	0.05123	0.04597	0.02879	-0.0044	0.0671	0.32791
0.24532	0.22591	-0.07101	0.06912	-0.04716	0.1402	0.25048	0.0778	0.41707	0.02601	-0.01053	0.05577	0.01944	0.02024	0.08983	0.31606
0.24465	0.24398	-0.03579	0.07996	-0.00596	0.15197	0.30368	0.10369	0.41623	0.03508	0.04532	0.0118	0.00242	0.03524	0.06458	0.28085
0.25004	0.19629	-0.03429	0.15036	-0.13452	0.13961	0.08772	-0.06092	0.42967	0.00116	-0.00852	0.05961	-0.00319	-0.0181	0.02573	0.28104
0.25349	0.25006	-0.04503	0.16055	-0.16134	0.10979	0.14444	0.00859	0.43213	-0.00538	0.1164	0.03836	-0.0369	0.01046	0.03664	0.32493
0.24847	0.16653	0.04068	0.17045	-0.08291	0.043	0.07046	-0.0526	0.45216	0.01937	0.00013	0.08164	-0.01413	-0.01932	0.06565	0.32343
0.23572	0.23927	-0.06263	0.06583	-0.01396	0.09642	-0.04404	0.01425	0.43972	-0.00355	-0.02357	0.06266	-0.01189	0.0015	0.05753	0.32498
0.25092	0.18341	-0.0683	0.16174	-0.103	0.10797	0.10093	0.02397	0.43503	-0.00783	0.09504	0.02509	-0.02822	-0.00266	0.02275	0.35288
0.25857	0.17659	-0.0277	0.17713	-0.10884	0.10513	-0.16417	-0.03994	0.45081	0.01065	-0.0657	0.03704	-0.04023	-0.00617	0.04851	0.32842
0.22466	0.28829	-0.05751	0.0927	-0.12962	0.14171	0.20627	-0.01945	0.44661	0.03722	-0.0266	0.08899	0.00995	-0.03107	0.04377	0.25914
0.22884	0.22699	-0.04028	0.11465	-0.05487	0.1483	-0.01621	0.06144	0.44766	0.03653	-0.02603	0.03851	0.02901	0.0084	0.0544	0.30784

ARE_RE	RF2_PA	TING_PATH	DELETIONS_	TRANSL	EAR_RE	IN_LIPID	ECEPTOR	IGOMER	IDE_EXC	REPAIR_IN	CAN_BIO	RMATION	ERENTIA	GA_6_FAT	FATTY_A
0.06947	0.18157	0.00468	0.18601	0.28387	-0.1302	-0.00335	0.11343	0.03659	-0.02867	0.15134	0.07738	0.00232	-0.12349	0.01436	0.12175
0.00591	0.15868	-0.00856	0.25436	0.30226	-0.22588	-0.09359	0.06868	0.01698	0.04359	0.19647	0.11335	0.26214	0.11199	0.0097	0.05585
0.04786	0.14765	0.0345	-0.02096	0.2882	-0.22453	-0.01358	0.09066	0.04348	0.01944	0.17031	0.04602	0.08095	0.11646	0.07574	0.06701
0.05615	0.18158	0.04742	0.32411	0.29981	-0.35676	0.00658	0.115	0.07438	-0.01203	0.15747	0.09582	0.01431	0.18598	0.11712	0.11294
0.07254	0.18744	0.04575	0.11784	0.29928	-0.33105	-0.07285	0.1148	0.06968	-0.00999	0.1637	0.08066	0.12236	0.11237	0.08124	0.08471
0.0476	0.17308	-0.01787	-0.41941	0.3213	-0.32746	0.03546	0.09931	0.01752	0.05022	0.19072	0.07494	0.2686	0.14604	0.11248	0.09252
0.02988	0.14252	0.07114	-0.02378	0.28042	-0.36903	-0.07507	0.08662	0.08372	0.01297	0.16657	0.08924	0.30101	0.13589	0.07881	0.09578
0.05751	0.1853	0.05804	0.23393	0.29748	-0.31233	-0.04194	0.12907	0.08878	-0.05524	0.13452	0.1181	0.13313	0.15811	0.05451	0.12871
0.02053	0.189	0.04922	0.10727	0.29691	-0.26617	-0.0276	0.13253	0.12343	-0.06615	0.12871	0.09348	0.01786	0.26494	0.03608	0.08908
0.03414	0.18695	0.06007	0.17086	0.29492	-0.30284	-0.07567	0.12275	0.10713	-0.00048	0.16154	0.07908	0.05275	0.17873	0.10385	0.11038
0.05246	0.17754	0.02877	0.04034	0.27129	-0.25549	0.02454	0.11268	0.05042	0.01233	0.17549	0.11131	0.09764	-0.07704	0.0928	0.05135
-0.01818	0.17818	0.01365	0.14427	0.32143	-0.22531	0.04476	0.12234	0.04069	0.01971	0.16968	0.08469	-0.02416	0.11914	0.06888	0.05444
-0.00593	0.15218	0.0277	0.24221	0.29683	-0.14923	-0.06352	0.07894	-0.00456	0.05068	0.18836	0.10382	0.10522	-0.00279	0.05521	0.06877
0.02463	0.17325	0.02107	0.23058	0.28965	-0.30592	-0.10984	0.11449	0.07062	-0.03767	0.15099	0.11601	0.15581	-0.09543	0.09886	0.14532
-0.00217	0.18403	0.01976	0.17668	0.31432	-0.27616	-0.04005	0.11275	0.04589	-0.008	0.16019	0.09234	0.14602	0.12307	0.02648	0.0777
0.01942	0.16949	0.02739	-0.03611	0.30879	-0.18025	-0.05397	0.08178	0.0562	0.02897	0.17975	0.05943	0.0748	0.13831	0.0386	0.0271
0.03165	0.15719	0.0234	0.12401	0.31115	-0.29597	-0.13649	0.10275	0.03951	-0.06842	0.14452	0.06503	0.01631	0.20741	0.08813	0.09155
0.05233	0.18055	0.02309	0.22019	0.29248	-0.29464	-0.07063	0.10985	0.07073	-0.03807	0.13788	0.07882	0.08803	0.17295	0.1389	0.08622

4_M_SIGN	RBON_M	BOLISM_A	RECEPT	ENTIATIO	OBLAST_	OCLAST_	PONTIN_	AS_IN_	TORIAN_INF	DN_PATH	RONS_ME	_NANOP	AMATORY_	_BY_CYT	OE_DAMAG	E_PHOSP	E_STRES
0.06133	0.16364	0.16635	0.08812	0.05034	-0.2122	0.04349	0.15257	-0.03994	-0.06439	0.11708	0.05814	-0.01271	-0.07259	0.04509	0.00689	0.15584	0.12627
0.03913	0.15071	0.14146	0.07447	0.03061	-0.30005	0.02954	0.1125	-0.05323	-0.00412	0.12513	-0.00798	0.00673	-0.14023	0.03074	-0.00432	0.15495	0.04825
0.05521	0.17231	0.18384	0.07215	0.03343	-0.13058	0.05323	0.14852	-0.02916	-0.03683	0.11132	0.12418	0.04522	-0.11848	0.0269	0.05748	0.13941	0.1036
0.05811	0.18084	0.17649	0.10653	0.06953	-0.05334	0.07457	0.13888	-0.10518	-0.05793	0.09743	0.14692	0.04674	-0.08333	0.03094	0.11808	0.16658	0.1427
0.08325	0.13999	0.18158	0.09758	0.05876	-0.23993	-0.04121	0.11202	-0.14594	-3E-05	0.06067	0.07449	0.06904	-0.13755	0.02538	0.07759	0.12979	0.14036
0.04634	0.16546	0.16958	0.0507	-0.01029	-0.23347	0.04897	0.03008	0.08367	-0.01922	0.09819	0.07175	0.05536	-0.10143	-0.04323	0.13696	0.15641	0.0771
0.06873	0.19514	0.15164	0.08652	0.07368	-0.08089	0.03237	0.15896	-0.03441	-0.07382	0.06725	0.18861	0.08408	-0.02404	0.00891	0.17955	0.1674	0.08952
0.06827	0.11302	0.1851	0.1132	0.04382	-0.07826	0.05554	0.16521	-0.19694	-0.06574	0.08806	0.18212	0.10252	-0.03861	0.0361	0.12304	0.15874	0.16624
0.07116	0.12415	0.1692	0.11608	0.04867	-0.11922	0.05793	0.11663	-0.15985	-0.0696	0.08392	0.16783	0.09579	0.07763	0.01482	0.13421	0.16041	0.13773
0.0862	0.15059	0.17345	0.0982	0.03522	-0.21726	0.02858	0.14005	-0.26253	-0.02283	0.04341	0.15866	0.08784	0.00116	-0.01081	0.15426	0.18645	0.13801
0.01557	0.21436	0.19874	0.07858	0.05964	-0.19451	-0.02413	0.08316	-0.04665	-0.06088	0.10623	0.06355	0.05561	-0.10567	0.03127	0.04518	0.19234	0.07729
0.02016	0.16319	0.15924	0.06382	0.03014	-0.02794	-0.04613	0.08284	-0.11415	-0.06781	0.02877	0.07171	0.01194	-0.13419	0.00581	0.09436	0.13823	0.11992
0.04074	0.15938	0.19972	0.09762	0.07105	0.0327	0.01664	0.11905	-0.03095	-0.04813	0.10994	-0.01732	0.00076	-0.13289	0.02367	0.01238	0.04757	-0.01007
0.05009	0.11461	0.18274	0.08011	0.04363	-0.12851	0.03382	0.13519	-0.09131	-0.02845	0.11247	0.01486	0.05878	-0.10608	0.07671	0.06901	0.14257	0.11724
0.04506	0.1867	0.19666	0.0849	0.02161	-0.09218	0.05097	0.1588	-0.05829	-0.07751	0.09656	0.04791	0.00735	-0.06632	-0.00255	0.05521	0.12041	0.123
0.01742	0.22928	0.17408	0.06853	0.04988	-0.22845	-0.02969	0.08671	-0.03644	-0.04385	0.09394	-0.05046	-0.01386	-0.14127	-0.06128	-0.05782	0.1424	0.02954
0.04264	0.15447	0.18016	0.09623	0.0425	-0.29534	0.06007	0.11327	-0.12059	-0.10406	0.09598	0.0976	0.05659	-0.08656	0.03832	0.00305	0.1758	0.108
0.06334	0.15365	0.15272	0.08904	0.05116	-0.12181	-0.0155	0.07859	-0.06991	-0.03469	0.05825	0.18185	0.05858	-0.1707	0.05301	0.08819	0.18532	0.12174

ERIVED_F	_SIGNAL	IPTIONAL	OPY_NUM	DENOCA	RIC_CANC	_39_5_DI	PROTEA	V_2_MEDI	CHERICH	CAL_ROL	5_IN_ADE	METABOL	FR_BETA	GF_PAT	PHOSPHA	E_PATHW	EPTIDE_G	2_INDUC
0.00124	0.08486	0.01619	0.27497	0.07517	0.18911	0.09806	0.22398	0.03525	0.29582	0.38401	0.0195	0.06536	0.04254	0.14817	0.33945	0.18962	-0.17657	0.24602
-0.0508	0.0326	0.0314	0.30591	0.05475	0.06416	0.09255	0.25302	-0.09293	0.27928	0.3789	0.01979	0.09424	0.00248	0.07958	0.33917	0.18175	-0.14579	0.32279
-0.00651	0.07205	0.03794	0.29011	0.0731	0.01367	0.06738	0.24801	-0.01265	0.29788	0.34525	0.04909	0.0573	0.04241	0.14826	0.37121	0.26756	-0.02953	0.29126
0.01508	0.06972	0.05324	0.29906	0.07298	0.1773	0.08716	0.25413	0.09882	0.30313	0.32447	-0.0415	0.10612	0.07994	0.13461	0.32753	0.20809	0.0277	0.26501
0.03239	0.08114	0.01368	0.30287	0.09493	0.04435	0.11518	0.26185	0.12507	0.28887	0.31214	0.02828	0.13068	0.07052	0.13194	0.35359	0.22108	0.00793	0.3779
-0.02129	0.07782	0.03792	0.2881	0.03607	0.11813	0.12335	0.25058	0.10636	0.2766	0.28184	0.0399	0.07624	0.03741	0.11424	0.32854	0.2017	-0.20845	0.34515
-0.02553	0.06306	0.05765	0.2781	0.09041	0.00891	0.09095	0.24491	0.17325	0.2916	0.33856	-0.01414	0.07632	0.06987	0.15893	0.35267	0.20698	-0.00958	0.30535
-0.01479	0.05317	0.03882	0.2972	0.07776	0.07419	0.08029	0.26014	0.03973	0.30595	0.29311	0.00608	0.08893	0.09323	0.14018	0.33785	0.221	0.07394	0.33833
-0.01698	0.0544	0.05034	0.29407	0.0815	0.10687	0.11696	0.26313	0.07366	0.29546	0.30368	-0.00509	0.08893	0.10144	0.13243	0.3431	0.22596	0.07584	0.36759
-0.02476	0.0949	0.04477	0.28726	0.07629	0.06967	0.11256	0.25409	0.22826	0.28533	0.25506	0.00712	0.14151	0.09311	0.14165	0.34424	0.22155	0.01191	0.3593
0.00017	0.02556	0.06322	0.31354	0.06431	0.15601	0.06808	0.27592	0.05246	0.30805	0.32764	-0.007	0.01505	0.01076	0.08453	0.36267	0.2416	-0.20251	0.25586
0.02707	0.08068	0.05111	0.29011	0.05877	0.24711	0.06721	0.2552	0.11315	0.30762	0.31883	0.03297	0.1154	0.02966	0.1106	0.34786	0.21045	-0.30958	0.27626
-0.03108	0.07984	0.04841	0.34251	0.0884	0.04188	0.04235	0.27175	-0.12526	0.31981	0.36375	0.05099	-0.01195	0.01801	0.11768	0.31736	0.19459	-0.36285	0.29723
0.03098	0.06008	0.02607	0.30346	0.06562	0.13808	0.08078	0.26395	0.08395	0.31357	0.31196	-0.01714	0.06895	0.03202	0.11563	0.35589	0.23027	-0.04607	0.2479
0.00948	0.05127	0.06435	0.28873	0.04283	0.13227	0.07439	0.25349	0.07923	0.30258	0.33715	0.02145	0.04329	0.01866	0.11812	0.35078	0.20772	-0.10338	0.29586
-0.00624	0.0341	0.04311	0.29502	0.06696	0.12139	0.11758	0.24727	-0.08009	0.30191	0.33944	0.03852	0.06197	-0.00797	0.0846	0.34726	0.19033	-0.34067	0.21262
-0.00907	0.06118	0.02438	0.29897	0.07406	0.11327	0.05819	0.24664	0.03559	0.32342	0.32817	0.03451	0.06701	0.05618	0.13391	0.33128	0.20332	-0.07107	0.29863
-0.00317	0.07681	0.02845	0.29661	0.07711	0.08261	0.08244	0.25777	0.14346	0.31798	0.26546	0.0091	0.11872	0.08911	0.1488	0.35438	0.23746	0.08012	0.34856

UTOPHAG	ANSFOR	IASES_IN_	NOSITIDE	_INDUCE	INDUCE	UCED_NF	INDUCED	IDUCED_	ES_AND_	OLOGICAL	_THERAP	OR_VITAM	_SIGNAL	YTIC_AST	IM_SIGN	AS_WITH	RAL_MES	CELL_DIR	ALPHA_	SIGNALIN	_AND_AN	X_RECEP	LANTATI	NTAL_GL
0.12845	-0.02005	0.04672	-0.11978	-0.0592	0.11245	0.05568	-0.00994	0.26829	0.13861	0.17837	-0.08798	0.12913	0.02407	0.13868	0.07392	-0.11108	0.0189	0.02546	0.21254	0.07684	0.05225	0.02013	0.23859	0.11622
0.13752	-0.0711	0.10325	-0.1392	-0.06197	0.10621	-0.0288	-0.03615	0.24913	0.09874	0.16823	-0.12207	0.10786	0.00875	0.08606	-0.01032	-0.09372	0.02946	0.00749	0.12784	0.0472	0.06382	0.03954	0.2951	0.13792
0.15531	-0.0903	0.02834	-0.13156	-0.08317	0.10045	0.0077	-0.04044	0.28981	0.13071	0.17618	-0.08224	0.14916	0.0147	0.14236	0.09757	-0.0752	0.03059	0.02931	0.21556	0.08636	0.08224	0.0451	0.22966	0.13131
0.16626	0.16475	0.0676	-0.09524	-0.12667	0.09336	0.05549	-0.04832	0.26066	0.10551	0.17487	-0.06933	0.17061	0.04654	0.03921	0.10702	-0.06336	-0.00074	0.01969	0.24212	0.13175	0.01402	0.07134	0.23243	0.09065
0.15736	-0.01607	0.09388	-0.05682	-0.07892	0.09832	0.05005	-0.0178	0.25951	0.13678	0.20842	-0.09038	0.16085	0.00342	0.12729	0.0224	-0.05276	0.00101	0.06644	0.24814	0.11434	0.02998	0.07261	0.21399	0.08213
0.11866	0.12144	0.08314	-0.14441	-0.14288	0.08199	0.06941	-0.1373	0.26053	0.13289	0.19311	-0.11118	0.1059	0.01285	0.10777	-0.03554	-0.14033	0.01251	0.05381	0.2571	0.09879	0.02951	0.05626	0.18991	0.09407
0.14345	-0.0291	0.05293	-0.15026	-0.09703	0.13032	-0.01817	-0.01554	0.26299	0.12815	0.17823	-0.05594	0.16456	0.05056	0.08657	0.0352	-0.15405	0.03499	0.05794	0.2312	0.08852	0.03571	0.06738	0.17967	0.13527
0.17597	0.10145	0.09727	-0.07376	-0.11323	0.10843	0.07733	-0.02579	0.27574	0.12102	0.1998	-0.10548	0.15891	0.03735	0.11826	0.07826	0.01007	-0.01003	0.04251	0.24838	0.15544	-0.01419	0.13989	0.19459	0.06651
0.18016	0.12758	0.07946	-0.05408	-0.09236	0.11527	0.04044	0.07069	0.2813	0.13595	0.19731	-0.09545	0.17894	0.03132	0.10753	0.06746	0.11812	0.00657	0.05342	0.25301	0.14762	0.00068	0.09016	0.22332	0.0945
0.15957	0.03193	0.08679	-0.08729	-0.09401	0.11379	0.06488	-0.01001	0.25254	0.11829	0.21803	-0.10781	0.17033	0.01815	0.13171	0.06506	-0.05977	0.00164	0.07816	0.23222	0.12352	0.009	0.06629	0.18601	0.04892
0.11654	-0.10663	0.09194	-0.13049	-0.0449	0.11875	0.00934	-0.04159	0.30034	0.12381	0.13647	-0.06271	0.13139	0.00845	-0.02139	0.09226	-0.19084	0.02605	0.14484	0.19515	0.07515	0.04634	0.04918	0.28618	0.10419
0.11859	0.08946	0.11969	-0.13397	-0.04512	0.10552	0.0131	-0.04716	0.31326	0.11651	0.16928	-0.11274	0.12562	0.01172	0.08586	0.10516	-0.13358	0.01999	0.18895	0.28465	0.09565	0.06483	0.05337	0.24299	0.11938
0.13851	-0.07424	0.11334	-0.18816	-0.04321	0.12812	-0.04035	-0.10333	0.30061	0.14285	0.14879	-0.0121	0.08392	0.04827	0.06889	0.09256	-0.10495	0.0522	0.04437	0.23085	0.04004	0.07576	0.02238	0.27087	0.14816
0.13725	-0.01156	0.09825	-0.08565	-0.09472	0.10116	-0.00131	-0.0239	0.25801	0.10441	0.14805	-0.08946	0.16179	0.01262	0.08073	0.07448	-0.04016	0.00045	0.01175	0.23066	0.13468	0.05641	0.05573	0.23623	0.07248
0.12235	-0.09857	0.08909	-0.14296	-0.10616	0.08104	0.04026	-0.02905	0.30114	0.1348	0.15979	-0.13933	0.13979	-0.00166	0.12217	0.00691	-0.12284	0.00961	0.01538	0.21007	0.07298	0.02766	0.0955	0.2367	0.10401
0.13636	-0.09031	0.04801	-0.16015	-0.02434	0.11255	-0.027	-0.04757	0.26537	0.12068	0.1371	-0.052	0.13222	0.01761	0.08159	0.11141	-0.1918	0.0327	0.10952	0.17829	0.04472	0.0543	0.02021	0.25504	0.15143
0.14997	0.0203	0.08155	-0.09811	-0.10904	0.1065	0.00766	-0.04555	0.24727	0.11016	0.17697	-0.06889	0.14337	0.04172	0.0488	0.09494	-0.03926	0.0178	0.01859	0.23813	0.10636	0.04534	0.05781	0.27234	0.11697
0.17899	0.05863	0.08233	-0.07588	-0.1037	0.11247	0.05764	-0.08119	0.24463	0.13927	0.17813	-0.07405	0.18346	0.03376	0.03869	0.01484	0.0032	-0.00259	0.02193	0.23905	0.12713	0.01613	0.06182	0.21554	0.09771

VARIAN_	_DISEASE	SOCIATED	N_SIGNA	IENE_ME	IENE_ME	GLANDIN	SYNTHES	SOME_D	LYCAN_B	L_TUBUL	ALING_O	IN_CYT	ED_REGU	NERGIC_	SINE_MET	SM_AND_	OLISM_A	_KB_AP	1_P38_M
0.10301	0.1185	0.18553	0.13809	0.09403	0.09403	0.03003	0.23834	0.30047	0.03626	0.21236	-0.06583	0.18448	0.08322	0.23877	0.08069	0.11528	-0.0799	-0.14995	0.08704
0.11607	0.10775	0.21958	0.08674	0.13444	0.13444	0.01614	0.24356	0.30252	-0.16738	0.20075	-0.06802	0.1672	0.11135	0.20424	0.08807	0.08823	-0.08322	-0.22471	0.05817
0.10472	0.12882	0.19636	0.12763	0.15887	0.15887	0.02975	0.27288	0.27577	-0.04931	0.25669	-0.01964	0.15036	0.15352	0.2081	0.13178	0.16007	-0.05406	-0.23287	0.08291
0.07437	0.07038	0.18477	0.13327	0.14277	0.14277	0.04255	0.23356	0.2879	-0.02784	0.2408	-0.01141	0.18982	0.04598	0.28723	0.16669	0.13932	-0.08248	-0.23093	0.07943
0.09031	0.12888	0.19225	0.14787	0.10759	0.10759	0.02005	0.24193	0.31795	0.05947	0.20278	0.01883	0.16958	0.0778	0.24273	0.21598	0.19172	-0.0865	-0.21163	0.09284
0.08328	0.12579	0.22862	0.11037	0.12051	0.12051	-0.10244	0.21602	0.31298	-0.03548	0.16852	-0.02285	0.1608	0.01891	0.19631	0.16604	0.14861	-0.05501	-0.22959	0.04359
0.08764	0.11236	0.20876	0.14218	0.15345	0.15345	0.04666	0.247	0.28314	-0.07988	0.19216	-0.00814	0.13631	0.05685	0.24528	0.1589	0.15789	-0.13021	-0.22441	0.07517
0.07185	0.10542	0.18783	0.16284	0.11097	0.11097	0.00237	0.22171	0.30876	0.05746	0.24806	-0.00692	0.19426	0.01789	0.30242	0.18258	0.16202	-0.01206	-0.17078	0.08092
0.07718	0.129	0.20322	0.15125	0.0994	0.0994	0.13066	0.23232	0.32407	0.00799	0.24764	0.02727	0.18044	-0.00114	0.28937	0.18179	0.16334	-0.03446	-0.14516	0.07966
0.07965	0.12423	0.18632	0.15706	0.08408	0.08408	0.03397	0.20993	0.31634	-0.03902	0.21329	0.01927	0.17343	-0.11482	0.26588	0.17497	0.17462	-0.08066	-0.20707	0.07697
0.09369	0.11744	0.23099	0.09672	0.05308	0.05308	-0.08803	0.22145	0.28315	-0.0203	0.19905	-0.06884	0.17152	0.16347	0.19981	0.15563	0.17545	-0.04129	-0.23961	0.07008
0.09363	0.1178	0.18342	0.11079	0.0122	0.0122	-0.03645	0.19376	0.29566	-0.03002	0.18193	-0.05455	0.16065	-0.00052	0.17229	0.21038	0.20119	-0.07919	-0.31107	0.05398
0.10159	0.12056	0.21299	0.10722	0.00341	0.00341	-0.178	0.19332	0.27419	-0.09045	0.22864	-0.0127	0.15435	0.12644	0.22568	0.14122	0.17543	-0.12116	-0.27245	0.0791
0.07341	0.10294	0.19389	0.11246	0.16809	0.16809	0.05645	0.26359	0.29013	0.00319	0.23676	-0.04343	0.19278	0.08043	0.27489	0.16597	0.15029	-0.06258	-0.18939	0.07722
0.11396	0.11674	0.18845	0.12	0.14666	0.14666	0.00027	0.24871	0.29234	-0.01014	0.2361	-0.05011	0.15554	0.09987	0.20188	0.15877	0.16475	-0.02622	-0.2482	0.04518
0.1409	0.11331	0.20285	0.0967	0.00136	0.00136	-0.07196	0.20967	0.28692	-0.09174	0.20127	-0.06828	0.16275	0.1275	0.16947	0.17746	0.18143	-0.08971	-0.32531	0.07531
0.09375	0.08787	0.18756	0.1258	0.13973	0.13973	0.0183	0.22779	0.27935	-0.09826	0.23349	-0.03986	0.20637	0.02023	0.29028	0.1354	0.12325	-0.10775	-0.23313	0.07119
0.08635	0.11868	0.17973	0.15446	0.09914	0.09914	-0.02378	0.25243	0.307	-0.01727	0.22735	-0.00758	0.19469	0.07118	0.28708	0.18993	0.16733	-0.07275	-0.19793	0.09076

TREAM_	RNK_SIGN	KININ_PA	AS_SIGNAL	_TUBUL	OF_ACTI	_PARATHY	PARATIO	SIGNALI	N_OF_THE	N_INFLA	IN_ALDO	EGULAT	G_PATH	STOMA_G	ROME_C	_AND_C	GNALING	DEVELO
0.21493	0.04345	0.01286	0.1004	0.0415	0.17863	-0.01685	0.15512	0.09461	0.1077	0.01633	0.13086	0.00574	-0.10834	0.07913	0.03121	0.04838	0.30932	-0.04026
0.18127	0.00566	-0.00855	0.07	0.05366	0.12233	0.00334	0.22006	0.11759	0.06021	0.02343	0.12847	-0.04658	-0.13325	0.11345	0.10058	0.0292	0.30534	-0.07969
0.1591	0.0343	0.05382	0.07717	0.04154	0.17193	0.0222	0.20841	0.18814	0.0621	0.02633	0.10977	0.00852	-0.09327	0.12761	0.04667	0.07506	0.32836	0.05415
0.19154	0.06918	0.14047	0.11284	0.06993	0.17911	-0.05832	0.17078	0.12406	0.10578	0.05537	0.17547	0.0312	-0.0833	0.07929	0.00371	-0.01093	0.38528	0.09231
0.21911	0.05548	0.08237	0.12974	0.09156	0.18725	-0.00862	0.17758	0.06877	0.10062	0.07206	0.13403	0.06458	-0.09645	0.09353	0.0482	0.01797	0.35909	0.00793
0.16952	-0.01534	0.21257	0.08567	-0.00284	0.1622	-0.1104	0.22677	0.07318	0.03928	-0.02463	0.1566	-0.0625	-0.12639	0.12724	0.03624	-0.01472	0.30415	0.00069
0.19187	0.05583	0.19039	0.08158	0.07109	0.164	0.0178	0.19263	0.08419	0.08722	0.05797	0.08687	0.00086	-0.05119	0.09776	0.02493	0.0671	0.34636	0.05862
0.19181	0.07028	0.16302	0.10729	0.04913	0.19496	-0.01943	0.1255	0.08768	0.08913	0.0497	0.1436	0.05784	-0.09562	0.00654	0.0035	-0.01018	0.39077	0.06355
0.18859	0.07033	0.19336	0.10124	0.07457	0.18435	-0.01985	0.09385	0.08258	0.08866	0.06987	0.12418	0.03939	-0.03035	0.03963	0.00138	-0.01153	0.39802	0.09102
0.18773	0.06157	0.17424	0.11281	0.0747	0.18562	-0.0217	0.15677	0.02886	0.09564	0.03696	0.1575	0.06086	-0.07791	0.03389	0.0141	0.02639	0.38975	0.04366
0.17619	-0.00282	0.06044	0.07556	0.06629	0.17524	0.02321	0.18702	0.05391	0.08007	0.01635	0.09187	-0.02404	-0.11557	0.15514	0.07139	0.00486	0.29279	-0.02419
0.17335	0.02358	0.14001	0.07249	0.01799	0.16777	0.01918	0.19093	0.08654	0.09757	0.00833	0.1551	-0.02273	-0.09252	0.12277	0.04359	0.0623	0.29116	-0.01613
0.21149	-0.01859	0.09955	0.10035	0.10115	0.17019	0.10464	0.19574	0.07925	0.0744	0.05469	0.03733	-0.10089	-0.01351	0.1399	0.07483	0.02571	0.2525	-0.00973
0.20542	0.02377	0.05666	0.1066	0.03916	0.17677	-0.00878	0.17757	0.15526	0.08703	0.04838	0.16503	0.04993	-0.00062	0.03588	0.04725	-0.03224	0.34097	0.0095
0.16916	0.03417	0.10768	0.07661	0.04108	0.17207	-0.05442	0.17558	0.11783	0.06188	-0.0096	0.13154	0.01251	-0.15589	0.09217	0.01839	-0.02527	0.31406	-0.0271
0.17349	0.00231	-0.0263	0.07637	0.0679	0.15631	0.08816	0.20073	0.13929	0.06085	0.08233	0.08969	-0.05791	-0.03642	0.12355	0.04234	0.0698	0.26988	-0.04174
0.19852	0.03779	0.07577	0.10412	0.04575	0.19267	-0.03851	0.14953	0.13739	0.09272	0.07572	0.1321	0.00789	-0.07011	0.01406	0.04467	0.02227	0.35796	0.05021
0.19282	0.06602	0.08395	0.11164	0.06837	0.18393	0.01122	0.13659	0.12199	0.08565	0.06612	0.13514	0.03549	-0.07322	0.03974	-0.0056	0.0376	0.37331	0.06648

_AND_CC	_ANTAGO	SION_AN	OXIDATIV	FORMATI	KINE_REC	CRONUT	INE_META	ONIN_AND	_ANXIET	1_GROUP	2_AND_B	FOR_4_6_	_TRANSP	SDUCTIO	NAFIL_TR	EP_REGU	CELL_LU	AMAGE_C	UPSKI_S
0.28435	0.07944	0.03097	0.05009	0.1454	-0.43003	0.18052	0.08515	0.07554	-0.00989	0.19098	0.23081	0.26519	-0.24103	-0.09706	0.0706	0.07521	-0.0451	-0.0152	0.03593
0.30287	0.02873	0.01078	-0.00759	0.06587	-0.4184	0.16884	0.10387	0.0455	-0.06354	0.14125	0.17244	0.17795	-0.23521	-0.15244	0.10866	0.11058	-0.05815	0.03891	0.01089
0.36108	-0.01107	0.03362	0.01389	0.17255	-0.34404	0.17956	0.19493	0.05234	-0.00696	0.15944	0.20624	0.21295	-0.18246	-0.11965	0.12684	0.13862	-0.03952	0.04352	0.03562
0.39306	0.00092	0.05058	0.04169	0.26458	-0.29871	0.19819	0.05984	0.14362	0.0953	0.17417	0.21153	0.20238	-0.18498	-0.10586	0.10456	0.10667	-0.05102	0.00633	0.02337
0.34695	0.01681	0.04756	0.03891	0.19903	-0.32603	0.18467	0.11782	0.15714	0.12065	0.20091	0.23165	0.23643	-0.19798	-0.07224	0.11101	0.22076	-0.04046	0.0019	0.05222
0.23993	-0.05275	0.03949	-0.00557	0.23889	-0.3253	0.19715	0.2442	0.04071	0.02053	0.13925	0.09448	0.16542	-0.17002	-0.12604	0.12187	0.10377	-0.05327	0.01037	0.04904
0.33267	-0.0146	0.05343	0.03032	0.18671	-0.22381	0.21719	0.21126	0.10143	0.06972	0.15926	0.17235	0.21628	-0.14465	-0.08694	0.12516	0.12414	-0.01444	0.04236	0.02652
0.40697	0.02799	0.06377	0.034	0.23179	-0.23723	0.1984	0.08691	0.12353	0.12022	0.1858	0.19703	0.22778	-0.16109	-0.07185	0.07547	0.14249	-0.04445	-0.0338	0.04061
0.41394	0.0026	0.08445	0.02856	0.24766	-0.07354	0.2095	0.0416	0.09085	0.10952	0.16808	0.14341	0.21674	0.05903	-0.02357	0.09067	0.16617	-0.03044	-0.01222	0.04753
0.39757	-0.01137	0.07313	0.04933	0.24009	-0.19157	0.21635	0.03167	0.16275	0.13723	0.19344	0.18877	0.22602	-0.14997	-0.06493	0.10995	0.1288	-0.04771	0.00782	0.03434
0.17383	0.04237	0.01274	0.07123	-0.07669	-0.44947	0.1902	0.23811	-0.01406	-0.10103	0.148	0.16983	0.19428	-0.15856	-0.13262	0.10309	0.11314	-0.01789	0.05277	0.07447
0.31493	0.06146	0.03261	0.08125	-0.06035	-0.38922	0.2039	0.20866	0.06095	-0.00739	0.19691	0.2026	0.22116	-0.22103	-0.1268	0.10814	0.13633	-0.03564	0.038	0.03316
0.17482	0.02677	0.0215	0.00092	-0.18064	-0.44148	0.16688	0.18518	-0.01706	-0.1495	0.15302	0.23743	0.22577	-0.11541	-0.09395	0.07176	0.11789	-0.02479	0.07633	0.08375
0.36532	0.04211	0.00757	0.0209	0.1993	-0.29805	0.1845	0.04812	0.07265	0.01454	0.15354	0.19608	0.20255	-0.1915	-0.09698	0.09129	0.10097	-0.06535	0.0321	0.02689
0.32736	0.08604	0.06142	0.03441	0.18695	-0.42442	0.19234	0.19802	0.03768	-0.03751	0.16444	0.21363	0.2425	-0.14735	-0.10121	0.05598	0.08445	-0.06074	0.03943	0.04345
0.28994	0.1068	0.02798	-0.02127	-0.03588	-0.46211	0.15619	0.2291	-0.0138	-0.15866	0.1452	0.21744	0.22588	-0.1481	-0.13978	0.14401	0.07262	-0.01301	0.14556	0.04583
0.3326	-0.00056	0.02825	0.03147	0.25001	-0.36464	0.18805	0.12145	0.03459	0.04463	0.1681	0.23065	0.21486	-0.19602	-0.13239	0.12741	0.10031	-0.03605	0.01179	0.04074
0.38904	-0.00921	0.04348	0.02446	0.15354	-0.20147	0.18225	0.03122	0.15457	0.10077	0.17703	0.18131	0.2294	-0.15002	-0.08074	0.11357	0.15291	-0.03813	0.03419	0.03185

SEX_DE	ATIONSHI	ABOLISM	METABOL	ID_METAB	GOLIPID	AL_CORD	A_REGU	OLESTER	CLE_DIF	OF_CHOL	OID_BIOS	NT_BIND	ASAKI_LC	LE_CON	DROME_	AND_ARO	TRANSF	C_METAB
0.04281	-0.07258	0.10841	-0.04016	0.10841	0.19455	0.12408	0.05397	-0.04755	0.03347	0.00507	-0.04231	0.04639	-0.02503	0.31756	0.18381	-0.40285	0.10935	-0.05034
0.21583	-0.02698	0.10889	-0.01443	0.10889	0.19985	0.15661	0.06589	-0.05629	0.08504	-0.02779	-0.0448	0.04613	-0.04325	0.3544	0.1693	-0.40831	0.02646	-0.09999
-0.03144	-0.02693	0.05771	-0.04454	0.05771	0.18954	0.13344	0.03892	-0.09785	0.05603	0.04487	-0.02446	0.04556	-0.0667	0.34789	0.13781	-0.45535	0.10464	-0.13082
-0.07544	-0.01613	0.17624	0.03965	0.17624	0.24487	0.15442	-0.00244	-0.0547	-0.04603	0.14238	0.1534	0.06217	-0.01661	0.31711	0.16208	-0.25753	0.13654	-0.11607
0.09496	-0.04631	0.07228	-0.02811	0.07228	0.19578	0.15169	0.04282	-0.0881	-0.06752	0.10168	0.0179	0.06172	-0.018	0.32778	0.18035	-0.42029	0.15753	-0.1687
-0.0745	-0.09666	0.15271	0.01825	0.15271	0.22973	0.12648	0.0637	-0.08475	0.01663	0.13524	0.35251	0.0508	-0.12433	0.3277	0.18507	-0.31231	-0.00905	-0.28079
0.0625	-0.05788	0.0814	0.00356	0.0814	0.19031	0.15648	0.03122	-0.08476	-0.03235	0.09331	0.2265	0.04853	-0.09268	0.30266	0.14164	-0.3981	0.01621	-0.23091
-0.25684	-0.06278	0.13501	0.01476	0.13501	0.24291	0.14713	0.02106	-0.07232	-0.02943	0.16867	0.15944	0.08069	0.00753	0.33793	0.16146	-0.16576	0.2229	-0.06151
-0.10798	-0.02786	0.15503	0.02816	0.15503	0.24575	0.1901	0.04651	-0.09714	-0.02038	0.17461	0.21441	0.06624	0.04802	0.33716	0.17001	-0.26951	0.18082	-0.07376
-0.30836	-0.09683	0.13042	0.00461	0.13042	0.2216	0.16447	0.00387	-0.10589	0.01666	0.16637	0.11958	0.06564	0.01171	0.3128	0.15716	-0.31108	0.13516	-0.01654
-0.07159	-0.1045	0.11102	-0.01179	0.11102	0.19137	0.11686	0.00361	-0.14572	-0.29606	0.00498	-0.03658	0.0451	-0.10048	0.31265	0.15702	-0.46433	0.01967	-0.1686
-0.24884	-0.06777	0.09542	-0.03575	0.09542	0.18687	0.13736	0.02731	-0.14497	-0.03138	0.10267	-0.05273	0.04501	-0.06626	0.32333	0.17813	-0.45129	-0.00788	-0.22405
0.04454	0.0491	0.10728	0.00557	0.10728	0.22138	0.12469	0.08097	-0.05385	-0.12407	0.08602	0.00252	0.06446	-0.16128	0.3332	0.17908	-0.43984	-0.02755	-0.1172
-0.31952	0.04324	0.08938	0.01576	0.08938	0.18589	0.1154	0.01492	-0.0827	-0.05585	0.07909	-0.03761	0.0536	-0.00933	0.3222	0.17758	-0.47507	0.15076	-0.20415
0.04715	-0.09679	0.05657	-0.01473	0.05657	0.14479	0.14289	0.06311	-0.03363	-0.07387	0.07359	0.04098	0.08338	-0.07705	0.34018	0.16277	-0.45728	0.12978	-0.17903
0.00423	0.0246	0.03147	-0.05025	0.03147	0.15152	0.10231	0.04822	-0.03654	-0.16518	-0.05369	-0.03176	0.0444	-0.15292	0.33825	0.14758	-0.45605	-0.02446	-0.19168
0.03566	-0.04092	0.10189	0.0006	0.10189	0.22524	0.13726	0.04949	-0.08137	-0.08311	0.10075	0.01484	0.0491	-0.04943	0.32299	0.18022	-0.45499	0.09425	-0.179
-0.17229	-0.00912	0.13701	0.00175	0.13701	0.23699	0.1259	0.02542	-0.16721	-0.09486	0.11085	-0.01052	0.02612	-0.00638	0.3367	0.16914	-0.32112	0.17018	-0.11816

_MEDIAT	SOCIATE	C_VESIC	AMIDES_A	FEN_ME	RAPAMY	AR_SYND	REBS_O	PYRUVA	CLE_IN_S	AND_INV	RECEPT	IGNALIN	ELLS_FOR	SIGNAL	D_SIGNAL	TION_OF
0.10175	-0.0139	0.27705	0.01186	0.12947	-0.03754	0.08224	0.32693	0.33718	0.23681	0.26397	0.02767	0.02767	0.16038	0.06327	-0.13554	-0.05848
0.1164	-0.02184	0.27662	0.0417	-0.18851	-0.0102	0.06918	0.29015	0.27559	0.17039	0.21238	0.05481	0.05481	0.17528	0.03147	-0.0547	0.13669
0.07988	0.00765	0.29094	0.0294	-0.03939	-0.01164	0.07871	0.34507	0.33241	0.24332	0.19894	0.01951	0.01951	0.19251	0.06047	-0.05845	0.0731
0.0797	0.01494	0.30545	0.09176	-0.07837	0.00414	0.07163	0.27653	0.28906	0.20117	0.20433	0.05442	0.05442	0.15183	0.04891	-0.07636	0.02026
0.13448	0.01971	0.28575	0.00755	0.00291	-0.00888	0.10633	0.28477	0.27791	0.20953	0.23883	0.00909	0.00909	0.11032	0.05547	-0.13043	0.099
0.06595	-0.00649	0.2855	-0.01378	-0.23319	0.02657	0.10121	0.27929	0.27164	0.17786	0.11057	0.03419	0.03419	0.11325	0.04467	-0.03222	0.0185
0.10765	0.01962	0.27547	0.06404	-0.17112	-0.00559	0.09453	0.32569	0.30552	0.208	0.19247	0.08098	0.08098	0.20742	0.0805	0.02808	0.0916
0.12568	0.01226	0.31566	0.12306	0.1207	0.00671	0.08344	0.27725	0.2996	0.20621	0.23645	0.04385	0.04385	0.19323	0.04704	-0.0246	0.05091
0.07721	0.02147	0.30007	0.0929	-0.02251	0.00976	0.08744	0.26039	0.25864	0.22289	0.20911	0.04151	0.04151	0.1537	0.05809	-0.05133	0.04668
0.1315	0.00759	0.2837	0.07199	0.05612	0.00166	0.11692	0.29438	0.26748	0.21245	0.2235	0.03917	0.03917	0.13376	0.05288	-0.08135	-0.06341
0.02712	-0.0172	0.2593	0.00685	-0.10452	-0.02107	0.09067	0.32489	0.32917	0.23614	0.26013	0.03424	0.03424	0.19517	0.05822	0.00833	0.17069
0.03294	-0.01545	0.2795	0.02537	-0.16827	0.0005	0.11269	0.33202	0.32219	0.2349	0.25136	0.01072	0.01072	0.1689	0.05232	0.00842	-0.0007
-0.01862	0.03569	0.29198	0.02835	-0.09459	0.03956	0.07233	0.26732	0.30916	0.22379	0.23525	0.02768	0.02768	0.17512	0.0845	-0.04019	0.15765
0.14434	-0.00212	0.30791	0.06222	-0.04472	-0.01285	0.06519	0.32027	0.32712	0.2141	0.20116	0.01809	0.01809	0.16264	0.04487	-0.05911	0.06729
0.0854	-0.00367	0.27392	0.02105	-0.00658	0.00449	0.07424	0.27941	0.28684	0.21781	0.21953	0.02409	0.02409	0.20872	0.04661	-0.05671	0.08896
0.01588	0.0159	0.2583	-0.0565	-0.21186	0.00667	0.10664	0.36991	0.32965	0.23485	0.27643	0.04306	0.04306	0.17008	0.06708	-0.11498	0.15114
0.06429	0.02426	0.31704	0.05889	-0.0666	-0.0167	0.05691	0.3308	0.29442	0.22307	0.19682	0.0056	0.0056	0.16115	0.0503	-0.062	0.06306
0.12209	0.00399	0.30213	0.08965	0.00512	-0.00946	0.09317	0.3109	0.29904	0.22146	0.23734	0.01711	0.01711	0.22006	0.0601	-0.07258	-0.04369

IFFEREN	ERMOGE	METABO	OPOIET	ND_PERIP	HORMON	OID_HOR	ALING_AN	A_SIGNAL	OF_APOP	EPTOR_S	R_SIGNA	P53_NET	Y_NRF2	SCADE_R	RS_SKI	E_MIRNA	R_REG
0.00375	0.06987	0.14718	0.07483	0.0071	0.15989	0.21279	-0.06891	0.05204	0.02952	-0.03146	-0.06589	0.03092	0.07093	-0.09585	0.12259	-0.01454	0.00431
-0.02249	0.07063	0.12222	0.03046	-0.01615	0.12367	0.14214	-0.11895	0.0571	0.02861	-0.0633	-0.0784	0.04637	0.01127	-0.13017	0.14782	-0.04224	-0.03284
-0.03473	0.05305	0.16977	0.08675	-0.00611	0.14001	0.1682	-0.09735	0.06809	0.05158	-0.01982	-0.06316	0.1167	0.09033	-0.0008	0.12886	-0.01891	-0.03386
-0.0033	0.05494	0.1457	0.09281	0.00888	0.17105	0.16432	-0.0662	0.06983	0.04546	0.01882	-0.04789	0.03074	0.09276	-0.03491	-0.00547	-0.06257	0.0158
0.06094	0.08021	0.14349	0.10425	-0.01639	0.17185	0.2068	-0.05125	0.0969	0.0517	0.0079	-0.01605	0.09832	0.13208	0.00944	0.09367	0.01913	0.01814
0.05642	0.07258	0.14814	0.05383	-0.00744	0.1457	0.16164	-0.11163	0.0347	0.02313	-0.05002	-0.07911	0.05529	0.08053	-0.10513	0.12942	-0.03478	-0.03105
0.05562	0.04488	0.12085	0.09084	-0.00232	0.167	0.20464	-0.10087	0.06986	0.06363	0.0011	-0.05567	0.03097	-0.02413	-0.20628	0.12557	-0.03322	-0.07708
0.01946	0.0517	0.20729	0.1206	-0.01084	0.17312	0.19682	-0.02005	0.08005	0.03591	0.02026	0.01569	0.03364	0.13488	-0.03002	0.02679	-0.03819	0.00476
0.04983	0.0557	0.16225	0.12164	-0.00841	0.15978	0.16979	-0.05423	0.08686	0.05087	0.06398	0.01625	0.09021	0.10172	-0.04798	0.05451	-0.04608	0.02366
0.06092	0.05188	0.1855	0.12954	-0.02123	0.17777	0.1919	-0.04811	0.09573	0.04574	0.03623	0.01199	0.08597	0.08113	-0.04806	0.12212	-0.02582	-0.0129
-0.0347	0.03903	0.14241	0.00674	-0.02193	0.14563	0.16752	-0.15012	0.04288	0.01776	-0.07437	-0.10572	0.09659	0.04162	-0.18638	0.0695	-0.04929	-0.03068
0.00674	0.05424	0.20833	0.04753	-0.0169	0.16013	0.17192	-0.11718	0.04245	0.00419	-0.03544	-0.0879	0.10889	0.00888	-0.19548	0.14627	-0.09721	-0.01713
-0.0692	0.11094	0.12242	0.00256	0.04809	0.16015	0.19067	-0.16105	0.05638	0.03612	-0.07783	-0.13344	0.08632	-0.03631	-0.19419	0.12698	-0.03825	-0.08081
-0.00336	0.06416	0.1729	0.06106	-0.01855	0.15351	0.19559	-0.06196	0.07239	0.02968	-0.0082	-0.03651	0.0095	0.09087	-0.00182	0.13946	-0.03848	0.02304
-0.03731	0.05782	0.11677	0.0565	0.01247	0.14832	0.23735	-0.08369	0.06076	0.04178	-0.03379	-0.05805	0.0597	0.06281	-0.08215	0.10249	-0.09956	0.02464
-0.06023	0.04851	0.19651	0.02783	-0.006	0.13653	0.19863	-0.16191	0.04564	0.04221	-0.06743	-0.09611	0.05653	-0.00571	-0.05495	0.15395	-0.02929	-0.02741
-0.02536	0.07091	0.18743	0.05943	-0.00506	0.17479	0.17304	-0.14311	0.06182	0.02876	-0.02004	-0.08659	0.10317	0.06198	-0.0077	0.07496	-0.01286	-0.0291
0.03311	0.04647	0.19477	0.11358	-0.01357	0.16947	0.19147	-0.03396	0.08845	0.05347	0.02193	-0.00732	0.08057	0.08369	-0.11952	0.043	0.02609	-0.00717

SLATION_	CHRONI	BON_ME	ULFURAT	LYCERID	GULATOR	SM_LEA	OPHAN_M	ISSOR_A	RY_REN	NTERFER	DIABETE	TERFER	THE_CON	D_SIGNA	L_NETW	LISM_AN
0.25987	0.11586	0.17098	0.214	-0.0569	0.14553	0.07452	0.19675	0.04465	0.14372	0.09527	0.0041	0.18084	0.10507	0.01562	0.10307	0.01696
0.27297	0.09495	0.15075	0.17662	-0.04978	0.14311	0.04525	0.219	0.12106	0.09904	0.05161	0.01782	0.1919	0.09287	0.02417	0.04253	-0.1027
0.26481	0.11677	0.18449	0.18295	-0.01612	0.15508	-0.02376	0.22632	0.05713	0.12949	0.13861	-0.06898	0.21981	0.10912	0.08128	0.13271	-0.02272
0.26249	0.12703	0.15112	0.09306	0.0032	0.16616	0.03543	0.20202	-0.01985	0.11685	0.11506	0.02003	0.22538	0.14476	0.07839	0.21159	0.13144
0.27672	0.12302	0.16434	0.16054	-0.05905	0.11984	0.0978	0.2201	0.06046	0.1306	0.09644	-0.03127	0.20913	0.04375	0.06333	0.16952	0.00912
0.26192	0.08352	0.19448	0.19707	-0.05269	0.12538	-0.03771	0.20528	0.04071	0.15833	0.11465	-0.13153	0.19229	0.11831	0.03514	0.08756	-0.02364
0.25354	0.14013	0.18355	0.20883	-0.10967	0.17111	0.0301	0.22836	-0.00444	0.11665	0.17299	-0.08291	0.21126	0.14795	0.09678	0.15449	-0.18365
0.2579	0.11151	0.16056	0.12192	0.0002	0.14581	0.03462	0.21432	-0.0338	0.12993	0.17065	0.01282	0.22597	0.21182	0.10461	0.22808	0.07716
0.25417	0.12108	0.15281	0.14514	-0.01229	0.14585	0.04112	0.20448	0.00992	0.08269	0.14146	-0.01089	0.28254	0.15129	0.09133	0.24075	-0.05504
0.24608	0.12346	0.15078	0.09748	-0.05438	0.14127	0.01662	0.21019	-0.04237	0.09699	0.19831	-0.03377	0.27043	0.09212	0.13943	0.23373	0.00294
0.27162	0.09344	0.20678	0.21229	-0.11692	0.16408	-0.06787	0.18489	0.02284	0.18399	0.0126	-0.11405	0.13326	0.12537	-0.05536	0.02989	-0.23305
0.27321	0.10403	0.19141	0.22472	-0.00617	0.13583	-0.05528	0.20345	0.0372	0.12612	0.11551	-0.0784	0.18595	0.12212	0.07757	0.08	-0.08992
0.27527	0.11968	0.19702	0.22411	-0.02604	0.14152	0.05815	0.13415	0.11951	0.15674	0.01849	-0.06297	0.13478	0.07503	-0.02499	-0.08425	-0.10735
0.25915	0.11207	0.16201	0.09204	-0.09736	0.13629	0.0991	0.23172	0.01483	0.13099	0.06217	-0.01219	0.15738	0.1121	0.02881	0.15241	0.04692
0.27867	0.10408	0.19244	0.16943	-0.12393	0.11995	0.01916	0.18222	0.03965	0.08255	0.10354	-0.03928	0.19979	0.12203	0.05805	0.11282	0.13158
0.2866	0.11048	0.21764	0.23465	-0.07682	0.12161	0.04781	0.20387	0.0544	0.16216	0.01622	-0.03748	0.17576	0.07496	0.05041	-0.05513	0.10535
0.27105	0.1232	0.16778	0.10475	-0.0613	0.18743	-0.05884	0.20079	0.08794	0.1326	0.0429	-0.03855	0.14232	0.0694	-0.01818	0.13973	-0.01315
0.25225	0.12162	0.13759	0.11811	-0.07995	0.15082	0.06333	0.23454	-0.01365	0.12441	0.13871	-0.03252	0.23226	0.09642	0.11487	0.1913	0.01999

CTIVATION	WAY_DUR	TION_IN	AND_CO	PTOR_SI	ARS_SYN	VE_COLI	D_PROTE	ND_ASS	METABOL	E_AND_RE	EDIATED	OIC_ACI	DULATED	VEGFR2	I_AND_A	S_CAROT	N_B12_D
-0.02685	0.02058	0.00168	-0.03525	-0.00689	0.27736	-0.17735	0.0637	0.23263	0.05203	0.13637	0.09808	0.2559	0.25252	0.20605	-0.13511	0.03809	0.02546
-0.05389	-0.0174	0.00523	-0.04149	-0.05111	0.23114	-0.20687	0.02143	0.18573	0.04277	0.04481	0.11127	0.18515	0.24571	0.19734	-0.02642	0.01148	-0.03685
-0.01415	0.00858	0.01098	-0.0119	-0.02467	0.26389	-0.14358	0.08717	0.2271	0.08834	0.02655	0.13089	0.24736	0.20375	0.20263	-0.26193	0.04841	-0.15635
-0.00094	0.01676	0.04947	-0.0385	-0.01295	0.25869	-0.11133	0.10106	0.16973	0.03179	0.03452	0.15114	0.20299	0.26074	0.19815	-0.45799	0.03062	-0.01341
0.03354	0.06552	0.05319	0.00961	0.04892	0.19612	-0.02908	0.09976	0.21577	0.05547	0.11636	0.14888	0.21787	0.25257	0.20038	-0.30069	0.0238	-0.20109
0.01445	0.02037	-0.0014	0.03313	0.01461	0.18361	-0.11629	0.06082	0.22767	0.14767	0.15529	0.07374	0.2082	0.21765	0.18833	-0.33733	0.00393	-0.02386
0.04443	0.04377	0.03243	0.02443	0.03116	0.22726	-0.1016	0.07481	0.21859	0.15195	0.04211	0.12579	0.21538	0.17588	0.19916	-0.41448	-0.01709	-0.17526
0.00815	0.03284	0.06322	-0.00832	0.02432	0.26981	-0.01915	0.10824	0.17738	0.04228	0.02027	0.15684	0.21499	0.261	0.20092	-0.43069	0.047	-0.0185
0.01501	0.03711	0.04854	0.00071	0.0295	0.22167	-0.00944	0.15461	0.15494	0.01409	-0.08354	0.16634	0.187	0.25299	0.2053	-0.36023	0.00679	-0.14422
0.04097	0.06002	0.08788	0.03144	0.07705	0.21078	-0.01956	0.12562	0.16425	0.01384	-0.0144	0.1377	0.22251	0.23901	0.19514	-0.19834	-0.0029	-0.11852
-0.01895	-0.01912	0.02304	-0.03007	-0.0506	0.26895	-0.19848	0.04806	0.26941	0.19621	0.17209	0.11679	0.22427	0.25055	0.20678	-0.18302	0.00436	0.07103
-0.00546	0.00348	0.01989	0.00486	-0.01444	0.24095	-0.19164	0.07319	0.22034	0.1206	-0.01518	0.0737	0.26323	0.24575	0.20489	-0.23111	-0.07671	-0.03854
-0.04863	-0.03008	-0.04387	-0.02905	-0.06655	0.26819	-0.28639	0.06176	0.22238	0.16955	0.11335	0.06005	0.21029	0.24771	0.21382	-0.2112	-0.00015	-0.05112
-0.00415	0.03733	0.00064	0.01129	-0.00669	0.26836	-0.09784	0.07503	0.20456	0.09615	0.03901	0.12376	0.25826	0.25074	0.20239	-0.36886	0.04286	0.02635
-0.03097	0.00929	-0.01828	-0.0481	-0.01476	0.28123	-0.19374	0.09213	0.20715	0.11266	0.04591	0.12991	0.25942	0.25549	0.20164	-0.26616	0.07994	-0.03516
-0.05795	-0.0152	0.03377	-0.02426	-0.06911	0.24951	-0.30973	0.07468	0.27777	0.20306	0.09682	0.03577	0.26128	0.21396	0.19861	-0.23498	-0.04874	-0.07467
-0.02762	0.00424	-0.01148	-0.01381	-0.02718	0.23798	-0.15782	0.07534	0.21686	0.05589	-0.00198	0.11522	0.25486	0.25114	0.20193	-0.39035	-0.00466	0.00697
0.03176	0.04101	0.04643	0.01508	0.03201	0.23928	-0.03822	0.09008	0.1781	0.02434	-0.02689	0.12611	0.20908	0.25912	0.20495	-0.31883	0.05707	-0.13412

N_B12_	MENT_AND	INFLAM	IN_D_ME	RECEPT	ALCIUM	D_ACTIVA	UBIQUITI	CELL_DI	SIGNALIN	INT_SIGN	LING_IN	SIGNALIN	PATHWAY	C_HOME	AL_RUFFLE_FORMATION
0.16849	0.19857	0.01034	-0.01841	0.07761	0.16124	-0.04635	0.14078	-0.00827	0.08575	-0.02037	0.08547	0.01633	0.03759	0.13751	0.20223
0.13619	0.18876	-0.0238	-0.07117	0.0618	0.14805	-0.05716	0.18508	-0.05997	0.11806	-0.03772	0.08078	0.04177	0.03232	-0.0482	0.04422
0.14387	0.24458	-0.05049	-0.02119	0.06518	0.16637	0.03349	0.18821	-0.05744	0.15307	0.01803	0.13614	0.07186	0.05942	-0.12442	0.23959
0.18157	0.14115	-0.03408	-0.01486	0.12619	0.14372	0.05347	0.17495	-0.01795	0.09377	-0.02033	0.08434	0.06208	0.05923	-0.01407	-0.16619
0.14441	0.18479	0.03947	0.04574	0.05971	0.15288	-0.01204	0.16725	0.03427	0.02552	-0.01863	0.05366	0.0422	0.04954	-0.07135	-0.05016
0.18068	0.19476	-0.04774	-0.02536	0.07389	0.13458	0.09198	0.1561	-0.01418	0.07232	0.01183	0.07036	0.03777	0.07095	-0.03468	-0.38567
0.20759	0.18461	0.00364	-0.03507	0.10455	0.07448	0.10751	0.1634	-0.07999	0.05858	0.00899	0.08945	0.04149	0.0478	-0.21646	-0.22019
0.17491	0.12571	0.00994	0.06208	0.09884	0.13532	0.0554	0.1626	-0.03888	-0.00355	-0.00843	0.0192	0.05389	0.03852	-0.01896	-0.24434
0.19071	0.15778	-0.00115	0.06569	0.11691	0.12372	0.10328	0.16709	0.01228	0.0473	-0.00987	0.04378	0.05866	0.04534	-0.07441	-0.21287
0.19811	0.1602	0.02131	-0.01298	0.0994	0.11076	0.0706	0.13902	0.03172	0.04291	-0.01915	-0.02738	0.07658	0.02411	-0.03313	-0.19242
0.18034	0.20767	-0.06156	-0.00668	0.07678	0.14584	0.03974	0.18982	-0.12883	0.07256	-0.00704	0.07317	-0.01106	0.038	-0.11136	0.13422
0.21588	0.19047	-0.05321	-0.04766	0.05269	0.13123	0.15279	0.14625	-0.05703	0.1014	-0.02038	0.04797	0.03945	0.01619	-0.05191	0.21049
0.1551	0.23885	-0.07472	-0.10555	0.01129	0.13822	0.05934	0.17364	-0.10472	0.15219	0.01876	0.17745	0.0749	0.09497	-0.01989	0.23039
0.15602	0.15107	-0.00731	-0.02058	0.075	0.14574	0.03507	0.19483	-0.03901	0.07957	-0.02127	0.06503	0.05231	0.05183	-0.07126	-0.11621
0.16494	0.20546	-0.04604	-0.07495	0.07779	0.10826	0.04892	0.15546	-0.06629	0.06998	-0.03239	0.08433	0.02637	0.03355	-0.05751	0.0591
0.13249	0.2461	-0.07716	-0.14228	0.00425	0.09316	0.02371	0.1988	-0.06045	0.15546	0.01447	0.1185	0.07436	0.06604	-0.13218	0.16888
0.17092	0.21209	-0.06699	-0.08591	0.0895	0.16675	-0.0561	0.17276	-0.05595	0.06445	-0.02799	0.0491	0.04398	0.06788	-0.11458	0.08818
0.15417	0.17872	0.02617	0.02489	0.09559	0.14303	0.01079	0.18033	-0.00834	0.0813	0.01332	0.08949	0.06519	0.03991	-0.04124	-0.19119

Supplementary Table 6. Estimate score

	StromalScore_estimate	ImmuneScore_estimate	ESTIMATEScore_estimate	TumorPurity_estimate
HGSOC1	-254.921335	298.5455769	43.62424198	0.818850519
HGSOC2	-191.0290818	52.84007252	-138.1890093	0.833876241
HGSOC3	61.24996139	420.0345211	481.2844825	0.780310496
HGSOC4	316.5122877	711.385707	1027.897995	0.727674584
HGSOC5	20.58773737	828.7664351	849.3541725	0.745399401
HGSOC6	-7.929692974	420.5749541	412.6452611	0.786571887
HGSOC7	369.7950576	532.2418593	902.0369169	0.740222062
HGSOC8	495.5783055	804.8608343	1300.43914	0.699658737
HGSOC9	651.1273449	916.6772277	1567.804573	0.671086701
HGSOC10	536.4999082	962.9727314	1499.47264	0.678489097
HGSOC11	-594.3689503	13.01219212	-581.3567582	0.86799265
HGSOC12	-242.2369388	322.3091878	80.07224897	0.815767795
HGSOC13	-758.4997342	-547.7403587	-1306.240093	0.915821984
HGSOC14	124.7799969	601.1912457	725.9712426	0.757349736
HGSOC15	-173.7232274	310.3371506	136.6139232	0.810939398
HGSOC16	-746.4304717	-489.4571065	-1235.887578	0.911626088
HGSOC17	-1.363278676	485.9465503	484.5832716	0.780007575
HGSOC18	299.5163654	801.1382281	1100.654594	0.720307692

Supplementary Table 7. Expression spectrum of the ssGSEA-transformed platinum dataset annotated by h.all.v2023.2.Hs.symbols.gmt

	HALLMARK_ADIPOGENESIS	LLOGRAF	NDROGE	RK_ANGIO	_APICAL	K_APICAL	ARK_APO	ILE_ACID	LESTERO	RK_COAG	RK_COMP	ARK_DNA	RK_E2F_T	AL_MESEN	OGEN_RI	ROGEN_R	TTY_ACID	_G2M_CH	ARK_GLYC	EDGEHO	HEME_M	MARK_HYP	IL2_STAT5	_JAK_ST	LAMMATO	FERON_A	FERON_G	KRAS_SIG	KRAS_SIG	_MITOTIC	MTORC1	_MYC_TA	_MYC_TA	ARK_MYOG	_NOTCH_	ATIVE_PH	RK_P53_	PANCREAS	RK_PERO	K_AKT_M	PROTEIN_	E_OXYGE	_SPERMA	TGF_BETA	A_SIGNA	LDED_PR	_UV_RES	_UV_RES	BETA_CA	NOBIOTIC	Group
HGSOC1	0.364544837	0.280223	0.298029	0.056553	0.271248	-0.09701	0.261142	0.173461	0.297637	0.142779	0.29757	0.068167	0.279854	0.05035	0.131381	0.230094	0.504622	0.384901	0.421665	0.213781	0.247526	0.393776	0.248998	0.052575	0.092545	0.197358	0.281448	-0.04988	0.076243	0.132021	0.54324	0.701201	0.144004	0.104065	0.048875	0.536619	0.153557	0.401011	0.331061	0.292032	0.376232	0.429252	0.104502	0.257971	-0.01724	0.342233	-0.01382	0.374574	0.028402	0.27087	sensitive
HGSOC2	0.265847098	0.192461	0.242641	-0.11463	0.251157	-0.13976	0.229976	0.082962	0.204873	0.100671	0.157264	0.153975	0.357857	0.037815	0.085267	0.201221	0.443622	0.435716	0.408481	0.288209	0.204597	0.299889	0.205913	0.016049	0.022178	0.239865	0.239233	-0.05547	-0.02329	0.126008	0.505595	0.760017	0.249148	0.097015	-0.00928	0.507464	0.130385	0.516275	0.248508	0.264281	0.340718	0.434412	0.180415	0.335214	-0.05279	0.42414	-0.06806	0.322706	0.083423	0.191848	sensitive
HGSOC3	0.354740072	0.307285	0.262094	-0.01051	0.275063	0.022604	0.284957	0.179181	0.253323	0.229413	0.306487	0.121805	0.402216	0.075682	0.098072	0.225066	0.518964	0.435188	0.40042	0.260979	0.252005	0.307728	0.236227	0.116642	0.078689	0.307018	0.371572	-0.07851	0.015724	0.134673	0.563707	0.749582	0.277683	0.185584	0.011124	0.542927	0.170646	0.409537	0.288682	0.268769	0.336006	0.45393	0.083269	0.230623	-0.05205	0.399616	-0.03242	0.365606	0.139003	0.297475	sensitive
HGSOC4	0.310633469	0.352231	0.251445	0.035232	0.270496	-0.04698	0.280669	0.188924	0.186494	0.389745	0.44121	0.097546	0.289921	0.123168	0.090181	0.178172	0.48391	0.352079	0.356897	0.182505	0.224291	0.34358	0.266265	0.153113	0.190749	0.374053	0.429735	0.017035	0.125532	0.101895	0.531653	0.702538	0.15169	0.1465	0.119411	0.485168	0.219667	0.53955	0.323347	0.281649	0.398583	0.492516	0.036989	0.234619	-0.01911	0.340513	-0.07173	0.349714	0.098536	0.333594	sensitive
HGSOC5	0.28825585	0.393781	0.219636	-0.03229	0.223961	-0.08281	0.313515	0.205461	0.220065	0.24682	0.414318	0.110994	0.287503	-0.0096	-0.01545	0.119845	0.474899	0.363708	0.365733	0.138154	0.354673	0.321331	0.238375	0.09973	0.081521	0.289821	0.373905	-0.14296	0.080956	0.120326	0.548975	0.712841	0.183647	0.030231	0.139291	0.465533	0.162596	0.559859	0.277411	0.29005	0.346145	0.48471	0.03251	0.13755	-0.02818	0.331231	-0.17012	0.331774	-0.06116	0.312173	sensitive
HGSOC6	0.3055658	0.355232	0.298374	0.210665	0.184994	-0.13921	0.300737	0.283378	0.178119	0.450794	0.399202	0.12143	0.410247	0.15378	-0.01762	0.157882	0.465288	0.403	0.402365	0.098541	0.384629	0.338587	0.256456	0.034089	0.004684	0.321402	0.364642	-0.08902	0.114528	0.101844	0.573866	0.747399	0.305889	0.11683	0.093316	0.467761	0.148739	0.531594	0.335288	0.318084	0.328271	0.479923	0.133331	0.187687	-0.11766	0.38856	-0.04958	0.317085	-0.0748	0.332044	sensitive
HGSOC7	0.3336639	0.342912	0.243048	0.24552	0.219861	-0.106	0.383575	0.162399	0.242664	0.497307	0.438299	0.113963	0.363393	0.224152	0.08063	0.18275	0.479623	0.402415	0.414254	0.271192	0.153959	0.345202	0.325431	0.13102	0.100327	0.456669	0.499022	-0.1003	0.072224	0.078116	0.540515	0.741917	0.357039	0.173965	0.122428	0.530698	0.178408	0.493463	0.312293	0.292149	0.291875	0.469877	0.084978	0.294747	-0.0446	0.417847	-0.04556	0.380385	-1.8E-05	0.32846	sensitive
HGSOC8	0.340148236	0.396681	0.260119	0.175936	0.285677	0.056133	0.304996	0.235682	0.19844	0.431719	0.482507	0.049972	0.183762	0.172351	0.041299	0.142659	0.490354	0.284824	0.366823	0.23356	0.309031	0.364229	0.273312	0.208705	0.220471	0.351603	0.412995	-0.05771	0.131052	0.109386	0.536902	0.660197	0.050307	0.142737	0.11485	0.489926	0.221059	0.505951	0.296639	0.287911	0.419368	0.504625	-0.00519	0.193255	0.037664	0.331234	-0.10995	0.337322	0.018314	0.361629	sensitive
HGSOC9	0.310081543	0.44999	0.217721	0.070461	0.254568	0.116566	0.344294	0.254423	0.227693	0.420232	0.500347	0.034721	0.213634	0.170205	-0.00982	0.098189	0.474999	0.31518	0.365782	0.225457	0.263339	0.349533	0.299801	0.294567	0.256888	0.42975	0.522282	0.049655	0.188113	0.120919	0.529822	0.670459	0.063144	0.197233	-0.08335	0.460866	0.164808	0.443855	0.296113	0.286411	0.39625	0.500397	-0.0312	0.202832	0.137556	0.336203	-0.09186	0.333712	-0.02975	0.344499	sensitive
HGSOC10	0.316018106	0.446048	0.229736	0.143124	0.224124	-0.08709	0.34467	0.146385	0.167482	0.401032	0.490695	0.097278	0.250157	0.056368	-0.02619	0.119817	0.469163	0.342497	0.354488	0.272581	0.214309	0.320946	0.26625	0.23974	0.209416	0.505118	0.543403	0.057228	0.1446	0.097493	0.528717	0.681889	0.081358	0.086925	-0.00289	0.499144	0.167884	0.538424	0.26632	0.311143	0.360331	0.498036	-0.01254	0.196167	0.04035	0.339225	-0.11429	0.361977	-0.2029	0.333196	sensitive
HGSOC11	0.378649576	0.254244	0.24767	0.076628	0.259944	-0.0354	0.251203	0.185209	0.242234	0.277673	0.244956	0.115611	0.430634	0.064364	0.092688	0.221644	0.503101	0.421735	0.453723	0.218253	0.187889	0.382107	0.231925	-0.07105	-0.02861	0.209598	0.254539	-0.01819	0.002529	0.096241	0.578987	0.770082	0.257586	0.146	0.183707	0.552711	0.172034	0.45347	0.359737	0.26254	0.330045	0.428301	0.126231	0.264078	-0.07781	0.431547	-0.1303	0.349034	0.028248	0.283057	resistant
HGSOC12	0.346010195	0.319907	0.306425	0.123869	0.289041	-0.15463	0.297009	0.166331	0.297009	0.378808	0.357022	0.099807	0.37176	0.140856	0.065418	0.182943	0.517145	0.372704	0.423601	0.162762	0.233057	0.352794	0.294325	0.003558	0.052523	0.355237	0.383859	-0.0264	0.039406	0.07904	0.557788	0.754997	0.255852	0.146674	-0.02057	0.542245	0.197307	0.370135	0.290215	0.288635	0.285459	0.475403	0.035552	0.214634	-0.07514	0.368978	0.033829	0.371398	-0.06826	0.314574	resistant
HGSOC13	0.310160946	0.110657	0.273162	0.095928	0.272673	-0.04224	0.214521	0.077521	0.323537	0.27129	0.150113	0.120296	0.411134	-0.00345	0.16551	0.233007	0.459379	0.415269	0.391354	0.108461	0.237273	0.344738	0.204084	-0.04849	-0.08216	0.206129	0.254371	-0.08519	-0.0894	0.155867	0.55669	0.768846	0.277041	0.158666	0.081105	0.435278	0.199961	0.376081	0.343345	0.327355	0.331093	0.306836	0.131984	0.287874	-0.15002	0.393874	-0.05932	0.332884	0.067298	0.263291	resistant
HGSOC14	0.350614096	0.337167	0.255035	0.068842	0.280626	-0.15564	0.292902	0.189734	0.226461	0.311671	0.387668	0.098138	0.229671	0.050318	0.099934	0.186835	0.518922	0.34794	0.389533	0.141939	0.233885	0.352957	0.252682	0.077278	0.089838	0.217683	0.307693	-0.08371	0.065173	0.109964	0.538232	0.704411	0.161239	0.093243	0.130539	0.518003	0.207722	0.435137	0.340819	0.259327	0.371709	0.431724	0.052861	0.242759	-0.03177	0.402008	-0.15762	0.36017	0.037608	0.33314	resistant
HGSOC15	0.341074891	0.294596	0.326368	0.226091	0.25763	-0.03268	0.281463	0.1898	0.280548	0.346587	0.351646	0.117715	0.343928	0.126938	0.120175	0.204378	0.490098	0.379504	0.399744	0.104021	0.208032	0.358129	0.222052	0.039013	-0.01091	0.283536	0.331705	-0.03766	0.060291	0.105923	0.564258	0.758328	0.261174	0.135769	-0.07977	0.480035	0.181865	0.436344	0.29491	0.279331	0.33078	0.493641	0.092293	0.256872	-0.03304	0.416973	-0.07798	0.359265	0.013495	0.2853	resistant
HGSOC16	0.383826588	0.15126	0.258677	-0.15509	0.249491	-0.2198	0.240672	0.09792	0.307848	0.083349	0.093445	0.127575	0.416291	-0.02208	0.133089	0.213637	0.524146	0.427049	0.436562	0.127076	0.197756	0.355161	0.206928	-0.07892	-0.0825	0.251952	0.269101	-0.01767	-0.1715	0.126391	0.522992	0.780199	0.430524	0.162037	0.007842	0.571495	0.147941	0.428698	0.320378	0.2647	0.303786	0.405395	0.188941	0.274851	-0.13499	0.380965	-0.05041	0.313396	-0.02383	0.245368	resistant
HGSOC17	0.353797965	0.299666	0.21996	0.083115	0.307803	0.013319	0.283683	0.133997	0.273391	0.283473	0.358353	0.093128	0.245285	0.05447	0.129398	0.230436	0.486606	0.326124	0.354364	0.185303	0.213796	0.364116	0.256467	0.066832	0.079409	0.194218	0.273441	0.025149	0.092655	0.128425	0.502286	0.698712	0.165071	0.181665	-0.00232	0.574651	0.223896	0.457071	0.283576	0.275572	0.381162	0.460025	-0.00978	0.229954	-0.02121	0.365265	-0.04762	0.339036	0.036822	0.27879	resistant
HGSOC18	0.323582664	0.37465	0.239145	0.002247	0.23366	-0.12128	0.320977	0.230692	0.184461	0.292145	0.419184	0.067676	0.244913	0.097081	0.039116	0.145587	0.509328	0.329647	0.353357	0.127486	0.315526	0.340741	0.262046	0.153708	0.16867	0.421895	0.482588	-0.01255	0.038089	0.108101	0.535339	0.67777	0.083268	0.135936	0.091322	0.53514	0.188894	0.580634	0.319144	0.280406	0.402271	0.472689	-0.0147	0.196346	0.031621	0.337807	-0.16217	0.325912	-0.03112	0.324499	resistant

Supplementary Table 8. Expression spectrum of the ssGSEA-transformed platinum dataset annotated by c7.all.v2023.2.Hs.symbols.gmt

	Antigen_Processing_and_Presentation	ıtimicrobi	gnalingPa	kine_Rec	hemokine	ine_Rece	Cytokines	nterleukin	ukins_Re	er_Cell_C	gnalingPa	Family_M	Group
HGSOC1	0.295520497	0.16644	-0.00807	-0.25433	0.12343	-0.12063	0.13866	0.07786	0.17958	0.1235	-0.0246	-0.53069	sensitive
HGSOC2	0.309493534	0.11844	-0.06444	-0.12462	-0.05127	-0.10993	0.04726	-0.04262	-0.38773	0.0471	-0.08112	-0.53143	sensitive
HGSOC3	0.298445268	0.16929	-0.0062	-0.07772	-0.03218	-0.14858	0.06512	0.03713	-0.28003	0.10604	-0.0351	-0.46092	sensitive
HGSOC4	0.333944605	0.2164	0.07793	-0.05974	0.07015	-0.1174	0.12924	0.12256	-0.13056	0.14342	-0.0004	-0.43529	sensitive
HGSOC5	0.33789036	0.19993	0.10314	-0.06289	0.05826	-0.14574	0.11446	0.09499	-0.38575	0.18953	0.06227	-0.38883	sensitive
HGSOC6	0.301399606	0.14293	-0.02436	-0.23757	0.0726	-0.19781	0.10453	0.00368	-0.32355	0.117	0.0132	-0.42099	sensitive
HGSOC7	0.304198394	0.20418	0.03491	-0.04911	0.2205	-0.11312	0.17754	0.07237	-0.12221	0.13103	0.0304	-0.33977	sensitive
HGSOC8	0.349370392	0.22232	0.11048	0.00314	0.13035	-0.13872	0.15518	0.12459	-0.40114	0.17207	0.02297	-0.39189	sensitive
HGSOC9	0.38257388	0.23027	0.102	0.03634	0.1245	-0.10803	0.16598	0.32189	-0.41828	0.18061	0.03089	-0.41387	sensitive
HGSOC10	0.356610584	0.24539	0.0975	-0.09608	0.18156	-0.10809	0.17128	0.09676	-0.34773	0.20319	0.06396	-0.33879	sensitive
HGSOC11	0.275729753	0.18584	-0.054	-0.23863	0.17639	-0.13575	0.13354	-0.04904	0.06352	0.03889	-0.06215	-0.53373	resistant
HGSOC12	0.311023064	0.19188	-0.04597	-0.31688	0.13659	-0.20419	0.13671	0.11224	-0.2209	0.06955	-0.05247	-0.16364	resistant
HGSOC13	0.226743398	0.10836	-0.10896	-0.17251	-0.03234	-0.08031	0.03276	0.05965	0.11166	-0.01865	-0.09038	-0.53637	resistant
HGSOC14	0.328577697	0.20801	0.02831	-0.11685	0.11099	-0.11267	0.13678	0.05129	-0.27651	0.10158	-0.00324	-0.47437	resistant
HGSOC15	0.325208979	0.21962	-0.03308	-0.16578	0.13495	-0.16756	0.13775	0.05464	-0.15276	0.09137	-0.06386	-0.45879	resistant
HGSOC16	0.265671533	0.10589	-0.12413	-0.21843	-0.08656	-0.11504	-0.02117	-0.1334	-0.61743	-0.01927	-0.09307	-0.58752	resistant
HGSOC17	0.289419427	0.18577	0.0335	-0.08804	0.07626	-0.12782	0.12563	0.06398	-0.21255	0.10089	-0.0236	-0.52999	resistant
HGSOC18	0.343804367	0.19099	0.10447	-0.04115	0.06248	-0.1197	0.09743	0.13329	-0.50049	0.1802	0.04478	-0.46404	resistant

Supplementary Table 9. Expression spectrum of the ssGSEA-transformed TCGA-GTEX dataset annotated by manual gene list of immue cells

	Activated CD4 T cell	Activated CD8 T cell	Activated dendritic cell	CD56bright natural killer cell	CD56dim natural killer cell	Central memory CD4 T cell	Central memory CD8 T cell	Effector memeory CD4 T cell	Effector memeory CD8 T cell	Gamma delta T cell
HGSOC1	-0.059339915	0.083342064	0.189208745	-0.03941635	0.084659794	0.222033404	0.300238817	-0.036090928	-0.039120601	0.202119712
HGSOC2	-0.225014066	-0.025114911	0.229772642	-0.026990466	0.058784703	0.227407838	0.312344595	0.002869468	-0.151192063	0.298959789
HGSOC3	-0.129099338	0.037021503	0.263962817	-0.036206539	0.012388896	0.243530139	0.306565724	-0.039518888	0.008594357	0.246780696
HGSOC4	-0.180964027	0.04574528	0.284176591	-0.022973472	0.14804039	0.256666747	0.382050692	-0.044671324	0.064211766	0.299890231
HGSOC5	-0.230504259	0.212978226	0.262793785	0.018733393	0.15628787	0.238238033	0.337592075	0.058862489	0.13508811	0.277382549
HGSOC6	-0.131752743	0.194084231	0.257866987	-0.080558151	0.017857644	0.232796924	0.284036087	-0.016574434	-0.002405421	0.340369847
HGSOC7	-0.011097857	0.161729856	0.29010424	-0.068601322	-0.040375065	0.248478514	0.346129532	0.003002879	0.084290428	0.266215057
HGSOC8	-0.197316983	0.027099449	0.281330147	0.076595384	0.16686713	0.280019662	0.390034831	0.019608402	0.078514313	0.315851672
HGSOC9	-0.225603478	0.086543214	0.292950196	0.017358216	0.092859106	0.290775362	0.427119857	0.014329302	0.197525546	0.276125807
HGSOC10	-0.025941059	0.240237757	0.28396541	0.006806279	0.141190943	0.259734769	0.402469756	0.027557753	0.171045866	0.263104483
HGSOC11	-0.020219369	0.06528576	0.247066357	0.015380258	-0.061984694	0.191503534	0.22000093	-0.089947812	-0.070968366	0.244217468
HGSOC12	-0.084629979	0.150306814	0.257812049	-0.056183312	0.019117959	0.226368923	0.311273039	-0.117984478	0.101440323	0.279310454
HGSOC13	-0.15711391	0.022934116	0.198100271	-0.008820316	0.138726689	0.101532956	0.26159519	-0.143744599	-0.139206426	0.263927951
HGSOC14	-0.123235365	0.105170294	0.236428063	0.001251097	0.150140868	0.225734517	0.353641276	-0.001306679	0.097421012	0.314034825
HGSOC15	-0.058636108	0.071168103	0.239332389	0.011925923	0.016958004	0.205344625	0.295669025	-0.03593179	-0.030047289	0.269054013
HGSOC16	-0.036108033	0.061764203	0.205997635	-0.061310871	-0.05817839	0.124803408	0.16197503	-0.15637303	0.018028099	0.290079013
HGSOC17	-0.17894349	0.079028865	0.20848031	0.016915938	0.117667294	0.263716449	0.341927162	-0.029613413	-0.052408659	0.289300205
HGSOC18	-0.231048747	0.13186929	0.302201259	0.001057208	0.138390653	0.249980787	0.396005353	0.053058998	0.14272208	0.247121487

	Immature B cell	Immature dendritic cell	Macrophage	Mast cell	MDSC	Monocyte	Natural killer cell	Natural killer T cell
HGSOC1	0.018967293	0.356489368	0.035728124	0.223295372	0.293637589	0.178630113	0.197823736	-0.035530979
HGSOC2	-0.10285846	0.33535007	-0.062206849	0.057623845	0.305147221	0.240235877	0.173287752	-0.007207953
HGSOC3	0.052580255	0.337028139	-0.001936321	0.099122004	0.300024058	0.200854466	0.183570366	0.015194177
HGSOC4	0.171142764	0.313366828	0.169927488	0.198837454	0.414125067	0.264667269	0.26516181	0.027471365
HGSOC5	0.15496854	0.285087363	0.118066221	0.24525947	0.342130423	0.255045384	0.192971956	-0.019744365
HGSOC6	-0.005839071	0.33629304	-0.10789664	0.156290935	0.249661586	0.216519821	0.106179989	-0.025318304
HGSOC7	0.07027933	0.337572304	0.015184189	0.072576154	0.33457229	0.161221878	0.190581482	-0.023559498
HGSOC8	0.193986883	0.340702726	0.242766473	0.265090251	0.449288564	0.309722156	0.239136705	0.044550632
HGSOC9	0.252482993	0.296542077	0.224608845	0.238011985	0.404131352	0.26382976	0.261892781	0.063569427
HGSOC10	0.235392728	0.285031881	0.227368208	0.238816605	0.429405135	0.285933452	0.257294778	0.020216766
HGSOC11	-0.075954895	0.36962989	-0.081614911	0.242836257	0.156474042	0.129559306	0.097059319	0.01417593
HGSOC12	-0.003096081	0.366677555	-0.081182448	0.139552319	0.212364121	0.168540647	0.087759924	-0.015618007
HGSOC13	-0.050043292	0.337458286	-0.257950459	-0.068807737	-0.010172526	0.152697078	0.139170499	0.008334985
HGSOC14	0.099296176	0.339517616	0.054367236	0.200146253	0.334746848	0.208754903	0.190347958	0.01275319
HGSOC15	0.083149018	0.383974615	0.006607495	0.222307364	0.27769532	0.239763357	0.18821359	-0.01062847
HGSOC16	-0.174602948	0.324214627	-0.245235104	-0.083225423	0.023283017	0.171013523	0.062245236	-0.04795374
HGSOC17	0.050672711	0.319091792	0.077298333	0.159154343	0.378483442	0.221844579	0.224580741	-0.003384723
HGSOC18	0.245195037	0.360170271	0.197348089	0.187192738	0.417959417	0.245258919	0.217061196	0.050846827

	Neutrophil	Plasmacytoid dendritic cell	Regulatory T cell	T follicular helper cell	Type 1 T helper cell	Type 17 T helper cell
HGSOC1	-0.404684417	0.151728326	-0.035977532	-0.07216535	0.063900319	-0.044524914
HGSOC2	-0.438752402	0.191965678	0.028761039	-0.047862114	0.030623572	0.012267595
HGSOC3	-0.385116989	0.133420557	0.087121385	0.022260689	0.065497969	0.046773503
HGSOC4	-0.362223065	0.221120981	0.313496338	0.095044249	0.103014778	0.010917612
HGSOC5	-0.217619263	0.161910671	0.088494763	0.039528376	0.081871224	0.117919959
HGSOC6	-0.432571372	0.118915326	-0.035983454	0.044957671	0.122929146	0.107364913
HGSOC7	-0.430124392	0.152986341	0.116649382	-0.028353274	0.115456401	-0.103526721
HGSOC8	-0.299356456	0.216888837	0.295717437	0.146473295	0.083413718	0.157128514
HGSOC9	-0.461605786	0.138619999	0.302885397	0.148478997	0.122429387	0.185825289
HGSOC10	-0.161528362	0.15087077	0.246967822	0.054646389	0.113735238	0.155923786
HGSOC11	-0.060033291	0.082779986	-0.147897849	-0.044124329	-0.002496076	-0.252982316
HGSOC12	-0.277063919	0.11997096	-0.0009011	-0.078347504	0.120590174	-0.071452436
HGSOC13	-0.550711436	0.119653979	-0.238812133	-0.034693877	-0.014399419	0.058514588
HGSOC14	-0.25414227	0.184359024	0.104588664	0.077998487	0.060668779	-0.008721622
HGSOC15	-0.083023974	0.187820895	-0.079370151	-0.046997067	-0.009022218	-0.048353996
HGSOC16	-0.27892095	0.095223114	-0.256057624	-0.088044764	-0.036280228	-0.184779414
HGSOC17	-0.387372676	0.191044711	0.152339414	0.008986582	0.055707255	-0.05036389
HGSOC18	-0.428326753	0.212935861	0.207103943	0.080943064	0.085329559	0.14453938

Supplementary Table 10. Immunohistochemical results

Patient ID	group	SH3YL1 tumor intensity	SH3YL1 tumor %	SH3YL1 tumor Hscore	CD4 total intensity	CD4 total %	CD4 total Hscore	DFS	DFS time (month)
HGSOC1	sensitive	2	30	60	1	60	60	1	18
HGSOC2	sensitive	1	30	30	1	15	15	1	29.8
HGSOC3	sensitive	2	80	160	1	40	40	1	12.6
HGSOC4	sensitive	2	60	120	1	20	20	1	16.1
HGSOC5	sensitive	2	55	110	1	35	35	1	20
HGSOC6	sensitive	1	50	50	1	40	40	1	24
HGSOC7	sensitive	2	70	140	1	20	20	1	40.1
HGSOC8	sensitive	2	60	120	1	40	40	1	18.7
HGSOC9	sensitive	1	20	20	1	20	20	1	29
HGSOC10	sensitive	1	20	20	1	60	60	1	13.6
HGSOC-expand1	sensitive	3	90	270	1	80	80	1	13
HGSOC-expand2	sensitive	3	80	240	1	5	5	1	13.3
HGSOC-expand3	sensitive	2	70	140	1	30	30	0	49
HGSOC-expand4	sensitive	2	60	120	1	15	15	1	45
HGSOC-expand5	sensitive	2	60	120	1	80	80	1	13
HGSOC-expand6	sensitive	2	80	160	2	40	80	0	37
HGSOC-expand7	sensitive	3	80	240	2	80	160	0	21
HGSOC-expand8	sensitive	3	80	240	1	60	60	1	13
HGSOC-expand9	sensitive	3	80	240	1	60	60	1	13
HGSOC-expand10	sensitive	3	80	240	2	60	120	0	61
HGSOC-expand11	sensitive	2	80	160	2	20	40	0	37
HGSOC-expand12	sensitive	3	60	180	2	30	60	1	19
HGSOC-expand13	sensitive	1	80	80	2	70	140	1	13
HGSOC-expand14	sensitive	2	70	140	1	80	80	1	23
HGSOC-expand15	sensitive	0	0	0	0	0	0	1	19
HGSOC-expand16	sensitive	2	70	140	1	40	40	0	32
HGSOC-expand17	sensitive	2	75	150	2	40	80	0	49
HGSOC-expand18	sensitive	1	70	70	1	40	40	1	13
HGSOC-expand19	sensitive	1	10	10	1	2	2	1	16
HGSOC-expand20	sensitive	2	30	60	2	70	140	1	47
HGSOC-expand21	sensitive	2	80	160	2	30	60	1	13
HGSOC-expand22	sensitive	1	60	60	2	60	120	1	17
HGSOC11	resistant	2	60	120	1	2	2	1	2.5
HGSOC12	resistant	3	60	180	2	20	40	1	3
HGSOC13	resistant	2	70	140	0	0	0	1	3
HGSOC14	resistant	2	80	160	2	20	40	1	5
HGSOC15	resistant	2	60	120	1	2	2	1	4.4
HGSOC16	resistant	2	80	160	0	0	0	1	0
HGSOC17	resistant	3	80	240	1	40	40	1	0
HGSOC18	resistant	1	30	30	1	20	20	1	4
HGSOC-expand23	resistant	3	80	240	1	40	40	1	5
HGSOC-expand24	resistant	3	80	240	2	60	120	1	5
HGSOC-expand25	resistant	2	80	160	1	5	5	1	5
HGSOC-expand26	resistant	3	80	240	2	20	40	1	4.2
HGSOC-expand27	resistant	3	80	240	2	20	40	1	4
HGSOC-expand28	resistant	0	0	0	0	0	0	1	5.1
HGSOC-expand29	resistant	3	80	240	2	70	140	1	0
HGSOC-expand30	resistant	3	80	240	1	70	70	1	2
HGSOC-expand31	resistant	1	50	50	2	70	140	1	5.6
HGSOC-expand32	resistant	1	30	30	1	20	20	1	5
HGSOC-expand33	resistant	2	70	140	1	60	60	1	2
HGSOC-expand34	resistant	3	70	210	1	20	20	1	5.7
HGSOC-expand35	resistant	3	80	240	1	10	10	1	3.2
HGSOC-expand36	resistant	3	75	225	1	40	40	1	0
HGSOC-expand37	resistant	2	70	140	2	20	40	1	5.7
HGSOC-expand38	resistant	2	75	150	1	40	40	1	0
HGSOC-expand39	resistant	2	70	140	1	70	70	1	0

Supplementary Table 11. List of Signature Genes used for cell type annotation

Signature Genes for ovary ascites				
**tissueType**	**cellName**	**geneSymbolmore1**	**geneSymbolmore2**	**shortName**
Ascites	B cells	CD79A,MS4A1,SDC1,CD19		B cells
Ascites	CD4+ T cells	CD3D,CD4	CD8A,CD8B	CD4+ T cells
Ascites	CD8+ T cells	CD3D,CD8A,CD8B	CD4	CD8+ T cells
Ascites	NK	NCAM1,NKG7		NK
Ascites	Monocyte	CD14		Monocyte
Ascites	Macrophage	CD86, HLA-DRA		Macrophage
Ascites	DC	HLA-DRA	CD14	DC
Ascites	Fibroblast	ACTA2,PDGFRA,COL1A2		Fibroblast
Ascites	Mesothelial cells	MSLN,UPK3B,WT1		Mesothelial cells
Ascites	Other stromal cells	CSPG4,TRPC6,PDGFRB		Other stromal cells
Ascites	Epithelial cells(Cancer cells)	EPCAM		Tumor cells
Ascites	HSC	CD34,KIT		HSC
Ascites	Proliferative cells	MKI67		Proliferative cells

Signature Genes for CD4$^+$ T cells				
**tissueType**	**cellName**	**geneSymbolmore1**	**geneSymbolmore2**	**shortName**
CD4$^+$ T cells	CD4+ naïve T cells	7,IL7R,SELL,PLAC8,GIMAP7,MAL,LDHB,GIMAP4,COTL1,DGKA		CD4 Tn
CD4$^+$ T cells	CD4+ follicular helper T cells	F1,CD40LG,CXCR5,IL6ST,TOX2,CXCL13,IL21		CD4 Tfh
CD4$^+$ T cells	CD4+ memory T cells	L16,GZMK,IFNGR1,GPR35,CD44,ZFP36,FTH1,SRGN,FAM177A1,TNFAI		CD4 Tm
CD4$^+$ T cells	CD4+ effector memory T cells	NFSF9,CCL3L3,IFNG,FASLG,CCL3,C	CCR7	CD4 Tem
CD4$^+$ T cells	CD4+ terminal effector memory T cell	CCL4,GZMA,GZMM,CCL3,FASLG,G	CD27,CD28,CCR7	CD4 Temra
CD4$^+$ T cells	CD4+ T helper 17 cells	R6,RORC,CEBPD,ID2,IL26,IL17F,IL17A,GZMA,CKLF,IL22,TNFSF14,CXCI		CD4 Th17
CD4$^+$ T cells	CD4+ T helper 1 cells	,GZMB,CCL3,GZMA,CCL5,CSF2,CXCR6,CCR5,CD4,CCL4L2		CD4 Th1
CD4$^+$ T cells	CD4+ T helper 2 cells	T6,IL4,IL5,IL13,CCR3,CCR8,PTGDR2,IL4R,IL9R,IL1RL1		CD4 Th2
CD4$^+$ T cells	CD4+ proliferating T cells	TOP2A,MKI67		CD4 prf
CD4$^+$ T cells	CD4+ regulatory T cells	3,TIGIT,IL10RA,CCR4,IL2RA,CD74,CTLA4		CD4 Treg
CD4$^+$ T cells	CD4+ Central memory T cells	LEF1,BACH2,KLF2,CD45RO,CD44,F	GZMA,GZMB	CD4 Tcm

Signature Genes for CD8$^+$ T cells				
**tissueType**	**cellName**	**geneSymbolmore1**	**geneSymbolmore2**	**shortName**
CD8$^+$ T cells	CD8+ naïve T cells	,TXK,BACH2,LTB,CCR7,IL7R,IL6R,IFNGR2,SELL,MAL		CD8 Tn
CD8$^+$ T cells	CD8+ memory T cells	4,ZFP36,TNFAIP3,GPR183,CCL5,IL32,GZMA,TNF,CKLF,CD52,SIT1		CD8 Tm
CD8$^+$ T cells	CD8+ effector memory T cells	CL4,CCR5,CXCR3,HLA-DRB1,HLA-D	CCR7,CD45RA	CD8 Tem
CD8$^+$ T cells	CD8+ terminal effector memory T cell	2,TBX21,GZMB,GZMM,GZMA,CX3CR1,GNLY,NKG7,KLRD1		CD8 Temra
CD8$^+$ T cells	CD8+ tissue-resident memory T cells	GZMB,XCL1,CCL5,GZMA,XCL2,CXCR6,CXCR3,LGALS3,SH3BGRL3,LGA		CD8 Trm
CD8$^+$ T cells	CD8+ exhausted T cells	,TOX2,FAM3C,GZMB,CSF1,CD70,FASLG,LAYN,ENTPD1,KRT86,TNFRS		CD8 Tex
CD8$^+$ T cells	CD8+ effector T cells	D8A,GNLY,GZMA,GZMK,GZMB,GZMH		CD8 Tef
CD8$^+$ T cells	Tc17	NR1D1,CCL20,IL17A,TNF,CCR6,IL23R,IL7R,IL17RE,IL18RAP,KLRB1		Tc17
CD8$^+$ T cells	CD8+ Central memory T cells	CD44, IL7R, TCF7, LEF1, CD27, BACH2, KLF2,CD28,ICOS		CD8 Tcm

Signature Genes for B cells				
**tissueType**	**cellName**	**geneSymbolmore1**	**geneSymbolmore2**	**shortName**
B cells	Follicular B cells	1,CD23,CD279,CD360,IGD,IGM,IKAROS,PAX5,CXCR4		B Fol
B cells	proliferating B cells	SPM,CENPF,CCNB2,CDC20,PLK1,STMN1,MYBL1,HMGB2,MEF2B		B prf
B cells	Memory B cells	FRSF13B,FCRL4,FCRL5,RGS1,FCRL3,CCR7,GPR183,BANK1,BLK		B m
B cells	naïve B cells	A,FCER2,IL4R,CD72,BACH2,RFTN1,CR2		B n
B cells	germinal center B cells	CDA,MKI67,PAX5,RGS13,MEF2B,CXCR4,CD83,CD10,CD38,CD95,IRF4,F		GCB
B cells	Plasma cells	SSR4,PRDX4,RRBP1,FKBP2,TXNDC	CD19,CD24,CD93,CD40	Plasma cells
B cells	Regulatory B cells	L35,CD19,CD20,CD25,PRDM1,CD96,OAS1,CSH1,FCRL5,BTLA,IRF4,FCF		Bregs
B cells	Plasmablasts	TNFRSF17,TNFRSF13B,FCRL5,IGH,	,CD19,CD20,PAX5,BCL6,FKBP11,S	Plasmablasts

Signature Genes for Macrophage				
**tissueType**	**cellName**	**geneSymbolmore1**	**geneSymbolmore2**	**shortName**
Macrophage	Macrophage cells	D68,CD14,PLTP,C1QB, C1QC, APOE		Macrophage cells
Macrophage	M2-like macrophage	CD163,CD206,MRC1,ARG1		M2-like macrophage
Macrophage	M1-like macrophage	NLRP3,IL1B,CD86,IRF5,TNF, S100A8,FCN1,LYZ,VCAN		M1-like macrophage
Macrophage	M2a macrophage	ARG,HLA-DRB5,STAT6,CD200R1,CLEC10A,IL10,TGFB1,CLEC10A		M2a Φs
Macrophage	M2b macrophage	,CD86,HLA-DRA,TLR2,TLR4,FCGR2A,FCGR3A,CCL1		M2b Φs
Macrophage	M2c macrophage	6,MRC1,IL10,TGFB1,TLR1,MARCO,CD14,MERTK		M2c Φs
Macrophage	M2d macrophage	L16,PD-L1,CD163,CD274,MMP9,IL10,IL12,RANTES,CXCL10,CXCL16		M2d Φs
Macrophage	M0 macrophage	CD68,CD11b,ITGAM,CSF1R,FCGR3A,LYZ		M0 Φs
Macrophage	TAM	3,CSF1R,CCR2,VEGFA,CCL2,C1QA,C1QC,C1QB,APOC1		Tumor-Associated Macrophages
Macrophage	LAM	POE,TREM2,ABCA1,PLIN2,SPP1,LPL		Lipid-Associated Macrophages

Supplementary Table 12. Kyoto Encyclopedia of Genes and Genomes enrichment analysis of upregulated lipid process related genes in the platinum-resistant group

ID	Description	GeneRatio	BgRatio	pvalue	p.adjust	qvalue	geneID	Count	Fold_enrichment
hsa04146	Peroxisome	14366	83/8521	3.4E-05	0.00355	0.00327	23417/10654/8309/4598/3155	5	3.718087584
hsa00100	Steroid biosynthesis	14305	20/8521	9.6E-05	0.00493	0.00454	6713/2222/10682	3	5.034303768
hsa00280	ine and isoleucine	14305	48/8521	0.00133	0.04566	0.04199	549/594/3155	3	3.771885579
hsa00900	oid backbone biosy	14277	23/8521	0.00486	0.11443	0.10525	10654/4598	2	4.247927513
hsa00563	idylinositol (GPI)-ar	14277	26/8521	0.00619	0.11443	0.10525	84720/54872	2	4.071247819
hsa00650	utanoate metabolis	14277	27/8521	0.00667	0.11443	0.10525	622/3155	2	4.016139703
hsa00640	opanoate metabolis	14277	32/8521	0.00928	0.13662	0.12566	23417/594	2	3.771885579
hsa00780	Biotin metabolism	14246	2418332	0.01367	0.176	0.16188	54995	1	6.18646094
hsa00510	-Glycan biosynthes	14277	53/8521	0.02429	0.27796	0.25566	79053/79644	2	3.042644337
hsa00140	id hormone biosynt	14277	62/8521	0.03248	0.33459	0.30775	79154/79644	2	2.817623258
hsa00920	Sulfur metabolism	14246	2418546	0.04486	0.38911	0.35789	10380	1	4.449561375
hsa03320	'AR signaling pathw	14277	75/8521	0.04594	0.38911	0.35789	10580/8309	2	2.543495883
hsa00980	xenobiotics by cyt	14277	78/8521	0.04929	0.38911	0.35789	4259/29785	2	2.485426827
hsa00533	ycan biosynthesis -	14246	14/8521	0.06225	0.38911	0.35789	6482	1	3.964398632
hsa04211	evity regulating pat	14277	89/8521	0.06231	0.38911	0.35789	8660/1386	2	2.295723025
hsa00604	lipid biosynthesis -	14246	15/8521	0.06655	0.38911	0.35789	6482	1	3.864928972
hsa00603	iosynthesis - globo	14246	16/8521	0.07083	0.38911	0.35789	6482	1	3.771885579
hsa04070	tidylinositol signalin	14277	97/8521	0.07243	0.38911	0.35789	8396/90809	2	2.169925001
hsa00120	iry bile acid biosynt	14246	17/8521	0.07509	0.38911	0.35789	8309	1	3.683696454
hsa04151	<-Akt signaling path	14336	354/8521	0.07703	0.38911	0.35789	3696/117145/2066/1386	4	1.304511042
hsa00061	atty acid biosynthes	14246	18/8521	0.07933	0.38911	0.35789	54995	1	3.601696516
hsa00785	poic acid metabolis	14246	19/8521	0.08356	0.39119	0.35981	594	1	3.523561956
hsa04668	NF signaling pathw	14277	114/8521	0.09552	0.42775	0.39344	153090/1386	2	1.937344392
hsa04935	ne synthesis, secre	14277	120/8521	0.10412	0.43496	0.40006	8660/1386	2	1.86393845
hsa04152	IPK signaling pathv	14277	121/8521	0.10557	0.43496	0.40006	23417/8660	2	1.851998837
hsa00062	Fatty acid elongatio	14246	27/8521	0.11666	0.46217	0.42509	117145	1	3.016139703
hsa04910	ulin signaling pathv	14277	137/8521	0.12955	0.4716	0.43376	10580/8660	2	1.673556424
hsa04915	ogen signaling path	14277	137/8521	0.12955	0.4716	0.43376	10499/1386	2	1.673556424
hsa00410	a-Alanine metaboli	14246	31/8521	0.13278	0.4716	0.43376	23417	1	2.817623258
hsa00512	ype O-glycan biosy	14246	36/8521	0.15252	0.52365	0.48164	6482	1	2.601696516
hsa04022	P-PKG signaling pa	14277	167/8521	0.17739	0.56974	0.52404	8660/1386	2	1.389566812
hsa04930	pe II diabetes mellit	14246	46/8521	0.1907	0.56974	0.52404	8660	1	2.247927513
hsa05030	Cocaine addiction	14246	49/8521	0.20182	0.56974	0.52404	1386	1	2.15704371
hsa00565	ther lipid metabolis	14246	50/8521	0.20549	0.56974	0.52404	79153	1	2.12763328
hsa05134	Legionellosis	14246	56/8521	0.2272	0.56974	0.52404	1917	1	1.963474124
hsa00480	utathione metabolis	14246	57/8521	0.23076	0.56974	0.52404	4259	1	1.937344392
hsa01212	atty acid metabolis	14246	57/8521	0.23076	0.56974	0.52404	54995	1	1.937344392
hsa04923	on of lipolysis in ad	14246	58/8521	0.2343	0.56974	0.52404	8660	1	1.914564523
hsa00590	hidonic acid metab	14246	61/8521	0.24484	0.56974	0.52404	145482	1	1.839959587
hsa04213	ılating pathway - m	14246	61/8521	0.24484	0.56974	0.52404	8660	1	1.839959587
hsa05207	cinogenesis - recep	14277	212/8521	0.25311	0.56974	0.52404	4259/1386	2	1.042644337
hsa04927	ol synthesis and se	14246	65/8521	0.25867	0.56974	0.52404	1386	1	1.748461233
hsa00830	Retinol metabolism	14246	68/8521	0.26888	0.56974	0.52404	29785	1	1.682573297
hsa04920	ytokine signaling p	14246	69/8521	0.27226	0.56974	0.52404	8660	1	1.66448284
hsa05031	nphetamine addicti	14246	69/8521	0.27226	0.56974	0.52404	1386	1	1.66448284
hsa05204	carcinogenesis - DN	14246	69/8521	0.27226	0.56974	0.52404	4259	1	1.66448284
hsa05230	arbon metabolism	14246	70/8521	0.27561	0.56974	0.52404	5163	1	1.641546029
hsa04810	ation of actin cytosk	14277	229/8521	0.28217	0.56974	0.52404	3696/8396	2	0.933572638
hsa00982	tabolism - cytochro	14246	72/8521	0.28228	0.56974	0.52404	4259	1	1.599317794
hsa00562	ol phosphate metab	14246	73/8521	0.2856	0.56974	0.52404	8396	1	1.580145484
hsa01524	itinum drug resistar	14246	73/8521	0.2856	0.56974	0.52404	4259	1	1.580145484
hsa04918	roid hormone synth	14246	75/8521	0.29218	0.56974	0.52404	1386	1	1.541019153
hsa05140	Leishmaniasis	14246	77/8521	0.2987	0.56974	0.52404	1917	1	1.50589093
hsa05412	c right ventricular c	14246	77/8521	0.2987	0.56974	0.52404	3696	1	1.50589093
hsa00983	etabolism - other e	14246	80/8521	0.30837	0.5775	0.53117	4259	1	1.448900951
hsa04012	bB signaling pathw	14246	85/8521	0.32421	0.59014	0.5428	2066	1	1.361768359
hsa04911	Insulin secretion	14246	86/8521	0.32733	0.59014	0.5428	1386	1	1.344828497
hsa04512	M-receptor interact	14246	89/8521	0.33662	0.59014	0.5428	3696	1	1.292781749
hsa05410	rtrophic cardiomyo	14246	90/8521	0.33969	0.59014	0.5428	3696	1	1.280956314
hsa04520	Adherens junction	14246	93/8521	0.34881	0.59014	0.5428	10580	1	1.232660757
hsa05414	lated cardiomyopat	14246	96/8521	0.35781	0.59014	0.5428	3696	1	1.189033824
hsa04925	one synthesis and	14246	98/8521	0.36374	0.59014	0.5428	1386	1	1.15704371
hsa05231	ne metabolism in c	14246	98/8521	0.36374	0.59014	0.5428	1120	1	1.15704371
hsa00564	ophospholipid meta	14246	99/8521	0.36669	0.59014	0.5428	1120	1	1.14404637
hsa04928	ione synthesis, sec	14246	106/8521	0.38694	0.59028	0.54292	1386	1	1.042644337
hsa04922	agon signaling path	14246	107/8521	0.38978	0.59028	0.54292	1386	1	1.028569152
hsa03013	eocytoplasmic tran	14246	108/8521	0.39261	0.59028	0.54292	1917	1	1.014355293
hsa04931	Insulin resistance	14246	108/8521	0.39261	0.59028	0.54292	8660	1	1.014355293
hsa04066	F-1 signaling pathw	14246	109/8521	0.39543	0.59028	0.54292	5163	1	1
hsa04010	PK signaling pathv	14277	301/8521	0.40306	0.59307	0.54549	2066/1386	2	0.5360529
hsa04919	hormone signaling	14246	121/8521	0.42824	0.62125	0.57141	10499	1	0.855989697
hsa04926	axin signaling path	14246	129/8521	0.44914	0.62782	0.57745	1386	1	0.757023247
hsa04068	xO signaling pathw	14246	131/8521	0.45425	0.62782	0.57745	8660	1	0.739848103
hsa04728	opaminergic synap	14246	132/8521	0.45679	0.62782	0.57745	1386	1	0.731183242
hsa04210	Apoptosis	14246	136/8521	0.46682	0.62782	0.57745	153090	1	0.687060688
hsa05418	ar stress and ather	14246	139/8521	0.47423	0.62782	0.57745	4259	1	0.650764559
hsa04140	Autophagy - anima	14246	141/8521	0.47911	0.62782	0.57745	8660	1	0.632268215
hsa04120	iitin mediated prote	14246	142/8521	0.48153	0.62782	0.57745	10273	1	0.622930351
hsa04936	lcoholic liver disea	14246	142/8521	0.48153	0.62782	0.57745	23417	1	0.622930351
hsa01240	osynthesis of cofact	14246	153/8521	0.50748	0.63545	0.58447	54995	1	0.516015147
hsa04261	c signaling in cardi	14246	154/8521	0.50978	0.63545	0.58447	1386	1	0.50589093
hsa04932	lcoholic fatty liver d	14246	155/8521	0.51206	0.63545	0.58447	8660	1	0.495695163
hsa04934	Cushing syndrome	14246	155/8521	0.51206	0.63545	0.58447	1386	1	0.495695163
hsa04514	ll adhesion molecu	14246	158/8521	0.51886	0.63622	0.58518	3696	1	0.464668267
hsa05161	Hepatitis B	14246	162/8521	0.52777	0.63953	0.58823	1386	1	0.432959407
hsa05225	patocellular carcino	14246	168/8521	0.54084	0.64775	0.59579	4259	1	0.378511623
hsa04141	essing in endoplasr	14246	173/8521	0.55146	0.65288	0.6005	10273	1	0.333423734
hsa05034	Alcoholism	14246	188/8521	0.58191	0.6811	0.62646	1386	1	0.214124805
hsa04510	Focal adhesion	14246	203/8521	0.61034	0.6874	0.63225	3696	1	0.111031312
hsa05415	abetic cardiomyopa	14246	203/8521	0.61034	0.6874	0.63225	5164	1	0.111031312
hsa05203	Viral carcinogenesis	14246	204/8521	0.61217	0.6874	0.63225	1386	1	0.097610797
hsa05205	oteoglycans in canc	14246	205/8521	0.61399	0.6874	0.63225	2066	1	0.097610797
hsa05166	cell leukemia virus	14246	222/8521	0.64366	0.70329	0.64687	1386	1	-0.029146346
hsa05208	ogenesis - reactive	14246	223/8521	0.64534	0.70329	0.64687	4259	1	-0.029146346
hsa05163	cytomegalovirus ir	14246	225/8521	0.64866	0.70329	0.64687	1386	1	-0.043943348
hsa04714	Thermogenesis	14246	232/8521	0.66007	0.7082	0.65138	1386	1	-0.089267338
hsa04020	cium signaling path	14246	253/8521	0.69216	0.73497	0.67601	2066	1	-0.217591435
hsa05020	Prion disease	14246	272/8521	0.71864	0.7553	0.69471	1386	1	-0.321928095
hsa05206	MicroRNAs in cance	14246	310/8521	0.7651	0.79601	0.73215	8660	1	-0.514573173
hsa05165	n papillomavirus int	14246	331/8521	0.78748	0.8111	0.74603	3696	1	-0.59946207
hsa05014	otrophic lateral scle	14246	364/8521	0.8185	0.82915	0.76263	2066	1	-0.736965594
hsa04080	ve ligand-receptor i	14246	367/8521	0.8211	0.82915	0.76263	2908	1	-0.736965594
hsa05010	Alzheimer disease	14246	384/8521	0.83511	0.83511	0.76812	8660	1	-0.810966176

Supplementary Table 13. Kyoto Encyclopedia of Genes and Genomes enrichment analysis of upregulated lipid process related genes in the platinum-resistant group

ID	Description	GeneRatio	BgRatio	pvalue	p.adjust	qvalue	geneID	Count	Fold_enrichment
hsa04664	ilon RI signaling pa	11871	68/8521	4.3E-09	8.8E-07	5E-07	4067/240/3635/1432/241/5293/5335	7	4.776630423
hsa04933	ing pathway in diab	11871	100/8521	6.6E-08	6.7E-06	3.8E-06	776/4790/5330/5580/1432/5293/533	7	4.220329955
hsa04750	ediator regulation o	11841	98/8521	1.4E-06	9.6E-05	5.4E-05	5330/6714/5580/1432/5293/5335	6	4.026800059
hsa04917	actin signaling path	11810	70/8521	5.5E-06	0.00016	9.2E-05	6776/4790/6714/1432/5293	5	4.249445341
hsa05120	naling in Helicobact	11810	70/8521	5.5E-06	0.00016	9.2E-05	4067/4790/6714/1432/5335	5	4.249445341
hsa04062	okine signaling pa	11871	192/8521	5.5E-06	0.00016	9.2E-05	067/4790/5330/6714/5580/5293/533	7	3.279471296
hsa04611	Platelet activation	11841	124/8521	5.6E-06	0.00016	9.2E-05	4067/5330/6714/6916/1432/5293	6	3.687060688
hsa05417	d and atheroscler	11871	215/8521	1.2E-05	0.0003	0.00017	067/4790/5330/6714/1432/5293/533	7	3.116031993
hsa04666	a R-mediated phag	11810	97/8521	2.7E-05	0.00058	0.00033	4067/3635/5580/5293/5335	5	3.77925972
hsa05131	Shigellosis	11871	247/8521	2.9E-05	0.00058	0.00033	790/5330/6714/5580/1432/5293/533	7	2.916476644
hsa04979	holesterol metabolis	11780	51/8521	3.6E-05	0.00065	0.00036	1593/4035/10577/336	4	4.384049807
hsa04625	in receptor signalir	11810	104/8521	3.8E-05	0.00065	0.00036	4790/6714/5580/1432/5293	5	3.678071905
hsa01212	atty acid metabolis	11780	57/8521	5.6E-05	0.00086	0.00048	35/2180/401494/3295	4	4.224194664
hsa04370	GF signaling pathw	11780	59/8521	6.5E-05	0.00086	0.00048	6714/1432/5293/5335	4	4.173926932
hsa04613	l extracellular trap	11841	191/8521	6.5E-05	0.00086	0.00048	4790/5330/6714/1432/5293/5335	6	3.063502942
hsa05167	a-associated herpe	11841	194/8521	7.1E-05	0.00086	0.00048	4067/4790/6714/1432/5293/5335	6	3.042644337
hsa04722	rophin signaling pa	11810	119/8521	7.3E-05	0.00086	0.00048	4790/5580/1432/5293/5335	5	3.484138131
hsa04935	ne synthesis, secre	11810	120/8521	7.6E-05	0.00086	0.00048	6776/5330/1432/5293/5335	5	3.472487771
hsa05203	Viral carcinogenesis	11841	204/8521	9.4E-05	0.00101	0.00057	4067/6776/55697/4790/6714/5293	6	2.969012308
hsa04926	axin signaling path	11810	129/8521	0.00011	0.00109	0.00061	4790/5330/6714/1432/5293	5	3.367371066
hsa05135	Yersinia infection	11810	137/8521	0.00014	0.00131	0.00074	4790/6714/1432/5293/5335	5	3.280956314
hsa00562	ol phosphate metal	11780	73/8521	0.00015	0.00131	0.00074	5330/3635/5293/5335	4	3.866907978
hsa05418	ar stress and ather	11810	139/8521	0.00015	0.00131	0.00074	4790/6714/1432/5293/857	5	3.260025656
hsa05208	ogenesis - reactive	11841	223/8521	0.00015	0.00131	0.00074	4790/6714/5580/847/1432/5293	6	2.839959587
hsa03320	AR signaling pathw	11780	75/8521	0.00017	0.00135	0.00076	1593/2180/336/10999	4	3.827819025
hsa04662	eceptor signaling p	11780	84/8521	0.00026	0.00201	0.00113	4067/4790/3635/5293	4	3.66448284
hsa04012	oB signaling pathw	11780	85/8521	0.00027	0.00203	0.00114	6776/6714/5293/5335	4	3.64731451
hsa05161	Hepatitis B	11810	162/8521	0.00031	0.00225	0.00127	6776/4790/6714/1432/5293	5	3.039138394
hsa05235	nd PD-1 checkpoir	11780	89/8521	0.00032	0.00225	0.00127	4790/1432/5293/5335	4	3.581351247
hsa04658	nd Th2 cell differen	11780	92/8521	0.00036	0.00247	0.00139	6776/4790/1432/5335	4	3.533563348
hsa04912	RH signaling pathw	11780	93/8521	0.00038	0.00249	0.0014	5330/6714/5580/1432	4	3.517275693
hsa04070	idylinositol signalir	11780	97/8521	0.00044	0.00283	0.00159	5330/3635/5293/5335	4	3.456806149
hsa05142	Chagas disease	11780	102/8521	0.00054	0.00333	0.00187	4790/5330/1432/5293	4	3.384049807
hsa04620	receptor signaling	11780	104/8521	0.00058	0.00347	0.00195	4790/1432/5293/6696	4	3.35614381
hsa04659	17 cell differentiati	11780	108/8521	0.00067	0.00378	0.00213	6776/4790/1432/5335	4	3.301587647
hsa04931	Insulin resistance	11780	108/8521	0.00067	0.00378	0.00213	4790/5580/5293/10999	4	3.301587647
hsa05415	betic cardiomyopa	11810	203/8521	0.00087	0.0048	0.0027	4790/5330/5580/1432/5293	5	2.713695815
hsa05205	oteoglycans in can	11810	205/8521	0.00091	0.00489	0.00275	6714/1432/5293/857/5335	5	2.698218478
hsa04015	p1 signaling pathw	11810	210/8521	0.00101	0.00497	0.00279	5330/6714/1432/5293/5335	5	2.66448284
hsa04071	golipid signaling pa	11780	121/8521	0.00102	0.00497	0.00279	4790/5330/1432/5293	4	3.137503524
hsa04660	eceptor signaling p	11780	121/8521	0.00102	0.00497	0.00279	4790/1432/5293/5335	4	3.137503524
hsa04919	hormone signaling	11780	121/8521	0.00102	0.00497	0.00279	5330/6714/5293/5335	4	3.137503524
hsa05163	cytomegalovirus ir	11810	225/8521	0.00138	0.00655	0.00369	4790/5330/6714/1432/5293	5	2.565597176
hsa04915	ogen signaling path	11780	137/8521	0.00162	0.00752	0.00423	5330/6714/5580/5293	4	2.957914599
hsa04929	GnRH secretion	11749	64/8521	0.00172	0.00778	0.00438	5330/5293/6696	3	3.641546029
hsa00120	ry bile acid biosynt	11720	17/8521	0.00179	0.00796	0.00447	1593/3295	2	4.969472865
hsa05221	ute myeloid leuker	11749	67/8521	0.00196	0.0085	0.00478	6776/4790/5293	3	3.575312331
hsa05223	-small cell lung ca	11749	72/8521	0.00241	0.01023	0.00575	6776/5293/5335	3	3.472487771
hsa05220	onic myeloid leuke	11749	76/8521	0.00281	0.01169	0.00658	6776/4790/5293	3	3.393690764
hsa05100	l invasion of epithe	11749	77/8521	0.00291	0.01189	0.00669	6714/5293/857	3	3.374343989
hsa01521	sine kinase inhibito	11749	79/8521	0.00314	0.01254	0.00705	6714/5293/5335	3	3.337711092
hsa04146	Peroxisome	11749	83/8521	0.00361	0.01415	0.00796	847/2180/3295	3	3.266036894
hsa04211	evity regulating pat	11749	89/8521	0.00439	0.01689	0.0095	4790/847/5293	3	3.166715445
hsa01040	sis of unsaturated	11720	27/8521	0.00452	0.01709	0.00961	401494/3295	2	4.301587647
hsa04621	receptor signaling	11780	186/8521	0.0049	0.01818	0.01022	4790/5330/5580/1432	4	2.518535139
hsa01522	ndocrine resistanc	11749	98/8521	0.00575	0.02093	0.01177	6714/1432/5293	3	3.026800059
hsa05146	Amoebiasis	11749	102/8521	0.00642	0.02298	0.01292	4790/5330/5293	3	2.969012308
hsa05169	tein-Barr virus infe	11780	202/8521	0.00655	0.02304	0.01296	4067/4790/1432/5293	4	2.397802962
hsa04510	Focal adhesion	11780	203/8521	0.00667	0.02304	0.01296	6714/5293/6696/857	4	2.392317423
hsa04064	ppa B signaling pa	11749	104/8521	0.00678	0.02304	0.01296	4067/4790/5335	3	2.941106311
hsa04066	F-1 signaling pathw	11749	109/8521	0.00771	0.02512	0.01413	4790/5293/5335	3	2.873813198
hsa05170	unodeficiency viru	11780	212/8521	0.00776	0.02512	0.01413	4790/1432/5293/5335	4	2.327687364
hsa05207	cinogenesis - recep	11780	212/8521	0.00776	0.02512	0.01413	6776/4790/6714/5293	4	2.327687364
hsa05145	Toxoplasmosis	11749	111/8521	0.00811	0.02584	0.01454	240/4790/1432	3	2.847996907
hsa04668	NF signaling pathw	11749	114/8521	0.00872	0.02738	0.0154	4790/1432/5293	3	2.809414444
hsa04670	e transendothelial	11749	115/8521	0.00893	0.02761	0.01553	1432/5293/5335	3	2.797012978
hsa05166	cell leukemia virus	11780	222/8521	0.0091	0.0277	0.01558	6776/55697/4790/5293	4	2.263034406
hsa05171	virus disease - CC	11780	232/8521	0.01058	0.03175	0.01786	4790/1432/5293/5335	4	2.198494154
hsa00071	atty acid degradati	11720	43/8521	0.01121	0.03314	0.01864	35/2180	2	3.631104282
hsa04380	teoclast differentiat	11749	128/8521	0.01195	0.03483	0.01959	4790/1432/5293	3	2.641546029
hsa04068	xO signaling pathw	11749	131/8521	0.01272	0.03615	0.02033	847/1432/5293	3	2.608809243
hsa04930	pe II diabetes melli	11720	46/8521	0.01276	0.03615	0.02033	5580/5293	2	3.533563348
hsa04973	rate digestion and	11720	47/8521	0.01329	0.03715	0.0209	5330/5293	2	3.502075956
hsa05162	Measles	11749	138/8521	0.01463	0.04033	0.02268	6776/4790/5293	3	2.533563348
hsa04072	lipase D signaling	11749	148/8521	0.01762	0.0473	0.02661	5330/5293/5335	3	2.432959407
hsa04723	e endocannabinoi	11749	148/8521	0.01762	0.0473	0.02661	5330/1432/221955	3	2.432959407
hsa05020	Prion disease	11780	272/8521	0.01808	0.0479	0.02694	5580/1432/5293/857	4	1.970853654
hsa04932	coholic fatty liver d	11749	155/8521	0.01991	0.05207	0.02928	4790/1432/5293	3	2.364572432
hsa04218	Cellular senescenc	11749	156/8521	0.02025	0.05228	0.02941	4790/1432/5293	3	2.35614381
hsa04730	ong-term depressic	11720	60/8521	0.02112	0.05386	0.03029	4067/5330	2	3.150559677
hsa00590	nidonic acid metab	11720	61/8521	0.02179	0.0542	0.03048	240/6916	2	3.125981654
hsa04213	ulating pathway - m	11720	61/8521	0.02179	0.0542	0.03048	847/5293	2	3.125981654
hsa04920	ytokine signaling p	11720	69/8521	0.02742	0.06738	0.0379	4790/2180	2	2.948600847
hsa04622	receptor signaling	11720	71/8521	0.02891	0.0702	0.03948	4790/1432	2	2.906890596
hsa05152	Tuberculosis	11749	180/8521	0.02935	0.07045	0.03962	4790/6714/1432	3	2.150559677
hsa04360	Axon guidance	11749	182/8521	0.0302	0.07163	0.04029	6714/5293/5335	3	2.13422094
hsa05022	rodegeneration - m	11810	476/8521	0.03076	0.07212	0.04057	4790/5330/847/1432/5335	5	1.485426827
hsa05214	Glioma	11720	75/8521	0.03198	0.07414	0.0417	5293/5335	2	2.827819025
hsa05133	Pertussis	11720	76/8521	0.03277	0.07428	0.04178	4790/1432	2	2.809414444
hsa05212	Pancreatic cancer	11720	76/8521	0.03277	0.07428	0.04178	4790/5293	2	2.809414444
hsa05140	Leishmaniasis	11720	77/8521	0.03357	0.07525	0.04232	4790/1432	2	2.790772038
hsa05130	nic Escherichia coli	11749	198/8521	0.0374	0.08293	0.04664	4790/6714/1432	3	2.010779839
hsa04540	Gap junction	11720	88/8521	0.04281	0.09391	0.05282	5330/6714	2	2.596935142
hsa05222	mall cell lung canc	11720	92/8521	0.04639	0.10067	0.05662	4790/5293	2	2.533563348
hsa04657	17 signaling pathw	11720	94/8521	0.04821	0.10353	0.05823	4790/1432	2	2.503348735
hsa05215	Prostate cancer	11720	97/8521	0.051	0.10813	0.06082	4790/5293	2	2.456806149
hsa04925	one synthesis and	11720	98/8521	0.05195	0.10813	0.06082	5330/221955	2	2.440952198
hsa05231	ne metabolism in c	11720	98/8521	0.05195	0.10813	0.06082	5293/5335	2	2.440952198
hsa05010	Alzheimer disease	11780	384/8521	0.05397	0.11122	0.06255	4790/5330/4035/5293	4	1.469885976
hsa00604	ipid biosynthesis -	11689	15/8521	0.05492	0.11203	0.06301	3073	1	4.14974712
hsa04914	ne-mediated oocyte	11720	102/8521	0.05578	0.11266	0.06337	1432/5293	2	2.384049807
hsa04014	as signaling pathw	11749	236/8521	0.05772	0.11544	0.06493	4790/5293/5335	3	1.757023247
hsa00603	osynthesis - globo	11689	16/8521	0.05847	0.11581	0.06514	3073	1	4.056583528
hsa00061	tty acid biosynthes	11689	18/8521	0.06555	0.12621	0.07098	2180	1	3.886550147
hsa00511	er glycan degrada	11689	18/8521	0.06555	0.12621	0.07098	3073	1	3.886550147
hsa05132	Salmonella infectio	11749	249/8521	0.06567	0.12621	0.07098	4790/1432/5293	3	1.682573297
hsa04144	Endocytosis	11749	250/8521	0.06631	0.12621	0.07098	6714/1601/857	3	1.678071905
hsa04725	Cholinergic synaps	11720	113/8521	0.06682	0.12621	0.07098	5330/5293	2	2.23572706
hsa01200	Carbon metabolism	11720	115/8521	0.0689	0.12693	0.07139	847/35	2	2.211012193
hsa04726	erotonergic synaps	11720	115/8521	0.0689	0.12693	0.07139	240/5330	2	2.211012193
hsa00531	aminoglycan degra	11689	19/8521	0.06906	0.12693	0.07139	3073	1	3.808385051
hsa00100	Steroid biosynthesi	11689	20/8521	0.07257	0.13217	0.07434	3930	1	3.734438666
hsa04142	Lysosome	11720	132/8521	0.08741	0.15505	0.08721	10577/3073	2	2.010779839
hsa04650	ler cell mediated c	11720	132/8521	0.08741	0.15505	0.08721	5293/5335	2	2.010779839
hsa04728	opaminergic synap	11720	132/8521	0.08741	0.15505	0.08721	5330/1432	2	2.010779839
hsa04270	smooth muscle co	11720	134/8521	0.08968	0.15771	0.0887	5330/5580	2	1.989139007
hsa04210	Apoptosis	11720	136/8521	0.09196	0.16035	0.09018	4790/5293	2	1.970853654
hsa04371	elin signaling pathw	11720	139/8521	0.09543	0.16446	0.0925	5330/6696	2	1.937344392
hsa00062	atty acid elongatio	11689	27/8521	0.09674	0.16446	0.0925	401494	1	3.301587647
hsa00650	utanoate metabolis	11689	27/8521	0.09674	0.16446	0.0925	35	1	3.301587647
hsa04140	Autophagy - anima	11720	141/8521	0.09776	0.16468	0.09262	5580/5293	2	1.918386234
hsa04936	coholic liver diseas	11720	142/8521	0.09893	0.16468	0.09262	4790/1432	2	1.906890596
hsa04550	s regulating pluripo	11720	143/8521	0.1001	0.16468	0.09262	1432/5293	2	1.895302621
hsa05017	pinocerebellar atax	11720	143/8521	0.1001	0.16468	0.09262	5330/5293	2	1.895302621
hsa00630	and dicarboxylate	11689	30/8521	0.10691	0.17309	0.09735	847	1	3.150559677
hsa01523	ntifolate resistanc	11689	30/8521	0.10691	0.17309	0.09735	4790	1	3.150559677
hsa05206	licroRNAs in cance	11749	310/8521	0.10916	0.17534	0.09862	4790/5293/5335	3	1.367371066
hsa00410	a-Alanine metaboli	11689	31/8521	0.11028	0.17575	0.09885	35	1	3.102658131
hsa04261	c signaling in cardi	11720	154/8521	0.11328	0.17695	0.09952	5330/1432	2	1.790772038
hsa04921	ocin signaling path	11720	154/8521	0.11328	0.17695	0.09952	5330/6714	2	1.790772038
hsa00640	opanoate metabolis	11689	32/8521	0.11363	0.17695	0.09952	35	1	3.056583528
hsa05160	Hepatitis C	11720	158/8521	0.11819	0.18265	0.10273	4790/5293	2	1.752748591
hsa05165	n papillomavirus inf	11749	331/8521	0.12623	0.19362	0.1089	4790/5293/6696	3	1.269033146
hsa04630	STAT signaling pat	11720	166/8521	0.12815	0.19459	0.10944	6776/5293	2	1.682573297
hsa04960	-regulated sodium	11689	37/8521	0.13021	0.19459	0.10944	5293	1	2.847996907
hsa05143	ican trypanosomia	11689	37/8521	0.13021	0.19459	0.10944	5330	1	2.847996907
hsa05225	patocellular carcinc	11720	168/8521	0.13068	0.19459	0.10944	5293/5335	2	1.66448284
hsa05164	Influenza A	11720	171/8521	0.13448	0.1988	0.11181	4790/5293	2	1.63691458
hsa04216	Ferroptosis	11689	41/8521	0.14326	0.20876	0.11741	2180	1	2.698218478
hsa05219	Bladder cancer	11689	41/8521	0.14326	0.20876	0.11741	6714	1	2.698218478
hsa04151	-Akt signaling path	11749	354/8521	0.14599	0.20899	0.11754	4790/5293/6696	3	1.176322773
hsa00380	yptophan metabolis	11689	42/8521	0.1465	0.20899	0.11754	847	1	2.66448284
hsa00513	pes of N-glycan bi	11689	42/8521	0.1465	0.20899	0.11754	3073	1	2.66448284
hsa04975	igestion and absor	11689	43/8521	0.14972	0.2121	0.11929	10999	1	2.629939409
hsa00280	ine and isoleucine	11689	48/8521	0.16565	0.23305	0.13108	35	1	2.472487771
hsa00520	and nucleotide sug	11689	49/8521	0.1688	0.23384	0.13152	3073	1	2.440952198
hsa05030	Cocaine addiction	11689	49/8521	0.1688	0.23384	0.13152	4790	1	2.440952198
hsa00565	ther lipid metabolis	11689	50/8521	0.17194	0.23384	0.13152	79888	1	2.414135533
hsa05110	brio cholerae infect	11689	50/8521	0.17194	0.23384	0.13152	5335	1	2.414135533
hsa05144	Malaria	11689	50/8521	0.17194	0.23384	0.13152	4035	1	2.414135533
hsa04913	arian steroidogene	11689	51/8521	0.17507	0.23651	0.13302	240	1	2.384049807
hsa00600	hingolipid metaboli	11689	53/8521	0.18129	0.24172	0.13595	3073	1	2.327687364
hsa04961	factor-regulated c	11689	53/8521	0.18129	0.24172	0.13595	5330	1	2.327687364
hsa05134	Legionellosis	11689	56/8521	0.19054	0.2524	0.14196	4790	1	2.250961574
hsa04923	on of lipolysis in ad	11689	58/8521	0.19665	0.25715	0.14463	5293	1	2.198494154
hsa05213	Endometrial cance	11689	58/8521	0.19665	0.25715	0.14463	5293	1	2.198494154
hsa05416	Viral myocarditis	11689	60/8521	0.20271	0.26339	0.14814	857	1	2.150559677
hsa04024	MP signaling pathw	11720	225/8521	0.20655	0.26668	0.14999	4790/5293	2	1.244887059
hsa04810	ition of actin cytosk	11720	229/8521	0.21207	0.2721	0.15304	6714/5293	2	1.220329955
hsa04714	Thermogenesis	11720	232/8521	0.21623	0.27411	0.15417	1432/2180	2	1.201633861
hsa04927	l synthesis and se	11689	65/8521	0.21768	0.27411	0.15417	5330	1	2.03562391
hsa05321	nmatory bowel dis	11689	65/8521	0.21768	0.27411	0.15417	4790	1	2.03562391
hsa04720	ong-term potentiatio	11689	67/8521	0.22359	0.27983	0.15738	5330	1	1.989139007
hsa00830	Retinol metabolism	11689	68/8521	0.22653	0.28178	0.15848	216	1	1.970853654
hsa04924	Renin secretion	11689	69/8521	0.22945	0.28198	0.1586	5330	1	1.948600847
hsa05211	Renal cell carcinom	11689	69/8521	0.22945	0.28198	0.1586	5293	1	1.948600847
hsa05230	arbon metabolism	11689	70/8521	0.23237	0.28385	0.15965	5293	1	1.925999419
hsa04137	Mitophagy - anima	11689	72/8521	0.23817	0.2875	0.1617	6714	1	1.887525271
hsa05218	Melanoma	11689	72/8521	0.23817	0.2875	0.1617	5293	1	1.887525271
hsa01524	tinum drug resistar	11689	73/8521	0.24106	0.28927	0.1627	5293	1	1.867896464
hsa04020	cium signaling path	11720	253/8521	0.24554	0.29102	0.16368	5330/5335	2	1.070389328
hsa04623	lic DNA-sensing pa	11689	75/8521	0.2468	0.29102	0.16368	4790	1	1.827819025
hsa04918	roid hormone synth	11689	75/8521	0.2468	0.29102	0.16368	5330	1	1.827819025
hsa04971	astric acid secretic	11689	76/8521	0.24965	0.2927	0.16462	5330	1	1.807354922
hsa04742	Taste transduction	11689	86/8521	0.27762	0.31997	0.17996	5330	1	1.632268215
hsa04911	Insulin secretion	11689	86/8521	0.27762	0.31997	0.17996	5330	1	1.632268215
hsa05210	Colorectal cancer	11689	86/8521	0.27762	0.31997	0.17996	5293	1	1.632268215
hsa04512	M-receptor interact	11689	89/8521	0.28581	0.32573	0.1832	6696	1	1.580145484
hsa04727	GABAergic synaps	11689	89/8521	0.28581	0.32573	0.1832	6714	1	1.580145484
hsa04520	Adherens junction	11689	93/8521	0.29659	0.33428	0.18801	6714	1	1.516015147
hsa04970	Salivary secretion	11689	93/8521	0.29659	0.33428	0.18801	5330	1	1.516015147
hsa04713	ircadian entrainme	11689	97/8521	0.30722	0.34435	0.19368	5330	1	1.459431619
hsa00564	ophospholipid meta	11689	99/8521	0.31247	0.34699	0.19516	79888	1	1.427606173
hsa04010	PK signaling pathw	11720	301/8521	0.31297	0.34699	0.19516	4790/1432	2	0.82374936
hsa04916	Melanogenesis	11689	101/8521	0.31769	0.35031	0.19703	5330	1	1.40053793
hsa04972	Pancreatic secretio	11689	102/8521	0.32028	0.35127	0.19757	5330	1	1.384049807
hsa04928	one synthesis, sec	11689	106/8521	0.33056	0.36061	0.20282	5330	1	1.327687364
hsa04922	agon signaling path	11689	107/8521	0.3331	0.36145	0.20329	5330	1	1.316145742
hsa04724	utamatergic synap	11689	115/8521	0.35313	0.38116	0.21438	5330	1	1.214124805
hsa04152	PK signaling pathw	11689	121/8521	0.36777	0.39487	0.22209	5293	1	1.137503524
hsa04114	Oocyte meiosis	11689	131/8521	0.39146	0.4181	0.23516	1432	1	1.021479727
hsa05014	otrophic lateral scle	11720	364/8521	0.39971	0.42469	0.23886	847/1432	2	0.545968369
hsa04910	ulin signaling pathw	11689	137/8521	0.40526	0.42836	0.24092	5293	1	0.956056652
hsa05224	Breast cancer	11689	147/8521	0.42758	0.44962	0.25288	5293	1	0.855989697
hsa05226	Gastric cancer	11689	149/8521	0.43195	0.45189	0.25416	5293	1	0.839959587
hsa04934	Cushing syndrome	11689	155/8521	0.44486	0.46286	0.26033	5330	1	0.782408565
hsa04150	OR signaling pathw	11689	156/8521	0.44698	0.46286	0.26033	5293	1	0.773996325
hsa04217	Necroptosis	11689	159/8521	0.4533	0.46704	0.26268	6776	1	0.739848103
hsa04022	P-PKG signaling pa	11689	167/8521	0.46983	0.48163	0.27089	5330	1	0.669026766
hsa04530	Tight junction	11689	170/8521	0.4759	0.48504	0.2728	6714	1	0.650764559
hsa04310	nt signaling pathw	11689	171/8521	0.4779	0.48504	0.2728	5330	1	0.641546029
hsa05202	ional misregulation	11689	193/8521	0.52025	0.5254	0.2955	4790	1	0.464668267
hsa05012	Parkinson disease	11689	266/8521	0.63823	0.64137	0.36073	5335	1	0
hsa05016	Huntington disease	11689	306/8521	0.6904	0.6904	0.38831	5330	1	-0.200912694

Supplementary Table 14. Ascites lipid levels in patients with ovarian cancer ascites
"1" in DFS represents death or recurrence

Patient ID	Gender	group	Age	CHOL (mmol/L)	TG (mmol/L)	HDL-C (mmol/L)	LDL-C (mol/L)	Lpa (nmol/L)	ApoA1 (g/L)	ApoB (g/L)
Ascites1	Female	sensitive	55	2.38	0.45	0.44	1.69	7.5	0.39	0.36
Ascites2	Female	sensitive	64	2.85	0.47	0.42	2.04	6.5	0.42	0.46
Ascites3	Female	sensitive	62	2.78	0.47	0.34	2.03	2.5	0.34	0.45
Ascites4	Female	sensitive	53	1.27	0.3	0.25	0.91	1.3	0.28	0.26
Ascites5	Female	sensitive	57	2.12	0.46	0.36	NA	6.7	0.37	0.34
Ascites6	Female	sensitive	65	1.47	0.45	0.34	0.97	8.4	0.36	0.23
Ascites7	Female	sensitive	49	2.39	0.26	NA	NA	NA	0.57	0.3
Ascites8	Female	sensitive	69	2.91	0.34	0.66	1.65	30.2	0.5	0.33
Ascites9	Female	sensitive	52	3.92	1.97	0.58	2.68	13.8	0.74	0.7
Ascites10	Female	sensitive	66	2.32	0.33	0.56	1.67	26.3	0.53	0.34
Ascites11	Female	sensitive	63	4.28	0.49	0.89	3.19	14.2	0.73	0.59
Ascites12	Female	sensitive	52	5.23	0.82	0.79	3.74	6.2	0.36	0.76
Ascites13	Female	sensitive	56	3.93	1.21	0.52	2.66	20.8	0.61	0.8
Ascites14	Female	sensitive	63	4.92	1.2	0.47	3.69	11.3	0.52	0.89
Ascites15	Female	sensitive	72	5.15	1.42	0.48	3.79	7.1	0.79	0.75
Ascites16	Female	sensitive	51	5.11	0.81	1.28	3.61	17.8	0.91	0.72
Ascites17	Female	sensitive	67	5.2	1.67	0.71	3.64	7.6	0.81	0.98
Ascites18	Female	sensitive	50	4.01	0.58	1.04	2.84	37.6	0.88	0.65
Ascites19	Female	sensitive	41	2.89	0.58	0.5	2.08	5.7	0.57	0.49
Ascites20	Female	sensitive	54	6.09	1.51	0.54	4.66	4.6	0.92	0.89
Ascites21	Female	sensitive	41	3.12	0.8	0.56	2.23	51.6	0.68	0.58
Ascites22	Female	sensitive	69	3.57	0.53	0.74	2.56	10.9	0.76	0.63
Ascites23	Female	sensitive	71	2.55	0.48	0.45	1.86	3.6	0.44	0.48
Ascites24	Female	sensitive	60	4.19	0.87	0.76	2.98	4.3	0.74	0.76
Ascites25	Female	sensitive	47	4.61	0.71	0.83	3.23	48.7	0.71	0.82
Ascites26	Female	sensitive	54	2.84	0.47	0.62	1.96	12.4	0.5	0.52
Ascites27	Female	sensitive	51	2.17	0.49	0.63	1.4	5.2	0.63	0.37
Ascites28	Female	resistant	70	1.57	0.31	0.31	1.13	11.6	0.3	0.3
Ascites29	Female	resistant	53	3.33	0.66	0.42	2.45	8	0.42	0.67
Ascites30	Female	resistant	73	3.89	0.89	0.96	2.36	14.5	0.74	0.64

Supplementary Table 15. Quantitative Statistics of Western Blot results

SH3YL1

gene	value	group
SH3YL1	0.38464	SKOV3
SH3YL1	0.88864	SKOV3/DDP
SH3YL1	0.34195	SKOV3
SH3YL1	0.87749	SKOV3/DDP
SH3YL1	0.76111	SKOV3
SH3YL1	1.14942	SKOV3/DDP

CD4 T - cholesterol - CD44

group	actin	CD44	value
Ctrl	25906.8	24972.054	0.96392
CHO	27440	22485.196	0.81943
Ctrl	70297.3	72650.028	1.03347
CHO	79001.1	54474.2	0.68954
Ctrl	74577.4	70953.978	0.95141
CHO	86089.3	34903.359	0.40543
Ctrl	67629	71770.886	1.06124
CHO	79210.7	61721.3	0.7792
Ctrl	73418.4	67431.019	0.91845
CHO	73026.5	29751.057	0.4074
Ctrl	79332.3	69666.735	0.87816
CHO	78983.6	49790.179	0.63039

CD4 T - triglyceride - CD44

group	actin	CD44	value
Ctrl	34209.246	30503.8	0.89168
TG	34013.983	7946.98	0.23364
Ctrl	72080.886	69358.5	0.96223
TG	73340.279	49567.7	0.67586
Ctrl	70640.986	63662.8	0.90122
TG	75102.057	38320.1	0.51024
Ctrl	75306.543	63909.2	0.84865
TG	78165.208	40224.9	0.51461
Ctrl	78478.543	65641.2	0.83642
TG	77659.765	42250.6	0.54405
Ctrl	75070.329	74456.4	0.99182
TG	71762.794	24502.8	0.34144

CD4 T - cholesterol - CD69

group	actin	CD69	value
Ctrl	30306.7	22506.3	0.74262
CHO	29213.5	16231.9	0.55563
Ctrl	29129	23375.6	0.80249
CHO	29324.2	14229.1	0.48524
Ctrl	74577.4	62770.2	0.84168
CHO	86089.3	37824.3	0.43936
Ctrl	67629	70170.4	1.03758
CHO	79210.7	52783.3	0.66637
Ctrl	73418.4	68093.9	0.92748
CHO	73026.5	45462.1	0.62254

CD4 T - triglyceride - CD69

group	actin	CD69	value
Ctrl	26832.5	21962.1	0.81849
TG	28131.7	15096.2	0.53663
Ctrl	79100.1	71606.3	0.90526
TG	78978.2	41777.7	0.52898
Ctrl	80551.8	69560.1	0.86355
TG	84290.7	34825.5	0.41316
Ctrl	75606.3	68822	0.91027
TG	75012.4	41459.4	0.5527
Ctrl	78478.5	67832.9	0.86435
TG	77659.8	45450	0.58524

CD4 T - cholesterol - PD1

group	actin	PD-1	value
Ctrl	25983.9	27107.874	1.04326
CHO	25315	33258.116	1.31377
Ctrl	76972.6	54844.605	0.71252
CHO	63627.5	69478.22	1.09195
Ctrl	80216.1	50954.714	0.63522
CHO	76822.1	71975.957	0.93692
Ctrl	76737.1	43933.451	0.57252
CHO	69990.3	67058.785	0.95812
Ctrl	78515.1	45594.593	0.58071
CHO	79718.5	80698.028	1.01229

CD4 T - triglyceride - PD1

group	actin	PD-1	value
Ctrl	26700.497	15029.3	0.56289
TG	25239.69	31106.3	1.23244
Ctrl	76408.179	42039.8	0.5502
TG	73041.815	64747.1	0.88644
Ctrl	71710.836	41116.3	0.57336
TG	76030.978	63047	0.82923
Ctrl	81930.957	38462.6	0.46945
TG	82339.35	78722.6	0.95607

CD4 T - cholesterol - 2B4

group	actin	2B4	value
Ctrl	82414.5	41787.9	0.50704
CHO	77878	73512.9	0.94395
Ctrl	83723.2	31475.7	0.37595
CHO	79731	76726.2	0.96231
Ctrl	84340.6	46861.6	0.55562
CHO	85623.5	72626.6	0.84821
Ctrl	27721.2	15567.5	0.56157
CHO	29095.7	24046.4	0.82646
Ctrl	81859.2	28723.8	0.35089
CHO	80247	67338.4	0.83914

CD4 T - triglyceride - 2B4

group	actin	2B4	value
Ctrl	77207.7	44048	0.57051
TG	69492.9	72393.8	1.04174
Ctrl	70841.5	38013.2	0.53659
TG	69100.1	78121.2	1.13055
Ctrl	84802.5	57182.6	0.6743
TG	81188.5	73390.5	0.90395
Ctrl	74883.5	43366.1	0.57911
TG	74467.7	78573	1.05513
Ctrl	84354.6	38637.7	0.45804
TG	83916.2	67772	0.80761

CD4 T - cholesterol - CTLA4

group	actin	CTLA4	value
Ctrl	30706.3	12904.2	0.42025
CHO	30644.7	16280.6	0.53127
Ctrl	27721.2	13298.3	0.47972
CHO	29095.7	19757.1	0.67904
Ctrl	74803.2	52603.9	0.70323
CHO	70766.2	67910.2	0.95964
Ctrl	81062.9	50748.5	0.62604
CHO	77251.9	67135.1	0.86904
Ctrl	79529.7	40364	0.50753
CHO	79906.5	67426.5	0.84382
Ctrl	81859.2	22812.3	0.27868
CHO	80247	70967.5	0.88436
Ctrl	84579.2	33396.7	0.39486
CHO	82245.4	61169.9	0.74375

CD4 T - triglyceride - CTLA4

group	actin	CTLA4	value
Ctrl	30706.3	12904.2	0.42025
TG	30869.2	38328.5	1.24164
Ctrl	74205.6	22275.1	0.30018
TG	71267.7	59898.9	0.84048
Ctrl	84354.6	48346.1	0.57313
TG	83916.2	65776.3	0.78383

CD8 T - cholesterol - CD44

group	actin	CD44	value
Ctrl	67908.6	67489.07	0.99382
CHO	67881.4	47409.078	0.69841
Ctrl	69959.3	63046.484	0.90119
CHO	76809.4	32510.459	0.42326
Ctrl	75633	69020.099	0.91257
CHO	79610.4	39346.078	0.49423
Ctrl	75203.5	65147.726	0.86629
CHO	73983.1	42208.806	0.57052

CD8 T - triglyceride - CD44

group	actin	CD44	value
Ctrl	75777.342	62629.1	0.82649
TG	80230.078	45050.5	0.56152
Ctrl	72934.513	60830.1	0.83404
TG	72537.329	36887.3	0.50853
Ctrl	75779.43	74305.2	0.98055
TG	76158.158	27475.1	0.36076
Ctrl	82010.865	82557.4	1.00666
TG	81028.108	30347.9	0.37454

CD8 T - cholesterol - CD69

group	actin	CD69	value
Ctrl	67908.6	59259.5	0.87264
CHO	67881.4	26202.3	0.386
Ctrl	69959.3	59231.3	0.84665
CHO	76809.4	37092.8	0.48292
Ctrl	75605.6	60542.6	0.80077
CHO	82757.5	40049.2	0.48393
Ctrl	75633	62156.1	0.82181
CHO	79610.4	40540.4	0.50923
Ctrl	75203.5	62818.7	0.83532
CHO	73983.1	16493	0.22293

CD8 T - triglyceride - CD69

group	actin	CD69	value
Ctrl	29561	24040.9	0.81327
TG	30816.3	17763.1	0.57642
Ctrl	76323.3	68229.8	0.89396
TG	76199.7	49466.3	0.64917
Ctrl	79775.3	69378	0.86967
TG	82076.5	48905.5	0.59585
Ctrl	82010.9	71763	0.87504
TG	81028.1	34839.2	0.42996

CD8 T - cholesterol - PD1

group	actin	PD-1	value
Ctrl	29443.5	8598.296	0.29203
CHO	30580.9	22003.539	0.71952
Ctrl	77533.6	24403.593	0.31475
CHO	71062.5	72427.563	1.01921
Ctrl	76812.2	39034.484	0.50818
CHO	72944.5	65956.099	0.9042
Ctrl	83383.9	18842.785	0.22598
CHO	83645.8	60169.392	0.71934
Ctrl	72912	42325.865	0.58051
CHO	83964.1	68629.735	0.81737

CD8 T - triglyceride - PD1

group	actin	PD-1	value
Ctrl	71380.128	32998.8	0.4623
TG	71041.279	58355.5	0.82143
Ctrl	76706.057	24688.1	0.32185
TG	75238.723	59079.2	0.78522
Ctrl	76726.907	20384.7	0.26568
TG	73502.037	65165	0.88657

CD8 T - cholesterol - 2B4

group	actin	2B4	value
Ctrl	35595.9	11770.8	0.33068
CHO	35699.1	18667.9	0.52292
Ctrl	85215.2	20140	0.23634
CHO	87188.4	66069.7	0.75778
Ctrl	85120.2	49685.9	0.58371
CHO	79475.9	71103.8	0.89466
Ctrl	71658.2	41460.7	0.57859
CHO	73295.3	62507.4	0.85281

CD8 T - triglyceride - 2B4

group	actin	2B4	value
Ctrl	27777.7	15769.8	0.56771
TG	26417.4	22341.6	0.84571
Ctrl	72509.2	38532.6	0.53142
TG	69633.4	63213.2	0.9078
Ctrl	78743.4	48157.8	0.61158
TG	76086.3	68758.3	0.90369
Ctrl	77343.6	47029	0.60805
TG	79487.6	73925.9	0.93003
Ctrl	73266.4	42743.5	0.5834
TG	86265.6	73548.3	0.85258
Ctrl	78863	39785	0.50448
TG	73341	80545.8	1.09824

CD8 T - cholesterol - CTLA4

group	actin	CTLA4	value
Ctrl	35595.9	26013.9	0.73081
CHO	35699.1	47339.2	1.32606
Ctrl	84136.2	42950	0.51048
CHO	78084.6	69316.5	0.88771
Ctrl	71658.2	26492.1	0.3697
CHO	73295.3	83353.4	1.13723

CD8 T - triglyceride - CTLA4

group	actin	CTLA4	value
Ctrl	73266.4	46000.3	0.62785
TG	86265.6	72300.8	0.83812
Ctrl	78863	42218.6	0.53534
TG	73341	73075.4	0.99638
Ctrl	24067.4	16808.5	0.69839
TG	22613.2	21021	0.92959

CD4 T - siRNA - CD44

group	actin	CD44	value
CD4 T-siRNA1	32839.9	22964.58	0.69929
CD4 T-siRNA2	34221.1	14596.53	0.42654
CD4 T-siRNA3	41877.4	22323.97	0.53308
CD4 T-siCtrl	31523.7	25190.27	0.79909
CD4 T-siRNA1	51400.8	23352.55	0.45432
CD4 T-siRNA2	44858.3	28764.63	0.64123
CD4 T-siRNA3	37611.4	22964.59	0.61057
CD4 T-siCtrl	36399.7	31646.39	0.86941
CD4 T-siRNA1	34949.2	14785.36	0.42305
CD4 T-siRNA2	37308.8	21610.93	0.57925
CD4 T-siRNA3	38239.2	17815.82	0.4659
CD4 T-siCtrl	35388.4	37567.87	1.06159

CD8 T - siRNA - CD44

group	actin	CD44	value
CD8 T-siRNA1	29068.58	18248.6	0.62778
CD8 T-siRNA2	37810.97	21616.7	0.57171
CD8 T-siRNA3	38290.66	26031.2	0.67983
CD8 T-siCtrl	36397.3	33174.9	0.91147
CD8 T-siRNA1	35931.46	22346.5	0.62192
CD8 T-siRNA2	41513.51	23182.5	0.55843
CD8 T-siRNA3	38161.34	17296.4	0.45324
CD8 T-siCtrl	40206.78	32729.1	0.81402
CD8 T-siRNA1	37261.25	19897.5	0.534
CD8 T-siRNA2	43581.39	28359.2	0.65072
CD8 T-siRNA3	39252.9	24187.1	0.61619
CD8 T-siCtrl	42078.22	35148.7	0.83532

SH3YL1-baseline

group	actin	SH3YL1	value
SKOV3	34662.4	31430.652	0.90677
Caov-3	38990.8	12270.125	0.31469
OVCAR-3	23021	19792.56	0.85976
SKOV3	29092.2	22951.409	0.78892
Caov-3	30958.6	5269.054	0.1702
OVCAR-3	32010.7	23913.439	0.74705
SKOV3	28097.3	12355.49	0.43974
Caov-3	32085.5	4508.03	0.1405
OVCAR-3	33908.1	14795.63	0.43635
SKOV3	29660.4	23889.125	0.80542
Caov-3	33945.5	9581.095	0.28225
OVCAR-3	29266.5	22121.882	0.75588
SKOV3	27857.5	18957.54	0.68052
Caov-3	33723.2	6019.05	0.17848
OVCAR-3	26875	19215.2	0.71499
SKOV3	27062.9	18022.27	0.66594
Caov-3	31142.1	9285.35	0.29816
OVCAR-3	25060.2	16071.42	0.64131
SKOV3	27750.4	15939.52	0.57439
Caov-3	29357.8	4909.71	0.16724
OVCAR-3	29351.5	14698.83	0.50079

SKOV3 - SH3YL1 - Overexpression

group	actin	SH3YL1	value
SKOV3-Ctrl	22368.4	28280.9	1.26432
SKOV3-OE	26408.42	44898	1.70014
SKOV3-Ctrl	22583.723	17999.5	0.79701
SKOV3-OE	26241.38	32312	1.23134
SKOV3-Ctrl	26241.12	27263.5	1.03896
SKOV3-OE	22124.97	42632.6	1.9269
SKOV3-Ctrl	21384.3	23361.5	1.09246
SKOV3-OE	21537.54	35129.9	1.6311
SKOV3-Ctrl	39428.97	25516	0.64714
SKOV3-OE	37994.02	41059.1	1.08067

Caov3 - SH3YL1 - Overexpression

group	actin	SH3YL1	value
Caov3-Ctrl	64226.9	39594.5	0.61648
Caov3-OE	62702.5	55979	0.89277
Caov3-Ctrl	59744.8	45861.1	0.76762
Caov3-OE	58468.1	58657.1	1.00323
Caov3-Ctrl	63606.1	42567.5	0.66924
Caov3-OE	65374.2	57421.3	0.87835
Caov3-Ctrl	68592.6	43778.9	0.63825
Caov3-OE	68101	57141	0.83906
Caov3-Ctrl	50341.4	28977.2	0.57561
Caov3-OE	47688.3	38636.1	0.81018
Caov3-Ctrl	49934.5	16504.1	0.33052
Caov3-OE	50004.6	39761.3	0.79515

SKOV3/DDP - SH3YL1 - Knock Down

group	actin	SH3YL1	value
SKOV3/DDP-shCtrl	41516.6	32449.7	0.78161
SKOV3/DDP-sh1	41401.7	24707.1	0.59676
SKOV3/DDP-sh2	40646.3	28971.6	0.71277
SKOV3/DDP-sh3	38528.4	35276.9	0.91561
SKOV3/DDP-shCtrl	37855.4	37173.2	0.98198
SKOV3/DDP-sh1	39817.1	30338.5	0.76195
SKOV3/DDP-sh2	36040.7	24718.6	0.68585
SKOV3/DDP-sh3	31540.4	15125.2	0.47955
SKOV3/DDP-shCtrl	32450.2	34066.4	1.04981
SKOV3/DDP-sh1	42559.3	17211.3	0.40441
SKOV3/DDP-sh2	45110.5	14326	0.31757
SKOV3/DDP-sh3	42262.5	16610.3	0.39303
SKOV3/DDP-shCtrl	38437.2	28626.8	0.74477
SKOV3/DDP-sh1	41988.4	23268.4	0.55416
SKOV3/DDP-sh2	44408.1	28676.2	0.64574
SKOV3/DDP-sh3	40400.5	37542.4	0.92926
SKOV3/DDP-shCtrl	34333.8	23484.4	0.684
SKOV3/DDP-sh1	32284.2	17327	0.5367
SKOV3/DDP-sh2	40964.2	24417.7	0.59607
SKOV3/DDP-sh3	40975	49424.4	1.20621

OVCAR3 - SH3YL1 - Knock Down

group	actin	SH3YL1	value
OVCAR3-shCtrl	41516.6	32449.7	0.78161
OVCAR3-sh1	41401.7	24707.1	0.59676
OVCAR3-sh2	40646.3	28971.6	0.71277
OVCAR3-sh3	38528.4	35276.9	0.91561
OVCAR3-shCtrl	37855.4	37173.2	0.98198
OVCAR3-sh1	39817.1	30338.5	0.76195
OVCAR3-sh2	36040.7	24718.6	0.68585
OVCAR3-sh3	31540.4	15125.2	0.47955
OVCAR3-shCtrl	32450.2	34066.4	1.04981
OVCAR3-sh1	42559.3	17211.3	0.40441
OVCAR3-sh2	45110.5	14326	0.31757
OVCAR3-sh3	42262.5	16610.3	0.39303
OVCAR3-shCtrl	38437.2	28626.8	0.74477
OVCAR3-sh1	41988.4	23268.4	0.55416
OVCAR3-sh2	44408.1	28676.2	0.64574
OVCAR3-sh3	40400.5	37542.4	0.92926
OVCAR3-shCtrl	34333.8	23484.4	0.684
OVCAR3-sh1	32284.2	17327	0.5367
OVCAR3-sh2	40964.2	24417.7	0.59607
OVCAR3-sh3	40975	49424.4	1.20621

Supplementary Table 16. Quantitative Statistics of Flow Cytometry results

Lipid Treated CD4$^+$ T cells Panel1

Marker	Freq	Group
CD4+	83.6	CHO
CD4+ CD44+	80	CHO
CD4+ GZMB+	28.3	CHO
CD4+ IFN+	4.24	CHO
CD4+ TCF1+	6.76	CHO
CD4+	83.5	CHO
CD4+ CD44+	81	CHO
CD4+ GZMB+	30.5	CHO
CD4+ IFN+	4.61	CHO
CD4+ TCF1+	8.39	CHO
CD4+	83.1	CHO
CD4+ CD44+	68.9	CHO
CD4+ GZMB+	34.6	CHO
CD4+ IFN+	6.42	CHO
CD4+ TCF1+	6.42	CHO
CD4+	94.4	Ctrl
CD4+ CD44+	86	Ctrl
CD4+ GZMB+	10.6	Ctrl
CD4+ IFN+	10.2	Ctrl
CD4+ TCF1+	1.66	Ctrl
CD4+	94.3	Ctrl
CD4+ CD44+	86	Ctrl
CD4+ GZMB+	11	Ctrl
CD4+ IFN+	9.78	Ctrl
CD4+ TCF1+	1.91	Ctrl
CD4+	93.4	Ctrl
CD4+ CD44+	85.6	Ctrl
CD4+ GZMB+	13.4	Ctrl
CD4+ IFN+	9.71	Ctrl
CD4+ TCF1+	2.74	Ctrl
CD4+	84.2	TG
CD4+ CD44+	69.3	TG
CD4+ GZMB+	33.7	TG
CD4+ IFN+	6.15	TG
CD4+ TCF1+	6.1	TG
CD4+	83.4	TG
CD4+ CD44+	69.2	TG
CD4+ GZMB+	34.8	TG
CD4+ IFN+	5.86	TG
CD4+ TCF1+	6.19	TG
CD4+	93.2	TG
CD4+ CD44+	78	TG
CD4+ GZMB+	13.6	TG
CD4+ IFN+	8.13	TG
CD4+ TCF1+	2.74	TG

Lipid Treated CD4$^+$ T cells Panel2

Marker	Freq	Group
CD4+	71	CHO
CD4+ PD1+	16.3	CHO
CD4+ TOX+	95.3	CHO
CD4+	71.2	CHO
CD4+ PD1+	16.2	CHO
CD4+ TOX+	95.1	CHO
CD4+	85.2	CHO
CD4+ PD1+	13.3	CHO
CD4+ TOX+	95.9	CHO
CD4+	83.4	Ctrl
CD4+ PD1+	13	Ctrl
CD4+ TOX+	93.1	Ctrl
CD4+	83.2	Ctrl
CD4+ PD1+	13.6	Ctrl
CD4+ TOX+	92.4	Ctrl
CD4+	83.6	Ctrl
CD4+ PD1+	13.4	Ctrl
CD4+ TOX+	92.4	Ctrl
CD4+	86.1	TG
CD4+ PD1+	14.5	TG
CD4+ TOX+	95.6	TG
CD4+	84.8	TG
CD4+ PD1+	14.5	TG
CD4+ TOX+	94.3	TG
CD4+	85.7	TG
CD4+ PD1+	14.5	TG
CD4+ TOX+	95.3	TG

Lipid Treated CD8$^+$ T cells Panel1

Marker	Freq	Group
CD8+	40.1	CHO
CD8+ CD44+	93.4	CHO
CD8+ GZMB+	13.3	CHO
CD8+ IFN+	22.6	CHO
CD8+ TCF1+	6.49	CHO
CD8+	40.3	CHO
CD8+ CD44+	93.6	CHO
CD8+ GZMB+	13.4	CHO
CD8+ IFN+	22.1	CHO
CD8+ TCF1+	6.03	CHO
CD8+	66	CHO
CD8+ CD44+	90.7	CHO
CD8+ GZMB+	59.1	CHO
CD8+ IFN+	27.5	CHO
CD8+ TCF1+	18	CHO
CD8+	63.3	Ctrl
CD8+ CD44+	91.6	Ctrl
CD8+ GZMB+	59.4	Ctrl
CD8+ IFN+	30	Ctrl
CD8+ TCF1+	12.9	Ctrl
CD8+	63.7	Ctrl
CD8+ CD44+	92	Ctrl
CD8+ GZMB+	58.9	Ctrl
CD8+ IFN+	29.8	Ctrl
CD8+ TCF1+	12.5	Ctrl
CD8+	63.9	Ctrl
CD8+ CD44+	92.1	Ctrl
CD8+ GZMB+	58.9	Ctrl
CD8+ IFN+	30.4	Ctrl
CD8+ TCF1+	12.9	Ctrl
CD8+	39.5	TG
CD8+ CD44+	94.2	TG
CD8+ GZMB+	8.51	TG
CD8+ IFN+	15.1	TG
CD8+ TCF1+	2.71	TG
CD8+	40.1	TG
CD8+ CD44+	94.4	TG
CD8+ GZMB+	9.17	TG
CD8+ IFN+	15.2	TG
CD8+ TCF1+	3.17	TG
CD8+	51.3	TG
CD8+ CD44+	88.4	TG
CD8+ GZMB+	59.2	TG
CD8+ IFN+	30.2	TG
CD8+ TCF1+	9.92	TG

Lipid Treated CD8$^+$ T cells Panel2

Marker	Freq	Group
CD8+	55.4	CHO
CD8+ PD1+	3.71	CHO
CD8+ TOX+	97.6	CHO
CD8+	54.7	CHO
CD8+ PD1+	3.41	CHO
CD8+ TOX+	97.5	CHO
CD8+	55	CHO
CD8+ PD1+	3.92	CHO
CD8+ TOX+	97.4	CHO
CD8+	73.6	Ctrl
CD8+ PD1+	0.77	Ctrl
CD8+ TOX+	95.2	Ctrl
CD8+	72.9	Ctrl
CD8+ PD1+	0.62	Ctrl
CD8+ TOX+	95.5	Ctrl
CD8+	71.6	Ctrl
CD8+ PD1+	0.57	Ctrl
CD8+ TOX+	95.2	Ctrl
CD8+	48.9	TG
CD8+ PD1+	3.88	TG
CD8+ TOX+	96.8	TG
CD8+	49.5	TG
CD8+ PD1+	3.89	TG
CD8+ TOX+	96.9	TG
CD8+	49.9	TG
CD8+ PD1+	3.7	TG
CD8+ TOX+	96.6	TG

siRNA Treated CD4$^+$ T cells Panel1

Marker	Freq	Group
CD4+	79.8	siCtrl
CD4+ CD44+	69.5	siCtrl
CD4+ GZMB+	16.3	siCtrl
CD4+ IFN+	33	siCtrl
CD4+ TCF1+	0.19	siCtrl
CD4+	80	siCtrl
CD4+ CD44+	69.8	siCtrl
CD4+ GZMB+	16.4	siCtrl
CD4+ IFN+	32.5	siCtrl
CD4+ TCF1+	0.27	siCtrl
CD4+	80	siCtrl
CD4+ CD44+	69.2	siCtrl
CD4+ GZMB+	16.5	siCtrl
CD4+ IFN+	32.7	siCtrl
CD4+ TCF1+	0.23	siCtrl
CD4+	66.2	siCtrl
CD4+ CD44+	60.9	siRNA
CD4+ GZMB+	20.7	siRNA
CD4+ IFN+	25.1	siRNA
CD4+ TCF1+	0.44	siRNA
CD4+	67.1	siRNA
CD4+ CD44+	61	siRNA
CD4+ GZMB+	20.9	siRNA
CD4+ IFN+	25.5	siRNA
CD4+ TCF1+	0.45	siRNA
CD4+	66.6	siRNA
CD4+ CD44+	61.6	siRNA
CD4+ GZMB+	21.2	siRNA
CD4+ IFN+	25.8	siRNA
CD4+ TCF1+	0.43	siRNA

siRNA Treated CD4$^+$ T cells Panel2

Marker	Freq	Group
CD4+	66.7	siCtrl
CD4+ PD1+	14.4	siCtrl
CD4+ TOX+	67.5	siCtrl
CD4+	88.6	siCtrl
CD4+ PD1+	14.8	siCtrl
CD4+ TOX+	85.6	siCtrl
CD4+	86.8	siCtrl
CD4+ PD1+	14.7	siCtrl
CD4+ TOX+	85.7	siCtrl
CD4+	69.2	siRNA
CD4+ PD1+	20.6	siRNA
CD4+ TOX+	72	siRNA
CD4+	87.3	siRNA
CD4+ PD1+	14	siRNA
CD4+ TOX+	81	siRNA
CD4+	86.5	siRNA
CD4+ PD1+	14.1	siRNA
CD4+ TOX+	78.8	siRNA

siRNA Treated CD8$^+$ T cells Panel1

Marker	Freq	Group
CD8+	84.8	siCtrl
CD8+ CD44+	85.1	siCtrl
CD8+ GZMB+	75.7	siCtrl
CD8+ IFN+	58	siCtrl
CD8+ TCF1+	0.26	siCtrl
CD8+	84.6	siCtrl
CD8+ CD44+	85.2	siCtrl
CD8+ GZMB+	76.4	siCtrl
CD8+ IFN+	58.3	siCtrl
CD8+ TCF1+	0.26	siCtrl
CD8+	84.5	siCtrl
CD8+ CD44+	84.1	siCtrl
CD8+ GZMB+	75	siCtrl
CD8+ IFN+	57.1	siCtrl
CD8+ TCF1+	0.096	siCtrl
CD8+	68.3	siRNA
CD8+ CD44+	70.4	siRNA
CD8+ GZMB+	69.9	siRNA
CD8+ IFN+	24.8	siRNA
CD8+ TCF1+	0.75	siRNA
CD8+	69.7	siRNA
CD8+ CD44+	70.8	siRNA
CD8+ GZMB+	70.7	siRNA
CD8+ IFN+	24.3	siRNA
CD8+ TCF1+	0.5	siRNA
CD8+	68.7	siRNA
CD8+ CD44+	70.4	siRNA
CD8+ GZMB+	71.4	siRNA
CD8+ IFN+	24.4	siRNA
CD8+ TCF1+	0.98	siRNA

siRNA Treated CD8$^+$ T cells Panel2

Marker	Freq	Group
CD8+	87.2	siCtrl
CD8+ PD1+	7.8	siCtrl
CD8+ TOX+	95.9	siCtrl
CD8+	87.3	siCtrl
CD8+ PD1+	7.62	siCtrl
CD8+ TOX+	96	siCtrl
CD8+	86.9	siCtrl
CD8+ PD1+	7.66	siCtrl
CD8+ TOX+	95.9	siCtrl
CD8+	78.6	siRNA
CD8+ PD1+	14.1	siRNA
CD8+ TOX+	97.5	siRNA
CD8+	79.4	siRNA
CD8+ PD1+	13.9	siRNA
CD8+ TOX+	97.6	siRNA
CD8+	78.8	siRNA
CD8+ PD1+	14.5	siRNA
CD8+ TOX+	97.6	siRNA

Supplementary Table 17. Kyoto Encyclopedia of Genes and Genomes enrichment analysis of genes positively associated with SH3YL1 expression

ID	Description	GeneRatio	BgRatio	pvalue	p.adjust	qvalue	geneID	Count	Fold_enrichment	type
hsa04530	Tight junction	12/130	170/8521	1.01E-05	0.002616	0.002392	l2/9475/8531/54566/87/5584/5872/94	12	4.63	Positive correlation
hsa05130	enic Escherichia coli	11/130	198/8521	0.00021	0.019155	0.017517	23365/347733/147179/84617/10381/4	11	3.64	Positive correlation
hsa04210	Apoptosis	9/130	136/8521	0.000222	0.019155	0.017517	7846/7277/143/330/10018/27429/3845	9	4.34	Positive correlation
hsa04540	Gap junction	7/130	88/8521	0.000372	0.024117	0.022054	46/7277/347733/84617/10381/2778/38	7	5.21	Positive correlation
hsa04910	sulin signaling pathw	8/130	137/8521	0.001139	0.048111	0.043995	98/10580/8660/5584/3636/3845/868/2	8	3.83	Positive correlation
hsa04120	uitin mediated protec	8/130	142/8521	0.001435	0.048111	0.043995	264/7317/9040/8065/9820/330/868/65	8	3.69	Positive correlation
hsa05132	Salmonella infection	11/130	249/8521	0.001439	0.048111	0.043995	'5/347733/330/84617/10381/3320/107	11	2.9	Positive correlation
hsa04144	Endocytosis	11/130	250/8521	0.001486	0.048111	0.043995	26767/84612/8723/5584/147179/8022	11	2.88	Positive correlation
hsa05012	Parkinson disease	11/130	266/8521	0.002426	0.069817	0.063844	317/7277/509/347733/498/84617/2742	11	2.71	Positive correlation
hsa01230	synthesis of amino a	5/130	75/8521	0.005629	0.145801	0.133327	95/5214/65263/2875/6470	5	4.37	Positive correlation
hsa01200	Carbon metabolism	6/130	115/8521	0.008155	0.19202	0.175593	25796/26007/2821/5214/2875/6470	6	3.42	Positive correlation
hsa00030	tose phosphate path	3/130	30/8521	0.010429	0.225102	0.205844	25796/2821/5214	3	6.55	Positive correlation
hsa04215	ptosis - multiple spe	3/130	32/8521	0.012463	0.248308	0.227065	330/10018/27429	3	6.14	Positive correlation
hsa04520	Adherens junction	5/130	93/8521	0.013653	0.252589	0.230979	1457/10580/9475/87/56288	5	3.52	Positive correlation
hsa04310	Vnt signaling pathwa	7/130	171/8521	0.015563	0.268729	0.245739	157/56998/1457/8323/9475/81839/650	7	2.68	Positive correlation
hsa00500	h and sucrose metal	3/130	36/8521	0.017168	0.277915	0.254139	2632/2821/2997	3	5.46	Positive correlation
hsa05216	Thyroid cancer	3/130	37/8521	0.018479	0.281536	0.25745	7157/10342/3845	3	5.31	Positive correlation
hsa05020	Prion disease	9/130	272/8521	0.02309	0.323185	0.295536	457/7277/1386/509/347733/498/84617	9	2.17	Positive correlation
hsa04810	ation of actin cytosk	8/130	229/8521	0.023709	0.323185	0.295536	8/3696/9475/87/23365/3845/10787/10	8	2.29	Positive correlation
hsa01524	atinum drug resistan	4/130	73/8521	0.025075	0.324722	0.296942	7157/330/2950/2067	4	3.59	Positive correlation
hsa04814	Motor proteins	7/130	193/8521	0.028055	0.334093	0.305511	46/7277/4635/347733/84617/10381/46	7	2.38	Positive correlation
hsa05010	Alzheimer disease	11/130	384/8521	0.032405	0.334093	0.305511	323/7277/8660/509/347733/498/84617	11	1.88	Positive correlation
hsa04390	ppo signaling pathw	6/130	157/8521	0.032774	0.334093	0.305511	8323/84612/330/5584/84962/56288	6	2.5	Positive correlation
hsa01210	carboxylic acid meta	2/130	19/8521	0.033341	0.334093	0.305511	95/2875	2	6.9	Positive correlation
hsa04935	one synthesis, secre	5/130	120/8521	0.03629	0.334093	0.305511	1398/1386/8660/2778/3845	5	2.73	Positive correlation
hsa05203	Viral carcinogenesis	7/130	204/8521	0.036326	0.334093	0.305511	7157/1386/87/2968/9093/3845/7874	7	2.25	Positive correlation
hsa05205	roteoglycans in canc	7/130	205/8521	0.03715	0.334093	0.305511	'157/6382/8323/9475/2066/23365/384	7	2.24	Positive correlation
hsa04152	MPK signaling pathw	5/130	121/8521	0.037408	0.334093	0.305511	23417/8660/5214/5862/2997	5	2.71	Positive correlation
hsa04919	hormone signaling p	5/130	121/8521	0.037408	0.334093	0.305511	9862/7157/10499/5214/3845	5	2.71	Positive correlation
hsa04012	rbB signaling pathwa	4/130	85/8521	0.040636	0.343933	0.314509	1398/2066/3845/868	4	3.08	Positive correlation
hsa00220	Arginine biosynthesis	2/130	22/8521	0.043729	0.343933	0.314509	95/2875	2	5.96	Positive correlation
hsa00340	Histidine metabolism	2/130	22/8521	0.043729	0.343933	0.314509	138199/501	2	5.96	Positive correlation
hsa04151	K-Akt signaling path	10/130	354/8521	0.043822	0.343933	0.314509	/3696/1386/117145/2066/10018/3320/	10	1.85	Positive correlation
hsa04211	gevity regulating path	4/130	89/8521	0.046809	0.356574	0.326068	7157/1386/8660/3845	4	2.95	Positive correlation
hsa05014	otrophic lateral scle	10/130	364/8521	0.051208	0.373898	0.34191	5/7277/509/2066/347733/498/9631/84	10	1.8	Positive correlation
hsa05163	n cytomegalovirus in	7/130	225/8521	0.056279	0.373898	0.34191	'157/1398/9475/1386/23365/2778/384	7	2.04	Positive correlation
hsa04915	rogen signaling path	5/130	137/8521	0.058191	0.373898	0.34191	10499/1386/2778/3320/3845	5	2.39	Positive correlation
hsa05135	Yersinia infection	5/130	137/8521	0.058191	0.373898	0.34191	1398/9475/23365/147179/10552	5	2.39	Positive correlation
hsa04923	ion of lipolysis in adi	3/130	58/8521	0.05838	0.373898	0.34191	8660/2778/4881	3	3.39	Positive correlation
hsa05022	urodegeneration - m	12/130	476/8521	0.059085	0.373898	0.34191	/8323/7277/509/347733/498/84617/27	12	1.65	Positive correlation
hsa04360	Axon guidance	6/130	182/8521	0.059588	0.373898	0.34191	2049/84612/9475/23365/3845/56288	6	2.16	Positive correlation
hsa05215	Prostate cancer	4/130	97/8521	0.060632	0.373898	0.34191	7157/2950/3320/3845	4	2.7	Positive correlation
hsa05165	an papillomavirus infe	9/130	331/8521	0.065976	0.397388	0.363391	910/8323/3696/84612/5584/2778/3845	9	1.78	Positive correlation
hsa00410	ta-Alanine metabolis	2/130	31/8521	0.080614	0.455441	0.416478	23417/501	2	4.23	Positive correlation
hsa04922	cagon signaling path	4/130	107/8521	0.08062	0.455441	0.416478	1386/5214/2778/2997	4	2.45	Positive correlation
hsa00010	olysis / Gluconeoge	3/130	67/8521	0.08227	0.455441	0.416478	2821/5214/501	3	2.93	Positive correlation
hsa04145	Phagosome	5/130	152/8521	0.082648	0.455441	0.416478	7846/7277/347733/84617/10381	5	2.16	Positive correlation
hsa04130	eractions in vesicula	2/130	33/8521	0.089777	0.471569	0.431226	55850/8675	2	3.97	Positive correlation
hsa04510	Focal adhesion	6/130	203/8521	0.089971	0.471569	0.431226	1398/3910/3696/9475/87/330	6	1.94	Positive correlation
hsa05230	carbon metabolism i	3/130	70/8521	0.091037	0.471569	0.431226	7157/5214/3845	3	2.81	Positive correlation
hsa00051	e and mannose met	2/130	34/8521	0.094468	0.474452	0.433862	26007/5214	2	3.86	Positive correlation
hsa05016	Huntington disease	8/130	306/8521	0.095637	0.474452	0.433862	7/7846/7277/509/347733/498/84617/1	8	1.71	Positive correlation
hsa04137	Mitophagy - animal	3/130	72/8521	0.097089	0.474452	0.433862	7157/1457/3845	3	2.73	Positive correlation
hsa04015	ap1 signaling pathwa	6/130	210/8521	0.101685	0.48771	0.445986	1398/84612/5584/2778/3845/56288	6	1.87	Positive correlation
hsa03008	ne biogenesis in euk	5/130	164/8521	0.105582	0.497196	0.45466	1457/10557/1736/55272/55341	5	2	Positive correlation
hsa00250	artate and glutamate	2/130	37/8521	0.108947	0.498308	0.455677	56954/2875	2	3.54	Positive correlation
hsa05220	ronic myeloid leuke	3/130	76/8521	0.109666	0.498308	0.455677	7157/1398/3845	3	2.59	Positive correlation
hsa05412	ic right ventricular ca	3/130	77/8521	0.112905	0.50418	0.461047	3696/3728/4000	3	2.55	Positive correlation
hsa03018	RNA degradation	3/130	79/8521	0.119491	0.522467	0.477769	51013/5214/5073	3	2.49	Positive correlation
hsa04611	Platelet activation	4/130	124/8521	0.121035	0.522467	0.477769	9475/23365/5584/2778	4	2.11	Positive correlation
hsa00260	erine and threonine r	2/130	40/8521	0.123965	0.526345	0.481315	6470/501	2	3.28	Positive correlation
hsa05219	Bladder cancer	2/130	41/8521	0.129078	0.539214	0.493083	7157/3845	2	3.2	Positive correlation
hsa03083	comb repressive cor	3/130	84/8521	0.136555	0.561392	0.513364	1912/7874/6500	3	2.34	Positive correlation
hsa04068	oxO signaling pathwa	4/130	131/8521	0.139797	0.563536	0.515324	8660/10018/3845/7874	4	2	Positive correlation
hsa04714	Thermogenesis	6/130	232/8521	0.143376	0.563536	0.515324	1386/509/498/2778/3845/4881	6	1.7	Positive correlation
hsa05210	Colorectal cancer	3/130	86/8521	0.143604	0.563536	0.515324	7157/10018/3845	3	2.29	Positive correlation
hsa04270	r smooth muscle cor	4/130	134/8521	0.148177	0.572803	0.523798	9475/23365/2778/4881	4	1.96	Positive correlation
hsa04512	CM-receptor interact	3/130	89/8521	0.154398	0.588075	0.537764	6382/3910/3696	3	2.21	Positive correlation
hsa05418	ar stress and athero	4/130	139/8521	0.162563	0.604695	0.552962	7157/6382/2950/3320	4	1.89	Positive correlation
hsa05222	Small cell lung cance	3/130	92/8521	0.16544	0.604695	0.552962	7157/3910/330	3	2.14	Positive correlation
hsa00280	cine and isoleucine d	2/130	48/8521	0.166089	0.604695	0.552962	549/501	2	2.73	Positive correlation
hsa05030	Cocaine addiction	2/130	49/8521	0.171521	0.604695	0.552962	1386/2778	2	2.68	Positive correlation
hsa05131	Shigellosis	6/130	247/8521	0.175684	0.604695	0.552962	7157/1398/9475/87/6500/10552	6	1.59	Positive correlation
hsa00330	ne and proline metal	2/130	50/8521	0.176983	0.604695	0.552962	65263/501	2	2.62	Positive correlation
hsa05110	brio cholerae infecti	2/130	50/8521	0.176983	0.604695	0.552962	2778/9414	2	2.62	Positive correlation
hsa05414	ilated cardiomyopatl	3/130	96/8521	0.180513	0.604695	0.552962	3696/2778/4000	3	2.05	Positive correlation
hsa03450	-homologous end-joi	1/130	13/8521	0.181273	0.604695	0.552962	4361	1	5.04	Positive correlation
hsa04666	na R-mediated phag	3/130	97/8521	0.184339	0.604695	0.552962	1398/3636/10552	3	2.03	Positive correlation
hsa01522	Endocrine resistance	3/130	98/8521	0.188186	0.604695	0.552962	7157/2778/3845	3	2.01	Positive correlation
hsa04925	rone synthesis and s	3/130	98/8521	0.188186	0.604695	0.552962	1386/2778/4881	3	2.01	Positive correlation
hsa05226	Gastric cancer	4/130	149/8521	0.192757	0.604695	0.552962	7157/8323/3728/3845	4	1.76	Positive correlation
hsa00510	I-Glycan biosynthesi	2/130	53/8521	0.193523	0.604695	0.552962	201595/79644	2	2.47	Positive correlation
hsa00533	ycan biosynthesis -	1/130	14/8521	0.193782	0.604695	0.552962	6482	1	4.68	Positive correlation
hsa04916	Melanogenesis	3/130	101/8521	0.199852	0.61357	0.561078	8323/2778/3845	3	1.95	Positive correlation
hsa05146	Amoebiasis	3/130	102/8521	0.203779	0.61357	0.561078	3910/87/2778	3	1.93	Positive correlation
hsa03460	inconi anemia pathw	2/130	55/8521	0.204659	0.61357	0.561078	57599/2067	2	2.38	Positive correlation
hsa00604	olipid biosynthesis - g	1/130	15/8521	0.206103	0.61357	0.561078	6482	1	4.37	Positive correlation
hsa04921	ytocin signaling path	4/130	154/8521	0.208479	0.61359	0.561097	9475/2778/3845/4881	4	1.7	Positive correlation
hsa04625	tin receptor signaling	3/130	104/8521	0.211687	0.616034	0.563331	23365/3845/868	3	1.89	Positive correlation
hsa00480	lutathione metabolis	2/130	57/8521	0.215865	0.616835	0.564064	10314/2950	2	2.3	Positive correlation
hsa00603	iosynthesis - globo a	1/130	16/8521	0.218236	0.616835	0.564064	6482	1	4.1	Positive correlation
hsa04928	mone synthesis, sec	3/130	106/8521	0.219662	0.616835	0.564064	1386/3727/2778	3	1.86	Positive correlation
hsa05213	Endometrial cancer	2/130	58/8521	0.221489	0.616835	0.564064	7157/3845	2	2.26	Positive correlation
hsa04217	Necroptosis	4/130	159/8521	0.224558	0.618729	0.565796	51652/100526767/330/3320	4	1.65	Positive correlation
hsa00120	ary bile acid biosyntl	1/130	17/8521	0.230185	0.627558	0.57387	8309	1	3.86	Positive correlation
hsa04730	Long-term depressio	2/130	60/8521	0.232773	0.628001	0.574275	2778/3845	2	2.18	Positive correlation
hsa04213	ulating pathway - mu	2/130	61/8521	0.238429	0.636629	0.582164	8660/3845	2	2.15	Positive correlation
hsa00140	oid hormone biosyntl	2/130	62/8521	0.244092	0.63714	0.582631	79154/79644	2	2.11	Positive correlation
hsa00561	lycerolipid metabolis	2/130	63/8521	0.249761	0.63714	0.582631	26007/501	2	2.08	Positive correlation
hsa03420	cleotide excision rep	2/130	63/8521	0.249761	0.63714	0.582631	2968/2067	2	2.08	Positive correlation
hsa05217	Basal cell carcinoma	2/130	63/8521	0.249761	0.63714	0.582631	7157/8323	2	2.08	Positive correlation
hsa04022	P-PKG signaling pat	4/130	167/8521	0.25092	0.63714	0.582631	9475/1386/8660/4881	4	1.57	Positive correlation
hsa05225	epatocellular carcino	4/130	168/8521	0.254263	0.638121	0.583528	7157/8323/2950/3845	4	1.56	Positive correlation
hsa04670	te transendothelial n	3/130	115/8521	0.256234	0.638121	0.583528	9475/87/7070	3	1.71	Positive correlation
hsa04927	ol synthesis and sec	2/130	65/8521	0.26111	0.641344	0.586476	1386/2778	2	2.02	Positive correlation
hsa00100	Steroid biosynthesis	1/130	20/8521	0.264957	0.641344	0.586476	6713	1	3.28	Positive correlation
hsa00670	e carbon pool by fol	1/130	20/8521	0.264957	0.641344	0.586476	6470	1	3.28	Positive correlation
hsa04141	cessing in endoplasn	4/130	173/8521	0.271112	0.642235	0.587291	23640/201595/3320/6500	4	1.52	Positive correlation
hsa05221	cute myeloid leukem	2/130	67/8521	0.272464	0.642235	0.587291	3728/3845	2	1.96	Positive correlation
hsa04722	otrophin signaling pat	3/130	119/8521	0.272764	0.642235	0.587291	7157/1398/3845	3	1.65	Positive correlation
hsa04071	ngolipid signaling pat	3/130	121/8521	0.281074	0.644806	0.589642	7157/9475/3845	3	1.63	Positive correlation
hsa04924	Renin secretion	2/130	69/8521	0.283814	0.644806	0.589642	2778/4881	2	1.9	Positive correlation
hsa05031	mphetamine addictic	2/130	69/8521	0.283814	0.644806	0.589642	1386/2778	2	1.9	Positive correlation
hsa05211	Renal cell carcinoma	2/130	69/8521	0.283814	0.644806	0.589642	1398/3845	2	1.9	Positive correlation
hsa05218	Melanoma	2/130	72/8521	0.300808	0.667433	0.610333	7157/3845	2	1.82	Positive correlation
hsa05223	n-small cell lung can	2/130	72/8521	0.300808	0.667433	0.610333	7157/3845	2	1.82	Positive correlation
hsa00562	tol phosphate metab	2/130	73/8521	0.30646	0.667433	0.610333	51477/3636	2	1.8	Positive correlation
hsa04010	APK signaling pathw	6/130	301/8521	0.310994	0.667433	0.610333	7157/1398/1386/2066/3727/3845	6	1.31	Positive correlation
hsa04926	laxin signaling pathw	3/130	129/8521	0.314508	0.667433	0.610333	1386/2778/3845	3	1.52	Positive correlation
hsa03320	PAR signaling pathw	2/130	75/8521	0.317737	0.667433	0.610333	10580/8309	2	1.75	Positive correlation
hsa04918	yroid hormone synthe	2/130	75/8521	0.317737	0.667433	0.610333	1386/2778	2	1.75	Positive correlation
hsa05214	Glioma	2/130	75/8521	0.317737	0.667433	0.610333	7157/3845	2	1.75	Positive correlation
hsa05212	Pancreatic cancer	2/130	76/8521	0.32336	0.667433	0.610333	7157/3845	2	1.72	Positive correlation
hsa05100	al invasion of epithel	2/130	77/8521	0.328973	0.667433	0.610333	1398/10552	2	1.7	Positive correlation
hsa00563	tidylinositol (GPI)-an	1/130	26/8521	0.329892	0.667433	0.610333	84720	1	2.52	Positive correlation
hsa00980	f xenobiotics by cytc	2/130	78/8521	0.334572	0.667433	0.610333	2950/29785	2	1.68	Positive correlation
hsa05206	MicroRNAs in cance	6/130	310/8521	0.335368	0.667433	0.610333	7157/1398/8660/10018/6541/3845	6	1.27	Positive correlation
hsa04062	nokine signaling patl	4/130	192/8521	0.336559	0.667433	0.610333	1398/9475/3845/56288	4	1.37	Positive correlation
hsa00062	Fatty acid elongatior	1/130	27/8521	0.340147	0.667433	0.610333	117145	1	2.43	Positive correlation
hsa00650	Butanoate metabolisr	1/130	27/8521	0.340147	0.667433	0.610333	622	1	2.43	Positive correlation
hsa00790	Folate biosynthesis	1/130	27/8521	0.340147	0.667433	0.610333	6697	1	2.43	Positive correlation
hsa01521	osine kinase inhibitor	2/130	79/8521	0.340159	0.667433	0.610333	10018/3845	2	1.66	Positive correlation
hsa00983	netabolism - other er	2/130	80/8521	0.345731	0.673266	0.615667	3615/2950	2	1.64	Positive correlation
hsa05162	Measles	3/130	138/8521	0.352233	0.680808	0.622564	7157/1457/868	3	1.42	Positive correlation
hsa04392	aling pathway - multi	1/130	29/8521	0.360191	0.682549	0.624155	84962	1	2.26	Positive correlation
hsa04146	Peroxisome	2/130	83/8521	0.362355	0.682549	0.624155	23417/8309	2	1.58	Positive correlation
hsa04662	receptor signaling pa	2/130	84/8521	0.367862	0.682549	0.624155	3636/3845	2	1.56	Positive correlation
hsa00053	ate and aldarate met	1/130	30/8521	0.369986	0.682549	0.624155	501	1	2.18	Positive correlation
hsa00630	and dicarboxylate n	1/130	30/8521	0.369986	0.682549	0.624155	6470	1	2.18	Positive correlation
hsa01523	Antifolate resistance	1/130	30/8521	0.369986	0.682549	0.624155	6470	1	2.18	Positive correlation
hsa01232	lucleotide metabolisr	2/130	85/8521	0.373351	0.682549	0.624155	3615/79077	2	1.54	Positive correlation
hsa04911	Insulin secretion	2/130	86/8521	0.378821	0.682549	0.624155	1386/2778	2	1.52	Positive correlation
hsa00052	Galactose metabolisr	1/130	32/8521	0.389131	0.682549	0.624155	5214	1	2.05	Positive correlation
hsa00640	ropanoate metabolis	1/130	32/8521	0.389131	0.682549	0.624155	23417	1	2.05	Positive correlation
hsa05224	Breast cancer	3/130	147/8521	0.389735	0.682549	0.624155	7157/8323/3845	3	1.34	Positive correlation
hsa05235	and PD-1 checkpoint	2/130	89/8521	0.395112	0.682549	0.624155	1457/3845	2	1.47	Positive correlation
hsa05410	ertrophic cardiomyop	2/130	90/8521	0.400499	0.682549	0.624155	3696/4000	2	1.46	Positive correlation
hsa05170	nunodeficiency virus	4/130	212/8521	0.40613	0.682549	0.624155	1398/8065/3845/6500	4	1.24	Positive correlation
hsa05207	rcinogenesis - recep	4/130	212/8521	0.40613	0.682549	0.624155	1386/2778/3320/3845	4	1.24	Positive correlation
hsa03020	RNA polymerase	1/130	34/8521	0.407698	0.682549	0.624155	51082	1	1.93	Positive correlation
hsa04710	Circadian rhythm	1/130	34/8521	0.407698	0.682549	0.624155	6500	1	1.93	Positive correlation
hsa01240	osynthesis of cofacto	3/130	153/8521	0.414461	0.682549	0.624155	6697/6470/23475	3	1.29	Positive correlation
hsa05417	oid and atheroscleros	4/130	215/8521	0.416492	0.682549	0.624155	7157/9475/3320/3845	4	1.22	Positive correlation
hsa04912	nRH signaling pathw	2/130	93/8521	0.416527	0.682549	0.624155	2778/3845	2	1.41	Positive correlation
hsa04261	ic signaling in cardio	3/130	154/8521	0.418553	0.682549	0.624155	1386/4635/2778	3	1.28	Positive correlation
hsa04657	-17 signaling pathwa	2/130	94/8521	0.421823	0.682549	0.624155	3727/3320	2	1.39	Positive correlation
hsa04934	Cushing syndrome	3/130	155/8521	0.422636	0.682549	0.624155	8323/1386/2778	3	1.27	Positive correlation
hsa00350	Tyrosine metabolism	1/130	36/8521	0.425705	0.682549	0.624155	81889	1	1.82	Positive correlation
hsa00512	type O-glycan biosy	1/130	36/8521	0.425705	0.682549	0.624155	6482	1	1.82	Positive correlation
hsa04218	Cellular senescence	3/130	156/8521	0.42671	0.682549	0.624155	7157/4361/3845	3	1.26	Positive correlation
hsa00760	and nicotinamide m	1/130	37/8521	0.434504	0.682549	0.624155	23475	1	1.77	Positive correlation
hsa01250	thesis of nucleotide	1/130	37/8521	0.434504	0.682549	0.624155	2821	1	1.77	Positive correlation
hsa04960	e-regulated sodium r	1/130	37/8521	0.434504	0.682549	0.624155	3845	1	1.77	Positive correlation
hsa05143	frican trypanosomias	1/130	37/8521	0.434504	0.682549	0.624155	3910	1	1.77	Positive correlation
hsa04514	ell adhesion molecul	3/130	158/8521	0.434828	0.682549	0.624155	1001/6382/3696	3	1.24	Positive correlation
hsa00564	rophospholipid meta	2/130	99/8521	0.447926	0.698872	0.639082	23171/2820	2	1.32	Positive correlation
hsa05161	Hepatitis B	3/130	162/8521	0.450938	0.699358	0.639527	7157/1386/3845	3	1.21	Positive correlation
hsa04914	ne-mediated oocyte	2/130	102/8521	0.463272	0.71342	0.652386	3320/3845	2	1.29	Positive correlation
hsa03440	nologous recombina	1/130	41/8521	0.468382	0.71342	0.652386	4361	1	1.6	Positive correlation
hsa04216	Ferroptosis	1/130	41/8521	0.468382	0.71342	0.652386	7157	1	1.6	Positive correlation
hsa04064	appa B signaling pat	2/130	104/8521	0.473364	0.71342	0.652386	1457/330	2	1.26	Positive correlation
hsa00380	ryptophan metabolis	1/130	42/8521	0.476532	0.71342	0.652386	501	1	1.56	Positive correlation
hsa00513	ypes of N-glycan bio	1/130	42/8521	0.476532	0.71342	0.652386	201595	1	1.56	Positive correlation
hsa00071	atty acid degradatio	1/130	43/8521	0.484557	0.721267	0.659561	501	1	1.52	Positive correlation
hsa03410	Base excision repair	1/130	44/8521	0.492461	0.721697	0.659955	143	1	1.49	Positive correlation
hsa04962	n-regulated water re	1/130	44/8521	0.492461	0.721697	0.659955	2778	1	1.49	Positive correlation
hsa04931	Insulin resistance	2/130	108/8521	0.493206	0.721697	0.659955	8660/2997	2	1.21	Positive correlation
hsa03022	sal transcription fact	1/130	45/8521	0.500245	0.727884	0.665612	2968	1	1.46	Positive correlation
hsa05145	Toxoplasmosis	2/130	111/8521	0.507778	0.730825	0.668302	3910/330	2	1.18	Positive correlation
hsa04930	ype II diabetes mellit	1/130	46/8521	0.50791	0.730825	0.668302	8660	1	1.42	Positive correlation
hsa00620	Pyruvate metabolism	1/130	47/8521	0.515458	0.737589	0.674487	501	1	1.39	Positive correlation
hsa04668	NF signaling pathwa	2/130	114/8521	0.522078	0.741505	0.678068	1386/330	2	1.15	Positive correlation
hsa04724	Glutamatergic synaps	2/130	115/8521	0.526784	0.741505	0.678068	54407/2778	2	1.14	Positive correlation
hsa04726	Serotonergic synaps	2/130	115/8521	0.526784	0.741505	0.678068	2778/3845	2	1.14	Positive correlation
hsa00520	and nucleotide suga	1/130	49/8521	0.530211	0.742296	0.678791	2821	1	1.34	Positive correlation
hsa05144	Malaria	1/130	50/8521	0.53742	0.748343	0.684321	6382	1	1.31	Positive correlation
hsa04913	varian steroidogenes	1/130	51/8521	0.544519	0.754174	0.689653	2778	1	1.29	Positive correlation
hsa05034	Alcoholism	3/130	188/8521	0.550447	0.758329	0.693452	1386/2778/3845	3	1.05	Positive correlation
hsa04660	receptor signaling pa	2/130	121/8521	0.554359	0.759677	0.694685	3845/868	2	1.08	Positive correlation
hsa04961	r factor-regulated ca	1/130	53/8521	0.558394	0.76118	0.696059	2778	1	1.24	Positive correlation
hsa05202	ptional misregulation	3/130	193/8521	0.568365	0.770715	0.704779	7157/330/3728	3	1.02	Positive correlation
hsa00230	Purine metabolism	2/130	128/8521	0.585085	0.789255	0.721733	3615/4881	2	1.02	Positive correlation
hsa00240	Pyrimidine metabolisr	1/130	58/8521	0.591274	0.792613	0.724804	79077	1	1.13	Positive correlation
hsa04370	EGF signaling pathw	1/130	59/8521	0.597553	0.792613	0.724804	3845	1	1.11	Positive correlation
hsa05169	stein-Barr virus infec	3/130	202/8521	0.599506	0.792613	0.724804	7157/10018/7874	3	0.97	Positive correlation
hsa04728	opaminergic synaps	2/130	132/8521	0.601936	0.792613	0.724804	1386/2778	2	0.99	Positive correlation
hsa05415	abetic cardiomyopat	3/130	203/8521	0.602876	0.792613	0.724804	509/498/2997	3	0.97	Positive correlation
hsa00590	chidonic acid metabo	1/130	61/8521	0.609824	0.794138	0.726198	8529	1	1.07	Positive correlation
hsa00190	idative phosphorylat	2/130	134/8521	0.610168	0.794138	0.726198	509/498	2	0.98	Positive correlation
hsa00310	Lysine degradation	1/130	63/8521	0.621724	0.798685	0.730356	501	1	1.04	Positive correlation
hsa04929	GnRH secretion	1/130	64/8521	0.627538	0.798685	0.730356	3845	1	1.02	Positive correlation
hsa04371	pelin signaling pathw	2/130	139/8521	0.630184	0.798685	0.730356	4635/3845	2	0.94	Positive correlation
hsa04140	Autophagy - animal	2/130	141/8521	0.637967	0.798685	0.730356	8660/3845	2	0.93	Positive correlation
hsa00970	noacyl-tRNA biosynth	1/130	66/8521	0.638901	0.798685	0.730356	10056	1	0.99	Positive correlation
hsa04936	Alcoholic liver diseas	2/130	142/8521	0.64181	0.798685	0.730356	23417/501	2	0.92	Positive correlation
hsa04720	ong-term potentiatio	1/130	67/8521	0.644454	0.798685	0.730356	3845	1	0.98	Positive correlation
hsa04550	's regulating pluripot	2/130	143/8521	0.645621	0.798685	0.730356	8323/3845	2	0.92	Positive correlation
hsa00830	Retinol metabolism	1/130	68/8521	0.649921	0.798685	0.730356	29785	1	0.96	Positive correlation
hsa04664	silon RI signaling pa	1/130	68/8521	0.649921	0.798685	0.730356	3845	1	0.96	Positive correlation
hsa04920	cytokine signaling pa	1/130	69/8521	0.655305	0.798685	0.730356	8660	1	0.95	Positive correlation
hsa05204	carcinogenesis - DN	1/130	69/8521	0.655305	0.798685	0.730356	2950	1	0.95	Positive correlation
hsa04917	lactin signaling path	1/130	70/8521	0.660607	0.798685	0.730356	3845	1	0.94	Positive correlation
hsa05166	-cell leukemia virus 1	3/130	222/8521	0.663334	0.798685	0.730356	7157/1386/3845	3	0.89	Positive correlation
hsa04072	olipase D signaling p	2/130	148/8521	0.664202	0.798685	0.730356	2778/3845	2	0.89	Positive correlation
hsa04622	e receptor signaling	1/130	71/8521	0.665827	0.798685	0.730356	26007	1	0.92	Positive correlation
hsa05208	ogenesis - reactive	3/130	223/8521	0.666325	0.798685	0.730356	509/498/3845	3	0.88	Positive correlation
hsa00982	tabolism - cytochror	1/130	72/8521	0.670969	0.798685	0.730356	2950	1	0.91	Positive correlation
hsa04024	AMP signaling pathw	3/130	225/8521	0.672252	0.798685	0.730356	9475/2778/4881	3	0.87	Positive correlation
hsa04115	53 signaling pathwa	1/130	74/8521	0.681016	0.802451	0.7338	7157	1	0.89	Positive correlation
hsa04623	olic DNA-sensing pa	1/130	75/8521	0.685926	0.802451	0.7338	51082	1	0.87	Positive correlation
hsa04932	lcoholic fatty liver di	2/130	155/8521	0.688901	0.802451	0.7338	8660/10018	2	0.85	Positive correlation
hsa04971	Gastric acid secretio	1/130	76/8521	0.69076	0.802451	0.7338	2778	1	0.86	Positive correlation
hsa04150	TOR signaling pathw	2/130	156/8521	0.692306	0.802451	0.7338	8323/3845	2	0.84	Positive correlation
hsa04110	Cell cycle	2/130	157/8521	0.69568	0.802451	0.7338	7157/6500	2	0.83	Positive correlation
hsa05160	Hepatitis C	2/130	158/8521	0.699025	0.802451	0.7338	7157/3845	2	0.83	Positive correlation
hsa04612	processing and pres	1/130	78/8521	0.700208	0.802451	0.7338	3320	1	0.84	Positive correlation
hsa04260	diac muscle contrac	1/130	87/8521	0.7393	0.843519	0.771355	4635	1	0.75	Positive correlation
hsa04727	GABAergic synapse	1/130	89/8521	0.747276	0.845172	0.772866	54407	1	0.74	Positive correlation
hsa04976	Bile secretion	1/130	89/8521	0.747276	0.845172	0.772866	2778	1	0.74	Positive correlation
hsa05032	Morphine addiction	1/130	91/8521	0.755009	0.850206	0.777469	2778	1	0.72	Positive correlation
hsa04970	Salivary secretion	1/130	93/8521	0.762507	0.853442	0.780429	2778	1	0.7	Positive correlation
hsa03015	NA surveillance path	1/130	97/8521	0.776827	0.853442	0.780429	23708	1	0.68	Positive correlation
hsa04070	atidylinositol signaling	1/130	97/8521	0.776827	0.853442	0.780429	3636	1	0.68	Positive correlation
hsa04713	Circadian entrainmer	1/130	97/8521	0.776827	0.853442	0.780429	2778	1	0.68	Positive correlation
hsa04750	nediator regulation o	1/130	98/8521	0.780271	0.853442	0.780429	2778	1	0.67	Positive correlation
hsa05231	ine metabolism in ca	1/130	98/8521	0.780271	0.853442	0.780429	3845	1	0.67	Positive correlation
hsa04621	e receptor signaling	2/130	186/8521	0.780949	0.853442	0.780429	330/3320	2	0.7	Positive correlation
hsa04933	aling pathway in diab	1/130	100/8521	0.787002	0.856222	0.78297	3845	1	0.66	Positive correlation
hsa04972	Pancreatic secretion	1/130	102/8521	0.793528	0.856222	0.78297	2778	1	0.64	Positive correlation
hsa05142	Chagas disease	1/130	102/8521	0.793528	0.856222	0.78297	2778	1	0.64	Positive correlation
hsa04974	n digestion and absc	1/130	103/8521	0.796716	0.856222	0.78297	54407	1	0.64	Positive correlation
hsa05167	na-associated herpe	2/130	194/8521	0.800496	0.856729	0.783434	7157/3845	2	0.68	Positive correlation
hsa03013	eocytoplasmic trans	1/130	108/8521	0.811939	0.857906	0.78451	9631	1	0.61	Positive correlation
hsa04350	F-beta signaling path	1/130	108/8521	0.811939	0.857906	0.78451	6500	1	0.61	Positive correlation
hsa04659	h17 cell differentiatic	1/130	108/8521	0.811939	0.857906	0.78451	3320	1	0.61	Positive correlation
hsa04066	F-1 signaling pathwa	1/130	109/8521	0.814845	0.857906	0.78451	5214	1	0.6	Positive correlation
hsa04725	Cholinergic synapse	1/130	113/8521	0.82603	0.866161	0.792059	3845	1	0.58	Positive correlation
hsa04380	steoclast differentiati	1/130	128/8521	0.862317	0.898402	0.821543	3727	1	0.51	Positive correlation
hsa04114	Oocyte meiosis	1/130	131/8521	0.868617	0.898402	0.821543	6500	1	0.5	Positive correlation
hsa04142	Lysosome	1/130	132/8521	0.870653	0.898402	0.821543	10947	1	0.5	Positive correlation
hsa04650	iller cell mediated cy	1/130	132/8521	0.870653	0.898402	0.821543	3845	1	0.5	Positive correlation
hsa05322	emic lupus erythema	1/130	137/8521	0.880371	0.904826	0.827416	87	1	0.48	Positive correlation
hsa04020	lcium signaling pathv	2/130	253/8521	0.902816	0.924227	0.845158	2066/2778	2	0.52	Positive correlation
hsa04080	ive ligand-receptor ir	3/130	367/8521	0.923627	0.939717	0.859323	10424/10857/56288	3	0.54	Positive correlation
hsa03010	Ribosome	1/130	167/8521	0.925204	0.939717	0.859323	6204	1	0.39	Positive correlation
hsa05152	Tuberculosis	1/130	180/8521	0.939009	0.950013	0.868738	23365	1	0.36	Positive correlation
hsa05171	avirus disease - CO\	1/130	232/8521	0.973121	0.978546	0.89483	6204	1	0.28	Positive correlation
hsa04014	Ras signaling pathwa	1/130	236/8521	0.974768	0.978546	0.89483	3845	1	0.28	Positive correlation
hsa04740	Olfactory transductio	1/130	439/8521	0.999022	0.999022	0.913554	83988	1	0.15	Positive correlation

Supplementary Table 18. Kyoto Encyclopedia of Genes and Genomes enrichment analysis of genes negatively associated with SH3YL1 expression

ID	Description	GeneRatio	BgRatio	pvalue	p.adjust	qvalue	geneID	Count	Fold_enrichment	type
hsa05203	Viral carcinogenesis	25/147	204/8521	6.17E-15	1.47E-12	1.18E-12	67/8368/8370/554313/3107/10379/753	25	7.1	Negative correlation
hsa04613	il extracellular trap f	23/147	191/8521	1.22E-13	1.46E-11	1.17E-11	8366/8367/8368/8370/554313/60/3683	23	6.98	Negative correlation
hsa05322	emic lupus erythema	17/147	137/8521	1.51E-10	1.2E-08	9.57E-09	363/8364/8365/8366/8367/8368/8370/	17	7.19	Negative correlation
hsa05169	stein-Barr virus infec	20/147	202/8521	2.07E-10	1.24E-08	9.87E-09	772/3683/567/1432/3105/5704/3106/4	20	5.74	Negative correlation
hsa05034	Alcoholism	15/147	188/8521	7.55E-07	3.61E-05	2.88E-05	62/8363/8364/8365/8366/8367/8368/8	15	4.62	Negative correlation
hsa04612	processing and pres	9/147	78/8521	7.02E-06	0.000279	0.000223	/6892/6891/3107/567/3105/3106/1520/	9	6.69	Negative correlation
hsa05171	avirus disease - CO	15/147	232/8521	1.03E-05	0.000353	0.000281	32/6173/6773/5579/834/713/4790/6230	15	3.75	Negative correlation
hsa05170	nunodeficiency virus	14/147	212/8521	1.63E-05	0.000487	0.000388	07/567/1432/3105/3106/59345/8905/55	14	3.83	Negative correlation
hsa05167	na-associated herpe	13/147	194/8521	2.84E-05	0.000755	0.000602	1432/3105/3106/4067/59345/6773/479	13	3.88	Negative correlation
hsa05416	Viral myocarditis	7/147	60/8521	7.08E-05	0.001693	0.00135	60/3107/3683/1982/3105/3106/836	7	6.76	Negative correlation
hsa04650	iller cell mediated cy	10/147	132/8521	8.9E-05	0.001933	0.001541	52/3683/3105/3106/5579/3937/7305/83	10	4.39	Negative correlation
hsa05135	Yersinia infection	10/147	137/8521	0.000121	0.00242	0.001929	2/391/7456/834/4790/7454/63916/393	10	4.23	Negative correlation
hsa05133	Pertussis	7/147	76/8521	0.000319	0.005628	0.004487	1432/834/713/4790/714/836/929	7	5.34	Negative correlation
hsa05100	al invasion of epithe	7/147	77/8521	0.000346	0.005628	0.004487	60/55752/3059/391/63916/2335/867	7	5.27	Negative correlation
hsa04380	steoclast differentiati	9/147	128/8521	0.000353	0.005628	0.004487	/6772/1432/6773/4790/3937/2209/730	9	4.08	Negative correlation
hsa04625	tin receptor signalin	8/147	104/8521	0.000413	0.00617	0.004919	379/6772/1432/6773/834/4790/4046/68	8	4.46	Negative correlation
hsa05163	cytomegalovirus in	12/147	225/8521	0.000496	0.006978	0.005563	91/3107/567/1432/3105/3106/59345/5	12	3.09	Negative correlation
hsa05162	Measles	9/147	138/8521	0.000614	0.00815	0.006497	10379/6776/6772/9367/6773/4790/460	9	3.78	Negative correlation
hsa05164	Influenza A	10/147	171/8521	0.000725	0.009123	0.007273	9/10379/6772/6773/5579/834/4790/46	10	3.39	Negative correlation
hsa05205	roteoglycans in canc	11/147	205/8521	0.000811	0.009696	0.007729	/1432/288/3059/5579/967/960/2335/8	11	3.11	Negative correlation
hsa05152	Tuberculosis	10/147	180/8521	0.001077	0.012028	0.009588	72/1432/1520/4790/4046/2209/836/92	10	3.22	Negative correlation
hsa03050	Proteasome	5/147	46/8521	0.001107	0.012028	0.009588	5699/5704/5698/5719/5721	5	6.3	Negative correlation
hsa04145	Phagosome	9/147	152/8521	0.001225	0.012482	0.009951	0/6891/60/3107/3105/3106/1520/2209/	9	3.43	Negative correlation
hsa04144	Endocytosis	12/147	250/8521	0.001253	0.012482	0.009951	3106/23362/51100/7456/1175/30011/8	12	2.78	Negative correlation
hsa04666	a R-mediated phag	7/147	97/8521	0.001387	0.013257	0.010568	4082/4067/5579/3635/7454/2209/6850	7	4.18	Negative correlation
hsa04933	ling pathway in diab	7/147	100/8521	0.001655	0.015218	0.012131	6776/6772/1432/5579/4790/2335/836	7	4.06	Negative correlation
hsa05143	rican trypanosomias	4/147	37/8521	0.003608	0.03194	0.025462	3620/3039/3040/5579	4	6.27	Negative correlation
hsa05132	Salmonella infection	11/147	249/8521	0.003794	0.032384	0.025816	/1432/9367/391/834/4790/63916/3071/	11	2.56	Negative correlation
hsa04611	Platelet activation	7/147	124/8521	0.005532	0.045589	0.036342	60/83706/1432/4067/3937/2244/6850	7	3.27	Negative correlation
hsa04062	nokine signaling pat	9/147	192/8521	0.005908	0.045675	0.036411	794/4067/59345/6773/5579/4790/7454	9	2.72	Negative correlation
hsa05160	Hepatitis C	8/147	158/8521	0.005924	0.045675	0.036411	39/10379/7532/6772/6773/4790/4600/8	8	2.93	Negative correlation
hsa04664	silon RI signaling pa	5/147	68/8521	0.006222	0.04647	0.037045	1432/4067/3635/3937/6850	5	4.26	Negative correlation
hsa05130	nic Escherichia coli	9/147	198/8521	0.007188	0.051318	0.04091	432/3059/7456/834/4790/3071/836/64	9	2.63	Negative correlation
hsa05020	Prion disease	11/147	272/8521	0.0073	0.051318	0.04091	4/713/4700/5719/1340/714/836/1345/3	11	2.34	Negative correlation
hsa05146	Amoebiasis	6/147	102/8521	0.008253	0.056355	0.044925	5579/4790/2335/836/929/3915	6	3.41	Negative correlation
hsa05140	Leishmaniasis	5/147	77/8521	0.010428	0.069234	0.055192	6772/1432/5579/4790/2209	5	3.76	Negative correlation
hsa04662	receptor signaling pa	5/147	84/8521	0.014813	0.094628	0.075436	4067/5579/4790/3635/6850	5	3.45	Negative correlation
hsa04621	e receptor signaling	8/147	186/8521	0.015045	0.094628	0.075436	9/10379/6772/1432/6773/140609/834/4	8	2.49	Negative correlation
hsa05134	Legionellosis	4/147	56/8521	0.015606	0.095638	0.076241	834/4790/836/929	4	4.14	Negative correlation
hsa04932	lcoholic fatty liver di	7/147	155/8521	0.017618	0.105268	0.083918	1432/4790/4700/1340/836/1345/4697	7	2.62	Negative correlation
hsa04217	Necroptosis	7/147	159/8521	0.019974	0.116436	0.09282	817/10379/6776/6772/6773/834/29082	7	2.55	Negative correlation
hsa05161	Hepatitis B	7/147	162/8521	0.02188	0.12451	0.099257	6776/6772/1432/6773/5579/4790/836	7	2.5	Negative correlation
hsa05415	abetic cardiomyopat	8/147	203/8521	0.024083	0.133858	0.106709	7/1432/5579/4790/4700/1340/1345/46	8	2.28	Negative correlation
hsa05150	ylococcus aureus inf	5/147	96/8521	0.024924	0.135382	0.107924	3683/713/2209/3858/714	5	3.02	Negative correlation
hsa05131	Shigellosis	9/147	247/8521	0.026951	0.140376	0.111905	55752/1432/3059/834/4790/960/63916/	9	2.11	Negative correlation
hsa05330	Allograft rejection	3/147	38/8521	0.027323	0.140376	0.111905	3107/3105/3106	3	4.58	Negative correlation
hsa05165	n papillomavirus inf	11/147	331/8521	0.027788	0.140376	0.111905	9/6772/3105/3106/6773/4790/2335/460	11	1.93	Negative correlation
hsa05221	cute myeloid leukem	4/147	67/8521	0.028193	0.140376	0.111905	6776/4790/2209/929	4	3.46	Negative correlation
hsa05417	oid and atheroscleros	8/147	215/8521	0.032409	0.151974	0.121151	317/7184/1432/4067/834/4790/836/929	8	2.16	Negative correlation
hsa04917	lactin signaling path	4/147	70/8521	0.03243	0.151974	0.121151	6776/6772/1432/4790	4	3.31	Negative correlation
hsa05120	naling in Helicobacte	4/147	70/8521	0.03243	0.151974	0.121151	1432/4067/4790/836	4	3.31	Negative correlation
hsa04064	appa B signaling pat	5/147	104/8521	0.03366	0.154704	0.123327	4067/5579/4790/929/6850	5	2.79	Negative correlation
hsa05332	aft-versus-host disea	3/147	42/8521	0.035386	0.159383	0.127057	3107/3105/3106	3	4.14	Negative correlation
hsa04936	lcoholic liver diseas	6/147	142/8521	0.036011	0.159383	0.127057	1432/713/4790/714/836/929	6	2.45	Negative correlation
hsa04940	ype I diabetes mellit	3/147	43/8521	0.037573	0.162535	0.12957	3107/3105/3106	3	4.04	Negative correlation
hsa05166	-cell leukemia virus 1	8/147	222/8521	0.038084	0.162535	0.12957	07/6776/3683/567/3105/3106/55697/47	8	2.09	Negative correlation
hsa04623	olic DNA-sensing pa	4/147	75/8521	0.040279	0.168887	0.134634	834/25939/4790/836	4	3.09	Negative correlation
hsa05145	Toxoplasmosis	5/147	111/8521	0.042694	0.175928	0.140247	6772/1432/4790/836/3915	5	2.61	Negative correlation
hsa05144	Malaria	3/147	50/8521	0.054748	0.220938	0.176128	3039/3040/3683	3	3.48	Negative correlation
hsa04514	ell adhesion molecul	6/147	158/8521	0.055466	0.220938	0.176128	3107/3683/1462/3105/3106/6614	6	2.2	Negative correlation
hsa04012	rbB signaling pathwa	4/147	85/8521	0.058941	0.227626	0.181459	817/6776/5579/867	4	2.73	Negative correlation
hsa03060	Protein export	2/147	23/8521	0.059049	0.227626	0.181459	23478/6729	2	5.04	Negative correlation
hsa04610	ent and coagulation	4/147	86/8521	0.061023	0.231499	0.184547	3818/713/2244/714	4	2.7	Negative correlation
hsa05320	immune thyroid dise	3/147	53/8521	0.063075	0.235544	0.187771	3107/3105/3106	3	3.28	Negative correlation
hsa05235	and PD-1 checkpoin	4/147	89/8521	0.0675	0.245108	0.195395	6772/1432/27436/4790	4	2.61	Negative correlation
hsa05010	Alzheimer disease	11/147	384/8521	0.067687	0.245108	0.195395	/3416/4790/4700/5719/1340/23385/83	11	1.66	Negative correlation
hsa04658	and Th2 cell different	4/147	92/8521	0.074319	0.261211	0.208233	6776/6772/1432/4790	4	2.52	Negative correlation
hsa05222	Small cell lung cance	4/147	92/8521	0.074319	0.261211	0.208233	4790/2335/836/3915	4	2.52	Negative correlation
hsa00240	yrimidine metabolis	3/147	58/8521	0.078162	0.266109	0.212137	1633/1890/1806	3	3	Negative correlation
hsa04966	ecting duct acid secr	2/147	27/8521	0.078379	0.266109	0.212137	1188/10723	2	4.29	Negative correlation
hsa04657	-17 signaling pathwa	4/147	94/8521	0.079053	0.266109	0.212137	7184/1432/4790/836	4	2.47	Negative correlation
hsa04370	EGF signaling pathw	3/147	59/8521	0.081354	0.269245	0.214637	7867/1432/5579	3	2.95	Negative correlation
hsa05016	Huntington disease	9/147	306/8521	0.082238	0.269245	0.214637	/5704/1175/4700/5719/1340/836/1345/	9	1.7	Negative correlation
hsa05012	Parkinson disease	8/147	266/8521	0.088963	0.287326	0.229051	17/5704/4700/5719/1340/836/1345/46	8	1.74	Negative correlation
hsa01523	Antifolate resistance	2/147	30/8521	0.093984	0.297703	0.237323	4790/2350	2	3.86	Negative correlation
hsa03250	Viral life cycle - HIV-	3/147	63/8521	0.094667	0.297703	0.237323	25939/4600/29082	3	2.76	Negative correlation
hsa05014	otrophic lateral scle	10/147	364/8521	0.097978	0.304113	0.242433	32/5704/834/4700/5719/1340/836/134	10	1.59	Negative correlation
hsa05142	Chagas disease	4/147	102/8521	0.099436	0.304683	0.242888	1432/713/4790/714	4	2.27	Negative correlation
hsa04620	receptor signaling p	4/147	104/8521	0.104881	0.317298	0.252944	6772/1432/4790/929	4	2.23	Negative correlation
hsa04723	de endocannabinoid	5/147	148/8521	0.112533	0.336192	0.268006	1432/59345/5579/4700/4697	5	1.96	Negative correlation
hsa04659	h17 cell differentiatio	4/147	108/8521	0.116169	0.34277	0.273249	6776/6772/1432/4790	4	2.15	Negative correlation
hsa03030	DNA replication	2/147	36/8521	0.127482	0.367288	0.292795	84153/6118	2	3.22	Negative correlation
hsa05223	n-small cell lung can	3/147	72/8521	0.127552	0.367288	0.292795	6776/5579/27436	3	2.42	Negative correlation
hsa04218	Cellular senescence	5/147	156/8521	0.13227	0.37634	0.300011	3107/1432/3105/3106/4790	5	1.86	Negative correlation
hsa04670	te transendothelial m	4/147	115/8521	0.137131	0.385579	0.307377	60/3683/1432/5579	4	2.02	Negative correlation
hsa05340	nary immunodeficie	2/147	38/8521	0.139198	0.386842	0.308383	6890/6891	2	3.05	Negative correlation
hsa04971	Gastric acid secretio	3/147	76/8521	0.143315	0.389231	0.310288	817/60/5579	3	2.29	Negative correlation
hsa05220	ronic myeloid leuke	3/147	76/8521	0.143315	0.389231	0.310288	6776/4790/867	3	2.29	Negative correlation
hsa03266	Virion - Herpesvirus	1/147	2418516	0.145033	0.389471	0.310479	3416	1	6.44	Negative correlation
hsa04722	trophin signaling pa	4/147	119/8521	0.149751	0.397672	0.317017	817/1432/4790/397	4	1.95	Negative correlation
hsa04935	ne synthesis, secret	4/147	120/8521	0.152975	0.401768	0.320282	6776/6772/1432/5579	4	1.93	Negative correlation
hsa04015	ap1 signaling pathwa	6/147	210/8521	0.154926	0.402472	0.320843	60/3683/1432/51466/5579/3937	6	1.66	Negative correlation
hsa00130	other terpenoid-quin	1/147	2418577	0.17431	0.447958	0.357104	51004	1	5.27	Negative correlation
hsa02010	ABC transporters	2/147	45/8521	0.181781	0.462187	0.368447	6890/6891	2	2.58	Negative correlation
hsa05208	ogenesis - reactive	6/147	223/8521	0.187582	0.471916	0.376203	1432/4790/4700/1340/1345/4697	6	1.56	Negative correlation
hsa04142	Lysosome	4/147	132/8521	0.193572	0.473822	0.377722	2799/8905/1520/967	4	1.76	Negative correlation
hsa04728	opaminergic synaps	4/147	132/8521	0.193572	0.473822	0.377722	817/1432/59345/5579	4	1.76	Negative correlation
hsa00514	oes of O-glycan bios	2/147	47/8521	0.194287	0.473822	0.377722	8985/79709	2	2.47	Negative correlation
hsa04512	CM-receptor interacti	3/147	89/8521	0.198397	0.47447	0.378239	960/2335/3915	3	1.95	Negative correlation
hsa05022	urodegeneration - m	11/147	476/8521	0.198523	0.47447	0.378239	5704/5579/4790/4700/5719/1340/836/	11	1.34	Negative correlation
hsa00190	idative phosphorylat	4/147	134/8521	0.200648	0.4748	0.378501	4700/1340/1345/4697	4	1.73	Negative correlation
hsa04810	ation of actin cytosk	6/147	229/8521	0.203502	0.476834	0.380123	60/3683/10788/7114/2335/3071	6	1.52	Negative correlation
hsa04210	Apoptosis	4/147	136/8521	0.2078	0.482177	0.384383	60/1520/4790/836	4	1.7	Negative correlation
hsa04714	Thermogenesis	6/147	232/8521	0.211645	0.486376	0.38773	60/1432/4700/1340/1345/4697	6	1.5	Negative correlation
hsa04912	nRH signaling pathw	3/147	93/8521	0.21628	0.492294	0.392448	817/1432/5579	3	1.87	Negative correlation
hsa05418	ar stress and athero	4/147	139/8521	0.218666	0.49303	0.393034	60/7184/1432/4790	4	1.67	Negative correlation
hsa04961	er factor-regulated ca	2/147	53/8521	0.23236	0.514152	0.409873	5579/1175	2	2.19	Negative correlation
hsa04070	tidylinositol signaling	3/147	97/8521	0.234488	0.514152	0.409873	5579/3635/3613	3	1.79	Negative correlation
hsa04713	Circadian entrainmer	3/147	97/8521	0.234488	0.514152	0.409873	817/59345/5579	3	1.79	Negative correlation
hsa04750	nediator regulation o	3/147	98/8521	0.239084	0.519463	0.414106	817/1432/5579	3	1.77	Negative correlation
hsa04640	matopoietic cell linea	3/147	99/8521	0.243695	0.524712	0.418291	960/2209/929	3	1.76	Negative correlation
hsa04510	Focal adhesion	5/147	203/8521	0.272917	0.582386	0.464268	60/5579/2335/7791/3915	5	1.43	Negative correlation
hsa04730	ong-term depressio	2/147	60/8521	0.277273	0.586445	0.467503	4067/5579	2	1.93	Negative correlation
hsa00531	saminoglycan degra	1/147	19/8521	0.281789	0.590768	0.470949	2799	1	3.05	Negative correlation
hsa04330	otch signaling pathw	2/147	62/8521	0.290107	0.595521	0.474738	7090/23385	2	1.87	Negative correlation
hsa04066	IF-1 signaling pathwa	3/147	109/8521	0.290461	0.595521	0.474738	817/5579/4790	3	1.6	Negative correlation
hsa00100	Steroid biosynthesis	1/147	20/8521	0.294207	0.595521	0.474738	3930	1	2.9	Negative correlation
hsa00310	Lysine degradation	2/147	63/8521	0.296515	0.595521	0.474738	8985/79709	2	1.84	Negative correlation
hsa03420	cleotide excision rep	2/147	63/8521	0.296515	0.595521	0.474738	5436/6118	2	1.84	Negative correlation
hsa00770	enate and CoA bios	1/147	21/8521	0.306411	0.606083	0.483158	1806	1	2.76	Negative correlation
hsa05321	mmatory bowel dise	2/147	65/8521	0.309304	0.606083	0.483158	6772/4790	2	1.78	Negative correlation
hsa04725	Cholinergic synapse	3/147	113/8521	0.309381	0.606083	0.483158	817/59345/5579	3	1.54	Negative correlation
hsa04668	NF signaling pathwa	3/147	114/8521	0.31412	0.610363	0.48657	1432/4790/836	3	1.53	Negative correlation
hsa04726	Serotonergic synaps	3/147	115/8521	0.318861	0.610868	0.486973	59345/5579/836	3	1.51	Negative correlation
hsa04630	-STAT signaling pat	4/147	166/8521	0.321653	0.610868	0.486973	10379/6776/6772/6773	4	1.4	Negative correlation
hsa04720	ong-term potentiatio	2/147	67/8521	0.322048	0.610868	0.486973	817/5579	2	1.73	Negative correlation
hsa03430	Mismatch repair	1/147	23/8521	0.330195	0.621391	0.495361	6118	1	2.52	Negative correlation
hsa05031	mphetamine addictio	2/147	69/8521	0.334734	0.624188	0.497591	817/5579	2	1.68	Negative correlation
hsa04530	Tight junction	4/147	170/8521	0.337368	0.624188	0.497591	60/23370/3059/7454	4	1.36	Negative correlation
hsa04071	ngolipid signaling pat	3/147	121/8521	0.347307	0.624188	0.497591	1432/5579/4790	3	1.44	Negative correlation
hsa04660	receptor signaling pa	3/147	121/8521	0.347307	0.624188	0.497591	1432/4790/3937	3	1.44	Negative correlation
hsa04919	hormone signaling p	3/147	121/8521	0.347307	0.624188	0.497591	60/6772/5579	3	1.44	Negative correlation
hsa04622	e receptor signaling	2/147	71/8521	0.347351	0.624188	0.497591	1432/4790	2	1.63	Negative correlation
hsa00562	ol phosphate metab	2/147	73/8521	0.359891	0.641894	0.511706	3635/3613	2	1.59	Negative correlation
hsa04918	roid hormone synth	2/147	75/8521	0.372342	0.654336	0.521624	7184/5579	2	1.55	Negative correlation
hsa05214	Glioma	2/147	75/8521	0.372342	0.654336	0.521624	817/5579	2	1.55	Negative correlation
hsa05212	Pancreatic cancer	2/147	76/8521	0.378532	0.660358	0.526425	6772/4790	2	1.53	Negative correlation
hsa04926	laxin signaling pathw	3/147	129/8521	0.385047	0.666857	0.531606	1432/59345/4790	3	1.35	Negative correlation
hsa04721	Synaptic vesicle cycl	2/147	78/8521	0.390835	0.672011	0.535715	1175/8775	2	1.49	Negative correlation
hsa04114	Oocyte meiosis	3/147	131/8521	0.394415	0.673323	0.53676	817/7532/1432	3	1.33	Negative correlation
hsa00983	netabolism - other er	2/147	80/8521	0.40303	0.68315	0.544595	1890/1806	2	1.45	Negative correlation
hsa00030	tose phosphate path	1/147	30/8521	0.407236	0.685419	0.546403	6888	1	1.93	Negative correlation
hsa04151	K-Akt signaling path	7/147	354/8521	0.411318	0.687447	0.54802	'184/7532/59345/4790/2335/6850/391	7	1.15	Negative correlation
hsa00410	ta-Alanine metabolis	1/147	31/8521	0.417498	0.690042	0.550089	1806	1	1.87	Negative correlation
hsa04010	APK signaling pathw	6/147	301/8521	0.418645	0.690042	0.550089	7867/1432/5579/4790/836/929	6	1.16	Negative correlation
hsa04215	ptosis - multiple spe	1/147	32/8521	0.427584	0.699949	0.557987	836	1	1.81	Negative correlation
hsa01232	ucleotide metabolis	2/147	85/8521	0.433	0.702509	0.560027	1633/1890	2	1.36	Negative correlation
hsa04130	teractions in vesicula	1/147	33/8521	0.437496	0.702509	0.560027	9570	1	1.76	Negative correlation
hsa04911	Insulin secretion	2/147	86/8521	0.438899	0.702509	0.560027	817/5579	2	1.35	Negative correlation
hsa04260	diac muscle contrac	2/147	87/8521	0.444765	0.702509	0.560027	1340/1345	2	1.33	Negative correlation
hsa00051	e and mannose met	1/147	34/8521	0.447238	0.702509	0.560027	57103	1	1.7	Negative correlation
hsa03020	RNA polymerase	1/147	34/8521	0.447238	0.702509	0.560027	5436	1	1.7	Negative correlation
hsa05017	Spinocerebellar ataxi	3/147	143/8521	0.449723	0.702509	0.560027	5704/5579/5719	3	1.22	Negative correlation
hsa04727	GABAergic synapse	2/147	89/8521	0.456395	0.708301	0.564644	59345/5579	2	1.3	Negative correlation
hsa05410	ertrophic cardiomyop	2/147	90/8521	0.462157	0.712617	0.568085	60/7273	2	1.29	Negative correlation
hsa05032	Morphine addiction	2/147	91/8521	0.467885	0.716823	0.571438	59345/5579	2	1.27	Negative correlation
hsa01250	thesis of nucleotide	1/147	37/8521	0.475469	0.719223	0.573351	55577	1	1.57	Negative correlation
hsa04960	-regulated sodium r	1/147	37/8521	0.475469	0.719223	0.573351	5579	1	1.57	Negative correlation
hsa04520	Adherens junction	2/147	93/8521	0.479231	0.720334	0.574237	60/7454	2	1.25	Negative correlation
hsa05414	ilated cardiomyopat	2/147	96/8521	0.495972	0.720334	0.574237	60/7273	2	1.21	Negative correlation
hsa04921	ytocin signaling path	3/147	154/8521	0.49859	0.720334	0.574237	817/60/5579	3	1.13	Negative correlation
hsa05207	rcinogenesis - recep	4/147	212/8521	0.499818	0.720334	0.574237	7184/6776/5579/4790	4	1.09	Negative correlation
hsa05215	Prostate cancer	2/147	97/8521	0.501477	0.720334	0.574237	7184/4790	2	1.2	Negative correlation
hsa00260	erine and threonine m	1/147	40/8521	0.502268	0.720334	0.574237	669	1	1.45	Negative correlation
hsa04925	rone synthesis and s	2/147	98/8521	0.506944	0.720334	0.574237	817/5579	2	1.18	Negative correlation
hsa05231	ine metabolism in ca	2/147	98/8521	0.506944	0.720334	0.574237	5579/7454	2	1.18	Negative correlation
hsa03440	nologous recombina	1/147	41/8521	0.510895	0.720334	0.574237	6118	1	1.41	Negative correlation
hsa04216	Ferroptosis	1/147	41/8521	0.510895	0.720334	0.574237	6520	1	1.41	Negative correlation
hsa05219	Bladder cancer	1/147	41/8521	0.510895	0.720334	0.574237	1890	1	1.41	Negative correlation
hsa00564	rophospholipid meta	2/147	99/8521	0.512372	0.720334	0.574237	10554/23646	2	1.17	Negative correlation
hsa00380	ryptophan metabolis	1/147	42/8521	0.519374	0.724838	0.577827	3620	1	1.38	Negative correlation
hsa04916	Melanogenesis	2/147	101/8521	0.52311	0.724838	0.577827	817/5579	2	1.15	Negative correlation
hsa00860	Porphyrin metabolism	1/147	43/8521	0.527706	0.724838	0.577827	644	1	1.35	Negative correlation
hsa04975	digestion and absorp	1/147	43/8521	0.527706	0.724838	0.577827	10554	1	1.35	Negative correlation
hsa04974	n digestion and abso	2/147	103/8521	0.53369	0.727722	0.580126	80781/6520	2	1.13	Negative correlation
hsa04962	in-regulated water re	1/147	44/8521	0.535895	0.727722	0.580126	397	1	1.32	Negative correlation
hsa04928	none synthesis, sec	2/147	106/8521	0.54926	0.741656	0.591234	5579/4209	2	1.09	Negative correlation
hsa04931	Insulin resistance	2/147	108/8521	0.559437	0.74721	0.595662	5579/4790	2	1.07	Negative correlation
hsa04973	lrate digestion and a	1/147	47/8521	0.559626	0.74721	0.595662	5579	1	1.23	Negative correlation
hsa04310	Wnt signaling pathwa	3/147	171/8521	0.569573	0.754784	0.6017	817/7090/5579	3	1.02	Negative correlation
hsa00520	and nucleotide suga	1/147	49/8521	0.574773	0.754784	0.6017	55577	1	1.18	Negative correlation
hsa05030	Cocaine addiction	1/147	49/8521	0.574773	0.754784	0.6017	4790	1	1.18	Negative correlation
hsa00565	Ether lipid metabolism	1/147	50/8521	0.582151	0.756164	0.602799	23646	1	1.16	Negative correlation
hsa05110	brio cholerae infecti	1/147	50/8521	0.582151	0.756164	0.602799	60	1	1.16	Negative correlation
hsa04979	holesterol metabolis	1/147	51/8521	0.589402	0.761444	0.607009	336	1	1.14	Negative correlation
hsa04724	lutamatergic synaps	2/147	115/8521	0.593769	0.762961	0.608218	59345/5579	2	1.01	Negative correlation
hsa00510	I-Glycan biosynthesi	1/147	53/8521	0.603531	0.771358	0.614912	4248	1	1.09	Negative correlation
hsa03460	nconi anemia pathw	1/147	55/8521	0.617178	0.784604	0.625471	6118	1	1.05	Negative correlation
hsa05206	MicroRNAs in cance	5/147	310/8521	0.624249	0.789394	0.62929	4082/5579/4790/960/836	5	0.93	Negative correlation
hsa04814	Motor proteins	3/147	193/8521	0.651775	0.815572	0.650158	60/4650/64005	3	0.9	Negative correlation
hsa05202	tional misregulation	3/147	193/8521	0.651775	0.815572	0.650158	4790/2209/929	3	0.9	Negative correlation
hsa00561	ycerolipid metabolis	1/147	63/8521	0.667253	0.830591	0.662131	10554	1	0.92	Negative correlation
hsa04929	GnRH secretion	1/147	64/8521	0.673036	0.833448	0.664409	5579	1	0.91	Negative correlation
hsa04915	rogen signaling path	2/147	137/8521	0.688594	0.845439	0.673968	7184/3858	2	0.85	Negative correlation
hsa00010	olysis / Gluconeoge	1/147	67/8521	0.689793	0.845439	0.673968	669	1	0.87	Negative correlation
hsa04371	oelin signaling pathw	2/147	139/8521	0.696253	0.849002	0.676808	59345/4209	2	0.83	Negative correlation
hsa04920	cytokine signaling pa	1/147	69/8521	0.700488	0.84983	0.677469	4790	1	0.84	Negative correlation
hsa05230	carbon metabolism i	1/147	70/8521	0.705697	0.851826	0.67906	57103	1	0.83	Negative correlation
hsa01524	atinum drug resistan	1/147	73/8521	0.720791	0.859957	0.685541	836	1	0.79	Negative correlation
hsa04115	53 signaling pathwa	1/147	74/8521	0.725649	0.859957	0.685541	836	1	0.78	Negative correlation
hsa04072	olipase D signaling p	2/147	148/8521	0.728818	0.859957	0.685541	10554/6850	2	0.78	Negative correlation
hsa01230	synthesis of amino a	1/147	75/8521	0.730424	0.859957	0.685541	6888	1	0.77	Negative correlation
hsa03320	PAR signaling pathw	1/147	75/8521	0.730424	0.859957	0.685541	336	1	0.77	Negative correlation
hsa05412	ic right ventricular ca	1/147	77/8521	0.739726	0.864626	0.689264	60	1	0.75	Negative correlation
hsa01240	osynthesis of cofact	2/147	153/8521	0.74561	0.864626	0.689264	3620/51004	2	0.76	Negative correlation
hsa01521	sine kinase inhibitor	1/147	79/8521	0.74871	0.864626	0.689264	5579	1	0.73	Negative correlation
hsa04261	c signaling in cardio	2/147	154/8521	0.74886	0.864626	0.689264	817/1432	2	0.75	Negative correlation
hsa04150	TOR signaling pathw	2/147	156/8521	0.755255	0.86726	0.691364	5579/6520	2	0.74	Negative correlation
hsa04390	ppo signaling pathw	2/147	157/8521	0.758399	0.86726	0.691364	60/7532	2	0.74	Negative correlation
hsa05210	Colorectal cancer	1/147	86/8521	0.777795	0.879026	0.700743	836	1	0.67	Negative correlation
hsa04014	Ras signaling pathwa	3/147	236/8521	0.778792	0.879026	0.700743	59345/5579/4790	3	0.74	Negative correlation
hsa04540	Gap junction	1/147	88/8521	0.785473	0.879026	0.700743	5579	1	0.66	Negative correlation
hsa03010	Ribosome	2/147	167/8521	0.787979	0.879026	0.700743	6173/6230	2	0.69	Negative correlation
hsa04211	gevity regulating path	1/147	89/8521	0.789213	0.879026	0.700743	4790	1	0.65	Negative correlation
hsa05225	patocellular carcino	2/147	168/8521	0.790756	0.879026	0.700743	60/5579	2	0.69	Negative correlation
hsa04970	Salivary secretion	1/147	93/8521	0.803534	0.881634	0.702822	5579	1	0.62	Negative correlation
hsa05323	Rheumatoid arthritis	1/147	93/8521	0.803534	0.881634	0.702822	3683	1	0.62	Negative correlation
hsa04141	cessing in endoplasm	2/147	173/8521	0.804168	0.881634	0.702822	7184/54431	2	0.67	Negative correlation
hsa01522	Endocrine resistance	1/147	98/8521	0.820084	0.894978	0.71346	1432	1	0.59	Negative correlation
hsa04914	ne-mediated oocyte	1/147	102/8521	0.832321	0.900112	0.717552	1432	1	0.57	Negative correlation
hsa04972	Pancreatic secretion	1/147	102/8521	0.832321	0.900112	0.717552	5579	1	0.57	Negative correlation
hsa04922	cagon signaling path	1/147	107/8521	0.846461	0.91128	0.726455	817	1	0.54	Negative correlation
hsa01200	Carbon metabolism	1/147	115/8521	0.866661	0.928843	0.740456	6888	1	0.5	Negative correlation
hsa00230	Purine metabolism	1/147	128/8521	0.894006	0.95052	0.757736	1633	1	0.45	Negative correlation
hsa04068	oxO signaling pathwa	1/147	131/8521	0.899479	0.95052	0.757736	1432	1	0.44	Negative correlation
hsa04024	AMP signaling pathw	2/147	225/8521	0.904372	0.95052	0.757736	817/4790	2	0.52	Negative correlation
hsa04270	r smooth muscle co	1/147	134/8521	0.904672	0.95052	0.757736	5579	1	0.43	Negative correlation
hsa04910	sulin signaling pathw	1/147	137/8521	0.909597	0.95052	0.757736	867	1	0.42	Negative correlation
hsa04140	Autophagy - animal	1/147	141/8521	0.915774	0.95052	0.757736	51100	1	0.41	Negative correlation
hsa04120	uitin mediated prote	1/147	142/8521	0.917251	0.95052	0.757736	867	1	0.41	Negative correlation
hsa04550	s regulating pluripot	1/147	143/8521	0.918703	0.95052	0.757736	1432	1	0.41	Negative correlation
hsa04934	Cushing syndrome	1/147	155/8521	0.93427	0.956572	0.762561	817	1	0.37	Negative correlation
hsa04020	lcium signaling pathw	2/147	253/8521	0.936117	0.956572	0.762561	817/5579	2	0.46	Negative correlation
hsa04110	Cell cycle	1/147	157/8521	0.93656	0.956572	0.762561	7532	1	0.37	Negative correlation
hsa04022	P-PKG signaling pat	1/147	167/8521	0.946873	0.96299	0.767678	4209	1	0.35	Negative correlation
hsa04360	Axon guidance	1/147	182/8521	0.959303	0.971497	0.774459	817	1	0.32	Negative correlation
hsa03040	Spliceosome	1/147	216/8521	0.977797	0.986048	0.786059	10915	1	0.27	Negative correlation
hsa04060	-cytokine receptor in	1/147	297/8521	0.994811	0.99899	0.796376	3603	1	0.2	Negative correlation
hsa04740	Olfactory transductio	1/147	439/8521	0.999608	0.999608	0.796869	817	1	0.13	Negative correlation

Supplementary Table 19. Cell Counting Kit-8 results

Confirmation of cisplatinum resistance

Cisplatinum dose (μg/ml)	SKOV3			SKOV3/DDP		
0.2	119.8885	95.61426684	97.15945771	106.9463667	97.65912678	92.7077107
0.8	92.658	76.90885073	83.69916075	96.5612	98.36927933	101.3448894
1	85.96656667	66.15587847	80.89089735	82.8748	93.74013677	91.96054991
2	43.58733333	52.25891678	58.71530019	71.9395	86.34928985	86.96951584
4	33.92193333	37.04095112	35.34538412	59.2847	84.69226723	69.57561267
16	34.08923333	23.93659181	39.99354422	53.78266667	52.68279853	51.91273162
32	27.881	21.05680317	22.78889606	47.31773333	42.37243556	40.16736402
64	21.28253333	18.30911493	19.52872821	45.32326667	25.74960547	27.4656306

Inhibitor Validation-SKOV3 vs. SKOV3/DDP

Cisplatinum dose (μg/ml)	SKOV3			SKOV3+EIPA			SKOV3/DDP			SKOV3/DDP+EIPA		
0	100	100	100	100	100	100	100	100	100	100	100	100
0.5	95.18486672	98.31144465	97.85025945	94.42508711	101.4104372	91.37440758	94.89414695	110.0183486	98.50107066	91.04683196	103.0285381	97.32525864
1	95.52880482	95.04690432	90.98097356	89.06459394	93.44146685	83.90995261	91.87422167	103.4862385	96.38650964	90.90909091	94.05940594	92.4552107
2	85.03869304	87.99249531	79.51569064	76.60144733	93.47672779	74.88151659	89.57036115	100.6238532	91.40792291	79.17355372	89.92428655	79.20767096
4	73.6027515	84.27767355	66.98789227	60.19833825	79.90126939	60.82938389	82.68991283	108.5504587	80.43361884	72.89256198	79.84857309	72.59651779
8	58.89939811	75.00938086	55.49789968	47.27954972	63.68124118	51.70616114	73.44333748	72.18348624	68.65631692	53.99449036	58.67792662	59.34897805
16	34.7090857	36.84803002	28.76204596	28.81265076	33.18053597	34.1943128	38.54296389	42.89908257	45.15524625	35.81267218	35.06115317	40.62578854
32	34.04987102	39.2120075	22.16456635	31.94853927	31.17066291	20.16587678	44.17808219	40.22018349	35.70663812	31.98347107	30.63482819	29.57355539
64	29.97993694	21.23827392	13.31850754	14.44652908	17.348378	8.009478673	31.22665006	27.81651376	22.77837259	16.63911846	16.13278975	11.58213475

Inhibitor Validation-SKOV3-Ctrl vs. SKOV3-OE

Cisplatinum dose (μg/ml)	SKOV3-Ctrl			SKOV3-Ctrl+EIPA			SKOV3-OE			SKOV3-OE+EIPA		
0	100	100	100	100	100	100	100	100	100	100	100	100
0.5	91.68413697	96.82726204	106.4640411	83.28119569	87.63853367	91.84801382	96.79795069	102.0896528	95.74841883	94.6890088	91.88332276	83.55074769
1	85.98881901	99.23619271	98.71575342	79.63156065	87.95112248	78.48013817	97.79058598	96.25884732	79.86647927	79.94887816	95.4977806	76.16926503
2	88.50454228	87.69095182	82.83390411	66.84045881	73.71412333	65.45768566	86.71149536	90.05729693	80.32326072	72.84862255	84.36905517	71.9376392
4	68.34381551	79.23031727	53.38184932	57.28189086	67.14975845	31.29533679	76.40089657	84.59723627	71.82009838	56.23402442	72.35256817	61.97900095
8	45.07337526	72.62044653	32.74828767	39.86791797	47.88292128	28.56649396	59.62215818	68.92483991	50.56219255	37.91536495	56.37285986	48.32962138
16	31.44654088	43.65452409	26.66952055	20.82029892	28.84342143	20.93264249	33.07716939	44.96124031	36.15600843	23.00482817	37.92010146	28.69869551
32	24.24877708	22.94359577	16.99486301	18.42196733	22.64847968	8.773747841	27.76176753	31.58072127	28.25017569	21.41437092	27.96448954	23.38530067
64	17.40041929	25.79318449	12.20034247	12.99965242	19.92043194	6.804835924	21.67787384	25.51398719	17.53338018	14.39931838	21.30627774	9.704104359

Inhibitor Validation-SKOV3-shCtrl vs. SKOV3-sh1/sh2

Cisplatinum dose (μg/ml)	SKOV3-shCtrl			SKOV3-shCtrl+EIPA			SKOV3-sh1			SKOV3-sh1+EIPA			SKOV3-sh2			SKOV3-sh2+EIPA		
0	100	100	100	100	100	100	100	100	100	100	100	100	100	100	100	100	100	100
0.5	105.228505	98.00695982	111.4506498	98.63317325	99.45149126	88.50746269	90.87516961	88.87748118	91.66422931	106.1837456	83.74322634	96.99582754	99.31451613	96.75174014	101.9291339	93.36065574	84.81254782	97.36263736
1	98.25716499	92.66055046	104.2149631	81.19689693	87.7271169	84.68656716	82.08955224	66.80355921	89.23661889	85.64487633	79.57482284	94.60361613	94.75806452	80.80875041	103.3070866	89.18032787	82.28768171	93.94034537
2	79.70565453	84.37203417	94.48542325	75.72958995	72.02605417	82.50746269	82.15739484	53.7303217	81.25182802	74.07243816	53.31388078	89.98609179	95.80645161	47.63009612	96.92913386	83.93442623	67.13848508	87.566719
4	77.18822618	74.34356216	93.53705655	62.17214629	63.62701406	80.56716418	55.97014925	12.9705681	48.55220825	54.77031802	21.84243435	71.29346314	78.22580645	15.74411667	38.03149606	78.89344262	21.65263963	50.29827316
8	70.4879938	63.9038279	89.60309097	55.55966014	49.84573192	74.68656716	48.23609227	14.78439425	24.10061421	36.92579505	18.88286786	31.29346314	58.70967742	16.2081538	21.57480315	48.1147541	20.31369549	24.05023548
16	47.56003098	34.60930085	58.02599227	40.00738825	23.72300309	53.07462687	15.12890095	8.281998631	12.45978356	13.33922261	10.25427261	26.53685675	15.32258065	10.44083527	9.133858268	13.31967213	13.50420811	14.94505495
32	22.69558482	22.55615312	39.86652617	14.22238641	21.56324991	35.85074627	14.89145183	3.490759754	4.44574437	15.94522968	3.626511046	5.257301808	11.16935484	4.110043089	4.803149606	13.97540984	4.28462127	4.144427002
64	12.58714175	7.3710851	14.12012645	12.8186184	5.930750771	17.46268657	12.07598372	1.916495551	3.656039778	13.56007067	2.292621926	4.589707928	9.35483871	3.016241299	4.448818898	8.524590164	4.093343535	5.368916797

Inhibitor Validation-Caov3-Ctrl vs. Caov3-OE

Cisplatinum dose (μg/ml)	Caov3-Ctrl			Caov3-Ctrl+EIPA			Caov3-OE			Caov3-OE+EIPA		
0	100	100	100	100	100	100	100	100	100	100	100	100
0.5	77.00033478	89.14523449	51.09717868	77.46368276	118.7800481	86.81238616	93.63894812	96.22149837	70.95871239	81.72278094	79.14296549	92.32585596
1	59.85939069	61.49773071	42.5477344	63.48645465	93.99038462	60.36429872	67.02203269	55.27687296	51.71448565	68.08045474	69.01782328	80.51948052
2	45.89889521	27.15582451	16.07295526	59.20691009	22.41586538	19.74499089	50.42643923	59.70684039	35.68929321	49.62833406	21.12248768	22.19598583
4	15.66789421	15.05295008	9.404388715	39.41892422	10.78725962	11.62112933	25.90618337	25.50488599	22.2183345	19.02055094	13.3105802	13.97087761
8	11.24874456	13.01059002	7.466514677	20.88731841	9.224759615	7.504553734	12.65103056	15.99348534	13.3659902	11.7184084	9.290860827	9.838646202
16	8.804820891	7.602118003	4.531205472	9.893992933	7.061298077	6.666666667	8.919687278	10.45602606	6.927921624	7.520769567	6.5604854	9.405745769
32	6.160026783	2.420574887	2.080364776	6.910090302	1.472355769	5.173041894	6.503198294	8.501628664	5.878236529	6.209007433	4.247250664	4.407713499
64	4.251757616	1.02118003	0.797948133	6.164114645	0.360576923	0.837887067	4.051172708	3.615635179	2.834149755	4.591167468	1.403109594	2.439984258

Inhibitor Validation-OVCAR3-shCtrl vs. OVCAR3-sh1/sh2

Cisplatinum dose (μg/ml)	OVCAR3-shCtrl			OVCAR3-shCtrl+EIPA			OVCAR3-sh1			OVCAR3-sh1+EIPA			OVCAR3-sh2			OVCAR3-sh2+EIPA		
0	100	100	100	100	100	100	100	100	100	100	100	100	100	100	100	100	100	100
0.5	86.24907339	69.40222898	63.34690855	64.64174455	54.45783133	37.62597984	68.79334258	60.96965469	47.84565916	54.00895401	60.4743083	43.35166613	84.06956522	65.83278473	54.48789572	73.49528644	52.61669024	51.27967931
1	65.08524833	60.28368794	39.54091077	33.1152648	42.58175559	27.36095558	48.23162275	51.16846878	31.12540193	44.4037444	50.88932806	28.04917502	68.59130435	56.64911126	38.88268156	58.15808557	47.24186704	32.46993525
2	39.73313566	39.20972644	22.62125139	21.37071651	24.75043029	15.75214632	19.59084605	10.08022323	6.52733119	19.57671958	9.486166008	8.21740537	21.63478261	9.644502962	7.039106145	21.10224801	9.264497878	7.893925378
4	19.57005189	21.54677474	11.88448723	12.52336449	14.11359725	8.622620381	11.71983356	6.487617719	4.019292605	11.5995116	7.872200264	3.849886768	16.06956522	6.188281764	4.618249534	13.63306744	7.001414427	5.180388529
8	12.04595997	11.04356636	7.626804887	8.878504673	8.846815835	5.300485256	8.079056865	4.220439484	1.479099678	9.238909239	4.644268775	1.682303462	10.46956522	3.587886768	3.054003724	11.09499637	3.429985856	3.206907185
16	8.450704225	8.409321175	5.072195483	6.074766355	6.127366609	3.50877193	5.92926491	2.929891873	1.061093248	7.163207163	2.635046113	0.9382077	7.756521739	2.304147465	2.830540037	8.448150834	1.909476662	1.603453592
32	6.449221646	6.011482607	3.702332469	3.894080997	4.199655766	1.978350131	8.044382802	3.174049529	2.090032154	7.488807489	3.12911726	0.87350372	10.22608696	2.567478604	4.283054004	7.469180566	2.227722772	2.096823928
64	5.782060786	3.411009794	4.627915587	2.897196262	2.822719449	1.829040687	7.073509015	2.16253924	2.443729904	5.168905169	1.910408432	0.194111938	9.32173913	2.633311389	4.554934823	5.438723713	1.732673267	1.665124884

Supplementary Table 20. Quantitative Statistics of Fluorescent tracer results

SKOV3 vs. SKOV3/DDP

Probe	value	group
BODIPY-C16	7338317.667	SKOV3 + BODIPY-C16
BODIPY-C16	9217667.667	SKOV3 + BODIPY-C16
BODIPY-C16	9007480.667	SKOV3 + BODIPY-C16
BODIPY-C16	7764660.667	SKOV3 + BODIPY-C16
BODIPY-C16	7071767.667	SKOV3 + BODIPY-C16
BODIPY-C16	8717533	SKOV3 + BODIPY-C16
BODIPY-C16	9622593	SKOV3 + BODIPY-C16
BODIPY-C16	7686236	SKOV3 + BODIPY-C16
BODIPY-C16	9750376	SKOV3 + BODIPY-C16
BODIPY-C16	1226003.667	SKOV3 + BODIPY-C16 + EIPA
BODIPY-C16	3674788.667	SKOV3 + BODIPY-C16 + EIPA
BODIPY-C16	3176220.667	SKOV3 + BODIPY-C16 + EIPA
BODIPY-C16	5169621.667	SKOV3 + BODIPY-C16 + EIPA
BODIPY-C16	3884174.667	SKOV3 + BODIPY-C16 + EIPA
BODIPY-C16	1120197	SKOV3 + BODIPY-C16 + EIPA
BODIPY-C16	1230173	SKOV3 + BODIPY-C16 + EIPA
BODIPY-C16	1995343	SKOV3 + BODIPY-C16 + EIPA
BODIPY-C16	1836272	SKOV3 + BODIPY-C16 + EIPA
BODIPY-C16	1590578	SKOV3 + BODIPY-C16 + EIPA
BODIPY-C16	1190904	SKOV3 + BODIPY-C16 + EIPA
BODIPY-C16	1088191	SKOV3 + BODIPY-C16 + EIPA
BODIPY-C16	1267502	SKOV3 + BODIPY-C16 + EIPA
BODIPY-C16	11411493.67	SKOV3/DDP + BODIPY-C16
BODIPY-C16	10924735	SKOV3/DDP + BODIPY-C16
BODIPY-C16	9043078	SKOV3/DDP + BODIPY-C16
BODIPY-C16	11109988	SKOV3/DDP + BODIPY-C16
BODIPY-C16	10793944	SKOV3/DDP + BODIPY-C16
BODIPY-C16	10093148.5	SKOV3/DDP + BODIPY-C16
BODIPY-C16	9536543.5	SKOV3/DDP + BODIPY-C16
BODIPY-C16	9562484.5	SKOV3/DDP + BODIPY-C16
BODIPY-C16	9622360.5	SKOV3/DDP + BODIPY-C16
BODIPY-C16	2636239.667	SKOV3/DDP + BODIPY-C16 + EIPA
BODIPY-C16	2693874.5	SKOV3/DDP + BODIPY-C16 + EIPA
BODIPY-C16	9136303.5	SKOV3/DDP + BODIPY-C16 + EIPA
BODIPY-C16	7968651.5	SKOV3/DDP + BODIPY-C16 + EIPA
BODIPY-C16	5520187.5	SKOV3/DDP + BODIPY-C16 + EIPA
BODIPY-C16	1407497	SKOV3/DDP + BODIPY-C16 + EIPA
BODIPY-C16	1549657	SKOV3/DDP + BODIPY-C16 + EIPA
BODIPY-C16	4014015	SKOV3/DDP + BODIPY-C16 + EIPA
BODIPY-C16	1921304	SKOV3/DDP + BODIPY-C16 + EIPA
BODIPY-C16	1495624	SKOV3/DDP + BODIPY-C16 + EIPA
BODIPY-C16	4347565	SKOV3/DDP + BODIPY-C16 + EIPA
BODIPY-C16	1077250	SKOV3/DDP + BODIPY-C16 + EIPA
BODIPY-C16	3521384	SKOV3/DDP + BODIPY-C16 + EIPA
TMR-Dextran	71163.33333	SKOV3 + TMR-Dextran
TMR-Dextran	83002.33333	SKOV3 + TMR-Dextran
TMR-Dextran	64670.33333	SKOV3 + TMR-Dextran
TMR-Dextran	73775.33333	SKOV3 + TMR-Dextran
TMR-Dextran	51475.33333	SKOV3 + TMR-Dextran
TMR-Dextran	40658.33333	SKOV3 + TMR-Dextran
TMR-Dextran	53425.33333	SKOV3 + TMR-Dextran
TMR-Dextran	70426.33333	SKOV3 + TMR-Dextran
TMR-Dextran	22703.33333	SKOV3 + TMR-Dextran
TMR-Dextran	44091.33333	SKOV3 + TMR-Dextran
TMR-Dextran	26920.33333	SKOV3 + TMR-Dextran
TMR-Dextran	56895.33333	SKOV3 + TMR-Dextran
TMR-Dextran	39415.33333	SKOV3 + TMR-Dextran + EIPA
TMR-Dextran	46965.33333	SKOV3 + TMR-Dextran + EIPA
TMR-Dextran	71459.33333	SKOV3 + TMR-Dextran + EIPA
TMR-Dextran	57029.33333	SKOV3 + TMR-Dextran + EIPA
TMR-Dextran	5520.333333	SKOV3 + TMR-Dextran + EIPA
TMR-Dextran	9073.333333	SKOV3 + TMR-Dextran + EIPA
TMR-Dextran	45227.33333	SKOV3 + TMR-Dextran + EIPA
TMR-Dextran	40217.33333	SKOV3 + TMR-Dextran + EIPA
TMR-Dextran	53510.33333	SKOV3 + TMR-Dextran + EIPA
TMR-Dextran	37212.33333	SKOV3 + TMR-Dextran + EIPA
TMR-Dextran	34068.33333	SKOV3 + TMR-Dextran + EIPA
TMR-Dextran	37952.33333	SKOV3 + TMR-Dextran + EIPA
TMR-Dextran	174080	SKOV3/DDP + TMR-Dextran
TMR-Dextran	149882	SKOV3/DDP + TMR-Dextran
TMR-Dextran	239755	SKOV3/DDP + TMR-Dextran
TMR-Dextran	171668	SKOV3/DDP + TMR-Dextran
TMR-Dextran	251476	SKOV3/DDP + TMR-Dextran
TMR-Dextran	120941.5	SKOV3/DDP + TMR-Dextran
TMR-Dextran	123053.5	SKOV3/DDP + TMR-Dextran
TMR-Dextran	125328.5	SKOV3/DDP + TMR-Dextran
TMR-Dextran	126263.5	SKOV3/DDP + TMR-Dextran
TMR-Dextran	69279	SKOV3/DDP + TMR-Dextran + EIPA
TMR-Dextran	75536	SKOV3/DDP + TMR-Dextran + EIPA
TMR-Dextran	59152	SKOV3/DDP + TMR-Dextran + EIPA
TMR-Dextran	23772	SKOV3/DDP + TMR-Dextran + EIPA
TMR-Dextran	64280.5	SKOV3/DDP + TMR-Dextran + EIPA
TMR-Dextran	76733.5	SKOV3/DDP + TMR-Dextran + EIPA
TMR-Dextran	93532.5	SKOV3/DDP + TMR-Dextran + EIPA
TMR-Dextran	95721.5	SKOV3/DDP + TMR-Dextran + EIPA
TMR-Dextran	58760.5	SKOV3/DDP + TMR-Dextran + EIPA
TMR-Dextran	82607.5	SKOV3/DDP + TMR-Dextran + EIPA
TMR-Dextran	86084.5	SKOV3/DDP + TMR-Dextran + EIPA
TMR-Dextran	106257.5	SKOV3/DDP + TMR-Dextran + EIPA

SKOV3-Ctrl vs. SKOV3-OE

Probe	value	group
BODIPY-C16	3669266.333	SKOV3-Ctrl + BODIPY-C16
BODIPY-C16	1971060.667	SKOV3-Ctrl + BODIPY-C16
BODIPY-C16	2885233.667	SKOV3-Ctrl + BODIPY-C16
BODIPY-C16	2671296.667	SKOV3-Ctrl + BODIPY-C16
BODIPY-C16	2088646.333	SKOV3-Ctrl + BODIPY-C16
BODIPY-C16	3330562.333	SKOV3-Ctrl + BODIPY-C16
BODIPY-C16	1872955.667	SKOV3-Ctrl + BODIPY-C16
BODIPY-C16	2405556.333	SKOV3-Ctrl + BODIPY-C16
BODIPY-C16	3299571.667	SKOV3-Ctrl + BODIPY-C16
BODIPY-C16	3182752.667	SKOV3-Ctrl + BODIPY-C16
BODIPY-C16	2781448.667	SKOV3-Ctrl + BODIPY-C16
BODIPY-C16	2639299.667	SKOV3-Ctrl + BODIPY-C16
BODIPY-C16	1632716.667	SKOV3-Ctrl + BODIPY-C16 + EIPA
BODIPY-C16	2337752.667	SKOV3-Ctrl + BODIPY-C16 + EIPA
BODIPY-C16	1825213.667	SKOV3-Ctrl + BODIPY-C16 + EIPA
BODIPY-C16	1728772.667	SKOV3-Ctrl + BODIPY-C16 + EIPA
BODIPY-C16	2765990.333	SKOV3-Ctrl + BODIPY-C16 + EIPA
BODIPY-C16	3012397.333	SKOV3-Ctrl + BODIPY-C16 + EIPA
BODIPY-C16	2627346.333	SKOV3-Ctrl + BODIPY-C16 + EIPA
BODIPY-C16	1791819.667	SKOV3-Ctrl + BODIPY-C16 + EIPA
BODIPY-C16	2069224.667	SKOV3-Ctrl + BODIPY-C16 + EIPA
BODIPY-C16	1662501.667	SKOV3-Ctrl + BODIPY-C16 + EIPA
BODIPY-C16	2183163.667	SKOV3-Ctrl + BODIPY-C16 + EIPA
BODIPY-C16	1830451.667	SKOV3-Ctrl + BODIPY-C16 + EIPA
BODIPY-C16	2896271	SKOV3-OE + BODIPY-C16
BODIPY-C16	3990273	SKOV3-OE + BODIPY-C16
BODIPY-C16	2198147	SKOV3-OE + BODIPY-C16
BODIPY-C16	3561250	SKOV3-OE + BODIPY-C16
BODIPY-C16	3344336	SKOV3-OE + BODIPY-C16
BODIPY-C16	4209026	SKOV3-OE + BODIPY-C16
BODIPY-C16	4367834	SKOV3-OE + BODIPY-C16
BODIPY-C16	3897591	SKOV3-OE + BODIPY-C16
BODIPY-C16	3352145	SKOV3-OE + BODIPY-C16
BODIPY-C16	4367857	SKOV3-OE + BODIPY-C16
BODIPY-C16	4072041	SKOV3-OE + BODIPY-C16
BODIPY-C16	2566691	SKOV3-OE + BODIPY-C16
BODIPY-C16	1766394	SKOV3-OE + BODIPY-C16 + EIPA
BODIPY-C16	2832115	SKOV3-OE + BODIPY-C16 + EIPA
BODIPY-C16	1816717	SKOV3-OE + BODIPY-C16 + EIPA
BODIPY-C16	3415347	SKOV3-OE + BODIPY-C16 + EIPA
BODIPY-C16	3487010	SKOV3-OE + BODIPY-C16 + EIPA
BODIPY-C16	3325655	SKOV3-OE + BODIPY-C16 + EIPA
BODIPY-C16	2476932	SKOV3-OE + BODIPY-C16 + EIPA
BODIPY-C16	2059953	SKOV3-OE + BODIPY-C16 + EIPA
BODIPY-C16	2438661	SKOV3-OE + BODIPY-C16 + EIPA
BODIPY-C16	2293879	SKOV3-OE + BODIPY-C16 + EIPA
BODIPY-C16	2841838	SKOV3-OE + BODIPY-C16 + EIPA
BODIPY-C16	2481812	SKOV3-OE + BODIPY-C16 + EIPA
TMR-Dextran	90536	SKOV3-Ctrl + TMR-Dextran
TMR-Dextran	92056	SKOV3-Ctrl + TMR-Dextran
TMR-Dextran	64084	SKOV3-Ctrl + TMR-Dextran
TMR-Dextran	67465	SKOV3-Ctrl + TMR-Dextran
TMR-Dextran	94767.66667	SKOV3-Ctrl + TMR-Dextran
TMR-Dextran	5616.666667	SKOV3-Ctrl + TMR-Dextran
TMR-Dextran	45065.66667	SKOV3-Ctrl + TMR-Dextran
TMR-Dextran	56584.66667	SKOV3-Ctrl + TMR-Dextran
TMR-Dextran	48549.66667	SKOV3-Ctrl + TMR-Dextran
TMR-Dextran	68611.66667	SKOV3-Ctrl + TMR-Dextran
TMR-Dextran	31157	SKOV3-Ctrl + TMR-Dextran
TMR-Dextran	84412.66667	SKOV3-Ctrl + TMR-Dextran
TMR-Dextran	12298.33333	SKOV3-Ctrl + TMR-Dextran + EIPA
TMR-Dextran	107505	SKOV3-Ctrl + TMR-Dextran + EIPA
TMR-Dextran	144306	SKOV3-Ctrl + TMR-Dextran + EIPA
TMR-Dextran	58640.33333	SKOV3-Ctrl + TMR-Dextran + EIPA
TMR-Dextran	70889.33333	SKOV3-Ctrl + TMR-Dextran + EIPA
TMR-Dextran	143271.6667	SKOV3-Ctrl + TMR-Dextran + EIPA
TMR-Dextran	95557	SKOV3-Ctrl + TMR-Dextran + EIPA
TMR-Dextran	44938.66667	SKOV3-Ctrl + TMR-Dextran + EIPA
TMR-Dextran	102473.6667	SKOV3-Ctrl + TMR-Dextran + EIPA
TMR-Dextran	15167.66667	SKOV3-Ctrl + TMR-Dextran + EIPA
TMR-Dextran	89860.66667	SKOV3-Ctrl + TMR-Dextran + EIPA
TMR-Dextran	58698.66667	SKOV3-Ctrl + TMR-Dextran + EIPA
TMR-Dextran	130491.6667	SKOV3-OE + TMR-Dextran
TMR-Dextran	106274.3333	SKOV3-OE + TMR-Dextran
TMR-Dextran	97212.33333	SKOV3-OE + TMR-Dextran
TMR-Dextran	182123.6667	SKOV3-OE + TMR-Dextran
TMR-Dextran	158084.3333	SKOV3-OE + TMR-Dextran
TMR-Dextran	75258.33333	SKOV3-OE + TMR-Dextran
TMR-Dextran	91978.33333	SKOV3-OE + TMR-Dextran
TMR-Dextran	270639.6667	SKOV3-OE + TMR-Dextran
TMR-Dextran	153418.6667	SKOV3-OE + TMR-Dextran
TMR-Dextran	40679.33333	SKOV3-OE + TMR-Dextran
TMR-Dextran	136649.6667	SKOV3-OE + TMR-Dextran
TMR-Dextran	54346.66667	SKOV3-OE + TMR-Dextran
TMR-Dextran	48761.33333	SKOV3-OE + TMR-Dextran + EIPA
TMR-Dextran	69105.33333	SKOV3-OE + TMR-Dextran + EIPA
TMR-Dextran	39080.33333	SKOV3-OE + TMR-Dextran + EIPA
TMR-Dextran	37118.33333	SKOV3-OE + TMR-Dextran + EIPA
TMR-Dextran	80358.33333	SKOV3-OE + TMR-Dextran + EIPA
TMR-Dextran	62800.33333	SKOV3-OE + TMR-Dextran + EIPA
TMR-Dextran	73167.33333	SKOV3-OE + TMR-Dextran + EIPA
TMR-Dextran	81165.33333	SKOV3-OE + TMR-Dextran + EIPA
TMR-Dextran	70721.33333	SKOV3-OE + TMR-Dextran + EIPA
TMR-Dextran	30182.30556	SKOV3-OE + TMR-Dextran + EIPA
TMR-Dextran	13756.30556	SKOV3-OE + TMR-Dextran + EIPA
TMR-Dextran	3325.305556	SKOV3-OE + TMR-Dextran + EIPA

Caov3-Ctrl vs. Caov3-OE

Probe	value	group
BODIPY-C16	1959270	Caov3-Ctrl + BODIPY-C16
BODIPY-C16	2089785	Caov3-Ctrl + BODIPY-C16
BODIPY-C16	4448674.333	Caov3-Ctrl + BODIPY-C16
BODIPY-C16	2301627	Caov3-Ctrl + BODIPY-C16
BODIPY-C16	2964635.333	Caov3-Ctrl + BODIPY-C16
BODIPY-C16	3655246.333	Caov3-Ctrl + BODIPY-C16
BODIPY-C16	2896172.333	Caov3-Ctrl + BODIPY-C16
BODIPY-C16	2888808.333	Caov3-Ctrl + BODIPY-C16
BODIPY-C16	2370470.333	Caov3-Ctrl + BODIPY-C16
BODIPY-C16	4032770.333	Caov3-Ctrl + BODIPY-C16
BODIPY-C16	1595386	Caov3-Ctrl + BODIPY-C16
BODIPY-C16	3350413.333	Caov3-Ctrl + BODIPY-C16
BODIPY-C16	1857046	Caov3-Ctrl + BODIPY-C16 + EIPA
BODIPY-C16	3519440.333	Caov3-Ctrl + BODIPY-C16 + EIPA
BODIPY-C16	1958898	Caov3-Ctrl + BODIPY-C16 + EIPA
BODIPY-C16	2122044	Caov3-Ctrl + BODIPY-C16 + EIPA
BODIPY-C16	3282681.333	Caov3-Ctrl + BODIPY-C16 + EIPA
BODIPY-C16	1397616	Caov3-Ctrl + BODIPY-C16 + EIPA
BODIPY-C16	2847382.333	Caov3-Ctrl + BODIPY-C16 + EIPA
BODIPY-C16	3312204.333	Caov3-Ctrl + BODIPY-C16 + EIPA
BODIPY-C16	2809864.333	Caov3-Ctrl + BODIPY-C16 + EIPA
BODIPY-C16	2504285.333	Caov3-Ctrl + BODIPY-C16 + EIPA
BODIPY-C16	3001606.333	Caov3-Ctrl + BODIPY-C16 + EIPA
BODIPY-C16	2960825.333	Caov3-Ctrl + BODIPY-C16 + EIPA
BODIPY-C16	3161033.333	Caov3-OE + BODIPY-C16
BODIPY-C16	5356212.333	Caov3-OE + BODIPY-C16
BODIPY-C16	3207505.333	Caov3-OE + BODIPY-C16
BODIPY-C16	5475700.333	Caov3-OE + BODIPY-C16
BODIPY-C16	5498137.333	Caov3-OE + BODIPY-C16
BODIPY-C16	3651320.333	Caov3-OE + BODIPY-C16
BODIPY-C16	3628123.333	Caov3-OE + BODIPY-C16
BODIPY-C16	3673153.333	Caov3-OE + BODIPY-C16
BODIPY-C16	6756998.333	Caov3-OE + BODIPY-C16
BODIPY-C16	4791291.333	Caov3-OE + BODIPY-C16
BODIPY-C16	3389010.333	Caov3-OE + BODIPY-C16
BODIPY-C16	3405035.333	Caov3-OE + BODIPY-C16
BODIPY-C16	3094842.333	Caov3-OE + BODIPY-C16 + EIPA
BODIPY-C16	2311083.333	Caov3-OE + BODIPY-C16 + EIPA
BODIPY-C16	2907346.333	Caov3-OE + BODIPY-C16 + EIPA
BODIPY-C16	2287598.333	Caov3-OE + BODIPY-C16 + EIPA
BODIPY-C16	3905999.333	Caov3-OE + BODIPY-C16 + EIPA
BODIPY-C16	3299754.333	Caov3-OE + BODIPY-C16 + EIPA
BODIPY-C16	3136710.333	Caov3-OE + BODIPY-C16 + EIPA
BODIPY-C16	3106929.333	Caov3-OE + BODIPY-C16 + EIPA
BODIPY-C16	2969022.333	Caov3-OE + BODIPY-C16 + EIPA
BODIPY-C16	2953450.333	Caov3-OE + BODIPY-C16 + EIPA
BODIPY-C16	2767988.333	Caov3-OE + BODIPY-C16 + EIPA
BODIPY-C16	2318221.333	Caov3-OE + BODIPY-C16 + EIPA
TMR-Dextran	121917.6667	Caov3-Ctrl + TMR-Dextran
TMR-Dextran	129533.3333	Caov3-Ctrl + TMR-Dextran
TMR-Dextran	58393.66667	Caov3-Ctrl + TMR-Dextran
TMR-Dextran	70987.66667	Caov3-Ctrl + TMR-Dextran
TMR-Dextran	84935.66667	Caov3-Ctrl + TMR-Dextran
TMR-Dextran	34268.33333	Caov3-Ctrl + TMR-Dextran
TMR-Dextran	41886.66667	Caov3-Ctrl + TMR-Dextran
TMR-Dextran	87379.66667	Caov3-Ctrl + TMR-Dextran
TMR-Dextran	48338.33333	Caov3-Ctrl + TMR-Dextran
TMR-Dextran	45442.33333	Caov3-Ctrl + TMR-Dextran + EIPA
TMR-Dextran	52300.66667	Caov3-Ctrl + TMR-Dextran + EIPA
TMR-Dextran	67866.66667	Caov3-Ctrl + TMR-Dextran + EIPA
TMR-Dextran	117489.6667	Caov3-Ctrl + TMR-Dextran + EIPA
TMR-Dextran	127574.6667	Caov3-Ctrl + TMR-Dextran + EIPA
TMR-Dextran	100946.6667	Caov3-Ctrl + TMR-Dextran + EIPA
TMR-Dextran	107462.3333	Caov3-Ctrl + TMR-Dextran + EIPA
TMR-Dextran	145125.6667	Caov3-Ctrl + TMR-Dextran + EIPA
TMR-Dextran	53307.33333	Caov3-Ctrl + TMR-Dextran + EIPA
TMR-Dextran	161415.5	Caov3-OE + TMR-Dextran
TMR-Dextran	211386	Caov3-OE + TMR-Dextran
TMR-Dextran	172431.3333	Caov3-OE + TMR-Dextran
TMR-Dextran	204860.3333	Caov3-OE + TMR-Dextran
TMR-Dextran	198209.5	Caov3-OE + TMR-Dextran
TMR-Dextran	219146.5	Caov3-OE + TMR-Dextran
TMR-Dextran	249086.5	Caov3-OE + TMR-Dextran
TMR-Dextran	215264.5	Caov3-OE + TMR-Dextran
TMR-Dextran	167996.5	Caov3-OE + TMR-Dextran
TMR-Dextran	112965	Caov3-OE + TMR-Dextran + EIPA
TMR-Dextran	29563.5	Caov3-OE + TMR-Dextran + EIPA
TMR-Dextran	49728.33333	Caov3-OE + TMR-Dextran + EIPA
TMR-Dextran	94545.33333	Caov3-OE + TMR-Dextran + EIPA
TMR-Dextran	134359.3333	Caov3-OE + TMR-Dextran + EIPA
TMR-Dextran	108258.3333	Caov3-OE + TMR-Dextran + EIPA
TMR-Dextran	109305.3333	Caov3-OE + TMR-Dextran + EIPA
TMR-Dextran	33061.5	Caov3-OE + TMR-Dextran + EIPA
TMR-Dextran	48945.5	Caov3-OE + TMR-Dextran + EIPA

SKOV3-shCtrl vs. SKOV3-sh1/sh2

Probe	value	group
BODIPY-C16	2658142.333	SKOV3/DDP-sh1 + BODIPY-C16
BODIPY-C16	2007872.333	SKOV3/DDP-sh1 + BODIPY-C16
BODIPY-C16	3336714.333	SKOV3/DDP-sh1 + BODIPY-C16
BODIPY-C16	2548813.333	SKOV3/DDP-sh1 + BODIPY-C16
BODIPY-C16	1921309	SKOV3/DDP-sh1 + BODIPY-C16
BODIPY-C16	1804313	SKOV3/DDP-sh1 + BODIPY-C16
BODIPY-C16	1392177	SKOV3/DDP-sh1 + BODIPY-C16
BODIPY-C16	1962892	SKOV3/DDP-sh1 + BODIPY-C16
BODIPY-C16	3586826	SKOV3/DDP-sh1 + BODIPY-C16
BODIPY-C16	4261953	SKOV3/DDP-sh1 + BODIPY-C16
BODIPY-C16	3015778	SKOV3/DDP-sh1 + BODIPY-C16
BODIPY-C16	4979913	SKOV3/DDP-sh1 + BODIPY-C16
BODIPY-C16	5203471.667	SKOV3/DDP-sh1 + BODIPY-C16 + EIPA
BODIPY-C16	1618247.333	SKOV3/DDP-sh1 + BODIPY-C16 + EIPA
BODIPY-C16	1631954.333	SKOV3/DDP-sh1 + BODIPY-C16 + EIPA
BODIPY-C16	1945976.333	SKOV3/DDP-sh1 + BODIPY-C16 + EIPA
BODIPY-C16	1593400	SKOV3/DDP-sh1 + BODIPY-C16 + EIPA
BODIPY-C16	5641272.667	SKOV3/DDP-sh1 + BODIPY-C16 + EIPA
BODIPY-C16	1347454	SKOV3/DDP-sh1 + BODIPY-C16 + EIPA
BODIPY-C16	1203293	SKOV3/DDP-sh1 + BODIPY-C16 + EIPA
BODIPY-C16	1327890	SKOV3/DDP-sh1 + BODIPY-C16 + EIPA
BODIPY-C16	4521879.667	SKOV3/DDP-sh1 + BODIPY-C16 + EIPA
BODIPY-C16	1583149.333	SKOV3/DDP-sh1 + BODIPY-C16 + EIPA
BODIPY-C16	4436253.667	SKOV3/DDP-sh1 + BODIPY-C16 + EIPA
BODIPY-C16	2811662.667	SKOV3/DDP-sh2 + BODIPY-C16
BODIPY-C16	3015439.667	SKOV3/DDP-sh2 + BODIPY-C16
BODIPY-C16	2926814.667	SKOV3/DDP-sh2 + BODIPY-C16
BODIPY-C16	3071293.667	SKOV3/DDP-sh2 + BODIPY-C16
BODIPY-C16	6825679.667	SKOV3/DDP-sh2 + BODIPY-C16
BODIPY-C16	4397332.667	SKOV3/DDP-sh2 + BODIPY-C16
BODIPY-C16	5121787.667	SKOV3/DDP-sh2 + BODIPY-C16
BODIPY-C16	4176154.667	SKOV3/DDP-sh2 + BODIPY-C16
BODIPY-C16	2944363.333	SKOV3/DDP-sh2 + BODIPY-C16
BODIPY-C16	3490898.333	SKOV3/DDP-sh2 + BODIPY-C16
BODIPY-C16	3321623.333	SKOV3/DDP-sh2 + BODIPY-C16
BODIPY-C16	3837072.333	SKOV3/DDP-sh2 + BODIPY-C16
BODIPY-C16	1698443.667	SKOV3/DDP-sh2 + BODIPY-C16 + EIPA
BODIPY-C16	2899631.667	SKOV3/DDP-sh2 + BODIPY-C16 + EIPA
BODIPY-C16	2650603.667	SKOV3/DDP-sh2 + BODIPY-C16 + EIPA
BODIPY-C16	2681216.667	SKOV3/DDP-sh2 + BODIPY-C16 + EIPA
BODIPY-C16	4900724.667	SKOV3/DDP-sh2 + BODIPY-C16 + EIPA
BODIPY-C16	5231669.667	SKOV3/DDP-sh2 + BODIPY-C16 + EIPA
BODIPY-C16	4595176.667	SKOV3/DDP-sh2 + BODIPY-C16 + EIPA
BODIPY-C16	4649739.667	SKOV3/DDP-sh2 + BODIPY-C16 + EIPA
BODIPY-C16	3736998.333	SKOV3/DDP-sh2 + BODIPY-C16 + EIPA
BODIPY-C16	4203573.333	SKOV3/DDP-sh2 + BODIPY-C16 + EIPA
BODIPY-C16	4841585.333	SKOV3/DDP-sh2 + BODIPY-C16 + EIPA
BODIPY-C16	4837778.333	SKOV3/DDP-sh2 + BODIPY-C16 + EIPA
BODIPY-C16	22518343.33	SKOV3/DDP-shCtrl + BODIPY-C16
BODIPY-C16	17593943.33	SKOV3/DDP-shCtrl + BODIPY-C16
BODIPY-C16	4115572.333	SKOV3/DDP-shCtrl + BODIPY-C16
BODIPY-C16	9518755.333	SKOV3/DDP-shCtrl + BODIPY-C16
BODIPY-C16	20648450.33	SKOV3/DDP-shCtrl + BODIPY-C16
BODIPY-C16	16878257.33	SKOV3/DDP-shCtrl + BODIPY-C16
BODIPY-C16	18968692.33	SKOV3/DDP-shCtrl + BODIPY-C16
BODIPY-C16	3898677.333	SKOV3/DDP-shCtrl + BODIPY-C16
BODIPY-C16	16526670.33	SKOV3/DDP-shCtrl + BODIPY-C16
BODIPY-C16	2824942.333	SKOV3/DDP-shCtrl + BODIPY-C16
BODIPY-C16	13481094.33	SKOV3/DDP-shCtrl + BODIPY-C16
BODIPY-C16	15609748.33	SKOV3/DDP-shCtrl + BODIPY-C16
BODIPY-C16	8685341.333	SKOV3/DDP-shCtrl + BODIPY-C16 + EIPA
BODIPY-C16	5681741.333	SKOV3/DDP-shCtrl + BODIPY-C16 + EIPA
BODIPY-C16	2505297.333	SKOV3/DDP-shCtrl + BODIPY-C16 + EIPA
BODIPY-C16	3545303.333	SKOV3/DDP-shCtrl + BODIPY-C16 + EIPA
BODIPY-C16	1982915.333	SKOV3/DDP-shCtrl + BODIPY-C16 + EIPA
BODIPY-C16	7449131.333	SKOV3/DDP-shCtrl + BODIPY-C16 + EIPA
BODIPY-C16	6257018.333	SKOV3/DDP-shCtrl + BODIPY-C16 + EIPA
BODIPY-C16	6349093.333	SKOV3/DDP-shCtrl + BODIPY-C16 + EIPA
BODIPY-C16	7961441.333	SKOV3/DDP-shCtrl + BODIPY-C16 + EIPA
BODIPY-C16	6592879.333	SKOV3/DDP-shCtrl + BODIPY-C16 + EIPA
BODIPY-C16	2577183.333	SKOV3/DDP-shCtrl + BODIPY-C16 + EIPA
BODIPY-C16	6883680.333	SKOV3/DDP-shCtrl + BODIPY-C16 + EIPA
TMR-Dextran	63664.66667	SKOV3/DDP-sh1 + TMR-Dextran
TMR-Dextran	82858.66667	SKOV3/DDP-sh1 + TMR-Dextran
TMR-Dextran	52628.66667	SKOV3/DDP-sh1 + TMR-Dextran
TMR-Dextran	73597.66667	SKOV3/DDP-sh1 + TMR-Dextran
TMR-Dextran	96385.33333	SKOV3/DDP-sh1 + TMR-Dextran
TMR-Dextran	84363.33333	SKOV3/DDP-sh1 + TMR-Dextran
TMR-Dextran	84543.33333	SKOV3/DDP-sh1 + TMR-Dextran
TMR-Dextran	97520.33333	SKOV3/DDP-sh1 + TMR-Dextran
TMR-Dextran	153078.6667	SKOV3/DDP-sh1 + TMR-Dextran
TMR-Dextran	153490.6667	SKOV3/DDP-sh1 + TMR-Dextran
TMR-Dextran	173569.6667	SKOV3/DDP-sh1 + TMR-Dextran
TMR-Dextran	194716.6667	SKOV3/DDP-sh1 + TMR-Dextran
TMR-Dextran	79383.66667	SKOV3/DDP-sh1 + TMR-Dextran + EIPA
TMR-Dextran	81390.66667	SKOV3/DDP-sh1 + TMR-Dextran + EIPA
TMR-Dextran	80993.66667	SKOV3/DDP-sh1 + TMR-Dextran + EIPA
TMR-Dextran	141263.6667	SKOV3/DDP-sh1 + TMR-Dextran + EIPA
TMR-Dextran	164729.3333	SKOV3/DDP-sh1 + TMR-Dextran + EIPA
TMR-Dextran	168382.3333	SKOV3/DDP-sh1 + TMR-Dextran + EIPA
TMR-Dextran	148843.3333	SKOV3/DDP-sh1 + TMR-Dextran + EIPA
TMR-Dextran	139843.3333	SKOV3/DDP-sh1 + TMR-Dextran + EIPA
TMR-Dextran	168018.6667	SKOV3/DDP-sh1 + TMR-Dextran + EIPA
TMR-Dextran	137774.6667	SKOV3/DDP-sh1 + TMR-Dextran + EIPA
TMR-Dextran	162756.6667	SKOV3/DDP-sh1 + TMR-Dextran + EIPA
TMR-Dextran	200662.6667	SKOV3/DDP-sh1 + TMR-Dextran + EIPA
TMR-Dextran	98205.66667	SKOV3/DDP-sh2 + TMR-Dextran
TMR-Dextran	132700.6667	SKOV3/DDP-sh2 + TMR-Dextran
TMR-Dextran	84117.66667	SKOV3/DDP-sh2 + TMR-Dextran
TMR-Dextran	80163.66667	SKOV3/DDP-sh2 + TMR-Dextran
TMR-Dextran	93697	SKOV3/DDP-sh2 + TMR-Dextran
TMR-Dextran	87767	SKOV3/DDP-sh2 + TMR-Dextran
TMR-Dextran	102555	SKOV3/DDP-sh2 + TMR-Dextran
TMR-Dextran	185426	SKOV3/DDP-sh2 + TMR-Dextran
TMR-Dextran	110058	SKOV3/DDP-sh2 + TMR-Dextran
TMR-Dextran	91072	SKOV3/DDP-sh2 + TMR-Dextran
TMR-Dextran	91978	SKOV3/DDP-sh2 + TMR-Dextran
TMR-Dextran	86724	SKOV3/DDP-sh2 + TMR-Dextran
TMR-Dextran	85454.66667	SKOV3/DDP-sh2 + TMR-Dextran + EIPA
TMR-Dextran	76128.66667	SKOV3/DDP-sh2 + TMR-Dextran + EIPA
TMR-Dextran	72926.66667	SKOV3/DDP-sh2 + TMR-Dextran + EIPA
TMR-Dextran	75641.66667	SKOV3/DDP-sh2 + TMR-Dextran + EIPA
TMR-Dextran	96470	SKOV3/DDP-sh2 + TMR-Dextran + EIPA
TMR-Dextran	134254	SKOV3/DDP-sh2 + TMR-Dextran + EIPA
TMR-Dextran	81307	SKOV3/DDP-sh2 + TMR-Dextran + EIPA
TMR-Dextran	107410	SKOV3/DDP-sh2 + TMR-Dextran + EIPA
TMR-Dextran	74155	SKOV3/DDP-sh2 + TMR-Dextran + EIPA
TMR-Dextran	87733	SKOV3/DDP-sh2 + TMR-Dextran + EIPA
TMR-Dextran	93309	SKOV3/DDP-sh2 + TMR-Dextran + EIPA
TMR-Dextran	67440	SKOV3/DDP-sh2 + TMR-Dextran + EIPA
TMR-Dextran	1035605.333	SKOV3/DDP-shCtrl + TMR-Dextran
TMR-Dextran	553513.3333	SKOV3/DDP-shCtrl + TMR-Dextran
TMR-Dextran	153629	SKOV3/DDP-shCtrl + TMR-Dextran
TMR-Dextran	223768	SKOV3/DDP-shCtrl + TMR-Dextran
TMR-Dextran	428108.3333	SKOV3/DDP-shCtrl + TMR-Dextran
TMR-Dextran	422184.3333	SKOV3/DDP-shCtrl + TMR-Dextran
TMR-Dextran	956925.3333	SKOV3/DDP-shCtrl + TMR-Dextran
TMR-Dextran	458219.3333	SKOV3/DDP-shCtrl + TMR-Dextran
TMR-Dextran	264581	SKOV3/DDP-shCtrl + TMR-Dextran
TMR-Dextran	306490	SKOV3/DDP-shCtrl + TMR-Dextran
TMR-Dextran	907167.3333	SKOV3/DDP-shCtrl + TMR-Dextran
TMR-Dextran	910706.3333	SKOV3/DDP-shCtrl + TMR-Dextran
TMR-Dextran	257684.3333	SKOV3/DDP-shCtrl + TMR-Dextran + EIPA
TMR-Dextran	177096	SKOV3/DDP-shCtrl + TMR-Dextran + EIPA
TMR-Dextran	369872.3333	SKOV3/DDP-shCtrl + TMR-Dextran + EIPA
TMR-Dextran	180127	SKOV3/DDP-shCtrl + TMR-Dextran + EIPA
TMR-Dextran	253451.3333	SKOV3/DDP-shCtrl + TMR-Dextran + EIPA
TMR-Dextran	163772.3333	SKOV3/DDP-shCtrl + TMR-Dextran + EIPA
TMR-Dextran	258143.3333	SKOV3/DDP-shCtrl + TMR-Dextran + EIPA
TMR-Dextran	223823.3333	SKOV3/DDP-shCtrl + TMR-Dextran + EIPA
TMR-Dextran	197714	SKOV3/DDP-shCtrl + TMR-Dextran + EIPA
TMR-Dextran	346581.3333	SKOV3/DDP-shCtrl + TMR-Dextran + EIPA
TMR-Dextran	358225.3333	SKOV3/DDP-shCtrl + TMR-Dextran + EIPA
TMR-Dextran	133655	SKOV3/DDP-shCtrl + TMR-Dextran + EIPA

OVCAR3-shCtrl vs. OVCAR3-sh1/sh2

Probe	value	group
BODIPY-C16	5833058.333	OVCAR3-sh1 + BODIPY-C16
BODIPY-C16	6346285.333	OVCAR3-sh1 + BODIPY-C16
BODIPY-C16	5797518.333	OVCAR3-sh1 + BODIPY-C16
BODIPY-C16	6072174.333	OVCAR3-sh1 + BODIPY-C16
BODIPY-C16	6522809.667	OVCAR3-sh1 + BODIPY-C16
BODIPY-C16	6393299.667	OVCAR3-sh1 + BODIPY-C16
BODIPY-C16	6288810.667	OVCAR3-sh1 + BODIPY-C16
BODIPY-C16	6424337.667	OVCAR3-sh1 + BODIPY-C16
BODIPY-C16	4190073.333	OVCAR3-sh1 + BODIPY-C16
BODIPY-C16	4028891.333	OVCAR3-sh1 + BODIPY-C16
BODIPY-C16	3684489.333	OVCAR3-sh1 + BODIPY-C16
BODIPY-C16	3657392.333	OVCAR3-sh1 + BODIPY-C16
BODIPY-C16	5317029.333	OVCAR3-sh1 + BODIPY-C16 + EIPA
BODIPY-C16	6350206.333	OVCAR3-sh1 + BODIPY-C16 + EIPA
BODIPY-C16	6752906.333	OVCAR3-sh1 + BODIPY-C16 + EIPA
BODIPY-C16	5169699.333	OVCAR3-sh1 + BODIPY-C16 + EIPA
BODIPY-C16	7780022.667	OVCAR3-sh1 + BODIPY-C16 + EIPA
BODIPY-C16	6333563.667	OVCAR3-sh1 + BODIPY-C16 + EIPA
BODIPY-C16	6182256.667	OVCAR3-sh1 + BODIPY-C16 + EIPA
BODIPY-C16	6129978.667	OVCAR3-sh1 + BODIPY-C16 + EIPA
BODIPY-C16	4139409.333	OVCAR3-sh1 + BODIPY-C16 + EIPA
BODIPY-C16	4217025.333	OVCAR3-sh1 + BODIPY-C16 + EIPA
BODIPY-C16	3996119.333	OVCAR3-sh1 + BODIPY-C16 + EIPA
BODIPY-C16	4070721.333	OVCAR3-sh1 + BODIPY-C16 + EIPA
BODIPY-C16	6134815.333	OVCAR3-sh2 + BODIPY-C16
BODIPY-C16	4454662.333	OVCAR3-sh2 + BODIPY-C16
BODIPY-C16	5125694.333	OVCAR3-sh2 + BODIPY-C16
BODIPY-C16	5896627.333	OVCAR3-sh2 + BODIPY-C16
BODIPY-C16	4408596.333	OVCAR3-sh2 + BODIPY-C16
BODIPY-C16	4565003.333	OVCAR3-sh2 + BODIPY-C16
BODIPY-C16	5721195.667	OVCAR3-sh2 + BODIPY-C16
BODIPY-C16	7214955.667	OVCAR3-sh2 + BODIPY-C16
BODIPY-C16	4267372.333	OVCAR3-sh2 + BODIPY-C16
BODIPY-C16	4233709.333	OVCAR3-sh2 + BODIPY-C16
BODIPY-C16	4208785.333	OVCAR3-sh2 + BODIPY-C16
BODIPY-C16	5124385	OVCAR3-sh2 + BODIPY-C16
BODIPY-C16	5748852.333	OVCAR3-sh2 + BODIPY-C16 + EIPA
BODIPY-C16	6035896.333	OVCAR3-sh2 + BODIPY-C16 + EIPA
BODIPY-C16	6191357.333	OVCAR3-sh2 + BODIPY-C16 + EIPA
BODIPY-C16	5766863.333	OVCAR3-sh2 + BODIPY-C16 + EIPA
BODIPY-C16	6196518.667	OVCAR3-sh2 + BODIPY-C16 + EIPA
BODIPY-C16	6310782.333	OVCAR3-sh2 + BODIPY-C16 + EIPA
BODIPY-C16	4899773	OVCAR3-sh2 + BODIPY-C16 + EIPA
BODIPY-C16	4304609	OVCAR3-sh2 + BODIPY-C16 + EIPA
BODIPY-C16	4440727	OVCAR3-sh2 + BODIPY-C16 + EIPA
BODIPY-C16	3347581	OVCAR3-sh2 + BODIPY-C16 + EIPA
BODIPY-C16	4651747	OVCAR3-sh2 + BODIPY-C16 + EIPA
BODIPY-C16	6311814	OVCAR3-sh2 + BODIPY-C16 + EIPA
BODIPY-C16	14622122.67	OVCAR3-shCtrl + BODIPY-C16
BODIPY-C16	13929542.67	OVCAR3-shCtrl + BODIPY-C16
BODIPY-C16	13310777.67	OVCAR3-shCtrl + BODIPY-C16
BODIPY-C16	7604770.333	OVCAR3-shCtrl + BODIPY-C16
BODIPY-C16	12209286	OVCAR3-shCtrl + BODIPY-C16
BODIPY-C16	11621669	OVCAR3-shCtrl + BODIPY-C16
BODIPY-C16	13869056	OVCAR3-shCtrl + BODIPY-C16
BODIPY-C16	13063779	OVCAR3-shCtrl + BODIPY-C16
BODIPY-C16	14685852.67	OVCAR3-shCtrl + BODIPY-C16
BODIPY-C16	9558432.333	OVCAR3-shCtrl + BODIPY-C16
BODIPY-C16	7742002.333	OVCAR3-shCtrl + BODIPY-C16
BODIPY-C16	9294534.333	OVCAR3-shCtrl + BODIPY-C16
BODIPY-C16	4176741.667	OVCAR3-shCtrl + BODIPY-C16 + EIPA
BODIPY-C16	4716223.667	OVCAR3-shCtrl + BODIPY-C16 + EIPA
BODIPY-C16	4234360.667	OVCAR3-shCtrl + BODIPY-C16 + EIPA
BODIPY-C16	4523026.667	OVCAR3-shCtrl + BODIPY-C16 + EIPA
BODIPY-C16	4062439	OVCAR3-shCtrl + BODIPY-C16 + EIPA
BODIPY-C16	4097784	OVCAR3-shCtrl + BODIPY-C16 + EIPA
BODIPY-C16	4212924	OVCAR3-shCtrl + BODIPY-C16 + EIPA
BODIPY-C16	4337986	OVCAR3-shCtrl + BODIPY-C16 + EIPA
BODIPY-C16	8052529.333	OVCAR3-shCtrl + BODIPY-C16 + EIPA
BODIPY-C16	7162829.333	OVCAR3-shCtrl + BODIPY-C16 + EIPA
BODIPY-C16	7428650.333	OVCAR3-shCtrl + BODIPY-C16 + EIPA
BODIPY-C16	7710916.333	OVCAR3-shCtrl + BODIPY-C16 + EIPA
TMR-Dextran	80649.66667	OVCAR3-sh1 + TMR-Dextran
TMR-Dextran	92057.66667	OVCAR3-sh1 + TMR-Dextran
TMR-Dextran	97094.66667	OVCAR3-sh1 + TMR-Dextran
TMR-Dextran	77164.66667	OVCAR3-sh1 + TMR-Dextran
TMR-Dextran	42102	OVCAR3-sh1 + TMR-Dextran
TMR-Dextran	57520	OVCAR3-sh1 + TMR-Dextran
TMR-Dextran	48423	OVCAR3-sh1 + TMR-Dextran
TMR-Dextran	33039	OVCAR3-sh1 + TMR-Dextran
TMR-Dextran	39889.66667	OVCAR3-sh1 + TMR-Dextran
TMR-Dextran	34040.66667	OVCAR3-sh1 + TMR-Dextran
TMR-Dextran	6122.666667	OVCAR3-sh1 + TMR-Dextran
TMR-Dextran	17507.66667	OVCAR3-sh1 + TMR-Dextran
TMR-Dextran	57144.66667	OVCAR3-sh1 + TMR-Dextran + EIPA
TMR-Dextran	47588.66667	OVCAR3-sh1 + TMR-Dextran + EIPA
TMR-Dextran	57880.66667	OVCAR3-sh1 + TMR-Dextran + EIPA
TMR-Dextran	45394.66667	OVCAR3-sh1 + TMR-Dextran + EIPA
TMR-Dextran	15042	OVCAR3-sh1 + TMR-Dextran + EIPA
TMR-Dextran	10552	OVCAR3-sh1 + TMR-Dextran + EIPA
TMR-Dextran	21282	OVCAR3-sh1 + TMR-Dextran + EIPA
TMR-Dextran	16955	OVCAR3-sh1 + TMR-Dextran + EIPA
TMR-Dextran	39232.66667	OVCAR3-sh1 + TMR-Dextran + EIPA
TMR-Dextran	78133.66667	OVCAR3-sh1 + TMR-Dextran + EIPA
TMR-Dextran	23714.66667	OVCAR3-sh1 + TMR-Dextran + EIPA
TMR-Dextran	78954.66667	OVCAR3-sh1 + TMR-Dextran + EIPA
TMR-Dextran	101150.3333	OVCAR3-sh2 + TMR-Dextran
TMR-Dextran	53956.33333	OVCAR3-sh2 + TMR-Dextran
TMR-Dextran	75207.33333	OVCAR3-sh2 + TMR-Dextran
TMR-Dextran	45569	OVCAR3-sh2 + TMR-Dextran
TMR-Dextran	55996	OVCAR3-sh2 + TMR-Dextran
TMR-Dextran	45094	OVCAR3-sh2 + TMR-Dextran
TMR-Dextran	46935	OVCAR3-sh2 + TMR-Dextran
TMR-Dextran	80843.33333	OVCAR3-sh2 + TMR-Dextran
TMR-Dextran	28905.33333	OVCAR3-sh2 + TMR-Dextran
TMR-Dextran	52513.33333	OVCAR3-sh2 + TMR-Dextran
TMR-Dextran	102509.3333	OVCAR3-sh2 + TMR-Dextran
TMR-Dextran	55390.33333	OVCAR3-sh2 + TMR-Dextran
TMR-Dextran	85487.33333	OVCAR3-sh2 + TMR-Dextran + EIPA
TMR-Dextran	57467.33333	OVCAR3-sh2 + TMR-Dextran + EIPA
TMR-Dextran	29439	OVCAR3-sh2 + TMR-Dextran + EIPA
TMR-Dextran	43196.33333	OVCAR3-sh2 + TMR-Dextran + EIPA
TMR-Dextran	58852	OVCAR3-sh2 + TMR-Dextran + EIPA
TMR-Dextran	88358.33333	OVCAR3-sh2 + TMR-Dextran + EIPA
TMR-Dextran	61303	OVCAR3-sh2 + TMR-Dextran + EIPA
TMR-Dextran	45543	OVCAR3-sh2 + TMR-Dextran + EIPA
TMR-Dextran	49884.33333	OVCAR3-sh2 + TMR-Dextran + EIPA
TMR-Dextran	76580.33333	OVCAR3-sh2 + TMR-Dextran + EIPA
TMR-Dextran	52973.33333	OVCAR3-sh2 + TMR-Dextran + EIPA
TMR-Dextran	52272.33333	OVCAR3-sh2 + TMR-Dextran + EIPA
TMR-Dextran	670300	OVCAR3-shCtrl + TMR-Dextran
TMR-Dextran	722932	OVCAR3-shCtrl + TMR-Dextran
TMR-Dextran	1301008.333	OVCAR3-shCtrl + TMR-Dextran
TMR-Dextran	160136	OVCAR3-shCtrl + TMR-Dextran
TMR-Dextran	707709	OVCAR3-shCtrl + TMR-Dextran
TMR-Dextran	595747	OVCAR3-shCtrl + TMR-Dextran
TMR-Dextran	587182	OVCAR3-shCtrl + TMR-Dextran
TMR-Dextran	1517670.333	OVCAR3-shCtrl + TMR-Dextran
TMR-Dextran	1199423.333	OVCAR3-shCtrl + TMR-Dextran
TMR-Dextran	143336	OVCAR3-shCtrl + TMR-Dextran
TMR-Dextran	154944	OVCAR3-shCtrl + TMR-Dextran
TMR-Dextran	1043974	OVCAR3-shCtrl + TMR-Dextran
TMR-Dextran	198308	OVCAR3-shCtrl + TMR-Dextran + EIPA
TMR-Dextran	108296	OVCAR3-shCtrl + TMR-Dextran + EIPA
TMR-Dextran	171348	OVCAR3-shCtrl + TMR-Dextran + EIPA
TMR-Dextran	104444	OVCAR3-shCtrl + TMR-Dextran + EIPA
TMR-Dextran	124054.3333	OVCAR3-shCtrl + TMR-Dextran + EIPA
TMR-Dextran	61156	OVCAR3-shCtrl + TMR-Dextran + EIPA
TMR-Dextran	77296.33333	OVCAR3-shCtrl + TMR-Dextran + EIPA
TMR-Dextran	119259.3333	OVCAR3-shCtrl + TMR-Dextran + EIPA
TMR-Dextran	65911	OVCAR3-shCtrl + TMR-Dextran + EIPA
TMR-Dextran	77187	OVCAR3-shCtrl + TMR-Dextran + EIPA
TMR-Dextran	260991.3333	OVCAR3-shCtrl + TMR-Dextran + EIPA
TMR-Dextran	81986	OVCAR3-shCtrl + TMR-Dextran + EIPA

Supplementary Table 21. Single-cell metabolome of ovarian cancer cell lines

	SKOV3/DDP	SKOV3/DDP	SKOV3/DDP	SKOV3/DDP	SKOV3/DDP	SKOV3/DDP	SKOV3/DDP	SKOV3/DDP	SKOV3/DDP	SKOV3/DDP	SKOV3/DDP	SKOV3/DDP	SKOV3/DDP	SKOV3/DDP	SKOV3/DDP	SKOV3/DDP	[illegible]
Bilirubin	0	6.681093317	0	6.41206582	8.209331651	0	0	8.583219424	0	6.886748815	5.104603871	7.12430314	9.066901761	5.949059883	8.52080008	6.220230138	[illegible]
Inosine triphosphate	0	0	0	4.962111037	3.315504299	0	0	0	0	0	2.832610758	2.000065118	0	0	0	0	[illegible]
NADPH	0	0	0	12.55116798	12.49931576	11.65660975	11.04402363	12.05438555	0	8.108664509	3.350267487	10.25985315	13.2856993	9.178930511	10.336247	10.81072974	[illegible]
Liothyronine	0	0	0	9.892172113	12.07769531	9.893108463	8.367652267	8.649637078	0	7.693706334	0	9.327661184	11.26396767	7.941897764	9.801711565	9.108561704	[illegible]
Uridine triphosphate	0	0	0	0	3.289532485	2.617996445	2.215283551	0	0	0	3.344213669	2.441344397	0	0	0	0	[illegible]
CE(18:2(9Z,12Z))	0	0	0	10.11315228	11.98638488	8.45717795	7.521446245	10.92729168	0	5.813583127	0	8.808104768	10.65866242	0	8.572920145	9.679041502	[illegible]
Heparan sulfate	0	0	0	11.13471631	11.86801863	9.189447297	9.251051461	8.953102086	3.050422621	7.030572933	0	9.955317823	9.285698175	5.02891305	8.698314459	9.012289029	[illegible]
Lithocholyltaurine	0	0	0	0	0	0	2.026870852	0	0	2.300652922	3.102907202	2.882693441	0	2.789049684	0	2.214633323	[illegible]
N-Glycoloylganglioside GM2	4.64852452	0	0	12.13956634	12.37586034	10.18407196	10.19878231	12.21521014	6.902848299	8.50119272	11.20332022	10.30395852	12.02583408	6.235589041	10.66305721	11.04786765	[illegible]
3'-Sialyllactose	0	5.531683732	0	10.85521238	11.58218704	9.040210068	8.430561245	5.380483525	2.594197129	4.792466951	0	9.782668139	12.01248508	0	8.749016339	8.958258741	[illegible]
Tauroursodeoxycholic acid	0	0	0	0	0	0	0	0	0	0	0	2.653143859	3.80020994	0	5.087733346	2.221006743	[illegible]
Uroporphyrin IV	0	0	0	13.06861827	12.56371427	11.81775536	11.47165165	12.05228064	0	10.398126	4.979898234	12.00911952	13.46209714	6.620411923	11.81209277	10.16324397	[illegible]
Cer(d18:1/23:0)	0	0	0	9.006900197	9.853964232	7.620152279	7.490281125	9.667423946	0	6.186607607	0	8.741078087	11.26227938	4.785055337	8.017404293	8.216981557	[illegible]
UDP-D-Xylose	0	0	0	7.772825621	7.360268428	2.395617416	5.748818315	10.56569637	0	0	7.268456013	3.013136583	7.6905245	0	0	6.838490279	[illegible]
Uroporphyrinogen III	0	0	0	12.00110848	12.19617466	11.96358167	11.33521287	12.6849712	0	9.96093717	7.273080597	11.90027664	13.39557493	2.695215439	11.67241185	10.62250901	[illegible]
Phosphoadenosine phosphosulfate	0	0	0	0	0	4.600685613	3.887832084	4.492994142	0	3.74466619	3.943423784	2.987169386	0	0	3.968156987	2.300039536	[illegible]
Adenosine diphosphate ribose	0	0	0	7.190960087	7.316555037	0	0	8.447964677	0	0	0	4.742333298	7.393790117	0	6.473985611	6.833722803	[illegible]
Diadenosine tetraphosphate	0	5.5749128	0	13.14498631	13.27119756	12.45461069	11.0425242	12.83050447	4.042527912	8.946110347	7.074375672	11.45895111	14.00876849	7.047076471	12.21174707	11.80991985	[illegible]
FAD	0	4.773816669	0	12.38541271	11.80243827	12.00848791	11.61570422	11.57423312	4.747888797	9.330365334	5.818319451	12.09040604	13.89772869	4.794997131	11.84807359	10.44346566	[illegible]
Diguanosine tetraphosphate	0	4.614476547	0	12.09124619	12.51624839	10.59282757	10.27470616	11.70332297	4.961927162	10.5441834	4.467581671	11.91617259	12.95146108	0	10.79632544	11.03733078	[illegible]
Thymidine 5'-triphosphate	0	2.681293602	0	0	5.189314101	0	0	0	0	3.600504126	2.597787068	0	4.893712824	0	4.878653505	3.401404454	[illegible]
Guanosine triphosphate adenosine	0	0	0	12.19545361	11.46057563	11.64727253	11.08819041	12.09397432	0	8.989938451	0	11.87501881	13.47037284	0	11.66917735	10.35772998	[illegible]
-[6-deoxy-a-L-galactopyranosyl-(1->4	0	0	0	11.11892112	10.75366063	11.35975499	10.82809275	11.27481335	0	8.939351437	3.710910352	10.21965968	12.27731543	0	12.14664558	9.544776758	[illegible]
Astaxanthin	0	0	0	0	3.700011811	0	0	2.92912547	0	0	5.081236842	2.423112693	3.527050691	0	0	3.179308335	[illegible]
Acrylyl-CoA	0	0	0	13.05612219	12.99689405	10.84100904	11.74207625	12.71097161	3.420260161	9.249498181	5.907681499	10.69645099	12.36319474	7.666494429	10.97877608	12.25105088	[illegible]
Crocin	0	3.235873706	0	8.799612853	11.29925316	10.45750162	8.856844276	9.797014324	0	7.454240673	7.436525636	9.922329696	11.57729167	0	10.57054899	9.457543452	[illegible]
cochenodeoxycholic acid 3-glucuron	0	0	0	5.175759388	6.734233096	6.473082513	4.888124692	6.20589347	0	4.428644788	2.20659223	6.646285689	7.703066613	0	3.24505555	6.030179612	[illegible]
yo-Inositol 1,3,4,5,6-pentakisphospha	0	0	0	10.11494694	9.63048383	9.150746772	6.942945913	8.867270726	0	3.880096509	6.993123033	9.025645495	9.152977394	0	9.235800354	6.823962947	[illegible]
(2E)-Tetradecenoyl-CoA	0	4.016369815	0	10.22156765	11.76135228	11.22815145	10.63736156	12.04443273	2.208820256	8.467922912	5.659288121	10.42875541	12.63141727	0	11.85218758	9.867125248	[illegible]
hosphatidylinositol-3,4,5-trisphosphat	0	0	0	10.73373138	10.98645051	10.5595882	8.097503736	10.41096516	0	6.442643748	0	9.839845313	11.49533087	0	9.484908735	8.73128759	[illegible]
GlcCer(d18:1/26:1(17Z))	0	0	0	8.475549988	8.375164189	7.260488825	6.667422684	7.923398839	0	6.409030894	0	8.200521647	9.872449115	4.844052885	7.33940328	6.523074819	[illegible]
GlcCer(d18:1/26:0)	0	4.872614485	0	11.82345442	11.9395819	10.80958808	10.41278535	12.06420825	3.470816949	10.3508106	0	12.15357847	12.13867553	8.052771218	10.9985325	11.24197471	[illegible]
[illegible]	[illegible]	[illegible]	[illegible]	[illegible]	[illegible]	[illegible]	[illegible]	[illegible]	[illegible]	[illegible]	[illegible]	[illegible]	[illegible]	[illegible]	[illegible]	[illegible]	[illegible]

Acetylpuerarin	0	0	0	4.558696024	4.14021838	3.169068655	3.94491307	4.040259327	0	2.870641399	5.378796074	3.389736443	3.882249715	0
rihydroxy-6-(hydroxymethyl)oxan-2-	0	4.793009671	0	11.54172138	12.86485311	10.62605655	10.26998072	11.81622246	0	9.885897136	4.654354322	11.19719899	12.50299407	7.803561111
Annamycin	0	0	0	9.871463626	12.38371191	9.138256877	8.807199687	11.27002101	0	7.77135058	0	9.39482017	12.29079187	8.530159178
apicidin	0	0	0	5.544211613	6.064180424	3.033592891	2.046445603	5.815697138	0	3.02000835	0	3.903174476	6.205085051	2.865554151
Apomine	0	0	0	2.137617203	0	2.749917337	0	0	0	0	2.609970529	3.27731819	3.65534006	0
Arg-Pro-Pro-Gly-Phe-Ser-Pro	0	0	0	8.49873104	8.487243176	7.819694507	5.838227374	7.051092897	0	5.920484955	0	7.85956389	10.50013112	3.898186538
Asp-Asp-Asp-Asp-Lys	0	0	0	4.112914008	4.205577653	4.12603698	3.924156124	3.716847177	0	3.616646103	5.437438702	3.881914748	5.19612355	0
Astacin	0	4.338334367	0	7.44477359	9.093221855	7.900937775	4.807738638	4.938851096	5.603689527	5.536308734	8.280539607	6.574127193	8.241322731	0
Astragaloside I	0	0	0	12.86668458	13.42611167	11.89396901	11.62314198	12.8437629	0	8.284852899	4.542487452	11.23088636	13.15408792	6.69681114
oanamido)-3-(3-methylbutanoyl)-6-oxo	0	2.312981055	0	0	0	0	0	0	0	0	0	0	0	0
Atorvastatin lactone	0	3.594990045	0	2.575624881	2.289967139	2.350212437	0	0	0	0	0	2.562707228	2.711969841	0
Atrimustine	0	0	0	11.62918739	11.71950173	10.03504162	10.20900298	11.20351944	0	7.266874879	0	11.21662428	13.11200955	0
Avridine	0	4.343456058	0	9.713740632	11.89741176	9.003157969	6.918225424	9.706838659	0	0	0	8.970376264	10.58330857	0
Bachuoside VI	0	0	0	12.28579672	11.22309978	11.24874121	10.12829772	11.93708785	5.370867297	9.392073023	4.357620415	11.57623533	12.3052925	8.305009235
Bapta-AM	0	0	0	12.91065856	12.93972263	11.7822423	10.66880136	12.67242205	0	9.037529569	0	10.50865075	13.48873509	7.825781777
Benzoyl-fvr-pna	0	6.204593941	0	11.17250991	11.7817389	10.62618242	9.019324418	10.20938178	0	3.723609938	0	9.257743329	12.36585181	7.213491996
beta-Casomorphin-7	0	0	0	6.069886277	8.00562778	6.585772926	5.543830001	7.512242075	0	3.64533212	0	6.159013205	8.028605864	4.276093703
C-Telopeptide	0	0	0	12.84080515	11.65057322	11.98340151	11.74697759	12.83026499	0	10.26317863	4.197858288	11.98004332	13.45051009	7.123414604
Carminomycin II	0	0	0	7.46274376	6.287908565	5.953315324	4.636084803	6.073771179	0	3.997733424	0	5.374898664	8.247569721	2.759379337
Carzelesin	0	0	2.047469433	11.61216872	11.51781666	10.13851758	10.75389868	12.13807938	4.768562296	9.040847296	0	11.40965896	13.0590825	8.798400159
u-Thr-Asp 7-amido-4-trifluoromethylco	0	4.575788965	0	13.12254224	13.01838795	11.09482189	12.06300899	12.97129015	0	9.609379751	6.538061907	11.37686435	13.17191343	7.304680246
Cefcanel daloxate	0	0	0	10.29179658	11.78471611	8.791231512	7.380770777	10.24180521	0	7.922156902	0	10.55222248	12.56374487	7.488490918
cefteram pivoxil	0	0	0	3.188912409	2.045845343	0	2.642348694	3.042056272	0	0	3.994746704	2.105391898	0	0
Cephamycin A	0	0	0	9.966315025	9.461807748	8.387950956	8.05491578	8.725448891	0	4.804110918	0	7.607857659	9.449725843	0
-4-O-methyl-3-(4-((2,4,6-trimethylphe	0	0	0	11.45153512	12.70044654	10.88334881	9.876595538	10.32287121	3.205352832	7.435460948	7.023574341	10.73994734	9.622774629	0
Ala-Thr-Trp-Leu-Pro-Pro-Arg	0	0	0	11.8778823	12.34575513	10.81629891	10.60826971	11.29776518	3.66924677	8.130309164	3.282064635	9.680183419	12.74634617	5.155291246
Galactocerebroside	0	0	0	0	4.205909696	3.176847636	2.288131123	3.45692228	0	3.171219033	5.010523652	2.60008345	3.497407468	0
5-Azanonane deriv.	0	0	0	5.1808934	2.886442511	2.747932822	0	2.652770524	0	0	0	0	4.807947962	2.169534411
Chemistry 2	0	0	0	0	0	0	0	0	0	0	2.433086968	2.495260648	2.601107727	0
Closantel	0	0	0	10.51874061	10.72130549	9.331356881	6.92952562	10.93841816	0	4.154442474	0	10.6299911	12.5103327	5.187355849
propanoyl]pyrrolidin-2-yl]methylamino	0	0	0	0	6.757434548	4.44062195	5.527622263	0	0	0	5.623898209	4.940079014	4.857111145	0
-carboxyphenyl)amino]-1-naphthyl]-L-	0	0	0	11.95407647	10.83753266	10.31729502	9.718583827	10.62977482	0	7.188031735	0	10.80735851	12.82267857	6.541116974
Crustacean Cardioactive Peptide	0	4.69487555	0	12.88620451	12.12802249	12.17649154	11.41543328	12.38697267	4.043298601	10.27775627	5.030698368	11.17426969	13.1557373	4.220284143
Cryptophycin 52	0	0	0	10.23547057	10.93175092	9.712074479	9.261950422	9.855381265	0	6.445903051	0	9.713839935	13.09765893	6.423619013
Cyclin D1	4.129698074	2.332761301	0	11.47634301	12.26400054	12.43746667	10.32399852	11.57856883	4.406837105	10.45866544	4.689437657	12.39730193	13.18471148	0
Dechlorane plus	0	0	0	10.78384483	11.47000765	9.87955759	7.43044381	8.781291719	0	0	0	9.721409539	11.5496077	8.676792561
Deltaline	0	0	0	0	0	0	3.590647462	0	0	2.804830571	0	3.4166146	4.182295675	0
Deltorphin	2.723289201	3.196013722	0	12.70243012	12.14618471	12.40126769	11.65618621	11.88796898	0	10.67989792	0	11.6102963	12.62070774	7.132144955
Deltorphin II	0	2.107258029	0	12.03668973	12.67642748	11.31076018	11.36231843	12.41674265	3.45489875	8.171592957	5.189373658	11.80569276	12.87718339	5.725031162
Dpp-III	0	0	0	12.17241087	12.36467451	11.97285497	11.65660805	13.11437018	6.211702817	9.945750568	0	11.74977462	13.99991762	6.21609253
Epothilone B	0	0	0	0	0	0	0	0	0	0	3.460380012	0	4.249408705	0
F1alpha	0	0	0	10.62475647	11.1899454	9.954427303	9.039986597	11.17078351	0	4.863425997	6.3113789	10.4054841	11.7745755	5.399262749
Fluo-3	0	0	0	12.70060746	13.35186272	11.76840818	10.81517507	11.9085777	0	7.564106102	5.965855387	10.63082435	13.45541853	8.432052927
Fura-PE3	0	0	0	7.76806247	7.669853783	6.517756438	5.638513778	7.145959469	0	4.635872258	0	6.982721491	8.236647987	3.176236786
Glecaprevir	0	0	0	13.16273091	13.94507089	11.59177068	12.0332531	12.91041702	5.159574236	9.005595597	0	10.93835713	11.97824358	6.3215684
Gliadins	0	0	0	4.347751894	4.434413448	2.935274025	2.326705543	3.707382161	0	2.391209787	2.967928817	3.42810406	4.644948445	0
ohexylcarbamoyl)amino]ethyl]phenyl	0	0	0	0	2.867138627	2.046864092	0	2.198100885	0	0	3.023576541	0	0	0
Hemiasterlin	0	0	0	0	0	2.052046921	0	0	0	0	2.969991152	0	0	0
Hexa-His	0	0	0	10.34084242	10.38597496	9.335489872	9.52110898	10.73955567	0	7.105638153	4.467156806	9.150912796	11.26657994	5.349391407
[illegible]	[illegible]													

[illegible]

Supplementary Table 22. Training and Validation Sets for Logistic regression model

Training set

	SH3YL1	MLYCD	CD44	LIU_OVARIAN_CANCER_TUMORS_AND_XENOGRAFTS_KINASES_DN	KEGG_FATTY_ACID_METABOLISM	KEGG_MISMATCH_REPAIR	REACTOME_MET_ACTIVATES_RAP1_AND_RAC1	WP_COMPLEMENT_SYSTEM	WP_DNA_REPAIR_PATHWAYS_FULL_NETWORK	MANUAL_DORSAL_RUFFLE_FORMATION	GOBP_FATTY_ACID_CATABOLIC_PROCESS	GOBP_POSITIVE_REGULATION_OF_RUFFLE_ASSEMBLY	HOEGERKORP_CD44_TARGETS_DIRECT_DN	TCR_SIGNALING_PATHWAY	Group
HGSOC1	-0.77355	-1.083625	-0.591858	-0.172075834	0.444225337	-0.631949235	-0.251188366	-1.129114876	-0.9631917	1.134597588	0.820661607	0.249755777	0.881533287	-0.195098137	sensitive
HGSOC2	-0.77355	-1.083625	-0.317514	-0.520666834	-2.171706213	0.964193034	0.054047001	-1.442558855	1.347824306	0.336259812	-0.920478633	-0.492124766	0.320913975	-1.290576513	sensitive
HGSOC3	-0.77355	-1.083625	0.4269746	-0.308563535	0.353241409	1.152412626	-0.175253773	-0.491407813	0.621172094	1.323339151	-0.255743418	0.845886169	1.006767195	-0.398591296	sensitive
HGSOC4	-0.77355	0.7187563	0.7321608	0.836972143	-0.066241869	0.254969009	-1.205159847	0.75163838	-0.399839218	-0.726862938	0.391095888	-0.243815905	-0.413308272	0.27382643	sensitive
HGSOC5	-0.77355	0.8643953	0.3840012	0.666739238	-0.346602312	-0.396659373	-2.566829458	-0.261392995	-0.2351547	-0.140595024	-0.6994705	-0.985898253	-0.653293804	1.488391221	sensitive
HGSOC6	-0.77355	-1.083625	0.0592401	0.338482732	-0.132816269	1.067758054	-1.129211438	0.990695604	1.133027684	-1.835795079	-1.260310841	-0.501561922	-0.239322403	0.537446887	sensitive
HGSOC7	-0.77355	-1.083625	0.500455	0.519896243	0.272231895	0.01505658	0.050223247	1.163683289	0.467122495	-0.999705995	-0.302835961	1.067833565	-0.379129926	0.870642756	sensitive
HGSOC8	-0.77355	-1.083625	1.0956847	1.059088994	-0.302040894	-1.096225332	1.919607918	1.117732252	-1.659736385	-1.121723604	-0.12694728	-0.582566242	-2.116347399	0.726687298	sensitive
HGSOC9	-0.77355	-1.083625	1.5379735	1.413790392	-0.918191969	-1.034709421	-0.123902189	1.189666995	-1.60606358	-0.96272167	-0.71344368	-1.11691919	-0.945837684	0.880242176	sensitive
HGSOC10	-0.77355	-1.083625	0.922579	1.25215892	-0.483447553	0.08781316	-0.44514474	1.088117466	-0.225985974	-0.859379344	-1.328382562	-1.953906943	-0.326012155	1.521126787	sensitive
HGSOC11	1.2335875	0.7873158	-1.275373	-1.558312464	1.138018741	0.738070174	0.57679775	-0.520308799	0.253908063	0.790989222	0.952792728	0.56116349	0.47159155	-0.922872598	resistant
HGSOC12	-0.77355	0.8785062	-0.379911	-0.435389901	1.200262416	0.932654278	0.064326475	0.680559344	0.802891919	1.176352007	0.339302893	0.855874485	-0.37865549	-0.735297332	resistant
HGSOC13	1.1080115	1.0128634	-2.489645	-1.799475599	-1.971247674	1.496080815	0.885803849	-0.761956175	1.27696175	1.276896744	-1.249005056	0.880429475	2.130948527	-1.469934208	resistant
HGSOC14	1.180296	0.969811	0.2328823	0.329034857	0.667986892	-1.130382078	0.843807357	0.128117647	-0.49638403	-0.474324603	1.087825408	1.792679079	-0.609860175	0.218715522	resistant
HGSOC15	1.3831097	0.7740365	-0.309151	-0.39391778	-0.642729345	-0.020639435	-0.492723222	0.107297552	-0.046900407	0.411416681	-0.286564263	-0.506402993	-0.10192675	-0.955969591	resistant
HGSOC16	1.3158042	0.9201114	-1.469926	-1.98002268	1.227548848	0.802259099	0.231640848	-2.272939573	1.571999264	0.966083021	2.535527372	0.92979715	0.121423896	-1.522182933	resistant
HGSOC17	1.2245326	0.7739232	0.0904566	0.198795225	0.653034151	-1.559820875	0.912515629	-0.267355479	-0.998487916	0.558331555	0.572652446	0.514920778	1.79148396	-0.17583966	resistant
HGSOC18	1.063711	0.9692808	0.8509694	0.553465885	1.078474408	-1.640881081	0.850642958	-0.070473963	-0.843163665	-0.853157522	0.443323853	-1.315143753	-0.560968331	1.149283191	resistant

Validation set

	SH3YL1	CD44	MLYCD	LIU_OVARIAN_CANCER_TUMORS_AND_XENOGRAFTS_KINASES_DN	KEGG_FATTY_ACID_METABOLISM	KEGG_MISMATCH_REPAIR	REACTOME_MET_ACTIVATES_RAP1_AND_RAC1	WP_COMPLEMENT_SYSTEM	WP_DNA_REPAIR_PATHWAYS_FULL_NETWORK	MANUAL_DORSAL_RUFFLE_FORMATION	GOBP_FATTY_ACID_CATABOLIC_PROCESS	GOBP_POSITIVE_REGULATION_OF_RUFFLE_ASSEMBLY	HOEGERKORP_CD44_TARGETS_DIRECT_DN	TCRsignalingPathway	Group
372440	0.6508696	-1.727721	0.7925714	-1.344135081	0.800584678	-1.006043188	-0.032453398	-1.873198632	0.04051547	1.249724968	0.85252919	-0.178438632	-0.473504944	-0.420769521	sensitive
40353_A3	-0.351837	-2.447237	1.2547874	-0.352484215	1.760511319	-0.758888702	-1.465660095	-0.241253104	-0.497544151	-0.492800184	1.324175304	1.08448971	0.067088695	-2.725917277	resistant
S-14-0007480_A6	-1.624778	0.2599613	-1.785644	-1.739073083	-1.685793017	0.802438984	0.162405166	1.613036929	1.151549778	-1.668371592	-1.837945937	1.139969684	2.141855777	-1.600865488	resistant
493391	0.3422558	0.2503206	-1.640064	0.882075377	-0.653571956	1.016847901	0.13472995	-1.270085496	1.324490854	0.62172488	-0.33612706	-0.386608819	-0.215568767	2.178245261	sensitive
37440_A1	1.8871898	-1.05267	-0.171656	-1.129795891	0.627132325	0.018185341	-0.062835262	-1.211008404	-0.131007227	0.900321673	0.144478329	2.09783811	-0.556890231	-0.138021882	sensitive
208877	0.7484664	-1.265528	-0.995543	-0.879492262	0.100879875	1.800450014	-0.607479301	-1.327788972	1.244678701	0.235793802	0.151286965	-0.209695529	-0.542845633	-1.43046551	sensitive
231865;230368	0.2556075	-2.038909	-0.157171	-2.163688942	0.19845145	1.17974096	-2.839254375	-2.177429754	1.836583179	0.590662557	0.373862432	0.872008863	-2.234589879	-2.095689913	resistant
40885_B1	0.6778185	-0.668572	2.3608136	-0.588456094	1.923429426	-0.355283338	0.103993251	-1.459078705	-0.662554026	1.731138402	1.726745555	1.524845845	-2.106061634	0.416870848	resistant
210413	0.5008677	-0.327402	0.6080124	1.130882479	-0.878907256	-1.373917122	0.151101967	0.738189909	-0.980591145	0.538620732	-0.490691267	0.179498298	0.898981953	0.476269688	resistant
S-14-0030753_E1	-0.675365	-0.633708	0.5423548	-1.243507853	-0.272292887	1.787196821	-1.18443049	-1.085568833	1.501245869	-1.514978723	-0.430080463	-0.3844098	0.588566649	-1.33616766	sensitive
360612	-0.11025	-0.265929	0.2298089	0.498755145	1.478161675	-0.760863677	-0.018074294	-0.101661498	-0.125756188	1.259829597	1.882991277	1.296045527	-0.524193526	1.632307747	resistant
S-11-0010626_B3	-0.589119	0.4571509	-1.016533	0.395105527	-1.265608301	-0.45218785	-0.086661546	0.149639938	-0.189727221	-1.739300009	-1.365007818	-0.528255089	1.225594396	-0.839740721	sensitive
37530_B9	-0.24942	-0.477543	1.3597701	0.339924513	0.87042134	-0.61100245	-0.248346675	-0.2302687	-1.497164275	1.131286459	0.450417552	0.363856671	-0.915686835	0.057753755	sensitive
215376	-0.239027	2.7682232	-1.625779	1.554932007	-1.015453437	1.998553936	1.761543811	0.392544436	1.466879518	0.351655716	-1.166766356	0.292129411	-1.461411647	0.77119841	resistant
S-10-0035607_A4	-0.394824	-0.575731	-1.298764	-0.223916642	-1.033633667	0.311019144	0.576497426	-0.214050058	1.44680051	-0.663741793	-1.878362154	-0.75912286	0.854748789	-0.530039286	sensitive
S-11-0025953_B7	-0.275117	2.0945903	-1.935552	-0.227808217	-1.747183443	0.27274372	0.926295876	1.711496926	0.03557979	0.553382871	-1.551058295	-1.002443549	1.292490091	0.809053825	sensitive
37572_A1	-1.264708	0.2816119	1.045078	0.231507588	1.327352052	-1.237074669	0.072898138	0.494014214	-1.620291122	-0.173779765	1.477529216	0.377704334	-1.140285182	1.029585308	sensitive
39433_A4	0.6187014	-0.308076	0.5565785	-0.114915608	1.661639427	-0.710247946	0.420038744	1.141701955	-1.24413745	0.65537081	1.21109734	-0.751030388	0.268034211	0.366665737	sensitive
38791_A1	0.4516162	0.3014269	-0.081986	0.827707408	0.848187755	0.367803412	0.832275979	-0.640433244	0.346817659	0.599371729	-0.084160803	-1.145080892	1.326953937	-0.326531773	resistant
555178;555177	-0.102724	-0.356102	-0.483199	0.134123095	0.744357038	1.073067282	-0.455575505	-0.401349052	0.537608115	0.800713723	0.855314093	-0.157771245	-0.007041963	-1.000272149	sensitive
36754_C4	-0.091511	-1.470008	0.568014	-1.352786454	-0.737030857	-0.19475195	-0.025778508	-0.47265762	-0.008879067	-0.398506497	-0.488285666	1.164889983	0.07865051	-1.797888503	sensitive
654490	-0.3946	-0.378476	1.0012691	-0.686658395	0.867940074	1.432358942	0.401595472	-0.796872754	1.406752895	-0.048165924	0.818132186	-1.206739284	0.315047261	0.487831686	sensitive
S-07-0032072_A8	-0.816947	-0.981566	-0.02418	-0.774800468	-0.844809818	1.386420341	0.925036948	0.608091788	1.976723492	-0.806013889	-1.330078865	-1.268633324	0.493312127	-1.703260405	sensitive
S-11-0009142_B2	-0.700277	0.7895987	-1.085649	-1.597011532	-0.804305056	1.989167092	0.321412312	0.223805967	1.842818013	0.830003024	-0.884055435	-1.830759959	-0.100387767	-0.061751506	sensitive
517774	0.2174061	-0.676254	0.1126095	-0.529569546	0.248999959	-0.003074648	0.336614811	-0.686579148	0.409720122	0.222190771	0.728042914	-0.297860399	-0.850116034	-1.092065571	sensitive
S-17-0024563_A14	-3.521026	1.4941913	0.1758152	1.460344827	-0.80954545	0.394892371	1.743624803	1.151789535	0.715102481	-3.266285062	-0.700134591	-1.37081026	-0.411056604	1.45717928	resistant
41079_A6	-2.556409	-0.83904	1.4129102	-0.528584671	1.070049189	-0.962302466	0.377581189	0.461420326	-0.249391667	-2.359821185	1.713550003	0.96150109	0.909317476	-1.044554861	sensitive
297929	0.2799636	-1.149462	0.2709397	-0.068537311	1.670781658	-0.914122921	-1.062279322	-0.826571784	-1.056990323	0.847401338	2.009787662	1.361586033	-0.635251316	-1.810868348	sensitive
S-12-0032630_B14	1.0929961	2.4132913	-1.051815	-0.836957248	-1.132654806	-0.188298193	2.148241321	0.95617415	0.190257713	1.272482578	-1.395888795	-0.408126049	1.806693469	-0.17053715	sensitive
555596	0.586919	1.0525585	-0.546921	1.995949629	0.021955642	-0.573105847	1.353419229	1.407224031	-0.205047208	-0.919651155	-0.426279436	0.93524715	0.646255398	0.394288153	sensitive
S-17-0012504_C1	2.6476016	0.6476714	-0.203926	0.519226046	-0.448696337	1.982221367	1.29039023	-1.09313044	2.39571649	-0.057426434	-0.771247714	0.506966792	-0.612120926	0.561995209	resistant
42695_C3	0.7474258	-0.831687	-1.128104	0.906601433	1.030096504	-0.991037587	1.204657781	-0.421186964	-0.094602567	-0.218351538	0.641860919	-0.208139657	-0.577754377	-0.129961631	resistant
S-07-0023499_A2	-0.150858	-0.8248	-0.113186	0.360525921	-0.559853511	-1.44639649	0.373781487	1.34459757	-0.918387662	-1.326468548	-0.386094471	0.818047883	0.0385049	-0.947915986	resistant
S-07-0027436_B6	-1.923861	0.9652684	1.1999585	2.064447428	-0.001467592	-0.853716225	1.231908891	1.812588375	-0.54518525	-2.287580639	0.05094412	-0.665906624	-0.15638639	1.008894892	resistant
525976;525977	1.6068642	0.0091176	-1.028508	1.416154686	-1.180735364	-0.939494758	0.060478888	1.011303636	-0.510131408	0.071738046	-0.910053655	0.077335051	-0.09226183	0.118786923	sensitive
277568	0.146297	1.949703	0.8371211	0.532231981	-0.515549759	0.078088281	0.889500442	0.452800511	-0.409235204	0.414787695	0.174487172	-0.315967482	-1.412691414	-0.028397213	sensitive
527499	1.4893341	-0.270846	-0.912124	1.370896471	0.174131422	-0.58291693	-0.121279482	0.259645038	0.356902809	0.259367054	0.014413604	-0.140545738	-0.466444179	-0.599524253	sensitive
40885_B1.1	0.6778185	-0.668572	2.3608136	-0.588456094	1.923429426	-0.355283338	0.103993251	-1.459078705	-0.662554026	1.731138402	1.726745555	1.524845845	-2.106061634	0.416870848	resistant
S-14-0024532_A2	-1.601511	0.7495847	-1.172289	-0.787443397	-1.696798559	-0.30445695	0.145916482	0.972326883	0.554772491	-1.619366739	-1.572243426	-2.404781112	1.379142959	-0.316220598	sensitive
500550	-1.17517	0.0521962	-0.183222	1.210374926	-0.609982399	-1.65925111	0.726927313	1.067818353	-1.106094705	-1.128472798	-0.385026747	0.301917822	0.706430354	1.659409114	sensitive
S-12-0007994_B6	-0.52123	-0.464904	-0.304839	-1.590754614	-0.558149162	-0.709951448	1.672751321	-0.728859544	-1.125159729	-0.248238917	-0.162193901	1.114126308	1.024318201	-0.227452297	sensitive
271005	-0.161776	-0.52841	0.1744148	0.691012768	-1.050036861	-0.145452047	-0.651959219	-0.477659663	0.235278389	-0.547952502	-0.568750091	1.357497833	0.456339166	-0.036357928	sensitive
39352_B1	-0.423027	-0.946661	-0.122578	0.054740051	1.135662771	-2.03464308	-0.30235638	-0.102762321	-2.044587105	0.548675031	1.459880531	-1.300329571	-1.667732595	-0.492586414	resistant
500395	2.3402691	-0.756477	0.3288074	-0.721438538	0.070789157	0.414651376	-0.076283405	-1.242530604	0.179274407	1.009821972	0.367106055	-0.973969728	-0.133538816	-0.159998463	sensitive
37712_C1	1.0116564	0.3242125	-1.793676	0.828251034	-0.09366954	0.141483359	0.461417053	0.246421826	0.067570202	0.635954371	-0.258212849	0.076802018	-0.569654736	1.50641139	sensitive
352244	1.0032944	-0.59353	-0.600164	0.152484123	-0.575772082	0.263395514	-2.007974408	-0.405589915	0.482011483	0.332045154	-0.434203351	-0.148103915	-0.231872702	-0.145629186	resistant
555588	0.401296	0.3192472	-1.413671	2.281854772	-0.930768312	-1.573100106	0.300214085	1.61461487	-1.424688988	-0.336572077	-1.317867491	0.661977798	0.673881252	0.59343832	sensitive
S-13-0021879_A8	-0.88826	0.2198141	-0.81347	-1.076269572	-0.294339856	0.765998791	0.075089806	0.563661821	0.525548267	0.096859321	-0.655010992	-1.148653964	0.723953945	-0.475832791	sensitive
37504_B1	-0.875412	0.1148106	0.0817613	-1.482605774	0.316319941	0.820248571	0.346048398	-1.262387646	0.223876663	0.24462927	0.098075864	0.497605608	-0.824859057	0.60876244	resistant
S-11-0020094_A3	-0.114136	0.8027532	0.180479	-0.167973888	0.184745277	1.468545408	0.718464674	1.442343184	0.378392874	0.762000602	-0.091746397	1.015671972	0.075745938	0.249050571	sensitive
296927	0.7453906	-0.58993	1.4584338	0.091204586	-0.861554075	-0.389525333	-0.030136962	0.405549727	-0.599707076	0.858265747	-0.22027139	0.524305159	0.875046867	0.802840802	sensitive
654490.1	-0.3946	-0.378476	1.0012691	-0.686658395	0.867940074	1.432358942	0.401595472	-0.796872754	1.406752895	-0.048165924	0.818132186	-1.206739284	0.315047261	0.487831686	sensitive
206053	-1.665875	-0.103478	-0.281041	-0.36421986	-1.186339489	0.467201162	-0.991737387	0.174885902	0.433590483	-1.34943334	-1.0505237	0.295697555	0.697959321	0.400772224	sensitive
232982	0.5052159	-0.019892	-0.305151	0.231759208	-1.072914143	-0.07004733	-1.253047296	-0.071391819	-0.705885606	-0.079640179	-0.765204039	1.581913491	1.603361717	0.030806133	sensitive
S-13-0021202_A5	-0.800735	0.2965566	-0.780822	1.30318781	-0.35792879	-0.389597135	0.916313484	0.824418134	-0.224427132	-1.239606941	-1.213907474	-0.569958704	1.040093026	-0.064917164	resistant
212713	0.561603	1.6248258	1.3377422	-1.064556949	1.190133932	0.364429386	-1.055288045	-1.17538322	0.132987937	0.485162802	1.115225787	-0.848489889	-0.322109291	-0.592343581	sensitive
S-07-0006305_A1	0.1260435	-0.337122	-1.089892	-0.250545855	-0.625531998	0.926194213	-0.429254613	-1.33032792	1.41503083	-0.419245073	-0.913540835	-2.019211047	-0.457395192	-1.874881402	sensitive
38735_A6	0.0657184	-0.069358	0.8481494	-0.496299968	1.130003435	-0.028576099	-1.104920219	0.323280289	-0.683164439	1.289645676	0.617854744	-0.390446234	-2.244606059	0.652492051	resistant
517758	0.6268928	0.3895624	-0.24615	0.522654563	-0.743066539	-0.617195026	-0.527685143	0.310422043	-0.543045326	-0.030367456	-0.736451827	0.564905476	0.218008219	0.057961876	sensitive
S-11-0016358_A3	-0.463893	1.2418469	0.2733711	0.437158474	-0.418908128	-0.832056345	0.24762608	1.874974214	-1.049982691	0.047996342	-0.17055504	0.588922305	0.431126117	0.173222618	sensitive
S-07-0021920_B3	-0.45585	1.5534446	-1.366193	-0.183058536	-1.421302658	1.421761401	1.229349391	0.48176879	1.291578963	-0.572744651	-1.462965074	-0.782674709	0.804910443	-0.053912626	sensitive
37917_D6	-0.092048	0.8542787	-0.17621	1.836211633	0.044842127	-1.934266034	0.940215021	0.950480685	-1.844315998	-0.212981006	0.132985798	-0.096481579	0.339869729	2.30523386	sensitive
39099_A2	0.5774275	1.6673597	-0.206091	1.578374173	0.192470629	0.763720104	0.822717066	-1.438097751	0.342046405	0.092247003	0.143230739	-0.64191757	-1.710082256	2.515594995	sensitive
40885_B1.2	0.6778185	-0.668572	2.3608136	-0.588456094	1.923429426	-0.355283338	0.103993251	-1.459078705	-0.662554026	1.731138402	1.726745555	1.524845845	-2.106061634	0.416870848	resistant
41911_C6	-0.53053	-0.852881	0.1910937	-0.981346913	1.377755717	-1.244852731	-0.330398553	1.046827089	-2.006257535	0.058850222	0.882287008	0.151280169	1.250202142	-0.407423226	sensitive
38999_B2	0.1175397	-0.939938	1.800631	-0.117615031	1.599918791	0.680143024	0.265788592	-1.33045127	0.279656013	1.210628453	1.751593059	-0.51792078	0.793520737	0.626407509	sensitive
474534;474536	0.0369038	-0.351582	-0.105599	0.592875378	-1.198757131	-0.427135845	-1.188403653	0.013952889	-0.635626493	-1.143162335	-0.592602996	0.678613834	0.617870289	-0.125350466	sensitive
503836;503834	0.0435052	-0.236381	-0.382089	0.464858981	-0.960971347	-0.394227674	-0.891067773	0.027203294	-0.894791803	-0.815836861	-0.567722901	1.050269667	0.555438909	0.005719655	sensitive
S-13-0010181_B3	-0.940432	1.2151704	-0.398002	-0.630662656	0.887204559	-0.320948474	-1.651770609	-0.393313527	0.200716724	0.277243921	0.348737763	-1.163217056	-1.066236201	0.136380256	sensitive
654490.2	-0.3946	-0.378476	1.0012691	-0.686658395	0.867940074	1.432358942	0.401595472	-0.796872754	1.406752895	-0.048165924	0.818132186	-1.206739284	0.315047261	0.487831686	sensitive
351913;352452	1.2816279	-0.623417	0.4281935	-0.749753591	0.740720402	0.334074767	-2.561611229	0.232930885	-0.237258685	0.767320735	1.532163891	-0.520077089	-0.964783135	-0.367695063	sensitive
555175	0.4334046	0.6510831	-0.178796	1.163696434	-0.526317139	-0.982495354	-0.720549714	1.014836956	-0.076791441	-0.449865784	-0.426794683	0.492834375	0.099016105	0.965355778	sensitive
S-14-0021444_B4	0.5575416	0.0070061	-0.308479	-1.181346426	-0.467900601	1.15438496	-0.397370407	0.382809362	-0.432716387	0.403149907	-0.799642345	-1.490024214	1.018231278	0.226732809	sensitive
218201	0.6733106	-0.074904	0.609732	-0.034705092	-0.004469507	0.044203735	-0.507309741	-0.418496466	-0.684118255	1.185648249	0.568970822	2.110332736	0.037210253	-0.495320382	sensitive
228608	0.1192611	1.3317907	0.7493397	0.750914673	-0.635797714	-1.103277816	-2.790807312	1.517735315	-1.192230773	-0.674928569	0.088242959	-0.522244943	0.893677256	0.574204	sensitive

Parameters of the logistic regression model

Call:
glm(formula = Group ~ ., family = binomial(), data = scaled_data_fix_CPTAC)

Coefficients:

	Estimate	Std. Error	z value	Pr(>|z|)
(Intercept)	3.57	71451.959	0	1
SH3YL1	-14.865	196154.63	0	1
MLYCD	-9.009	133442.94	0	1
CD44	15.004	293417.03	0	1
LIU_OVARIAN_CANCER_TUMORS_AND_XENOGRAFTS_KINASES_DN	-24.033	532385.39	0	1
KEGG_FATTY_ACID_METABOLISM	-14.656	206569.28	0	1
KEGG_MISMATCH_REPAIR	8.134	306956.41	0	1
REACTOME_MET_ACTIVATES_RAP1_AND_RAC1	-6.337	121912.41	0	1
WP_COMPLEMENT_SYSTEM	-1.3	271589	0	1
WP_DNA_REPAIR_PATHWAYS_FULL_NETWORK	-13.279	196864.36	0	1
MANUAL_DORSAL_RUFFLE_FORMATION	-8.387	176354.45	0	1
GOBP_FATTY_ACID_CATABOLIC_PROCESS	10.532	259625.66	0	1
GOBP_POSITIVE_REGULATION_OF_RUFFLE_ASSEMBLY	2.891	100038.04	0	1
HOEGERKORP_CD44_TARGETS_DIRECT_DN	5.106	149840.43	0	1
TCRsignalingPathway	12.657	242077.14	0	1

(Dispersion parameter for binomial family taken to be 1)

Null deviance: 2.4731e+01 on 17 degrees of freedom
Residual deviance: 3.2125e-10 on 3 degrees of freedom
AIC: 30

Number of Fisher Scoring iterations: 24

Supplementary Table 23. Prediction results of the logistic regression model

	Predicted	Actual
372440	resistant	sensitive
40353_A3	resistant	resistant
S-14-0007480_A6	sensitive	resistant
493391	sensitive	sensitive
37440_A1	resistant	sensitive
208877	resistant	sensitive
231865;230368	resistant	resistant
40885_B1	resistant	resistant
210413	resistant	resistant
S-14-0030753_E1	sensitive	sensitive
360612	resistant	resistant
S-11-0010626_B3	sensitive	sensitive
37530_B9	resistant	sensitive
215376	sensitive	resistant
S-10-0035607_A4	resistant	sensitive
S-11-0025953_B7	sensitive	sensitive
37572_A1	sensitive	sensitive
39433_A4	resistant	sensitive
38791_A1	resistant	resistant
555178;555177	resistant	sensitive
36754_C4	sensitive	sensitive
654490	sensitive	sensitive
S-07-0032072_A8	resistant	sensitive
S-11-0009142_B2	sensitive	sensitive
517774	resistant	sensitive
S-17-0024563_A14	sensitive	resistant
41079_A6	sensitive	sensitive
297929	resistant	sensitive
S-12-0032630_B14	sensitive	sensitive
555596	resistant	sensitive
S-17-0012504_C1	resistant	resistant
42695_C3	resistant	resistant
S-07-0023499_A2	resistant	resistant
S-07-0027436_B6	sensitive	resistant
525976;525977	resistant	sensitive
277568	sensitive	sensitive
527499	resistant	sensitive
40885_B1.1	resistant	resistant
S-14-0024532_A2	sensitive	sensitive
500550	sensitive	sensitive
S-12-0007994_B6	sensitive	sensitive
271005	sensitive	sensitive
39352_B1	resistant	resistant
500395	resistant	sensitive
37712_C1	sensitive	sensitive
352244	resistant	resistant
555588	resistant	sensitive
S-13-0021879_A8	sensitive	sensitive
37504_B1	sensitive	resistant
S-11-0020094_A3	sensitive	sensitive
296927	resistant	sensitive
654490.1	sensitive	sensitive
206053	sensitive	sensitive
232982	sensitive	sensitive
S-13-0021202_A5	resistant	resistant
212713	sensitive	sensitive
S-07-0006305_A1	resistant	sensitive
38735_A6	resistant	resistant
517758	sensitive	sensitive
S-11-0016358_A3	sensitive	sensitive
S-07-0021920_B3	sensitive	sensitive
37917_D6	sensitive	sensitive
39099_A2	sensitive	sensitive
40885_B1.2	resistant	resistant
41911_C6	sensitive	sensitive
38999_B2	resistant	sensitive
474534;474536	sensitive	sensitive
503836;503834	sensitive	sensitive
S-13-0010181_B3	sensitive	sensitive
654490.2	sensitive	sensitive
351913;352452	sensitive	sensitive
555175	sensitive	sensitive
S-14-0021444_B4	sensitive	sensitive
218201	sensitive	sensitive
228608	sensitive	sensitive

Supplementary Table 24. List of reagents and resource

REAGENT or RESOURCE	SOURCE	IDENTIFIER
**Antibodies**		
Rabbit monoclonal anti-β-actin	Boao Rui Jing	Cat# Ab1015t-hrp<br>RRID: AB_2146403
Rabbit monoclonal anti-CD4 (IHC)	Cell Signaling Technology	Cat# 25229T<br>RRID: AB_2798898
Alexa Fluor® 555 Anti-CD4 antibody (IF)	Abcam	Cat# AB280849<br>RRID: AB_3107080
Anti-CD8 alpha antibody (IF)	Abcam	Cat# AB251596<br>RRID: AB_1834350
Pan Cytokeratin Monoclonal Antibody (IF)	Invitrogen	Cat# 53-9003-82<br>RRID: N/A
Mouse monoclonal anti-CD44 (IF)	Novus Biologicals	Cat# NBP2-34520PCP<br>RRID: N/A
Rabbit monoclonal anti-Granzyme B (IF)	Cell Signaling Technology	Cat# 17215T<br>RRID: AB_2798780
Alexa Fluor® 647 Anti-PD1 antibody (IF)	Abcam	Cat# AB201825<br>RRID: AB_3073680
Rabbit monoclonal anti-TCF1/TCF7 (IF)	Cell Signaling Technology	Cat# 2203T<br>RRID: N/A
Rabbit monoclonal anti-TOX (IF)	Abcam	Cat# AB322259<br>RRID: N/A
Rabbit monoclonal anti-PD-1 (WB)	Cell Signaling Technology	Cat# 86163T<br>RRID: AB_2728833
Rabbit monoclonal anti-CTLA-4 (WB)	Cell Signaling Technology	Cat# 53560T<br>RRID: AB_3107072
Rabbit monoclonal anti-CD44 (WB)	Cell Signaling Technology	Cat# 37259T<br>RRID: AB_2750879
Rabbit monoclonal anti-INF-γ (WB)	Cell Signaling Technology	Cat# 8455S<br>RRID: AB_2797644
Rabbit monoclonal anti-CD69 (WB)	Cell Signaling Technology	Cat# 39275S<br>RRID: N/A
Rabbit monoclonal anti-2B4 (WB)	Cell Signaling Technology	Cat# 54560S<br>RRID: N/A
PE/Cyanine7 anti-human CD4 Antibody (FCM)	Biolegend	317414<br>RRID: AB_571959
BD Horizon™ BV711 Mouse Anti-Human CD8 (FCM)	BD Biosciences	Cat# 563677<br>RRID: N/A
FITC anti-mouse/human CD44 Antibody (FCM)	Biolegend	Cat# 103006<br>RRID: AB_312957
PE/Dazzle™ 594 anti-human/mouse Granzyme B Recombinant Antibody (FCM)	Biolegend	Cat# 372216<br>RRID: AB_2728383
BD Horizon™ BV421 Mouse Anti-Human IFN-γ (FCM)	BD Biosciences	Cat# 562988<br>RRID: AB_2737934
PE anti-human CD279 (PD-1) Antibody (FCM)	Biolegend	Cat# 329906<br>RRID: AB_940483
PE anti-TCF1 (TCF7) Antibody (FCM)	Biolegend	Cat# 655207<br>RRID: AB_2728491
Alexa Fluor® 594 anti-TOX Antibody (FCM)	Biolegend	Cat# 682604<br>RRID: AB_2566786
Zombie Aqua™ Fixable Viability Kit (FCM)	Biolegend	Cat# 423102<br>RRID: N/A
Rabbit Polyclonal anti-SH3YL1 (IHC)	Aviva Systems Biology	Cat# ARP67810_P050<br>RRID: N/A
Rabbit Polyclonal anti-SH3YL1 (WB)	Abcam	Cat# AB154123<br>RRID: N/A
Peroxidase AffiniPure™ Goat Anti-Rabbit IgG (H+L)	Jackson ImmunoResearch	Cat# 111-035-003<br>RRID: AB_2313567
**Critical reagents**		
Cisplatin for Injection	Qilu-pharma	BP:EA4A019B
Caprylic/Capric Triglyceride	Yuanye Bio-Technology	Cat# HY-135087
Cholesterol-Water Soluble	Sigma-Aldrich	Cat# C4951
CD8 MicroBeads, human	Milteny Biotec	Cat# 130-045-201
Fetal bovine serum	PAN	Cat# ST30-3302
Newborn calf serum	Gibco	Cat# 26010074
ImmunoCult™-XF T Cell Expansion Medium	Stemcell Technolohies	Cat# 10981
ImmunoCult™ Human CD3/CD28/CD2 T Cell Activator	Stemcell Technolohies	Cat# 10970
Human IL-2 Recombinant Protein	Thermo Fisher	Cat# 200-02-100UG
McCoy's 5A	Procell	Cat# PM150710
DMEM basic (1X)	Gibco	Cat# C11995500BT
RPMI Medium 1640 basic (1X)	Gibco	Cat# C11875500BT
Lipofectamine™ 3000 Transfection Reagent	Invitrogen	Cat# L3000015
5-(N-ethyl-N-isopropyl)-amiloride (EIPA)	Selleck	Cat# HY-101840
BODIPY™ FL C16	Invitrogen	Cat# D3821
Dextran, Tetramethylrhodamine, 70000 MW	Invitrogen	Cat# D1818
**Critical commercial assays**		
Cell Counting Kit-8	Dojindo	Cat# CK04
Human CD4 Naïve T Cell Isolation Kit	Biolegend	Cat# 480041
**Experimental cells**		
Human Ovarian cancer cells: SKOV3, SKOV3/DDP	National Intrastructure of Cell Line Resource	3101HUMTCHu185 | http://www.cellresource.com.cn/fdetail.aspx?id=7339
Human Ovarian cancer cells: OVCAR3	National Intrastructure of Cell Line Resource	3101HUMTCHu228 | http://www.cellresource.com.cn/fdetail.aspx?id=7338
Human Ovarian cancer cells: Caov3	National Intrastructure of Cell Line Resource	3101HUMTCHu260 | http://www.cellresource.com.cn/fdetail.aspx?id=10308
Mouse Fat cells:3T3-L1	National Intrastructure of Cell Line Resource	1101MOU-PUMC000155 | http://www.cellresource.com.cn/fdetail.aspx?id=3997
Peripheral blood monoculear cell: $CD4^{+}$T cell,$CD8^{+}$T cell	This paper	N/A
**Nucleotide Sequences**		
siCD44-1	MiaoLing Plasmid	sense: CCACAAUGGCCCAGAUGGA<br>antisense: UCCAUCUGGGCCAUUGUGG
siCD44-2	MiaoLing Plasmid	sense: CCGCUUUGCAGGUGUAUUC<br>antisense: GAAUACACCUGCAAAGCGG
siCD44-3	MiaoLing Plasmid	sense: GCGCAGAUCGAUUUGAAUA<br>antisense: UAUUCAAAUCGAUCUGCGC
siCtrl	MiaoLing Plasmid	sense: UUCUCCGAACGUGUCACGU<br>antisense: ACGUGACACGUUCGGAGAAT
shSH3YL1-1	MiaoLing Plasmid	GAAATGGACCTGATAAGATCA
shSH3YL1-2	MiaoLing Plasmid	GAGCTGTTTGATTGAAAGGAA
shSH3YL1-3	MiaoLing Plasmid	GCTCTCAAAGTAACAGAAATG
**Deposited data**		
NCC HGSOC FFPE - Proteome	This paper	https://proteomecentral.proteomexchange.org/cgi/GetDataset?ID=PXD044439
Mendelian randomization - Total cholesterol:<br>Dataset: ieu-a-301	ieu open gwas project	https://gwas.mrcieu.ac.uk/datasets/ieu-a-301/
Mendelian randomization - Cisplatin drug response (IC50):<br>Study: GCST90011776	The NHGRI-EBI Catalog of human genome-wide association studies	https://www.ebi.ac.uk/gwas/studies/GCST90011776
SKOV3 - LncRNA/mRNA expression in SKOV3/SKOV3_DDP/IOSE80:<br>GSE104776	Gene Expression Omnibus	https://www.ncbi.nlm.nih.gov/geo/query/acc.cgi
Ascites - single cell sequence of ovarian cancer:<br>PRJCA005422	China National Center for Bioinformation<br>National Genomics Data Center	https://ngdc.cncb.ac.cn/bioproject/browse/PRJCA005422
PTRC HGSOC FFPE Discovery - Proteome:<br>PDC Study Identifier: PDC000360<br>Study ID: 97332d25-b8bb-4094-8bed-92290c729599	Clinical Proteomic Tumor Analysis Consortium	https://pdc.cancer.gov/pdc/study/PDC000360

Supplementary Table 25. Protein expression profiles

	HGSOC1	HGSOC2	HGSOC3	HGSOC4	HGSOC5	HGSOC6	HGSOC7	HGSOC8	HGSOC9	HGSOC10	HGSOC11	HGSOC12	HGSOC13	HGSOC14	HGSOC15	HGSOC16	HGSOC17	HGSOC18
NUDT4	0	11.97578693	12.74135743	14.21493537	14.42270617	0	0	13.78602415	13.3197836	0	12.59323757	12.95174386	12.41722582	0	13.97057743	12.1279043	13.2958366	14.01847595
RBM47	17.69789764	15.8712403	17.53988177	16.41825167	16.09331504	16.16658682	17.4371186	16.85110478	16.9464737	16.61406355	16.99587055	16.99672578	17.44792127	17.18553031	17.44152848	17.65548738	15.43081468	16.57888619
UBA6	17.2507804	16.77426587	17.54124636	16.82276142	17.58574194	16.9338537	17.3494605	16.94076084	17.1813866	17.4606085	17.87132661	17.78881211	18.50911003	17.40268513	16.68022333	17.90791781	16.97543675	17.25294401
ESYT2	17.99513821	15.77247074	17.19990669	17.30390669	16.98162224	17.77971214	18.039559	16.97232926	16.68625996	16.97915416	19.11686712	17.6309614	18.71655595	18.29000098	17.26140329	17.95944635	17.20668739	17.0980165
SHTN1	16.48633152	17.00623498	16.93018138	16.60415695	17.51716043	15.6748444	16.27966683	17.45386127	18.18450114	17.59454023	16.62612275	16.34338472	16.74812998	16.87664546	16.32831897	16.72681966	17.08825269	17.54497646
TMEM223	14.91054246	11.71784102	12.97052354	12.93939825	13.66933146	13.97425913	14.31097328	13.16440689	13.74739317	12.44179896	13.04849552	13.19207334	14.80295273	13.18108733	12.53850535	15.77484325	14.58187613	13.51658445
MEX3A	0	15.73306085	0	0	0	0	0	0	0	0	0	0	0	0	0	0	0	0
ILVBL	15.35016023	14.51662172	16.66882819	16.60378162	14.60289274	15.85459963	15.32274004	15.50101069	15.90695058	15.02926464	17.15396264	15.93501708	16.96949982	16.84053361	16.30781547	16.94999364	16.28167949	16.00005091
PLEKHG3	15.23229385	14.38479402	14.20266521	16.29221265	0	15.38894354	0	14.41048254	15.13672098	15.33468574	13.21897482	13.73761128	14.8906044	15.52952087	10.6009732	14.13443556	15.97208178	17.16549757
SH3PXD2B	16.11106433	16.37704037	13.99508135	15.50706776	16.048188	15.69309799	15.72677404	15.38079528	15.70546723	13.73633782	16.14423377	15.35739011	14.97548582	15.26112591	15.74377782	15.70620753	16.09890385	15.53886692
MACROD2	0	0	0	0	0	0	0	0	10.67286307	0	13.01202704	0	10.18154326	0	0	11.89512934	0	0
ZNF320	0	16.13933354	16.52030911	14.54254519	15.47632199	14.58781139	15.8008456	14.56543101	13.10138376	13.83074613	16.21327439	14.85488398	16.71470857	14.80573443	14.94514347	16.64481457	16.46235736	15.29247976
NBAS	13.44239102	14.35449583	13.67347795	14.09571814	13.76451285	14.59651452	14.42378056	14.18062702	13.75378364	13.82521647	14.52703463	14.40822555	14.49319678	14.5116307	14.67407328	13.95993111	13.77640994	14.55934044
DENND3	15.76209711	15.27143653	15.63640004	15.27187133	15.43032212	11.40035635	14.51851581	14.46802027	15.1611368	14.38060361	14.76175543	16.1638442	14.56313959	14.65116953	12.78727837	15.649303	15.73772535	15.6900534
VWA8	14.51687247	14.09270844	13.75588858	14.4022482	13.96832631	14.72075788	15.61835783	13.66564668	13.83233222	14.8423184	13.61646236	15.14222939	14.59882627	12.73383345	12.46411046	15.96248602	15.13991368	13.96757483
SBNO1	15.03567841	14.5821375	14.99877625	14.39305627	14.47049554	14.93393301	15.79396045	0	14.187864	15.02203774	14.34215444	14.89065161	15.15608524	13.09488534	0	15.20247182	14.73178285	13.68913987
WDR91	14.45912439	14.6319892	15.736073	15.36280426	15.16638654	15.36880483	15.55591399	15.43242835	15.54259762	15.20403988	15.71917676	14.65170757	14.80745005	15.91867392	15.05204327	14.73294527	15.51818261	15.70584845
VPS37C	15.71848292	14.14232648	14.89564304	15.31091089	14.97195509	0	0	15.55548809	15.50524702	13.62826686	14.31681791	14.55160407	14.38912149	14.98923389	15.15952847	16.17462249	14.99544568	15.2896103
PEDS1	12.89535589	13.67026368	0	13.10928676	0	0	13.88537986	0	0	0	11.96731123	0	14.51566567	14.14817864	0	13.09703533	14.66708186	0
PEDS1-UBE2V1	12.89535589	13.67026368	0	13.10928676	0	0	13.88537986	0	0	0	11.96731123	0	14.51566567	14.14817864	0	13.09703533	14.66708186	0
CNOT1	16.42194603	16.8649975	16.81691392	16.61344485	16.3082243	16.48794697	16.74581884	16.14282675	16.31188493	16.42363119	17.05886144	16.1144647	16.21985344	16.77189765	16.15696768	16.45835317	16.56231813	16.11545489
CCDC88B	14.65340809	13.70920753	14.42535025	15.20737201	16.20940443	15.25036001	16.3233301	16.86156535	16.28709812	16.11279487	14.67276997	14.79202033	13.98546205	15.37159029	15.11171818	13.55534778	14.98865167	18.04619562
MAP1LC3B2	16.23746309	16.26691905	16.27569173	16.01448298	16.75399924	15.29961567	16.36138589	16.64792465	16.63176057	16.99426503	16.97426736	16.09780775	14.97568322	15.95524984	15.61787781	14.97588312	15.84323916	16.78968507
PALM3	15.27092605	15.21839162	15.93645454	16.16293738	0	14.22658359	0	0	12.41615659	0	14.2929859	15.52759429	17.35146796	16.00678028	15.5119747	11.36775387	14.66972948	0
PGP	17.36161788	15.68699383	15.02381881	16.63584397	16.86972966	16.67024703	15.78260386	16.41830945	16.07768724	16.89230517	17.31368271	16.36900047	16.05562766	16.73371028	17.47169097	17.41176414	16.00896779	17.07816775
RIMOC1	14.79160767	15.89549809	14.96721166	15.16620155	13.0611401	15.45756387	0	15.27077923	0	15.38356838	15.11272886	15.19873159	0	15.22110665	15.95619392	14.88710475	14.6675681	14.58651181
TCAF2	14.47966557	13.79263165	13.20898565	14.67838469	15.3636625	15.1452563	15.96355909	14.42998923	14.03087993	15.09260747	13.2837121	14.60819187	14.97012029	16.38816789	13.10341973	13.09600716	14.7823241	15.07343489
TUBAL3	19.12797323	19.42393274	18.27722737	19.63002933	19.59314261	19.91216645	18.8143194	19.36362414	19.77529439	19.26709056	19.23326068	18.64212178	19.83522053	19.68672204	19.00728465	19.55917086	19.4238612	19.34059447
SMCHD1	14.98566894	15.11064759	15.29878459	14.8037827	15.74392652	15.67032452	15.86014772	14.6756641	14.68754377	16.19335044	14.10189843	16.0346042	14.20893714	14.73328277	15.47736771	15.20418962	14.90929928	14.75321389
UNC119B	14.96157599	13.82733127	15.89469955	15.79520012	15.19632652	0	16.13511399	15.04029357	15.64675041	15.30228415	15.53270501	14.65460413	15.40250884	16.10171525	15.46293805	14.2364109	15.37065442	15.39680727
METTL15	0	0	0	0	0	0	11.49365331	0	0	0	10.97630347	12.46442074	0	0	0	10.52194423	0	0
CCDC85C	12.45738702	11.85295796	14.29342492	13.19575021	13.17161143	0	0	0	12.37776894	0	13.91442811	12.45348353	15.32778383	13.52937836	12.53435098	14.77960164	12.03955499	11.28203979
RTL8C	14.9194824	14.94413832	13.87349387	13.55254211	13.89837295	14.12219544	13.69883849	14.36779745	13.50916392	13.34705927	15.71092096	15.02888793	14.18002158	14.74900324	15.26193479	14.39002013	13.95222758	14.65631073
KRTAP9-7	0	14.81838606	0	0	0	0	0	0	0	0	0	0	0	0	0	0	0	0
KRTAP16-1	10.42298648	16.34044303	0	13.71698541	13.37096099	0	0	10.38785654	0	0	0	0	0	13.25818098	0	0	14.07664029	0
C19orf38	0	0	13.01579088	14.84406352	12.86981107	0	0	14.48761951	14.19954041	14.13221212	13.46097139	0	0	13.08318064	14.66393275	0	13.58534998	0
NUDT19	16.12353495	13.70146511	15.04289891	13.04819599	15.20283048	14.46078733	15.693033	13.14925452	14.00037694	13.93321654	15.49907216	15.87754523	15.33916578	14.90805117	13.42434329	16.05219505	15.12599103	13.51861642
UPK3BL1	16.20492959	0	15.7302146	16.67271786	0	0	0	12.24807103	0	0	14.52242984	[illegible]	[illegible]	[illegible]	[illegible]	[illegible]	[illegible]	[illegible]
MYO1G	16.15782522	14.53963014	16.7986827	17.261536	17.63212459	17.69079728	18.18937275	17.27715868	17.86126348	18.18303288	15.46295639	[illegible]	[illegible]	[illegible]	[illegible]	[illegible]	[illegible]	[illegible]
FHAD1	0	16.636957	15.50744709	13.93991257	16.74878915	16.58045905	14.80718313	16.18131637	15.857551	15.64029194	13.71058102	[illegible]	[illegible]	[illegible]	[illegible]	[illegible]	[illegible]	[illegible]
SMIM1	12.29639156	0	10.9658984	12.31850544	10.62766613	16.23535472	0	12.01805871	13.05150287	0	12.62215697	[illegible]	[illegible]	[illegible]	[illegible]	[illegible]	[illegible]	[illegible]
GPR89B	16.11802983	15.02553702	14.01322367	14.44170526	15.89101825	0	13.76329201	14.61608034	14.02253511	0	15.29027019	[illegible]	[illegible]	[illegible]	[illegible]	[illegible]	[illegible]	[illegible]
GPR89A	16.11802983	15.02553702	14.01322367	14.44170526	15.89101825	0	13.76329201	14.61608034	14.02253511	0	15.29027019	[illegible]	[illegible]	[illegible]	[illegible]	[illegible]	[illegible]	[illegible]
PPP1R3G	15.84438454	0	15.47738821	15.19650533	13.46733415	14.38250622	14.01252719	0	0	0	16.35199424	[illegible]	[illegible]	[illegible]	[illegible]	[illegible]	[illegible]	[illegible]
[illegible]	[illegible]	[illegible]	[illegible]	[illegible]	[illegible]	[illegible]	[illegible]	[illegible]	[illegible]	[illegible]	[illegible]	[illegible]	[illegible]	[illegible]	[illegible]	[illegible]	[illegible]	[illegible]

CALU	19.7576186	19.34212812	19.40023529	18.62802094	18.39223592	18.60747831	18.70493105	18.4366442	18.84279746	18.25667805	19.59802012	20.16083716	18.93105118	18.70862737	18.8892414	18.02570699	18.07591047	18.61868739
AHCYL1	17.81374959	17.57635067	18.07816172	16.95972262	17.47466636	17.26279266	18.01600605	17.03114639	17.27721871	17.21487195	17.63912262	16.95197076	17.92862545	17.16022259	17.54625015	17.29834035	16.96576879	17.33145181
CD5L	14.89174969	14.58341038	15.62890382	17.57310951	16.87644987	17.94686283	18.03574032	17.31223735	17.33650247	18.53787659	14.70752733	16.85636416	16.49623903	16.73450518	16.38966705	13.77154518	15.20051057	16.23155446
TYROBP	16.57480554	16.42173921	16.5769064	17.87558232	16.95826569	15.69076436	16.33795517	17.96609553	17.33351367	17.80151911	14.61244312	15.59145067	14.34258283	16.52552702	16.16030416	15.46136916	16.25918308	17.31342074
NDUFS5	16.08007761	15.39728515	15.14924785	15.35070237	15.78204558	15.35395243	15.84555657	15.42235445	15.86389701	15.95852413	15.66468468	14.96869746	13.51521858	14.84602336	15.0948351	14.80633357	15.20103296	16.58536443
PDE6D	12.61843356	13.86651583	14.48102717	13.83669858	14.44644891	14.29249476	14.60560803	14.07127789	14.04523223	14.13112848	14.05085601	14.77394295	14.21569032	14.07151899	13.98462124	13.62992597	14.40859187	0
RAD21	17.24965905	17.45470744	17.41555727	17.17956123	17.33093629	17.44310113	17.37128744	16.67558937	16.78577651	17.25870117	17.3424811	17.30208943	17.44666888	17.49807872	17.40325029	17.67955033	17.06337369	16.83160936
AKR1B10	0	0	9.511555842	15.95089357	9.948458581	13.44241583	0	16.07352037	12.99457684	13.24565367	12.50032318	9.317777762	12.56138748	13.10740946	11.96060069	0	10.2445125	12.14028558
TIMM8A	17.08562563	18.55778901	17.53402818	16.48114965	17.60157959	17.44236128	17.958214	17.16099991	15.91761747	16.72398074	17.91744565	17.65304995	17.63061786	17.68191502	17.55546176	18.70669741	17.22748345	17.16023029
DHX16	14.77135597	15.14360446	14.24808247	14.74469159	14.56711008	14.9676538	14.26583978	13.91071857	14.19228589	14.46897982	15.1112285	14.31754428	14.27524359	14.75731869	14.49067096	15.1944321	14.28878189	14.27309426
ZNRD2	17.71768102	16.46034053	16.9040119	16.07581795	16.23223265	16.99746895	16.82083991	15.79562513	16.30880118	15.95317568	16.93928776	17.65792451	16.55416186	16.2494937	16.95416631	18.38063307	15.64026767	16.38751384
GMFG	17.51139508	16.91877818	16.90450573	17.11377141	18.03329545	17.73302505	16.98197536	17.91798842	17.45285567	18.72715558	15.82058402	17.02915914	15.82884172	16.95381359	16.81696517	14.9427833	17.51246826	17.71560962
BNIP3L	15.18850464	14.16829229	14.0170143	14.1620738	13.27148212	14.08559597	16.93548935	14.8586128	13.82242807	13.94147107	14.18096825	13.39416513	14.67261407	14.07800727	13.13999921	14.1561195	14.30166671	14.55572387
PCDH7	14.43351413	9.354367636	14.19602498	0	0	0	15.72493918	15.93977629	0	13.01848919	14.12316991	17.43616761	15.00748296	13.66705273	12.11137832	16.29153342	13.60348056	13.56358732
PRPSAP2	15.61842718	15.61529343	15.90004811	15.55862203	16.8196289	17.12537059	16.66258928	16.25747777	16.18977103	16.49855452	15.55953149	16.51501304	16.08930645	15.21590857	16.15564327	14.82869193	15.39314231	16.22574597
KLK8	0	0	0	0	0	0	0	0	0	0	0	0	13.96653212	0	0	10.97825772	0	0
SMARCA5	17.84741621	17.90873894	17.29180065	17.1510215	16.89921096	17.57906844	16.98682254	16.41443427	17.14697149	17.00517033	17.18837982	16.9789109	18.05271828	17.13504821	17.07611751	16.7411638	16.84808725	16.7248267
KIAA0513	0	0	0	12.92075137	14.00482909	0	0	13.90699167	0	0	0	0	0	13.21737395	13.37734731	0	0	0
SPAG9	15.75869849	15.46705087	15.39581285	15.34395869	15.37046792	15.09879909	15.23430816	15.93121597	16.07144158	15.50873391	15.27089497	15.60573925	14.40090327	15.69055295	15.89735267	15.43121154	15.89638341	15.90146888
KIF5C	0	13.81609225	0	10.22397178	10.64481325	0	13.0189827	0	11.28904048	0	15.38886604	13.52802385	13.08792344	9.658204726	14.25820759	15.02681451	13.89377395	0
SIPA1L3	14.75825646	14.20996212	14.19648672	12.42733753	12.68882122	15.82975747	15.59798569	14.28550579	14.34448962	15.14210908	14.06080212	13.70445355	15.47217927	11.57480323	0	14.12239152	13.39237088	13.246838
AQR	15.29519117	15.50390227	14.37583423	15.01587048	14.74310022	15.71950267	14.30373212	14.21722912	14.4724484	14.76152648	13.92032241	14.94369999	14.11728481	15.0217679	14.79120205	14.63254321	15.07365471	13.98881771
OPA1	17.77263585	17.10785368	16.9955718	16.43531876	17.24411772	16.03274243	15.55363726	15.98391351	15.75898683	16.27165451	15.57496175	16.62608988	17.14714948	16.69166355	17.03407974	16.18657976	17.07009277	16.45762761
ZEB2	0	11.91604573	13.85328185	15.27169212	14.25931988	0	0	14.50300353	15.24879274	15.28887691	0	0	0	15.12484829	13.483317	0	14.70679815	13.9778774
KIF1B	16.75922909	17.62224658	16.09648708	15.17229473	15.89663793	16.76274324	12.52032457	15.30905385	15.80221154	15.67932191	15.23274061	13.61286032	17.19770174	15.56774874	15.86659167	15.89124035	15.96001104	15.38780686
KDM1A	14.45602848	15.81860734	15.57187749	14.99515952	14.86536213	15.36499357	15.52557648	14.35514567	14.67346469	13.8694181	15.35693508	14.90905036	16.28344788	15.21992994	14.769938	15.65830019	14.95537029	14.7157288
FZD6	14.24101429	0	15.359042	0	0	0	14.44125314	0	0	0	12.49628256	13.21521689	15.24620797	13.49626634	13.75303144	14.59388324	13.17413099	13.04576166
PPL	19.01464507	18.8848231	18.96282118	18.64346583	17.00485343	16.41314574	17.48328268	17.06177923	16.04659782	16.33078329	20.19079314	18.31592927	19.91905944	19.6465619	17.9971258	20.55824413	19.91241811	18.3267903
GSDME	11.3395642	14.26611582	13.32562223	13.18852893	13.87822121	10.45402324	12.82757831	14.82565447	14.61571011	14.21375421	12.64428914	13.83725517	0	14.60839539	14.0927723	12.23402479	14.38337523	15.30020465
LY75	13.67139691	13.84887276	12.95616227	13.49712602	13.88025379	0	13.82640525	13.41254413	14.70709062	12.3184642	12.89620988	13.67380303	0	13.38766397	12.91959233	14.96642131	14.42384915	13.41474303
LY75-CD302	13.67139691	13.84887276	12.95616227	13.49712602	13.88025379	0	13.82640525	13.41254413	14.70709062	12.3184642	12.89620988	13.67380303	0	13.38766397	12.91959233	14.96642131	14.42384915	13.41474303
NRP2	15.20883179	15.96541705	15.84786568	13.99666131	14.77872667	0	15.97387023	14.68397817	14.63940927	15.95586236	16.31827607	15.1530533	14.58626497	14.82862095	13.63366954	14.58751773	14.5142775	14.41849167
MAN1A2	13.87178109	0	12.72856484	13.32867616	11.97891832	0	12.96776469	0	12.71099483	12.75760497	13.40255945	12.85719455	0	12.53982617	13.0371719	12.83989614	0	12.85618281
ACSL4	16.12707508	15.96550609	15.24719725	16.55903078	16.48869409	15.28681746	16.06937306	16.64275894	16.4391505	16.49405011	16.73930442	14.84903995	15.58971869	15.87008736	16.08809107	14.5453337	15.91354832	16.3573634
SNX3	19.04189422	18.5648755	18.84876877	19.43937133	18.37031785	17.92786529	18.15813212	19.52734528	19.71291716	19.45786354	17.80782677	17.50786564	17.63851579	18.52117016	18.28167227	17.91251798	18.64011759	19.24763653
DOK2	15.73529065	15.32525096	16.61908619	17.2256489	17.63896437	16.21769565	16.72546372	17.5823762	17.73026903	17.77218468	13.97688773	16.05220036	13.27414251	16.99542576	15.07864533	13.33532084	16.48170866	17.43131562
STX10	16.1676313	15.01588869	15.53850014	15.90861022	15.26600427	15.77363766	14.68366716	15.78813244	15.51300647	15.27094158	13.40674154	15.68041361	15.18275623	15.29887924	15.37068807	14.42549355	15.8696138	15.72444849
OGA	16.12371791	15.97224335	15.48614269	15.75089709	15.9203106	16.42410656	15.99331375	15.47355613	15.76023427	15.85712467	17.35664902	16.85625121	16.44897542	15.69132799	16.18668705	15.41602589	15.85174275	15.54132956
SORBS3	16.44702671	15.43021631	16.60493854	16.18528833	15.41483308	15.26757168	14.3780658	15.19761176	15.48453492	15.13600293	15.9284075	15.93827869	15.95049868	14.95254371	15.6977702	17.80621457	16.4987284	15.3055039
SYNCRIP	20.17014833	20.72999065	20.44827665	19.73137269	19.93110719	19.88397595	19.90378303	19.53614048	19.81476355	19.85044602	19.93541067	19.94172484	20.00162621	19.39625121	20.24370033	20.06096683	19.82238965	19.68709606
CDC40	13.90976814	13.81938683	15.15207188	14.16845575	14.61063615	0	0	13.32134764	13.9447881	14.38408955	14.44482874	13.90100386	13.47288156	14.33677091	14.17019984	14.47205517	13.84038378	14.15634837
NEMF	15.76235122	14.60588289	14.73883456	15.35788191	15.27878562	14.50093315	15.00054199	14.78401513	15.23796965	15.37106964	14.33300053	14.30030075	15.42401491	15.44334829	15.19252084	14.69888394	14.93122986	14.89978874
GMDS	17.63427165	16.57941596	16.97262605	17.30366733	16.96177485	17.42930151	16.75844883	16.79967154	15.95473164	16.39910043	17.34122643	17.04469344	16.25283316	16.18731326	16.84133649	17.15587778	15.72940832	16.93995829
NMT2	0	0	13.45533287	0	13.8579383	0	0	0	0	13.60023961	12.83157351	14.53905975	14.72727911	13.12352469	13.0768915	14.06181185	12.16993631	12.19731765
PLOD3	18.37885589	18.45902556	18.26263297	18.30936602	17.72684959	17.63489611	17.52243295	18.58533588	19.077008	18.31627573	18.39350096	18.30838014	17.04389255	17.52778338	17.58309574	17.0491323	17.42777984	18.14891764
EIF4E2	13.3992285	13.43616112	13.73563958	14.29858787	13.49794147	15.39014363	13.97088536	13.4582683	13.1994146	12.85904021	12.58277058	12.89345263	13.52749944	12.5865212	13.21457748	12.76130764	14.21516103	13.11797515
[illegible]	[illegible]	[illegible]	[illegible]	[illegible]	[illegible]	[illegible]	[illegible]	[illegible]	[illegible]	[illegible]	[illegible]	[illegible]	[illegible]	[illegible]	[illegible]	[illegible]	[illegible]	[illegible]

0	13.50016025	0	0	11.36567633	0	13.89759548	0	0	[illegible]
12.813631	12.80221263	15.13737065	0	13.7633909	13.94809217	0	0	12.83298328	[illegible]
14.13450858	17.26884729	15.39201431	17.77276851	17.54424991	17.28165615	15.95193129	17.17186611	14.98196839	[illegible]
14.30408222	14.75341917	14.90176311	14.09501062	13.93932606	14.87323757	14.88998292	0	0	[illegible]
0	13.4479446	14.10340276	15.0691357	14.75968362	13.35249569	14.40245302	13.35168173	14.75587259	[illegible]
14.91522602	13.64917667	14.95331576	15.11931811	14.37184788	15.14301896	14.96050666	14.33034837	14.61617908	[illegible]
18.72076957	18.60028499	18.14031788	19.92488806	19.11118471	18.83586653	18.85737682	18.54294966	19.36162671	[illegible]
16.11203855	13.82520861	15.6557316	14.90427611	16.56129342	14.92659651	16.3069403	15.10160674	15.98533827	[illegible]
16.24894153	16.5837066	16.84240089	18.09373993	16.75414577	16.39912534	16.96935947	17.1465244	16.07998617	[illegible]
12.0757388	12.93199113	15.71961756	16.06305566	12.23397624	14.06458194	15.39931131	14.20006255	0	[illegible]
15.34821162	16.35396185	16.86081959	15.28412485	15.31247613	16.58035151	16.1438358	16.24740763	15.28045787	[illegible]
18.2138294	19.21954404	18.15125923	18.74140814	18.71809227	18.85193667	19.4744245	18.51795875	18.62586544	[illegible]
15.7727802	15.96089432	15.166915	15.66862945	16.52746949	15.42812295	16.00314351	15.62017037	16.09778573	[illegible]
14.17487038	15.20667428	14.75257155	12.4557994	14.84301617	14.68763102	14.95800242	14.16397039	15.49120942	[illegible]
18.17860954	17.99848722	18.04729269	18.10811906	18.36370835	17.99721894	17.87282281	18.2019341	18.46951476	[illegible]
15.30106548	11.94962771	15.53756193	11.84042345	11.88519448	13.52483962	12.98824582	12.56631795	12.84648932	[illegible]
0	14.97686781	14.42623135	15.02180212	12.65136774	15.66876631	14.2873215	14.50071875	13.49863328	[illegible]
15.87929494	15.34069375	15.10643259	15.51794878	14.91899177	15.68536177	15.89253114	14.79568774	15.72185779	[illegible]
0	13.86069742	17.93857834	0	14.42670637	12.98651331	0	13.65220274	0	[illegible]
15.65184966	15.31629341	15.39498897	15.11708534	15.52593163	15.32275723	14.78011219	15.03108833	15.51028138	[illegible]
17.2136676	17.7758812	16.62673304	16.64928019	17.08268962	16.88669154	17.16320066	17.08200006	17.42362914	[illegible]
15.71498405	16.22311673	15.98413001	17.22473906	16.16291357	15.26861952	16.82929081	16.50299492	16.3866556	[illegible]
15.03829577	16.32648805	15.62733282	16.39379002	15.71032447	15.70965853	16.43866116	16.20148592	14.95433682	[illegible]
14.59249833	15.63424617	15.20930253	14.86171868	15.32004427	14.6998899	15.29112094	14.94861355	15.44993123	[illegible]
12.10572791	14.97413814	13.75220657	15.79089224	15.24347314	14.3278074	15.3373147	14.7034683	13.87568421	[illegible]
15.93484922	16.9700828	17.87038532	17.24760306	17.00329006	17.22398015	17.23939779	17.15556453	16.35425974	[illegible]
0	15.05744495	14.30492557	14.01825435	14.71219626	14.6172058	14.5825966	16.12885148	14.73703872	[illegible]
16.41083315	18.3396713	17.22847054	17.06492297	16.50407133	16.64915847	17.07576214	16.09370576	17.31032071	[illegible]
0	0	0	0	0	0	11.10720334	0	0	[illegible]
21.86628839	22.70350917	23.61694879	22.82506776	21.80967924	22.02845649	22.50757424	22.00214303	22.69431718	[illegible]
18.30352227	14.39258353	15.54470413	13.98737384	17.43688459	17.97298329	14.7239394	16.87694957	18.00659112	[illegible]
19.07967732	20.39452236	19.12958772	17.55878632	18.9952791	18.51167135	20.42268012	19.62592205	19.34002461	[illegible]
12.48223459	14.27071793	15.14662399	13.88314275	12.51183914	13.83722205	15.77278302	9.169170221	13.9813443	[illegible]
18.85851919	18.8771053	18.86405708	20.51230597	19.28360331	19.50349665	18.47919237	19.33617732	19.27874847	[illegible]
18.35717853	17.47097333	17.71208435	18.27941236	18.00297533	17.89398581	18.25998428	18.53907537	18.23416487	[illegible]
20.92762779	20.70208209	21.00933334	19.4391179	20.93367292	20.55568561	20.21026452	21.30315531	21.21393824	[illegible]
13.81390818	13.42612499	13.68691681	12.64286016	13.62504895	12.80841591	12.41437194	14.643448	14.2589997	[illegible]
18.18923746	18.72047902	18.65523394	18.68369318	17.87544593	18.93838666	19.14904344	18.33607609	18.40449558	[illegible]
18.44567176	18.42371877	18.48452767	17.66314198	18.29322114	18.10899847	17.66468685	17.79081818	17.63348175	[illegible]
20.34026196	15.90078944	17.99537667	17.75110872	18.93824046	19.11507573	15.83415998	19.1677495	18.47444146	[illegible]
18.44980142	18.82161771	17.7465911	17.36266664	18.16302525	17.27398052	16.36180459	17.48751537	17.82431075	[illegible]
18.01091459	18.9869547	18.00339301	18.70660004	17.65044591	18.42404016	17.94511435	16.78108632	18.37743111	[illegible]
18.16985208	19.58519752	18.49487511	18.19670432	18.26460968	18.21579588	18.38243907	18.3043087	18.49989989	[illegible]
0	16.45454731	15.20202874	14.13727105	13.37656063	14.19213325	16.29392077	12.35084479	14.2371138	[illegible]
21.49248536	22.30604945	21.66046102	23.51558098	22.39794758	21.72567673	22.97324542	21.35344731	21.82391422	[illegible]
16.27201704	17.71391045	16.86403365	17.23393632	17.17588711	18.61453709	18.30530502	17.63667639	16.91614954	[illegible]
20.69117984	18.1489952	19.51387274	19.87977299	19.00618618	20.97426797	18.97283705	19.69903184	19.32711593	[illegible]
16.01947841	14.89190976	15.3861193	14.57119042	15.57804105	15.41946956	13.18458089	14.38165384	14.90050975	[illegible]
20.89676236	20.07534008	20.27013504	18.43664699	19.40173629	20.31795271	16.73023119	18.37178807	19.7099499	[illegible]
19.77991589	18.32328749	19.1330638	17.26973338	17.72862109	18.4794787	14.96998342	17.24508407	18.99600585	[illegible]
17.06130757	15.03480282	15.34729141	15.04895526	14.44882423	15.83760108	15.41647452	14.29222543	15.16063177	[illegible]
15.80551432	14.74045009	16.44204141	14.95347394	14.75342764	15.44966341	14.79391759	15.26458992	15.30037267	[illegible]
13.00443514	14.49782821	14.50480529	12.71926869	13.38219378	0	0	13.80445957	13.56352122	[illegible]
21.87065783	19.05820235	21.03025256	18.56233199	18.3387081	19.10147193	18.02741503	20.21625267	18.25288079	[illegible]
16.75266913	15.77938032	16.97369343	16.78450865	16.02154257	16.55491047	16.32336268	15.98409193	15.59900307	[illegible]
15.60839956	12.83272509	15.64153804	14.31709956	13.98748834	15.8047902	12.55829902	14.70882772	13.17609628	[illegible]
18.97017077	18.434125	19.09740431	18.06071457	17.67669164	18.71475426	17.53158802	16.87352203	17.96524449	[illegible]
15.54189693	13.23156677	14.03024616	14.88714009	14.90990114	13.99030075	12.93392743	14.42701853	15.64650004	[illegible]
17.79993443	17.99741502	16.73647316	17.5276685	18.05906203	17.62113801	17.57764572	18.9418543	17.79345775	[illegible]

[illegible]

ANXA4	19.66406941	20.06051998	20.74034642	20.4954558	19.80776998	18.69726667	19.29101384	20.04734195	20.42016353	20.00863207	20.02739259	18.84347841	19.76713708	20.09862746	19.64592136	20.60780686	19.61734942	20.51179765
CNP	16.93360116	16.10836652	15.79922074	16.80448241	15.86564389	16.67043615	16.27616176	16.39765818	16.15415368	16.95649829	16.92073674	16.75501856	16.85889305	16.16555416	15.91440083	16.340623	17.12373022	16.65018884
HMOX1	16.84037744	16.62807202	17.41933877	18.92187988	17.865452	15.86069096	16.6047187	19.64301788	18.21848076	18.45695858	14.85638886	15.3866506	12.67497386	17.24854102	16.52349591	14.49871386	17.94952708	18.03814838
DLD	17.32138728	16.81905085	18.39864898	16.19043403	16.62135112	17.11884478	17.2059519	17.09865444	16.4253961	16.38090068	18.4946625	17.61375934	16.82253573	17.56415182	17.06311097	16.81983313	16.70003251	17.13489128
HNRNPA1	20.01572657	20.89334357	20.94856908	20.45334361	20.54344689	20.7614583	20.95536505	20.31704931	20.26890945	20.44943735	21.03491899	19.87040978	20.59914113	20.69595963	20.6944174	19.92931819	20.75068695	20.42100797
SNRPA1	18.73108852	19.06322357	18.99589962	18.4300094	18.52261038	18.75454941	18.58581093	18.02521663	18.11143945	18.20809814	18.76692475	18.49227839	18.47562274	18.72527399	18.59324783	18.8614653	18.56893676	18.07710126
CTSH	16.79679169	15.64202072	15.65296671	16.76830469	15.12317954	0	14.81361427	16.70504182	16.9056662	16.29944449	13.22465664	14.68594826	0	16.30556875	14.99625788	13.76012846	16.25731345	17.07477601
COX6C	18.8293635	18.40843885	18.18239587	18.17237562	18.23765856	18.43049275	18.99905714	18.72773686	18.09684735	18.45521734	18.26831626	18.41589531	17.63835205	18.12802784	17.67529345	17.62239584	18.63603566	18.62630237
TACSTD2	18.60939125	16.31938491	15.98780942	17.30483118	14.47407477	15.78755488	17.30423739	14.42612844	0	14.54902269	19.14654941	18.1295459	19.40117822	16.78708513	17.89314995	19.22272416	18.10363839	15.97526688
FGR	14.75119149	13.55491457	14.71071501	16.17791585	15.80975292	14.88011111	15.1492511	16.06408076	16.28101382	15.93607553	14.99271771	14.48514558	12.10575364	15.66746667	15.64390396	0	15.51510667	15.12725759
C1S	15.21597165	15.0654186	17.02222827	16.94763061	16.82719566	19.56848535	21.71867376	17.19320108	17.72767652	17.7207392	17.0613023	17.25030637	16.08667984	16.82206575	17.20266342	14.57905486	15.4408769	16.2579772
PARP1	18.36002456	19.8784465	18.55106316	18.0938666	18.69177983	19.33478829	19.08241838	18.05996938	18.45441502	18.69598851	18.65320787	19.29064297	19.02598083	18.70840299	18.50657429	18.10689755	18.51270768	18.28844059
IFIT2	19.63258414	19.85439808	19.63755156	19.54772721	20.29101833	20.31977161	18.05095496	19.09699594	20.69559236	20.40207719	18.51084275	17.42625631	19.43223221	15.87760074	19.5824133	18.50507786	19.39139229	20.14430411
IFIT1	16.31215756	16.15021677	17.62951771	17.3440664	16.56040281	14.9739851	18.65499148	16.97541226	17.72980172	18.91573289	16.42372774	17.92071021	15.99719126	16.52581696	17.41307044	16.60260565	15.79835511	18.33439692
ALOX5	18.47483826	17.16690381	17.90399775	20.32686119	18.6901955	22.61667615	19.96134927	20.2748604	19.81217858	20.79955624	15.43565589	14.84609903	12.9802999	17.76849844	20.10915187	15.9746215	20.30334445	17.09088451
UCHL1	17.52067215	19.13699393	15.15360414	15.89432302	17.7895029	18.17118046	16.33788754	15.40445868	19.48025955	18.24730346	16.4167974	14.49402178	14.93645023	19.60328529	16.21125083	18.7102306	16.70543486	15.6207192
LTA4H	19.73581474	19.63515581	19.01940527	19.72612677	20.37407713	18.85189265	17.90751519	20.41418946	19.80875075	19.76301314	18.28020807	18.23883591	17.10407785	19.4813836	19.68371427	17.96539908	19.73622096	18.74728724
ALDOC	18.81130736	18.12945676	15.99687987	17.73938652	17.37337842	17.36573613	17.28825343	17.04904736	17.29675311	17.59686526	17.3763786	16.39639155	17.18548207	16.95663476	17.86886999	17.82271484	17.76426003	17.15831475
C4A	16.92878319	0	16.11622385	17.22107581	17.6637798	17.99630452	0	16.94557832	16.18487035	16.81504266	16.56041657	17.60255083	15.9272716	16.95962172	16.13802941	0	17.46209695	16.91601
C4B	16.92878319	0	16.11622385	17.22107581	17.6637798	17.99630452	0	16.94557832	16.18487035	16.81504266	16.56041657	17.60255083	15.9272716	16.95962172	16.13802941	0	17.46209695	16.91601
C4B_2	17.90377016	17.42661783	19.05723124	19.70279834	18.59874439	21.18297804	20.49450772	19.93888949	19.74577289	19.83309092	18.40029146	20.05229225	18.23965534	19.20296466	19.5620762	17.05820218	19.1633894	19.00520768
H2AZ1	20.74339879	20.98811967	20.12912907	20.33956787	20.79884444	20.6373018	19.52423202	19.96467428	20.24391572	20.85759219	20.08535672	20.49526242	19.88191081	19.93758063	20.93344646	19.78543032	20.46634736	20.33678371
KRTAP2-4	0	16.96029462	0	0	0	0	0	14.46619324	0	0	0	0	0	0	0	0	0	0
KRTAP2-3	0	16.96029462	0	0	0	0	0	14.46619324	0	0	0	0	0	0	0	0	0	0
GSTT2	0	14.065964	0	0	0	0	0	0	0	0	0	0	0	0	15.48896089	0	12.69722308	0
TRAPPC2	13.58047285	0	13.95511731	0	14.13623329	0	0	15.28042629	0	0	13.72191149	0	14.87042888	15.32888567	14.05839642	13.70541904	14.3851512	0
SAA1	13.24639703	0	0	9.612138906	11.34965379	0	17.37994149	13.55174369	0	15.93339873	17.03346434	17.20039323	14.87547152	16.28640167	13.0340831	9.220585276	15.68008884	0
SAA2	0	0	0	0	0	0	16.11669011	0	0	14.37958011	17.44286316	17.69523063	15.96460845	13.81097498	13.8760841	0	15.00598461	0
SULT1A3	15.05645448	13.58074483	14.87588272	15.8041649	16.52108831	0	13.79118118	16.92237353	16.22043162	15.31726424	15.32286379	14.61925056	14.31972448	15.87533165	16.04114226	14.92632821	14.66655581	16.60755721
SULT1A4	15.05645448	13.58074483	14.87588272	15.8041649	16.52108831	0	13.79118118	16.92237353	16.22043162	15.31726424	15.32286379	14.61925056	14.31972448	15.87533165	16.04114226	14.92632821	14.66655581	16.60755721
CT45A5	0	17.70233008	0	0	15.67675899	0	16.40933202	13.78685757	17.44898802	15.00127503	0	0	10.75580842	14.9326673	0	14.02097468	0	0
CT45A6	0	17.70233008	0	0	15.67675899	0	16.40933202	13.78685757	17.44898802	15.00127503	0	0	10.75580842	14.9326673	0	14.02097468	0	0
CT45A7	0	17.70233008	0	0	15.67675899	0	16.40933202	13.78685757	17.44898802	15.00127503	0	0	10.75580842	14.9326673	0	14.02097468	0	0
HSPA1A	22.14356772	22.86190256	22.39197381	21.70259852	22.0991346	22.0328196	21.63739567	21.78865282	21.683974	21.99913734	22.85796989	21.93061184	22.05964796	22.15568311	22.65955315	22.86068615	21.53812652	21.96968715
HSPA1B	22.14356772	22.86190256	22.39197381	21.70259852	22.0991346	22.0328196	21.63739567	21.78865282	21.683974	21.99913734	22.85796989	21.93061184	22.05964796	22.15568311	22.65955315	22.86068615	21.53812652	21.96968715
U2AF1	18.50442623	18.63671577	18.52583166	18.08760868	18.23761071	18.7226114	18.12214961	18.11450204	17.81051682	18.12648172	18.3743997	18.41470227	18.11999323	18.42581212	18.13036522	18.01700648	18.36100069	17.75824477
LOC102724594	18.50442623	18.63671577	18.52583166	18.08760868	18.23761071	18.7226114	18.12214961	18.11450204	17.81051682	18.12648172	18.3743997	18.41470227	18.11999323	18.42581212	18.13036522	18.01700648	18.36100069	17.75824477
CBS	18.1347113	16.82213229	15.60583215	14.46228284	12.63557056	15.49419639	17.11758572	14.43344269	15.31699298	13.81339419	14.79963528	17.13168586	17.05901217	15.02976559	14.78072518	19.1925717	15.82220145	14.77026454
LOC102724560	18.1347113	16.82213229	15.60583215	14.46228284	12.63557056	15.49419639	17.11758572	14.43344269	15.31699298	13.81339419	14.79963528	17.13168586	17.05901217	15.02976559	14.78072518	19.1925717	15.82220145	14.77026454
[illegible]																		

[illegible]	17.45894786	17.119453	17.48066486	17.79324033
[illegible]	0	0	13.46571635	14.77901895
[illegible]	17.07101252	16.95304776	16.93297694	16.46619922
[illegible]	14.63115796	12.71480204	13.16632677	13.83559392
[illegible]	17.95651885	17.76695739	18.9693253	19.12791453
[illegible]	17.58651822	17.19115141	17.62256821	17.48834056
[illegible]	19.11769937	18.34908222	18.63209929	17.97086641
[illegible]	21.40316419	22.15346111	20.90624793	21.55120742
[illegible]	17.45891107	17.93118413	16.1988594	15.74771738
[illegible]	16.52404761	16.50271043	16.12985118	16.04524657
[illegible]	14.79251141	11.34694894	15.18151399	15.60734725
[illegible]	11.63103758	0	0	12.05550719
[illegible]	12.0084688	15.58501845	0	11.32299372
[illegible]	16.52519555	14.75774788	16.71923694	18.05215607
[illegible]	12.2991686	13.00384333	15.68888865	0
[illegible]	13.57745407	10.29356987	15.96665084	16.9333773
[illegible]	18.85376727	19.54443919	19.00004712	19.03481323
[illegible]	18.49643861	19.88241795	18.82720226	18.33099891
[illegible]	17.39095547	19.25075395	18.15267369	17.40102884
[illegible]	14.59529641	15.32456829	15.25538925	14.39266515
[illegible]	17.71984647	18.00344568	17.71153612	18.848836
[illegible]	19.62244522	18.69497781	21.03675097	20.76454565
[illegible]	15.02374336	15.82782433	15.81631437	15.41637761
[illegible]	17.42476691	17.66855642	16.67227847	17.11441673
[illegible]	17.80513365	17.33358109	17.54616158	17.76038444
[illegible]	18.84843899	19.32247393	19.45931404	18.34834833
[illegible]	18.0815465	18.10459332	16.96191047	17.86062724
[illegible]	0	12.20459398	0	13.02294023
[illegible]	17.55008305	18.55744384	16.81496241	16.68544594
[illegible]	17.69301481	17.95160257	17.72738943	18.61523044
[illegible]	21.60501068	21.9168319	20.85581737	20.77444525
[illegible]	15.08766354	14.64213236	15.84558959	14.67783231
[illegible]	19.62132529	21.12665785	20.53592271	20.29725439
[illegible]	19.62132529	21.12665785	20.53592271	20.29725439
[illegible]	14.91606277	14.6452745	15.26976191	15.20868149
[illegible]	16.94768384	15.95568981	16.64147839	16.18217837
[illegible]	22.06809827	22.46335449	22.85449653	22.43462377
[illegible]	18.31951516	14.40171663	15.63147583	14.71388581
[illegible]	15.49586747	15.09051774	15.2147494	16.36654047
[illegible]	18.05793274	17.22672498	18.29388078	18.65774803
[illegible]	18.68878793	18.27748634	19.46831666	18.46636092
[illegible]	0	11.25020371	14.88398033	15.4327719
[illegible]	16.76752558	17.54804543	18.622486	16.89529228
[illegible]	15.86787436	12.31593258	15.97974519	13.99651805
[illegible]	13.25890988	14.85484185	15.38676781	14.68448743
[illegible]	17.89961692	0	14.29582876	11.70493052
[illegible]	14.3558206	0	15.42836081	15.27169611
[illegible]	13.90064177	0	13.89197268	16.71881964
[illegible]	16.86835941	12.99875551	15.83049819	16.41400437
[illegible]	17.68358578	18.11644359	17.20087676	17.06283191
[illegible]	16.47742725	18.59710756	16.4516107	17.78115077
[illegible]	14.53197856	15.83466489	14.51388527	14.82964184
[illegible]	20.33461005	20.49751419	20.44165803	20.04753683
[illegible]	17.75798038	16.94210701	16.96967345	16.90369662
[illegible]	20.60321215	20.30471352	19.5897164	20.42133678
[illegible]	17.57106435	12.83596634	16.18222672	16.78124314
[illegible]	18.1917019	17.66820721	18.59684126	19.93233624
[illegible]	15.62116463	15.7402447	16.49213362	11.83149987
[illegible]	18.82769634	18.21229674	17.8353894	19.21826612
[illegible]	[illegible]	20.85276437	20.85791135	20.83878768
[illegible]	[illegible]	14.78696991	13.7874377	13.22931978
[illegible]	[illegible]	12.23918318	15.24657173	14.97310367
[illegible]	[illegible]	17.5635545	17.0739111	17.72528356
[illegible]	[illegible]	13.00097894	12.70112547	0
[illegible]	[illegible]	21.21737957	20.0401509	19.97396803
[illegible]	[illegible]	21.06518059	21.26080987	22.02328803
[illegible]	[illegible]	18.02613672	18.58759487	18.32497241
[illegible]				

DLST	20.22515874	19.70113584	20.95889888	19.77371452	19.23229283	19.50637078	19.67581224	19.31363953	19.13997424	19.27608407	19.81050437	19.34039929	19.98607065	20.47664449	19.93282209	19.58612532	19.70097007	19.60367863
GPX4	15.77109952	17.05471559	16.30497067	16.68287203	16.83892787	16.03803649	16.91230992	16.65566228	16.7712943	16.95254255	15.84210726	16.56994644	15.65177271	16.8716934	16.9454667	16.05172058	16.62713753	16.62582086
CFHR2	0	0	0	13.75013848	0	13.80893266	14.29423973	12.84444392	13.06918767	14.11923166	0	13.05324945	11.7365836	13.90083825	13.98203527	0	0	13.14467878
SRP14	17.91538141	17.93192027	17.17567106	17.91928426	18.28651349	18.13518493	17.87951987	17.53919443	17.34771706	17.68330392	17.66807928	17.62273777	18.18653412	17.55527194	18.04770539	17.62504014	17.51569599	18.05085552
NUP62	14.79581782	14.98466097	15.74863644	14.5708078	15.70290242	14.81438346	14.54022053	15.02382388	14.93877286	15.06206904	14.93049318	15.39817611	15.54640548	15.08651545	15.08250544	15.66023707	15.36055644	15.16096914
HPCAL1	17.27921283	17.32011164	17.18900047	17.40089468	17.42522666	17.60660695	18.17667171	17.33890935	17.81335631	17.43952288	17.49684817	17.00883157	16.34188701	17.66090935	17.58986804	16.68772925	17.31350364	17.65559983
FDFT1	13.46167253	0	15.45610333	13.38627945	0	0	14.12824375	12.08126383	0	0	12.58199378	12.82214532	16.66642752	12.47575903	13.79668953	15.0736936	13.50702238	0
TAGLN2	21.12836428	21.35441862	21.22749241	21.12714232	20.98143149	21.2239653	20.87192525	20.94462065	21.21868606	20.63565159	21.45400252	21.72735785	21.03630829	21.59346521	21.14055989	21.44732441	20.95765401	21.12800093
TALDO1	20.25474391	19.68007027	20.61461145	20.09313173	20.81355243	20.1681293	20.50389196	20.21952011	20.44076998	20.46142799	19.59253996	20.42553543	19.36036072	20.60062664	19.95753481	19.57099489	20.00221052	20.30300281
SNCA	14.91672605	0	14.30430536	0	17.30390665	17.13235224	0	15.49743562	15.36580833	14.71490421	0	0	14.35919995	14.03747138	0	0	14.53320424	16.53738052
ETFB	19.38890492	19.09369996	19.93621557	19.120647	19.13798925	18.84941904	19.80123964	19.14099012	19.12620394	19.57272532	19.92497375	19.34290966	19.08590939	19.06188122	18.4912482	20.48905856	18.87492264	19.4870883
RBMX	20.46536662	20.88140882	20.26191442	19.86125316	20.08460907	20.22816033	20.29173926	19.54863227	19.79587637	20.19715459	20.31537887	20.00325454	19.98748548	19.91558711	19.96705375	20.11573236	20.15620377	19.72855539
COIL	14.75557894	15.33917299	13.43219681	14.73534209	13.73783838	14.80261842	14.10214841	14.1304045	13.73913802	13.66449796	15.86200523	14.54460568	14.06648867	14.76888052	14.72011731	15.20437532	15.04653704	14.04899749
GGCX	14.93787153	15.39978432	14.38258181	15.48111835	13.6453021	14.09129171	14.63668203	14.86188732	14.07773141	14.2778694	14.85777327	15.29662905	16.01204819	14.83007482	13.69459594	16.28592312	14.59251361	0
ITGAE	16.09816785	15.24733563	16.31076822	13.72989899	14.69555509	14.78359628	16.23753136	14.48628572	13.74207933	14.611855	14.91617232	15.89510391	15.55979181	14.7547548	15.16594159	16.21891009	14.95085781	15.2904187
LIPA	16.47449885	15.37385405	16.19501198	18.60332933	17.06055213	0	17.21408607	17.98697336	18.00298837	17.78176451	14.16542995	15.86093086	12.30280694	16.65481803	15.09789694	14.00195687	18.01341963	17.23147932
ATP6V1A	18.82298737	18.16315596	19.11028114	19.01777147	18.51825022	17.94742728	18.28936729	18.86356575	19.04078184	18.84766481	18.89219204	17.80301582	18.524552	18.80541021	18.50236348	18.19043976	18.94867477	18.88474197
HSPA9	21.29596731	21.31598923	22.34710132	21.01671139	20.99985235	21.58565462	21.54838333	20.80001849	20.62568332	20.68762781	22.6197243	21.40497093	21.74482963	21.25759084	20.85537086	23.01540127	21.36491799	21.11517243
EIF4A3	17.64033762	18.46311996	18.31961314	17.57783101	17.94554939	18.47398165	18.47195399	17.25073313	17.54167353	17.75538283	18.15852248	17.80690406	17.92440768	17.90572422	18.34414472	18.17138191	18.1604265	17.62221384
RPS19	19.3761825	18.98110882	18.97724996	19.51536863	19.09910859	19.4666075	18.86914159	19.20078638	19.20126619	19.21159455	19.42985327	19.23572546	19.26635678	19.53960281	19.13297393	19.52926957	19.61598597	18.91887679
RPL3	18.36822655	19.00182444	19.32826773	18.82158356	18.71145735	19.21067788	19.69828404	19.00479652	19.11572205	18.69855366	19.58279975	18.69896923	18.93744647	19.1755226	19.35961633	19.15325915	19.01873979	18.40337122
COL18A1	14.14925839	15.02632233	15.71042253	14.76132712	16.62445502	16.12844782	16.66333779	14.44649599	17.42639786	14.78190165	14.61660549	15.57169294	15.25925019	14.53587574	14.08285623	14.90777409	15.23251344	13.40175686
DDOST	19.56992107	20.49358112	19.79410447	20.12977337	19.76995363	19.99013037	19.87104529	19.90960344	19.96458589	19.77207883	20.08367312	19.61635993	20.05428253	20.09785452	19.75000902	19.90537975	19.94459751	20.03728788
ANP32A	19.02855968	18.87886844	17.53082565	18.99881541	19.11908772	18.35269355	18.87828221	18.39667468	19.19628844	19.10935978	18.65654783	18.52764201	18.35265032	19.34035025	19.01131019	18.46767171	18.99720421	18.98638126
FEN1	17.74771256	17.99472296	18.0523853	17.72928	18.20175691	18.10520182	18.41232911	17.13144064	17.69406941	17.85945858	18.14564798	18.07257751	17.43947989	17.3109791	18.41868957	18.47131576	17.05491329	17.43272017
CAPG	18.80257967	18.08330499	18.95144925	19.52520715	18.97951504	17.80798493	18.85970596	19.21445327	19.21721358	19.47829922	19.94412634	19.64014496	16.84869142	19.31576899	18.5839689	20.19194085	19.59754749	19.50760993
CEACAM6	15.59251475	0	13.94920752	14.50482735	15.72596364	0	16.12700121	16.27636994	14.18624122	16.53769794	17.21521437	14.73309955	0	16.49652541	17.18161605	0	15.35341177	0
TXLNA	16.56054252	16.6420161	15.66665538	16.34119235	15.99185206	15.41144905	16.06557999	16.56536267	16.33945588	15.98659985	16.21867039	16.06634151	16.38132379	15.46932558	16.50038105	15.68694234	15.99212911	16.3607169
CCT6A	19.33749778	18.75023214	19.07438976	18.74814872	19.13271042	18.70574712	18.88622991	18.6680729	18.98116066	18.86730561	19.59492617	19.01918297	19.07839288	18.72586569	19.42763715	19.68835597	18.66213441	19.11333585
NNMT	19.32020752	16.8226213	18.55668134	17.91130484	15.92841554	18.87560238	18.09152949	18.02361664	17.83310714	14.9301683	16.495665	17.59753874	16.27284375	16.96425369	17.58838972	16.94913733	14.78480026	18.35834911
PSMB10	17.34439344	16.62575666	16.28318079	17.701232	17.18976485	16.73973282	16.73342398	17.60854417	17.79346261	18.0848799	15.40723236	17.43295625	14.2940724	16.17666456	16.02792658	15.57515351	16.50359964	17.64497769
PBXI	13.87307382	16.74957148	15.66879195	15.47533527	15.37026676	0	0	0	0	0	15.70773921	0	15.669522	15.4130707	14.66575282	15.12573349	15.92214274	0
RPL13A	19.03033424	19.13325653	18.81591661	18.99918793	18.51607679	18.49536991	18.24331478	18.59140296	18.70631199	18.69579337	18.76860051	18.75995288	18.78006673	18.72042537	19.13595958	18.92287862	18.79673784	18.00426625
ARL1	15.8509855	15.84260039	15.43295166	16.07214062	15.48745735	15.32828349	15.52991662	15.83248289	15.75933669	15.28914422	15.30710281	15.44929086	15.66326872	16.10354058	15.82034367	16.32104727	15.7567138	16.02708138
STAT3	18.40998195	17.59347672	16.95767021	17.30679446	17.88368338	17.57715634	17.26464089	18.05437214	17.69223972	17.79083418	17.41704589	17.70575281	17.47513584	17.38416572	17.06887109	17.22220603	17.24587457	17.78686684
USP6	16.14852996	15.44164397	16.10813135	16.24662957	15.53251312	0	16.14889236	15.81867247	15.65567211	15.35894598	15.94673899	15.35884956	14.84995146	15.82380405	15.68756609	16.69541459	15.90453981	15.48224383
PEX19	0	0	0	12.12538777	13.46425993	0	0	0	0	0	11.47732324	13.16200986	13.8515895	11.21383207	13.66886768	13.20460069	0	0
MDH1	21.1867972	20.72464239	21.01086765	20.50687315	21.16399095	20.78286167	19.92904562	20.56527934	20.5229683	20.71117647	21.42886362	20.48437622	20.79200304	20.12655009	20.6548414	21.58618719	20.88136662	20.59954526
MDH2	21.24597131	21.22138179	22.56414077	20.81217574	21.13824445	21.43684457	21.79314882	20.88048053	20.5540254	20.92331207	21.97272164	21.579161	21.91577892	21.5524517	20.34928541	21.73073195	21.49823786	20.99997816
RFC5	14.01751816	14.42958156	15.56392707	15.1434564	14.77548641	14.30293325	14.5524877	14.62356952	14.17389791	13.8727719	15.28542578	15.38724403	15.48142943	14.41687302	14.85750781	15.13521186	13.66227275	15.04581698
RFC3	14.88316448	15.89603531	16.17338954	14.5854489	14.94348833	15.30824694	15.19535118	14.20134996	13.95964144	14.52940041	15.5543352	15.72487248	15.82178692	14.40396979	15.53894055	15.87222528	13.90878254	13.63107234
[illegible]	[illegible]	[illegible]	[illegible]	[illegible]	[illegible]	[illegible]	[illegible]	[illegible]	[illegible]	[illegible]	[illegible]	[illegible]	[illegible]	[illegible]	[illegible]	[illegible]	[illegible]	[illegible]

MRPL12	19.39290355	18.52211071	17.4454428	17.51865087	17.61068481	18.38555332	17.7838715	16.72230527	17.27271777	17.39291534	16.61747139	18.29161098	17.7143755	17.00724324	16.85118725	19.11186588	18.42088183	17.4060734
THOP1	15.48353289	15.81658088	16.3310439	15.00303454	15.23555895	15.5818798	16.31593355	15.30592091	15.57291413	15.75516784	14.9952885	15.43502798	15.75844171	14.98976893	16.59733072	16.3620808	14.81620693	16.04023486
CAPZA1	17.907019	18.01782447	18.37213241	18.81076862	18.94059122	18.27941519	18.93290326	19.18747411	18.91713977	18.93706593	18.42982965	18.35290513	18.14751798	19.92889458	18.15252763	17.83203827	18.4132993	18.83104257
HMGA2	15.71553746	0	0	15.67872649	14.80901522	0	0	14.70215228	0	0	13.19892158	0	0	15.73378102	0	0	17.18099753	13.82930882
CRIP2	19.58043375	20.18770591	19.26754767	18.75676581	18.05856223	18.30179117	19.89897284	18.00158532	17.14729608	18.87109654	19.97603508	19.63115252	19.48795862	18.82803079	19.5599892	19.86739413	19.42862853	17.53844851
NUP98	16.92980867	16.96984785	17.03265013	16.386734	16.56015811	16.50708373	16.84463777	15.98424947	16.06625675	16.30252542	16.4525708	16.6563583	16.49792661	16.78766915	16.44396159	16.60285787	16.68848991	16.42570995
BLVRA	18.72207936	19.14204861	19.36529667	19.39631746	19.42247546	18.81411919	19.30345385	19.89061478	20.02709877	19.35145043	18.07924729	19.32707357	18.70487903	19.19848881	18.54962535	17.7285091	18.75117933	19.65463319
SLC25A1	17.99300634	17.88316513	16.43604901	16.40055831	17.5166472	14.68968185	17.4410117	16.97550349	16.44804306	17.39439034	16.64176689	17.11612484	18.24955807	17.446419	17.45159649	17.47593649	16.88155072	16.87918508
PPP5C	14.3373573	15.7653895	15.78342123	15.24336345	15.60120676	15.70275703	15.24490252	14.78353613	14.47942751	15.22201937	15.47184003	15.04273696	16.56763201	14.64428815	14.02552165	15.53940556	15.97990207	15.20677144
ARFIP2	14.35594477	13.19756617	13.32135727	0	13.48728756	15.45347763	14.61705196	0	12.80353815	0	12.65622135	14.18366809	13.72745394	15.28696083	12.05682274	14.07733617	14.20138571	12.96084148
ARFIP1	17.5679338	17.57329955	16.59139508	17.08677118	16.89902366	16.26176826	17.2449626	17.21865192	16.72081016	16.9012456	17.08150506	16.45872175	17.5303412	17.29476797	16.73733411	17.578583	16.86214422	17.53271333
NUBP1	17.30272733	16.64421805	16.78802776	17.80348898	16.77814452	16.37613732	17.05418331	17.4777679	17.82121032	17.98181035	15.9632256	17.01270108	15.48422156	16.51591142	17.88432836	17.63621039	17.40946112	16.53479064
ACLY	18.36170769	17.78292255	18.17665847	17.87052643	18.65682297	18.4204622	17.96138018	18.58472297	18.45538994	18.46104847	18.06565904	18.28251454	18.04787443	18.23638374	17.41403626	17.69848774	17.78449517	18.75811421
METAP1	16.44241937	16.48492735	16.02214326	14.95148625	16.57023501	17.02310289	16.83658755	16.5478251	16.34064187	16.20117628	15.73856535	16.15510326	16.85329099	15.89703723	15.64237732	16.4424724	15.13925328	16.48554313
SUCLG1	18.19171108	16.90456792	17.10351753	18.00860045	18.13280987	17.44040665	16.80051031	17.21010783	17.21610772	17.7545226	17.11485276	17.7686103	16.37031119	17.25504485	17.21801537	18.08607012	18.28799652	17.89462296
MVD	15.69589158	0	15.01676035	14.80115062	15.43287153	13.7888352	14.66386686	15.36774694	15.44980003	15.69231443	14.79745572	15.66567801	14.79622106	14.67866063	16.16819986	14.79246097	14.19705663	15.202839
PGGT1B	14.76162853	14.80289693	14.17844131	14.21773987	15.72240419	14.79421803	14.474888	12.08743403	14.86503177	14.26201597	13.96350679	13.3263738	14.58603905	13.73310327	13.44463908	15.44290086	14.8211157	14.9784797
RABGGTB	16.35203817	14.86223741	14.89701636	15.17792284	15.56110984	15.1240009	14.18009248	15.19512781	15.43772465	15.59054548	15.18497625	15.03867757	15.32399398	14.71351269	15.55480768	14.61726006	14.81231325	15.09321831
COPB1	19.3712784	18.82929362	18.8443308	19.18813454	18.76562098	18.6637342	18.26924299	19.17608578	18.95701965	18.92763683	18.8865977	18.65240091	18.51857236	18.80065141	18.66384504	18.36157585	18.97334458	18.87250468
COPA	19.98769997	19.51855916	19.97055242	19.84679995	19.51346341	19.48087819	19.52256034	19.79572579	19.77580145	19.47324757	19.94895213	19.46578335	19.01739481	19.9019922	19.5845069	19.46847017	19.55160782	19.58176398
CTSC	17.77266585	16.90922272	18.54738998	18.23199281	18.23393529	17.25738223	18.02168912	18.72161355	19.07879271	19.32902218	17.3793075	17.15026606	14.62921619	17.96244534	17.94256685	15.86919111	17.72604869	19.04862257
CRYBB1	0	12.83396713	0	15.53539381	0	0	0	15.10263301	14.81905592	14.30074425	0	12.05703089	0	13.98872048	0	10.08961729	13.93591004	0
AP3M2	0	14.67944821	0	0	0	0	0	0	11.98000715	0	11.70913315	0	14.48179438	13.02971691	13.30272732	11.7029712	13.53859996	0
AP2S1	17.18124691	17.39226566	17.63029224	17.48284365	17.68278504	17.04390056	17.56157906	17.78698626	17.95379734	17.39498047	16.23072632	16.60212852	17.11657844	17.05329685	16.5227951	16.28887058	17.20108263	17.9199536
HCCS	14.41202294	14.39958995	14.07532278	14.11701191	14.07308441	14.65266634	15.11706203	14.15523793	14.01031775	14.50681469	12.94204077	14.24044387	15.04084688	13.53102491	0	13.61837676	13.49038403	13.67107097
POLR2K	17.70573352	17.00675255	17.1489831	16.21335805	16.96966423	16.6937629	18.00194794	16.28173962	16.1153971	16.54582021	16.58599185	16.78765531	17.49179354	16.28065652	17.07840998	16.9453681	16.47787052	17.18254924
SLC16A1	14.80734274	0	15.53203744	0	12.86597156	0	0	15.15105898	14.60138837	14.76118314	14.74962617	14.97759935	0	13.83632605	15.43896744	14.65322318	13.55617192	15.39648706
IST1	16.45933556	16.59138298	16.35915383	16.14685484	16.71825264	16.12070774	16.89292787	17.041801	16.68621892	16.69811751	15.7106651	15.68866534	16.50043722	15.95427149	16.69616961	15.34518269	15.89503872	16.82312677
SEC24C	18.56772136	18.52774568	18.12745928	18.03234426	17.94583824	18.17985717	17.71935989	17.71554385	17.97729678	17.54528715	17.57238127	17.52075822	17.31505819	17.28417499	17.72241212	18.2543192	17.67621041	17.45906297
SUB1	20.41654861	20.3695027	19.86120857	20.10515945	20.60425029	20.32288125	19.35245043	19.50083835	19.74214524	20.70411483	18.86973446	19.61360874	20.48288167	20.71645474	20.81226869	19.65232213	19.78620152	20.12492069
CLNS1A	16.27003977	15.9703308	16.57332867	15.4602899	15.54829254	17.14158237	16.83386756	15.12895828	16.03006847	17.70646609	17.75596394	14.91302671	16.06849064	16.09065261	16.28569401	18.32712635	16.2320937	16.65326933
RARS1	18.30642555	18.1751979	17.68275943	17.92454623	18.14753847	18.18454169	18.27075485	17.97490844	17.62810129	17.79924249	18.33852408	18.537706	18.04782302	17.97674494	18.69841324	19.0986901	17.80559392	18.02887512
ATXN3	15.40223219	15.14614508	15.76201801	15.25919716	16.58698337	15.9239015	15.48826283	14.8489522	15.80687801	15.87836174	15.612111	15.06138702	15.57960954	15.72133944	15.66141235	15.82598659	15.52858038	15.5713476
YARS1	18.46628661	18.09913059	17.90138583	17.47495594	18.17035818	17.83274325	18.10396455	17.46042015	17.69831404	17.90654283	18.22931245	18.48020703	18.94021393	17.74978914	18.07245256	18.47759388	17.37382617	17.71774628
USP14	18.51675695	18.64236288	19.48514976	18.45488035	19.12397439	19.40853668	18.82306971	18.71344294	18.90331287	18.68944043	19.97234775	19.95519138	18.53642509	18.82254739	19.13968437	19.63167243	18.64077394	18.75501102
PRKAG1	16.51833788	16.27447058	15.42939065	16.12962246	15.6822173	15.70221814	15.45785502	15.35393709	15.88068713	16.02182177	15.57389504	14.90197775	15.75011363	15.47045396	15.69745641	14.83521118	15.59951813	15.68957821
HSPA2	16.12608764	16.66518116	16.86511893	16.89750879	16.30817013	16.91304662	16.39135479	16.47047059	16.92538593	16.74327109	17.77872997	16.70824489	17.63026301	16.60850627	16.82551919	15.70885319	16.4242264	16.03095636
BCAT1	16.96061562	14.79832129	16.2081891	15.53868046	17.08792311	16.8602905	15.84083765	18.36428978	16.24404904	16.7095195	17.40266002	13.58057509	20.656827	18.9840058	17.06582615	15.66724736	15.56358115	15.67815426
ATP1B3	17.1884334	16.74422481	18.22174759	17.16304706	16.91660406	17.28979433	18.68482758	17.22598038	17.39457508	17.40593478	18.77629547	17.52370767	17.26971283	16.77158085	17.94657766	18.45967888	17.26191069	17.75932328
RAD23A	18.64345901	18.25267814	17.66680922	17.13227646	18.40974881	17.87925268	17.48284992	17.61106319	17.49752516	18.05093371	17.46737905	18.28825934	18.05518163	16.71339606	17.52509397	17.43627476	17.50898604	17.51942234
RAD23B	19.12543207	19.79803487	18.96369966	18.50491292	18.9529766	18.43067548	19.50019709	19.12790414	18.74548117	18.85243902	19.254313	19.03502626	19.59641932	19.23108858	19.27505897	19.0717931	18.42588061	19.16076131
EPHB3	0	0	15.25287343	0	0	0	0	0	0	0	10.70590971	0	12.25101309	12.01818574	13.52367501	14.2607826	11.96617625	0
GALC	14.73178171	14.96577548	14.60808536	16.0606402	14.92294692	0	12.83948215	15.30684173	15.80059622	15.22331533	12.23050015	12.85220259	11.9743008	14.56884007	14.80478164	14.18761168	15.68092412	15.10071431
AK2	19.8281823	19.72324232	19.07538595	18.98211519	19.05755849	19.46320554	19.64070169	19.66342498	18.78496636	19.62196331	19.61801049	19.04652255	19.40335419	18.88889279	19.74633841	19.29294308	19.47346497	19.42277032
PRRX1	0	0	0	0	0	0	0	0	0	0	0	0	14.15330632	10.24401346	0	13.40065889	0	0
EMP3	12.60577553	12.81584627	12.32579472	14.73933007	0	11.49792856	0	14.38532107	14.72000487	14.17455437	10.19612842	12.18038269	0	14.27394595	12.56259414	0	14.13730826	13.76194165
ALDH18A1	17.31452986	17.1813866	17.34028247	16.37481981	15.90415959	17.52426527	17.26761996	16.2879692	16.17270157	15.59909515	17.91006369	17.76123499	16.45409505	17.16130029	17.25357543	17.0945183	16.71841205	15.89240107
NAPA	18.53578499	18.85210198	18.32788331	19.81551728	18.92108837	21.55122479	19.74512517	20.49573916	20.19529365	20.85947441	17.95071945	19.97966078	19.31439004	18.4467273	19.88453238	17.6283809	19.54974343	18.2250475
ADPRH	0	12.4077912	13.04337312	14.55432478	0	0	0	13.73501706	0	13.88588271	0	0	0	13.99238311	0	11.1051684	13.18657462	13.96224612
AIF1	16.53737637	17.52416278	17.17645654	18.4124321	17.84917485	15.94830297	16.86509438	18.47778507	18.25643417	18.77192142	15.14414569	16.67978032	14.3285829	16.92228586	16.25418654	13.78209859	17.32678235	18.54402133
EIF5	17.77703323	17.31913942	18.29096351	17.3140369	17.88730225	18.00982008	17.23803133	17.62167027	17.79590886	18.48404217	17.56650005	18.66303609	17.76724086	17.47551464	18.15102382	18.22500409	17.38484316	17.74881596
SLC12A2	16.32835472	17.20763132	16.8687409	14.56097613	13.47618786	0	16.51241123	13.11579633	13.42472527	13.75364145	14.67122736	14.09705768	13.40783631	12.28133254	14.04295734	17.15762742	13.54280883	15.20734877
PSMD4	16.69549785	17.24813677	17.13081766	16.62532793	17.41020069	17.25170199	17.14970139	17.39119363	17.04442931	16.97033799	16.83975701	17.07096652	17.2337032	16.70626899	16.88167524	17.0868673	16.8443376	17.14786014
DRG2	17.59040876	16.66885201	16.94643407	16.76578707	16.92478125	16.2431115	15.6973074	16.30021527	16.27079405	16.5296331	17.26053439	16.41616754	16.61366595	17.09757261	16.97029726	17.97207889	16.8143641	16.77319526
PLTP	15.99638608	16.94171526	17.08043322	17.72628022	19.81033187	16.43702383	17.52849799	18.0341392	18.38512394	18.21059124	15.80419425	20.26747524	14.68749252	18.45962648	16.02755356	14.47997079	19.21591613	18.49919603
CSE1L	19.27938146	20.18889667	19.63519169	18.83049615	19.51829321	19.846272	20.28278062	18.54521502	18.73747667	18.59551997	19.30484412	18.73024933	20.63589046	19.64243042	19.01638529	20.26475212	18.836359	18.74051267
TMBIM6	17.58532992	16.06942879	16.36924402	16.8404393	16.4424438	16.95616272	15.57878452	16.91718394	15.81289988	16.52360574	16.8945125	15.72473855	16.93057376	16.98575318	14.67521699	16.0037459	17.06986372	17.11680384
VCP	20.83344942	21.16462202	20.80400698	19.83640783	20.38165727	20.41003657	20.13939022	20.47444988	20.41002402	20.31609077	19.1748201	19.91837057	20.52641076	20.24565425	20.71188833	19.47749692	20.28106718	20.08105671
MFAP1	15.54242428	16.09413859	15.25977285	14.81554298	14.3750336	14.18492799	15.84749115	14.25037158	14.38954312	15.50764622	14.44236368	14.21507451	14.96364243	14.50297756	14.51370893	14.55116199	15.37761937	14.92561112
MFAP4	0	0	0	0	0	0	14.33138073	0	0	0	0	12.32087187	0	0	0	0	0	0
HADHB	20.32743628	19.15966889	19.81389889	19.77297496	19.94094486	19.73305464	20.02099367	19.74025593	19.85512825	19.98311773	19.39457521	19.6808898	19.07572575	19.84048463	19.31703812	20.59849861	20.09016396	19.84697177
MANF	16.95658416	17.23602865	16.66657661	17.43085066	17.34317623	17.36754646	17.39980388	17.64992954	17.51384275	17.65079977	17.21762082	18.35657233	17.14495132	17.7845411	17.92155614	17.03296181	17.2546998	17.00633378
NCKAP1L	15.16619619	15.28497698	16.22843927	16.77371935	16.71070282	15.60274235	16.58851976	17.20057792	17.42399094	17.20369256	14.42752232	15.40990299	11.69028918	16.41183317	15.40432887	13.01142554	16.15465821	16.98780942
AFDN	17.5543619	17.94702742	16.57423399	15.63402898	14.07750601	16.65414815	15.47643313	14.03403556	14.32449454	13.30427876	16.59853632	15.669003	16.96302667	16.30096163	15.47433389	16.65148292	15.94874204	14.53685784
NAP1L1	17.79313126	18.59143126	17.83659804	18.19815229	18.24333003	19.22213172	18.24164965	18.55410025	18.33069741	18.18798569	19.89203901	17.92747652	18.11482163	18.08971652	18.85606889	17.77795537	18.39739797	17.98595439
CASP7	15.37368548	15.75864187	17.26006054	16.19774433	15.49032123	15.80372398	16.87667619	15.30191994	16.23030371	16.39250413	16.4967436	15.39840917	16.50370554	16.31655348	15.81549822	16.92508371	15.72104984	15.64903016
CASP9	14.12594958	14.36796457	13.50035795	15.04739671	14.23988532	0	14.42573325	14.67215317	14.8436951	14.89270382	10.89428156	12.58618719	15.34100866	14.26410537	13.29258985	13.48461174	14.44039676	14.41771991
CASP6	13.60215038	13.63678446	14.40482947	13.55117256	14.24167656	15.61110992	15.87213574	14.14785238	13.86823496	14.27693684	13.84864204	14.63601408	13.34693163	14.28194361	13.4867172	13.54880427	14.54905408	14.45749336
ADK	17.58391957	16.38736961	17.87244493	16.90506064	17.05540243	17.06991582	17.47966273	16.70896924	16.66083299	16.80786636	17.08930467	17.75291306	15.85513157	16.79414027	16.58639635	19.41439195	16.87754392	18.00779876
ADAR	17.12717492	18.06972935	17.44905013	17.18082366	17.33245948	17.92581718	17.27468797	16.78291675	17.22521729	17.47913278	17.03553058	17.77494577	18.81438375	16.93735245	17.43110631	17.32559478	17.21258277	17.48088398
LAMB2	15.01075926	16.22502337	14.3905437	15.31343776	14.729455	15.41439066	14.59358945	15.87781864	14.76595639	12.7196114	15.90611835	15.86189773	16.2281963	14.39191352	16.05347386	17.7872116	16.79629781	14.55234076
CDH6	0	15.79417846	15.7367441	15.58353721	13.45123891	15.71550468	14.79815148	15.52968969	12.75644689	0	17.34405638	15.81706972	14.98318264	15.87477465	16.71113445	16.06702661	15.71885538	16.01989762
TPD52	18.47146213	15.78415985	16.98561249	16.33364728	16.62036348	18.09568583	17.38285763	15.0635381	15.46084943	16.52994997	17.99259402	16.61511132	17.99416618	16.87027215	17.5163993	19.18705556	17.68651164	17.32279476
SEC13	17.58573358	16.86604572	17.21229391	16.94749399	16.43973769	16.84099128	16.98478254	17.21515555	16.65887447	16.48050694	17.22080518	16.93632729	17.01068159	17.85720091	17.16312494	17.28851705	16.95829516	16.36863544
SNU13	18.18533673	19.28530231	19.1530219	17.73987762	18.432903	18.67909948	19.34070937	17.77062438	18.02794479	18.2835759	18.37871079	18.81633341	18.33845023	18.26029433	18.85953232	19.35129298	19.20760675	18.07994224
NPEPPS	19.74440571	19.19440023	18.69793697	18.98187096	18.25396359	18.53688925	18.11933758	18.39878922	17.99919171	18.23770246	19.21557773	19.14198892	19.62577717	19.05695121	18.72392472	18.85425529	18.47163057	18.37419523
GFER	14.73279698	14.89404087	13.79113208	14.27409402	13.22866459	15.07545646	14.18554196	11.7131442	12.06615424	14.41239683	13.47805994	15.09403563	14.35248586	14.5172657	14.43290605	14.72910497	13.66598968	13.97536984
HNRNPH2	16.75109268	17.63010123	16.93117376	16.74445632	16.87987234	17.51774819	16.83920561	16.95929024	16.82195251	17.14911463	17.03593878	16.43063809	17.06493514	17.87954034	17.37269691	17.33870415	17.28826991	16.92337927
OXCT1	17.06596384	16.32421297	15.54434567	16.32620247	16.95064043	15.69107726	15.24857663	15.21267211	14.93181399	15.87041185	17.91539043	17.35986938	14.20792185	15.20106872	17.18650359	15.84404906	16.06013655	16.39119284
EIF3B	18.33787733	18.58958303	18.13814413	18.45510932	18.38574736	18.49133384	18.64289915	18.24541619	18.24140933	18.37151877	18.0336858	18.25343116	19.99179827	18.57044129	18.08662527	18.43769684	18.67093379	18.10795805
FCGRT	16.96296818	18.01797752	18.03408646	18.99966692	17.54496608	15.54327165	17.95642818	19.00058789	19.69544454	18.70305329	14.77009029	16.32949595	14.29152246	18.5388449	16.73928587	16.61648894	17.7975987	18.25860772
BID	16.45428034	15.51882505	16.8222885	16.58476482	17.32219031	16.18146986	16.36898369	16.9866015	17.16185291	17.24047328	16.06295507	16.1267484	15.6686565	16.28478372	17.22373483	17.98879284	16.56410889	17.13447348
ATP5MF	18.27167645	18.32396857	19.34204414	18.54186905	18.53870308	19.21302158	19.77259749	18.73652621	18.61624527	18.72675803	19.4927533	18.05507751	19.15948089	19.04953883	18.68592228	19.23400973	19.30476629	19.05246507
RRP1	15.22156205	16.12473094	15.66068689	14.75270404	14.3836803	14.59548366	15.97944718	13.4416051	14.33209557	14.82236835	14.59201441	15.04229428	14.53866306	15.10919397	15.20734892	16.01615946	14.64416985	14.19592154
MARS1	19.1195426	19.61877621	18.86693676	18.29033165	18.32951798	19.00751033	18.54770309	17.88461781	17.72763175	18.37683257	19.09446417	18.76869863	18.23914908	18.51830257	18.76705209	18.88343934	18.46655896	17.86777908
ARPP19	18.06427109	17.47386922	17.72959741	17.69598299	17.25481108	19.02412733	17.9588403	17.44230483	17.21324931	16.99738694	17.53800713	17.23890776	16.87151725	17.22752238	17.13994614	17.7354266	17.65522542	16.86183641
AP1S2	15.18466502	15.81808809	16.39811765	16.88954073	16.96764527	16.97578964	17.25448731	17.37787615	17.48982334	17.36483919	14.55740001	15.69356065	14.02068837	16.18475763	16.15472372	13.33506754	16.02357671	17.20522007
ATP5F1E	18.29337735	19.98859776	18.16120955	18.64136307	17.18860801	17.55330586	18.53779296	18.01548158	17.38671948	18.06567432	18.70415505	17.45597783	18.73419474	17.92780373	18.04937248	18.45078524	18.67151073	17.82451286
ATP5ME	19.14175682	18.20048962	17.65730144	17.28782424	17.21652533	0	17.43429175	17.40397771	17.98175993	17.78027963	16.75897159	17.20086838	17.54774968	17.46689372	17.79661745	17.88169409	17.96112464	17.77203503
EIF6	19.15018423	19.73087029	18.47659221	18.66415222	18.96815445	18.8144968	18.64963686	18.38942338	18.02565276	18.46190505	17.84983104	18.77514527	18.8731126	18.42066291	18.88353433	19.30175113	18.23718822	18.26522958
CTBP2	15.25604308	16.66793521	16.69993416	15.80225425	15.90751043	16.68300401	16.65742684	15.69156133	16.25813871	15.51072747	17.20081077	16.33265314	16.52650127	16.36928503	16.2332955	16.80107008	17.29681821	16.04283296
NDUFA6	16.9115808	15.92110372	16.65766387	15.98294989	16.74945425	16.05223038	16.77770731	15.16831373	15.52017756	15.92279733	15.91296835	15.9625666	15.61896491	15.43716941	15.14899256	15.55389502	15.82191282	15.76172591
CLDN6	15.59141038	15.57858495	12.81415733	14.89515172	0	0	0	13.20500538	0	13.78649159	0	15.22021727	15.4842309	16.69133183	13.0545717	15.59203541	15.89759844	14.22376134
WDR4	13.39088595	13.75661078	14.23053987	13.38128154	12.90972712	0	0	11.84285489	0	0	0	12.5289827	13.09754432	13.32505328	13.49882134	14.42036967	13.65111795	0
TMEM33	15.63593041	16.16194023	17.78060885	15.57880329	16.54477971	16.97682796	17.02974437	16.90731668	17.01183583	15.933504	17.64491554	16.32232346	17.1651884	18.01341043	16.38139205	16.58595244	15.79744147	16.63233075
SYNJ2BP	15.80692372	15.43100783	15.66476594	15.60614447	14.65265376	15.29150025	14.98170306	15.51520847	15.67643706	15.37505057	15.90958394	14.54598879	15.56581634	15.03351677	15.72630226	15.67684399	15.41921994	16.28009788
GEMIN4	12.26633347	13.50688489	11.86585822	13.13970341	12.66290737	0	13.20143866	12.54960561	11.96785447	12.53245284	13.02997465	12.69151405	12.68012619	12.58773975	13.36677669	13.47947821	13.28552131	12.82329405
PCBP3	16.6324549	17.08945339	17.19737719	17.09600394	17.45273228	0	16.27195466	16.1095632	16.70593417	15.02614601	16.3473905	16.56254837	16.24201791	17.10858555	15.82472077	15.78995167	16.10816661	15.36239803
RAB25	15.92983918	15.37075005	14.47944675	15.17062316	0	14.78719934	13.84991511	14.48949545	14.43831305	0	15.68689202	16.44358125	15.91094232	12.30124582	14.46450873	16.06416548	14.70255667	12.7342058
CORO7	17.41305572	17.03958779	17.37043493	17.95787785	18.23712125	17.03473668	17.48768157	18.22289732	18.49342121	18.4452685	15.32522392	16.78724857	14.755441	17.43921925	16.99441369	15.33267568	17.3098406	18.35704976
NUP107	16.76753679	16.5267515	16.42812641	16.23831318	16.20741208	15.94086047	16.36926239	15.62092253	15.9043578	16.01018438	15.8558322	16.06713906	16.09690315	16.41241388	15.79066677	16.16734387	16.29453718	15.77868496
GSDMD	18.06366449	17.46625063	18.30173927	17.61667929	17.93795331	19.09140778	18.3328588	17.91435602	17.83234343	18.15282886	18.11648024	17.89196379	18.79734429	17.34617865	18.2269513	19.23251637	17.52378674	17.61346306
EEFSEC	15.98517155	16.56023428	16.39529525	15.87600295	15.62763574	16.1014325	16.04263511	15.79238441	16.29422753	16.10940601	15.91146313	15.65026237	15.0260141	15.63248563	15.47732039	16.12364924	15.56055383	16.16354972
EPPK1	19.16572435	18.4531974	19.17171267	16.08658785	16.8383929	16.61136009	15.75913723	15.2335224	14.81965677	15.85799599	18.44117397	13.97458841	20.56723324	18.46601483	18.7405629	20.11523388	18.6739685	16.9484683
MTPN	18.20831102	18.08460453	17.93512961	18.13197295	18.29193889	18.70583052	17.91943357	17.92589485	18.17700156	18.25021224	18.21454206	17.78763249	18.57923663	18.43189887	18.08018434	17.09417597	18.06548599	17.83641207
DEFA1	24.99652521	20.81154197	22.05835034	23.63712388	25.12884346	22.52810209	21.90968071	25.48223693	23.47142194	25.93431681	26.17258087	24.1861224	19.69648276	24.84278758	26.32818157	19.68814122	24.12220208	22.91118752
DEFA1B	24.99652521	20.81154197	22.05835034	23.63712388	25.12884346	22.52810209	21.90968071	25.48223693	23.47142194	25.93431681	26.17258087	24.1861224	19.69648276	24.84278758	26.32818157	19.68814122	24.12220208	22.91118752
GNG2	15.96539705	15.99205034	15.83375216	17.24579596	18.6696231	18.47732546	0	17.72175684	17.61811123	17.93412834	16.05433851	16.26187311	13.8068728	17.05178479	15.96462052	0	17.29104912	17.36203423
ARPC4	19.59340241	19.38699404	19.6004624	20.01884164	19.73874283	19.29272744	19.61388836	20.02852006	19.97493976	19.84847968	19.61879894	19.69639728	19.51730583	19.91788985	19.68832822	19.47845427	19.70882331	20.00587739
CD81	15.63002933	16.40453976	17.04934256	16.60361763	16.70875459	0	19.21740753	17.52227262	17.74435325	17.17422153	16.89986442	16.46723176	17.46023315	16.8060632	16.52192124	17.70266129	16.65105355	17.34831253
SEC61G	15.12485366	13.89698366	11.92857937	15.04864651	16.41860529	0	12.2750891	13.96140288	13.72230589	14.33423154	13.69915568	15.67329603	15.82319802	13.71745826	13.71555809	12.95118154	13.26229274	16.30866399
TPI1	20.85388299	22.55247117	21.80748277	21.18734059	21.19572484	21.86568249	21.68755552	21.04712558	21.189148	21.09241778	22.46483301	20.44237482	22.00532883	21.8385764	21.71189043	21.84708507	21.63184248	21.07687919
EIF3E	17.34232212	18.15055799	17.75003949	17.83416307	17.98391003	18.35332171	17.82188762	18.07542823	18.02184735	17.63761026	17.39064577	17.93272253	17.80747452	17.73397998	17.71581341	17.64677446	18.256938	17.78377311
KRTAP10-1	19.13472164	17.367768	19.27027308	0	18.93552118	0	0	0	18.68474231	0	19.02250328	18.8665097	19.07125088	18.27910908	19.61414108	19.08007631	19.01467094	0
SEC61B	17.59415042	18.3720871	17.4393797	17.6340057	17.80660075	18.73490732	17.39449881	18.24619985	17.95343228	17.86831947	17.92416146	17.39020189	17.08706136	17.58498291	17.14874344	16.72230683	17.98387725	17.69521852
PPP4C	16.33092823	16.60836741	15.68423122	16.73549054	16.62807458	16.65856911	15.61280957	16.48464014	16.18091545	16.55829855	16.29705773	15.99299632	15.83606009	15.94278053	16.24161809	16.35270083	15.9339575	16.45095032
GABARAPL2	13.96216968	14.1055668	14.65629283	15.45166679	15.52565533	13.40940894	13.64079799	15.95920108	15.34694989	15.93072101	14.14121314	13.92247389	12.74538146	14.35095806	13.91725242	12.22595803	14.68091726	16.00308259
UBE2G2	16.16878748	16.75518578	15.97727573	16.39834002	16.80831554	0	16.10170146	16.44410356	15.9791279	15.96456791	15.83576978	16.14099074	15.79566218	11.47938569	0	16.64864526	15.74257486	16.64061256
MYL6	19.06968937	18.956276	18.92689398	19.00953086	19.28860463	19.25578455	21.03738575	19.26014441	19.07602484	19.02990255	19.809298	18.6450406	18.87037356	18.90814337	18.68977154	18.97888385	19.27806372	19.46135741
ACTB	26.29850644	26.32384092	26.40074995	26.339903	26.62747655	26.27957488	26.34585134	26.49918642	26.70268866	26.74076711	26.0524989	26.49188734	26.26777182	26.16196836	25.96950817	25.64003416	26.30089057	26.36213165
EIF4A1	20.55450196	20.5553191	20.93049769	20.49286183	20.6239768	20.44647125	20.4483156	20.83423291	20.70033398	20.40358927	20.73364088	20.53333437	20.54222139	20.52867277	20.6022792	20.69460784	20.40243858	20.69914593
RPS20	20.2443724	20.56096658	20.21542043	20.19330517	20.22046616	20.32377854	20.60469416	20.01925883	19.85038526	19.96380327	20.38424294	20.2751812	20.14094738	20.05308037	20.27248969	20.26992823	20.22054173	20.16672487
PRPS1	14.62179892	14.73420073	13.58263427	14.55769647	15.61667032	16.21760898	15.04489235	14.84544889	15.1000601	15.32642181	14.37224814	14.86704755	14.39449697	14.58780956	14.57492885	13.1744893	14.75825758	15.27198215
PSMA6	18.68356876	18.07823731	18.05205107	18.16964843	18.55732743	18.407174	18.39647139	18.59592966	18.53379548	18.68301523	18.15196261	18.36375593	18.64285014	17.87658448	18.31893332	18.4743048	17.98657389	18.27817548
S100A10	22.03488844	20.90517489	20.10063331	21.17016667	21.2570694	19.94661166	20.29330465	21.43433502	21.56241375	21.05368431	20.69654958	21.10260658	21.6517402	20.87458003	20.46066261	20.25030015	20.55669951	21.30939877
CDC42	18.01723201	18.02116434	17.13894624	18.38350049	18.32008161	17.73092335	18.31848171	18.39533775	18.12339112	18.49250761	17.40734765	17.71272335	18.2792902	17.78664264	17.69228623	17.5398477	18.18063961	18.36361385
DSTN	18.06644317	18.12634638	17.25898418	18.31841869	18.07968058	18.03457633	16.90008008	17.51907254	17.27022616	16.98152861	17.38428194	18.24476812	18.10910818	17.77258571	18.5686753	18.73742881	17.84351378	17.83686141
GMFB	16.84450216	15.88296576	16.9178396	16.46707551	16.6463705	17.42929972	16.07173501	17.32436626	17.0834118	16.58408845	16.81616317	16.00439622	17.03485772	16.97797686	16.7226287	16.21744244	16.2187632	16.94746251
RAB8A	16.02522525	15.66172853	15.28453382	16.34311061	16.26525048	16.19594871	15.41457428	16.49856706	16.4805315	16.25913968	16.0163842	16.03553881	16.28372735	15.81377229	15.81929707	15.53796496	16.09791639	16.46712216
SPCS3	16.57609182	16.79992109	16.57579866	16.86061436	16.73777001	17.05050931	16.46190992	16.9861323	16.75294327	17.11606057	16.42400683	16.32849963	15.79956088	17.16175452	16.29233666	16.08323649	16.80077926	17.12185487
SRP54	16.62295823	16.35052123	15.91728138	16.24376932	16.33852803	16.1373153	15.80380565	16.25943741	15.9554	16.13518008	15.58615786	15.97012038	15.51615893	15.96430252	16.35935829	15.35035504	15.71948554	16.11625305
RAB4B	15.7079955	15.60237877	16.32099813	16.87335399	16.94294505	16.01792469	16.50935073	16.98264427	17.18964452	17.00882764	16.14104502	16.49495944	16.74723007	16.29262986	15.96894561	15.78588774	16.52485038	17.73489337
RAB2A	18.78068573	17.47019314	17.74296479	18.05863836	17.60262004	17.83228451	18.08415066	17.69328744	17.64963489	17.38488649	18.3120775	17.94304542	18.65057498	18.15033393	17.98676561	18.30789105	18.00934035	18.11292273
RAB5B	17.67158231	16.98779762	15.72575036	17.04664344	16.76244997	16.29054103	14.5741641	16.54223249	16.3347047	16.49194408	16.58941022	15.58020588	17.59516868	16.41010711	16.79731866	16.02590431	16.55540933	16.06901078
CKS1B	14.4766849	14.04884155	15.14323357	13.90925129	14.39245671	15.73588373	15.4257056	0	13.5229057	0	14.67767814	12.36299818	15.59646715	13.45171986	13.87575141	15.7299724	13.98912758	13.62953039
RAB10	17.33005959	17.71404182	18.58896403	18.91597162	18.57279921	18.57487735	18.23720923	18.96374798	18.86945632	18.52201813	19.23974856	18.35267214	18.90207779	18.30096665	18.2831627	18.95832703	17.87039501	19.09939469
UBE2D3	20.54958881	19.91095631	19.46422528	19.66353639	19.94591119	19.85004249	19.76926018	19.9971028	19.72174287	20.17660887	19.33689169	20.07131521	20.41905505	19.01066622	19.85697019	20.00115801	19.2111403	19.5081236
UBE2M	17.47541209	18.57881526	17.68739704	17.93633025	16.91387332	17.55197234	17.60054498	16.88240909	17.65160708	17.10066426	18.9634372	18.0090676	18.28127834	17.98748799	18.26047869	18.89614455	18.4623098	16.96678539
UBE2K	18.71276806	17.93771316	17.89187278	17.64610666	18.00891772	17.38770459	17.34483525	17.85071088	17.68629159	17.96253412	18.10222243	17.86200466	18.71480969	17.93851256	18.0730128	18.43253897	17.36143329	17.85612733
UBE2N	19.92098546	19.98095599	18.90309649	19.29808215	19.75958681	19.8508461	19.06061183	19.04362744	19.3277863	19.6209213	18.65209253	18.94415861	19.23711322	18.97654201	19.3126664	18.70913	19.4990191	19.28391665
RAB14	17.68319992	17.43744947	16.98157451	18.45826579	17.88935356	16.86230468	17.50825649	18.34933496	17.72009915	18.05507735	17.68035545	17.98060924	17.84920292	17.54195729	17.90312684	17.85472555	17.82279545	18.06265539
ACTR3	21.15492408	20.70879342	20.42095929	21.29828636	21.34540064	20.53278564	20.27206048	21.23844468	21.17589077	21.55133112	20.42132355	20.71521265	20.83021973	20.64717612	20.69031711	20.15053707	20.93392671	21.21471591
ACTR2	19.97764485	19.93909932	19.878217	20.29604225	20.66102454	19.89365269	19.55317787	20.49185615	20.52793773	20.71040498	19.51901757	20.07605633	19.98212721	19.94955299	19.85428098	19.77177769	20.16685081	20.36551989
ACTR1A	17.79752132	17.43276259	17.97537902	17.70208072	17.2729552	17.49685914	17.86853067	17.83143463	17.79409118	17.21751373	17.41304319	16.39987296	17.05991988	17.13100944	17.59106334	16.99429603	17.34399364	17.21846361
TMEM258	15.82885566	16.692185	16.1967747	16.96258337	16.53174125	16.04714988	0	16.65245485	16.68575946	16.58567869	16.40067049	15.65368481	16.27676919	16.34427057	16.48482265	16.09821381	16.67353089	16.45173582
COPS2	15.58221975	15.76320689	15.89913552	15.14674547	16.29089041	16.16263288	15.95684148	15.86759501	15.48641905	15.32321161	16.67467644	16.02760233	16.25019171	15.90331866	16.16033263	15.99475783	15.55865158	15.564544
ARF3	19.68193089	19.58692692	19.6080321	19.81297144	20.04131591	19.80052009	19.40810852	19.96526704	19.66720988	19.9209742	19.60205884	19.65050234	19.90643254	19.66422797	19.65154161	19.57932423	19.77723515	19.78080036
ABCE1	16.57464134	16.67045063	16.37765921	16.6585204	16.90611651	16.95550554	17.22740824	16.944721	16.69220576	17.12112116	16.77434551	16.80307417	16.91981816	16.93553935	16.84930077	16.98072969	16.50133695	17.09592751
RAP2B	14.99925082	16.5469087	17.23583632	17.51164542	16.94151289	17.23569289	16.74288607	18.10242333	17.82621785	17.77246107	16.44968292	16.751352	16.45121677	17.25249942	16.53522708	16.07720967	16.87827604	18.3284569
MAX	15.2230989	14.63371421	14.98795198	15.00207795	15.03692247	15.68256035	14.22852309	15.39622508	15.36744021	15.68297905	14.59250711	13.99329138	0	14.60204705	14.8034742	13.58444784	14.36437809	15.08972396
RPS3A	19.0962588	19.91594528	20.00640518	19.46173747	19.55165206	19.89752034	20.10877185	19.6158458	19.74233658	19.49989685	20.07008376	19.78758497	19.6508977	19.858947	19.84210659	20.05135301	19.62422485	19.44444332
RPL26	19.18825454	19.94096448	20.01370211	19.89349929	19.19768165	19.73941609	20.28546544	19.4691765	19.47177244	20.05518378	20.37930988	19.54223521	19.39681942	20.11815121	19.50038895	19.7626292	19.80102763	19.33061043
PSME3	17.24093119	17.33713663	16.21724376	17.1119459	17.08511501	16.86353834	16.7069858	17.10363214	17.08918682	16.70882699	17.24741473	17.51948701	17.14534105	17.20817957	16.69351503	17.1779485	16.78868628	17.24245905
RPL15	20.58162987	20.63422766	20.12131234	20.69119155	20.08523028	20.39551805	20.47328771	20.31616937	20.14071028	20.31044124	20.37940587	20.26965976	20.25599181	20.47387194	20.59603787	20.10306397	20.55821014	20.18024837
MAGOH	13.45598297	14.86370819	14.14831691	15.5777984	15.19282466	0	0	14.62719843	14.5212767	14.62704087	15.17199953	14.75974375	15.28240594	15.38768974	0	15.14914192	15.57145663	15.0159273
RPL27	19.91764396	20.15388705	19.59652715	19.32092061	19.96892435	19.99866583	19.880191	19.47151435	19.40719514	19.52995629	19.46500284	19.74679168	19.6854422	19.3434544	19.79969634	19.68914425	19.30730408	19.72059424
ATP6V0D1	18.60799351	18.44926	18.80921556	19.51696526	18.43103385	17.73109059	18.25105442	19.36012773	19.54983519	19.40801338	18.25239402	17.70443772	17.91273423	19.1205774	18.31351512	17.40703509	18.57835207	19.34859053
PCBD1	19.80031222	18.24041694	19.36782397	18.57449017	18.97137191	20.175603	18.94985743	17.89122802	18.37617773	17.4508737	18.92557829	19.28695688	18.6226473	17.97003302	18.61246111	20.26069303	18.42158513	18.15775616
RPL37A	15.97679554	16.26044308	15.60931978	17.04481932	16.50180099	16.93437963	17.01077377	16.70415948	16.17537929	16.89336338	16.40831937	16.74029114	16.74591893	17.12948081	17.59351821	16.32312487	16.50728521	16.18386099
RHOA	18.28801807	18.05432493	18.60978575	18.60924999	18.8412764	18.15213039	18.58071654	18.81975197	19.02583049	18.95676342	18.10031248	18.55715399	18.22411814	18.59592648	18.53469127	18.60163241	18.40613778	19.02398001
NAA20	15.16412833	15.64127475	16.02031772	15.25310983	14.77997979	15.74387764	15.0335255	13.46226698	14.94391179	14.92496161	15.17862302	14.72023643	15.09250521	14.970923	14.86226088	15.76565648	15.57099408	14.98611511
NCALD	0	0	0	0	0	13.98141668	14.11590536	0	0	12.264762	14.79338472	12.7294636	0	14.43205401	11.62998298	13.40162237	13.93254467	12.93910541
HSPE1	21.91564947	21.08773831	22.48620764	20.24001347	21.07160491	20.92588915	21.85755517	20.70443075	20.48514399	20.59833345	22.83970608	21.80694024	21.25727302	21.72261119	21.7587548	22.49903662	20.58384521	21.39980987
SEC61A1	16.72169198	17.17042573	16.69172446	17.4730678	16.696807	16.78030397	16.18339898	17.34309013	17.00275219	16.45684042	16.62341435	16.74144693	16.39219162	16.26991568	16.08783072	15.70161391	17.08455182	16.98178814
LYZ	20.69030315	18.39996825	19.34535435	20.89030752	21.44363853	20.13130404	20.12491832	21.23987964	20.86250749	21.46956109	21.56559975	19.30601773	17.10562945	21.04078586	21.74067118	15.45490645	21.27220803	19.53927172
VBP1	17.0829686	16.39425491	16.24728376	15.99997197	16.60873862	17.17831851	16.54026393	16.18705994	16.64219	16.38604021	16.51321254	16.76175928	16.94223	15.87797727	16.5929302	17.043288	16.09571862	16.41337278
B2M	18.71257381	18.35562424	18.5022468	18.76219801	18.78908803	18.65087973	19.44343986	19.37588526	19.35566094	19.59637154	17.78870997	19.00153808	16.13104179	18.42693154	17.81789942	18.52107345	18.11691518	19.18622217
DAD1	17.60512997	18.0908267	17.02513905	18.1427512	17.50879713	18.39534455	17.45937558	17.81218181	17.56943578	17.76391897	17.55783574	17.26671492	17.95750399	17.73385982	17.6645151	17.45304376	17.79116627	17.98777039
NPC2	18.62683027	16.90068645	15.71243432	18.29061134	17.33514716	17.21473295	16.67903943	18.24661855	18.18466457	17.97970639	14.6055774	16.30376166	15.17196381	16.94082203	17.32725042	15.40211507	17.11585363	17.86345194
COPZ1	17.99619977	17.48023712	17.13848071	17.52805378	17.47073706	15.50339243	16.8626925	17.62311368	17.3395638	17.90083033	17.52322788	17.13070535	17.36826506	17.30405046	17.25886704	16.84549417	17.25136798	17.37186436
SUMO2	19.59638224	19.50685367	18.34553045	18.56341334	18.86580283	20.51536141	20.33829298	19.25185712	18.84731078	18.91822424	18.63467392	18.79947571	19.15748125	17.79580256	19.61100047	18.38700693	18.83423696	19.23921071
UFM1	16.85792235	17.4319185	16.75541665	16.82736374	17.7185959	16.62628231	16.72235145	16.68179109	16.4109942	17.37832688	16.09253263	16.83586672	16.56530886	16.65139577	16.45205292	17.23244201	17.75349038	17.31557194
DCAF7	15.05168076	15.73644549	15.30864677	15.0478004	15.11068386	15.04376793	15.94407342	14.69724196	14.19073091	14.73707473	15.26543821	15.09082993	16.2790616	15.78900243	15.59098855	15.72251854	15.72796843	13.64565821
WDR5	15.59317533	16.31096967	15.86127986	14.89002301	15.8089787	17.46928496	16.11952202	14.76979215	15.23743756	15.50802335	15.95253136	15.46433398	16.13886298	15.68926584	15.53695729	15.73649308	15.4722601	15.17256973
AP1S1	15.81355859	15.06298108	14.65854383	14.86617508	15.04442647	15.38928131	15.08534067	14.97079998	15.14699883	14.40146184	15.40249036	14.88418711	16.0803758	14.85285693	15.58691742	15.20076932	15.19296353	14.89164911
NUTF2	18.06313698	17.60958479	18.76930901	17.38585332	18.60326004	18.44898525	18.06457125	18.18087555	18.11800653	18.32902505	18.64582046	18.06569535	18.93715513	17.85331374	18.1070507	17.48745293	17.44251353	18.66689426
HNRNPK	21.47286223	22.11767686	21.64922745	21.45023299	21.56774828	21.4200107	21.32882063	21.13884315	20.96967213	21.42156086	21.53069756	21.39393086	21.44669563	21.58246614	21.58511349	21.37923372	21.49905317	21.34454596
YWHAG	19.44979021	19.28541788	18.58231911	18.7260384	18.8869329	19.74761536	19.17505669	19.82050319	19.64114	19.15713843	18.46823623	18.68960231	19.67479473	18.46964542	18.00734899	17.78648421	18.54736409	18.61911799
RRAS2	14.4798931	0	13.06382229	0	12.24364658	12.30340479	13.65978254	0	12.93236355	0	12.64059998	13.81549617	13.21826979	11.47985475	11.41820701	13.71218293	12.72446493	12.46653701
TIMM10	16.00511891	0	16.78444884	15.86759416	16.0110013	16.80127734	16.78295593	15.15545722	15.54672493	15.10557015	17.26640139	16.06107465	14.8024291	15.80316465	16.06969163	16.18451037	15.53732838	14.58333818
RPS7	15.17005616	16.89483127	16.64258169	16.87674849	17.94053683	18.80828421	16.5917013	18.5198433	16.55304132	16.98510561	16.08999302	17.8417298	16.46377268	17.94529978	16.79166897	17.47179477	17.83769328	16.81347629
PPP1CA	19.73273126	19.53904282	19.56939661	19.83207421	19.52279713	19.76752669	19.52591766	19.58482514	19.60092977	19.44104235	19.77966696	19.44934868	20.0148313	19.98891434	19.383025	20.04377093	19.90136733	19.48279885
PPP1CB	18.02822107	18.12074428	17.85633386	17.57565984	17.33853809	18.2423897	17.63044411	17.0051316	16.9518486	17.52588529	18.18683845	17.52092927	18.00810834	17.96246961	17.61454698	18.22453197	17.66507053	17.25924429
PSMC1	18.24123751	18.38922826	18.82226574	18.19517738	18.72298374	18.25397368	18.33330551	18.31804087	18.64772773	18.47333751	18.56128165	18.52011052	18.45705392	18.4612215	18.60543193	18.51826655	18.054628	18.36641534
PSMC5	17.60880877	17.78988085	17.66019789	17.62769941	17.94795747	17.73145225	17.61128196	17.97314134	18.07171832	17.85643284	17.69829114	17.69868506	17.56591207	17.77828863	17.71030786	17.51986465	17.70181334	17.92707751
RPS8	19.94900567	19.96736234	19.68328582	20.24163787	19.71501999	20.07288773	20.28667479	19.87219963	19.86542009	19.85544215	19.83879018	19.88402515	19.83724467	20.1115547	20.33513039	20.09309416	19.89436151	19.3975429
RPS15A	20.22996603	20.30050338	20.39242067	20.02799287	20.08793622	20.10311937	20.5896452	20.14874716	20.24095332	19.98411758	20.33375919	20.33562751	20.04554771	19.96811709	20.59357942	20.2520144	19.9569393	20.05189795
RPS16	20.14715259	20.72433357	20.79594006	20.58066698	20.41679031	20.76278094	21.04804595	20.58491503	20.66853804	20.45360624	20.93918847	20.60481437	20.54814341	20.8775032	20.91190477	20.84735838	20.66570425	20.41151952
UBE2G1	14.25124604	13.09290067	14.45468774	14.73848804	14.55114627	14.57969861	13.55312654	14.91449362	14.23482737	14.41747978	14.63255974	13.85628361	13.86720869	14.81370311	14.44577341	13.50227581	14.37057088	12.68570279
UBE2H	15.15862278	13.63737511	15.3287145	14.72657439	13.93428873	16.23553017	15.52822874	14.29316205	14.50912187	14.38038259	16.20704963	15.05102518	16.98585088	16.16192624	15.01345814	14.76821189	14.52113901	13.50386261
YWHAE	17.77729796	18.10736635	16.94007365	17.76907117	17.72104014	17.86107233	17.00929831	17.17915511	18.24805076	18.09877602	19.34477837	17.92307717	18.17193801	17.76124889	17.97257196	18.32388737	18.22057435	17.80311803
RPS14	20.83451464	21.19957246	20.20105451	20.65612881	20.3980674	20.47897003	20.09923093	20.53676063	20.29430818	20.4787616	19.85149581	20.4664315	20.33283326	20.17001351	20.69843507	20.30787354	20.47898693	20.47551872
RPS23	18.28509978	18.66255038	17.99919584	18.67513416	18.62764899	18.09917442	18.61690147	18.57273549	18.26582176	18.03333041	18.58976885	18.67719713	18.16125846	19.03282582	18.68799317	18.36791806	18.69579995	18.56586281
RPS18	20.49727443	20.48570318	20.40474451	20.60939114	20.22557271	20.51156145	20.73542783	20.56941921	20.34791112	20.21272213	20.84378863	20.4658486	20.08338242	20.96306108	20.86677087	20.62377133	20.69489165	19.95178921
RPS29	17.78674726	18.36614876	18.15532735	17.82266953	17.96784541	18.1052234	18.38479455	18.13722101	17.92382798	17.16875061	18.98615661	18.23183289	17.57683145	18.5118307	18.18734044	18.30858345	18.235091	17.97199832
RPS13	20.79405944	20.66217614	20.67642059	20.31885445	20.13391968	20.53497771	20.88241385	20.28177583	20.38128682	20.28556153	20.43938705	20.53914816	20.40636501	20.25615411	20.90677474	20.64104769	20.20479873	20.16211289
RPS11	18.35103681	18.36971262	18.05537396	18.35162202	17.83929367	18.42691453	18.63770378	18.39109737	18.11394576	17.81664171	18.35507225	18.39608158	18.15139303	18.26356407	18.7223602	18.29941766	18.10878605	18.05457352
SNRPE	18.22118381	18.89760333	18.75255981	18.62195952	19.4686837	18.7918601	18.61526682	18.32108844	18.24613909	18.22796474	18.53183095	18.46118365	18.7883824	18.90221565	18.67407705	18.64903103	18.86688998	18.45970076
SNRPF	17.13212612	16.48976284	16.5069868	16.46490453	16.07180505	15.93426218	16.30911659	16.02382388	15.72198583	16.86910638	15.76748638	16.89105146	16.27129788	15.16449912	16.29352062	15.81683367	16.23329433	15.78650186
LSM3	18.45290656	19.43181638	18.55961107	18.72216078	18.31510118	18.5628014	18.5294072	18.01323832	18.31049116	18.40604693	19.49442629	18.21787045	19.49448367	18.59119703	18.48799516	18.2954213	19.19186557	18.30393303
LSM6	17.05210432	18.19544971	16.98498249	16.79956592	17.37109756	17.17476739	17.24580974	16.9370194	18.38107725	17.0557655	17.30050031	16.71515325	17.61693511	17.05239939	16.95456065	16.62658534	16.88775852	17.27408235
SNRPD1	19.47435741	19.68638629	19.31059767	19.07763395	19.16949506	19.00264782	19.45377878	18.43012722	18.72679524	19.08790985	18.65064137	19.12894601	19.26756882	18.65256667	19.25746194	18.74142337	18.91414312	18.87748195
SNRPD2	18.461568	19.47141437	19.27448181	18.46629358	18.82569765	18.95799968	19.63206677	18.34051257	18.52024147	18.53315349	18.86689536	18.97432636	19.12034674	18.70580237	18.74909176	18.79033806	18.69650171	18.73782539
SNRPD3	19.34339177	19.59851134	18.70102741	18.88314601	18.84919356	18.6350856	19.12990147	18.52035347	18.28425846	18.60805963	18.78565111	18.82808844	19.79684148	18.74673511	18.97231322	18.6657461	18.77098382	18.93421043
TMSB4X	20.16392341	20.14160965	19.46176569	19.94518063	20.96273462	21.4543913	18.89209835	20.69383127	20.33990504	20.35399468	19.39200212	19.85352506	20.14335332	18.97067125	19.51969527	17.65272585	19.71765017	20.56792347
ARF6	17.2217734	16.36168561	16.48034524	17.41937723	17.12791106	16.76245808	17.24519116	17.31877338	17.18829153	17.12806483	16.59154129	16.54041955	17.29474624	17.15155701	16.84585151	16.75717543	17.21105517	16.83519913
PSMC6	15.78260756	16.15503916	17.13189997	16.26726076	16.88133347	17.49155294	16.9391047	16.81743047	16.97415522	16.92570836	17.30818875	16.73254186	16.65402235	17.37178329	16.63827283	16.82489146	16.55622701	16.73865781
SELENOT	14.63046955	14.6130663	15.46876286	15.02544341	15.51871774	15.37818516	15.35816973	15.45070383	15.67639563	15.43642816	15.44533873	15.05292487	15.23530111	15.36165696	15.34613198	14.5152311	14.84092215	15.12957781
RPL7A	19.8080917	20.05626924	19.2482697	19.93773758	19.39356904	19.35197026	19.45106053	19.4750725	19.40400019	19.68361673	19.58994928	19.60956281	19.21276427	19.93795412	20.08616409	19.83950157	19.6424258	19.26384843
POLR2G	15.05495914	15.25934631	14.54224596	14.38651299	14.63344272	15.09257715	14.99440584	14.50802129	14.7409718	14.75828931	14.20632521	14.33846111	14.70637007	13.79998925	14.53593137	13.93480775	14.57160962	13.58409774
ETF1	17.65200159	17.69011758	17.99936464	18.11328036	17.75765084	17.86047408	18.2841298	17.7486913	17.84182662	17.70049147	18.32420225	17.78529112	17.52856476	18.0807398	18.31431356	18.46678347	17.59403375	18.07494191
CNBP	17.79056397	17.71887962	17.77890138	17.28729543	17.33253118	17.44478477	17.11461832	17.42846957	17.02892856	16.62126134	17.71983509	16.10512171	17.84883915	16.67614454	16.94810634	18.03537385	17.74747041	17.77613128
RPS4X	21.47101403	21.42695393	21.67431901	21.49961028	21.24758669	21.42786857	21.80115299	21.24389713	21.16222566	21.26339458	21.73790923	21.25684948	21.2056313	21.57257649	21.60907275	21.64129393	21.35295441	21.40512068
RHOB	14.34098951	14.87737392	13.94014752	14.62358828	14.07402677	14.56686013	14.09968715	13.9482035	15.14452693	12.90600271	13.48586505	13.92733255	13.49548568	15.74483846	14.37438099	13.31140589	14.7150268	14.22194756
RPL23A	20.38379353	20.41030629	19.4830286	20.14673134	19.96285692	19.70559442	19.41106301	19.82631184	19.77825493	20.3739792	19.47189025	19.89786816	19.7617111	19.61019919	20.27427406	19.80350977	19.91644114	19.68877042
RPS6	21.30644599	21.5776811	20.94971853	21.50004205	21.24815574	21.30552074	21.11996364	21.25607203	21.20002647	21.17215874	21.02290993	21.26819031	20.94694267	21.10866531	21.4207734	21.19172539	21.26528496	21.0859742
H4C9	26.78250328	26.72461356	26.17090673	26.10159119	26.61937449	26.77238647	26.21071831	26.04125508	25.93445828	26.72984869	25.7976269	26.70338402	25.57547877	25.56708512	25.65690499	25.70732216	25.23892102	25.84394041
H4C1	26.78250328	26.72461356	26.17090673	26.10159119	26.61937449	26.77238647	26.21071831	26.04125508	25.93445828	26.72984869	25.7976269	26.70338402	25.57547877	25.56708512	25.65690499	25.70732216	25.23892102	25.84394041
H4C4	26.78250328	26.72461356	26.17090673	26.10159119	26.61937449	26.77238647	26.21071831	26.04125508	25.93445828	26.72984869	25.7976269	26.70338402	25.57547877	25.56708512	25.65690499	25.70732216	25.23892102	25.84394041
H4C6	26.78250328	26.72461356	26.17090673	26.10159119	26.61937449	26.77238647	26.21071831	26.04125508	25.93445828	26.72984869	25.7976269	26.70338402	25.57547877	25.56708512	25.65690499	25.70732216	25.23892102	25.84394041
H4C12	26.78250328	26.72461356	26.17090673	26.10159119	26.61937449	26.77238647	26.21071831	26.04125508	25.93445828	26.72984869	25.7976269	26.70338402	25.57547877	25.56708512	25.65690499	25.70732216	25.23892102	25.84394041
H4C11	26.78250328	26.72461356	26.17090673	26.10159119	26.61937449	26.77238647	26.21071831	26.04125508	25.93445828	26.72984869	25.7976269	26.70338402	25.57547877	25.56708512	25.65690499	25.70732216	25.23892102	25.84394041
H4C3	26.78250328	26.72461356	26.17090673	26.10159119	26.61937449	26.77238647	26.21071831	26.04125508	25.93445828	26.72984869	25.7976269	26.70338402	25.57547877	25.56708512	25.65690499	25.70732216	25.23892102	25.84394041
H4C8	26.78250328	26.72461356	26.17090673	26.10159119	26.61937449	26.77238647	26.21071831	26.04125508	25.93445828	26.72984869	25.7976269	26.70338402	25.57547877	25.56708512	25.65690499	25.70732216	25.23892102	25.84394041
H4C2	26.78250328	26.72461356	26.17090673	26.10159119	26.61937449	26.77238647	26.21071831	26.04125508	25.93445828	26.72984869	25.7976269	26.70338402	25.57547877	25.56708512	25.65690499	25.70732216	25.23892102	25.84394041
H4C5	26.78250328	26.72461356	26.17090673	26.10159119	26.61937449	26.77238647	26.21071831	26.04125508	25.93445828	26.72984869	25.7976269	26.70338402	25.57547877	25.56708512	25.65690499	25.70732216	25.23892102	25.84394041
H4C13	26.78250328	26.72461356	26.17090673	26.10159119	26.61937449	26.77238647	26.21071831	26.04125508	25.93445828	26.72984869	25.7976269	26.70338402	25.57547877	25.56708512	25.65690499	25.70732216	25.23892102	25.84394041
H4C14	26.78250328	26.72461356	26.17090673	26.10159119	26.61937449	26.77238647	26.21071831	26.04125508	25.93445828	26.72984869	25.7976269	26.70338402	25.57547877	25.56708512	25.65690499	25.70732216	25.23892102	25.84394041
H4-16	26.78250328	26.72461356	26.17090673	26.10159119	26.61937449	26.77238647	26.21071831	26.04125508	25.93445828	26.72984869	25.7976269	26.70338402	25.57547877	25.56708512	25.65690499	25.70732216	25.23892102	25.84394041
H4C15	26.78250328	26.72461356	26.17090673	26.10159119	26.61937449	26.77238647	26.21071831	26.04125508	25.93445828	26.72984869	25.7976269	26.70338402	25.57547877	25.56708512	25.65690499	25.70732216	25.23892102	25.84394041
RAB1A	17.90107873	17.3039106	15.67342339	17.90550646	17.04175456	14.89997705	11.73290998	17.33636926	16.55381371	17.65401612	15.17687742	16.93101853	16.74233586	15.51644102	16.91191995	16.06926602	17.30098633	17.35122314
RAN	19.70110136	19.86977832	18.97100934	19.27987054	19.4604149	19.71049054	19.37892996	18.84866171	18.49409016	19.24122572	18.65092081	18.97904981	19.58864808	19.071464	19.53013387	18.95474353	19.14605525	19.1789988
RPL23	19.17884277	19.22856712	19.32950786	19.30745654	19.91594118	19.60922485	19.46831106	19.40696064	19.41516201	19.15449845	19.58922035	19.26277317	19.20684625	19.45453087	19.72538103	19.58131898	19.30110555	18.97634124
RAP1A	19.59696054	18.25312238	18.44100764	19.23109605	18.98232726	18.74667658	18.8673387	19.27953308	19.00885003	19.14008723	18.88997327	18.46646312	18.30036653	18.57682868	18.8059365	17.94809368	18.87329193	19.18295698
RPS15	18.95427824	18.00747492	16.81439246	17.28335377	18.03350853	17.66327187	17.57866568	17.61430197	17.22805608	17.98228696	17.07749265	17.59735187	16.84488732	16.89092032	17.31415158	17.63342798	17.53003156	17.3991436
RPS24	17.26489798	17.64223385	17.13819148	17.73128754	18.12778278	18.36919276	18.2121514	17.76503038	17.94063674	17.89357576	17.79156733	17.59900034	17.65146726	17.87233558	17.94719129	17.7823074	17.63349439	17.26076351
RPS25	21.15494308	21.18193476	20.90067263	20.79826077	20.83160753	20.9996123	21.04454804	20.57511045	20.97593733	21.15339493	20.71058113	20.94550715	20.79805406	20.47294649	21.02836779	20.8683661	20.46315215	20.75157389
RPS26	19.41513634	19.84310763	18.10984051	19.41126025	19.34457346	20.16831343	19.96861398	20.23903979	19.49000312	19.00382618	19.012952	18.04219373	18.47319516	18.49713248	19.44153696	18.54123029	19.20584141	19.4005832
RPS28	20.1345656	20.40530834	20.47821341	20.36540268	20.43462696	20.53562305	20.05494971	20.37498148	20.36708382	20.26508819	20.66507986	20.26410652	20.07429437	20.47574963	20.4445558	20.72609423	20.49924227	20.45390083
GNB1	18.0632188	18.00070943	16.61769535	18.0936155	17.67938044	16.24762523	21.39600826	17.32276053	16.90544366	17.95576855	16.6717434	16.46150905	17.94827784	16.62048397	16.86906551	16.88259361	17.93073986	17.7451304
POLR2L	16.53675755	15.66134189	15.49266102	15.07069225	15.45014714	14.78470074	15.98141564	14.85355009	14.75625548	15.44973682	15.23092929	15.29884737	15.1505222	15.13596646	15.19161742	15.55237313	15.09123424	15.40417227
RBX1	17.1762281	16.12890732	16.36303187	16.56380747	16.45694154	16.65429404	16.67609794	15.53028928	0	15.62511318	16.9973909	16.09364194	16.43904724	16.03223158	14.64893561	16.7302201	16.02167913	16.56521146
GNB2	18.96449238	18.46415185	18.47739303	18.35941151	18.58605464	18.45826667	18.90243501	18.99158863	18.90157853	19.04397163	18.55025806	18.69535777	19.57852127	18.93021162	18.89932908	18.38254672	19.38058247	18.53337774
RPL30	19.85011509	19.71591215	19.32743642	20.0711065	19.68674565	19.79600821	20.27846761	19.66558694	19.52007478	19.5106064	19.856691	19.84250573	19.62287202	20.08745782	20.13454712	19.58782798	19.69198424	19.62775684
RPL39	18.3264406	17.88828999	19.17653507	18.47156942	18.41627838	18.64005877	19.04191644	18.42400735	18.89047313	18.49076729	18.445365	18.76835558	18.58905984	18.37419311	18.86300296	18.80543038	18.15074979	18.14269138
RPL31	19.61030621	19.89814476	19.31926073	19.8404248	19.25282594	19.52380648	19.46975073	19.49696285	19.28265253	19.42328111	19.25853929	19.49230724	19.32343896	19.50258855	19.65655088	19.43617282	19.6701422	19.3776281
RPL10A	19.64563579	19.79698443	19.71146523	19.94162885	19.78947076	20.57817883	20.0052644	20.08162451	19.7873548	20.2200553	20.13194894	19.69115598	19.60761353	20.09756586	20.19910901	20.0811593	19.99770203	19.63635003
RPL32	18.71956617	19.01815976	18.41217907	19.0523702	18.48439687	18.76923374	19.31318516	18.81289508	18.45998686	18.75844161	18.97957644	18.58598383	18.49401091	19.06760521	18.83761879	18.63089943	19.0534132	18.6116451
RPL11	19.23646783	19.10449478	18.97984173	19.17084027	18.92067669	19.57808275	19.36080235	19.34899581	19.96862364	19.02357891	19.06176012	18.96506161	18.99257622	19.12143155	19.40095485	19.27729123	19.01659884	18.71969317
RPL8	21.17615351	21.1199122	20.86426588	21.3028535	20.61898115	21.01127999	21.19409704	20.90420195	20.77618561	20.8669823	21.10194554	20.92700298	20.82186067	21.02934301	21.14974805	20.92630839	21.18139858	20.97531543
PPIA	22.86656734	22.74777402	23.78982942	22.77316356	22.90580537	23.30864247	22.80453155	22.55477935	23.01374112	22.8204916	23.30216211	23.34266475	23.46172498	23.33928569	22.93527343	23.81180839	23.23979269	22.77991629
FKBP1A	17.05693484	18.3518886	17.81464573	17.60204773	17.96496558	17.87315288	17.97481472	18.26603774	17.92186781	18.19530657	17.62331332	17.42312951	17.26554938	17.17858234	18.5309854	17.65008375	17.19009684	18.04830144
RPS27A	22.77308194	23.17167755	23.00539206	22.84285451	23.24565483	23.403005	23.20138421	22.97585776	23.19359469	23.27799422	23.01017379	23.06777981	23.54192835	23.10020675	23.21392712	23.12428873	23.00592312	23.29128623
GRB2	18.78914157	19.06446303	18.88749247	20.12996025	19.17702346	18.41467749	19.11891811	20.11684729	19.97383923	20.1709052	17.33082034	18.19448214	17.87829428	19.41729485	18.7440863	17.82103082	19.45937696	19.75388257
TRA2B	17.74624152	17.85384654	18.14496936	17.6387259	18.04017532	18.30846875	18.66286044	17.5741105	17.71023386	17.95992112	18.14922908	17.86803785	18.20128429	17.64521105	17.95409574	17.58716575	17.83529119	17.87632021
RAC1	20.03227531	19.30267532	19.77131929	20.17594679	20.49065725	20.12609284	19.86595646	20.5637807	20.29135607	20.69296662	19.4244954	20.02355116	19.49368568	20.01144942	19.76794155	19.55706223	19.96307352	20.32969165
AP2B1	17.90070672	17.57187622	16.9908914	17.7676559	17.97013328	17.66076609	16.73362951	17.93079589	17.52935596	17.79301007	17.30497123	17.4849942	17.83056134	17.48381934	17.40295427	17.25511302	18.10774914	17.80738254
VAMP2	18.65291093	18.36028396	18.23353308	18.82479379	18.54199804	18.12181457	17.52807668	18.36530415	18.19160672	18.33551085	17.46730155	17.40315366	19.03273419	17.91777178	17.87769687	17.76469638	18.03123593	17.79263324
GNAS	17.84539989	17.13347542	17.19628424	17.1797313	16.89563445	17.51813662	17.64856627	16.9757056	17.16877172	17.19668861	17.76517166	16.91607316	19.1260889	17.35989564	17.56954703	17.43912082	18.13999874	17.16901772
PPP3R1	16.01939272	13.8291249	13.98121362	14.86569458	15.89511779	14.66398692	15.29076178	14.81212382	14.51128299	15.02500756	15.09443196	13.45360146	14.68375603	14.78476373	14.1389995	14.57187922	14.3109278	16.15787748
YWHAZ	21.11075553	19.52818175	20.03500662	19.5370002	19.8328401	19.73220159	19.99862305	19.77785938	19.05467652	19.82913235	19.81621875	20.50023612	21.0899914	20.12622064	19.90836788	20.68203326	20.34852191	20.22917366
PPP2R2A	17.66895162	16.42315657	16.9770298	17.13491869	16.78997873	16.6988191	16.22007361	16.83374887	16.50128604	16.57704302	15.32642126	14.19058067	16.4842968	16.45795406	16.86849516	17.08242748	17.31160434	16.64405342
SUMO1	19.1903965	18.32430431	19.42483558	18.1816074	19.14044895	18.32405581	19.68202876	17.15582437	18.71420841	18.42475346	19.57301784	19.69072898	19.08079557	17.73156049	18.86179155	19.31123139	18.1028835	19.85341594
DYNLL1	14.96607889	15.0514106	14.83867536	14.71887398	16.21317751	0	13.05297858	15.46456656	15.60813241	14.4888403	15.6083127	13.43088319	15.82898637	15.46096651	12.94592324	15.20031285	16.08397073	15.59961325
RPL38	19.07537991	17.55400443	17.98130388	17.93851947	18.18758662	18.45049467	19.14678618	17.94797414	17.92511432	18.31725885	18.14282768	18.36452891	18.46826109	18.00678764	18.78717891	18.40524718	18.152997	18.2062708
SKP1	18.77541587	18.83084118	18.00572939	18.65142555	18.94074229	18.77278342	18.36790181	18.62913246	18.60919633	18.40945037	18.91799161	18.3938039	18.44649649	18.68777004	18.94027255	19.02486591	18.6205597	18.52394619
GNG5	17.39397458	17.5413851	16.88631821	18.55782943	18.36612261	0	16.30617283	18.0234119	18.43871077	18.59957794	16.70803877	17.28459327	18.6267796	16.78693054	16.18005422	15.63173237	18.12663422	18.88674843
RPS21	18.94737413	19.21868635	19.50700471	19.03116827	19.19411884	19.32272436	19.83375254	19.33142693	19.11489199	19.27301541	19.52794668	19.16433323	19.30668281	19.05845225	19.36275411	19.46090804	19.27862896	19.26821687
EIF5A	19.80362999	19.4159015	19.75591597	20.13303322	20.03425173	20.35823964	20.04646926	19.17000602	19.8020414	20.00226289	20.11817831	19.71618783	20.06222636	19.98507694	19.81009059	19.78110363	20.11698432	20.21229941
RACK1	20.59618179	20.58870126	20.09318768	20.39841831	20.19862848	20.2086669	20.42813241	20.20436442	20.03707233	20.13551923	20.09677756	20.31174887	20.22964764	19.9415685	20.59252479	20.24199188	20.08425352	20.01233366
ACTG1	18.34486937	19.24706032	18.36984825	19.01009472	19.49804231	19.49775306	17.03362598	19.22021727	18.3666133	19.11326732	19.94300213	18.99926682	19.04834817	19.19496751	18.65822489	19.16372568	19.79446307	18.59195588
ACTG2	20.14697056	20.77460641	21.42299623	20.86155731	21.46213965	21.94652867	21.46027578	21.5741522	21.63573948	20.6416862	20.93434012	21.43178536	21.39133617	21.07001085	20.84795339	20.26687874	21.51086109	21.10143602
SUPT4H1	0	14.5058703	15.00206318	14.36633049	14.36827771	15.53110085	14.5850298	0	14.09717104	14.50276881	13.35047253	13.47671308	14.89525298	12.77367981	14.22989095	12.88780053	14.08011714	14.2797711
UBE2I	18.88449223	19.53440976	19.44902682	19.01495301	18.82463153	19.1918201	19.52648993	18.2699984	18.47361076	19.08190386	18.96439445	19.34229564	19.27239016	19.31981873	18.74871934	19.15962023	19.43522299	18.63213701
TMSB10	19.82620127	19.58851827	19.43154504	19.70335138	19.49413266	19.57047429	18.64550881	18.87393734	20.27517125	20.19157962	17.78661443	18.44257681	18.56501687	18.32684337	18.67890698	18.34485624	19.21640988	20.06962447
PPP2CA	17.93093076	17.29673141	18.17148698	18.15559309	17.82874809	17.99966485	17.68871239	17.8683548	17.9568997	17.69954788	17.89690506	17.4758848	18.73555551	18.05589538	17.70855433	18.16172853	18.38453926	17.7045403
YBX1	19.35545434	19.25338817	17.30640983	19.26988144	18.52346492	18.50741745	16.22591156	18.69443866	17.94555457	18.59937636	17.28134033	17.74808267	18.41755678	17.08831724	18.70715731	17.58540798	18.39358201	18.1066862
SEC11A	18.34172729	18.58051863	18.75986117	18.43650696	18.57908157	18.1939055	18.62609456	18.42337075	18.52578352	18.64695405	17.81974905	17.85522493	17.71148067	18.24952552	17.89413536	18.12948883	18.14158268	18.75199979
CSNK2B	16.9609605	16.89503298	17.49166536	16.55487172	17.10194346	17.37405033	16.95097871	16.62010458	16.72119444	16.77426286	16.84654675	16.85084915	17.64483976	16.71425086	16.96344876	16.78473093	16.8322792	16.75593451
TPM4	22.83026741	22.83426117	22.59137499	22.59092792	22.36094511	22.26811348	22.5745001	22.28098396	22.58606976	22.50939181	22.64243384	22.93887101	22.93306306	22.00414055	22.66192474	22.69455216	22.728239	22.488363
ACTC1	20.58926425	21.1516146	21.89174783	21.50625624	21.95401699	21.79875487	21.65075825	21.941192	22.38991382	21.99006621	21.67541331	21.79205659	21.92391337	21.86924478	21.34337049	21.12127613	22.03620011	21.34003711
UBE2L3	18.10280919	18.29564629	18.28572082	17.89103968	17.84927623	18.11142194	18.99262134	18.03273419	17.95411322	17.90939158	17.74640022	18.27158128	18.63914714	18.47050621	18.12594234	18.44350031	18.12867641	17.62906374
EEF1A1	23.14051355	22.48961574	22.29672062	22.39723501	22.2692036	22.71444384	22.08793816	22.44397954	22.60009393	22.51064406	22.6349259	21.71380056	22.73988946	22.08221695	22.85208826	22.26266308	22.0830678	22.33882437
TUBA4A	20.38780107	20.5068009	20.87044393	20.7912901	20.88243092	21.3690576	21.12787203	20.49390027	20.63194181	20.5976067	21.58610974	20.77681412	21.89411991	21.16807129	20.74422286	21.28549253	20.99916986	21.14461219
TUBB4B	23.35696933	23.79170761	23.62346247	23.81619872	23.17814706	23.30185423	23.46425582	23.12505711	23.46478453	23.10507918	23.81044484	23.05292785	24.32656993	23.34560801	23.43528493	24.04801617	22.97736955	23.36622909
CSNK2A1	16.56153746	17.26852515	17.68072661	17.10276547	16.83830748	17.12705334	17.21508117	16.72200804	16.78909738	17.14438083	18.02587677	17.28428374	19.99102682	17.31726796	17.5824133	18.30622458	17.21186186	17.06615227
PAFAH1B2	18.14810771	19.21178747	19.75823064	18.55737804	18.87040103	19.12732592	18.85403961	19.005549	18.77259226	18.44539216	18.92091994	19.05887465	20.3260834	19.28614598	19.09063855	19.19942418	18.67434531	19.61776719
H3C1	23.24693684	24.7225621	24.13244184	24.08937871	24.90455773	24.87535415	24.68385067	24.01950114	23.95752587	24.99604328	24.11486197	24.52469594	23.67806978	24.65843422	24.50326892	23.56959153	24.30197212	24.28089959
H3C4	23.24693684	24.7225621	24.13244184	24.08937871	24.90455773	24.87535415	24.68385067	24.01950114	23.95752587	24.99604328	24.11486197	24.52469594	23.67806978	24.65843422	24.50326892	23.56959153	24.30197212	24.28089959
H3C3	23.24693684	24.7225621	24.13244184	24.08937871	24.90455773	24.87535415	24.68385067	24.01950114	23.95752587	24.99604328	24.11486197	24.52469594	23.67806978	24.65843422	24.50326892	23.56959153	24.30197212	24.28089959
H3C6	23.24693684	24.7225621	24.13244184	24.08937871	24.90455773	24.87535415	24.68385067	24.01950114	23.95752587	24.99604328	24.11486197	24.52469594	23.67806978	24.65843422	24.50326892	23.56959153	24.30197212	24.28089959
H3C11	23.24693684	24.7225621	24.13244184	24.08937871	24.90455773	24.87535415	24.68385067	24.01950114	23.95752587	24.99604328	24.11486197	24.52469594	23.67806978	24.65843422	24.50326892	23.56959153	24.30197212	24.28089959
H3C8	23.24693684	24.7225621	24.13244184	24.08937871	24.90455773	24.87535415	24.68385067	24.01950114	23.95752587	24.99604328	24.11486197	24.52469594	23.67806978	24.65843422	24.50326892	23.56959153	24.30197212	24.28089959
H3C12	23.24693684	24.7225621	24.13244184	24.08937871	24.90455773	24.87535415	24.68385067	24.01950114	23.95752587	24.99604328	24.11486197	24.52469594	23.67806978	24.65843422	24.50326892	23.56959153	24.30197212	24.28089959
H3C10	23.24693684	24.7225621	24.13244184	24.08937871	24.90455773	24.87535415	24.68385067	24.01950114	23.95752587	24.99604328	24.11486197	24.52469594	23.67806978	24.65843422	24.50326892	23.56959153	24.30197212	24.28089959
H3C2	23.24693684	24.7225621	24.13244184	24.08937871	24.90455773	24.87535415	24.68385067	24.01950114	23.95752587	24.99604328	24.11486197	24.52469594	23.67806978	24.65843422	24.50326892	23.56959153	24.30197212	24.28089959
H3C7	23.24693684	24.7225621	24.13244184	24.08937871	24.90455773	24.87535415	24.68385067	24.01950114	23.95752587	24.99604328	24.11486197	24.52469594	23.67806978	24.65843422	24.50326892	23.56959153	24.30197212	24.28089959
HBB	26.74114209	25.99094773	26.81763305	25.32544422	28.73261017	29.72674099	25.000265	27.72949669	27.27850135	26.93298775	24.53825573	25.88899318	26.43293383	26.03917644	25.6627655	24.25185076	26.19628874	27.91483446
NOMO3	13.07059415	0	12.09484812	13.20336354	13.36705782	0	0	13.41838731	0	0	0	0	12.36891657	12.58081302	13.12191999	12.42633387	0	13.02428242
HBG1	26.43629217	25.81274598	26.79387147	25.9521674	28.5362841	29.47211989	24.94788338	27.79436966	27.07193889	26.34580168	24.35020691	26.02016204	26.02444746	26.25765429	25.65199064	24.26670878	26.58209097	26.92307046
HBG2	17.68993422	15.94095632	15.65339923	16.09284971	17.34778071	20.14561238	14.95001305	17.30591019	18.31021253	15.70239254	15.08267857	15.26232558	17.62659781	14.89608374	13.07548209	14.62168257	0	19.55311177
HBA1	26.64937395	26.10542069	25.70194899	25.64515288	27.36796098	28.63575476	24.75500694	26.56863391	25.95925035	26.4435251	24.03409368	25.15265234	24.9793582	25.10498435	24.41193232	24.02479626	24.70846151	26.79935485
HBA2	26.64937395	26.10542069	25.70194899	25.64515288	27.36796098	28.63575476	24.75500694	26.56863391	25.95925035	26.4435251	24.03409368	25.15265234	24.9793582	25.10498435	24.41193232	24.02479626	24.70846151	26.79935485
CXADR	15.99233842	16.68374297	15.24920809	14.04087413	15.47199515	0	15.44211774	14.0712851	0	13.36671107	14.10395927	12.73154941	15.51541495	14.9848352	0	15.83298222	14.14850078	14.43492468
NOP14	13.50932886	14.61607921	15.14325225	13.94475307	14.16190994	13.99774139	14.56484097	12.3458508	13.72192212	13.81212304	14.27200579	13.91335429	14.22708134	14.16805032	14.38422343	14.91867129	14.58722837	13.22884232
IGBP1	16.29014136	16.81307198	16.12871385	16.0263789	16.23048629	16.79619441	15.73292354	15.89246778	15.92219466	15.94271816	16.43374193	16.30350262	16.69864427	16.54776662	16.31931917	16.70762053	15.58425418	15.73900603
SIRPA	14.73705131	13.8503431	14.78044705	15.53186544	15.12083049	0	0	16.74560785	15.19539579	15.58855441	14.30227564	11.66254071	12.08680746	14.20904918	16.14164442	14.62346314	15.13676149	15.83509492
CYP4F2	0	0	14.17653065	11.85526277	13.50100571	0	14.10824878	11.35626403	0	13.37203713	13.35433876	13.7986565	14.5291094	12.90884593	14.51151487	13.28991194	11.25089499	13.38076662
PSPH	15.94918917	15.80964921	15.78294444	13.71867982	15.66707158	17.31125428	17.0074679	14.53356773	14.63183182	15.2416242	14.99194208	16.50653483	16.53020592	14.49144835	15.01210651	16.49912911	12.20856153	14.5640904
RBM6	13.89090984	15.45588354	13.71532389	13.73828733	15.07147316	14.62641556	14.365955	13.21385668	14.39660478	12.78848068	14.06624574	14.97756092	14.18979109	14.25319915	14.30584063	14.36621121	14.97409463	14.58630701
EIF4G2	17.14610529	17.64748478	17.77364199	17.05423582	17.20338774	17.6610365	17.58122196	16.99997033	17.25683639	17.05625866	16.615739	17.82852062	16.92439781	16.93041973	17.10787379	17.25881961	17.03786898	17.02963519
RPP38	13.55634736	13.82604509	14.40179357	13.14420561	13.49245297	13.605744	14.4483548	13.48580794	13.21158311	11.69439912	14.12258803	12.81583689	14.54770592	14.65347872	13.92323353	14.04298104	14.01055832	13.55699186
RPP30	15.71502124	15.54204931	15.5574741	14.57824829	14.8721612	0	15.0555906	14.19948619	14.17734562	15.04696793	15.40642289	15.2230989	15.41020263	15.37570738	15.5310017	16.23071122	14.77406183	14.21539599
GTF2I	17.97902268	18.17192855	19.53591417	17.95922081	17.26252	18.46203365	17.7035399	16.50499048	17.035743	17.15313914	18.1958873	17.3925137	18.67550721	17.41465156	16.92726554	17.52263816	17.23516172	16.9319621
[illegible]	[illegible]	[illegible]	[illegible]	[illegible]	[illegible]	[illegible]	[illegible]	[illegible]	[illegible]	[illegible]	[illegible]	[illegible]	[illegible]	[illegible]	[illegible]	[illegible]	[illegible]	[illegible]

MRPS10	14.44121894	14.03946747	14.72566852	13.84335265	15.11981249	14.03307544	14.57416503	13.20030566	13.46849453	0	12.82319967	14.25788976	0	13.28628095	13.01055884	14.8249999	14.12097156	13.75501071
MRPS35	15.13603329	13.53313709	14.58074564	13.33691348	14.07517358	14.71509311	14.38828169	14.02529944	14.25332526	13.77933673	14.61358423	14.54336346	13.9810433	13.87852508	13.48907595	15.22923763	14.71740679	13.87086164
MRPS5	15.19110893	14.36697861	15.52962846	15.05398768	15.30100071	14.33556386	14.25451975	14.31901976	15.02569398	13.42799461	15.25644684	15.77857007	15.1321878	14.77352257	14.94170084	16.35831477	14.75886955	14.92918706
MRPS36	15.64544141	15.13433027	16.08214206	15.76800437	16.01742029	16.6061526	15.66291226	15.92934095	15.41090236	15.33407029	14.94087104	14.89446457	15.0703796	15.32861494	15.15555465	15.01797022	15.43578101	14.80840737
MRPS11	14.47966014	13.43075893	13.8590368	13.97944997	13.99887841	0	14.27364438	13.37148726	13.42274312	0	12.96071424	14.47849216	14.37667906	12.18578708	14.22257663	14.9416712	14.56929565	13.92104913
MRPS21	13.14539999	14.64370054	14.56397756	14.80210788	13.97021923	0	14.08296876	14.32598791	0	14.543069	14.92738528	14.91088698	14.30316702	14.66399343	14.37349738	15.52188147	15.10091718	14.64633589
MRPS34	16.13540274	16.47333429	17.17712097	16.39074428	16.42694254	16.72464229	17.56950752	16.03819043	16.2267607	16.59395992	16.68502966	17.20903326	16.62568778	16.51337931	16.55667432	17.80189975	17.13256531	16.52501569
MRPS6	15.25060578	15.35102063	15.78636556	15.77194277	14.71694879	16.55537095	15.56895473	15.63740341	15.58924584	16.03113125	14.61409748	15.94681857	15.30608534	14.67451018	15.07199256	15.9951986	16.59690028	15.07278487
MRPS9	14.88956746	14.05289204	14.5518047	14.12458992	14.33432485	14.40375061	14.58407376	13.60749355	13.70308699	14.25046917	13.85679233	14.61121632	14.2812039	13.89886493	13.99759402	15.65491699	14.6991418	14.25235461
HMGN5	14.8015725	13.5795467	0	0	0	12.68473931	0	13.68932866	0	0	15.21459705	13.45797622	11.23323771	14.08094062	13.72639395	13.95922886	14.87959615	13.18378332
SARNP	19.8182581	20.12472069	19.92425952	19.04060511	19.57321494	19.45392129	19.57090389	18.81657795	19.13832212	19.46348646	19.40480414	19.10283145	19.49941844	19.31507243	19.63497962	19.45686586	19.59411795	19.45050876
LACTB	15.7677057	15.66968894	15.67073858	16.14297223	16.38983428	14.33800633	15.49985768	16.27809549	16.62586265	15.77788797	13.60098148	15.4139579	12.03403606	15.80732161	15.32217133	14.52589771	15.32288978	16.6863492
COG7	14.82578566	12.48769421	15.32794346	14.58276404	13.54448341	15.07063788	14.59544963	14.85003285	14.49117247	15.03296215	14.59898433	14.94698844	13.61750961	14.90542603	14.84752862	14.71264601	14.48082076	14.68624724
RPL24	19.21824438	19.71264538	19.370116	19.29547511	18.83017136	19.90435137	19.87836333	18.95494121	19.25591692	19.8698012	19.59611626	19.5233631	19.11261704	19.38252403	19.73965578	19.66242423	19.31068794	18.76127933
RPL36A	18.98597584	19.134054	17.34377764	18.3170217	18.76111779	19.24734954	18.42017831	18.12491485	18.58638043	18.90987256	17.98683695	18.0806497	18.30802539	17.52981251	17.96374349	17.71677049	17.97106996	17.84503133
CBX1	16.13071226	16.17100343	0	14.86751432	14.44610281	13.83795606	0	15.02891946	14.90247918	0	12.77898387	15.72571573	14.80722519	14.47968283	16.09659469	13.88194684	16.23617073	13.4433211
ARF5	17.92698832	17.15574594	17.4657161	17.69904858	17.93858776	18.4792675	16.77499351	17.68189433	17.36093694	17.55975622	16.80297609	17.08298435	17.34808083	17.0770349	17.25874745	16.02317498	16.89506489	17.35916656
ERH	18.51464122	19.08828408	18.82365363	18.522865	18.19116798	18.73625317	18.58688107	17.90025646	18.38749583	17.99206564	18.11393559	18.39621972	18.73953857	18.21811354	18.01388557	18.22171676	18.51472922	17.81647775
RHOG	17.10938704	16.84358949	17.31695004	17.988589	17.91191072	17.25903333	17.71151144	18.02648376	18.07305221	17.90863239	16.64454957	16.97232602	15.99949041	17.45705906	17.03171567	15.4679014	17.44592884	17.52317355
RPL19	20.48705885	20.42194166	20.26481095	20.78099874	20.07133993	20.24635963	20.09767902	20.37654102	20.22933827	20.20522052	20.29248692	20.32474311	19.97558325	20.66712963	20.52311982	20.28115083	20.48946813	20.30519242
SRSF3	20.48777577	21.27291449	21.1345479	20.10466737	20.43831648	20.7624598	20.55162941	20.00577324	20.35898501	20.3166877	21.18537076	20.44607564	20.73600423	20.70385955	20.71039268	20.77152121	20.59011083	19.95241058
MXRA7	16.00165353	15.50146791	0	14.49403497	13.0020266	15.51580858	0	0	12.58556237	0	12.82339785	14.1007451	15.81028744	12.03212024	14.29580844	12.72598069	14.55325945	13.9072358
FOXK1	15.48967404	16.13862601	16.5632007	14.00222889	15.63242983	14.94299739	16.54084614	14.75585385	15.18606239	14.85055952	14.81390525	15.50143815	15.67771996	15.42674578	15.39564945	15.80381413	15.46558496	15.49622452
CCZ1	15.81486132	14.52734289	14.83785907	15.33359692	15.24800065	15.24380709	14.81778683	15.32704615	15.01689719	15.06366794	14.3888378	14.5730161	14.68282211	15.28188292	14.86348115	13.78612931	15.06387774	14.72527908
CCZ1B	15.81486132	14.52734289	14.83785907	15.33359692	15.24800065	15.24380709	14.81778683	15.32704615	15.01689719	15.06366794	14.3888378	14.5730161	14.68282211	15.28188292	14.86348115	13.78612931	15.06387774	14.72527908
DAB2	15.17892178	16.15179852	17.01496969	18.11784028	16.26660599	15.35660309	15.72036034	18.70085147	16.10513706	17.26619734	13.26642041	14.28085514	13.32189012	16.56458702	16.47100199	13.6018704	16.8223382	17.26873702
FBLN2	8.995925659	0	8.788454492	13.54972816	15.24157706	14.3272304	15.97537456	0	12.92346795	14.87334122	0	13.89908508	13.77176197	11.68942251	0	8.476388625	9.254458044	14.1282277
HSPG2	15.35519071	16.088468	14.61154166	15.966945	17.82890553	15.1250284	16.99316749	13.0156769	16.60443706	14.57032663	15.11814836	14.68395488	16.01883281	13.03573629	14.14489921	15.87778747	14.46622909	12.83369262
ARHGAP4	15.49084623	14.17291242	15.77524998	16.7755491	16.96484566	16.01794593	16.21966825	17.23090189	16.68520862	17.27781932	16.13445546	16.38377495	15.09923013	16.13850727	15.66023294	15.99047726	16.09373267	16.98923458
EFNB1	12.1947241	16.78918755	13.36601479	12.25899295	0	0	0	13.31861245	0	12.5668866	15.06859398	12.68176032	15.58188785	12.04426753	0	13.30439074	13.78364014	0
RBM10	16.8055219	17.51427088	17.28201094	16.03641327	15.97974284	16.03553478	16.74197414	15.72721252	15.71630234	16.26604675	16.4621786	16.36290724	17.117154	16.1151186	16.61592436	16.60171514	16.17817366	15.97046985
RBM3	16.74328475	15.8704671	15.93169931	16.83602569	16.2274742	16.96748433	17.50914205	16.65757578	16.55630982	16.05518759	16.32294299	15.03276782	15.72912552	17.12568967	15.74315067	16.92028779	17.4404728	17.53524642
ATP2C1	14.5716691	14.38812098	15.06026911	14.09978794	14.32696348	0	0	14.15597487	14.03473349	0	15.78627122	14.24530448	14.11791398	14.38829588	12.98823368	14.10843512	13.78533812	14.07578139
CYCS	18.70169502	18.49528024	19.18646989	18.88632103	18.89692086	18.89621478	19.45071314	17.87471322	18.12148087	18.0526702	18.97383073	18.90252177	17.79019217	18.37874889	17.8375547	18.78328683	19.27110335	17.80563968
MPP1	15.11504476	15.63343807	16.58356969	17.65474921	17.15084816	17.23027453	16.34370284	17.69949864	17.82759536	17.29968372	13.56291025	15.13248247	13.92378301	16.45091748	15.24810506	13.20336414	16.31129737	17.21317914
TFAM	18.034622	17.86427722	17.96247711	17.04667841	17.55412098	17.70244771	17.57925528	16.58636932	16.50662538	17.33996251	17.78117379	17.66068341	15.85196535	16.5190381	16.72751428	18.53278347	17.50862439	17.14274201
PITPNA	15.72972404	15.81561763	15.20517442	15.33752404	15.3063912	15.21822679	15.39953115	15.9253238	15.90239177	16.64276411	13.62615997	14.993203	14.24257869	15.44046925	14.21523837	14.06051453	14.77613078	16.43953632
SLC25A3	17.80948721	18.81600785	18.41082539	17.86475593	17.91488974	17.6677606	18.12004261	17.81018347	17.5904934	17.5462028	17.90915413	17.98096662	17.7765745	17.91404382	18.27418561	17.98586443	17.63492934	17.469555
HDLBP	19.16463691	17.76927228	18.28008117	18.0671975	17.80434809	17.34878501	17.49702378	18.40116546	18.33056626	17.93796929	17.97595499	17.70708209	18.23484944	17.81620937	18.57287355	18.63360225	17.97469667	18.08666203
GTF2B	16.13301879	16.20418992	15.74659643	15.59851838	15.10906848	15.45698727	15.46144335	14.56237869	14.95897488	16.00344328	15.95683351	15.32179243	15.58215795	15.27463679	15.61289481	14.28815541	15.35496764	15.51688172
CDK5	14.86095747	17.81483659	14.85117485	15.32374158	14.73428468	17.31898722	15.05305733	15.02600515	14.96738485	14.86516802	16.17961514	17.64892914	15.5667278	16.32210529	15.666861	17.46392555	16.80582071	15.13097803
PURA	17.54899907	17.20356368	16.89726744	16.54250717	17.01404193	16.30463304	17.88945541	16.73011269	16.68494943	16.88014811	17.19408847	17.5781195	16.51569479	17.33722975	15.88220149	18.09180951	16.92428535	16.9732853
CDC42EP1	12.95871554	17.14700908	16.61929154	0	12.70093875	0	15.84561256	0	12.14524371	0	14.47381349	13.86098483	14.74020589	12.59845193	15.03241472	16.21684693	15.81450795	12.16195268
CLTC	20.94178375	21.05225128	20.96188257	21.41787504	20.70996988	20.82961329	20.58672711	21.26489369	21.30703428	20.80795733	20.5712322	20.8494807	20.93803357	21.42850996	20.68338596	20.93379806	21.42799683	21.0813851
HSF1	16.31404589	15.98553458	15.66193583	14.60672283	14.60960565	14.99153032	15.29543685	14.57723461	14.05440774	14.88149788	14.74925966	15.11726626	16.73867347	15.01052811	15.41881218	16.11897053	15.09037511	14.83007764
NFKB2	15.35857115	16.39460635	16.07786112	15.38108227	15.60221757	16.17051681	16.5219295	15.35198878	16.00109443	15.77415123	15.97013284	15.46688501	15.67697759	15.30062836	16.0054791	15.13828368	15.50170637	15.63896558
[illegible]	[illegible]	[illegible]	[illegible]	[illegible]	[illegible]	[illegible]	[illegible]	[illegible]	[illegible]	[illegible]	[illegible]	[illegible]	[illegible]	[illegible]	[illegible]	[illegible]	[illegible]	[illegible]

NAE1	15.82371077	16.19370176	16.42418808	15.464796	16.15325637	15.91887361	15.89476069	15.30375901	15.57100624	15.97763533	16.24726521
IRF5	13.86685688	13.16693491	15.07562702	15.1698212	14.8449239	0	15.28357406	15.23009786	15.55528118	15.32694017	0
LAPTM5	14.14800636	15.00397797	15.33566036	16.60715081	15.45792314	0	14.14567301	16.08526837	16.47361336	16.60847187	14.00741418
ITPK1	16.59131289	16.17339137	17.43430346	16.05889116	17.20056131	17.25366357	16.00710223	16.63657351	16.89042609	15.95108724	15.46846682
SNW1	15.75145253	16.15643726	16.11512622	15.57342125	15.58732189	16.33920657	15.27145519	15.4928834	15.51180138	16.14734795	15.53385678
IQGAP2	16.04920494	16.73885445	16.32437876	17.25027919	16.97549264	16.00979206	16.24923732	16.99828827	17.68137462	17.58150173	14.35581025
STIM1	16.69155781	16.80990518	15.87782051	16.50874382	16.4890615	17.20322028	16.82980808	16.22107138	16.13388128	16.19830625	17.01850634
TRA2A	15.05760938	14.88709015	15.23951317	15.44692654	14.84090381	16.2371338	16.17207006	14.83304179	15.56278277	15.66279222	15.7050394
SNX1	18.02419938	17.47368513	17.54617783	18.23686149	17.70303522	16.20558939	17.15260298	18.22918217	18.08548316	17.90854453	16.72727464
KRR1	16.12003739	16.38734581	15.430207	15.01236453	15.09097494	15.31370208	15.57205485	13.99742941	14.75333674	14.96998807	16.03496986
MTMR2	16.04487417	16.0200038	14.98826489	14.9774762	16.32582395	15.39433604	15.73829991	14.6187603	15.07525226	14.8838259	14.96563784
CUL1	15.49171795	15.57129147	16.00991026	15.37373419	15.25292799	15.8907901	15.77018437	15.2112746	15.1027219	15.1181135	16.24849078
CUL2	15.62705189	15.7145128	15.73094656	15.79680506	15.97479564	16.38401865	15.70172431	15.92709062	15.62543752	15.70397558	16.84980013
CUL3	17.23752786	16.65247367	16.73560674	16.21287987	16.81869119	16.58256904	16.77776299	16.34277243	16.50325776	16.39416317	17.36266142
CUL4A	0	13.09654145	14.32559217	14.73211907	15.34462195	14.79963182	15.04714389	0	13.51920179	15.04525707	14.1919541
CUL4B	15.25915793	15.46796755	15.00868302	14.8105279	14.92651915	14.87126785	14.44719953	14.75881487	14.9775052	15.30827195	15.481745
TP53BP2	16.18082245	16.04918483	15.94206861	16.12734812	14.35316015	15.98642555	15.70969144	15.5083045	15.72300197	17.73927083	15.94951452
DYRK1A	14.61046156	14.91058409	14.46901809	13.73696267	12.94752289	13.74988968	13.11799179	13.54862886	14.05433156	13.58530757	13.91879519
GFUS	18.27873486	18.36663731	18.38811033	16.10215129	17.11222886	17.72700046	17.61375373	16.55564747	16.26842478	16.8033048	18.57443104
RAB31	16.6899146	16.48531034	16.53768975	17.21627724	16.78717313	15.57688138	17.054234	16.74546784	17.05342795	17.46351689	16.08731991
RAB32	15.99307132	15.65480219	14.83008613	16.5655442	16.26891061	14.70271698	14.54886059	16.4599436	16.36296715	15.8900683	14.82974412
TPBG	15.07105837	15.67516561	14.7140392	13.89434707	13.89442442	15.92779424	13.27537802	13.77682044	0	12.2138426	16.35483652
FHL1	0	17.03209905	0	0	14.97870252	15.05287911	13.74652303	15.79930534	15.45856081	14.95937038	15.24430649
RIN1	0	0	0	12.04748974	0	0	0	0	0	0	13.26587111
AAMP	14.70759242	14.53224842	14.31115418	13.83975403	14.9047974	15.10319655	15.6551434	14.37196138	13.95454778	14.15813659	15.39406999
MOGS	18.41952035	19.57620141	16.83873479	18.27643257	18.58814194	18.06271764	17.71965265	17.82729676	18.17515418	18.06260648	17.83934432
ALCAM	16.14789134	16.94619711	18.44654382	14.35074351	15.96853409	16.33831026	15.84304752	15.03808072	17.28899446	14.49222802	14.90608586
LAMB3	15.872772	14.44336069	15.20336742	14.45003472	0	14.67318721	12.68026433	0	14.34976311	0	14.8277816
LAMC2	14.0064586	12.23914981	14.09130624	13.3241822	13.66818015	0	0	12.64964344	11.38325709	12.57901686	14.10883517
RUNX3	14.83934182	14.14642806	14.0989782	13.6921734	15.47460472	15.57915225	14.24760842	14.15383279	14.67051784	14.99443329	13.55204683
SPTAN1	19.13871115	18.57371984	19.39960963	18.1155603	18.56162835	19.1158893	18.85588	18.21685507	18.16827425	18.11239242	19.26940923
GNL2	12.13438261	14.14521727	14.01129364	12.22983918	13.93943843	14.94403123	14.38968398	12.87016839	13.75608563	0	13.83338833
AUH	15.14583119	15.61870585	14.6713752	14.11678956	14.95019972	12.91351883	15.66460418	14.67526319	12.96781851	13.90562149	16.45869872
DDX39B	15.59144177	17.60234411	17.82938151	16.73717615	17.66978473	17.60563719	18.38825764	17.1143805	17.26491187	16.95725972	17.93096197
BLMH	16.33740985	17.03623869	16.87494824	16.38021082	17.32269108	17.76166941	16.11292814	17.38043887	17.86538422	17.23464672	16.51214197
EXOSC2	14.10967063	14.91433869	14.19791137	14.37724761	14.57749268	14.46418177	15.63356378	13.70940791	14.26287938	13.97483738	14.0995703
SNTB1	17.00754849	16.80471646	16.76359149	16.44681765	16.4139632	16.06332266	15.81527305	16.26945203	16.00294992	16.13712452	15.14791727
TUBB2A	16.3270186	17.04637017	15.8363091	17.16805017	16.73189683	0	0	17.38461527	17.04295216	16.29307082	16.84020896
KLF5	14.28200613	13.17167343	15.65570682	13.8225332	0	0	0	0	0	0	13.54603202
BYSL	14.13618025	15.5087234	14.61363077	14.87148041	14.64570131	15.17605122	15.47778321	14.33736275	14.40488169	14.1767093	15.10052215
IDI1	15.02442937	13.16304952	14.82363925	14.24562031	14.53542988	14.60169905	14.77428377	14.44731987	14.15154384	13.651	14.14004931

[illegible]

CPEB4	12.62241919	0	11.73615416	12.65946082	11.95200721	0	0	11.51214173	12.10903387	11.31657391	10.67755395	11.47174814	12.35301002	13.75057625	[illegible]
DOK7	0	0	0	0	0	0	0	0	0	0	13.91212779	0	15.16331676	0	[illegible]
KNOP1	12.73219486	15.66429753	13.60968657	13.06212455	13.52559357	0	12.45339775	0	0	13.24258843	12.8111179	13.37406227	14.00500717	13.6066475	[illegible]
HNRNPUL2	19.63816112	19.12346754	19.16891599	18.71058817	19.07266484	19.27690519	19.06391723	18.64450919	18.82021179	19.06838712	18.91803858	18.78438424	19.31271417	18.7487002	[illegible]
INF2	15.78006472	16.03459514	16.39620639	16.62268148	16.33538599	15.06716385	16.70023015	16.10433336	15.9112797	15.61205575	15.82034824	15.45519827	18.79700126	16.47947019	[illegible]
PDS5A	16.30962442	15.99861926	16.58716209	15.47689005	16.01850617	16.00185794	16.44036913	15.42171623	15.36718876	15.98703115	15.8211488	16.2072153	16.72808596	15.73234114	[illegible]
CLEC16A	16.04503188	15.88434289	14.89060096	17.9325198	16.01423744	19.54916903	17.58643392	17.48141551	18.71972345	19.2333015	0	16.48467762	17.10497716	15.18093487	[illegible]
AAK1	17.96294233	16.81301824	16.39475182	17.28568357	17.10518679	16.3043548	15.56087366	16.85700276	17.12476706	16.86866405	16.29931508	17.08799269	16.96529655	16.62780827	[illegible]
MPEG1	13.12427327	0	0	14.59466687	14.00662484	0	0	14.13601811	14.3695208	15.42896416	0	0	12.49409456	0	[illegible]
WASHC4	15.02191832	14.60686084	15.67281852	16.48649061	15.50081186	15.34706671	16.10285227	16.19852927	16.32096013	16.09744836	14.72951827	14.19943272	14.11108408	15.90839235	[illegible]
TSR1	13.82133374	15.72651889	14.87684571	14.24845097	14.86352066	15.80404109	14.83213459	14.72706031	14.51653372	14.10643163	16.16852079	14.94961631	15.65749651	15.60466226	[illegible]
VMAC	12.80828372	0	13.06717633	11.57074747	13.58339902	12.52911869	14.23459264	12.8050743	13.2773734	10.25532138	12.36866791	12.57949694	10.92723253	14.3300206	[illegible]
IAH1	16.68689341	16.34889575	14.64165721	15.40475841	14.99014171	15.30877313	15.19425097	15.35653163	15.48503377	15.60263578	15.41229344	15.80294474	16.49023785	15.1410667	[illegible]
ALG11	18.41157874	17.62803686	13.68700441	13.92921632	14.2373722	17.04183759	18.69551388	13.89473239	13.90055342	14.11974364	16.15455562	17.31547023	14.2493603	17.68102496	[illegible]
QRICH1	15.00721055	16.10855508	14.76243091	15.47180617	15.75045011	15.98478732	15.07644996	14.75772235	15.24859545	15.24868447	14.26670363	14.89388885	15.84812394	14.88610587	[illegible]
SMU1	16.44430973	17.80523326	17.53493059	16.51008975	16.92110944	17.25293188	16.91888625	16.54191547	16.54975154	16.43843781	16.38555437	16.8386042	16.8450724	16.612985	[illegible]
ZNF800	0	0	0	13.3152842	0	0	0	0	0	12.63760075	14.67170637	14.00655328	14.43957944	15.18004101	[illegible]
HLA-DRB5	18.84312527	18.20461592	17.23740065	18.86104401	19.1271735	18.22496642	19.26679312	18.15518573	18.90913407	19.44323142	19.42648488	18.64925947	16.28613131	18.93993023	[illegible]
LRRFIP1	16.67238196	15.93214833	15.66768276	16.98050971	16.59085416	15.61045221	15.20990339	16.65012627	16.30839153	16.66339371	15.02513568	16.61846795	16.58781333	15.65438768	[illegible]
P3H1	0	13.82759362	13.9460933	13.8597885	14.11223109	0	0	15.58495012	0	0	13.54334824	13.27582513	14.08347592	13.97936911	[illegible]
EML3	16.40291746	13.02248905	16.09247134	15.29338591	16.04332322	15.77782117	15.81249766	15.69079888	15.60429487	16.03297442	14.93962351	14.65379434	15.38465519	16.22124091	[illegible]
MAP7D1	16.11695784	16.12984064	15.98624898	16.50011574	16.42207247	14.81440175	14.3782197	16.48289496	16.46408826	16.41095837	15.86218197	15.80085794	16.20480499	15.99339073	[illegible]
UAP1L1	12.74862456	15.16676271	15.0058135	15.83093555	16.09279004	13.13432494	14.90495805	15.60920783	16.33690708	15.48643034	13.75017641	12.48843079	12.07182632	14.90534945	[illegible]
TKFC	15.89781112	15.3097783	16.90761879	16.34560801	16.74067973	16.29028946	16.3295214	16.28590633	16.67089468	16.69272648	17.61747341	16.25785581	16.49691215	17.17268234	[illegible]
LSM12	16.88753635	16.79148943	15.09814841	16.32246403	16.01837475	0	16.2389866	16.07304975	15.60778632	15.42278007	15.29815601	15.91113319	15.84999182	15.00453733	[illegible]
TMEM30B	12.96520943	11.78299061	13.06737659	12.0693629	0	0	0	0	0	0	14.18899353	0	14.61190339	12.99769187	[illegible]
ADCK5	13.60412482	0	14.88470434	11.92905709	0	0	13.65480317	12.05297311	11.21086825	0	12.89187871	14.34516482	12.40306808	12.11343887	[illegible]
KRT71	17.57354908	16.33964072	12.85353096	15.97173491	0	0	0	0	0	0	15.52616919	15.29899304	15.47538401	14.97108198	[illegible]
CCDC88A	0	0	12.96663887	13.86882444	13.63886221	0	0	13.84399358	14.3449796	12.79666764	10.66865174	0	0	13.96114416	[illegible]
TMEM176B	0	0	13.81326098	14.81708759	0	0	15.09788071	0	0	16.10416697	12.10211812	0	0	14.22131747	[illegible]
RABL6	16.91016418	17.03918246	17.10224646	16.70165737	17.06338027	15.74541921	17.0578992	16.73790882	17.30193375	17.1872193	16.07232416	16.44334323	17.24902995	16.18760111	[illegible]
VMA21	19.0805017	18.7762064	17.49518092	18.70355839	16.99410833	17.22221104	17.01776281	17.97121743	18.16084577	18.00815622	18.55824413	18.05668318	16.92958386	18.10049745	[illegible]
TUBB6	17.27565323	18.14410056	17.76486532	17.34678667	17.92550754	17.69238031	18.23986769	16.98080167	18.01345439	17.91603069	18.87877557	17.3982411	18.79306198	14.41058333	[illegible]
TIMM50	15.98418235	15.74196643	16.73431487	16.48470073	16.04753284	16.06961218	16.78737353	16.00549623	15.87562686	16.1032798	16.0938255	16.68732965	16.45482226	16.59300819	[illegible]
PARP14	15.45505839	15.90661191	16.00925081	16.31043292	15.8133888	16.02692154	16.72691131	16.32738091	16.18372608	16.70473996	15.97660328	16.11758778	16.48697112	15.12198337	[illegible]
GLYR1	16.36153917	17.37826747	16.49640045	16.83017707	16.33623631	16.46725366	16.61781146	16.05201325	16.1531031	17.08210453	16.72598308	16.41398153	16.42198595	16.93240545	[illegible]
COX19	15.60043665	15.35321684	13.99470518	13.69293619	15.00225602	15.42289899	15.38465716	0	14.01919892	15.33351981	13.48733663	14.54539121	13.45159196	14.96288136	[illegible]
VPS26B	14.54329242	14.55493693	15.53445393	15.64327194	15.51619993	15.20793689	15.85447351	15.81285426	15.80051811	16.18343745	15.46253098	15.54172776	16.36364847	15.4138758	[illegible]
LARP7	16.1236475	16.98394455	16.45289638	14.67109711	15.44286581	15.70798455	14.79802725	14.99037754	15.00028288	15.10594127	16.11677576	15.35382768	15.96511227	15.92496424	[illegible]
NADK2	14.79762828	16.03528759	15.40343052	14.13304843	14.31302943	14.23244435	14.97065317	14.35008575	14.10522756	15.35035221	14.79647348	15.17798471	14.0230428	14.82679896	[illegible]
ACSF3	15.9547375	15.16672426	14.71267697	15.38591377	15.95733412	0	0	14.42023576	15.70623304	15.83221312	13.78105399	14.65637314	14.74714103	15.98255535	[illegible]
TBC1D10B	16.11290031	15.34310315	14.49595113	16.31245992	14.56593949	0	13.43238999	15.4331165	14.32604526	14.67119464	14.91427526	14.69918725	15.35992579	14.98216029	[illegible]
TPRN	16.30262819	16.67901355	16.27625975	14.40006274	15.02083891	15.66663193	16.38907527	13.03155146	13.23783781	13.86981954	15.18259455	14.98669514	17.99065231	16.02594738	[illegible]
ANO6	15.28743537	0	15.75939806	15.3849397	14.26233389	14.74687746	15.33152247	15.38301946	15.59073346	15.136422	15.10848474	14.33810538	12.20591522	14.71660975	[illegible]
PLCH1	15.58643896	15.52477603	15.47668863	15.75474298	15.8733186	12.34252844	15.01144345	15.47668775	15.74543676	15.59388517	15.28015751	14.94375077	15.2000094	14.31173501	[illegible]
FILIP1L	15.20848324	0	14.57012056	15.83888508	15.7631614	15.95967682	15.59978359	16.32306163	15.83188128	15.84364249	15.33642497	15.71398248	15.60594843	16.08753778	[illegible]
SVEP1	0	0	0	0	12.37853829	0	0	0	0	0	0	0	0	0	[illegible]

[illegible]

ARHGEF18	14.77226503	13.44338155	13.75723617	12.93699615	14.2686308	13.44885283	14.4137236	13.85726679	14.30354554	14.03157989	12.4557885	14.01308716	11.97920511	12.71925762	13.1008325	12.36139018	13.29399245	14.17288648
UBR3	0	0	14.55798049	14.0511203	0	0	0	0	14.02036792	13.28625796	0	0	14.07773043	14.70544836	13.18467365	14.94742363	0	14.63582781
TMPPE	0	13.90874865	13.37963087	15.24621435	14.54929009	0	15.19051783	14.74151081	15.45097523	15.32872204	16.04621794	15.03358415	14.88334611	16.18790024	14.47655562	14.59969757	14.62048408	15.94046783
PIK3AP1	12.83336893	13.55115659	14.74318037	15.1937804	14.05336232	11.00846145	14.78556791	15.73166334	16.1648988	15.72333774	11.86098105	13.06511418	0	14.71622498	13.50606976	11.39951889	14.60945517	15.33030077
ARHGAP27	14.0867192	13.60558046	13.85454446	15.29558707	14.99116309	14.76866855	15.02242877	15.19731705	15.27697985	15.42861677	13.96602871	14.4740926	13.74881432	15.53933254	13.71568984	13.50975616	14.68789271	14.65528242
TOM1L2	0	0	0	13.14012844	0	0	0	0	13.27060602	0	14.94653572	12.82441555	15.72240305	13.60365577	0	0	13.84911165	0
NCCRP1	13.66359654	12.32130414	10.90920367	14.0386342	0	0	13.70316716	11.64821241	0	0	0	0	16.18261925	17.71564952	14.20139842	11.60275051	17.72041535	13.55139578
VPS13C	16.11391302	15.72987441	15.76634015	16.22189064	16.45121023	15.83007762	16.44132116	16.03121304	16.28246576	16.41671353	14.67902848	15.30349426	14.26096116	15.65510693	15.90950173	16.40217389	15.95543166	16.34833807
MOB2	15.62898823	15.85024789	15.57229615	14.45702411	15.98641427	15.04302561	15.68104749	15.2014735	15.45597407	15.61549	14.97780012	16.10963015	15.70444658	14.90382017	14.92034559	14.86890279	14.92430682	15.77083445
UNC13D	15.11401902	14.92379625	14.4180075	15.73625503	15.85027117	14.63663436	14.70964801	15.60089723	14.29840188	15.03250267	14.70985233	12.96186536	11.95940027	15.51030276	14.90356314	10.85892329	15.78689844	14.96122057
IKBIP	15.29675353	16.91132798	16.41187467	15.92225873	15.25268954	15.34562628	15.24270675	17.06565131	16.3194377	16.0906904	14.79419859	15.04776945	14.7602536	16.26307359	16.28492235	15.35228568	15.73150922	15.25079644
UBE2R2	15.63252856	15.54346574	15.00643138	14.29379703	15.21627176	0	15.28319958	15.17860498	15.20870634	15.04521607	15.15363351	14.37060973	14.64007797	14.21897245	14.30221232	14.44773479	14.80073898	15.05188368
TMUB2	14.481711	14.10530446	0	14.14972712	10.78388828	0	0	13.45158266	12.36036836	0	0	13.84221662	0	0	13.407212	12.78776073	13.92302284	13.52029975
MED25	15.22851443	15.96748521	15.18745719	14.98360252	0	0	0	0	0	0	15.07619516	14.12302147	15.23336207	14.73308165	14.2437577	15.48258199	15.56647857	0
TUBA1A	22.95088468	23.64754505	23.05354031	23.35709098	23.00299283	23.25133259	23.02582255	23.26579558	23.37424725	22.94512293	23.6308911	22.74948883	24.11485355	23.71901524	23.28641365	23.71099367	23.48193625	23.41113596
RPS27L	19.1119959	19.67202537	19.52590392	19.59812226	19.06204515	20.00652093	19.48513723	19.53977909	19.98096532	19.79403266	19.63386429	19.42668923	18.96410462	19.61246595	19.37556687	19.45271004	19.56858242	19.38328597
PGAP1	14.84371848	0	14.81571183	16.3563664	16.35956312	14.48100241	14.48914935	15.47175111	15.79865581	16.62967801	12.29199072	0	0	15.35130417	14.53491564	0	15.31773278	15.18796803
CCDC80	0	0	10.04418264	0	0	0	0	13.3556194	0	0	0	0	0	9.734948135	12.11866548	0	0	0
BTN2A1	14.54295439	0	0	12.76523126	0	0	0	12.08012739	12.91329101	0	13.81704083	13.61467278	13.86594542	12.87068689	13.83477164	14.23729517	12.9022162	0
SUPT6H	15.38421579	16.23986464	16.19095089	15.68393332	16.01165876	15.76648948	15.75913144	15.50299771	15.79322058	15.57421481	16.0006527	15.40236662	15.42523486	15.96268935	15.77043	15.7358512	15.68554165	15.3234714
SND1	19.62971902	19.59481082	20.18870987	19.47381015	19.20029324	19.43580606	20.27181221	19.47267225	19.67559486	19.46734298	19.40654278	19.24253319	19.21499539	20.01103936	19.99169362	19.27154538	19.14287472	19.29351136
MARK2	17.0042479	15.75371989	15.43874453	15.57052467	15.60634421	15.28682733	16.00196494	14.93159769	15.29324711	15.58609118	15.7677357	15.77316125	15.8864382	15.79331317	15.63158429	17.06597912	15.46973161	15.23617233
DDX46	17.46234275	17.55293593	17.61605195	16.90802203	17.37064364	17.40539664	17.77376904	16.63331367	17.00309786	17.17979947	17.83995562	17.53627124	17.31122363	16.97009272	17.6654454	17.81739709	17.23920824	17.19583565
RUFY3	12.53997309	12.2215737	14.86263603	13.9496603	14.82057233	0	0	15.13122113	15.11081861	14.37377269	12.69004445	14.99288562	13.19648687	14.00962157	12.68666348	0	13.82647323	14.89376738
TRMT10C	16.42625016	17.02524097	15.89182903	16.04214914	15.93910694	16.80088875	16.82277493	15.05493133	15.49807226	15.83995619	16.56339414	16.59366592	16.34580019	16.35155045	15.70346175	17.44543207	16.93609628	15.72836149
BZW1	16.83548299	16.66076543	16.68025424	16.54543917	17.17337398	17.10620221	16.97219768	16.86676738	16.98401507	16.96554925	17.04996999	17.39193541	16.73431042	16.80611966	17.34630082	16.76966293	16.7220681	17.066125
MON1B	15.80837741	15.11056949	15.67570372	15.57443092	15.65222386	15.75829652	15.89881706	15.85353229	15.42493845	15.77223845	15.16704811	15.67540793	15.07652287	15.46707488	16.11453143	15.34944309	15.47764551	15.17438156
LRRC8D	0	0	0	0	0	0	13.66701406	0	0	13.20160317	12.67574624	12.90274137	14.04398881	0	0	0	12.67272886	0
ASRGL1	14.76615056	14.65119494	16.15033811	13.64279209	17.2563894	16.95531982	17.04366681	14.61127295	15.59483667	16.23401353	16.43957804	15.06930373	16.29733612	14.41194952	17.00689205	15.20784614	13.80728485	14.30721221
DHX30	14.53786901	16.30015325	13.89979833	15.21982273	14.53718498	16.02101909	15.40264987	14.37733129	14.46634362	14.66703507	14.24240613	15.18319587	16.19819562	15.10867169	14.83965601	16.14479654	15.66892544	14.59917928
EIF3M	15.9571412	16.27073931	16.05653767	16.27364907	16.3679747	16.68175399	16.59105911	16.15288052	16.28878245	16.02989329	16.33866335	16.64283549	16.28067917	16.52204903	16.08782618	16.56853812	17.04430539	16.00370593
MEPCE	16.89222836	16.60964025	15.59531918	15.56617067	14.75311839	14.43754154	13.87165045	14.90397431	14.84839151	14.75829602	14.84964562	15.23346447	15.65251383	14.91553696	16.08259575	15.04282979	15.65438156	14.94919859
ARMCX2	0	13.60578468	10.72653489	10.26620663	0	12.57615239	13.35516704	11.55811366	12.83920042	0	14.62222647	10.79045768	13.89194479	13.96067695	13.82632659	13.64051272	12.42927686	12.86858634
PARS2	15.38478415	15.72952843	15.02497713	13.36954037	13.32870163	14.65881249	15.6794278	13.51241858	12.68902008	12.28904471	16.05919128	14.07329579	14.93545122	14.56365795	14.63743171	15.21624541	14.4108924	13.33850218
RSRC2	15.59409437	15.73098366	15.71899226	14.82666553	14.51740868	15.17774404	14.43226841	14.9180971	14.28775507	14.40195081	14.7384365	14.81225723	13.73592297	14.72940334	15.97536389	14.98420043	15.3357086	14.49276002
RRAGA	15.15765039	14.71006177	15.15369118	15.59533933	15.27017925	16.13779762	14.94362908	16.14961022	15.89208769	15.47861831	15.65512822	15.25915491	15.05121923	15.67932342	15.29996607	15.2682767	15.03052941	15.74376498
CYFIP1	19.10279814	18.64707113	19.3026459	19.25423268	19.01791773	19.22677436	19.58506534	18.87788237	19.20212203	18.91691734	19.70027132	19.00547978	19.27643739	19.42609567	18.79630098	18.46154564	19.49942807	18.8342166
DOK3	0	11.64904573	15.25138908	15.68497821	15.00834467	0	14.74373447	15.93321573	16.17867093	15.58935661	14.16821792	13.37025145	11.57804508	15.18827794	14.97957278	11.93103233	14.55830544	15.80630102
NDUFAF7	15.15371607	14.89480703	13.98723183	14.73068635	14.37730083	15.24328819	15.11138867	13.52129731	13.90763254	13.28627141	14.18275623	15.09141327	15.25402719	13.93925404	13.35612322	16.23343215	14.96419472	13.18499683
GET4	17.56766613	16.90013243	15.9341125	16.56209741	16.8572479	18.11972354	16.84238228	16.43572608	16.77956307	16.35583901	16.61076095	17.11335477	16.33805735	16.03485755	16.16776726	15.94538632	16.51279446	16.331967
GDPD3	15.70188777	13.23794398	13.61896615	16.10804965	0	0	15.64022949	14.58282397	0	13.04901727	16.40730874	15.58605051	15.66388152	15.56690324	16.50810111	15.25451744	13.49997201	15.02650369
COPS6	16.89429128	16.48505392	16.4837902	16.05605288	16.47267522	16.49656381	16.62136599	16.28185109	16.3886786	16.29854647	16.85428164	16.30296469	16.59495896	16.4416251	16.61096354	16.52083326	16.05905109	16.13546694
LPCAT2	17.18776918	16.74105731	16.62668342	17.65591674	17.2183254	14.48955131	17.09066133	17.56250505	17.45634401	17.60904298	16.57616381	16.64657629	16.16584577	17.46555756	17.70640253	14.23097296	16.70503138	17.87080916
MAEA	0	13.25816761	13.69856299	14.33277468	14.41008071	14.21062141	14.19123727	14.21920457	0	13.89931866	15.25060969	14.37221559	14.01023937	14.44387777	13.19010468	14.33406401	13.97317367	15.53448254
EPM2AIP1	14.60052433	15.40911198	15.43714883	15.41923164	14.46016489	14.28187443	14.03305897	15.43306975	16.19673103	16.09907391	15.16773205	14.11300702	13.50545396	15.0630336	14.33787502	16.40839855	15.10225976	14.96178677
FASTKD5	0	15.61517082	14.75329531	14.16745138	0	0	15.24551006	13.770904	13.86212227	0	16.07301755	15.6923155	13.35247454	13.06313978	14.38395723	15.47872963	13.92901725	14.10763709
KDM3B	15.66684291	15.15832523	14.7339682	14.66219874	14.64085656	13.30000812	14.1094092	14.25270123	14.12889521	13.62168659	14.60169588	14.41054607	14.84237269	14.13667389	14.63464047	15.42918707	14.8158862	13.86260444
CHMP1B	15.4574777	0	15.97485604	16.11200895	16.29173799	16.77031526	17.31407882	16.47632372	16.53405396	16.90082444	16.37225093	16.1167185	16.25310304	16.91023254	17.17397017	16.96772073	15.01497292	16.65064421
RRM2B	15.90518379	12.86202084	15.08322512	14.75485917	14.90338339	14.62051093	14.43248927	15.2146129	14.70164532	14.86611517	12.93658609	15.54795379	14.61864987	15.14902793	15.79110346	15.28324297	15.3385147	15.06442224
[illegible]	[illegible]	[illegible]	[illegible]	[illegible]	[illegible]	[illegible]	[illegible]	[illegible]	[illegible]	[illegible]	[illegible]	[illegible]	[illegible]	[illegible]	[illegible]	[illegible]	[illegible]	[illegible]

PTGR2	17.34668807	14.80782087	16.26962681	15.42878773	0	0	14.68595264	13.44974249	14.44063463	0	14.36567206	16.01948214	16.18328115	14.65575322	15.90430772	16.56485748	15.13543782	15.44015517
ENAH	13.92427311	15.74963357	14.70755966	15.11006524	11.51583384	16.04606103	13.68238546	13.87507812	15.0524795	13.800298	16.05787178	15.32754058	13.71517493	13.18849588	15.65707896	15.1366279	14.97911455	14.57102013
GPATCH11	13.19519466	12.41190363	12.39347452	12.68101735	0	0	0	0	12.11990429	0	12.61039307	12.17902599	0	13.55036364	13.19132403	0	12.26141362	0
MRPL43	16.29654098	15.01886303	15.09476794	14.80475269	15.58962635	13.49796936	14.42937673	13.42946779	14.74814544	15.15408846	14.50130378	15.75479832	15.23249602	14.31964001	14.27392547	13.77687545	15.41191862	15.18808835
ASCC1	14.16952763	14.6526974	14.56389845	14.91369266	14.17971915	15.01844573	14.5661975	14.71253088	14.84603244	14.43352546	15.27784925	14.83955452	14.30908258	14.70754629	15.01439887	15.84249489	15.06521445	15.15319219
LRRC57	16.21901988	14.57673851	15.07697083	15.66132329	15.41420353	13.88413772	13.50902108	15.0724748	14.84349604	14.61219089	16.15129036	14.72040126	14.37370964	15.63124612	16.02469854	15.45184962	15.63503998	14.82240541
EIF1AD	15.06752922	12.09924956	13.9131838	14.23974725	15.00342578	14.63125556	14.40124138	14.65057476	14.34186408	14.30083319	14.59440821	13.95920922	15.76882733	14.50369971	14.80347617	14.41408569	13.41927846	14.52364163
KRI1	15.22483074	14.25939531	11.83228615	13.34366191	13.08103574	14.1019167	0	12.45373345	12.01755656	0	14.18724816	13.45842364	14.12365603	12.74247022	13.75213948	13.42296942	14.11791271	12.74109229
CCDC71L	13.28608209	11.51004337	13.26433206	13.32391924	11.7735431	11.38325551	12.08418522	0	11.74690186	12.45684619	12.5972649	14.37934558	13.70402984	15.19422692	14.25957605	0	12.79358853	11.01298848
ZNF579	13.11644946	15.23062474	14.65917759	13.31555287	0	0	14.46116704	13.69122777	13.47611887	12.76454494	14.94461895	0	16.09645426	14.52795515	13.60879072	14.38082553	14.39340512	13.32590024
PRPF38A	16.3945603	16.993149	16.81425485	15.67026	15.96064735	16.10372994	16.31778397	15.67681472	15.95564689	15.94282946	16.1261253	16.0885416	16.27373358	15.88967904	16.32016234	15.88266534	15.90545366	15.68507127
GATD1	15.62515254	15.24055087	16.20062955	15.79683892	16.06444527	15.32270483	16.19160823	16.22815857	16.15561749	15.8124907	16.08545285	16.04510572	16.43363459	16.39476242	15.59007747	16.56647648	16.22990532	16.2039394
NHLRC2	14.66689837	15.87144957	15.48917337	14.29312809	14.80632305	15.28061249	13.89561448	15.00856779	14.82076844	14.39846815	16.03515265	15.99388572	14.88927631	14.58662759	15.40549104	15.55352219	15.10566014	14.40029133
AVL9	16.24009837	16.14480479	15.39183776	14.97747489	15.5868367	16.22207333	15.69481143	14.42824502	15.53512655	14.53644762	15.33687263	15.19796054	16.85455683	15.33690136	15.14184848	15.28397104	15.04244141	15.39200002
GOLM1	15.89573752	15.76700712	14.97863689	15.10104546	15.55704574	13.07699822	15.66772964	14.92411614	13.40643132	14.59307306	15.03493981	16.38572959	15.18835341	15.68138985	15.88814755	15.16193546	15.68307942	14.2786525
COLGALT1	18.60869588	18.68429362	19.01245132	19.18579146	18.8984034	17.5494968	18.49558852	19.52062803	19.7962273	19.08552384	18.09236342	18.13631952	17.82382717	18.26921316	18.41391195	17.54313002	18.27770498	19.05968344
SUMF2	16.78087313	15.95830853	16.06109905	16.83688432	16.83280312	15.67603066	16.01067903	17.21490633	17.27842479	16.97155083	16.66966202	16.73770957	17.31275987	16.42693832	16.04293497	16.348018	15.6863819	16.51336124
SIDT2	12.65557974	12.86689008	14.4270752	15.17923896	14.63438478	0	14.09401468	15.80993148	15.90443232	14.99248918	13.33359706	12.112022	13.17619537	13.97837696	13.22738165	12.18132517	13.47998361	16.05145591
SUMF1	14.77663513	14.8607301	14.33416347	15.63247653	15.44757867	16.36057037	14.95399302	15.25964572	15.64563645	15.44995994	13.89411611	14.25583626	16.21926573	14.60459485	14.65966366	15.21548175	15.14752357	14.89055579
POGLUT1	14.80013158	15.97839084	16.07767486	14.91187982	15.56444957	15.69384243	15.19614448	15.45353176	15.88691281	15.40099067	16.06016454	15.67781963	14.08614789	14.79236971	15.49871484	15.4559945	16.06431336	15.12071724
PCYOX1L	0	14.70561636	14.16286257	15.41728841	15.19158067	13.94005035	14.4662881	15.17133304	14.84359668	0	15.3169916	14.95899974	15.26470875	14.91711791	15.09428117	13.34935089	14.59350419	15.76362624
TMEM87A	14.58922126	0	13.99879192	11.94495516	12.96709615	0	14.82867266	12.14595546	0	0	14.35371449	11.27274837	12.4438832	14.15167761	12.59850782	15.23754959	13.60132841	12.33978586
RDH13	17.75948797	17.06005665	16.29579822	16.01109227	16.4950124	17.11901709	17.09544532	14.76937187	15.20438577	15.68488546	15.23423827	16.04146983	18.19120428	15.52045359	14.76592797	17.53592935	17.28330303	15.9040789
TTC13	14.30236253	14.75553493	14.33856108	13.92786195	14.20329184	0	0	11.24484722	14.11897465	13.81513611	15.22321677	15.82901357	12.48910781	12.59197505	14.04160368	13.53996268	14.05724595	13.93372919
HSD17B11	18.05954647	15.12525264	14.09970464	16.23386451	16.16714921	14.93777987	14.61952059	16.09195475	16.11474889	15.51723974	17.64002555	15.622846	9.696981	16.30808623	15.98546865	14.56249387	16.50207886	16.46675933
TXNDC5	18.87427946	18.67254555	17.89399636	18.26352195	17.74118778	18.06000518	16.77800191	18.70336754	18.62063856	18.55099966	18.28683861	18.60094496	16.98053481	18.08452036	18.33124103	17.04034625	17.83089977	18.53702284
ATAD1	16.95205184	16.56907762	15.94100559	15.69272521	15.72452343	15.83124722	16.7068444	15.23439222	14.99628556	15.34280647	15.74383502	16.42275851	15.88169745	14.62191012	15.68615628	15.69336561	15.99583045	15.90234475
SCCPDH	19.06065471	17.91784779	17.50134776	17.68536242	17.03232526	16.70077094	17.62914714	17.68250881	17.89484773	17.7872416	18.6662292	20.16633474	16.99258348	17.8585281	18.46228471	18.97197982	17.29651941	17.69659574
UXS1	0	0	12.81775024	0	0	13.0484601	12.91616266	11.8317404	12.61011108	11.10011305	13.37529098	12.26131203	13.36787968	13.53348718	10.55075359	13.16291649	12.40973353	13.03069205
RETREG2	13.6328356	13.87455339	13.46379762	13.50651909	13.94488173	0	0	15.07388561	13.58699345	0	16.60346596	13.82124964	14.96832508	14.6119232	12.7378851	15.43870659	12.90260375	14.48125597
SERBP1	20.46545132	20.32139705	19.7330892	19.80638727	19.4916327	18.93869193	18.68962214	19.3626883	19.53501807	19.99441007	19.38331379	19.96214258	19.49037509	18.9359496	20.2101163	19.8206254	19.4592944	19.42316799
C5orf15	0	15.21440584	12.53688937	0	14.6207211	0	12.38180036	0	0	0	12.50345734	0	14.41678735	0	13.89329949	15.75547584	14.22019113	0
LEMD2	18.11842088	18.22507693	17.73216463	16.72514207	17.34408307	16.47450335	16.77736118	16.63449756	16.47861386	16.61327442	17.57420973	17.54489194	17.72467653	17.37903774	17.38257311	17.18225628	17.03015656	17.25841996
FAM98A	15.42201419	15.93304829	15.89968338	14.99332611	15.45697311	15.45431649	16.79669348	15.55800397	14.85391458	15.53656216	15.9595439	15.5295136	14.65921004	16.22679758	15.77648625	16.48483506	14.94611704	15.27984264
[illegible]	[illegible]	[illegible]	[illegible]	[illegible]	[illegible]	[illegible]	[illegible]	[illegible]	[illegible]	[illegible]	[illegible]	[illegible]	[illegible]	[illegible]	[illegible]	[illegible]	[illegible]	[illegible]

TMBIM1	18.22601949	17.11633825	18.11642519	17.87835931	17.66840331	16.34223666	16.99014414	18.46504657	18.58501971	18.02428377	18.12106012	17.76540883	18.63240331	17.89186054	17.2088492	18.08920266	17.75210466	18.19183651
ERGIC1	17.88260229	18.22182973	15.69871982	17.18084217	18.09610901	17.86469341	17.57874974	16.81662999	17.33019749	16.97540693	18.23950108	17.95102551	18.10816023	17.63680736	17.83740975	17.99168644	17.59643638	17.17434178
UTP4	14.82939012	15.6052367	15.12049561	14.97803379	14.87898712	0	15.59661768	0	0	11.60448084	14.77646014	13.01865541	14.81260253	14.46130707	14.46601844	15.48557396	14.61489774	0
TBRG4	14.8845009	14.26327422	16.28402768	14.84391521	14.82047193	15.23956808	15.88427972	14.09108425	14.25018303	13.5474893	16.60997552	14.90399508	16.38882611	15.25661039	14.76377605	16.60392217	15.27144678	14.27889676
MTARC2	14.52668966	10.87735987	12.88366558	12.73647202	13.01260186	0	0	15.58659575	11.65855701	0	16.00898215	17.38477163	0	11.91372671	16.30229726	16.46705746	14.27920517	11.53923633
H2BC1	18.38040571	19.64829602	18.27532189	17.91984179	19.20205401	19.54331331	19.52011364	18.28118168	18.33038954	19.39900914	18.64894351	19.42411002	18.48610929	18.93618043	19.37939992	18.02368482	18.18117289	18.61772651
FAM162A	18.37362256	19.2097526	18.05152113	17.13240308	17.5354561	18.40947821	17.69796464	16.66969932	16.8942638	17.53052576	18.21682252	17.33736792	19.1054696	17.15174558	16.89707768	18.45664957	18.13321843	17.16636677
CCDC47	17.88589109	16.97618117	17.43365127	17.40012152	16.72067366	16.7050069	17.45022267	17.15910188	16.81846896	16.74702698	16.91182561	17.01149396	16.74955645	17.1479362	17.56526807	17.30386392	17.12118094	17.30686565
MRPL24	15.02213259	15.29454657	15.36894734	15.28974931	15.56472354	14.86951308	15.19153067	14.38831165	15.25771715	14.95752657	14.14684536	16.08548591	14.81421133	14.72147306	14.60648993	14.11381194	15.97645513	15.62708976
SYAP1	16.47240353	16.38193974	17.18502694	15.51096622	15.57134911	15.7603757	16.29238441	15.76546923	15.25182101	15.98312809	16.20047735	16.31637849	18.01729846	14.98675147	17.04808088	16.43000927	15.12409638	15.28480109
TMEM230	16.50681651	17.66001799	0	16.55358664	15.69689018	0	0	15.53618744	14.43325738	16.02810014	15.68690965	15.71056915	15.75265086	14.50556735	17.28400395	15.28304963	16.1445691	15.50257472
EXOC4	15.68521921	14.77436039	15.33293786	15.76533305	15.23868273	15.05424808	15.42708633	15.62667389	15.81613719	15.64496592	15.61909739	15.77607273	15.84868855	15.68062114	15.61592862	16.07336771	15.43675665	15.82522608
MAGOHB	18.32383213	18.60038856	16.76685885	17.92825188	17.76082023	17.84728985	17.11585664	16.81407341	16.6597071	17.77825183	16.42825614	17.25428762	18.16522521	17.02826593	18.02568148	16.53421144	17.65208378	17.21681201
RFT1	15.25709746	16.33051565	15.85512074	16.69891976	16.12449416	0	15.99808697	16.65905474	16.27759233	16.52141497	16.26037919	16.75192851	17.14384218	16.34883741	16.70281609	16.6039551	15.99504071	16.23810687
ISOC2	15.94684579	15.98926898	17.47632249	16.09323846	16.26916307	0	17.15786145	16.10780947	15.15535437	15.88832009	17.16990467	15.95884154	16.43534	16.68878014	15.42052054	16.90026227	17.18837212	15.41880047
FERMT2	15.00627026	0	15.41302782	14.48453848	13.13611483	15.05412567	0	15.108464	14.06197447	0	15.22523539	14.19566852	14.53526968	14.7111105	16.30491346	15.13401717	14.43927734	14.44588364
FUBP1	19.64452426	20.86807083	19.86013702	19.72967499	19.96821678	19.64830831	19.39641625	19.32723575	19.4992099	19.89984027	20.15197994	19.07496411	19.57009473	19.51448496	19.83838289	19.7638745	19.13111419	19.57790441
SLC25A46	16.45650445	16.0383842	15.18154629	15.34370216	15.4320753	0	15.76100342	14.99467006	14.70158377	15.91957096	15.93070693	15.22408487	15.60812519	14.81965628	15.43092325	16.45951204	15.72860469	15.81510835
LRRC59	20.10889657	20.29688429	19.43650149	19.58440138	19.42613241	19.62062648	18.62064751	19.65616155	19.07563697	19.22925186	18.89959073	19.6687477	19.22400668	19.31592568	19.85427403	19.09095718	19.57778688	19.19176138
PBXIP1	16.22459202	16.79456847	13.43285051	14.59341438	16.20382233	15.56327205	13.47587021	14.85501054	15.26564151	14.75204595	16.12072957	15.67761298	18.15445596	15.78481095	14.83157776	14.64600358	16.04557454	16.02432419
MCUR1	14.00774864	13.10425449	11.09672159	13.27786613	12.09754995	0	0	12.81058536	0	12.95001928	13.09319187	13.479447	0	12.59634595	12.4046385	13.35099379	13.39103127	13.03003074
KIAA1143	15.85369847	17.06201055	16.04093197	15.66863183	16.06572049	16.99118074	16.63154289	15.45383001	15.83968916	16.12807757	14.24403626	16.09700108	16.73535718	16.11599598	16.85154709	16.73673357	15.71692504	16.11270611
RPE	17.52020406	17.75605519	18.04272611	16.93844329	17.26061292	17.17408328	17.37951811	16.9726007	17.29588241	17.48720843	18.74435181	17.80281965	18.12995381	17.57791973	17.49039823	19.13143719	17.03464417	17.52896268
VPS33A	15.55670741	15.45421343	15.92284904	15.7468254	16.11731351	15.22814579	15.90427822	15.99555178	16.06921914	15.73187119	15.40573175	14.78690532	14.90227362	15.691893	15.31018756	15.23972554	15.58180983	16.27366714
FKBP10	16.98637798	15.96415523	15.99847913	15.97951041	13.9114196	17.56385249	16.62822747	17.10878684	15.69080985	15.19480464	18.06454757	15.86971216	17.73406994	16.93304054	16.89847952	17.41484831	16.69402146	16.19045377
ISG20	14.81331364	14.85961987	15.02465645	14.21060655	15.77194922	16.55031525	17.22168137	14.62890694	15.86302774	17.02168624	14.70647663	16.2054656	14.63018912	15.09325055	15.47208918	13.57078059	14.9562955	15.78235559
C18orf25	14.45390748	15.50227533	16.04905866	13.40158305	0	0	16.14381976	0	0	13.36584504	15.82623628	13.22820628	16.8995534	14.39437568	14.26224356	15.70005316	14.10272431	0
EXOC8	15.02580211	15.5961372	0	14.57512095	14.94758558	0	0	14.874647	14.12937987	14.56326529	12.33940735	13.90313419	13.91771406	13.50697506	14.59248361	13.33443902	14.31989479	14.41317971
AKT1S1	14.11332838	13.79029062	14.52083398	14.74891012	12.02675542	0	12.21119705	13.92915626	13.82357133	11.64258599	14.89744555	14.13233514	15.54733973	14.44275826	14.02312963	14.89591628	15.10671788	13.48454143
TOMM6	13.58997122	0	0	13.20928796	12.1487892	0	0	12.47342409	0	15.16590679	14.58545784	13.69499087	13.56004426	12.44923796	12.95700681	13.55877978	13.13782435	13.71269093
ZNF428	15.32896495	16.83760128	14.74239682	15.06887289	14.94635875	14.25638032	0	15.12662319	13.95090194	15.14819509	15.12734072	14.34516915	15.2958317	13.78262555	14.3193047	15.34099046	15.77129924	14.44839362
SH3KBP1	16.28522836	15.35526721	16.29045231	16.64299817	17.73277431	17.37976597	17.27471796	16.80573384	17.22200536	17.86060461	15.22915311	16.56798276	14.11455033	16.76852631	16.07295558	13.70548274	16.81964976	17.02273032
AIDA	15.68612453	16.08425546	16.60876997	15.83542883	15.56828691	15.74218606	15.73977103	15.70327216	16.34998739	15.37035044	15.5768612	17.00128689	15.48738215	15.02859509	15.62050466	15.93627381	15.91670846	15.58757931
ARL8A	0	0	13.64194247	15.08614659	0	0	0	15.11390428	0	13.61657756	13.89535728	12.78332151	0	15.35559669	0	0	14.74444287	0

[illegible]

RAD9A	14.05507337	13.12305842	14.24404207	14.2003118	14.23771588	13.9758032	14.97768957	13.57714377	13.74087887	14.33380258	14.72306115	13.98716624	14.88472241	14.34568746	14.45088351	15.09206802	13.86949604	0
SDHC	15.75545812	16.05129492	15.63950753	15.01317425	14.31452779	15.50437167	16.41787324	14.76064615	16.47160985	16.42069618	15.54401709	15.89707583	15.63942672	15.50159255	15.87857063	16.80903642	16.11238621	15.50920175
CHP1	16.55342622	16.96361227	16.60018163	16.87504333	16.36762364	15.8807036	16.2147371	16.44438961	16.31006382	15.99068716	16.65935302	15.76596296	16.68197795	16.94541972	16.25669475	16.28776563	16.75153446	16.30226002
KIF2C	0	14.49218082	15.2709423	13.61805811	12.76787933	15.49902105	13.64882867	12.41462369	12.45272235	0	14.79681733	13.03275554	13.89823814	11.78155862	13.89132389	14.13613205	12.15635609	12.8989259
ATXN2	14.5960918	14.52020526	14.8677706	13.94639873	14.26778668	0	0	14.23839133	14.09860818	0	15.10529935	0	13.85774262	13.56782497	14.34810583	13.65964809	14.17839724	0
HSD17B10	19.5511465	20.33603222	19.23960523	19.03877635	19.35918319	19.07544879	19.7890935	18.86263726	19.13758438	19.17978353	19.62686435	19.72836803	18.43584763	19.59280123	19.31635363	19.98556646	19.07886501	19.71417119
COL12A1	12.48104171	12.40087631	15.30739067	13.9852606	14.10558965	0	13.7331043	0	15.74928565	0	14.73354811	14.01749557	0	11.87430676	14.2246222	13.22027145	0	14.56161414
HNRNPAB	16.62126827	18.34457928	18.35228002	17.86583954	18.36803107	17.96247349	18.51661643	17.67208592	17.92005286	17.31746644	18.59853803	17.76249062	18.1157057	18.57041743	18.34878109	18.02295495	18.0225131	18.07798528
NAP1L4	17.83741832	18.14624376	18.37344494	17.80461298	18.70914599	18.71493983	18.7499134	18.15501383	18.30134129	18.32601398	18.47760277	18.77510768	17.85621882	18.12369628	17.98859602	17.88756973	18.36202433	18.14011533
MGST2	19.83459372	19.62965344	18.97833777	19.53670479	19.03271015	19.57838836	19.23997517	19.55701103	19.8523612	19.74628899	19.29990698	18.57608617	19.46778948	18.8324377	19.22257442	18.85700884	18.96982721	19.8225154
NAPG	14.84474598	15.39948721	15.20993788	16.37335134	15.20141921	16.2001669	15.15907046	15.56809772	15.76187521	15.27527272	16.48010877	16.2521926	13.35461326	15.80969425	15.5731124	17.11523245	15.38144935	15.46346313
TXN2	14.2408478	12.99812124	14.80306441	14.22183106	14.16117526	14.89744029	15.55397069	14.86013257	14.36207893	14.41451557	14.28414999	13.97003302	15.16973038	14.79341072	13.34160682	15.26232142	15.04147451	14.6384126
DMAC2L	15.05947386	0	14.83878999	15.09702344	14.82301741	15.08720108	14.4053882	15.20539176	14.91359744	14.69058407	14.73855236	15.05179591	14.88102207	15.71962539	14.8054972	15.16805154	15.37632481	14.58127645
MIPEP	15.59405381	15.27363925	14.3600409	14.59331452	14.95137028	14.41758627	15.86220562	14.57295021	14.22589538	14.77683419	14.83260371	14.69603618	15.56822474	15.341412	14.477995	16.39608576	16.14671906	14.63442777
ACO2	19.91058958	19.6690905	20.26531616	19.08853464	19.80744592	19.04072968	20.17599292	19.19052416	19.38831112	19.48104294	18.2379399	19.38690644	19.50109709	19.27319459	18.68175764	20.20366509	20.30514718	19.33920331
TM9SF2	17.43353107	17.45636081	17.160907	17.78547951	17.20738829	17.07104396	18.17290738	17.54062932	17.49453872	17.40826145	17.74621578	17.06168745	16.72007785	17.45155046	17.81563405	17.34677598	17.96036206	16.77352589
TSG101	16.71254253	15.27628914	14.94618979	15.75098937	15.23532302	14.6104806	14.10273904	15.17694634	14.96320125	14.84028609	14.29371159	14.99993637	15.42337011	14.90100902	15.10612999	15.25045818	15.56148642	15.12136404
CIB1	14.88721643	14.33048119	14.78098513	14.73454342	14.51513218	14.11176704	14.61129322	14.64444274	14.28708191	14.18956067	15.2844371	15.66786972	16.44209217	14.99517678	12.05473927	15.20595295	15.50174852	14.95551316
CPNE1	18.22388048	20.25408098	18.81629444	17.97031324	18.95402285	19.14015138	18.81053859	18.41201518	18.65143737	18.51927367	18.95554784	18.16969231	18.38788047	19.25023003	18.59851009	19.5082739	18.90539784	18.43849683
CCT7	18.4966033	18.34498596	18.56646974	17.76370586	18.36529561	18.34390896	18.47034559	17.95042988	18.26199374	18.24900796	19.06499121	18.4201535	18.6169165	18.14769171	18.79005681	19.16165673	17.68440791	18.06133559
MYD88	14.96220025	14.81754611	15.76484851	15.40686504	15.87866633	15.68710525	15.81400708	15.66251214	15.64714482	15.74528482	14.98886685	15.39626977	15.50747674	15.41675102	15.43033922	15.40518653	15.27054785	15.7177102
EBNA1BP2	16.91366619	17.81082362	16.45776098	16.20527676	16.83814539	16.41042431	16.53849955	14.88674911	15.25953223	15.71552678	16.27615934	16.66279787	16.30091542	16.46324995	17.04928871	17.07328614	16.15315755	15.79288311
ARID3A	0	0	0	13.79271226	0	0	0	0	0	0	0	0	0	14.39256859	0	0	0	0
PRMT1	16.69356672	18.0025843	17.89545852	17.52009061	17.37932178	18.4151326	18.17850575	17.38369006	17.22745231	17.42355093	17.50250066	17.45561042	18.47289532	17.63163047	17.147767	17.97273698	18.19630495	17.34188647
BAG1	15.62690679	15.63278403	15.08947343	14.32130236	15.21967445	0	14.92475298	14.68166876	14.33906626	14.64547923	13.55405351	14.68061814	16.0588867	14.19098449	13.80653812	12.1677077	15.12839354	14.99765449
AGPAT1	16.90652604	16.21608848	15.640812	16.19591208	15.99676258	15.94734853	15.67702987	15.89986617	15.66143116	16.0777288	15.73501613	16.62465079	15.42815261	15.47652645	15.84750329	15.57210783	15.34628702	15.62129488
PTPN18	0	0	11.09776114	12.82353508	13.72655371	0	0	12.67988681	12.33505159	0	0	0	0	0	11.7510281	0	13.58841018	13.3000971
PKP2	17.93917977	17.96608936	17.71488252	16.33215568	17.46497418	17.76846331	16.92298366	15.71788347	17.73222747	15.91234775	18.54374576	16.70306776	18.03411887	17.81412104	17.8415106	17.1138334	16.49838238	18.59685707
SH3GL1	17.81333273	15.98315027	16.58381684	16.46392032	15.96850711	16.24130734	15.16184461	16.55622923	16.36550921	16.28502935	15.50845587	15.55231985	16.4524279	16.53989892	16.65543257	16.77948362	16.2970023	16.11404461
RARRES2	13.54934262	11.43606096	13.80044791	14.39951172	14.05491196	15.93317862	16.34720597	15.0280008	14.01571177	15.8386116	13.15459935	14.77478465	10.78143386	13.51463027	13.24673437	12.23724819	14.61481968	14.49880612
TEP1	13.54740245	0	13.35309674	13.52317774	13.9751766	13.90388304	13.69428455	14.62992786	14.5966145	14.19895215	14.5187313	13.11576125	11.99374046	14.59816055	12.68990075	12.86765457	13.69077374	14.421547
VRK1	15.76078085	17.51145891	17.22738033	15.38439697	16.24083427	16.6839725	16.7481949	14.77341472	15.3467772	16.09909767	15.65350463	15.00859224	15.45612953	15.73829351	15.9772787	17.10518247	14.53123223	16.00210027
GDF15	0	0	0	0	0	0	0	12.34965041	0	0	12.76064899	16.46016727	0	0	13.91799252	0	0	0
AKAP9	14.78572424	14.33796292	14.21696827	13.30707348	14.88370566	0	13.97886143	13.89133057	14.12315222	14.45978692	13.26773925	13.66246726	15.00721569	14.38326936	12.75559136	13.85744754	13.61251522	13.9316957
[illegible]	[illegible]	[illegible]	[illegible]	[illegible]	[illegible]	[illegible]	[illegible]	[illegible]	[illegible]	[illegible]	[illegible]	[illegible]	[illegible]	[illegible]	[illegible]	[illegible]	[illegible]	[illegible]

PPIL3	15.61849686	15.12963975	16.14948029	15.27519868	16.39972305	15.78019301	16.0073199	15.61579766	15.72645548	15.9143291	15.17947968	15.61758158	15.20257674	14.97638949	16.05544927	15.58349956	15.7831925	16.35273086
LRMDA	13.30100462	0	14.27747655	14.78616003	14.00739022	0	13.10711744	14.97263506	0	13.486497	13.10469872	12.78860922	0	14.84698851	14.50265775	12.29227206	14.63043034	14.17254318
PDCL3	15.41845037	14.93014411	15.35123878	15.46704589	15.29836734	16.561099	16.74770478	15.61490391	15.26625011	15.52634246	15.061799	14.99390593	16.41679199	15.08883465	15.09440248	15.7219777	15.44252201	15.44975155
TAOK3	16.3984752	15.32761432	16.28648218	16.68225096	16.51205201	15.71095994	15.93571324	16.35557424	16.66580269	16.50127291	15.30403751	15.79567318	12.99285011	16.2667448	15.59308161	15.01501479	16.45680183	16.66212223
RAB3GAP2	16.57729005	16.0824944	16.84319995	16.45791663	16.41464318	16.25255023	15.64177075	16.6704087	16.74580591	16.66512672	16.41029697	17.06209144	15.82904988	15.75682199	16.23624271	16.14693297	16.48033944	16.61919861
ADNP	15.176457	16.08186974	14.36322843	14.58762509	14.62440125	15.82977373	15.28975775	13.97636895	14.48859015	14.65105487	14.86728836	13.19657751	15.01784605	14.50638555	14.58926402	15.29355135	15.43056957	13.75390807
DHX36	17.16268482	16.47462762	16.011763	15.57786408	16.70474059	16.95288279	15.58627184	15.43552377	15.39926084	14.85492724	15.58821687	14.99832185	15.98971559	15.56871229	15.65113843	15.80416993	16.48860412	16.54308458
PPA2	17.96390088	18.29414759	16.78758048	17.63204258	17.40103418	17.46942831	17.57534904	17.34810002	17.01304557	17.79817067	17.6781318	17.97358965	17.73770256	17.37388033	16.98243111	17.85060568	17.38472869	18.41862717
SPNS1	15.4479277	15.96867892	14.20377992	16.34652902	15.82629186	15.37915054	0	16.33298879	15.90510261	16.1993743	13.89528377	13.74958959	13.64788151	15.38559176	16.14569742	14.16848682	16.17336819	15.93219015
MS4A6A	0	14.60042814	15.80230573	15.96804729	14.05121326	0	0	15.83812	17.20489751	16.67997302	0	0	0	14.54313144	0	0	14.17410995	15.72838713
MRPL46	15.7642974	16.0852627	15.53433376	14.66286001	15.12166583	15.32180743	15.49615697	14.41795949	14.66865055	14.56939324	15.1823454	15.82696882	14.86500014	14.61931337	14.44640673	16.15687512	15.30118804	15.03838001
PNN	17.94244515	18.24122266	18.21782698	17.20877167	17.42664278	17.99263828	17.3688059	16.52737797	17.1171226	17.30705054	17.11023796	17.24177314	17.68445607	17.14163086	17.41379912	17.50896235	17.70341761	16.9166651
EPB41L4B	12.52956781	0	0	12.19484491	0	14.00205133	13.02169183	0	0	0	11.59142716	12.18768205	14.46363263	13.50610076	12.20890158	14.10670813	13.71292031	11.10644281
TMEM245	15.65999742	15.49934861	13.48737344	13.78257906	15.15085467	16.92770446	15.80012121	13.93503696	15.36974603	16.46510389	16.92406312	14.98163469	15.51830425	16.79579315	14.66853999	14.51082742	15.48914224	14.77843151
CPVL	17.80124507	17.29073296	18.65980442	19.10586744	19.73615571	17.55991559	18.51642839	18.33850504	19.2819999	18.66795675	16.66074063	17.61115609	14.10767556	18.8029342	17.43159108	15.96164204	18.60496636	18.82672653
C11orf68	16.7712943	16.12733726	16.64355765	15.88679543	16.36078066	16.42935923	16.62470221	16.03285776	16.17886906	16.6006576	15.92995392	16.63468389	15.61787613	16.03478755	16.86198895	16.97414138	15.88482626	16.25463393
GHITM	17.5607538	16.6555661	15.12595668	15.35358448	15.93698446	15.90387486	15.41160732	16.37423955	15.02006367	15.31561561	15.65665879	14.84339416	15.01067168	14.52190531	15.57588485	17.8451084	15.34749526	15.51334232
BOLA2	18.40965831	17.52708966	17.9438451	17.91404336	18.2409993	18.22932492	18.70121465	17.88727529	17.69338954	17.8766981	18.03836829	18.02289622	18.39152536	17.65821214	18.91963879	18.64970505	17.97514493	17.65357789
BOLA2B	18.40965831	17.52708966	17.9438451	17.91404336	18.2409993	18.22932492	18.70121465	17.88727529	17.69338954	17.8766981	18.03836829	18.02289622	18.39152536	17.65821214	18.91963879	18.64970505	17.97514493	17.65357789
TXNIP	13.62262094	12.09660032	13.5019692	15.28126759	14.29872307	0	14.92265436	15.07785427	0	15.84270837	14.45417979	14.7061407	16.82274965	14.92939721	13.65846484	14.7550902	0	0
TMX1	18.83069682	18.18709045	18.41358338	18.84811658	18.89895735	19.06360887	17.88093149	18.7727494	18.51205297	19.07261186	17.7544757	18.14320618	17.789115	18.31786262	18.67452947	17.02778977	17.89057944	19.05956
NELFA	16.09696442	17.2763637	15.376198	14.91679692	15.81184131	13.77932821	14.90587256	14.11488135	14.54335532	15.14478472	14.93148678	13.14324385	15.99394176	15.53433459	16.00326054	16.40715299	15.11768748	14.83464098
ACBD3	17.87004313	17.17198976	17.31168929	16.90097171	19.08424378	17.96269412	16.7364072	17.81371133	17.70659835	17.03295913	16.93329093	17.8890087	16.8064271	17.19566101	17.57401727	16.64471491	16.84259847	17.64486957
CDC42EP4	15.55925489	17.53121212	14.64140309	14.3405631	15.65191665	15.69635186	16.55545111	14.30311294	14.69311087	14.63375245	14.98969593	14.98796533	16.09024167	14.65705312	16.01163369	16.01600639	16.27110008	14.11580792
TPK1	15.48821011	0	14.66320237	14.63475511	16.05253538	14.77168995	15.2303883	15.70596949	15.78940947	16.30760357	12.15081006	15.7155074	12.84302978	15.12327886	13.5780937	12.85853863	0	15.81135696
PTPN23	16.51333641	17.24240642	16.18217003	16.45992947	16.32570388	16.43929409	15.9711505	16.35318803	16.43391598	16.17044452	15.17686913	16.08326343	17.19168609	16.25460019	16.68255124	16.98200819	16.7271212	16.07100106
UNC45A	16.24955865	15.90185387	16.49100214	16.03790164	15.75801587	15.85876882	16.02920094	15.59850418	15.56642677	15.96345864	16.27625662	16.1149336	16.62244299	16.04905085	16.26467124	17.66872866	15.66485598	15.85377375
MFSD1	15.36057814	15.72049426	16.65568259	17.25097393	16.04408656	0	0	17.81056595	17.36290911	16.24891982	14.39646969	0	14.81863971	16.01447072	15.58203514	14.5874937	16.16656062	17.40104199
DSN1	0	13.07833131	11.90865878	0	0	0	0	0	0	0	12.50744213	0	0	0	0	10.96195047	0	0
CHMP4B	18.9087743	19.13402547	17.37553824	18.33120918	17.93513465	18.0642497	16.50225615	17.8195069	17.32958888	17.71853852	17.84952537	18.03198517	17.51693893	17.82861059	18.76983512	17.14417837	17.90899825	17.54131408
RWDD1	15.12706988	13.02800113	13.73247013	14.90128869	15.35392161	14.46290536	13.06585638	15.18698591	14.94185	14.72971606	15.08599462	14.40167458	14.71340036	14.50245356	15.07677565	13.69822426	14.4633001	15.01760464
RBKS	14.72357804	15.57943817	14.54453942	14.14788855	14.95462676	14.60897984	15.59700466	14.20915972	14.07530711	13.49933182	13.68988848	13.64462064	15.33989403	14.36369752	14.72556818	16.63415874	15.5645369	13.46924806
FN3K	15.23259542	16.32429181	14.10505615	13.24231468	15.59490385	15.98684762	16.52521049	13.740475	13.51483623	13.71649619	13.85189989	15.62764008	16.38574894	14.44774765	15.11636189	16.49434632	16.10734432	14.9745422
POFUT1	15.46449639	16.77067448	16.41792911	16.50975592	16.21777655	16.0900019	15.04014471	15.94509765	16.34766232	15.09232535	16.86489872	16.05123732	16.87701203	16.22964529	15.5897684	16.10381732	16.14445474	15.82376227

MRPL16	16.10635527	15.38941261	15.65880443	15.57758453	15.27145391	0	15.87171295	0	14.84446193	15.63624049	15.95502594	16.27162232	15.80766976	14.39476242	14.3502061	0	16.01951268	15.22953103
NHP2	17.01525578	17.70182687	16.78511468	16.94185528	16.1384843	17.07174516	17.51428509	15.83234565	16.29158644	16.77473218	17.1604185	16.96851124	16.70317065	17.25406195	16.78679846	17.19678385	16.6216867	16.12806058
OCIAD1	18.72022964	17.95519857	17.91636506	17.35547356	17.86800845	17.79431512	17.83444511	17.62727771	17.1462405	17.8099301	17.20230321	17.36230104	18.16850533	17.42136092	18.16635037	18.62959386	17.80392564	17.87508834
ADPRS	18.29788924	19.47334605	18.04284265	17.92791356	18.13909352	17.63356711	18.70313875	17.4422761	17.69779172	18.11732509	18.00150114	17.53587906	18.74470175	17.80481659	18.27474782	18.23144518	17.32395716	17.92875293
MARCHF5	15.12855402	15.7297061	14.48814758	14.03512563	13.95721933	14.44656754	15.55873144	15.02821011	12.60844096	0	15.14971487	14.77614846	14.09572184	14.19805425	14.4031547	15.5079272	14.52237787	14.58625626
HYPK	11.83682819	13.13152809	13.33160283	13.11871332	14.40102818	13.42718098	0	13.55308786	13.81575902	14.3533901	14.26000884	13.53443956	13.48105687	14.57801697	13.57383715	14.23990993	13.86103483	13.761591
LYAR	14.36556341	14.72934732	14.89777761	14.888383	15.04352047	15.78180572	15.60505014	13.54135154	14.68238321	14.75762267	15.76863342	14.86031598	15.32400882	15.29295852	14.3895334	15.71479115	14.21967253	14.68772092
BPNT2	16.26525978	0	14.73340277	15.49851459	16.40183288	16.02336215	15.52924025	14.51242063	14.539743	15.44077161	15.99325941	15.49033604	15.47265899	14.58605899	15.10504784	15.98818686	15.35818208	15.20449831
CHCHD3	18.45795444	17.91953114	17.55142113	17.70432995	17.58670718	17.55636303	17.61900321	16.95500323	17.17961939	17.5381592	17.25819867	17.15326689	17.54904538	17.45033608	17.66565438	17.78789927	17.57468845	17.6151011
CMTM6	15.29015627	14.51478832	12.89638904	14.76436513	13.80153234	11.57903103	13.39577128	14.25367987	14.47848117	13.4610521	13.81322311	12.81873137	16.01278308	13.96806188	12.56398465	13.88958913	14.0376877	14.52949394
SIRT5	16.11234962	14.74134652	13.27340164	13.13071273	14.52646175	14.12037205	14.5703923	12.94258618	12.09228276	13.88981604	14.59188285	15.01567128	15.36434242	13.89483312	13.65323893	15.42066795	14.32724356	14.90541473
MIOS	14.63944847	14.12358199	15.80291626	14.65650855	14.9418534	14.34335165	15.55398171	14.71001088	15.01902666	14.63702856	14.34995554	14.39576109	14.63680337	15.4887751	14.03409536	15.27023101	14.80670222	15.07548568
SMPD4	14.83810682	15.38448932	15.28203116	14.35750422	14.49812767	14.81316369	14.99722704	14.52232423	14.68864474	14.38620142	15.62862453	15.16870517	15.33011869	15.13402061	14.65249074	15.37657348	15.24041214	14.62444944
TEX10	14.66134145	16.29896144	15.6336892	14.25030839	14.60895999	15.2214051	15.28514325	13.82148361	14.24875453	14.23524997	15.89797901	14.89814273	15.37824484	15.11794504	14.97864021	15.00555756	14.79597939	13.97532007
DCAF16	12.47346869	13.32569029	12.4733468	10.37005971	13.07855983	0	12.80112308	0	11.55527802	12.23084459	12.88760691	13.32311016	12.41841665	10.49846371	0	13.9618718	0	0
THUMPD1	16.13876536	17.26373727	16.59244791	15.91937047	16.40011044	15.77151073	16.42231094	15.77792709	15.71301558	16.21584475	16.51577658	16.25327216	16.02799405	16.78414437	17.08517597	16.79102941	16.486613	16.41810945
TOR4A	14.99871411	14.48879428	15.20451458	16.11227436	15.98465887	13.09845607	15.12105538	16.42753766	16.22045996	15.75681293	13.89256357	13.35308004	14.5352228	15.32314082	14.83094821	13.39634294	15.36033058	15.83737732
TRMT1	15.30696255	16.29894061	15.4190764	14.77268729	14.29055566	14.23513542	0	14.07380146	14.37319281	11.16081531	15.22721094	15.25669359	15.137812	14.27961511	14.92359743	15.02081789	14.96536896	14.37434915
BABAM2	14.97032895	15.38547999	15.15391117	15.05782684	15.52550265	14.35560395	15.09462863	15.60284767	15.36321546	15.50947771	15.8126931	15.46440092	15.22317198	15.25010075	15.63190219	15.44312567	15.53725917	15.79584744
QPCTL	13.93219258	16.07164601	16.41504915	14.85382861	15.95720754	16.00949498	15.84456728	15.11723788	14.68027367	14.83512076	15.57529491	15.83751938	15.97343287	15.52801657	15.56833273	16.26907216	15.24312894	14.82271805
CDKN2AIP	14.44646343	15.66471376	14.35898677	14.53148451	14.8232751	15.31718958	15.61445172	13.8325844	13.29704681	15.16384159	14.00671951	14.56980776	14.95863679	14.8211414	14.87997421	14.15306707	15.00170059	14.91895538
DNAJB12	14.46611531	13.2719303	13.59830096	14.74617159	14.72752841	14.89341986	13.10518439	14.32985819	14.17341638	14.6581086	15.12663973	14.25964141	13.80167205	14.22578082	14.3574077	15.70920774	13.92848539	14.31546691
DDX43	16.82109409	17.32411337	16.28467106	16.58006001	17.53864779	15.83120942	16.81228024	16.09048511	16.97164871	16.79971047	16.1784983	16.47285269	17.00950369	14.93039527	16.65730078	16.88352921	16.19946417	16.94078243
GAR1	17.85651415	19.04318502	18.18567034	17.13891406	17.74787858	17.88656047	18.75126781	16.93883674	17.25368954	17.61520956	18.07345025	18.05754394	18.32475053	18.03569888	17.91643322	18.50850858	17.93632756	18.10584426
STAB1	15.93937162	17.06716516	17.63408228	18.61317255	16.64899638	19.9191613	18.70990017	18.72650673	17.64919389	17.64307152	16.36005095	17.86010628	16.03827417	17.23506192	15.6944021	15.65250879	17.58406424	18.10467553
PPP4R2	14.49873492	15.64645115	15.01358814	14.30796176	14.89449862	15.2691742	13.92104768	14.11476096	13.2073452	14.78128356	14.48211442	14.40940414	14.73666938	14.02891271	14.34549161	15.44244721	14.22992248	14.23101487
DPP3	19.05368414	18.28355951	19.24594027	17.76369198	18.45341182	18.20975439	17.75502437	17.97039461	18.14978464	18.11747189	18.19849315	18.23894215	19.15065622	17.76265606	18.38020923	19.26260358	18.18576137	18.25902586
AATF	15.23463166	15.01629942	14.66043665	14.82606944	14.55254727	15.28427908	14.56283958	13.38970197	13.55993402	13.82058976	14.20716375	14.43973592	14.58268032	14.65122144	15.36166192	14.91927771	14.18871394	13.31021336
DDX56	14.00776113	15.90749796	15.54416912	14.25729389	15.236926	15.39466614	15.33417725	13.90757543	13.91949213	13.41150659	14.91026741	14.70124522	14.80837741	14.95270126	15.07990595	15.7408799	14.76150812	14.06189803
TERF2IP	14.2214824	13.80228768	14.49452652	13.80467265	14.82773509	14.52719348	14.0776892	13.54973309	14.08816048	14.96825142	14.05166931	13.95101047	13.35690111	13.74069084	14.38034268	11.66920093	14.14305353	13.8201099
ABI2	17.04247414	16.09527741	14.97594406	16.08485704	16.04346958	16.10536617	16.00996817	15.44404111	15.68006133	16.49759703	16.07371076	15.95174697	17.01478704	15.70582019	15.81307305	17.15481471	16.11635078	15.90896949
BCLAF1	17.87951183	17.49742221	16.95112496	17.39736285	16.89398368	16.8410894	16.27270154	16.73375848	16.51958911	17.06450415	16.24379968	16.89101149	17.30510501	16.9966601	17.10195612	16.49552265	17.47659023	16.78343132
PSD3	16.5307128	16.44670244	16.19524739	17.95621058	16.61906882	19.82903971	18.62954517	18.45833226	18.8374368	18.62168804	15.1785638	18.33256	16.41064582	16.7104383	17.63804099	15.00393955	17.88830251	15.45846532
COA4	15.2854152	13.56887064	12.73286725	14.8588352	14.96117499	15.29223259	16.71072363	0	12.72813372	13.64836406	15.02073736	15.05984283	15.24383047	14.33820143	15.26863329	17.08869509	14.53110358	14.76143619
[illegible]	[illegible]	[illegible]	[illegible]	[illegible]	[illegible]	[illegible]	[illegible]	[illegible]	[illegible]	[illegible]	[illegible]	[illegible]	[illegible]	[illegible]	[illegible]	[illegible]	[illegible]	[illegible]

MINPP1	14.76297483	14.64671317	14.34903969	14.44978807	14.07507726	16.50667658	13.57789162	15.30970865	13.97974354	12.61315175	15.21886901	14.70495784	14.43422849	14.04806525	14.15846624	14.06823492	14.07312381	15.1722494
NOVA2	9.976525055	14.36887539	0	0	0	0	0	0	0	0	13.60191924	11.3606139	14.88690993	11.22899611	0	10.71265703	0	12.16259401
RPL26L1	19.70010662	19.60085504	19.00542016	18.84509222	19.230382	19.19844489	19.93195083	18.93428801	19.03941057	19.13457124	19.51688942	19.2778189	19.24452411	19.14757744	19.61534159	19.2559957	18.90692912	19.17655561
WDR3	13.75660824	14.48666491	14.28818371	14.01328416	13.97996991	14.28280805	15.41083341	12.7510237	13.13814147	12.47618477	13.57481085	13.64555531	13.7488875	13.92476993	13.11578189	14.98504757	14.17759168	13.60019536
NSFL1C	17.0257548	19.51110416	17.28633073	17.92275842	18.20474769	17.82848663	18.07241511	17.42176874	17.80886169	18.01140711	17.30672675	18.00347862	16.44358112	17.32817267	18.92137608	18.37351105	17.64193156	17.95366868
MACF1	16.77012064	17.41397417	16.54041293	16.3343078	16.57225335	17.22454448	17.0057729	16.36735756	16.43276858	15.89208547	16.33738684	16.04140532	18.43712203	16.83394706	16.16230112	16.88204529	16.31598204	16.6370489
PPP6R1	15.69578965	15.88912932	15.9083699	16.03561515	16.54169278	16.29908194	16.13673881	16.43156787	16.44168348	16.89560283	15.48771837	16.11599233	15.60267519	16.37327207	15.38465255	15.12397155	16.22322635	16.26442155
TRIM33	13.05905972	13.23771107	13.09329344	13.56855807	0	13.65040559	13.36582902	12.99839571	12.48259774	13.30757105	14.19099157	13.79246742	13.47071596	13.81176978	13.54463245	13.92823426	12.64343258	12.51906366
LIMCH1	14.87736746	12.49847174	13.41458576	12.84218948	13.53248684	13.70816288	13.96900632	0	0	0	12.56396651	13.56038812	13.82098896	13.85098036	12.69967517	13.46402256	13.2194526	11.70821478
SMG5	15.86748614	15.45110834	15.38772365	15.13751559	15.61596779	15.01579139	15.57392508	14.4205641	14.48278802	14.64197411	14.77776961	14.91509834	15.30938989	15.09853044	14.81073028	15.41555288	15.20932708	15.10514474
EXOC7	16.11488961	14.89945613	14.59540342	15.60077804	15.62891706	15.25406555	14.46233776	15.16574091	15.3438534	15.28759656	14.62666431	15.03096158	14.99941333	14.92205416	15.13450466	15.52200843	15.18942207	15.31228915
ZC3H4	15.62976615	16.15082973	16.3735996	15.93558098	15.87296649	15.9002631	16.08012756	15.30191924	15.55273347	15.83014747	15.5890755	15.02537058	15.89538363	16.13670268	15.5289454	15.94287277	15.83679978	15.50090824
USP24	19.7856163	15.52645064	19.83883941	19.55636935	19.78776898	18.23842588	19.73769998	19.52640038	19.8682065	19.81555334	19.47660692	18.88476321	19.74468071	19.34302993	19.4535555	19.29264011	19.19575247	14.80025474
TBC1D2B	13.68519001	12.05708578	13.88790676	13.97645784	14.56846216	0	0	14.82870771	14.74402182	14.69785534	13.34045403	8.850906268	0	14.15505074	13.36007829	0	14.00000189	15.45734969
DICER1	14.94719646	14.86488059	15.75130766	15.83342597	14.95022945	14.1637722	14.22849901	15.67950362	15.90725818	15.28611185	14.04811548	13.22876791	14.43812723	15.1130172	14.23377004	14.92243741	15.08812602	14.85221326
SHOC2	13.40363478	13.31024332	14.48727689	14.15850738	14.59327975	13.62026682	0	13.89943815	14.04395211	14.78644752	14.77753857	14.86371263	14.01397227	15.47371504	14.34284864	15.28928848	13.75251112	12.81422942
SRRM2	18.50632635	18.63806851	18.59181017	18.30149424	18.20803948	18.25699974	18.2073023	17.92288415	17.6905375	18.23653775	18.53479254	17.78373165	18.46682354	18.48241162	18.32065612	18.34928595	18.46182913	17.93879045
PA2G4	19.60310516	19.33932049	19.66096189	19.40478154	19.53046742	19.98217439	19.72943975	19.13925796	19.21315585	19.23933048	19.71904492	18.90267237	19.08328973	19.23333705	19.66480141	19.29246473	19.37910624	19.31139813
BAIAP2	17.5413968	16.50004071	16.42771617	16.39570592	15.49324028	17.19249736	17.26509707	15.89076719	15.24553124	15.20987514	16.65209483	16.94167837	17.28652843	17.01757066	16.55023715	18.34319466	17.85348032	15.39548602
GAB2	12.60591012	0	12.85032554	12.11946266	13.6742537	0	14.00400113	0	12.91094524	11.79814525	14.13445828	0	13.22588553	11.79588498	14.09404337	14.83096242	0	12.92671612
SMC3	16.47215559	16.88321977	16.96822891	16.16345175	16.96870423	17.21892709	16.53675873	16.0474235	16.43455862	16.5493197	17.02010302	16.87116386	16.64562402	16.71989638	16.64417359	16.98340316	16.21590206	16.30719831
CHMP2B	16.61795121	17.28311818	15.84065683	17.23951127	16.72667261	16.1920917	14.59263599	16.96764105	16.42907747	16.66192768	15.28639532	16.52779208	17.89606507	16.22462249	16.77528967	16.57242129	17.1878483	16.83430776
ZNF148	14.65873155	15.01091323	15.50232693	14.96809432	15.01662928	15.72947595	15.78549667	14.65041019	14.69123384	14.60769919	16.45597657	15.49342573	16.38979267	15.32417368	14.49634681	15.68034536	15.40998648	14.83146653
MTMR6	15.02783548	14.61447643	14.42932156	15.51062933	14.41970537	0	13.33189677	15.10848075	14.52074036	15.11112325	13.80428654	14.11170497	13.45307642	15.07728042	13.80794937	15.04160735	15.72692274	14.82115581
[illegible]	[illegible]	[illegible]	[illegible]	[illegible]	[illegible]	[illegible]	[illegible]	0	12.45733053	13.01738784	14.18040378	13.17623297	13.93562036	14.29266609	14.31695432	14.24249423	13.91970216	0

1 | DETAILED MATERIALS AND METHODS

1.1 | Proteomics Data Generation

Paraffin was removed from the FFPE sections using a dewaxing liquid, and the sections were subsequently transferred to an Eppendorf tube and broken by sonication (300 mM Tris-HCl 50% TFE) for 30 min. Then, the protein was heated at 90 °C for 90 min to de-crosslink the proteins, cooled to room temperature, and ultrasonically broken again for 30 min. Addition of reducing and alkylating agents to break disulfide bonds in proteins. After completion of the reaction, the volume of lysate in the Eppendorf tube was reduced to about 20 μl using a vacuum heat dryer, and Trypsin dissolved in 10% TFE (H_2O) was added for overnight digestion for 16 h. After digestion, TFA was added to the enzyme digestion solution to make a final concentration of 1% and the enzyme digestion solution was transferred to SDB-RPS-Tip for desalting. The concentration of the desalted peptide fragments was determined by NanoDrop and frozen at -80°C for storage. Three samples were taken from each group to obtain the distribution pattern of peptides using the conventional Data-Dependent Acquisition (DDA) mass spectrometry acquisition scheme and the PD mass spectrometry data analysis software, and then divided into the mass spectrometry variable acquisition window, and then the samples were analyzed by Data-Independent Acquisition (DIA) mass spectrometry. DIA mass spectrometry data were parsed using the directDIA function in spectronaut.

1.2 | Cell lines and cell culture

SKOV3, Caov3 and OVCAR3 cell lines were obtained from the National Intrastructure of Cell Line Resource. SKOV3 cells were cultured in McCoy's 5A (Wuhan Pricella Biotechnology Co., Ltd.) medium containing 10% Fetal Bovine Serum (FBS, PAN). Caov3 cells were cultured in Dulbecco's modified Eagle medium (DMEM, Gibco) medium containing 10% FBS (PAN). OVCAR3 cells were cultured in Roswell Park Memorial Institute 1640 (RPMI 1640, Gibco) medium containing 20% FBS (Gibco). All cell lines were incubated at 37 °C in a humidified atmosphere with 5% CO_2.

1.2.1 | Establishing cisplatin-resistant ovarian cancer cell line

SKOV3 cells were grown stably for about one week, and then 0.2 μg/ml of cisplatin (Qilu-pharma) was added to the medium. After that, the concentration of cisplatin in the medium was gradually increased by 0.4, 0.5, and 0.8 μg/ml every other week to induce drug resistance (passaging was carried out on a 1:3 basis whenever the cells grew up to 70% to 80% of the petri dish). Obtained in vitro-induced cisplatin-resistant ovarian cancer cell line SKOV3/DDP.

1.3 | Peripheral blood $CD4^+$ and $CD8^+$ T cell isolation and activation in vitro

$CD4^+$ and $CD8^+$ T cells were derived from healthy human peripheral blood. Peripheral blood samples were added to centrifuge tubes containing separating solution, and cells of different densities were stratified by gradient centrifugation, with lymphocytes settling in the middle layer. After aspiration, specific CD8 (Milteny Biotec) and CD4 Naïve (Biolegend) magnetic beads were added for sorting to obtain $CD4^+$ and $CD8^+$ T cells. T cells were activated in vitro using ImmunoCult™ Human CD3/CD28/CD2 T Cell Activator (25μl/10^5 cell, Stemcell Technologies) and were incubated in ImmunoCult™-XF T Cell Expansion Medium (Stemcell Technologies) containing 100 U/mL of penicillin/streptomycin, cultured at 37°C in 5% CO_2, and 2.5 ng/ml IL-2 (ThermoFisher Scientific) was added every 72h.

1.4 | Immunohistochemistry and Scoring

IHC staining was performed using a fully automated immunohistochemistry machine, Roche BenchMark GX, and a fully automated immunohistochemistry staining system, BenchMark ULTRA. Sections were blocked with goat nonimmune serum, and the primary antibodies used were SH3YL1 (1:300, Aviva Systems Biology) and CD4 (1:300, Cell Signaling Technology). Staining was analyzed and scored by two experienced pathologists. Samples were scored on a scale from 0 to 3 (0, negative; 1, weakly positive; 2, moderately positive; 3, strongly positive) based on the intensity and the percentage of positive staining. The staining intensity score was multiplied by the percentage of the slices exhibiting staining for the object of interest to calculate the "Hscore" of the object of interest.

1.5 | Multiplex immunofluorescence staining

FFPE tissue sections were deparaffinized, rehydrated, then subjected to a two-step antigen retrieval protocol. First, slides were incubated in an acidic retrieval buffer; subsequently, they were transferred to an alkaline retrieval buffer. After blocking nonspecific binding, nuclei were counterstained with DAPI. Multiplex staining was performed using an iterative "stain–image–bleach" cycle: in each round, slides were incubated with DAPI plus up to four fluorescently labeled primary antibodies (CD4 (1:100, Abcam); CD8 (1:100, Abcam); Panck (1:200, Invitrogen); CD44 (1:200, Novus Biologicals); GZMB (Granzyme B, 1:400 Cell Signaling Technology); PD1 (1:100, Abcam); TCF1 (1:200, Cell Signaling Technology); TOX (1:200, Abcam)), followed by imaging on the Cell DIVE platform; subsequent chemical bleaching was used to inactivate fluorophores, allowing additional rounds of staining. Autofluorescence images were acquired prior to antibody staining in each cycle for background correction. Multi-channel images were analyzed using HALO software to identify cell–cell spatial relationships across the tissue.

1.6 | siRNA transfection and lentiviral transduction

For transient knockdown experiments that targeting CD44 in T cells, we used three synthetic siRNAs and a non-targeting control siRNA (Table1). T cells were plated at 8 × 10^5 cells per well in a 12-well plate, transfected with siRNA (or control) in serum-free medium using Lipofectamine 3000 (Invitrogen), and after 8 hours the transfection media was replaced with complete medium. Knockdown efficiency was assessed 48 hours later by WB.

For SH3YL1 manipulation in ovarian cancer cell lines, the full coding sequence (NM_015677.4) was cloned into the pLV3-CMV-3×FLAG-MCS-Puro vector for overexpression; the empty vector was used as control. For knockdown, three shRNA sequences targeting SH3YL1 (Table S24) were cloned into the pLKO.1-Puro plasmid. Lentiviral particles were produced by co-transfecting HEK293T packaging cells with the expression or shRNA plasmid plus helper plasmids using Lipofectamine 3000, and viral supernatants were harvested at 48 and 96 hours post-transfection. Recipient ovarian cancer cell lines (SKOV3, SKOV3/DDP, Caov3, OVCAR3) were infected with the lentivirus in the presence of 10 μg/mL polybrene (Santa Cruz Biotechnology) for 8 hours at 37 °C, then the medium was replaced with fresh complete medium. Stable cell populations were selected by puromycin, and overexpression or knockdown efficiency was verified by WB.

1.7 | Flow cytometry

Cells were first stained with Zombie (live/dead dye, Biolegend) at 4 °C for 15 min, centrifuged at 1500 rpm for 5 min, resuspended in 100 μL staining buffer, after which surface-marker antibodies (CD4 (2.5ul, Biolegend), CD8 (2.5ul, BD Biosciences), CD44 (2.5ul, Biolegend), PD-1 (2.5ul, Biolegend)) were added and incubated in the dark for 15 min at 4 °C. Cells were then washed, fixed and permeabilized using the kit (Staining Buffer Set, eBioscience) before addition of intracellular antibodies (IFN-γ (2.5ul, BD Biosciences), GZMB (2.5ul, Biolegend), TCF-1 (2.5ul, Biolegend), TOX (2.5ul, Biolegend)), incubated for 30 min in the dark, washed twice, resuspended in 200 μL buffer, and acquired on a flow cytometer (BD LSRFortessa).

Data were processed using FlowJo (v10.10.0) for high-dimensional analysis. Initial gating excluded debris (forward vs side scatter), doublets (FSC-A vs FSC-H), and dead cells (live/dead dye negative); subsequent identification of cell populations was based on marker expression, candidate populations were identified by unsupervised methods (e.g., t-SNE + FlowSOM + Cluster Explorer) and optimized polygon gates maximizing purity and yield were generated using an automated gating tool (e.g., HyperFinder) and applied uniformly across samples for downstream quantification or sorting (Figure S12).

1.8 | Cell Counting Kit-8

1.8.1 | Detection of sensitivity of ovarian cancer cell lines to cisplatin

The IC_{50} of cell lines against cisplatin were determined as follows: SKOV3 and SKOV3/DDP cells in the logarithmic growth phase were counted, diluted, and inoculated in 96-well plates with 6 replicate wells in each group. After cell adhesion, serum-containing medium with gradient concentration of cisplatin (0 μg/ml, 0.5 μg/ml, 1 μg/ml, 2 μg/ml, 4 μg/ml, 8 μg/ml, 16 μg/ml, 32 μg/ml, and 64 μg/ml) were applied to the cells and then incubated for 36 h in an incubator, and the cell proliferation was determined by using CCK-8 (Dojindo) reagent. Proliferation inhibition rate = (absorbance of the experimental group - absorbance of the blank group) / (absorbance of the control group - absorbance of the blank group) × 100%. Each group of tests was repeated at least 3 times.

1.8.2 | Effect of macropinocytosis on the sensitivity of cell lines to cisplatin

In the logarithmic growth phase, SKOV3, SKOV3/DDP, SKOV3-Ctrl, SKOV3-OE, Caov3-Ctrl, Caov3-OE, SKOV3/DDP-shCtrl, SKOV3/DDP-sh1, SKOV3/DDP-sh2, OVCAR3-shCtrl, OVCAR3-sh1 and OVCAR3-sh2 cells were counted, diluted, and inoculated in 96-well plates with 3 replicate wells per group. After cell adhesion, the serum-containing medium containing a gradient concentration of cisplatin (as previously described) with 12.5 μM EIPA (Selleck) or an equal volume of Dimethyl Sulfoxide (DMSO, as solvent of EIPA) was added, and then incubated for 36 h, and cell proliferation was determined using CCK-8 reagent. Each set of experiments was repeated at least 3 times.

1.9 | Western blot

Samples were lysed in a mixture of Ripa lysate and protease inhibitor, and the supernatant was collected as protein after centrifugation at 14,000 rpm for 10 minutes at 4 °C. The concentration of proteins in the lysate was measured using the BCA Protein Assay Kit (Thermo Fisher). The extracts were resolved by SDS-PAGE on a 4%–20% gradient gel (ACE Biotechnology), transferred, and incubated with the indicated antibodies, followed by detection using enhanced chemiluminescent regent (Applygen). The optical density values of the immunoreactive bands were calculated using Image J software (v1.54i).

1.10 | Single-cell metabolome assay

Cell suspensions of SKOV3 and SKOV3/DDP were centrifuged at 1000 rpm for 4 min at 4 °C and the supernatant was discarded. Add 500 μl of cell buffer, gently aspirate, and dispense to mix and wash the cells. Cell smears were prepared using the above cell suspension samples, and a single-cell mass spectrometer (SinCell-100, CII Tech Co., Ltd.) was activated, which enables automated single-cell localisation, single-cell metabolite extraction, and ionisation of single-cell samples. The single-cell metabolite

data were obtained by mass spectrometry detection using a Thermo Q Exactive HF mass spectrometer.

1.11 | Image analysis

All image processing and analysis were performed using Image J software (v1.54i). Convert the WB image to 8-bit, using subtract background function to eliminate the background influence, set Area, Mean grey value, Integrated Density as the quantitative parameters, pixels as the quantitative unit, using rectangle tool to select the target bands and analyze to get the grey value of the bands, and use the grey value of the internal control protein to normalization.

1.12 | Proteomic data analysis

We used the minimum value to fill in the missing values in the protein expression profile and performed a log2 transformation. We then used mixOmics for PLS-DA dimensionality reduction; limma for differential analysis; function enrichKEGG of clusterProfiler for KEGG enrichment; pheatmap and Complex-Heatmap for heatmapping; circlize for chord mapping; GSVA for ssGSEA (annotated gene sets including: c2.all.v202-3.2.Hs.symbols.gmt, c5.go.bp.v2023.2.Hs.symbols.gmt, c5.go.cc.v2024.1.Hs.symbols.gm, h.all.v2023.2.Hs.symbols.gmt, c7.all.v2023.2.Hs.symbols.gmt, c5.go.bp.v20-23.2.Hs.symbols.gmt); IOBR for immuno-scoring; gwasrapidd, TwoSampleMR and MendelianRandomization for Mendelian Randomization analyses; drc to plot dose-response curves; function scale of base for data normalization; function glm of stats to construct logistic regression models; pRRophetic for IC50 prediction; networkD3 to plot Sankey diagrams; pROC to plot ROC curves.

1.13 | Single-cell data analysis

We cited single-cell sequencing data of ovarian cancer ascites from a related study at Shanghai Jiaotong University School of Medicine; low-quality cells were filtered out if cells had fewer than 600 genes or more than 5000 genes expressed, as well as >30% unique molecular identifiers (UMIs) linked to mitochondrial genes, then ues the NormalizeData and ScaleData functions of Seurat for logarithmic normalization and linear regression of gene expression matrices; harmony for data integration; scTyper for cell type annotation by scoring individual clusters according to their given cell markers (Table S11); SCpubr, sscVis and scRNAtoolVis for single-cell data visualization; CellChat for cell communication analysis. The potential interaction strength between two cell subsets was predicted based on the expression of ligand–receptor pairs. To characterize the subtype preference distribution of cell subclusters, $R_{o/e}$ was calculated by package Startrac.

Software / Package	Version	Reference
mixOmics	6.28	
limma	3.60.4	[1]

Software / Package	Version	Reference
clusterProfiler	4.12.6	[2]
pheatmap	1.0.12	
ComplexHeatmap	2.20.0	[3]
circlize	0.4.16	[4]
GSVA	1.48.3	[5]
IOBR	0.99.9	[6]
gwasrapidd	0.99.17	[7]
TwoSampleMR	0.6.5	
MendelianRandomization	0.10.0	
drc	3.0-1	
base	4.4.1	
stats	4.4.1	
pRRophetic	0.5	[8]
networkD3	0.4	
pROC	1.18.5	[9]
Seurat	5.1.0	[10]
Harmony	1.2.1	[11]
scTyper	0.1.0	[12]
SCpubr	2.0.2.9000	[13]
sscVis	0.1.0	
scRNAtoolVis	0.1.0	
CellChat	1.6.1	[14]
Startrac	0.1.0	[15]
R	4.4.1	
FlowJo	10.10.0	
ImageJ	1.54i	

Table S26. Software and computational tools used.

REFERENCES

1. Phipson B, Lee S, Majewski IJ, Alexander WS, Smyth GK. ROBUST HYPERPARAMETER ESTIMATION PROTECTS AGAINST HYPERVARIABLE GENES AND IMPROVES POWER TO DETECT DIFFERENTIAL EXPRESSION. *Ann Appl Stat*. 2016;10(2):946-963.
2. Wu T, Hu E, Xu S, et al. clusterProfiler 4.0: A universal enrichment tool for interpreting omics data. *Innovation (Camb)*. 2021;2(3):100141.
3. Gu Z, Eils R, Schlesner M. Complex heatmaps reveal patterns and correlations in multidimensional genomic data. *Bioinformatics*. 2016;32(18):2847-2849.
4. Gu Z, Gu L, Eils R, Schlesner M, Brors B. circlize Implements and enhances circular visualization in R. *Bioinformatics*. 2014;30(19):2811-2812.

5. Hänzelmann S, Castelo R, Guinney J. GSVA: gene set variation analysis for microarray and RNA-seq data. *BMC Bioinformatics*. 2013;14:7.
6. Zeng D, Ye Z, Shen R, et al. IOBR: Multi-Omics Immuno-Oncology Biological Research to Decode Tumor Microenvironment and Signatures. *Front Immunol*. 2021;12:687975.
7. Magno R, Maia A-T. gwasrapidd: an R package to query, download and wrangle GWAS catalog data. *Bioinformatics*. 2020;36(2):649-650.
8. Geeleher P, Cox N, Huang RS. pRRophetic: an R package for prediction of clinical chemotherapeutic response from tumor gene expression levels. *PLoS One*. 2014;9(9):e107468.
9. Robin X, Turck N, Hainard A, et al. pROC: an open-source package for R and S+ to analyze and compare ROC curves. *BMC Bioinformatics*. 2011;12:77.
10. Hao Y, Stuart T, Kowalski MH, et al. Dictionary learning for integrative, multimodal and scalable single-cell analysis. *Nat Biotechnol*. 2024;42(2):293-304.
11. Korsunsky I, Millard N, Fan J, et al. Fast, sensitive and accurate integration of single-cell data with Harmony. *Nat Methods*. 2019;16(12):1289-1296.
12. Choi J-H, In Kim H, Woo HG. scTyper: a comprehensive pipeline for the cell typing analysis of single-cell RNA-seq data. *BMC Bioinformatics*. 2020;21(1):342.
13. Blanco-Carmona E. Generating publication ready visualizations for Single Cell transcriptomics using SCpubr. *bioRxiv*. 2022:2022.02.28.482303.
14. Jin S, Guerrero-Juarez CF, Zhang L, et al. Inference and analysis of cell-cell communication using CellChat. *Nat Commun*. 2021;12(1):1088.
15. Zhang L, Yu X, Zheng L, et al. Lineage tracking reveals dynamic relationships of T cells in colorectal cancer. *Nature*. 2018;564(7735):268-272.
